# UTP14A, DKC1, DDX10, PinX1, and ESF1 Modulate Cardiac Angiogenesis Leading to Obesity-Induced Cardiac Injury

**DOI:** 10.1155/2022/2923291

**Published:** 2022-06-13

**Authors:** Xiaoyu Pan, Shuchun Chen, Xing Chen, Qingjuan Ren, Lin Yue, Shu Niu, Zelin Li, Ruiyi Zhu, Xiaoyi Chen, Zhuoya Jia, Ruoxi Zhen, Jiangli Ban

**Affiliations:** ^1^Department of Internal Medicine, Hebei Medical University, Shijiazhuang, China; ^2^Department of Endocrinology, Hebei General Hospital, Shijiazhuang, China

## Abstract

**Background:**

This study is aimed at exploring the key genes and the possible mechanism of heart damage caused by obesity.

**Methods:**

We analyzed the GSE98226 dataset. Firstly, differentially expressed genes (DEGs) were identified in heart tissues of obese and normal mice. Then, we analyzed DEGs using Gene Ontology (GO) and Kyoto Encyclopedia of Genes and Genomes (KEGG) pathway enrichment analysis. Thirdly, we constructed a protein-protein interaction (PPI) network and key modules and searched hub genes. Finally, we observed the pathological changes associated with obesity through histopathology.

**Results:**

A total of 763 DEGs were discovered, including 629 upregulated and 134 downregulated genes. GO enrichment analysis showed that these DEGs were mainly related to the regulation of transcription, DNA-templated, nucleic acid binding, and metal ion binding. KEGG pathway analysis revealed that the DEGs were enriched in long-term depression, gap junction, and sphingolipid signaling pathways. Finally, we identified UTP14A, DKC1, DDX10, PinX1, and ESF1 as the hub genes. Histopathologic analysis showed that obesity increased the number of collagen fibers and decreased the number of microvessels and proliferation of the endothelium and increased endothelial cell damage which further leads to dysfunction of cardiac microcirculation.

**Conclusion:**

UTP14A, DKC1, DDX10, PinX1, and ESF1 have been identified as hub genes in obesity-induced pathological changes in the heart and may be involved in obesity-induced cardiac injury by affecting cardiac microcirculatory function.

## 1. Introduction

Obesity is a metabolic disease with excessive accumulation of body fat. It is an independent risk factor for cardiovascular disease, metabolic disease, and type 2 diabetes [1]. With the improvement of the quality of life, the number of obese people in the world is increasing, especially in developed countries [2]. BMI is often used to measure obesity, which is defined by the World Health Organization as having a BMI ≥ 30 [3]. Obesity has been identified as an independent risk factor for cardiovascular diseases in the context of increasing morbidity and mortality [4, 5]. The essence of obesity is the abnormal accumulation of lipids and the increase in inflammatory factors in the body, which will inevitably augment the workload and impair the function of the heart and lungs. In addition, inflammatory factors affect the metabolism of the heart, impairing cardiac function [6]. For some obese patients, sleep apnea syndrome, elevated blood pressure, and blood sugar are risk factors for heart damage [7].

The mechanisms underlying obesity-induced cardiac dysfunction have not been fully elucidated [8]. Previous studies showed that obesity causes alterations in cardiac energy metabolism in cardiomyocytes, particularly fatty acid metabolism [9, 10], and analysis has revealed that acyl-CoA synthetase long-chain family member 1 (ACSL-1) and glucose transporter member 4 (GLUT-4) may be the key proteins responsible for heart damage [11]. Other studies discovered that obesity contributes to a chronic inflammatory state in the body and interferes with a variety of metabolic processes [12]. In this context, metabolic disorders will inevitably lead to abnormal metabolism of the heart, as well as heart microcirculation dysfunction, which plays an important role in the occurrence and development of cardiovascular diseases [13, 14]. However, at present, the diagnosis and treatment of cardiac microcirculation are not mature.

Oxidative stress plays an important role in obesity and its complications and can interfere with angiogenesis, so oxidative stress, obesity, and angiogenesis are closely related. Angiogenesis is considered to be the most important change in the premorbid process of obesity [15]. Angiogenesis is a multistep process involved in the healing of damaged tissues, organ repair, and fetal development under physiological conditions, while under pathological conditions, it can promote the development of multiple cancers and multiple vascular complications [16, 17]. For the cardiovascular system, obesity significantly increases the likelihood of acute myocardial infarction and microcirculatory disturbance. Microcirculation dysfunction is one of the pathological changes of heart damage in obese patients, where the imbalance of the angiogenesis regulation mechanism plays a significant role.

In this study, we further revealed biomarkers of obesity-induced cardiac damage through the analysis of GSE98226 gene expression profiling and immunohistochemical analysis of cardiac tissue in obese mice. The identification of key genes and pathways is helpful to better understand the pathophysiological mechanisms of disease development, and it provides new ideas of cardiovascular protection for obese patients.

## 2. Materials and Methods

### 2.1. Microarray Data

The gene expression data utilized in this study (GSE98226) were downloaded from the NCBI Gene Expression Omnibus (GEO; http://www.ncbi.nlm.nih.gov/geo). The data was based on the GPL21163 Platform (Agilent-074809 SurePrint g 3mouse GE v 28x60k Microarray), and it came from heart tissues of three mice on a normal diet and three mice on a high-fat diet.

### 2.2. Screening of Differentially Expressed Genes

The GEO2R (https://www.ncbi.nlm.nih.gov/geo/geo2r/) online tool in the GEO database was applied to screen the differentially expressed genes (DEGs) in normal and hyperlipidemic mouse heart tissues. The determining criteria of the DEGs were adjusted *P* value < 0.01 and |log2foldchange(FC)| ≥ 2. The Benjamini and Hochberg (false discovery rate) method is used to calculate the adjusted *P* value. The study deleted gene probes that do not correspond to gene names and replaced them with averages for multiple probes for one gene name. After initial processing, we input the DEG data into Excel for further analysis.

### 2.3. GO and KEGG Pathway Enrichment Analysis of DEGs

The study applied an online Database for Annotation, Visualization and Integrated Discovery (DAVID) (https://david.ncifcrf.gov) for Gene Ontology (GO) and Kyoto Encyclopedia of Genes and Genomes (KEGG) pathway enrichment analysis. The data was collated, and the results were visualized using the online tool Bioinformatics (http://www.bioinformatics.com.cn/).

### 2.4. PPI Network Construction

We constructed a PPI network of DEGs through STRING (http://www.string-db.org), an online database, to determine protein-protein interactions. An interaction score that is equal to or greater than 0.7 is defined as having interaction between proteins, while the remaining options are set to default.

### 2.5. Module Analysis and Selection of Hub Genes

The PPI interaction map was imported into Cytoscape software, and the MCODE plug-in was used to extract and visualize major modules in the network. We screened the key genes in the network through three algorithms (Maximum Neighborhood Component (MNC), Density of Maximum Neighborhood Component (DMNC), and Maximal Clique Centrality (MCC)) in the plug-in CytoHubba.

### 2.6. Mice and Groups

Specific pathogen-free male C57BL/6 mice were purchased from Hebei Yiweiwo Biotechnology Co. Ltd. Six C57 mice were randomly divided into normal chow diet (NCD, *n* = 3) and high-fat diet (HFD, *n* = 3) after adaptive feeding for 1 week. The mice were fed for the next 24 weeks according to their groups, and their bodyweight was monitored weekly. We evaluated the results of obesity modeling by comparing the weight of mice in the high-fat group with that in the control group. The modeling would be considered successful if the body weight of mice in the high-fat group was 20% or higher than that in the control group. All mice were raised in a comfortable and sterile environment with specific conditions as follows: humidity 45%-65%, temperature 20-24°C, time ratio of night to day 1 : 1, adequate food and water supply, and bedding change twice a week. This experiment was subject to approval by the animal ethics association of Hebei General Hospital.

### 2.7. Preparation of Tissue Sample

The fully anesthetized and completed mice were placed on an icebox for blood collection. The chest cavity was opened, and the heart tissue was dissected. The blood vessels were cut off from the bottom of the heart, and the heart was harvested and flushed with saline. The excess blood was squeezed out with a filter paper and the surface fluid drained, and then, the heart tissue was fixed in 4% paraformaldehyde for later use.

### 2.8. HE and Masson Staining

Heart tissue soaked in paraformaldehyde was taken out and embedded in paraffin and sliced. Slices were placed in hematoxylin solution for 5 minutes, separated by 1% alcohol for 5 seconds, added 0.5% eosin for 30 seconds, and rinsed in running water. Then, dehydrate with 95% alcohol and 100% alcohol, respectively, for 5 minutes; then, soak in xylene and seal the tablets with neutral gum.

In Masson staining, conventional paraffin embedding and section treatment were performed. Sections were placed in hematoxylin for 5 minutes, Masson compound dyeing solution for 5 minutes, 1% phosphomolybdic acid for 5 minutes, 2% bright green dyeing solution for 5 minutes, and 1% phosphomolybdic acid for 5 minutes. Further treatment included alcohol dehydration, xylene treatment, and neutral gum sealing, and we observed the sections using a microscope.

### 2.9. Immunofluorescence Analysis

The study examined cardiac tissue using CD34 immunochemistry to determine the density of blood vessels in the heart. The heart tissue was sectioned after conventional treatment and incubated with the first and secondary antibodies. The vessel density was calculated after observing under a microscope.

In order to identify changes in the cardiovascular function of obese mice, the study measured the vWF factor of heart tissue. The expression of vWF was observed by the microscope after the heart tissues were sectioned and incubated with the first and secondary antibodies, respectively.

The study identified the proliferation of cardiac endothelium cells by double immunofluorescence staining and applied CD31 and Ki-67 dual staining to detect the proliferation of cardiac endothelium cells. All operations were performed in strict accordance with the manufacturer's instructions and images were evaluated using Image-Pro Plus 6.0 software.

### 2.10. Statistical Analysis

All data are presented as mean ± standard deviation. Data were analyzed using GraphPad software, and independent samples *t*-tests were performed. *P* < 0.05 means the result is statistically significant, and *P* < 0.01 means the result is extremely statistically significant.

## 3. Results

### 3.1. Identification of DEGs

**Figure 1:**
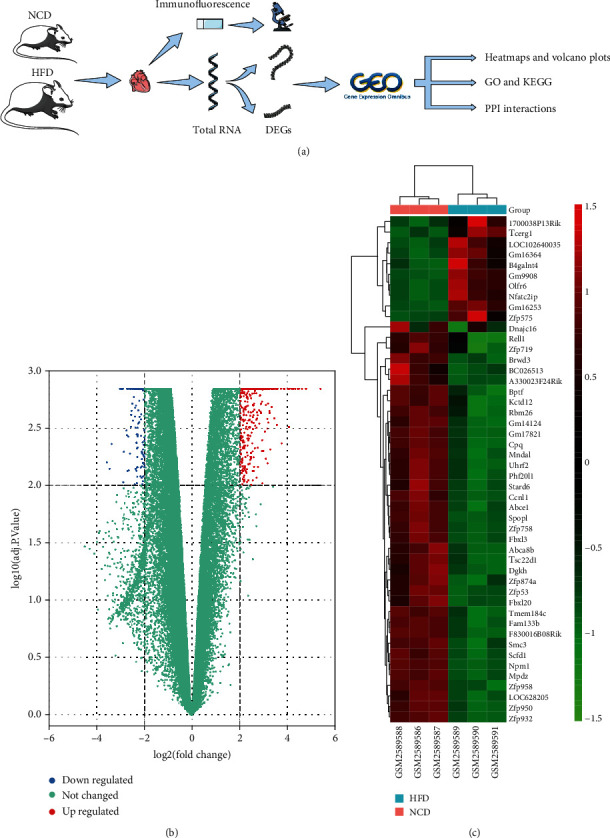
Screening and visualization of DEGs. (a) Experimental workflow. The hearts of NCD and HFD mice were harvested for DEGs analysis and pathological examination (*n* = 3). (b) The volcano plots of DEGs. The screening criteria for DEGs were adjusted *P* value < 0.01 and |log2foldchange(FC)| ≥ 2. (c) The heatmaps of DEGs. Top 50 DEGs between NCD and HFD. Red: greater expression; blue: less expression; DEGs: differentially expressed genes; NCD: normal chow diet; HFD: high-fat diet; GO: Gene Ontology; KEGG: Kyoto Encyclopedia of Genes and Genomes; PPI: protein-protein interaction.

A total of 763 DEGs were identified in the heart tissue of the high-fat mice, including 629 upregulated genes and 134 downregulated genes.  Figure 1 and Supplemental File 1 show the volcano plots of all DEGs and the heatmaps of the first 50 DEGs.

### 3.2. GO Enrichment Analysis

**Figure 2:**
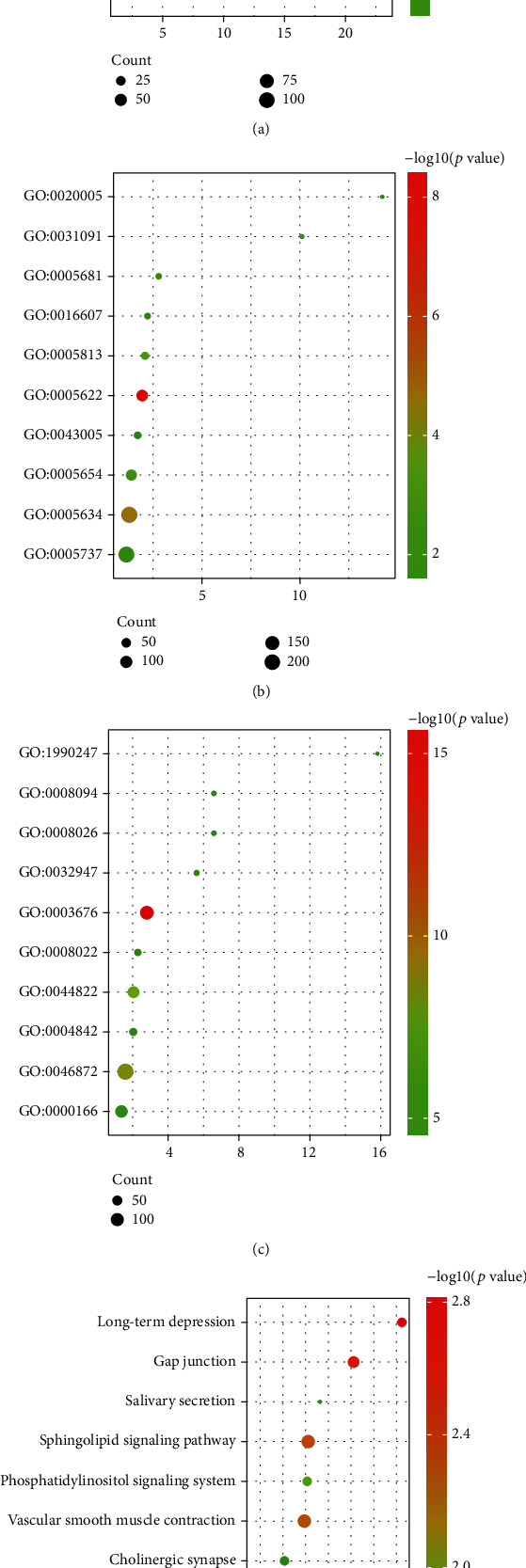
GO terms and KEGG pathway enrichment analysis. The *x*-axis represents the fold enrichment of each functional group gene. The *y*-axis represents the different functional groups. The size of the dots indicates the number of genes contained in different functional groups, and the color of the dots reflects the different range of −log10(*P* value). The larger the number of genes, the larger the dot. The gradient from green to red represents a change in *P* value from large to small. GO analysis divided DEGs into three functional groups: BP (a), CC (b), and MC (c). (d) Top 10 KEGG pathway enrichment analysis of DEGs. GO: Gene Ontology; KEGG: Kyoto Encyclopedia of Genes and Genomes; BP: biological processes; CC: cell composition; MF: molecular function; DEGs: differentially expressed genes.

This study conducted the GO enrichment analysis of DEGs based on the DAVID database and screened out enrichment terms with adjusted *P* value < 0.05. As shown in Figure 2 and Supplemental File 2, the result contained 60 enrichment terms. Biological processes (BP) were mainly enriched in regulation of transcription, DNA-templated, mRNA processing, and RNA splicing; cellular component (CC) was mainly enriched in intracellular, nucleus, and centrosome; molecular function (MF) was mainly enriched in nucleic acid binding, metal ion binding, and poly(A) RNA binding.

### 3.3. KEGG Pathway Analysis

Figure 2 shows the top 10 KEGG enrichment pathways of DEGs, and Supplemental File 3 shows all pathway information. Main pathways include long-term depression, gap junction, and sphingolipid signaling pathway.

### 3.4. PPI Network Construction and Module Analysis

**Figure 3:**
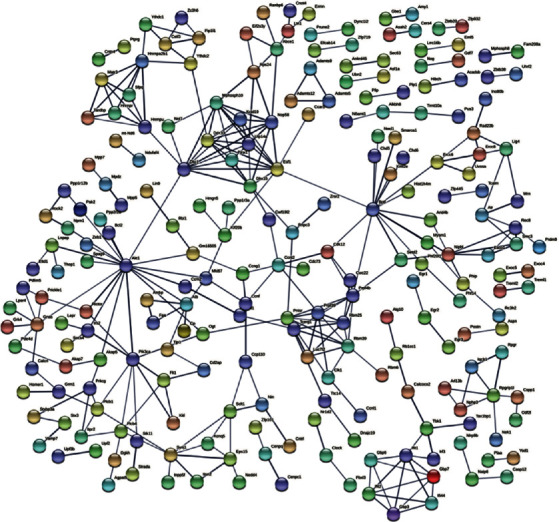
Results of PPI network analysis of DEGs. The circles represent genes; the lines represent the PPI between genes, and the images within the circles represent protein structures. The line color represents the PPI evidence level. PPI: protein-protein interaction; DEGs: differentially expressed genes.

The study constructed a PPI network to better understand the biological characteristics of DEGs.  Figure 3 shows the details of the PPI network and the corresponding modules. There are 584 nodes and 316 edges in this network. The three major modules were generated by importing the network into Cytoscape.

### 3.5. Hub Gene Selection and Analysis

**Figure 4:**
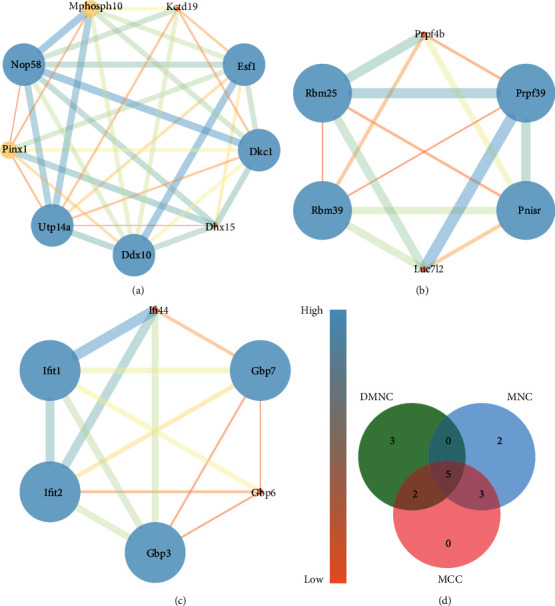
The protein-protein interaction module constructed based on the DEGs (a–c). The size of the circle and the thickness of the line represent the degree of importance; the larger the circle and the thicker the line, the more important it is. The color represents the degree value. Orange means the smallest degree value, while blue means the largest degree value. (d) Venn diagram of common hub genes based on three methods. DEGs: differentially expressed genes; DMNC: Density of Maximum Neighborhood Component; MCC: Maximal Clique Centrality; MNC: Maximum Neighborhood Component.

Based on the key module, we screened out hub genes by applying the cytoHubba plug-in from Cytoscape. Three algorithms, MCC, DNMC, and MNC, were used to identify the top 10 hub genes, and five hub genes were identified in obese mice compared to controls: UTP14A, Dkc1, DDX10, PinX1, and ESF1, as shown in Table 1 and Figure 4.

### 3.6. General Conditions of Obese Mice

**Figure 5:**
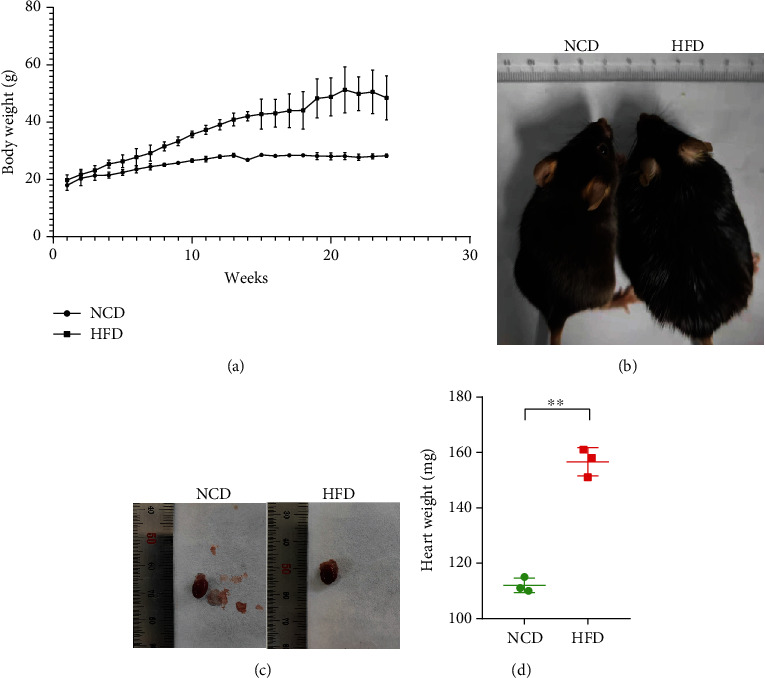
Effect of high-fat diet on mice. (a) Comparison of NCD and HFD mice. (b) Effect of high-fat diet on body weight of mice (*n* = 3). (c) A representative macroscopic image of the heart. (d) Comparison of heart weights between the two groups of mice (*n* = 3). Data were processed using independent samples *t*-test. ∗∗ represents *P* < 0.01. NCD: normal chow diet; HFD: high-fat diet.

After 24 weeks of feeding, the weight of mice in the high-fat group was significantly higher than that in the control group. Compared with the high-fat group, the control group had faster movements, smoother and darker hair, smaller hearts, and less epicardial fat. For the HFD group, mice's heart weight was significantly higher compared with that of the NCD group (*P* < 0.01). The results are shown in Figure 5.

### 3.7. Myocardial Histopathology

**Figure 6:**
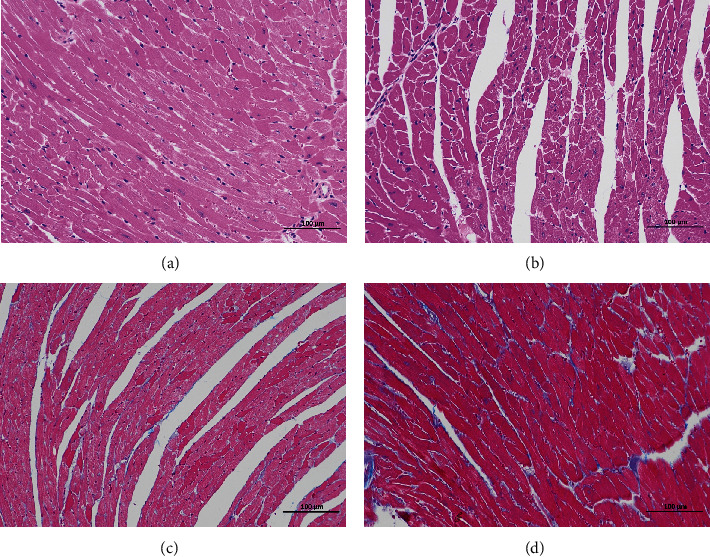
HE and Masson staining of rat myocardial tissue in each group (200x). HE staining of rat myocardial tissue in each group (a, b). Masson staining of rat myocardial tissue in each group (b, c). The left side of the graph represents the normal group, and the right side represents the high-fat group.

The results of HE staining indicated that the cardiomyocytes from the control group were arranged compactly and orderly with less extracellular matrix, while the cardiomyocytes from the obese group were enlarged and slightly disordered with more extracellular matrix. Masson staining showed that the collagenous tissue in the control group was slender and orderly distributed, and the collagenous tissue in the obese group was obviously increased, disarranged, and unevenly distributed, and inflammatory cells were found around the vessels, as shown in Figure 6.

### 3.8. Effects of Obesity on Cardiac Angiogenesis, Proliferation, and Function

**Figure 7:**
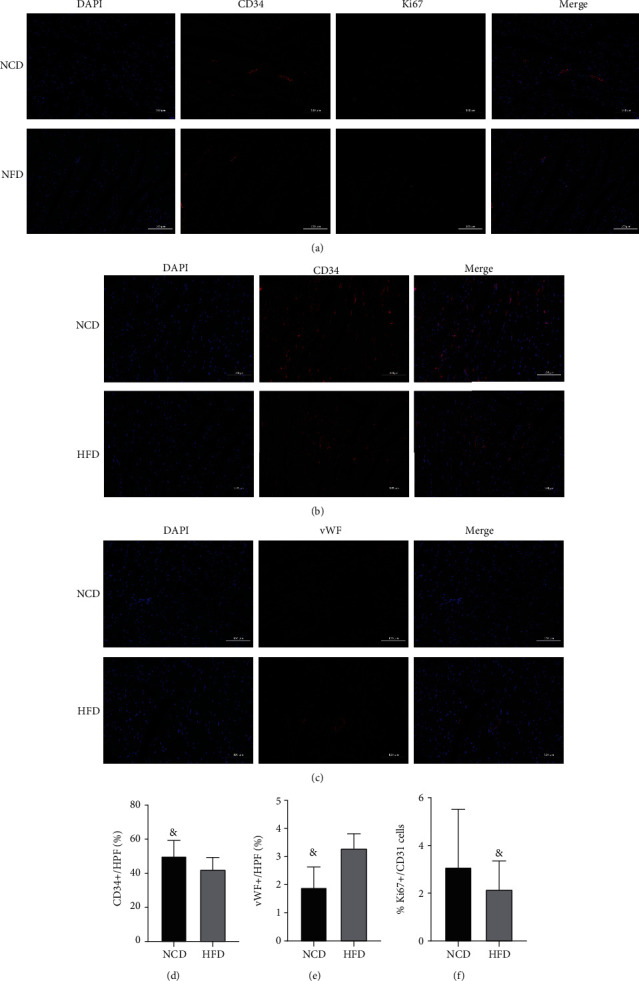
Comparison of cardiac angiogenesis, endothelial cell proliferation, and injury between NCD-heart and HFD-heart. (a) Immunofluorescent staining of Ki67 and CD31 to detect proliferative endothelial cells in NCD and HFD hearts; (b) CD34 immunofluorescence staining to determine the number of cardiac microvessels. (c) VWF immunofluorescence staining to determine the proportion of endothelial cell damage. (d) Comparison of CD34-positive cells per HPF between NCD and HFD hearts (^&^*P* > 0.05, *n* = 3). (e) Comparison of vWF-positive cells per HPF between NCD and HFD hearts (^&^*P* > 0.05, *n* = 3). (f) The comparison of the percentage of Ki67-positive cells between NCD and HFD hearts (^&^*P* > 0.05, *n* = 3). NCD: normal chow diet; HFD: high-fat diet; HPF: high-power field.

To determine the effect of obesity on cardiovascular angiogenesis, we measured the capillary density of mouse heart tissue. Compared to the control group, the cardiac capillary density was slightly decreased in obese mice, but not statistically significant (*P* > 0.05). Subsequently, we observed a slight increase in vWF factor expression in the hearts of obese mice. Double immunofluorescence staining showed that the proliferation capacity of endothelial cells in the hearts of obese mice was decreased compared with the control group, but there was no statistical difference between the two groups (*P* > 0.05), as shown in Figure 7. The expression levels of UTP14A, DKC1, DDX10, PinX1, and ESF1 were significantly associated with impaired cardiac angiogenesis (Supplemental File 4).

## 4. Discussion

Cardiovascular disease is the main cause of death worldwide, and its prevalence continues to increase. The main risk factors include dyslipidemia, insulin resistance, and type 2 diabetes. Obesity is closely linked to these risk factors [18, 19]. As a result, obese people are more apt to get cardiovascular disease and have higher mortality rates than people of normal weight. For obese patients, abnormal increases in adipose tissues can increase the release of cytokines and bioactive mediators, such as leptin, IL-6, and TNF, which can activate the inflammatory mechanism and impair the normal metabolism of the heart [20]. Cardiac microcirculation plays an important role in cardiac metabolism, and the endothelium is the major cell type in microcirculation. Therefore, when the microcirculation is in a state of low inflammation caused by obesity-related insulin resistance and cytokines released by fat cells, it inevitably leads to endothelial dysfunction [21, 22]. In this study, we demonstrated the effect of obesity on cardiac microcirculation by immunocytochemistry. The results showed that obesity can lead to vascular endothelial dysfunction, increase cell damage, and reduce cell proliferation.

Angiogenesis is essential for the maintenance of normal physiological functions. Numerous studies have shown that obesity promotes angiogenesis in various tumor and adipose tissues [23–25]. However, the pathophysiology of cardiac microvasculature in the obese state is still worth exploring. Studies have shown that cardiac microvessel density in obese mice is significantly higher than that in normal mice, but it decreases as the disease progresses. Another study of human heart tissue showed that obesity significantly reduced the number of microvessels [26–28]. In this study, the results of pathological examination showed that obesity can increase the diameter of myocardial cells, increase collagen fibers, and decrease the number of microvessels.

To determine the genetic mechanisms underlying the effects of obesity on cardiac microcirculation, we analyzed GSE98266 data and found 763 DEGs, including 629 upregulated genes and 134 downregulated genes. GO enrichment analysis showed that these DEGs were mainly associated with the regulation of transcription, DNA-templated, nucleic acid binding, and metal ion binding. KEGG pathway analysis revealed that the DEGs were enriched in long-term depression, gap junction, and sphingolipid signaling pathway. Finally, we identified UTP14A, DKC1, DDX10, PinX1, and ESF1 as the hub genes between the two groups.

U Three Protein 14A (UTP14A) is a small nucleolar RNA related to protein 14 homologue of U 3, belonging to the UTP14 family, and plays a key role in the synthesis of ribosomes and 18s rRNA [29]. In a pathological state, UTP14A plays its role mainly by promoting angiogenesis. It has been noted that UTP14A is involved in the development of colorectal cancer by promoting angiogenesis, while the inhibition of UTP14A can improve the prognosis of patients [30, 31]. The upregulation of UTP14A was associated with the progression of esophageal cancer [32]. Similarly, PIN2/TRF1-interacting telomerase inhibitor 1 (PinX1) is involved in the development of cancer through angiogenesis [33]. In this study, UTP14A and PinX1 were identified as key genes in the heart of obese mice, which may be involved in the pathophysiology of obesity-related cardiac effects by regulating the balance of cardiac microvasculature, but there is no relevant research to verify this.

The dyskeratosis congenita 1 (DKC1) gene, located on the X chromosome xq28, was first discovered because of a mutation that causes dyskeratosis congenita [34], as well as a variety of cancers and pulmonary fibrosis [35, 36]. Studies have shown that DKC1 can be involved in cancer progression by promoting angiogenesis and is closely related to oxidative stress [37, 38]. DEAD/H box RNA helicase 10 (DDX10) is associated with a variety of cancers and diseases of the blood system [39, 40]. However, little research has been done on ESF1, and its role is still unclear.

This study found the key genes and related pathways of obesity-induced heart injury through gene chip. It is worth noting that UTP14A, DKC1, and PinX1 are all closely associated with angiogenesis in various diseases, so we speculate that all three may affect cardiac angiogenesis in obesity, but their angiogenic roles in cardiac tissue remain unclear. Moreover, we proved that obesity can increase the size of myocardial cells, increase the number of collagen fibers in the myocardial matrix, and decrease the number of microvessels in the heart and reduce endothelium proliferation which can affect the microcirculation of the heart, leading to impairment of cardiac function. This still needs further experimental verification.

## 5. Conclusions

UTP14A, DKC1, DDX10, PinX1, and ESF1 may be involved in obesity-induced cardiac injury by affecting angiogenesis in the heart.

## Figures and Tables

**Table 1:** Hub genes based on cytoHubba.

Projects	Methods in cytoHubba		
	DMNC	MNC	MCC
Top 10 gene symbols	**Utp14a**	**Utp14a**	**Utp14a**
	**Dkc1**	**Dkc1**	**Dkc1**
	**Ddx10**	**Ddx10**	**Ddx10**
	**Esf1**	**Esf1**	**Esf1**
	**Pinx1**	Nop58	**Pinx1**
	Dhx15	**Pinx1**	Dhx15
	Kctd19	Mphosph10	Mphosph10
	Ifit1	Rbm39	Nop58
	Gbp3	Rbm25	Kctd19
	Gbp7	Prpf4b	Rbm39

Bold gene symbols were the overlap hub gene. MCC: Maximal Clique Centrality; DMNC: Density of Maximum Neighborhood Component; MNC: Maximum Neighborhood Component.

## Data Availability

The data used to support the results of this study are available from the first author upon request.

## Supplementary Materials

Supplementary Materials GSE98226 datasets were downloaded from GEO (http://www.ncbi.nlm.nih.gov/geo/). Supplemental File 1: DEGs. Supplemental File 2: GO enrichment analysis. Supplemental File 3: KEGG pathway analysis. Supplemental File 4: correlation between UTP14A, DKC1, DDX10, PinX1, and ESF1 expression and cardiac angiogenesis.
