# Supplementary material for: UTP14A, DKC1, DDX10, PinX1, and ESF1 Modulate Cardiac Angiogenesis Leading to Obesity-Induced Cardiac Injury
Source: J Diabetes Res. 2022 Jun 13;2022:2923291. doi: 10.1155/2022/2923291 (PMC9208995; doi:10.1155/2022/2923291)
Supplement: Supplementary Materials — GSE98226 datasets were downloaded from GEO (http://www.ncbi.nlm.nih.gov/geo/). Supplemental File 1: DEGs. Supplemental File 2: GO enrichment analysis. Supplemental File 3: KEGG pathway analysis. Supplemental File 4: correlation between UTP14A, DKC1, DDX10, PinX1, and ESF1 expression and cardiac angiogenesis. [file 2923291.f1.zip › Supplemental File 1 (1).docx]

Supplemental File 1: Differentially expressed gene results

| GENE_SYMBOL | adj.P.Val | logFC |
| --- | --- | --- |
| 4930442B02Rik | 0.00144 | -1.25118919 |
| Avil | 0.02161 | 1.53091483 |
| Hpse2 | 0.00145 | -1.22193674 |
| Cldn34b4 | 0.00144 | -1.30017714 |
| 4930429P21Rik | 0.04472 | -1.01963123 |
| Mid1 | 0.25062 | -0.42706053 |
| Gm30414 | 0.00144 | -1.28116048 |
| Gm36642 | 0.00145 | -1.2388121 |
| Ppp2r3d | 0.00144 | -1.23579703 |
| Casp3 | 0.89046 | 0.06421503 |
| Ube2s | 0.15655 | -0.50861974 |
| Hoxb9 | 0.00144 | -1.25927895 |
| Rfc3 | 0.89741 | 0.02440075 |
| Pla2g4d | 0.00158 | -1.22853957 |
| Cox17 | 0.01599 | -0.65487978 |
| Itga8 | 0.29118 | -0.43895549 |
| Tns3 | 0.09675 | -1.38146573 |
| Fbxo47 | 0.62739 | -0.29803229 |
| Gm39939 | 0.42009 | -0.33225202 |
| Dad1 | 0.42879 | -0.32252195 |
| Inafm1 | 0.03111 | -0.41476222 |
| Gm6040 | 0.81167 | 0.08965579 |
| Gm17250 | 0.72903 | 0.18715454 |
| Gm34166 | 0.05812 | -0.65295496 |
| Lyg2 | 0.00144 | -1.24811894 |
| LOC102634502 | 0.0034 | -1.50533838 |
| Vmn1r229 | 0.00144 | -1.29051721 |
| AU016765 | 0.0024 | -1.60838479 |
| LOC102633724 | 0.03605 | -2.04698198 |
| 1700097N02Rik | 0.07419 | -0.74138821 |
| Slamf6 | 0.00164 | -1.29347279 |
| Gm11110 | 0.1248 | -0.79743914 |
| Cacng4 | 0.03995 | -0.98967077 |
| Chrna6 | 0.34501 | -0.39535256 |
| Vmn1r64 | 0.00144 | -1.29051721 |
| Macrod2os1 | 0.00462 | -1.33841592 |
| Gm10584 | 0.14041 | -0.88885876 |
| 8030443G20Rik | 0.05778 | -1.59281408 |
| 9130015L21Rik | 0.00144 | -1.20555712 |
| Cd9 | 0.15666 | -0.26969195 |
| Ino80d | 0.00158 | -1.2446827 |
| 9430083A17Rik | 0.04668 | -0.6309349 |
| Azin2 | 0.00608 | -1.14536307 |
| 4930447I22Rik | 0.00144 | -1.30713006 |
| 4930402C01Rik | 0.27711 | 0.28370491 |
| D030018L15Rik | 0.00158 | -1.17515383 |
| Gm10445 | 0.00144 | -1.28898079 |
| Rps10 | 0.00144 | -1.34640742 |
| Rpl31 | 0.00193 | -0.81790963 |
| Gm6403 | 0.00144 | 1.19283869 |
| LOC105244085 | 0.07897 | -1.9956082 |
| F730016J06Rik | 0.02613 | -0.99601094 |
| Olfr787 | 0.69539 | -0.13768938 |
| 4933400L20Rik | 0.00144 | -1.37964577 |
| Plxdc1 | 0.04234 | -0.51244114 |
| Psmd3 | 0.9611 | 0.03011255 |
| D930030D11Rik | 0.00145 | -1.22376446 |
| Gm32282 | 0.01288 | -0.87427545 |
| Clec4b2 | 0.03631 | -0.98251086 |
| Sdk1 | 0.05135 | -0.58072813 |
| A130006I12Rik | 0.23712 | -0.62586779 |
| Rps19 | 0.00144 | -1.17437787 |
| Akp3 | 0.08614 | -0.825383 |
| 6530403H02Rik | 0.80592 | -0.35443523 |
| Gm11981 | 0.02732 | -1.26219879 |
| Ccdc97 | 0.00853 | -1.00769151 |
| F730048M01Rik | 0.00758 | -1.09996726 |
| Selk | 0.78914 | 0.05639218 |
| Gm11690 | 0.02137 | -0.90596085 |
| A630076J17Rik | 0.66988 | -0.18116384 |
| 1700034K08Rik | 0.3562 | -0.31479205 |
| Aff3 | 0.50629 | 0.124674 |
| Gm17762 | 0.87917 | -0.07514338 |
| Gm15825 | 0.00144 | -1.40202659 |
| Krtap31-2 | 0.54516 | -0.32238639 |
| Lhpp | 0.2467 | -0.77627458 |
| Tia1 | 0.98436 | 0.0096241 |
| E130111B04Rik | 0.00929 | -1.0433256 |
| Gm16876 | 0.02613 | -0.99601094 |
| 1700027I24Rik | 0.00144 | -1.24613586 |
| Gm8364 | 0.00273 | -1.40433124 |
| 7420701I03Rik | 0.01686 | -1.32057938 |
| Cuta | 0.01751 | -0.79737308 |
| Map1a | 0.00571 | -1.05580988 |
| Plscr3 | 0.00372 | -0.78308875 |
| Smyd2 | 0.62243 | 0.22493444 |
| D430018E03Rik | 0.64013 | 0.1912087 |
| Adora1 | 0.00144 | -1.29864962 |
| Stx12 | 0.49616 | -0.1777597 |
| Poc5 | 0.03947 | -0.42257712 |
| 9930111H07Rik | 0.49625 | -0.30404241 |
| Gm34868 | 0.23742 | -0.5784271 |
| Nptx2 | 0.00145 | -1.2879918 |
| Dclre1c | 0.05542 | 0.83486848 |
| Mbnl3 | 0.74687 | -0.21107256 |
| Clec2i | 0.36859 | -0.65152696 |
| Wdr76 | 0.20552 | -0.78728582 |
| Ddx23 | 0.00489 | -1.19415878 |
| Lsp1 | 0.84148 | -0.05692293 |
| Wtap | 0.79768 | -0.04085136 |
| 4930594M17Rik | 0.0037 | -1.11214255 |
| Gm41669 | 0.24615 | -0.75582677 |
| A230108P19Rik | 0.00144 | -2.90214244 |
| Vmn1r223 | 0.00144 | -1.26560808 |
| Kmt2a | 0.65324 | 0.41170947 |
| LOC102633032 | 0.00448 | -0.99815121 |
| 1810024B03Rik | 0.48896 | 0.26908953 |
| Atp5a1 | 0.4007 | 0.47057091 |
| Olfr120 | 0.01239 | -1.02834537 |
| D430001F17Rik | 0.81474 | 0.10901276 |
| Rgs9 | 0.02742 | 0.67475587 |
| Gm32688 | 0.01344 | -0.9146277 |
| 1700041A01Rik | 0.00288 | -1.09711326 |
| Wdr64 | 0.00144 | -1.60728483 |
| Gm38733 | 0.05684 | -0.4037605 |
| 4833418N17Rik | 0.00502 | -1.46570614 |
| Acadsb | 0.00277 | -0.74106541 |
| 4930443O20Rik | 0.56998 | -0.35172388 |
| A830011I04 | 0.01789 | -1.65633744 |
| Muc19 | 0.00144 | -1.29085005 |
| Itga4 | 0.02951 | -1.63395827 |
| C030037F17Rik | 0.00144 | -1.20579466 |
| Tmem2 | 0.0629 | 0.65488815 |
| 5031410I06Rik | 0.11403 | -0.73596428 |
| 2010001A14Rik | 0.0188 | -0.94673677 |
| A930002C04Rik | 0.01874 | 0.98732073 |
| Ppp2r2cos | 0.10104 | -1.09018341 |
| Gm34278 | 0.75594 | -0.17598026 |
| Gm39463 | 0.10568 | -1.07275646 |
| A930017M01Rik | 0.04616 | -1.00148197 |
| Brpf1 | 0.47817 | -0.21931193 |
| Cacna1b | 0.03832 | -0.49813985 |
| LOC105246765 | 0.00144 | -1.30713006 |
| 1700027H10Rik | 0.00145 | -1.26219879 |
| 1700109I08Rik | 0.0046 | -1.56403079 |
| 6030466F02Rik | 0.94271 | 0.02632381 |
| Brd2 | 0.01812 | -1.84877989 |
| Wfdc9 | 0.00209 | -1.37685025 |
| Gm7894 | 0.00441 | -1.32611356 |
| Olfr750 | 0.02314 | -0.63200076 |
| Gm34029 | 0.00144 | -1.29085005 |
| Thop1 | 0.03447 | -0.44497724 |
| Trip12 | 0.00696 | -0.65586325 |
| E030019B13Rik | 0.00144 | -1.29085005 |
| 1700011C11Rik | 0.15468 | -0.55834595 |
| BC037704 | 0.46522 | -0.13800623 |
| Olfr119 | 0.00144 | -1.27165604 |
| Gpr137b | 0.13355 | -0.29549205 |
| Gm12404 | 0.00145 | -1.20627532 |
| 4930539M17Rik | 0.00144 | -1.28116048 |
| Dcpp1 | 0.06599 | -1.68504575 |
| Tbc1d16 | 0.14888 | -0.47161637 |
| Greb1l | 0.04631 | 0.70702374 |
| Gm17821 | 0.00144 | 2.79669609 |
| Gm15478 | 0.17639 | -0.75676341 |
| Crispld1 | 0.00144 | 1.55586514 |
| Eps8l1 | 0.08584 | -0.87391275 |
| Ccdc62 | 0.02032 | 0.76973276 |
| Gm38692 | 0.00144 | -1.30017714 |
| 4930599A14Rik | 0.03861 | -2.04731648 |
| Ppap2b | 0.00998 | 0.5723867 |
| C130063K03Rik | 0.31411 | -0.6800006 |
| 4930504C09Rik | 0.00144 | -1.29746368 |
| Mkln1os | 0.91873 | 0.08425291 |
| Tspan32 | 0.23234 | -0.70324341 |
| Wnt4 | 0.0102 | -1.22464953 |
| Slc25a53 | 0.00144 | -1.30626366 |
| A630028G03Rik | 0.00144 | -1.26934956 |
| Zbp1 | 0.01256 | -0.79382684 |
| 4833417C18Rik | 0.34936 | -0.42822987 |
| Cct5 | 0.1755 | 0.35994852 |
| Tmem229a | 0.01598 | -1.60604492 |
| Gm5 | 0.00929 | -1.0433256 |
| LOC102641805 | 0.31908 | 0.46922933 |
| Zfp444 | 0.72457 | 0.14363782 |
| Vwa3a | 0.0278 | 0.89108458 |
| Pla2g4c | 0.00144 | -1.26560808 |
| Lhfpl1 | 0.00144 | -1.08541217 |
| Barhl2 | 0.00144 | -1.48564159 |
| Fcor | 0.00761 | -1.15951348 |
| Mtx1 | 0.39176 | -0.35364595 |
| Stfa2 | 0.29222 | 0.81746999 |
| Gm12409 | 0.53169 | 0.24531467 |
| Gm11734 | 0.00144 | 1.10137462 |
| Ralbp1 | 0.03112 | -0.76899665 |
| Helz | 0.04853 | -0.36207108 |
| Cpq | 0.00144 | 2.36246272 |
| Pcsk2os2 | 0.90846 | 0.05863225 |
| Ppt2 | 0.65329 | -0.2393648 |
| Igfbp6 | 0.00145 | -1.02454633 |
| Gm19359 | 0.64952 | -0.3676036 |
| Gm19866 | 0.00144 | -1.29864962 |
| C330006A16Rik | 0.00158 | -1.08970239 |
| Taf1b | 0.00409 | 0.78535923 |
| Mia3 | 0.02218 | 0.75761413 |
| Sf1 | 0.00422 | -1.46494571 |
| Tmem123 | 0.05338 | -0.37033352 |
| Mtmr12 | 0.00299 | -1.41159044 |
| Tdpoz1 | 0.07764 | -0.91095373 |
| 2010106C02Rik | 0.38249 | -0.37708151 |
| Mei1 | 0.00144 | -1.28116048 |
| Gm3279 | 0.00144 | -1.26560808 |
| Platr27 | 0.00144 | -1.26560808 |
| Ap1s1 | 0.04946 | -0.6092589 |
| Prkag3 | 0.00145 | -1.23937365 |
| Gm26641 | 0.06731 | 0.82717798 |
| Nadsyn1 | 0.51633 | -0.13415273 |
| Prlr | 0.51241 | 0.14283121 |
| Gm15614 | 0.01471 | 1.391988 |
| Minpp1 | 0.01933 | 1.49330983 |
| Lrp4 | 0.05093 | -0.70276174 |
| Dctn4 | 0.00264 | -0.85731002 |
| Cog1 | 0.07379 | -0.39738913 |
| Bmp2k | 0.1259 | -1.1607575 |
| Tomm40 | 0.07768 | -0.4668851 |
| Adgrl4 | 0.05793 | 0.39051516 |
| Itgb8 | 0.00224 | 1.38178707 |
| Cbfa2t2 | 0.59229 | 0.2253781 |
| 1700081B01Rik | 0.03945 | -0.75987879 |
| Gm30539 | 0.02078 | -1.15821745 |
| Epb4.1l4aos | 0.00636 | -0.64050834 |
| Kctd3 | 0.42613 | 0.15530591 |
| 6330576A10Rik | 0.00144 | -1.29085005 |
| 4930555O08Rik | 0.06217 | -0.8418205 |
| Tmem55b | 0.00976 | -0.63600159 |
| Slc6a8 | 0.12192 | -0.66592078 |
| Dennd1c | 0.05314 | -0.5196334 |
| 4930515B02Rik | 0.42681 | -0.25040158 |
| LOC101056015 | 0.01152 | -2.05953097 |
| Mroh4 | 0.70896 | -0.08982022 |
| E430016F16Rik | 0.22756 | -0.30593313 |
| Safb | 0.0216 | -0.4584627 |
| Stmn2 | 0.00597 | -0.78006936 |
| Hras | 0.00144 | -1.11734623 |
| Dtymk | 0.84838 | -0.19453503 |
| 5830444B04Rik | 0.57559 | -0.21739128 |
| Zkscan16 | 0.0025 | -1.68549172 |
| Otof | 0.00144 | -1.24811894 |
| Primpol | 0.00642 | -1.55898609 |
| Mrgpra9 | 0.00145 | -1.2388121 |
| Mroh3 | 0.04597 | -1.18067012 |
| Ftsj3 | 0.03805 | 0.46984773 |
| E130006D01Rik | 0.45137 | -0.49940951 |
| Vmn1r177 | 0.00145 | -1.22376446 |
| Pmpcb | 0.98682 | -0.00428786 |
| Mov10 | 0.0111 | -0.79112428 |
| Gm10125 | 0.73274 | -0.08681419 |
| Pstpip2 | 0.08821 | 0.47235222 |
| Efcab9 | 0.00598 | -1.22199969 |
| Ppp2r5a | 0.08517 | 0.49881554 |
| Gm31493 | 0.11133 | -0.80691329 |
| Gsdmc2 | 0.01514 | -1.05083598 |
| Npw | 0.00144 | -1.67293753 |
| Cdr1 | 0.00144 | -1.31884045 |
| Gclm | 0.23807 | -0.25490157 |
| Gm35884 | 0.00144 | -1.31495282 |
| Gm9908 | 0.00144 | -2.43330709 |
| Syk | 0.00162 | -0.88729922 |
| Kif24 | 0.02468 | -0.63190183 |
| Lox | 0.11459 | 0.59239726 |
| Slc45a2 | 0.00541 | -1.24095692 |
| Gm21269 | 0.00144 | -1.29864962 |
| A930031H19Rik | 0.00144 | -1.3480823 |
| Fyn | 0.01111 | 0.69575949 |
| Cfb | 0.01955 | -1.18769677 |
| Sp8 | 0.00144 | -1.30017714 |
| Prdm14 | 0.00966 | 1.00319785 |
| Nhsl1 | 0.00858 | -0.9100191 |
| Sirpb1a | 0.03339 | -1.00077005 |
| Apol7d | 0.00218 | -1.18878227 |
| Spata7 | 0.00144 | 1.29679637 |
| Marf1 | 0.69355 | 0.06911598 |
| 4930529K09Rik | 0.00144 | -1.20555712 |
| Atp2b3 | 0.22722 | -0.55471651 |
| Rsph14 | 0.00257 | -1.1333102 |
| Cit | 0.00208 | -1.15498993 |
| Glis3 | 0.00158 | -1.2446827 |
| Gm16793 | 0.00144 | 1.50372642 |
| 1700061J23Rik | 0.13368 | -0.74957552 |
| Alkbh6 | 0.27768 | -0.57564954 |
| Gm10544 | 0.00144 | -1.28116048 |
| Trappc10 | 0.52657 | -0.17406022 |
| LOC102636254 | 0.00145 | -1.26219879 |
| Gm16553 | 0.00144 | -1.24353547 |
| LOC105244530 | 0.10791 | -0.56193098 |
| Gm15767 | 0.06498 | -1.10326942 |
| 2210420H20Rik | 0.98049 | -0.00830566 |
| Whamm | 0.0222 | -0.75839292 |
| Mrpl48 | 0.05198 | 0.80172143 |
| Olfr1390 | 0.00158 | -1.2446827 |
| Ehd4 | 0.06635 | -0.31930925 |
| Olfr775 | 0.01056 | -1.24865146 |
| Mir142hg | 0.64854 | -0.1255253 |
| Gm39168 | 0.00188 | 1.45185148 |
| 1700100L14Rik | 0.00144 | -1.33632959 |
| Gm13974 | 0.004 | -1.5298028 |
| 1810021B22Rik | 0.04994 | -0.72493416 |
| Tmem136 | 0.23317 | -0.44451011 |
| Gm12326 | 0.01268 | -1.91763571 |
| 4833419G08Rik | 0.8883 | -0.06734793 |
| Mtf2 | 0.00234 | 0.76178474 |
| Hmces | 0.40265 | -0.14356594 |
| Gm33374 | 0.00279 | -1.43192856 |
| Gm31410 | 0.00144 | -1.28116048 |
| Slc7a4 | 0.08885 | -0.51438796 |
| Gm17116 | 0.96887 | -0.02356362 |
| Gm11563 | 0.00144 | -1.31884045 |
| Setd1a | 0.96656 | 0.01067013 |
| Sprr2h | 0.00144 | -1.28898079 |
| Gm11738 | 0.00144 | -1.58869852 |
| Rab2a | 0.00298 | 0.85150179 |
| Abca8b | 0.00144 | 2.93369244 |
| Veph1 | 0.03797 | -1.33216463 |
| 1700017N19Rik | 0.03586 | -3.22697402 |
| Mtg1 | 0.05651 | -1.23332981 |
| 4930421P07Rik | 0.06042 | 0.90246416 |
| Cdh13 | 0.03062 | 1.00106748 |
| Stx1b | 0.37421 | -0.66666667 |
| Gm40617 | 0.01237 | -0.92161086 |
| Clec4b1 | 0.97117 | -0.03409331 |
| 2810449D17Rik | 0.30088 | -0.22019543 |
| Cd101 | 0.85602 | -0.0353404 |
| Uba52 | 0.7881 | 0.11306468 |
| Gm36149 | 0.59775 | 0.19634427 |
| C030029H02Rik | 0.00215 | -1.59576966 |
| Htra1 | 0.14353 | -0.36792773 |
| Myh7b | 0.06459 | -0.34213573 |
| 1700110K17Rik | 0.03405 | -2.16946161 |
| Wdr12 | 0.59225 | -0.20702967 |
| Acox2 | 0.91698 | 0.02470189 |
| Gm9891 | 0.00144 | -1.29085005 |
| Osbpl1a | 0.01166 | -1.51787518 |
| Znrd1 | 0.06841 | -0.42698872 |
| Gm26844 | 0.00726 | -1.43411591 |
| L1cam | 0.00466 | -1.44318043 |
| Ykt6 | 0.05408 | -1.38039258 |
| Rdh5 | 0.09396 | -0.30652044 |
| Plcb2 | 0.00158 | -0.91233277 |
| Gm15245 | 0.04989 | -0.39646245 |
| Fbxw5 | 0.7393 | 0.27444209 |
| B230319C09Rik | 0.42563 | 0.47043904 |
| 1110058D11Rik | 0.04833 | -1.49146285 |
| Sun1 | 0.07003 | -0.48699308 |
| Selo | 0.1809 | -0.61676801 |
| Gm34534 | 0.03514 | -0.54463209 |
| Gm609 | 0.00834 | -1.47796612 |
| 6030407O03Rik | 0.70824 | 0.12267834 |
| Dock2 | 0.82344 | -0.08033392 |
| 4930512M02Rik | 0.02827 | -1.01421764 |
| Olfr872 | 0.00286 | -1.71624359 |
| Rhox10 | 0.00144 | -1.31495282 |
| Gm26761 | 0.04778 | -1.4243395 |
| Dnajc16 | 0.00144 | -2.93028893 |
| Scgb2b23-ps | 0.00145 | -1.22193674 |
| Gm4107 | 0.15428 | 0.60254798 |
| Magea5 | 0.00144 | -1.27699683 |
| Tmem164 | 0.0029 | -1.20027811 |
| Mthfsd | 0.49152 | -0.20452536 |
| LOC102639987 | 0.48738 | -0.25476207 |
| Alg3 | 0.78041 | 0.18436821 |
| Scarb1 | 0.00611 | -0.80072042 |
| Nudt15 | 0.03843 | 0.83378875 |
| Xirp2 | 0.38044 | 0.20691212 |
| Thyn1 | 0.4653 | 0.33057602 |
| Pnp | 0.38557 | -0.14996919 |
| Scml1 | 0.09999 | -1.27142478 |
| Gm39214 | 0.01817 | -0.71120729 |
| Ntm | 0.36587 | 0.33945729 |
| Hottip | 0.29887 | -0.26728091 |
| Grm7 | 0.00144 | -1.31884045 |
| Eef2kmt | 0.24359 | -0.23098587 |
| Dxo | 0.02205 | -0.52216899 |
| Esr1 | 0.00479 | -1.73535992 |
| Gm16130 | 0.00144 | -1.30017714 |
| Ceacam16 | 0.00144 | -1.28898079 |
| Dsc3 | 0.04189 | -1.09663077 |
| Gm9917 | 0.00229 | -1.18115647 |
| Cacna2d1 | 0.002 | -0.80923624 |
| Gfm1 | 0.12433 | 0.40035117 |
| Gm12298 | 0.00829 | -0.69090435 |
| Gm41148 | 0.00145 | -1.18741414 |
| Igsf9b | 0.04482 | -0.50864434 |
| 3110009F21Rik | 0.00739 | -1.94288848 |
| Fgd6 | 0.00747 | 0.83041313 |
| LOC102638199 | 0.91085 | -0.15612158 |
| C230091D08Rik | 0.13541 | 0.49117898 |
| Gm4710 | 0.00472 | -0.95770123 |
| Gm9883 | 0.16547 | 0.39599121 |
| B130006D01Rik | 0.18281 | 0.52013112 |
| Gm5524 | 0.09459 | -1.26165185 |
| Gm34471 | 0.00641 | -1.77951002 |
| Pear1 | 0.02031 | -0.5298984 |
| 4930467K11Rik | 0.00144 | -1.25118919 |
| Tctn2 | 0.05775 | -0.49403161 |
| Gadl1 | 0.00144 | -1.30713006 |
| Gm36159 | 0.00144 | -1.28898079 |
| Rbm3os | 0.03983 | -0.64768637 |
| Gm9054 | 0.02785 | -0.92312327 |
| Gm39121 | 0.27164 | -0.4511859 |
| A930038B10Rik | 0.00145 | -1.22376446 |
| Tlr1 | 0.27189 | -0.9530667 |
| Otud7b | 0.03029 | -0.50945155 |
| Gm6328 | 0.02158 | -0.95671402 |
| Clec16a | 0.00144 | -1.29085005 |
| Gm13982 | 0.59021 | -0.36797081 |
| D130079A08Rik | 0.00144 | -1.29746368 |
| Hoxaas2 | 0.53841 | 0.35065708 |
| Agmo | 0.8193 | 0.04143857 |
| Gpr161 | 0.42139 | 0.28165428 |
| Gm37041 | 0.00144 | -1.29085005 |
| Gm36101 | 0.15964 | -3.46018552 |
| Rem1 | 0.30668 | 0.18585576 |
| Ccdc17 | 0.41141 | 0.13614291 |
| 4831440E17Rik | 0.02215 | 1.71785193 |
| 3830432H09Rik | 0.0475 | -0.92312902 |
| Eomes | 0.03965 | -0.81835739 |
| 4930538E20Rik | 0.00144 | -1.28116048 |
| LOC105246632 | 0.00769 | -1.64070378 |
| Lrp2 | 0.00144 | -1.65909331 |
| 9030204H09Rik | 0.08156 | -0.34892722 |
| Pnpla1 | 0.00144 | -1.39552011 |
| Mpp1 | 0.53719 | -0.18069937 |
| Tbc1d8 | 0.03848 | -0.74055726 |
| Arid3b | 0.13137 | -0.40170835 |
| Kifc1 | 0.72551 | -0.20228444 |
| 4930597A21Rik | 0.68622 | 0.30421227 |
| Hephl1 | 0.00144 | -1.47614798 |
| Fam166b | 0.0156 | -0.74720793 |
| Smpd2 | 0.50253 | -0.34423738 |
| Gm10871 | 0.01729 | -0.65672326 |
| Gm36823 | 0.00144 | -1.28116048 |
| Arhgap12 | 0.95036 | -0.01055309 |
| Prdx1 | 0.12338 | -0.5717922 |
| Slc7a14 | 0.0093 | -0.58903815 |
| 4930545H06Rik | 0.00836 | -0.88324425 |
| Mb21d1 | 0.0125 | 1.20088801 |
| Ttf2 | 0.01409 | -0.59321282 |
| Tmem42 | 0.65795 | -0.13719764 |
| Gm12022 | 0.00144 | -1.29051721 |
| Ppwd1 | 0.03255 | 0.77778368 |
| Klhl33 | 0.02531 | -0.51108684 |
| Ss18 | 0.07765 | 0.42484375 |
| 1700060L04Rik | 0.00144 | -1.30713006 |
| Khdrbs3 | 0.9419 | 0.02957959 |
| Gm9047 | 0.00144 | -1.28116048 |
| Gm41959 | 0.02961 | -0.94782714 |
| Fgd4 | 0.00144 | 1.41378247 |
| Gm34078 | 0.00144 | -1.28116048 |
| 1700055C04Rik | 0.00144 | -1.40054462 |
| Gdpgp1 | 0.69596 | 0.12386849 |
| Itpkb | 0.00288 | -0.74558657 |
| Mad2l2 | 0.28745 | -0.30525306 |
| Syndig1 | 0.21586 | -1.11874395 |
| Spink14 | 0.00144 | -1.28898079 |
| Gm14858 | 0.00149 | -1.25408 |
| Olfr813 | 0.52241 | 0.22141969 |
| 5330429C05Rik | 0.00144 | -1.22465584 |
| Samd12 | 0.10655 | 0.57410732 |
| Olfr1028 | 0.00145 | -1.26219879 |
| Gltscr1l | 0.03414 | -0.77470546 |
| Gm33786 | 0.7881 | -0.16044377 |
| 2610017A05Rik | 0.00144 | -1.28898079 |
| Agtr1b | 0.02856 | -0.88872563 |
| Stard9 | 0.81305 | -0.07322174 |
| Hspa8 | 0.09618 | -0.36289071 |
| Slc43a1 | 0.01522 | 1.43193877 |
| Zscan29 | 0.00354 | 1.6601492 |
| Zfp951 | 0.00158 | -1.22853957 |
| Gpd1 | 0.85743 | -0.07859898 |
| Gm30173 | 0.09012 | -2.33696277 |
| Gm30836 | 0.34903 | -0.42044952 |
| Gm5606 | 0.15569 | -0.84142486 |
| Clmp | 0.00649 | -1.30792117 |
| Mecom | 0.01604 | -0.93750278 |
| 4921517O11Rik | 0.00622 | -1.16266692 |
| Vmn1r1 | 0.00968 | -0.99308048 |
| Gm30892 | 0.00144 | -1.20555712 |
| Enpp6 | 0.00198 | -1.12868614 |
| Clip4 | 0.43081 | -0.14128894 |
| Prrx2 | 0.02636 | -1.16976395 |
| 4930590J08Rik | 0.78938 | 0.1433991 |
| D730045A05Rik | 0.90163 | -0.04649436 |
| Ly96 | 0.00794 | 1.07339417 |
| Psg18 | 0.00145 | -1.2388121 |
| S100a10 | 0.00754 | -0.70402364 |
| Gm35722 | 0.00144 | -1.28116048 |
| Otof | 0.07389 | -0.3297271 |
| Pdap1 | 0.07768 | 0.50892989 |
| A730045E13Rik | 0.00144 | -1.27165604 |
| Ptchd4 | 0.00145 | -1.22376446 |
| 5730412P04Rik | 0.00144 | -1.29049509 |
| Gm9933 | 0.01949 | -0.51101237 |
| Sp1 | 0.88634 | -0.0248195 |
| Olfr310 | 0.03237 | -0.89971117 |
| Olfr1183 | 0.00144 | -1.2383131 |
| Hook2 | 0.36694 | 0.33175739 |
| Clspn | 0.00144 | -1.29085005 |
| Olfr452 | 0.00144 | -1.30713006 |
| Camsap1 | 0.34782 | 0.22589043 |
| Elane | 0.02331 | -0.54280918 |
| 9130409J20Rik | 0.35601 | -0.49092978 |
| Vps11 | 0.03979 | -0.85514435 |
| Bcl2l14 | 0.00883 | -0.8492789 |
| 9330104G04Rik | 0.13625 | -0.68750549 |
| Gm10747 | 0.06073 | 0.6482224 |
| Shisa6 | 0.21843 | -0.21183349 |
| Arl6ip4 | 0.00897 | -0.63159211 |
| D830044I16Rik | 0.8675 | 0.03756303 |
| Zbtb8os | 0.51469 | 0.13546456 |
| Coq7 | 0.00523 | -0.71338457 |
| AF067063 | 0.01326 | -1.72535225 |
| 2810029C07Rik | 0.90024 | -0.07106943 |
| Cks1b | 0.22339 | -0.45623337 |
| Adamts17 | 0.08287 | 0.77710108 |
| Tmem258 | 0.00305 | -1.04698507 |
| Per3 | 0.157 | -0.57361576 |
| Ppia | 0.01601 | -0.53308822 |
| Asb17os | 0.5471 | -0.54668123 |
| Ube2e1 | 0.02949 | 0.53832746 |
| Tmem141 | 0.68612 | -0.21419025 |
| Hcn3 | 0.52934 | -0.19666781 |
| Xpo7 | 0.53764 | -0.41076968 |
| Slc26a8 | 0.0603 | -1.21290582 |
| Gm11413 | 0.18928 | -0.73707107 |
| Klk1b21 | 0.90626 | 0.02912232 |
| Fgf3 | 0.00144 | -1.75096634 |
| Gm20257 | 0.00144 | -1.28116048 |
| Med14 | 0.85996 | -0.06112845 |
| Fam13c | 0.07799 | -0.84199975 |
| Mill1 | 0.01647 | -1.07142346 |
| Gss | 0.59736 | -0.18106344 |
| Nuggc | 0.00321 | -1.27646041 |
| Nadk | 0.04535 | -0.4540466 |
| Stard6 | 0.00144 | 2.02740032 |
| Olfr1329 | 0.14654 | -0.99984712 |
| 1700019D03Rik | 0.00144 | -1.27165604 |
| Gm41183 | 0.04552 | -0.81581966 |
| 9330182O14Rik | 0.06559 | -0.86188542 |
| 4933432K03Rik | 0.32982 | 0.57997905 |
| Armc1 | 0.07912 | 0.78026237 |
| Dppa2 | 0.00145 | -1.2388121 |
| Irak1bp1 | 0.06865 | -0.38871705 |
| Fam65b | 0.9932 | -0.0049415 |
| Lrrc10b | 0.8936 | -0.03357526 |
| B230217O12Rik | 0.00377 | 0.91748532 |
| 4930505K13Rik | 0.00144 | -1.22304353 |
| Ptprn2 | 0.0399 | -2.10110804 |
| Cabp1 | 0.0017 | -1.24950944 |
| Peg13 | 0.01132 | -0.57193792 |
| Fez1 | 0.10545 | -0.57551439 |
| Ccdc142 | 0.00627 | -1.56500132 |
| Gmppa | 0.58095 | 0.28482151 |
| Trio | 0.8606 | 0.02875376 |
| 4930556N09Rik | 0.11981 | -0.40893669 |
| 1700023F02Rik | 0.02144 | -0.79765928 |
| Olfr1098 | 0.01576 | -1.32466826 |
| Gm12532 | 0.01145 | -1.19499308 |
| 1700061G19Rik | 0.39518 | -1.4838904 |
| A630034I12Rik | 0.00583 | 1.11125118 |
| Ankrd35 | 0.27442 | -0.30327057 |
| Ackr3 | 0.06719 | -0.48460241 |
| 1810012K08Rik | 0.00811 | -0.92360188 |
| Zfp958 | 0.00144 | 3.11371958 |
| Jup | 0.03706 | -0.75383902 |
| Slc25a19 | 0.6821 | -0.14500552 |
| Dner | 0.00144 | -1.31884045 |
| Hoxa11os | 0.01505 | 0.50524152 |
| Gm39888 | 0.00829 | -1.66364016 |
| Olfr287 | 0.83843 | -0.05547819 |
| Chp1 | 0.00449 | -1.1498686 |
| Cnot3 | 0.72024 | -0.13642709 |
| Gm19576 | 0.03372 | -0.73648991 |
| Gm6756 | 0.00144 | -1.28116048 |
| 1700003I22Rik | 0.00144 | -1.28116048 |
| Sycp2l | 0.00159 | -1.26431393 |
| Cdc123 | 0.00165 | 1.306723 |
| Nagk | 0.47037 | -0.27486706 |
| Olfr453 | 0.00144 | -1.30713006 |
| Actg1 | 0.74624 | -0.11626529 |
| Osbpl5 | 0.00341 | -0.89197282 |
| 4930459C07Rik | 0.10672 | -0.79618843 |
| Zbtb2 | 0.61974 | 0.14685753 |
| Olfr56 | 0.15552 | -0.31030802 |
| Scarletltr | 0.79755 | -0.04413832 |
| Gm21221 | 0.01277 | -1.70999226 |
| Olfr311 | 0.00144 | -1.27165604 |
| Tmem181a | 0.26112 | -0.31549338 |
| Ggnbp1 | 0.0067 | 0.99858872 |
| Zfp72 | 0.17021 | -0.31528063 |
| Sptlc1 | 0.00508 | -0.67177624 |
| Klhdc1 | 0.03143 | -0.41465829 |
| Atxn7l3 | 0.00364 | -0.76495098 |
| Gm9456 | 0.00144 | -1.36473674 |
| C030007H22Rik | 0.01566 | -1.16329091 |
| 1700003D09Rik | 0.03804 | -0.39876937 |
| LOC101056011 | 0.32604 | 0.22784342 |
| Ift88os | 0.02476 | -1.25854869 |
| Zbtb7c | 0.00179 | -1.73643108 |
| Gm36851 | 0.02145 | -1.67940874 |
| 1700048O14Rik | 0.00144 | -1.28819962 |
| Cfap52 | 0.00144 | -1.29049509 |
| Atad2b | 0.84803 | 0.03941345 |
| Gm32914 | 0.00601 | -0.75422072 |
| Colgalt1 | 0.99177 | 0.00492024 |
| Gm41744 | 0.06098 | -0.91531813 |
| 4930573O16Rik | 0.15921 | -0.91815731 |
| Peli2 | 0.0324 | -0.78066596 |
| Olfr132 | 0.00144 | -1.29085005 |
| 1700049E22Rik | 0.03027 | -1.24545467 |
| Bmpr1b | 0.16605 | -0.77892679 |
| Ddx41 | 0.23073 | -0.46046804 |
| A230045G11Rik | 0.15352 | 0.25921728 |
| Arhgap27os3 | 0.33651 | -0.85107977 |
| 7030419G21Rik | 0.00144 | -1.30017714 |
| Vmn1r17 | 0.00158 | -1.2446827 |
| 4933400B14Rik | 0.00144 | -1.27454685 |
| Hsh2d | 0.00144 | -1.27454685 |
| Ern2 | 0.13613 | -0.7860182 |
| Rasgrf2 | 0.0021 | -1.09360304 |
| Crlf2 | 0.00192 | -0.93830153 |
| Ppil4 | 0.00184 | 1.08711018 |
| Zfhx2 | 0.04393 | -0.4428831 |
| Ift172 | 0.78242 | -0.10632229 |
| Rbm4b | 0.12825 | 0.56357135 |
| Ntn3 | 0.30038 | -0.56357915 |
| Mff | 0.02323 | 0.67558804 |
| Rai14 | 0.00321 | 1.01042248 |
| 1700095J07Rik | 0.00144 | -1.28116048 |
| Cnbd2 | 0.01586 | -1.05743197 |
| Gm40289 | 0.0051 | -1.40054462 |
| Gm550 | 0.22371 | -0.50072162 |
| Gm6760 | 0.00144 | -1.33632959 |
| Wee1 | 0.00374 | 0.92477373 |
| Olfr367-ps | 0.00149 | -1.2703832 |
| Olfr64 | 0.00544 | -1.10518964 |
| Olfr735 | 0.00144 | -1.21977358 |
| Gm32755 | 0.55731 | -0.18188215 |
| Gm7134 | 0.00144 | -1.31627578 |
| Olfr1200 | 0.12692 | -1.00574663 |
| Trim40 | 0.00144 | -1.27165604 |
| Cfl1 | 0.23071 | -1.20979464 |
| Lefty2 | 0.2464 | 0.73446536 |
| Coasy | 0.01631 | -0.68675603 |
| Ctnna3 | 0.0071 | -0.99357624 |
| Gtsf1 | 0.07887 | -2.65845678 |
| BC048602 | 0.00144 | -1.26911831 |
| Tex16 | 0.00145 | -1.17894292 |
| Renbp | 0.0209 | -0.68146114 |
| 9530082P21Rik | 0.91429 | 0.03886283 |
| Cnot6l | 0.6715 | 0.08011062 |
| Znrf3 | 0.10208 | -0.39275602 |
| Cyp26c1 | 0.00144 | -1.28116048 |
| Tmem132d | 0.00144 | -1.26934956 |
| Wfdc11 | 0.00144 | -1.24865146 |
| Gm14062 | 0.11256 | -2.97954644 |
| Tmeff2 | 0.00144 | -1.50450276 |
| Gm5914 | 0.26561 | 0.33004818 |
| Fam63b | 0.56787 | 0.22747377 |
| Apobec3 | 0.00702 | 0.71904419 |
| Gm12505 | 0.00145 | -1.33249771 |
| Lhx1 | 0.00415 | -1.06195552 |
| Mtss1l | 0.63404 | -0.57587328 |
| Apol10b | 0.18342 | -0.57740285 |
| D130009I18Rik | 0.00144 | -1.42084929 |
| Abcb5 | 0.0089 | -1.98550712 |
| Eef2 | 0.00178 | -0.99510363 |
| Mmadhc | 0.25905 | 0.2278111 |
| Gm12264 | 0.04534 | -0.6457245 |
| Polr3c | 0.51671 | 0.26359461 |
| Cacna1h | 0.05473 | -0.44261172 |
| Spag1 | 0.00144 | 1.9366949 |
| Cox10 | 0.13277 | -0.54583704 |
| Olfr671 | 0.28416 | -0.68085014 |
| Dcst2 | 0.13729 | -0.40395392 |
| Rrp1 | 0.17855 | -0.21147368 |
| 4930583P06Rik | 0.00149 | -1.19348234 |
| LOC102636896 | 0.00144 | -1.50578853 |
| Nlrp9b | 0.00288 | -2.2628928 |
| Abhd6 | 0.52787 | -0.13530841 |
| 6330537M06Rik | 0.11125 | -0.92577285 |
| Otof | 0.25333 | -0.20122215 |
| Cdh24 | 0.01588 | -0.99849484 |
| A330008L17Rik | 0.00144 | -1.30713006 |
| Fip1l1 | 0.21708 | -0.20978228 |
| Stx5a | 0.65731 | 0.12356137 |
| 1700016P03Rik | 0.39267 | 0.73588066 |
| Smg9 | 0.02134 | -0.57085875 |
| Sarnp | 0.76962 | -0.12354779 |
| Gm26871 | 0.00144 | -1.25118919 |
| Ldb1 | 0.06035 | -0.50357206 |
| 4930570D08Rik | 0.19735 | -0.78520753 |
| Wdr1 | 0.1341 | -0.32946451 |
| Gm3740 | 0.01563 | -0.62346834 |
| Stx3 | 0.28818 | -0.54444184 |
| Gm34086 | 0.34382 | 0.44844825 |
| Stx2 | 0.7364 | 0.17689574 |
| Cyp2d11 | 0.97962 | -0.00579817 |
| Vps51 | 0.05225 | -0.57611215 |
| Tgfb1 | 0.01061 | -0.8379426 |
| BC055402 | 0.01395 | -1.01228913 |
| Psg25 | 0.00144 | -1.26560808 |
| Gm40798 | 0.38131 | 0.48004369 |
| Gm35319 | 0.18749 | 0.3470036 |
| F730035M05Rik | 0.00165 | -1.64577724 |
| Zdhhc18 | 0.05253 | -0.38014032 |
| Gm15870 | 0.00257 | -1.1333102 |
| Gm38999 | 0.04356 | -0.59081987 |
| Capn9 | 0.00997 | -0.7402015 |
| 4930513D17Rik | 0.01759 | -2.84050043 |
| LOC102641977 | 0.00144 | 1.23869738 |
| Dennd5b | 0.2529 | 0.4336951 |
| Olfr605 | 0.00144 | -1.28116048 |
| 4930545L08Rik | 0.00144 | -1.25118919 |
| Gm5591 | 0.17574 | -0.69501203 |
| Gm14285 | 0.02018 | -0.92403253 |
| Gm12853 | 0.00827 | -0.65788503 |
| BE691133 | 0.09694 | -2.05784331 |
| Gm9921 | 0.56376 | -0.3491019 |
| Gm14569 | 0.00409 | -2.00016332 |
| Zfp950 | 0.00144 | 2.96012903 |
| Olfr722 | 0.00144 | -1.64649884 |
| Gm31267 | 0.02073 | -0.8330171 |
| Habp2 | 0.00149 | -1.19348234 |
| Pard3b | 0.37103 | -0.18429917 |
| Rpl7l1 | 0.50978 | 0.23808084 |
| Gm26881 | 0.00362 | -1.11124141 |
| Dennd3 | 0.76172 | -0.09704282 |
| Gm32004 | 0.00144 | -1.29085005 |
| AU021063 | 0.00943 | -1.46262384 |
| Tmem108 | 0.03849 | -0.45713027 |
| Clcnka | 0.00144 | -1.29878664 |
| LOC628205 | 0.00144 | 2.40742886 |
| Gm30871 | 0.00149 | -1.22582153 |
| Gm9099 | 0.06196 | -0.60957619 |
| Gm31828 | 0.00144 | -1.25118919 |
| Glt1d1 | 0.00964 | -1.68047009 |
| AI481877 | 0.03225 | 0.74582592 |
| 4930518P08Rik | 0.26538 | -0.85721467 |
| Mgat4d | 0.0028 | -1.16569658 |
| E130215H24Rik | 0.1447 | 0.32276285 |
| Clec7a | 0.78238 | 0.12688957 |
| 1700019B21Rik | 0.0021 | -1.63265249 |
| D930030I03Rik | 0.08368 | -0.86316526 |
| Clec18a | 0.09396 | -0.87423081 |
| Ermard | 0.00451 | 1.04091021 |
| Tyr | 0.0023 | -1.08189352 |
| Ccny | 0.05884 | -0.463019 |
| Rab4b | 0.00474 | -0.83122405 |
| Spata6 | 0.00144 | 1.57714862 |
| Fbxo32 | 0.19332 | 0.3406778 |
| Ccdc137 | 0.74786 | 0.11356339 |
| E130309D02Rik | 0.2059 | -2.40703802 |
| Card9 | 0.00153 | -1.82867074 |
| Slc25a12 | 0.00221 | -0.89715911 |
| Gm41724 | 0.49742 | -0.503116 |
| B230317F23Rik | 0.43159 | 0.39520153 |
| Xkr9 | 0.04553 | -1.35855313 |
| A930004J17Rik | 0.03913 | 0.45380354 |
| Yrdc | 0.92227 | 0.02698612 |
| Etv1 | 0.48254 | -0.12391242 |
| 9630013A20Rik | 0.00145 | -1.25646236 |
| Apex1 | 0.08624 | -0.43266513 |
| 4930517G24Rik | 0.19226 | -0.75559551 |
| 1700094M24Rik | 0.72565 | -0.09470479 |
| Gm12426 | 0.10674 | 0.63826526 |
| Manf | 0.48649 | 0.19719636 |
| Gm8149 | 0.00144 | -1.29746368 |
| Gm28497 | 0.00144 | -1.35320495 |
| Pdhx | 0.15892 | 0.49508628 |
| Nkx2-2os | 0.00144 | -1.20555712 |
| Eif3i | 0.44364 | -0.27749158 |
| Slco3a1 | 0.2817 | -0.25080434 |
| Nctc1 | 0.21983 | -0.50189851 |
| Tex21 | 0.00564 | -1.17300636 |
| Gm32960 | 0.00144 | -1.30017714 |
| Nudt15 | 0.00144 | -1.38156382 |
| Per3 | 0.24705 | 0.2095146 |
| Ubox5 | 0.63694 | -0.20958581 |
| Gm3960 | 0.01047 | -1.00099289 |
| Krtap5-1 | 0.54511 | 0.26306759 |
| 1700044K03Rik | 0.00144 | -1.31495282 |
| Ifi35 | 0.00219 | -0.82360762 |
| Teddm1b | 0.00973 | -2.08711891 |
| Slc35e2 | 0.98945 | 0.00301102 |
| Gm15441 | 0.72543 | -0.10358406 |
| Kis2 | 0.00144 | -1.27730451 |
| Gcnt4 | 0.00158 | -1.2446827 |
| Serpina1b | 0.00345 | -1.13628123 |
| Ankub1 | 0.36498 | -0.24832769 |
| 9630013D21Rik | 0.00144 | 1.90221639 |
| LOC102635658 | 0.02122 | -0.96350362 |
| Pde6h | 0.04551 | -0.91232186 |
| BC026513 | 0.00144 | 2.41566375 |
| Gm20744 | 0.46992 | 0.24219491 |
| Wdsub1 | 0.00338 | -0.88176051 |
| G730013B05Rik | 0.01514 | -1.03228718 |
| Pcca | 0.01301 | -1.0212011 |
| Gpr39 | 0.00877 | -1.68292746 |
| Dsg1b | 0.00144 | -1.33632959 |
| Gm36849 | 0.15484 | -0.55321502 |
| Qrich2 | 0.08746 | 0.4323054 |
| D11Wsu47e | 0.00386 | -0.81725641 |
| Mest | 0.0135 | -1.67821538 |
| 4933401H06Rik | 0.00145 | -1.25646236 |
| Nlrp9b | 0.00182 | -1.32924611 |
| 1700045H11Rik | 0.08095 | -0.52578311 |
| Ap4e1 | 0.07563 | 0.48116336 |
| 5330411J11Rik | 0.0057 | -0.95803672 |
| Mmp17 | 0.27951 | -0.73810637 |
| Nos1ap | 0.18934 | -0.46106137 |
| Lsamp | 0.05683 | -0.89257193 |
| LOC102638515 | 0.01112 | -0.6877074 |
| Pter | 0.5436 | 0.11824603 |
| Gm38562 | 0.01418 | -1.43784992 |
| Oas1b | 0.18668 | 0.33033707 |
| 4930591E09Rik | 0.00144 | -1.28116048 |
| Ptgdr2 | 0.10024 | -0.97826553 |
| Psmg1 | 0.07298 | -0.47323821 |
| Duox2 | 0.00607 | -0.90809973 |
| Otx2os1 | 0.00145 | -1.2388121 |
| Mphosph9 | 0.02366 | -1.05348117 |
| Gm4952 | 0.00144 | -1.26934956 |
| Gm3858 | 0.01307 | -0.8913628 |
| Rps5 | 0.01104 | -0.89397553 |
| Sirt4 | 0.19012 | -0.28949909 |
| Gm30455 | 0.00202 | -1.82759249 |
| Slc25a32 | 0.00144 | -1.40720366 |
| Olfr798 | 0.00144 | -1.25118919 |
| Gm30052 | 0.06603 | -1.27132146 |
| Rusc2 | 0.01434 | -0.73298457 |
| Nln | 0.66745 | 0.0808843 |
| Kcnab2 | 0.00144 | -1.29049509 |
| Olfr917 | 0.023 | -1.71818581 |
| 6530437J22Rik | 0.1341 | -2.42065113 |
| Gm37359 | 0.10962 | -0.83607133 |
| Hdac10 | 0.01598 | -0.63962358 |
| 4631405K08Rik | 0.16998 | 0.60577113 |
| Slco2a1 | 0.03006 | -0.5111802 |
| Gm38829 | 0.3003 | -0.52889299 |
| Ctr9 | 0.13673 | 0.5857302 |
| Anln | 0.01734 | 1.42852697 |
| A430110L20Rik | 0.03579 | 2.04206796 |
| Gm11419 | 0.00144 | -1.28898079 |
| Ptcd3 | 0.00189 | -0.8818083 |
| Atl2 | 0.00304 | 1.10351626 |
| G630071F17Rik | 0.00256 | -1.28183002 |
| Tomm40l | 0.01749 | -0.55813734 |
| Parn | 0.52875 | 0.23676799 |
| Gm12680 | 0.00144 | -1.30737044 |
| Aim2 | 0.06868 | -0.99047078 |
| Gm4208 | 0.00375 | -2.67722329 |
| Lif | 0.00981 | -1.03615246 |
| Skp2 | 0.00477 | 1.06324449 |
| Glul | 0.81561 | -0.09558451 |
| Ang3 | 0.14421 | -0.26279644 |
| I830077J02Rik | 0.19814 | -0.61372926 |
| Ift46 | 0.26265 | -0.27889032 |
| Pcnxl3 | 0.43876 | 0.21220453 |
| 4930522L14Rik | 0.63673 | -0.26863765 |
| Rbm26 | 0.00144 | 2.0732222 |
| Sfrp1 | 0.67529 | -0.11197485 |
| Dnah7a | 0.02229 | -0.66678118 |
| 3110021N24Rik | 0.66528 | 0.17771768 |
| Tmtc3 | 0.78094 | -0.05108851 |
| Mettl25 | 0.17631 | 0.25288898 |
| Axin2 | 0.01995 | -1.34524895 |
| Slc25a12 | 0.14353 | 0.79693444 |
| Pramel1 | 0.00144 | -1.26560808 |
| Pde6c | 0.08896 | -1.42168566 |
| LOC102631612 | 0.00144 | -1.28116048 |
| Olfr116 | 0.00879 | -1.05401892 |
| Gm5079 | 0.04533 | -0.48979952 |
| Gm20098 | 0.00804 | -1.40715177 |
| Abcc12 | 0.00577 | -1.05980796 |
| Lrrtm3 | 0.005 | -0.88070774 |
| Ceacam1 | 0.18216 | 0.37632094 |
| Tefm | 0.0447 | 1.02934236 |
| Gm20063 | 0.11656 | -0.45558903 |
| Gm30075 | 0.17147 | -0.51571014 |
| Gm14244 | 0.00144 | -1.27558215 |
| Olfr978 | 0.33821 | -0.67874349 |
| Uba1y | 0.35249 | -0.22115583 |
| 4930452G13Rik | 0.5185 | -0.43731467 |
| Gm2087 | 0.00271 | -1.10730936 |
| 2610306M01Rik | 0.00144 | -1.26911831 |
| Tnfrsf22 | 0.75259 | 0.14364675 |
| Nlrp9c | 0.00144 | -1.27454685 |
| Fam47e | 0.00144 | -1.28116048 |
| Gm28068 | 0.41443 | -0.25517552 |
| 4930551L18Rik | 0.00314 | -1.13684067 |
| Efna5 | 0.13104 | -0.30902239 |
| B3galnt2 | 0.00309 | -1.05527353 |
| Kif17 | 0.17494 | -0.33839133 |
| Vps11 | 0.0123 | -0.69590975 |
| Kif12 | 0.10163 | -1.70409898 |
| C330013E15Rik | 0.31436 | -0.43343602 |
| C030017D09Rik | 0.00144 | -1.31884045 |
| Haus6 | 0.00217 | 0.98654742 |
| Aym1 | 0.00233 | -1.08640103 |
| Vmn1r232 | 0.00144 | -1.31495282 |
| Gm40812 | 0.00846 | -1.27272598 |
| Snx30 | 0.04373 | -0.44673416 |
| Dlx6os1 | 0.00222 | -1.1481805 |
| Gnrhr | 0.00148 | -1.43466873 |
| Gm3331 | 0.00728 | -1.45121232 |
| C79562 | 0.00144 | -1.29746368 |
| Phlpp1 | 0.44692 | 0.52864587 |
| Alpl | 0.02045 | -0.55031297 |
| Trpm3 | 0.02511 | 0.91417729 |
| C2cd4d | 0.004 | -1.01833101 |
| 4930401A07Rik | 0.00144 | -1.27165604 |
| Gm16108 | 0.07563 | -2.43335423 |
| A330076H08Rik | 0.00158 | -1.2446827 |
| Tex29 | 0.00234 | 1.20117558 |
| Gm7135 | 0.00144 | -1.29864962 |
| Pxn | 0.00144 | -1.1906677 |
| Col8a2 | 0.00144 | -1.27165604 |
| Gm3728 | 0.15126 | -0.84601109 |
| Vps25 | 0.85503 | -0.08082542 |
| Mettl15 | 0.04413 | -0.83468084 |
| C330021F23Rik | 0.02617 | -1.51545018 |
| Rps14 | 0.00144 | -1.19530456 |
| Cyp20a1 | 0.00758 | 0.83328318 |
| Gm36169 | 0.00144 | -1.27165604 |
| Slc26a7 | 0.30463 | -0.58301281 |
| LOC105245924 | 0.00144 | -1.24613586 |
| 4930529L06Rik | 0.15155 | -1.39435879 |
| Mmp1b | 0.00144 | -1.24811894 |
| Cdip1 | 0.28135 | -0.3746051 |
| Gm5093 | 0.01678 | -0.77219603 |
| Akr1e1 | 0.01206 | 0.58124319 |
| Aste1 | 0.26045 | 0.41617354 |
| Pik3ip1 | 0.80817 | -0.04021243 |
| Rev3l | 0.03653 | 0.83792787 |
| Atcay | 0.15448 | -0.28741016 |
| 1700006H21Rik | 0.00144 | -1.29864962 |
| Gm12915 | 0.00145 | -1.97052679 |
| Cracr2a | 0.03134 | -0.50681269 |
| Slc14a2 | 0.00144 | -1.24353547 |
| Fundc2 | 0.82175 | 0.06705151 |
| AI115009 | 0.00144 | -1.25118919 |
| Gm13580 | 0.00144 | -0.97880914 |
| Ndufa10 | 0.31265 | -0.31635905 |
| Prpf38a | 0.08287 | -0.60631398 |
| Chd8 | 0.23417 | -0.27549395 |
| Mccc1 | 0.00371 | 1.0626981 |
| Pam16 | 0.18892 | -0.43764678 |
| Kcnk9 | 0.00144 | -1.20579466 |
| Gm27151 | 0.00144 | -1.27992547 |
| Gm12292 | 0.01877 | -1.80948131 |
| Itga11 | 0.02981 | -1.00826146 |
| 1700095J03Rik | 0.00236 | -1.37083112 |
| Mcm9 | 0.54819 | 0.13494468 |
| Nedd4l | 0.36321 | 0.19411258 |
| Slc10a1 | 0.03669 | -0.94779588 |
| Ppil2 | 0.0052 | -1.10379914 |
| Gm32058 | 0.00335 | -1.76663393 |
| Lhfpl2 | 0.00144 | -1.31495282 |
| 9330136K24Rik | 0.67069 | -0.14450064 |
| 4930405L22Rik | 0.06448 | -1.32397723 |
| Tbc1d17 | 0.01179 | -0.73505641 |
| Psenen | 0.00206 | -0.97574656 |
| Pkd1l3 | 0.0931 | -0.52502698 |
| Gm13201 | 0.50688 | 0.23577537 |
| Prdm11 | 0.82434 | 0.04869847 |
| Tspy-ps | 0.12585 | -3.11619354 |
| 2810433D01Rik | 0.29588 | -0.52282479 |
| Rpl35 | 0.00144 | -1.16525204 |
| Gm12888 | 0.00145 | -1.22376446 |
| Nup88 | 0.2833 | 0.18719731 |
| E330021D16Rik | 0.00144 | -1.28898079 |
| A630009H07Rik | 0.70039 | -0.09753295 |
| Bvht | 0.00851 | -0.66328598 |
| Idh3g | 0.93516 | -0.02877119 |
| 4930441J16Rik | 0.00151 | -1.17792995 |
| Trappc12 | 0.49025 | -0.25442374 |
| A530013C23Rik | 0.91215 | 0.02057483 |
| Gm35525 | 0.0399 | -0.80437207 |
| Csmd2 | 0.00145 | -1.2388121 |
| Fam133b | 0.00144 | 2.17559502 |
| 5730416F02Rik | 0.00144 | -1.1618576 |
| Gm33926 | 0.00144 | -1.31310402 |
| Cep126 | 0.00586 | -2.09996726 |
| Gm4132 | 0.00713 | -0.64716475 |
| Cracr2a | 0.96107 | 0.03519882 |
| Gpr15 | 0.02563 | -1.3826329 |
| 2610203C22Rik | 0.03014 | -1.33150482 |
| Supt7l | 0.74857 | 0.15292682 |
| Pdcd6ip | 0.05691 | 0.42954797 |
| Nsun5 | 0.43528 | -0.16684207 |
| Lasc1 | 0.18403 | -0.68636255 |
| Mettl23 | 0.25629 | -0.40211277 |
| Dnajb9 | 0.00144 | -1.0476356 |
| Slitrk5 | 0.11388 | -0.92797408 |
| Gm53 | 0.00154 | -1.31035088 |
| Pus7 | 0.06065 | -0.64088037 |
| Gm10706 | 0.03479 | -0.68373025 |
| Vwa5b2 | 0.14013 | -1.01963123 |
| 4930413F20Rik | 0.02018 | -1.4114709 |
| Hint2 | 0.00163 | -0.90337447 |
| 4930470O06Rik | 0.00145 | -1.22376446 |
| Ppm1k | 0.00145 | -1.17631656 |
| Chil4 | 0.00144 | -1.33059316 |
| 4932418E24Rik | 0.00541 | -1.47676176 |
| 5930422O12Rik | 0.00158 | -1.2446827 |
| Gm33560 | 0.04145 | -0.55573446 |
| LOC102632357 | 0.5091 | 0.38120097 |
| 9830166K06Rik | 0.00144 | -1.38527672 |
| Gm26843 | 0.00145 | -1.2388121 |
| Slc22a19 | 0.00144 | -1.31495282 |
| Gm36394 | 0.07497 | -0.83721856 |
| 4930451E10Rik | 0.00144 | -1.21212043 |
| Nid1 | 0.0059 | 1.84652099 |
| Caap1 | 0.37078 | 0.20194991 |
| Rhd | 0.67163 | -0.19676812 |
| Aldoc | 0.2171 | -0.67404672 |
| LOC675947 | 0.00144 | -1.28116048 |
| Hesx1 | 0.1698 | -0.61149755 |
| Gm3704 | 0.00583 | -0.75371401 |
| 9530026P05Rik | 0.05117 | 1.16001382 |
| Prkch | 0.14847 | 0.34667405 |
| Tor1aip2 | 0.01797 | -0.97449752 |
| Gm12670 | 0.00145 | -1.2388121 |
| Hnrnpll | 0.0044 | 0.89530922 |
| LOC102642832 | 0.53432 | 0.15042301 |
| BC021767 | 0.93496 | 0.05485992 |
| Ppip5k1 | 0.50807 | 0.22836057 |
| Asns | 0.81855 | 0.04823123 |
| Ankfy1 | 0.04108 | 0.40088173 |
| BC051665 | 0.00144 | -1.25927895 |
| Ankrd50 | 0.80892 | -0.06640083 |
| Rfc5 | 0.02615 | -0.49769108 |
| Nfyc | 0.4739 | -0.12382721 |
| Gm6249 | 0.08649 | -2.52090451 |
| 9230112E08Rik | 0.34122 | 0.24120796 |
| Gm26843 | 0.06322 | -0.90159113 |
| Sil1 | 0.40414 | -0.34022256 |
| Rpe | 0.15538 | -0.25974036 |
| Gm3227 | 0.33445 | -0.57835535 |
| 9430024E24Rik | 0.00512 | -1.06802371 |
| Il15 | 0.46552 | 0.17117247 |
| Aoah | 0.0031 | -1.09550759 |
| E330012B07Rik | 0.15594 | -0.65861265 |
| Sarm1 | 0.05503 | -0.6732776 |
| Gm36877 | 0.03784 | -0.57880934 |
| Gm40292 | 0.14303 | -0.87531143 |
| Olfr834 | 0.00144 | -1.31495282 |
| 4930503B20Rik | 0.08855 | -1.17830593 |
| Gm14635 | 0.01869 | -1.56126036 |
| Gm4791 | 0.00144 | -1.24613586 |
| LOC100048452 | 0.0163 | -0.82449541 |
| C2cd2l | 0.03668 | -0.48354816 |
| Tspan31 | 0.41526 | -0.24363313 |
| Mup3 | 0.00313 | -0.87038593 |
| Olfr1186 | 0.07815 | -0.94568505 |
| 4930559C10Rik | 0.03557 | -0.93227475 |
| Diablo | 0.34677 | 0.48628013 |
| Mpped2 | 0.09372 | 0.37584896 |
| AU017674 | 0.13866 | -0.83084896 |
| Bnc2 | 0.59903 | -0.68330446 |
| B230307C23Rik | 0.06842 | 0.51683238 |
| Gm10637 | 0.00144 | -1.94127829 |
| BC024386 | 0.11631 | -1.11958829 |
| Wbp1l | 0.31173 | -0.36318206 |
| Gm20560 | 0.92622 | 0.03116163 |
| 4930429E23Rik | 0.37005 | -0.27568677 |
| BC049987 | 0.00144 | -1.33632959 |
| 1810044D09Rik | 0.02032 | -0.67934756 |
| Gm35239 | 0.00144 | -1.28375735 |
| Fgr | 0.10172 | -0.56804159 |
| Gm30019 | 0.00144 | -1.25927895 |
| Suco | 0.09057 | 0.59869378 |
| 4933412O06Rik | 0.01816 | -1.02516522 |
| B930049P21Rik | 0.27277 | -0.348652 |
| Snx25 | 0.15389 | 0.31366397 |
| Fcnaos | 0.04276 | -0.7592884 |
| Nol10 | 0.00144 | -1.4406408 |
| Hhip | 0.00144 | -1.28116048 |
| BC019684 | 0.21631 | -0.54881266 |
| 4930471D02Rik | 0.00144 | -1.54342655 |
| Gm8633 | 0.00144 | -1.29085005 |
| Prg3 | 0.01385 | -0.8491626 |
| E330020D12Rik | 0.00229 | -1.13943754 |
| Nr3c2 | 0.00499 | -0.73437264 |
| 9430077A04Rik | 0.0102 | -1.60613015 |
| Myzap | 0.0065 | 0.97445307 |
| Cyp2d13 | 0.00144 | -1.29746368 |
| Cep68 | 0.0567 | 0.38860598 |
| Gm29669 | 0.00144 | -1.30713006 |
| 2310043O21Rik | 0.00144 | -1.29049509 |
| Pot1a | 0.48927 | 0.14533348 |
| Ccdc141 | 0.30495 | -0.27232104 |
| Fzd4 | 0.01704 | 0.6750463 |
| Gm36367 | 0.3452 | 0.36823246 |
| Gm4319 | 0.64683 | -0.20812044 |
| Gm41790 | 0.00158 | -1.2446827 |
| Il20 | 0.01695 | -0.98480632 |
| 4931440J10Rik | 0.00144 | -1.27165604 |
| Atpaf2 | 0.48521 | 0.3037947 |
| LOC105245058 | 0.2957 | -0.18759455 |
| Kifc2 | 0.08018 | 0.35871055 |
| 1700015F17Rik | 0.00144 | -1.28898079 |
| Poldip2 | 0.20205 | -0.67765602 |
| Fxn | 0.03557 | -0.78384575 |
| Ndufaf5 | 0.83772 | 0.06078144 |
| Sema3a | 0.05678 | 0.60951306 |
| Topaz1 | 0.00144 | -1.26560808 |
| Uqcrb | 0.89321 | -0.0294478 |
| Atg4b | 0.27341 | -0.18360392 |
| Epha6 | 0.00144 | -1.26560808 |
| Vmn1r36 | 0.00144 | -1.28116048 |
| Gm14261 | 0.38873 | 0.19303658 |
| Oma1 | 0.14237 | 0.34813353 |
| Ube2i | 0.02066 | -0.51729333 |
| Olfr532 | 0.02264 | -0.92886545 |
| C230057M02Rik | 0.63032 | 0.10813905 |
| Ptgs2os2 | 0.00427 | -1.24530897 |
| Gm30516 | 0.02132 | -1.5880954 |
| 2010009K17Rik | 0.07538 | -0.73397847 |
| 4933429O19Rik | 0.59239 | -0.7470027 |
| Hs3st5 | 0.0382 | 0.5762251 |
| Mgat4d | 0.00144 | -1.28116048 |
| Tmem196 | 0.06137 | -0.36742041 |
| Pigv | 0.02794 | -1.07394409 |
| Lhx8 | 0.00716 | -1.51747993 |
| Lrrc75aos1 | 0.00144 | -1.27165604 |
| Osbpl1a | 0.15337 | -0.92814321 |
| 9830144P21Rik | 0.01113 | -1.62271449 |
| Asic3 | 0.32939 | 0.36927714 |
| Napsa | 0.02666 | -1.08315732 |
| Siglece | 0.02261 | -0.45898941 |
| Gm10373 | 0.00144 | -1.24466058 |
| Gm36456 | 0.01468 | -1.88264479 |
| Nf1 | 0.00251 | -0.90140143 |
| Srsf9 | 0.00304 | -0.90983943 |
| 4930405A21Rik | 0.00689 | -1.02798188 |
| Usb1 | 0.00144 | -1.75983809 |
| D930048G16Rik | 0.19042 | 0.34096011 |
| A330023F24Rik | 0.00144 | 2.48351617 |
| Gnpnat1 | 0.00503 | 1.29415332 |
| Cx3cl1 | 0.00834 | -0.74586484 |
| Gm10190 | 0.6313 | -0.20637721 |
| Nr4a3 | 0.00814 | -1.92654036 |
| A830018L16Rik | 0.00144 | -1.28116048 |
| A630089K24Rik | 0.00766 | -0.98393102 |
| C330020E22Rik | 0.16509 | -0.43954062 |
| Entpd8 | 0.02075 | -1.11881732 |
| Pafah1b2 | 0.74546 | -0.05960564 |
| 4430402I18Rik | 0.2597 | 0.32477403 |
| G630055G22Rik | 0.14665 | -1.01206277 |
| Mrpl21 | 0.54771 | 0.17420356 |
| Gm19590 | 0.02622 | -0.9501226 |
| Calcoco1 | 0.10429 | 0.70934724 |
| Gria3 | 0.74772 | 0.10941207 |
| Rad54b | 0.30958 | -0.30483459 |
| Topbp1 | 0.34676 | -0.47625445 |
| Adam23 | 0.00276 | -1.19115562 |
| Gm36455 | 0.00158 | -1.2446827 |
| 2010315B03Rik | 0.00144 | 1.22610615 |
| D030055H07Rik | 0.05265 | -2.06810804 |
| Akr1c13 | 0.37683 | 0.21429552 |
| Gm14061 | 0.62511 | -0.35593846 |
| Gm33188 | 0.49034 | 0.15966464 |
| Myl6 | 0.00716 | -0.61761175 |
| Ercc1 | 0.10365 | -0.45942244 |
| Cks2 | 0.05109 | -1.00343378 |
| Mlph | 0.05522 | -0.69538555 |
| Gm4430 | 0.11638 | -0.47093761 |
| Pitpna | 0.20132 | 0.25722727 |
| Gpn3 | 0.16626 | 0.54153331 |
| 4933416M06Rik | 0.01786 | -1.76196512 |
| Gm536 | 0.00383 | -1.13029182 |
| Gm36879 | 0.00144 | -1.26560808 |
| Mmp28 | 0.01102 | -1.04033337 |
| LOC102643180 | 0.00988 | 1.06133344 |
| 1700024B18Rik | 0.18261 | -0.5371681 |
| 4930445B16Rik | 0.00144 | -1.2963229 |
| Gm31590 | 0.56013 | -1.44223488 |
| Gm9959 | 0.02335 | -0.71799747 |
| Wdr49 | 0.00145 | -1.2388121 |
| 4833419F23Rik | 0.04164 | -0.43513157 |
| Gm33602 | 0.21 | -0.67449612 |
| Fbrsl1 | 0.01334 | -0.69158052 |
| Zmym1 | 0.09224 | 0.64843826 |
| Gm31738 | 0.00144 | -1.32155392 |
| Gm13016 | 0.00296 | -1.24082201 |
| Iqcb1 | 0.00628 | 2.09937089 |
| Commd2 | 0.92454 | -0.02555253 |
| Gm34433 | 0.04298 | -0.4612587 |
| Gm5608 | 0.27793 | 0.28895198 |
| Plin2 | 0.01191 | -0.62473887 |
| Nckap1l | 0.72592 | -0.09473632 |
| Pdx1 | 0.00144 | -1.27165604 |
| Jmjd4 | 0.08038 | -0.63742219 |
| 4930547E08Rik | 0.37147 | -0.42584374 |
| Tomm40 | 0.00438 | -1.05044915 |
| E2f8 | 0.02013 | -1.5232852 |
| 4930505K14Rik | 0.00144 | -1.33632959 |
| 4930594O21Rik | 0.22206 | -0.56868477 |
| 2310030A07Rik | 0.00144 | -1.29051721 |
| Drd5 | 0.02189 | -1.72814152 |
| Olfr527 | 0.00144 | -1.25118919 |
| Aurkc | 0.00571 | 1.02746961 |
| Atp8a1 | 0.10868 | 0.33810957 |
| Sycp1 | 0.00144 | -1.33182817 |
| Olfr117 | 0.00144 | -1.30017714 |
| Gm31026 | 0.02417 | -0.89118547 |
| Synrg | 0.01708 | -0.49695941 |
| Tsc22d2 | 0.00687 | -0.74128832 |
| Ptprj | 0.07898 | 0.77473038 |
| Cdhr3 | 0.14136 | -0.66666667 |
| Kcnmb1 | 0.55024 | 0.21830539 |
| Cnga4 | 0.00144 | -1.33632959 |
| Vwa5a | 0.02454 | -0.6053578 |
| Ulk4 | 0.0029 | -1.06147486 |
| A830011K09Rik | 0.00145 | -1.2388121 |
| Mettl22 | 0.66998 | -0.19378404 |
| 1700016D02Rik | 0.00702 | -1.56335487 |
| Gm14064 | 0.00144 | -1.28116048 |
| Ythdf2 | 0.21148 | -0.27755285 |
| Gm12530 | 0.22186 | -0.72259807 |
| Ptf1aos | 0.00144 | -1.29085005 |
| Ost4 | 0.47889 | -0.66295009 |
| Gm41764 | 0.00152 | -1.25878908 |
| Mon2 | 0.0029 | 0.82353352 |
| Znhit1 | 0.00144 | -1.14381945 |
| Gm11377 | 0.00145 | -1.22376446 |
| Gm31938 | 0.00144 | -1.32188652 |
| LOC102642914 | 0.00704 | -1.19266991 |
| Tvp23a | 0.1957 | -1.00976241 |
| Gm32172 | 0.00144 | -1.23155796 |
| LOC105245440 | 0.01051 | -1.1474527 |
| Myl12b | 0.00743 | -0.6882598 |
| Olfr1273-ps | 0.00144 | -1.26301121 |
| Gdap7 | 0.04353 | 0.93365121 |
| Ahnak2 | 0.00423 | -0.78363507 |
| Sstr5 | 0.10331 | -0.33079589 |
| Gm36608 | 0.04207 | -1.07589557 |
| Trip4 | 0.24426 | 0.52116251 |
| Sec22a | 0.22132 | 0.43959129 |
| Arhgap20 | 0.00409 | 1.3081511 |
| AA545190 | 0.00144 | -1.30713006 |
| 4930425K10Rik | 0.00144 | -1.58979569 |
| Otud4 | 0.00158 | 1.06064939 |
| Ddx6 | 0.01279 | -0.76040444 |
| Olfr899 | 0.00145 | -1.25646236 |
| C920008G01Rik | 0.00144 | -1.29051721 |
| Cd300ld | 0.01689 | 0.72454915 |
| Gm1110 | 0.00144 | -1.29051721 |
| Gm38497 | 0.09908 | -0.71591047 |
| Casc4 | 0.01044 | -0.75382861 |
| Art1 | 0.48604 | 0.18185095 |
| Anxa4 | 0.65326 | -0.07953252 |
| Dalrd3 | 0.01122 | -1.05282886 |
| Gm4265 | 0.00218 | -1.18878227 |
| Gm11681 | 0.00897 | -1.15797706 |
| Gm20616 | 0.02017 | -1.75696755 |
| Gm41175 | 0.02666 | -0.95694161 |
| Gm10069 | 0.22833 | 0.51144548 |
| Gpr26 | 0.79217 | -0.05282062 |
| Lmx1b | 0.00145 | -1.17020054 |
| Arhgap8 | 0.88019 | -0.04644441 |
| Mfap4 | 0.06754 | -0.50104126 |
| Grik1 | 0.75136 | -0.30267038 |
| 9430096G21Rik | 0.2844 | 1.1104504 |
| Gm40956 | 0.02627 | -1.30779993 |
| A430089I19Rik | 0.11118 | -2.93913693 |
| Gm10099 | 0.41767 | -0.49647872 |
| Klc1 | 0.46989 | 0.13027954 |
| Gm9465 | 0.09751 | -0.60201624 |
| Ror2 | 0.01784 | -0.52598916 |
| Gm4633 | 0.0038 | -1.21641907 |
| Ugt2b38 | 0.00144 | -1.50444427 |
| A230028O05Rik | 0.00144 | -1.28116048 |
| Chmp3 | 0.00145 | -1.49718739 |
| LOC102638255 | 0.00145 | -1.26219879 |
| Txnip | 0.11039 | 0.28193423 |
| Irf2bp2 | 0.0216 | -0.87570787 |
| Gm40177 | 0.00144 | -1.89303873 |
| Gm11837 | 0.11382 | 0.39302854 |
| Spp2 | 0.15528 | -2.09102466 |
| Gm36070 | 0.00144 | -1.29085005 |
| Cr1l | 0.22172 | 0.20691129 |
| Map3k3 | 0.25976 | -0.18549718 |
| Arl16 | 0.66797 | -0.06770596 |
| Tmem236 | 0.00242 | -1.19552001 |
| Slc35e1 | 0.87978 | 0.03394687 |
| 4833427F10Rik | 0.03357 | 0.64855372 |
| Hnrnpul2 | 0.71577 | -0.11821241 |
| Ninj1 | 0.55384 | -0.3457806 |
| LOC102636360 | 0.00383 | -0.81958525 |
| 1700010K24Rik | 0.04369 | -1.10808438 |
| Tlr8 | 0.00693 | 1.54118458 |
| Gm10390 | 0.0017 | -1.24950944 |
| Dnajc8 | 0.00328 | 0.81069377 |
| Flot1 | 0.85303 | 0.11818964 |
| Tcstv3 | 0.28317 | -0.55792392 |
| Ctdnep1 | 0.08629 | -0.57832404 |
| Glg1 | 0.14706 | -0.3333754 |
| Gm31131 | 0.00144 | -1.27165604 |
| LOC102635801 | 0.24805 | -0.69501203 |
| Znf512b | 0.00144 | -1.57975555 |
| LOC100862268 | 0.00144 | -1.31495282 |
| Hectd3 | 0.36248 | -0.20022319 |
| Mapkbp1 | 0.14002 | -1.23276799 |
| A630073D07Rik | 0.00145 | -1.24191372 |
| Gm32556 | 0.00472 | -1.39396076 |
| Gm13610 | 0.04552 | -2.34867765 |
| C87487 | 0.02695 | -1.81630032 |
| Gm9934 | 0.0156 | -1.08617297 |
| Ppih | 0.00492 | -0.682283 |
| AI414108 | 0.01185 | 0.54247188 |
| Tas2r103 | 0.00144 | -1.29049509 |
| Rims4 | 0.00439 | -1.24199403 |
| Gm12290 | 0.00236 | -1.13834583 |
| Prlhr | 0.00606 | -1.11156938 |
| Zfp473 | 0.09155 | -0.90326025 |
| Rbms2 | 0.1815 | -0.22191908 |
| Gm26511 | 0.28258 | -0.18942711 |
| Fgf2os | 0.00144 | -1.25118919 |
| Cct4 | 0.08294 | 0.34728935 |
| Sgcz | 0.00564 | -1.17300636 |
| Zdhhc21 | 0.83693 | 0.08617483 |
| 4930549C15Rik | 0.00144 | -1.26153594 |
| Gnl3l | 0.41375 | 0.34938987 |
| 4930433I11Rik | 0.00144 | -1.28898079 |
| Htr3a | 0.22839 | -1.17652587 |
| Gm32401 | 0.56542 | -0.15324242 |
| Trpm5 | 0.01052 | -1.06034672 |
| Gm41795 | 0.00144 | -1.33632959 |
| Olfr792 | 0.70015 | -0.0826425 |
| 1700112H15Rik | 0.05458 | -0.91197564 |
| Gm1322 | 0.00144 | -1.29746368 |
| Gm281 | 0.14521 | -0.34756689 |
| Pkp3 | 0.00951 | -1.35822768 |
| Gipc3 | 0.01733 | -0.62716831 |
| Psmd11 | 0.30762 | -0.17707098 |
| Map3k13 | 0.52317 | -0.22160475 |
| LOC105243866 | 0.18749 | -1.3566309 |
| Zfp408 | 0.51492 | -0.46526037 |
| Rpgrip1 | 0.95746 | 0.02742467 |
| 5730437C11Rik | 0.00144 | -1.24811894 |
| Apoc2 | 0.26342 | -0.55826559 |
| H2-Ob | 0.89902 | -0.04859432 |
| Proser2 | 0.19665 | 0.29733681 |
| Dcaf7 | 0.00483 | -1.3789654 |
| Sgpp2 | 0.00144 | -1.30017714 |
| Gm39273 | 0.1603 | 0.25915751 |
| Pcdh18 | 0.59775 | -0.10482196 |
| B230334C09Rik | 0.00144 | -1.29085005 |
| Ccdc101 | 0.0071 | -0.73438514 |
| 4930528G23Rik | 0.17953 | -0.40219902 |
| Hs3st6 | 0.00144 | -1.30713006 |
| Hmox2 | 0.64438 | -0.1079466 |
| Gm26876 | 0.01495 | -1.18465827 |
| 9630015K15Rik | 0.23476 | 0.49148901 |
| Rnf185 | 0.91509 | 0.08679805 |
| 4931415C17Rik | 0.00267 | -1.20283908 |
| Gm17745 | 0.01326 | 1.3675892 |
| Epb4.1l1 | 0.00191 | -1.9518151 |
| 4930567H17Rik | 0.00144 | -1.28898079 |
| Gm11985 | 0.00149 | -1.28183002 |
| Otud3 | 0.40961 | -0.15651935 |
| Ptprtos | 0.03989 | -1.03910346 |
| Gm36352 | 0.12805 | -1.16234536 |
| Gm32444 | 0.03868 | -0.89592444 |
| 1810035K13Rik | 0.00144 | -1.26934956 |
| 4631405J19Rik | 0.03971 | -0.60603027 |
| Nlrp4a | 0.00145 | -1.25646236 |
| Igfbpl1 | 0.02359 | 0.87053204 |
| Zfp963 | 0.0151 | 1.75765969 |
| Zfp110 | 0.15601 | 0.29670483 |
| Pde3b | 0.79285 | -0.13036887 |
| Gm16287 | 0.0059 | -0.84053461 |
| 1700128F08Rik | 0.00144 | -1.31884045 |
| Aldh2 | 0.67629 | -0.11492011 |
| Fam126a | 0.00294 | -1.00932308 |
| Map4k2 | 0.54446 | -0.10042661 |
| Vac14 | 0.03955 | -0.44029723 |
| Prkaa1 | 0.16304 | -0.29800173 |
| Gm16548 | 0.00144 | -1.25118919 |
| Lamc1 | 0.00145 | -1.22878849 |
| Gm15628 | 0.00198 | -1.11811652 |
| LOC105247398 | 0.04557 | -0.70338824 |
| Onecut1 | 0.00637 | -1.02877696 |
| Hist1h4h | 0.06298 | 0.38951843 |
| 2310020H05Rik | 0.18823 | -0.68806724 |
| Csn1s2b | 0.61072 | -0.31897709 |
| Mcm3 | 0.02 | -0.53903596 |
| 2810408A11Rik | 0.90172 | 0.02879477 |
| Lmx1b | 0.00144 | -1.25118919 |
| Atcayos | 0.00926 | -0.64465144 |
| Ttc33 | 0.00144 | -1.50994032 |
| A130088B03Rik | 0.90026 | -0.04997621 |
| LOC73899 | 0.13871 | 1.23374147 |
| Olfr324 | 0.08907 | -2.0246327 |
| Gabrb3 | 0.41727 | 0.40457351 |
| Gm3143 | 0.0559 | -0.90728623 |
| Gm14264 | 0.39858 | -0.52832083 |
| Defb30 | 0.00144 | -1.30017714 |
| Traip | 0.02049 | -1.50198128 |
| Dmwd | 0.42748 | -0.26786448 |
| Ddx17 | 0.78871 | -0.08804498 |
| Ctsl | 0.07227 | 0.47252936 |
| Olfr582 | 0.00144 | -1.29746368 |
| Prm3 | 0.74669 | 0.10263127 |
| Lepr | 0.00144 | -1.29878664 |
| Gm6598 | 0.08286 | -0.55432167 |
| Gm31208 | 0.11082 | -1.87110824 |
| Gm39910 | 0.0247 | -0.96581009 |
| Serpinf1 | 0.04284 | -0.53067417 |
| LOC102636700 | 0.02381 | -1.1404073 |
| Ttn | 0.54288 | 0.16214494 |
| Pnkd | 0.01798 | -0.78949616 |
| Gm10847 | 0.00244 | -1.16878422 |
| Gm4055 | 0.48878 | -0.17235381 |
| Ptprh | 0.01262 | -0.84118732 |
| Usp29 | 0.07609 | -0.66510423 |
| Gm11541 | 0.00144 | -1.25118919 |
| Cd80 | 0.01187 | 0.77817459 |
| Phyhipl | 0.01481 | -0.91763571 |
| 6530413G14Rik | 0.05452 | -0.84662487 |
| Olfr1322 | 0.00883 | -1.16882948 |
| Arsb | 0.05084 | -0.7603941 |
| Med9os | 0.08244 | -0.37333006 |
| Gm10941 | 0.01647 | -1.10379914 |
| 6330420H09Rik | 0.17333 | -0.70827904 |
| D830032E09Rik | 0.03338 | 1.58134804 |
| Naa16 | 0.24092 | 0.36426922 |
| Speer4a | 0.108 | 0.53617699 |
| Gm29707 | 0.00744 | -1.03871255 |
| Stk38l | 0.28934 | -0.26160979 |
| 4930520P13Rik | 0.00606 | -1.27303074 |
| Slc25a23 | 0.01887 | -0.81277208 |
| Fmnl2 | 0.30155 | 0.32749931 |
| Tox4 | 0.00505 | -0.73186525 |
| Gfy | 0.71853 | -0.11702742 |
| Kif3a | 0.00145 | -1.24191372 |
| Gm11210 | 0.17155 | -0.74907163 |
| A4galt | 0.0052 | -1.13545088 |
| Wipf3 | 0.00161 | -1.28508245 |
| 4933402J15Rik | 0.0018 | -1.56982499 |
| Gm4491 | 0.00144 | -1.28898079 |
| Tars2 | 0.59273 | -0.25774379 |
| Gm12295 | 0.00145 | 2.51480929 |
| Gm31763 | 0.00254 | -1.22203309 |
| Gm40882 | 0.00715 | -1.06546574 |
| 1700065O20Rik | 0.00144 | -1.31495282 |
| Psd4 | 0.00144 | -1.24894227 |
| Slc25a1 | 0.00371 | -0.80426394 |
| Gm11733 | 0.22749 | -0.98097876 |
| Gm16090 | 0.07688 | -2.37480575 |
| Zfp292 | 0.04667 | -0.57378431 |
| Slfn10-ps | 0.04597 | -0.90881164 |
| 1700051A21Rik | 0.00144 | -1.31884045 |
| LOC102642252 | 0.15841 | -0.3679381 |
| Zfp46 | 0.0304 | -0.92435468 |
| Hadhb | 0.93134 | 0.01953385 |
| 4930579P08Rik | 0.03963 | -2.12383671 |
| Nav2 | 0.3879 | -0.41735532 |
| Mmp19 | 0.05981 | -1.15718556 |
| Gm20389 | 0.00144 | 1.94555094 |
| 4921501I09Rik | 0.00145 | -1.27532353 |
| Ncor1 | 0.37147 | -0.14793663 |
| Cpeb4 | 0.01348 | -1.09140624 |
| Mug2 | 0.09471 | -0.89030722 |
| Mmp9 | 0.3493 | 0.52756027 |
| Fads2 | 0.50882 | -0.24000958 |
| Slc25a53 | 0.23635 | -0.45298628 |
| Rab23 | 0.00472 | 1.02959233 |
| Adap2os | 0.67449 | 0.33210945 |
| Nhlrc2 | 0.77653 | -0.06231679 |
| Gm31336 | 0.01767 | -1.65068818 |
| Gm30329 | 0.34362 | -0.82090225 |
| Inpp5a | 0.91062 | -0.03649907 |
| 1700054K19Rik | 0.06047 | -0.91785585 |
| Rad23b | 0.21842 | -0.27655013 |
| Slc4a8 | 0.82487 | 0.1331081 |
| Ttn | 0.02748 | 1.38739524 |
| B930099G20 | 0.00149 | -1.32850014 |
| Klk1b24 | 0.04739 | -0.7938097 |
| Gm10050 | 0.0237 | 0.7236529 |
| Gm36660 | 0.00317 | -1.11935153 |
| Mks1 | 0.21634 | 0.40163029 |
| Gdpd2 | 0.32259 | -0.67976642 |
| Theg | 0.01235 | -0.88463289 |
| Tor3a | 0.21163 | 0.44828308 |
| Capza2 | 0.02525 | 0.52825476 |
| 3110082J24Rik | 0.00211 | -1.52477175 |
| Apba1 | 0.55004 | -0.1144682 |
| Gm30564 | 0.00144 | -1.30713006 |
| Gm4224 | 0.0401 | -0.95716175 |
| A430048G15Rik | 0.00216 | -1.10905491 |
| Cela2a | 0.0112 | -0.8455366 |
| 1700084D21Rik | 0.00169 | -1.16968746 |
| 4930429C20Rik | 0.00144 | -1.28898079 |
| Mvb12b | 0.00144 | -1.12285874 |
| Olfr776 | 0.00144 | -1.31884045 |
| Hk1os | 0.00624 | -0.72027196 |
| Tbca | 0.96579 | -0.00950266 |
| Ccdc158 | 0.00144 | -1.4406427 |
| Efcab5 | 0.0744 | 0.9541321 |
| Npm1 | 0.00144 | 2.04077801 |
| C6 | 0.22518 | -0.68718489 |
| Gm5441 | 0.00144 | -1.20052149 |
| Gm34517 | 0.00208 | -1.15498993 |
| Ccdc37 | 0.32389 | -0.7670007 |
| Gm16619 | 0.05146 | -1.26799156 |
| Wbp2 | 0.04848 | -0.62946102 |
| A830019L24Rik | 0.00383 | -1.13029182 |
| Rab8b | 0.09923 | 0.48931589 |
| Ccdc59 | 0.45005 | 0.13204252 |
| Nf1 | 0.139 | -0.25115825 |
| Wdr75 | 0.00387 | 0.78449385 |
| Gm30234 | 0.07077 | -1.92665949 |
| Podnl1 | 0.57684 | -0.22727468 |
| Glb1 | 0.99219 | 0.00451787 |
| Gm6329 | 0.00772 | -1.07069847 |
| Olfr1137 | 0.99098 | -0.01463904 |
| Mtmr11 | 0.35225 | -0.4370902 |
| Ap1g1 | 0.00894 | 0.76013471 |
| Pdia3 | 0.03617 | 0.5174872 |
| Tg | 0.00633 | -1.82573544 |
| Eci3 | 0.85245 | 0.07411547 |
| Cmas | 0.00144 | -1.08909505 |
| Pigg | 0.03028 | -0.67011392 |
| D030024E09Rik | 0.0573 | -1.83671475 |
| Gsap | 0.28135 | 0.70717761 |
| Clcn5 | 0.04221 | -0.77046581 |
| Olfr69 | 0.00159 | -1.26431393 |
| Trim56 | 0.02849 | -1.01814926 |
| 4930453N24Rik | 0.02463 | 1.00142168 |
| Gm10305 | 0.06471 | -0.61387924 |
| 2610037D02Rik | 0.00145 | -1.24191372 |
| Rell1 | 0.00144 | -2.32690035 |
| Gm6225 | 0.32082 | -0.58038446 |
| Alg5 | 0.77194 | -0.05546664 |
| Gm11944 | 0.1606 | 0.35398858 |
| Gm6567 | 0.86467 | 0.07045187 |
| Gpr137c | 0.81674 | -0.11862514 |
| 4930406D18Rik | 0.02147 | -1.06017214 |
| Phldb3 | 0.13209 | -0.63333982 |
| Gm2011 | 0.4953 | 0.15016027 |
| Commd1 | 0.24522 | -2.30046776 |
| Cldn20 | 0.78805 | 0.09879817 |
| AU019823 | 0.21145 | -0.47479413 |
| Atp5o | 0.00201 | -0.87435282 |
| Gm5174 | 0.0029 | -1.20702967 |
| Unc5c | 0.00144 | -1.29085005 |
| Ngb | 0.44567 | -0.1467379 |
| Svet1 | 0.01162 | -1.21461873 |
| Gale | 0.06031 | -0.69990539 |
| L1Md-Tf29 | 0.00144 | 1.90642397 |
| Gm38569 | 0.074 | -0.37486748 |
| Hyls1 | 0.62413 | 0.15697089 |
| Rfx1 | 0.78831 | -0.0976406 |
| 4933417A18Rik | 0.09958 | -0.79271211 |
| Gm11525 | 0.0476 | -1.24266807 |
| Pnkp | 0.01453 | -0.78592288 |
| Kif2c | 0.00144 | -1.29746368 |
| Ctnna2 | 0.01022 | -0.9045813 |
| Rars | 0.70194 | -0.07089778 |
| Gm34184 | 0.04539 | -0.86550211 |
| Gm28979 | 0.11067 | 1.2807283 |
| Gm7644 | 0.00144 | -1.30017714 |
| Shox2 | 0.41419 | -0.43282437 |
| Rnf44 | 0.02939 | -0.97205614 |
| 2810468N07Rik | 0.00649 | -0.78900112 |
| Gm3896 | 0.08855 | -0.62041657 |
| Gm14124 | 0.00144 | 2.61246078 |
| Pou6f1 | 0.33628 | -1.07901307 |
| Acbd6 | 0.0026 | -0.77539015 |
| Tctn1 | 0.04241 | -0.42374209 |
| 1700008C04Rik | 0.00144 | -1.28116048 |
| Gm15510 | 0.01728 | -0.54456797 |
| Gm11538 | 0.66674 | -0.28282267 |
| Lekr1 | 0.02138 | 0.66356259 |
| Birc6 | 0.05658 | 0.50143929 |
| Gm14642 | 0.00144 | -1.62591475 |
| Crhr2 | 0.04915 | -0.53066231 |
| Gm16253 | 0.00144 | -2.11423551 |
| Gm12511 | 0.00144 | -1.25927895 |
| 4921518K17Rik | 0.41 | -0.34064756 |
| Hepacam2 | 0.28759 | -0.31419618 |
| Gm32511 | 0.00144 | -1.28116048 |
| Asic3 | 0.01377 | 1.91786289 |
| Hist2h2ac | 0.00144 | -1.3727256 |
| Gm32849 | 0.81779 | 0.13253005 |
| Tdp1 | 0.00144 | -1.49973687 |
| Gm26756 | 0.49151 | -0.48185315 |
| Rps3 | 0.012 | -0.78767967 |
| Fam183b | 0.11025 | -2.96355961 |
| 1700012O15Rik | 0.00144 | -1.22465584 |
| Hrh3 | 0.00144 | -1.3319652 |
| 1700074H08Rik | 0.00144 | -1.24613586 |
| Gm41386 | 0.00145 | -1.2388121 |
| Gabrg3 | 0.09648 | -1.62650157 |
| Gm12223 | 0.00144 | -1.31495282 |
| LOC101055630 | 0.00144 | -1.27165604 |
| Nasp | 0.05186 | 0.90321211 |
| Gm29398 | 0.00261 | -1.11284335 |
| C2cd3 | 0.91355 | -0.17398423 |
| Cast | 0.05022 | 0.79316225 |
| Rab26os | 0.00354 | -0.78140887 |
| Dmrta2os | 0.00144 | -1.27165604 |
| Gm13869 | 0.0063 | -1.45898328 |
| Gm10482 | 0.114 | -0.53219431 |
| 2610002J02Rik | 0.00612 | -0.71162154 |
| Mtus2 | 0.92596 | 0.06171525 |
| Olfr1089 | 0.00144 | -1.28116048 |
| Nup133 | 0.61438 | -0.17715028 |
| Taf10 | 0.04439 | -0.61682005 |
| Olfr1006 | 0.32686 | -0.49214601 |
| Frmd4a | 0.00144 | -1.29085005 |
| 5430416N02Rik | 0.00766 | -1.12466538 |
| Gprin2 | 0.0578 | -1.19026424 |
| 2900076A07Rik | 0.00571 | 0.69577314 |
| Cdc25c | 0.66976 | 0.21505979 |
| Synpo | 0.06615 | -0.72962598 |
| Kcnq5 | 0.04532 | -1.35103711 |
| Cyp2d9 | 0.00291 | -1.6991851 |
| Ralgapa1 | 0.00309 | 1.14116415 |
| L3mbtl1 | 0.0242 | -1.03617482 |
| Mipep | 0.50648 | -0.15476421 |
| Krt222 | 0.00144 | -1.24811894 |
| Aldh1l1 | 0.51205 | -0.39272496 |
| Ice1 | 0.04174 | -0.80184128 |
| Gm6306 | 0.01814 | -0.9096825 |
| Adgrf5 | 0.2942 | -0.26144743 |
| 4930544I03Rik | 0.02754 | -1.27328196 |
| Usmg5 | 0.26635 | -0.41524008 |
| Gm19990 | 0.00144 | -1.28116048 |
| Hacl1 | 0.50738 | 0.12659081 |
| Numbl | 0.00167 | -0.99759269 |
| Rnf165 | 0.20718 | 0.5787149 |
| Gm38736 | 0.00144 | -1.26560808 |
| Herc4 | 0.00144 | 1.47498423 |
| Chd7 | 0.35658 | -0.43536771 |
| 7530416G11Rik | 0.00144 | -1.27165604 |
| 9330175E14Rik | 0.17775 | -0.38057604 |
| Anks1b | 0.32625 | -0.5607423 |
| Pax5 | 0.00389 | -1.36404709 |
| Card9 | 0.01104 | -1.31646818 |
| AA474331 | 0.90277 | 0.18269837 |
| Gm13262 | 0.00451 | -0.66592845 |
| AA415398 | 0.102 | -1.08072897 |
| Scamp3 | 0.73768 | -0.17401564 |
| Gm15395 | 0.00144 | -1.25118919 |
| Gm1045 | 0.00144 | -1.28898079 |
| LOC102636989 | 0.00144 | -1.28116048 |
| Pydc3 | 0.0904 | 0.54869812 |
| 1700009C05Rik | 0.0366 | -2.0146614 |
| 4933439K11Rik | 0.226 | -0.39206133 |
| Adarb2 | 0.95134 | -0.09376204 |
| Kcnj15 | 0.48754 | 0.33206687 |
| Rnf144b | 0.73408 | 0.06615157 |
| BC106175 | 0.34548 | -0.47167917 |
| 2310034O05Rik | 0.01974 | -0.52733785 |
| Psd4 | 0.07492 | -0.74639596 |
| Dab2ip | 0.00144 | -1.30349246 |
| Nup43 | 0.11724 | 0.40836089 |
| Adcyap1 | 0.00144 | -1.26042915 |
| Pex26 | 0.13129 | -0.45466795 |
| Ttc38 | 0.03675 | -0.51787846 |
| Trim9 | 0.00873 | -1.13435495 |
| Ppox | 0.37189 | -0.17907902 |
| Cox4i2 | 0.00256 | -0.76714521 |
| Gm26793 | 0.00144 | -1.31884045 |
| Fut11 | 0.50261 | 0.1301767 |
| 9430093N23Rik | 0.01737 | -1.05152568 |
| Gm41408 | 0.24055 | -0.26293991 |
| Gm33619 | 0.00144 | -1.28116048 |
| Rpl9 | 0.79733 | -0.06247021 |
| Tomt | 0.02507 | -0.8750453 |
| C030023E24Rik | 0.00144 | -1.20555712 |
| Pdzk1ip1 | 0.39773 | -0.36551106 |
| Enpp2 | 0.65339 | -0.17322534 |
| Gm28777 | 0.00459 | -1.52614275 |
| Gm36888 | 0.00145 | -1.22376446 |
| Dis3l2 | 0.00144 | -1.33059316 |
| Olfr1031 | 0.01955 | -1.18606919 |
| 9330117O12Rik | 0.01235 | -1.14224876 |
| Slfn5 | 0.13346 | 0.29177944 |
| Ptprd | 0.0331 | 0.7685803 |
| A130094D17Rik | 0.04227 | -1.07671797 |
| 1700066B17Rik | 0.06162 | -1.46712957 |
| Platr9 | 0.40091 | 0.1918949 |
| Sptbn4 | 0.0028 | -1.05366396 |
| Gm31160 | 0.00144 | -1.28898079 |
| Atp8a1 | 0.9172 | 0.02273389 |
| Bend7 | 0.36995 | -0.79635146 |
| Gm15545 | 0.42635 | -0.12517933 |
| Rimbp3 | 0.98468 | -0.00383188 |
| BB094273 | 0.11253 | 0.60127175 |
| Kit | 0.02481 | 0.62869315 |
| Ifna15 | 0.0096 | -0.8986266 |
| Fam122b | 0.03557 | -1.24701501 |
| Trpc5 | 0.15969 | -2.25086523 |
| A930024N18Rik | 0.42205 | -0.67670754 |
| Trappc2 | 0.00484 | 0.93582798 |
| Fxyd6 | 0.62118 | -0.22502935 |
| Zfyve1 | 0.01465 | -0.50928362 |
| Gtf2a1 | 0.00149 | 1.12117302 |
| Ptcra | 0.06457 | -1.26492906 |
| Med16 | 0.02184 | -0.88541758 |
| Entpd7 | 0.02138 | -1.0980998 |
| Slc5a11 | 0.01181 | -1.1280166 |
| Slc4a8 | 0.80615 | 0.0564261 |
| Slc9a8 | 0.81097 | 0.04561707 |
| Prom1 | 0.00144 | -1.30713006 |
| Mtmr2 | 0.02955 | 0.45750294 |
| Atp5f1 | 0.16054 | 0.48571723 |
| Olfr1193 | 0.00144 | -1.26560808 |
| Fggy | 0.00144 | -1.30017714 |
| LOC105244413 | 0.00144 | -1.46059356 |
| Cse1l | 0.02878 | 0.84509747 |
| Sec14l3 | 0.00144 | -1.24353547 |
| 4930417O13Rik | 0.00144 | -1.29085005 |
| Fam109b | 0.00212 | -0.88718771 |
| AA623943 | 0.00605 | -1.08150631 |
| Gm26777 | 0.93737 | 0.01618538 |
| Gm17455 | 0.01967 | -1.06407376 |
| Nrk | 0.01282 | 0.93259632 |
| Olfr1242 | 0.96575 | 0.01774016 |
| Zc3hc1 | 0.57015 | -0.2079915 |
| 1600019K03Rik | 0.00144 | -1.66516151 |
| 2410127L17Rik | 0.00144 | -1.24673457 |
| Eef1a1 | 0.39874 | -0.19063511 |
| A930012N16Rik | 0.00144 | -1.27165604 |
| Fzd5 | 0.47415 | -0.18610463 |
| Zfp735 | 0.00145 | -1.25646236 |
| C7 | 0.17733 | -0.47764788 |
| Atad2 | 0.45365 | 0.43466114 |
| D230022J07Rik | 0.04335 | -0.8262792 |
| Gm1647 | 0.04982 | -3.53890873 |
| Smtn | 0.11925 | -0.38898719 |
| Plekhd1 | 0.01736 | -0.53716239 |
| Trpt1 | 0.00394 | -1.14470896 |
| Mettl16 | 0.46973 | -0.12857796 |
| 4930440I19Rik | 0.42272 | -0.61770108 |
| Slc13a2os | 0.00144 | -1.24613586 |
| Olfr65 | 0.14239 | 0.8428636 |
| Pdlim1 | 0.5212 | 0.26372944 |
| Abi3bp | 0.1855 | -0.38977696 |
| Gdap1 | 0.00144 | -1.57547831 |
| Krt8 | 0.04827 | -1.07652729 |
| Gm518 | 0.00651 | -1.53264006 |
| D330046F09Rik | 0.00144 | -1.29746368 |
| AI846148 | 0.71604 | -0.07362451 |
| Gm38572 | 0.00158 | -1.2446827 |
| 1700101O22Rik | 0.00144 | -1.30017714 |
| Gm3300 | 0.00144 | -1.48248633 |
| Dct | 0.00437 | -0.8025649 |
| Gm12603 | 0.0063 | -1.45898328 |
| Prox2 | 0.4102 | 0.32818833 |
| Slc8b1 | 0.00672 | -1.01585177 |
| D830036C21Rik | 0.07419 | -0.68274928 |
| 9430041J12Rik | 0.00395 | -1.19099662 |
| Hoxb5os | 0.00144 | -1.33361546 |
| Hist2h3c2 | 0.21424 | -0.5298156 |
| Upp1 | 0.00573 | -0.94985923 |
| Bcat1 | 0.69349 | 0.11426009 |
| Vmn1r185 | 0.00746 | -1.57369717 |
| Slc25a34 | 0.37243 | -0.25341022 |
| 2210404O09Rik | 0.01041 | 1.27816496 |
| Gm2560 | 0.00144 | -1.30017714 |
| Gm41830 | 0.03824 | 0.94307415 |
| Lim2 | 0.00144 | -1.28898079 |
| Gm16252 | 0.43479 | 0.21970385 |
| 4933434M16Rik | 0.00144 | -1.31884045 |
| 8030451O07Rik | 0.00144 | -1.27992547 |
| Ilkap | 0.02938 | -0.44341608 |
| Mecr | 0.85075 | -0.07315608 |
| Nrxn1 | 0.00144 | -1.29085005 |
| Gm19510 | 0.48967 | 0.21891909 |
| Clcn4-2 | 0.06527 | 0.41068235 |
| Gm9519 | 0.00144 | -1.24613586 |
| Tnk1 | 0.55734 | -0.2834634 |
| Gm26592 | 0.00144 | -1.29878664 |
| BC030343 | 0.19853 | -0.74203372 |
| Oscp1 | 0.95515 | -0.03741921 |
| 2210411M09Rik | 0.31676 | -0.29949119 |
| Smpd5 | 0.00144 | -1.29085005 |
| Lhfpl5 | 0.00144 | -1.28898079 |
| Bdkrb2 | 0.10166 | -0.70750205 |
| Sptlc2 | 0.09865 | 0.72921972 |
| 4921533I20Rik | 0.00144 | -1.29085005 |
| Itgad | 0.0251 | -1.20164736 |
| Nudcd1 | 0.00144 | -1.28898079 |
| Hnrnpu | 0.0093 | 0.6401442 |
| Tomm6os | 0.36793 | 0.36706718 |
| Gm15713 | 0.00145 | -1.22376446 |
| Brca1 | 0.23723 | -0.3737334 |
| Rd3l | 0.0115 | -1.63432972 |
| Ptger3 | 0.01499 | -1.53292277 |
| B930071A02 | 0.00144 | -1.26934956 |
| Senp1 | 0.85057 | 0.04225829 |
| Cnksr1 | 0.03622 | -0.73456763 |
| Olfr206 | 0.00144 | -1.25118919 |
| Frrs1 | 0.04207 | -1.26052549 |
| AV064505 | 0.06922 | -1.41265781 |
| Prr14l | 0.93803 | 0.03761352 |
| Gm32643 | 0.38556 | 0.41328474 |
| Gm156 | 0.00144 | -1.31495282 |
| Echs1 | 0.00144 | -1.35970939 |
| Taar7f | 0.00144 | -1.27165604 |
| 4833438C02Rik | 0.00737 | -0.64756151 |
| Rabepk | 0.58266 | -0.37033214 |
| Sult5a1 | 0.00939 | -1.07004417 |
| Gm32699 | 0.10879 | 0.84425177 |
| 2810471M01Rik | 0.07745 | -0.35141313 |
| Magea10 | 0.14028 | -0.79695848 |
| Gm11770 | 0.03947 | -1.1058042 |
| Mgat5 | 0.25097 | 0.2849426 |
| Helz | 0.73195 | -0.06230134 |
| 4933430M04Rik | 0.37684 | -0.65572287 |
| Ifna12 | 0.00144 | -1.29864962 |
| Nxf1 | 0.02031 | 0.64271322 |
| Gm4632 | 0.37463 | -0.63199684 |
| Olfr663 | 0.00144 | -1.29878664 |
| Lix1l | 0.09473 | -0.31210993 |
| Clspn | 0.04167 | -1.60901501 |
| Parpbp | 0.00225 | -1.40363226 |
| Vmn1r4 | 0.00149 | -1.22582153 |
| Abcf3 | 0.16958 | -0.26364106 |
| Ism2 | 0.23343 | -0.53740849 |
| 6030408B16Rik | 0.00655 | -0.9438718 |
| Gm5860 | 0.38345 | 0.37151547 |
| Ppp4r2 | 0.70875 | 0.06407268 |
| Zfp652 | 0.45494 | 0.12175969 |
| Gm15816 | 0.0609 | -0.69879402 |
| Pdzk1 | 0.00144 | -1.31884045 |
| Hk3 | 0.01536 | -0.73379906 |
| Tial1 | 0.1641 | 0.40391335 |
| Tnni1 | 0.01295 | -1.23970419 |
| 3110015C05Rik | 0.10226 | -3.26022899 |
| D330023K18Rik | 0.50672 | 0.25015148 |
| Abca16 | 0.00145 | -1.2388121 |
| Gm28209 | 0.02848 | -1.57032346 |
| Gm38695 | 0.00144 | -1.30017714 |
| Arntl | 0.25266 | -0.60700995 |
| A530046M15Rik | 0.00144 | -1.31884045 |
| Krtcap2 | 0.00556 | -0.90308661 |
| Tulp1 | 0.08354 | -0.58918464 |
| Ascl2 | 0.12945 | -0.80890014 |
| Atxn10 | 0.09832 | 1.11525698 |
| Gm31075 | 0.00144 | -1.21977358 |
| Tle2 | 0.00722 | 0.60317413 |
| Gm26550 | 0.00144 | -1.30713006 |
| Gm30906 | 0.00145 | -1.22376446 |
| 7420426K07Rik | 0.08859 | -2.63183718 |
| Col6a3 | 0.00145 | -1.35466696 |
| Clec2d | 0.04201 | -0.69982221 |
| Rad52 | 0.16413 | 0.3501002 |
| Ing3 | 0.00153 | 0.94263528 |
| Gm28181 | 0.0017 | -1.24950944 |
| Gm35501 | 0.26495 | -0.65711895 |
| Spopl | 0.00144 | 2.13433075 |
| Npm3 | 0.13442 | -0.45676027 |
| 4930426I24Rik | 0.38769 | 0.37030494 |
| Zfat | 0.0332 | -0.78427281 |
| Mina | 0.11388 | 0.33601007 |
| 1700101E01Rik | 0.00144 | -1.28116048 |
| Ube2e3 | 0.11374 | 0.72126605 |
| Gm14169 | 0.34081 | -0.52785405 |
| Glrx3 | 0.33607 | 0.81528658 |
| Gm20647 | 0.00324 | 2.55199121 |
| Gm34055 | 0.00144 | -1.26911831 |
| Ankrd16 | 0.00144 | 1.08897009 |
| LOC328448 | 0.05528 | -0.95718388 |
| 5730405O15Rik | 0.00832 | 1.14580177 |
| Eif2b3 | 0.0204 | 0.77101516 |
| Gm38419 | 0.01229 | -0.87416673 |
| Fuca1 | 0.0359 | -0.43848314 |
| C230099D08Rik | 0.00144 | -1.30017714 |
| Gm30439 | 0.00144 | -1.29049509 |
| Tom1 | 0.01157 | -0.72238213 |
| Tnfsf8 | 0.02937 | 0.77192023 |
| Gm2568 | 0.78543 | -0.12529158 |
| 4930553C11Rik | 0.00144 | -1.30713006 |
| Gdf5 | 0.96216 | 0.02215152 |
| Fmo9 | 0.00201 | -1.41000187 |
| LOC102632465 | 0.0179 | -1.5479068 |
| G630064G18Rik | 0.04547 | 1.00693667 |
| Olfr954 | 0.04982 | -1.46464312 |
| 5830433I10Rik | 0.005 | -1.04448964 |
| Gm16160 | 0.00145 | -1.31786111 |
| Pigq | 0.00798 | -0.69892035 |
| Polr1c | 0.16682 | -0.46345975 |
| Endov | 0.06986 | -0.83011923 |
| Mbd3 | 0.0034 | -0.84589547 |
| Arhgap27os1 | 0.01484 | -0.88626496 |
| Gm12273 | 0.15964 | -0.33844932 |
| Gm10523 | 0.00776 | -0.71871138 |
| 2700070H01Rik | 0.03588 | -1.02614883 |
| Cbfa2t2 | 0.7377 | 0.1173777 |
| Dock9 | 0.01044 | -0.55024627 |
| 4930548K13Rik | 0.00144 | -1.31884045 |
| Olfr1335 | 0.51328 | 0.16454911 |
| 2810449G22Rik | 0.36293 | 0.45786615 |
| Olfr1082 | 0.00144 | -1.27165604 |
| Ncstn | 0.0197 | -0.63419991 |
| 8430423G03Rik | 0.29601 | -0.58093549 |
| Gm26663 | 0.12389 | -1.14491598 |
| Gm3435 | 0.35787 | 0.2304018 |
| D730003K21Rik | 0.83047 | 0.13580811 |
| Tppp3 | 0.00189 | -1.10567046 |
| Zswim4 | 0.08692 | -0.68486499 |
| Cfap61 | 0.0046 | -1.12385295 |
| Vmn1r214 | 0.60619 | -0.2383131 |
| Gm33790 | 0.92498 | -0.04529988 |
| Gm40275 | 0.00144 | -1.3348153 |
| Supv3l1 | 0.43876 | 0.26712177 |
| Gm5635 | 0.13927 | 0.45196928 |
| Gm29927 | 0.00144 | -1.27699683 |
| AA415398 | 0.02803 | 0.74553051 |
| Zbtb44 | 0.32781 | 0.23583927 |
| Fam92b | 0.00246 | -1.81936874 |
| 2010111I01Rik | 0.02122 | -0.96350362 |
| Gm10851 | 0.15326 | -0.99863187 |
| Gm16499 | 0.006 | -0.62818603 |
| Gm13025 | 0.43783 | 0.13390669 |
| L3mbtl1 | 0.22794 | -0.64776933 |
| Cops8 | 0.0313 | -0.79809298 |
| Chid1 | 0.26875 | -0.36167528 |
| Gm4792 | 0.08556 | -0.87755765 |
| Wfdc16 | 0.19453 | -0.32641381 |
| Gm20750 | 0.06446 | -1.44022024 |
| Gm32391 | 0.06905 | 0.45708527 |
| Rdm1 | 0.32122 | -0.49870653 |
| Mpz | 0.12852 | -1.27409756 |
| Dhdh | 0.52928 | 0.17028345 |
| Dhrs4 | 0.67879 | -0.11918838 |
| Mboat4 | 0.00144 | -1.27165604 |
| Cttn | 0.1094 | 1.20143588 |
| 1700080G11Rik | 0.00356 | -1.08631 |
| Grm7 | 0.00144 | -1.30713006 |
| 4732414G09Rik | 0.62203 | -0.37547266 |
| Gm19651 | 0.1437 | 0.66714415 |
| Cdk5rap3 | 0.2551 | -0.33080459 |
| Pax9 | 0.17654 | -0.52140131 |
| Casr | 0.00145 | -1.2388121 |
| Olfr1461 | 0.10983 | -2.98589416 |
| Car8 | 0.00283 | 1.23885705 |
| Stk32a | 0.95114 | -0.03731109 |
| 6030442E23Rik | 0.00735 | -2.66274699 |
| AY339874 | 0.00144 | -1.27165604 |
| Tmem181a | 0.01228 | 1.02976396 |
| U2surp | 0.39105 | 0.47278601 |
| Gm17115 | 0.16083 | -2.39782658 |
| Gm32352 | 0.05878 | -0.71618786 |
| Prr14l | 0.09414 | 0.65242314 |
| Gm8787 | 0.00144 | -1.2261378 |
| 2310043L19Rik | 0.00144 | -1.58116948 |
| Alg13 | 0.00646 | 1.96103081 |
| Tmem51os1 | 0.19276 | -0.43460531 |
| Phb2 | 0.5577 | -0.28229119 |
| Gm31786 | 0.42138 | 0.24667926 |
| Ky | 0.01322 | -1.29333807 |
| Trabd2b | 0.16553 | -0.27664928 |
| Smtnl2 | 0.0166 | -0.78055829 |
| Brk1 | 0.62089 | -0.16742921 |
| Trmt1l | 0.00934 | -0.69342443 |
| Gm13861 | 0.00145 | -1.50871772 |
| Vps13a | 0.01706 | 0.5875789 |
| 9630028H03Rik | 0.00144 | -1.31518407 |
| 4930500L23Rik | 0.00326 | -1.1259681 |
| Gm13429 | 0.10553 | -0.70214069 |
| Gm16833 | 0.00144 | -1.30935626 |
| Rufy2 | 0.10276 | 0.51704926 |
| Dand5 | 0.66081 | -0.14634667 |
| Obox1 | 0.3085 | 0.60111907 |
| Gm32975 | 0.00144 | -1.25118919 |
| Celf6 | 0.01144 | -2.31202941 |
| Mpc1 | 0.01664 | 1.59854314 |
| Gm20655 | 0.01041 | 1.24228539 |
| Gm11426 | 0.00144 | -1.29049509 |
| Gm8013 | 0.23974 | 0.67723629 |
| Prkcg | 0.49526 | -0.93241217 |
| Gm28505 | 0.91798 | -0.04386162 |
| Ano7 | 0.72242 | -0.08330941 |
| Gm29682 | 0.00144 | -1.31495282 |
| Gm14707 | 0.00144 | -1.20555712 |
| 9830137J09Rik | 0.00158 | -1.2446827 |
| BC030870 | 0.64508 | -0.14274607 |
| 4930524O05Rik | 0.26064 | -0.26286658 |
| 4930563M20Rik | 0.00592 | -1.07650415 |
| Ccng2 | 0.0062 | 1.20438425 |
| Zfp703 | 0.00144 | -1.47629713 |
| 9230102O04Rik | 0.00145 | 2.02872412 |
| Mrpl48 | 0.01737 | 0.61763397 |
| Zfp64 | 0.00258 | -0.98958607 |
| Rhof | 0.03134 | -0.54402965 |
| Mrpl47 | 0.89431 | -0.02333137 |
| Nmnat2 | 0.53072 | -0.39976331 |
| Itgb3 | 0.1236 | -0.63667677 |
| Drd4 | 0.12499 | -3.03654058 |
| 4933406I18Rik | 0.00149 | -1.19348234 |
| Olfr328 | 0.26746 | -0.70032742 |
| BC024978 | 0.00198 | -0.93056802 |
| 4921517D16Rik | 0.09406 | 0.84731654 |
| Sf3a2 | 0.00544 | -0.71217 |
| Olfr43 | 0.00144 | -1.28116048 |
| Hs2st1 | 0.90195 | -0.02378863 |
| Gm34168 | 0.01946 | -1.02877696 |
| Tfeb | 0.08265 | -0.67975484 |
| Tarsl2 | 0.00144 | -1.30713006 |
| Cd300lf | 0.48328 | -0.44066848 |
| Aqp9 | 0.01635 | -0.54544679 |
| Zcchc9 | 0.03606 | 0.44853914 |
| D530049I02Rik | 0.00457 | -1.1369777 |
| Ttk | 0.10182 | 0.69669056 |
| Tubb1 | 0.00144 | -1.43031347 |
| Kcnd1 | 0.06615 | -0.81320771 |
| Flt3 | 0.00484 | -0.93933546 |
| 1700011M02Rik | 0.00144 | -1.30713006 |
| Trappc3l | 0.00144 | -1.26560808 |
| Spink5 | 0.07702 | -1.10630805 |
| Gm11998 | 0.00149 | -1.25408 |
| C130073E24Rik | 0.14925 | -0.90240608 |
| 4933407E24Rik | 0.00261 | -1.11284335 |
| Pycrl | 0.02019 | -0.85763328 |
| Gm11454 | 0.94273 | -0.03168942 |
| Gm14015 | 0.15782 | -1.41519544 |
| Rpl3 | 0.38563 | -0.18904648 |
| 1700010I02Rik | 0.00221 | -1.77231187 |
| 4930459L07Rik | 0.00359 | -2.28874954 |
| Pygm | 0.84291 | -0.12223104 |
| Ifitm10 | 0.05867 | -1.17683824 |
| 1700074I03Rik | 0.00144 | -1.29049509 |
| 1700025N23Rik | 0.09021 | -2.57268006 |
| Gm12 | 0.35862 | 0.1637339 |
| Cass4 | 0.04978 | -0.96247806 |
| 1700019G24Rik | 0.00144 | -1.28116048 |
| Tap1 | 0.01047 | -0.97668953 |
| Cntn2 | 0.75998 | -0.16519893 |
| Dync1li1 | 0.06846 | 0.57445429 |
| Gm11476 | 0.17367 | -0.57697193 |
| Hdc | 0.05024 | -0.85431206 |
| Tlr13 | 0.26729 | -1.00315466 |
| Setd8 | 0.62356 | 0.14946853 |
| Hrh1 | 0.00144 | -1.31495282 |
| D830035M03Rik | 0.34228 | -0.81988803 |
| A930005H10Rik | 0.89974 | -0.05078269 |
| Gcfc2 | 0.19496 | 0.66499423 |
| Chodl | 0.03804 | -1.96301454 |
| Bard1 | 0.13452 | 0.56586242 |
| Vmn2r37 | 0.00144 | -1.26560808 |
| Atp13a1 | 0.03945 | -0.49739794 |
| Pnn | 0.00298 | 1.21340635 |
| LOC102632664 | 0.20444 | -0.38676369 |
| A430027H14Rik | 0.44088 | 0.31575784 |
| Gm30794 | 0.4145 | -0.57164643 |
| Gm11292 | 0.00144 | -1.29049509 |
| Plg | 0.00144 | -1.46015036 |
| Gm26911 | 0.90519 | 0.094525 |
| Slc43a2 | 0.00149 | -1.03427714 |
| Gm3942 | 0.30399 | -0.89292875 |
| Hist2h3c1 | 0.21025 | 0.26185497 |
| Ube2uos | 0.15938 | -0.91230504 |
| Snd1 | 0.21196 | -0.63560665 |
| Tcte2 | 0.00144 | -1.26560808 |
| Catsper2 | 0.00144 | 1.07910867 |
| Ripply2 | 0.08233 | -0.5594168 |
| Sh3glb1 | 0.35725 | 0.21824556 |
| Catsper3 | 0.01055 | -1.62067246 |
| Gm19619 | 0.00144 | -1.24613586 |
| Kif26a | 0.00145 | -1.10372466 |
| Cyb5a | 0.13617 | -0.27555046 |
| D930032P07Rik | 0.00166 | -1.21062785 |
| Gm4673 | 0.00144 | -1.42896738 |
| LOC105242387 | 0.59524 | -0.3068831 |
| Muc1 | 0.59681 | 0.2031324 |
| Nde1 | 0.00798 | -0.90752666 |
| Gm28800 | 0.04895 | -1.01963123 |
| Bptf | 0.00144 | 2.18798379 |
| Rsph3a | 0.06878 | -0.47365873 |
| Slc34a2 | 0.01891 | -1.30732989 |
| 1700008F21Rik | 0.102 | -0.77524994 |
| Gm38554 | 0.00144 | -1.28898079 |
| 4930573C15Rik | 0.80597 | 0.09039975 |
| 2410127L17Rik | 0.0015 | 0.89149801 |
| Maged1 | 0.26414 | -0.31186817 |
| 4930550C17Rik | 0.02906 | -0.75881559 |
| 4933427J07Rik | 0.00145 | -1.2388121 |
| H2afy2 | 0.54727 | -0.1056162 |
| Six3os1 | 0.00144 | -1.29051721 |
| Gpc5 | 0.00144 | -1.29746368 |
| Ppp1r10 | 0.0045 | -0.85996408 |
| Lctl | 0.02742 | -2.17453372 |
| Gm12992 | 0.37792 | -0.40172386 |
| Olfr517 | 0.1249 | -3.16455595 |
| Vkorc1l1 | 0.82121 | -0.05324927 |
| 4930465K09Rik | 0.00149 | -1.2703832 |
| Cyb5r3 | 0.78113 | -0.13542834 |
| Cpxcr1 | 0.05318 | -1.44258181 |
| Rpl30 | 0.01292 | -0.5098162 |
| Nhs | 0.84182 | -0.08958269 |
| Zfp575 | 0.00144 | -2.29235939 |
| LOC100503048 | 0.02694 | -1.36347569 |
| Olfr1272 | 0.07021 | -0.41500016 |
| 4930447M23Rik | 0.00144 | -1.27165604 |
| Ino80d | 0.00958 | -0.8532965 |
| Chm | 0.02762 | 0.69833256 |
| Mettl5 | 0.00381 | 1.16005536 |
| 3100003L05Rik | 0.00673 | -1.35122687 |
| Atp8b4 | 0.34675 | 0.20098414 |
| Stard6 | 0.00144 | 1.95152488 |
| Vmn1r10 | 0.00144 | -1.29746368 |
| 1700063D05Rik | 0.00174 | -1.21911628 |
| Ddx42 | 0.42093 | 0.44534422 |
| 4933401L05Rik | 0.00144 | -1.37531994 |
| Taf12 | 0.02824 | -0.43264014 |
| Dhodh | 0.54889 | 0.20373328 |
| B230303O12Rik | 0.03951 | 0.97916008 |
| Dlg5 | 0.04779 | -0.55931706 |
| Atp5b | 0.31392 | -0.31181401 |
| Cfap44 | 0.00145 | -1.25646236 |
| 9330158H04Rik | 0.02645 | 0.50365397 |
| Srgn | 0.00296 | -1.40718181 |
| Helq | 0.00481 | 0.86910939 |
| Mapk15 | 0.86425 | -0.09189754 |
| A930003O13Rik | 0.4312 | -1.53506671 |
| 2900076A07Rik | 0.12877 | 0.47571326 |
| Epgn | 0.00144 | -1.27165604 |
| Il1rapl2 | 0.00184 | -1.19286777 |
| Cox14 | 0.01301 | -0.90445591 |
| Prr33 | 0.00783 | -0.76055601 |
| Nob1 | 0.04227 | -0.47617799 |
| Plod1 | 0.08989 | -0.38962717 |
| Mapk1ip1l | 0.05183 | 0.36157249 |
| Abca4 | 0.22258 | -0.3476328 |
| 4933428C19Rik | 0.00145 | -1.26219879 |
| Arid4b | 0.0027 | 2.4236067 |
| Notum | 0.10901 | -0.50191169 |
| Gm13749 | 0.00145 | -1.24191372 |
| 4931418L13Rik | 0.06643 | -1.17438574 |
| 4931408C20Rik | 0.14804 | -0.98973449 |
| Smtnl2 | 0.15909 | -0.53118416 |
| R3hdml | 0.39273 | -0.43130457 |
| Aip | 0.02963 | -0.82048873 |
| 1700057H15Rik | 0.01943 | -0.80561887 |
| Hnf1aos1 | 0.08501 | -2.66875127 |
| Tmtc4 | 0.01014 | -0.70318051 |
| Lppos | 0.21947 | -2.14122735 |
| Gm36584 | 0.09495 | -0.82135365 |
| Capn9 | 0.04192 | 0.76628149 |
| Gm33543 | 0.72123 | 0.06941211 |
| Dcx | 0.00268 | -1.36211029 |
| 4930551E15Rik | 0.59279 | -0.22168347 |
| Gm38483 | 0.08039 | -1.20632717 |
| 0610005C13Rik | 0.00144 | -1.47167917 |
| 2310033P09Rik | 0.03192 | -1.12948231 |
| Hhat | 0.43909 | -0.26854065 |
| Nppb | 0.01686 | -1.06165009 |
| Pdha2 | 0.0049 | -1.08617297 |
| Nadk2 | 0.00364 | 1.03694524 |
| Gm13425 | 0.10246 | -0.74317082 |
| Pde12 | 0.00144 | 1.07609342 |
| Alg1 | 0.02069 | -0.6227019 |
| Zfa-ps | 0.00144 | -1.30017714 |
| Duxbl2 | 0.38622 | -0.36234572 |
| Gm16083 | 0.01135 | -1.6853254 |
| Gm9648 | 0.63513 | -0.11514428 |
| Prc1 | 0.45859 | 0.87560059 |
| Slc16a9 | 0.74121 | -0.17021332 |
| AI481877 | 0.59896 | -0.25589553 |
| A930009A15Rik | 0.0023 | -1.05175941 |
| Ccdc47 | 0.77828 | -0.06125773 |
| Hspe1 | 0.73298 | -0.10312283 |
| Gm35705 | 0.00216 | -1.10905491 |
| Olfr1014 | 0.00144 | -1.4913104 |
| Gm39055 | 0.00145 | -1.24191372 |
| Rapgef4os3 | 0.00144 | -1.28116048 |
| Taf2 | 0.00144 | -1.30017714 |
| Gm29856 | 0.00144 | -1.29085005 |
| Gm26703 | 0.03494 | -1.11367897 |
| Il34 | 0.07411 | -0.88411385 |
| A330035P11Rik | 0.01119 | -1.29523212 |
| Diras2 | 0.29618 | -0.43161388 |
| Nxf3 | 0.00333 | -1.13565473 |
| LOC102636367 | 0.85656 | 0.08252968 |
| Gm20735 | 0.12349 | 0.39588558 |
| Mtmr9 | 0.00844 | -1.82144545 |
| Gm4681 | 0.00144 | -1.27165604 |
| Sptssb | 0.06271 | -1.38427925 |
| A230020J21Rik | 0.02388 | -0.90349779 |
| Klf1 | 0.00336 | -1.61292113 |
| Map1lc3b | 0.43243 | -0.23923863 |
| Cntnap5b | 0.165 | -0.66979957 |
| Chmp1a | 0.19972 | -0.42490855 |
| Zfp788 | 0.56762 | -0.17951689 |
| Skint10 | 0.00315 | -2.38201597 |
| Fut8 | 0.00144 | 1.59755691 |
| Slc17a1 | 0.02203 | -1.455453 |
| Mrrf | 0.90108 | 0.04070479 |
| Gm13553 | 0.00144 | -1.33632959 |
| Rnf19b | 0.57911 | -0.14941705 |
| Cmtm2b | 0.11329 | -0.42834924 |
| Ppard | 0.0086 | -0.76304613 |
| Clcnkb | 0.29327 | 0.31256748 |
| Gm12414 | 0.00158 | -1.99791367 |
| Mpdz | 0.00144 | 2.15903737 |
| BC049352 | 0.0061 | -0.7192049 |
| Ipcef1 | 0.27757 | -0.42080903 |
| Ubxn2a | 0.98935 | -0.00214078 |
| Arfgap2 | 0.00483 | -0.88753906 |
| A730020E08Rik | 0.00257 | -1.1333102 |
| Pip4k2a | 0.31521 | -0.36640439 |
| Ager | 0.33594 | 0.37305123 |
| Obox6 | 0.00158 | -1.2446827 |
| Krt73 | 0.00145 | -1.2388121 |
| Wdr12 | 0.38398 | 0.23194065 |
| 4930405J17Rik | 0.12505 | -0.60245164 |
| Ralgapa2 | 0.00186 | -1.44274655 |
| Tlcd2 | 0.51356 | 0.14948239 |
| 9530046B11Rik | 0.05194 | 0.56274814 |
| Phldb3 | 0.70825 | 0.21099631 |
| LOC548103 | 0.10782 | 1.09623927 |
| Ipo13 | 0.11405 | -0.55920993 |
| Krtap1-4 | 0.77723 | -0.2340106 |
| 4930586N03Rik | 0.07765 | 0.33377162 |
| LOC102635521 | 0.00144 | -1.43149941 |
| Capn13 | 0.00144 | -1.28116048 |
| Gm34093 | 0.50398 | -0.3900899 |
| Akap13 | 0.45149 | 0.17000814 |
| D630024D03Rik | 0.01232 | -0.64589366 |
| 4930453H23Rik | 0.0341 | -0.77314041 |
| Mrps17 | 0.2032 | -0.30226035 |
| Cap1 | 0.02203 | -0.50959586 |
| Imp3 | 0.01409 | -0.7677009 |
| Cyb5d2 | 0.01697 | -0.59891608 |
| LOC100503212 | 0.31771 | 0.42576139 |
| Gm28178 | 0.34572 | -0.44330161 |
| Gm11780 | 0.04158 | -0.92159312 |
| Rasd1 | 0.38294 | -0.15005313 |
| Wdr36 | 0.39896 | 0.14972591 |
| 3110002H16Rik | 0.28195 | 0.39968439 |
| Slc25a48 | 0.6669 | -0.24303449 |
| Gm14342 | 0.00151 | -1.28615177 |
| Lrp3 | 0.00858 | -0.78007579 |
| Gm33508 | 0.00144 | -1.28898079 |
| Gm13049 | 0.05436 | -0.39278703 |
| Gm44 | 0.01375 | -1.05871899 |
| Hoxa9 | 0.0048 | -1.56250022 |
| Hspe1 | 0.24729 | -0.28607335 |
| Ctsh | 0.85312 | 0.06584272 |
| Mcpt8 | 0.00144 | -1.28116048 |
| LOC105243337 | 0.69631 | 0.12640418 |
| Elfn2 | 0.00231 | -0.87217529 |
| Osmr | 0.06606 | -0.57439197 |
| Adam10 | 0.83984 | -0.05803842 |
| Ggta1 | 0.10488 | 0.63027403 |
| Syndig1l | 0.00149 | -1.22582153 |
| Gm36693 | 0.00145 | -1.2388121 |
| Dnaic2 | 0.021 | -1.14915299 |
| Gm9910 | 0.53224 | 0.16978906 |
| Ssh1 | 0.00192 | -1.00934107 |
| Gm6557 | 0.00144 | -0.9703974 |
| 4933430H16Rik | 0.00144 | -1.27165604 |
| 9230102K24Rik | 0.04854 | -2.4977537 |
| Gpx5 | 0.00144 | -1.31495282 |
| Gm9548 | 0.0848 | -0.68080297 |
| 6030468B19Rik | 0.02988 | -1.52386116 |
| Gm14718 | 0.00145 | -1.2388121 |
| Scly | 0.60744 | -0.35482958 |
| Atp6v0d2 | 0.39593 | -0.44788429 |
| Mcm7 | 0.03939 | -0.46217674 |
| 1700026D11Rik | 0.00144 | -1.28898079 |
| Gm17232 | 0.00145 | -1.22376446 |
| Ttc16 | 0.01247 | -0.53470708 |
| Gm34353 | 0.00144 | -1.29051721 |
| Maob | 0.07598 | -0.38127609 |
| Serpina1e | 0.00452 | -1.15773587 |
| Prkx | 0.97829 | 0.00864829 |
| Adgrg6 | 0.1371 | -0.70951112 |
| Map2k3 | 0.69697 | -0.11609054 |
| Eef1d | 0.0102 | -0.79546453 |
| Adamts17 | 0.11922 | -1.1474527 |
| Art2a-ps | 0.03615 | -0.91785585 |
| Eif3f | 0.12825 | -0.4409929 |
| Zfp451 | 0.0075 | 0.85342678 |
| Heatr3 | 0.75228 | 0.13429959 |
| Bok | 0.00144 | -1.76710004 |
| Erlec1 | 0.00144 | 1.95815602 |
| E430025E21Rik | 0.01168 | 0.63742408 |
| Unc79 | 0.00152 | -1.25878908 |
| Mtap7d3 | 0.02329 | 1.5548563 |
| Spint2 | 0.02764 | -0.71617525 |
| Meis3 | 0.18356 | 0.38946799 |
| Ctrcos | 0.00955 | -0.91109037 |
| Gm4881 | 0.37464 | -0.27149985 |
| 4930515I15 | 0.12671 | -0.66339187 |
| 2310015K22Rik | 0.44985 | 0.40797943 |
| 1700023C21Rik | 0.03355 | -1.25646236 |
| Grik1 | 0.00222 | -1.389975 |
| Slc25a41 | 0.14537 | -0.52851861 |
| Gm15704 | 0.01154 | 1.44100272 |
| Dctn6 | 0.72662 | -0.1013532 |
| E130218I03Rik | 0.04581 | -0.46721843 |
| Smim13 | 0.32612 | 0.20751131 |
| Gm21057 | 0.00144 | -1.30713006 |
| 9130017K11Rik | 0.00144 | -1.29085005 |
| Kcnn1 | 0.07441 | -0.38743093 |
| Vamp3 | 0.06286 | 0.62507159 |
| 4930452N14Rik | 0.00144 | -1.86212526 |
| Pfas | 0.36086 | 0.21317154 |
| Ak5 | 0.00145 | -1.57601082 |
| Tmem39a | 0.00595 | 0.6214588 |
| D630008O14Rik | 0.00207 | -1.72354019 |
| Tmprss9 | 0.00336 | -1.20864476 |
| Zp2 | 0.30071 | -0.53203856 |
| Gm11999 | 0.00145 | -1.27532353 |
| Arhgef16 | 0.0065 | -1.42363028 |
| Gm35959 | 0.41675 | 0.37138938 |
| Gm5427 | 0.98835 | 0.01223337 |
| Wsb1 | 0.11559 | 0.47725073 |
| Gm42293 | 0.0955 | -0.64503682 |
| Sorl1 | 0.0955 | 0.52088198 |
| Olfr168 | 0.00144 | -1.26560808 |
| Lrrc74a | 0.00144 | -1.29085005 |
| Fbxo30 | 0.04225 | 0.37472269 |
| Metrnl | 0.14053 | -0.66038534 |
| 4921511C10Rik | 0.43401 | -0.40835552 |
| Lpl | 0.00398 | 1.3836081 |
| Lars2 | 0.66509 | 0.11287525 |
| A630035G10Rik | 0.92103 | -0.06875759 |
| Ppp4r4 | 0.00144 | -1.23002154 |
| Gm28258 | 0.13545 | -0.78723879 |
| 1110002O04Rik | 0.02702 | -0.67261559 |
| Gm35677 | 0.08131 | -0.42330419 |
| Dlg2 | 0.00257 | -1.31286649 |
| Ulbp1 | 0.83768 | 0.05143282 |
| Dysf | 0.0279 | -0.62729846 |
| Gdap1l1 | 0.0132 | -0.79479773 |
| Noc4l | 0.03945 | -0.5475056 |
| Mkx | 0.00144 | -1.28898079 |
| Atat1 | 0.87273 | 0.13218443 |
| Lmod1 | 0.00144 | -1.29746368 |
| 1700030J22Rik | 0.0114 | -0.58288673 |
| Lad1 | 0.808 | 0.09296839 |
| 4930480M12Rik | 0.00144 | -1.31495282 |
| Gm11832 | 0.06502 | -0.86648736 |
| Mog | 0.00468 | -0.82324028 |
| H2afy | 0.20986 | -0.47762166 |
| A330048O09Rik | 0.01295 | 1.12450147 |
| C1s1 | 0.03056 | -1.49285892 |
| Gm36899 | 0.00304 | -1.26219879 |
| Adss | 0.00144 | 1.49226981 |
| 6720432D03Rik | 0.00144 | -1.31884045 |
| Cep112 | 0.0017 | 1.3378303 |
| 4930504D19Rik | 0.07182 | 0.83460676 |
| Zfp369 | 0.24044 | -0.42600817 |
| Anapc1 | 0.0979 | 0.56329472 |
| Anks6 | 0.01274 | -0.72398287 |
| G6pc3 | 0.99504 | -0.00420511 |
| Gm21284 | 0.00144 | -1.29085005 |
| Bre | 0.84831 | -0.06167459 |
| LOC100043728 | 0.00874 | -1.0083373 |
| Pex2 | 0.31587 | 0.26600016 |
| Kif9 | 0.00145 | -1.21051401 |
| 4922502N22Rik | 0.00144 | -1.27165604 |
| Gm29590 | 0.00144 | -1.56660554 |
| Prkrip1 | 0.42685 | 0.15642641 |
| Dep1 | 0.01205 | 1.6484305 |
| 9130007G19Rik | 0.00144 | -1.31884045 |
| 4930447J18Rik | 0.00144 | -1.20579466 |
| Gm39590 | 0.27603 | -0.54519619 |
| Ccdc169 | 0.11821 | -2.96560314 |
| Gm10129 | 0.21083 | -0.38809966 |
| 4930422C21Rik | 0.00144 | -1.26560808 |
| B230312C02Rik | 0.00525 | -0.86455023 |
| Gm15834 | 0.01313 | -1.70878907 |
| Gm15343 | 0.03119 | -1.03523648 |
| Gm829 | 0.0073 | -1.17129313 |
| A330087D11Rik | 0.00158 | -1.2446827 |
| Gm13660 | 0.00144 | -1.27165604 |
| Gm30509 | 0.00144 | -1.7129058 |
| C1rb | 0.142 | -1.03819921 |
| E330040D14Rik | 0.00144 | -1.27165604 |
| Ceacam1 | 0.09336 | 0.66514661 |
| Elovl3 | 0.04837 | -1.01265322 |
| C230024C17Rik | 0.12371 | -2.95683135 |
| Zdhhc23 | 0.00163 | -1.11663244 |
| Fam13c | 0.79936 | -0.24891792 |
| Clk2 | 0.01184 | 0.57077 |
| 4930578C19Rik | 0.8458 | -0.07275451 |
| Gm30103 | 0.50423 | 0.148205 |
| Olfr301 | 0.00166 | -1.21062785 |
| Crxos | 0.00145 | -1.26219879 |
| 0610033M10Rik | 0.00145 | -1.22376446 |
| LOC105242408 | 0.00144 | -1.28116048 |
| Pmepa1 | 0.00144 | -1.08932736 |
| Gpt2 | 0.00163 | -1.34198119 |
| Rab35 | 0.41604 | -0.14991816 |
| Vmn1r56 | 0.00842 | -1.05664167 |
| AA619741 | 0.0051 | -0.76088833 |
| Gm35204 | 0.00144 | -1.26560808 |
| 7420461P10Rik | 0.00144 | -1.23532597 |
| Olfr1105 | 0.00144 | -1.28116048 |
| Thsd7b | 0.00145 | -1.22376446 |
| Pspc1 | 0.21839 | -0.38023141 |
| Ddo | 0.00144 | 1.31426418 |
| Olfr169 | 0.00144 | -1.28116048 |
| Tox2 | 0.04178 | -0.4882245 |
| Rheb | 0.01458 | 1.16383566 |
| Cdkl4 | 0.00152 | -1.67108641 |
| 4930448I18Rik | 0.56502 | 0.31198415 |
| Fbxo48 | 0.00434 | -1.0948178 |
| 4930578M01Rik | 0.18546 | -2.44653631 |
| Gsto1 | 0.47211 | 0.41007667 |
| Ankrd45 | 0.24834 | 0.97544166 |
| Ceacam5 | 0.00144 | -1.29051721 |
| Gm29676 | 0.07109 | -0.34401753 |
| Gm40918 | 0.00144 | -1.30017714 |
| Ift52 | 0.00624 | -0.82630532 |
| BB283400 | 0.00144 | -1.28898079 |
| B020004J07Rik | 0.00144 | -1.25118919 |
| Tsen34 | 0.36503 | -0.41274537 |
| Bcl2l1 | 0.31501 | -0.21133456 |
| Scn1b | 0.00629 | -0.74707497 |
| Tmcc1 | 0.30537 | 0.33795836 |
| Mfsd8 | 0.12482 | -0.6199015 |
| Bsx | 0.07663 | -2.64599265 |
| 1010001N08Rik | 0.48896 | 0.2688586 |
| Mbd5 | 0.28879 | 0.32110956 |
| A230050P20Rik | 0.00719 | -0.96042152 |
| Pagr1a | 0.05696 | -0.63600057 |
| Tctn3 | 0.00144 | -1.28898079 |
| 1700092C10Rik | 0.04275 | -1.55975967 |
| Ly6h | 0.00144 | -1.33656085 |
| Igkv14-126 | 0.00144 | -1.26934956 |
| Gm41793 | 0.00932 | -0.99616812 |
| Pde8a | 0.59938 | -0.17150712 |
| Spats2 | 0.56201 | -0.1010001 |
| E430002N23Rik | 0.10023 | -0.85867646 |
| Gm15346 | 0.09369 | -0.85473465 |
| Eda2r | 0.5778 | -0.27447042 |
| Gm13125 | 0.00144 | -1.05729147 |
| Olfr936 | 0.00144 | -1.22925777 |
| Spata31 | 0.32169 | 0.37968801 |
| D030040B21Rik | 0.00144 | -1.28898079 |
| Cap2 | 0.02729 | 0.68517239 |
| BC025920 | 0.02642 | 0.51541584 |
| Cdca2 | 0.85672 | 0.08662238 |
| Tmem178b | 0.00144 | -1.28116048 |
| Havcr1 | 0.00272 | -1.09759392 |
| Tsr1 | 0.0037 | 0.82902367 |
| Gm12758 | 0.09637 | 0.48013304 |
| Rab3gap1 | 0.00147 | -1.32513272 |
| Wdr38 | 0.7658 | 0.08391091 |
| Arl14epl | 0.56162 | -0.33668455 |
| 2610316D01Rik | 0.00144 | -1.31884045 |
| Slc2a12 | 0.08574 | -1.18870116 |
| A830092H15Rik | 0.00144 | -1.29085005 |
| Ssbp2 | 0.00144 | 1.29300385 |
| Fpgs | 0.00354 | -0.90345713 |
| Cux2 | 0.40585 | 0.15135465 |
| 4930540I17Rik | 0.04551 | -0.5367473 |
| 4930547E14Rik | 0.91616 | 0.10618969 |
| Gm29536 | 0.03188 | -1.01963123 |
| Map2k7 | 0.06259 | -0.41947352 |
| Mrgpra4 | 0.01637 | 1.76588494 |
| Pih1d1 | 0.00144 | -1.09188161 |
| Gm26995 | 0.13675 | -2.52265203 |
| Gm9860 | 0.00208 | -1.15498993 |
| Clp1 | 0.0786 | 0.34910694 |
| Calm3 | 0.002 | -1.03820401 |
| Lars2 | 0.11091 | -0.33014161 |
| 4931423N10Rik | 0.00447 | -1.07435095 |
| Gm9889 | 0.07188 | -0.60051406 |
| 9630009A06Rik | 0.00144 | -1.30713006 |
| 2900060N18Rik | 0.01102 | -1.5151021 |
| Tinf2 | 0.00559 | -0.68723166 |
| Zfp445 | 0.22374 | 0.27355877 |
| Gm9926 | 0.00216 | -1.10905491 |
| 4930505G20Rik | 0.00296 | -1.24082201 |
| Rnf24 | 0.64358 | 0.11906084 |
| 1700028P15Rik | 0.01266 | -1.31495282 |
| Wipf1 | 0.05333 | 0.58777291 |
| Gm27008 | 0.00144 | -1.30713006 |
| Gpi1 | 0.32433 | -0.38378894 |
| Gm30606 | 0.07413 | -2.28262699 |
| Lcp2 | 0.36344 | -1.26830926 |
| Cd8b1 | 0.12655 | -0.35871793 |
| Slc28a1 | 0.0017 | -1.24950944 |
| A730063M14Rik | 0.60302 | 0.17107398 |
| 5430425K12Rik | 0.00447 | -1.01287609 |
| Ptrh1 | 0.35115 | 0.45072432 |
| Traf3ip2 | 0.09127 | -0.50584908 |
| Gm34047 | 0.44661 | -0.42858483 |
| Slc44a5 | 0.39994 | -0.41076363 |
| Gm15423 | 0.00144 | -1.31884045 |
| Snhg6 | 0.33902 | -0.23239918 |
| Gm16157 | 0.52708 | -0.53282226 |
| Xpnpep3 | 0.92011 | -0.0189665 |
| Omt2b | 0.00144 | -1.28116048 |
| Mtf1 | 0.03176 | -0.4063869 |
| Gm10845 | 0.01279 | -1.10654349 |
| Git1 | 0.00144 | -1.28902986 |
| Aldh16a1 | 0.00145 | -0.98818031 |
| LOC102635527 | 0.00144 | -1.43115851 |
| 9630002D21Rik | 0.00144 | -1.27992547 |
| E330023G01Rik | 0.42379 | -0.3150246 |
| Defb33 | 0.00144 | -1.70741952 |
| 1700042O10Rik | 0.5631 | -0.13400106 |
| BB019430 | 0.00144 | -1.28898079 |
| Gm10091 | 0.08082 | -0.90512202 |
| Gm38997 | 0.00145 | -1.22376446 |
| Pnpla5 | 0.71239 | -0.27232334 |
| Mettl8 | 0.21133 | 0.3395502 |
| AI197445 | 0.00144 | -1.27165604 |
| Nt5dc3 | 0.03958 | -0.79117884 |
| Gm34312 | 0.30993 | -0.63564611 |
| Slc25a5 | 0.72728 | 0.12076571 |
| Gm10790 | 0.22668 | -0.70574589 |
| Ctnnb1 | 0.00352 | -0.87042183 |
| Kcnd3 | 0.08043 | 0.43688847 |
| Olfr248 | 0.0765 | -0.85914525 |
| Gm35194 | 0.08878 | -2.62315888 |
| A630095N17Rik | 0.93216 | -0.04492592 |
| AU015228 | 0.00144 | -1.28116048 |
| Mkl2 | 0.3533 | -0.14451554 |
| Pax6os1 | 0.02533 | -1.83241769 |
| Wdr26 | 0.52799 | 0.15237671 |
| Etnppl | 0.01366 | -0.74582069 |
| Gm12082 | 0.00144 | -1.28819962 |
| Glra1 | 0.97267 | 0.00787665 |
| Ddx41 | 0.71356 | -0.13943002 |
| Cdhr3 | 0.01183 | -1.04972215 |
| Mitd1 | 0.0496 | 0.6784815 |
| Acox2 | 0.07633 | -0.73680659 |
| 6330410L21Rik | 0.00149 | -1.19348234 |
| Tmem28 | 0.01204 | -0.62699015 |
| Tulp4 | 0.28921 | -0.21624443 |
| Zfp622 | 0.00565 | -0.64611598 |
| 9030619P08Rik | 0.0032 | -0.88473195 |
| Crybg3 | 0.80117 | -0.07387388 |
| Gm16006 | 0.07692 | -0.8228402 |
| Arrdc4 | 0.85623 | -0.0395448 |
| 4833418N02Rik | 0.15427 | 0.49430945 |
| 4930524C18Rik | 0.00419 | -0.71980254 |
| Gm13387 | 0.00163 | -1.21015905 |
| Kcnq5 | 0.00719 | -1.40463309 |
| 4932442G11Rik | 0.00144 | -1.26560808 |
| Atoh1 | 0.00144 | -1.26560808 |
| Slc9a4 | 0.00144 | -1.25118919 |
| Tkt | 0.02588 | -0.67697648 |
| Upp2 | 0.57176 | 0.19350785 |
| Gm1720 | 0.01329 | -2.2281979 |
| Rsl1d1 | 0.00191 | 1.19703437 |
| Slc4a11 | 0.36808 | -0.1748824 |
| Gm30146 | 0.27536 | -0.24293888 |
| Gm8298 | 0.00144 | -1.26560808 |
| 1110032F04Rik | 0.13662 | 0.53108349 |
| Itpkc | 0.19656 | 0.22417104 |
| Opn1mw | 0.06577 | -0.83177042 |
| 1700030M09Rik | 0.37837 | 0.40704505 |
| Prss35 | 0.6709 | 0.27700524 |
| Gm16041 | 0.01585 | -1.79535281 |
| A330043C09Rik | 0.04482 | -1.53854548 |
| Gm9776 | 0.42858 | -0.35705734 |
| E030046B03Rik | 0.47486 | 0.14390607 |
| 4930474N09Rik | 0.10761 | -0.70238295 |
| Cep131 | 0.00179 | -1.31883586 |
| 4930401O10Rik | 0.00144 | -1.29746368 |
| 1700001K23Rik | 0.00257 | -1.1333102 |
| Tmem74bos | 0.84281 | 0.13663506 |
| Clca2 | 0.00144 | -1.31884045 |
| Sytl3 | 0.05546 | -0.813494 |
| Phc3 | 0.59694 | -0.12784747 |
| Gm11946 | 0.44362 | -0.35893853 |
| 4921501E09Rik | 0.06077 | -1.45934226 |
| Nploc4 | 0.64258 | -0.11892984 |
| Ifi27 | 0.01674 | -0.62730499 |
| Gm41781 | 0.33205 | -0.62290951 |
| Nvl | 0.60806 | -0.38292295 |
| Tmem59 | 0.55623 | -0.09876193 |
| Pycard | 0.39062 | 0.5303257 |
| Gls2 | 0.00144 | -1.52794963 |
| Cggbp1 | 0.01047 | 0.80364944 |
| Prdm1 | 0.86791 | 0.08265117 |
| Arhgef28 | 0.13541 | 0.2903265 |
| Zfp292 | 0.98771 | -0.0042834 |
| Hist1h3c | 0.00144 | -1.34029927 |
| 4933407G14Rik | 0.00144 | -1.58908829 |
| 6430710C18Rik | 0.01667 | 0.62221435 |
| Phf6 | 0.30646 | -0.99002814 |
| Gm32894 | 0.00916 | -1.07883087 |
| Prickle4 | 0.00144 | -1.31495282 |
| Naaladl1 | 0.00158 | -1.2446827 |
| 4930448I06Rik | 0.00144 | -1.49567869 |
| Ccnl1 | 0.00144 | 2.26516886 |
| 1700061F12Rik | 0.45973 | -0.54971974 |
| Gart | 0.10864 | -3.12965908 |
| Riok2 | 0.00181 | 1.56441604 |
| Ablim3 | 0.26678 | 0.188841 |
| Gm39822 | 0.03487 | -1.04429809 |
| 4930471G24Rik | 0.00144 | -1.29051721 |
| Gm28382 | 0.01132 | -2.32097118 |
| 4930555G21Rik | 0.00145 | -1.22193674 |
| Sim2 | 0.00144 | -1.30713006 |
| Prss22 | 0.08652 | -1.18879248 |
| Kcnn1 | 0.98559 | -0.01129907 |
| 4931431B13Rik | 0.76964 | -0.14981584 |
| Cyp2w1 | 0.00183 | -1.80216447 |
| Phf7 | 0.00595 | -0.67214439 |
| Kank4os | 0.06098 | -0.60872351 |
| 4930556C24Rik | 0.00144 | -1.28116048 |
| 2810428I15Rik | 0.04827 | -0.87396903 |
| Rhbdd1 | 0.85332 | 0.04186609 |
| Tekt3 | 0.00144 | -1.30017714 |
| LOC102635783 | 0.00875 | 0.820326 |
| Gm5485 | 0.05138 | -3.17918066 |
| 5033426O07Rik | 0.00145 | -1.27532353 |
| Psat1 | 0.86566 | 0.07514004 |
| Gnpda1 | 0.3252 | 0.38515785 |
| Gm35439 | 0.01633 | -1.13077247 |
| Lce6a | 0.10124 | -0.7370314 |
| Opn1sw | 0.53292 | -0.20055123 |
| Chmp6 | 0.19416 | -0.39578015 |
| A430105I19Rik | 0.00367 | -0.86086889 |
| Cenpa | 0.95409 | -0.02726926 |
| Cyp2c39 | 0.00149 | -1.19696847 |
| Gm1113 | 0.00463 | -1.48164402 |
| Gm13657 | 0.01536 | -0.71521646 |
| Gm28651 | 0.06395 | 0.72403053 |
| Trim7 | 0.06269 | 0.44506133 |
| Olfr331 | 0.93833 | -0.03101786 |
| Lsmem1 | 0.07439 | 0.36190217 |
| D730002M21Rik | 0.00144 | -1.29864962 |
| Gm34016 | 0.00144 | -1.40054462 |
| Slc23a3 | 0.12633 | -1.21701912 |
| Fras1 | 0.20522 | -0.4465893 |
| BC026863 | 0.79442 | 0.11566404 |
| Gm13398 | 0.00144 | -1.29085005 |
| Gars | 0.03651 | -0.41888544 |
| Rab7b | 0.00948 | -0.70058528 |
| C330021F23Rik | 0.0108 | -0.64909116 |
| Gm29514 | 0.0316 | -1.93538406 |
| Pou2f2 | 0.20993 | -0.63783911 |
| Usp46 | 0.00428 | -0.87387268 |
| Kctd11 | 0.89562 | -0.0704184 |
| Gm4524 | 0.11192 | 0.41757946 |
| Rab11fip4os2 | 0.00144 | -1.25118919 |
| Olfr1251 | 0.00144 | -1.44970716 |
| Gm14809 | 0.00144 | -1.21717671 |
| Tceal6 | 0.14139 | -0.46968653 |
| Cic | 0.00144 | -1.62618978 |
| Gm33517 | 0.00144 | -1.26560808 |
| Tmem200b | 0.0061 | -0.90578258 |
| Gm33680 | 0.44667 | -0.54728252 |
| LOC105247294 | 0.04282 | 1.30488697 |
| Katnb1 | 0.00612 | -0.82114733 |
| Gm7480 | 0.96421 | 0.01915009 |
| 2410152P15Rik | 0.00694 | -2.31345088 |
| 4930441H08Rik | 0.14981 | -0.65984766 |
| Cwc25 | 0.00376 | -0.73545266 |
| Gm12159 | 0.00144 | -1.29746368 |
| Gm15413 | 0.00183 | -2.21775544 |
| Ints12 | 0.03287 | 0.40903739 |
| Gm30082 | 0.00453 | -1.04133679 |
| Idi2 | 0.00191 | -1.4076954 |
| Cercam | 0.00598 | -0.85930151 |
| A530050N04Rik | 0.00531 | -1.26246852 |
| Ebp | 0.31902 | -0.32059563 |
| Ceacam19 | 0.00144 | -1.29085005 |
| LOC102639143 | 0.6772 | -0.11105936 |
| Gm2090 | 0.00144 | -1.29085005 |
| Gm29824 | 0.02611 | -1.0980435 |
| Pck1 | 0.60299 | -0.30404241 |
| Gm15622 | 0.00193 | -1.24713717 |
| Runx2os2 | 0.01421 | -1.46410581 |
| Map3k8 | 0.00352 | 1.48822308 |
| Spata24 | 0.37824 | 0.59567831 |
| Gm773 | 0.00144 | -1.30713006 |
| Gm20319 | 0.00198 | -1.1455615 |
| Mirg | 0.25538 | -0.50882088 |
| Avpr1b | 0.00144 | -1.26560808 |
| Snrpa | 0.01529 | -0.65062917 |
| Gm35657 | 0.98733 | -0.00406418 |
| Mrpl4 | 0.02989 | -0.81757327 |
| Tbc1d20 | 0.32104 | -0.2967807 |
| Cntnap5a | 0.00144 | -1.31884045 |
| Gm6602 | 0.00144 | -1.31495282 |
| A730085E03Rik | 0.00144 | -1.54866398 |
| Gm19335 | 0.31435 | 0.3926661 |
| 4930503B20Rik | 0.00323 | -0.97368468 |
| Htr2a | 0.35777 | 0.69552296 |
| Rnf152 | 0.01787 | 0.89841208 |
| Pms2 | 0.00144 | 1.46463767 |
| Cd79a | 0.01289 | -1.0205606 |
| 4930572K03Rik | 0.00144 | -1.29746368 |
| Gm12916 | 0.14 | -0.64147826 |
| Ggcx | 0.0023 | -1.06413318 |
| Smkr-ps | 0.23555 | -0.68680175 |
| Tsc22d1 | 0.32063 | -0.16611249 |
| Rad51d | 0.00898 | 1.17888028 |
| Trim46 | 0.70816 | -0.08869462 |
| Pate2 | 0.00244 | -0.8225222 |
| Gm41496 | 0.67971 | 0.18713624 |
| Ddx18 | 0.82524 | 0.05858835 |
| Ramp2 | 0.00415 | -0.74208669 |
| 4930442G15Rik | 0.63772 | -0.14770875 |
| 4930432B10Rik | 0.0227 | -0.6476899 |
| Mms22l | 0.00144 | -1.30713006 |
| Tmem74b | 0.03707 | 1.12051555 |
| Dnajb6 | 0.08864 | 0.40205303 |
| Olfr205 | 0.00144 | -1.29085005 |
| Birc6 | 0.51346 | -0.11317268 |
| Gm19277 | 0.98617 | -0.00565622 |
| Tmem233 | 0.81608 | 0.12085669 |
| Vprbp | 0.00662 | 0.60362841 |
| Wdr55 | 0.43831 | 0.28811037 |
| 4933436I20Rik | 0.24876 | -0.27669167 |
| Top2a | 0.02541 | 1.14220262 |
| Mansc4 | 0.12488 | -0.81124085 |
| Hs6st2 | 0.00562 | 1.08129072 |
| Rpl10a | 0.33828 | -0.16382448 |
| Gm16086 | 0.00144 | -1.28116048 |
| Gm26907 | 0.74412 | -0.09415961 |
| LOC101055849 | 0.02616 | 0.53559135 |
| Sfi1 | 0.84317 | -0.04370462 |
| Gpr45 | 0.1028 | -0.48761007 |
| Olfr193 | 0.00249 | -1.24679598 |
| Orc3 | 0.41867 | -0.21023534 |
| Pls3 | 0.00144 | 1.7692883 |
| Sohlh1 | 0.06082 | -0.72264821 |
| Ston2 | 0.00195 | -1.1949875 |
| Nrbf2 | 0.04709 | -0.48559287 |
| D6Ertd527e | 0.19844 | 0.29166784 |
| 1700058M13Rik | 0.31183 | -1.75313186 |
| Trpv2 | 0.0018 | -1.00624082 |
| Mfsd2b | 0.00873 | 1.78715899 |
| Tmem245 | 0.00559 | -0.65183337 |
| 4930544D05Rik | 0.05047 | -1.23534167 |
| Gm34265 | 0.02571 | -0.93883676 |
| 4930404A05Rik | 0.00144 | -1.27165604 |
| Adamts5 | 0.09638 | 0.6707298 |
| 1810007C17Rik | 0.00144 | -1.49377414 |
| Zfp760 | 0.00311 | 2.43268655 |
| L3mbtl2 | 0.09672 | -0.40562495 |
| Ms4a1 | 0.03237 | -1.21321084 |
| Trappc2l | 0.04908 | -0.90497976 |
| Ifnar1 | 0.79746 | 0.07935163 |
| Tcf12 | 0.97809 | -0.01902519 |
| Vax2os | 0.10868 | -1.21399848 |
| Dusp5 | 0.00331 | -0.90811584 |
| Elmo3 | 0.62961 | 0.27088203 |
| Gm14488 | 0.00965 | -0.72502291 |
| Samd11 | 0.83422 | -0.10864661 |
| Grik3 | 0.00144 | -1.27165604 |
| LOC102633677 | 0.03839 | -0.75418042 |
| Erich2 | 0.11474 | -0.96046193 |
| Duoxa1 | 0.00926 | -0.61664764 |
| A430078G23Rik | 0.00503 | -1.31649592 |
| Aqr | 0.63021 | -0.08010668 |
| E330037M01Rik | 0.74751 | 0.13430927 |
| Pglyrp2 | 0.00144 | -1.30017714 |
| Evl | 0.01689 | -0.59686203 |
| Sik3 | 0.06342 | -0.59887296 |
| Kcnb1 | 0.36883 | -0.20192233 |
| Gm29811 | 0.00144 | -1.31495282 |
| Usf1 | 0.0025 | -0.94409883 |
| Plbd2 | 0.32526 | -0.33392244 |
| Klrk1 | 0.26673 | -0.65876883 |
| A430072P03Rik | 0.00144 | -1.29085005 |
| A230103J11Rik | 0.00144 | -1.38185621 |
| Gpr119 | 0.04492 | -0.51201763 |
| Apol10b | 0.14311 | -0.66682163 |
| Fnbp1 | 0.15611 | -0.29801645 |
| Dnal1 | 0.42665 | -0.58691273 |
| C730027H18Rik | 0.11246 | -1.03958412 |
| Sstr5 | 0.74704 | 0.05343324 |
| B430212C06Rik | 0.06633 | 0.85829667 |
| Zbtb4 | 0.01872 | -0.61446719 |
| Vezt | 0.00622 | -1.47491297 |
| Cldn34-ps | 0.00144 | -1.30017714 |
| Olfr666 | 0.9922 | -0.0033171 |
| Tmem138 | 0.65164 | -0.16720433 |
| Usp36 | 0.21739 | -0.26152938 |
| 9330182L06Rik | 0.00144 | -1.24613586 |
| Csnk1a1 | 0.06852 | 0.32251242 |
| Gm5118 | 0.00144 | -1.33632959 |
| Taf10 | 0.00589 | -0.78339287 |
| Nr1h3 | 0.00442 | -0.78834772 |
| Plcg1 | 0.00916 | -0.5799224 |
| 4930461G14Rik | 0.83328 | -0.21884087 |
| Hnrnpd | 0.14322 | -0.47686374 |
| Sssca1 | 0.08252 | -0.78415644 |
| Gm33474 | 0.23085 | -1.9794286 |
| 1700069B07Rik | 0.67069 | -0.07113455 |
| Gm32486 | 0.00144 | -1.27558215 |
| Gm35082 | 0.00144 | -1.20292746 |
| Gm9936 | 0.19226 | -1.64068886 |
| 4930445N18Rik | 0.00536 | -0.8431193 |
| 4930557B15Rik | 0.00145 | -1.22376446 |
| Cit | 0.51146 | -0.20769489 |
| Uhrf1 | 0.01451 | -1.36667627 |
| Gm10410 | 0.03842 | -0.87202135 |
| Got1 | 0.30926 | 0.55498538 |
| 1500012K07Rik | 0.00144 | -1.28116048 |
| Rpl7 | 0.00144 | -1.49031828 |
| Atg7 | 0.002 | -0.81367074 |
| Fbxo38 | 0.00144 | 1.40831154 |
| Clec4a1 | 0.87103 | -0.04421541 |
| Gm10703 | 0.02194 | -1.13969756 |
| Gm12299 | 0.00144 | -1.31884045 |
| Ero1l | 0.02521 | 0.51594139 |
| LOC102638888 | 0.02055 | -0.62057662 |
| Ciita | 0.00356 | -1.45857428 |
| Zcchc4 | 0.13522 | -0.28657332 |
| A630091E08Rik | 0.81036 | 0.05368347 |
| Pus7 | 0.28895 | -0.38541518 |
| Gm6135 | 0.52787 | -0.34776561 |
| C530050E15Rik | 0.05313 | -2.51422672 |
| Wnt7a | 0.00328 | -1.41692424 |
| Gm17673 | 0.16039 | -0.90682582 |
| Pld3 | 0.00598 | -0.86868995 |
| Wdr91 | 0.00144 | -0.95742904 |
| 4930455H04Rik | 0.37982 | -0.93974238 |
| 9230110F15Rik | 0.00144 | -1.27165604 |
| Itln1 | 0.00144 | -1.28116048 |
| Akr1cl | 0.34215 | -0.49704683 |
| Dnal1 | 0.54622 | 0.34757766 |
| Ubxn1 | 0.01669 | -0.52007132 |
| Axl | 0.01999 | -0.5406332 |
| Olfr553 | 0.72688 | -0.09445856 |
| Tcea2 | 0.44068 | -0.21213704 |
| Hsd3b4 | 0.00144 | -1.24811894 |
| Erich1 | 0.0089 | 0.59029487 |
| Gm9981 | 0.93597 | -0.01963123 |
| 1700047G03Rik | 0.01311 | -1.60728483 |
| H2afb1 | 0.11833 | -0.71112914 |
| Gm10369 | 0.00795 | -0.81460209 |
| Gm1564 | 0.02389 | -1.88405336 |
| Phf19 | 0.06565 | -0.45367656 |
| Gm28077 | 0.0059 | -1.77300354 |
| Nme8 | 0.00144 | -1.45349378 |
| 1700010K23Rik | 0.00144 | -1.29051721 |
| Dcst2 | 0.00144 | -1.28898079 |
| Stx7 | 0.00315 | -1.73944166 |
| Adgrf1 | 0.00144 | -1.26560808 |
| AU022754 | 0.24878 | -0.29082016 |
| Fam78a | 0.2593 | -0.46127658 |
| BC049265 | 0.01262 | -1.94527942 |
| Enpp4 | 0.74056 | 0.10777378 |
| Gm41043 | 0.00144 | -1.29085005 |
| Tmem80 | 0.98386 | -0.00453443 |
| B930092H01Rik | 0.00144 | -1.31495282 |
| Tmem147os | 0.00249 | -0.89321656 |
| Lag3 | 0.00733 | -1.14281464 |
| Lipt2 | 0.47213 | 0.23823033 |
| Tubal3 | 0.00144 | -1.28116048 |
| A730085K08Rik | 0.00929 | -1.55332705 |
| Gm39182 | 0.00527 | -1.09019374 |
| Ahsa2 | 0.11441 | 0.49194797 |
| 9530020I12Rik | 0.03202 | -0.46709443 |
| Abracl | 0.00439 | 0.75217744 |
| Gpr165 | 0.32425 | 0.51067726 |
| Gm19897 | 0.01106 | -1.20123908 |
| Ackr4 | 0.02598 | -0.5078507 |
| LOC102639683 | 0.54158 | -0.30923705 |
| Tdo2 | 0.58354 | -0.28189062 |
| Fam135b | 0.08332 | -1.56561413 |
| Egfros | 0.7147 | 0.08690312 |
| LOC102637491 | 0.00144 | -1.12664334 |
| Map3k14 | 0.08561 | -0.56702498 |
| Scnn1a | 0.00158 | -1.0696305 |
| C530034L13Rik | 0.04908 | -0.90497976 |
| Vmn1r213 | 0.00239 | -1.61118827 |
| Oxt | 0.18468 | -0.57157462 |
| Cdca7 | 0.00144 | -1.30713006 |
| Olfr607 | 0.05125 | -1.15766469 |
| Srrm1 | 0.11337 | 0.27068839 |
| Zbtb16 | 0.58442 | 0.14931429 |
| Tuba8 | 0.00298 | -1.17889936 |
| H60b | 0.00261 | -1.70492782 |
| B3glct | 0.5455 | 0.41381273 |
| Gm9962 | 0.0017 | -1.24950944 |
| Mog | 0.67793 | -0.25561104 |
| 4930541O19Rik | 0.00144 | -1.20555712 |
| 6430531B16Rik | 0.04437 | -0.45740707 |
| Sumf2 | 0.37256 | -0.50083345 |
| Gm35961 | 0.00144 | -1.27165604 |
| 4930533P14Rik | 0.00144 | -1.27699683 |
| Hepacam | 0.00144 | -1.30713006 |
| Prkrip1 | 0.07201 | -0.48998716 |
| 1700067G17Rik | 0.04281 | -0.46517145 |
| Ftsj1 | 0.32225 | 0.3577806 |
| Lrpprc | 0.2051 | 0.23025314 |
| Atp6v0a4 | 0.12585 | -0.92206191 |
| Gm19937 | 0.02626 | -0.90748867 |
| Foxk1 | 0.76325 | -0.06957991 |
| Slco2b1 | 0.13572 | -0.38765147 |
| Psg-ps1 | 0.00144 | -1.45718629 |
| Tmem205 | 0.00145 | -1.15684682 |
| Kctd12 | 0.00144 | 2.52693188 |
| Gm20324 | 0.64627 | -0.21724366 |
| 4933406K04Rik | 0.02143 | -2.86259692 |
| Vmn2r60 | 0.00144 | -1.28375735 |
| 2310040G24Rik | 0.69625 | 0.09529722 |
| Gm19696 | 0.07389 | -2.13262127 |
| Gm36388 | 0.00145 | -1.16044081 |
| Gm16311 | 0.00144 | -1.25927895 |
| Gm382 | 0.0765 | -0.85914525 |
| Naip5 | 0.23996 | -0.35677619 |
| Tango6 | 0.0102 | -0.87652522 |
| LOC102640586 | 0.14644 | -0.51264035 |
| 2310007L24Rik | 0.03775 | 0.6866075 |
| Gm17757 | 0.00145 | -1.26219879 |
| 4930517E11Rik | 0.00144 | -1.22925777 |
| BC042761 | 0.00144 | -1.28116048 |
| Gm28523 | 0.01441 | 0.53499649 |
| Gm30363 | 0.90044 | 0.07648218 |
| Gatad1 | 0.84136 | 0.04152274 |
| Maoa | 0.20529 | -0.44094752 |
| Olfr910 | 0.04657 | -1.00288136 |
| 4930479D17Rik | 0.35757 | 0.30617099 |
| Crx | 0.02417 | -0.98467126 |
| Gm20756 | 0.1092 | -0.85431694 |
| 1700072O05Rik | 0.66423 | -0.37231064 |
| Gm12069 | 0.18245 | -0.58899906 |
| Inpp5e | 0.84066 | 0.05775161 |
| 4930519L02Rik | 0.74642 | -0.06263856 |
| Cmtm6 | 0.98779 | 0.00277953 |
| Gm20687 | 0.00144 | -1.20616817 |
| Zfp354c | 0.03733 | 1.50112533 |
| Dusp3 | 0.01976 | -0.48637338 |
| Gm5432 | 0.17503 | -0.77086341 |
| C330004P14Rik | 0.00144 | -1.30713006 |
| 5430403N17Rik | 0.13477 | -0.43601732 |
| A1cf | 0.00144 | -1.25927895 |
| Ankrd13c | 0.00465 | 1.08303493 |
| A230103O09Rik | 0.01231 | -0.91293643 |
| Ica1l | 0.03229 | 0.54793215 |
| 4930563I02Rik | 0.00144 | -1.28116048 |
| Gm26520 | 0.58362 | -0.38812894 |
| 4930557F10Rik | 0.00144 | -1.25118919 |
| 4930433J02Rik | 0.02739 | -1.31645101 |
| Zfp870 | 0.44883 | 0.19486329 |
| Rapsn | 0.38781 | -0.37453673 |
| Eif4e1b | 0.49311 | -0.32920611 |
| BC046251 | 0.00144 | -1.28898079 |
| 4930447M23Rik | 0.00144 | -1.51126328 |
| 2810442N19Rik | 0.09244 | -0.83776848 |
| Gm14204 | 0.00208 | -1.15498993 |
| Pla2g7 | 0.07278 | 0.43349583 |
| Smarcd3 | 0.98436 | 0.01224704 |
| Smarca1 | 0.42705 | 0.59918556 |
| Trappc11 | 0.13443 | -0.69203433 |
| Gm8350 | 0.07732 | -0.47329483 |
| Olfr952 | 0.00144 | -1.29085005 |
| Olfr1283 | 0.00144 | -1.30017714 |
| D930030F02Rik | 0.00462 | -0.75579745 |
| Plaa | 0.57485 | 0.10155481 |
| Kel | 0.00144 | -1.26934956 |
| Chd1l | 0.26954 | -0.54024107 |
| 4930455B14Rik | 0.00144 | -1.27165604 |
| Mcpt-ps1 | 0.75041 | 0.23062712 |
| Eif2b5 | 0.03197 | -0.50806173 |
| Gsdmc | 0.00149 | -1.31665311 |
| Dlc1 | 0.71894 | -0.06188455 |
| Arhgap27os2 | 0.06165 | -0.49449687 |
| Rrm2b | 0.98834 | -0.00367079 |
| Isoc2b | 0.04709 | -0.88788682 |
| Mmp7 | 0.00144 | -1.29085005 |
| Gm9870 | 0.48373 | -0.26901274 |
| Atg9b | 0.00144 | -1.19740533 |
| Pax2 | 0.00144 | -1.80631311 |
| Gm38402 | 0.00145 | -1.25646236 |
| Sh3tc2 | 0.12274 | -0.29245818 |
| Gm5144 | 0.00236 | -1.90072517 |
| 4933429K18Rik | 0.56683 | -0.36460363 |
| Gm20554 | 0.68169 | -0.37916784 |
| Ppp5c | 0.27292 | -0.24254068 |
| Tmem136 | 0.20218 | -0.21469756 |
| Ech1 | 0.0035 | -1.09571548 |
| Nod2 | 0.00144 | -1.30713006 |
| Safb2 | 0.03047 | 0.52981983 |
| Rabif | 0.17985 | 0.27807568 |
| Vmn1r37 | 0.00145 | -1.22376446 |
| Taar6 | 0.07841 | -0.87476151 |
| Nup107 | 0.27071 | -0.183266 |
| Gm19835 | 0.08086 | -1.7893037 |
| Basp1 | 0.00325 | -1.04039162 |
| Gm15408 | 0.00253 | -1.47905303 |
| Olfr884 | 0.5092 | -0.38299114 |
| Serpina16 | 0.00144 | -1.30017714 |
| 6330418K02Rik | 0.11635 | -0.80380875 |
| Lama1 | 0.61612 | -0.25155213 |
| 1700010H22Rik | 0.00144 | -1.21868187 |
| Oca2 | 0.00144 | -1.24466058 |
| Nt5c | 0.60947 | 0.1773583 |
| 3300002A11Rik | 0.03136 | -0.84149151 |
| Lrp1b | 0.00144 | -1.60435619 |
| Gm16907 | 0.28531 | -0.49974961 |
| Sharpin | 0.69855 | -0.16762528 |
| C87198 | 0.00144 | -1.28116048 |
| Lypd6b | 0.58131 | -0.29593296 |
| Mlycd | 0.95265 | 0.04945604 |
| Pdss2 | 0.20148 | 0.53893656 |
| Tmem61 | 0.00144 | -1.64046339 |
| Pamr1 | 0.01332 | -0.94782714 |
| Glis3 | 0.01402 | -1.17749836 |
| Krtap4-13 | 0.00144 | -1.2479551 |
| Gm4131 | 0.02076 | -0.78538127 |
| Yy1 | 0.5436 | -0.10260744 |
| Gm26510 | 0.64331 | 0.37970468 |
| Gm567 | 0.00149 | -1.25408 |
| 4930563H03Rik | 0.07058 | -0.34580093 |
| LOC105246506 | 0.02734 | -1.22279473 |
| Rb1cc1 | 0.00955 | 1.70253056 |
| Gm16701 | 0.07688 | -0.91636288 |
| LOC102641965 | 0.00865 | -0.83700638 |
| Thap7 | 0.00932 | -0.91854829 |
| Cars | 0.19207 | -0.22510564 |
| 4932443I19Rik | 0.00144 | -1.27165604 |
| H1f0 | 0.00275 | -1.3826329 |
| Slc17a2 | 0.00144 | -1.22925777 |
| Gm16578 | 0.00144 | -1.3421574 |
| Xlr | 0.06559 | 0.89246958 |
| 2310016D03Rik | 0.01224 | -0.87699043 |
| Aadacl2 | 0.00144 | -1.29049509 |
| LOC102641639 | 0.08618 | -0.88608781 |
| 4933432I09Rik | 0.07336 | -0.45582427 |
| Gm34567 | 0.25293 | -0.23465437 |
| Gm31359 | 0.00144 | -1.2383131 |
| Gm26685 | 0.19597 | -0.64669808 |
| Catip | 0.06962 | -0.37372287 |
| Lztr1 | 0.57405 | -0.13939879 |
| Smim4 | 0.14546 | 0.4217885 |
| Krt86 | 0.0769 | -2.23451484 |
| Cyp4a14 | 0.00144 | -1.26560808 |
| Ntrk3 | 0.69205 | -0.13911367 |
| Dpp10 | 0.51049 | 0.18506862 |
| Angpt1 | 0.01867 | 1.15238967 |
| Platr6 | 0.17279 | -0.57253342 |
| 1700064F23Rik | 0.00584 | -1.03353025 |
| Gm35219 | 0.52814 | -0.4078627 |
| Gpt | 0.31669 | -0.4077032 |
| Man1a | 0.15968 | 0.59638143 |
| Strn3 | 0.00144 | 1.31664542 |
| Gm14137 | 0.05536 | -0.93361024 |
| Smc2os | 0.87027 | 0.0944356 |
| Trim30d | 0.05413 | -0.48232855 |
| Hnrnpa0 | 0.71589 | 0.05847884 |
| A630019I02Rik | 0.07323 | -1.55859336 |
| Ndufv1 | 0.60055 | -0.25303412 |
| Chrac1 | 0.32674 | -0.25062335 |
| Spdya | 0.47896 | 0.32115625 |
| Pax8 | 0.31812 | -0.38184842 |
| Nr4a2 | 0.45553 | 0.2649919 |
| Olfr1490 | 0.00449 | -1.7260218 |
| Olfr457 | 0.11683 | -0.65984766 |
| Slc39a12 | 0.00144 | -1.33632959 |
| Patz1 | 0.4383 | -0.1444399 |
| Ciao1 | 0.3143 | -0.28675878 |
| Olfr147 | 0.12587 | 0.34425835 |
| Gm12503 | 0.85649 | -0.09298111 |
| Gabrg1 | 0.00144 | -1.26934956 |
| Sag | 0.0191 | -0.671997 |
| Sh2d2a | 0.79195 | 0.05850798 |
| Cep112os2 | 0.32108 | -0.43987264 |
| Fxyd3 | 0.11241 | -1.00470007 |
| 1700071M16Rik | 0.00144 | -1.28116048 |
| Gm38411 | 0.00144 | -1.27585605 |
| Eif2ak1 | 0.03567 | -0.45376268 |
| Gm12052 | 0.00144 | -1.28116048 |
| Gm9484 | 0.01271 | -2.08801719 |
| Atxn1l | 0.13634 | -0.39856792 |
| Pcdh10 | 0.00205 | -1.21461873 |
| Snrpn | 0.30525 | 0.26308436 |
| Pcgf5 | 0.01452 | 2.21301551 |
| Gm4606 | 0.02376 | -1.96735786 |
| 4930555G07Rik | 0.00144 | -1.31495282 |
| Ada | 0.02254 | -1.01331753 |
| Gm7854 | 0.00492 | -1.13895867 |
| Ddx56 | 0.0528 | -0.66450197 |
| Hspa1b | 0.30805 | -0.72106453 |
| Ern1 | 0.65268 | -0.09756113 |
| Rnf38 | 0.12389 | -0.33864718 |
| Gm10426 | 0.91951 | 0.01817071 |
| Rab3ip | 0.30488 | -0.25554449 |
| Gm2848 | 0.02028 | -0.7390141 |
| Pctp | 0.06205 | -0.88151665 |
| Supt4a | 0.00182 | -0.84666945 |
| Gm36814 | 0.00999 | -0.63574796 |
| Gm17518 | 0.00144 | -1.27730451 |
| Xrcc6bp1 | 0.0017 | -1.24950944 |
| Gm38409 | 0.00298 | -0.85302661 |
| Rit1 | 0.3016 | -0.40446888 |
| Fbxl17 | 0.05188 | 0.82615909 |
| Gk5 | 0.18586 | 0.39872766 |
| Raet1a | 0.00158 | -1.2446827 |
| Gm38397 | 0.00145 | -1.2388121 |
| Zfp654 | 0.05224 | -0.40915796 |
| Gm26629 | 0.87678 | -0.22165007 |
| Gm33045 | 0.00144 | -1.31495282 |
| Prrc2c | 0.86529 | 0.0300656 |
| Gm16861 | 0.24685 | -0.54607278 |
| Gm9801 | 0.00783 | 1.42960486 |
| Lhfp | 0.51714 | -0.15940434 |
| Gm30695 | 0.00144 | -1.32766452 |
| Tmbim4 | 0.33806 | -0.16018236 |
| 8430426J06Rik | 0.00314 | 1.80818768 |
| Nrtn | 0.03044 | -1.05518559 |
| Tspear | 0.22348 | -0.54878768 |
| Ndufa6 | 0.00852 | -0.63786108 |
| Capsl | 0.00144 | 1.64233735 |
| D930015M05Rik | 0.03103 | -0.87062541 |
| Pms2 | 0.82822 | 0.13315623 |
| Crxos | 0.00841 | -0.59539848 |
| Rbfox1 | 0.45013 | -0.12573349 |
| 4930526L06Rik | 0.00145 | -1.22376446 |
| Gm16299 | 0.24534 | -0.53889045 |
| Dhrs13os | 0.72457 | -0.22774777 |
| Mlx | 0.07335 | 0.46946954 |
| Gm13261 | 0.00994 | -1.91709835 |
| Hapln1 | 0.4927 | 0.28433253 |
| Awat1 | 0.00144 | -1.27699683 |
| Lipo4 | 0.6108 | 0.27402853 |
| Nup37 | 0.15734 | 0.38182161 |
| Akap13 | 0.60264 | 0.09688553 |
| Appl1 | 0.01329 | 2.04692622 |
| Zpr1 | 0.00977 | -0.6095835 |
| Gm12589 | 0.3002 | -0.57482488 |
| Gm26710 | 0.27165 | -0.63925372 |
| Olfr1511 | 0.00144 | -1.27699683 |
| Zfyve1 | 0.04961 | -0.42365013 |
| Exosc9 | 0.00386 | 0.83731994 |
| Gm16894 | 0.18858 | 0.78677848 |
| Gm33115 | 0.04145 | -0.44488807 |
| Ybx1 | 0.60707 | -0.08623983 |
| Kcnq2 | 0.00144 | -1.22925777 |
| 1700065J11Rik | 0.03643 | -2.64190392 |
| Mapk10 | 0.03592 | 0.89066846 |
| Mtmr7 | 0.36377 | 0.2964523 |
| Bloc1s6os | 0.00781 | -1.08229866 |
| Gm36617 | 0.00144 | -1.29085005 |
| Als2cr12 | 0.11977 | -2.34310889 |
| Gm26748 | 0.00144 | -1.27165604 |
| E030025P04Rik | 0.05148 | -1.69962508 |
| Gm38575 | 0.00144 | -1.38447896 |
| Srrd | 0.00195 | -0.92416109 |
| Gm5535 | 0.76597 | -0.10534306 |
| Sec24a | 0.10506 | -0.30561285 |
| Det1 | 0.00152 | -1.0075312 |
| Gm14317 | 0.00198 | -1.13811458 |
| Dlk1 | 0.40422 | -0.60876728 |
| Sp7 | 0.79678 | -0.04107892 |
| A530065N20Rik | 0.00144 | -1.29049509 |
| Olfr1317 | 0.00803 | -1.49672015 |
| Traf6 | 0.6882 | 0.11602154 |
| Gm34908 | 0.0044 | -0.91067338 |
| Cenpw | 0.03326 | -0.51228202 |
| Sdk1 | 0.09169 | -2.866054 |
| Gm35058 | 0.01467 | -0.5384535 |
| Pvrl3 | 0.55894 | 0.14200994 |
| Eps15l1 | 0.02707 | 0.68641367 |
| Cops5 | 0.08674 | 0.77060359 |
| Atp1b2 | 0.00144 | -1.79387151 |
| E130018O15Rik | 0.00145 | -1.27532353 |
| Vgf | 0.02991 | -0.45970397 |
| Amdhd1 | 0.01056 | -1.0104056 |
| 4930595D18Rik | 0.03264 | -2.08333229 |
| Olfr1024 | 0.00145 | -1.2388121 |
| Pan3 | 0.33851 | 0.17254398 |
| Avl9 | 0.93089 | 0.04380625 |
| 4930429F24Rik | 0.03345 | -1.83158823 |
| Clcn6 | 0.13117 | 0.46972826 |
| Cd180 | 0.40855 | 0.36457397 |
| 4933436H12Rik | 0.01008 | -1.44278479 |
| Mical2 | 0.73434 | -0.09020531 |
| Fam120aos | 0.54723 | -0.17963607 |
| Cdk13 | 0.01001 | 1.25442296 |
| Gm26583 | 0.90336 | 0.03985179 |
| Fsd2 | 0.0125 | -1.43594495 |
| 4930567H12Rik | 0.01758 | -1.31129597 |
| Nms | 0.01898 | -0.53351895 |
| A530095I07Rik | 0.06593 | -1.23991742 |
| C330022B21Rik | 0.00144 | -1.29049509 |
| D830050J10Rik | 0.64349 | 0.32053354 |
| A330033J07Rik | 0.00144 | -1.27165604 |
| 6330416G13Rik | 0.34815 | -0.34412949 |
| Ap3d1 | 0.01173 | -0.58260356 |
| 1700015C15Rik | 0.0321 | -1.78454565 |
| Gm41597 | 0.0104 | -1.5520152 |
| A430028G04Rik | 0.00144 | -1.26560808 |
| A630012P03Rik | 0.10258 | -0.69672773 |
| Fgf4 | 0.00258 | -1.16351105 |
| Gm31107 | 0.2575 | -0.79934369 |
| Calu | 0.05061 | 0.483721 |
| Rad54l2 | 0.51463 | -0.10038117 |
| Eif2ak2 | 0.00195 | 1.33771848 |
| Dgkh | 0.05129 | -0.68738961 |
| Gldnos | 0.00144 | -1.30017714 |
| Tcirg1 | 0.14864 | -0.37797109 |
| Exoc6b | 0.01634 | -0.53521511 |
| Hs6st3 | 0.01709 | -0.99496437 |
| C230030N03Rik | 0.13046 | -0.67103496 |
| Gm572 | 0.0071 | -0.94689659 |
| Syn3 | 0.00966 | -1.02700962 |
| Gm32568 | 0.02808 | -1.93288393 |
| Dazap1 | 0.04105 | -0.42750977 |
| Mcemp1 | 0.0027 | -1.69482984 |
| Wdr25 | 0.10146 | -0.67909769 |
| Ugt2a1 | 0.14034 | 0.63646958 |
| C4b | 0.00144 | -1.8269499 |
| Atp6v1e1 | 0.52814 | 0.23226175 |
| Psma1 | 0.06145 | 0.63307973 |
| Nlk | 0.00144 | 1.05756952 |
| Tro | 0.40394 | 0.22657055 |
| Olfr742 | 0.00144 | -1.28898079 |
| Cdh13 | 0.47332 | -0.18257502 |
| Mgat4b | 0.05176 | -0.72801102 |
| Gm34589 | 0.0048 | 1.44950501 |
| 9530036M11Rik | 0.00144 | -1.53687637 |
| LOC102637646 | 0.05308 | -2.53500008 |
| Smyd2 | 0.34853 | -0.54143675 |
| 4930471C04Rik | 0.80628 | -0.06289827 |
| Katna1 | 0.16162 | -0.26053604 |
| 4930565N06Rik | 0.09076 | -3.00524394 |
| Gm15984 | 0.36456 | 0.40464361 |
| Txlng | 0.00144 | 1.32992362 |
| Gm27242 | 0.25936 | -0.54186816 |
| Lrpap1 | 0.11842 | -0.32852751 |
| Cdh16 | 0.01902 | -0.61812426 |
| Polr2a | 0.00563 | -0.76024113 |
| Kif26b | 0.61766 | 0.21157808 |
| Gm11548 | 0.00749 | -1.05513651 |
| 4930578N18Rik | 0.00144 | -1.57624836 |
| Gm20627 | 0.01855 | 1.25057687 |
| Gm13599 | 0.00149 | -1.22582153 |
| Zfp385b | 0.00144 | -1.35296456 |
| Gm30238 | 0.01684 | 1.1116752 |
| Edem2 | 0.02849 | -0.82451362 |
| 5031434O11Rik | 0.00144 | 1.48015297 |
| 4930572O13Rik | 0.00144 | -1.31096193 |
| Ddr1 | 0.01103 | -0.90460618 |
| Usp21 | 0.00834 | -0.81570219 |
| Atpaf1 | 0.03609 | 0.81787656 |
| C230034O21Rik | 0.47856 | -0.36063059 |
| Smad2 | 0.00983 | 0.75098577 |
| Selt | 0.00144 | 1.64944466 |
| 4930525D18Rik | 0.1944 | -0.72353959 |
| 4633402D09Rik | 0.38848 | -0.41888321 |
| Vps8 | 0.79028 | -0.05293627 |
| Cdon | 0.49143 | -0.37343141 |
| 4930587A21Rik | 0.00144 | -1.26560808 |
| Zfp934 | 0.58825 | 0.21664317 |
| LOC105246071 | 0.0286 | -1.1247985 |
| 4930511O05Rik | 0.00144 | -1.28375735 |
| Sdk1 | 0.03882 | -0.85588951 |
| Slc23a3 | 0.01381 | -1.03043839 |
| Gm14321 | 0.00144 | -1.04706581 |
| Gm16142 | 0.00144 | -1.30713006 |
| Gm35363 | 0.00144 | -1.25118919 |
| LOC434348 | 0.00144 | -1.28116048 |
| D630010B17Rik | 0.51623 | -0.36186729 |
| Atxn2 | 0.0051 | -1.61854705 |
| Tmem168 | 0.00472 | 0.98866822 |
| Agbl2 | 0.02393 | -0.63294383 |
| Gria1 | 0.29603 | -0.87473164 |
| Igf2os | 0.15989 | -0.7046495 |
| Mafg | 0.00596 | -0.65503295 |
| LOC101055751 | 0.00144 | -1.29085005 |
| 4930473D10Rik | 0.18647 | -0.71781502 |
| 9030417H13Rik | 0.01853 | -0.69026086 |
| Rps28 | 0.00144 | -1.08027108 |
| Col22a1 | 0.02327 | -1.07083461 |
| 2010010I01Rik | 0.0015 | -1.29503459 |
| Gm29949 | 0.00144 | -1.26934956 |
| LOC102639814 | 0.00144 | -1.26560808 |
| Gm13848 | 0.00144 | -1.24811894 |
| Tmprss7 | 0.00144 | -1.26428512 |
| Il17rc | 0.00144 | -1.46134994 |
| H2-M9 | 0.0018 | -1.21153109 |
| Aldh3a2 | 0.00436 | 1.49853508 |
| Gm20268 | 0.00186 | -1.39297126 |
| Selp | 0.1205 | -2.28189151 |
| Nlgn2 | 0.00144 | -1.28116048 |
| Mro | 0.07514 | 0.54503992 |
| 2210039B01Rik | 0.024 | 0.5721564 |
| C230052I12Rik | 0.03613 | -0.46498442 |
| Ms4a6d | 0.00451 | 0.85839115 |
| Pm20d2 | 0.44518 | -0.19105267 |
| Flt3 | 0.37104 | -0.40855849 |
| Ndfip2 | 0.00735 | 0.72626605 |
| Surf1 | 0.04321 | -0.50482467 |
| Mfsd10 | 0.30562 | -0.32108913 |
| Hoxa9 | 0.00144 | -1.28116048 |
| Cst3 | 0.02343 | -0.75837356 |
| Gm17597 | 0.03775 | -1.36051914 |
| Gm32949 | 0.00144 | -1.30017714 |
| Pwwp2b | 0.00144 | -1.19276125 |
| Soat2 | 0.2083 | -0.80399151 |
| Gm3428 | 0.00232 | -1.15063496 |
| Gm38436 | 0.15107 | -0.53462119 |
| Trmt13 | 0.00144 | 1.53930409 |
| Taf13 | 0.05344 | 0.54414672 |
| Sh2d6 | 0.00735 | -0.84129895 |
| Mcmdc2 | 0.00144 | -1.18342326 |
| Gm19569 | 0.00487 | -1.50801822 |
| 4933437I04Rik | 0.00144 | -1.27165604 |
| 2610028H24Rik | 0.02076 | 0.65213221 |
| 4930598F16Rik | 0.00144 | -1.31495282 |
| Rcn3 | 0.00202 | -0.89269976 |
| Mup6 | 0.00144 | -1.29878664 |
| Gm5546 | 0.00149 | -1.19348234 |
| D930045L01 | 0.13119 | -0.86696801 |
| Pias1 | 0.16681 | 0.22903582 |
| Vmn2r122 | 0.00474 | -1.41803103 |
| Gm765 | 0.00144 | -1.27699683 |
| AA386476 | 0.12156 | -2.86016762 |
| Gm2629 | 0.00144 | 1.41363167 |
| Maml2 | 0.00664 | 0.94596763 |
| Lcn11 | 0.16008 | -0.65238353 |
| LOC101056093 | 0.42874 | -0.66912113 |
| Gas2l2 | 0.07291 | -1.18318572 |
| Gm39102 | 0.00144 | -1.70561604 |
| Dusp16 | 0.00409 | 0.76673902 |
| Arhgap31 | 0.00758 | 1.21869415 |
| LOC102641980 | 0.32153 | -0.31534785 |
| Defb3 | 0.0648 | -0.75582677 |
| Zfp791 | 0.35223 | 0.29416072 |
| F5 | 0.68705 | 0.14556635 |
| Tssk3 | 0.05588 | -0.48008524 |
| Ndufb5 | 0.99551 | 0.00280674 |
| 1700031M16Rik | 0.13422 | -0.46561122 |
| Vmn2r69 | 0.0018 | -1.21153109 |
| Lrrc55 | 0.06072 | -0.73341642 |
| Il23r | 0.00144 | -1.28116048 |
| Gm16233 | 0.00144 | -1.28116048 |
| LOC105243223 | 0.00144 | -1.96709705 |
| Gm19500 | 0.28903 | -0.57652869 |
| Sdhb | 0.00144 | -1.04489186 |
| Hcst | 0.00247 | -1.31453066 |
| Gm20736 | 0.00144 | -1.26042915 |
| Mdc1 | 0.14225 | -0.44731254 |
| Eif5a | 0.0078 | -0.9338012 |
| 1700054A03Rik | 0.09201 | -0.79344826 |
| 4930429D17Rik | 0.82275 | 0.09592122 |
| Ptpn2 | 0.02014 | 1.06950565 |
| Ints9 | 0.23858 | -0.45919148 |
| Ldhb | 0.33803 | 0.58841956 |
| Rbfox2 | 0.00144 | -1.27739384 |
| LOC102637966 | 0.00585 | -1.07062058 |
| Platr5 | 0.01252 | -1.12803231 |
| B4galnt4 | 0.00144 | -2.25760281 |
| Prr15l | 0.00144 | -1.22925777 |
| Xkr5 | 0.00144 | -1.46941966 |
| Gm36550 | 0.0047 | -1.17385111 |
| Tac2 | 0.00733 | -1.10984709 |
| C530038A18Rik | 0.06807 | -2.68599724 |
| Setd2 | 0.00144 | 1.17718729 |
| Gm10427 | 0.05781 | -1.00299626 |
| Smarcd2 | 0.04124 | -0.59078954 |
| Olfr975 | 0.19834 | -0.78926705 |
| B130055M24Rik | 0.00722 | 1.36644458 |
| Olfr699 | 0.3546 | -1.72749417 |
| Dym | 0.36028 | 0.52435124 |
| Ccbl1 | 0.24982 | -0.37348733 |
| Gstm5 | 0.00839 | -0.65442907 |
| Fbxo31 | 0.91702 | -0.05754307 |
| LOC432596 | 0.52754 | -0.14537231 |
| Zfp790 | 0.0023 | 1.26752064 |
| Tmem171 | 0.97429 | 0.02855858 |
| 1700016H13Rik | 0.00354 | -0.83998511 |
| Slc36a3os | 0.00208 | -1.15498993 |
| Gm9991 | 0.00144 | -1.28116048 |
| 4930567K20Rik | 0.00144 | -1.28898079 |
| Tmem88b | 0.00825 | -0.7355168 |
| Ssrp1 | 0.03018 | 0.41918308 |
| Klra10 | 0.00144 | -1.40960623 |
| Hist1h2ai | 0.00144 | -1.27558215 |
| Tubgcp4 | 0.00568 | 0.8233352 |
| Gabpb2 | 0.77489 | -0.16618857 |
| Sp2 | 0.23546 | 0.19736203 |
| Gm15319 | 0.12909 | 0.71113547 |
| Oraov1 | 0.29918 | -0.2847691 |
| Cyb561a3 | 0.16215 | 0.63906294 |
| Zfp593 | 0.884 | -0.02999557 |
| Lonrf3 | 0.91225 | -0.04369242 |
| P2rx1 | 0.90797 | -0.05105608 |
| LOC102634128 | 0.00144 | -1.28116048 |
| Fam19a5 | 0.00144 | -1.28898079 |
| Gm24175 | 0.05069 | -2.20333043 |
| Nhlh1 | 0.98724 | 0.00470445 |
| 4933417D19Rik | 0.80495 | -0.12182519 |
| Mthfd2l | 0.00253 | 1.14852967 |
| Ptprq | 0.00144 | -1.31884045 |
| BC023719 | 0.102 | -0.70232622 |
| Galnt1 | 0.01795 | 0.73786721 |
| Gm17783 | 0.06859 | -2.41902041 |
| Haus6 | 0.74984 | 0.17657148 |
| Fhad1os1 | 0.00144 | -1.36950815 |
| Rnf207 | 0.00144 | 1.51029188 |
| Hoxa10 | 0.00144 | -1.27992547 |
| Tectb | 0.00236 | -1.13100373 |
| Tmem72 | 0.00144 | -1.28898079 |
| Adat1 | 0.59051 | -0.46108849 |
| Igsf8 | 0.02494 | -1.92312902 |
| 1700007P06Rik | 0.26941 | -0.3167928 |
| Nfxl1 | 0.00429 | -1.37617779 |
| Champ1 | 0.42116 | 0.13362411 |
| G630018N14Rik | 0.06336 | -1.44000952 |
| 1700071A11Rik | 0.46725 | -0.28724534 |
| AU040096 | 0.0015 | -1.15488942 |
| Tead4 | 0.36819 | -0.60247348 |
| Gm38728 | 0.89895 | -0.0224607 |
| 1700038P13Rik | 0.00144 | -2.45697723 |
| Gm32364 | 0.77629 | 0.13081815 |
| Sipa1l3 | 0.0042 | -2.07701217 |
| Sox12 | 0.77195 | -0.15026575 |
| Gm11240 | 0.09622 | -0.31674726 |
| A930027H12Rik | 0.00144 | -1.27454685 |
| Crebzf | 0.00328 | 1.53550123 |
| Hdac4 | 0.14298 | -0.37400031 |
| Gm12339 | 0.09415 | -2.15579304 |
| A530021J07Rik | 0.00176 | -1.24266807 |
| C130046B21Rik | 0.00263 | -1.25281511 |
| Sfta2 | 0.32274 | -0.2570022 |
| Tceb1 | 0.96887 | 0.00754968 |
| Scel | 0.00144 | -1.59894142 |
| Coro1c | 0.02557 | -0.45263892 |
| Gm6687 | 0.42684 | -0.57318285 |
| Gm5122 | 0.21136 | -0.58979569 |
| Gm5126 | 0.03268 | -0.65058161 |
| LOC105242553 | 0.55841 | -0.46602648 |
| Bag6 | 0.0235 | -0.69956401 |
| Pabpc1 | 0.01209 | 0.57712322 |
| 1810012K16Rik | 0.77527 | 0.07345249 |
| Gm10579 | 0.00144 | -1.28898079 |
| Klf3 | 0.00144 | 1.26604421 |
| Ctps2 | 0.22964 | 0.72076196 |
| Gm12374 | 0.0108 | -1.08231701 |
| 1700007F19Rik | 0.00145 | -1.26219879 |
| Gnas | 0.52979 | -0.18389872 |
| Supt6 | 0.28424 | 0.19167025 |
| C630028M04Rik | 0.00145 | -1.2388121 |
| Cirh1a | 0.14225 | -0.24229989 |
| Tbrg3 | 0.02188 | 1.32044146 |
| Gm36236 | 0.34625 | -0.48280789 |
| Snord7 | 0.87099 | 0.04024061 |
| Sgca | 0.21154 | -0.3622576 |
| Tmem255b | 0.00144 | -1.36544467 |
| Mapt | 0.01283 | -0.5546391 |
| Zc3h8 | 0.00144 | 1.67959485 |
| Tmod1 | 0.06098 | 0.86131732 |
| 1810010D01Rik | 0.2549 | 0.42449599 |
| Foxl2os | 0.04991 | -0.93578497 |
| Atp5j | 0.64096 | -0.11175507 |
| Gm5922 | 0.00144 | -1.29085005 |
| Gm15972 | 0.01863 | -0.57994998 |
| Spag8 | 0.00268 | -1.17452065 |
| Cltb | 0.00375 | -0.8790215 |
| Snrnp48 | 0.05203 | 0.69185656 |
| Serpinb3b | 0.00144 | -1.28898079 |
| Gm19966 | 0.00144 | -0.97676961 |
| Gm12359 | 0.8476 | 0.05486871 |
| Lyrm7os | 0.12886 | -3.12013006 |
| Chmp2a | 0.94554 | -0.0174369 |
| Olfr1513 | 0.06542 | 0.77248604 |
| Gm29776 | 0.00144 | -0.96940062 |
| Siglec1 | 0.00399 | -1.2523575 |
| Gmppb | 0.09248 | -0.47985284 |
| Nfia | 0.21051 | 0.62527122 |
| Hyou1 | 0.00169 | -1.08462507 |
| Gm35665 | 0.06462 | -0.53660118 |
| Prrt4 | 0.01034 | -0.78755712 |
| Gpn1 | 0.32313 | 0.24412094 |
| Anp32b | 0.02942 | 0.52314855 |
| Gm30123 | 0.00145 | -1.2388121 |
| Prl3d1 | 0.00144 | -1.28898079 |
| Il3ra | 0.00268 | -1.18805763 |
| Bend5 | 0.78256 | -0.04439455 |
| 4930579D09Rik | 0.08132 | -0.65812424 |
| Stat5a | 0.00144 | -1.22077835 |
| 4930528J11Rik | 0.1127 | 0.74352887 |
| Gabrp | 0.04965 | -1.12361257 |
| Gm26535 | 0.00145 | -1.2388121 |
| Stag3 | 0.00144 | -1.27454685 |
| Agpat9 | 0.00286 | -1.04693312 |
| Mprip | 0.00144 | -1.27378934 |
| Hmgb4os | 0.0373 | -0.85954928 |
| Rc3h2 | 0.00265 | 0.92323365 |
| Kcnip1 | 0.43089 | -0.19555797 |
| Tnfrsf21 | 0.05405 | -0.47814048 |
| Olfr1432 | 0.00144 | -1.22925777 |
| Atm | 0.00345 | 1.35456633 |
| Gm34746 | 0.0136 | -1.47969685 |
| Olfr145 | 0.05104 | -1.25626325 |
| Gm38520 | 0.00739 | -1.26188251 |
| Vwa1 | 0.05278 | -0.59038167 |
| 1700008K24Rik | 0.01134 | -1.02600084 |
| Ccdc178 | 0.09051 | -3.53954178 |
| Iapp | 0.00144 | -1.27730451 |
| Rbm25 | 0.00446 | 1.17146704 |
| Gm13031 | 0.00486 | -1.08982022 |
| Phex | 0.00144 | -1.27165604 |
| Pon2 | 0.16955 | 0.29138466 |
| Cd96 | 0.03551 | -0.8201655 |
| Gm7325 | 0.00144 | -1.29085005 |
| Gm32854 | 0.14692 | -0.42736251 |
| 4930533B01Rik | 0.01647 | -0.62725504 |
| Hoxaas3 | 0.1211 | -0.55996324 |
| Pla2g7 | 0.07355 | 0.49922479 |
| Bnc2 | 0.00144 | -1.31495282 |
| Bach2 | 0.00933 | 0.85084953 |
| Gm38585 | 0.03006 | 1.03503904 |
| Akr1d1 | 0.00144 | -1.56804473 |
| Hopxos | 0.40316 | -0.89172424 |
| 5033404E19Rik | 0.00296 | -1.42334488 |
| 1810049I09Rik | 0.01051 | -1.1474527 |
| 4930421J07Rik | 0.43205 | -0.49426932 |
| D930030F02Rik | 0.03433 | -1.24473038 |
| Vmn1r-ps103 | 0.09495 | -0.82135365 |
| F730035P03Rik | 0.00144 | -1.29746368 |
| 6720483E21Rik | 0.00208 | -1.68601549 |
| Adamts6 | 0.73588 | 0.26177621 |
| Bpifa3 | 0.60443 | -0.3505101 |
| Gga3 | 0.52754 | 0.09762027 |
| Gm40568 | 0.15613 | 0.89022176 |
| Gm9915 | 0.00144 | -1.28898079 |
| Gm608 | 0.34383 | -0.24390653 |
| Rspo3 | 0.00149 | -1.25408 |
| Cdh18 | 0.07069 | -1.37159448 |
| S100a16 | 0.00149 | -0.96813669 |
| Gm16063 | 0.27848 | -0.74376403 |
| Olfr885 | 0.45973 | -0.58955815 |
| Gm13498 | 0.00144 | -1.32766452 |
| Ndst1 | 0.00848 | -1.91260456 |
| Rab3gap1 | 0.03343 | 0.49442934 |
| 4930528P14Rik | 0.00144 | -1.27992547 |
| Zfp558 | 0.01473 | 0.78992583 |
| Pogz | 0.19441 | 0.56043797 |
| Mta3 | 0.07677 | -0.34833081 |
| Cyp4x1 | 0.00144 | -1.29746368 |
| A630095N17Rik | 0.00144 | -1.28898079 |
| Krtap1-3 | 0.00144 | -1.27730451 |
| Gm34095 | 0.00144 | -1.27165604 |
| Slc6a7 | 0.00144 | -1.20381157 |
| Trim67 | 0.00247 | -1.41779016 |
| Wwc2 | 0.3419 | 0.18578343 |
| Atp5l | 0.00333 | -0.81584114 |
| Tvp23bos | 0.4746 | 0.30638457 |
| Dnah17 | 0.01892 | -1.37730124 |
| Il1b | 0.87578 | -0.0835456 |
| 1700060C16Rik | 0.01051 | -1.1474527 |
| Gm32478 | 0.00144 | -1.31886942 |
| Etaa1os | 0.14167 | 0.4336551 |
| Grtp1 | 0.72275 | 0.2186063 |
| Ccnb1 | 0.00145 | -1.2388121 |
| Gm4971 | 0.0881 | -0.90540141 |
| Trim50 | 0.00144 | -1.23532597 |
| Grm7 | 0.00144 | -1.28116048 |
| Adgrg6 | 0.0121 | 0.94763415 |
| Samd4b | 0.03073 | -0.45842058 |
| Gm30529 | 0.04389 | -1.85603528 |
| Gm7978 | 0.38411 | -0.41285221 |
| Olfr888 | 0.01143 | -1.58830914 |
| A730043L09Rik | 0.11765 | -0.2697186 |
| Gm16068 | 0.02783 | -1.06344206 |
| Parp8 | 0.009 | 1.6638344 |
| Pgm3 | 0.25695 | 0.2289644 |
| Gpr55 | 0.00144 | -1.31495282 |
| Rnpep | 0.19166 | -0.40289236 |
| Ypel2 | 0.63237 | 0.10708866 |
| P3h2 | 0.83575 | 0.03482438 |
| A830035O19Rik | 0.0763 | -0.61851708 |
| Gm16152 | 0.68116 | -0.4833405 |
| Gm30789 | 0.00442 | -1.1333102 |
| Ankrd13d | 0.01778 | -0.75442592 |
| Pno1 | 0.02878 | -0.58634405 |
| Inpp1 | 0.00144 | -1.31014844 |
| Plcxd3 | 0.18781 | 0.67475355 |
| LOC105243711 | 0.03338 | -0.79432264 |
| 2310061I04Rik | 0.01365 | -0.84614715 |
| Olfr1168 | 0.0401 | -0.95716175 |
| Tlr12 | 0.00145 | -1.14210969 |
| 1700026F02Rik | 0.00144 | -1.29051721 |
| Vmn1r180 | 0.00144 | -1.30017714 |
| Dnmt3bos | 0.04014 | -0.95775877 |
| Gm38793 | 0.04741 | -0.75767283 |
| Gm29260 | 0.00232 | -1.15063496 |
| H3f3a | 0.15978 | -0.3069821 |
| Paupar | 0.73347 | 0.2448649 |
| 5930412G12Rik | 0.00144 | -1.27165604 |
| Svs3b | 0.03573 | -1.8999063 |
| 2310046K23Rik | 0.00234 | -1.43684968 |
| Frs2 | 0.01746 | 0.68504544 |
| LOC102632821 | 0.11053 | 0.45501564 |
| Tekt5 | 0.00144 | -1.40024753 |
| Gm6537 | 0.00157 | -0.93298551 |
| 2900089D17Rik | 0.42152 | 0.20562485 |
| Fam188b | 0.14888 | -2.00787852 |
| Hist2h2ab | 0.00621 | -1.73431335 |
| Gm33791 | 0.00144 | -1.33632959 |
| Gm7904 | 0.30485 | 0.53978721 |
| E130116L18Rik | 0.78225 | -0.23156607 |
| 4930487D11Rik | 0.00145 | -1.24082201 |
| Ms4a18 | 0.05119 | -0.58706097 |
| B430319G15Rik | 0.08887 | 0.95896474 |
| Gm19639 | 0.06327 | -1.02180233 |
| 5430435K18Rik | 0.01107 | -1.00198097 |
| Gm19434 | 0.00732 | -1.14137549 |
| Zfp36l2 | 0.23989 | -0.26795071 |
| Lvrn | 0.03642 | -2.36435806 |
| Abcg8 | 0.00336 | -0.90144852 |
| Slc35a1 | 0.01705 | 0.65059231 |
| Pzp | 0.48537 | 0.22702066 |
| Gm41676 | 0.00145 | -1.2388121 |
| Sco2 | 0.03892 | -0.78220665 |
| Olfr1392 | 0.00259 | -1.31244712 |
| Olfr1333 | 0.28506 | 0.67787944 |
| Gm26651 | 0.05653 | -0.78533208 |
| Gm14133 | 0.00144 | -1.29049509 |
| Chtop | 0.1639 | 0.23468808 |
| 3110039I08Rik | 0.0047 | 2.75526976 |
| Tmem126a | 0.0606 | -0.36132134 |
| 1700085C21Rik | 0.23503 | -0.57771417 |
| Gm15813 | 0.00144 | -1.31884045 |
| Slc47a2 | 0.20224 | -1.35725343 |
| Tmem28 | 0.51212 | -0.68010889 |
| Dbpht2 | 0.00144 | -1.28116048 |
| Gm33677 | 0.00409 | -1.26528643 |
| Eno4 | 0.00572 | -1.28900438 |
| Uckl1os | 0.95644 | -0.01209957 |
| Vps26b | 0.46495 | -0.16337891 |
| Sspo | 0.24576 | 0.30628409 |
| Smim6 | 0.38256 | 0.63158397 |
| Ubtfl1 | 0.00357 | -1.52608928 |
| C820005J03Rik | 0.01193 | -2.13805323 |
| Gm3325 | 0.25545 | -0.6938875 |
| 1700010J16Rik | 0.00144 | -1.29051721 |
| 1700042O13Rik | 0.00145 | -1.17894292 |
| Defb36 | 0.00144 | -1.23546088 |
| Cdh3 | 0.00145 | -1.60870824 |
| LOC100861913 | 0.00144 | -1.24613586 |
| Itpr3os | 0.32765 | -0.670333 |
| Cdk6 | 0.53628 | 0.30779381 |
| C1qtnf2 | 0.00148 | -1.11256688 |
| Elmod2 | 0.55543 | 0.5137112 |
| Prdx1 | 0.00737 | -0.60177023 |
| 2310039L15Rik | 0.00144 | -1.31495282 |
| AW060742 | 0.91372 | -0.06337941 |
| Gpr6 | 0.07093 | -1.00048066 |
| Gnpda2 | 0.02624 | 0.71839486 |
| Nkx2-1 | 0.00144 | -1.27992547 |
| 1300017J02Rik | 0.0464 | -1.72353959 |
| Gm19582 | 0.00144 | -1.51281896 |
| Rexo1 | 0.00268 | -0.83352242 |
| Atox1 | 0.00144 | -1.08693607 |
| 4921521D15Rik | 0.08659 | -0.99076289 |
| Hspa14 | 0.74352 | 0.15734486 |
| LOC105242800 | 0.00144 | -1.19002334 |
| Nespas | 0.00144 | -1.30713006 |
| Gm13415 | 0.30825 | -0.524763 |
| 4930459I23Rik | 0.00605 | -1.08150631 |
| Gm19689 | 0.00176 | 2.34489434 |
| Rlf | 0.0044 | 1.4576791 |
| Gm33255 | 0.77779 | -0.11134593 |
| Tmem62 | 0.19344 | 0.23087708 |
| Ripply1 | 0.10055 | -1.18932981 |
| LOC102637283 | 0.19123 | -2.31314342 |
| Slc15a2 | 0.9343 | 0.09355772 |
| Gm13546 | 0.04919 | -1.23118444 |
| 4930412B13Rik | 0.00144 | -1.20555712 |
| Gm33301 | 0.00598 | -0.85835468 |
| Synpo | 0.00153 | -1.0651319 |
| Ube4bos1 | 0.4587 | -0.25503332 |
| Il31ra | 0.00144 | -1.27585605 |
| Prss23os | 0.08815 | -1.02400212 |
| 1110036E04Rik | 0.17607 | 0.73559677 |
| Stc1 | 0.01072 | -1.05977457 |
| Rsrp1 | 0.75081 | -0.05839042 |
| Gm36551 | 0.04601 | -0.9836968 |
| Dpp10 | 0.00144 | -1.49016641 |
| Gm29507 | 0.01514 | -0.95990192 |
| Sh2b2 | 0.13523 | -0.35138963 |
| Pacsin2 | 0.13411 | 0.36639283 |
| Ccdc33 | 0.02081 | -0.64668889 |
| Gm15559 | 0.00292 | -1.09742983 |
| Cerkl | 0.01909 | -1.09965565 |
| Tspan2os | 0.01094 | -1.11366036 |
| Alg2 | 0.01073 | -0.59957052 |
| Pld5 | 0.00145 | -1.26219879 |
| 9130011E15Rik | 0.10893 | 0.89432757 |
| Unc45a | 0.01787 | -0.85450188 |
| Gm14164 | 0.00144 | -1.29085005 |
| 4930507L24Rik | 0.00176 | -1.24266807 |
| Snrnp27 | 0.77527 | 0.08922974 |
| Wdr62 | 0.00925 | -0.97271178 |
| Casq2 | 0.08154 | 0.68718704 |
| Thsd7a | 0.08344 | -1.05862749 |
| 4930528D03Rik | 0.00811 | -1.60498937 |
| 5730420D15Rik | 0.08331 | -0.91134937 |
| Gm14637 | 0.81727 | -0.24082201 |
| Gm5103 | 0.87808 | -0.08754991 |
| Ip6k2 | 0.66238 | 0.09679516 |
| Leprot | 0.89971 | -0.02339195 |
| Platr30 | 0.01033 | -0.91204365 |
| 5430427G11Rik | 0.00145 | -1.24191372 |
| Sdr9c7 | 0.31601 | -0.68008033 |
| Ccdc109b | 0.03927 | -0.40113329 |
| Erdr1 | 0.61666 | -0.41532722 |
| Prcp | 0.06896 | 0.59716865 |
| Gm2233 | 0.00144 | -1.27992547 |
| Mme | 0.04901 | 1.39511548 |
| Galnt14 | 0.97911 | -0.01039267 |
| 4930509K18Rik | 0.00144 | -1.34306733 |
| Gm5486 | 0.12344 | -0.47315171 |
| Mfng | 0.00144 | -1.19695286 |
| Mylk3 | 0.0986 | -0.33071124 |
| Pdia3 | 0.0076 | 0.76469538 |
| 4930458A03Rik | 0.45499 | -0.73696559 |
| Gm14391 | 0.11194 | 0.56381786 |
| Cops3 | 0.04054 | 0.96105249 |
| Lyar | 0.27716 | 0.30896581 |
| 4930444F02Rik | 0.05982 | -0.83084896 |
| Gm38947 | 0.84016 | 0.06572196 |
| Ggn | 0.28359 | -1.08033603 |
| Aoc1 | 0.00144 | -1.27992547 |
| Slc5a9 | 0.00257 | -1.1333102 |
| Gm9878 | 0.0043 | 1.74131155 |
| Psmd6 | 0.02094 | 1.10933544 |
| Gm3907 | 0.00144 | -1.44608204 |
| Adad1 | 0.04801 | -1.17193878 |
| Atg16l2 | 0.48135 | -0.39364991 |
| Akr1d1 | 0.02627 | -1.04360839 |
| A730082K24Rik | 0.00144 | -1.28898079 |
| 4930407G08Rik | 0.00144 | -1.27730451 |
| Srm | 0.0534 | -0.55333105 |
| Sema5b | 0.04569 | 0.56290348 |
| Itgb1 | 0.59971 | -0.14747826 |
| Sdcbp | 0.23293 | 0.28924005 |
| Lep | 0.99897 | -0.00023991 |
| Nlrc5 | 0.29592 | -0.39753788 |
| LOC102642104 | 0.77844 | -0.09025827 |
| Nipal3 | 0.83262 | -0.07102828 |
| Gm725 | 0.75459 | 0.12973346 |
| Gm31633 | 0.00145 | -1.22376446 |
| Bpifa1 | 0.00289 | -0.90289558 |
| Pla2g2e | 0.06059 | -0.8047872 |
| Krtap16-1 | 0.00144 | -1.50131777 |
| 4930554G24Rik | 0.7694 | 0.20326835 |
| Gm15821 | 0.10141 | -2.69626275 |
| Snx4 | 0.33482 | -0.15834455 |
| Gm2511 | 0.18126 | -0.80005109 |
| A730004F24Rik | 0.40892 | -0.38875907 |
| Gm10253 | 0.00144 | -1.30017714 |
| Spink11 | 0.00144 | -1.29085005 |
| Abhd16a | 0.05587 | -0.51633835 |
| Isl1 | 0.00284 | -1.15314387 |
| Wac | 0.00477 | -0.66814875 |
| Abca1 | 0.04616 | -0.51467444 |
| Adar | 0.00925 | -0.69863618 |
| Npy4r | 0.21776 | -0.6699947 |
| Rnf146 | 0.00335 | 0.82022633 |
| Dpy19l3 | 0.00225 | -1.18527205 |
| Gm11186 | 0.00314 | -1.14703327 |
| Ubl3 | 0.00594 | 0.95187465 |
| Paqr3 | 0.34268 | -0.51419585 |
| Gm17821 | 0.00144 | 2.63184688 |
| Gm28876 | 0.00144 | -1.4157514 |
| AI413582 | 0.00413 | -0.87835971 |
| Eif1a | 0.01997 | 0.76924022 |
| Glt6d1 | 0.00144 | -1.5654412 |
| Ddx56 | 0.2406 | -0.59835997 |
| E230014E18Rik | 0.03408 | -1.12689901 |
| Glrx2 | 0.40936 | -0.12666404 |
| Arhgap26 | 0.13433 | -0.38283489 |
| Gm19461 | 0.05873 | 0.78726546 |
| Gm10371 | 0.00144 | -1.31627578 |
| E130120K24Rik | 0.00144 | -1.26560808 |
| Gm10463 | 0.80525 | -0.16087395 |
| Gm5111 | 0.07689 | -0.90547877 |
| Gm32950 | 0.83488 | -0.11811652 |
| Rln3 | 0.52166 | -0.29270985 |
| Olfr1201 | 0.00144 | -1.26934956 |
| Fam175a | 0.00268 | 0.91526913 |
| Gm20745 | 0.00144 | -1.28116048 |
| Gm39348 | 0.00144 | -1.26560808 |
| Kcnh4 | 0.036 | -0.96355852 |
| 4930519G04Rik | 0.17647 | -0.95106795 |
| Gm16208 | 0.38688 | -0.37060516 |
| Lsamp | 0.86587 | 0.09181521 |
| Casp12 | 0.02478 | 0.91074911 |
| Sprr2i | 0.11394 | -0.68887906 |
| C430042M11Rik | 0.00144 | -1.25118919 |
| Gm33609 | 0.03715 | 1.1677846 |
| Rgsl1 | 0.0381 | -0.83414817 |
| N4bp2l2 | 0.0023 | 1.27033894 |
| Figf | 0.46248 | -0.19709889 |
| 4930487H11Rik | 0.00144 | -1.25118919 |
| Gm9564 | 0.00145 | -1.22518835 |
| Gm27162 | 0.00144 | -1.25927895 |
| Isg20 | 0.01413 | -0.63593903 |
| Ash1l | 0.0401 | 0.61004789 |
| Zfp36l1 | 0.32585 | 0.63084135 |
| Fam47c | 0.00144 | -1.24811894 |
| Tmem192 | 0.47429 | -0.30954731 |
| Ubac1 | 0.60814 | 0.23221338 |
| Ppp1r7 | 0.36646 | 0.36189163 |
| Shf | 0.00826 | -0.92200647 |
| 1700010B09Rik | 0.00188 | -1.1975031 |
| Gm5737 | 0.06218 | -1.19299148 |
| Olfr292 | 0.00144 | -1.31884045 |
| Pdilt | 0.00145 | -1.67907415 |
| Phactr2 | 0.00221 | -0.82511602 |
| E330035G20Rik | 0.00144 | -1.30017714 |
| 4921513I08Rik | 0.00144 | -1.26560808 |
| Relt | 0.0232 | -0.56264899 |
| Scn4b | 0.38987 | -0.44743761 |
| C230079O03Rik | 0.00144 | -1.26560808 |
| Cux1 | 0.00144 | -1.55884611 |
| Tmem184c | 0.00144 | 2.34910552 |
| Gm13598 | 0.00666 | -1.84151548 |
| A830092I05Rik | 0.11576 | -0.79535281 |
| Bex6 | 0.00144 | -1.28116048 |
| Gm19600 | 0.00144 | -1.28898079 |
| Tmprss11c | 0.00424 | -1.20978554 |
| Skida1 | 0.00993 | -1.36337518 |
| Mphosph10 | 0.00377 | 1.38843721 |
| Trpm2 | 0.0089 | -1.14560391 |
| Slc36a2 | 0.55788 | 0.26834006 |
| Unc13b | 0.01127 | 1.07927415 |
| Aqp6 | 0.42955 | -0.22754946 |
| BC029722 | 0.00145 | -0.94761754 |
| Gm33308 | 0.00144 | -1.26428512 |
| Tas2r134 | 0.00315 | -1.34034747 |
| 3110001L01Rik | 0.01969 | -2.63561827 |
| Cacna1i | 0.20098 | -0.38289547 |
| Pex6 | 0.00441 | -1.42247556 |
| Crabp1 | 0.00904 | -1.4406427 |
| Gm36738 | 0.01272 | -1.40219487 |
| 1700126G02Rik | 0.01696 | -1.12385295 |
| Myo1e | 0.31624 | -1.07465862 |
| Lamp5 | 0.53464 | -0.17239003 |
| Raxos1 | 0.31406 | -0.42573543 |
| Zfp641 | 0.61759 | 0.11782237 |
| Paqr9 | 0.19479 | 0.2785242 |
| Gm30074 | 0.02862 | -1.03550528 |
| Slpi | 0.03172 | -1.05920832 |
| Myo1f | 0.00238 | -1.0226713 |
| Trim72 | 0.00681 | -0.92551825 |
| C230072F16Rik | 0.2958 | -1.12128828 |
| Fanci | 0.00144 | -1.28116048 |
| Gm30489 | 0.00721 | -1.4499447 |
| Tmem211 | 0.00144 | -1.53063196 |
| Flg2 | 0.04031 | -1.92958365 |
| Sgol1 | 0.18812 | -0.66591232 |
| Gm10688 | 0.00144 | -1.30713006 |
| AI987944 | 0.00144 | 1.8949573 |
| Wdr37 | 0.01024 | -1.28769669 |
| Gm19269 | 0.01051 | -2.87091202 |
| Gm19272 | 0.00144 | -1.29746368 |
| LOC105245970 | 0.00144 | -1.27165604 |
| Gm39874 | 0.81141 | 0.13289536 |
| B3gntl1 | 0.34098 | -0.31601513 |
| 4930488L21Rik | 0.00144 | -1.25118919 |
| Rpl12 | 0.00704 | -0.72081244 |
| Rab29 | 0.01199 | -0.66686405 |
| Psen2 | 0.97323 | 0.0230647 |
| Tmem120b | 0.01475 | -0.71075937 |
| Prpf39 | 0.05646 | 0.9256554 |
| Tdgf1 | 0.69004 | 0.27173927 |
| Gm4872 | 0.00145 | -1.26219879 |
| A930012L18Rik | 0.38391 | 0.29745739 |
| Prkcq | 0.02237 | -0.87942611 |
| F830004D09Rik | 0.0391 | 1.21868219 |
| Gm4598 | 0.00385 | -1.15734821 |
| Btbd19 | 0.3485 | -0.43398977 |
| Olfr1316 | 0.00144 | -1.28116048 |
| Irak3 | 0.03948 | -0.45532054 |
| Gm38434 | 0.08585 | -0.84459662 |
| Olfr127 | 0.00173 | -1.6426896 |
| D330050G23Rik | 0.04 | -0.72528931 |
| Ranbp17 | 0.01269 | 0.79862216 |
| Slc26a2 | 0.04878 | 0.43470113 |
| Gm9831 | 0.00144 | -1.52481061 |
| LOC102639076 | 0.01404 | -0.89319377 |
| 1700119H24Rik | 0.11449 | -0.78791609 |
| Nr1h2 | 0.03037 | -0.6571194 |
| H2-T10 | 0.00525 | -0.9508884 |
| Olfr1252 | 0.68423 | -0.18672709 |
| Gm36177 | 0.0048 | -1.14198351 |
| Gm12320 | 0.10524 | -0.83545089 |
| Rfpl4b | 0.00144 | -1.30713006 |
| Brwd3 | 0.00144 | 2.77692841 |
| 5033403F01Rik | 0.77946 | 0.09154079 |
| Vps13a | 0.54346 | 0.46694121 |
| Mms19 | 0.69678 | 0.10729353 |
| 4921517D22Rik | 0.00145 | -1.22376446 |
| Trmt61b | 0.00144 | -1.20555712 |
| Kif13b | 0.1787 | 0.26943558 |
| Gm38403 | 0.88341 | 0.05840934 |
| Tnpo3 | 0.68302 | 0.06862518 |
| BB131254 | 0.99374 | -0.00662781 |
| Gm5600 | 0.00244 | -1.72607099 |
| 2500002B13Rik | 0.32311 | -0.28660022 |
| Mfsd10 | 0.07764 | -0.47686123 |
| Grm6 | 0.02743 | -0.97379672 |
| Gm3336 | 0.00411 | -1.63160592 |
| 4930439A04Rik | 0.00145 | -1.2388121 |
| Nudt19 | 0.29932 | 0.47038447 |
| Zc3hc1 | 0.04986 | -0.57698193 |
| Gm15834 | 0.52402 | 0.28769669 |
| 4930432M17Rik | 0.00145 | -0.92255046 |
| Prdm11 | 0.00144 | -1.40538302 |
| 1700026J14Rik | 0.25335 | -0.44006014 |
| LOC102642672 | 0.00144 | -1.25118919 |
| Klhl36 | 0.00144 | -1.5298028 |
| Gm2491 | 0.25287 | -0.72293101 |
| 2610035F20Rik | 0.00144 | -1.29746368 |
| Ccbl2 | 0.29599 | -0.53022538 |
| Tubb4b | 0.08871 | -0.50911494 |
| St8sia4 | 0.9203 | -0.02923525 |
| Gpd1l | 0.0076 | -0.70482398 |
| Gm34751 | 0.01213 | -1.35424525 |
| Zfp626 | 0.06424 | 0.48815753 |
| Rap1b | 0.36424 | 0.17082448 |
| Mettl17 | 0.90297 | -0.06530382 |
| Nprl3 | 0.05801 | -0.52904566 |
| 9530034A14Rik | 0.00465 | -2.2904563 |
| Myh13 | 0.02718 | -0.48483861 |
| Mt1 | 0.26381 | -0.28730434 |
| Arfgef2 | 0.30225 | -0.3279971 |
| Edar | 0.00494 | -1.04898329 |
| Dnah14 | 0.01546 | -1.02967477 |
| Rwdd1 | 0.00278 | 0.84610495 |
| Hpse2 | 0.00144 | -1.14247101 |
| 4933424L21Rik | 0.01428 | -2.01871323 |
| Olfr592 | 0.00145 | -1.31323225 |
| Gm26588 | 0.26804 | 0.78707941 |
| Meaf6 | 0.00537 | -0.86679188 |
| Gm13269 | 0.00144 | -1.47699301 |
| Gm19276 | 0.10296 | -0.99308048 |
| V1ra8 | 0.05207 | -1.42471505 |
| 9230114J08Rik | 0.00144 | -1.26560808 |
| Fam122b | 0.01746 | 2.14114411 |
| Cd2ap | 0.07124 | 0.50703622 |
| Ccnd1 | 0.13959 | -0.28594545 |
| Srrm2 | 0.09861 | -0.35963478 |
| Ppid | 0.00985 | 0.75449976 |
| Zfp407 | 0.03128 | 0.61783501 |
| Sorcs2 | 0.29917 | 1.23101889 |
| 5430431A17Rik | 0.2581 | 0.31732046 |
| Mrs2 | 0.03522 | 0.41194809 |
| 1700072B07Rik | 0.21359 | -0.74704078 |
| Cyba | 0.0031 | -0.84575622 |
| LOC102632690 | 0.00144 | -1.29746368 |
| Orm3 | 0.10363 | -0.95793004 |
| Trpm1 | 0.00144 | -1.2383131 |
| Hecw1 | 0.02393 | -0.6416984 |
| 1700018A04Rik | 0.00632 | -0.61565145 |
| Kirrel3 | 0.01785 | -0.63901698 |
| Abhd12 | 0.00563 | -0.82552371 |
| Ube2j2 | 0.00981 | -0.67892228 |
| Faddos | 0.00145 | -1.26219879 |
| 2410006H16Rik | 0.00144 | -1.09176063 |
| Hoxc8 | 0.09445 | -1.12250304 |
| Fbxw8 | 0.05546 | -0.5746001 |
| 4930593C16Rik | 0.00144 | -1.29864962 |
| Klhl28 | 0.21068 | -0.22345197 |
| Daxx | 0.00583 | -0.67515599 |
| Milr1 | 0.13661 | -0.39688406 |
| Psme3 | 0.04581 | -0.42052911 |
| Tlr5 | 0.0165 | -1.16215991 |
| Nudt13 | 0.33299 | 0.24285785 |
| Rax | 0.00329 | -1.19409336 |
| Gm20865 | 0.00144 | -1.30713006 |
| Gm10782 | 0.05074 | -1.11328333 |
| E030013I19Rik | 0.63046 | -0.32080561 |
| Grid2ip | 0.00882 | -1.99099957 |
| Tmem178b | 0.09453 | -0.81152909 |
| Prdm9 | 0.04089 | 0.99841739 |
| Vipr2 | 0.68982 | 0.2047821 |
| Gm33936 | 0.05502 | -2.15217138 |
| Mplkip | 0.15725 | 0.24768133 |
| Nxpe2 | 0.22894 | -0.22794814 |
| Gm32167 | 0.00144 | -1.28116048 |
| Pdp2 | 0.0328 | -0.54545022 |
| Pfpl | 0.00232 | -1.15063496 |
| 2310015A10Rik | 0.63042 | -0.20831605 |
| 2610016A17Rik | 0.00144 | -1.30017714 |
| B230119M05Rik | 0.00463 | -1.48164402 |
| Cplx4 | 0.00296 | -1.24082201 |
| A830008E24Rik | 0.05757 | -1.45656689 |
| A730006G06Rik | 0.01247 | -0.78924393 |
| LOC105246305 | 0.02666 | -0.95694161 |
| Pdcd2l | 0.37597 | -0.33874837 |
| Recql5 | 0.85475 | -0.07322861 |
| BC048609 | 0.04814 | -0.42917187 |
| 5430427N15Rik | 0.00145 | -1.2388121 |
| Gm10931 | 0.51388 | -0.24119552 |
| Fdft1 | 0.58935 | -0.17050846 |
| 9430021M05Rik | 0.03518 | -0.93175814 |
| Trmt10b | 0.0626 | 0.39223312 |
| Grin2d | 0.00144 | -1.90626628 |
| LOC102635521 | 0.02224 | -1.41972937 |
| Asb14 | 0.02057 | 0.71250661 |
| Trav14n-1 | 0.26008 | -0.34809737 |
| 1700066B19Rik | 0.08448 | -0.77838463 |
| Smc3 | 0.00144 | 2.06949317 |
| Pde8b | 0.28317 | -0.32031938 |
| Enpp1 | 0.46666 | -0.13682992 |
| Gm17644 | 0.03594 | -0.87369633 |
| Gramd2 | 0.00288 | -1.01721208 |
| Olfr6 | 0.00144 | -2.13545309 |
| Shkbp1 | 0.33168 | -0.7750226 |
| Sctr | 0.00144 | -1.29746368 |
| Ube2f | 0.9001 | -0.09467814 |
| Gm9799 | 0.07578 | -0.55899748 |
| Vsig10 | 0.00472 | -0.78831702 |
| Susd1 | 0.00253 | -1.43915615 |
| Sox17 | 0.01504 | -0.78664219 |
| Gm1661 | 0.72956 | -0.12826843 |
| Cfap54 | 0.42932 | -0.16788935 |
| Asap2 | 0.14964 | -0.55147379 |
| Prlh | 0.43523 | -0.29407087 |
| Runx1t1 | 0.55923 | -0.79371374 |
| Uchl1os | 0.4251 | -0.44427248 |
| Olfr290 | 0.00145 | -1.2388121 |
| Epha3 | 0.00145 | -1.22376446 |
| Gm815 | 0.00144 | -1.26560808 |
| Epb4.1l5 | 0.00144 | -1.31495282 |
| 9530003J23Rik | 0.60779 | -0.55641245 |
| Gm11634 | 0.00144 | -1.37850499 |
| Msantd2 | 0.00144 | 1.83037087 |
| 4930402H24Rik | 0.04758 | -0.36719756 |
| Dgki | 0.00794 | -0.87763815 |
| Med6 | 0.04858 | -0.4008674 |
| Frem2 | 0.00465 | -0.76657406 |
| 4930503O07Rik | 0.00144 | -1.25118919 |
| 2610028E06Rik | 0.20888 | -1.59536635 |
| C130046K22Rik | 0.3317 | 0.35435996 |
| Zrsr2 | 0.91638 | -0.07085342 |
| Zfp53 | 0.00144 | 2.39228351 |
| Gm19783 | 0.00864 | -1.07099791 |
| Tmprss11g | 0.01926 | -0.98294245 |
| Mtus2 | 0.03618 | -1.05018656 |
| Ppp1r12a | 0.15221 | 0.61436723 |
| LOC105245763 | 0.08746 | 0.46320567 |
| Sgol2a | 0.00269 | 0.94884144 |
| 4930517O19Rik | 0.00336 | -1.20864476 |
| A230083G16Rik | 0.35526 | -0.69219304 |
| Prr16 | 0.05088 | -0.96545331 |
| B930082K07Rik | 0.02448 | -1.31216903 |
| Olfr467 | 0.00144 | -1.28116048 |
| Gm34785 | 0.0187 | -0.81031247 |
| Olfr1385 | 0.05831 | -1.06521001 |
| Olfr781 | 0.00144 | -1.25118919 |
| Lipm | 0.00144 | -1.29049509 |
| Dok2 | 0.03722 | -1.85839818 |
| Matn2 | 0.64261 | 0.20892756 |
| Rad51c | 0.09393 | -0.47811477 |
| Gm4262 | 0.98802 | -0.00440633 |
| Pcdh9 | 0.05632 | 1.32090423 |
| Lnpep | 0.00144 | 1.0566677 |
| Acacb | 0.00176 | -1.31470426 |
| Ccdc82 | 0.02343 | 0.713661 |
| Tmem19 | 0.89583 | -0.04839496 |
| Gm39307 | 0.00144 | -1.30713006 |
| B930019K04Rik | 0.00145 | -1.25646236 |
| Fnip2 | 0.39354 | -0.21339216 |
| Skint5 | 0.12979 | -0.81369993 |
| 4930428E07Rik | 0.03716 | -0.96620766 |
| Olfr1249 | 0.00144 | -1.29085005 |
| Gm7244 | 0.00777 | -1.20707535 |
| Tmem145 | 0.48858 | -0.43671292 |
| Lrcol1 | 0.00144 | -1.2383131 |
| C730014E05Rik | 0.00708 | 1.12742125 |
| Sla | 0.10727 | -0.45123049 |
| Taf1a | 0.04601 | 0.94443732 |
| Gm33778 | 0.04218 | -0.84136009 |
| Gabrq | 0.00144 | -1.25118919 |
| Cpd | 0.81538 | 0.04226008 |
| Ict1 | 0.03219 | -0.76403383 |
| Ptn | 0.72681 | -0.16313605 |
| Haus2 | 0.25522 | 0.34394841 |
| S100z | 0.00144 | -1.30713006 |
| Gm6117 | 0.00144 | -1.29085005 |
| 4930451G21Rik | 0.52194 | -0.37957322 |
| Apol9b | 0.00943 | -0.95113377 |
| Lipg | 0.00766 | -1.24795066 |
| Gm614 | 0.82025 | -0.15923612 |
| Tnnt3 | 0.69677 | -0.10072423 |
| Men1 | 0.00144 | -1.04070531 |
| Odf3l2 | 0.03218 | -0.65256717 |
| Clcnkb | 0.2326 | 0.33419439 |
| Sart1 | 0.01293 | -0.69871748 |
| Sqstm1 | 0.05645 | -0.36254153 |
| Gpr82 | 0.03371 | -0.94444466 |
| Suv420h2 | 0.05 | -0.47561107 |
| Kansl1l | 0.12361 | 0.3157318 |
| 4930428O21Rik | 0.07412 | -1.71995711 |
| 9630041A04Rik | 0.00144 | -1.21153109 |
| Dnajb12 | 0.43247 | -0.47821836 |
| Polr3h | 0.20852 | -0.51796856 |
| Gm36556 | 0.01052 | -0.55617589 |
| Megf9 | 0.04029 | -0.65133793 |
| C030013E06Rik | 0.0037 | -1.28081519 |
| Nr3c2 | 0.04551 | 0.56843764 |
| Far1os | 0.00144 | -1.27699683 |
| BC018473 | 0.00157 | -1.19247859 |
| C330024C12Rik | 0.00144 | -1.28898079 |
| Gm35188 | 0.01129 | -1.29878664 |
| Unc80 | 0.12454 | -0.90551228 |
| Clk1 | 0.0031 | 1.59206643 |
| Kif6 | 0.36068 | 0.16224174 |
| 2700081O15Rik | 0.0557 | -0.37860233 |
| Cdh11 | 0.07402 | 0.69460513 |
| Zc3h12b | 0.00145 | -1.2388121 |
| 4933433F19Rik | 0.04475 | -0.53872597 |
| Gm12519 | 0.25795 | -0.43230211 |
| Afg3l1 | 0.03093 | -0.57369751 |
| 4930578E11Rik | 0.03119 | -0.83378946 |
| Gm13944 | 0.00144 | -1.28898079 |
| Gm7489 | 0.00144 | -1.4913104 |
| 4930594O21Rik | 0.00144 | -1.51201763 |
| Elf5 | 0.00158 | -1.2446827 |
| Snx10 | 0.90784 | -0.05905665 |
| 4930542C21Rik | 0.61793 | 0.33217529 |
| Gm11342 | 0.00144 | -1.27165604 |
| Nif3l1 | 0.60103 | 0.28648641 |
| Arpc3 | 0.0443 | -0.42685828 |
| LOC105246996 | 0.57975 | -0.1471699 |
| Dlst | 0.42681 | -0.16890967 |
| Iffo2 | 0.01008 | -0.7273611 |
| Gm12606 | 0.93894 | 0.07710852 |
| Ska3 | 0.69985 | -0.20565763 |
| Rab25 | 0.00144 | -1.26934956 |
| D030045P18Rik | 0.00271 | -1.17180261 |
| Vmn1r55 | 0.00144 | -1.26934956 |
| Tmem91 | 0.02117 | -0.82822404 |
| Atp2b4 | 0.03043 | -0.79807492 |
| LOC102642253 | 0.00144 | -1.28116048 |
| Wdr64 | 0.11532 | -0.88511728 |
| Gm10319 | 0.08823 | -2.19348234 |
| Capn12 | 0.0049 | -0.81050248 |
| LOC105244433 | 0.03399 | -0.60084668 |
| Actr3 | 0.24221 | -0.25231073 |
| Gm15232 | 0.33003 | -0.28092009 |
| LOC666331 | 0.00145 | -1.22376446 |
| Kri1 | 0.45012 | -0.15535383 |
| Taar7e | 0.00144 | -1.28116048 |
| Ankrd28 | 0.59546 | -0.14995076 |
| Olfr970 | 0.01386 | -1.69113109 |
| Trim38 | 0.5188 | 0.2670648 |
| Eppk1 | 0.01023 | -1.34121185 |
| Adamts18 | 0.01478 | -0.82325997 |
| Gm37896 | 0.00144 | -1.30713006 |
| Dact2 | 0.034 | -0.84774094 |
| Gm15894 | 0.00144 | -1.29746368 |
| Nov | 0.01218 | 0.75581436 |
| Gm10561 | 0.00916 | -1.06669723 |
| Extl1 | 0.08866 | -2.27211457 |
| Olfr545 | 0.00144 | -1.46816894 |
| Tango6 | 0.00721 | -0.82635183 |
| Myo18b | 0.00144 | -1.36034142 |
| Gm32635 | 0.46582 | -0.55621953 |
| LOC105247620 | 0.00149 | -1.22582153 |
| St8sia3os | 0.15649 | -0.75164493 |
| Gm12371 | 0.00144 | -1.25118919 |
| Spats2 | 0.22953 | 0.44936291 |
| 4933404K13Rik | 0.00149 | -1.28183002 |
| Gm12794 | 0.00144 | -1.28898079 |
| 5730424H11Rik | 0.19011 | -0.29962727 |
| Mtpn | 0.00149 | 1.18234531 |
| Noa1 | 0.00144 | -1.07001557 |
| Gm3693 | 0.00144 | -1.26560808 |
| Xrn2 | 0.16481 | -1.25587413 |
| Nlrp4g | 0.01885 | -2.85379801 |
| Rsph9 | 0.01669 | -1.39831189 |
| Cd33 | 0.56592 | 0.10885311 |
| LOC102643083 | 0.25925 | -0.5186232 |
| Prss35 | 0.04513 | -2.09658197 |
| Fermt1 | 0.00145 | -1.22376446 |
| Olfr298 | 0.00144 | -1.33632959 |
| Asun | 0.00144 | 1.17861739 |
| Mamdc4 | 0.54339 | -0.15398622 |
| Ikzf3 | 0.14167 | -0.58562212 |
| Catsper4 | 0.03114 | -1.01362769 |
| LOC102638195 | 0.63728 | -0.10269154 |
| Aldh16a1 | 0.29956 | 0.47397463 |
| Ggps1 | 0.00221 | 1.08855364 |
| Slc4a3 | 0.72124 | 0.07895915 |
| C230021G24Rik | 0.00193 | -1.24713717 |
| Fhitos | 0.00145 | -1.2388121 |
| Fgd1 | 0.00144 | -1.27801956 |
| A630075F10Rik | 0.02815 | -1.95284873 |
| Nova2 | 0.02616 | -0.55184607 |
| Gm4984 | 0.91909 | -0.02887351 |
| Vps9d1 | 0.81914 | 0.11365064 |
| Ces3b | 0.00144 | -1.25118919 |
| Gcsh | 0.05367 | -0.39369153 |
| Rnase11 | 0.00144 | -1.28898079 |
| Gm31932 | 0.39168 | 0.147548 |
| 4930528H21Rik | 0.00144 | -1.27165604 |
| Abcc6 | 0.60333 | 0.23873568 |
| Gm15199 | 0.05742 | -0.78178978 |
| Nox4 | 0.00249 | 1.67150196 |
| Gm26627 | 0.0683 | -1.16401352 |
| Runx3 | 0.00144 | -1.33632959 |
| Nae1 | 0.00144 | 1.68668139 |
| 1700123I01Rik | 0.06046 | -1.28640711 |
| Gm5127 | 0.00144 | -1.29746368 |
| Il1f6 | 0.00144 | -1.26560808 |
| Slc22a27 | 0.00144 | -1.26934956 |
| Sh2b3 | 0.98375 | -0.00381949 |
| Gm33201 | 0.04016 | -0.95353231 |
| Syndig1l | 0.21096 | -0.41976076 |
| Gm12227 | 0.00144 | -1.29085005 |
| Cd3d | 0.00667 | -1.0936149 |
| Trim8 | 0.00758 | -0.84167771 |
| Ube4b | 0.8034 | -0.04679213 |
| Plcb3 | 0.02278 | -0.46070958 |
| Ypel4 | 0.18524 | -0.33144298 |
| Dmr | 0.15757 | -0.88632103 |
| Tnfsf10 | 0.00144 | -1.3226194 |
| Dag1 | 0.00144 | -1.2267887 |
| C030018K13Rik | 0.00144 | -1.46357118 |
| Gm35676 | 0.1166 | -0.83468084 |
| LOC102636313 | 0.687 | 0.24424823 |
| Kbtbd12 | 0.0197 | 0.50671092 |
| Ehf | 0.08172 | -0.92819591 |
| Ddrgk1 | 0.03545 | -0.94725632 |
| 1700112K13Rik | 0.14885 | -0.33015119 |
| Gm36807 | 0.00144 | -1.32313902 |
| Gm11517 | 0.0022 | -0.90582648 |
| Olfr293 | 0.03453 | -2.01415838 |
| Cldn24 | 0.08367 | -0.52617874 |
| Tpi1 | 0.65159 | -0.21903928 |
| Mtfr2 | 0.00144 | -1.31495282 |
| Dlgap4 | 0.29907 | -0.31515443 |
| 2410021H03Rik | 0.30874 | -0.66206473 |
| 1700030A11Rik | 0.00144 | -1.25588837 |
| Spag17os | 0.01042 | -2.45992365 |
| Ttc25 | 0.01554 | -1.72104773 |
| Tmem125 | 0.00144 | -1.27699683 |
| Gm39139 | 0.00144 | -1.26560808 |
| Gm30352 | 0.02156 | -1.70069667 |
| 4933431G14Rik | 0.72286 | 0.06435895 |
| Cd55 | 0.04245 | -0.46283545 |
| Gm36241 | 0.01786 | -1.04100801 |
| Cdh19 | 0.00144 | 1.34897877 |
| Rpia | 0.42186 | -0.16201626 |
| Stk33 | 0.01468 | -0.95185142 |
| Prex2 | 0.12077 | 0.6537331 |
| Gm30333 | 0.90905 | -0.03997896 |
| Bloodlinc | 0.33026 | -0.61474612 |
| 5033403H07Rik | 0.07369 | -0.87729452 |
| Rad51d | 0.02184 | -0.75892137 |
| Snapc2 | 0.01608 | -0.88114302 |
| Matr3 | 0.00145 | 1.67086541 |
| C530025M09Rik | 0.00144 | -1.28116048 |
| 9330162B11Rik | 0.01044 | -1.44248876 |
| Amer2 | 0.00432 | -1.70237662 |
| A530053M12Rik | 0.00589 | -1.02871888 |
| Hoxc11 | 0.00144 | -1.27165604 |
| Gm7616 | 0.00733 | -1.53778094 |
| Gm12153 | 0.00144 | -1.30713006 |
| Gm6416 | 0.00409 | 1.16736807 |
| Kcnd1 | 0.03408 | -0.91373947 |
| Bpifb6 | 0.01149 | -0.96933705 |
| Gm13481 | 0.51464 | 0.28874954 |
| Mettl20 | 0.02629 | 0.4991046 |
| Dlec1 | 0.00144 | -1.27699683 |
| Tyw5 | 0.00149 | 1.87354643 |
| Alas1 | 0.65393 | 0.34662119 |
| Gm2464 | 0.14227 | -3.33177635 |
| Apold1 | 0.0258 | 0.71336045 |
| Desi2 | 0.00144 | 1.37733355 |
| Gm13630 | 0.00144 | -1.30017714 |
| Farsb | 0.01188 | 0.93726609 |
| Rest | 0.09889 | 0.29904538 |
| Ppp2r1a | 0.69969 | 0.19734788 |
| Krtap5-4 | 0.01586 | -0.82493912 |
| BC031181 | 0.9326 | -0.02760985 |
| Srp68 | 0.78755 | 0.07945237 |
| 2310005A03Rik | 0.00437 | -1.35450997 |
| 1700029J08Rik | 0.17007 | -0.72266071 |
| Lacc1 | 0.01271 | 1.92412985 |
| 1810063I02Rik | 0.00144 | -1.67061238 |
| Gm38416 | 0.05699 | 0.68935476 |
| 4930556G01Rik | 0.68413 | 0.10018831 |
| Scfd1 | 0.93324 | -0.11970065 |
| 2210414B05Rik | 0.00258 | -1.19958943 |
| Gm34481 | 0.01892 | -1.19120088 |
| Gm35771 | 0.007 | -0.71631698 |
| Unc80 | 0.00144 | -1.36950815 |
| Lrguk | 0.00144 | -1.28898079 |
| Gm39216 | 0.22733 | -0.5661085 |
| Cald1 | 0.00145 | 1.61380794 |
| Gm26787 | 0.04642 | -2.48165071 |
| Iah1 | 0.27215 | -0.40527687 |
| Mptx2 | 0.00144 | -1.24466058 |
| A730009L09Rik | 0.01019 | -1.44396052 |
| Gm26806 | 0.11294 | -0.9914635 |
| 4930534H18Rik | 0.98293 | 0.0088326 |
| Pds5a | 0.00468 | 0.92961594 |
| Gm715 | 0.25283 | -0.37908382 |
| Rnf26 | 0.04967 | -0.55878621 |
| Rps6ka5 | 0.70595 | 0.06452848 |
| Kcng2 | 0.14686 | -0.54970229 |
| Gm11278 | 0.00184 | -1.19286777 |
| Hmgb3 | 0.05397 | -0.50336099 |
| Olfr1535 | 0.00144 | -1.29746368 |
| Gm14379 | 0.47062 | 0.50173361 |
| Smpdl3b | 0.46548 | -0.2633589 |
| 9430018G01Rik | 0.00144 | -1.32313902 |
| 5930435M05Rik | 0.03914 | -0.90904707 |
| Gm31134 | 0.00144 | -1.63222844 |
| Slc25a1 | 0.00144 | -1.4123348 |
| Gm30094 | 0.00604 | -1.58998782 |
| Gm8773 | 0.00144 | -1.27165604 |
| Glmp | 0.05976 | -0.63304411 |
| Lgr5 | 0.07362 | -1.01612101 |
| Gm33335 | 0.11208 | -2.05909417 |
| Olfr1129 | 0.00145 | -1.22376446 |
| Xpnpep2 | 0.85857 | -0.19965645 |
| E430020m11rik | 0.00749 | -1.31372447 |
| Ppia | 0.04293 | -0.39560142 |
| Olfr146 | 0.00145 | -1.2388121 |
| Lgalsl | 0.94931 | -0.01384687 |
| Cela3b | 0.88289 | 0.06469568 |
| LOC105244175 | 0.02269 | 0.74413126 |
| Olfr358 | 0.00824 | -1.10802128 |
| Gm13056 | 0.00254 | -1.10534241 |
| 5330413P13Rik | 0.00232 | -1.15063496 |
| Asz1 | 0.00144 | -1.33632959 |
| Gm15689 | 0.49498 | -0.22370638 |
| 4930550C14Rik | 0.00317 | -1.12815152 |
| Gm11961 | 0.00144 | -1.27699683 |
| Gm28516 | 0.00144 | -1.26301121 |
| Reln | 0.77095 | -0.17792995 |
| Rad23a | 0.344 | -0.29747468 |
| Gm7628 | 0.21828 | 0.80941708 |
| Rad1 | 0.3164 | 0.23523556 |
| Camk4 | 0.02862 | -1.03550528 |
| 8030462N17Rik | 0.00708 | -0.81749183 |
| Oxct1 | 0.02381 | 0.68537572 |
| Nop58 | 0.00327 | 1.82387795 |
| Tigd5 | 0.00455 | -0.82099601 |
| Sdsl | 0.52833 | -0.38923576 |
| Arhgef38 | 0.00254 | -1.26636244 |
| Ccdc9 | 0.14086 | -0.3002964 |
| Acss2 | 0.15143 | -0.37512099 |
| Gm8439 | 0.00144 | -1.30713006 |
| A230009B12Rik | 0.00144 | -1.27165604 |
| LOC105244659 | 0.01191 | -1.40503413 |
| Cideb | 0.80208 | -0.07319526 |
| Cyth1 | 0.99562 | -0.00139703 |
| 4831410D14 | 0.02583 | -1.12164699 |
| Kcnk16 | 0.20623 | -0.31145477 |
| Banf2os | 0.81467 | -0.06076721 |
| Mtg1 | 0.07796 | -0.65302235 |
| 4930543N07Rik | 0.12562 | -0.81981054 |
| Gm29154 | 0.01447 | -0.82239636 |
| Cd2 | 0.00336 | -1.17136338 |
| Gm31887 | 0.00144 | -1.38007777 |
| Defb4 | 0.00144 | -1.29864962 |
| Dock7 | 0.16384 | 0.4483785 |
| N4bp2os | 0.00144 | -1.25622482 |
| Gtpbp10 | 0.21068 | 0.55108303 |
| Gm28703 | 0.00267 | -1.01564035 |
| Gm31908 | 0.00166 | -1.29367065 |
| Gm9872 | 0.01047 | -1.90014657 |
| E030018B13Rik | 0.0557 | -0.75060626 |
| Pfkp | 0.25472 | 0.30614067 |
| 7330404K18Rik | 0.00144 | -1.27165604 |
| Smarce1 | 0.20792 | 0.21293266 |
| Gm33948 | 0.00145 | -1.2388121 |
| Xrcc6 | 0.10905 | -0.27751624 |
| 1700031F05Rik | 0.00186 | -1.1994385 |
| Cyp4x1os | 0.00383 | -1.13029182 |
| 4930502A04Rik | 0.19719 | -0.74444472 |
| Gm14634 | 0.13762 | 0.58422084 |
| Nek7 | 0.25655 | 0.37158576 |
| Ppp1r3e | 0.01286 | -0.54896611 |
| 2610528A11Rik | 0.70804 | -0.21099411 |
| Psmc4 | 0.01682 | -0.61568417 |
| 4930428D20Rik | 0.00144 | -1.26934956 |
| D830013O20Rik | 0.09146 | 2.35050199 |
| Csnk1d | 0.71289 | -0.09936353 |
| 1700039E22Rik | 0.08257 | -0.91866935 |
| Gm973 | 0.42315 | -0.26068449 |
| Uggt1 | 0.06275 | -0.32767751 |
| Myo5b | 0.02278 | -0.67278975 |
| 4930556J02Rik | 0.10831 | -0.56772296 |
| 4930557F08Rik | 0.34959 | -0.6185367 |
| E2f2 | 0.06193 | -0.64683288 |
| Bbs2 | 0.01233 | 1.16163496 |
| Abhd16a | 0.32694 | -0.33282717 |
| Cfap69 | 0.00281 | 1.20769921 |
| Auh | 0.12562 | 0.79479504 |
| Zfp872 | 0.04958 | -0.96748156 |
| Gm13177 | 0.5185 | -0.16623963 |
| Zfp385c | 0.04916 | -0.72847327 |
| Rgag1 | 0.05736 | -1.0690351 |
| Rbakdn | 0.26401 | -0.75419597 |
| A930013F10Rik | 0.00964 | 1.83748753 |
| D630013N20Rik | 0.00144 | -1.30713006 |
| Celrr | 0.0596 | -1.53591275 |
| Slain1os | 0.00215 | -1.13684067 |
| Prkar2a | 0.99417 | 0.00147447 |
| Txndc16 | 0.0041 | 1.08746439 |
| 4632428C04Rik | 0.00144 | -1.29085005 |
| Psg22 | 0.00295 | -1.38077972 |
| Eif4e | 0.00144 | -1.29085005 |
| Gm27166 | 0.00144 | -1.29864962 |
| Kcng2 | 0.5979 | -0.23899366 |
| Foxn4 | 0.13022 | -0.82431196 |
| Pwwp2a | 0.00789 | 1.62547688 |
| Gm13089 | 0.00144 | -1.29746368 |
| Ucp3 | 0.07656 | -0.40373728 |
| Tmem207 | 0.03092 | -1.38784312 |
| Gm1600 | 0.07195 | -0.79618843 |
| Adgrl2 | 0.01042 | 0.74868466 |
| Klhl40 | 0.00328 | -0.95673624 |
| Gm38635 | 0.26525 | -0.39902646 |
| 4930540M03Rik | 0.7679 | -0.18368419 |
| Car5a | 0.00149 | -1.25408 |
| Ndfip2 | 0.03021 | 0.87162488 |
| Lin52 | 0.67832 | 0.06750507 |
| 4930502E09Rik | 0.22185 | -0.78472743 |
| Dgkeos | 0.05094 | -2.0891601 |
| Klhl7 | 0.0112 | -0.65542518 |
| Rmdn2 | 0.00144 | -1.44780261 |
| Cbr1 | 0.17887 | -0.29492992 |
| Afap1l1 | 0.23301 | -0.18631209 |
| Gm33800 | 0.00144 | -1.38836933 |
| Fbxw10 | 0.24002 | 0.28247939 |
| Gm11487 | 0.95265 | -0.01895958 |
| A530076I17Rik | 0.01925 | -1.1221074 |
| Zfp932 | 0.00144 | 3.00163754 |
| Dock2 | 0.08327 | 0.58267893 |
| Ncbp1 | 0.78866 | 0.04481691 |
| Olfr294 | 0.00145 | -1.2388121 |
| Capza2 | 0.00589 | -0.84685713 |
| 4930458D05Rik | 0.00284 | 0.77073699 |
| 9330169L03Rik | 0.37033 | -0.31219694 |
| Ttc39c | 0.06559 | 0.46517171 |
| Setd8 | 0.00144 | -1.53513511 |
| Aifm3 | 0.27108 | 0.46172835 |
| Cet1 | 0.06819 | -1.85564604 |
| 4930466F19Rik | 0.00144 | -1.27730451 |
| E130317F20Rik | 0.37928 | -0.34940709 |
| Heatr1 | 0.42604 | -0.13584652 |
| A630044M17Rik | 0.35871 | -0.55852168 |
| Gm30873 | 0.17373 | -0.31768619 |
| Spred3 | 0.0355 | -1.2887702 |
| Espn | 0.11231 | -1.05255991 |
| Gm17619 | 0.4021 | -0.29057805 |
| Gm10277 | 0.01413 | -2.44947844 |
| Nptn | 0.02528 | -0.62747466 |
| Defb23 | 0.02967 | -0.95751671 |
| LOC102638985 | 0.00144 | -1.29281267 |
| 1700030O20Rik | 0.00328 | -1.10633687 |
| Rgmb | 0.01542 | 1.11399763 |
| 5730435O14Rik | 0.14422 | -0.84270555 |
| Rhbdd1 | 0.06436 | -0.82847561 |
| Plxdc1 | 0.05017 | -1.39793238 |
| LOC102640035 | 0.00144 | -2.50691289 |
| Rap1gapos | 0.09102 | -1.21563008 |
| Fam166a | 0.00145 | -1.21796995 |
| Trpc7 | 0.6176 | -0.57916579 |
| Gm853 | 0.02036 | -0.91256225 |
| Gm21859 | 0.00145 | -1.33072139 |
| 1700022A22Rik | 0.08815 | -0.84051778 |
| Sphkap | 0.01323 | -1.05283004 |
| Gm2670 | 0.00144 | -1.32934245 |
| Gm26671 | 0.18132 | 0.49599124 |
| Crybb2 | 0.49174 | -0.59229655 |
| Fam198a | 0.36066 | -0.40198591 |
| AW121686 | 0.00144 | -1.29085005 |
| B230334C09Rik | 0.49225 | -0.31635434 |
| Xkrx | 0.01921 | -1.01433763 |
| Fra10ac1 | 0.08047 | 0.64225402 |
| Gdf3 | 0.00144 | -1.28116048 |
| Olfr1042 | 0.00144 | -1.28116048 |
| Atp5k | 0.00144 | -0.96237058 |
| Myl12a | 0.3358 | -0.32703582 |
| Dyrk2 | 0.15978 | -0.37741277 |
| 1700001J04Rik | 0.00494 | -0.85125589 |
| Tomm20 | 0.71943 | -0.07766695 |
| Olfr1357 | 0.00144 | -1.26560808 |
| Gm36756 | 0.25072 | -0.382058 |
| Ears2 | 0.89546 | -0.05923087 |
| Gm32312 | 0.97675 | -0.03736287 |
| 1110020A21Rik | 0.84838 | -0.05282206 |
| LOC545466 | 0.00144 | -1.28898079 |
| Gm12616 | 0.00626 | -3.57213166 |
| 1700029I15Rik | 0.6755 | 0.09532555 |
| Wdr95 | 0.00144 | -1.29085005 |
| Smox | 0.07596 | -0.33485207 |
| Gm10714 | 0.00144 | -1.29049509 |
| Tas2r126 | 0.50413 | -0.28749883 |
| Fam217b | 0.01158 | -0.81749258 |
| Ankra2 | 0.31383 | -0.25750968 |
| 4930550L05Rik | 0.15193 | -0.62266921 |
| Thap6 | 0.05772 | -0.40922714 |
| 6030443J06Rik | 0.18749 | 0.67973668 |
| Taf2 | 0.00378 | 0.96783613 |
| Rhox3a | 0.02012 | -1.18709549 |
| Srp72 | 0.04788 | -0.42950293 |
| Nlrp9c | 0.01557 | 1.60349736 |
| Rcor3 | 0.04348 | -0.47498624 |
| Gm33337 | 0.09874 | -0.89245938 |
| Prox2 | 0.09019 | -0.3189145 |
| Gm38293 | 0.55088 | 0.29989578 |
| Polr3k | 0.14822 | -0.42872075 |
| Gm7256 | 0.12834 | -1.0053594 |
| 4922502H24Rik | 0.40784 | 0.34305188 |
| Vmn1r179 | 0.00144 | -1.25118919 |
| Gm9958 | 0.48547 | 0.19180802 |
| Gm36560 | 0.00271 | -1.12385295 |
| Gm19395 | 0.67283 | -0.21303625 |
| Gpaa1 | 0.18172 | -0.46411396 |
| 1700027F09Rik | 0.00144 | -1.27165604 |
| Arhgef33 | 0.1738 | -0.72989609 |
| A930035D04Rik | 0.55333 | -0.13796881 |
| Gm32815 | 0.01947 | -2.01284665 |
| Dennd2a | 0.14363 | -0.68093008 |
| 5830416I19Rik | 0.02223 | -1.82597771 |
| Gm40330 | 0.00144 | -1.28116048 |
| Olfr1462 | 0.00192 | -1.37617779 |
| Gm32265 | 0.36181 | 0.37690806 |
| Gm11465 | 0.89964 | 0.05602474 |
| Lect1 | 0.52514 | 0.28999123 |
| Ide | 0.25795 | -0.21643586 |
| Akt1 | 0.01374 | -0.5440537 |
| Defb21 | 0.00144 | -1.20555712 |
| Gm40658 | 0.13238 | -2.24559739 |
| Dnah2os | 0.03336 | -0.51387083 |
| Syt11 | 0.05907 | -0.36448362 |
| Mdk | 0.01239 | -0.66663507 |
| Mettl21e | 0.00145 | -1.27532353 |
| Gm29678 | 0.02331 | -0.58391497 |
| Adcy2 | 0.00144 | -1.24353547 |
| Olfr955 | 0.00144 | -1.29085005 |
| Gm41257 | 0.15768 | -1.38941022 |
| Glis3 | 0.71848 | -0.13446333 |
| Gm8025 | 0.00733 | -1.10984709 |
| Krtdap | 0.00144 | -1.40930122 |
| Dnase2a | 0.14609 | -0.46035302 |
| Oscp1 | 0.01633 | -1.10278386 |
| Gm35404 | 0.38618 | 0.27651044 |
| Gm30790 | 0.00145 | -1.22518835 |
| LOC102642578 | 0.86137 | -0.07013551 |
| Kcna10 | 0.00144 | -1.21977358 |
| Nom1 | 0.00572 | 0.74459503 |
| 4930521E06Rik | 0.00144 | -1.30713006 |
| Gm16364 | 0.00144 | -2.38534377 |
| Loxl4 | 0.37588 | -0.50691447 |
| Nfkbid | 0.00729 | -1.78832879 |
| Gipc2 | 0.11991 | -0.25864701 |
| Dhrs2 | 0.01195 | -1.82332733 |
| Hif1a | 0.0195 | 1.03058823 |
| LOC102640562 | 0.00144 | 1.13908606 |
| Zfp839 | 0.90803 | -0.1355868 |
| Zc3h12d | 0.00144 | -1.25118919 |
| Gm33224 | 0.17185 | -0.35330523 |
| Nuak1 | 0.00149 | -1.2467961 |
| 1700028D13Rik | 0.05662 | -2.19554883 |
| Rpl41 | 0.00201 | -1.03384552 |
| Mrap2 | 0.81573 | 0.10445536 |
| Smarcb1 | 0.0325 | -0.56818137 |
| Ptprn2 | 0.94479 | 0.03626228 |
| 3300005D01Rik | 0.28053 | -0.50983262 |
| Efcab6 | 0.00144 | -1.28116048 |
| 5430428K19Rik | 0.00205 | -1.21461873 |
| 4933408N05Rik | 0.00145 | -1.24191372 |
| Glyat | 0.52914 | -0.3573786 |
| Fkbp5 | 0.01028 | 1.26432194 |
| B230112J18Rik | 0.00941 | -1.30205933 |
| Pak3 | 0.08264 | 0.88441215 |
| Prcp | 0.00367 | -0.77361714 |
| Rpf2 | 0.04278 | 0.612608 |
| Gm12059 | 0.79257 | -0.09981727 |
| Gm20742 | 0.00144 | -1.26934956 |
| Gm960 | 0.27136 | -1.01825107 |
| Gm13856 | 0.00144 | -1.29085005 |
| Fam135b | 0.00342 | -1.57269586 |
| 8430430B14Rik | 0.00144 | -1.25118919 |
| Gm3404 | 0.00144 | -1.66099786 |
| Gm16432 | 0.00144 | -1.30713006 |
| Gm35438 | 0.01076 | -1.24102684 |
| Cdkal1 | 0.10571 | -0.97033557 |
| Gm34973 | 0.00144 | -1.26560808 |
| Gm9926 | 0.00144 | -1.27165604 |
| Megf10 | 0.00359 | -1.77579417 |
| Rabl2 | 0.01201 | 1.33570954 |
| Aph1a | 0.00144 | -1.1515497 |
| Gm34568 | 0.00144 | -1.27165604 |
| Gsdmd | 0.24431 | -0.27483365 |
| Acsf2 | 0.00144 | -1.59941161 |
| 9130604C24Rik | 0.00151 | -1.4602828 |
| Jak2 | 0.00342 | 1.07358059 |
| B3glct | 0.01321 | 0.69717481 |
| Limd1 | 0.68294 | 0.14190348 |
| B230322F03Rik | 0.14491 | -0.71217734 |
| Kmo | 0.61642 | -0.25873658 |
| Stau1 | 0.02991 | -0.42227818 |
| 2610020F03Rik | 0.00144 | -1.28898079 |
| Mrps18b | 0.00638 | -0.74056863 |
| Rgs10 | 0.24328 | -0.20300209 |
| 1700044C05Rik | 0.00655 | -1.51963168 |
| Lhfpl3 | 0.00144 | -1.43884151 |
| 9630029G12Rik | 0.34975 | -0.55178394 |
| Adam32 | 0.28704 | 0.39670024 |
| Upf1 | 0.02913 | -0.78597994 |
| Magi2 | 0.41155 | -0.52339998 |
| 1700084C01Rik | 0.02396 | -1.25366643 |
| Syce3 | 0.07497 | -0.81983258 |
| Tmem217 | 0.00144 | -1.29085005 |
| Ptk6 | 0.6687 | 0.34679292 |
| Nckipsd | 0.17806 | -0.36199032 |
| Cln6 | 0.25043 | -0.35754089 |
| Ffar3 | 0.00144 | -1.15410761 |
| Gm3045 | 0.50074 | -0.44008232 |
| Rmdn2 | 0.08842 | -0.49702887 |
| Olfr1265 | 0.00144 | -1.27730451 |
| Ranbp1 | 0.57034 | -0.19386163 |
| Slc7a1 | 0.02032 | -0.58049252 |
| Zscan5b | 0.25764 | -0.54691517 |
| Camsap1 | 0.32205 | -0.19172574 |
| LOC433227 | 0.00144 | -1.28898079 |
| Ccdc170 | 0.05413 | -0.81098647 |
| Olfr628 | 0.00145 | -1.24191372 |
| Grm5 | 0.00187 | -1.25897127 |
| Vhl | 0.00928 | -0.5691986 |
| Exd2 | 0.0795 | -0.71140577 |
| Sae1 | 0.26208 | 0.31978738 |
| Pot1a | 0.10311 | 0.94040806 |
| Ccdc136 | 0.32604 | -0.17951263 |
| Olfr156 | 0.08267 | -1.59213966 |
| Gm4951 | 0.83976 | 0.08782727 |
| Psma4 | 0.24241 | 0.25863154 |
| 1700022H01Rik | 0.00562 | -0.86938471 |
| Hsd3b1 | 0.00144 | -1.44780261 |
| 1700100I10Rik | 0.26457 | -0.71027506 |
| 2010107E04Rik | 0.00144 | -0.95338256 |
| AI427809 | 0.37508 | -0.56537157 |
| Akap4 | 0.36069 | -0.31433122 |
| Nlrp4g | 0.00144 | -1.25118919 |
| Chn1 | 0.00717 | -1.41838511 |
| D130017N08Rik | 0.89973 | -0.05363094 |
| Crp | 0.0902 | -0.68145569 |
| Gna14 | 0.01964 | -0.53686669 |
| Cct3 | 0.12734 | 0.43903152 |
| Ppp2r3a | 0.00243 | 1.21711317 |
| Sec61a1 | 0.01634 | -0.74055024 |
| Wbscr16 | 0.99724 | -0.00179205 |
| Tbc1d30 | 0.62926 | -0.20492788 |
| Sez6l | 0.2235 | -0.9581925 |
| 1700071K01Rik | 0.90193 | -0.07324859 |
| Fam136a | 0.0021 | -0.88483093 |
| Ldlrap1 | 0.56315 | -0.14216941 |
| Brd8 | 0.00684 | 0.71769987 |
| Ccdc30 | 0.01431 | -0.9383227 |
| Gm13298 | 0.02001 | -1.56544511 |
| C330003G01Rik | 0.50843 | 0.18186275 |
| Gm17252 | 0.01905 | -1.7575786 |
| Daf2 | 0.04866 | 0.60654055 |
| Gm30505 | 0.00145 | -1.2388121 |
| Strap | 0.22347 | 0.58669099 |
| Itgb1bp1 | 0.90623 | 0.04243083 |
| Btnl2 | 0.00383 | -1.13029182 |
| 2700049A03Rik | 0.15275 | 0.40383159 |
| Pramef6 | 0.00144 | -1.28898079 |
| Gm31463 | 0.0037 | -1.11214255 |
| AA692955 | 0.07692 | -0.74542971 |
| Ccdc175 | 0.00144 | -1.28116048 |
| Gm39164 | 0.01106 | -1.18694642 |
| A930037O16Rik | 0.31936 | 0.36490622 |
| Slc5a2 | 0.00237 | -1.35169066 |
| Nlrp1b | 0.0111 | -1.02598223 |
| Ercc8 | 0.64583 | 0.16878422 |
| Pik3r4 | 0.17381 | -0.223973 |
| 4831407H17Rik | 0.00144 | -1.25118919 |
| Gm10700 | 0.00144 | -1.30713006 |
| D5Ertd615e | 0.00521 | -1.2836982 |
| Mir124a-1hg | 0.00144 | -1.30713006 |
| A430034D21Rik | 0.00144 | -1.31495282 |
| Lypla2 | 0.00144 | -1.03926728 |
| Gm5106 | 0.00144 | -1.54279327 |
| Gm36607 | 0.00144 | -1.30017714 |
| Lca5l | 0.43463 | -0.40446788 |
| Nccrp1 | 0.15723 | -0.48985412 |
| Gdap9 | 0.00169 | 1.30174171 |
| l7Rn6 | 0.26825 | 0.39621651 |
| Secisbp2 | 0.41886 | -0.14890223 |
| Vmn1r189 | 0.00158 | -1.2446827 |
| 0610031O16Rik | 0.01225 | -1.30950748 |
| Grxcr2 | 0.00144 | -1.28116048 |
| Slc35f5 | 0.00144 | -1.38265051 |
| Dmxl2 | 0.01292 | 0.59283862 |
| Tnfsf15 | 0.00144 | -1.23155796 |
| Hs3st3b1 | 0.72372 | -0.07467667 |
| Raet1c | 0.00144 | -1.25118919 |
| Gm11827 | 0.00935 | 1.72188075 |
| Gm32123 | 0.20901 | -0.69421527 |
| E030044B06Rik | 0.00144 | -1.30017714 |
| Saa3 | 0.00712 | -2.36985631 |
| Gm35822 | 0.97342 | 0.02634409 |
| 1700003G13Rik | 0.00294 | -1.68738961 |
| AU015791 | 0.42678 | -0.27341466 |
| Iqsec2 | 0.1605 | -1.18179336 |
| BC048559 | 0.45048 | -1.18687708 |
| Rhoj | 0.04707 | -0.51004326 |
| Nctc1 | 0.03307 | -0.57454555 |
| Rpa3 | 0.35851 | 0.31107806 |
| Hist1h2bn | 0.48608 | -0.2321223 |
| Ttc3 | 0.29056 | -0.18250825 |
| Gm4906 | 0.20647 | -1.0111686 |
| Dtx3l | 0.07142 | -0.59596076 |
| Crebbp | 0.25356 | -0.22993267 |
| Gm33766 | 0.00144 | -1.52534313 |
| Sh3d19 | 0.00145 | 1.10578442 |
| Dtna | 0.01909 | 0.64800676 |
| 2010300F17Rik | 0.00144 | -1.29085005 |
| 5730407M17Rik | 0.04487 | -0.67859746 |
| Bcr | 0.02935 | -0.77454453 |
| Usf2 | 0.02849 | -0.67958027 |
| Asna1 | 0.20743 | -0.48597893 |
| Tsnax | 0.00516 | 0.8726522 |
| Ufsp2 | 0.00144 | -1.12194768 |
| Gm15239 | 0.00144 | -1.30017714 |
| Gabpb1 | 0.60188 | 0.14415907 |
| Bcas1os2 | 0.00144 | -1.28116048 |
| Drp2 | 0.012 | -3.14050773 |
| Sec31b | 0.91473 | -0.01818833 |
| Eddm3b | 0.00144 | -1.25118919 |
| Dhrs1 | 0.86304 | -0.0755981 |
| A930014D07Rik | 0.00144 | -1.31884045 |
| Galnt13 | 0.00144 | -1.24811894 |
| Olfr33 | 0.03157 | -0.71448215 |
| Gm32743 | 0.00747 | -1.8436121 |
| 4930562C15Rik | 0.08949 | -0.8276943 |
| Lca5l | 0.02406 | -0.81861675 |
| Gm36529 | 0.04375 | -0.92735116 |
| Arv1 | 0.07396 | 0.40731564 |
| Gm12349 | 0.00556 | -1.55729019 |
| Mid1 | 0.01689 | -1.31978601 |
| Alox5ap | 0.20155 | -0.26775009 |
| A230051N06Rik | 0.00144 | -1.29085005 |
| Cyth2 | 0.03855 | -0.51990301 |
| Pars2 | 0.00265 | -1.36419466 |
| Gm17182 | 0.00144 | -1.28116048 |
| Prkca | 0.24562 | -0.2200879 |
| Jakmip2 | 0.91749 | 0.04180477 |
| Rag2 | 0.00144 | -1.25118919 |
| Gm10548 | 0.03056 | -0.81442373 |
| Gm28548 | 0.00254 | -1.1839779 |
| 1110014L15Rik | 0.08048 | -0.53946603 |
| Gm13986 | 0.00144 | -1.28116048 |
| Gm9567 | 0.01356 | -1.10879133 |
| 4930430E12Rik | 0.0049 | -1.11996532 |
| Ap4s1 | 0.3177 | 0.21800415 |
| Szrd1 | 0.01286 | -0.72916866 |
| Dock4 | 0.00575 | 0.85014507 |
| Lmo7 | 0.00436 | -0.67553702 |
| Trim34a | 0.00344 | 1.50515665 |
| Cald1 | 0.00437 | 1.33800622 |
| 1700109H08Rik | 0.80045 | 0.08365386 |
| Lrrc75a | 0.71693 | -0.15312621 |
| Dgcr2 | 0.0461 | -0.78129399 |
| Mettl21b | 0.00305 | -1.39343567 |
| Rbbp5 | 0.00311 | -0.77123459 |
| 4930405E02Rik | 0.00144 | -1.24811894 |
| Olfr859 | 0.00149 | -1.22582153 |
| Pax7 | 0.00144 | -1.28898079 |
| Olfr777 | 0.00363 | -1.23873568 |
| Vsig10l | 0.07636 | -0.41688838 |
| Sytl3 | 0.00424 | -1.1237073 |
| Etfa | 0.3705 | 0.15825744 |
| Slfn8 | 0.22204 | -0.81744158 |
| Ces5a | 0.00144 | -1.26560808 |
| Macf1 | 0.79851 | -0.04823029 |
| 4833422C13Rik | 0.15056 | 0.5374607 |
| Acsl5 | 0.0948 | -0.41709328 |
| Gm31793 | 0.43458 | 0.12653064 |
| Mamld1 | 0.40274 | -0.60988836 |
| LOC102635707 | 0.00149 | -1.2703832 |
| Dpp6 | 0.00145 | -1.25646236 |
| Gm31126 | 0.00145 | -1.22193674 |
| Smpd4 | 0.22399 | -0.41160566 |
| 4930503H13Rik | 0.00144 | -1.31495282 |
| Gm5429 | 0.00144 | -1.28898079 |
| Iglon5 | 0.00144 | -1.20555712 |
| Gm14964 | 0.05156 | 0.65800161 |
| Gm12169 | 0.00454 | -1.12501865 |
| Ccdc14 | 0.11598 | -0.9318051 |
| Tpm1 | 0.01586 | -0.56238462 |
| Olfr874 | 0.1356 | -0.91210233 |
| Qsox2 | 0.27484 | 0.29690315 |
| Dnajc6 | 0.13494 | -0.78799322 |
| Morc3 | 0.75795 | 0.05856589 |
| 4833412C05Rik | 0.0165 | -1.08638211 |
| 4930570N18Rik | 0.00961 | -1.84728799 |
| A130033P14 | 0.00596 | 0.81659244 |
| Rtca | 0.15825 | 0.46957097 |
| Gm32461 | 0.23107 | -0.42262696 |
| Crnde | 0.14298 | -0.31613078 |
| 1700025M24Rik | 0.59146 | -0.20280125 |
| Olfr484 | 0.07006 | -1.63211998 |
| Gm26891 | 0.09812 | -0.70700513 |
| Gm5155 | 0.00144 | -1.20555712 |
| Gm15319 | 0.11598 | -0.432839 |
| Gcdh | 0.25351 | 0.28656632 |
| Gm26851 | 0.00144 | -1.29085005 |
| Cacna2d4 | 0.03044 | -1.13668918 |
| Lyzl4os | 0.00144 | -1.26934956 |
| AA543401 | 0.01229 | -0.61286154 |
| Zfp61 | 0.01341 | -0.61594488 |
| Plagl1 | 0.0655 | -0.78771849 |
| 1600014C10Rik | 0.01067 | -0.93205595 |
| Gm15706 | 0.88016 | 0.02882238 |
| Gm13383 | 0.00328 | -1.10633687 |
| Plcd4 | 0.0843 | -1.40054462 |
| Uqcrfs1 | 0.68873 | -0.36573505 |
| Adnp | 0.04543 | 0.45753786 |
| Gt(ROSA)26Sor | 0.489 | -0.18486911 |
| Nfkbib | 0.7569 | 0.14754743 |
| Hao1 | 0.00158 | -1.2446827 |
| Gdf10 | 0.4072 | -0.21949718 |
| Hspb11 | 0.00144 | -1.30713006 |
| Tor1aip1 | 0.00144 | -1.26304659 |
| Fbxl18 | 0.65537 | -0.25368709 |
| Ctage5 | 0.00166 | 1.00838126 |
| Olfr1049 | 0.00144 | -1.30713006 |
| Fam57b | 0.10641 | -0.90857791 |
| Arpc1b | 0.93458 | -0.02117532 |
| Rps25 | 0.05523 | -0.50468455 |
| Lipt1 | 0.00528 | -1.78710991 |
| Gm20597 | 0.01981 | 1.50198067 |
| Ush1g | 0.04904 | -0.92735116 |
| Bche | 0.11256 | 0.28993525 |
| Pou2f3 | 0.07421 | -0.92037315 |
| Slc15a4 | 0.26647 | -0.60053712 |
| Cyp2d40 | 0.96784 | -0.04924381 |
| B430119L08Rik | 0.00447 | -1.07435095 |
| Spryd7 | 0.00192 | 1.32449896 |
| Oas1g | 0.17177 | -0.4639211 |
| Gm19519 | 0.91962 | -0.11935153 |
| Ivd | 0.66208 | 0.26067892 |
| Gprin2 | 0.00144 | -1.26560808 |
| Enpp5 | 0.02849 | 0.42802038 |
| Gm38058 | 0.00144 | -1.32000433 |
| Uevld | 0.01348 | -0.7374464 |
| Rnf19b | 0.90869 | 0.03799395 |
| Gm29675 | 0.39043 | -0.44761744 |
| Gm13889 | 0.00144 | -1.04741936 |
| Ush2a | 0.00184 | -1.19286777 |
| E030007A22Rik | 0.01909 | -1.17738282 |
| Tceal5 | 0.00496 | 0.90649266 |
| Cyp2c44 | 0.00144 | -1.25118919 |
| Tcerg1 | 0.00144 | 2.79251354 |
| Krt24 | 0.00144 | -1.27165604 |
| Morn3 | 0.00144 | -1.30713006 |
| Bpifa2 | 0.00144 | -1.29085005 |
| Gm8817 | 0.95499 | -0.03320552 |
| Msmo1 | 0.02272 | 0.78379583 |
| Tnks1bp1 | 0.13277 | -0.26696103 |
| Svil | 0.40551 | 0.23834208 |
| Dync1i2 | 0.00435 | -0.98568164 |
| Ascc2 | 0.73602 | 0.0820689 |
| Coro1c | 0.01818 | -0.75155826 |
| Slc30a8 | 0.00144 | -1.27165604 |
| Nell1 | 0.1173 | -0.74813958 |
| Trak1 | 0.00145 | -1.06915835 |
| LOC105243964 | 0.69631 | 0.13405387 |
| Mcidas | 0.01201 | -0.55088105 |
| Oog2 | 0.05278 | -1.99916438 |
| Pgr | 0.50051 | -0.21779049 |
| Gm19691 | 0.30301 | -0.81195458 |
| Kirrel3os | 0.00778 | -1.06398377 |
| Gm20187 | 0.00144 | -1.33632959 |
| Slc25a21 | 0.00144 | -1.33632959 |
| 4930448C13Rik | 0.00144 | -1.27165604 |
| Csmd1 | 0.00144 | -1.29746368 |
| Gm20635 | 0.04969 | -1.22673268 |
| Gm38811 | 0.00144 | -1.28116048 |
| 4930553E22Rik | 0.02379 | -2.18931869 |
| Lrp5 | 0.00181 | -0.89626889 |
| Grin1os | 0.00364 | -0.88262466 |
| Gm12709 | 0.00145 | -1.22376446 |
| Zfp248 | 0.00144 | 1.62191638 |
| Slc15a2 | 0.16997 | -0.84211569 |
| 4921528I07Rik | 0.00195 | -1.14271413 |
| Slc34a3 | 0.00144 | -1.31884045 |
| Abat | 0.49338 | -0.19762981 |
| 4930509E16Rik | 0.0204 | -1.08695888 |
| Gm18409 | 0.00144 | -1.28898079 |
| AF366264 | 0.16087 | -1.15882664 |
| Scrn3 | 0.83928 | 0.04198945 |
| Amy1 | 0.00159 | 2.20514803 |
| Sufu | 0.12968 | 0.50886825 |
| 4932413F04Rik | 0.00585 | -1.07062058 |
| Inpp1 | 0.52689 | 0.14489185 |
| Slc1a3 | 0.08072 | -0.63603212 |
| Pcid2 | 0.55249 | -0.15698315 |
| E230032D23Rik | 0.0416 | -1.78093264 |
| Slx4ip | 0.92998 | -0.05503674 |
| Pold3 | 0.00241 | 0.894631 |
| Tbl1x | 0.16416 | -0.32578102 |
| Trim14 | 0.00144 | -1.28898079 |
| Zfp758 | 0.00144 | 2.44768388 |
| Ciart | 0.00594 | 0.7901364 |
| Camk1g | 0.00144 | -1.28116048 |
| Rpl28 | 0.00144 | -1.02221165 |
| Uvssa | 0.08236 | -0.5447634 |
| Ccdc34os | 0.35904 | -0.24381659 |
| Gm2172 | 0.13767 | -0.8143145 |
| Myrf | 0.00144 | -1.27699683 |
| Gm35147 | 0.03337 | 0.70517251 |
| Cog6 | 0.00217 | -0.806078 |
| Ccdc186 | 0.07697 | 0.730076 |
| Gm39927 | 0.00254 | -1.22203309 |
| Ckap4 | 0.10277 | -0.76820817 |
| Gm9925 | 0.23471 | 0.25045338 |
| Cyp11a1 | 0.34971 | -0.15609317 |
| Ccni | 0.00302 | 1.17452065 |
| E230034D01Rik | 0.00144 | -1.25927895 |
| Olfr26 | 0.00144 | -1.29085005 |
| Ccdc167 | 0.00562 | -0.84359472 |
| Gm14051 | 0.00261 | 3.44901013 |
| Caskin2 | 0.09327 | -1.93180977 |
| Gm9898 | 0.00353 | -1.08965911 |
| Arap1 | 0.01547 | -0.64533082 |
| Nfyb | 0.02041 | -0.46619073 |
| Apol6 | 0.20485 | -0.69558341 |
| Myl2 | 0.0022 | -0.86852915 |
| Acpt | 0.00144 | -1.51680878 |
| Gm14167 | 0.15779 | 0.40184324 |
| Ppp1r9a | 0.02397 | 0.76436029 |
| LOC102636792 | 0.26775 | -0.51963168 |
| Vamp5 | 0.20056 | -0.46009602 |
| Tcp11l2 | 0.0579 | 1.63613021 |
| U2af1 | 0.79589 | 0.07568546 |
| Gsk3b | 0.35204 | -0.17884923 |
| Gm33104 | 0.07371 | -0.87252901 |
| Zfp874a | 0.00144 | 2.04389097 |
| Gm11725 | 0.00144 | -1.31884045 |
| Ugt3a2 | 0.00144 | -1.31884045 |
| Zfyve28 | 0.15013 | -0.64980151 |
| Gm36736 | 0.15617 | -0.94128101 |
| Gm16496 | 0.00144 | -1.29085005 |
| Rars2 | 0.00375 | 1.29830695 |
| BC001981 | 0.00145 | -1.24191372 |
| Sfxn2 | 0.01885 | 0.6947358 |
| Gm30698 | 0.06588 | -1.5745115 |
| Tas2r143 | 0.00144 | -1.26560808 |
| C630043F03Rik | 0.00145 | -1.22376446 |
| 5033426E14Rik | 0.00144 | -1.27165604 |
| Gfra2 | 0.12629 | -0.85456712 |
| Gm12002 | 0.00839 | 1.01922493 |
| Gm32026 | 0.77675 | -0.23331547 |
| Gm9903 | 0.10673 | -0.71299949 |
| 2610020H08Rik | 0.25236 | 0.33588489 |
| Lrrc7 | 0.00144 | -1.31495282 |
| Efcab13 | 0.01728 | -0.936162 |
| Zc3h7a | 0.198 | 0.40328855 |
| Gm40556 | 0.00508 | -1.30078065 |
| Rsf1 | 0.04321 | 0.98613 |
| Gm16618 | 0.00149 | -1.21097148 |
| Gm30692 | 0.05025 | -0.90748867 |
| 4930524B17Rik | 0.03776 | -0.69501203 |
| Gm10421 | 0.00145 | -1.2388121 |
| Rsrc1 | 0.12683 | -0.33413727 |
| Olfr749 | 0.00144 | -1.30713006 |
| Gata5os | 0.00144 | -1.37898565 |
| Ubb | 0.00589 | -0.7526655 |
| Anks3 | 0.04465 | -0.43004959 |
| Slc25a22 | 0.56592 | -0.15537641 |
| Rrh | 0.00144 | -1.29051721 |
| Olfr198 | 0.03023 | -0.79513267 |
| LOC102636795 | 0.00144 | -1.28116048 |
| Sptbn5 | 0.17126 | -0.32915072 |
| Gm15915 | 0.00144 | -1.31495282 |
| 9430019J16Rik | 0.00144 | -1.37434134 |
| Gm12703 | 0.00149 | -1.22582153 |
| Tmem216 | 0.00145 | -1.54550932 |
| Ppp1r8 | 0.50747 | -0.34448601 |
| Zdhhc22 | 0.0391 | -3.46908925 |
| Ribc2 | 0.95995 | -0.01748914 |
| Mogat1 | 0.36068 | -0.62482304 |
| Gm32939 | 0.34349 | -0.77212997 |
| Wap | 0.34344 | -0.44101621 |
| Gif | 0.04617 | -0.37626784 |
| Gm34883 | 0.90849 | 0.07972922 |
| 9130015A21Rik | 0.00987 | -1.86696801 |
| Gm30500 | 0.13589 | 0.43783811 |
| Lsm14a | 0.00144 | 1.21296334 |
| Enpep | 0.03003 | -1.70916848 |
| Lcor | 0.13739 | -0.248418 |
| Fndc3b | 0.31846 | 0.19049739 |
| Gm3646 | 0.00815 | -0.77356681 |
| E530001K10Rik | 0.06291 | -0.79809298 |
| AU015836 | 0.00144 | -1.49245118 |
| Sipa1l3 | 0.02059 | -1.02926879 |
| Gm12592 | 0.82201 | 0.11436603 |
| 4933430I17Rik | 0.00144 | -1.27165604 |
| 5330432E05Rik | 0.03556 | -0.99595333 |
| Pnrc1 | 0.01251 | -0.82908598 |
| Anp32e | 0.00163 | 1.17937593 |
| LOC105244220 | 0.04481 | -0.37201648 |
| Gm1987 | 0.00659 | -0.85714809 |
| Zc4h2 | 0.11598 | 0.78796646 |
| 4930590A17Rik | 0.43331 | -0.56586379 |
| F630028O10Rik | 0.02514 | 1.15070326 |
| 2610027K06Rik | 0.06113 | -0.55069223 |
| Slc39a8 | 0.00144 | -1.30713006 |
| Gm13748 | 0.43547 | -0.15256229 |
| Pitpnc1 | 0.01556 | -0.81131273 |
| Gm31087 | 0.59922 | 0.37458776 |
| Elmo1 | 0.04616 | 0.72264766 |
| 1810019D21Rik | 0.10499 | 0.35523229 |
| Gm35177 | 0.48431 | -0.19541371 |
| Kcng4 | 0.10571 | -2.73600515 |
| Gm21286 | 0.03118 | -1.32659559 |
| Gm17501 | 0.01702 | -1.00335122 |
| B4galnt3 | 0.41679 | -0.3051706 |
| Nek10 | 0.00178 | -1.32898749 |
| Map3k10 | 0.00238 | -0.99408863 |
| Gm9376 | 0.10037 | -2.45648178 |
| Ctsw | 0.17889 | -0.59416161 |
| Mest | 0.00749 | -1.05513651 |
| H2-Ke6 | 0.14258 | -0.46977001 |
| Zfr | 0.00417 | 0.80905769 |
| Atpif1 | 0.15043 | -0.32425688 |
| Gm3289 | 0.03242 | -0.67368721 |
| Gm34653 | 0.01226 | -1.35568261 |
| Trib1 | 0.09199 | -0.3328124 |
| Gm31169 | 0.05097 | -2.97295441 |
| AA387883 | 0.16878 | -0.72011048 |
| Drg2 | 0.71113 | -0.1294186 |
| 4930568B11Rik | 0.01356 | -1.10879133 |
| Lrwd1 | 0.5285 | -0.12320941 |
| Lats1 | 0.50043 | -0.57016446 |
| AU019823 | 0.01468 | 0.7691731 |
| Vmn2r26 | 0.00145 | -1.2388121 |
| Fbxo18 | 0.29885 | 0.68396879 |
| Caln1 | 0.14206 | -3.49256023 |
| Arsb | 0.777 | -0.07294243 |
| Telo2 | 0.01184 | -0.60303242 |
| Bcl11b | 0.00152 | -1.37678442 |
| Eri3 | 0.87246 | -0.0778506 |
| Aldh8a1 | 0.18018 | 0.82188513 |
| E130018N17Rik | 0.14486 | -0.71424672 |
| Tmc8 | 0.03024 | -0.54520191 |
| Gm9888 | 0.06155 | -0.57102826 |
| Pdhb | 0.09672 | 0.5139294 |
| Bahd1 | 0.05521 | -0.80949473 |
| Zan | 0.23764 | -0.29906661 |
| N4bp3 | 0.02128 | -0.61647496 |
| 4930571N24Rik | 0.00144 | -1.31495282 |
| Plch1 | 0.00144 | -1.2856619 |
| Ttc7 | 0.05141 | -2.46386554 |
| 1700106O07Rik | 0.00144 | -1.28116048 |
| Gm33460 | 0.63393 | -0.51563149 |
| Phactr2 | 0.00145 | -1.2388121 |
| Slc2a9 | 0.00144 | -1.6917903 |
| Ssr2 | 0.01648 | -0.72442579 |
| Gm15939 | 0.04617 | -1.58350904 |
| Stk25 | 0.65502 | -0.10631189 |
| Gm6521 | 0.00386 | -1.70999226 |
| Slc25a30 | 0.00144 | -1.28116048 |
| Il22ra1 | 0.02551 | -0.97509087 |
| Gpr85 | 0.00144 | -1.28116048 |
| Pla2g10os | 0.38428 | 0.28749883 |
| Irf2bp2 | 0.00144 | -1.21252367 |
| 2810001A02Rik | 0.00246 | -0.94813098 |
| Gm33050 | 0.46925 | 0.38727803 |
| Gm15234 | 0.00145 | -1.22376446 |
| A230087F16Rik | 0.34477 | -0.32687823 |
| Gm38549 | 0.01843 | -1.71733436 |
| Hist1h2bb | 0.81707 | 0.09557093 |
| LOC433354 | 0.0034 | -1.42584466 |
| Foxl1 | 0.02011 | -0.90876885 |
| Lbhd1 | 0.11035 | -0.42483598 |
| Gm32051 | 0.07307 | -0.61790352 |
| Aurkb | 0.08841 | -0.71600373 |
| Lce6a | 0.186 | -0.28458958 |
| 3632451O06Rik | 0.30336 | -0.45881977 |
| Olfr1418 | 0.0049 | -1.08617297 |
| 2510003B16Rik | 0.03809 | -0.40806518 |
| Gm15764 | 0.00365 | -0.91137085 |
| Gm19345 | 0.11916 | -0.54119378 |
| Gpr21 | 0.13103 | -0.706907 |
| Cftr | 0.11686 | -0.48871372 |
| Gm31747 | 0.00144 | -1.32346451 |
| Depdc1b | 0.00144 | -1.27165604 |
| Nr4a3 | 0.05279 | -0.92405197 |
| Acot9 | 0.12327 | 0.28741061 |
| Amer2 | 0.04193 | -0.73901607 |
| F630040K05Rik | 0.38677 | -0.15011962 |
| B230214G05Rik | 0.04674 | -2.0328236 |
| Rasgrp4 | 0.0603 | -0.78122923 |
| Clec9a | 0.68237 | -0.25162917 |
| Arl4aos | 0.00144 | -1.28898079 |
| Crygb | 0.00145 | -1.24191372 |
| Gm35161 | 0.40099 | -1.13063592 |
| Zfp639 | 0.03067 | 0.54407077 |
| Olfr1424 | 0.00144 | -1.25118919 |
| Rbfox3 | 0.00158 | -1.2446827 |
| Slc38a1 | 0.78925 | -0.21128134 |
| Hrsp12 | 0.0015 | 1.62710407 |
| Fnbp1l | 0.00235 | 2.89973958 |
| Rcvrn | 0.01444 | -0.79995826 |
| Fez1 | 0.1232 | -0.58614062 |
| 4931420L22Rik | 0.85207 | 0.0891601 |
| Hibadh | 0.13207 | 0.36191557 |
| Ddx31 | 0.01107 | -1.17472831 |
| Tmem55a | 0.03944 | 0.47815949 |
| Thra | 0.02489 | -0.54681882 |
| Olfr726 | 0.0222 | -1.05814862 |
| Pald1 | 0.03712 | -0.51260669 |
| B230340J04Rik | 0.25202 | 0.64584035 |
| 1700001J11Rik | 0.15597 | -0.54444614 |
| Gm15455 | 0.0249 | -0.45575217 |
| Gm20751 | 0.00144 | -1.08140413 |
| Fto | 0.31995 | -0.19956784 |
| Pard3bos2 | 0.01356 | -1.10879133 |
| Gab2 | 0.48898 | -0.11528264 |
| LOC102635502 | 0.3281 | -0.98267205 |
| Trim71 | 0.00144 | -1.18933657 |
| Col6a5 | 0.08402 | -0.8823614 |
| Gm12802 | 0.02761 | -2.09577112 |
| Stk-ps2 | 0.00144 | -1.26934956 |
| Tiam2 | 0.12013 | 0.28560752 |
| Mgat4a | 0.00286 | 1.15904833 |
| Lpcat4 | 0.01014 | -0.81034078 |
| Col4a4 | 0.79257 | -0.06212147 |
| Gm9984 | 0.06242 | -2.07492584 |
| Fopnl | 0.34005 | 0.44583323 |
| Ccdc28b | 0.00168 | -0.95484113 |
| Mthfsd | 0.98768 | 0.00329407 |
| Bsph1 | 0.04702 | -1.21169009 |
| Spop | 0.00229 | 0.85336229 |
| Nfix | 0.00144 | -1.49413523 |
| Gm20557 | 0.00144 | -1.30713006 |
| Gnpat | 0.40821 | 0.21738909 |
| 2700089I24Rik | 0.00284 | -1.43386937 |
| Myh15 | 0.00144 | -1.32647189 |
| Gm31066 | 0.00144 | -1.30713006 |
| 2700012I20Rik | 0.00825 | -0.97250801 |
| Nfatc2ip | 0.00144 | -2.07877077 |
| Olfr951 | 0.00637 | -1.02877696 |
| Plxnb3 | 0.23477 | -0.36224213 |
| Gm17746 | 0.00505 | -1.15422657 |
| 1700016J18Rik | 0.21619 | -0.24272217 |
| Olfr1002 | 0.04758 | -2.19897838 |
| 4931403E22Rik | 0.2925 | -0.46362515 |
| Btrc | 0.25658 | -0.20154762 |
| Apcdd1 | 0.04062 | -0.53278917 |
| Kcnq1 | 0.56173 | -0.16159558 |
| Ube2z | 0.00961 | -0.70918139 |
| Mlxip | 0.74579 | 0.09328369 |
| Trdmt1 | 0.22302 | 0.32660786 |
| Gm13497 | 0.00144 | -1.28898079 |
| Fendrr | 0.00144 | -1.20555712 |
| Krt72 | 0.02451 | -0.7826411 |
| Nat10 | 0.00572 | -0.88044367 |
| Olfr356 | 0.00144 | -1.29878664 |
| Gm38407 | 0.13528 | -0.78951426 |
| Ufl1 | 0.00144 | 1.20517227 |
| C8a | 0.07497 | -2.59049649 |
| Gm2085 | 0.00144 | -1.35112763 |
| Gm26705 | 0.05434 | -0.40560496 |
| 4930432O09Rik | 0.00358 | -1.62949723 |
| Meis2 | 0.23591 | -0.27838495 |
| Pou4f1 | 0.0049 | -1.08617297 |
| A630050E04Rik | 0.00144 | -1.30713006 |
| Casz1 | 0.21837 | -0.48365344 |
| 4930555A03Rik | 0.57757 | -0.241398 |
| Wbp2 | 0.10337 | -0.29873837 |
| Diras1 | 0.6505 | -0.35391341 |
| Gm38715 | 0.31436 | -0.6417141 |
| Gm15927 | 0.57305 | -0.25567206 |
| Cops4 | 0.21776 | -0.23075321 |
| Vmn1r191 | 0.03554 | -1.9926579 |
| Gm36757 | 0.80515 | 0.16418229 |
| Calb1 | 0.99683 | -0.00531385 |
| Gm5099 | 0.01176 | -1.65502389 |
| 5430421F17Rik | 0.00144 | -1.28898079 |
| Gyg | 0.09762 | 0.60897271 |
| Gpt | 0.25647 | -0.3386175 |
| 9430078K24Rik | 0.00424 | -1.04304679 |
| Ndfip2 | 0.79868 | -0.08176703 |
| Gm41011 | 0.04442 | -1.15565948 |
| 1700119I11Rik | 0.06076 | -0.47735173 |
| Irs2 | 0.00332 | -2.00598323 |
| Agpat5 | 0.47188 | 0.69139364 |
| Hacl1 | 0.90804 | 0.03738091 |
| Nemf | 0.00144 | 1.60711245 |
| Hmga1 | 0.38118 | -0.23875308 |
| Tex35 | 0.01198 | -1.4367066 |
| 1700092K14Rik | 0.00236 | -1.18056199 |
| Tollip | 0.00144 | -1.31884045 |
| Ano3 | 0.00144 | -1.27165604 |
| Nrf1 | 0.26599 | 0.26577887 |
| Gm648 | 0.00144 | -1.29878664 |
| Gm2449 | 0.92827 | 0.03921224 |
| Sil1 | 0.03533 | -2.90435578 |
| 4930430F21Rik | 0.00144 | -1.29085005 |
| Dio3os | 0.00659 | -0.80064417 |
| 4921511I17Rik | 0.51879 | -0.3227667 |
| 4930565D16Rik | 0.01709 | -0.99496437 |
| Gmip | 0.22034 | 0.21641551 |
| Stat2 | 0.10451 | -1.72587456 |
| A330102I10Rik | 0.00705 | -1.37898565 |
| 1700029J03Rik | 0.29214 | -0.53751611 |
| 1810073J14Rik | 0.55393 | -0.26934956 |
| LOC102636309 | 0.76493 | -0.1280166 |
| Gdap6 | 0.00144 | -1.27165604 |
| Gm9866 | 0.00144 | -1.31884045 |
| Tsnax | 0.05479 | -0.50880069 |
| Dlgap2 | 0.00483 | -0.72768206 |
| Pkmyt1 | 0.04604 | -0.42346099 |
| Vmn2r78 | 0.00145 | -1.22376446 |
| Cep68 | 0.00672 | -0.79852884 |
| Syce1l | 0.74117 | -0.08085913 |
| Myrfl | 0.502 | -0.86722607 |
| Zfp1 | 0.03601 | -0.47981642 |
| Tbc1d13 | 0.00145 | -0.88801534 |
| 1700072I22Rik | 0.00455 | -1.16728778 |
| Gm4123 | 0.03567 | -0.70989586 |
| Kalrn | 0.92272 | 0.0505822 |
| Th | 0.00145 | -1.2388121 |
| D130037M23Rik | 0.00144 | -1.30713006 |
| A630001O12Rik | 0.06177 | -2.08632294 |
| St7l | 0.26323 | -0.18640993 |
| Otos | 0.9907 | 0.01038347 |
| Akap5 | 0.00144 | 1.90995368 |
| Gm9706 | 0.04813 | -0.55564591 |
| Lce1k | 0.15976 | -0.85512282 |
| Krt6b | 0.91798 | -0.05380203 |
| Urah | 0.00758 | -1.09996726 |
| Lce3b | 0.00289 | -1.69211316 |
| Ptgs2os | 0.02396 | -1.36353418 |
| Ormdl3 | 0.23684 | -0.38856993 |
| Saxo2 | 0.00144 | -1.26560808 |
| Gm15527 | 0.00144 | -1.31884045 |
| AB124611 | 0.08361 | -0.90959635 |
| Gm15997 | 0.00144 | -1.28116048 |
| Cyp2u1 | 0.00144 | -0.98481078 |
| 4732490B19Rik | 0.47582 | -0.33587106 |
| Recql | 0.24437 | 0.28955798 |
| Cd38 | 0.15781 | 0.40546006 |
| Frem1 | 0.00144 | -1.28898079 |
| Pdcd10 | 0.00144 | 1.55307087 |
| Cd46 | 0.00446 | 1.07594612 |
| Ptpn11 | 0.00144 | -1.25693264 |
| E030042O20Rik | 0.0909 | -1.2841476 |
| Sfi1 | 0.08097 | -0.85903142 |
| Reep5 | 0.4011 | 0.15652287 |
| Gm10748 | 0.00144 | -1.36555301 |
| Igfbp5 | 0.48967 | 0.22016203 |
| Efemp2 | 0.00173 | -1.1086313 |
| Dhx57 | 0.2195 | 0.46518672 |
| Gm867 | 0.01348 | -1.33072097 |
| Gm9924 | 0.00366 | -1.12376995 |
| Gm32947 | 0.00144 | -1.33978133 |
| Vmn1r201 | 0.22396 | -1.22656039 |
| Col9a3 | 0.00144 | -1.29049509 |
| Zfp433 | 0.30108 | 0.22841681 |
| Gm13375 | 0.17012 | -0.50428326 |
| 5430437J10Rik | 0.29444 | -0.66795078 |
| 4933406M09Rik | 0.33633 | -0.52954918 |
| Qars | 0.00471 | -0.81874917 |
| Krtap5-5 | 0.34398 | -0.16389715 |
| Itga9 | 0.19051 | -0.24287461 |
| Spaca6 | 0.00871 | 1.41358411 |
| Afmid | 0.31581 | -0.46479814 |
| Zfp27 | 0.04051 | 0.37817192 |
| Gm11730 | 0.09487 | -0.42628277 |
| Asphd1 | 0.01328 | -0.86264761 |
| Lamb1 | 0.03573 | 0.40368114 |
| Gm32817 | 0.11894 | 0.74689168 |
| Armc4 | 0.00144 | -1.26934956 |
| Gm2176 | 0.00413 | -1.23116232 |
| Kdm2a | 0.12112 | -0.53092349 |
| Gm30189 | 0.98915 | 0.00678015 |
| LOC102634429 | 0.00144 | -1.34887284 |
| Gm10304 | 0.00495 | -1.01777749 |
| Fmr1 | 0.03057 | 0.83129448 |
| Gm41177 | 0.0015 | -1.15488942 |
| Abcc9 | 0.35392 | -0.34857253 |
| Lin52 | 0.0164 | -0.99673439 |
| 6530401F13Rik | 0.00144 | -1.27165604 |
| AW822252 | 0.2268 | -0.56477166 |
| Prox1 | 0.00441 | -0.76099748 |
| Zfp142 | 0.01303 | -0.61378391 |
| Slc18a1 | 0.38479 | -0.44447458 |
| Fbxl3 | 0.00144 | 2.22029 |
| Col1a1 | 0.01418 | -0.72085655 |
| Gm10419 | 0.01163 | 1.48298756 |
| Acad9 | 0.89143 | -0.03604537 |
| LOC100503594 | 0.03869 | -0.92436403 |
| Etnk2 | 0.00165 | -1.69861935 |
| Haghl | 0.01669 | -0.49132972 |
| Gm31641 | 0.16902 | -0.81032635 |
| 1110025L11Rik | 0.00144 | -1.29746368 |
| 2310026L22Rik | 0.30145 | -0.45715854 |
| Ogfod3 | 0.03425 | -0.63780168 |
| 1700013N06Rik | 0.00144 | -1.45866826 |
| Gm6297 | 0.51709 | -0.18249593 |
| Ly6k | 0.59401 | -0.31310402 |
| Acat3 | 0.09343 | 0.77703289 |
| Gm11190 | 0.06628 | -0.95678824 |
| A530001N23Rik | 0.0363 | 0.94190028 |
| Gm7694 | 0.2559 | -0.39611426 |
| Cldn34a | 0.02739 | -0.77697229 |
| Hydin | 0.02481 | -0.97331558 |
| Pcdhb6 | 0.06085 | -0.84459662 |
| F830016B08Rik | 0.00144 | 2.67033477 |
| Armc2 | 0.15722 | -0.4367922 |
| BC059841 | 0.03421 | -0.58846164 |
| 4933402C06Rik | 0.0018 | -1.21153109 |
| Gm5533 | 0.14056 | -0.53478176 |
| Vmn2r-ps54 | 0.00144 | -1.31884045 |
| Mmab | 0.17118 | -0.26812897 |
| Gm3367 | 0.05087 | -0.95844191 |
| Gpn2 | 0.0266 | -0.58751851 |
| Gm5817 | 0.09336 | -0.28362842 |
| Chmp3 | 0.02706 | -0.6040958 |
| Gm30881 | 0.00144 | -1.35320495 |
| Gm28644 | 0.02739 | -3.49310291 |
| A230001M10Rik | 0.03398 | -1.09815604 |
| Zfp595 | 0.01095 | 1.140952 |
| Rcc1 | 0.00222 | -1.1481805 |
| Gm5785 | 0.01553 | 0.89379468 |
| Dppa4 | 0.00144 | -1.27165604 |
| BC003965 | 0.63349 | -0.1525748 |
| Gltscr1 | 0.01606 | -0.74917098 |
| Hist1h2bp | 0.05733 | -0.98051773 |
| AY512915 | 0.01748 | -2.0433256 |
| 1810059C17Rik | 0.10163 | -0.64654933 |
| Tnrc18 | 0.00144 | -1.54776076 |
| Brd7 | 0.35832 | 0.34943805 |
| Ttc37 | 0.53563 | 0.12290501 |
| Psg29 | 0.00144 | -1.26514459 |
| Rnpepl1 | 0.17761 | -0.53747379 |
| Sapcd2 | 0.2451 | -0.62338404 |
| 4933409F18Rik | 0.00144 | -1.27165604 |
| 8030442B05Rik | 0.00144 | -1.27165604 |
| A130030D18Rik | 0.00144 | -1.29085005 |
| Tti2 | 0.00683 | 0.84988479 |
| Vmn2r98 | 0.22839 | -0.73942934 |
| Bcs1l | 0.8914 | -0.082655 |
| Tssk4 | 0.0857 | -0.7604986 |
| Gm31080 | 0.28017 | -0.56403712 |
| Cep250 | 0.60406 | -0.20552273 |
| Slc22a30 | 0.00144 | -1.30713006 |
| Dcdc2b | 0.37346 | -1.12366019 |
| 6430590A10Rik | 0.00144 | -1.30713006 |
| Akr1e1 | 0.11617 | 0.43831173 |
| Gm11571 | 0.11807 | -0.80610421 |
| Eqtn | 0.04302 | -1.25963959 |
| Tmem240 | 0.00144 | -1.08395915 |
| Gm1527 | 0.00144 | -1.28898079 |
| LOC102642338 | 0.00802 | 1.58662891 |
| Tmem256 | 0.00933 | -0.89526355 |
| Gm24105 | 0.02914 | -1.40041095 |
| Gm40438 | 0.00144 | -1.28116048 |
| Pdcd2l | 0.27501 | -0.38708217 |
| Nudt18 | 0.18803 | -0.58456153 |
| Col26a1 | 0.00442 | -0.87752457 |
| Npat | 0.00144 | 1.30013134 |
| Vps29 | 0.53127 | 0.14459247 |
| Gm16070 | 0.82715 | -0.08071117 |
| Kpna7 | 0.00215 | -1.23937386 |
| Gm20420 | 0.46243 | -0.3661487 |
| B230323A14Rik | 0.00144 | -1.2383131 |
| Gm26670 | 0.07358 | -0.66114203 |
| Mtrr | 0.0611 | 0.60361508 |
| Hist1h2ao | 0.00704 | -1.28670855 |
| Gm2990 | 0.00144 | -1.26560808 |
| 4833432E10Rik | 0.06827 | -0.31838284 |
| Etv1 | 0.00149 | -1.2703832 |
| D230030E09Rik | 0.0998 | 0.91587044 |
| 4930425L21Rik | 0.0111 | -1.02598223 |
| Gm5468 | 0.11242 | -1.96493925 |
| Ppp6r2 | 0.00144 | -1.075612 |
| Kansl2 | 0.03924 | 0.42649325 |
| Katnbl1 | 0.00671 | 0.81488681 |
| 1700065L07Rik | 0.27614 | -0.58750023 |
| Sstr1 | 0.01801 | -0.96799104 |
| Bach2os | 0.35676 | -0.278778 |
| Gm15698 | 0.00144 | -1.25118919 |
| Cd3eap | 0.18163 | -0.2512889 |
| C530044C16Rik | 0.95133 | -0.03221368 |
| Lig1 | 0.00521 | -0.96692382 |
| 4930438E09Rik | 0.023 | -0.61801476 |
| Psmc1 | 0.08712 | -0.29806942 |
| Slc26a2 | 0.07383 | 0.77862736 |
| Usp46 | 0.64267 | 0.29913973 |
| 1700048O20Rik | 0.00144 | -1.26934956 |
| Krt79 | 0.01685 | -1.08368725 |
| Xpa | 0.0033 | 0.94688509 |
| Ptprt | 0.00434 | -1.0948178 |
| Disp1 | 0.00399 | -0.78087966 |
| Acss2os | 0.00144 | -1.29085005 |
| Ensa | 0.51574 | -0.17675175 |
| A230048O21Rik | 0.03064 | -0.80153379 |
| 4930599N23Rik | 0.0049 | 1.22005787 |
| Vrtn | 0.02818 | -0.92785017 |
| Alms1 | 0.00144 | -1.29085005 |
| Zfp583 | 0.68317 | 0.39488217 |
| Ccdc107 | 0.0025 | -0.84902036 |
| Bnip2 | 0.10234 | 0.43785506 |
| C230040D14 | 0.32143 | -0.49334415 |
| A530010L16Rik | 0.61477 | -0.25211929 |
| Gm31166 | 0.1481 | 0.57278134 |
| Gm10865 | 0.04789 | -0.93528843 |
| 4921504A21Rik | 0.8603 | -0.0930065 |
| Tex9 | 0.08121 | -2.56857484 |
| Akap4 | 0.00144 | -1.28898079 |
| Gm5112 | 0.11896 | -2.92121465 |
| Speer4b | 0.10309 | -1.00939623 |
| Samd12 | 0.49755 | -0.39118641 |
| 1700010I14Rik | 0.07128 | -2.4222071 |
| Olfr1095 | 0.00144 | -1.28898079 |
| Vezf1 | 0.00182 | 0.85784499 |
| 1700095K22Rik | 0.18123 | -0.64619982 |
| Gm33602 | 0.02285 | -1.0076792 |
| Wtip | 0.00286 | -0.73294063 |
| Olfr1000 | 0.00144 | -1.30713006 |
| Tmem184b | 0.0017 | -1.3003838 |
| Asb18 | 0.31276 | -0.29132147 |
| Snx6 | 0.00351 | 1.99007506 |
| 4933416E03Rik | 0.04211 | -2.02520428 |
| Gm26911 | 0.00144 | -1.28116048 |
| Gm15411 | 0.01497 | -1.35131487 |
| Entpd1 | 0.21027 | -0.74096372 |
| Esyt2 | 0.00144 | 1.27592915 |
| 1500015A07Rik | 0.09868 | -1.12701563 |
| Rit2 | 0.00144 | -1.22925777 |
| 1700016P04Rik | 0.00145 | -1.22518835 |
| B230311B06Rik | 0.00531 | -0.99494008 |
| Slc38a8 | 0.51145 | -0.3901909 |
| Trpv4 | 0.01883 | -0.54449991 |
| 1700109G15Rik | 0.00144 | -1.30713006 |
| A430060F13Rik | 0.00144 | -1.26428512 |
| BC037034 | 0.23372 | -0.39843763 |
| Olfr1471 | 0.00144 | -1.30713006 |
| Snhg12 | 0.17333 | -0.33727074 |
| 9130221F21Rik | 0.00498 | -1.10677474 |
| Brsk1 | 0.00144 | -1.62304265 |
| Paxbp1 | 0.00344 | 1.55474486 |
| Rhox1 | 0.04496 | -1.376405 |
| Olfr1029 | 0.00144 | -1.25118919 |
| LOC102641859 | 0.07393 | -1.23129896 |
| C330011F03Rik | 0.00144 | -1.28116048 |
| Pdss1 | 0.05977 | 0.46751767 |
| Gm34403 | 0.0731 | -0.8956869 |
| Gm20611 | 0.64344 | -0.33312816 |
| Psg28 | 0.01657 | -1.46878669 |
| Olfr115 | 0.00229 | -1.15758681 |
| Pdxdc1 | 0.02592 | -0.88517528 |
| Serpini1 | 0.00165 | 1.46793336 |
| Cfl2 | 0.03977 | 0.72157151 |
| Prkcz | 0.1794 | 0.34512579 |
| E130008D07Rik | 0.05395 | -1.47382126 |
| Zim3 | 0.00144 | -1.28898079 |
| Lactb | 0.01436 | -0.69582881 |
| Ocel1 | 0.01181 | -0.53958051 |
| 5330439A09Rik | 0.00144 | -1.28898079 |
| 9830132P13Rik | 0.00145 | -1.22376446 |
| Hgs | 0.36117 | 0.18711092 |
| AW011738 | 0.13137 | 0.410212 |
| 9530052C20Rik | 0.04863 | -1.06195552 |
| Sbno2 | 0.16778 | -0.26129898 |
| Wasl | 0.07847 | -0.43724328 |
| Fbxw16 | 0.12256 | -0.74482175 |
| Dmc1 | 0.01175 | -1.85388756 |
| 1810043H04Rik | 0.00144 | -1.36279271 |
| 9530026F06Rik | 0.00162 | -1.6026829 |
| Ncaph2 | 0.12945 | -0.55673872 |
| Adgrg2 | 0.29077 | 0.60023638 |
| Adamts12 | 0.00166 | -1.21062785 |
| Mtmr9 | 0.09618 | 0.36789832 |
| Gm32618 | 0.06252 | 1.0296429 |
| Kif5b | 0.00144 | 1.27637116 |
| Tsc22d1 | 0.00144 | 3.09479143 |
| Vps8 | 0.17273 | 1.01915532 |
| Gm34188 | 0.00749 | -1.05513651 |
| Gm1070 | 0.04399 | -1.29458587 |
| Fads2 | 0.01447 | -0.92689729 |
| Fam65b | 0.02742 | 1.17738403 |
| Dirc2 | 0.58655 | -0.12748466 |
| Zdbf2 | 0.00144 | -1.30713006 |
| Parpbp | 0.41825 | -0.43625891 |
| Med15 | 0.21825 | 0.4753218 |
| Zc3h7a | 0.00348 | 2.50286517 |
| Bclaf1 | 0.40265 | 0.40377548 |
| C030004N09Rik | 0.60309 | 0.14085182 |
| Nipsnap1 | 0.14574 | -0.3693699 |
| Sos1 | 0.48597 | 0.4650566 |
| Ppt1 | 0.55936 | -0.32460977 |
| Mme | 0.00187 | 1.33806154 |
| Xrcc4 | 0.00506 | 1.19994015 |
| Sdc1 | 0.00164 | -0.96111829 |
| LOC497252 | 0.20646 | 0.79040252 |
| Taf7l | 0.01326 | -1.02109997 |
| Hyou1 | 0.00396 | -1.02337145 |
| Kif3a | 0.00144 | 1.4148211 |
| Mapk1ip1l | 0.17841 | 0.56594206 |
| Alg10b | 0.02013 | 0.57088047 |
| Smarca4 | 0.33661 | -0.18169899 |
| Atp8b2 | 0.30194 | -1.07063915 |
| Elovl6 | 0.49659 | 0.42432741 |
| Gm33777 | 0.00145 | -1.25646236 |
| Fyttd1 | 0.06804 | 0.41752054 |
| Atp6v0a2 | 0.52284 | 0.2389521 |
| Zdhhc23 | 0.05743 | 1.12643144 |
| Luc7l2 | 0.1599 | 0.67281863 |
| Ncor1 | 0.06646 | 0.84294483 |
| Zfp710 | 0.06728 | -0.68036045 |
| Telo2 | 0.01193 | -0.77259928 |
| Tmem92 | 0.00144 | -1.27165604 |
| Adamts4 | 0.1586 | -0.78879726 |
| Vdr | 0.49059 | -0.30120515 |
| Ttr | 0.50534 | 0.20237953 |
| Tmtc3 | 0.04158 | 1.05116572 |
| Asb7 | 0.03227 | -0.4387999 |
| Klf9 | 0.00456 | 0.77001196 |
| Cdc27 | 0.01686 | 0.73160737 |
| Abce1 | 0.00144 | 2.52609483 |
| Ttc3 | 0.02383 | 1.39298522 |
| Gm39922 | 0.00144 | -1.30017714 |
| Atp11b | 0.18207 | 0.60073949 |
| Ssu72 | 0.27115 | -0.25437948 |
| Tomm22 | 0.77909 | -0.12718152 |
| Mapk8ip2 | 0.0357 | -0.46030975 |
| Senp6 | 0.06457 | 0.48417293 |
| 6030446J10Rik | 0.01627 | 1.12033706 |
| Rfc4 | 0.83832 | 0.04610287 |
| Meis1 | 0.79855 | -0.13040383 |
| Tusc3 | 0.0281 | -0.95564746 |
| Rock2 | 0.16903 | 0.27464568 |
| Gm19818 | 0.00144 | -1.37898565 |
| Sh3bp4 | 0.13365 | -0.27762179 |
| Snhg4 | 0.02386 | 0.62894701 |
| H2-D1 | 0.00244 | -1.02794837 |
| Tlk2 | 0.00413 | 1.17072518 |
| Nsun3 | 0.00408 | -1.39360444 |
| Soga1 | 0.04177 | -0.5651315 |
| Alg10b | 0.85348 | -0.03432756 |
| Rpap1 | 0.29398 | -0.44768475 |
| Ap2m1 | 0.01211 | -0.62190196 |
| Gnb2 | 0.00875 | -0.79407486 |
| Hdlbp | 0.65881 | 0.24079889 |
| Pds5a | 0.00281 | 0.98976887 |
| Zfp382 | 0.00364 | 1.82681332 |
| Nln | 0.83672 | -0.03484852 |
| Dgkh | 0.00144 | 2.38882022 |
| Synj2 | 0.00947 | 1.0375642 |
| Zfp397 | 0.00144 | 1.96433726 |
| Pex2 | 0.00145 | 1.9061345 |
| Cd99l2 | 0.00592 | -0.7303535 |
| Far2 | 0.00279 | -1.51563149 |
| Gm3764 | 0.00144 | -1.25118919 |
| Palb2 | 0.67977 | -0.18167877 |
| Btbd18 | 0.00144 | -1.28116048 |
| Nsun4 | 0.02299 | 0.81581952 |
| 4931406P16Rik | 0.02395 | 0.79764985 |
| Ankrd66 | 0.05102 | -0.60853683 |
| Ppm1h | 0.88695 | -0.02910624 |
| Nbeal1 | 0.00547 | 1.54309445 |
| Grin1 | 0.00319 | -1.44399392 |
| Mtmr9 | 0.00276 | 0.76574766 |
| Ptch1 | 0.93446 | -0.03195502 |
| Btf3 | 0.63995 | 0.13928124 |
| Gm4961 | 0.01634 | -1.64429527 |
| Snhg7 | 0.23531 | 0.53444846 |
| Cep295 | 0.01034 | 1.14355222 |
| Scarb2 | 0.00216 | 0.8832097 |
| Pnisr | 0.00705 | 2.22205267 |
| Plag1 | 0.15259 | 0.78360005 |
| Usp32 | 0.25914 | -0.29804119 |
| Zfp281 | 0.00192 | 0.85957957 |
| Tardbp | 0.00268 | 2.69891255 |
| Ranbp1 | 0.11902 | -0.32630109 |
| Nrp1 | 0.00616 | 1.13820787 |
| Ccnd2 | 0.97528 | -0.00984033 |
| Cep112 | 0.00612 | 0.8655697 |
| Nrp1 | 0.01322 | 1.16307172 |
| Ankrd28 | 0.06502 | 0.49899304 |
| Tbc1d10b | 0.35159 | -0.22833613 |
| Tbc1d31 | 0.58482 | 0.45226885 |
| Zfp719 | 0.00144 | 2.08183568 |
| Itsn1 | 0.00655 | -1.24924848 |
| Il31ra | 0.00215 | -1.16778291 |
| Jmjd6 | 0.10694 | -0.63741021 |
| Gm16513 | 0.00144 | -1.25118919 |
| Runx2 | 0.8741 | 0.24271031 |
| Hnrnpul2 | 0.01869 | 0.76656814 |
| Gspt1 | 0.00218 | 0.83522161 |
| Simc1 | 0.14731 | 0.61924634 |
| E430010N07Rik | 0.02771 | 1.017089 |
| Yme1l1 | 0.00144 | 1.42058609 |
| Mbd1 | 0.00166 | 0.85426761 |
| Kdm2a | 0.03761 | 1.7182727 |
| Rpf1 | 0.00192 | 1.5807533 |
| Cenpl | 0.09162 | 0.56512975 |
| Gpc6 | 0.05191 | 0.7587861 |
| Dnajc13 | 0.00516 | 1.08413283 |
| Sik2 | 0.023 | 0.56144512 |
| Imp4 | 0.00397 | 0.71667441 |
| Herc3 | 0.00433 | -0.83634971 |
| Ercc4 | 0.33437 | 0.27653005 |
| Nxn | 0.24765 | -0.33902786 |
| Gm41538 | 0.00144 | -1.27992547 |
| Phkb | 0.00349 | 1.17322165 |
| Fuca2 | 0.00144 | 1.16319503 |
| Apobec3 | 0.02865 | -0.44623554 |
| Vgll1 | 0.00144 | -1.31769968 |
| Dock8 | 0.27314 | -0.71034782 |
| Map2k1 | 0.02578 | 0.73050435 |
| Shc1 | 0.00229 | -0.84102742 |
| Mbnl1 | 0.06888 | -0.3397601 |
| Gse1 | 0.05909 | -0.99327404 |
| Tmppe | 0.20773 | -0.30264458 |
| Mvb12b | 0.55864 | -0.21197366 |
| Ate1 | 0.06534 | -0.50388682 |
| C1qtnf1 | 0.00428 | -0.81845814 |
| Pdzd2 | 0.3392 | 0.47404706 |
| Braf | 0.03891 | 1.25972322 |
| Pias1 | 0.00144 | 1.38436757 |
| Tpm1 | 0.15406 | -0.71968646 |
| Nkiras2 | 0.36721 | -0.59019144 |
| Socs6 | 0.00144 | 1.33179565 |
| Esr1 | 0.02115 | 1.2378233 |
| Igf2bp3 | 0.01808 | 1.16083799 |
| Mndal | 0.00144 | 2.25700606 |
| Hbp1 | 0.54623 | 0.37571461 |
| 4933427D06Rik | 0.00144 | -1.39629016 |
| Uhrf2 | 0.00144 | 2.01267401 |
| Slc22a15 | 0.6199 | 0.20809485 |
| Gm16845 | 0.45977 | -0.21295556 |
| LOC102643228 | 0.3498 | 0.40825497 |
| Eps8 | 0.16863 | -0.71249495 |
| Pdzd2 | 0.61661 | -0.13691106 |
| Ascc2 | 0.32164 | 0.1744054 |
| Smim20 | 0.01909 | -0.81172844 |
| Prelp | 0.00144 | -1.22696574 |
| Stam2 | 0.00144 | -1.08521282 |
| Gtf3c1 | 0.23522 | -0.3602793 |
| Nova1 | 0.00144 | -1.25118919 |
| Taok2 | 0.3797 | -0.19295475 |
| B4galt5 | 0.20194 | -0.3061032 |
| Kpna1 | 0.01291 | -0.58103089 |
| E130314K07Rik | 0.56492 | 0.29654473 |
| Mcmbp | 0.15549 | -0.39409171 |
| Satb1 | 0.00798 | 0.71543294 |
| Zmiz1 | 0.31698 | 0.17458486 |
| Btaf1 | 0.01176 | -1.21636428 |
| Hdac3 | 0.30803 | -0.32213083 |
| Gm35497 | 0.30166 | 0.24219665 |
| B330018G23Rik | 0.11931 | 1.2403832 |
| Nol8 | 0.38597 | 0.32187786 |
| Cmtr1 | 0.0104 | -0.64361433 |
| LOC102642348 | 0.87902 | -0.2747763 |
| Fam117a | 0.21196 | -0.73586514 |
| Ttc39b | 0.06077 | 0.42808847 |
| Ms4a2 | 0.00144 | -1.54014117 |
| Smarca2 | 0.02417 | -0.96863964 |
| Gapvd1 | 0.00535 | 1.40470072 |
| Cpm | 0.00144 | 1.0287419 |
| Pml | 0.00973 | -1.04374818 |
| Junos | 0.00279 | 1.25734653 |
| Tatdn1 | 0.22636 | 0.58172127 |
| Rsf1 | 0.05888 | 1.72172002 |
| Tgfbr3 | 0.43997 | 0.12749982 |
| Cdhr4 | 0.12771 | -0.6607603 |
| Polh | 0.76333 | 0.17751512 |
| Tfap2c | 0.00188 | -1.37510274 |
| Pknox1 | 0.3728 | 0.42753947 |
| Adgre5 | 0.98797 | -0.00352763 |
| Lamb1 | 0.00165 | -1.28317511 |
| Lrrc45 | 0.15083 | 0.40503413 |
| Rmst | 0.92922 | -0.03557756 |
| Mapkapk5 | 0.85637 | -0.04719612 |
| Tulp4 | 0.01265 | 0.6799304 |
| Mut | 0.21177 | 0.23730944 |
| Ipo9 | 0.0083 | 0.82963783 |
| Phtf2 | 0.0111 | 0.61340618 |
| Pcgf3 | 0.1574 | 0.42637946 |
| Arih1 | 0.05243 | 0.88385139 |
| Mid1 | 0.53801 | 0.23929097 |
| Nt5dc1 | 0.00341 | 0.8035662 |
| Top2a | 0.20764 | -0.52203449 |
| Focad | 0.04219 | 1.18883026 |
| Arfgap1 | 0.12817 | -0.44562514 |
| Map3k2 | 0.05436 | 0.67751572 |
| Pcdh19 | 0.00327 | 1.30787292 |
| Cuedc1 | 0.01519 | 1.01834589 |
| Gldc | 0.14918 | -0.60796708 |
| Apbb2 | 0.85355 | 0.0411745 |
| Usp13 | 0.70321 | -0.0809417 |
| Fam208b | 0.18642 | 0.47559523 |
| Mrps15 | 0.04765 | -0.37001158 |
| Zfp251 | 0.03746 | -0.42213044 |
| Nptn | 0.19796 | 0.51834301 |
| Scaf8 | 0.56959 | 0.27784078 |
| B3galnt2 | 0.1209 | 0.33861501 |
| Thoc2 | 0.02557 | 1.23064346 |
| Bcl9l | 0.01357 | -0.79689707 |
| Mrpl15 | 0.5462 | 0.24761763 |
| Ralbp1 | 0.02666 | -0.53206782 |
| Myo5a | 0.89612 | -0.05172042 |
| Snx29 | 0.7746 | 0.12544938 |
| Cops4 | 0.03651 | 1.37033148 |
| Wdr92 | 0.02414 | -0.8451519 |
| Apaf1 | 0.34278 | -0.40852421 |
| 2810029C07Rik | 0.4239 | 0.25502641 |
| Safb2 | 0.21483 | 0.5258273 |
| 4930551A22Rik | 0.01091 | -0.62928948 |
| 5031439G07Rik | 0.00561 | -0.96615827 |
| Gm36480 | 0.00144 | -1.30626366 |
| Nipbl | 0.00174 | 2.478944 |
| Rrnad1 | 0.42153 | -0.2703904 |
| Alyref | 0.70635 | 0.06945486 |
| 9530059O14Rik | 0.00144 | -1.24811894 |
| Deptor | 0.52609 | 0.43496562 |
| Zfp850 | 0.94348 | -0.05896621 |
| Fam178a | 0.00145 | 1.06285946 |
| Sypl | 0.64358 | -0.08393547 |
| Sdad1 | 0.14587 | -0.29012575 |
| Pdlim7 | 0.30211 | -0.50403667 |
| Snx9 | 0.08025 | 0.34586204 |
| AU020206 | 0.32187 | 0.27351299 |
| Wdr37 | 0.03525 | 0.55779328 |
| 2310067E19Rik | 0.00208 | 3.34488584 |
| Phip | 0.01809 | 1.47476836 |
| Trpm2 | 0.00144 | -1.29746368 |
| Gm15496 | 0.00166 | -1.25694302 |
| Nek4 | 0.01177 | 0.64207087 |
| B3gntl1 | 0.03666 | 0.49075517 |
| Trim41 | 0.71933 | 0.07655653 |
| Rbm47 | 0.42925 | 0.18406852 |
| Vps39 | 0.28075 | -0.18518338 |
| Mtmr7 | 0.76135 | -0.33541966 |
| Il18rap | 0.00144 | -1.28116048 |
| Gm31863 | 0.99243 | -0.00362512 |
| Nsmaf | 0.23587 | 0.21841482 |
| Sac3d1 | 0.00347 | -1.10976892 |
| Tlk1 | 0.19833 | -0.4855938 |
| Tmem164 | 0.49268 | -0.24398922 |
| Cr1l | 0.64202 | 0.09165287 |
| Rsrc2 | 0.05876 | 0.98253479 |
| Ttc19 | 0.05689 | 0.51627859 |
| Ndufb2 | 0.00144 | -1.16475267 |
| Taldo1 | 0.51847 | -0.27387125 |
| LOC105247521 | 0.06196 | 1.24208248 |
| Pikfyve | 0.15038 | 0.85499358 |
| Myoz2 | 0.32646 | 0.34412511 |
| Parg | 0.27713 | 0.34408473 |
| Fgf10 | 0.00144 | -1.28116048 |
| Fbxo3 | 0.00144 | 1.60781081 |
| Dcp2 | 0.00144 | 1.25622722 |
| Ank3 | 0.00516 | 0.93408536 |
| Ubn2 | 0.02979 | 2.41470278 |
| Anapc16 | 0.13359 | -0.38254542 |
| Ephb2 | 0.0874 | -0.75039268 |
| Thbs1 | 0.15267 | 1.08665657 |
| Xpc | 0.00185 | -1.11189166 |
| Jup | 0.03286 | -0.61987349 |
| Ints10 | 0.10806 | 0.75590768 |
| Osbpl8 | 0.00177 | 1.17529297 |
| Cd300lb | 0.12415 | -0.72394151 |
| Actn2 | 0.00251 | -0.78653748 |
| Parp6 | 0.16161 | 0.96655529 |
| Naa20 | 0.0412 | 1.20202429 |
| C86695 | 0.00145 | -1.27532353 |
| LOC102634296 | 0.2082 | -2.21267492 |
| Fam20b | 0.00373 | -1.2220099 |
| Pxdn | 0.10472 | 0.42580064 |
| Lims1 | 0.1299 | 0.29974099 |
| Rnf13 | 0.02292 | 1.56379586 |
| Sugp1 | 0.00552 | -1.14048792 |
| Bcat1 | 0.0447 | 1.02557354 |
| Frmd4b | 0.12972 | 0.43575567 |
| Sprtn | 0.0278 | 0.44747893 |
| B930053N05Rik | 0.78006 | 0.09632378 |
| Bicd1 | 0.93549 | -0.06100606 |
| Kif1b | 0.01953 | 1.12415947 |
| Dnaja2 | 0.53106 | 0.10806916 |
| Polr2c | 0.13035 | -0.42308747 |
| Sec16a | 0.89214 | -0.04334456 |
| Mutyh | 0.25427 | 0.29346885 |
| Lmna | 0.00364 | -1.1217097 |
| A130010C12Rik | 0.66468 | -0.11227718 |
| Abcb1a | 0.0016 | 1.05910151 |
| Snrnp48 | 0.01985 | 0.86305984 |
| Slc25a13 | 0.0645 | 0.51157998 |
| Meis1 | 0.69277 | 0.21429938 |
| Adamts18 | 0.03049 | -1.64252682 |
| Dnaja1 | 0.05878 | 1.17172472 |
| Arhgef2 | 0.02621 | -0.49600293 |
| Acvr2b | 0.00317 | -0.88288573 |
| Gabbr1 | 0.60893 | 0.09352824 |
| Runx3 | 0.06532 | 0.35293014 |
| Hmgb2 | 0.00624 | 0.77626734 |
| Naa10 | 0.00273 | -0.94940136 |
| Pde4d | 0.92231 | 0.0202335 |
| Oas1c | 0.10402 | -0.67447756 |
| Gsk3b | 0.00415 | 1.28534729 |
| Timp2 | 0.02187 | -0.81791123 |
| Shmt2 | 0.96151 | -0.03281277 |
| Pin1rt1 | 0.1563 | -0.58078808 |
| Rbm39 | 0.00563 | 2.46194506 |
| Gm29999 | 0.00204 | -1.2031737 |
| Ralgps2 | 0.20067 | 0.6741226 |
| Itprip | 0.00145 | -1.16475783 |
| Gm15941 | 0.06072 | -1.48608862 |
| Srpr | 0.00144 | -1.21230763 |
| Slc25a43 | 0.62243 | -0.12808007 |
| Fut9 | 0.01776 | -1.77506774 |
| 8030451A03Rik | 0.00144 | -1.28116048 |
| Phf20l1 | 0.00144 | 2.10946855 |
| 2310039F13Rik | 0.0017 | -1.14580177 |
| Prpf4b | 0.00367 | 2.37109662 |
| Ipw | 0.09749 | -2.17765736 |
| Fbxl20 | 0.00144 | 2.29688162 |
| Nvl | 0.50918 | 0.23128599 |
| Prkx | 0.00144 | 1.34262746 |
| Uba6 | 0.05526 | 0.7855252 |
| Zkscan7 | 0.02149 | 1.39375783 |
| Mob1b | 0.00245 | 1.35845116 |
| Tmem181a | 0.0108 | 2.27097591 |
| Ctr9 | 0.02832 | 0.86778301 |
| Mef2c | 0.00237 | 1.33398628 |
| Nif3l1 | 0.34013 | 0.47769811 |
| Arhgap28 | 0.78718 | 0.11936004 |
| Ivd | 0.06104 | 0.89005433 |
| Cpsf6 | 0.01545 | 2.23268085 |
| Cnot2 | 0.14081 | -0.56788428 |
| Gm28729 | 0.00144 | -1.28898079 |
| Fam126b | 0.0029 | 0.90343665 |
| Usp14 | 0.00144 | 1.45074829 |
| Ldb3 | 0.0583 | 0.4515289 |
| Fgfr1 | 0.02295 | -0.47835308 |
| Gm32846 | 0.01105 | -1.62385054 |
| Tbc1d10a | 0.09437 | -0.34775054 |
| Prss44 | 0.00144 | -1.20555712 |
| Tenm2 | 0.01313 | -2.00857561 |
| Bmp2k | 0.00598 | 0.72967782 |
| Mtmr3 | 0.95839 | -0.01959117 |
| Phip | 0.77453 | -0.13669892 |
| Nasp | 0.00144 | 1.49119272 |
| Gm33248 | 0.01305 | -1.4362411 |
| Col6a4 | 0.00144 | -1.24811894 |
| Fam53c | 0.00144 | -1.05582173 |
| Akap11 | 0.00983 | 0.6142695 |
| Foxp2 | 0.326 | -0.50442197 |
| Gm36463 | 0.12483 | -1.0277656 |
| Letm1 | 0.5418 | -0.11167263 |
| Tanc2 | 0.24991 | 0.28290361 |
| Sytl2 | 0.00145 | -1.2388121 |
| Gcnt3 | 0.01345 | -1.60256462 |
| Eif4g3 | 0.14275 | 0.63335349 |
| Lmo7 | 0.27889 | 0.5677735 |
| Tanc2 | 0.00205 | 0.92459465 |
| Tgfb2 | 0.73106 | -0.06362074 |
| Kdm5a | 0.49946 | 0.15767085 |
| C77563 | 0.76578 | 0.38271473 |
| Arfgap1 | 0.74056 | -0.19973038 |
| Dpysl3 | 0.02211 | 0.69624695 |
| Scfd1 | 0.00144 | 2.37159166 |
| Cmtm7 | 0.18313 | -0.4226686 |
| 2610037D02Rik | 0.36798 | 0.33860674 |
| Mvp | 0.25894 | -0.33872451 |
| Ammecr1 | 0.00537 | 0.70471124 |
| Swt1 | 0.03068 | 0.86585286 |
| Wbp2 | 0.68146 | -0.22199547 |
| Tcf4 | 0.41597 | 0.39563052 |
| Tmem44 | 0.77727 | 0.06185226 |
| Erlin1 | 0.34856 | 0.33423705 |
| LOC552908 | 0.00144 | 1.93840663 |
| Wwp2 | 0.07937 | -0.61350446 |
| Cdca2 | 0.32392 | 0.72033982 |
| Kdm5a | 0.64852 | -0.07585523 |
| Raph1 | 0.20747 | 1.21811067 |
| Taok1 | 0.00474 | 1.1342 |
| Tmpo | 0.0614 | 0.62845248 |
| Rpp14 | 0.1085 | 0.30069495 |
| Lpin1 | 0.42696 | 0.29807584 |
| Srsf6 | 0.00348 | 0.93018908 |
| Atp2b1 | 0.00273 | 1.03574003 |
| Marveld1 | 0.02564 | -0.86347015 |
| Cks2 | 0.02773 | 1.36527409 |
| Gbe1 | 0.01869 | 1.00396495 |
| H60c | 0.00144 | -1.39297126 |
| Thrap3 | 0.24293 | 0.51837699 |
| Prdm2 | 0.82019 | 0.08227005 |
| Pcf11 | 0.48734 | 0.23278924 |
| Zfp106 | 0.00144 | 1.23836933 |
| Hdac1 | 0.00501 | -0.9453738 |
| Ssbp1 | 0.0871 | 0.35821072 |
| Prkdc | 0.00298 | 1.17924001 |
| Stat1 | 0.58102 | 0.21584137 |
| Nup160 | 0.86251 | -0.04764431 |
| Acsl3 | 0.54514 | -0.77954978 |
| Ccnt2 | 0.00212 | 1.80910245 |
| Zfp213 | 0.05937 | -0.58709603 |
| Zyg11b | 0.9941 | -0.00152017 |
| Znrf3 | 0.06 | -0.4215158 |
| Sox11 | 0.00144 | -1.24613586 |
| Scrn3 | 0.4325 | 0.12055951 |
| Neat1 | 0.00144 | 2.31394926 |
| Spata1 | 0.00144 | 1.49605445 |
| Obsl1 | 0.31852 | -0.29241437 |
| LOC105243035 | 0.45365 | -0.59721005 |
| Tsnax | 0.06459 | 0.70117349 |
| Thumpd3 | 0.24926 | 0.89155472 |
| Pik3ca | 0.00144 | 3.30093938 |
| Tpp2 | 0.47997 | 0.11407337 |
| 2610524H06Rik | 0.72011 | 0.0915713 |
| Oma1 | 0.73144 | -0.10853268 |
| Cbx4 | 0.01418 | -0.69372554 |
| Rreb1 | 0.01813 | -0.59479449 |
| Usp16 | 0.43054 | -0.17012082 |
| Arih2 | 0.19448 | -0.22740223 |
| Trim12a | 0.00551 | -1.3258774 |
| Rab43 | 0.00853 | -0.67126921 |
| Cdc42se2 | 0.89823 | -0.04079487 |
| LOC102631979 | 0.00262 | 2.72627472 |
| Smg1 | 0.00144 | 1.85839679 |
| Pabpc1l | 0.38064 | -0.22372407 |
| Chmp1b | 0.00144 | 1.41730902 |
| Dph5 | 0.00514 | 1.1303594 |
| Smc2 | 0.00144 | 1.87183657 |
| Zmiz1 | 0.45374 | -0.19517779 |
| Tpr | 0.11916 | 0.72190416 |
| Gapvd1 | 0.47159 | -0.13894232 |
| Fbln1 | 0.0282 | -0.87035659 |
| Cux1 | 0.01503 | 0.95887412 |
| 2810427A07Rik | 0.05897 | -0.98837767 |
| Larp4b | 0.69401 | 0.11941182 |
| Fut8 | 0.14054 | 1.15688068 |
| Adamts18 | 0.00144 | -1.28116048 |
| Zcchc7 | 0.70543 | -0.12118346 |
| Dis3 | 0.51868 | 0.36383767 |
| Herc4 | 0.15058 | 0.64141807 |
| Coq3 | 0.40219 | 0.23681324 |
| Gpr161 | 0.00144 | 1.54087674 |
| Abhd2 | 0.01921 | -0.47371619 |
| Tlk2 | 0.10545 | 0.75586327 |
| Eno1 | 0.01593 | -1.31234719 |
| Sh3kbp1 | 0.00436 | 0.68322835 |
| 6230400D17Rik | 0.21363 | -2.23045781 |
| Derl1 | 0.46382 | -0.21989779 |
| Spred2 | 0.18414 | 1.02333801 |
| Ikbkb | 0.0374 | 1.00576867 |
| Foxo3 | 0.61084 | 0.12246728 |
| Gpalpp1 | 0.13498 | 0.261687 |
| Atrx | 0.21692 | 0.60074693 |
| Patz1 | 0.09609 | -0.42133347 |
| D14Ertd426e | 0.00144 | -1.28898079 |
| Dync1h1 | 0.50604 | -0.24210659 |
| Kif22 | 0.33434 | 0.23549991 |
| Plekhm1 | 0.9821 | -0.00925512 |
| Axl | 0.55873 | 0.0951614 |
| Gm19313 | 0.2627 | -0.28942978 |
| Plod1 | 0.04701 | -0.59344105 |
| Emd | 0.00144 | -1.15618694 |
| Gm2701 | 0.01177 | 1.14472166 |
| Fip1l1 | 0.00144 | 2.06070704 |
| Elac2 | 0.2638 | 0.27215367 |
| Gm10512 | 0.00144 | -1.28116048 |
| Slc10a7 | 0.4243 | 0.48395809 |
| Dna2 | 0.15742 | -0.56107787 |
| Luc7l2 | 0.01239 | 0.73552598 |
| Klf9 | 0.00144 | 1.97630452 |
| Gm30868 | 0.92484 | -0.07375395 |
| Snrpb | 0.20295 | -0.31360842 |
| Rock1 | 0.00144 | 1.52357555 |
| Tpd52l2 | 0.85631 | -0.06162029 |
| Cage1 | 0.31137 | -0.60862721 |
| Brd8 | 0.15959 | 0.61797371 |
| Nr2c2 | 0.00341 | 1.27505698 |
| Myh7 | 0.04698 | 1.06076508 |
| Trim14 | 0.04616 | -0.44158599 |
| Cav1 | 0.62318 | 0.11642139 |
| AU017193 | 0.09346 | 0.98191059 |
| Cobll1 | 0.17394 | 0.59776651 |
| Skiv2l | 0.01087 | -0.55110537 |
| 1700034H15Rik | 0.07362 | 0.39678247 |
| Slc25a48 | 0.00144 | -1.49155079 |
| Pms2 | 0.29207 | -0.55415729 |
| Ncor1 | 0.00316 | 1.39044998 |
| Maml3 | 0.0028 | -0.81115734 |
| Ppil2 | 0.11743 | -0.53636549 |
| Trip12 | 0.93801 | -0.01475838 |
| Sap18 | 0.09595 | -0.38990114 |
| Fam21 | 0.02408 | -0.52771985 |
| Map2 | 0.75197 | -0.23161956 |
| Cbx5 | 0.01297 | -0.80187617 |
| Serf2 | 0.54652 | -0.10617906 |
| Ddx20 | 0.26827 | 0.43532885 |
| Fubp1 | 0.00431 | 2.51831399 |
| Ccdc88a | 0.00144 | 1.44737399 |
| Bmp5 | 0.00144 | 1.82398398 |
| Mcm10 | 0.04708 | -1.56472691 |
| Mbd5 | 0.04651 | 0.62069684 |
| Klf7 | 0.00144 | 1.00367048 |
| Galt | 0.3325 | -0.36314845 |
| Ddi2 | 0.00566 | 0.63446935 |
| Mid1 | 0.06689 | -0.78578232 |
| Cdkal1 | 0.49172 | -1.29260116 |
| Dync1h1 | 0.01383 | -0.66628591 |
| Msmo1 | 0.89817 | 0.05056388 |
| Vps36 | 0.46541 | -0.11146306 |
| Cir1 | 0.21113 | 0.41185393 |
| Ap2a2 | 0.02849 | -0.43429579 |
| Wtap | 0.0133 | 0.87071699 |
| Gm3515 | 0.00144 | -1.22098834 |
| Gm32743 | 0.00241 | -1.47167917 |
| Mipol1 | 0.00144 | -1.28898079 |
| Tfrc | 0.00852 | -1.63579706 |
| Ints4 | 0.01106 | 0.9003896 |
| Zglp1 | 0.12194 | -0.30180374 |
| Hat1 | 0.00238 | 1.31666445 |
| Sec22b | 0.51674 | 0.2836446 |
| Tcf7 | 0.20721 | -0.71604735 |
| Myh10 | 0.27056 | 0.53491236 |
| Mipep | 0.01765 | -1.03545621 |
| Fgfr3 | 0.16228 | -0.29782351 |
| Phip | 0.87734 | -0.13180393 |
| Sugp2 | 0.07875 | -0.39843436 |
| Smc6 | 0.02953 | 0.59418195 |
| Rpl37rt | 0.00144 | -1.10937715 |
| Fam171b | 0.10407 | 0.59795246 |
| Zfp445 | 0.00144 | 2.99349769 |
| Entpd1 | 0.21414 | 0.20987364 |
| Ctsc | 0.00145 | 1.31466975 |
| Tuba4a | 0.79133 | -0.14541989 |
| B630005N14Rik | 0.00144 | 1.53619695 |
| Srgap2 | 0.96025 | 0.0320065 |
| Cyyr1 | 0.00144 | 2.83998296 |
| Dbpht2 | 0.00144 | -1.49155079 |
| Nedd4 | 0.00144 | 2.36600334 |
| Hnrnpab | 0.19977 | 0.30558358 |
| Clasp1 | 0.01107 | -0.8768167 |
| Naa11 | 0.71007 | -0.35404057 |
| Slc39a10 | 0.14172 | 0.37062163 |
| Vezt | 0.10901 | -0.67980055 |
| Dhx36 | 0.00144 | 2.5116007 |
| Abcf1 | 0.00259 | -0.83660374 |
| Ebf3 | 0.06422 | 0.53867211 |
| Rad18 | 0.00145 | 1.51054713 |
| Gm6150 | 0.0177 | -1.99721174 |
| ND1 | 0.00536 | 0.96233085 |
| Zfp182 | 0.01189 | 1.75959639 |
| B130021K23Rik | 0.82251 | -0.06404768 |
| Arhgap15 | 0.33384 | -0.38226333 |
| Cts8-ps | 0.0095 | -1.65033555 |
| Szrd1 | 0.02076 | -1.25091474 |
| Peg3 | 0.00144 | 1.93589178 |
| Olfr288 | 0.00144 | -1.33632959 |
| Vps54 | 0.13164 | 0.49973497 |
| Iqck | 0.00144 | -1.26560808 |
| Srrm2 | 0.1935 | 0.2850187 |
| Fbxw11 | 0.01722 | 1.35273375 |
| Dtl | 0.32725 | -0.74677144 |
| Ston2 | 0.053 | 0.53959695 |
| Gm21992 | 0.04201 | -0.56491899 |
| Myo1d | 0.04111 | 1.49533832 |
| Eapp | 0.18696 | 0.55318973 |
| Edil3 | 0.00144 | -1.28375735 |
| Serpinb6a | 0.01691 | 0.72644353 |
| Pkp2 | 0.00846 | 2.16817862 |
| Cwc25 | 0.27206 | -0.27813798 |
| E030024N20Rik | 0.00149 | 1.48783324 |
| Fubp1 | 0.00144 | 4.23597732 |
| D830012I16Rik | 0.01243 | 1.91039753 |
| Ypel1 | 0.52826 | 0.16410188 |
| Larp1 | 0.15235 | -0.24572182 |
| Ppp4r2 | 0.01503 | 0.90647401 |
| Mga | 0.67461 | -0.10684306 |
| Zfp207 | 0.05888 | 1.15206794 |
| Itga6 | 0.00144 | 1.56490701 |
| Atp6v1c1 | 0.00399 | 0.72957898 |
| Man1a | 0.00158 | 1.98199745 |
| Lamc1 | 0.142 | 0.8695553 |
| Utp14a | 0.00311 | 2.40049736 |
| Ube3c | 0.00726 | 1.34141585 |
| Mpdz | 0.15197 | -0.51443929 |
| Ube3c | 0.01951 | 0.8109281 |
| P4ha1 | 0.02587 | -0.65300975 |
| Erap1 | 0.02713 | 0.55765271 |
| Xpa | 0.00155 | 1.89999532 |
| Nrxn2 | 0.00144 | -1.58198479 |
| Tvp23b | 0.01318 | -0.80097507 |
| Rufy2 | 0.92751 | 0.0220476 |
| Pfkp | 0.61584 | 0.16255261 |
| Sgtb | 0.58191 | 0.18975672 |
| Sgip1 | 0.0036 | 0.95851611 |
| Vcp | 0.04779 | -0.54666792 |
| Tnfaip1 | 0.06046 | -0.3701967 |
| E030022I16Rik | 0.05989 | 0.67753857 |
| 9330182L06Rik | 0.00879 | -1.05401892 |
| Atrx | 0.01247 | -1.12568988 |
| Lyn | 0.03303 | 1.33722661 |
| Riok1 | 0.65118 | 0.12512081 |
| Spag5 | 0.05585 | 0.46229549 |
| Noc2l | 0.2844 | -0.40142577 |
| Ccnh | 0.00796 | 1.71260628 |
| Mdn1 | 0.26044 | 0.23758548 |
| Etv1 | 0.05653 | 1.3352469 |
| Nav2 | 0.22443 | 0.5101796 |
| Ikzf4 | 0.00363 | -1.41933878 |
| Kbtbd8 | 0.00144 | -1.29746368 |
| Stx1b | 0.83154 | 0.09691839 |
| Dnajc3 | 0.0024 | 1.32593803 |
| St3gal1 | 0.03569 | -0.41148333 |
| Ubr4 | 0.00144 | -1.42043204 |
| Fuca2 | 0.07443 | 0.80845779 |
| Jmjd1c | 0.026 | 1.79608735 |
| Shroom4 | 0.00691 | 0.9547083 |
| Rpap2 | 0.0138 | 0.66554796 |
| Acot7 | 0.42328 | -0.41877267 |
| Ptbp3 | 0.00144 | 2.06654595 |
| 2210016L21Rik | 0.08728 | -0.30743018 |
| Rpl39 | 0.00884 | -0.80554655 |
| Dennd4a | 0.00144 | 3.021589 |
| Atp6v1a | 0.7722 | -0.27443216 |
| Gp49a | 0.00506 | 2.53878662 |
| St8sia2 | 0.00541 | -1.15274627 |
| Mier1 | 0.07842 | 0.6441259 |
| LOC105247524 | 0.84489 | 0.17476227 |
| Gas2l1 | 0.48475 | -0.34797217 |
| Traf3ip3 | 0.20515 | -0.52430496 |
| Mapk1 | 0.08556 | 0.40228246 |
| Rnf13 | 0.00144 | 1.61374157 |
| Vav3 | 0.98605 | 0.0310494 |
| Ltn1 | 0.8921 | 0.02647348 |
| Trim23 | 0.00144 | 2.22803513 |
| Mtdh | 0.00684 | 0.93218707 |
| Ppargc1b | 0.0044 | 1.12781104 |
| Kif1b | 0.52819 | 0.18599226 |
| Rps6ka2 | 0.0332 | 0.47922095 |
| Celsr2 | 0.00144 | -1.29746776 |
| Brd7 | 0.03059 | 0.57392041 |
| Rrnad1 | 0.04259 | 0.98356705 |
| Mrpl24 | 0.01418 | 0.94483307 |
| Rnf2 | 0.00752 | -1.16426206 |
| Stk26 | 0.6918 | -0.18158207 |
| Serinc1 | 0.00144 | 2.95601016 |
| Ccnt1 | 0.09154 | 0.34525277 |
| Uqcc1 | 0.83014 | -0.07915013 |
| 1190007I07Rik | 0.31995 | -0.34420117 |
| Eif2c5 | 0.00144 | -1.20555712 |
| Susd1 | 0.8777 | 0.04605012 |
| Zfp597 | 0.00144 | 1.08805829 |
| Pogk | 0.13291 | 0.53784143 |
| Sec22b | 0.35892 | 0.30518987 |
| Xlr | 0.00636 | 2.09302638 |
| Gstm4 | 0.68555 | -0.16051975 |
| Ldlr | 0.94508 | 0.0531594 |
| C2cd5 | 0.39589 | -0.58626844 |
| Braf | 0.04406 | 1.47480656 |
| Aen | 0.21971 | 0.20653655 |
| Zfp322a | 0.00144 | 1.20804996 |
| 6430537K16Rik | 0.07317 | 0.95196371 |
| Sptbn1 | 0.03961 | -0.66442659 |
| Rtn4 | 0.00162 | 1.11285821 |
| Prrg4 | 0.82805 | 0.07817745 |
| Clpb | 0.40676 | -0.3426724 |
| Kcnj12 | 0.69985 | -0.10916409 |
| Prune | 0.01134 | 1.38485901 |
| Med28 | 0.22872 | -0.39062298 |
| Ccdc50 | 0.00204 | 1.02772616 |
| Chd2 | 0.08702 | 0.56573711 |
| Rnf6 | 0.62003 | -0.0974692 |
| Usp7 | 0.00199 | -0.99359929 |
| Epha4 | 0.98227 | 0.00752064 |
| Tgs1 | 0.00145 | 1.78642249 |
| Snx2 | 0.00144 | 1.46747383 |
| Meg3 | 0.61748 | -0.13303199 |
| Uhrf2 | 0.00144 | 2.75903419 |
| Olfml3 | 0.05415 | -0.45154098 |
| Hp1bp3 | 0.00144 | -1.37586312 |
| Abca5 | 0.02287 | 0.89171814 |
| Fto | 0.87045 | 0.0456102 |
| Orc1 | 0.31383 | 0.50763158 |
| Rhbdl2 | 0.00144 | -1.29085005 |
| Pnmal2 | 0.17089 | -0.23926792 |
| BC003331 | 0.12168 | 1.12639883 |
| Gm19451 | 0.41443 | -0.29561145 |
| Vgll4 | 0.00144 | -1.02923895 |
| Wnk2 | 0.35555 | -0.29383152 |
| Kremen1 | 0.30238 | -0.34142332 |
| 9130011E15Rik | 0.13937 | -0.39507481 |
| 1700025G04Rik | 0.00158 | 1.06126472 |
| Pde4b | 0.00383 | 1.18656901 |
| Idh1 | 0.00144 | -1.39356003 |
| Cadm2 | 0.01718 | -0.77229162 |
| Mef2c | 0.09025 | 0.43153956 |
| Kat6a | 0.02321 | -0.67879654 |
| Ubfd1 | 0.0204 | -0.50370053 |
| Tasp1 | 0.91997 | 0.12939884 |
| Rarb | 0.00144 | -1.31884045 |
| Gria2 | 0.00144 | -1.29746368 |
| Fam46c | 0.31347 | -0.3345504 |
| Ankhd1 | 0.00144 | 3.20767451 |
| Tardbp | 0.00298 | 0.87610559 |
| Atf7 | 0.0123 | 0.62047831 |
| Ttc3 | 0.83309 | 0.04702403 |
| Rabep2 | 0.31903 | 0.19176133 |
| Rab14 | 0.00876 | 1.81305432 |
| COX1 | 0.00713 | -0.65409117 |
| Psmd3 | 0.32543 | -0.6729724 |
| L1cam | 0.53456 | 0.14552355 |
| Hook2 | 0.08571 | 0.76988326 |
| Crcp | 0.02411 | 0.4964985 |
| Gm30494 | 0.00144 | -1.29864962 |
| Uhrf2 | 0.01163 | 1.56029815 |
| Ugcg | 0.01513 | 0.9886987 |
| Wwc2 | 0.0141 | 0.65012325 |
| 2900097C17Rik | 0.01074 | -0.60769807 |
| Caskin1 | 0.00388 | -0.74358202 |
| Gm11744 | 0.04517 | -0.45354718 |
| Ccdc116 | 0.59559 | 0.2524602 |
| Zfp937 | 0.26331 | 0.46178326 |
| Wdr82 | 0.46634 | 0.16943876 |
| D030024E09Rik | 0.02016 | -0.98313485 |
| Luc7l2 | 0.26209 | 0.42997222 |
| Btg4 | 0.00144 | -1.27699683 |
| 2900097C17Rik | 0.00144 | -1.12220686 |
| Vmn1r69 | 0.0017 | -1.24950944 |
| Matr3 | 0.02448 | 0.77731369 |
| Tmem181b-ps | 0.62295 | -0.24578337 |
| St8sia6 | 0.00144 | 2.92304212 |
| Rbm5 | 0.00661 | 1.62798081 |
| Iws1 | 0.87091 | 0.05950795 |
| Celf2 | 0.49919 | 0.23964441 |
| Iqgap1 | 0.34936 | -0.24205136 |
| Maf1 | 0.00153 | -1.0274568 |
| Pkp4 | 0.71913 | 0.06845601 |
| Eef2k | 0.00729 | -0.79922368 |
| Tenm4 | 0.0458 | -0.8584786 |
| Rasa2 | 0.95295 | 0.026056 |
| Tuft1 | 0.00144 | -1.28116048 |
| Bmpr2 | 0.49066 | 0.19600014 |
| Tcaf3 | 0.27954 | -0.69415405 |
| Gm31001 | 0.00296 | -1.49566245 |
| Ampd3 | 0.0063 | 0.8739054 |
| Ccdc47 | 0.00304 | 0.87743969 |
| Ssh2 | 0.07547 | 0.7968349 |
| Gm36369 | 0.11219 | -1.13267702 |
| Ak3 | 0.32187 | -0.16131641 |
| Zcrb1 | 0.02141 | 1.93076925 |
| LOC102638940 | 0.82675 | -0.08322866 |
| Slco2a1 | 0.7453 | 0.27476571 |
| Zfp91 | 0.1167 | 1.31616588 |
| 4933436I20Rik | 0.14511 | -0.91501687 |
| Grm7 | 0.03868 | -0.89592444 |
| Klhl35 | 0.1017 | -0.75710067 |
| Olfr1259 | 0.00145 | -1.22376446 |
| F630111L10Rik | 0.0385 | -0.86465043 |
| Satb1 | 0.00312 | -1.22465584 |
| Gm41518 | 0.00381 | -0.74632384 |
| Ikbke | 0.07796 | -0.91914462 |
| Dtymk | 0.60562 | -0.09255863 |
| BC048594 | 0.08674 | -0.42469731 |
| Ing5 | 0.66982 | -0.07689327 |
| Atf7ip | 0.20454 | 0.5461115 |
| Ank2 | 0.58151 | 0.18859373 |
| Olfr1420 | 0.00555 | -0.89020322 |
| Tmx1 | 0.39802 | 0.42219585 |
| Arfgef1 | 0.06289 | 0.89652011 |
| Tctn2 | 0.96299 | 0.02758014 |
| Syf2 | 0.45634 | -0.11689841 |
| Mapk8ip3 | 0.09437 | -0.6994136 |
| Rpl41 | 0.00293 | -1.22465261 |
| Col11a1 | 0.0111 | -1.02598223 |
| Hcrtr2 | 0.00144 | -1.27165604 |
| Gnas | 0.08153 | 1.27527652 |
| Kctd18 | 0.13335 | 0.59320924 |
| Tor1b | 0.00144 | -1.06676925 |
| LOC102634132 | 0.03396 | -1.28901887 |
| D430020J02Rik | 0.00144 | -2.00203425 |
| 9830163H01Rik | 0.67464 | -0.17687702 |
| Gm4876 | 0.00215 | -1.518735 |
| Nt5dc1 | 0.00276 | 1.31104388 |
| Satb2 | 0.07888 | 0.38326062 |
| Hrasls5 | 0.03791 | -0.42594145 |
| Cbfa2t3 | 0.00264 | -0.77369596 |
| Tacc1 | 0.01738 | -0.57599775 |
| Atp6v1b2 | 0.94008 | -0.02271849 |
| Pes1 | 0.0175 | -0.60756214 |
| Pfkl | 0.00144 | -1.25118919 |
| Olfr316 | 0.00144 | -1.32934245 |
| Gm33733 | 0.61895 | -0.33427167 |
| Cc2d2b | 0.10684 | 0.71496102 |
| Itch | 0.27673 | 0.26492187 |
| Vamp1 | 0.00383 | -1.13029182 |
| Gm17382 | 0.98039 | 0.01371858 |
| 5430401H09Rik | 0.01778 | -1.27487413 |
| Gm33228 | 0.00383 | -1.13029182 |
| Anp32a | 0.02433 | -0.6541099 |
| Gm4035 | 0.00144 | -1.38944037 |
| Faim | 0.70907 | -0.15774415 |
| Gcg | 0.06192 | -0.43200212 |
| C130093G08Rik | 0.17287 | -0.7678042 |
| 4833423E24Rik | 0.00144 | -1.29085005 |
| Car3 | 0.15167 | -0.65700698 |
| Hif1a | 0.00385 | 0.74445387 |
| 4930503L19Rik | 0.00144 | 2.053844 |
| 2410137M14Rik | 0.00442 | -0.7613049 |
| Hmcn1 | 0.10603 | -0.5949957 |
| 9830163H01Rik | 0.4006 | -0.34479003 |
| Arl15 | 0.60277 | -0.59902266 |
| Neil2 | 0.50004 | 0.27547611 |
| AW146154 | 0.63094 | 0.15868677 |
| Slc25a16 | 0.37901 | -0.19508778 |
| Tbck | 0.13477 | -1.70238174 |
| Pif1 | 0.00145 | -1.24191372 |
| Bcl7a | 0.00951 | -1.10679531 |
| Nlrp5 | 0.33169 | -0.1600718 |
| Nlrp5 | 0.01421 | 1.12805808 |
| Rnf150 | 0.04409 | -0.62897326 |
| Myof | 0.19138 | 0.25050186 |
| Il2 | 0.00149 | -1.21097148 |
| Abcc5 | 0.02323 | 0.52941596 |
| Epb4.1l3 | 0.01663 | -0.93427981 |
| Sppl2b | 0.02143 | -0.66396994 |
| Pink1 | 0.09827 | -0.40281432 |
| Iigp1 | 0.0341 | 1.04524147 |
| Dhx15 | 0.02362 | 0.74927122 |
| Dhx15 | 0.00144 | 2.00636863 |
| A330023F24Rik | 0.74597 | 0.13787765 |
| A530072M11Rik | 0.25369 | -0.43397931 |
| Timm9 | 0.06367 | 0.74641852 |
| Anxa1 | 0.00144 | -1.26560808 |
| Rnf150 | 0.32859 | -0.97560609 |
| St3gal1 | 0.77041 | 0.07644748 |
| Ilvbl | 0.02993 | -0.70017201 |
| Hectd1 | 0.02745 | 1.08436941 |
| Hectd1 | 0.13312 | 0.86973122 |
| Gemin6 | 0.88033 | -0.04286756 |
| Gm16529 | 0.01043 | 0.66972733 |
| Gm16529 | 0.21192 | -1.27622494 |
| Vps13a | 0.79447 | 0.14285557 |
| Metap1 | 0.97794 | -0.00991084 |
| Tgfa | 0.55171 | -0.40177297 |
| Emc3 | 0.01568 | 1.21338266 |
| Sugp1 | 0.40234 | 0.41556841 |
| Erap1 | 0.00149 | -1.2703832 |
| Dlgap1 | 0.30143 | 0.31863068 |
| Wdr18 | 0.01995 | -0.67569694 |
| Frs2 | 0.17206 | -0.34990459 |
| Gm9946 | 0.00144 | -1.41515133 |
| Tulp4 | 0.01039 | 1.26389908 |
| Ppp2r5c | 0.35816 | -0.2270332 |
| Psmd7 | 0.40591 | -0.26827438 |
| Mctp2 | 0.00256 | -1.12235164 |
| Itgal | 0.29755 | -0.35735079 |
| Zc3h11a | 0.55754 | 0.13301124 |
| Gm16163 | 0.45788 | -0.1874157 |
| Gm15834 | 0.11184 | -0.52228481 |
| Armcx2 | 0.00144 | -1.26934956 |
| Edem3 | 0.32079 | 1.00006996 |
| Fuk | 0.00317 | -1.50158293 |
| Sos1 | 0.95514 | 0.02890582 |
| Pip4k2c | 0.18647 | 0.52977943 |
| Jmy | 0.01123 | -0.66585404 |
| C230062I16Rik | 0.25618 | 0.19529826 |
| Ccnk | 0.00144 | -1.32287053 |
| Gm32014 | 0.17058 | -0.62545622 |
| A330076C08Rik | 0.24116 | -0.66367041 |
| Pcdh17 | 0.00144 | 2.17172159 |
| Arhgap5 | 0.00353 | 1.70313978 |
| Cd180 | 0.01581 | -1.01479804 |
| Cd180 | 0.10379 | -0.48244815 |
| Podxl2 | 0.08889 | -0.89317129 |
| Tep1 | 0.0052 | -0.94932459 |
| Nipal2 | 0.00145 | -1.22376446 |
| Rfx2 | 0.07268 | 1.47216828 |
| Gnas | 0.19136 | 0.49677416 |
| E130314K07Rik | 0.0281 | -0.56519642 |
| Gm13111 | 0.70361 | 0.14437923 |
| Rmst | 0.16754 | 0.37846531 |
| 9630033C03Rik | 0.00245 | 2.57579259 |
| 9630033C03Rik | 0.0029 | 2.82635565 |
| Rmst | 0.12125 | 0.55018955 |
| Ppp1r14c | 0.03929 | -2.18018086 |
| Ppp1r14c | 0.05955 | -1.1013354 |
| Gtf2ird2 | 0.94682 | -0.02383368 |
| C030034L19Rik | 0.95272 | 0.03649638 |
| Mob3a | 0.06177 | -0.56399382 |
| 9530059O14Rik | 0.00144 | -1.69248136 |
| 9530059O14Rik | 0.12375 | -0.99938307 |
| Api5 | 0.00144 | 2.74779386 |
| LOC102636317 | 0.68726 | 0.21165021 |
| 4930403D09Rik | 0.00144 | -1.26560808 |
| Kctd1 | 0.13961 | 1.02801029 |
| Foxn3 | 0.82964 | 0.1167688 |
| 9530036O11Rik | 0.00174 | -1.30713006 |
| 9530036O11Rik | 0.00144 | -1.4333006 |
| Arhgap44 | 0.06397 | -0.98161948 |
| Lpp | 0.02083 | 1.20190761 |
| Cttnbp2nl | 0.04772 | -0.67889094 |
| Iqgap3 | 0.11307 | -1.24082201 |
| Gm13571 | 0.00145 | -1.22376446 |
| Hspbap1 | 0.68063 | 0.16313967 |
| C330019F10Rik | 0.00144 | -1.27165604 |
| Cdv3 | 0.28099 | -0.27133963 |
| Olfr1299 | 0.00144 | -1.28898079 |
| Mov10 | 0.08747 | -0.9558499 |
| Lsm1 | 0.30524 | 0.3253414 |
| Rbbp8 | 0.00214 | 1.88545042 |
| Zmiz1os1 | 0.03432 | -0.68370137 |
| Suds3 | 0.00157 | -1.19247859 |
| Dock4 | 0.99132 | -0.00274017 |
| Sidt1 | 0.01889 | -1.29807826 |
| Kit | 0.48375 | -0.26511184 |
| Kit | 0.19342 | 0.66828828 |
| Gm4876 | 0.97672 | 0.02112069 |
| Mdh1 | 0.21828 | 0.48629172 |
| Krit1 | 0.07446 | 1.24946987 |
| Zfp950 | 0.42435 | 0.30860881 |
| Rfwd3 | 0.44844 | 0.33608103 |
| Lpp | 0.01621 | 1.20690644 |
| Pola2 | 0.64075 | -0.23726396 |
| Pola2 | 0.39426 | -0.39556265 |
| Cblb | 0.11535 | 1.01113097 |
| Cblb | 0.15118 | 0.82862952 |
| A930011G23Rik | 0.56338 | -0.18006583 |
| A830039H05Rik | 0.01341 | 0.72313957 |
| Stmn2 | 0.00144 | 1.61742286 |
| LOC102640344 | 0.00144 | -1.30713006 |
| 9330158H04Rik | 0.29917 | 0.43582383 |
| Rad9b | 0.99579 | 0.00338843 |
| LOC102635844 | 0.01177 | 1.06452517 |
| Stau1 | 0.18324 | -0.43301886 |
| Acss2 | 0.37026 | -0.38874351 |
| Dpp3 | 0.34517 | -0.41435285 |
| A630001O12Rik | 0.15891 | -2.22340165 |
| Xpo7 | 0.01517 | -0.66098183 |
| Gm27230 | 0.00144 | -1.20555712 |
| Clic5 | 0.54061 | -0.1845922 |
| C030034L19Rik | 0.00291 | -1.1949875 |
| C030034L19Rik | 0.0096 | -1.61891225 |
| Timm9 | 0.03027 | -0.73065044 |
| A330015K06Rik | 0.00144 | -1.29049509 |
| A230098N10Rik | 0.01453 | -1.37508608 |
| C130026L21Rik | 0.9083 | -0.04015041 |
| A230072E10Rik | 0.33733 | -1.79708671 |
| A230072E10Rik | 0.66914 | -0.15925276 |
| Gm17344 | 0.0018 | -1.21110851 |
| Gabrg1 | 0.00158 | -1.80943224 |
| Gabrg1 | 0.00144 | -1.29085005 |
| Slc38a9 | 0.28914 | 0.446014 |
| Map3k4 | 0.00144 | -1.25118919 |
| Map3k4 | 0.01165 | -0.90700802 |
| 9330210C06 | 0.02097 | -3.55508613 |
| Hmgxb3 | 0.92633 | -0.02977734 |
| Lta4h | 0.12643 | 0.49956763 |
| Nrde2 | 0.94995 | -0.02135147 |
| Wdr18 | 0.84688 | -0.08762018 |
| Sh3bgrl2 | 0.27015 | -0.3410072 |
| Sh3bgrl2 | 0.00144 | -1.28116048 |
| BC039771 | 0.21719 | 0.79456687 |
| Dera | 0.58011 | 0.19017969 |
| Usp25 | 0.10082 | 0.6873739 |
| Slc35g1 | 0.00145 | -1.2388121 |
| Gm10619 | 0.00144 | -1.27165604 |
| Sh3bgrl2 | 0.00158 | -1.2446827 |
| Nr2c1 | 0.00144 | -1.31495282 |
| Lman2 | 0.23457 | -0.43503935 |
| 5430401H09Rik | 0.30086 | -0.79030357 |
| Magi2 | 0.44153 | -0.35402032 |
| Galnt6os | 0.41679 | 0.32599123 |
| D730003I15Rik | 0.02401 | -0.49461163 |
| Spata9 | 0.1145 | -2.763239 |
| 2810403A07Rik | 0.00144 | 1.59204105 |
| Plekha6 | 0.49043 | 0.21038971 |
| 1810024B03Rik | 0.02628 | -0.67253712 |
| 6230400D17Rik | 0.6126 | -0.09172266 |
| 5830403F22Rik | 0.24565 | -0.43991627 |
| Atp10d | 0.85431 | 0.07718937 |
| Rab3gap2 | 0.01255 | 0.71747501 |
| Gmpr2 | 0.08858 | 0.66330744 |
| Uba6 | 0.89678 | -0.03390243 |
| Ppp2r3c | 0.0629 | 0.8939602 |
| Car3 | 0.02711 | -0.67870883 |
| 4930500H12Rik | 0.00144 | -1.27165604 |
| Luc7l2 | 0.40344 | -0.2204028 |
| 4930430M16Rik | 0.85165 | 0.06341223 |
| 4833447I15Rik | 0.35942 | 0.40347013 |
| Gm15270 | 0.93927 | 0.02737108 |
| Impa1 | 0.61443 | 0.30680739 |
| Exosc1 | 0.49725 | -0.19796189 |
| Hip1 | 0.24071 | -0.28043408 |
| Pfkl | 0.00271 | -1.74421812 |
| P2ry6 | 0.94521 | -0.01883426 |
| Mpc2 | 0.21418 | 0.32727673 |
| 4932702P03Rik | 0.00144 | -1.28116048 |
| 4932702P03Rik | 0.27965 | 0.27631464 |
| 1700019L22Rik | 0.09551 | -1.49803939 |
| D130017N08Rik | 0.23757 | -0.68876164 |
| Srr | 0.01673 | 0.71213867 |
| Dnmt3b | 0.00144 | -1.29049509 |
| Gja3 | 0.62628 | -0.16001908 |
| Psme4 | 0.11001 | 0.5696193 |
| Pira6 | 0.00144 | -1.26560808 |
| Scnn1b | 0.22098 | -0.68331077 |
| Atp8b2 | 0.03457 | -1.6999475 |
| Gmppa | 0.47742 | 0.53534137 |
| Tph1 | 0.0018 | -1.21153109 |
| Acy1 | 0.56781 | 0.47783949 |
| Ano10 | 0.32122 | -0.38031228 |
| Pitpnm1 | 0.22017 | -0.47518898 |
| Rps5 | 0.00601 | -0.92765134 |
| Fstl5 | 0.00144 | -1.28116048 |
| Skil | 0.03166 | 0.51808907 |
| Adra1a | 0.62324 | -0.24334873 |
| Gm5859 | 0.24878 | -0.44138991 |
| Gjb6 | 0.00317 | -1.11935153 |
| Ttc39aos1 | 0.56642 | 0.11237766 |
| LOC102641978 | 0.49135 | -0.31272308 |
| Stx6 | 0.27775 | -0.19241759 |
| Slmo2 | 0.86396 | 0.03028255 |
| Tmem126a | 0.65356 | 0.13716427 |
| Mboat7 | 0.05612 | -0.61873347 |
| Rplp1 | 0.00144 | -1.40569224 |
| Cenpk | 0.7816 | 0.11012239 |
| Ywhab | 0.01786 | 0.55879432 |
| Cc2d2b | 0.00144 | -1.57415534 |
| Gm16367 | 0.06847 | -0.74119416 |
| Gm3893 | 0.67245 | -0.29978126 |
| Cbll1 | 0.0022 | -1.40110407 |
| Fbxo10 | 0.23544 | -0.75283985 |
| Csrp2bp | 0.04171 | -0.43035901 |
| Zmynd8 | 0.59651 | -0.37389627 |
| Tpm3 | 0.00753 | -0.67545436 |
| Sun1 | 0.06579 | -0.87715946 |
| Gm27227 | 0.33142 | -1.67055431 |
| Upk1b | 0.00144 | -1.33632959 |
| Mgea5 | 0.00211 | 1.15840025 |
| Tmem209 | 0.28916 | 0.19437562 |
| Rarres1 | 0.19259 | 0.28622271 |
| Micu3 | 0.81655 | -0.08133092 |
| Snip1 | 0.09722 | -0.32908585 |
| Itpr1 | 0.46725 | -0.21010633 |
| Nrxn2 | 0.60171 | -0.1786843 |
| Greb1 | 0.00144 | -1.29049509 |
| Clip1 | 0.03741 | 0.85299006 |
| Mtr | 0.00167 | -0.87909015 |
| Cttn | 0.04711 | -0.68912041 |
| Cpt1c | 0.02481 | -0.59742276 |
| Dhx30 | 0.01314 | -1.15614013 |
| B4galnt1 | 0.12946 | -0.61779235 |
| Parp9 | 0.27022 | -0.49916655 |
| Matn4 | 0.0088 | -0.85951544 |
| Fam160a2 | 0.09092 | -0.6116892 |
| Cdc73 | 0.01964 | 0.69108897 |
| Cldnd1 | 0.18657 | 0.4684584 |
| Twsg1 | 0.02355 | 0.54677431 |
| Ptgr2 | 0.01607 | 0.64802187 |
| Rbm15b | 0.00156 | -1.13798902 |
| Rfk | 0.98771 | 0.00317652 |
| Ormdl1 | 0.05983 | 0.44516413 |
| Hs3st5 | 0.0325 | -0.72459244 |
| Cryz | 0.57027 | -0.17175831 |
| Stard3 | 0.02698 | -0.72514374 |
| Elmod3 | 0.00187 | -1.08724743 |
| Smpx | 0.04224 | -0.3785079 |
| Fnbp4 | 0.00144 | 1.96565706 |
| Arl6ip4 | 0.15149 | -0.36818125 |
| Osbpl10 | 0.58584 | 0.17159532 |
| Rdh11 | 0.356 | 0.19752689 |
| Zfp934 | 0.00667 | 0.82633769 |
| Zfp207 | 0.04558 | 1.50648416 |
| Olfr58 | 0.00144 | -1.31884045 |
| Gid4 | 0.86192 | -0.07048965 |
| Rxra | 0.00356 | -0.88385591 |
| Fbn1 | 0.31329 | 0.19856623 |
| Herpud2 | 0.40946 | 0.20856508 |
| Stard8 | 0.03014 | -0.57069652 |
| Dlg4 | 0.05119 | -0.48175115 |
| Lyrm5 | 0.00432 | 0.77230052 |
| Acaca | 0.07413 | -1.00159218 |
| Hsph1 | 0.04266 | -0.85111894 |
| Mep1b | 0.06358 | -1.13077247 |
| Trim41 | 0.01102 | 1.02459116 |
| Epb4.1l1 | 0.07178 | -0.51201763 |
| Cdon | 0.20385 | -0.93843865 |
| Ttll7 | 0.00509 | -0.68619093 |
| Cnga3 | 0.05129 | -0.59956685 |
| Phc2 | 0.26303 | -0.39325888 |
| Ntrk3 | 0.00144 | -1.36162963 |
| Usp35 | 0.03669 | -0.94779588 |
| Gm26614 | 0.13003 | -2.80264431 |
| Zic4 | 0.09466 | -2.72001876 |
| Sema6d | 0.24913 | -0.57587591 |
| Baz2b | 0.57232 | 0.43469783 |
| Bcl3 | 0.00145 | -1.2388121 |
| Tex101 | 0.03079 | -1.49925177 |
| Prdm9 | 0.86144 | 0.24411386 |
| Actr3 | 0.00559 | -0.74746505 |
| Fam134a | 0.00144 | -1.07953887 |
| Cfap221 | 0.00144 | -1.24613586 |
| LOC102640325 | 0.15522 | -0.63874388 |
| Nova1 | 0.00144 | -1.30713006 |
| Gm16096 | 0.00144 | -1.28898079 |
| Klf8 | 0.00144 | -1.29864962 |
| Tmbim4 | 0.05471 | -0.61973013 |
| Slc6a2 | 0.00158 | -1.17284736 |
| 2700046A07Rik | 0.00144 | -1.24811894 |
| Tsc22d1 | 0.48165 | 0.2506803 |
| Gm33586 | 0.00222 | -1.1481805 |
| Grm4 | 0.00145 | -1.2388121 |
| Plekha1 | 0.00232 | -1.06571598 |
| 4930431P22Rik | 0.12057 | 0.30284808 |
| App | 0.03333 | 0.49789503 |
| Gm28154 | 0.00744 | -1.03871255 |
| Gid8 | 0.04216 | -0.56874272 |
| Fam47e | 0.01596 | -0.88179945 |
| Lrrc16b | 0.00144 | -2.14976802 |
| Pgr | 0.00144 | -1.30713006 |
| Gm33464 | 0.23234 | -0.61004446 |
| Il7 | 0.06202 | -0.86718816 |
| Mroh5 | 0.00144 | -1.26560808 |
| Pilrb1 | 0.32618 | -0.31218013 |
| Polg2 | 0.6603 | -0.0971998 |
| C230035I16Rik | 0.02826 | -1.24124528 |
| Etf1 | 0.03643 | -0.58633783 |
| Ccna2 | 0.36915 | -0.38366052 |
| Nudt8 | 0.21079 | -1.85247766 |
| Soat1 | 0.00144 | -1.41803081 |
| Mical2 | 0.8294 | -0.09221621 |
| Aldh3a2 | 0.16248 | -0.60710541 |
| Aatk | 0.00149 | -1.19696847 |
| Prss3 | 0.64738 | -0.19546123 |
| Il13ra2 | 0.05194 | -1.60330962 |
| Lamb3 | 0.86236 | -0.05084655 |
| Gsn | 0.00389 | -0.95991147 |
| Elk4 | 0.00144 | -1.25118919 |
| Vti1a | 0.00764 | -0.67721755 |
| Snap47 | 0.00144 | -1.19386552 |
| Slco4a1 | 0.02908 | -1.01028165 |
| Rbpj | 0.00211 | -1.16847448 |
| Ccdc125 | 0.07175 | -0.7871359 |
| Gm355 | 0.00227 | -1.81159752 |
| Rec8 | 0.00144 | -1.28898079 |
| Fpgs | 0.01215 | -1.30968803 |
| Ssx2ip | 0.01044 | -0.93236333 |
| Wdsub1 | 0.70962 | 0.16827201 |
| St6galnac4 | 0.03465 | -0.67482415 |
| Fbxo7 | 0.86374 | 0.06074155 |
| Slc39a11 | 0.00145 | -1.2388121 |
| Lpin3 | 0.04098 | -1.23679773 |
| Nup54 | 0.15794 | -0.39965924 |
| Apex1 | 0.81272 | 0.16900031 |
| Eps8 | 0.01505 | -1.32135476 |
| Dpy19l1 | 0.42895 | 0.41600999 |
| Gcn1l1 | 0.07479 | -0.44410959 |
| LOC102638761 | 0.00158 | -1.17515383 |
| Dcun1d4 | 0.0961 | -0.71501678 |
| 4930423D24Rik | 0.03149 | -1.17638884 |
| 2310003H01Rik | 0.00827 | -0.8822204 |
| Bcl9l | 0.10201 | -0.75228937 |
| Top3b | 0.01001 | -1.57512587 |
| Tectb | 0.72123 | -0.27201185 |
| Ruvbl2 | 0.93955 | 0.02071172 |
| LOC102635252 | 0.00144 | -1.28116048 |
| Gm40627 | 0.00144 | -1.28116048 |
| Pkp4 | 0.24526 | 0.59221791 |
| Eme2 | 0.0662 | -0.77416326 |
| Kifc2 | 0.00574 | -1.62503401 |
| Hsd17b6 | 0.00144 | -1.28116048 |
| Lrrc14 | 0.01693 | -0.91119376 |
| Ell2 | 0.02841 | -0.68197196 |
| Cyb5rl | 0.00145 | -1.26219879 |
| B3gat1 | 0.26019 | -0.74068037 |
| Fam198a | 0.02728 | -1.4299159 |
| Mgam | 0.1639 | 0.376708 |
| Ccnt1 | 0.0091 | -0.67246769 |
| Furin | 0.07511 | -0.43460092 |
| Brwd1 | 0.04196 | 0.56501251 |
| Sort1 | 0.00145 | -1.31271171 |
| 4933427D14Rik | 0.03365 | 0.49454668 |
| Syt11 | 0.08347 | -0.59741601 |
| Slc37a3 | 0.01513 | -0.84381566 |
| Cxcl14 | 0.00288 | -1.18478579 |
| Pde4dip | 0.57782 | 0.13342435 |
| Fen1 | 0.02765 | -0.6221729 |
| Ppp1r13l | 0.00144 | -1.22925777 |
| Atp1a2 | 0.2204 | -0.22710772 |
| Gucy2f | 0.14106 | -0.72217027 |
| Pnkp | 0.19825 | -1.00745594 |
| Oxnad1 | 0.77182 | 0.0637211 |
| Crisp2 | 0.00144 | -1.28116048 |
| Fam63a | 0.84152 | -0.04956769 |
| Pla2g6 | 0.00144 | -1.29289397 |
| Pcsk2os1 | 0.00144 | -1.27454685 |
| Herc3 | 0.08346 | -0.5026854 |
| Tnip1 | 0.00513 | -2.08279407 |
| Ephb4 | 0.09156 | -0.31720624 |
| Adhfe1 | 0.08293 | 0.58585907 |
| Gm2824 | 0.00144 | -1.28898079 |
| Taf8 | 0.40503 | -0.21023657 |
| Rcc1 | 0.58134 | -0.27899619 |
| Nyap2 | 0.00144 | -1.30017714 |
| Slc25a42 | 0.46243 | 0.58799884 |
| 4930577N17Rik | 0.13654 | -0.31412324 |
| Gbf1 | 0.00144 | -1.32845339 |
| Cxxc4 | 0.90155 | 0.11672522 |
| Epb4.1l2 | 0.05379 | -0.63243728 |
| Kansl1l | 0.02038 | -0.98980568 |
| Pitpnm3 | 0.00144 | -1.27165604 |
| Traip | 0.05371 | -1.32744438 |
| Lzic | 0.00303 | -0.8650694 |
| Tti1 | 0.04108 | -0.5821224 |
| Slc43a2 | 0.03667 | -0.78563034 |
| Csk | 0.00469 | -0.83379387 |
| Nufip2 | 0.07547 | -0.82218927 |
| Rnf130 | 0.14684 | -0.2630242 |
| Cpq | 0.34149 | -0.17694943 |
| Fbxo11 | 0.0031 | 1.07233915 |
| Lnpep | 0.98195 | 0.00561294 |
| Mbtps2 | 0.74451 | -0.05322567 |
| 4930430M16Rik | 0.0088 | -1.24004069 |
| Ccser1 | 0.00708 | 1.25135538 |
| Atrx | 0.00144 | 1.86147907 |
| Fez1 | 0.21873 | -0.4570589 |
| Wdfy1 | 0.00144 | -1.61724968 |
| Serpinc1 | 0.01642 | -1.6317909 |
| Gnao1 | 0.21341 | -0.31385535 |
| Sema4d | 0.19047 | -0.39163888 |
| Gm13629 | 0.00249 | -1.75485624 |
| Penk | 0.05022 | -0.83700308 |
| Pkd2l2 | 0.76398 | -0.15867042 |
| Ralgds | 0.00144 | -1.1941589 |
| Clec10a | 0.09446 | -0.93353463 |
| Gm11423 | 0.16999 | -0.23464204 |
| Osgepl1 | 0.1598 | 0.29822088 |
| Ggact | 0.24887 | -0.20837993 |
| Dync1i2 | 0.00193 | -1.32582505 |
| Ap1g1 | 0.00144 | -1.33960781 |
| Susd4 | 0.00144 | -1.31495282 |
| Setd5 | 0.84428 | 0.05581711 |
| Plekha2 | 0.08399 | 0.30036741 |
| Lsamp | 0.21289 | -0.58462128 |
| Kctd2 | 0.01083 | 0.86130173 |
| Etohd2 | 0.00144 | -1.36893564 |
| Dusp14 | 0.1624 | 0.87861804 |
| Rhobtb2 | 0.01347 | -1.01042984 |
| Pgpep1 | 0.11364 | -0.61832611 |
| Mturn | 0.00151 | -1.01234262 |
| Lrrc29 | 0.00144 | 1.37956929 |
| Pramel6 | 0.00144 | -1.28898079 |
| Nupr1 | 0.75177 | 0.23089646 |
| Myl1 | 0.10061 | -1.64361298 |
| Hdgfrp2 | 0.03835 | -0.43877856 |
| Mx1 | 0.00144 | -1.26934956 |
| Crkl | 0.68852 | -0.0643456 |
| Rae1 | 0.86207 | -0.0568764 |
| Pkp4 | 0.00438 | -0.80769562 |
| Tprn | 0.00241 | -0.90294395 |
| Hax1 | 0.81466 | -0.10044595 |
| 5530601H04Rik | 0.0042 | 1.3095205 |
| Cdc25b | 0.12794 | -0.94342544 |
| Crb3 | 0.11833 | -0.9170858 |
| Mfsd9 | 0.41669 | -0.30575664 |
| Igf2bp1 | 0.00149 | -1.22582153 |
| Fendrr | 0.26242 | 0.37430498 |
| A930017M01Rik | 0.00145 | -1.1216355 |
| Rpl39 | 0.00144 | -1.14351222 |
| Chrdl1 | 0.25136 | -0.70032315 |
| Cnot11 | 0.91357 | -0.03364582 |
| Vmn1r31 | 0.04156 | -1.99541389 |
| Olfr370 | 0.01689 | -0.8452116 |
| Olfr1180 | 0.03887 | -1.97441964 |
| Gm3669 | 0.00145 | -1.17894292 |
| Psmb2 | 0.84306 | -0.1046791 |
| Gm30302 | 0.26291 | -0.23772009 |
| Olfr725 | 0.02671 | -1.29950918 |
| Gm38666 | 0.00602 | -1.53663094 |
| Rybp | 0.00353 | -1.06721129 |
| AA388235 | 0.4316 | -0.42210318 |
| Susd1 | 0.20366 | -0.47553103 |
| Gm36279 | 0.00723 | -0.90647339 |
| Acacb | 0.01122 | -0.71877565 |
| Gprasp2 | 0.18232 | -0.91280252 |
| Gm3383 | 0.01497 | 1.1862666 |
| Map4 | 0.00248 | -1.31225853 |
| Atp5d | 0.03051 | -0.75110809 |
| Gm10406 | 0.05097 | 0.63868422 |
| Cdc5l | 0.0018 | -0.92987728 |
| Mdfic | 0.91962 | 0.05226609 |
| Tlr3 | 0.00839 | -0.83879588 |
| Gm20236 | 0.63397 | -0.11393954 |
| Ttc34 | 0.18437 | -0.80098822 |
| Uba1y | 0.78543 | 0.112197 |
| Gan | 0.83663 | -0.04526842 |
| Glyctk | 0.01241 | -1.02801808 |
| Ebf4 | 0.00894 | -0.66223145 |
| LOC102640359 | 0.00144 | -1.20579466 |
| Igbp1 | 0.29039 | -0.98133668 |
| Serbp1 | 0.94936 | -0.01612101 |
| Aida | 0.04072 | 0.57204624 |
| Hpca | 0.00144 | -1.34462115 |
| Ubxn10 | 0.02745 | -1.24415004 |
| Gm34872 | 0.05528 | -0.95718388 |
| 4930417O13Rik | 0.00573 | -1.52832083 |
| Gm38460 | 0.00144 | -1.28116048 |
| Ccnd3 | 0.00144 | -1.11418967 |
| C330007P06Rik | 0.52503 | 0.1644617 |
| Sos1 | 0.00545 | 1.36748903 |
| Abcb8 | 0.07358 | -1.0643884 |
| Chd1 | 0.35717 | 0.32361788 |
| A530040E14Rik | 0.03429 | -0.41764383 |
| Safb2 | 0.20239 | -0.21278429 |
| Eef2 | 0.12382 | -0.5626504 |
| Zmat5 | 0.04167 | -0.61103989 |
| Hmgcl | 0.48533 | -0.26961522 |
| Astl | 0.00827 | -2.05478767 |
| Mfhas1 | 0.76587 | -0.10635496 |
| Nlrp9a | 0.86549 | -0.0867076 |
| Surf6 | 0.02056 | 0.52132787 |
| Pard3 | 0.00638 | -0.96388536 |
| Ammecr1l | 0.00929 | -1.04626933 |
| C86695 | 0.00144 | -1.20555712 |
| 2810002D19Rik | 0.08996 | 0.34921482 |
| Gm21975 | 0.00184 | -1.27646041 |
| Kcnv2 | 0.2384 | -0.41464186 |
| Slc38a10 | 0.18889 | -0.38703993 |
| Il6st | 0.00144 | -1.2083687 |
| Nceh1 | 0.00144 | -1.12872984 |
| Zim1 | 0.87791 | -0.04635468 |
| Zranb3 | 0.00486 | -1.01533148 |
| Gria1 | 0.65063 | -0.10558444 |
| Zfp157 | 0.87412 | 0.04689313 |
| Pdzrn3 | 0.00184 | -1.73961398 |
| Vezt | 0.02564 | -0.49239277 |
| Zfp692 | 0.0103 | 0.72534859 |
| Ccnd2 | 0.00158 | -1.06761849 |
| Bbx | 0.66682 | -0.08785387 |
| Gpr19 | 0.17381 | -0.32050711 |
| Siah2 | 0.06872 | -0.6097762 |
| Pcf11 | 0.03405 | 1.1806116 |
| Fam212a | 0.00145 | -0.94638786 |
| Tbl2 | 0.00144 | -1.26239926 |
| Sh3rf2 | 0.13155 | -0.57239018 |
| 1700001O22Rik | 0.07289 | -0.68260568 |
| Hnrnpr | 0.14414 | -0.34747666 |
| Flrt2 | 0.28461 | -0.24219673 |
| LOC101056032 | 0.02985 | -0.53697924 |
| Rsf1 | 0.97808 | -0.00750457 |
| Rps4x | 0.05006 | -0.37629782 |
| Olfr845 | 0.00144 | -1.27699683 |
| Mical1 | 0.29739 | 0.26375923 |
| Kdm1b | 0.13106 | -0.651334 |
| Naglu | 0.00335 | -0.99673281 |
| Rdh13 | 0.3073 | -0.22768991 |
| Tmem200c | 0.10779 | -3.00774276 |
| Hcn1 | 0.42861 | 0.5660999 |
| Oit1 | 0.00217 | -1.62382842 |
| 1700003E16Rik | 0.0365 | -0.86792012 |
| Strbp | 0.67856 | -0.14383349 |
| Gm17577 | 0.00145 | -1.22376446 |
| 4930426L09Rik | 0.01138 | -1.0539015 |
| Vmn1r49 | 0.00605 | -0.86664024 |
| Vmn1r50 | 0.00144 | -1.29746368 |
| Vmn1r218 | 0.0025 | -1.68549172 |
| Vmn1r30 | 0.01625 | -0.74338084 |
| Vmn1r20 | 0.00144 | -1.29085005 |
| Vmn1r39 | 0.00144 | -1.28116048 |
| Vmn1r35 | 0.00144 | -1.29051721 |
| Vmn1r198 | 0.01939 | -2.71496042 |
| Vmn1r200 | 0.00145 | -1.24191372 |
| Ly6h | 0.02057 | -1.74563066 |
| Wdr38 | 0.00344 | -1.01612101 |
| Ppp1r10 | 0.00149 | -1.50169256 |
| Rpl15 | 0.11167 | -0.64923852 |
| Gm4737 | 0.02496 | -0.7064778 |
| Atg3 | 0.00144 | 1.14021739 |
| B4gat1 | 0.18227 | -0.27766919 |
| Cdkn2aip | 0.02448 | 0.76834507 |
| Gssos1 | 0.00144 | -1.45426645 |
| Ing3 | 0.00185 | 1.50007392 |
| Ptk2b | 0.07978 | -0.5425697 |
| Gjc3 | 0.00144 | -1.31495282 |
| Wisp1 | 0.01239 | -0.56638524 |
| Rgs7bp | 0.38353 | -0.1503663 |
| Ppip5k2 | 0.00144 | 1.86505529 |
| Tmco3 | 0.00144 | -1.47619583 |
| Brd9 | 0.02002 | 0.45008223 |
| Rab28 | 0.579 | -0.0988139 |
| Kidins220 | 0.0328 | 0.67245004 |
| Dusp14 | 0.00144 | -1.41634415 |
| 1700066O22Rik | 0.00144 | -1.29051721 |
| Plekhb2 | 0.40537 | -0.24306887 |
| Alcam | 0.17822 | 0.47666494 |
| Sel1l | 0.22887 | -0.21196298 |
| Pdzd2 | 0.00315 | 0.77366011 |
| Prr13 | 0.0035 | -0.78530963 |
| Rfpl4 | 0.23629 | 0.43466873 |
| Prkcdbp | 0.00324 | -0.72269153 |
| Lsmem2 | 0.01208 | -0.64464506 |
| Setd2 | 0.00277 | 2.16465089 |
| Trim36 | 0.23549 | 0.60436259 |
| Tceal3 | 0.01043 | -0.63509557 |
| Ist1 | 0.00149 | -1.34661972 |
| Trit1 | 0.54634 | -0.16531916 |
| Ndufs8 | 0.00583 | -0.8555134 |
| Fam174b | 0.03691 | -0.60972945 |
| Gm29886 | 0.22159 | -0.48439088 |
| Pramef25 | 0.00144 | -1.31884045 |
| Nsun4 | 0.07818 | -0.30774866 |
| Zswim6 | 0.24959 | 0.19542362 |
| Ddx3x | 0.37366 | -0.19630296 |
| Glycam1 | 0.00144 | -1.48029524 |
| Swsap1 | 0.01325 | -0.68191371 |
| Fam35a | 0.80611 | -0.04706563 |
| Akap11 | 0.00581 | 1.02175915 |
| Magee1 | 0.46388 | 0.11866552 |
| Agtr2 | 0.5517 | -0.33614993 |
| Map2k6 | 0.04647 | 0.3654724 |
| Kcnip4 | 0.07005 | -2.22504311 |
| Rpl3 | 0.05344 | -0.47935374 |
| Lnp | 0.00375 | 1.15801697 |
| Sash1 | 0.02642 | 0.46371585 |
| Fkrp | 0.13142 | -0.47599941 |
| 1190002N15Rik | 0.04873 | 0.45810939 |
| Psmg3 | 0.08477 | -0.46743037 |
| Setd2 | 0.01718 | 0.54365732 |
| Pwp1 | 0.56943 | -0.09719902 |
| Cnot2 | 0.02771 | 0.62787164 |
| Ubqln2 | 0.05893 | 0.4240794 |
| Sfxn2 | 0.01134 | 0.8148819 |
| Ube3a | 0.00304 | -0.78269978 |
| Fam101b | 0.0118 | -0.65014814 |
| Slc25a14 | 0.73566 | -0.24592085 |
| Tuba3a | 0.00883 | -0.95783134 |
| Usp39 | 0.0542 | -0.4038321 |
| Wls | 0.00182 | -0.82290014 |
| Mrps22 | 0.11629 | 0.47153436 |
| Pnisr | 0.59536 | 0.25699885 |
| Cadps | 0.00144 | -1.29049509 |
| Fam126b | 0.28124 | 0.40439367 |
| Tef | 0.59279 | -0.16145324 |
| Atg4b | 0.00338 | -0.78828448 |
| Ano10 | 0.12047 | -0.39216787 |
| Mbtps1 | 0.15471 | -0.24227545 |
| Slc30a5 | 0.01314 | 0.62599411 |
| Ube3a | 0.52354 | 0.17288197 |
| Ndn | 0.00354 | -0.72531084 |
| Cd44 | 0.01552 | -0.76693726 |
| C1d | 0.03402 | 0.54350342 |
| Slc4a4 | 0.21247 | -0.20531145 |
| Poldip2 | 0.84976 | 0.11321203 |
| Polr1d | 0.83121 | 0.04442131 |
| Hmgcs2 | 0.00144 | -2.30046855 |
| Ifit1 | 0.00187 | 2.9365448 |
| Aldh18a1 | 0.02293 | -0.50999437 |
| Stxbp5 | 0.00144 | 1.34726566 |
| Cntrob | 0.7165 | 0.06302512 |
| Dnajb4 | 0.60595 | -0.18609498 |
| Zfp389 | 0.17828 | -0.41900814 |
| Slc26a6 | 0.00578 | -0.98117867 |
| Dsg2 | 0.12046 | 0.38624937 |
| Gtf3c2 | 0.24275 | 0.20400459 |
| Rdh10 | 0.66982 | 0.19797303 |
| Pomgnt2 | 0.01256 | -0.91027439 |
| Tmem161b | 0.00339 | 1.35323275 |
| Gins4 | 0.6375 | -0.08256997 |
| Hdgf | 0.21611 | -0.27310624 |
| Atp10a | 0.82231 | -0.06296105 |
| Acp1 | 0.07091 | 0.4079232 |
| Snx24 | 0.00864 | 0.66677816 |
| Rps3a1 | 0.04466 | -0.37812908 |
| Fam198b | 0.4825 | 0.13191106 |
| Lox | 0.05927 | 0.67800617 |
| Dcbld1 | 0.06113 | 0.53469766 |
| 1700019D03Rik | 0.17152 | 0.37470165 |
| Fam134b | 0.4182 | 0.15410528 |
| Nr1d2 | 0.00144 | 2.04551526 |
| Zmynd11 | 0.00144 | 1.06770171 |
| Hspa8 | 0.089 | 0.6868883 |
| Ncald | 0.00305 | -0.79683202 |
| Appl1 | 0.02002 | 0.52431996 |
| Fgr | 0.07793 | -0.83916986 |
| Arl5a | 0.00946 | 0.87153012 |
| Creb1 | 0.00222 | 1.36147626 |
| Ythdf3 | 0.01414 | 0.59477095 |
| Ctsb | 0.01385 | -0.51339547 |
| Stxbp5l | 0.03491 | -0.97425098 |
| Hltf | 0.00177 | 1.43205568 |
| Dvl2 | 0.00411 | -0.71617172 |
| Tbl3 | 0.00178 | -1.05380055 |
| Mrps27 | 0.35062 | -0.26082162 |
| Fos | 0.07111 | -0.71082331 |
| Dedd | 0.00466 | -0.89817212 |
| Rap2c | 0.00297 | 0.82152379 |
| Macf1 | 0.06219 | 0.4049773 |
| Slc35e3 | 0.24604 | 0.21142076 |
| Frzb | 0.15652 | -0.36503122 |
| Btbd1 | 0.18173 | 0.42535947 |
| Mthfd1 | 0.53846 | -0.17233322 |
| Rpl4 | 0.27071 | 0.18607415 |
| Glt8d1 | 0.01519 | 0.67851657 |
| Smek2 | 0.00243 | 1.07722751 |
| Lrrc57 | 0.01795 | 0.64016527 |
| Tgfbr2 | 0.55115 | -0.09043965 |
| Dhrs3 | 0.01081 | -0.90552065 |
| Fam102a | 0.00144 | -0.94622434 |
| Stx3 | 0.00144 | 2.32077027 |
| Usp32 | 0.00529 | 0.9133301 |
| Rpl7 | 0.69352 | 0.07516603 |
| Tmem30a | 0.02518 | 0.58818795 |
| Atp2b1 | 0.00361 | 0.9629467 |
| A730017C20Rik | 0.00144 | -1.72197021 |
| Rpp14 | 0.51307 | -0.13720237 |
| Btg2 | 0.00144 | -1.36954259 |
| Rab7 | 0.07772 | 0.33038434 |
| Dhfr | 0.10612 | 0.42176002 |
| Grn | 0.00145 | -1.35380373 |
| Hspa8 | 0.13283 | 0.58278398 |
| Rab1 | 0.00144 | 1.19760955 |
| Gm34561 | 0.05442 | -2.22750594 |
| Plscr4 | 0.00427 | 1.45449177 |
| Arl3 | 0.0233 | -0.43985887 |
| Kcnj14 | 0.11363 | -0.57593523 |
| Ncam2 | 0.00149 | -1.22582153 |
| C2cd2 | 0.03161 | -0.41961931 |
| Kansl1 | 0.02251 | 0.90643191 |
| Bcl7b | 0.07299 | -0.47208187 |
| Dok4 | 0.00144 | -1.11533371 |
| Timp4 | 0.12494 | -0.50381136 |
| Qdpr | 0.32175 | -0.24932774 |
| Neurod1 | 0.00144 | -1.24613586 |
| Dusp1 | 0.00988 | -0.73639274 |
| Sod2 | 0.65602 | -0.07732233 |
| Gdf10 | 0.20014 | 0.3243103 |
| Klhl5 | 0.31013 | 0.2280923 |
| Arfgap1 | 0.00953 | -0.55380733 |
| Ppic | 0.20869 | 0.21636607 |
| Sh3bgrl | 0.00415 | 1.60988241 |
| Gpc3 | 0.08357 | -0.53008527 |
| Srsf6 | 0.00963 | 0.56592212 |
| Entpd5 | 0.26621 | -0.26741467 |
| Ilf3 | 0.85296 | -0.03597637 |
| Pkib | 0.11709 | 0.88081472 |
| Ddx4 | 0.21145 | 0.4681487 |
| Rora | 0.15744 | 0.37120197 |
| Kif5b | 0.11202 | 0.48677685 |
| Cav2 | 0.00144 | 2.74632387 |
| Dck | 0.15408 | 0.43746618 |
| Nrep | 0.40803 | -0.15507577 |
| Bcl10 | 0.11928 | -0.29307617 |
| Catsperd | 0.0676 | -0.39479207 |
| Actr2 | 0.0019 | 1.11142849 |
| Gm34463 | 0.20263 | -2.19876696 |
| Runx1 | 0.0523 | -1.76828768 |
| Bcat1 | 0.06008 | -0.49118701 |
| Pdk1 | 0.05725 | -0.35740534 |
| Fmn2 | 0.00144 | -1.29746368 |
| Zfp516 | 0.32359 | -0.17794533 |
| Gdap1 | 0.00144 | -1.29051721 |
| Atg2a | 0.00298 | -0.97816596 |
| Fam214a | 0.47509 | -0.16392099 |
| Trim75 | 0.00158 | -1.2446827 |
| Zfp661 | 0.07754 | -2.05290209 |
| 1600015I10Rik | 0.06784 | -1.11765884 |
| Ldlr | 0.14646 | -0.74677144 |
| Ncaph2 | 0.11535 | -0.57799008 |
| Mfsd5 | 0.00145 | -1.0608115 |
| Padi2 | 0.69032 | 0.40215029 |
| Tle3 | 0.00369 | -0.81609683 |
| N4bp2l2 | 0.01474 | 0.66052845 |
| 9430020K01Rik | 0.1921 | -0.28663413 |
| Rad23b | 0.82065 | -0.03930174 |
| Zfp800 | 0.06819 | 0.46404288 |
| Slitrk3 | 0.07382 | -0.86014901 |
| Tbc1d4 | 0.00275 | -1.05771896 |
| Ppp1r11 | 0.00339 | -0.82672723 |
| Kcnh1 | 0.00144 | -1.29746368 |
| Rab33a | 0.32921 | -0.77747885 |
| Lcmt2 | 0.00315 | -1.08463652 |
| Rnf170 | 0.64383 | -0.14958257 |
| Pold2 | 0.00403 | -0.79731756 |
| Fam129a | 0.00488 | -0.87793772 |
| Supv3l1 | 0.044 | -0.44757565 |
| Gpr171 | 0.00497 | 1.2327858 |
| Armc9 | 0.97398 | -0.00735258 |
| Bzw2 | 0.01256 | -0.53519062 |
| Rgs12 | 0.00144 | -1.51350554 |
| Gabra5 | 0.05361 | -0.77117195 |
| Asl | 0.01248 | -0.51561467 |
| Glb1 | 0.0164 | -0.64495053 |
| Rb1cc1 | 0.20647 | 0.59179459 |
| Lhx1 | 0.4225 | -0.8286578 |
| Srpk2 | 0.05534 | 0.55978101 |
| C130026I21Rik | 0.99343 | -0.00223939 |
| Skil | 0.01031 | 0.76949768 |
| Psmd10 | 0.63657 | -0.09849694 |
| Acad8 | 0.28185 | -0.18490456 |
| Ampd2 | 0.02683 | -1.12164699 |
| Prdx1 | 0.18482 | -0.31893275 |
| Ikzf5 | 0.06252 | 0.44132045 |
| Baz1b | 0.36884 | -0.19553526 |
| Hist2h2be | 0.47866 | 0.12654638 |
| Toporsl | 0.00144 | -1.29864962 |
| Ldhc | 0.00158 | -1.22727468 |
| Lix1l | 0.04706 | -0.62748974 |
| 1200014J11Rik | 0.44164 | -0.12309183 |
| 1600020E01Rik | 0.57692 | -0.09419719 |
| Fbxl6 | 0.00144 | -1.14120857 |
| 1700086L19Rik | 0.11978 | -3.47735002 |
| 1700011H14Rik | 0.09502 | -0.75632578 |
| Ddi1 | 0.00521 | -1.05862264 |
| 1700013G24Rik | 0.00144 | -1.24811894 |
| Fam168a | 0.03265 | -0.40510285 |
| Glrb | 0.69138 | -0.19163628 |
| Hnrnpu | 0.03193 | 0.48344851 |
| Zfp667 | 0.18397 | -0.5889665 |
| Mss51 | 0.0288 | 0.75234676 |
| Prickle1 | 0.00144 | 2.33637579 |
| Ackr1 | 0.96266 | -0.02288545 |
| Lcat | 0.17269 | 0.63866588 |
| Triml1 | 0.00145 | -1.26219879 |
| Ankrd9 | 0.00163 | -1.00026708 |
| Sssca1 | 0.00202 | -0.91700564 |
| Gorasp2 | 0.11166 | -0.32939319 |
| Spata6 | 0.00144 | 1.45349874 |
| 1700123L14Rik | 0.37696 | -0.51231366 |
| Yif1a | 0.01962 | -0.97482915 |
| LOC100862431 | 0.00226 | -1.09509883 |
| H19 | 0.01318 | -1.04085425 |
| Relt | 0.31807 | -0.21720675 |
| Serpinb9 | 0.68023 | -0.13827809 |
| Slit2 | 0.00145 | -1.26219879 |
| Gpr88 | 0.00144 | -1.28116048 |
| Atp2b2 | 0.00981 | -0.89390277 |
| Vwc2 | 0.84753 | -0.0531594 |
| Pdgfc | 0.94002 | 0.01453162 |
| Actr3 | 0.02665 | 0.42726944 |
| Ywhaz | 0.49847 | -0.12213828 |
| Itgb1 | 0.79272 | -0.04475297 |
| Hs2st1 | 0.29314 | 0.19777296 |
| Serpine2 | 0.00294 | -0.96574198 |
| Azin1 | 0.19576 | 0.27233232 |
| Myadm | 0.63097 | -0.08372712 |
| Gm35240 | 0.79411 | 0.37964927 |
| Dnph1 | 0.1984 | -0.21435184 |
| Myt1 | 0.00159 | -1.26431393 |
| Cebpg | 0.02062 | 0.77237179 |
| Six4 | 0.00144 | -1.29049509 |
| Hoxb3 | 0.10534 | -0.29956296 |
| Gm36393 | 0.02243 | -0.75785015 |
| LOC102641621 | 0.57016 | -0.17171278 |
| Gm10069 | 0.64793 | 0.17165288 |
| Zfp772 | 0.9806 | 0.00912345 |
| Rbm3 | 0.13819 | 0.33665014 |
| 1700093A15Rik | 0.00372 | -1.14963365 |
| Actr2 | 0.02939 | -0.53552612 |
| Mrps26 | 0.15127 | -0.36716106 |
| Gm35037 | 0.02941 | -1.71826877 |
| Cd244 | 0.00145 | -0.97160974 |
| Lpl | 0.36245 | -0.24619057 |
| Cox7a2 | 0.00775 | -0.75584173 |
| 2010107G23Rik | 0.51584 | 0.46161011 |
| LOC105244656 | 0.00527 | -1.09019374 |
| Hlf | 0.62318 | 0.10856709 |
| Seh1l | 0.05148 | -0.41116232 |
| Pir | 0.47997 | 0.22881779 |
| Trim24 | 0.76227 | -0.05526271 |
| Gm36862 | 0.00144 | -1.20555712 |
| Gm16938 | 0.00213 | -1.67549074 |
| Esr1 | 0.13204 | -1.27558215 |
| Grk6 | 0.00962 | -0.6414374 |
| Sirt5 | 0.33799 | -0.19395675 |
| Wdr75 | 0.01621 | -0.77886385 |
| Fndc1 | 0.03185 | -0.81953232 |
| Gm20199 | 0.08021 | 1.05837019 |
| Pde4b | 0.64655 | -0.11919652 |
| Tmem65 | 0.00144 | 1.28279697 |
| Spred3 | 0.1452 | -0.74752579 |
| Rdh9 | 0.04011 | 0.73695934 |
| Acaca | 0.08834 | -0.5632999 |
| Psg23 | 0.23445 | 0.23677852 |
| C130046K22Rik | 0.00144 | -1.24613586 |
| Fads1 | 0.01538 | -2.69563073 |
| Gm35343 | 0.00268 | 0.90237276 |
| Trappc10 | 0.38294 | -0.30393982 |
| Dnajc18 | 0.85303 | 0.03165242 |
| Anxa1 | 0.32664 | -0.51580086 |
| Mrps2 | 0.78866 | -0.06929395 |
| Garem | 0.02417 | -0.45786802 |
| Sox21 | 0.00144 | -1.13577463 |
| Npc1 | 0.94995 | 0.01232117 |
| Ccl28 | 0.01688 | -1.03197481 |
| Ttc39aos1 | 0.18695 | 0.61197469 |
| Gm10413 | 0.00144 | 2.50037484 |
| LOC102641500 | 0.90905 | 0.04474532 |
| Nsg2 | 0.00144 | -1.26560808 |
| Gm5458 | 0.00144 | 2.61194876 |
| Eml3 | 0.01451 | -0.74897413 |
| Fam58b | 0.0075 | -0.86568354 |
| Colec12 | 0.11235 | 0.55776624 |
| Mcmdc2 | 0.02869 | 0.93150694 |
| Pcdh1 | 0.0021 | -1.10793124 |
| Kcnh6 | 0.30483 | -0.20868348 |
| Tspan18 | 0.47449 | -0.36425238 |
| Slc3a2 | 0.00144 | -1.23174693 |
| Aida | 0.16243 | -0.34622772 |
| Hook3 | 0.0133 | 0.94067079 |
| Gm29941 | 0.00144 | -1.29085005 |
| Lnp1 | 0.01538 | -1.12812796 |
| Ccdc88c | 0.05681 | -0.37693214 |
| A830049F12Rik | 0.20057 | -0.34489366 |
| Pald1 | 0.00459 | -0.69412662 |
| Ube2k | 0.11877 | 0.26988906 |
| Ncbp2 | 0.01927 | 0.69355164 |
| Defb1 | 0.00144 | -1.20555712 |
| Dhps | 0.00522 | -1.17381985 |
| Angpt2 | 0.00145 | -1.26219879 |
| Gm35340 | 0.00144 | -1.41392798 |
| Gm40881 | 0.01528 | -0.62735257 |
| Gm36816 | 0.00145 | -1.26219879 |
| Fam60a | 0.04019 | -0.83341593 |
| Hmgb2 | 0.12671 | -0.43206734 |
| Piga | 0.05504 | 1.01440802 |
| Jund | 0.00144 | -1.34464757 |
| Nap1l1 | 0.00489 | -1.04394795 |
| Gm26564 | 0.65969 | 0.1917822 |
| Gm32549 | 0.01326 | -1.56879576 |
| Slc25a36 | 0.58719 | -0.17121215 |
| Scn1a | 0.00144 | -1.29085005 |
| Olfr314 | 0.00152 | -1.25878908 |
| Olfr459 | 0.02728 | -0.8983632 |
| Olfr1349 | 0.01244 | -0.87999748 |
| Olfr857 | 0.00144 | -1.28898079 |
| Gmip | 0.05351 | 0.36147228 |
| Zfp27 | 0.05828 | 0.75592841 |
| Ptpn5 | 0.0085 | -1.50570917 |
| Gm32281 | 0.04356 | -2.11217381 |
| Ube2n | 0.73269 | 0.0527392 |
| Asrgl1 | 0.00144 | -1.19016999 |
| Mbp | 0.02359 | -0.93390357 |
| Gm16152 | 0.01241 | -1.02801808 |
| Fam83g | 0.67273 | -0.17513522 |
| Pramel7 | 0.09902 | -1.13774775 |
| Smyd4 | 0.75081 | -0.14565511 |
| AU040320 | 0.00144 | -1.81253706 |
| Adgrl3 | 0.16788 | -0.61742286 |
| Tle3 | 0.01042 | -0.61784932 |
| LOC102643260 | 0.73052 | -0.14389649 |
| 4930563F08Rik | 0.01134 | -1.02600084 |
| Cabin1 | 0.6941 | -0.11959955 |
| Slc1a2 | 0.00144 | -1.29746368 |
| LOC102642664 | 0.87655 | -0.03637768 |
| Vps33a | 0.23284 | -0.20389128 |
| Osbpl6 | 0.23847 | 0.47549254 |
| Aurkb | 0.19865 | 0.83871778 |
| Zfp622 | 0.00144 | -1.20616817 |
| Gm20604 | 0.00792 | 0.95349758 |
| Znrf2 | 0.15045 | 0.26917947 |
| Zic4 | 0.00144 | -1.28898079 |
| Tcf4 | 0.02259 | -0.92157284 |
| Slc7a8 | 0.00158 | -1.2446827 |
| Numb | 0.61381 | 0.14858748 |
| Rcor2 | 0.00714 | -1.08170417 |
| Fubp3 | 0.05377 | -0.85314247 |
| Lpin1 | 0.00793 | -0.80894015 |
| Baz1a | 0.03582 | -0.79997687 |
| E330014E10Rik | 0.00144 | -1.28116048 |
| Gm15418 | 0.07979 | -1.0023985 |
| Olfr1377 | 0.01672 | -0.84478901 |
| Fchsd2 | 0.97656 | 0.00611972 |
| Cables2 | 0.04294 | -0.4438907 |
| Gm12522 | 0.01536 | -1.32821982 |
| G630064G18Rik | 0.04466 | -1.33315114 |
| Cry1 | 0.00557 | -1.16347015 |
| Ctbp1 | 0.00144 | -1.11502172 |
| Arid4b | 0.64583 | -0.16395103 |
| Wdr25 | 0.13735 | 0.7703097 |
| Nupr1l | 0.06085 | -0.46803325 |
| Jmjd4 | 0.40739 | 0.26186893 |
| Pigk | 0.00717 | -0.75861195 |
| Gad1 | 0.14593 | -0.58239876 |
| Gm38448 | 0.00328 | -1.10633687 |
| Prss3 | 0.17613 | -0.7940029 |
| Rpl35a | 0.00175 | -0.84683856 |
| Gabarapl2 | 0.97964 | 0.00707994 |
| Tprgl | 0.42272 | -0.20491331 |
| Gimap8 | 0.00837 | 1.11646442 |
| Srrm4os | 0.85829 | -0.17828673 |
| Tmem59 | 0.09021 | 0.40515981 |
| Gm34975 | 0.00144 | 1.57274301 |
| Pdcd11 | 0.77319 | -0.04607042 |
| Brd8 | 0.38933 | -0.23140714 |
| Gm33676 | 0.01383 | -1.07643452 |
| Nrg1 | 0.4459 | -0.23333761 |
| 4930579G18Rik | 0.00145 | -1.18904624 |
| Lactbl1 | 0.00158 | -1.2446827 |
| Kcnh1 | 0.37276 | -0.28254165 |
| Dda1 | 0.11902 | -0.40281686 |
| Fbxl14 | 0.36269 | -0.17703794 |
| Smyd3 | 0.01385 | 0.8587738 |
| Cd300a | 0.01231 | -0.8999537 |
| Corin | 0.05033 | 0.60115572 |
| Slc26a1 | 0.50638 | -0.20019093 |
| Wbscr17 | 0.12257 | -1.39919861 |
| LOC102641065 | 0.08186 | -0.56944067 |
| BC024139 | 0.02802 | -0.85512225 |
| Slc9a8 | 0.01239 | -1.58249875 |
| Zfp870 | 0.2944 | -0.24302491 |
| Btg3 | 0.00812 | 0.7541197 |
| Lrp2 | 0.6823 | -0.20511205 |
| Ipmk | 0.89712 | 0.03404549 |
| E130308A19Rik | 0.32012 | -1.46475965 |
| Adpgk | 0.20931 | -0.34070891 |
| Rfc2 | 0.00539 | -0.69916023 |
| Kansl1l | 0.98076 | -0.00889557 |
| C330013J21Rik | 0.07044 | 0.85999734 |
| Mpped1 | 0.00144 | -1.26428512 |
| Tbck | 0.00773 | -0.84695968 |
| Gper1 | 0.00314 | -1.05780799 |
| Acap2 | 0.0531 | 1.44127643 |
| Ccl11 | 0.12232 | 0.38042518 |
| Adsl | 0.31427 | -0.19825986 |
| Ap3m2 | 0.54984 | -0.1276757 |
| Cacna2d1 | 0.0033 | 0.92209274 |
| Sfxn5 | 0.23477 | -0.61934146 |
| Gm16246 | 0.08354 | -2.71593849 |
| Ank3 | 0.91452 | -0.03103647 |
| Rdh18-ps | 0.04967 | 1.37075826 |
| Dcaf15 | 0.01185 | -0.6840697 |
| Alg11 | 0.03666 | 0.49940839 |
| Son | 0.00144 | 1.56486555 |
| Narf | 0.08474 | -0.34913761 |
| M6pr | 0.06411 | 0.78606236 |
| Pbrm1 | 0.00144 | 1.18524355 |
| Zfp207 | 0.07995 | -0.3629096 |
| Dpf1 | 0.00144 | -1.26560808 |
| Gm33542 | 0.00144 | -1.27699683 |
| 9230116N13Rik | 0.02194 | -0.86613882 |
| Cd24a | 0.55078 | 0.17268298 |
| 2700060E02Rik | 0.10922 | -0.27755251 |
| 9130019P16Rik | 0.4589 | -0.54120804 |
| Habp4 | 0.00524 | -0.80215973 |
| Gm36559 | 0.00144 | -1.29085005 |
| Agtrap | 0.1575 | -0.29704338 |
| Zfp764 | 0.20307 | 0.38459251 |
| Arl4c | 0.02664 | -0.47077748 |
| Timp3 | 0.03362 | 0.81517162 |
| Nudt4 | 0.0217 | -0.93656638 |
| Gm3120 | 0.00144 | -1.29085005 |
| Spocd1 | 0.05682 | -0.89689065 |
| Adam1a | 0.00411 | 1.52564943 |
| 4833427G06Rik | 0.00144 | -1.28116048 |
| Dsn1 | 0.1223 | 0.52264672 |
| Gm36243 | 0.00144 | -1.29864962 |
| 4930500A05Rik | 0.00144 | -1.26560808 |
| Ctnna3 | 0.00558 | 1.44945803 |
| Gm16430 | 0.00144 | -1.28898079 |
| E130307A14Rik | 0.00739 | -1.24104409 |
| Lbr | 0.18749 | 0.30805089 |
| 4930564I24Rik | 0.01433 | -0.83374634 |
| Ralgps2 | 0.04597 | -0.90881164 |
| Gm32964 | 0.02032 | -1.6985337 |
| Gm38462 | 0.00353 | -1.00997267 |
| Vti1a | 0.00562 | 1.03756755 |
| 4930405O22Rik | 0.19481 | 0.55053669 |
| Amigo3 | 0.884 | 0.11701165 |
| Gm33349 | 0.20899 | 0.46709294 |
| Gm32394 | 0.49601 | -0.47683964 |
| Gm16364 | 0.00144 | -1.20555712 |
| G430095P16Rik | 0.00144 | -1.28898079 |
| Lxn | 0.1163 | -0.36312819 |
| Eif1 | 0.11788 | -0.42128077 |
| Carm1 | 0.0443 | -0.484816 |
| Rhobtb1 | 0.62544 | -0.08499285 |
| Srcap | 0.94623 | 0.01384256 |
| Gm8327 | 0.70687 | 0.42835357 |
| LOC105247377 | 0.26062 | 0.44714221 |
| Brox | 0.0047 | -0.98401328 |
| Camk2d | 0.00144 | 1.57278171 |
| LOC102642140 | 0.55517 | 0.2764514 |
| Gm36208 | 0.07852 | -0.8482538 |
| Scfd2 | 0.00276 | -1.12355196 |
| Mak16 | 0.07701 | 0.36028584 |
| Gm13154 | 0.00144 | 2.75532926 |
| Rock1 | 0.02012 | 0.86435794 |
| Gm12786 | 0.00202 | -1.14316776 |
| Papola | 0.32464 | 0.27153262 |
| Lgals12 | 0.10937 | -0.92693771 |
| Rpl39 | 0.01778 | -0.638447 |
| Sass6 | 0.01083 | 0.6641253 |
| Sacs | 0.32654 | 0.27740969 |
| Bzw1 | 0.00503 | 0.748614 |
| Gm10413 | 0.0027 | 2.23573288 |
| Faf2 | 0.04369 | -0.37002419 |
| 2310075K07Rik | 0.43539 | -0.29971322 |
| Mrpl27 | 0.81605 | 0.07842334 |
| Grin3a | 0.1437 | -0.62134758 |
| Itfg2 | 0.44717 | -0.15682883 |
| Ccnd3 | 0.00516 | -1.07001368 |
| Casq1 | 0.11451 | -0.59231432 |
| Pxdn | 0.19771 | -0.23287584 |
| Notch3 | 0.00214 | -0.94775664 |
| Nr6a1 | 0.17475 | -0.24658446 |
| Ddx55 | 0.87782 | -0.06513821 |
| Usp15 | 0.05368 | 1.41856768 |
| Map6 | 0.00249 | -1.17452065 |
| Gm17202 | 0.00969 | -0.99774049 |
| Prkar2a | 0.02807 | -0.43214549 |
| Rnf166 | 0.00711 | -0.72647888 |
| Gm16049 | 0.95362 | 0.01789339 |
| Mylk2 | 0.00144 | -1.24173194 |
| Usp48 | 0.19621 | -0.57812095 |
| Pigt | 0.00221 | -1.11814941 |
| Nt5m | 0.00144 | -1.32766452 |
| Cp | 0.02557 | 1.05723739 |
| Dzip1l | 0.06102 | -0.37731443 |
| Stap2 | 0.08485 | 0.35580634 |
| Lrriq3 | 0.00144 | -1.25927895 |
| Irak4 | 0.81023 | -0.05656664 |
| Ftl1 | 0.00144 | -1.04666309 |
| Gm30752 | 0.5455 | 0.23796269 |
| Teddm2 | 0.00436 | -0.99450396 |
| Ccdc157 | 0.81229 | -0.11621599 |
| Stmn3 | 0.00144 | -1.33579708 |
| LOC102643083 | 0.0029 | -1.20702967 |
| Gm30661 | 0.05087 | -0.98251086 |
| Zfp385a | 0.23505 | -0.225735 |
| Braf | 0.01602 | 0.5701054 |
| Zswim4 | 0.0142 | -0.63511188 |
| Uimc1 | 0.07879 | -0.43554175 |
| Bcl7c | 0.00144 | -1.25118919 |
| Map3k9 | 0.59609 | -0.36562051 |
| Myo6 | 0.02603 | 1.06201194 |
| Gm35688 | 0.00144 | -1.27165604 |
| Ces1d | 0.53841 | 0.28236905 |
| Zfp36l1 | 0.04737 | -0.37834016 |
| Gm11418 | 0.39097 | -0.61197591 |
| Tsn | 0.15514 | 0.29316482 |
| Snrpa | 0.012 | -1.0598408 |
| Mical3 | 0.08634 | -0.32746147 |
| Fam155a | 0.00551 | -1.37916784 |
| Kif5b | 0.3389 | 0.26855288 |
| Klhdc8a | 0.97587 | 0.01699493 |
| Olfr153 | 0.19291 | 0.82510975 |
| Slc7a2 | 0.80845 | -0.09255029 |
| Olfr1286 | 0.00163 | -1.21015905 |
| Olfr806 | 0.05074 | -1.35026667 |
| Olfr1037 | 0.00144 | -1.28625228 |
| Olfr1162 | 0.0027 | -1.11012596 |
| Olfr1123 | 0.03533 | 1.25896637 |
| LOC105246799 | 0.00158 | -1.2446827 |
| Ube2l3 | 0.39819 | 0.2390167 |
| Rcn3 | 0.01031 | -0.85374281 |
| Paip2b | 0.24077 | 0.18682761 |
| Blmh | 0.02158 | -0.48466143 |
| Prkag1 | 0.01078 | -0.81055901 |
| Akt1s1 | 0.02976 | -0.88675583 |
| Efhd2 | 0.00623 | -0.65909024 |
| Hnrnpl | 0.003 | -0.73172617 |
| Psmd2 | 0.0022 | -0.81559408 |
| E330021D16Rik | 0.01789 | -0.93558464 |
| Man2a2 | 0.00356 | -1.49319452 |
| Olfr1199 | 0.00144 | -1.28116048 |
| 1600002H07Rik | 0.04026 | -0.47144206 |
| Cops5 | 0.50433 | 0.12413608 |
| Wdr37 | 0.00232 | 1.08017745 |
| Mogat1 | 0.00144 | -1.3348153 |
| Gpr149 | 0.00533 | -1.24292841 |
| Gm35409 | 0.03639 | -0.7199297 |
| LOC102634904 | 0.00439 | 1.17877815 |
| Synj1 | 0.00144 | -1.26560808 |
| Trp53bp1 | 0.00224 | -0.80020231 |
| Hspd1 | 0.00144 | 1.44088412 |
| Gltscr2 | 0.00191 | -0.86223417 |
| St18 | 0.07258 | -2.44550715 |
| Gm34752 | 0.01544 | -0.85697914 |
| Gm1976 | 0.78044 | -0.08252812 |
| Ddx17 | 0.00145 | -1.37792666 |
| Btg1 | 0.00783 | -0.6308771 |
| Tacstd2 | 0.92827 | -0.04119937 |
| H3f3a | 0.3085 | 0.17979253 |
| Harbi1 | 0.21704 | 0.21548072 |
| Eif4b | 0.02538 | -0.4778425 |
| BC027231 | 0.15927 | 0.31481896 |
| 4922502B01Rik | 0.8951 | 0.0221457 |
| Ttll1 | 0.4637 | -0.25269637 |
| Eif2s3y | 0.00271 | 1.51864083 |
| C920006O11Rik | 0.0113 | -0.60751295 |
| Znhit6 | 0.26151 | 0.34056421 |
| Wdr37 | 0.32376 | -0.44701231 |
| Gas5 | 0.73059 | -0.09366145 |
| Malt1 | 0.71711 | -0.13033068 |
| Ssr1 | 0.5745 | -0.11363854 |
| Rsf1 | 0.01375 | 1.25179071 |
| Arrdc3 | 0.01911 | 0.92629016 |
| Hnrnph2 | 0.04866 | 0.65383834 |
| Gm35973 | 0.00691 | -0.89516328 |
| Rmi1 | 0.09099 | 0.45965493 |
| Fnd3c2 | 0.0151 | -1.57415534 |
| Tcp10a | 0.00144 | -1.25118919 |
| Bcl7c | 0.00324 | -0.81826963 |
| Atp5j2 | 0.01879 | -0.77136285 |
| Gm5891 | 0.00521 | -1.05862264 |
| Wdr92 | 0.05139 | 0.9717316 |
| Gm30478 | 0.04205 | -2.36380298 |
| Gm10439 | 0.00144 | -1.29085005 |
| C330007P06Rik | 0.00195 | 1.34308912 |
| Gm30313 | 0.00144 | -1.29085005 |
| Gm31987 | 0.32776 | -0.59253586 |
| Kazn | 0.95168 | -0.0144792 |
| Pcgf3 | 0.10152 | 0.32231634 |
| Dhx36 | 0.83605 | 0.05250904 |
| Decr2 | 0.34598 | 0.24331493 |
| Alb | 0.00508 | -2.86007921 |
| Fam120c | 0.29601 | 0.27351492 |
| Dennd4a | 0.0023 | 1.11636371 |
| Atad2b | 0.00162 | 1.20526346 |
| Opa1 | 0.0099 | 0.79103922 |
| Upf3b | 0.00166 | -1.36066784 |
| Gm29766 | 0.00144 | -1.37728621 |
| Svopl | 0.20977 | -0.38661184 |
| Rbm38 | 0.00447 | -1.23300721 |
| Plekhg1 | 0.00164 | 0.97542403 |
| Gpr75 | 0.25486 | 1.06822699 |
| Mpv17l | 0.53428 | -0.15662492 |
| Gm31727 | 0.00144 | -1.27165604 |
| Sdc1 | 0.00522 | -1.10129424 |
| Tarsl2 | 0.01822 | -0.59570292 |
| Lin28a | 0.00229 | -1.71801697 |
| Plau | 0.00181 | -1.22749089 |
| Foxn3 | 0.00144 | -1.11614834 |
| Ube4a | 0.55153 | 0.16356305 |
| Gm19327 | 0.10564 | -0.35622865 |
| 4933424M12Rik | 0.03418 | -0.9383227 |
| Gm32017 | 0.9058 | 0.08446355 |
| Gm34368 | 0.00144 | -1.30713006 |
| Gm38559 | 0.00145 | -1.33072139 |
| Gm32431 | 0.48204 | -0.44384133 |
| Gm36855 | 0.07356 | 0.65783229 |
| Gm34349 | 0.00144 | -1.30229687 |
| Cacng8 | 0.48967 | -0.39960187 |
| Brwd3 | 0.00154 | 1.33867894 |
| Pcnx | 0.06686 | -0.3964394 |
| LOC102638002 | 0.13386 | -1.45712798 |
| Rps19 | 0.32575 | -0.39052935 |
| Focad | 0.34716 | -0.47530861 |
| Gm35358 | 0.00144 | 4.3229931 |
| Il16 | 0.20975 | -0.60215023 |
| Mtfmt | 0.03658 | -2.20513454 |
| Gm38444 | 0.02977 | -0.5962688 |
| Phc2 | 0.00149 | -1.22582153 |
| Panx1 | 0.50448 | -0.19570903 |
| Dgkh | 0.00145 | -1.26219879 |
| Ercc6 | 0.00336 | -1.20864476 |
| Iqsec3 | 0.28173 | -0.81272705 |
| Cdhr3 | 0.02269 | -1.44440992 |
| Ddx19a | 0.55669 | 0.14988798 |
| Wdr20 | 0.13021 | -0.80350734 |
| Ptchd2 | 0.12061 | -2.9423326 |
| 4932430A15Rik | 0.06162 | -0.34427718 |
| Usp12 | 0.24202 | 0.24077868 |
| Gm32621 | 0.11037 | -1.06076958 |
| Gm31704 | 0.00145 | -1.22376446 |
| Rai1 | 0.93639 | -0.03348851 |
| Gm16193 | 0.00144 | -1.5716978 |
| Vwa1 | 0.96887 | -0.01498973 |
| Gm31805 | 0.39481 | -0.69453138 |
| Zbed4 | 0.06949 | -1.23452648 |
| Eprs | 0.09214 | -0.45398553 |
| Lppos | 0.03542 | -0.62073028 |
| Nsd1 | 0.72509 | 0.05565762 |
| Apoa4 | 0.00385 | -1.14134209 |
| Sst | 0.00418 | -1.84228383 |
| Mllt6 | 0.00538 | -2.04815278 |
| Olfr707 | 0.06028 | -2.26045324 |
| Olfr75-ps1 | 0.99832 | -0.00085951 |
| Olfr45 | 0.27445 | -0.41619398 |
| Olfr1247 | 0.01288 | -1.49349134 |
| Olfr1294 | 0.19421 | 1.26954089 |
| Gm30816 | 0.49218 | -0.21335333 |
| Gm32739 | 0.01866 | -1.45248974 |
| Gm32280 | 0.01318 | 2.27806031 |
| Atp6v0b | 0.00725 | -0.86069237 |
| Acsm2 | 0.00144 | -1.29746368 |
| Gm19554 | 0.00144 | -1.28116048 |
| Plcl2 | 0.01336 | -0.83591508 |
| Polr3k | 0.78984 | 0.05737821 |
| Pdha1 | 0.00725 | 1.18290136 |
| Gm36356 | 0.80194 | 0.10164056 |
| Gspt1 | 0.0032 | -0.89782838 |
| Gm21284 | 0.00144 | -1.31495282 |
| Ago3 | 0.05767 | 0.61254608 |
| Gm13133 | 0.0028 | -1.16569658 |
| Dexi | 0.54607 | 0.17248579 |
| Gm39307 | 0.00668 | -0.93852514 |
| Gm33318 | 0.38053 | 0.14019189 |
| Gm28321 | 0.07605 | -1.35296456 |
| St18 | 0.00144 | -1.27165604 |
| AW551984 | 0.00158 | -1.22727468 |
| Gm34483 | 0.00144 | -1.31495282 |
| Clmn | 0.00145 | -1.2388121 |
| Setd5 | 0.0795 | 1.10752951 |
| Gm12359 | 0.49694 | -0.20794578 |
| 4930546K05Rik | 0.00144 | -1.28116048 |
| Gm13964 | 0.03315 | -1.32660737 |
| Gm36221 | 0.33754 | -0.49514228 |
| Ddx26b | 0.0018 | 1.34196501 |
| Tspan15 | 0.04819 | -0.56094156 |
| Mir99ahg | 0.97254 | -0.01605513 |
| Hrasls | 0.15351 | 0.28925279 |
| Map4 | 0.79595 | 0.05735179 |
| Hpcal4 | 0.13559 | -0.55798917 |
| Tspan11 | 0.00366 | -1.1352771 |
| Hipk1 | 0.01793 | 0.71700573 |
| Prmt8 | 0.15508 | -1.18678434 |
| Pcyt2 | 0.0363 | -0.53333265 |
| Gm10530 | 0.00832 | -1.10818293 |
| Gm10612 | 0.00144 | -1.47728193 |
| Clec12a | 0.01521 | -0.67652603 |
| Dlgap5 | 0.0037 | -0.94322169 |
| Hcar1 | 0.00188 | -1.37510274 |
| Gm19343 | 0.00149 | -1.22582153 |
| Btbd7 | 0.12747 | 0.47167917 |
| Cd19 | 0.19255 | 0.5311409 |
| Stx11 | 0.14467 | 0.32202792 |
| 3110052M02Rik | 0.06025 | 0.69227866 |
| F730016J06Rik | 0.03992 | -1.06933101 |
| Mmp8 | 0.43268 | -0.3879788 |
| Zc3h12c | 0.00963 | -0.64080437 |
| Usp22 | 0.15431 | -0.28912432 |
| Tnfrsf9 | 0.23566 | -0.84027739 |
| Sash3 | 0.00623 | -1.03820359 |
| Selplg | 0.00823 | -1.03303644 |
| Fpr2 | 0.15525 | -0.29089128 |
| Cep83os | 0.03988 | 0.65992872 |
| Osgin2 | 0.13086 | 0.38229951 |
| Asnsd1 | 0.2732 | -0.17487565 |
| Met | 0.63995 | -0.28004468 |
| Zfp518b | 0.92535 | -0.01665448 |
| C5ar1 | 0.03822 | -0.66628109 |
| Il10ra | 0.01114 | -0.8860938 |
| Clcn7 | 0.02847 | -0.58663974 |
| Osm | 0.60725 | 0.27613351 |
| Cmpk2 | 0.90786 | -0.03179501 |
| Eif3a | 0.01597 | 0.92758228 |
| Wipf2 | 0.00202 | -1.03143797 |
| Nrp1 | 0.15893 | 0.38273633 |
| Apoa5 | 0.00144 | -1.28898079 |
| Zfp462 | 0.16757 | 0.54869294 |
| Hsp90b1 | 0.03104 | 0.68174988 |
| Hspb7 | 0.03294 | -0.63621429 |
| Cct8 | 0.5784 | -0.13699825 |
| I730030J21Rik | 0.021 | -0.52013798 |
| Cbx5 | 0.53443 | -0.09493968 |
| Slc11a2 | 0.04465 | -0.7217796 |
| Brwd1 | 0.72038 | -0.05975877 |
| Sugp1 | 0.76105 | -0.1866469 |
| Ftsj3 | 0.10176 | -0.42294893 |
| Cnot1 | 0.01673 | -0.50668287 |
| Smarcal1 | 0.67955 | -0.15810065 |
| Hnrnpm | 0.01333 | -1.10035356 |
| Chuk | 0.00762 | 0.68869024 |
| Adck3 | 0.09322 | -0.63004741 |
| Fis1 | 0.00326 | -0.86010795 |
| Gm11211 | 0.5277 | -0.2637636 |
| Zfp260 | 0.15662 | -0.44200725 |
| Yars | 0.79527 | -0.11588584 |
| Rab36 | 0.01535 | -1.42444313 |
| Gm32575 | 0.46948 | -0.82167354 |
| Pttg1ip | 0.00144 | -1.39485581 |
| Gm20670 | 0.0281 | -0.95564746 |
| Gm33936 | 0.00223 | -1.16781173 |
| Gm31555 | 0.02792 | -0.91509798 |
| Ndufb2 | 0.00149 | -1.01416418 |
| Gm35229 | 0.00145 | -1.2388121 |
| Gm30416 | 0.00149 | -1.25408 |
| Gm34882 | 0.00261 | -1.11284335 |
| Gm32023 | 0.00231 | -1.26219879 |
| Gm36086 | 0.00144 | -1.26560808 |
| Nnt | 0.00144 | -1.1947002 |
| Gm20684 | 0.05012 | -0.94393716 |
| Gm34799 | 0.00158 | -1.2446827 |
| Crnkl1 | 0.01564 | 0.49822391 |
| Gm15569 | 0.00144 | -1.40395392 |
| Gm29821 | 0.00144 | -1.27165604 |
| Gm32872 | 0.00144 | -1.29085005 |
| Daxx | 0.25933 | -0.47536242 |
| Gm33693 | 0.04439 | -1.95342403 |
| Gm35650 | 0.0292 | -1.5206263 |
| 1700065J18Rik | 0.00144 | -1.29085005 |
| 1700095A21Rik | 0.00144 | -1.43713248 |
| 1110032A03Rik | 0.60791 | -0.1009151 |
| LOC102632156 | 0.82178 | -0.23588167 |
| LOC102642028 | 0.00144 | -1.75660353 |
| Rp1l1 | 0.01742 | -0.73396746 |
| Grb14 | 0.00144 | -1.26560808 |
| Sncb | 0.12151 | -1.20630328 |
| Pard3b | 0.00583 | 0.7426522 |
| Asxl3 | 0.00144 | 2.18490437 |
| Gm30818 | 0.00144 | -1.31884045 |
| Gm36116 | 0.0055 | -1.76227476 |
| Lifr | 0.91853 | -0.01926203 |
| Capn1 | 0.0017 | -0.98033146 |
| Gm31630 | 0.00144 | -1.67731314 |
| Prokr1 | 0.00172 | -1.21364624 |
| Armc8 | 0.1569 | 0.27797988 |
| Zfp608 | 0.01903 | -0.52943588 |
| Oprl1 | 0.52257 | -0.25194636 |
| Prdm2 | 0.5569 | -0.14142741 |
| Pax6 | 0.00144 | -1.45544074 |
| Ebf1 | 0.3797 | -0.22089835 |
| Bsn | 0.00144 | -1.28116048 |
| Hectd1 | 0.11344 | 0.27559953 |
| Nras | 0.07344 | 0.58287212 |
| Pdk1 | 0.85506 | 0.03590206 |
| Gm29926 | 0.20029 | -0.61662296 |
| Scml4 | 0.03055 | 1.437517 |
| D11Wsu47e | 0.15255 | -0.44313121 |
| Nbea | 0.00144 | -1.29085005 |
| Zfp746 | 0.57391 | 0.11872858 |
| Scoc | 0.08838 | -1.01748914 |
| Ilf3 | 0.00144 | -1.12411114 |
| Setd2 | 0.00144 | 1.46420121 |
| Il22ra1 | 0.00144 | 4.77169587 |
| Rngtt | 0.13193 | 0.50235512 |
| Golga7 | 0.09667 | 0.30775825 |
| Gm34798 | 0.01921 | -1.26688261 |
| Pou4f1 | 0.66082 | -0.22574652 |
| Fam167a | 0.01289 | 0.83924047 |
| Gm30684 | 0.00877 | -1.43788683 |
| Npy5r | 0.00144 | -1.28898079 |
| Nedd1 | 0.05143 | 0.44148078 |
| Fam64a | 0.21224 | -0.72644316 |
| Phf23 | 0.03566 | -0.58028548 |
| Polk | 0.00307 | 1.07208735 |
| Mybbp1a | 0.83932 | -0.06126595 |
| Paip1 | 0.08548 | 0.35371398 |
| Esco1 | 0.01289 | 2.0921399 |
| Cdc23 | 0.40735 | 0.14695452 |
| Fam110a | 0.00144 | -1.35523346 |
| Rspry1 | 0.26026 | -0.46542877 |
| Slc30a6 | 0.20317 | -0.38715769 |
| Flrt2 | 0.52826 | 0.21389127 |
| Rtn2 | 0.35638 | -0.60348239 |
| Edc3 | 0.81046 | -0.05617866 |
| Siglecf | 0.01741 | -1.44766324 |
| Wnt7b | 0.00418 | -0.85972578 |
| Lrrc40 | 0.31342 | -0.39110453 |
| Aatk | 0.00144 | -1.29085005 |
| Itgax | 0.00144 | -1.28116048 |
| LOC102642586 | 0.16974 | -1.7131636 |
| Bptf | 0.47945 | 0.23624253 |
| Mphosph8 | 0.00205 | 2.28835063 |
| Gm33035 | 0.00144 | -1.29085005 |
| Gm6999 | 0.00144 | -1.28116048 |
| Gm36320 | 0.00416 | -1.82432208 |
| Alg13 | 0.0098 | 1.35219145 |
| Pold4 | 0.00178 | -0.96815324 |
| Nrn1 | 0.0651 | 0.67664651 |
| Apol11b | 0.20237 | -0.8500727 |
| Btc | 0.70408 | 0.49674795 |
| Egf | 0.04251 | 0.98000643 |
| Dennd5b | 0.02822 | -0.68397232 |
| Ubxn2a | 0.07203 | 0.42971686 |
| LOC102632885 | 0.0281 | -0.95564746 |
| Slc16a12 | 0.00323 | 1.85870662 |
| Rps6ka2 | 0.00235 | -0.82687775 |
| Gm26838 | 0.00469 | -1.36425238 |
| Chm | 0.00155 | 1.49595836 |
| Taf4b | 0.0322 | -0.55295912 |
| Dclre1b | 0.00322 | 0.97696488 |
| Pck2 | 0.17426 | -0.29545264 |
| Cnot4 | 0.00997 | 0.71813002 |
| Apcdd1 | 0.01785 | -0.59960252 |
| Hs1bp3 | 0.00388 | -0.98141659 |
| Scaf8 | 0.95295 | 0.03116262 |
| Stambpl1 | 0.00145 | -1.22376446 |
| Gm16189 | 0.00837 | -1.26913726 |
| Mecom | 0.17769 | -0.80914041 |
| Myl4 | 0.00144 | -1.31495282 |
| Suv420h1 | 0.80753 | 0.07120206 |
| Nup88 | 0.05568 | -1.02797416 |
| Gm32679 | 0.04824 | -0.86696801 |
| Lmo7 | 0.74629 | -0.12329847 |
| Mmp16 | 0.18379 | -0.61472297 |
| Mier1 | 0.5014 | -0.34390295 |
| Zfp248 | 0.00144 | -1.26934956 |
| Gm13481 | 0.53886 | -0.5197149 |
| Gm30931 | 0.89738 | -0.05586665 |
| Iffo1 | 0.48665 | -0.28606625 |
| Txlng | 0.00149 | 1.3955895 |
| Atp10a | 0.0098 | -0.99034031 |
| Gm32880 | 0.00201 | -1.28029898 |
| Gm30411 | 0.00273 | -1.28366646 |
| Rhob | 0.00158 | -0.87773832 |
| Nasp | 0.21312 | -0.58113062 |
| Ei24 | 0.95809 | -0.01812641 |
| Evi5 | 0.01058 | 1.25111582 |
| Dhodh | 0.00144 | -1.30017714 |
| Pdpr | 0.00197 | -1.33421599 |
| Wnt7a | 0.00177 | -1.65679785 |
| Sdhaf2 | 0.01524 | 1.44814479 |
| Etnk1 | 0.07461 | -0.48525537 |
| Tmem154 | 0.3392 | 0.35005854 |
| Slx4ip | 0.02166 | 0.74326285 |
| Gm31365 | 0.00144 | -1.39803146 |
| Atrnl1 | 0.00144 | 1.00741443 |
| Ncf2 | 0.57761 | -0.11765553 |
| Son | 0.31568 | -0.18588523 |
| Repin1 | 0.00824 | -0.8552118 |
| Map2 | 0.88935 | 0.06902168 |
| Gm33682 | 0.13932 | -0.61232046 |
| Dicer1 | 0.39934 | 0.19726468 |
| Nlk | 0.38393 | -0.4697145 |
| Fam134c | 0.01023 | -0.70725923 |
| Smarcc1 | 0.02802 | -0.54857277 |
| Ahcyl1 | 0.0148 | -0.5042727 |
| Gm35248 | 0.00144 | -1.69222902 |
| Jakmip3 | 0.00269 | -1.29769493 |
| Gm15736 | 0.04031 | -1.58430238 |
| Cxadr | 0.09687 | 0.36122598 |
| Tmpo | 0.00144 | 1.45093304 |
| Hip1r | 0.04437 | -0.40870181 |
| Hook3 | 0.00144 | 1.42687511 |
| Ppp1r10 | 0.02198 | -1.10542783 |
| Cetn3 | 0.0023 | 1.30238209 |
| Ibtk | 0.00144 | -1.31194506 |
| Hnrnpc | 0.00498 | -0.74982633 |
| Tbc1d4 | 0.0611 | -0.5057869 |
| Dpy19l1 | 0.428 | 0.19148735 |
| Ndufaf4 | 0.00144 | 2.09180497 |
| Sclt1 | 0.04997 | 1.2549057 |
| Pdlim5 | 0.00144 | 2.20944933 |
| Usp10 | 0.00144 | -1.27992547 |
| Snx13 | 0.10697 | 0.43927308 |
| Eif3k | 0.05196 | -0.90961238 |
| Ppp1r37 | 0.42294 | -0.59992024 |
| Dnmbp | 0.92975 | -0.02980144 |
| Marcks | 0.0447 | -0.74394924 |
| Gm11713 | 0.00164 | 1.29713973 |
| Alkbh1 | 0.02843 | 0.59392761 |
| Gm31814 | 0.12427 | -1.15280259 |
| Gm12610 | 0.00144 | -1.29049509 |
| Platr11 | 0.2251 | -0.73444999 |
| Fam184b | 0.01077 | -1.55536642 |
| Hdac4 | 0.13685 | -0.4045898 |
| 1810041L15Rik | 0.22217 | -0.62032026 |
| Tcaf2 | 0.00651 | 0.83067447 |
| Pcyt1a | 0.00499 | -0.77593571 |
| Gm15663 | 0.02172 | -0.71711569 |
| Rexo4 | 0.34981 | 0.29872163 |
| Cacna1e | 0.00144 | -1.29746368 |
| Gm20125 | 0.99409 | 0.01043638 |
| Eps15 | 0.99026 | -0.0042504 |
| Zhx2 | 0.00144 | -1.4049326 |
| Atg4c | 0.81357 | -0.08935481 |
| Mpp3 | 0.26342 | 0.29536335 |
| Zranb2 | 0.90651 | 0.09112898 |
| Pram1 | 0.00144 | -1.25118919 |
| Epb4.1 | 0.20128 | 0.43580951 |
| Mark1 | 0.02012 | 0.7514936 |
| Tmem170b | 0.29425 | -0.25154021 |
| Tapt1 | 0.04732 | 0.77063827 |
| Arnt | 0.0052 | 1.42727259 |
| Mis18bp1 | 0.00144 | -1.2383131 |
| Stard7 | 0.82929 | 0.05303239 |
| Slc25a53 | 0.01264 | 0.77281515 |
| Zmym3 | 0.00144 | -1.30713006 |
| Tspan15 | 0.00209 | -0.97077003 |
| Helz | 0.74076 | 0.08367898 |
| Ube2d1 | 0.73111 | -0.08815987 |
| Pfkfb2 | 0.35513 | -0.20231827 |
| Smoc1 | 0.00525 | -0.83341773 |
| Exo5 | 0.2578 | -0.4744698 |
| Prdm4 | 0.02145 | 0.58837278 |
| Herc3 | 0.74342 | -0.06920954 |
| Zfp871 | 0.46755 | 0.1265044 |
| Whsc1 | 0.00144 | 1.06248514 |
| Cnksr2 | 0.36794 | 0.37236479 |
| Trp53inp2 | 0.0053 | -0.93007657 |
| BC027231 | 0.00561 | 1.47619022 |
| Cngb3 | 0.00144 | -1.30017714 |
| Cfp | 0.05671 | -0.34323875 |
| Trib2 | 0.00258 | -1.20410499 |
| B3gat1 | 0.00144 | -1.27165604 |
| Tsga10 | 0.87284 | 0.1005797 |
| Adam4 | 0.31443 | 0.32504178 |
| Cirbp | 0.20982 | -0.26483152 |
| Ppm1n | 0.08244 | -1.16268753 |
| Zfp84 | 0.1683 | 0.23928084 |
| Ociad2 | 0.49919 | -0.15678165 |
| Dicer1 | 0.01282 | -0.5540643 |
| Amz2 | 0.10982 | 0.75166039 |
| Anapc1 | 0.48944 | 0.31404659 |
| Ercc5 | 0.07772 | -0.38923412 |
| Psmb4 | 0.56627 | -0.24643837 |
| Gpatch2 | 0.70635 | 0.09443688 |
| Sorcs2 | 0.02622 | -0.75585299 |
| Smap2 | 0.14997 | -0.37540048 |
| Dlgap1 | 0.03619 | -0.78004423 |
| Rasa2 | 0.00166 | 1.45821058 |
| Dusp18 | 0.61436 | -0.26882743 |
| Acot6 | 0.00229 | -1.73512867 |
| Hars2 | 0.02319 | 0.85495135 |
| Vps13c | 0.12704 | -0.44395149 |
| Rcbtb1 | 0.00717 | -1.0591794 |
| Nanos1 | 0.42179 | 0.37957571 |
| Cnpy1 | 0.00145 | -1.25646236 |
| Dmd | 0.1652 | 0.3576512 |
| Dym | 0.01191 | -0.63913343 |
| Trio | 0.00417 | -0.81870647 |
| Mccc1os | 0.73832 | 0.1180268 |
| Tada2b | 0.75392 | -0.05217201 |
| Chrdl1 | 0.25109 | -0.69695592 |
| Hoxd8 | 0.98802 | 0.00756694 |
| Cxcl1 | 0.0123 | 1.26512243 |
| Grtp1 | 0.55837 | -0.22136281 |
| Slitrk4 | 0.77976 | -0.22180637 |
| Tmem194b | 0.25894 | 0.51128461 |
| Tfe3 | 0.00249 | -0.82574998 |
| Gm31344 | 0.07711 | -1.20497096 |
| Ubr3 | 0.01286 | -1.67482789 |
| Nrg1 | 0.96397 | 0.00750444 |
| Dnajb9 | 0.00144 | 1.22886891 |
| Dcaf10 | 0.16234 | 0.25814337 |
| Actn1 | 0.56207 | -0.09302694 |
| Nudcd3 | 0.00144 | -1.29049509 |
| Tbc1d9 | 0.01416 | 1.02800312 |
| Bod1l | 0.00185 | 1.21648527 |
| Sowaha | 0.57614 | -0.45268487 |
| Cntnap2 | 0.91793 | 0.15560372 |
| Gm32633 | 0.00144 | -1.29051721 |
| Jmy | 0.41459 | 0.1345095 |
| Herpud1 | 0.33337 | 0.36912331 |
| Myo9a | 0.05996 | 0.42943253 |
| Srpk2 | 0.17816 | 0.52289906 |
| Sntb2 | 0.01137 | -0.99060319 |
| Speer4cos | 0.13616 | -1.16737189 |
| Hnmt | 0.07218 | 0.40835474 |
| Pcdh7 | 0.40303 | 0.18880881 |
| Ascc1 | 0.01094 | -0.59558644 |
| Dgkz | 0.00315 | -0.82209455 |
| Tmx4 | 0.00144 | -1.06905493 |
| Reps2 | 0.00368 | -1.97118478 |
| Medag | 0.27004 | 0.41937149 |
| Rasl10b | 0.29465 | -0.38464547 |
| Nefl | 0.00144 | -1.51183838 |
| Casq2 | 0.05983 | -0.42956229 |
| Commd3 | 0.29972 | -0.21040017 |
| Eid2 | 0.38375 | -0.21402819 |
| Kcnj12 | 0.00243 | -0.98660656 |
| Espl1 | 0.02086 | -0.64552535 |
| Hnrnpr | 0.07776 | -0.73596711 |
| Ubxn8 | 0.47611 | 0.1278587 |
| Gm20752 | 0.0145 | 0.64836941 |
| Tmem167 | 0.05107 | 1.19893756 |
| Tbx2 | 0.0734 | -0.39018049 |
| Supt20 | 0.23164 | -0.28448556 |
| Ptchd4 | 0.00144 | -1.23532597 |
| Ptp4a1 | 0.75206 | 0.08599212 |
| 2610507B11Rik | 0.00144 | -1.03432485 |
| Dlx2 | 0.30732 | -0.76484098 |
| Stxbp4 | 0.87844 | -0.05724349 |
| Adhfe1 | 0.00145 | 1.66783494 |
| Lpcat2 | 0.75838 | -0.10079347 |
| Ddn | 0.4541 | -0.40433124 |
| Atl3 | 0.81902 | -0.07461916 |
| Cpeb4 | 0.00154 | 0.93897629 |
| Tmem26 | 0.00383 | -1.13029182 |
| 2900097C17Rik | 0.28281 | 0.199236 |
| AI504432 | 0.3047 | -0.53022538 |
| Polr3d | 0.52934 | -0.13770343 |
| Sf3b1 | 0.53323 | -0.10787446 |
| Cul1 | 0.00144 | 1.42940564 |
| Fam109b | 0.0035 | -1.03754725 |
| Zfp60 | 0.5885 | 0.13137696 |
| Frem1 | 0.24704 | -0.52118117 |
| Trpm7 | 0.20699 | 0.37996138 |
| Glmp | 0.00144 | -1.04248027 |
| Hebp2 | 0.59496 | -0.11365514 |
| Terf2ip | 0.00486 | 0.76171571 |
| Erbb2 | 0.00148 | -1.05481351 |
| Rfc5 | 0.0114 | -0.73988197 |
| Prps2 | 0.0377 | 0.69582401 |
| Smim20 | 0.01923 | -0.72325783 |
| Gm35739 | 0.16205 | -0.59553212 |
| Cblb | 0.04705 | -0.66497014 |
| Aif1l | 0.0019 | -0.9356591 |
| Clptm1l | 0.01529 | -0.66434731 |
| Gm41724 | 0.0018 | -1.21153109 |
| Zfp422 | 0.01354 | 0.58870032 |
| Gm13373 | 0.00144 | -1.24613586 |
| Aldh1l2 | 0.0024 | 2.17980843 |
| Chd9 | 0.05296 | 0.8215163 |
| Arfgef2 | 0.02676 | 0.72967312 |
| F8a | 0.00731 | -0.98178778 |
| Saraf | 0.00571 | -0.75149582 |
| D19Bwg1357e | 0.78508 | 0.05659366 |
| Gldc | 0.24501 | 0.39727754 |
| Spint1 | 0.50331 | 0.27851555 |
| Alx1 | 0.07632 | -0.80538495 |
| Nup155 | 0.01533 | -0.74032038 |
| Arhgap5 | 0.00165 | 0.91727204 |
| Tnrc6a | 0.55512 | 0.14709564 |
| Sema3a | 0.21776 | 1.05983702 |
| Zranb1 | 0.20014 | 0.25732332 |
| Pcdh20 | 0.00144 | 2.01569798 |
| Pcm1 | 0.22016 | 0.43304658 |
| Gng2 | 0.10652 | 0.51438469 |
| Ppfia2 | 0.04171 | -0.78131813 |
| Gnl3l | 0.68907 | 0.07120198 |
| Metap1 | 0.56372 | 0.11274001 |
| Ska3 | 0.44717 | -0.37087629 |
| Tmod3 | 0.8398 | 0.04470916 |
| Slc34a2 | 0.00144 | -1.26934956 |
| Frmpd1 | 0.01106 | -1.04418778 |
| Gm36908 | 0.06477 | -2.29358273 |
| Mapk8 | 0.90717 | -0.02877441 |
| Chodl | 0.12901 | -0.94188657 |
| Pggt1b | 0.00144 | 2.78574308 |
| Zfp709 | 0.00179 | -1.10190923 |
| Limd2 | 0.00144 | -1.02402652 |
| Bcl6b | 0.10708 | -0.39025482 |
| Npr3 | 0.08219 | 0.6557138 |
| Syngr3 | 0.79058 | 0.14139158 |
| Rai2 | 0.01189 | -0.60662454 |
| Gm32810 | 0.00145 | -1.2388121 |
| Hmg20a | 0.53589 | -0.66581777 |
| Gm26885 | 0.80805 | 0.11245112 |
| Yy1 | 0.08065 | 0.35052002 |
| Copg1 | 0.05742 | -0.50906914 |
| Faah | 0.00489 | -0.94261996 |
| Notch4 | 0.00627 | -0.6572532 |
| Il1rap | 0.05051 | 0.77840714 |
| Paqr7 | 0.00622 | -1.17135446 |
| Dopey1 | 0.03511 | 0.58676865 |
| Rgs16 | 0.51623 | -0.22221878 |
| Zrsr2 | 0.02529 | 1.14373063 |
| Ankrd34b | 0.00544 | -1.10518964 |
| Ralyl | 0.00144 | -1.27699683 |
| Rnf141 | 0.07239 | -0.38829187 |
| Prodh | 0.45422 | 0.20379729 |
| Plxnb1 | 0.18678 | -0.24224568 |
| Xpo7 | 0.81474 | -0.15395858 |
| Bambi | 0.07119 | -0.72004573 |
| Krit1 | 0.32687 | -0.23669046 |
| Lss | 0.61509 | 0.10142051 |
| Atxn10 | 0.18322 | -0.21942432 |
| Usp9x | 0.06114 | -0.65726019 |
| Zfp553 | 0.02065 | -0.57473506 |
| Drd1 | 0.02785 | -0.84697243 |
| Tulp4 | 0.01401 | -0.53343866 |
| Crx | 0.05502 | -0.504131 |
| Gabra5 | 0.04481 | -0.93208132 |
| Eif3a | 0.00149 | -1.22582153 |
| Gm32592 | 0.00144 | -1.26934956 |
| Mthfr | 0.05476 | -0.45345397 |
| Gm32093 | 0.00144 | -1.30017714 |
| Ryk | 0.05144 | -0.48289502 |
| Avpr1a | 0.72747 | -0.14787891 |
| Megf11 | 0.00145 | -1.16044081 |
| Mxi1 | 0.08653 | -0.31695225 |
| Adcyap1 | 0.00144 | -1.26560808 |
| Cdk5rap3 | 0.00168 | -0.8538642 |
| Herc1 | 0.07105 | -0.41479021 |
| Spock1 | 0.14 | -0.26512267 |
| Akap9 | 0.09276 | 0.5024637 |
| Dpysl5 | 0.24446 | -0.44699018 |
| Zfp638 | 0.19571 | 0.55871471 |
| Kcns1 | 0.30093 | -0.2798708 |
| Frk | 0.07865 | 0.81450099 |
| Slc15a2 | 0.36805 | 1.53487725 |
| Zbtb14 | 0.46773 | -0.16761828 |
| Shank2 | 0.56775 | -0.09858559 |
| Anln | 0.23277 | -0.68219497 |
| Grasp | 0.00221 | -0.81613932 |
| Rilpl2 | 0.00944 | -0.7875833 |
| Sidt1 | 0.36071 | 0.40624941 |
| Ncf2 | 0.27209 | -0.22757902 |
| Gata6 | 0.83174 | -0.04624246 |
| AI662270 | 0.49498 | -0.47021969 |
| Smarca5 | 0.00542 | 1.32585095 |
| Gltscr1l | 0.62784 | -0.10211724 |
| Dmrt2 | 0.00144 | -1.27165604 |
| Olfml2b | 0.0093 | -0.927743 |
| Acadm | 0.00824 | 0.66491846 |
| Abcg3 | 0.38249 | 0.26192203 |
| Il13ra1 | 0.10483 | 0.48547837 |
| Tab2 | 0.28233 | -0.17630229 |
| Mknk2 | 0.01939 | -0.74059797 |
| Usp53 | 0.18033 | -0.38709239 |
| Fkbp7 | 0.62998 | -0.0902919 |
| Slco1a1 | 0.09682 | -1.11787898 |
| Lrrc8d | 0.6821 | -0.06530425 |
| Snhg7 | 0.25287 | 0.40547421 |
| Baat | 0.349 | -0.61477215 |
| Il2rg | 0.42317 | -0.14875192 |
| Nepn | 0.44682 | -0.20354146 |
| Stc1 | 0.00145 | 1.65541284 |
| Otop1 | 0.00231 | -1.67596867 |
| Camkmt | 0.00266 | 1.13504865 |
| Ebf1 | 0.1784 | 0.42357498 |
| Ror1 | 0.00151 | -0.87452629 |
| Slc25a45 | 0.10995 | -0.45034779 |
| Kbtbd2 | 0.75077 | -0.05305228 |
| Rnf157 | 0.85001 | -0.14853354 |
| Hace1 | 0.00529 | 1.27163555 |
| Gna13 | 0.0184 | 0.54456157 |
| Stim2 | 0.01201 | 0.77167923 |
| Cdc6 | 0.50884 | -0.24456057 |
| Zfhx3 | 0.65464 | -0.11755288 |
| Map2k3 | 0.01509 | -0.67664646 |
| Zdhhc17 | 0.04277 | -0.47145357 |
| Mir22hg | 0.00144 | 1.42684986 |
| Ep300 | 0.03165 | 0.91388218 |
| Gas1 | 0.0019 | -0.82086663 |
| Nup133 | 0.92538 | 0.03162821 |
| F2rl2 | 0.02049 | -1.72009297 |
| Mr1 | 0.19958 | 0.21201621 |
| Cnp | 0.0416 | -1.16076237 |
| Tm9sf4 | 0.04158 | -0.64123474 |
| Grik1 | 0.00144 | -1.29085005 |
| Magef1 | 0.00402 | 0.83781144 |
| Olfr558 | 0.94861 | -0.02249519 |
| Sp4 | 0.01901 | 0.56247484 |
| Ppp2r2a | 0.00443 | -0.84272653 |
| Gm34530 | 0.53365 | -0.24033601 |
| Gabrg1 | 0.00144 | -1.24811894 |
| 2610035D17Rik | 0.13592 | -0.28063784 |
| Nmur2 | 0.00144 | -1.76823119 |
| Mageh1 | 0.99241 | -0.00199445 |
| Atr | 0.12418 | -0.59806985 |
| Actr8 | 0.02989 | -0.43314446 |
| Creb1 | 0.39205 | 0.203992 |
| Rsrc2 | 0.06987 | 0.85995409 |
| Nfatc3 | 0.14132 | 0.59566637 |
| Igf1r | 0.44634 | -0.36163814 |
| Rspry1 | 0.64322 | -0.0777927 |
| Il1r2 | 0.06363 | 0.93757463 |
| Cd14 | 0.14116 | -0.41108249 |
| Usp7 | 0.0115 | 0.53869089 |
| Ehf | 0.0036 | -1.18318572 |
| Vav3 | 0.13104 | 0.4108479 |
| Map7 | 0.03774 | 0.47579523 |
| Krt6a | 0.14722 | -0.94944943 |
| Zfp759 | 0.1757 | 0.53640313 |
| Bms1 | 0.03372 | 0.59063287 |
| Klf16 | 0.00461 | -0.82498416 |
| Nlrp10 | 0.92806 | 0.01553582 |
| Sox6 | 0.13092 | 0.28456487 |
| Pid1 | 0.10063 | 0.81404457 |
| Slfn5 | 0.00241 | 1.95155899 |
| Zfp277 | 0.17938 | 0.25151575 |
| Susd5 | 0.77653 | -0.22820277 |
| Dusp15 | 0.07239 | -2.45248914 |
| Ttc14 | 0.00927 | 2.17865773 |
| Ppp1r16b | 0.05792 | -0.45804859 |
| Nfe2 | 0.03198 | -0.84192449 |
| Pten | 0.01583 | 0.66886775 |
| Slc35a1 | 0.00217 | 1.24081896 |
| Kcnh8 | 0.00144 | -1.30713006 |
| Gm34888 | 0.00144 | -1.25281511 |
| Gm14377 | 0.00144 | -1.28116048 |
| Tram1l1 | 0.07282 | -0.38994052 |
| Dcaf4 | 0.048 | -0.66964008 |
| 9530080O11Rik | 0.04349 | 0.42288372 |
| Calhm2 | 0.00213 | -1.04387647 |
| Gm20300 | 0.05179 | 0.57901307 |
| 9530053A07Rik | 0.0075 | -1.33182817 |
| Kctd7 | 0.00814 | -0.98670782 |
| Vamp3 | 0.76508 | -0.04852396 |
| Anapc2 | 0.02005 | -0.52818803 |
| Sh3bp4 | 0.00163 | -0.94980036 |
| Itpripl2 | 0.26413 | -0.29467847 |
| Mfap3l | 0.06158 | 0.89831848 |
| 9530014B07Rik | 0.01593 | -1.31041479 |
| Styk1 | 0.00144 | -1.22925777 |
| Gm10033 | 0.05758 | 0.76394154 |
| Fosl2 | 0.33294 | -0.25289534 |
| Mavs | 0.35836 | -0.29200633 |
| Gpr45 | 0.2864 | -0.51971217 |
| Sv2b | 0.41743 | -0.3800824 |
| Mmp16 | 0.42639 | -0.30957313 |
| Ptpn9 | 0.08176 | 0.31229958 |
| Nova1 | 0.69166 | -0.12465167 |
| Cpa4 | 0.02381 | -0.83045798 |
| Zfp282 | 0.021 | -0.59978674 |
| Rcn2 | 0.03955 | 0.49144781 |
| Acot2 | 0.00144 | -1.14694484 |
| Spg21 | 0.00738 | -0.64294177 |
| Erlec1 | 0.00458 | 0.85387254 |
| Neil3 | 0.06536 | 0.84484172 |
| Mis18bp1 | 0.02015 | 1.37029851 |
| Nrm | 0.00915 | -0.61998077 |
| Mis18bp1 | 0.187 | -0.64199981 |
| Gm13154 | 0.00343 | 1.72462731 |
| Mtap | 0.43696 | 0.12706008 |
| Igfbp3 | 0.58436 | -0.10975378 |
| Tmem38b | 0.50027 | -0.14598764 |
| Akap17b | 0.60773 | -0.15529323 |
| Tmtc1 | 0.01298 | 0.8737316 |
| Kbtbd8 | 0.19358 | -0.61050293 |
| Hoxb7 | 0.34057 | -0.80921683 |
| Gnai1 | 0.01464 | 0.7978018 |
| Oat | 0.36821 | 0.1875287 |
| Arl6ip1 | 0.00144 | -1.24198775 |
| Rasgrp3 | 0.00327 | 1.73013142 |
| Kdm7a | 0.61593 | -0.1677846 |
| Sspn | 0.1395 | -0.26428992 |
| Smurf1 | 0.06421 | -0.48206166 |
| Gbp2b | 0.00144 | -1.27165604 |
| Shpk | 0.00948 | -0.75672299 |
| Kcnn4 | 0.00144 | -1.65007361 |
| Gimap9 | 0.06277 | 0.53774536 |
| Ap3m1 | 0.01257 | 0.75164993 |
| Mrrf | 0.37327 | -0.24428511 |
| Cops8 | 0.48466 | 0.27526188 |
| LOC105244164 | 0.07096 | -2.32850014 |
| Whamm | 0.75164 | -0.0549966 |
| Ccdc146 | 0.46658 | -0.20923065 |
| Ttc39d | 0.06358 | -1.14181089 |
| Ccbe1 | 0.02431 | 0.85588813 |
| 1700019B03Rik | 0.03791 | -0.9860568 |
| Zfp110 | 0.14762 | 0.35614313 |
| Gm364 | 0.00144 | -1.27992547 |
| 4930455F16Rik | 0.37678 | -0.82975972 |
| Optn | 0.11459 | 0.28766196 |
| Sh3bp5 | 0.00396 | 0.71790318 |
| Diap1 | 0.00559 | -0.63596221 |
| Ms4a1 | 0.01292 | 1.6729749 |
| Kctd1 | 0.10733 | -0.28592023 |
| Ccnl2 | 0.77283 | 0.06948334 |
| Nrbp1 | 0.06022 | 0.50962093 |
| Fuca2 | 0.29471 | 0.33894876 |
| Ift81 | 0.00712 | 0.87740611 |
| Cep170 | 0.00761 | 0.90491448 |
| Loh12cr1 | 0.41047 | -0.3181912 |
| LOC102641067 | 0.29257 | 0.4687255 |
| Cntnap4 | 0.02018 | -1.76914284 |
| Ikbkb | 0.01002 | 0.66006929 |
| Pros1 | 0.0281 | 0.83280124 |
| Gm31445 | 0.00144 | -1.28116048 |
| Zfp597 | 0.00144 | 1.42545924 |
| Arhgef28 | 0.29573 | 0.50435851 |
| Eprs | 0.01314 | 0.5927527 |
| Slc35a3 | 0.59538 | -0.09229277 |
| Aldh5a1 | 0.01411 | 0.69853545 |
| Bmpr1a | 0.00405 | 0.74038306 |
| Amigo1 | 0.96337 | -0.02854204 |
| Gja1 | 0.00144 | -1.55335628 |
| Caprin1 | 0.01232 | 0.67188938 |
| Flt1 | 0.00144 | 2.42842689 |
| Hyi | 0.34404 | 0.31766728 |
| Kcnk13 | 0.00311 | -0.89409049 |
| Nrp2 | 0.02718 | -0.53033783 |
| AW047730 | 0.03594 | -0.87369633 |
| Cbl | 0.17641 | -0.23235228 |
| Fam120c | 0.0462 | -0.47280068 |
| 6430571L13Rik | 0.04218 | -0.99076953 |
| Col12a1 | 0.60054 | 0.31781268 |
| Zcchc4 | 0.02671 | -0.86465577 |
| Gm31319 | 0.00144 | -1.28898079 |
| Il1rapl1 | 0.00144 | -1.28898079 |
| Tbc1d10a | 0.06726 | -0.36386665 |
| LOC102639979 | 0.00144 | -1.28116048 |
| Kctd11 | 0.63578 | -0.14475138 |
| Rnase10 | 0.49467 | 0.15542423 |
| Gm14321 | 0.72005 | 0.10441174 |
| Atp6v1h | 0.96351 | -0.00847733 |
| BC021891 | 0.06559 | -0.55442132 |
| Zfp58 | 0.21413 | 0.33488873 |
| Socs6 | 0.01637 | 0.54372733 |
| Prpsap2 | 0.72977 | 0.05868869 |
| Lmbrd1 | 0.03062 | 0.45253514 |
| Efnb2 | 0.29139 | 0.24539385 |
| Ppwd1 | 0.05065 | 1.14157259 |
| Spats2l | 0.01163 | 0.70405344 |
| Rnf125 | 0.0018 | 1.29734213 |
| Tmem201 | 0.06158 | -0.34869569 |
| Slc6a15 | 0.01456 | -1.03105883 |
| Fut9 | 0.00144 | -1.27165604 |
| Rdh14 | 0.09749 | 0.46412094 |
| Psmf1 | 0.9722 | -0.00587561 |
| Pcmt1 | 0.51312 | -0.10050867 |
| Luzp1 | 0.05104 | 0.57105357 |
| Esrrg | 0.00346 | 0.99488665 |
| Ppapdc3 | 0.02528 | -1.00475461 |
| Sacm1l | 0.02946 | 0.51319882 |
| Ccnc | 0.02661 | 0.54863126 |
| Foxo1 | 0.00199 | -0.82958409 |
| Fam98a | 0.40265 | 0.16565304 |
| Txnl4a | 0.83238 | -0.05409744 |
| Arpc5l | 0.09237 | -0.28729302 |
| Acadl | 0.08453 | 0.31313619 |
| Eif2d | 0.01337 | -0.66013935 |
| Gskip | 0.92051 | 0.01648311 |
| Kank2 | 0.56876 | -0.14258241 |
| Cacul1 | 0.02136 | -0.45936562 |
| Abhd13 | 0.87925 | 0.02551809 |
| Shh | 0.00145 | -1.2388121 |
| Mnat1 | 0.76765 | -0.04980209 |
| Dnajb6 | 0.01322 | 0.82184719 |
| Cbwd1 | 0.0045 | 0.85384269 |
| Rcl1 | 0.04079 | -0.50501713 |
| 3110035E14Rik | 0.35129 | -0.32641897 |
| Csde1 | 0.01514 | -0.95990192 |
| Ankrd6 | 0.00144 | -1.28116048 |
| Psmd13 | 0.00184 | -0.81419136 |
| Zmym2 | 0.0329 | 0.56697778 |
| Sox9 | 0.10604 | -0.49321331 |
| Chd4 | 0.00145 | -0.9316099 |
| Mfsd1 | 0.19785 | -0.23322218 |
| Alad | 0.00267 | -1.0675382 |
| Psmd1 | 0.42149 | -0.14140501 |
| Meis2 | 0.003 | 0.85986168 |
| Npl | 0.01505 | -0.68626946 |
| Acer3 | 0.41806 | -0.17241198 |
| Arap2 | 0.04849 | -0.7326722 |
| Zbtb11 | 0.00149 | 1.18284611 |
| Atg12 | 0.2069 | 0.21196356 |
| Zeb1 | 0.93531 | -0.01743567 |
| Farsa | 0.00377 | -0.76581144 |
| Tead1 | 0.03616 | -0.44808113 |
| Rad54b | 0.94762 | -0.02205656 |
| Tmem183a | 0.48184 | -0.12479953 |
| Cldn1 | 0.08746 | -1.12579206 |
| Parvg | 0.33551 | 0.2331983 |
| Orc2 | 0.00679 | -1.40804231 |
| Rhoh | 0.02942 | -0.70790993 |
| Atpaf1 | 0.56787 | 0.12116177 |
| Trim11 | 0.04277 | -0.42777293 |
| Fbxl12 | 0.24205 | -0.21597193 |
| Glipr1l2 | 0.0475 | -0.92312902 |
| Canx | 0.00483 | 0.8596362 |
| Zfand3 | 0.01238 | -0.86627123 |
| Gm10634 | 0.00144 | -1.25048839 |
| Gm8140 | 0.00149 | -1.2703832 |
| Wbp11 | 0.93699 | -0.02485272 |
| 4933406D12Rik | 0.00145 | -1.52620111 |
| Vwa3a | 0.65013 | -0.24189034 |
| Ubxn10 | 0.21586 | -0.47267695 |
| Robo1 | 0.00251 | 1.08804508 |
| Thsd7b | 0.00846 | -1.09251133 |
| Ap5m1 | 0.0019 | -1.13608632 |
| Nras | 0.0243 | -0.48545697 |
| 4930549G23Rik | 0.00144 | -1.33632959 |
| Rnf38 | 0.44646 | -0.19869782 |
| Necap2 | 0.00144 | -1.03479492 |
| D7Ertd443e | 0.0144 | -1.94216987 |
| Tex30 | 0.00144 | 1.30703875 |
| Spag6 | 0.00158 | -1.2446827 |
| Smc4 | 0.03717 | 1.46502101 |
| Caps2 | 0.00144 | -1.25927895 |
| Spag17 | 0.16053 | -0.84180189 |
| 1700074P13Rik | 0.01943 | -1.12793531 |
| Spata19 | 0.00144 | -1.26560808 |
| Hgsnat | 0.00622 | -0.6402046 |
| Eid3 | 0.11703 | 0.64188059 |
| Zswim2 | 0.13573 | -0.80821891 |
| Cep126 | 0.0054 | -1.08878157 |
| Ift140 | 0.14545 | -0.26178888 |
| Uimc1 | 0.00256 | 1.13142788 |
| Gm34285 | 0.00144 | -1.47681752 |
| Mybl1 | 0.00144 | 1.81688087 |
| Lrriq3 | 0.00144 | -1.28898079 |
| Enkur | 0.00144 | 1.54250431 |
| Zfp36 | 0.52205 | -0.13937037 |
| Krt10 | 0.14251 | 0.29177695 |
| Ckap2 | 0.32943 | 0.59892071 |
| Slc9a3r1 | 0.00144 | -0.9884842 |
| Pir | 0.17676 | -0.27360224 |
| Ttn | 0.73143 | -0.062675 |
| Mtmr12 | 0.00577 | 0.6404323 |
| Ndrg1 | 0.00518 | -0.83194916 |
| Vcl | 0.03495 | 0.4264736 |
| Eln | 0.041 | -0.63555868 |
| Ttc28 | 0.01575 | -0.5313749 |
| Pkn2 | 0.14936 | 0.30594355 |
| Slc9a9 | 0.41316 | -0.17164728 |
| Khsrp | 0.01072 | -0.58067146 |
| Numbl | 0.01383 | -0.5843538 |
| Fsbp | 0.00501 | -0.81738903 |
| Gphn | 0.0017 | 0.88740894 |
| Rassf3 | 0.21956 | -0.1993445 |
| Ccny | 0.10215 | 0.40069895 |
| Fa2h | 0.06536 | -0.77712947 |
| Otub2 | 0.1189 | -0.30517335 |
| Zcchc24 | 0.13123 | -0.26468048 |
| Clcn4-2 | 0.51264 | 0.09967999 |
| Ninl | 0.33169 | -0.51166014 |
| Bag3 | 0.10322 | -0.46375419 |
| Dnmt3b | 0.31289 | 0.30514693 |
| Cep192 | 0.00203 | 1.72323211 |
| Eri1 | 0.15856 | 0.3534974 |
| Bbs7 | 0.00278 | 0.91441989 |
| Orc1 | 0.03781 | -0.92964662 |
| Ddx3y | 0.00254 | 1.08840528 |
| Pyroxd2 | 0.13798 | 0.43917952 |
| Supt7l | 0.23819 | 0.34457317 |
| Ptprr | 0.20008 | -0.33960358 |
| Haus3 | 0.24987 | 1.37724482 |
| Asf1b | 0.07474 | -0.51239436 |
| Ttc1 | 0.00657 | -0.85179626 |
| Mkrn1 | 0.35909 | 0.31463899 |
| Nudt21 | 0.02932 | -0.44434987 |
| Mycn | 0.07168 | -0.73979846 |
| Crem | 0.01352 | 0.94168038 |
| Usp42 | 0.07236 | -0.38818405 |
| Hsp90aa1 | 0.11705 | -0.60780066 |
| Rps8 | 0.00144 | -1.28012597 |
| Rpl7a | 0.06595 | -0.5092723 |
| Ears2 | 0.23952 | -0.38630873 |
| 2210408I21Rik | 0.12744 | 0.38589071 |
| Abca14 | 0.72127 | -0.17150794 |
| Fhl4 | 0.96836 | -0.01391945 |
| Snx3 | 0.40619 | -0.24410046 |
| Pgam2 | 0.00144 | -1.03338243 |
| Rfk | 0.47171 | -0.17081869 |
| Adipor1 | 0.01042 | -0.69887268 |
| Diablo | 0.01691 | -0.6240546 |
| Gm27211 | 0.17418 | -0.82847561 |
| Klhl5 | 0.00144 | -1.28898079 |
| Gm35167 | 0.30744 | -0.55205602 |
| Brd8 | 0.03473 | 0.41646535 |
| Cahm | 0.05689 | 0.72995695 |
| Faxc | 0.14944 | -0.89882361 |
| Dner | 0.40589 | -0.68715289 |
| Slc22a3 | 0.00144 | -1.16859794 |
| Fam126a | 0.24534 | -0.22902686 |
| Rgmb | 0.02374 | -0.72842906 |
| Phc3 | 0.0025 | 1.5870482 |
| Mphosph6 | 0.06149 | -0.34036359 |
| Cacna1b | 0.01881 | -0.87938833 |
| Grik5 | 0.00253 | -0.98887901 |
| Fam189a2 | 0.03055 | -0.66932683 |
| A330076C08Rik | 0.05779 | -0.97801886 |
| Phyhip | 0.06193 | -1.93215554 |
| Gm38666 | 0.00145 | -1.2388121 |
| Caml | 0.79803 | -0.0400743 |
| BC006965 | 0.69537 | -0.16647579 |
| Ndufb2 | 0.18303 | 0.68169596 |
| Dync1i1 | 0.18102 | -1.5849625 |
| Grin1 | 0.00144 | -1.28898079 |
| Cep120 | 0.17123 | 0.26641481 |
| Cdk5r2 | 0.07874 | -2.24633137 |
| Hnrnpll | 0.06295 | -0.66819856 |
| Dclk2 | 0.04618 | -0.97427738 |
| Gm31369 | 0.00153 | -1.24063982 |
| Higd1b | 0.36843 | -0.45704107 |
| Vps11 | 0.00527 | -1.43769573 |
| Dock1 | 0.04484 | 0.36342649 |
| Gm32178 | 0.27624 | -0.65895 |
| Synrg | 0.61386 | 0.25514138 |
| Ptpn14 | 0.29039 | 0.20323049 |
| Tmem18 | 0.02408 | 0.54515937 |
| Crebl2 | 0.01499 | -1.01847297 |
| Brinp2 | 0.00145 | -1.22376446 |
| Map3k10 | 0.00541 | -0.93957292 |
| Bahd1 | 0.07335 | -0.38649105 |
| Jph3 | 0.10143 | -1.01579887 |
| Klf15 | 0.11626 | 0.47284939 |
| Dlg2 | 0.00262 | -1.0890011 |
| Shprh | 0.00144 | 1.68508203 |
| Lmtk2 | 0.56254 | 0.20275431 |
| Rbm34 | 0.05066 | 0.67352961 |
| Panx2 | 0.05361 | -0.87063435 |
| Cacnb2 | 0.54132 | 0.11423549 |
| Frmd6 | 0.82566 | -0.06204765 |
| Ptpn5 | 0.38663 | -0.68778445 |
| 2610034B18Rik | 0.00722 | -0.78191743 |
| Tubb4a | 0.00197 | -1.06287166 |
| Trib1 | 0.84126 | -0.05530387 |
| Cln5 | 0.63553 | 0.07665743 |
| Fmo1 | 0.06113 | 0.46629245 |
| Nol11 | 0.54827 | 0.09117008 |
| Clstn1 | 0.00661 | -0.64292779 |
| Ubp1 | 0.00144 | 1.23705607 |
| Coq10b | 0.00523 | 1.41568734 |
| Chd5 | 0.01265 | -1.07139495 |
| Scn8a | 0.00145 | -1.22518835 |
| Pcmt1 | 0.33187 | -0.14969304 |
| Yars2 | 0.11109 | 0.50683888 |
| Map2 | 0.05446 | 0.7760574 |
| Sfr1 | 0.01233 | 0.52629736 |
| Pitx2 | 0.78896 | 0.23408813 |
| Gm30713 | 0.05254 | -0.5832085 |
| Ythdf2 | 0.66765 | 0.08153888 |
| Psma4 | 0.05824 | 0.39567793 |
| Bub3 | 0.01523 | -0.54663405 |
| Pgls | 0.00144 | -1.59003023 |
| Cox7b | 0.57011 | -0.09801183 |
| Slc22a5 | 0.71841 | 0.12142862 |
| Cela1 | 0.00168 | -1.33365648 |
| Gnpda1 | 0.20624 | -0.36190132 |
| Ppp1r2 | 0.00753 | 0.68992805 |
| Cyp51 | 0.0319 | -0.48021115 |
| Vamp4 | 0.02187 | 0.54759812 |
| 1700018C11Rik | 0.04637 | -0.51777609 |
| Prl8a8 | 0.00144 | -1.31884045 |
| Ddit4l | 0.00279 | -1.33072455 |
| Mbtps2 | 0.0048 | 1.09338082 |
| Cyb5r4 | 0.41748 | 0.21869811 |
| Skap1 | 0.00145 | -1.2388121 |
| Cog2 | 0.02658 | -0.53957827 |
| Mief2 | 0.01495 | -0.80735469 |
| Riok1 | 0.01303 | 0.6394458 |
| Klri2 | 0.00149 | -1.2703832 |
| Dcp1b | 0.08565 | -0.99995638 |
| Bbs2 | 0.01491 | -1.59969577 |
| Dmtf1 | 0.00144 | 2.24813784 |
| Vmn2r97 | 0.19245 | -0.34848099 |
| Fgf7 | 0.05207 | 0.93089785 |
| Trim33 | 0.42213 | -0.1766181 |
| Gm36223 | 0.00144 | -1.29049509 |
| Scoc | 0.02918 | 0.73319807 |
| Phospho2 | 0.50945 | 0.11147296 |
| Gm36279 | 0.01085 | -1.94265032 |
| 1700096J18Rik | 0.00753 | -1.58658673 |
| Sell | 0.98349 | -0.0036821 |
| Gm33558 | 0.72532 | 0.23865081 |
| Gm31208 | 0.0015 | -1.32982311 |
| Ar | 0.3573 | 0.3487989 |
| Ap5s1 | 0.17507 | -0.57045343 |
| Rbbp6 | 0.00971 | -0.61564369 |
| Gm30270 | 0.08413 | -0.47111823 |
| Gm36418 | 0.1684 | -1.9432641 |
| Mmp12 | 0.0759 | -1.00019218 |
| Gm14296 | 0.45122 | 0.1280778 |
| Pdp2 | 0.02882 | -0.52239948 |
| Wdr43 | 0.00152 | 1.07531871 |
| Fyb | 0.52354 | -0.13532257 |
| Gm15420 | 0.03557 | -0.55891207 |
| Gm24474 | 0.03717 | 0.56136032 |
| Egr3 | 0.00431 | 3.76142679 |
| Acads | 0.00947 | -1.08059225 |
| Gm27204 | 0.00149 | -1.19696847 |
| Gm35986 | 0.00144 | -1.28375735 |
| Cdr2 | 0.01581 | 0.98242171 |
| Dcaf6 | 0.2597 | -0.25707791 |
| Ptgfrn | 0.56921 | -0.09755103 |
| 1110032A03Rik | 0.01233 | -0.54732989 |
| Gm36796 | 0.00152 | -1.25878908 |
| Foxp1 | 0.00761 | 0.69779961 |
| Gm35035 | 0.00144 | -1.29049509 |
| Rps6ka3 | 0.03956 | 0.70602125 |
| Cct6a | 0.77323 | 0.05010858 |
| Gm32885 | 0.00144 | -1.29085005 |
| Acnat1 | 0.06697 | 1.10900553 |
| Desi2 | 0.00763 | 0.71413791 |
| Gm34152 | 0.00169 | -1.39182106 |
| Gm14114 | 0.00144 | -1.28898079 |
| Krt14 | 0.01799 | -1.14260151 |
| Dcpp2 | 0.00144 | -1.30713006 |
| Gm38619 | 0.00144 | -1.27165604 |
| Msx1 | 0.00211 | -1.04048108 |
| Pex19 | 0.01606 | -0.89229082 |
| Srsf11 | 0.56542 | 0.09210189 |
| Gm16104 | 0.0742 | -0.82227368 |
| Lpcat3 | 0.08887 | -0.54810622 |
| Gm31278 | 0.00298 | -1.78349908 |
| Ppp1r18 | 0.01724 | -0.56796716 |
| Lysmd2 | 0.7778 | -0.05935629 |
| Lhfp | 0.51661 | 0.15439791 |
| Rpl6 | 0.18883 | -0.32378875 |
| Eef1g | 0.03537 | -0.64380861 |
| Zdhhc6 | 0.08305 | 0.43868365 |
| Gm31819 | 0.00144 | -1.26560808 |
| Iws1 | 0.00144 | -1.30713006 |
| Gm16619 | 0.00144 | -1.29051721 |
| Tdgf1 | 0.00145 | -1.2388121 |
| Dlg1 | 0.22034 | 0.35878148 |
| Ahsa1 | 0.19761 | -0.23096911 |
| Gm16976 | 0.00144 | -1.28116048 |
| Gm33317 | 0.34635 | -0.73742782 |
| Ubxn2a | 0.14681 | 0.26487515 |
| Gm5595 | 0.85095 | -0.03056199 |
| Tmem41b | 0.03625 | 0.6861084 |
| Ap3s1 | 0.95507 | 0.01283975 |
| BC005624 | 0.75838 | -0.05578851 |
| Grpel2 | 0.00158 | -1.00307331 |
| Tpk1 | 0.00144 | 1.68510937 |
| Tmem178b | 0.75976 | -0.1474527 |
| Rab2a | 0.23199 | 0.21687987 |
| Ormdl2 | 0.44159 | -0.15060884 |
| Krtcap3 | 0.44003 | -1.18940785 |
| Gcn1l1 | 0.00152 | -0.94371614 |
| Gm11757 | 0.80984 | -0.283097 |
| 1700112D23Rik | 0.00145 | -1.2388121 |
| Rims3 | 0.09427 | -2.70495663 |
| Rps25 | 0.00145 | -0.89186299 |
| C030004G16Rik | 0.12604 | -0.54684712 |
| Zfp235 | 0.16326 | -0.40197585 |
| Gm28783 | 0.17982 | -0.75419143 |
| Rasgrf2 | 0.28581 | 0.54772782 |
| Tmem14c | 0.02112 | -0.74564549 |
| C920009B18Rik | 0.00455 | -1.1280166 |
| Gm29128 | 0.00145 | -1.2388121 |
| Sgip1 | 0.70836 | -0.10689242 |
| LOC735298 | 0.01241 | -1.02801808 |
| Tdrd12 | 0.00292 | -1.43632968 |
| 4930519P11Rik | 0.26142 | 0.22913496 |
| Armc7 | 0.73252 | 0.07836501 |
| Mthfd1l | 0.00317 | -1.33059316 |
| Lin28b | 0.0484 | -1.4212066 |
| Gm38560 | 0.10822 | 0.71658656 |
| Gm32507 | 0.37342 | 0.48773736 |
| Gm33663 | 0.00144 | -1.31884045 |
| Dfna5 | 0.6113 | -0.42815113 |
| Gm26868 | 0.0019 | -1.13608632 |
| Gm20506 | 0.75537 | -0.28819962 |
| Gm30421 | 0.00144 | -1.30713006 |
| 1700100I10Rik | 0.00145 | -1.24191372 |
| Gm36365 | 0.1203 | -0.69984522 |
| Gm38398 | 0.18975 | -1.55081145 |
| Gm1965 | 0.07056 | 0.77892738 |
| Egln1 | 0.17396 | -0.31043901 |
| Pcbp1 | 0.04289 | -0.68641687 |
| Fam169a | 0.07382 | -0.86014901 |
| Eef1b2 | 0.04327 | -0.43258869 |
| Gm34880 | 0.8883 | 0.08005466 |
| Gm12107 | 0.00288 | -1.09711326 |
| Kcnn2 | 0.25033 | 0.40974364 |
| Gm13001 | 0.96692 | -0.01317612 |
| Rpusd4 | 0.66682 | 0.12013867 |
| Scfd2 | 0.31204 | 0.30059476 |
| Gm34657 | 0.0038 | -1.07011841 |
| Dctn2 | 0.006 | -0.6266373 |
| Gm4988 | 0.00145 | -1.26219879 |
| Tab2 | 0.0049 | 0.95109436 |
| Hist3h2bb-ps | 0.0024 | -1.84638627 |
| Olfr196 | 0.00144 | -1.38703011 |
| Ccdc127 | 0.70587 | 0.11746727 |
| Zfp488 | 0.07183 | -0.45069964 |
| Gm32587 | 0.12434 | -0.87369633 |
| Col9a1 | 0.09409 | -0.8082463 |
| Myh10 | 0.01991 | 0.53357976 |
| AY702103 | 0.69036 | -0.14994376 |
| Gm11269 | 0.00144 | -1.31495282 |
| Gm16322 | 0.83081 | -0.24107716 |
| Gapdh | 0.01204 | -0.66567623 |
| Olfr1484 | 0.10962 | -0.83607133 |
| Gm13941 | 0.01025 | -1.73283138 |
| Gm4281 | 0.04198 | -0.83967303 |
| Zbtb18 | 0.14605 | 0.29604465 |
| Gm34595 | 0.0015 | -1.43594442 |
| Letm2 | 0.0105 | 1.08202715 |
| Gm19600 | 0.00159 | -1.26431393 |
| Rnf169 | 0.54657 | 0.17013377 |
| Gm36447 | 0.4265 | -0.39428802 |
| Plekhf2 | 0.09677 | -0.34780905 |
| Gm31115 | 0.00144 | -1.3826329 |
| Lrrc38 | 0.02449 | -0.8606076 |
| Mannr | 0.00144 | -1.28116048 |
| Gm13715 | 0.00395 | -0.98868564 |
| Ppp1r12c | 0.00313 | -0.88580971 |
| Nxt1 | 0.03041 | -0.59627974 |
| Lhcgr | 0.00144 | -1.28116048 |
| Gm35933 | 0.27307 | 0.39946599 |
| Gm16537 | 0.01697 | -0.81698462 |
| Gm34586 | 0.00144 | -1.33632959 |
| E130309D02Rik | 0.00386 | -1.12466797 |
| Gm36654 | 0.00144 | -1.52534313 |
| Gm38719 | 0.15943 | 0.44270981 |
| Gm35798 | 0.00144 | -1.51500476 |
| Slc35a4 | 0.00899 | -0.78631146 |
| Sfn | 0.00266 | -0.96972435 |
| Gm16013 | 0.93489 | -0.06822208 |
| Rbm3 | 0.01229 | 1.64288074 |
| LOC102635529 | 0.01496 | -1.20381157 |
| Kl | 0.00144 | -1.28898079 |
| Gm31645 | 0.39943 | 0.53438293 |
| Gm33531 | 0.33546 | -0.53145373 |
| Nmur1 | 0.00538 | -1.61597574 |
| Otp | 0.05439 | -2.31584419 |
| 9130409I23Rik | 0.00144 | -1.26560808 |
| Pkd2 | 0.80379 | 0.2182293 |
| Prelid1 | 0.01105 | -0.70613609 |
| Ube2d3 | 0.06843 | 0.39998177 |
| Tomm22 | 0.15451 | -0.35630968 |
| Gm15290 | 0.01715 | -1.29864962 |
| 2310047M10Rik | 0.00176 | -1.10340031 |
| Zbed4 | 0.26658 | 0.48100664 |
| Olfr818 | 0.06351 | -1.04767238 |
| Rpsa | 0.00144 | -1.50396406 |
| Utrn | 0.01185 | 0.79686807 |
| Utp14a | 0.82931 | 0.05039303 |
| Aqp9 | 0.00682 | -1.59255835 |
| Gm29040 | 0.78041 | -0.10042521 |
| Nkx1-2 | 0.03757 | -1.50096201 |
| Gm32930 | 0.00144 | -1.28116048 |
| Amy2b | 0.00149 | -1.19696847 |
| Olfr1243 | 0.00145 | -1.36286464 |
| Olfr1182 | 0.00144 | -1.32599123 |
| Olfr988 | 0.00144 | -1.20579466 |
| Gm13601 | 0.00144 | -1.29746368 |
| Gm13266 | 0.25355 | -0.55178394 |
| Eif4enif1 | 0.42569 | -0.12846066 |
| Ptcd1 | 0.04723 | -0.48887321 |
| Msl2 | 0.0318 | 0.67356122 |
| Dnaja3 | 0.92733 | -0.03264214 |
| Ifitm3 | 0.00229 | -0.91665261 |
| Zc3h15 | 0.11163 | 0.56744997 |
| Rybp | 0.00144 | 1.36097861 |
| LOC545086 | 0.00144 | -1.27730451 |
| Amd2 | 0.00753 | -0.93084982 |
| Adrm1 | 0.974 | -0.01871904 |
| Axin1 | 0.07362 | -0.43027737 |
| Gm33446 | 0.40354 | -0.38007492 |
| Ctsr | 0.03901 | -0.55785214 |
| Rpl36a | 0.00229 | -0.77504441 |
| Tpgs2 | 0.24018 | -0.25990845 |
| Mex3b | 0.04093 | -0.50055036 |
| Tgs1 | 0.16182 | -0.35964149 |
| Tctn1 | 0.00516 | -0.65908912 |
| Pkm | 0.00322 | -0.89381641 |
| Minpp1 | 0.88026 | -0.0245037 |
| Smarca5 | 0.09027 | 0.32291794 |
| Cyfip1 | 0.80062 | -0.07808554 |
| LOC102636380 | 0.00395 | -1.19099662 |
| Tpm4 | 0.01686 | 0.78469181 |
| 4930435F18Rik | 0.00144 | -1.28116048 |
| Gm12963 | 0.22145 | -1.96426073 |
| Defa24 | 0.24599 | -0.37517696 |
| Kbtbd7 | 0.00518 | 0.76906255 |
| Pgls | 0.49725 | -0.26221823 |
| Hap1 | 0.01005 | -0.58776702 |
| Unc119b | 0.00508 | -0.67967454 |
| Fus | 0.00144 | 1.59261726 |
| Dscc1 | 0.38305 | 0.67497379 |
| Usp44 | 0.00144 | -1.26560808 |
| Rrp12 | 0.1925 | -0.26457325 |
| A430010J10Rik | 0.05989 | -0.7911528 |
| Uchl3 | 0.00639 | 0.8735871 |
| Gm15517 | 0.90192 | 0.14375532 |
| E130309D14Rik | 0.18209 | -0.58743851 |
| Srp9 | 0.61642 | -0.11288737 |
| Spata5l1 | 0.49059 | -0.11756571 |
| Gm33485 | 0.0037 | -1.11214255 |
| Gm13051 | 0.0025 | 1.32411306 |
| Rcan1 | 0.01751 | -0.71210962 |
| Gm34979 | 0.03011 | -1.04761516 |
| Phb | 0.01615 | -0.72612454 |
| Irs4 | 0.00144 | -1.26301121 |
| Mrpl22 | 0.04347 | -0.57665239 |
| Phykpl | 0.08219 | 1.02075354 |
| Tmed4 | 0.17636 | -0.32571587 |
| Slc10a4 | 0.01884 | -1.04711862 |
| Taf6 | 0.0264 | -0.72835707 |
| Gm20753 | 0.00144 | 3.72312367 |
| Braf | 0.00144 | 1.62868658 |
| Emx2 | 0.00144 | -1.50785399 |
| Gm2065 | 0.00222 | -1.1481805 |
| Gm31537 | 0.53263 | -0.28816168 |
| Marcksl1 | 0.31574 | -0.42887989 |
| Hmox1 | 0.64901 | -0.16241653 |
| Bcap29 | 0.00144 | 1.5872861 |
| Gm33789 | 0.00468 | 1.82029446 |
| Spint3 | 0.00149 | -1.28183002 |
| Gm32271 | 0.33794 | -0.68369626 |
| Wrn | 0.00144 | 2.18545969 |
| Olfr1494 | 0.50044 | -0.20260506 |
| Olfr211 | 0.17933 | -0.56881267 |
| Olfr133 | 0.00298 | -1.42363422 |
| Olfr914 | 0.2049 | -0.29594184 |
| Olfr1451 | 0.00144 | -1.31884045 |
| Olfr809 | 0.00144 | -1.34390295 |
| Olfr960 | 0.17668 | 0.52684044 |
| Gm20016 | 0.00144 | -1.31495282 |
| Dnajc27 | 0.00144 | 1.59161634 |
| Usp53 | 0.04894 | 0.79064616 |
| Gm35591 | 0.00325 | -2.00785158 |
| Gm11228 | 0.00144 | -1.26560808 |
| A630024J24Rik | 0.68982 | 0.16667033 |
| Timm9 | 0.05371 | 0.3510154 |
| 1700106J16Rik | 0.02203 | -0.972494 |
| 4931428F04Rik | 0.25913 | -0.83976343 |
| Mrpl16 | 0.01712 | -0.55601676 |
| Appl1 | 0.01316 | -1.71426594 |
| Magea8 | 0.02464 | -0.94533166 |
| Magea5 | 0.00144 | -1.27165604 |
| Gm19872 | 0.00158 | -1.2446827 |
| A26c2 | 0.00144 | -1.25118919 |
| Atxn7l2 | 0.03239 | -0.43865502 |
| 4930543I03Rik | 0.02708 | -0.99199803 |
| Gm30984 | 0.28288 | -0.20229632 |
| Crybg3 | 0.06663 | -0.38745527 |
| Snx29 | 0.34686 | -0.27778356 |
| Pfdn5 | 0.00636 | -0.73653578 |
| Gm30340 | 0.00144 | -1.61915423 |
| Il21r | 0.70836 | -0.07093383 |
| Gm10103 | 0.00144 | -1.28116048 |
| Gm26578 | 0.10211 | -1.22376446 |
| Gm32219 | 0.00145 | -1.22376446 |
| AA589522 | 0.64036 | -0.34619004 |
| Hpgds | 0.00144 | -1.27730451 |
| 5730409K12Rik | 0.12509 | -2.07861973 |
| LOC102632821 | 0.79249 | 0.08412905 |
| Gm31227 | 0.00144 | -1.51491761 |
| Fscn2 | 0.01798 | -0.96948519 |
| D630033A02Rik | 0.8184 | -0.06854234 |
| Gm32710 | 0.00145 | -1.22376446 |
| Gm30104 | 0.00901 | -1.22196083 |
| Lcn4 | 0.11338 | 0.57748337 |
| Per3 | 0.00514 | 1.02977282 |
| LOC102634481 | 0.00144 | 1.69638017 |
| Gm28515 | 0.37511 | 0.31887271 |
| Gm31456 | 0.97975 | -0.02849874 |
| Bola1 | 0.00353 | -0.98601152 |
| BC030308 | 0.86678 | -0.04559018 |
| Chd9 | 0.00365 | 1.31505598 |
| Vip | 0.00144 | -1.27165604 |
| Arfrp1 | 0.04406 | -0.85239345 |
| Accs | 0.09123 | 0.47018607 |
| Psenen | 0.06928 | -0.64693034 |
| 1700110I07Rik | 0.04739 | -2.0506677 |
| Gm9918 | 0.04169 | -0.88587163 |
| Gm31904 | 0.00144 | -1.28898079 |
| Tmco2 | 0.05522 | -0.69538555 |
| Gm12440 | 0.22841 | 0.54652657 |
| Hnrnpr | 0.00958 | 2.95332512 |
| Ttc19 | 0.00144 | 1.02306044 |
| 1700027J07Rik | 0.01877 | -1.38064341 |
| Gm33758 | 0.00144 | -1.26560808 |
| Gm34648 | 0.00144 | -1.28116048 |
| 1700036G14Rik | 0.00216 | -1.10905491 |
| Cox7b2 | 0.49639 | 0.73302006 |
| Depdc1a | 0.52105 | -1.16110454 |
| Osr2 | 0.00144 | -1.33059316 |
| LOC102636590 | 0.00144 | -1.26560808 |
| Scp2d1 | 0.04318 | -1.48580321 |
| Gm4841 | 0.24142 | 0.38697634 |
| Olfr31 | 0.10699 | 0.49617484 |
| Paox | 0.09398 | -0.41371393 |
| Foxm1 | 0.59809 | -0.23351488 |
| Gas7 | 0.44273 | -0.11861364 |
| Gm33002 | 0.00144 | -1.27165604 |
| Cyp2c37 | 0.04911 | -0.95795792 |
| Tmem81 | 0.21095 | -0.37569109 |
| Hhat | 0.53812 | 0.25039257 |
| Zfp367 | 0.04657 | 0.7261081 |
| 3110057O12Rik | 0.01013 | 0.72711834 |
| Mier3 | 0.00145 | 1.52337812 |
| Edf1 | 0.16743 | -0.51827864 |
| LOC102641261 | 0.08278 | -1.40101443 |
| Rab6a | 0.00193 | 0.92979809 |
| Triap1 | 0.1338 | -0.36948047 |
| Cypt12 | 0.09117 | -0.79777091 |
| Fam120a | 0.07994 | 0.34206806 |
| Rnf113a2 | 0.01875 | 1.05600628 |
| Cml2 | 0.67797 | -0.07758098 |
| Gm27199 | 0.00144 | -1.53904003 |
| Smim7 | 0.14176 | 0.26279304 |
| Sephs2 | 0.00343 | -0.92843365 |
| Capn2 | 0.40315 | 0.14163496 |
| Ptger4 | 0.99963 | -0.00014936 |
| Icosl | 0.78407 | 0.09233889 |
| Imp3 | 0.07843 | -0.54132811 |
| Gm33721 | 0.00145 | -1.2388121 |
| Naa11 | 0.00144 | -1.31884045 |
| Cideb | 0.93358 | 0.01543147 |
| Creb3l1 | 0.01521 | -0.63252303 |
| Tll1 | 0.53518 | 0.16761463 |
| Hoxa9 | 0.00144 | -1.41788339 |
| Sox2 | 0.01514 | -0.95990192 |
| Thbs1 | 0.01957 | 2.12549957 |
| Runx1 | 0.00298 | 0.95715468 |
| Clock | 0.09275 | 0.4631863 |
| Nr1i3 | 0.01203 | -1.94959834 |
| Pdpn | 0.00649 | -0.75206748 |
| F9 | 0.00144 | -1.28116048 |
| Mthfd2 | 0.01073 | -0.85523514 |
| Pgk1 | 0.44085 | -0.14890311 |
| Dip2a | 0.62355 | -0.21933987 |
| Sh3pxd2a | 0.72609 | -0.2024445 |
| Ncor2 | 0.0042 | -1.0856395 |
| Xaf1 | 0.74035 | 0.10064373 |
| Adgb | 0.00144 | -1.28898079 |
| Ppnr | 0.02829 | 0.43918223 |
| Agmo | 0.00144 | -1.37833222 |
| Cxcr4 | 0.34611 | 0.36270026 |
| Epo | 0.00144 | -1.29085005 |
| Cbx1 | 0.00144 | 1.24141346 |
| Pde6b | 0.17124 | -2.61877392 |
| Sh3bp1 | 0.04576 | -0.41990833 |
| Glrb | 0.06779 | -2.34221751 |
| Myl10 | 0.00144 | -1.31495282 |
| H2-Q7 | 0.02229 | -1.02129593 |
| Xlr | 0.0041 | 0.91910758 |
| Nnmt | 0.01724 | -0.69979738 |
| Gt(ROSA)26Sor | 0.97438 | 0.00918803 |
| Smad4 | 0.11421 | 0.63026538 |
| Gdnf | 0.00144 | -1.28116048 |
| Wnt10b | 0.00144 | -1.28116048 |
| Gfi1 | 0.00144 | -1.33333333 |
| Zfp82 | 0.00144 | -1.99443376 |
| Elavl3 | 0.524 | -0.56089149 |
| Gm30948 | 0.69859 | 0.21599077 |
| Myb | 0.00144 | -1.27165604 |
| Max | 0.37336 | -0.15792124 |
| Hyou1 | 0.01374 | -0.81613203 |
| Itpr1 | 0.06857 | -0.48259135 |
| Amelx | 0.08882 | -0.61770108 |
| a | 0.06327 | -0.59582805 |
| Ptpn6 | 0.16379 | -0.64528989 |
| Amd2 | 0.06242 | -0.47869936 |
| Kitl | 0.12177 | 0.35509395 |
| Epha3 | 0.12668 | -0.42221316 |
| H2-T22 | 0.00296 | -0.96704189 |
| Igh-VJ558 | 0.00144 | -1.31495282 |
| Lyst | 0.00144 | -1.28116048 |
| Sin3b | 0.00144 | -1.30017714 |
| Nfkb1 | 0.06045 | 0.33920404 |
| Fas | 0.92455 | -0.03908992 |
| P2ry2 | 0.02872 | -1.2867124 |
| Epb4.1 | 0.01527 | 0.69835917 |
| Sh2d1a | 0.00145 | -1.22518835 |
| Becn2 | 0.00144 | -1.38381884 |
| Trpa1 | 0.00927 | -1.2698525 |
| Adam26a | 0.04887 | -1.17412126 |
| Amhr2 | 0.28594 | -1.81517902 |
| Amhr2 | 0.10606 | -1.96326414 |
| Klb | 0.00246 | -1.38358758 |
| Pla2g10 | 0.00144 | -1.30017714 |
| Nppa | 0.052 | -1.11313983 |
| Fer1l5 | 0.02985 | -1.00328201 |
| Mirc35hg | 0.04073 | -0.39268576 |
| Npas3 | 0.27066 | -0.4084766 |
| C4a | 0.00144 | -1.29666168 |
| Ppargc1a | 0.07616 | 0.34806736 |
| Bola1 | 0.00161 | -1.55754919 |
| Bola1 | 0.00576 | -1.49911595 |
| Gm884 | 0.01801 | -0.96799104 |
| Gm884 | 0.00502 | -1.51651905 |
| Gm884 | 0.04023 | -1.90934143 |
| Htr4 | 0.01194 | -0.75983815 |
| Jpx | 0.08501 | -0.74293956 |
| Nlrp6 | 0.00733 | -1.32164453 |
| Chrng | 0.01316 | -1.25311652 |
| Mybpc1 | 0.02042 | -0.67872454 |
| Mybpc1 | 0.00627 | -1.47007978 |
| Olr1 | 0.00144 | -1.26911831 |
| Shank3 | 0.03538 | -0.84594536 |
| Shank3 | 0.34249 | 0.35372375 |
| Nexn | 0.00144 | 1.65678033 |
| Sun1 | 0.01935 | -0.89532722 |
| Sun1 | 0.12177 | -1.06469295 |
| Scyl1 | 0.00723 | -0.76678795 |
| Rltpr | 0.55654 | -0.17207433 |
| Ager | 0.49058 | -0.56712561 |
| Ager | 0.07875 | -0.97173986 |
| Gabrb2 | 0.06085 | -1.64704352 |
| Tet2 | 0.00864 | 0.66776399 |
| Smad4 | 0.02209 | -0.86762088 |
| Pparg | 0.70086 | -0.12930344 |
| Scn5a | 0.95031 | -0.01513596 |
| Unc79 | 0.00144 | -1.31495282 |
| Dach2 | 0.00144 | -1.28116048 |
| Hdgfrp2 | 0.85972 | 0.04846777 |
| Gabpa | 0.14814 | 0.24625029 |
| Cdk17 | 0.2195 | 0.3502064 |
| Elk3 | 0.09099 | -0.38785003 |
| Elk1 | 0.02397 | -0.6539699 |
| Rfx6 | 0.23805 | -0.63794779 |
| Sox9 | 0.03494 | -0.57940645 |
| Cacna1b | 0.00144 | -1.29085005 |
| Raet1b | 0.49742 | -0.74851887 |
| Prkaa2 | 0.06614 | 0.58145382 |
| Park2 | 0.2039 | -0.57838703 |
| Kcnq1 | 0.00144 | -1.30713006 |
| Kcnq1 | 0.03311 | -0.95922871 |
| Ghrl | 0.15998 | -0.55306558 |
| Trpc3 | 0.00144 | 1.7455684 |
| Adarb1 | 0.12975 | -0.60417221 |
| Tert | 0.08365 | -0.53248048 |
| Ildr2 | 0.00207 | -1.12931932 |
| Nfatc4 | 0.00921 | -0.91953399 |
| Kdr | 0.00145 | -1.22376446 |
| Vegfc | 0.03768 | 0.55582341 |
| Vegfc | 0.11294 | 0.41945621 |
| Nox4 | 0.13555 | -1.56530808 |
| Mxra8 | 0.00493 | -0.84174805 |
| Fbxl21 | 0.08086 | -0.45662213 |
| Cdh23 | 0.03488 | -1.00416365 |
| Prrxl1 | 0.00896 | -0.83507686 |
| Hrh4 | 0.0017 | -1.24950944 |
| Irf3 | 0.05969 | -0.42537971 |
| 4930444F02Rik | 0.01352 | -0.99237968 |
| 4930444F02Rik | 0.00336 | -1.1857878 |
| 4930444F02Rik | 0.00144 | -1.31518407 |
| 4930444F02Rik | 0.00144 | -1.29085005 |
| Hk1 | 0.00144 | -1.29746368 |
| Smarce1 | 0.3229 | 0.16021464 |
| Gm21992 | 0.88737 | -0.04588166 |
| Gm29904 | 0.90108 | -0.13943754 |
| Erbb2ip | 0.02032 | -0.96811802 |
| Tmprss6 | 0.99628 | -0.00431913 |
| Setdb2 | 0.28259 | 0.342556 |
| Phf11c | 0.00177 | 1.35218348 |
| Skint8 | 0.00218 | -1.63746178 |
| Skint1 | 0.13176 | -0.81410185 |
| Ccdc136 | 0.01548 | -0.69790437 |
| Ecscr | 0.01099 | -0.63335862 |
| Strn3 | 0.34595 | 0.17326852 |
| Il20ra | 0.01982 | -1.20853483 |
| Tnip3 | 0.80658 | 0.12307794 |
| Skint7 | 0.00144 | -1.30017714 |
| Skint4 | 0.06992 | -2.32732521 |
| Brinp3 | 0.00144 | -1.29878664 |
| Sp1 | 0.01606 | -1.4796658 |
| Oprm1 | 0.01126 | -1.15703151 |
| Cnnm1 | 0.44974 | -0.50385641 |
| Epdr1 | 0.91764 | 0.02949699 |
| Mapkapk3 | 0.02104 | -0.70627043 |
| Aqp6 | 0.06305 | -0.51477351 |
| Art3 | 0.16346 | 0.26033963 |
| Rpain | 0.29252 | -0.32917316 |
| Il21 | 0.32361 | -0.3673075 |
| Dmrt1 | 0.00184 | -0.89639791 |
| Art3 | 0.51635 | 0.12748079 |
| Art3 | 0.30891 | 0.20353177 |
| Rnf165 | 0.00144 | -1.29864962 |
| Dst | 0.65268 | -0.07853286 |
| Elavl4 | 0.00951 | -1.21265076 |
| Gata4 | 0.00439 | -1.63054609 |
| Shroom2 | 0.59171 | 0.11381436 |
| Treml2 | 0.66863 | 0.2245419 |
| Eif4e1b | 0.1631 | 0.54955774 |
| Pde6g | 0.54478 | 0.16485877 |
| Akap6 | 0.35722 | 0.56692669 |
| Eml4 | 0.04691 | -0.89142301 |
| Tex101 | 0.00144 | -1.20555712 |
| Gas8 | 0.57578 | 0.1086556 |
| Rsph1 | 0.32389 | 0.40571505 |
| Rgs7 | 0.14012 | 0.80509286 |
| Rgs7 | 0.40196 | 0.3411584 |
| Zdhhc18 | 0.0173 | -0.71673057 |
| 5930412G12Rik | 0.07949 | -0.72298547 |
| Treml4 | 0.10885 | -0.65784048 |
| Treml1 | 0.00144 | -2.18882098 |
| Il15 | 0.13583 | 0.55143163 |
| Myh4 | 0.00144 | -1.26560808 |
| Myh4 | 0.00144 | -1.07221214 |
| Pinc | 0.01071 | -1.37945684 |
| Myh8 | 0.00461 | -1.08200933 |
| Myh3 | 0.03956 | -0.62142733 |
| Relb | 0.00879 | -0.68206178 |
| Rela | 0.02126 | -0.54152779 |
| Myh2 | 0.00144 | -1.27165604 |
| Tyr | 0.00144 | -1.27165604 |
| Six4 | 0.02733 | -0.99738805 |
| Lcp1 | 0.00679 | -1.00895123 |
| Chat | 0.96735 | -0.02261252 |
| Chat | 0.06409 | -0.74930917 |
| Chat | 0.08913 | -0.81098647 |
| Chat | 0.01976 | -1.5163329 |
| Farsa | 0.56273 | -0.21522087 |
| Sp100 | 0.2322 | 0.25788998 |
| Fam219a | 0.00556 | -0.71835481 |
| Flnc | 0.29508 | -0.51587264 |
| Tmem253 | 0.97726 | -0.020966 |
| Gpi1 | 0.99701 | -0.00187466 |
| Rbbp8nl | 0.01527 | -1.04033847 |
| Gm815 | 0.03051 | -1.06721129 |
| Sarnp | 0.01705 | 0.69473393 |
| Hfm1 | 0.00266 | -1.73619554 |
| Gm853 | 0.04404 | -1.10057162 |
| Prss53 | 0.40149 | -0.14013792 |
| Ccdc60 | 0.39461 | -0.44294917 |
| Tex40 | 0.00501 | -1.08747569 |
| Oxld1 | 0.01913 | -0.7715616 |
| Ms4a15 | 0.00144 | -1.27165604 |
| Sult2a6 | 0.00149 | -1.22582153 |
| Gm94 | 0.85687 | -0.10990182 |
| Slc24a4 | 0.84578 | -0.13649977 |
| Phyhd1 | 0.01955 | 0.67039757 |
| Fam196a | 0.01578 | 1.06703676 |
| Pik3r4 | 0.06007 | 0.5018232 |
| Nek1 | 0.00144 | 2.5826024 |
| Adgrd1 | 0.78183 | -0.08782733 |
| Epb4.1l3 | 0.02465 | 0.58153519 |
| Urb2 | 0.26681 | 0.20154215 |
| Tbc1d8b | 0.00144 | 1.49708966 |
| Ralgapa2 | 0.29084 | -0.41717959 |
| Yeats2 | 0.00332 | 1.86408847 |
| Otop3 | 0.13758 | 0.67731991 |
| Rint1 | 0.03399 | 0.44424029 |
| Abca7 | 0.00645 | -0.62450778 |
| Hecw2 | 0.01183 | -0.58769525 |
| Cisd3 | 0.00739 | -0.76960336 |
| Gm5897 | 0.03113 | -1.52448895 |
| Rnf224 | 0.41408 | -0.25557098 |
| Gm6034 | 0.00144 | -1.42017585 |
| Pabpn1l | 0.00159 | -1.26431393 |
| Lrig1 | 0.45389 | 0.39384797 |
| Rsad1 | 0.32503 | -0.19451781 |
| Gm813 | 0.00144 | -1.28898079 |
| Hcst | 0.00358 | -1.0577094 |
| 8030411F24Rik | 0.43627 | -0.53912799 |
| Triml1 | 0.00144 | -1.28898079 |
| Zdhhc23 | 0.00145 | -1.22376446 |
| Zfp329 | 0.00144 | 2.14430767 |
| Lipo1 | 0.00144 | 1.95882204 |
| Cnn1 | 0.41165 | -0.41493368 |
| Krtdap | 0.00144 | -1.29085005 |
| Cdh7 | 0.00144 | -1.26934956 |
| Irf7 | 0.00224 | -1.16676879 |
| Cfd | 0.91084 | -0.16608125 |
| Klrb1b | 0.01133 | -1.54943941 |
| Kncn | 0.28498 | -0.85826122 |
| BC023829 | 0.18793 | 0.62123447 |
| Epha6 | 0.10143 | -2.82439527 |
| Edem3 | 0.87018 | -0.07643939 |
| Etnk2 | 0.13653 | -0.57386952 |
| Epha3 | 0.22831 | -1.63972566 |
| Tpd52l1 | 0.21645 | 0.74772213 |
| Spata5 | 0.33008 | 0.15837687 |
| Fev | 0.00929 | -2.88138659 |
| Plce1 | 0.04504 | 0.40524229 |
| Rasgef1a | 0.01769 | -0.74785041 |
| Klhl29 | 0.00254 | -1.1839779 |
| Ctnnd2 | 0.00167 | -1.30945677 |
| A230046K03Rik | 0.00144 | 2.99624309 |
| Fiz1 | 0.18263 | -0.38775162 |
| Pcnp | 0.04983 | 0.38309028 |
| 4930474N05Rik | 0.00149 | -1.22582153 |
| Ptpn7 | 0.04959 | -0.84862089 |
| Rere | 0.15957 | 0.30653828 |
| Gp6 | 0.00144 | -1.28116048 |
| Gm5108 | 0.00144 | -1.30713006 |
| Tbc1d12 | 0.00199 | 1.44857929 |
| Adcy6 | 0.61243 | -0.09446837 |
| Fosb | 0.04003 | -2.03108343 |
| Ankk1 | 0.00144 | -1.30713006 |
| Pld6 | 0.00144 | -1.28898079 |
| Samd11 | 0.68105 | 0.11260997 |
| Prelid2 | 0.49666 | -0.14210474 |
| Ccl4 | 0.07796 | -1.50499998 |
| Pla2g4e | 0.12109 | 0.86462468 |
| Mau2 | 0.03642 | -0.64320312 |
| Rgr | 0.00144 | -1.20579466 |
| Rasef | 0.03399 | -0.80099288 |
| Tas1r2 | 0.13022 | -0.82431196 |
| Arv1 | 0.00217 | 0.99441297 |
| Efcab8 | 0.00144 | -1.41534266 |
| Fgf17 | 0.07645 | -0.88534853 |
| Psma8 | 0.52717 | -0.2010557 |
| Igsf1 | 0.4968 | -0.32319845 |
| Exd1 | 0.49948 | -0.36524533 |
| Cthrc1 | 0.51153 | -0.284542 |
| Cnih2 | 0.00144 | -1.26042915 |
| Ikzf4 | 0.33955 | -0.73502715 |
| Klhl4 | 0.00157 | 1.41698719 |
| Mup21 | 0.01808 | -1.54979812 |
| Cep170b | 0.10341 | -0.48202773 |
| Dock4 | 0.69608 | -0.08523827 |
| Ptpn20 | 0.00144 | -1.28116048 |
| Snph | 0.00144 | -1.87721205 |
| Adamtsl1 | 0.14082 | -0.52553276 |
| Camsap2 | 0.26448 | 0.40783565 |
| Ttll7 | 0.06278 | 0.91610428 |
| Trim7 | 0.01122 | 1.62390896 |
| Pola1 | 0.004 | 1.2506124 |
| Synpo2l | 0.98617 | 0.00736415 |
| Slc26a11 | 0.41047 | -0.15345359 |
| Sv2c | 0.00886 | -1.55709779 |
| Kif26b | 0.04555 | -1.50188002 |
| Fbxl16 | 0.95785 | -0.02794386 |
| Plcxd3 | 0.13672 | 0.27606209 |
| Ighmbp2 | 0.00667 | -0.66466326 |
| Odam | 0.00144 | -1.30713006 |
| Wdr86 | 0.04147 | -1.16332038 |
| Pik3c2b | 0.21048 | -0.23022753 |
| Slc37a1 | 0.00144 | -1.26911831 |
| Polh | 0.34681 | -0.99134851 |
| Timm44 | 0.04139 | 0.57192224 |
| Myh9 | 0.02548 | -0.49419385 |
| 9230110C19Rik | 0.07373 | 0.61892875 |
| Pcdh12 | 0.0407 | -0.64284768 |
| Pik3cb | 0.00959 | 0.75063103 |
| Pcp4l1 | 0.84147 | -0.09928997 |
| Slc16a7 | 0.00144 | 1.38369084 |
| Nipal1 | 0.00144 | -1.31495282 |
| Col17a1 | 0.01891 | -1.06983578 |
| Galnt16 | 0.53993 | -0.33573183 |
| Phyhipl | 0.24326 | -0.80387964 |
| Phxr1 | 0.55228 | 0.43846175 |
| Nanos3 | 0.00144 | -1.36068408 |
| Gdpd5 | 0.38197 | -0.18836932 |
| Muc19 | 0.93806 | 0.02994797 |
| Enpp3 | 0.31713 | -0.66387074 |
| Ispd | 0.01044 | -1.7162878 |
| Glipr1l2 | 0.58843 | -0.88148784 |
| Ganab | 0.97596 | 0.00784687 |
| Fam155a | 0.00144 | -1.20555712 |
| B3glct | 0.11042 | -0.57522639 |
| Tmem26 | 0.00144 | -1.26560808 |
| Nsun7 | 0.72513 | -0.61466143 |
| Ssu2 | 0.16514 | -0.43732975 |
| Hdx | 0.00471 | -1.51793578 |
| 2300009A05Rik | 0.22888 | -0.33055477 |
| Pdzd2 | 0.39982 | -0.14460511 |
| Ttll11 | 0.26318 | -0.73966501 |
| 1810022K09Rik | 0.86549 | -0.04839405 |
| Kcnk6 | 0.05087 | 0.49139402 |
| Itga10 | 0.00144 | 2.18143685 |
| Kif9 | 0.22204 | -0.54049791 |
| Neil2 | 0.03143 | -0.6395747 |
| Slc35e1 | 0.10504 | -0.52254343 |
| Rhbdl2 | 0.21113 | -0.60594129 |
| Scd1 | 0.05935 | -0.69870396 |
| Crlf1 | 0.00556 | -0.83488742 |
| 3110062M04Rik | 0.58746 | 0.23533709 |
| Csf2rb | 0.73178 | -0.12831263 |
| Cdh22 | 0.17433 | -0.60347049 |
| Cd209a | 0.5082 | -0.59757914 |
| Vps37a | 0.02351 | -2.15298487 |
| Cr2 | 0.00616 | -1.01044586 |
| Lce1m | 0.00145 | -1.22193674 |
| Tacc2 | 0.08123 | -0.38345895 |
| Rap1gap2 | 0.01241 | -1.28998455 |
| Cd300lb | 0.18596 | -0.7433352 |
| Slc35d2 | 0.53987 | -0.18431701 |
| Krt16 | 0.08133 | -0.36954342 |
| Apob | 0.00144 | -1.51500476 |
| Tsnaxip1 | 0.02477 | -1.01647779 |
| Smg5 | 0.92045 | 0.03288072 |
| Zfp608 | 0.2246 | -0.31801257 |
| Hjurp | 0.78948 | -0.07408662 |
| Klhl4 | 0.73148 | -0.83012076 |
| Sprr2h | 0.00151 | -1.17792995 |
| Derl3 | 0.996 | -0.0028684 |
| Svil | 0.00144 | -1.20141019 |
| 4933439C10Rik | 0.03072 | 1.13551054 |
| D830044I16Rik | 0.35599 | -0.18290801 |
| 1700021L22Rik | 0.00279 | -0.99821741 |
| Fbxo47 | 0.64706 | -0.29866743 |
| Tpo | 0.02577 | -0.98550712 |
| Fn1 | 0.00603 | -1.88630195 |
| N6amt1 | 0.00656 | 2.01718932 |
| Irs3 | 0.00588 | -1.01309816 |
| 1700006J14Rik | 0.01417 | -2.80544436 |
| Ube2g1 | 0.05247 | 0.42247339 |
| Iqsec1 | 0.00425 | -0.75659975 |
| Snhg8 | 0.00144 | -1.15704508 |
| Sipa1l1 | 0.18237 | 0.6654965 |
| Ror1 | 0.11874 | -0.54197809 |
| Abcc9 | 0.5656 | -0.30380203 |
| Pou6f2 | 0.90277 | -0.07413081 |
| LOC102640359 | 0.71192 | 0.12980365 |
| 1500004A13Rik | 0.02943 | -0.75653351 |
| Cfap57 | 0.00257 | -1.44633387 |
| Wdr83os | 0.42157 | -0.30918673 |
| Dhx9 | 0.43437 | -0.17732355 |
| Ubr3 | 0.00776 | 0.69936059 |
| D930016D06Rik | 0.00158 | 2.6949716 |
| Arid1a | 0.0875 | -0.33712693 |
| Zic3 | 0.00144 | -1.22925777 |
| Adgrv1 | 0.00544 | -1.10518964 |
| Ncoa7 | 0.72803 | 0.13385042 |
| Cnot6 | 0.63948 | 0.11341191 |
| Dhx57 | 0.00145 | 0.93133261 |
| Fbxo28 | 0.0558 | 0.34114773 |
| 3110043O21Rik | 0.24607 | 0.22240869 |
| Syne2 | 0.10626 | 0.29316324 |
| Prdm15 | 0.01125 | 0.56676402 |
| Huwe1 | 0.04194 | -1.60159213 |
| Hmga2 | 0.68559 | -0.10425955 |
| Katnal2 | 0.06403 | -0.45401315 |
| Hmg20a | 0.02113 | 0.78453947 |
| 3110006O06Rik | 0.12397 | -2.36300167 |
| Slc38a1 | 0.77852 | 0.07617239 |
| Il31ra | 0.01511 | -1.65352598 |
| Zfp825 | 0.00144 | 1.93874854 |
| Dennd4b | 0.12483 | -0.46257551 |
| Dcbld2 | 0.00854 | 0.75914873 |
| Zfp777 | 0.00609 | -0.78326609 |
| Zfp961 | 0.61061 | -0.21691801 |
| 2310045N01Rik | 0.12409 | -0.42223717 |
| Klra8 | 0.00144 | 1.97010636 |
| Klhl10 | 0.83055 | 0.07884556 |
| Fitm2 | 0.26793 | -0.46948171 |
| Usp29 | 0.00144 | -1.28116048 |
| D930015E06Rik | 0.32941 | -0.18916484 |
| Gm38758 | 0.00144 | 1.42884444 |
| A430035B10Rik | 0.00804 | -1.75453858 |
| Myo5c | 0.14163 | -0.47327107 |
| Smarcc2 | 0.00465 | -0.74256651 |
| Pik3c2g | 0.05154 | -1.47092209 |
| Fxyd7 | 0.0638 | 1.82456284 |
| Sys1 | 0.01139 | -0.75650407 |
| Sdhaf2 | 0.08442 | -1.62707947 |
| Osbp2 | 0.00316 | -0.8441642 |
| Cenpn | 0.2191 | 0.2294024 |
| Tsc22d2 | 0.00772 | 0.6608034 |
| Ganc | 0.02801 | 0.63068922 |
| Tex2 | 0.06797 | 0.64726482 |
| Ptpn5 | 0.00147 | -1.15996015 |
| Pcnx | 0.22777 | 0.39712275 |
| Cep170 | 0.0576 | -0.3830562 |
| Myt1l | 0.00144 | -1.28898079 |
| Scml2 | 0.00144 | -1.29085005 |
| Rhot1 | 0.46535 | -0.19927309 |
| Msl1 | 0.47844 | -0.21371349 |
| Cntn1 | 0.00144 | -1.24811894 |
| Robo2 | 0.36009 | 0.51293564 |
| Ppp2r2a | 0.13798 | 0.66962332 |
| 0610030E20Rik | 0.02509 | -0.91788021 |
| Ttc14 | 0.00477 | 1.92809388 |
| Mapk15 | 0.06586 | -0.35242606 |
| 1200014J11Rik | 0.86974 | -0.02814477 |
| Slc22a23 | 0.17615 | -0.25092692 |
| Nek5 | 0.06857 | -0.87718068 |
| Pbld1 | 0.00144 | -1.27699683 |
| Cd226 | 0.04698 | -0.91567309 |
| Paqr3 | 0.00144 | -1.23155796 |
| Rtca | 0.48208 | 0.15196707 |
| Fancg | 0.04749 | -0.4054644 |
| Zeb2 | 0.51404 | 0.42151416 |
| LOC105244102 | 0.00739 | -1.12870827 |
| Tex37 | 0.00145 | -1.22376446 |
| Enpp6 | 0.14899 | -0.48584895 |
| BC049692 | 0.08859 | -3.1658599 |
| 2410015M20Rik | 0.00144 | -1.12987589 |
| Hnrnph3 | 0.0026 | 0.85337106 |
| 1700016H13Rik | 0.0067 | -1.32599123 |
| Mok | 0.00148 | -1.38051317 |
| BC049265 | 0.00144 | -1.28116048 |
| Daam1 | 0.19727 | 0.21336765 |
| 1700029B22Rik | 0.00144 | -1.30713006 |
| Morn1 | 0.00144 | -1.26560808 |
| Fam204a | 0.45442 | 0.19568159 |
| Hrasls | 0.0915 | -0.38964361 |
| Myh7b | 0.93842 | 0.04637327 |
| Csnk2a2 | 0.86701 | -0.04380607 |
| Gm16120 | 0.00144 | -1.29049509 |
| 0610031O16Rik | 0.00144 | -1.26560808 |
| Trak1 | 0.00144 | -1.31495282 |
| Agl | 0.51552 | 0.31037725 |
| Herc2 | 0.00149 | 0.86778353 |
| Atp1a3 | 0.0232 | -0.6957503 |
| Zc3h12c | 0.00144 | -1.26560808 |
| Scaf4 | 0.02976 | 1.21605239 |
| Dock3 | 0.00774 | -1.00810978 |
| A1cf | 0.00145 | -1.22376446 |
| Rragd | 0.88737 | -0.0417501 |
| Sdk2 | 0.12442 | -0.97070055 |
| Baz2a | 0.00242 | -0.90001899 |
| Ccnf | 0.63994 | -0.13164106 |
| Vps13d | 0.07616 | 0.31592663 |
| Commd4 | 0.00671 | -0.71524082 |
| Iars2 | 0.88716 | 0.05313069 |
| Rho | 0.00144 | -1.2456552 |
| Slc26a3 | 0.00144 | -1.30713006 |
| Sh2d5 | 0.21623 | -0.33446542 |
| Plekha5 | 0.0017 | 1.04871908 |
| Suv39h2 | 0.00291 | 1.28885874 |
| Ncoa4 | 0.10527 | 0.31173671 |
| Dna2 | 0.05178 | -0.51175375 |
| Tnfrsf19 | 0.04277 | -1.14062679 |
| Lactbl1 | 0.09532 | -2.74891881 |
| Ugt1a6b | 0.73844 | -0.0760326 |
| Wfdc1 | 0.93639 | 0.04184057 |
| Itga6 | 0.00643 | 1.20754333 |
| Ccdc171 | 0.00144 | -1.30713006 |
| Ppp2r2a | 0.53816 | 0.10561534 |
| Calm1 | 0.35383 | 0.14364836 |
| Ttc14 | 0.18038 | 0.531367 |
| Eea1 | 0.14836 | 0.62839922 |
| Gon4l | 0.01353 | -0.60492805 |
| Psmg2 | 0.22956 | -0.27632126 |
| Napb | 0.50699 | 0.41715055 |
| Fubp1 | 0.01874 | 0.48335193 |
| Psen1 | 0.52172 | 0.25811572 |
| Gm11128 | 0.00144 | -1.53322165 |
| 3110057O12Rik | 0.00925 | 1.75317127 |
| Clca3a1 | 0.00248 | 1.69798796 |
| Zcchc14 | 0.01587 | -0.50856177 |
| Stambp | 0.2877 | -0.18380684 |
| Ggps1 | 0.64482 | 0.13187923 |
| Tmem8 | 0.00589 | -0.83167501 |
| Matn2 | 0.16126 | 0.46115583 |
| Tom1l1 | 0.03542 | 1.30186561 |
| Mettl8 | 0.09522 | 0.28824559 |
| Gnpat | 0.25473 | 0.48574467 |
| H2-T3 | 0.00526 | -0.82586272 |
| Nckap5 | 0.53709 | 0.21705468 |
| Kis2 | 0.00144 | -1.27992547 |
| Huwe1 | 0.03496 | 0.42012698 |
| Ptk2 | 0.1894 | -0.32448537 |
| Ghrh | 0.09387 | -1.04398572 |
| Chd8 | 0.69575 | 0.1479727 |
| Bpi | 0.00144 | -1.29051721 |
| Sp7 | 0.00144 | -1.25118919 |
| Aqp4 | 0.10238 | -0.43726572 |
| Aqp4 | 0.00144 | -1.27992547 |
| Grm8 | 0.00145 | -1.26219879 |
| D930015E06Rik | 0.01002 | -0.661542 |
| Alcam | 0.00144 | -1.29049509 |
| Pax6os1 | 0.67002 | -0.07598992 |
| Pax6os1 | 0.06425 | -1.74786068 |
| Mog | 0.21669 | -0.72814152 |
| Clca4a | 0.01356 | -1.74549754 |
| Xpo7 | 0.00145 | -1.2388121 |
| Adam23 | 0.04156 | -0.6128726 |
| Mid1 | 0.00144 | -1.26934956 |
| Mid1 | 0.07511 | -0.75828661 |
| Ikzf1 | 0.04255 | -1.09351215 |
| Lppr5 | 0.03538 | -4.28925232 |
| Scn8a | 0.04256 | -2.09278954 |
| Scn8a | 0.3711 | -0.60227183 |
| Scn8a | 0.05526 | -0.90222389 |
| Il31ra | 0.00144 | -1.28116048 |
| Il31ra | 0.61476 | 0.14897354 |
| Fcrls | 0.00356 | -1.47378786 |
| Gja1 | 0.38174 | 0.41070528 |
| Gja1 | 0.00144 | -1.44185726 |
| Gja1 | 0.0145 | 2.07363251 |
| Gja1 | 0.01507 | 1.84453811 |
| Gja1 | 0.91283 | 0.05268659 |
| Gja1 | 0.75945 | 0.13373938 |
| Nlrp5 | 0.37449 | -0.51758607 |
| H2-Ab1 | 0.01396 | -0.97190725 |
| Def6 | 0.02017 | -0.55583866 |
| Rassf5 | 0.27071 | -0.79158076 |
| Mro | 0.00144 | -1.30017714 |
| Mgam | 0.00144 | -1.29049509 |
| Clec18a | 0.05588 | 0.93917992 |
| Slc14a2 | 0.02825 | -3.10510323 |
| Tha1 | 0.72029 | -0.1474313 |
| Phf21a | 0.02916 | 0.59350921 |
| Muc3 | 0.00144 | -1.29878664 |
| Itsn1 | 0.85525 | -0.03437379 |
| Vegfa | 0.24491 | 0.21520349 |
| Clock | 0.489 | 0.25729798 |
| Pif1 | 0.19053 | -0.71636187 |
| Mtmr1 | 0.00827 | -0.70098725 |
| Mir155hg | 0.01134 | -1.02600084 |
| Dio3os | 0.01408 | -1.6434879 |
| Parp3 | 0.07333 | -0.63749552 |
| Scn3a | 0.00144 | -1.30713006 |
| Scn2a1 | 0.00149 | -1.22582153 |
| Sp6 | 0.14672 | -0.87874139 |
| Lingo1 | 0.15511 | -0.39291434 |
| Txndc5 | 0.00144 | -1.08899091 |
| Cspg4 | 0.28434 | -0.46879281 |
| Rnf130 | 0.58619 | 0.18016686 |
| Psd | 0.00144 | -2.29751765 |
| Trmt5 | 0.01458 | 0.82411948 |
| Pkd1 | 0.85139 | -0.0995014 |
| Ikzf1 | 0.01596 | -0.63534067 |
| Usp48 | 0.98391 | -0.00978431 |
| Zkscan3 | 0.14808 | 0.80489055 |
| Cux1 | 0.02692 | -0.45511626 |
| Fancm | 0.00144 | 1.67281749 |
| Ankrd26 | 0.01235 | 1.72848964 |
| Spidr | 0.53953 | 0.40304126 |
| Zfp521 | 0.13173 | -0.34328278 |
| Ppp6r3 | 0.79142 | 0.10841413 |
| Uvssa | 0.00283 | 2.50742792 |
| Zfp608 | 0.62998 | 0.08771696 |
| Fam149b | 0.00863 | 0.9345378 |
| Cpne1 | 0.02331 | 0.47195956 |
| Ap5z1 | 0.01121 | -0.53185216 |
| Ralgps1 | 0.01744 | 0.64575055 |
| Ncoa7 | 0.94933 | 0.02406798 |
| Kirrel3 | 0.19215 | -1.22298154 |
| Sez6l | 0.22144 | -0.64839131 |
| Nedd4l | 0.30173 | -0.4081648 |
| Zfp536 | 0.10852 | 0.31128053 |
| Ubap2l | 0.09582 | -0.38602722 |
| Rab14 | 0.25631 | -0.26239696 |
| Scn5a | 0.00334 | -1.34288208 |
| Nup93 | 0.19053 | -0.34222169 |
| Zfand6 | 0.25537 | 0.26928024 |
| Smad7 | 0.32805 | 0.19492089 |
| Itsn1 | 0.09873 | -0.27704776 |
| Mc2r | 0.02576 | -1.05616101 |
| Itsn1 | 0.09239 | -0.34044723 |
| Rian | 0.19152 | 0.90160045 |
| Dscaml1 | 0.0017 | -1.19401501 |
| Tln2 | 0.88662 | -0.05001786 |
| Plekhg2 | 0.10275 | 0.36894784 |
| Nudt6 | 0.34774 | -0.23414507 |
| Crhr1 | 0.00144 | -1.28116048 |
| Necap1 | 0.05629 | 0.38209615 |
| Ptprd | 0.00639 | 0.75993634 |
| Cyb5r3 | 0.25246 | -0.20105056 |
| Svil | 0.00268 | 1.11598583 |
| Kcnn1 | 0.00749 | -1.03299636 |
| Twsg1 | 0.84066 | 0.09832035 |
| Rnf17 | 0.00144 | -1.64611241 |
| Usp29 | 0.01712 | -1.22163927 |
| Nkx1-2 | 0.11159 | -2.25839569 |
| Tsc22d3 | 0.13139 | -0.30284914 |
| Add2 | 0.00257 | -1.1333102 |
| Pde6h | 0.36051 | -0.90051008 |
| Prok2 | 0.01892 | -1.76526189 |
| Rorc | 0.90948 | -0.0577311 |
| Kif1b | 0.49699 | -0.14702827 |
| Ankrd17 | 0.56001 | -0.18022985 |
| Il12b | 0.01342 | -1.75858879 |
| Unc13b | 0.00145 | 1.19011265 |
| Pou2f1 | 0.0618 | -0.33506011 |
| Herc2 | 0.00182 | 1.1740601 |
| Ciita | 0.02531 | -1.0328145 |
| C920025E04Rik | 0.00144 | -1.11764012 |
| Chrne | 0.00144 | -1.29049509 |
| Nkx2-6 | 0.31597 | -0.243204 |
| Traf2 | 0.11118 | -0.49599098 |
| Prdm9 | 0.00145 | 2.6636943 |
| Kif14 | 0.00144 | -1.28116048 |
| Swi5 | 0.37923 | -0.19833516 |
| Mtmr6 | 0.10588 | 0.31122055 |
| Acap2 | 0.9822 | -0.00536363 |
| Nyap1 | 0.07033 | -1.07341889 |
| H2-M5 | 0.00191 | -1.4076954 |
| Pou5f1 | 0.01283 | -0.62426419 |
| Sell | 0.00152 | -1.37779971 |
| Miat | 0.00144 | -1.29085005 |
| Miat | 0.00171 | -1.1206745 |
| Miat | 0.00144 | -1.27165604 |
| Vps13c | 0.02814 | -0.50499154 |
| Rnf125 | 0.00361 | 1.46923394 |
| Cacna1c | 0.00606 | -1.22779906 |
| Reln | 0.062 | -0.99010277 |
| Phf10 | 0.53596 | -0.1093854 |
| Dok7 | 0.00743 | -0.7966459 |
| Nox1 | 0.00144 | -1.29746368 |
| Klra17 | 0.77027 | -0.11601647 |
| Kcnmb2 | 0.1678 | 0.3960614 |
| Ptch1 | 0.42171 | -0.53107692 |
| Mark1 | 0.051 | 0.45075959 |
| Kcnn4 | 0.6748 | -0.22866524 |
| Fkbp1b | 0.38235 | -0.15728433 |
| Slc25a27 | 0.00144 | 1.56689623 |
| Stab2 | 0.03506 | -0.9858148 |
| Rsbn1 | 0.13336 | 0.40752871 |
| Incenp | 0.99666 | 0.00131861 |
| Erg | 0.00474 | 1.23109616 |
| Srrm3 | 0.00144 | -1.28898079 |
| Notch4 | 0.01107 | -0.69818951 |
| Cyth2 | 0.7797 | 0.11127284 |
| Cyth1 | 0.00197 | -1.67598728 |
| Epor | 0.40389 | -1.43206413 |
| Tnf | 0.7746 | -0.10143813 |
| Hpcal1 | 0.18949 | -0.25396789 |
| Ciapin1 | 0.0035 | -0.93092521 |
| Wdr26 | 0.0754 | 0.45853315 |
| Arl2 | 0.00218 | -0.9734224 |
| Acsf3 | 0.03211 | -0.5685613 |
| Trpm1 | 0.00249 | -1.33983982 |
| Gas5 | 0.29004 | 0.29203461 |
| Tbc1d9b | 0.00479 | -0.82976698 |
| Slc25a27 | 0.00183 | 1.94041936 |
| Lcmt1 | 0.79331 | -0.04765533 |
| Tgif2 | 0.58814 | 0.30583771 |
| Pskh1 | 0.0093 | -0.58915448 |
| Naa40 | 0.90945 | -0.07152036 |
| Mlf2 | 0.58843 | -0.24687223 |
| Morf4l1 | 0.53429 | 0.1477724 |
| Gorasp2 | 0.92962 | 0.03793564 |
| Clptm1 | 0.00144 | -1.10329696 |
| Usf2 | 0.55513 | -0.23538953 |
| Myo9b | 0.11363 | -0.72159661 |
| Tuba1c | 0.11783 | -0.50889013 |
| Hsp90aa1 | 0.5236 | -0.17941597 |
| Nckipsd | 0.06325 | -0.40207148 |
| Rnf213 | 0.39427 | -0.19899033 |
| Sorbs1 | 0.00853 | -0.67229569 |
| Flywch1 | 0.01139 | -0.7181994 |
| Srprb | 0.02469 | -0.92574091 |
| Xpo6 | 0.10665 | 0.74847446 |
| Sema4c | 0.04884 | -1.30089877 |
| Zfand6 | 0.01441 | -0.70225265 |
| Slc9a3r1 | 0.07663 | -0.44281429 |
| Pter | 0.01144 | 1.63896795 |
| Celf2 | 0.00529 | 0.71085613 |
| Sidt2 | 0.82524 | -0.05121956 |
| Sox10 | 0.01338 | -1.15947214 |
| Ist1 | 0.55308 | 0.20628235 |
| Hs2st1 | 0.01047 | 0.59154169 |
| Rab1b | 0.42696 | -0.32773801 |
| Mboat2 | 0.71517 | -0.10614535 |
| Pink1 | 0.68612 | 0.30236853 |
| Zdhhc16 | 0.48906 | -0.86055557 |
| Kctd3 | 0.21737 | -0.31756476 |
| Fbxw8 | 0.35572 | -0.35926699 |
| Tnfrsf19 | 0.00144 | -1.28898079 |
| Cnot10 | 0.28235 | 0.33850035 |
| Nrcam | 0.01784 | -1.041468 |
| Galns | 0.34507 | -0.2693283 |
| Ubqln4 | 0.14681 | -0.43337457 |
| Taf10 | 0.56036 | -0.12905191 |
| Trmt2a | 0.49245 | 0.18443804 |
| Zfp639 | 0.38553 | -0.3826329 |
| Lrch4 | 0.01514 | -0.75193512 |
| Gga3 | 0.02766 | -1.28488688 |
| Copz2 | 0.06319 | -0.46448719 |
| Apbb3 | 0.22526 | -0.26259114 |
| Clstn3 | 0.02016 | -1.03020085 |
| Mcl1 | 0.23358 | 0.20434407 |
| Cdc37 | 0.49771 | -0.22269556 |
| D230025D16Rik | 0.29661 | -0.21553652 |
| Sarnp | 0.86224 | -0.03116009 |
| Bag6 | 0.18586 | -0.46883643 |
| Rhbdd2 | 0.01818 | -1.27036902 |
| Erh | 0.23856 | 0.36541644 |
| Fam63b | 0.03334 | -0.44899442 |
| Gga2 | 0.78982 | 0.13547134 |
| Ptov1 | 0.395 | -0.45604962 |
| Uso1 | 0.00722 | 0.79364008 |
| Ip6k1 | 0.02646 | -0.57639071 |
| Mtx2 | 0.09091 | 0.65833168 |
| Itm2c | 0.00144 | -1.13315459 |
| Txnrd1 | 0.00144 | -1.17041693 |
| Msto1 | 0.00169 | -0.99403602 |
| Pde12 | 0.00149 | -0.86033996 |
| Aagab | 0.87038 | -0.08514208 |
| Slc39a7 | 0.00794 | -0.74676737 |
| Slc37a3 | 0.17362 | -0.81098647 |
| Fibp | 0.15968 | -0.40509487 |
| Surf2 | 0.01087 | 0.78625414 |
| Mtch1 | 0.09346 | -0.59075426 |
| 9530077C05Rik | 0.30402 | -0.41409195 |
| Wdr62 | 0.99479 | 0.00195091 |
| Eif1ax | 0.00144 | 1.99722455 |
| Rabl2 | 0.54872 | -0.28015672 |
| Tyrp1 | 0.13568 | -3.08669728 |
| Foxn3 | 0.66226 | -0.12212101 |
| Kansl1 | 0.30961 | -0.46387807 |
| Dzip1l | 0.19436 | -0.3744644 |
| Ap2s1 | 0.00144 | -1.24621808 |
| Arhgef10 | 0.0113 | -0.77130128 |
| Colgalt1 | 0.01017 | -0.59731748 |
| Cln5 | 0.00409 | -0.88694494 |
| Sh3kbp1 | 0.00225 | 0.90634586 |
| Cxxc1 | 0.07141 | -0.33819105 |
| Pigs | 0.82165 | 0.1200516 |
| Spata2 | 0.04115 | -0.80452028 |
| Ctage5 | 0.17934 | 0.24705948 |
| A830019P07Rik | 0.01232 | -0.87944093 |
| Ywhaq | 0.00837 | 0.64597728 |
| Trpm1 | 0.01696 | -1.12385295 |
| Pgs1 | 0.13086 | -0.33977717 |
| Tmem198b | 0.00182 | -1.10876194 |
| Appbp2 | 0.03894 | -0.39414891 |
| Zdhhc16 | 0.45028 | 0.22740819 |
| Afg3l2 | 0.00144 | -0.96007986 |
| Nrbp1 | 0.15058 | -0.25934586 |
| Fam134c | 0.21426 | -0.89997199 |
| Strip1 | 0.00177 | -1.10314433 |
| Chpf2 | 0.12748 | -0.32663784 |
| Rfng | 0.82025 | 0.24351311 |
| Tulp4 | 0.00318 | -0.75362924 |
| Zbtb43 | 0.9124 | 0.04190635 |
| Usp39 | 0.1662 | 0.47942203 |
| Tsen34 | 0.54069 | 0.1627816 |
| Fbln1 | 0.00144 | -1.12001718 |
| Crebl2 | 0.01317 | -0.77562688 |
| Serpinh1 | 0.01701 | -0.83361236 |
| Parl | 0.40783 | -0.18332687 |
| Itm2b | 0.03694 | -2.35225362 |
| Ube2d2a | 0.03381 | 0.69128134 |
| Gak | 0.82476 | -0.05699842 |
| Atp6v1b2 | 0.34949 | 0.17888226 |
| Polg | 0.87169 | -0.0575902 |
| Aebp1 | 0.01165 | -0.76074403 |
| Tbc1d9b | 0.96365 | -0.02927089 |
| Glyr1 | 0.00333 | -0.95925888 |
| Smug1 | 0.05087 | -0.56219622 |
| Sun1 | 0.32676 | 0.29942541 |
| Rab22a | 0.26218 | 0.46890761 |
| Ywhaq | 0.40236 | -0.13833486 |
| Eftud2 | 0.06277 | -0.93673464 |
| Nf2 | 0.01049 | -0.78458921 |
| Got1 | 0.32165 | 0.55533304 |
| Pygo2 | 0.00144 | -1.18957614 |
| Exoc2 | 0.11566 | -0.57405572 |
| Pkd1 | 0.21337 | -1.03747892 |
| Ednrb | 0.00577 | 0.79315348 |
| Ldlrad3 | 0.07355 | -1.38025955 |
| Nckipsd | 0.12657 | -0.98406593 |
| P4hb | 0.00145 | -1.26219879 |
| Morf4l1 | 0.5092 | -0.10547054 |
| Slc37a3 | 0.13726 | -1.55178706 |
| Sbk1 | 0.02869 | -0.80623507 |
| Flot2 | 0.15499 | -0.9130259 |
| Zfp276 | 0.01075 | -0.79062807 |
| Pkmyt1 | 0.13651 | -0.38488443 |
| Tmc6 | 0.00753 | -0.90578437 |
| Nupl1 | 0.21215 | 0.24009147 |
| Klhl30 | 0.0697 | -0.79042477 |
| Eno1b | 0.03594 | -0.45633622 |
| Wasf2 | 0.54729 | -0.0945166 |
| Speg | 0.00144 | -1.11134008 |
| Dkk3 | 0.12138 | -1.04776411 |
| Mthfd1 | 0.00144 | -1.7261908 |
| Faf1 | 0.51281 | 0.1774683 |
| Fam21 | 0.26805 | -0.22745543 |
| Ddr2 | 0.01669 | -1.62253068 |
| Srebf2 | 0.03088 | -0.46269971 |
| Usp19 | 0.02653 | -0.87144217 |
| Rad54l | 0.04821 | -0.72088054 |
| Rad23b | 0.72977 | -0.10648491 |
| Mapkapk2 | 0.00776 | -0.95903905 |
| Fkbp4 | 0.00887 | -0.80081959 |
| Neat1 | 0.00144 | 2.06131203 |
| Xpo6 | 0.21164 | 0.32719298 |
| Setd4 | 0.82329 | 0.12654042 |
| Bcl2l13 | 0.9229 | 0.01753236 |
| L3mbtl2 | 0.01683 | -0.55726617 |
| Smyd2 | 0.95265 | 0.01631761 |
| Pif1 | 0.00145 | -1.22376446 |
| Msantd2 | 0.47388 | 0.26293959 |
| Cited2 | 0.08655 | -0.60293863 |
| Snx12 | 0.00376 | -0.77726848 |
| Enthd2 | 0.15585 | -2.07627667 |
| Cdhr4 | 0.17501 | -1.44425655 |
| Rab11b | 0.79868 | -0.12308739 |
| Pcsk7 | 0.85624 | -0.03410693 |
| Slc38a10 | 0.98082 | 0.01359276 |
| Firre | 0.13746 | -0.72937653 |
| Tfeb | 0.01029 | -0.68326963 |
| Flad1 | 0.31788 | -0.41768332 |
| Arhgef25 | 0.73528 | -0.11428493 |
| Rhobtb3 | 0.1295 | -0.3037113 |
| Itpkc | 0.57905 | -0.17893174 |
| St3gal6 | 0.01537 | 0.66637253 |
| Ppp1r16a | 0.27174 | -0.54166572 |
| Gde1 | 0.01791 | -0.61424448 |
| Fads2 | 0.03246 | -0.71868822 |
| Ncdn | 0.01708 | -0.79901556 |
| Trpm1 | 0.00145 | -1.2388121 |
| Wisp1 | 0.28053 | -0.71810938 |
| Stim1 | 0.00549 | -1.19232539 |
| Pycr2 | 0.16634 | -0.47685037 |
| Tacc1 | 0.00662 | -0.64906537 |
| LOC100861738 | 0.00783 | -0.83992294 |
| Prc1 | 0.41167 | -0.44343821 |
| Tjap1 | 0.15162 | -0.42952381 |
| Arih2 | 0.35632 | 0.22494205 |
| Aph1a | 0.00396 | -0.9260886 |
| Trit1 | 0.33791 | -0.45830079 |
| Tyrp1 | 0.00144 | -1.30017714 |
| Mib2 | 0.00144 | -1.16077854 |
| Fam118a | 0.00492 | -1.6741226 |
| Abhd4 | 0.23921 | -0.58615317 |
| Tpd52l1 | 0.65712 | 0.1995813 |
| Hpcal1 | 0.2437 | -0.23092066 |
| Mettl9 | 0.01294 | 0.72904514 |
| Srpr | 0.00484 | -0.90434374 |
| Cdk10 | 0.77468 | 0.08041016 |
| Supt20 | 0.91563 | 0.06758183 |
| Ralgds | 0.15799 | -0.53052277 |
| Dclk2 | 0.11486 | -0.84118732 |
| Nacc1 | 0.13245 | -0.84656846 |
| Ddx56 | 0.73225 | -0.17668066 |
| Aldh16a1 | 0.25589 | -0.76305924 |
| Tars2 | 0.58906 | -0.22353395 |
| Prkaca | 0.00144 | -1.32424252 |
| Slc25a22 | 0.99278 | 0.00259394 |
| Triobp | 0.0235 | -0.53310779 |
| Nsun2 | 0.04917 | 0.61012938 |
| Zdhhc16 | 0.38596 | -0.28115134 |
| Dcaf10 | 0.06843 | -0.51568041 |
| St7 | 0.10294 | -0.64263995 |
| 4930402H24Rik | 0.42462 | -0.47417369 |
| Cdc14b | 0.00144 | -1.7626144 |
| Usp20 | 0.32921 | 0.39926348 |
| Dlst | 0.51396 | -0.16461101 |
| Sorbs1 | 0.05436 | -0.42832032 |
| Metap1d | 0.20788 | 0.29485986 |
| Cdkl3 | 0.23184 | 0.51828373 |
| Rbm7 | 0.00413 | 0.92728152 |
| Ncor1 | 0.40142 | 0.32198157 |
| Rbm39 | 0.01104 | -0.86649204 |
| Trmt2a | 0.24733 | -0.51222231 |
| Ccnh | 0.00645 | 1.63059898 |
| Samd4b | 0.09176 | -0.71920022 |
| Gtf2h1 | 0.33666 | 0.20798392 |
| Lsm4 | 0.07302 | -0.77077008 |
| Ube2g2 | 0.32894 | -0.51320066 |
| Ccdc97 | 0.40332 | -0.66590616 |
| Uba2 | 0.00351 | 0.79716706 |
| Pisd-ps3 | 0.00424 | 1.00851709 |
| Rbm42 | 0.3129 | -0.38590673 |
| Tacc2 | 0.01984 | -0.80296689 |
| Setd3 | 0.83845 | 0.03880302 |
| Osbpl5 | 0.40622 | 0.35627807 |
| Lrrc61 | 0.00144 | -1.41443942 |
| Rbms1 | 0.04446 | 0.38078445 |
| Prmt5 | 0.11126 | -0.32661983 |
| Mff | 0.01741 | 0.93774036 |
| Mrps2 | 0.67587 | 0.14964065 |
| Coro1b | 0.08151 | -0.64237993 |
| Tyms | 0.98128 | 0.00489972 |
| Ndufaf7 | 0.19992 | 0.44006794 |
| Xbp1 | 0.20584 | 0.42406699 |
| Cdc14b | 0.74673 | 0.18960937 |
| Acbd4 | 0.83189 | -0.1261617 |
| Paip2b | 0.24691 | 0.37264734 |
| Trpm4 | 0.04386 | -0.46713472 |
| Lrrc28 | 0.28323 | 0.42405546 |
| Mbd3 | 0.49761 | -0.28520898 |
| Rcbtb2 | 0.2769 | 0.21696707 |
| Rhobtb2 | 0.01423 | -1.43839551 |
| Sorbs3 | 0.01526 | -0.69046448 |
| Rad9a | 0.7637 | 0.09736841 |
| Bcar1 | 0.00451 | -1.6354985 |
| Ric8b | 0.00144 | 1.64739575 |
| Adck3 | 0.00144 | -1.30612874 |
| Prr14 | 0.85428 | 0.04826709 |
| Chmp6 | 0.13723 | -0.61059842 |
| Fbxo9 | 0.79084 | -0.05543438 |
| Zkscan3 | 0.36477 | 0.21809566 |
| Med22 | 0.00144 | -1.27699683 |
| Aprt | 0.00584 | -0.92291271 |
| Tmem234 | 0.03958 | -0.51648682 |
| Emc7 | 0.31588 | 0.41074572 |
| Prmt1 | 0.00509 | -0.80466168 |
| Appbp2 | 0.00297 | -0.77982267 |
| Trip13 | 0.01097 | -1.10038984 |
| Shisa4 | 0.00352 | -0.82740066 |
| Cyc1 | 0.00299 | -1.04285127 |
| Ubc | 0.05385 | -0.59125937 |
| Kif3c | 0.08224 | -0.94704583 |
| Pddc1 | 0.41091 | -0.39215468 |
| Aph1a | 0.02849 | -0.75530252 |
| Hdac11 | 0.94772 | -0.0356384 |
| Pja1 | 0.04409 | -1.11996532 |
| Mms19 | 0.24061 | -2.12902692 |
| Acsl4 | 0.36023 | 0.18909369 |
| Ogdh | 0.05649 | -0.49770707 |
| Abcf3 | 0.71508 | 0.11535243 |
| Pik3r2 | 0.66272 | -0.38844984 |
| Nap1l1 | 0.00455 | 0.97597347 |
| Oaz2 | 0.00144 | -1.25592773 |
| Slc3a2 | 0.39639 | -0.23907396 |
| Foxn3 | 0.66415 | 0.19150798 |
| Vps33b | 0.15324 | -0.75987879 |
| Dgcr8 | 0.69583 | -0.13490847 |
| Xylb | 0.08442 | 0.47287118 |
| Cdk5rap3 | 0.0017 | -1.24004482 |
| Pcbp4 | 0.49542 | -0.27640787 |
| Agap1 | 0.15783 | -0.73610771 |
| Fbxw11 | 0.00144 | -1.67988785 |
| Tmc6 | 0.00144 | -1.66920536 |
| Csad | 0.27544 | -0.29509188 |
| Tmem164 | 0.50132 | 0.30067217 |
| Eef2k | 0.48428 | -0.15174999 |
| Pank2 | 0.03056 | 0.45422352 |
| Tut1 | 0.89663 | 0.0282963 |
| Prmt2 | 0.52465 | -0.22547997 |
| Mapk12 | 0.30238 | -0.21527848 |
| Suclg2 | 0.00144 | -1.21952088 |
| Limd1 | 0.0197 | 1.01560102 |
| Klhdc2 | 0.26892 | 0.44718658 |
| Aldh2 | 0.07012 | -0.37229934 |
| Tmem181a | 0.65649 | -0.10639544 |
| Med17 | 0.25674 | 0.5427715 |
| Apbb3 | 0.31111 | 0.25897978 |
| Tcf25 | 0.0459 | -0.40972344 |
| Aldh9a1 | 0.03151 | 0.49481036 |
| Tmem106c | 0.00236 | -0.91443965 |
| Efr3a | 0.01797 | -0.85079595 |
| Bre | 0.93133 | -0.02466566 |
| Mpv17l2 | 0.08862 | -0.51611342 |
| Pradc1 | 0.52482 | 0.16626862 |
| Mpi | 0.63404 | -0.15223888 |
| Ttc13 | 0.06808 | -1.10429432 |
| Ctc1 | 0.59891 | -0.25702068 |
| Htra2 | 0.31085 | -0.37014173 |
| Zdhhc4 | 0.41047 | -0.23085182 |
| Fam133b | 0.01918 | -1.0068561 |
| Ms4a8a | 0.00653 | -1.60015618 |
| Tmem2 | 0.47373 | -0.67451824 |
| Galnt10 | 0.18189 | -0.64280792 |
| Cfap20 | 0.51889 | -0.12899817 |
| Tmem2 | 0.91661 | -0.0282806 |
| Camkmt | 0.00144 | -1.28898079 |
| Specc1l | 0.22555 | -0.34518637 |
| Peak1 | 0.5441 | -0.17020049 |
| a | 0.59365 | -0.32383803 |
| Uso1 | 0.22359 | 0.34963297 |
| Slc2a6 | 0.00149 | -1.39493641 |
| Man1a | 0.00144 | -1.27730451 |
| Suclg2 | 0.30846 | -0.48808288 |
| Cep68 | 0.33751 | -0.57263058 |
| Golga4 | 0.34782 | -0.36037892 |
| Larp4 | 0.09154 | 1.06147324 |
| Ints6 | 0.01798 | 1.58951048 |
| Atrx | 0.97059 | 0.01287609 |
| Rras2 | 0.31869 | -0.20595812 |
| Pacs1 | 0.7145 | -0.0831862 |
| Rnf111 | 0.94127 | 0.02046685 |
| Picalm | 0.02069 | 1.12589738 |
| Tmem143 | 0.03329 | 0.97558919 |
| Sipa1l1 | 0.0961 | -0.74967232 |
| 6030458C11Rik | 0.01137 | 0.60169633 |
| Mical1 | 0.23883 | -0.28270257 |
| Zfp653 | 0.20232 | -0.40945532 |
| Xdh | 0.35341 | -0.226856 |
| Tbc1d23 | 0.69192 | 0.06595122 |
| Jup | 0.73167 | -0.08252814 |
| Slc7a6os | 0.45091 | -0.46528259 |
| Irf5 | 0.66716 | -0.24417048 |
| Ppfibp2 | 0.50718 | -0.2561886 |
| F13a1 | 0.45353 | -0.26191921 |
| Bcl2l13 | 0.03478 | -1.45773424 |
| Acot11 | 0.01787 | -0.80354208 |
| Haao | 0.01595 | -0.97953315 |
| Trpv2 | 0.44164 | -0.19447109 |
| Qsox1 | 0.43721 | 0.18808076 |
| Pkm | 0.85371 | 0.1334488 |
| Pik3r6 | 0.00248 | 0.99942238 |
| Tsc2 | 0.99515 | -0.00341141 |
| Cabin1 | 0.49483 | 0.18139655 |
| Dennd1c | 0.095 | -0.77351977 |
| Akna | 0.40307 | -0.3468371 |
| Gprasp1 | 0.01491 | 1.92045508 |
| Uchl1 | 0.00144 | -0.96738326 |
| Cblb | 0.03165 | -0.60709109 |
| Zfp729b | 0.00144 | 1.80131326 |
| Zfp622 | 0.00401 | -0.94127829 |
| Tnfrsf1b | 0.0097 | -0.5680064 |
| Abcd1 | 0.19221 | -0.45722016 |
| LOC105243004 | 0.00229 | -1.13943754 |
| Sipa1 | 0.07723 | -0.59516072 |
| Pggt1b | 0.33445 | 0.27744404 |
| 4931406P16Rik | 0.01941 | 0.4902237 |
| Lpl | 0.032 | 0.63433537 |
| Ncor2 | 0.97924 | 0.00999 |
| Il6st | 0.07887 | -1.14604037 |
| Ehbp1l1 | 0.35762 | 0.35997957 |
| Itga1 | 0.00211 | 2.10594356 |
| Otud5 | 0.8381 | -0.03484321 |
| Cnot1 | 0.00636 | -0.85350321 |
| Psd4 | 0.98537 | 0.00743362 |
| Ikzf3 | 0.18454 | -0.82416259 |
| Acap1 | 0.00145 | -1.25646236 |
| Skap1 | 0.93451 | -0.05156809 |
| Hn1l | 0.30342 | 0.28670832 |
| Psma3 | 0.00655 | 0.66832349 |
| Rnf157 | 0.02173 | -1.20302799 |
| Ssfa2 | 0.01113 | 0.69982252 |
| Zxdc | 0.46539 | -0.20675172 |
| Wdfy2 | 0.11844 | -0.81098647 |
| Spata7 | 0.15323 | 0.77969982 |
| Smarcal1 | 0.04971 | -0.44069169 |
| Endou | 0.03002 | -0.51665607 |
| Anxa3 | 0.27491 | 0.206217 |
| Gss | 0.01914 | -0.65881646 |
| Bbs1 | 0.73463 | -0.08411158 |
| Mapre2 | 0.07631 | -0.81310408 |
| Errfi1 | 0.02987 | 0.89804141 |
| Ifih1 | 0.00915 | 1.2558513 |
| Helz2 | 0.00144 | -1.18486748 |
| Ccdc94 | 0.1048 | -0.33118977 |
| Mre11a | 0.17177 | 0.22584018 |
| Spc24 | 0.02258 | -0.6950761 |
| Golph3 | 0.02147 | -1.06017214 |
| Wdyhv1 | 0.86447 | -0.03543368 |
| Ogg1 | 0.02195 | -0.69965646 |
| Lig3 | 0.09925 | -0.70857039 |
| Chchd6 | 0.18796 | -0.54691676 |
| Glb1 | 0.10809 | 0.49133737 |
| Utp18 | 0.37917 | -0.38448169 |
| Rin3 | 0.16277 | 0.51505383 |
| Zc3hav1 | 0.67669 | 0.11642847 |
| Tmem104 | 0.31889 | 0.24048817 |
| Slc35b3 | 0.32326 | 0.53228239 |
| Tmem87b | 0.18981 | 0.68253135 |
| Sync | 0.00144 | -1.35521724 |
| Pbrm1 | 0.87785 | 0.03799203 |
| Atp9b | 0.46537 | -0.36347555 |
| Npr3 | 0.04588 | 0.90856692 |
| Notch4 | 0.00144 | -1.28898079 |
| Tbc1d9b | 0.04667 | -0.88534853 |
| Rnf130 | 0.10982 | -0.34094651 |
| Kcnma1 | 0.00455 | -1.33626376 |
| Ssh3 | 0.02824 | 0.41671814 |
| St13 | 0.0213 | -0.79642779 |
| Upf3a | 0.37809 | -0.32243977 |
| Rbm15 | 0.22488 | -0.28608589 |
| Diap3 | 0.00306 | -1.71990804 |
| Slc12a7 | 0.00197 | -1.26111664 |
| Mei4 | 0.00144 | -1.29085005 |
| Snx13 | 0.0538 | -0.81363126 |
| Dlc1 | 0.99305 | 0.00235339 |
| Wdr19 | 0.00159 | -1.64397313 |
| Rab31 | 0.01186 | 0.98365704 |
| Slc9a2 | 0.00144 | -1.27165604 |
| Pcnp | 0.37826 | 0.20642745 |
| Rtn2 | 0.00208 | -1.15498993 |
| Pvt1 | 0.02891 | -1.89143243 |
| 4930511M06Rik | 0.14392 | -0.29651012 |
| Otog | 0.00144 | -1.28898079 |
| Gm12296 | 0.00144 | -1.27165604 |
| Map1b | 0.16416 | 0.37335111 |
| Mapk12 | 0.05629 | -0.56183648 |
| Wrn | 0.00144 | -1.30713006 |
| Chtf8 | 0.00144 | -1.16499661 |
| Lyst | 0.20838 | 0.89014395 |
| Prune2 | 0.04037 | -0.7257654 |
| Vps26b | 0.03549 | -1.0709793 |
| Slc17a3 | 0.00144 | -1.28898079 |
| Tubgcp3 | 0.97407 | -0.01297135 |
| Tyk2 | 0.00281 | -1.88309826 |
| Spats1 | 0.03418 | 0.90121581 |
| Sirt3 | 0.48009 | 0.27758826 |
| Slc7a13 | 0.00144 | -1.30017714 |
| Gm15638 | 0.00144 | -1.29085005 |
| Cpsf7 | 0.97966 | 0.01726538 |
| Rps6kb1 | 0.00144 | -1.41658554 |
| Fam135a | 0.63104 | -0.2237266 |
| Gm38575 | 0.00144 | -1.26560808 |
| Herc2 | 0.00296 | -1.19248721 |
| Tctn2 | 0.4748 | 0.33936954 |
| Tnfsf13b | 0.6318 | 0.17731811 |
| Fndc7 | 0.01712 | -1.06950675 |
| Hspa12a | 0.05943 | -0.66743529 |
| C130026L21Rik | 0.00158 | -1.14134209 |
| Arih2 | 0.72162 | -0.12780017 |
| Uvrag | 0.00398 | 1.31255019 |
| Kdm6a | 0.24791 | -0.36089324 |
| Cdk12 | 0.01079 | 1.11034212 |
| Cpeb2 | 0.16267 | 1.02244782 |
| Glis1 | 0.05139 | -1.13195841 |
| Map2 | 0.14811 | -1.04697165 |
| Nup98 | 0.00176 | -0.94675425 |
| Abhd4 | 0.0513 | -0.98364225 |
| Malt1 | 0.01843 | 1.07071082 |
| Sdad1 | 0.87569 | 0.04209781 |
| Brca2 | 0.01352 | 0.96558111 |
| Hmmr | 0.00364 | -1.67596867 |
| Dhx29 | 0.07364 | -0.66666667 |
| Cacna2d1 | 0.04305 | 0.52771219 |
| Tmco6 | 0.00809 | -0.80495342 |
| Ankrd11 | 0.0097 | -0.69561381 |
| Tmem87b | 0.63293 | -0.3287553 |
| Psmb2 | 0.12229 | 0.42473979 |
| Usp15 | 0.04155 | 0.98227056 |
| Nrp1 | 0.03534 | 1.88560252 |
| Mbnl2 | 0.22981 | 0.45581289 |
| Hlcs | 0.06986 | -0.87092771 |
| Pid1 | 0.0242 | 1.08584844 |
| Clasp2 | 0.17639 | 0.39213451 |
| Midn | 0.00144 | -1.0968017 |
| Nipbl | 0.91425 | 0.06213771 |
| Sema3a | 0.16864 | -0.47721316 |
| Klhl36 | 0.00248 | -0.89633932 |
| Extl1 | 0.02531 | -1.15591755 |
| Jarid2 | 0.00485 | -1.27779779 |
| Dpagt1 | 0.00552 | -1.14698805 |
| Atp5j2 | 0.00803 | -1.04483898 |
| Senp7 | 0.10949 | 0.89697849 |
| Pdlim3 | 0.9176 | 0.04518289 |
| Ep400 | 0.33256 | -0.2043839 |
| Pja2 | 0.11508 | 0.73142567 |
| BC068281 | 0.00202 | -0.82813444 |
| Gria2 | 0.01141 | -1.67570729 |
| Hykk | 0.00145 | -1.27532353 |
| Ice2 | 0.59669 | 0.18851799 |
| Radil | 0.05628 | -0.89334157 |
| Poli | 0.02599 | -0.53309826 |
| Samd4 | 0.00475 | -1.16873434 |
| Serinc5 | 0.03119 | -0.83378946 |
| Lgi3 | 0.97472 | -0.01305244 |
| Col4a3bp | 0.29094 | 0.47040653 |
| Pxdn | 0.92926 | -0.03507954 |
| Prune2 | 0.5087 | 0.22724979 |
| Ifitm10 | 0.02857 | -0.75912044 |
| 5330434G04Rik | 0.01687 | -1.0543352 |
| Synj2 | 0.0037 | -0.9769394 |
| Sh3pxd2b | 0.98105 | 0.01543171 |
| Pik3c2a | 0.0668 | 0.67587835 |
| Adcy8 | 0.04089 | -0.99242664 |
| Homer2 | 0.01745 | -0.70713838 |
| Gm10767 | 0.8295 | -0.03612361 |
| Fkbp14 | 0.00144 | 1.59674269 |
| Tbc1d5 | 0.04144 | -2.21824817 |
| Pcnt | 0.00981 | -0.68942196 |
| Pstpip2 | 0.01133 | -1.64223381 |
| Xrn1 | 0.38087 | -0.50793695 |
| Mical2 | 0.0439 | -0.84983191 |
| Heatr1 | 0.55012 | -0.30253547 |
| Fam110b | 0.08229 | -2.12160894 |
| Lrrc41 | 0.112 | -0.83236325 |
| Ptprn | 0.00144 | -1.54866398 |
| A330068G13Rik | 0.99165 | 0.01449288 |
| Zc3h12a | 0.05502 | -0.80571535 |
| Prepl | 0.99028 | -0.00252799 |
| Oxa1l | 0.97018 | -0.0073017 |
| Bod1l | 0.31599 | 0.38011427 |
| Iqgap1 | 0.03279 | 1.03985336 |
| Terf2ip | 0.01711 | -0.60403615 |
| Itch | 0.01032 | -0.83367024 |
| Ccdc55 | 0.44575 | -0.30713006 |
| Tmbim4 | 0.02245 | -1.19330584 |
| Daam2 | 0.00144 | -1.32169056 |
| Rims1 | 0.00144 | -1.59270923 |
| Mapk4 | 0.1892 | 0.74707398 |
| BC048403 | 0.01234 | -1.3327164 |
| Zfp131 | 0.01393 | 1.2420745 |
| Npy1r | 0.0354 | -2.01812607 |
| Mbd1 | 0.78156 | -0.16507055 |
| Usp45 | 0.13784 | 0.74509291 |
| Tnrc6b | 0.66073 | 0.07032471 |
| Sltm | 0.09123 | 0.73708395 |
| Slc6a6 | 0.13399 | -0.72576818 |
| Camk1d | 0.30318 | -1.54858227 |
| Pum1 | 0.0136 | -0.53464074 |
| Arid5b | 0.01942 | -0.54521286 |
| Mpp7 | 0.00321 | 2.25250181 |
| Zyg11b | 0.67832 | 0.22121714 |
| Tiam2 | 0.60004 | -0.12969635 |
| Ccdc84 | 0.52483 | -0.2256355 |
| Ppip5k1 | 0.07646 | -0.33898089 |
| Naip7 | 0.00144 | -1.29495476 |
| Mprip | 0.97438 | 0.02274066 |
| Zfp507 | 0.00858 | -0.72157524 |
| Cacna1d | 0.43277 | -0.67860114 |
| Ccnf | 0.00144 | -2.4959548 |
| Dhx29 | 0.05672 | 0.8856062 |
| Zbtb44 | 0.91776 | -0.02946641 |
| Ddx50 | 0.00517 | 2.30787959 |
| Lnpep | 0.00487 | 1.53182231 |
| Rapgef5 | 0.05525 | -0.60807937 |
| Zbed3 | 0.01111 | -0.70990141 |
| Nin | 0.00144 | 2.00751443 |
| Btg4 | 0.89352 | 0.03129881 |
| Plekhg3 | 0.00208 | -1.23527915 |
| Enox1 | 0.00748 | -1.76359773 |
| Pkp2 | 0.87069 | -0.08233907 |
| Tshz2 | 0.08543 | 0.65041696 |
| Usp15 | 0.165 | 0.45126763 |
| Dgkb | 0.83151 | -0.08401064 |
| Lrch1 | 0.13481 | 1.08880101 |
| Mat2a | 0.39574 | 0.18723665 |
| Cnot10 | 0.93248 | -0.02170413 |
| BC106179 | 0.53622 | -0.20764345 |
| Arhgap17 | 0.57422 | 0.197375 |
| Asap1 | 0.02287 | 1.33608212 |
| Ttll13 | 0.27314 | -0.4762117 |
| Zfp458 | 0.02063 | 1.26293951 |
| Heatr5a | 0.08229 | 0.36777404 |
| 1700026D11Rik | 0.01672 | -1.57799391 |
| 4930404A12Rik | 0.00144 | -1.31286007 |
| Bbs1 | 0.00516 | -1.49385047 |
| Sec61a2 | 0.7845 | 0.09742235 |
| Dnah8 | 0.00158 | -1.2446827 |
| Rbbp6 | 0.01178 | 1.52853372 |
| Tmprss2 | 0.04338 | -0.97279541 |
| Cmtm8 | 0.64502 | -0.11963777 |
| BC037032 | 0.0712 | -0.42321101 |
| Mbd4 | 0.01356 | -1.10879133 |
| Gm29782 | 0.03109 | 0.46014461 |
| Eef1e1 | 0.56579 | -0.15302579 |
| 4930503L19Rik | 0.03906 | 0.9224874 |
| Rint1 | 0.30312 | -0.2880761 |
| Brwd1 | 0.91443 | -0.02189962 |
| Ubqln4 | 0.1511 | -0.50124496 |
| Gm11974 | 0.03993 | -0.48095235 |
| Tmed1 | 0.75257 | -0.20416814 |
| Glt8d1 | 0.07473 | 0.72950918 |
| Tbce | 0.94389 | -0.02251808 |
| Gm16226 | 0.08897 | -2.98788179 |
| Dst | 0.04383 | -0.75126053 |
| 4930405O22Rik | 0.08388 | -0.66013988 |
| Camkk2 | 0.06857 | -0.43480722 |
| Lyst | 0.00267 | 2.14103399 |
| Ccdc136 | 0.21773 | 0.44483136 |
| Prdm4 | 0.07349 | -0.97379672 |
| App | 0.37466 | 0.21333802 |
| 4833439L19Rik | 0.19961 | 0.61523425 |
| Per2 | 0.01049 | 1.00577761 |
| Kalrn | 0.0347 | 0.89120118 |
| Ryr2 | 0.01699 | -0.48490588 |
| Gm29514 | 0.12894 | -0.26471386 |
| Umad1 | 0.43408 | -0.26068449 |
| Vps13c | 0.89689 | 0.07291637 |
| Gm26947 | 0.24455 | 0.49916945 |
| Alk | 0.00144 | -1.28116048 |
| Irf2 | 0.01397 | 0.77063403 |
| LOC101056001 | 0.14315 | -0.2658236 |
| Prkar1a | 0.02555 | -0.5360358 |
| Rap1gds1 | 0.13544 | -0.78795495 |
| Zkscan17 | 0.00144 | -1.28116048 |
| Phb2 | 0.00673 | -1.13744792 |
| Lamb1 | 0.05778 | -0.47600896 |
| Hcn1 | 0.00149 | -1.25408 |
| Akna | 0.50151 | 0.24283467 |
| 5930412G12Rik | 0.0089 | -0.60985232 |
| Afap1l2 | 0.8097 | -0.18676141 |
| Gm14866 | 0.05213 | -2.13723632 |
| Tiam2 | 0.01196 | -1.93677152 |
| Lrig2 | 0.17722 | 0.32957379 |
| Tns2 | 0.16832 | -0.23078987 |
| Mrgprf | 0.00343 | -0.88698649 |
| BC006965 | 0.00145 | -1.2388121 |
| Gata3 | 0.41029 | -0.43039738 |
| Slc25a35 | 0.05122 | -0.83737786 |
| Immt | 0.00144 | -1.35708589 |
| Zfp638 | 0.05577 | 0.86435138 |
| Egf | 0.00144 | 1.53735161 |
| Atr | 0.15317 | 0.74024133 |
| Map1b | 0.00144 | 1.40052857 |
| Gigyf2 | 0.13975 | 0.53789274 |
| Dcc | 0.00144 | -1.7365511 |
| Zc3h7b | 0.25518 | -0.28013073 |
| Sema5a | 0.27301 | -1.78314716 |
| Gm830 | 0.00149 | -1.25408 |
| Tfec | 0.00144 | -1.32599123 |
| Cpox | 0.14596 | 0.51485835 |
| Etv6 | 0.00952 | -0.55568944 |
| Arhgef11 | 0.93699 | 0.02718502 |
| Notch2 | 0.04078 | -0.60365539 |
| Meg3 | 0.11974 | 0.6912433 |
| Phf6 | 0.00424 | 0.93863898 |
| Msi2 | 0.92523 | -0.05594565 |
| Tmem2 | 0.70635 | -0.10399605 |
| Wdr1 | 0.14782 | -0.32685067 |
| Lig3 | 0.22206 | -0.24581849 |
| Snx18 | 0.10872 | -0.61956099 |
| Igf1r | 0.02511 | -0.4526161 |
| Ints1 | 0.00144 | -1.31627578 |
| Pik3cd | 0.13227 | -1.16727001 |
| Parp8 | 0.87808 | 0.05363204 |
| Rn28s1 | 0.00299 | -1.47411691 |
| Mast3 | 0.0017 | -1.24950944 |
| Mkln1 | 0.0737 | 1.11972505 |
| Iqsec2 | 0.54506 | -0.21544949 |
| Slc37a4 | 0.01245 | -0.84415222 |
| Ciita | 0.00144 | -1.47229022 |
| 2310067B10Rik | 0.05151 | -0.58201435 |
| Dennd4c | 0.00611 | -1.25935846 |
| Zfp608 | 0.01588 | -0.99849484 |
| Tspan3 | 0.23254 | -0.49237787 |
| Ptpre | 0.28836 | 0.31981486 |
| Exoc3l4 | 0.23392 | -0.99837754 |
| Tmem82 | 0.05391 | 0.70515371 |
| A830008E24Rik | 0.00805 | -1.38739383 |
| Midn | 0.0036 | -0.78369004 |
| Plekhh3 | 0.00144 | -1.18521624 |
| Smyd4 | 0.28503 | -0.21467311 |
| Wdr7 | 0.00447 | 0.81400767 |
| Gm40699 | 0.26446 | 0.38529714 |
| Mctp1 | 0.00145 | -1.2388121 |
| Gm4673 | 0.98316 | 0.01107749 |
| Il7r | 0.08129 | 0.58253408 |
| Gm16754 | 0.02719 | -0.96413034 |
| Cyba | 0.05213 | -0.4696655 |
| Pim1 | 0.00478 | -1.29275681 |
| Cd96 | 0.8155 | -0.21864574 |
| Hmg20a | 0.01652 | -0.91406741 |
| Dek | 0.01165 | 1.20767882 |
| Hdlbp | 0.21958 | -0.19668156 |
| Sema3d | 0.76426 | -0.37779971 |
| Wrn | 0.11344 | 0.56899641 |
| Mettl14 | 0.564 | -0.50804925 |
| Ckap2l | 0.00158 | -1.2446827 |
| Agl | 0.02653 | 1.26419881 |
| Kansl1 | 0.0609 | 1.48740337 |
| Abr | 0.15735 | -0.68856576 |
| Abcd1 | 0.95362 | -0.03645671 |
| Gm15658 | 0.23839 | 0.3825915 |
| Slc4a7 | 0.11438 | 0.3997696 |
| 1700112E06Rik | 0.75963 | -0.06143858 |
| P2rx4 | 0.0078 | -1.13216063 |
| Rbpj | 0.55351 | 0.09768122 |
| Usp8 | 0.02917 | 0.48087301 |
| Gm36151 | 0.35536 | 0.32714468 |
| Adam8 | 0.00181 | -1.24639447 |
| Cdk6 | 0.15421 | -0.36607468 |
| Sidt2 | 0.0249 | -0.81733134 |
| Rgs10 | 0.00144 | -1.29085005 |
| Maf | 0.92063 | 0.05691654 |
| Rabep1 | 0.23923 | 0.32189484 |
| Ccrl2 | 0.1513 | -0.38781006 |
| Fcnb | 0.00145 | -1.88205316 |
| BC026585 | 0.69808 | -0.14449733 |
| Zc3h4 | 0.00256 | -1.39659111 |
| Ammecr1l | 0.35393 | -0.1670436 |
| Fbxo10 | 0.20703 | -1.292138 |
| Rock2 | 0.00144 | 2.78415704 |
| Uxt | 0.40952 | -0.33468931 |
| Gak | 0.42093 | -0.14473549 |
| Ssh3 | 0.00557 | 1.50355624 |
| Il10ra | 0.06138 | -0.57560919 |
| Inpp4a | 0.00437 | -0.85537471 |
| Hps3 | 0.24535 | -0.39963469 |
| Kcnma1 | 0.00527 | -0.74808173 |
| Gm11754 | 0.0015 | -1.15488942 |
| Gm10575 | 0.00818 | -0.82084131 |
| E2f6 | 0.11686 | -0.63266473 |
| Heatr6 | 0.44859 | 0.18286365 |
| Pibf1 | 0.17613 | -0.7940029 |
| Ngly1 | 0.00144 | 1.40439401 |
| Ctnnd1 | 0.11239 | 0.50684134 |
| Ipo7 | 0.33629 | 0.36139838 |
| Usp34 | 0.9068 | -0.04818647 |
| Chrna3 | 0.00144 | -1.29049509 |
| Apoe | 0.00415 | -0.79319894 |
| Syne1 | 0.0023 | 1.71955235 |
| Ccdc159 | 0.30162 | -0.26185893 |
| Chfr | 0.11391 | -0.70889271 |
| 4930503L19Rik | 0.009 | -1.1398278 |
| Tbc1d2b | 0.02469 | -0.45844811 |
| Aprt | 0.713 | -0.16923594 |
| Ulk1 | 0.57285 | 0.16977043 |
| Gm2115 | 0.00533 | -1.29896619 |
| Myo9b | 0.03756 | -0.73788601 |
| Snx13 | 0.01322 | 0.96001537 |
| Mrgprx2 | 0.10895 | -0.57323583 |
| Sh3rf1 | 0.17249 | -0.63603573 |
| Zfp119a | 0.01887 | 1.30675216 |
| Ccdc30 | 0.00276 | -1.14169705 |
| Pdcd6 | 0.19942 | -1.86219058 |
| Wdr19 | 0.15196 | -0.58361988 |
| Arfgef3 | 0.00366 | -1.06744883 |
| Zfp229 | 0.46924 | -0.39296213 |
| Anapc5 | 0.00883 | 1.14442627 |
| Zfp235 | 0.09717 | -1.14955238 |
| Zfp53 | 0.01642 | 0.58922365 |
| Sars | 0.00508 | -1.17914471 |
| Mical3 | 0.00144 | -1.41093324 |
| Fam53b | 0.00149 | -0.94611538 |
| Kif1a | 0.53189 | -0.18527205 |
| Agap1 | 0.51741 | -0.12015485 |
| Tnrc6c | 0.10434 | 0.36472679 |
| Pkd1 | 0.00335 | -0.77143605 |
| Meg3 | 0.00288 | 2.3076522 |
| Pafah1b1 | 0.00144 | 1.36015333 |
| Ehbp1 | 0.0748 | 1.15158683 |
| Vps13d | 0.27867 | 0.46481595 |
| Zbtb8b | 0.01228 | -1.52681706 |
| Flt1 | 0.31981 | 0.29059122 |
| Laptm4b | 0.00859 | -0.58120137 |
| Calml4 | 0.01915 | -0.87511357 |
| Zfp553 | 0.00149 | -0.96514509 |
| LOC102641455 | 0.02724 | -1.10021196 |
| Gm32880 | 0.51171 | -0.29505909 |
| Smco4 | 0.07252 | -0.51809197 |
| A430072P03Rik | 0.37258 | -0.88208321 |
| Gm17747 | 0.00144 | -1.36149651 |
| Pcgf5 | 0.08049 | 0.57423297 |
| Kdm4a | 0.85895 | -0.0482747 |
| Casc3 | 0.15361 | -0.2507492 |
| Clk3 | 0.1041 | -0.2828738 |
| Tfap2a | 0.00144 | -1.44399392 |
| Arglu1 | 0.02543 | -1.15911784 |
| Tnfrsf10b | 0.43863 | -0.36434499 |
| Wdr74 | 0.02269 | -0.62212069 |
| Gm5907 | 0.00613 | -1.05687292 |
| Maml3 | 0.01842 | -0.98296837 |
| Pole4 | 0.81405 | -0.2456552 |
| Gm15867 | 0.02672 | -0.85701504 |
| Syt17 | 0.07182 | -0.78176753 |
| 4930519P11Rik | 0.00144 | -1.31769968 |
| Gm10863 | 0.49641 | 0.13704012 |
| Gm35521 | 0.67117 | -0.09939695 |
| Gm7596 | 0.79532 | -0.13708401 |
| Kdm4b | 0.07105 | -0.34962848 |
| Gm34006 | 0.02036 | -3.34605256 |
| 5031415H12Rik | 0.08187 | -0.90779636 |
| Dock6 | 0.88312 | 0.05417025 |
| Pdgfra | 0.87246 | -0.06636697 |
| 9330188P03Rik | 0.30202 | 0.32947621 |
| Fam193a | 0.00535 | -0.71890187 |
| Actr3b | 0.01317 | -1.36082072 |
| Bach2os | 0.13594 | -0.92114594 |
| Gm13662 | 0.00144 | -1.29085005 |
| Mctp1 | 0.00144 | -1.24613586 |
| Man2a1 | 0.20694 | -0.33657255 |
| Krit1 | 0.00144 | 2.02918951 |
| Kdm6a | 0.79678 | -0.05849623 |
| Sipa1l3 | 0.05099 | -2.11625265 |
| Ttbk1 | 0.00144 | -1.31153409 |
| Casz1 | 0.03712 | -0.53333684 |
| LOC105242515 | 0.1782 | -2.17827576 |
| Tbc1d5 | 0.16255 | 0.85805581 |
| Acsf3 | 0.00686 | -0.99607411 |
| Ncoa7 | 0.90623 | -0.04483989 |
| Epb4.1l4b | 0.78399 | 0.13148192 |
| LOC105244993 | 0.00144 | -1.31495282 |
| LOC101055656 | 0.1496 | -0.41152725 |
| Ift172 | 0.00158 | -1.2446827 |
| Gm41341 | 0.00145 | -1.26219879 |
| Nek4 | 0.0051 | -0.97240036 |
| Lpar2 | 0.00145 | -0.94120347 |
| Rabl6 | 0.74937 | -0.13117881 |
| Specc1 | 0.06397 | -1.680679 |
| Tead3 | 0.6803 | -0.0648611 |
| Plk2 | 0.69232 | 0.10572666 |
| 1110019D14Rik | 0.01299 | -0.57642872 |
| Ptpn13 | 0.34033 | -0.49391393 |
| Map3k2 | 0.04397 | 1.22989612 |
| Gbf1 | 0.00144 | -1.2807135 |
| Cwc22 | 0.03182 | 1.12606747 |
| Ryr1 | 0.00144 | -1.29085005 |
| Arntl | 0.78641 | -0.28364269 |
| Ankrd10 | 0.12646 | -0.51800042 |
| Map4k1 | 0.02293 | -0.59782859 |
| Itpr2 | 0.31153 | 0.41199951 |
| Foxo3 | 0.00788 | -0.77600094 |
| Gm16046 | 0.17806 | -0.44704622 |
| Chat | 0.04464 | -2.34402605 |
| Lmo7 | 0.51063 | -0.15108761 |
| G630018N14Rik | 0.04474 | -1.14286665 |
| Hydin | 0.00145 | -1.24191372 |
| Scn7a | 0.64442 | -0.30229687 |
| Mdn1 | 0.4987 | 0.32221677 |
| Itgb4 | 0.02236 | -0.95167635 |
| Gm34289 | 0.00144 | -1.20555712 |
| Cpd | 0.01604 | 0.87620894 |
| Gm5475 | 0.0328 | -1.505597 |
| Endod1 | 0.01517 | -0.53394906 |
| Gm15050 | 0.01552 | -1.25397768 |
| Uty | 0.11882 | 0.57557724 |
| Cdc25a | 0.03504 | -0.49297873 |
| Pik3c2a | 0.12026 | 0.35575374 |
| Mtcp1 | 0.05622 | 1.46824127 |
| Robo1 | 0.00144 | 1.5735349 |
| Ndst3 | 0.21076 | -0.53452606 |
| Parp2 | 0.37725 | 0.20995481 |
| Mrvi1 | 0.1685 | 0.3392664 |
| Tbc1d8 | 0.6402 | 0.21462664 |
| BC055324 | 0.92236 | -0.0681195 |
| Xpr1 | 0.01156 | 1.29007461 |
| C2cd5 | 0.08446 | 0.71897873 |
| Gatad2b | 0.3223 | -0.17906709 |
| Acsf3 | 0.88326 | 0.1389371 |
| Gm3764 | 0.00144 | -1.29085005 |
| Lpin1 | 0.00173 | -1.0277763 |
| Gad2 | 0.00144 | -1.29085005 |
| Plcb1 | 0.01647 | 0.79517783 |
| Csnk1a1 | 0.00758 | 1.26845836 |
| Arnt | 0.06641 | 0.72229275 |
| Epb4.1l4a | 0.02948 | -0.75126486 |
| Nrp2 | 0.24993 | -0.61263203 |
| Eprs | 0.84697 | 0.06533662 |
| Tfap2a | 0.06551 | -1.9926579 |
| Limch1 | 0.75288 | -0.13820812 |
| Dym | 0.20909 | 0.48743642 |
| Jmjd1c | 0.42243 | -0.20568793 |
| Mettl1 | 0.00596 | -0.66337587 |
| Fam222b | 0.01227 | -0.61988791 |
| Epb4.1l4a | 0.50734 | -0.12067469 |
| Epb4.1l4a | 0.31659 | -0.21646255 |
| Gtf3c1 | 0.00144 | -1.66846872 |
| Zranb1 | 0.04779 | -0.57931703 |
| B3gat3 | 0.00176 | -1.26402932 |
| LOC101056011 | 0.00144 | -1.29049509 |
| Gm19412 | 0.01007 | -0.71159012 |
| Tdp2 | 0.00235 | 1.66649893 |
| Gkap1 | 0.01714 | 0.53305361 |
| Prnp | 0.93707 | 0.01967806 |
| Slc35a4 | 0.82264 | -0.08988977 |
| Gm11665 | 0.06963 | -0.82249579 |
| Gm42098 | 0.00144 | -1.24811894 |
| Wdr44 | 0.5691 | 0.12803233 |
| Rpl23 | 0.08631 | -0.2949265 |
| Ccdc82 | 0.10842 | 0.40782326 |
| Zfp410 | 0.99776 | -0.00123781 |
| Dnah14 | 0.00144 | -1.29746368 |
| Metap1d | 0.00239 | -1.38646478 |
| Slitrk3 | 0.00144 | -1.28898079 |
| Hnrnpa2b1 | 0.03406 | -0.61044381 |
| Siglech | 0.49482 | -0.5225844 |
| Gtf3c1 | 0.07565 | -0.7458331 |
| Zdhhc20 | 0.00649 | 1.53733976 |
| Elf2 | 0.00374 | -0.84761203 |
| Fiz1 | 0.57947 | 0.15259517 |
| Gm42274 | 0.95196 | 0.01051085 |
| Pdgfra | 0.4785 | -0.2048438 |
| AU015791 | 0.00366 | -1.64327337 |
| Orm1 | 0.00144 | -1.69419903 |
| Casz1 | 0.0133 | -0.97688161 |
| Tkfc | 0.01514 | -0.78008423 |
| Nrap | 0.00439 | -0.80134752 |
| Cd300lg | 0.91391 | 0.04029875 |
| Usp4 | 0.04621 | -0.84754406 |
| Slc2a4 | 0.01444 | -0.83277617 |
| Lama3 | 0.11881 | -2.32925896 |
| Ankrd11 | 0.8809 | 0.03942852 |
| 1700026H06Rik | 0.00414 | -1.07965768 |
| Cdc42bpa | 0.05235 | 1.51053124 |
| Dnaic2 | 0.72491 | 0.08641202 |
| Emc1 | 0.03323 | -0.65877225 |
| Astn1 | 0.15132 | 0.57384017 |
| Sptbn2 | 0.1841 | -2.32602523 |
| Map9 | 0.03064 | 0.77305034 |
| LOC102634058 | 0.8921 | -0.16460487 |
| Ahnak2 | 0.00241 | -0.88338888 |
| LOC101056159 | 0.00145 | -1.2388121 |
| Tut1 | 0.97414 | -0.02160001 |
| Gm36037 | 0.00144 | -1.29085005 |
| Ntm | 0.00159 | -1.26431393 |
| Trim30a | 0.89344 | 0.02932816 |
| Adcy8 | 0.99062 | 0.01042736 |
| Abca8b | 0.72583 | -0.12711868 |
| Dock1 | 0.266 | 0.42827441 |
| Smpd1 | 0.51948 | -0.14297206 |
| Txk | 0.00144 | -1.31552497 |
| Ilvbl | 0.48409 | 0.27878442 |
| Cenpc1 | 0.75508 | 0.15744889 |
| Ncor1 | 0.10893 | -0.49509742 |
| Spen | 0.51748 | 0.30671186 |
| D930015E06Rik | 0.75091 | -0.1734772 |
| Myo18a | 0.03633 | -0.48524246 |
| Eif3d | 0.00144 | -1.25927895 |
| Ebf1 | 0.00764 | 1.41477251 |
| Astn2 | 0.00144 | -1.29085005 |
| Sptbn1 | 0.03151 | 1.77240991 |
| G3bp1 | 0.05336 | -0.5806209 |
| Gm11744 | 0.08694 | -1.43580951 |
| Ccdc88c | 0.17131 | -0.29824485 |
| Gm3775 | 0.39516 | -0.33130679 |
| Nup98 | 0.34586 | -0.53263385 |
| Myh11 | 0.87693 | 0.07270334 |
| Itga1 | 0.04266 | 0.95455615 |
| Diap3 | 0.05979 | -1.43793531 |
| Blm | 0.18793 | 0.60711666 |
| Lrig3 | 0.50759 | 0.22135912 |
| Gad2 | 0.00144 | -1.30017714 |
| Glb1l2 | 0.21492 | -1.04319467 |
| Sec61a2 | 0.01328 | 1.36133294 |
| Pkd2l2 | 0.06611 | 0.57233293 |
| D030068K23Rik | 0.0017 | -1.24950944 |
| Strn | 0.00993 | -1.10643551 |
| A730082K24Rik | 0.0049 | -1.08617297 |
| Gm4203 | 0.5866 | 0.15708338 |
| Cbx3 | 0.87893 | 0.0374007 |
| Vps13a | 0.08374 | -0.32506823 |
| Ppfibp2 | 0.00506 | -1.00180345 |
| Dennd6a | 0.04234 | 0.78298359 |
| Nlrp4f | 0.00144 | -1.32647189 |
| Sytl2 | 0.0127 | -1.1649976 |
| Gm32442 | 0.00145 | -1.25646236 |
| Scn10a | 0.06702 | -2.16443169 |
| Pdzrn3 | 0.00144 | -1.29049509 |
| Pcnx | 0.83393 | 0.04579082 |
| LOC102641761 | 0.00144 | -1.28116048 |
| Gm38438 | 0.00144 | -1.93928284 |
| Vcl | 0.01178 | 1.42320453 |
| Zmym2 | 0.16837 | -0.39378467 |
| A730017L22Rik | 0.1648 | -0.30272711 |
| Nrp1 | 0.04345 | 1.27775843 |
| Dnm1l | 0.02162 | 0.95106043 |
| Cdc40 | 0.72099 | -0.27185848 |
| Ace2 | 0.96353 | -0.02305156 |
| Dedd2 | 0.99537 | 0.00694119 |
| Sars2 | 0.87592 | 0.04487134 |
| Socs5 | 0.00144 | -1.16898273 |
| Kcnh3 | 0.01067 | -0.77397469 |
| Bms1 | 0.31771 | -0.37029194 |
| Fam193a | 0.0078 | 1.28222015 |
| 2310002F09Rik | 0.00144 | -1.29746368 |
| Gm26908 | 0.02492 | 0.85852569 |
| Slc7a6os | 0.17377 | -0.80127102 |
| Cdh6 | 0.00145 | -1.2388121 |
| Rims1 | 0.00158 | -1.22727468 |
| Bod1l | 0.00144 | -1.23155796 |
| Trp53bp2 | 0.00932 | -0.75844203 |
| Nsmaf | 0.59316 | -0.19175998 |
| Gm15326 | 0.04147 | -2.02346311 |
| Txlng | 0.00779 | 1.90361981 |
| Sema3d | 0.00463 | -1.48164402 |
| Evi5 | 0.07355 | 0.61204965 |
| Btbd7 | 0.00938 | 1.77438177 |
| Gtf3c1 | 0.11883 | -0.68835696 |
| Gm15558 | 0.08005 | 0.66704458 |
| Stip1 | 0.16547 | -0.78689298 |
| Trpv2 | 0.00176 | -1.24266807 |
| Eif4g1 | 0.00144 | -1.02857014 |
| Tcf4 | 0.00458 | 1.71868701 |
| Usp15 | 0.00144 | 1.57238894 |
| 4930458D05Rik | 0.4723 | 0.28059946 |
| Tert | 0.05449 | -0.98980568 |
| Palm | 0.10964 | 0.53459546 |
| Epha5 | 0.00144 | -1.27165604 |
| Chrnb3 | 0.04381 | -2.34230356 |
| Ghrhr | 0.00626 | -1.49567869 |
| Palm2 | 0.03338 | 1.02178688 |
| Proser2 | 0.83665 | -0.06360384 |
| Map3k4 | 0.16368 | -0.77032878 |
| Naga | 0.53134 | -0.13554547 |
| Ick | 0.00144 | -1.35320495 |
| Lin7c | 0.26094 | 0.23660504 |
| Gm26588 | 0.8398 | -0.22229123 |
| Csf2rb | 0.00704 | -1.54311887 |
| 1700028P15Rik | 0.01778 | -1.05211616 |
| Arel1 | 0.40606 | 0.23317648 |
| Nphp3 | 0.51149 | -0.28817188 |
| AY702103 | 0.13253 | -3.29406574 |
| Dnah14 | 0.0056 | 0.90374337 |
| 4930500H12Rik | 0.00144 | -1.27165604 |
| Dnah12 | 0.00144 | -1.26560808 |
| Itgad | 0.00144 | -1.25118919 |
| Ankrd49 | 0.00145 | 1.21454652 |
| 1700030C12Rik | 0.00144 | -1.30017714 |
| A930007I19Rik | 0.00318 | 1.1682899 |
| 4930544I03Rik | 0.23901 | 0.64464167 |
| Mef2a | 0.51928 | 0.11338066 |
| Stx12 | 0.2639 | 0.45376841 |
| Gm19865 | 0.00144 | -1.27699683 |
| Pam | 0.00271 | -1.1204374 |
| Smgc | 0.00144 | -1.30713006 |
| Col6a2 | 0.01183 | -0.71785974 |
| Efcab11 | 0.00223 | -1.06136102 |
| Firre | 0.00144 | 2.30208932 |
| Rlf | 0.92281 | 0.02636375 |
| Acan | 0.00144 | -1.30017714 |
| Cdkn1c | 0.01134 | -0.71993746 |
| Rbbp7 | 0.02657 | 1.26989904 |
| BC051077 | 0.07594 | -0.64827514 |
| Fam173a | 0.02113 | -0.58741767 |
| Treml2 | 0.00144 | 4.02961149 |
| Gm17753 | 0.85305 | 0.04674226 |
| Sis | 0.00144 | -1.28116048 |
| Zfp933 | 0.16387 | 0.3386542 |
| Gm40421 | 0.00144 | -1.29746368 |
| Ubqln1 | 0.66822 | 0.10072121 |
| Sel1l2 | 0.10339 | -0.8216566 |
| Fam208b | 0.00144 | 1.81102752 |
| Rims1 | 0.23647 | -0.76638697 |
| Tpcn2 | 0.02394 | -1.8297405 |
| Ttc3 | 0.07114 | 0.79045568 |
| Uvrag | 0.71061 | 0.11726511 |
| Cd69 | 0.08557 | -0.83099121 |
| Il6 | 0.00144 | -1.29085005 |
| Rnf216 | 0.62641 | 0.16363805 |
| Trim30a | 0.04041 | 0.86842594 |
| Sbno2 | 0.0037 | -0.86976743 |
| Agk | 0.88039 | -0.20028985 |
| Gm12708 | 0.0281 | -0.95564746 |
| Gm16096 | 0.71977 | 0.06379337 |
| Irf4 | 0.00144 | -1.28898079 |
| Dctn4 | 0.2478 | 0.22072181 |
| F630040K05Rik | 0.23722 | -0.52726507 |
| Cd33 | 0.00144 | -1.33632959 |
| 6430573F11Rik | 0.96754 | 0.01460098 |
| Eftud1 | 0.24878 | -0.37246166 |
| Med13l | 0.45821 | -0.22054161 |
| Mapre2 | 0.21333 | -0.40778085 |
| E430014B02Rik | 0.09476 | -0.85232898 |
| Scoc | 0.40912 | 0.66686584 |
| Setd8 | 0.00395 | -0.71306744 |
| Gabpb2 | 0.53292 | -0.45591039 |
| Ptk7 | 0.00144 | -1.57547831 |
| Tctn2 | 0.50924 | 0.30779291 |
| Pdgfb | 0.01885 | -0.80324313 |
| Snrnp48 | 0.0349 | -0.84103361 |
| Smyd3 | 0.0148 | -1.05812364 |
| 2810429I04Rik | 0.09141 | -2.56886654 |
| Nup205 | 0.00326 | -0.92435117 |
| Nrcam | 0.2039 | -0.58614844 |
| Lrriq3 | 0.64709 | 0.28266564 |
| Kif15 | 0.11212 | -2.04614612 |
| Stard9 | 0.07187 | 0.95251133 |
| Gm41666 | 0.63115 | 0.19155663 |
| Hdac9 | 0.06841 | 2.04127887 |
| Top2b | 0.52148 | -0.21065124 |
| Mtdh | 0.03789 | 1.25016523 |
| Hyal2 | 0.32694 | -0.38276265 |
| Kctd9 | 0.07519 | -0.87458824 |
| Utrn | 0.05481 | 0.7213172 |
| Gns | 0.1735 | -0.44715059 |
| Tinagl1 | 0.04174 | -1.11446927 |
| LOC102641119 | 0.69947 | -0.16212132 |
| D930032P07Rik | 0.00144 | -1.55081145 |
| 2010111I01Rik | 0.24123 | -0.23664684 |
| Gpc4 | 0.12096 | 0.31006962 |
| E030003E18Rik | 0.29435 | 0.45049752 |
| Klhl22 | 0.98921 | 0.00942118 |
| Ptpn14 | 0.01098 | -0.84300743 |
| Fancm | 0.01033 | -0.75647164 |
| Slc36a2 | 0.88168 | -0.06887439 |
| Lpp | 0.31748 | 0.35783575 |
| Cpsf6 | 0.00144 | 1.16381639 |
| Anxa11 | 0.11729 | 0.52085149 |
| D830039M14Rik | 0.03515 | -0.66306 |
| Micall2 | 0.00435 | -1.08359888 |
| Lama4 | 0.01316 | -0.66610979 |
| Prodh | 0.08334 | -0.39292697 |
| D830024N08Rik | 0.05791 | 1.2179474 |
| Myh11 | 0.56892 | 0.24469296 |
| Pomt2 | 0.96054 | -0.01861513 |
| Slc15a1 | 0.00144 | -1.26934956 |
| Il15 | 0.00811 | 1.89142619 |
| Cmip | 0.93604 | -0.04016165 |
| Sh3pxd2a | 0.10507 | -0.5883082 |
| Dock4 | 0.03443 | 0.86222714 |
| Fam168a | 0.00153 | -1.02572889 |
| Egf | 0.50029 | -0.32459223 |
| Ankdd1b | 0.51635 | -0.42693637 |
| 9930038B18Rik | 0.00144 | -1.65333766 |
| Neo1 | 0.10602 | 0.63207882 |
| 4933412E12Rik | 0.22345 | -0.69882281 |
| C330007P06Rik | 0.00472 | -1.06270273 |
| A630065K11Rik | 0.09248 | 0.81454772 |
| 4833422M21Rik | 0.00144 | -1.29878664 |
| Cdc40 | 0.21285 | 0.72201351 |
| Cacna2d1 | 0.00387 | 0.97938903 |
| Tpp1 | 0.54506 | -0.09866533 |
| Prox1 | 0.00825 | -0.59239478 |
| Ralgapa1 | 0.01106 | 1.24394471 |
| Pax3 | 0.04621 | -1.96799104 |
| Cmah | 0.11233 | 0.51313137 |
| Supt20 | 0.30708 | 0.26365762 |
| Cyb5b | 0.16638 | 0.23905544 |
| Usp15 | 0.05474 | 1.21881356 |
| Rims2 | 0.00144 | -1.29746368 |
| Gm30382 | 0.45302 | 0.30229121 |
| Dync1li1 | 0.07063 | -0.95564746 |
| Gm41609 | 0.00144 | -1.29085005 |
| Dnajc21 | 0.07399 | 0.46380915 |
| Gm38414 | 0.00144 | -1.20052149 |
| Dock4 | 0.11553 | 0.54784092 |
| C230037L18Rik | 0.08237 | 0.33459174 |
| Igdcc4 | 0.34012 | 0.32389963 |
| Luzp2 | 0.02109 | -1.17660698 |
| Fignl1 | 0.00208 | -1.15498993 |
| Gm17750 | 0.00144 | -1.27165604 |
| Trpc3 | 0.11788 | -0.34363673 |
| Fbn2 | 0.19824 | -0.71979811 |
| Fbxo16 | 0.08256 | 1.03219954 |
| Prr16 | 0.00146 | -1.27088387 |
| Yeats4 | 0.91798 | 0.0218561 |
| Neto2 | 0.00144 | -1.31884045 |
| Matn2 | 0.05362 | 0.4365203 |
| Ankrd39 | 0.28424 | -0.42059785 |
| Fam35a | 0.3104 | -0.28779108 |
| Lrp5 | 0.80873 | -0.08544217 |
| Tmem189 | 0.13721 | -0.51717265 |
| Fbn1 | 0.97789 | -0.01398451 |
| Slc26a6 | 0.26745 | -0.53389411 |
| Nrn1l | 0.04879 | -0.34676184 |
| Fads1 | 0.01024 | -0.89882872 |
| 1700017B05Rik | 0.07533 | -0.35211683 |
| Kcnq2 | 0.03982 | -1.03581296 |
| Phf20l1 | 0.00457 | 1.62678519 |
| Zkscan2 | 0.00144 | -1.32599123 |
| Zfp467 | 0.01146 | -0.60142262 |
| Me2 | 0.65385 | -0.11575029 |
| Gm5914 | 0.33115 | 0.73411302 |
| Wdr33 | 0.0037 | 0.99937342 |
| Rmi2 | 0.13392 | 0.4289443 |
| Ptpn13 | 0.47382 | -0.37536019 |
| Trib1 | 0.03241 | -0.65821619 |
| Acsm5 | 0.0474 | -0.99177254 |
| Fcer2a | 0.23795 | -0.62779694 |
| Esyt1 | 0.04917 | 1.1387231 |
| Gm16283 | 0.00771 | -0.64762278 |
| Gstt2 | 0.37011 | -0.36110413 |
| Tmem19 | 0.05905 | 0.62297502 |
| A430010J10Rik | 0.00144 | -1.30017714 |
| Gdi2 | 0.0874 | 0.34955678 |
| Gabra4 | 0.02642 | -1.09848529 |
| Efcab14 | 0.00987 | 2.47616881 |
| Gtf2ird1 | 0.40422 | -0.52650204 |
| Atp11c | 0.01209 | 1.29824878 |
| 2310065F04Rik | 0.00144 | -1.30713006 |
| Eps15l1 | 0.32098 | -0.25686461 |
| Gm26910 | 0.00767 | 1.10684418 |
| 9130204K15Rik | 0.25788 | -0.74381313 |
| Capn2 | 0.25371 | 0.250922 |
| Pak1 | 0.00144 | -1.24900642 |
| Sptlc3 | 0.04225 | -0.39266131 |
| Zbtb20 | 0.42336 | -0.46684598 |
| Lactb | 0.00381 | 0.81113883 |
| C030014I23Rik | 0.0098 | -1.01108256 |
| Sltm | 0.0144 | 2.02223126 |
| 4930430F08Rik | 0.11827 | -0.63117052 |
| Mettl6 | 0.05898 | 0.80102634 |
| 1500004A13Rik | 0.20916 | 0.43569812 |
| Il18 | 0.63371 | 0.12547423 |
| Mrps31 | 0.38711 | -0.24130792 |
| Ttc5 | 0.09483 | -0.75447164 |
| Rad51ap1 | 0.23777 | 0.52482475 |
| Me2 | 0.63221 | -0.13803346 |
| Net1 | 0.31689 | -0.47105389 |
| Cc2d2a | 0.41525 | 0.20568649 |
| Gm32647 | 0.35581 | 0.26285972 |
| Lcor | 0.04739 | 1.04856054 |
| Wdr60 | 0.07502 | 0.59988287 |
| Gm34299 | 0.03444 | -1.04501 |
| Trpm3 | 0.08044 | -1.07120217 |
| Frmd4a | 0.03712 | 0.90088402 |
| Gm29371 | 0.00144 | -1.20555712 |
| Gm11482 | 0.78956 | -0.10869736 |
| Trp53rkb | 0.6984 | -0.21584138 |
| Rapgef1 | 0.01412 | -0.57733326 |
| 4930544M13Rik | 0.00144 | -1.29128727 |
| Gm29823 | 0.04166 | -0.76605024 |
| Gm38691 | 0.00144 | -1.28116048 |
| Brd3 | 0.00388 | -0.77223337 |
| Spata17 | 0.00144 | -1.29864962 |
| 4930474G06Rik | 0.00144 | -1.30017714 |
| 1700029M03Rik | 0.22663 | -0.60179152 |
| 4930503F20Rik | 0.00193 | -1.95182471 |
| Gm26611 | 0.12206 | -2.11179678 |
| Gdpd1 | 0.00144 | -1.22465584 |
| 4930417H01Rik | 0.01011 | -1.92648025 |
| Abca15 | 0.00233 | -1.4352793 |
| 1700061H18Rik | 0.00149 | -1.22582153 |
| Hemgn | 0.00144 | -1.20555712 |
| Fbxo21 | 0.09669 | -0.75001032 |
| Phf1 | 0.00653 | -0.62384281 |
| Kif23 | 0.32903 | -0.25279407 |
| Ppm1a | 0.16848 | 0.26156235 |
| Man2b2 | 0.24074 | -0.22265648 |
| Senp1 | 0.43732 | -0.16340303 |
| C77370 | 0.84062 | 0.24054363 |
| Acsl5 | 0.00144 | -1.30626366 |
| Fry | 0.17459 | -0.33890632 |
| Sema5a | 0.74681 | 0.12031867 |
| St6gal2 | 0.01131 | -1.11664749 |
| Prune2 | 0.01945 | 0.51259392 |
| Nek5 | 0.00736 | -1.22518835 |
| Rictor | 0.01653 | 0.84234362 |
| Arhgef10 | 0.78608 | -0.17507347 |
| Gatb | 0.86119 | 0.05937466 |
| Dock5 | 0.76713 | -0.19754923 |
| Slc6a1 | 0.84013 | -0.21334006 |
| Clvs2 | 0.00144 | -1.28898079 |
| Pofut1 | 0.00144 | -1.18109374 |
| Rassf4 | 0.00145 | -1.06854687 |
| Epb4.1l3 | 0.0663 | -0.79481818 |
| Ints6 | 0.44237 | -0.33593279 |
| Tspan3 | 0.00144 | -1.3570277 |
| Dnajc14 | 0.98701 | -0.00693588 |
| Afap1l2 | 0.15008 | -0.98262079 |
| Gata6 | 0.22105 | -0.52732076 |
| St5 | 0.1803 | -0.50099898 |
| Chl1 | 0.05694 | -1.8553876 |
| Heatr5b | 0.03293 | 0.92146759 |
| Ltbp2 | 0.00144 | -1.6957738 |
| Atp1a2 | 0.40425 | 0.62570744 |
| Nid2 | 0.05858 | -2.27433301 |
| Csn1s1 | 0.00145 | -1.22376446 |
| Oxr1 | 0.01269 | -0.75682554 |
| Cacna1a | 0.14885 | -0.37001999 |
| Phox2b | 0.01187 | -1.00553399 |
| Nktr | 0.33104 | 0.17736291 |
| Tbx3 | 0.2085 | -0.2890031 |
| Il7 | 0.00144 | -1.26301121 |
| Kif15 | 0.00144 | -1.25717427 |
| Sfmbt2 | 0.08163 | -0.95241994 |
| Tbcel | 0.00361 | -0.71905013 |
| Creb5 | 0.08207 | 0.80352033 |
| Palld | 0.00144 | -1.32313902 |
| Thsd7b | 0.00158 | -1.2446827 |
| Smarca1 | 0.64731 | -0.29075946 |
| Mme | 0.56958 | 0.41206607 |
| Hbegf | 0.01539 | -1.9527968 |
| Faf2 | 0.49828 | 0.22194573 |
| Pnn | 0.00144 | 1.86644679 |
| Dst | 0.60719 | 0.09111306 |
| Astn2 | 0.35073 | -0.44477061 |
| Inpp4a | 0.51552 | -0.26937082 |
| Gtf2ird1 | 0.77497 | -0.22591352 |
| Dennd4a | 0.15056 | 0.39444618 |
| Mir99ahg | 0.02758 | 1.58615166 |
| Ikbkb | 0.00144 | -1.24811894 |
| Taf2 | 0.01455 | -1.98524892 |
| Lama1 | 0.00144 | -1.27165604 |
| Xpo7 | 0.05695 | 0.63574942 |
| Ubap2l | 0.00144 | 1.14654097 |
| E430024P14Rik | 0.00327 | -1.43580951 |
| Rps6ka2 | 0.00249 | -1.99676887 |
| Dctn4 | 0.20144 | 0.55798697 |
| Slc25a40 | 0.18029 | 0.45386846 |
| Mpp7 | 0.0095 | 0.92973461 |
| Nr1h4 | 0.00146 | -1.23139222 |
| Lypla1 | 0.20824 | -0.32865102 |
| Nedd4 | 0.40475 | 0.14878151 |
| Gipr | 0.12201 | -0.49732049 |
| Acin1 | 0.00267 | -0.94886355 |
| Tnfsf13b | 0.21892 | -0.28207461 |
| Gpr137b | 0.8064 | -0.05154175 |
| Gm39079 | 0.0679 | -2.32889388 |
| LOC105246088 | 0.58098 | -0.33107382 |
| Gm33447 | 0.00144 | -1.80304123 |
| Jpx | 0.02025 | -0.64849271 |
| Gm20319 | 0.01421 | -0.91422642 |
| C530008M17Rik | 0.10093 | 0.61143586 |
| Pcdh8 | 0.00145 | -1.22376446 |
| Anapc7 | 0.03518 | 0.68926847 |
| A030001D20Rik | 0.43562 | -0.1795758 |
| Prmt3 | 0.80592 | -0.03988081 |
| Ogfod1 | 0.56489 | -0.48819501 |
| Trpc4 | 0.00144 | -1.61432628 |
| Adal | 0.71664 | 0.06188951 |
| Zdhhc13 | 0.24607 | 0.23573055 |
| Pik3c2a | 0.15525 | 0.23951185 |
| Sash1 | 0.91962 | 0.02480479 |
| Haus3 | 0.00205 | 1.51288804 |
| Tmem80 | 0.02928 | -0.54266892 |
| Zfp410 | 0.054 | 1.37447872 |
| Prss41 | 0.00158 | -1.2446827 |
| A230004M16Rik | 0.00144 | -1.29746368 |
| Tbc1d7 | 0.00144 | -1.30737044 |
| Itpr1 | 0.96493 | 0.01389695 |
| Slc13a5 | 0.00144 | -1.26560808 |
| Lrp11 | 0.01773 | -1.44052335 |
| C230072F16Rik | 0.00158 | -1.2446827 |
| Arhgef28 | 0.58324 | 0.33742917 |
| Antxr1 | 0.74579 | 0.05544895 |
| Pantr1 | 0.00144 | -1.26911831 |
| Gpc2 | 0.0093 | 0.86885207 |
| Nrcam | 0.00144 | -1.29051721 |
| B3gat3 | 0.72159 | 0.25938868 |
| Chsy3 | 0.02529 | -1.15268346 |
| Gabra2 | 0.00144 | -1.26934956 |
| Sh3tc2 | 0.0085 | 1.65599424 |
| Zfp608 | 0.98858 | -0.01548989 |
| 1700047M11Rik | 0.89841 | 0.04143182 |
| Kdm5d | 0.82928 | 0.10124124 |
| Cfap74 | 0.07372 | -0.53247929 |
| Dclk1 | 0.00145 | -1.2388121 |
| Fbxw8 | 0.12717 | -0.51683095 |
| C030009J22Rik | 0.49198 | -0.20490546 |
| Slc6a1 | 0.02311 | -0.63539746 |
| Gad1 | 0.00145 | -1.2388121 |
| Dcp1a | 0.08289 | 1.10912066 |
| Ganab | 0.25126 | -0.50964203 |
| LOC102637577 | 0.08293 | -0.50622777 |
| Lhx3 | 0.04092 | -0.72213975 |
| Gm10378 | 0.00447 | -1.07435095 |
| Ccnt2 | 0.45368 | -0.20002273 |
| Ptpn13 | 0.94247 | 0.01918743 |
| Sf3b3 | 0.02341 | -0.63449835 |
| Snx14 | 0.00178 | -1.03800811 |
| Clcn3 | 0.0162 | 0.59518034 |
| B430212C06Rik | 0.91144 | -0.02253451 |
| Xrcc1 | 0.00145 | -1.22193674 |
| Sema5a | 0.00866 | -1.67255267 |
| Cdh7 | 0.00144 | -1.39815941 |
| Adgrl3 | 0.12852 | 1.22289627 |
| B230354K17Rik | 0.38497 | 0.23312239 |
| Tmco6 | 0.02399 | 0.54270466 |
| Mmp16 | 0.00144 | -1.28116048 |
| Ints6 | 0.35722 | 0.30418845 |
| Usp53 | 0.81598 | -0.05394564 |
| Trp53bp1 | 0.04554 | -0.63336529 |
| Edil3 | 0.11243 | -0.60060285 |
| Polr1a | 0.00144 | -1.28898079 |
| Rpl11 | 0.09489 | -0.51128817 |
| Coro2a | 0.11025 | 0.90070187 |
| C230021G24Rik | 0.03459 | -0.83963955 |
| Trp53bp1 | 0.27389 | 0.50290584 |
| Smug1 | 0.00144 | -1.30792819 |
| Gm6145 | 0.86886 | 0.07834001 |
| Ezh1 | 0.0832 | -0.32273799 |
| Slc25a12 | 0.34056 | 0.28612337 |
| Ccdc15 | 0.18104 | -0.3648366 |
| Swap70 | 0.011 | 1.51097461 |
| Letmd1 | 0.00963 | -0.66105209 |
| Pbx1 | 0.71451 | 0.26972262 |
| Sema5a | 0.11212 | 0.46186565 |
| Mtpap | 0.95008 | 0.01290518 |
| Serbp1 | 0.00399 | 1.04863684 |
| Kat6b | 0.28447 | -0.31323696 |
| Huwe1 | 0.13294 | -0.33343733 |
| Meis2 | 0.81016 | -0.1186629 |
| Nos1ap | 0.2221 | 0.47130398 |
| Atad1 | 0.23211 | 0.70092966 |
| Dido1 | 0.54168 | -0.11611633 |
| B3galnt2 | 0.01347 | -1.01042984 |
| Fam73a | 0.00202 | 2.57846443 |
| Elp2 | 0.57075 | -0.34857136 |
| Cntn4 | 0.00372 | -2.29007592 |
| Camkk2 | 0.29221 | 0.36592112 |
| Zfp365 | 0.16253 | 0.40712813 |
| Cyfip1 | 0.14411 | -0.35289414 |
| Tob2 | 0.0532 | -0.85760108 |
| Pex5l | 0.08446 | -0.62224449 |
| Pprc1 | 0.13357 | -1.91227912 |
| Ppfibp1 | 0.05299 | -0.44369326 |
| Gm4793 | 0.02562 | -3.62043555 |
| Ankrd17 | 0.04919 | -0.77371792 |
| Cpsf7 | 0.11034 | 0.8631198 |
| AW011738 | 0.80029 | -0.04614885 |
| Ift27 | 0.00144 | -1.85412607 |
| Junb | 0.00794 | -1.44547589 |
| Grik1 | 0.00144 | -1.01563816 |
| Lrrfip1 | 0.41778 | -0.23233892 |
| Mboat7 | 0.11479 | -2.00380358 |
| Celf3 | 0.00892 | -0.56364821 |
| Gm38413 | 0.33663 | -1.20727663 |
| Abi3bp | 0.00144 | -1.28898079 |
| Rarb | 0.98802 | -0.00357309 |
| Dnajc9 | 0.35369 | -0.20766705 |
| Dok4 | 0.00144 | -1.03756789 |
| Ganab | 0.89841 | -0.06018487 |
| Syn2 | 0.08435 | 0.46551814 |
| Gm1604a | 0.00144 | -1.31884045 |
| Nup107 | 0.00496 | -1.32801949 |
| Nfrkb | 0.21068 | -0.54444758 |
| 9630013A20Rik | 0.00144 | -1.28116048 |
| Sema5a | 0.09869 | -2.71896964 |
| Brpf1 | 0.29609 | -0.22408924 |
| LOC102635912 | 0.03496 | 1.26279978 |
| Mef2c | 0.00929 | 1.8450377 |
| Nalcn | 0.08287 | -0.63429622 |
| Herpud2 | 0.16438 | -1.13415498 |
| Zfp638 | 0.04509 | 1.7857562 |
| Suclg2 | 0.02043 | -0.94123861 |
| D6Wsu163e | 0.46104 | 0.28934346 |
| Camkk2 | 0.05477 | -0.712352 |
| 1700008O03Rik | 0.00585 | -1.07062058 |
| Styx | 0.17253 | 0.22720903 |
| Zfp536 | 0.06803 | -0.48909094 |
| Ap5m1 | 0.00144 | -1.12607953 |
| Nron | 0.95362 | -0.01925628 |
| Minpp1 | 0.10782 | -0.45144949 |
| Rfc3 | 0.70753 | -0.10679983 |
| Man1a | 0.00888 | -0.61219867 |
| Lin9 | 0.1738 | -0.48255788 |
| E430016F16Rik | 0.00144 | -1.29085005 |
| Polk | 0.01461 | -1.00214209 |
| Reck | 0.02273 | 0.58470833 |
| Rnf115 | 0.01062 | 0.59842981 |
| Glb1 | 0.36963 | -0.40936869 |
| E430016F16Rik | 0.00144 | -1.26428512 |
| Lama2 | 0.04723 | -0.40622233 |
| Kitl | 0.82934 | -0.05087303 |
| Skap1 | 0.00162 | -1.25521165 |
| Cep152 | 0.50649 | 0.54540672 |
| Kifc1 | 0.1314 | -0.83670672 |
| Hmgxb3 | 0.68354 | -0.14871182 |
| Ptbp2 | 0.61673 | -0.26882662 |
| C78197 | 0.02303 | 1.38531843 |
| Lcor | 0.03319 | -0.55578449 |
| Epm2a | 0.03384 | 1.46929459 |
| Vwa9 | 0.84838 | -0.1153206 |
| Myof | 0.07609 | -0.73115991 |
| Nmnat3 | 0.09511 | -0.33766419 |
| Swap70 | 0.02614 | 0.8123283 |
| Ablim1 | 0.09304 | 0.97239444 |
| Qrsl1 | 0.5517 | 0.38743084 |
| Alpk1 | 0.31514 | -0.67067277 |
| Ammecr1l | 0.00144 | -1.29085005 |
| Pde3a | 0.00144 | -1.13975156 |
| Prkd2 | 0.94499 | -0.02263108 |
| Mr1 | 0.1778 | -0.74293956 |
| Gm34186 | 0.08469 | -0.42979097 |
| B130055M24Rik | 0.80261 | 0.15776272 |
| Dock10 | 0.11555 | -1.14145454 |
| Plg | 0.00144 | -1.31884045 |
| Acad12 | 0.14232 | 0.49514527 |
| Timp4 | 0.16212 | 0.40993301 |
| Neil1 | 0.01935 | -2.34677085 |
| Eda | 0.00144 | -1.29085005 |
| 4632404H12Rik | 0.2913 | 0.21566232 |
| Fkbp8 | 0.03856 | -0.70826465 |
| Cnst | 0.43859 | -0.31105775 |
| Bcam | 0.04793 | -0.66916176 |
| Ubap2l | 0.26588 | 0.39423615 |
| Cacfd1 | 0.00379 | -0.87260078 |
| B4galnt1 | 0.02289 | -1.82606467 |
| Ranbp2 | 0.26424 | 0.4451947 |
| Ldlrad4 | 0.00867 | -0.72646206 |
| Mgea5 | 0.04359 | -0.50880381 |
| Ccdc18 | 0.00158 | -1.15468698 |
| Smoc2 | 0.30463 | -0.567869 |
| Nedd4l | 0.88379 | 0.08233102 |
| Il21r | 0.90064 | 0.0496876 |
| Aqp11 | 0.8371 | 0.07716069 |
| Tns4 | 0.15557 | -0.73077324 |
| A430010J10Rik | 0.00144 | -1.31495282 |
| Piga | 0.18164 | -0.73545871 |
| Gfpt1 | 0.28525 | -0.80892988 |
| Mypn | 0.01295 | -0.72395214 |
| 9330164J24Rik | 0.00144 | -1.20555712 |
| Dpp9 | 0.11025 | -2.87743082 |
| Gm38832 | 0.00329 | 1.20615502 |
| Ophn1 | 0.00272 | -1.1074877 |
| Kidins220 | 0.12403 | 0.89147471 |
| Ephb1 | 0.11869 | -0.39500805 |
| Fam13c | 0.36903 | 0.36656911 |
| Gm14696 | 0.00144 | -1.25118919 |
| Gm10684 | 0.4021 | -0.87682572 |
| Nemf | 0.00336 | 0.76981917 |
| Brdt | 0.01701 | 1.31008036 |
| Tulp4 | 0.17755 | -1.55174447 |
| Gm14696 | 0.00145 | -1.25646236 |
| Rimklb | 0.00145 | -1.22518835 |
| Sema4g | 0.07438 | -0.66865249 |
| A230056P14Rik | 0.10582 | -0.59594878 |
| Magi2 | 0.96887 | -0.02252538 |
| Ptprm | 0.51552 | 0.29208411 |
| Shtn1 | 0.00145 | -1.22376446 |
| Gorab | 0.0212 | 0.92036244 |
| Ssbp2 | 0.06084 | -0.95838763 |
| Gm9801 | 0.00144 | 1.38587692 |
| 2010300C02Rik | 0.00144 | -1.32153155 |
| Nalcn | 0.19891 | 0.41950814 |
| Gm15624 | 0.04193 | 1.59003722 |
| Tldc1 | 0.00551 | -1.4828437 |
| Cspp1 | 0.00162 | 1.56918966 |
| Enpp2 | 0.00598 | -1.4377193 |
| P4htm | 0.00528 | -1.09451268 |
| Med15 | 0.01098 | 0.83587347 |
| Gm32639 | 0.13663 | -0.83880545 |
| Col4a3bp | 0.47031 | 0.30605859 |
| Gm39233 | 0.00787 | -1.01334489 |
| Csmd1 | 0.26475 | -0.54603011 |
| Lrp8 | 0.03667 | -1.01899805 |
| Rab2b | 0.14614 | 0.59231002 |
| Radil | 0.02634 | -0.75135621 |
| C030029H02Rik | 0.05566 | -0.69650564 |
| Gtf3c1 | 0.13194 | 0.75006411 |
| Drp2 | 0.14565 | 0.59498127 |
| Smarca1 | 0.11617 | -0.63392351 |
| Hebp2 | 0.07596 | -0.93936745 |
| Kcnv1 | 0.79071 | 0.21591387 |
| Them4 | 0.028 | 1.06611202 |
| Pyroxd2 | 0.01379 | -0.50023994 |
| Gramd3 | 0.56929 | 0.33978422 |
| Sec24a | 0.09405 | 1.20195083 |
| Vipas39 | 0.03714 | 0.99663917 |
| Gripap1 | 0.00475 | 1.21149466 |
| Eif3k | 0.01835 | -1.06953367 |
| Slc7a11 | 0.00144 | -1.26560808 |
| Med12l | 0.10482 | -2.48661946 |
| Arhgap4 | 0.1041 | -0.70786405 |
| Ermp1 | 0.35383 | -1.23342415 |
| Ankra2 | 0.01376 | 1.34458422 |
| 9530082P21Rik | 0.08148 | -0.54833964 |
| Mob2 | 0.00144 | 1.95269144 |
| Ar | 0.39344 | -0.31834418 |
| Cyp2j9 | 0.00916 | -1.06669723 |
| Tnpo1 | 0.5477 | 0.24601581 |
| Lnpep | 0.00245 | 2.17538374 |
| Gm36582 | 0.00144 | -1.29864962 |
| Krt80 | 0.44802 | -0.32332121 |
| Gpr31b | 0.39387 | -0.51318328 |
| Dsc1 | 0.27911 | -0.54146095 |
| Arhgef3 | 0.73132 | -0.17098168 |
| Cyth4 | 0.1479 | -1.15625256 |
| Ddx6 | 0.02649 | 1.41911463 |
| Ddx24 | 0.26602 | -0.24033325 |
| Rictor | 0.00601 | 2.43321387 |
| Klhl26 | 0.0325 | -0.49200049 |
| Raf1 | 0.78029 | 0.11362359 |
| Ldah | 0.00144 | 2.93617469 |
| Pde8b | 0.04897 | -1.11046515 |
| Armc9 | 0.85891 | 0.07614709 |
| Gm26803 | 0.00149 | -1.2703832 |
| Ltbp1 | 0.00484 | -0.73769134 |
| Dph6 | 0.55833 | -0.93483848 |
| Ank2 | 0.02249 | -0.45359722 |
| A730087J23Rik | 0.19765 | -0.62267822 |
| Ncoa1 | 0.27189 | 0.31799779 |
| Tbccd1 | 0.04085 | 0.50571571 |
| Xpo7 | 0.00144 | -1.28898079 |
| Ints9 | 0.58005 | -0.50239506 |
| 9630001P10Rik | 0.08651 | -0.61748094 |
| Usp37 | 0.07321 | -1.03660731 |
| BB123696 | 0.00528 | 1.29678591 |
| Chst15 | 0.01964 | -0.62816967 |
| Mtus1 | 0.01909 | 0.49978431 |
| Rangap1 | 0.31945 | 0.18504773 |
| Ppfibp2 | 0.14456 | -0.75632578 |
| Bmp3 | 0.00144 | -1.29085005 |
| Cab39l | 0.02312 | 1.27628608 |
| Dicer1 | 0.02106 | 0.79532426 |
| Rnf111 | 0.01237 | 1.5009345 |
| Lrig3 | 0.00587 | -1.14365968 |
| Smarca1 | 0.00144 | -1.27165604 |
| C530005A16Rik | 0.03994 | 0.50787351 |
| Slc25a36 | 0.00814 | 0.68810799 |
| Gucy1a2 | 0.00144 | 2.67323107 |
| Adamts10 | 0.05733 | 1.2640678 |
| Tiam1 | 0.21324 | 0.94838764 |
| Ptprm | 0.88413 | 0.04730354 |
| Nyap2 | 0.00144 | -1.30713006 |
| Adamts10 | 0.00472 | -0.68270581 |
| Gm40893 | 0.00144 | -1.28898079 |
| Gabrg2 | 0.00145 | -1.24191372 |
| 1110037F02Rik | 0.00827 | 0.67836749 |
| Klhl12 | 0.10975 | 0.29036427 |
| 9330159F19Rik | 0.17932 | -2.20468514 |
| Rnf13 | 0.33761 | -0.44505409 |
| Thrap3 | 0.64581 | 0.14925412 |
| Tbc1d5 | 0.02827 | 1.00305036 |
| Irak2 | 0.00162 | 1.69788366 |
| Lpin2 | 0.67349 | 0.10958817 |
| Pcgf3 | 0.24404 | -0.43388036 |
| Alkbh8 | 0.00144 | 1.268786 |
| Uhrf1bp1l | 0.04284 | 1.4948831 |
| Mbtd1 | 0.00144 | 1.43332185 |
| Btbd7 | 0.088 | 0.87364067 |
| Gm40932 | 0.00873 | -1.13435495 |
| 2210018M11Rik | 0.31401 | 0.31155463 |
| Masp1 | 0.007 | 1.48005329 |
| Atad2 | 0.25563 | -0.42454317 |
| Exoc2 | 0.00144 | -1.28898079 |
| Robo1 | 0.81884 | 0.09160139 |
| Dnajc14 | 0.03956 | -0.38640692 |
| Ap2b1 | 0.03235 | 1.24857144 |
| Mina | 0.00158 | -1.2446827 |
| 6430573F11Rik | 0.05997 | 0.72834919 |
| Tctn2 | 0.307 | -0.25121829 |
| Atp2c1 | 0.26086 | 0.56214004 |
| Calb2 | 0.17687 | -0.77971246 |
| Anxa7 | 0.02361 | 0.93594911 |
| Ilf2 | 0.38788 | 0.31839074 |
| Kcnj8 | 0.01295 | -0.54982271 |
| Sel1l3 | 0.00144 | -1.26934956 |
| Dock4 | 0.00144 | -1.30017714 |
| Ccnf | 0.73652 | -0.20424336 |
| Arid5b | 0.08078 | -1.25777379 |
| Pced1a | 0.39079 | -0.20306155 |
| 1700012D14Rik | 0.89564 | 0.04956951 |
| Antxr1 | 0.53415 | -0.18840636 |
| D19Bwg1357e | 0.65308 | 0.1532943 |
| L3mbtl3 | 0.04555 | 1.15281029 |
| Fads2 | 0.82269 | -0.1236041 |
| Sema3e | 0.00144 | -1.29051721 |
| Slco3a1 | 0.15251 | 0.69677457 |
| Sfpq | 0.00644 | 3.17164168 |
| Sema5a | 0.00144 | -1.26560808 |
| Tenm2 | 0.00144 | -1.28116048 |
| Msn | 0.157 | 0.42148979 |
| Cnot3 | 0.11042 | -0.66848289 |
| Bcl2l13 | 0.51502 | 0.26232988 |
| Dnah9 | 0.00145 | -1.2388121 |
| Cyth4 | 0.02972 | -0.68445514 |
| Cwc27 | 0.00376 | 1.50866892 |
| Cers4 | 0.01577 | 1.31811302 |
| Lmbr1 | 0.42364 | -0.1275584 |
| Tsr1 | 0.04072 | -0.44316675 |
| Gm41313 | 0.00144 | -1.51667806 |
| Ptpn21 | 0.07836 | 1.38200195 |
| Stard3nl | 0.44712 | 0.19861871 |
| Msra | 0.20294 | -1.46079314 |
| Stk4 | 0.05897 | 0.37807822 |
| Dleu2 | 0.02458 | 0.76017864 |
| Sirpb1b | 0.85479 | 0.14456772 |
| Gm40923 | 0.02268 | -1.04238921 |
| Tmprss5 | 0.10648 | -0.45281799 |
| Ccdc171 | 0.23635 | -0.38110712 |
| Gm9530 | 0.03467 | 0.66574951 |
| Sbf2 | 0.0078 | 0.69046298 |
| 0610010F05Rik | 0.02724 | -0.7533555 |
| Lysmd3 | 0.00145 | 1.44140779 |
| Fubp1 | 0.04339 | -0.67691464 |
| Cdc20 | 0.00253 | -1.32745858 |
| Ablim1 | 0.84533 | 0.04397087 |
| Kat8 | 0.45742 | -0.36514376 |
| Arhgef28 | 0.02575 | -1.20474598 |
| Abca12 | 0.00144 | -1.27165604 |
| Nat6 | 0.18305 | -0.31716217 |
| Sema4b | 0.40875 | -0.25904577 |
| Cdk19 | 0.04488 | 0.57666338 |
| Myo10 | 0.01955 | -0.76717969 |
| Slc9a6 | 0.76892 | 0.06287321 |
| Tia1 | 0.00144 | 1.09850617 |
| Ppm1l | 0.05865 | -0.51788733 |
| Gpank1 | 0.1196 | -0.39815236 |
| Adk | 0.0089 | -0.86845035 |
| Plin1 | 0.00144 | -1.29085005 |
| Klf12 | 0.02293 | 1.08429534 |
| Kidins220 | 0.96125 | -0.02374909 |
| Grm5 | 0.44001 | -0.61983877 |
| Parp6 | 0.9202 | -0.04333121 |
| C2cd5 | 0.59309 | -0.36295948 |
| Dlg2 | 0.11782 | -1.01541588 |
| Gnas | 0.0017 | -2.4413926 |
| Cfap97 | 0.74916 | 0.12920643 |
| Utrn | 0.00523 | 1.01307774 |
| Impg1 | 0.0095 | -1.65033555 |
| Ush2a | 0.00244 | -1.16878422 |
| A930006D01Rik | 0.00144 | -1.28898079 |
| A930001M01Rik | 0.04257 | 0.6664579 |
| Sh2d4b | 0.01098 | -0.65787741 |
| Rcsd1 | 0.15258 | -0.56492935 |
| Pvt1 | 0.34255 | 0.42523764 |
| 9530027D23Rik | 0.00144 | 2.01620972 |
| 9430065F17Rik | 0.07195 | -0.79618843 |
| Irak4 | 0.421 | 0.36797566 |
| Tmem57 | 0.65938 | -0.11439534 |
| 9230117E06Rik | 0.00717 | -1.0591794 |
| Mterf1a | 0.3824 | -0.15375201 |
| Chd9 | 0.98349 | -0.00536792 |
| Gfm2 | 0.00144 | -1.17001102 |
| Gm41664 | 0.15076 | -0.72967794 |
| Mtmr2 | 0.01426 | -0.66613881 |
| P2ry10 | 0.92371 | -0.0311401 |
| Gm32497 | 0.36173 | -0.29123585 |
| Lipe | 0.01786 | 1.01797098 |
| 4930573I07Rik | 0.03938 | -0.45851322 |
| 4930570G05Rik | 0.25459 | -0.48088065 |
| Nup210l | 0.0017 | -1.24950944 |
| 4930546J17Rik | 0.00149 | -1.2703832 |
| 1700086P04Rik | 0.49867 | -0.48046976 |
| 4930505M18Rik | 0.76803 | -0.09657649 |
| 4930513L16Rik | 0.05 | -0.87222379 |
| 4930455G09Rik | 0.21691 | -0.71580823 |
| 4930452B06Rik | 0.12856 | 0.7950262 |
| 4930445N06Rik | 0.00145 | -1.22193674 |
| 4930435C17Rik | 0.00144 | -1.4406427 |
| 3110057O12Rik | 0.0437 | 0.46611172 |
| Pycrl | 0.00144 | -1.22925777 |
| Nifk | 0.00419 | -0.85653042 |
| Dpf2 | 0.05621 | -0.57561482 |
| E030003E18Rik | 0.04566 | 1.48416415 |
| 1700039O17Rik | 0.00144 | -1.29746368 |
| 1700057G04Rik | 0.00144 | -1.29049509 |
| 1700037F03Rik | 0.1296 | -0.7775065 |
| 1700041B01Rik | 0.06595 | -0.89973537 |
| Zfp787 | 0.05131 | -0.56260522 |
| Fam71e1 | 0.00279 | -0.97916911 |
| Ldlr | 0.24679 | -1.21809485 |
| Anxa10 | 0.00144 | -1.31495282 |
| Bcas3os1 | 0.00144 | -1.28116048 |
| Agbl3 | 0.03695 | -0.94185317 |
| Tmod2 | 0.00145 | -1.21051401 |
| Dock8 | 0.10648 | -0.74525715 |
| Ap3m2 | 0.74307 | 0.13763392 |
| 5830416P10Rik | 0.397 | 0.51096452 |
| Adamts14 | 0.43796 | 0.17008788 |
| Zscan25 | 0.09682 | -1.3412565 |
| Cyp2c69 | 0.02503 | -1.03930772 |
| Enpp5 | 0.78824 | 0.05729718 |
| Recql4 | 0.03654 | -0.78274885 |
| Tsen54 | 0.00349 | -0.8674219 |
| Sall3 | 0.0035 | -1.66727772 |
| Pwp2 | 0.32155 | -0.28114911 |
| Klk11 | 0.00198 | -1.12868614 |
| Gata5 | 0.00144 | -1.26560808 |
| Cckbr | 0.65244 | -0.41808438 |
| Atxn7l1 | 0.18678 | 0.61196342 |
| Lin9 | 0.72818 | -0.11519914 |
| Fam114a1 | 0.00153 | 2.00870571 |
| Adamts7 | 0.11082 | -0.29421424 |
| Ulk4 | 0.05189 | -1.38525739 |
| Mlxipl | 0.34044 | 0.35366115 |
| Cdk19 | 0.61247 | 0.10672532 |
| Fam73a | 0.29826 | 0.24143375 |
| Rbak | 0.50841 | 0.19702721 |
| Mark4 | 0.16452 | -0.42475397 |
| Usp3 | 0.00145 | -0.94569111 |
| Btrc | 0.00144 | -1.28898079 |
| Xrn2 | 0.00144 | 1.47705911 |
| Arid1b | 0.02759 | -0.52722263 |
| 2310057M21Rik | 0.20196 | 0.21984267 |
| Nudt8 | 0.02417 | -1.03780376 |
| Cfap44 | 0.1822 | -0.39789868 |
| Sh2b2 | 0.00144 | -1.29085005 |
| Elmod1 | 0.19518 | -0.76217425 |
| Rce1 | 0.00144 | -1.11044616 |
| Slc25a51 | 0.40265 | 0.13632654 |
| Trim33 | 0.00144 | 2.05563462 |
| Slc7a6 | 0.00158 | 0.96177193 |
| Zkscan7 | 0.75447 | -0.0866185 |
| Dennd4c | 0.00144 | 1.81993478 |
| Tube1 | 0.66998 | 0.26586238 |
| Wnk4 | 0.49956 | -0.13777794 |
| Mcts1 | 0.02004 | 0.6154044 |
| Kif1b | 0.00145 | -1.92511713 |
| Fer1l4 | 0.009 | -1.22814825 |
| Ushbp1 | 0.32014 | -0.21984667 |
| Atg16l1 | 0.00244 | 1.67490161 |
| Atad2b | 0.61665 | 0.18727574 |
| Fam21 | 0.08751 | 0.39979996 |
| Dock1 | 0.03222 | -0.66647853 |
| Vps13d | 0.61996 | -0.13248138 |
| Plekhn1 | 0.02993 | 1.11694564 |
| Mylk | 0.15235 | 0.28545841 |
| Gnb1l | 0.00144 | -1.33632959 |
| Rock2 | 0.06155 | -0.34763765 |
| Polr3e | 0.31753 | -0.16334761 |
| Cyld | 0.0781 | 0.3342812 |
| Pbld1 | 0.06626 | -0.45258056 |
| Sil1 | 0.1148 | -1.8637091 |
| Lpcat2 | 0.52527 | 1.17957983 |
| Sox17 | 0.10394 | -0.33217889 |
| Slc7a10 | 0.11331 | -1.32448607 |
| Slc38a2 | 0.35175 | -0.24493021 |
| Jak3 | 0.17374 | -0.42316226 |
| Fgf1 | 0.07113 | -0.33021468 |
| BC026600 | 0.15105 | 0.39315656 |
| Sgip1 | 0.00144 | -1.31014844 |
| Sf3b2 | 0.08816 | -0.35780006 |
| Plin1 | 0.07182 | -2.59360873 |
| Ubtf | 0.00372 | -0.75236367 |
| Gnptg | 0.58445 | -0.09108013 |
| Snupn | 0.51559 | -0.13407123 |
| C330027C09Rik | 0.00144 | 1.29544774 |
| Wdr48 | 0.5271 | -0.23013126 |
| Glo1 | 0.54009 | 0.14610859 |
| Taf1b | 0.00604 | -0.67881497 |
| Hmgcr | 0.50701 | -0.35940839 |
| Cpq | 0.03566 | 0.85409922 |
| Slc22a30 | 0.00144 | -1.30017714 |
| Herc1 | 0.00144 | -1.33632959 |
| Epb4.1l4a | 0.1511 | -0.25719779 |
| Nr3c1 | 0.00398 | -0.70549703 |
| Top3b | 0.49542 | -0.25974257 |
| Zfp697 | 0.53412 | -0.12354825 |
| Ssb | 0.01131 | 1.14981981 |
| Bphl | 0.01743 | -0.91739544 |
| Commd8 | 0.00144 | 1.13877656 |
| Zfp398 | 0.12922 | 0.8228455 |
| Aagab | 0.12321 | 0.49547031 |
| Ipo5 | 0.31128 | 0.34766882 |
| Epha1 | 0.00144 | -1.25118919 |
| Mxi1 | 0.15987 | -0.49766665 |
| Tle4 | 0.03223 | 1.22199793 |
| C530020B09Rik | 0.08929 | -0.38682762 |
| Cherp | 0.55053 | -0.1813709 |
| 5530601H04Rik | 0.02388 | 1.03767011 |
| 5430414B12Rik | 0.00144 | -1.28116048 |
| Pde1c | 0.00653 | 1.62579333 |
| Iqgap1 | 0.00258 | 1.54952885 |
| 4930592I03Rik | 0.00144 | -1.31495282 |
| Aqr | 0.00904 | -0.81582187 |
| 4930568A12Rik | 0.07038 | -0.51104948 |
| 4931428L18Rik | 0.00907 | -1.03958412 |
| Sqrdl | 0.86549 | -0.05270454 |
| Exd2 | 0.13474 | -0.89782899 |
| 4930533D04Rik | 0.00144 | -1.28898079 |
| Kcnj9 | 0.00912 | -1.11367897 |
| Scd3 | 0.89864 | 0.06318701 |
| Zfp85os | 0.00144 | -1.22098834 |
| Kif16bos | 0.65589 | -0.29581812 |
| Tmod2 | 0.00144 | -1.49973687 |
| Spata16 | 0.02666 | -0.95694161 |
| 4930477N07Rik | 0.03346 | -0.94051005 |
| 1700067P10Rik | 0.00144 | -1.29049509 |
| 4930447F24Rik | 0.07763 | -0.62149501 |
| Htatip2 | 0.50966 | -0.22683884 |
| Ckap5 | 0.70037 | 0.1061353 |
| 4931403M11Rik | 0.92847 | 0.13284979 |
| Cfap43 | 0.00144 | -1.33632959 |
| 4930422M22Rik | 0.6984 | -0.12183017 |
| 4930421P22Rik | 0.01678 | -1.66529853 |
| Cxxc5 | 0.41899 | -0.25773104 |
| 4930405D11Rik | 0.00145 | -1.22376446 |
| Cml3 | 0.01668 | 1.1333102 |
| Ept1 | 0.01436 | -0.52279231 |
| Uba3 | 0.22493 | 0.19863445 |
| Pign | 0.13596 | -0.33544452 |
| Kcnmb2 | 0.00144 | -1.31495282 |
| Gm32566 | 0.00974 | -0.69225314 |
| Kif23 | 0.02684 | 1.01796864 |
| Map2k5 | 0.05393 | 0.89393848 |
| Anxa3 | 0.0292 | 0.5232852 |
| Mrpl42 | 0.54985 | 0.10489699 |
| Fmn2 | 0.00244 | -1.16878422 |
| Rcbtb2 | 0.00655 | 1.47647896 |
| Azi2 | 0.00144 | -1.20511198 |
| 2810429I04Rik | 0.81757 | 0.1341237 |
| Gbe1 | 0.00144 | 3.07295471 |
| Ninl | 0.20439 | 0.65200889 |
| Zfp248 | 0.07014 | 0.55712575 |
| Mgea5 | 0.00506 | -1.8408636 |
| Exosc3 | 0.95088 | 0.01227023 |
| Dnah17 | 0.00793 | -1.14764783 |
| Hbb-bh1 | 0.01531 | -1.074865 |
| Col25a1 | 0.0471 | -1.72538021 |
| Lypd1 | 0.01336 | -1.68304857 |
| Serf1 | 0.00553 | -0.8657374 |
| Ggps1 | 0.95169 | 0.01711432 |
| Rfc5 | 0.00152 | -1.33357767 |
| Ybx1 | 0.21797 | -0.51595945 |
| 2610204G07Rik | 0.00147 | -1.32115804 |
| Igf2bp3 | 0.00144 | -1.31884045 |
| Smarcad1 | 0.99714 | 0.00076697 |
| Agk | 0.00149 | -1.25408 |
| Rnf14 | 0.00144 | 1.24029642 |
| Rbms1 | 0.74176 | -0.12474369 |
| Pole2 | 0.04204 | -1.96567086 |
| 2410164B09Rik | 0.01485 | -0.63775473 |
| Phf12 | 0.02966 | 0.54174235 |
| Znhit6 | 0.35904 | 0.27357066 |
| Cdkn3 | 0.56943 | -0.26283196 |
| Eif2ak3 | 0.08757 | -0.5530477 |
| 2310081O03Rik | 0.00144 | -1.29085005 |
| Ppp2r5e | 0.00643 | 1.26911831 |
| Rnf14 | 0.69427 | 0.17323248 |
| Dqx1 | 0.9083 | -0.05198124 |
| 2310058D17Rik | 0.04719 | -0.55819812 |
| 2310047D07Rik | 0.08692 | -0.47127483 |
| Gp2 | 0.98548 | -0.02474722 |
| Zkscan14 | 0.00494 | -0.97593511 |
| Car14 | 0.81817 | -0.10147027 |
| Med27 | 0.4601 | -0.19741769 |
| Rbm39 | 0.00453 | 0.95425176 |
| Mettl21a | 0.95069 | 0.0142997 |
| 2210018M11Rik | 0.0027 | 0.85175206 |
| 2610203C22Rik | 0.56479 | -0.13484042 |
| Ppp1r2 | 0.95469 | 0.01039056 |
| Myo1e | 0.19011 | -0.44298038 |
| Lipn | 0.83387 | -0.16291573 |
| Slc44a1 | 0.02881 | 0.80701103 |
| 2210404E10Rik | 0.00158 | -1.78454565 |
| 2210022D18Rik | 0.19416 | -2.47152198 |
| Gtpbp2 | 0.43563 | -0.22968797 |
| Atp4b | 0.03276 | -1.14544934 |
| Pgam1 | 0.22888 | -0.31353854 |
| Cmc1 | 0.70594 | 0.07647712 |
| Tmem134 | 0.00303 | -1.20912515 |
| 2010109N18Rik | 0.00144 | -1.3348153 |
| Hps4 | 0.00144 | -1.02419909 |
| 2010203P06Rik | 0.01318 | -1.15782907 |
| 2010103J01Rik | 0.00144 | -1.27165604 |
| Map7 | 0.17228 | -0.45036498 |
| Fibp | 0.40344 | -0.25190473 |
| Tmigd1 | 0.72846 | 0.19047488 |
| Abhd11os | 0.03019 | -0.89421575 |
| Rnf13 | 0.00334 | -0.92121447 |
| Luc7l | 0.03029 | 0.98317213 |
| Ggps1 | 0.01563 | 1.33899069 |
| A930005H10Rik | 0.72993 | -0.11747448 |
| Zkscan6 | 0.35159 | -0.1483453 |
| Maea | 0.86207 | 0.06655705 |
| 1700122E12Rik | 0.03374 | -1.03043839 |
| Parp6 | 0.28962 | 0.36597547 |
| 1700116H05Rik | 0.04517 | -1.0148703 |
| Msi2 | 0.46804 | -0.17671155 |
| 1700096P03Rik | 0.00144 | -1.24613586 |
| 1700095J12Rik | 0.38093 | -0.4528452 |
| LOC102633456 | 0.56336 | -0.16967257 |
| 1700095A13Rik | 0.00144 | -1.27699683 |
| 1700003E24Rik | 0.0611 | -1.85671057 |
| Ptar1 | 0.00161 | -1.02376835 |
| 1700081N11Rik | 0.91749 | -0.02540154 |
| Ces5a | 0.00372 | -1.35870099 |
| 1700072G22Rik | 0.01798 | -1.31080856 |
| 1700066O22Rik | 0.09472 | -2.54311587 |
| 1700063H06Rik | 0.02272 | -1.18564018 |
| LOC105243184 | 0.5414 | 0.17868608 |
| Prdx2 | 0.10893 | -0.6434411 |
| Mettl6 | 0.71457 | 0.06755439 |
| 1700041M05Rik | 0.00144 | -1.31884045 |
| 1700038P13Rik | 0.00242 | -1.65752876 |
| 4930567K20Rik | 0.01086 | -1.64046339 |
| Fmn1 | 0.13395 | -0.43115851 |
| 1700037J18Rik | 0.00144 | -1.26934956 |
| R3hcc1l | 0.00144 | -1.30713006 |
| 4933429O19Rik | 0.00144 | -1.27165604 |
| 1700003P14Rik | 0.00144 | -1.30017714 |
| 1700030N03Rik | 0.00144 | -1.4554796 |
| 1700028E11Rik | 0.21423 | 0.42510344 |
| 1700027F06Rik | 0.57138 | 0.13582672 |
| Tsc22d4 | 0.004 | -0.7463166 |
| 1700022N22Rik | 0.04557 | -1.21153109 |
| Gm5563 | 0.69317 | -0.30309943 |
| Ttll8 | 0.34353 | -0.30273047 |
| Gabarapl1 | 0.44825 | 0.26444366 |
| Diap3 | 0.28564 | 0.35074332 |
| Pdcl2 | 0.00144 | -1.30253725 |
| Ahi1 | 0.15337 | -0.35468789 |
| 1700015C17Rik | 0.0015 | -1.30632114 |
| 4930534H18Rik | 0.02393 | -1.06802371 |
| Med9os | 0.25246 | -0.38151398 |
| Spata31d1a | 0.23052 | -0.47026189 |
| Rsph3a | 0.96672 | -0.014576 |
| 1700012C14Rik | 0.04597 | -0.90881164 |
| 4930542C16Rik | 0.00144 | -1.29085005 |
| Plac8l1 | 0.27413 | 0.31420394 |
| Ankrd36 | 0.00149 | -1.21097148 |
| Lrrc75aos2 | 0.00144 | -1.31884045 |
| Rab10os | 0.01305 | 0.78643532 |
| Rsf1os1 | 0.02356 | 1.3194384 |
| Srsf5 | 0.02151 | -0.75706602 |
| Dlst | 0.05338 | 1.85939089 |
| Prl7d1 | 0.30126 | -0.67287189 |
| Cnrip1 | 0.17182 | 0.26715513 |
| 1500035N22Rik | 0.00218 | -0.91461733 |
| Tra2a | 0.54057 | 0.11675373 |
| Nisch | 0.02685 | -0.65747466 |
| Gm7008 | 0.00317 | -1.11935153 |
| Anapc5 | 0.82329 | -0.0787316 |
| Slc35b2 | 0.00794 | -0.80492887 |
| Qk | 0.00145 | 1.272565 |
| Zfp235 | 0.16216 | 0.63739666 |
| Wdr27 | 0.00144 | -1.53488392 |
| Capn10 | 0.00352 | -0.79283743 |
| 1110019B22Rik | 0.00144 | 1.19113863 |
| Fam46c | 0.47509 | -0.17610546 |
| Dgkeos | 0.02233 | -0.81788516 |
| Sfi1 | 0.08524 | 0.37573208 |
| LOC102639672 | 0.04384 | -0.72876197 |
| Gm21861 | 0.04725 | -1.12694059 |
| Kcnq1ot1 | 0.00144 | 2.630852 |
| Snx4 | 0.02554 | -0.49057151 |
| Nsmce2 | 0.22944 | 0.21995826 |
| Sh2d4b | 0.04708 | -1.88252778 |
| Trim27 | 0.61832 | -0.13621043 |
| Gm30948 | 0.03925 | 0.69003243 |
| LOC102635133 | 0.00272 | -1.15217138 |
| Gm34735 | 0.31071 | -0.43301399 |
| Celsr3 | 0.06275 | 0.78594847 |
| Kcnq1ot1 | 0.00144 | 3.38557889 |
| Dlc1 | 0.60295 | 0.19361546 |
| Gm34280 | 0.49056 | -0.68207152 |
| Gm30482 | 0.4195 | -0.36500673 |
| N4bp2l1 | 0.25401 | 0.24263147 |
| Gm38488 | 0.00144 | 1.86614159 |
| Gm30522 | 0.01797 | -1.25517825 |
| Gm38442 | 0.00144 | -1.30713006 |
| Gm26763 | 0.00144 | -1.28116048 |
| Mup-ps12 | 0.00474 | -1.10247618 |
| Usp53 | 0.00594 | -1.41524904 |
| Usp53 | 0.11953 | -0.69264889 |
| Gm38473 | 0.44634 | 0.42182038 |
| Gm32952 | 0.00149 | -1.22582153 |
| Gm21075 | 0.16221 | 0.42308879 |
| LOC102641753 | 0.0108 | -1.14181089 |
| LOC102641156 | 0.36715 | -1.23113036 |
| LOC102642748 | 0.00144 | -1.30017714 |
| 1700095A21Rik | 0.00144 | -1.30017714 |
| LOC102642754 | 0.00144 | -1.29051721 |
| LOC102641139 | 0.00144 | 1.81769048 |
| Gm15205 | 0.09092 | -2.002951 |
| Gm35324 | 0.9651 | -0.02262177 |
| Gm35324 | 0.00913 | -1.96027748 |
| LOC102642116 | 0.00144 | -1.27165604 |
| Zfp119a | 0.00144 | -1.28898079 |
| Gm15320 | 0.04008 | 0.84263399 |
| Gm30255 | 0.00144 | -1.30713006 |
| Gm29907 | 0.00144 | -1.30713006 |
| Gm32970 | 0.00144 | -1.27730451 |
| Gm21464 | 0.00144 | -1.45144986 |
| Zmiz1os1 | 0.00654 | -1.01034857 |
| Zmiz1os1 | 0.00144 | -1.26560808 |
| 3830408C21Rik | 0.47213 | 0.26679492 |
| Gm32702 | 0.08995 | -2.20427195 |
| Gm31977 | 0.00276 | -1.14169705 |
| Gm38508 | 0.00272 | -1.09759392 |
| Gm38504 | 0.02739 | -1.87283484 |
| Gm36785 | 0.00144 | -1.33632959 |
| Gm36349 | 0.00144 | 2.17489621 |
| 2010111I01Rik | 0.57274 | -0.19718326 |
| 4930559C10Rik | 0.00144 | -1.31884045 |
| 1700083H02Rik | 0.18523 | -0.50443514 |
| Gm16342 | 0.05941 | -0.85634032 |
| Fam57a | 0.62503 | 0.18651043 |
| Fignl1 | 0.00144 | -1.28375735 |
| 4930471D02Rik | 0.00144 | -1.20616817 |
| Rtkn2 | 0.00144 | -1.27165604 |
| Gm5079 | 0.06911 | -0.64727086 |
| Gm38480 | 0.11361 | 1.22891501 |
| LOC102642723 | 0.00257 | -1.6701319 |
| LOC102641570 | 0.01783 | 1.05725575 |
| LOC102641570 | 0.55058 | -0.46559697 |
| Gm36033 | 0.00868 | -1.29837361 |
| Gm32695 | 0.13895 | -1.08525034 |
| Rccd1 | 0.00218 | -1.14892174 |
| 4930533N22Rik | 0.65312 | -0.34297076 |
| Gm33627 | 0.15324 | -2.28333048 |
| LOC102633308 | 0.00144 | -1.3348153 |
| Zfp853 | 0.00144 | -1.2841476 |
| Erp29 | 0.00325 | 1.2616581 |
| LOC102639958 | 0.00946 | -1.0032338 |
| LOC102641583 | 0.00234 | -1.48813035 |
| Gm33187 | 0.10007 | -0.39187955 |
| Gm33187 | 0.45652 | -0.36389994 |
| LOC102643206 | 0.00729 | -1.15082629 |
| LOC102643085 | 0.00144 | -1.26934956 |
| Fktn | 0.97134 | 0.02215126 |
| Gm30677 | 0.00145 | -1.2388121 |
| LOC102643270 | 0.00145 | -1.25646236 |
| LOC102637512 | 0.04458 | 1.52685244 |
| Gm12500 | 0.00144 | -1.30017714 |
| 1700017M07Rik | 0.10036 | -0.60176841 |
| 6430550D23Rik | 0.04012 | -0.97701755 |
| 6430550D23Rik | 0.19252 | 0.35659315 |
| 6430550D23Rik | 0.77255 | -0.14292897 |
| 6430550D23Rik | 0.11361 | -1.08020429 |
| 6430550D23Rik | 0.01872 | -1.08154892 |
| a | 0.00144 | -1.29085005 |
| a | 0.00144 | -1.27730451 |
| a | 0.00144 | -1.29051721 |
| Gm38479 | 0.79561 | -0.31459725 |
| Gm31699 | 0.00521 | -1.05862264 |
| LOC102642774 | 0.00145 | -1.2388121 |
| Gm7068 | 0.01074 | -1.04347137 |
| 1700025G04Rik | 0.00144 | -1.30713006 |
| Gm7759 | 0.00144 | -1.27165604 |
| Rbx1 | 0.05202 | -0.65770698 |
| Wdr46 | 0.08849 | -0.41574188 |
| Olfr316 | 0.09138 | -0.90686363 |
| Farsb | 0.27579 | -0.23806614 |
| Sly | 0.00526 | -1.02478608 |
| Gm20806 | 0.00144 | -1.28116048 |
| Gm20747 | 0.03695 | -2.68789823 |
| Frmpd3 | 0.00396 | -0.77640645 |
| Gm16404 | 0.00144 | 1.31003266 |
| Dmrt1 | 0.00144 | -1.25927895 |
| Gm38522 | 0.90415 | -0.0848258 |
| H2-T23 | 0.14014 | -0.31660506 |
| T | 0.00144 | -1.27165604 |
| Sim2 | 0.01033 | -1.57279839 |
| Hmgn1 | 0.46077 | -0.45618478 |
| Nsmce2 | 0.00839 | 0.56327878 |
| Abcd2 | 0.00145 | -1.2388121 |
| Tmem74 | 0.00144 | -1.28116048 |
| Gm35078 | 0.07797 | -1.16603108 |
| Gkap1 | 0.64492 | -0.14966519 |
| Fam172a | 0.6039 | 0.35098408 |
| Gpr137b | 0.00433 | -0.83105528 |
| Arhgap5 | 0.01212 | -1.18860717 |
| Tcl1b5 | 0.89939 | 0.10710317 |
| Pacs2 | 0.65665 | -0.19516234 |
| Ulk2 | 0.52789 | 0.10293301 |
| Il9r | 0.00144 | -1.28898079 |
| Tspan8 | 0.11871 | -0.55051004 |
| Rab21 | 0.54928 | -0.12627399 |
| Lims1 | 0.48931 | 0.21262591 |
| Cdc25a | 0.03993 | -0.40372119 |
| Cdc25a | 0.05867 | -0.34560765 |
| Dalrd3 | 0.16967 | -0.26943008 |
| Aph1c | 0.00725 | -0.77138433 |
| Pias1 | 0.01241 | 0.84080092 |
| Igdcc3 | 0.04009 | -2.07939829 |
| Dnm2 | 0.99579 | 0.00159909 |
| Fanca | 0.29007 | 0.33821582 |
| Nek5 | 0.00149 | -1.25408 |
| Olfr1532-ps1 | 0.00144 | -1.28116048 |
| Hs3st2 | 0.03098 | -0.49325025 |
| Hs3st2 | 0.02724 | -1.0081862 |
| Grin2d | 0.00903 | -1.18713134 |
| LOC105242401 | 0.00144 | -1.25118919 |
| Nlrp4e | 0.0017 | -1.0673118 |
| Gm4736 | 0.30463 | -0.19471707 |
| Klra6 | 0.00144 | -1.28116048 |
| Tmed5 | 0.10939 | -0.56068006 |
| Gm38457 | 0.00752 | -0.75535923 |
| Gm1043 | 0.00144 | -1.25118919 |
| Hmx1 | 0.48902 | 0.156997 |
| Lrrd1 | 0.00144 | -1.27699683 |
| Gucy1a3 | 0.86409 | -0.07132673 |
| 4930564D02Rik | 0.00144 | -1.24613586 |
| Ccna1 | 0.00247 | -1.61748094 |
| Tbl1xr1 | 0.26291 | -0.73836537 |
| Mfsd7b | 0.01282 | -1.47046078 |
| Rabgap1l | 0.18245 | -0.27009557 |
| Rabgap1l | 0.00145 | -1.22376446 |
| Tsga10 | 0.28479 | -0.51885759 |
| Vmn2r123 | 0.00144 | -1.28898079 |
| LOC102643251 | 0.94614 | 0.0359935 |
| LOC102642951 | 0.95916 | -0.03439897 |
| Sirpb1a | 0.00155 | -1.2255836 |
| LOC102641200 | 0.00165 | -1.56841357 |
| Gm7102 | 0.33546 | -0.2111312 |
| Rpl32 | 0.00144 | -1.06843664 |
| Gm33700 | 0.00144 | -1.33632959 |
| Gm19410 | 0.19626 | 0.46977949 |
| Gm38499 | 0.00193 | -1.05888546 |
| Gm1043 | 0.39467 | -0.49176971 |
| Lyn | 0.88482 | 0.03280829 |
| Zfp64 | 0.03363 | -1.47597563 |
| LOC102642530 | 0.00144 | -1.26560808 |
| 9230116L04Rik | 0.00144 | -1.29746368 |
| 6530402F18Rik | 0.05817 | -0.45632242 |
| Rian | 0.21755 | 0.50517061 |
| 1600029I14Rik | 0.00145 | -1.22376446 |
| Trmt61b | 0.78727 | -0.05144256 |
| Gm17066 | 0.00144 | 2.44783965 |
| Gm3086 | 0.18614 | 0.35662682 |
| Crnde | 0.10246 | -0.36878348 |
| Lrrc17 | 0.1046 | -0.4370989 |
| Rnu1a1 | 0.01614 | -0.75170551 |
| Rn7s1 | 0.00231 | -0.92719948 |
| Trim52 | 0.01143 | 1.12799204 |
| Gm1943 | 0.7901 | 0.06239164 |
| Rn7sk | 0.00144 | -1.13558495 |
| Rnu2-10 | 0.08834 | -0.34054127 |
| Rnu1b1 | 0.01362 | -0.82338612 |
| Nfkb2 | 0.05277 | -0.56613175 |
| Tnfsf4 | 0.00144 | -1.32599123 |
| Gm5886 | 0.00144 | -1.28116048 |
| Lgals6 | 0.04043 | 1.39568047 |
| Rel | 0.02963 | -0.92343493 |
| Sim2 | 0.00144 | -1.32766452 |
| Ager | 0.58787 | 0.15957209 |
| Nefm | 0.32259 | 0.41829822 |
| Adcy1 | 0.04566 | 0.4999099 |
| Gcm2 | 0.10529 | 0.56601244 |
| Rfx3 | 0.00932 | 1.11790739 |
| Zfp600 | 0.52339 | -0.35974574 |
| Rex2 | 0.10588 | -0.34190652 |
| Gabrr1 | 0.26813 | -0.26573881 |
| Tlcd2 | 0.42875 | 0.14486985 |
| Lsm11 | 0.02895 | -0.51223269 |
| Hist1h2bf | 0.0505 | -0.41027146 |
| Slamf6 | 0.00145 | -1.22376446 |
| Lrrc16b | 0.00684 | -0.66915885 |
| Srp54a | 0.25292 | -0.20913706 |
| Nudcd1 | 0.02302 | 0.59647963 |
| Hspa12a | 0.00819 | 0.765484 |
| Cd209g | 0.66582 | -0.08830666 |
| Zfp322a | 0.00992 | 0.97733108 |
| Tns4 | 0.41186 | -0.25984282 |
| Cldn34b1 | 0.00202 | -1.42919588 |
| Spata31d1c | 0.00145 | -1.25646236 |
| Fgfbp1 | 0.01307 | -0.9108167 |
| Sgk3 | 0.94092 | 0.0248845 |
| Olfr462 | 0.00144 | -1.28116048 |
| Gm7102 | 0.16921 | 0.3943901 |
| Olfr177 | 0.00197 | -1.37159448 |
| Olfr538 | 0.2009 | -0.76426058 |
| Ear12 | 0.0323 | -1.97782066 |
| Ear7 | 0.00144 | -1.30713006 |
| Ptma | 0.01634 | -0.97740973 |
| Rap1a | 0.2036 | 0.28654781 |
| Ppp4r1l-ps | 0.67781 | 0.11136887 |
| Gm5803 | 0.00449 | 0.87384747 |
| Tomm20 | 0.95397 | -0.02840561 |
| Rps15 | 0.00144 | -1.20462157 |
| Atp1b3 | 0.01021 | 0.70419425 |
| Gle1 | 0.79533 | -0.06534297 |
| Olfr205 | 0.00144 | -1.29864962 |
| Gm5592 | 0.04987 | -1.22739838 |
| Rb1cc1 | 0.00144 | 1.3180232 |
| Sly | 0.13212 | -1.91531813 |
| Sly | 0.00144 | -1.28116048 |
| Tuba1c | 0.49818 | -0.25651623 |
| Rps3a1 | 0.47227 | -0.1132466 |
| Dcun1d1 | 0.00292 | 0.76332138 |
| Sly | 0.00144 | -1.23532597 |
| Coro1c | 0.07224 | -0.37185583 |
| Frem1 | 0.9311 | -0.12249296 |
| Anapc16 | 0.78135 | 0.08160397 |
| Anapc16 | 0.93399 | -0.02457589 |
| Mast4 | 0.01824 | -1.16859575 |
| Mast4 | 0.01521 | 1.14229279 |
| Mast4 | 0.95497 | 0.02957208 |
| Mast4 | 0.01156 | 1.35129796 |
| Mast4 | 0.10308 | 0.75585528 |
| Pcdh15 | 0.056 | -0.63079701 |
| Nr2f6 | 0.07625 | -0.47654629 |
| Etv3 | 0.12679 | 0.59019304 |
| Atp13a3 | 0.80892 | -0.07698861 |
| Sema6d | 0.00144 | -1.28116048 |
| Myh9 | 0.20869 | -0.32738891 |
| Sgms1 | 0.1353 | 0.63415765 |
| Sgms1 | 0.19043 | 0.33307708 |
| Sgms1 | 0.48363 | 0.24245552 |
| Usp34 | 0.01241 | -1.02801808 |
| Sh3pxd2b | 0.00537 | 1.79474795 |
| Slc9a8 | 0.01818 | 1.00022919 |
| Rab3il1 | 0.02399 | -0.68076022 |
| Fxyd3 | 0.00144 | -1.45641127 |
| Diablo | 0.0352 | -0.94397117 |
| Fam110b | 0.68322 | -0.50457871 |
| Gyk | 0.81927 | -0.10029326 |
| Tmsb10 | 0.13037 | -0.62965837 |
| Camta1 | 0.00718 | 1.51145524 |
| Camta1 | 0.16586 | -0.74640568 |
| Gm38424 | 0.00152 | -1.25878908 |
| Kdm5b | 0.27829 | -0.39064043 |
| Kdm5b | 0.19051 | 0.52120122 |
| Cwc22 | 0.48056 | -0.34406014 |
| Casc3 | 0.6381 | -0.16180894 |
| Zfp275 | 0.00562 | -1.15214925 |
| Gmeb1 | 0.31432 | -0.49005968 |
| Arhgef2 | 0.00394 | -1.52545535 |
| Arhgef2 | 0.00532 | -1.2732049 |
| Arpc3 | 0.08643 | -2.70448602 |
| Snph | 0.00549 | -1.16183131 |
| Zrsr2 | 0.002 | 2.54550192 |
| Rbm25 | 0.00368 | 1.88927401 |
| Upb1 | 0.01809 | -0.93710794 |
| Birc6 | 0.69798 | -0.31326879 |
| Birc6 | 0.01843 | 0.7943757 |
| Aqr | 0.00827 | 1.66163104 |
| Ccdc93 | 0.26381 | 0.46711814 |
| Gtf2i | 0.0249 | -1.56837939 |
| Ap2m1 | 0.02857 | -1.12763957 |
| Tdrp | 0.00332 | -1.18526304 |
| Taf6 | 0.85401 | -0.06219525 |
| C4bp | 0.00144 | -1.25118919 |
| Camk1 | 0.87619 | -0.05880324 |
| Cdc14a | 0.27704 | -0.34937398 |
| Tnik | 0.2041 | 0.5493741 |
| Slc15a4 | 0.01668 | -0.61962199 |
| Snap47 | 0.00318 | -1.16999514 |
| Kctd18 | 0.77759 | -0.17667042 |
| Kctd18 | 0.73135 | 0.24488155 |
| Kctd18 | 0.03466 | 0.59155279 |
| Ccnl1 | 0.83267 | -0.10630201 |
| Zfp952 | 0.14998 | 0.41144839 |
| Slfn5 | 0.20561 | -1.09115896 |
| Upp2 | 0.06198 | -1.26934956 |
| Meis2 | 0.0047 | -1.17385111 |
| Fbxw17 | 0.74398 | 0.07294487 |
| Ankrd54 | 0.01052 | -1.06539119 |
| Actr3 | 0.23363 | 0.56730622 |
| Sncaip | 0.29552 | -0.30603061 |
| Maged1 | 0.53189 | 0.41873129 |
| Dlg1 | 0.0136 | 1.64544737 |
| Rbm39 | 0.0153 | 1.36458758 |
| Sppl2a | 0.11962 | 0.62904491 |
| Msn | 0.02579 | 0.71419697 |
| Irf6 | 0.6156 | -0.14593627 |
| Cab39 | 0.18228 | 0.43142444 |
| Pramef8 | 0.04259 | -1.00388764 |
| Chd6 | 0.00144 | 2.82811026 |
| Chd6 | 0.77492 | -0.11383615 |
| Cdh23 | 0.15573 | -0.78881293 |
| Nckap5 | 0.0766 | 1.32823397 |
| Eya1 | 0.10415 | 0.88672663 |
| Abhd16a | 0.0336 | -0.67627593 |
| Cela2a | 0.00144 | -1.30713006 |
| Tenm2 | 0.98062 | 0.02530004 |
| Prdm15 | 0.10193 | -0.51699686 |
| Stxbp5l | 0.00144 | -1.26911831 |
| Notch1 | 0.34675 | -0.28284868 |
| Uchl1 | 0.26813 | -0.56105941 |
| Cry2 | 0.26714 | -0.3215202 |
| 2810417H13Rik | 0.21177 | -0.72116624 |
| Pank3 | 0.0057 | 0.79515977 |
| Pak7 | 0.00144 | -1.29864962 |
| Zfp30 | 0.98915 | -0.00291564 |
| Tanc2 | 0.00237 | 1.30504853 |
| Tanc2 | 0.18219 | -0.2336695 |
| Tatdn3 | 0.98786 | 0.00534737 |
| Bcl7a | 0.82068 | -0.07250221 |
| Rgl1 | 0.32124 | -0.34325329 |
| U2surp | 0.16198 | -0.41662033 |
| U2surp | 0.27354 | 0.38651283 |
| U2surp | 0.03456 | 1.0656286 |
| H13 | 0.00144 | -1.32313902 |
| H13 | 0.00144 | -1.1370732 |
| Slc4a8 | 0.11089 | -1.71644689 |
| Ocrl | 0.38408 | 0.92802446 |
| BC055324 | 0.00144 | -1.28116048 |
| Psmd11 | 0.00144 | -1.28116048 |
| Rgl2 | 0.00144 | -1.25118919 |
| Thrap3 | 0.15351 | 0.67032776 |
| Snx14 | 0.21982 | 0.67978335 |
| Pigu | 0.13396 | 0.51379151 |
| Brd8 | 0.35564 | -0.31558559 |
| Exoc5 | 0.28259 | 0.42512085 |
| Anxa2 | 0.85518 | 0.09350195 |
| Skap1 | 0.00145 | -1.2388121 |
| Pde4b | 0.8231 | 0.04432984 |
| Ank1 | 0.82267 | -0.10879591 |
| Sorbs2 | 0.01635 | 1.3854996 |
| Sorbs2 | 0.1073 | 0.9150908 |
| Aldh4a1 | 0.05299 | -0.55455662 |
| Adnp | 0.23135 | 0.2430407 |
| Tm9sf4 | 0.00668 | -1.67737018 |
| Dnajc13 | 0.04908 | -3.6512356 |
| Tm4sf20 | 0.01104 | -0.87394329 |
| Kalrn | 0.19483 | 0.8060267 |
| Kalrn | 0.46462 | -0.22559412 |
| Ggnbp1 | 0.09687 | -0.59525303 |
| Mrps18a | 0.00672 | -1.04143731 |
| Zic4 | 0.00145 | -1.33072139 |
| Sun1 | 0.03454 | -0.62246197 |
| Elovl1 | 0.96598 | -0.03112782 |
| Hoxb3 | 0.90007 | 0.0576151 |
| C1qc | 0.02074 | -1.35176081 |
| Nsmf | 0.12612 | -0.58394443 |
| Wdpcp | 0.01219 | 0.82187765 |
| Ppp2r3a | 0.02141 | 1.20470615 |
| Sec14l1 | 0.00528 | -0.84433123 |
| Pex13 | 0.02696 | -0.71810938 |
| Kcnq3 | 0.00144 | -1.28116048 |
| Fkbp14 | 0.10267 | 0.55452411 |
| Lztfl1 | 0.00852 | 1.55541338 |
| Zhx3 | 0.37464 | -0.5325526 |
| Zfp672 | 0.56775 | 0.20197551 |
| Slc25a42 | 0.07857 | -0.79748499 |
| Epb4.1l1 | 0.00144 | -1.26560808 |
| Tsn | 0.02168 | -0.72017613 |
| Col13a1 | 0.2844 | -0.62643595 |
| Fbxw21 | 0.00517 | -1.21266062 |
| Ltbp1 | 0.59244 | 0.49713966 |
| 1200014J11Rik | 0.26253 | 0.40797708 |
| 1200014J11Rik | 0.14227 | 0.67470549 |
| Dtymk | 0.01007 | -0.67264204 |
| Kat6b | 0.22964 | 0.66644642 |
| Kat6b | 0.10697 | 0.43157391 |
| Btbd10 | 0.09892 | 0.96980093 |
| Ints2 | 0.25268 | -0.72077103 |
| Anapc1 | 0.3011 | -0.34153872 |
| Rpl26 | 0.80225 | -0.06208471 |
| Aox4 | 0.00144 | -1.28116048 |
| Metap2 | 0.27198 | 0.42490525 |
| Large | 0.04821 | 0.49042113 |
| Atic | 0.09613 | -0.39272677 |
| Atic | 0.00461 | -1.69544362 |
| Sf3b1 | 0.59027 | -0.46827461 |
| Rad51 | 0.13176 | -0.84345584 |
| Stx18 | 0.91196 | 0.05550089 |
| Npepps | 0.79746 | 0.2083612 |
| Plscr2 | 0.01657 | 1.70500235 |
| Ssbp1 | 0.00213 | -1.61264775 |
| Ank3 | 0.38092 | 0.25799878 |
| Ppip5k2 | 0.2232 | 0.65542455 |
| Ppip5k2 | 0.17671 | 0.75778715 |
| Pard3 | 0.32531 | -0.29840923 |
| Fndc3a | 0.03174 | 0.74620178 |
| Senp6 | 0.22282 | 0.48317479 |
| Ccdc47 | 0.95087 | -0.02909184 |
| Rabl3 | 0.22283 | -0.30289976 |
| Pick1 | 0.19028 | 0.4400511 |
| Osmr | 0.16543 | 0.42028031 |
| Wdr72 | 0.68453 | -0.10573006 |
| Grb14 | 0.2631 | -0.28065454 |
| Grb14 | 0.63875 | 0.32378044 |
| Slco1a6 | 0.00144 | -1.28116048 |
| Casp2 | 0.5542 | -0.26650446 |
| Dusp15 | 0.00144 | -1.20555712 |
| Zfp280d | 0.0663 | 1.12194966 |
| Zfp280d | 0.22566 | -0.77803917 |
| Zfp280d | 0.4157 | -1.04911039 |
| Zfp280d | 0.3078 | 0.48711077 |
| Frs3 | 0.00145 | -1.2388121 |
| Cux1 | 0.8323 | -0.08664004 |
| Atf1 | 0.03443 | -0.55006035 |
| Parp12 | 0.00909 | 1.16912203 |
| Gpatch8 | 0.10209 | 1.43487777 |
| Hmcn1 | 0.38117 | -0.49856351 |
| Vmn1r114 | 0.00158 | -1.2446827 |
| Asph | 0.70889 | 0.13630542 |
| Gpr45 | 0.00144 | -1.30017714 |
| Ggnbp2 | 0.9679 | 0.0132534 |
| Ahcyl2 | 0.79914 | -0.10129127 |
| Rin3 | 0.00144 | -1.28898079 |
| Rnpepl1 | 0.01504 | -0.89248191 |
| Plcd4 | 0.00754 | -1.65445252 |
| Macf1 | 0.69697 | -0.21763159 |
| Ralgapb | 0.0044 | -1.00416233 |
| Iqcb1 | 0.10461 | 1.31766289 |
| Cspp1 | 0.89647 | -0.07197237 |
| Cspp1 | 0.25279 | 0.66556584 |
| Cspp1 | 0.00144 | -1.14561076 |
| Gcc2 | 0.01095 | 2.09545948 |
| Iqca | 0.00566 | -1.16464089 |
| Slx4ip | 0.15559 | -0.6483469 |
| Mfsd4 | 0.00144 | -1.27730451 |
| Amz1 | 0.00813 | -1.37594701 |
| Tgif2 | 0.92351 | 0.03290623 |
| Nmnat3 | 0.01302 | 1.72689641 |
| Nmnat3 | 0.28051 | 1.29673011 |
| Ryk | 0.00762 | -1.14169705 |
| Stmn1 | 0.02576 | -1.8941726 |
| Gcnt1 | 0.27946 | -0.47345005 |
| Entpd7 | 0.83552 | 0.05536776 |
| Xrcc2 | 0.27645 | -0.66713473 |
| Eif3i | 0.0534 | -0.59378657 |
| Pld5 | 0.0211 | -1.75245203 |
| Mtch2 | 0.69329 | -0.13391664 |
| Mtch2 | 0.4753 | -0.19041354 |
| Senp1 | 0.00725 | 1.05987493 |
| Papola | 0.93976 | -0.02748739 |
| Ntng2 | 0.22702 | 0.31006644 |
| Ntng2 | 0.06116 | -0.90286462 |
| Unc79 | 0.00145 | -1.2388121 |
| 2310022A10Rik | 0.01687 | -1.09932273 |
| Arfgef1 | 0.00744 | 1.52175267 |
| Uba5 | 0.32265 | 0.4127276 |
| Dennd2a | 0.98264 | 0.00804458 |
| Nucks1 | 0.02244 | 1.69663505 |
| Thsd4 | 0.00315 | -0.92578141 |
| Srp72 | 0.79112 | -0.10102302 |
| Myo1b | 0.17594 | 0.30982623 |
| Ccndbp1 | 0.10416 | -0.65209992 |
| Klhl12 | 0.01994 | 1.02575346 |
| Klhl12 | 0.10245 | 0.95424632 |
| Sgk3 | 0.00144 | -1.27165604 |
| Sgk3 | 0.00144 | -1.25118919 |
| Sgk3 | 0.00447 | -0.86914837 |
| Hat1 | 0.00184 | 1.52724302 |
| Ola1 | 0.00144 | -1.27992547 |
| Csnk2a1 | 0.59141 | -0.18006949 |
| Arhgef1 | 0.27045 | -0.34915459 |
| Arhgef1 | 0.10588 | 0.55758088 |
| Spock3 | 0.00158 | -1.2446827 |
| Spock3 | 0.02891 | 1.28658228 |
| Smarce1 | 0.03853 | 1.43153288 |
| Foxp1 | 0.0287 | 0.82573544 |
| Foxp1 | 0.08868 | 0.66985321 |
| Baz1b | 0.94736 | -0.02901891 |
| Cnga1 | 0.00264 | -0.96230693 |
| Cnga1 | 0.00144 | -1.31495282 |
| Ccnt1 | 0.55374 | 0.20309692 |
| Slc35a5 | 0.15282 | 0.28837398 |
| Rbms3 | 0.00145 | -1.2388121 |
| Srsf11 | 0.5091 | 0.25871652 |
| Ap2b1 | 0.00216 | -1.10905491 |
| Usp16 | 0.97836 | -0.00866457 |
| Slc22a15 | 0.92344 | 0.04145272 |
| Slc22a15 | 0.00487 | 3.28068747 |
| Scfd2 | 0.22963 | -0.3443344 |
| Cnst | 0.18944 | -0.63932226 |
| Inadl | 0.85205 | 0.11466563 |
| Strada | 0.00144 | 3.06385538 |
| Abr | 0.0589 | -0.47721087 |
| Robo2 | 0.21712 | -0.50396635 |
| Robo2 | 0.05779 | -0.97801886 |
| Exph5 | 0.00144 | -1.27699683 |
| Unc80 | 0.02772 | -0.98161948 |
| Mms19 | 0.00149 | -1.22582153 |
| Letm1 | 0.04664 | -0.66666667 |
| Thsd7b | 0.00144 | -1.29085005 |
| Thsd7b | 0.88172 | 0.03040329 |
| Myo6 | 0.00941 | 1.50426794 |
| Csdc2 | 0.61326 | -0.10717886 |
| Wdr45b | 0.24629 | 0.40709542 |
| Ssh2 | 0.02459 | 1.30294366 |
| Pid1 | 0.13416 | -0.46892294 |
| Lppr1 | 0.00144 | -1.29085005 |
| Anapc4 | 0.00144 | -1.31884045 |
| Raly | 0.00199 | 0.91734604 |
| Ep300 | 0.83214 | 0.09789142 |
| Cdv3 | 0.48801 | -0.16827366 |
| Spats2l | 0.17594 | -0.69523108 |
| Inpp5k | 0.1551 | -0.87154284 |
| Kbtbd12 | 0.19935 | -0.38610377 |
| Itpr2 | 0.00144 | -2.96747813 |
| Mtg1 | 0.00296 | -1.24082201 |
| Ccdc68 | 0.01693 | -0.96347022 |
| Efcab6 | 0.38115 | -0.49036485 |
| Yap1 | 0.93703 | -0.02280432 |
| Isca1 | 0.77676 | 0.10820633 |
| Olfr138 | 0.21669 | -0.72814152 |
| Zmynd8 | 0.34968 | -0.32273408 |
| Wdr44 | 0.10415 | 0.49141608 |
| Wdr44 | 0.29183 | 0.60025625 |
| Pip5k1a | 0.22441 | -0.72256761 |
| Poglut1 | 0.19958 | -0.46410581 |
| Poglut1 | 0.92249 | -0.07341472 |
| Ybx1 | 0.15448 | 0.25024143 |
| Ybx1 | 0.28617 | 0.16595754 |
| Snrpa1 | 0.00144 | -1.20555712 |
| Dst | 0.18334 | 0.72206868 |
| Dst | 0.55453 | -0.28304375 |
| Ing4 | 0.07938 | -1.02605401 |
| Cd200 | 0.00144 | -1.26560808 |
| Lrp8 | 0.53436 | -0.16716038 |
| Copb2 | 0.98391 | 0.01284713 |
| Aen | 0.08756 | 0.56437661 |
| R3hdm1 | 0.74916 | -0.12083445 |
| R3hdm1 | 0.05024 | 1.47979284 |
| Tbxas1 | 0.00145 | -1.2388121 |
| Gls | 0.02312 | 0.97216195 |
| Gls | 0.61912 | 0.20339018 |
| Asnsd1 | 0.00144 | -1.30017714 |
| Slc4a10 | 0.00144 | -1.28797948 |
| Acad11 | 0.26637 | 0.76041244 |
| Acad11 | 0.27092 | -0.54444184 |
| Zfr | 0.05357 | 0.80532878 |
| Celf2 | 0.0429 | -2.04483989 |
| Celf2 | 0.50884 | 0.31938986 |
| Celf2 | 0.01448 | 1.37363646 |
| Celf2 | 0.07344 | 0.90240608 |
| Gtf2e1 | 0.56542 | 0.29531303 |
| Lamc2 | 0.00144 | -1.21993258 |
| Iqch | 0.04055 | -0.99350051 |
| Epb4.1l5 | 0.67413 | 0.22766017 |
| B9d1 | 0.00271 | -1.12385295 |
| Glt8d2 | 0.10366 | 0.77329796 |
| Hdac4 | 0.49441 | -0.17082124 |
| Angptl3 | 0.00144 | -1.24811894 |
| Vps39 | 0.45838 | -0.16974456 |
| Bcas3 | 0.00144 | -1.29051721 |
| Hadhb | 0.45125 | 0.30476861 |
| Pi4k2b | 0.00144 | -1.30713006 |
| Zdhhc19 | 0.00144 | -1.26560808 |
| Galk2 | 0.8046 | 0.3878156 |
| Syne2 | 0.02265 | 1.02586611 |
| Atg16l1 | 0.00777 | 1.22712551 |
| Ifnar1 | 0.15784 | 0.48843428 |
| Akr1b10 | 0.8594 | -0.1249293 |
| Bcorl1 | 0.10039 | 0.91304675 |
| Nt5e | 0.03689 | 1.1624875 |
| AA415398 | 0.16459 | 0.55967028 |
| Cux2 | 0.09075 | -0.49078062 |
| Carf | 0.00145 | 1.59344453 |
| Carf | 0.27501 | -0.55399617 |
| Carf | 0.19079 | 0.6198742 |
| 2010111I01Rik | 0.0142 | 0.76589812 |
| Fstl1 | 0.89166 | -0.03985238 |
| Phyhipl | 0.2978 | -0.48505915 |
| Phyhipl | 0.7978 | -0.16660389 |
| Nhsl2 | 0.0037 | -1.79951646 |
| Nrxn1 | 0.00149 | -1.22582153 |
| R3hcc1l | 0.48185 | -0.28179365 |
| Marcksl1 | 0.00265 | -1.00437006 |
| Snx11 | 0.08831 | 0.43442513 |
| Fam45a | 0.10094 | -2.48775253 |
| Kpna1 | 0.02925 | 1.27716259 |
| Kpna1 | 0.68045 | -0.4070617 |
| Gse1 | 0.07696 | -0.54176347 |
| Lsg1 | 0.12683 | -0.83017772 |
| Tbc1d13 | 0.01544 | -1.01925391 |
| Ptgs1 | 0.12274 | 0.84951996 |
| Ankrd44 | 0.04578 | 1.24953423 |
| Ankrd44 | 0.28652 | 0.37445168 |
| Llgl1 | 0.00144 | -1.28116048 |
| 2610020H08Rik | 0.00145 | -1.24191372 |
| Serpine2 | 0.81167 | -0.13255287 |
| Nfe2l2 | 0.01252 | 0.90600402 |
| Tulp1 | 0.00144 | -1.29085005 |
| Cntd1 | 0.00144 | -1.27992547 |
| Triobp | 0.20056 | -0.53566294 |
| Fbxl20 | 0.04409 | 0.84288834 |
| Mbd5 | 0.06628 | -0.88249632 |
| Uggt1 | 0.00144 | -1.20555712 |
| Rsu1 | 0.41469 | 0.33306191 |
| Psmf1 | 0.00144 | -1.35320495 |
| Anapc11 | 0.26897 | 0.33451808 |
| Fgf10 | 0.08212 | -0.72937653 |
| Atp2c1 | 0.37275 | 0.52933372 |
| Nfasc | 0.00144 | -1.26911831 |
| Taf15 | 0.45725 | -0.13188304 |
| Nefm | 0.00144 | -1.26560808 |
| Crot | 0.17415 | 1.0145605 |
| Ndrg4 | 0.00338 | 1.25905731 |
| Ndrg4 | 0.00232 | -1.15063496 |
| Dnmt3l | 0.04174 | 0.59096604 |
| Col5a2 | 0.73991 | 0.14645187 |
| Reps2 | 0.00144 | -1.28116048 |
| Psmd1 | 0.38519 | -0.3331797 |
| Apbb2 | 0.00158 | 1.92476638 |
| Osbpl2 | 0.55619 | -0.16664258 |
| Tmbim6 | 0.05216 | -0.8043637 |
| Gramd1b | 0.15906 | -0.3784104 |
| Gm10046 | 0.91484 | -0.03572954 |
| Polg | 0.20186 | -0.47902127 |
| Cdh2 | 0.14971 | -0.7373803 |
| Sptan1 | 0.03943 | 1.05783104 |
| Pwwp2a | 0.75098 | -0.21823928 |
| Raet1a | 0.34586 | -0.26672036 |
| Gfpt2 | 0.0053 | 1.04052066 |
| Dpy19l3 | 0.01577 | -0.6675049 |
| Fastkd1 | 0.86652 | -0.07215638 |
| Sdk2 | 0.344 | -0.59462733 |
| Il1b | 0.00929 | -1.13503714 |
| S100pbp | 0.91942 | 0.03492664 |
| Kdsr | 0.00144 | -1.28898079 |
| Ddx3y | 0.00144 | -1.3348153 |
| Primpol | 0.4146 | 0.29282615 |
| Arx | 0.15554 | -0.71434372 |
| Fkbp15 | 0.00362 | 1.71218396 |
| Gigyf2 | 0.02891 | 0.61735331 |
| Clmp | 0.16577 | 0.51682774 |
| Gin1 | 0.01009 | 1.9868061 |
| Gin1 | 0.70619 | 0.09251292 |
| Pamr1 | 0.00144 | -1.31884045 |
| Plod2 | 0.5276 | 0.2121788 |
| Vezt | 0.88115 | -0.07735832 |
| Tmem167 | 0.00232 | -1.15063496 |
| Lima1 | 0.00653 | 1.54886054 |
| Coro2b | 0.13599 | -0.9704248 |
| Gtf2h3 | 0.69556 | 0.19127398 |
| Rassf1 | 0.00349 | 1.05557783 |
| Dsn1 | 0.00145 | -1.2388121 |
| Icam2 | 0.00988 | -0.97886361 |
| Tma16 | 0.11693 | 0.63215524 |
| Suv420h1 | 0.01648 | 1.22769171 |
| Kat7 | 0.67394 | -0.10369213 |
| Ctsh | 0.00145 | -1.2388121 |
| Ctsh | 0.3692 | 0.44269451 |
| Dlg4 | 0.00409 | -1.08010478 |
| Il1f6 | 0.00144 | -1.25118919 |
| Ntmt1 | 0.2089 | -0.60905447 |
| Slc45a4 | 0.00445 | -1.14726092 |
| Syt2 | 0.10308 | -0.6025487 |
| Rheb | 0.99267 | -0.00320944 |
| Arhgap12 | 0.01912 | 1.85746068 |
| Actl6a | 0.7552 | 0.1016571 |
| Acox3 | 0.4657 | -0.23458391 |
| Trpc1 | 0.21203 | 0.64877237 |
| Fip1l1 | 0.00218 | 1.47144078 |
| Anks6 | 0.00144 | -1.20030135 |
| Focad | 0.00359 | -1.44768414 |
| Sirpa | 0.01626 | -1.51933182 |
| Akap9 | 0.00144 | 1.80653726 |
| Nisch | 0.04992 | -0.61727052 |
| Nisch | 0.0025 | 1.25065667 |
| Nisch | 0.02642 | -1.35286667 |
| Nox4 | 0.00323 | -1.50848716 |
| Themis | 0.00145 | -1.22193674 |
| Trappc13 | 0.00251 | 1.93786061 |
| Klhl20 | 0.02999 | 0.86857369 |
| Bid | 0.00145 | -1.26219879 |
| Copa | 0.0114 | 1.22517336 |
| Tsc22d3 | 0.01084 | -1.07413081 |
| Caprin2 | 0.14744 | -1.45721251 |
| Tmc7 | 0.56194 | -0.09376197 |
| Zfp385b | 0.00144 | -1.22925777 |
| Commd6 | 0.0181 | -1.29281267 |
| Rfx7 | 0.2221 | -0.25997585 |
| Rfx7 | 0.0589 | 0.79211812 |
| 2410141K09Rik | 0.00144 | -1.29051721 |
| E130114P18Rik | 0.00144 | -1.20555712 |
| Mtap | 0.59365 | -0.40129171 |
| 2210416O15Rik | 0.00336 | -0.79856271 |
| Nat9 | 0.57555 | -0.25888837 |
| Ints8 | 0.45152 | -0.33847883 |
| Aldh3a2 | 0.06189 | 0.57433152 |
| Zfhx2 | 0.06366 | -0.82297957 |
| Pomt1 | 0.00144 | -1.31518407 |
| Sympk | 0.06728 | -2.3365038 |
| Ahr | 0.13638 | -0.72937653 |
| Rrp1b | 0.00592 | -1.34179867 |
| Ptch2 | 0.02923 | -1.20665264 |
| Zfp940 | 0.00472 | -1.13105119 |
| Eng | 0.88747 | 0.05032957 |
| Usp40 | 0.00144 | 3.11252968 |
| Cracr2b | 0.00869 | -1.07182977 |
| Cracr2b | 0.01995 | -0.48518709 |
| Dmd | 0.15187 | 1.17842842 |
| Dmd | 0.00767 | 1.45014652 |
| Fchsd2 | 0.95539 | 0.03292788 |
| Fchsd2 | 0.16648 | 0.68727189 |
| Pdzrn3 | 0.07717 | -1.4152974 |
| Kif24 | 0.54498 | 0.21078073 |
| Ccdc77 | 0.46615 | -0.1715261 |
| Ccdc77 | 0.91726 | 0.05289125 |
| Suclg1 | 0.14602 | 0.45377865 |
| 1700021F05Rik | 0.7816 | -0.05993836 |
| Grid2 | 0.02279 | -1.78343614 |
| Setd1b | 0.91032 | 0.03162695 |
| Irgm1 | 0.04698 | -0.91567309 |
| Pla2g4a | 0.11057 | -0.96029465 |
| Cpne7 | 0.54054 | 0.21972639 |
| Abcc1 | 0.01613 | 1.00984837 |
| Aspa | 0.08701 | 0.69805443 |
| Poldip3 | 0.00144 | -1.29864962 |
| Ccdc59 | 0.14836 | -0.85716217 |
| Ppip5k1 | 0.21822 | 0.79077178 |
| Cdc73 | 0.00502 | 2.93314326 |
| Ophn1 | 0.25414 | -0.41294522 |
| Mtmr10 | 0.08305 | 0.70278797 |
| Pdxdc1 | 0.78135 | 0.15835544 |
| Ticam2 | 0.15155 | -0.63914042 |
| Kxd1 | 0.03736 | -0.74851228 |
| Ercc5 | 0.92293 | -0.04366944 |
| Impg2 | 0.00144 | -1.30017714 |
| Adam33 | 0.00144 | -1.28898079 |
| Hdgfrp3 | 0.8405 | 0.09350221 |
| Zfp637 | 0.06136 | 0.3964926 |
| Entpd5 | 0.00785 | 1.00532455 |
| Prpf40a | 0.50065 | 0.25182195 |
| Pigq | 0.66966 | 0.2581429 |
| Spink11 | 0.00149 | -1.22582153 |
| Rnpc3 | 0.00591 | 2.08948848 |
| Arhgef6 | 0.64464 | -0.15715257 |
| Smim14 | 0.04169 | 1.58606407 |
| St7l | 0.00727 | 1.74610907 |
| Ccdc28b | 0.02488 | -1.04214879 |
| Prkg1 | 0.00796 | 0.81087764 |
| Prkg1 | 0.00784 | -1.03698725 |
| Ftsj1 | 0.29346 | -0.37040288 |
| Dync1i2 | 0.00144 | 2.23422174 |
| Abhd5 | 0.34201 | 0.28404619 |
| Nat10 | 0.00144 | -1.29085005 |
| Cenph | 0.0189 | -1.389975 |
| Nfat5 | 0.14852 | 0.87666308 |
| Nfat5 | 0.02446 | 1.40085641 |
| Pax6 | 0.00144 | -1.26301121 |
| Pax6 | 0.00144 | -1.28116048 |
| Wt1 | 0.95278 | 0.01614276 |
| Zfr2 | 0.01234 | -1.10835412 |
| Pdlim4 | 0.00342 | -1.39332164 |
| Dnal4 | 0.00144 | -1.20579466 |
| Xpnpep1 | 0.00144 | -1.30713006 |
| Scn1a | 0.62575 | 0.20397376 |
| Ppil3 | 0.25053 | -0.81866976 |
| Crlf3 | 0.23018 | 0.47916331 |
| Camk2d | 0.95209 | -0.0202765 |
| Mrto4 | 0.11017 | -0.83729968 |
| Bnc2 | 0.01263 | -2.09914451 |
| Aldh16a1 | 0.01936 | -1.44822234 |
| Tango2 | 0.06929 | 0.92895715 |
| Npl | 0.31765 | -0.66206473 |
| Fam69c | 0.00144 | -1.28116048 |
| Ndst4 | 0.00144 | -1.28116048 |
| Col6a3 | 0.48188 | 0.31266033 |
| E2f8 | 0.01241 | -1.02801808 |
| Zbtb38 | 0.15419 | -0.71519227 |
| Zbtb38 | 0.87154 | 0.05620448 |
| Zbtb38 | 0.22242 | -0.41642916 |
| Zbtb38 | 0.74228 | -0.60247646 |
| Polr3f | 0.09249 | -0.43734266 |
| Ralb | 0.32883 | 0.32205525 |
| Pla2g6 | 0.01504 | -1.35870099 |
| Obfc1 | 0.02548 | -0.72862218 |
| Satb1 | 0.03467 | -1.68049221 |
| Satb1 | 0.40237 | 0.3635552 |
| Zfp2 | 0.00549 | 1.96258565 |
| Zfp2 | 0.23176 | 0.2761536 |
| Pms2 | 0.02298 | -1.79260595 |
| Psma3 | 0.36256 | 0.42013008 |
| Car5b | 0.07478 | -0.42353059 |
| Gcfc2 | 0.86652 | 0.08033603 |
| Arhgap6 | 0.00144 | 1.33605798 |
| C2cd3 | 0.00144 | -1.26560808 |
| Pik3c2g | 0.00144 | -1.34813137 |
| Cse1l | 0.62325 | 0.28180765 |
| Atr | 0.19428 | -0.63370825 |
| Atr | 0.00969 | 2.10823243 |
| Atr | 0.09897 | 0.52109095 |
| Atr | 0.00214 | 1.83875343 |
| Tia1 | 0.04083 | 0.82680495 |
| Tia1 | 0.29918 | 0.37144074 |
| Slco1b2 | 0.00144 | -1.28898079 |
| Slc25a23 | 0.88191 | -0.07058795 |
| Dock10 | 0.01917 | -0.94688061 |
| Dock10 | 0.42157 | 0.31536959 |
| Wscd2 | 0.14097 | -0.96478942 |
| Gabarapl2 | 0.00144 | -1.28116048 |
| Dnmt3a | 0.02698 | -0.7435934 |
| Dnmt3a | 0.09181 | -2.56098047 |
| Cdh10 | 0.00144 | -1.30713006 |
| Zfp182 | 0.00753 | 1.47882522 |
| Rpn2 | 0.01793 | -0.7420198 |
| Ptger4 | 0.00144 | -1.28898079 |
| 4932438A13Rik | 0.01698 | 1.30137077 |
| 4932438A13Rik | 0.17608 | 0.7467437 |
| Arhgap20 | 0.01029 | 3.39587043 |
| Cacna1e | 0.00144 | -1.29051721 |
| Cacna1e | 0.01693 | -0.82474288 |
| Hkdc1 | 0.00144 | -1.24811894 |
| Ube2t | 0.76064 | -0.08921684 |
| Ube2t | 0.05769 | -0.69807559 |
| Yars | 0.39589 | 0.35392124 |
| Cib1 | 0.00144 | -1.34202077 |
| Dnah7b | 0.08156 | 0.9462793 |
| Dnah7b | 0.00144 | -1.29746368 |
| Ikzf2 | 0.04678 | 1.56019987 |
| Nsun3 | 0.00144 | -1.28116048 |
| Unc5c | 0.00144 | -1.30017714 |
| Ggta1 | 0.00144 | -1.28898079 |
| Me3 | 0.0055 | 1.51971051 |
| Me3 | 0.52001 | -0.3230041 |
| Fam46c | 0.01929 | -1.04906203 |
| B3gnt7 | 0.00144 | -1.31014844 |
| Plekha1 | 0.51714 | 0.3000014 |
| Plekha1 | 0.98112 | -0.00804074 |
| Gabbr1 | 0.11176 | -0.77860016 |
| Bcl6 | 0.62347 | -0.31745175 |
| Smn1 | 0.00177 | -1.26528643 |
| Ube2e2 | 0.03513 | 1.31006194 |
| Txndc12 | 0.0702 | -2.79984023 |
| Hk1 | 0.00351 | -0.75217696 |
| Ercc8 | 0.002 | 3.09750534 |
| Mbtd1 | 0.02337 | 1.32645934 |
| Gk5 | 0.01858 | 1.46386964 |
| Syt12 | 0.39706 | 0.24497594 |
| Ppp2r1b | 0.00144 | -1.30713006 |
| Tox | 0.16729 | -0.64784435 |
| Mtdh | 0.04024 | 1.39620535 |
| Dpp4 | 0.28574 | -0.43612346 |
| Trim7 | 0.0629 | 1.0298366 |
| Mapre2 | 0.86994 | 0.09472059 |
| Ssbp3 | 0.04701 | -0.99868409 |
| Rbm18 | 0.01608 | 0.99284274 |
| Sf3a3 | 0.00492 | -1.13895867 |
| Sf3a3 | 0.02918 | -0.70234128 |
| Atp8a2 | 0.02133 | -0.66788548 |
| Ppil4 | 0.75163 | 0.12525788 |
| Vwf | 0.06622 | -0.65122269 |
| Fgfr1op2 | 0.84324 | 0.06675876 |
| Top1 | 0.05278 | 1.11855298 |
| Top1 | 0.02299 | 1.30286692 |
| Rxrb | 0.35275 | -0.40312675 |
| Zscan20 | 0.9366 | -0.04499415 |
| Tmem109 | 0.17513 | -0.5947571 |
| Pprc1 | 0.00144 | -1.29085005 |
| Pprc1 | 0.00276 | -1.01942033 |
| Pbx3 | 0.02344 | 1.15610679 |
| Lancl1 | 0.77549 | 0.2122773 |
| Slc15a2 | 0.00144 | -1.31884045 |
| Stard13 | 0.79083 | 0.20478407 |
| Pms1 | 0.00144 | -1.29746368 |
| Pms1 | 0.30372 | -0.51025269 |
| Tm6sf1 | 0.38188 | 0.31968247 |
| Afap1 | 0.24634 | 0.63958585 |
| Neb | 0.0175 | 1.25179355 |
| Psat1 | 0.00149 | -1.22582153 |
| Gm12789 | 0.00239 | -2.13540167 |
| Tmem87b | 0.31234 | 0.64199981 |
| Pax1 | 0.00144 | -1.27165604 |
| Rngtt | 0.81269 | -0.15522119 |
| Odf2 | 0.31544 | 0.37545636 |
| Odf2 | 0.06866 | -0.60115082 |
| Slc39a13 | 0.5642 | 0.3622802 |
| Cep128 | 0.00144 | -1.27454685 |
| Vopp1 | 0.58729 | -0.71250117 |
| Rhox4g | 0.03942 | -0.43889284 |
| Ttc13 | 0.11855 | -1.17475831 |
| Ttc13 | 0.34023 | -0.56325666 |
| Map3k8 | 0.08991 | 0.83396892 |
| Rsrc2 | 0.0775 | 0.95402554 |
| Myo18a | 0.04649 | -0.7796569 |
| Trmt1l | 0.02045 | 1.62135615 |
| Pde11a | 0.08409 | -1.09941343 |
| Gemin4 | 0.04519 | -0.45615443 |
| Gtf2h5 | 0.00145 | 1.48434731 |
| Des | 0.05118 | -2.00503793 |
| Wipf1 | 0.08237 | -1.1786843 |
| Whsc1 | 0.02666 | 1.40170998 |
| Trpm2 | 0.04169 | -0.88587163 |
| Dcxr | 0.39726 | -0.3235845 |
| Prrg1 | 0.02299 | 0.6403702 |
| Prrg1 | 0.04004 | 1.68729224 |
| Prrg1 | 0.03591 | 1.46162332 |
| Syk | 0.72268 | -0.15298124 |
| Rad54l2 | 0.06617 | -0.45798626 |
| Ccpg1 | 0.02438 | 0.84507937 |
| Pcsk2 | 0.04321 | -0.9554005 |
| Rasa2 | 0.06409 | 1.04598662 |
| Adamtsl1 | 0.00236 | -1.60633928 |
| Cdk14 | 0.23967 | 0.60784037 |
| Clu | 0.03283 | -1.90898914 |
| 2410089E03Rik | 0.07045 | 1.43935065 |
| Taok3 | 0.40436 | -0.25863776 |
| Strn4 | 0.02836 | 0.99302966 |
| Luc7l2 | 0.40066 | 0.20106103 |
| Luc7l2 | 0.00913 | 1.57429342 |
| Luc7l2 | 0.02599 | 1.28994363 |
| Zeb2 | 0.01595 | 0.94958218 |
| Opcml | 0.36564 | -0.45042493 |
| Opcml | 0.00144 | -1.28116048 |
| Opcml | 0.01365 | -1.10854086 |
| Lama4 | 0.00319 | -1.03018352 |
| Fam118b | 0.64025 | -0.21036065 |
| Fam118b | 0.20531 | 0.71700396 |
| Kmt2b | 0.0077 | -1.01080716 |
| Ttll4 | 0.49968 | -0.38215497 |
| Pxylp1 | 0.09716 | -0.71115368 |
| Pxylp1 | 0.00144 | -1.39487581 |
| Hectd2 | 0.19861 | -1.15714015 |
| Kdm2b | 0.0076 | -0.84732169 |
| Tmbim4 | 0.77492 | 0.12431737 |
| Phip | 0.69837 | -0.19712959 |
| Phip | 0.00144 | 1.77005384 |
| Phip | 0.3306 | 0.54237215 |
| Phip | 0.01945 | -0.52223649 |
| Phip | 0.31106 | -0.62731807 |
| Rgs20 | 0.00144 | -1.31627578 |
| Clcn7 | 0.42334 | -0.46478939 |
| Tepp | 0.00576 | -1.4913104 |
| 2700081O15Rik | 0.46204 | 0.25973637 |
| Nkain2 | 0.03705 | -1.01204217 |
| Nt5c3 | 0.00149 | -1.25408 |
| Nmrk1 | 0.10909 | 0.81058707 |
| Nmrk1 | 0.01445 | 1.2626652 |
| Dusp19 | 0.58318 | -0.21219842 |
| Vti1b | 0.02979 | 0.58707244 |
| Mdn1 | 0.1546 | 0.95121619 |
| Poldip2 | 0.5563 | -0.19887906 |
| Brwd1 | 0.06241 | 0.89816826 |
| Tnfsf4 | 0.00144 | -1.28116048 |
| Tanc1 | 0.98802 | -0.00482608 |
| Tanc1 | 0.2683 | 0.40353366 |
| Tanc1 | 0.00144 | 2.02867841 |
| Elovl5 | 0.00144 | -1.29746368 |
| Dzip1l | 0.14695 | -0.70855554 |
| Rcc1 | 0.00153 | -1.24063982 |
| Rcc1 | 0.03898 | -1.65913198 |
| Slc38a6 | 0.76795 | -0.18916323 |
| Tom1l1 | 0.00144 | -1.33632959 |
| Tom1l1 | 0.13614 | 0.89140393 |
| Tlr8 | 0.91465 | 0.11574318 |
| Pyroxd1 | 0.00144 | -1.2383131 |
| Sdcbp | 0.00144 | 1.7525791 |
| Smchd1 | 0.70575 | 0.13094179 |
| Smchd1 | 0.36958 | 0.37640846 |
| Xiap | 0.0217 | 0.57062887 |
| Pm20d1 | 0.00446 | -1.07797289 |
| Ptprn2 | 0.00144 | -1.27730451 |
| Cyb5d2 | 0.60625 | 0.16612314 |
| Scaf1 | 0.00144 | -1.27730451 |
| Raph1 | 0.0348 | 1.14702297 |
| Zyg11b | 0.06883 | 0.75417795 |
| Matr3 | 0.00144 | 1.94828755 |
| Atg4b | 0.15634 | -0.300171 |
| Cnbd2 | 0.86654 | -0.12553088 |
| Ndrg3 | 0.03624 | -0.89248819 |
| Ndrg3 | 0.17279 | 0.95504643 |
| Api5 | 0.02857 | 0.47382699 |
| Zfp101 | 0.08172 | -0.44758288 |
| Adgrd1 | 0.96887 | 0.02089004 |
| Rasa1 | 0.12711 | -0.60921391 |
| Camk2a | 0.00158 | -1.2446827 |
| Eva1a | 0.13085 | -0.56825559 |
| Phc3 | 0.00145 | -1.22376446 |
| Slc6a4 | 0.01332 | -1.88467179 |
| Jade2 | 0.16997 | -0.46946307 |
| Rrbp1 | 0.02229 | 1.36129297 |
| Brsk1 | 0.159 | -0.82710748 |
| Ndufs1 | 0.79005 | -0.24221924 |
| Ttc23 | 0.88305 | 0.07522005 |
| Ager | 0.00149 | -1.09695489 |
| Ager | 0.27206 | -0.7146735 |
| Ager | 0.28913 | 0.37913072 |
| Rnaseh2b | 0.03732 | 1.21191273 |
| Snrnp40 | 0.0571 | -0.99091235 |
| Stx2 | 0.80941 | -0.08708108 |
| Slc16a3 | 0.00145 | -1.26219879 |
| Tmem143 | 0.12577 | 0.62293601 |
| Zfp420 | 0.00144 | -1.29049509 |
| Ncf2 | 0.02296 | -1.74131765 |
| Ncf2 | 0.06288 | 1.78160163 |
| Wdr12 | 0.00149 | -1.19696847 |
| Nkiras2 | 0.00145 | -1.26219879 |
| Abhd2 | 0.6313 | 0.15232856 |
| Syt1 | 0.00144 | -1.44302143 |
| Syt1 | 0.37342 | -0.28221331 |
| Ube2f | 0.00437 | -1.16395103 |
| Ube2f | 0.17872 | -0.82632814 |
| Ube2f | 0.00145 | -1.07386469 |
| Prdm5 | 0.00144 | 2.80427484 |
| Itgav | 0.41569 | -0.17313728 |
| Ets1 | 0.46037 | -0.22837605 |
| Esyt3 | 0.00218 | -1.18878227 |
| Slc13a1 | 0.03113 | -1.44322125 |
| Slc13a1 | 0.80117 | -0.11667966 |
| Zfp560 | 0.43845 | -0.17070668 |
| Itch | 0.16763 | 0.37474739 |
| Plce1 | 0.01147 | -1.5374277 |
| Pkib | 0.21201 | 0.29670207 |
| Pkib | 0.02203 | -0.972494 |
| Hivep3 | 0.22248 | 0.47814754 |
| Mob1b | 0.07633 | 1.324751 |
| Coq10b | 0.45027 | 0.44897116 |
| Coq10b | 0.0258 | -0.88843189 |
| Fyb | 0.09084 | -0.96127634 |
| Skint1 | 0.00144 | -1.31048128 |
| Hmga2 | 0.00144 | -1.27730451 |
| Tcf12 | 0.00144 | 1.76909343 |
| Exoc1 | 0.03782 | 0.96693424 |
| Ablim2 | 0.09041 | 0.96727365 |
| Gle1 | 0.05225 | -0.83182145 |
| Gle1 | 0.56925 | 0.19165082 |
| Tgif1 | 0.00144 | -1.26560808 |
| Ankfy1 | 0.00891 | 1.87749167 |
| Dennd1a | 0.11081 | 0.63935025 |
| Exosc8 | 0.02178 | -1.2371435 |
| Asl | 0.07589 | -0.58958905 |
| Slc23a2 | 0.55668 | -0.31310402 |
| Stt3a | 0.00144 | 2.36170704 |
| Stt3a | 0.08153 | 0.76151907 |
| Paip2b | 0.02649 | 1.70508725 |
| Tsacc | 0.02767 | 0.74641551 |
| Adamts9 | 0.00144 | 3.11279864 |
| Diap2 | 0.07116 | -0.86506346 |
| Psmc2 | 0.9124 | 0.04674471 |
| St7 | 0.70804 | -0.53068486 |
| Rb1cc1 | 0.69549 | -0.14685753 |
| Rb1cc1 | 0.81951 | -0.06156862 |
| 3010026O09Rik | 0.00775 | -0.85196929 |
| Dnm3 | 0.01201 | 1.01971916 |
| Lama2 | 0.0637 | 0.9663093 |
| Cript | 0.32764 | 0.40783462 |
| Lcp1 | 0.0028 | -1.16569658 |
| Gm5148 | 0.00144 | -1.28116048 |
| Zzef1 | 0.3092 | 0.302967 |
| Pcmt1 | 0.24071 | -0.56144363 |
| Nrip1 | 0.48279 | 0.27303405 |
| Nrip1 | 0.13386 | 0.61558807 |
| Nrip1 | 0.0386 | 1.15369694 |
| Mina | 0.09549 | 0.40248518 |
| Hnf1a | 0.00159 | -1.26431393 |
| Tyms | 0.01545 | 0.96162808 |
| Chmp1a | 0.14753 | -0.69198295 |
| Spred3 | 0.20331 | 0.39835666 |
| Pdha1 | 0.06302 | -0.97733423 |
| Hs6st2 | 0.63638 | 0.12287656 |
| Foxj3 | 0.32125 | 0.4182478 |
| Evi5 | 0.1461 | -0.57248205 |
| Evi5 | 0.00423 | -0.76327852 |
| Gsta3 | 0.69924 | -0.22471121 |
| Atf6 | 0.10869 | 1.31272288 |
| Eftud2 | 0.13812 | 0.49507472 |
| Eftud2 | 0.51632 | 0.43223846 |
| Eftud2 | 0.13733 | 0.68419049 |
| Wsb2 | 0.67463 | -0.10480401 |
| Saxo2 | 0.00144 | -1.27165604 |
| Xpr1 | 0.01893 | 1.19940287 |
| Btbd3 | 0.00378 | -1.45121232 |
| Btbd3 | 0.00144 | -1.31518407 |
| Nacc2 | 0.08495 | -1.04375872 |
| Zbtb49 | 0.00212 | 1.87221559 |
| Kcnq1ot1 | 0.00144 | 3.32959447 |
| Atp11a | 0.40581 | -0.18896622 |
| Sh2d3c | 0.54874 | -0.30561878 |
| Pls3 | 0.12316 | 1.12782217 |
| Wdsub1 | 0.00144 | -1.29085005 |
| Zwilch | 0.00144 | -1.29049509 |
| Slain2 | 0.07627 | -0.57630352 |
| Rab1 | 0.13596 | 0.81839829 |
| Slc25a21 | 0.13119 | -0.95960483 |
| Nhsl1 | 0.85543 | 0.1303868 |
| Ctage5 | 0.22145 | 0.70587507 |
| Micu1 | 0.03257 | 0.59016251 |
| Rgs12 | 0.00144 | -1.25927895 |
| Prickle3 | 0.76527 | 0.08659389 |
| Serpini1 | 0.02632 | 0.75502741 |
| Sgcd | 0.04671 | 0.99782987 |
| Lef1 | 0.00538 | -1.51499183 |
| Lef1 | 0.00144 | -1.30017714 |
| Kctd13 | 0.00404 | -0.82688427 |
| Parp9 | 0.49738 | 0.36791692 |
| Acss3 | 0.04632 | 0.74499512 |
| Gm8369 | 0.00144 | -1.26934956 |
| Pard3b | 0.29907 | 0.46986456 |
| Rbm44 | 0.00186 | -1.56742429 |
| Eif2b1 | 0.04443 | -0.86996962 |
| Arid2 | 0.14744 | -0.67384439 |
| Ptpn5 | 0.00889 | -0.98750843 |
| Adgrl2 | 0.17496 | -0.301184 |
| Col1a1 | 0.00919 | -1.01394006 |
| Nfrkb | 0.19068 | -0.37838653 |
| Col3a1 | 0.02443 | 1.0758763 |
| Col3a1 | 0.75011 | -0.20273229 |
| Pkd2 | 0.44575 | -0.1791372 |
| Plcb4 | 0.00164 | 1.09185426 |
| Plcb4 | 0.00461 | 1.14096171 |
| Arpp19 | 0.91357 | 0.03166581 |
| Rnf122 | 0.37885 | -0.33230907 |
| Nprl3 | 0.4084 | -0.40195741 |
| Rmnd1 | 0.15255 | 0.70444716 |
| Trim2 | 0.00383 | -1.78478319 |
| Nfyb | 0.02725 | -1.246356 |
| Tctn1 | 0.0799 | -0.82849857 |
| Ccbe1 | 0.07879 | -0.65123545 |
| Gja5 | 0.00147 | -1.26946363 |
| Pmm2 | 0.01291 | -0.6571052 |
| Ap4e1 | 0.02892 | 0.52400782 |
| A730017C20Rik | 0.08128 | -0.87431012 |
| Znhit3 | 0.00144 | -1.34171688 |
| Pknox1 | 0.9161 | 0.03640385 |
| Grb10 | 0.87243 | -0.05683721 |
| Imp4 | 0.00327 | -0.80309412 |
| Slc35a3 | 0.00144 | 2.08619355 |
| Phf3 | 0.11017 | 1.23781356 |
| Dnajb4 | 0.24346 | 0.48792541 |
| Nktr | 0.06396 | 0.56634159 |
| Nktr | 0.01376 | 1.34228027 |
| Nktr | 0.04437 | 0.68093528 |
| Als2 | 0.92156 | 0.04508955 |
| Cln8 | 0.53426 | -0.22787714 |
| Rgs22 | 0.0045 | -1.82775945 |
| Ndufa10 | 0.87383 | -0.07321548 |
| Invs | 0.42096 | 0.89800585 |
| Arid4b | 0.83267 | -0.04177332 |
| Gipc2 | 0.00145 | -1.27532353 |
| Kcnq5 | 0.00144 | 2.60449538 |
| Fbxw2 | 0.42575 | -0.20020456 |
| Gabarap | 0.69985 | 0.11904077 |
| Creb3l2 | 0.10581 | -0.68207576 |
| Pou2f1 | 0.00144 | -1.28116048 |
| Spen | 0.38519 | -0.44300842 |
| Spen | 0.87177 | -0.05909978 |
| Nebl | 0.0315 | 1.39999557 |
| Lce3d | 0.10243 | -0.7912911 |
| Ash1l | 0.14597 | 0.81295736 |
| Prdm10 | 0.12676 | 0.5017773 |
| Mkln1 | 0.80941 | -0.0695247 |
| Sft2d1 | 0.088 | -0.86488168 |
| Usp42 | 0.0886 | 0.41973045 |
| Pon1 | 0.00144 | -1.28116048 |
| Adgrv1 | 0.00702 | -1.09418462 |
| Ccdc148 | 0.00144 | -1.30713006 |
| Ccdc148 | 0.00256 | 1.01894703 |
| Arhgap26 | 0.03474 | -0.74817297 |
| Arhgap26 | 0.88771 | -0.10096571 |
| Fam20b | 0.00144 | 2.78966411 |
| Slco4a1 | 0.08636 | -0.81230944 |
| Bin2 | 0.00266 | -0.8515214 |
| Smg1 | 0.30475 | 0.44942165 |
| 1700013F07Rik | 0.10146 | -2.80099843 |
| Faxc | 0.00144 | -1.29051721 |
| Cd52 | 0.00144 | -1.24811894 |
| Vav2 | 0.01736 | -0.88478444 |
| Zranb3 | 0.46024 | -0.62457458 |
| Zranb3 | 0.00696 | -1.52481061 |
| P2ry6 | 0.00467 | -0.79794166 |
| Grin1 | 0.00716 | -1.31181992 |
| Bnip1 | 0.67622 | -0.16569944 |
| Bnip1 | 0.90415 | 0.0454495 |
| Psip1 | 0.90195 | 0.04035708 |
| Six1 | 0.13934 | -0.26103617 |
| Ccser2 | 0.04987 | -1.11683242 |
| Ptpn18 | 0.34764 | 0.65950087 |
| Ptpn18 | 0.0914 | -2.70284149 |
| Dennd5a | 0.08469 | -0.6554846 |
| Exoc6b | 0.69623 | 0.154031 |
| Mpp3 | 0.50327 | -0.36651189 |
| Mpp3 | 0.02203 | -0.972494 |
| Dbf4 | 0.08199 | 0.74710555 |
| Akr1cl | 0.94682 | -0.02215126 |
| Akr1cl | 0.02665 | -0.75337231 |
| Stxbp4 | 0.00964 | 1.68286011 |
| Brca1 | 0.00144 | -1.28898079 |
| Fads6 | 0.00145 | -1.18741414 |
| Mlip | 0.41443 | 0.23373449 |
| Tulp3 | 0.30798 | 0.27874967 |
| Tulp3 | 0.12771 | -0.39804386 |
| Aars | 0.02584 | -0.81069095 |
| Serac1 | 0.07505 | -0.81367219 |
| Evi5l | 0.60197 | -0.75881125 |
| Cnrip1 | 0.69947 | 0.19774331 |
| Rnf220 | 0.05878 | -0.74759787 |
| Asxl3 | 0.03947 | -0.96052972 |
| Esr1 | 0.38688 | -0.55262102 |
| Fundc2 | 0.04131 | -0.42236128 |
| Mup11 | 0.00144 | -1.29085005 |
| Nrg3 | 0.00306 | -1.07962412 |
| Laptm5 | 0.00381 | -1.4316816 |
| Tmem29 | 0.05407 | 0.54377813 |
| Myo5a | 0.22399 | -1.18245977 |
| Lgals8 | 0.13238 | -1.0422887 |
| Brinp3 | 0.0461 | -3.02821935 |
| Zfp169 | 0.00292 | 1.14808048 |
| Pik3r5 | 0.00144 | -1.30017714 |
| Nlrp4e | 0.00144 | -1.82299602 |
| Sulf1 | 0.01945 | -1.71448426 |
| Acss2 | 0.00898 | 1.72575471 |
| Pcsk6 | 0.00144 | -1.28116048 |
| Cers6 | 0.05728 | -1.04254443 |
| Ikzf3 | 0.75781 | -0.09010122 |
| Dars | 0.02149 | 1.45175099 |
| Fam69b | 0.01084 | -1.45780085 |
| Thra | 0.28713 | -0.72689081 |
| Ube2e1 | 0.02582 | 1.00223019 |
| Tnks2 | 0.018 | 1.49841911 |
| Tmem108 | 0.3986 | -0.3155616 |
| Limk1 | 0.51171 | 0.25333501 |
| Taok2 | 0.18431 | -0.36719004 |
| Nudt5 | 0.52756 | 0.31920932 |
| Edil3 | 0.01467 | -1.10331848 |
| Edil3 | 0.07116 | -0.86506346 |
| Asb13 | 0.24699 | -0.44087571 |
| Fndc1 | 0.03537 | -0.84157058 |
| Lamp2 | 0.90596 | 0.03912484 |
| Irf8 | 0.03044 | -0.81965107 |
| Usp5 | 0.00208 | -1.15498993 |
| Usp5 | 0.16278 | -0.31689052 |
| Tshz3 | 0.22832 | 0.59896887 |
| Cdc23 | 0.02787 | -0.67641368 |
| Mtfp1 | 0.285 | -0.31006613 |
| Zdhhc21 | 0.39672 | 0.3361019 |
| Stk17b | 0.00682 | 2.22226287 |
| Fam126a | 0.90533 | -0.05224166 |
| Lpin2 | 0.77822 | -0.11173793 |
| Sqle | 0.00144 | -1.26934956 |
| Pax8 | 0.00144 | -1.24811894 |
| Gm21992 | 0.00554 | 1.95322305 |
| Pgap1 | 0.00144 | -1.26560808 |
| Pgap1 | 0.12519 | 1.122346 |
| Zfc3h1 | 0.00144 | 1.44676887 |
| 1700088E04Rik | 0.0424 | -1.73786883 |
| Tfdp2 | 0.01837 | 1.83690268 |
| 4930562C15Rik | 0.00144 | -1.29049509 |
| Pgd | 0.01952 | -1.51449863 |
| Pgd | 0.07049 | -0.73596428 |
| Cd276 | 0.14469 | -0.49476283 |
| Trim12a | 0.00156 | 1.10611008 |
| Map2k2 | 0.02201 | -0.75510419 |
| Map2k2 | 0.11588 | -0.69189454 |
| Cldn34c1 | 0.56343 | 0.37279309 |
| D430041D05Rik | 0.00145 | -1.2388121 |
| Arhgef7 | 0.04439 | -0.62649067 |
| Arhgef7 | 0.37549 | -0.19167611 |
| Ttc17 | 0.20153 | 0.62647525 |
| Nr1h2 | 0.05652 | -1.74196707 |
| Cpn1 | 0.00356 | -1.08631 |
| Cdc42bpa | 0.00196 | 2.88113315 |
| Cdc42bpa | 0.06876 | 1.14653231 |
| Cdc42bpa | 0.04054 | -0.50852311 |
| Csnk1a1 | 0.05904 | 0.69557975 |
| Dnajc1 | 0.40274 | 0.6670616 |
| Bicd1 | 0.00144 | -1.31495282 |
| Schip1 | 0.04508 | 0.92762015 |
| Mpdz | 0.00415 | 1.8760646 |
| Mpdz | 0.87804 | 0.05980951 |
| Chid1 | 0.1475 | -0.440051 |
| Rexo4 | 0.57555 | -0.49997547 |
| Rhpn2 | 0.00144 | -1.24613586 |
| Ang6 | 0.72681 | -0.06317288 |
| Slbp | 0.11304 | -0.65977248 |
| Cntrob | 0.0242 | -1.32966974 |
| Pex1 | 0.15167 | 0.48929567 |
| Rbbp4 | 0.30127 | -0.4164292 |
| Lgr4 | 0.03819 | -0.99516681 |
| Mccc1 | 0.63563 | 0.27997408 |
| Tectb | 0.00144 | -1.24811894 |
| Asb11 | 0.50582 | 0.17268792 |
| Sec31a | 0.86652 | 0.08353388 |
| Sec31a | 0.20498 | 0.41359268 |
| Tk1 | 0.49313 | 0.80397821 |
| Ppfia1 | 0.1479 | -0.48845338 |
| Ppfia1 | 0.13451 | -0.90484274 |
| Cd37 | 0.32216 | 0.33299782 |
| Nfya | 0.02497 | -0.49047191 |
| Nlrp9c | 0.00144 | -1.20555712 |
| Oxsm | 0.18428 | 0.59918434 |
| Pros1 | 0.31542 | -0.48098117 |
| Ankrd6 | 0.00144 | -1.29864962 |
| 1700039E15Rik | 0.71182 | 0.10960652 |
| Dlg2 | 0.2985 | -0.52732 |
| Dlg2 | 0.27069 | -0.49536242 |
| Kif4 | 0.00144 | -1.29085005 |
| Postn | 0.03613 | 0.78811933 |
| Serpinb6a | 0.0154 | 1.4603991 |
| Rc3h2 | 0.00421 | 2.18406869 |
| Ankrd42 | 0.51978 | -0.17515383 |
| Elmo1 | 0.00404 | 2.93988541 |
| Elmo1 | 0.00144 | 2.15247003 |
| Trpc4ap | 0.11403 | -1.05181949 |
| Rars2 | 0.2164 | -1.10990182 |
| Scyl2 | 0.93013 | -0.04781548 |
| Scyl2 | 0.04616 | 1.02523854 |
| Ppil2 | 0.01281 | -0.79369099 |
| Snap25 | 0.00144 | -1.26560808 |
| Ptprc | 0.24844 | -0.87512728 |
| Ptprc | 0.86566 | 0.0625483 |
| Ptprc | 0.70646 | 0.11276496 |
| Ptprc | 0.34715 | -0.65565707 |
| Ptprc | 0.73148 | -0.30420142 |
| Dlgap1 | 0.00203 | -1.17111094 |
| Usp14 | 0.69985 | -0.21880279 |
| Usp14 | 0.5157 | 0.22505051 |
| Ttll7 | 0.05045 | 0.68176612 |
| Ppp1r21 | 0.11579 | -0.50877719 |
| Rpia | 0.99994 | -0.00003483 |
| 2310067B10Rik | 0.00309 | -1.45535495 |
| Tgfbrap1 | 0.01183 | -1.04972215 |
| Xrn1 | 0.00619 | 1.02306096 |
| Xrn1 | 0.98696 | 0.0039339 |
| Cacna1b | 0.03395 | -3.73325716 |
| 2310035C23Rik | 0.09147 | 0.39088272 |
| Sypl | 0.09217 | 0.59554578 |
| Rab12 | 0.35752 | 0.54086242 |
| Mbd2 | 0.01696 | -1.12385295 |
| Tmx4 | 0.03115 | -0.9700101 |
| Tmx4 | 0.10374 | -1.27032541 |
| Slmap | 0.36646 | -0.40441509 |
| Topbp1 | 0.29383 | 0.43258526 |
| Topbp1 | 0.29702 | -0.52286092 |
| Topbp1 | 0.18707 | 0.33344232 |
| Mdm4 | 0.4078 | -0.20904702 |
| Mdm4 | 0.67464 | -0.16192483 |
| Taz | 0.14424 | -0.6977255 |
| Tnfaip8 | 0.05249 | -0.8155198 |
| Tnfaip8 | 0.02921 | -0.94844092 |
| Slc2a9 | 0.00144 | -1.39762478 |
| Il2ra | 0.63393 | -0.19052215 |
| Rassf2 | 0.1167 | -0.42611582 |
| Csn3 | 0.01684 | -0.71241609 |
| Arhgef40 | 0.35462 | -0.91258769 |
| Cchcr1 | 0.55301 | -0.38951641 |
| Mboat7 | 0.29378 | -0.36264866 |
| Mboat7 | 0.10974 | -0.93759021 |
| Ccdc174 | 0.0683 | 0.96728144 |
| Pigb | 0.9809 | 0.0111487 |
| Pigb | 0.0055 | -0.94520465 |
| Skp2 | 0.01996 | -2.1225488 |
| Dstyk | 0.14879 | -2.37745707 |
| Aarsd1 | 0.00811 | -1.19015431 |
| D8Ertd82e | 0.60256 | 0.30199079 |
| Crtc3 | 0.1408 | 0.76861468 |
| Dlc1 | 0.15061 | 0.79067366 |
| Glis2 | 0.11195 | -0.55596579 |
| Bmpr2 | 0.01599 | 0.651512 |
| C330018D20Rik | 0.09891 | 0.56997838 |
| Efr3a | 0.9456 | 0.02645937 |
| Efr3a | 0.57339 | -0.39262724 |
| Slc4a4 | 0.0905 | -0.51582927 |
| Slc4a4 | 0.01409 | -1.96687176 |
| Epn2 | 0.31117 | -0.42872602 |
| Specc1 | 0.00471 | 1.00140252 |
| Atp7a | 0.344 | 0.46001255 |
| Ino80d | 0.27603 | 0.55128256 |
| Ctnna3 | 0.0032 | 1.10498666 |
| A630001G21Rik | 0.90754 | -0.04802228 |
| Vps13d | 0.09238 | -0.60328653 |
| Grin3a | 0.00144 | -1.51875969 |
| Nfix | 0.02577 | -0.98550712 |
| Adgrb3 | 0.23375 | 0.50363276 |
| Zfy2 | 0.00144 | -1.29085005 |
| Daw1 | 0.01992 | -0.77514563 |
| Nucb1 | 0.80594 | -0.05206391 |
| Scmh1 | 0.04595 | 0.94846228 |
| Napb | 0.14011 | -0.53959649 |
| Apol6 | 0.00144 | -1.53116772 |
| Mroh1 | 0.00144 | -1.29049509 |
| Zfp600 | 0.32376 | -0.2228148 |
| Sucla2 | 0.57168 | 0.3618054 |
| Rbm5 | 0.4753 | -0.26568844 |
| Rbm5 | 0.81817 | -0.14360225 |
| Cdk12 | 0.00144 | 2.51937544 |
| Mtmr1 | 0.00144 | -1.29085005 |
| Ept1 | 0.00144 | 1.84174642 |
| Daglb | 0.00229 | -1.455006 |
| Daglb | 0.02785 | -0.48293803 |
| Supt3 | 0.25788 | 0.43697004 |
| Nbeal1 | 0.10214 | 0.97803882 |
| Nbeal1 | 0.82681 | -0.04973602 |
| Nbeal1 | 0.24805 | -0.69501203 |
| Lgi1 | 0.00144 | -1.31035088 |
| D7Ertd443e | 0.29887 | -0.61337622 |
| Capn6 | 0.00144 | -1.29746368 |
| Tsen15 | 0.42995 | 0.26865856 |
| Asxl2 | 0.06803 | 1.06103618 |
| Uckl1 | 0.44198 | -0.31838192 |
| Dtd1 | 0.00144 | -1.29864962 |
| Smim7 | 0.00144 | -1.28898079 |
| Tspan33 | 0.50719 | 0.16066567 |
| Cpm | 0.0339 | -0.88465874 |
| Atp10b | 0.00144 | -1.32599123 |
| Prc1 | 0.37472 | -0.63927791 |
| Tmtc4 | 0.41869 | 0.21558926 |
| Kansl3 | 0.63286 | -0.20091204 |
| Ncapg | 0.39413 | -0.92537354 |
| Hyls1 | 0.00442 | -1 |
| Mcf2l | 0.12446 | 0.39537369 |
| Lpl | 0.03002 | 0.72662518 |
| Def6 | 0.00144 | -1.41628821 |
| Rab37 | 0.00144 | -1.28116048 |
| Cep162 | 0.02627 | 0.90439452 |
| Cpa2 | 0.02027 | 0.56654733 |
| Cpa2 | 0.00145 | -1.25646236 |
| Elac2 | 0.06217 | -0.50750939 |
| Stag1 | 0.58758 | -0.28571742 |
| Bgn | 0.02699 | -0.63196069 |
| Mylk | 0.46107 | -0.32236179 |
| 2010315B03Rik | 0.00144 | 1.87775493 |
| BC005624 | 0.3073 | -0.35148259 |
| Hcn3 | 0.04363 | -1.6907899 |
| Uty | 0.0246 | 1.83867051 |
| Ulk4 | 0.58213 | -0.13495292 |
| Ick | 0.10448 | -0.60505814 |
| Tpi1 | 0.94373 | -0.02047554 |
| Adcy10 | 0.0017 | -1.24950944 |
| Adcy10 | 0.27666 | -0.9298055 |
| Prrc2c | 0.01304 | 1.84442932 |
| Srgn | 0.97573 | 0.01426162 |
| Etfdh | 0.03014 | 0.51658778 |
| Hspa14 | 0.93753 | 0.03259352 |
| Hspa14 | 0.12489 | -0.76426058 |
| Dock11 | 0.57225 | 0.3572966 |
| Mid2 | 0.31801 | 0.84980535 |
| Zfp407 | 0.00171 | 2.3255692 |
| Mdga2 | 0.00144 | -1.45290105 |
| Glud1 | 0.00512 | -0.72760288 |
| Uqcc1 | 0.22603 | -0.51388932 |
| Gatad1 | 0.02629 | 0.62370255 |
| Ppm1h | 0.3386 | -0.59553212 |
| Hnrnpl | 0.6172 | 0.13209233 |
| Rarg | 0.22319 | -0.80603896 |
| Rarg | 0.00144 | -1.28116048 |
| Ccdc88a | 0.28451 | -0.41597584 |
| Garnl3 | 0.00214 | -1.29719646 |
| Garnl3 | 0.03111 | -0.84904348 |
| Col1a2 | 0.01139 | 1.0255767 |
| 1700003F12Rik | 0.01412 | -0.94644062 |
| Trip12 | 0.0078 | 1.46165801 |
| Trip12 | 0.06845 | 0.35204235 |
| Trip12 | 0.06262 | 1.30601038 |
| Mtf1 | 0.35179 | -0.16213201 |
| Macrod2 | 0.00158 | -1.2446827 |
| Alkbh6 | 0.84044 | 0.1311436 |
| Slc43a2 | 0.75795 | -0.09060154 |
| Dock2 | 0.34487 | -0.45413509 |
| Mcu | 0.60521 | -0.19860926 |
| Mcu | 0.0574 | -0.79212529 |
| Mcu | 0.54516 | 0.29421222 |
| Pcnxl2 | 0.00144 | -1.26560808 |
| Timp3 | 0.00144 | 1.2576212 |
| Ncald | 0.01675 | -1.2613507 |
| Ncald | 0.97356 | -0.02056787 |
| Cstad | 0.0432 | -1.53973735 |
| Nudcd3 | 0.76417 | -0.09697387 |
| Ptprd | 0.62002 | -0.2087751 |
| Dnajc10 | 0.00144 | -1.48622667 |
| Tnnt2 | 0.39079 | 0.36244445 |
| Tnnt2 | 0.07565 | -0.59156004 |
| Cd86 | 0.13329 | 0.91901527 |
| Sorcs1 | 0.00144 | -2.57628958 |
| Sorcs1 | 0.00144 | -1.30713006 |
| Bub1 | 0.00144 | -1.24811894 |
| Chdh | 0.00144 | -1.26934956 |
| Sla | 0.00144 | -1.29085005 |
| Cnksr2 | 0.00144 | -1.24353547 |
| Prkag2 | 0.04193 | -0.83444958 |
| Rxfp1 | 0.00145 | -1.18741414 |
| Reln | 0.00144 | -1.21868187 |
| Reln | 0.12997 | 0.4253748 |
| Swt1 | 0.32353 | -0.27674053 |
| Swt1 | 0.0023 | 1.37675785 |
| Swt1 | 0.16581 | 0.56983663 |
| Agap1 | 0.03346 | -0.6951936 |
| Ppa1 | 0.9894 | 0.00314746 |
| Rab3gap1 | 0.77771 | -0.05510108 |
| Rab3gap1 | 0.0732 | -0.677488 |
| Rab3gap1 | 0.01579 | 1.13664191 |
| Actn4 | 0.26323 | -0.35122636 |
| Map1lc3b | 0.01405 | -1.06267913 |
| Rasal2 | 0.13944 | 0.89551075 |
| Phf20 | 0.13958 | -0.38677378 |
| Zfyve27 | 0.03327 | -1.1443198 |
| Zfyve27 | 0.3349 | -0.29100083 |
| Pold3 | 0.00601 | -1.49316309 |
| Cdk8 | 0.19226 | -0.75559551 |
| Copz2 | 0.0375 | -0.65925873 |
| Crbn | 0.11869 | -0.49961109 |
| Zcchc17 | 0.83735 | -0.06494464 |
| Atp13a4 | 0.41711 | 0.99439982 |
| Rad23b | 0.00903 | 2.36550687 |
| Otud5 | 0.13549 | -0.67697729 |
| Nupl2 | 0.01749 | -1.19088193 |
| Atf7 | 0.02491 | 0.96060925 |
| Senp3 | 0.5334 | 0.1980801 |
| Tcaf1 | 0.79176 | 0.09630435 |
| Ppp3cb | 0.02503 | 0.66828182 |
| Ppp3cb | 0.23753 | 0.56412035 |
| Glrx2 | 0.00292 | -1.81295493 |
| Myrf | 0.27486 | -0.49541521 |
| Zfp536 | 0.00145 | -1.22376446 |
| Zfp536 | 0.06495 | -0.92433994 |
| Rxfp2 | 0.03599 | -2.00745594 |
| Pdzd2 | 0.36091 | -0.25118919 |
| Pdzd2 | 0.0313 | 1.18195097 |
| Plag1 | 0.04051 | 1.08876918 |
| Hspa4l | 0.0213 | 1.27561783 |
| Dixdc1 | 0.08578 | 0.6185762 |
| Vmn1r67 | 0.00405 | -1.75764491 |
| Frmd4b | 0.08084 | -0.48611102 |
| Sccpdh | 0.92151 | -0.03907882 |
| Sptbn4 | 0.405 | -0.37872313 |
| Cacnb2 | 0.00144 | 1.19775527 |
| Zc3h7a | 0.19205 | -0.21380442 |
| Zc3h7a | 0.0058 | -1.08147291 |
| Dr1 | 0.29118 | -0.51019476 |
| Wbp2 | 0.04674 | -0.50862666 |
| Cyyr1 | 0.00219 | 2.61005602 |
| Adh5 | 0.00375 | -1.82287719 |
| Plxna2 | 0.00754 | 1.21520557 |
| Herc1 | 0.31807 | 0.41130559 |
| Herc1 | 0.34675 | -0.65732854 |
| Clcn3 | 0.00144 | 1.50204651 |
| Sec23b | 0.76076 | 0.09311981 |
| Itga4 | 0.35741 | -0.33621872 |
| Clec5a | 0.43328 | -0.25630403 |
| Snx10 | 0.09622 | 0.72994503 |
| Rbm6 | 0.10192 | 0.67943898 |
| Rbm6 | 0.62234 | 0.19189775 |
| Aff2 | 0.16247 | -0.7401643 |
| Itfg2 | 0.76652 | -0.11311239 |
| Tns1 | 0.04558 | -0.85437633 |
| Mrps5 | 0.00145 | -1.27532353 |
| Fgd1 | 0.04611 | -0.59496586 |
| Rint1 | 0.95897 | -0.01662655 |
| Paics | 0.07908 | 1.61213464 |
| Mrpl13 | 0.00738 | 1.780693 |
| Bbs9 | 0.94025 | 0.03597494 |
| Ctse | 0.00149 | -1.19348234 |
| Eps15 | 0.00149 | -1.28183002 |
| Pi4ka | 0.09462 | 0.65080418 |
| Pi4ka | 0.86619 | -0.09396448 |
| Rab14 | 0.99618 | -0.00242816 |
| Nup160 | 0.51344 | 0.23718637 |
| Micu3 | 0.22978 | -0.34948668 |
| Zfp600 | 0.30301 | -0.18116158 |
| Pstpip1 | 0.05325 | -0.7470027 |
| Pstpip1 | 0.03555 | 0.83839178 |
| Gfm2 | 0.01466 | 1.10199891 |
| Dph6 | 0.00145 | -1.22376446 |
| Rere | 0.57577 | -0.20180651 |
| Clic4 | 0.5415 | 0.25780329 |
| Ppp2r2b | 0.00381 | -2.00461686 |
| Nim1k | 0.06361 | 1.16050605 |
| Arhgap31 | 0.00558 | 1.76305377 |
| Tlr4 | 0.00574 | -0.69385233 |
| Noa1 | 0.70901 | 0.11175978 |
| Wdr62 | 0.32152 | -0.56749702 |
| Wrb | 0.00145 | -1.24191372 |
| BC052040 | 0.00144 | -1.22098834 |
| Trp53i13 | 0.01302 | -0.9168835 |
| Cpa6 | 0.00144 | -1.28898079 |
| Sirt7 | 0.28751 | -0.44084186 |
| Sirt7 | 0.80336 | -0.15309092 |
| Nmnat2 | 0.82425 | 0.12207114 |
| Nmnat2 | 0.05778 | -2.18205045 |
| Ppfibp1 | 0.10638 | 0.64460181 |
| Nfkbiz | 0.00144 | -1.30713006 |
| Wipf3 | 0.02311 | 0.98185693 |
| Cadm1 | 0.33928 | -0.67344955 |
| Nf1 | 0.8156 | 0.04469299 |
| Art3 | 0.00157 | -1.19247859 |
| Snap23 | 0.10621 | -0.55369046 |
| 9030624J02Rik | 0.03614 | -1.0408577 |
| Stx8 | 0.1086 | -0.29515298 |
| Abca12 | 0.00145 | -1.22376446 |
| Nav2 | 0.77932 | 0.16287152 |
| Nav2 | 0.56341 | -0.35353886 |
| Nav2 | 0.58947 | -0.14283245 |
| Nav2 | 0.00268 | -1.38302854 |
| Abcd2 | 0.88217 | -0.23127567 |
| LOC105245084 | 0.00144 | -1.49553518 |
| Bbs5 | 0.09419 | -0.78178693 |
| Plekha3 | 0.00247 | -1.14387982 |
| Iqce | 0.06839 | -0.92001081 |
| Slc9a9 | 0.38966 | 0.34933653 |
| Cntn6 | 0.0412 | -0.51701034 |
| Nt5m | 0.73884 | -0.2306259 |
| Gga2 | 0.62916 | 0.20015877 |
| Gga2 | 0.00625 | -0.94913233 |
| Pqlc1 | 0.04862 | -2.43774877 |
| Prex2 | 0.12889 | 1.01263263 |
| Frmd4a | 0.01537 | 2.22052939 |
| Frmd4a | 0.53413 | 0.18058066 |
| Bhmt | 0.00144 | -1.30017714 |
| Pofut1 | 0.85488 | 0.07465931 |
| Orc4 | 0.00204 | 1.40429508 |
| Ttc22 | 0.00608 | -1.41620077 |
| Zfp945 | 0.01505 | 1.26764298 |
| Gm13547 | 0.08495 | -0.42054464 |
| Vcp | 0.00161 | -1.05514474 |
| Sptbn1 | 0.00217 | 2.18053615 |
| Sptbn1 | 0.28704 | -0.29258631 |
| Ntan1 | 0.09661 | 0.30680693 |
| Ptpn1 | 0.00144 | -1.28116048 |
| Apc | 0.23635 | 0.47115587 |
| Hibch | 0.9578 | -0.0399745 |
| Dhx30 | 0.66973 | -0.12141027 |
| Cfap44 | 0.43371 | -0.50892945 |
| Plgrkt | 0.90095 | 0.05687938 |
| Arhgap29 | 0.37582 | 0.629784 |
| Sharpin | 0.0579 | -0.96335366 |
| Tenm4 | 0.00152 | -1.25878908 |
| Ndufs3 | 0.05445 | -0.84200562 |
| Rnf157 | 0.09883 | 0.78727089 |
| Rnf157 | 0.08647 | -0.51532975 |
| Ankrd11 | 0.28023 | -0.36659408 |
| Tenm1 | 0.35693 | -0.28266564 |
| Esrrg | 0.48709 | 0.35520114 |
| Rwdd3 | 0.51846 | -0.19327719 |
| Smg6 | 0.01141 | 1.40871877 |
| Lrfn5 | 0.00648 | -1.71166556 |
| Lrfn5 | 0.00144 | -1.28898079 |
| Dapk2 | 0.88038 | 0.05997599 |
| Hist2h2ac | 0.00144 | -1.43580951 |
| Eif2s2 | 0.03372 | 1.72594788 |
| Sat1 | 0.08808 | -0.42500939 |
| BC004004 | 0.20971 | -0.4876592 |
| Abat | 0.08585 | -0.95081427 |
| Lhfpl2 | 0.86111 | 0.07033267 |
| Bbx | 0.02662 | 1.35404939 |
| Bbx | 0.20927 | 0.42017585 |
| Crebbp | 0.68546 | -0.27010841 |
| Wdr31 | 0.55033 | 0.18068969 |
| Mtus1 | 0.59424 | 0.15294056 |
| Ccr5 | 0.74212 | -0.17698584 |
| Pam | 0.00144 | -1.27165604 |
| Baz2b | 0.035 | 0.83520653 |
| Baz2b | 0.01422 | 2.25638447 |
| 1700049G17Rik | 0.1606 | -0.26260659 |
| 1700049G17Rik | 0.47154 | -0.29803404 |
| 1700049G17Rik | 0.00934 | 1.51042386 |
| 1700049G17Rik | 0.00144 | -1.28797948 |
| Sos2 | 0.12581 | 0.90433445 |
| Ssbp2 | 0.00144 | 2.47336231 |
| Insc | 0.00149 | -1.2703832 |
| Wwox | 0.85624 | 0.08336681 |
| Wwox | 0.18746 | -1.89205316 |
| Flywch2 | 0.3407 | -0.47017401 |
| Cyp4f13 | 0.31442 | 0.53126528 |
| Myh10 | 0.07417 | -0.65840838 |
| Opa3 | 0.62825 | 0.27138884 |
| Zbtb44 | 0.96513 | 0.02285553 |
| Ly6g6d | 0.01729 | -1.12402572 |
| Rgs6 | 0.51229 | -0.32550496 |
| Eef1d | 0.31006 | -0.54750357 |
| Cxxc4 | 0.01859 | -0.79241049 |
| Zfat | 0.68819 | -0.18375776 |
| Zfp369 | 0.01893 | 0.67612927 |
| Ldlrad3 | 0.48795 | -0.34057774 |
| Sh3bgrl2 | 0.38749 | -0.21460173 |
| Skint10 | 0.00144 | -1.27165604 |
| Skint10 | 0.00675 | -1.49493983 |
| Btbd11 | 0.16413 | -0.36884491 |
| Gtpbp10 | 0.0796 | -0.58533601 |
| Lynx1 | 0.00144 | -1.34764358 |
| Brinp1 | 0.00144 | -1.25927895 |
| Apc2 | 0.86926 | -0.04177939 |
| Eaf2 | 0.44049 | -1.5829777 |
| Tank | 0.21722 | 0.83794351 |
| Fignl1 | 0.32225 | -0.56086621 |
| Map3k12 | 0.00144 | -1.28898079 |
| Map3k12 | 0.70098 | 0.22901305 |
| Zfp667 | 0.00144 | -1.20555712 |
| Zfp60 | 0.68726 | -0.1023632 |
| Vprbp | 0.56691 | -0.12603919 |
| Gabrg3 | 0.00336 | -1.20864476 |
| Mrps22 | 0.06037 | 0.37924197 |
| Pusl1 | 0.00144 | -1.20555712 |
| Zbtb40 | 0.76398 | 0.17171012 |
| Ormdl1 | 0.59629 | 0.18713592 |
| Lypla1 | 0.06983 | 0.85380259 |
| Slc1a3 | 0.00144 | -1.31014844 |
| Tmem140 | 0.74723 | 0.11327432 |
| Kdm6a | 0.01534 | 1.40528933 |
| Urm1 | 0.00536 | -0.91145475 |
| Lgals3bp | 0.24441 | 0.55150767 |
| Slc9c1 | 0.00733 | -1.84592979 |
| Gm10874 | 0.00144 | -1.27165604 |
| Tns3 | 0.09705 | -0.56874985 |
| Ttk | 0.10543 | -2.74841779 |
| Rab27b | 0.02162 | -0.95716175 |
| Phactr1 | 0.0487 | 1.13778173 |
| Zfp951 | 0.14177 | -0.67932262 |
| Med20 | 0.2917 | -1.00944233 |
| 5430421N21Rik | 0.00583 | -1.05828938 |
| Ccdc25 | 0.14236 | -0.3255963 |
| Aig1 | 0.059 | 1.02249258 |
| Rad52 | 0.69349 | -0.30137999 |
| Galnt1 | 0.1665 | 0.84380867 |
| Galnt1 | 0.03029 | 1.02621548 |
| Mbnl3 | 0.24704 | -0.59164448 |
| Mob3a | 0.81193 | 0.06851982 |
| Mroh5 | 0.06602 | -0.78477727 |
| Pnkd | 0.68614 | 0.08626474 |
| Plxna3 | 0.04971 | -0.56321996 |
| Plxna3 | 0.75492 | -0.18528713 |
| Ikbkb | 0.9419 | 0.03220512 |
| Ikbkb | 0.70631 | -0.16238742 |
| Cpne5 | 0.97679 | 0.02382141 |
| Sparc | 0.16972 | 0.61694842 |
| Emg1 | 0.35561 | -0.2986694 |
| Emg1 | 0.01444 | -0.6660774 |
| Lysmd1 | 0.02468 | -0.56524152 |
| Ripk1 | 0.00594 | 0.87994896 |
| Zfp111 | 0.00144 | -1.24811894 |
| Zfp111 | 0.12322 | -0.82044372 |
| Sidt1 | 0.11319 | -0.47017401 |
| Abca13 | 0.00149 | -1.2703832 |
| Fgfbp1 | 0.02388 | -1.10247618 |
| Dgcr8 | 0.82735 | -0.1166132 |
| Sp9 | 0.59866 | -0.35561759 |
| Arhgef10 | 0.06 | 0.6066412 |
| Rnpep | 0.20909 | 0.47090079 |
| Hepacam2 | 0.00204 | -1.2031737 |
| Tsga10 | 0.62095 | -0.19847536 |
| Katnal1 | 0.55668 | -0.32710617 |
| Arhgap24 | 0.1303 | 0.65133152 |
| Mat2b | 0.12153 | 0.80611332 |
| Stradb | 0.05811 | 1.73763234 |
| Stradb | 0.39912 | 0.42237323 |
| Pbx1 | 0.13402 | 0.70055449 |
| Dyrk1a | 0.49059 | -0.24877063 |
| Pou1f1 | 0.17743 | -2.45129468 |
| Blzf1 | 0.0153 | 1.58995514 |
| Zfp770 | 0.53911 | 0.24122694 |
| Il27ra | 0.00149 | -1.22890891 |
| Prss1 | 0.06859 | -0.70420962 |
| Kcnab1 | 0.96993 | 0.02256001 |
| Spata7 | 0.50868 | 0.77605412 |
| Spata7 | 0.03056 | 1.42101147 |
| Map2k3 | 0.14661 | -1.28510702 |
| Tsen2 | 0.14491 | 0.59253586 |
| Cd84 | 0.0073 | 1.45702553 |
| Dhx9 | 0.16772 | 0.71313672 |
| Dhx9 | 0.13878 | -0.76192444 |
| Dmxl1 | 0.00773 | 1.02644082 |
| Dmxl1 | 0.02298 | 2.14014016 |
| Fam19a1 | 0.00144 | -1.29746368 |
| Ift46 | 0.31525 | -0.33807328 |
| Elk1 | 0.49639 | -0.2487881 |
| Fmo2 | 0.93274 | -0.04922408 |
| Inip | 0.46745 | -0.1250001 |
| Inip | 0.00408 | 0.78378705 |
| B430306N03Rik | 0.00145 | -1.2388121 |
| Arhgap32 | 0.14546 | 0.55163879 |
| Top2a | 0.64305 | -0.24893836 |
| Pak2 | 0.00144 | 2.06239731 |
| Gm14085 | 0.72917 | 0.09991142 |
| Kdm5d | 0.00144 | -1.25927895 |
| Kdm5d | 0.79998 | -0.07488248 |
| Nxph1 | 0.00144 | -1.32000433 |
| 6330408A02Rik | 0.4302 | 0.35632242 |
| 6330408A02Rik | 0.68948 | -0.25986611 |
| Ccdc112 | 0.03222 | -1.02697333 |
| Qprt | 0.02216 | -0.99916438 |
| Msh2 | 0.26198 | -0.36205216 |
| Zmpste24 | 0.24505 | 0.67201316 |
| Gas7 | 0.00158 | -1.2446827 |
| St6galnac2 | 0.41336 | -0.29892798 |
| Mrpl23 | 0.00253 | -1.21845061 |
| Sipa1l3 | 0.02682 | -0.62616744 |
| Sipa1l3 | 0.34938 | 0.37290541 |
| Birc3 | 0.06115 | 1.20580915 |
| Birc3 | 0.38597 | 0.42651771 |
| Slc28a3 | 0.00144 | -1.49050422 |
| Slc28a3 | 0.00144 | -1.20555712 |
| Ddit3 | 0.52431 | -0.229363 |
| Sp110 | 0.0049 | -1.21517936 |
| Zfp273 | 0.53953 | 0.86732358 |
| Ankrd50 | 0.06224 | -0.41569629 |
| Tmprss5 | 0.7735 | 0.14409644 |
| Wdr4 | 0.40422 | -0.53270462 |
| Cep57l1 | 0.00272 | -1.09759392 |
| Jakmip1 | 0.00144 | -1.20228444 |
| Jakmip1 | 0.00144 | -1.24613586 |
| Rad51ap1 | 0.87178 | 0.16872846 |
| Tbr1 | 0.00218 | -1.18878227 |
| Med27 | 0.01031 | -1.01649833 |
| Lsm8 | 0.1045 | -2.73730421 |
| Phykpl | 0.16804 | -0.79536861 |
| Enpp1 | 0.00144 | -1.20555712 |
| Slc35f4 | 0.10513 | -0.88511728 |
| Gnb4 | 0.00194 | 1.84257624 |
| Gnb4 | 0.06389 | 0.5928346 |
| Parvb | 0.81994 | -0.07187661 |
| Begain | 0.00432 | -1.30168581 |
| Rlim | 0.03541 | 1.07454088 |
| Snx1 | 0.04223 | 0.93214618 |
| Pot1a | 0.00411 | 0.89466519 |
| Cenpq | 0.016 | 0.97548281 |
| Tmed3 | 0.92094 | 0.03961539 |
| Peg3 | 0.18846 | -2.16569716 |
| Ccm2 | 0.9121 | 0.06860682 |
| Heg1 | 0.13941 | 0.47018739 |
| Tmem168 | 0.01976 | 1.11801691 |
| Lingo2 | 0.00144 | -1.29085005 |
| Thop1 | 0.00158 | 2.61690407 |
| Bclaf1 | 0.016 | 0.94487923 |
| Ncbp1 | 0.03628 | -1.60571198 |
| Sema3c | 0.43793 | 0.48458512 |
| Park2 | 0.00144 | -1.29864962 |
| Park2 | 0.2076 | 0.67879281 |
| Npc1 | 0.06534 | 0.6760997 |
| Npc1 | 0.04108 | 1.2956754 |
| Snd1 | 0.43561 | -0.14172566 |
| Zeb1 | 0.00144 | 3.33035498 |
| Camk2b | 0.00144 | -1.26560808 |
| Zfp26 | 0.01552 | -0.97598014 |
| Pld1 | 0.80592 | 0.20253884 |
| Spag6 | 0.00144 | -1.20555712 |
| Bag2 | 0.13798 | -0.58982782 |
| Top3b | 0.0019 | -1.17487561 |
| Nmt2 | 0.27843 | 0.27534898 |
| Pus7 | 0.00144 | -1.29085005 |
| Rrm2b | 0.00144 | -1.27165604 |
| Clec4g | 0.24446 | -0.55053424 |
| Gm15881 | 0.00728 | -1.49415147 |
| Gm9899 | 0.11927 | -0.58557628 |
| Cdv3 | 0.05967 | -0.40725164 |
| H13 | 0.0607 | -0.63796659 |
| Ccdc132 | 0.0879 | 0.48912769 |
| Lrp4 | 0.04287 | -0.42750195 |
| Ndufa7 | 0.12683 | -0.49036485 |
| 1700012D01Rik | 0.13217 | -0.34885222 |
| Ccdc24 | 0.00389 | -1.20792335 |
| Mis12 | 0.00534 | 1.23782878 |
| Acsl1 | 0.03282 | 1.4167724 |
| Zfp932 | 0.06538 | 0.53368409 |
| Mtif2 | 0.0892 | 0.31642907 |
| Churc1 | 0.12154 | 0.51772018 |
| Nr6a1 | 0.00145 | -1.22193674 |
| Huwe1 | 0.00191 | -1.00604163 |
| Capza1 | 0.00759 | -1.4875599 |
| Phf8 | 0.874 | 0.09756356 |
| Stard6 | 0.00144 | 1.98376695 |
| 4932438A13Rik | 0.12367 | 0.89503253 |
| Atxn2 | 0.00554 | -1.61017593 |
| Atxn2 | 0.90651 | -0.04239641 |
| Synpr | 0.00144 | -1.31495282 |
| Eif4a2 | 0.80045 | -0.07563531 |
| Adam32 | 0.21891 | -0.67785085 |
| Adam32 | 0.98963 | 0.00539514 |
| Nae1 | 0.30004 | 0.44669188 |
| Armc9 | 0.00144 | -1.29049509 |
| Cnot6 | 0.00767 | 0.86273669 |
| Cnot6 | 0.00144 | 1.23819135 |
| Abca4 | 0.00144 | -1.27992547 |
| Chchd7 | 0.16941 | -0.31881076 |
| Phyh | 0.19463 | -0.20250461 |
| Tmem135 | 0.40175 | -0.23407501 |
| Usp32 | 0.00578 | -0.99717308 |
| Plekha1 | 0.47507 | -0.31790212 |
| Mag | 0.4929 | 0.18140615 |
| Smn1 | 0.01294 | -2.04538177 |
| Sec24b | 0.54087 | -0.36695528 |
| Timd2 | 0.00987 | -1.67001789 |
| Hk1 | 0.01487 | -1.44365757 |
| Ankzf1 | 0.00297 | -0.91178601 |
| Mbtd1 | 0.01218 | 0.80990762 |
| Mbtd1 | 0.47889 | -0.14149237 |
| Spag9 | 0.54238 | 0.39414897 |
| Spag9 | 0.78019 | 0.05237438 |
| Vwa5a | 0.00317 | -1.06812379 |
| Gk5 | 0.01782 | -2.06195552 |
| Cdh26 | 0.01532 | -1.3124439 |
| Fmo4 | 0.03448 | -1.90123828 |
| Rbm22 | 0.00144 | -1.27165604 |
| Sh3bgr | 0.49137 | 0.13071415 |
| Cd200r3 | 0.03201 | -2.15432002 |
| Prrg4 | 0.31514 | -1.32583003 |
| Parva | 0.19127 | 0.65930703 |
| Nudt19 | 0.23025 | 0.59775232 |
| Sacs | 0.16691 | 0.39400791 |
| Cenpf | 0.52318 | -0.24161009 |
| Ptprg | 0.00144 | 2.52880179 |
| Zfp558 | 0.55718 | -0.36396958 |
| Zfp558 | 0.00423 | -1.10677474 |
| Acin1 | 0.00144 | 1.67310505 |
| Stxbp5 | 0.0051 | -1.06784446 |
| Lancl1 | 0.49417 | -0.2393053 |
| Lancl1 | 0.00737 | -0.84117392 |
| Slc15a2 | 0.00144 | -1.20555712 |
| Slc15a2 | 0.20456 | -0.61288979 |
| Slc15a2 | 0.8218 | 0.35877783 |
| Pms1 | 0.00144 | -1.17424379 |
| Cpeb1 | 0.19495 | -0.50269351 |
| Smim9 | 0.09551 | -1.09233697 |
| Tm6sf1 | 0.03014 | 0.62608146 |
| Zfp62 | 0.10349 | -0.550476 |
| Zfp62 | 0.57485 | -0.23360993 |
| Neb | 0.96887 | 0.05021889 |
| Pde4d | 0.00144 | -1.28116048 |
| Dguok | 0.19136 | -0.2602095 |
| Pigr | 0.0144 | -2.22343406 |
| Atrx | 0.00144 | 1.63087009 |
| 2610301B20Rik | 0.00145 | -1.25646236 |
| Rsrc2 | 0.01374 | 1.19731253 |
| Ctcf | 0.1822 | -1.25264174 |
| Ankhd1 | 0.98257 | 0.01049555 |
| Stx4a | 0.03014 | 0.43866692 |
| Ttc29 | 0.00152 | -1.25878908 |
| Ttc29 | 0.08762 | -1.08951567 |
| Cct8 | 0.00677 | 1.10157527 |
| Alg6 | 0.06783 | -0.56443694 |
| Alg6 | 0.53443 | -0.18548679 |
| Alg6 | 0.37624 | 0.34640355 |
| Whsc1 | 0.014 | -0.5883878 |
| Mrrf | 0.41172 | 0.2663988 |
| Nipa2 | 0.00144 | -1.05891812 |
| Prrg1 | 0.78256 | -0.08414293 |
| Slc35d1 | 0.01163 | 1.08891713 |
| Bzrap1 | 0.80249 | -0.17770786 |
| Fnip2 | 0.0818 | -2.30478397 |
| Wnk1 | 0.01856 | -0.8262571 |
| 2410089E03Rik | 0.42844 | -0.18883734 |
| Trim3 | 0.20276 | 0.66524779 |
| Magohb | 0.15126 | 0.31114459 |
| 4430402I18Rik | 0.01561 | -0.57292913 |
| Gtf3c3 | 0.17321 | 0.61469715 |
| Osgin2 | 0.07564 | 1.0847724 |
| Mrpl22 | 0.77628 | 0.13233558 |
| Suz12 | 0.66453 | -0.19693208 |
| Piezo2 | 0.01122 | -1.23784157 |
| Smim8 | 0.18029 | 0.67591076 |
| Mitf | 0.29353 | 0.50797979 |
| Mier3 | 0.4927 | 0.33446708 |
| Ubr3 | 0.00144 | 1.85996196 |
| Bsdc1 | 0.06113 | -1.18090554 |
| Hnrnpm | 0.82852 | 0.20028205 |
| Mcm6 | 0.90174 | -0.02735063 |
| 1700057G04Rik | 0.0031 | -1.4243395 |
| 1700057G04Rik | 0.13174 | -3.0212454 |
| Srd5a3 | 0.21286 | -0.46998037 |
| Nt5c3 | 0.04845 | 0.60610323 |
| Nt5c3 | 0.00372 | 0.81685281 |
| Zfp426 | 0.53829 | -0.71589324 |
| Brwd1 | 0.86433 | 0.03449923 |
| Brwd1 | 0.19214 | 0.54905765 |
| Cfap46 | 0.00314 | -1.7108091 |
| Cnot6l | 0.03027 | 0.52869564 |
| Nsun2 | 0.00268 | -0.83607215 |
| Etl4 | 0.053 | 0.83472799 |
| Lrrc8d | 0.90645 | 0.05939586 |
| Map3k7 | 0.19237 | -0.33129049 |
| Kpna2 | 0.00144 | -1.28898079 |
| Ncoa6 | 0.8514 | -0.04950132 |
| Rnf20 | 0.12068 | -0.55076866 |
| Nup214 | 0.14415 | -0.92873054 |
| Ipo8 | 0.88454 | 0.03612053 |
| Smchd1 | 0.06819 | 0.57893048 |
| Smchd1 | 0.04339 | -0.83038016 |
| Nop58 | 0.00144 | 2.7421885 |
| Nop58 | 0.00144 | 2.05365427 |
| Nop58 | 0.14496 | 0.31407456 |
| Zfp326 | 0.03739 | 0.47786455 |
| Matr3 | 0.00405 | -0.81097847 |
| Cttnbp2 | 0.00232 | -1.43253471 |
| Cttnbp2 | 0.09683 | 0.52161247 |
| Api5 | 0.00378 | -0.85939507 |
| Adarb1 | 0.05179 | -0.54132784 |
| Slit2 | 0.12902 | -1.03882858 |
| Slit2 | 0.1452 | -2.72747026 |
| Rasa1 | 0.00144 | 1.94240093 |
| Mterf4 | 0.01849 | -1.00109171 |
| 3110043O21Rik | 0.00144 | -1.31473432 |
| 3110043O21Rik | 0.00144 | -1.2963229 |
| Cpne3 | 0.70755 | -0.16811468 |
| Ipo9 | 0.13143 | -0.4793213 |
| Dab2 | 0.06152 | -1.02801782 |
| Rpp40 | 0.1248 | -0.26167445 |
| Ttc23 | 0.06572 | -0.70546822 |
| Zfp804a | 0.00145 | -1.22376446 |
| Frmd7 | 0.00144 | -1.29085005 |
| Eif4g1 | 0.00498 | 1.07394741 |
| Eif4g1 | 0.2277 | -0.52663642 |
| AI314180 | 0.24223 | 0.50534479 |
| Fam171b | 0.1001 | -0.56987987 |
| Ccdc53 | 0.41577 | 0.35653631 |
| Rb1 | 0.35388 | 0.15010812 |
| Orc5 | 0.83319 | -0.08011689 |
| Stoml3 | 0.01912 | -0.94237278 |
| Mllt3 | 0.71378 | 0.07170203 |
| Arpp21 | 0.06218 | -0.47625065 |
| Eri2 | 0.17172 | -0.88511728 |
| Slco3a1 | 0.15238 | -0.57280133 |
| Itgav | 0.94347 | -0.01696167 |
| Itgav | 0.15013 | -0.78752336 |
| Atad5 | 0.31562 | -0.48550471 |
| Plce1 | 0.00388 | 1.07024977 |
| Sag | 0.70423 | -0.24000958 |
| Ap1g1 | 0.12112 | -0.82977507 |
| Ap1g1 | 0.89669 | 0.02087424 |
| Myo3a | 0.00144 | -1.29085005 |
| Aggf1 | 0.04588 | 0.48408665 |
| Sec22a | 0.96738 | 0.00754994 |
| Pkib | 0.45788 | -0.52865078 |
| Rpa2 | 0.0093 | -1.1233389 |
| Grin2b | 0.00144 | -1.35366536 |
| Exoc1 | 0.04038 | 1.29681807 |
| 1700112E06Rik | 0.81467 | -0.13177117 |
| Lrrc36 | 0.00144 | -1.28898079 |
| Luc7l | 0.00144 | 1.3727417 |
| Drp2 | 0.00144 | -1.28116048 |
| Cflar | 0.24021 | -0.41108731 |
| Ddx47 | 0.12171 | -0.38447896 |
| Zbtb37 | 0.36377 | -0.39989078 |
| Exosc8 | 0.00144 | 1.90364805 |
| Exosc8 | 0.18054 | 0.33516493 |
| Tec | 0.00144 | -1.31495282 |
| C330021F23Rik | 0.62486 | 0.26371923 |
| Plek | 0.50971 | 0.177823 |
| Oxsm | 0.00144 | -1.30017714 |
| Stt3a | 0.00575 | -0.62454033 |
| Mapk6 | 0.01169 | -0.82663569 |
| Col4a3 | 0.18139 | -1.26714694 |
| St7 | 0.33647 | -0.30186553 |
| St7 | 0.12324 | 0.84994899 |
| Aco1 | 0.00144 | -1.52351291 |
| Lama2 | 0.76595 | 0.10271843 |
| Tm9sf2 | 0.03256 | -0.62030658 |
| LOC102634852 | 0.00144 | -1.26560808 |
| Prkci | 0.04186 | -0.89185502 |
| Glmn | 0.2562 | 0.54365154 |
| Nmd3 | 0.03165 | -0.55197669 |
| Mapk1 | 0.25357 | 0.46523639 |
| Inpp5f | 0.02653 | -0.58725796 |
| Abi2 | 0.4078 | -0.21619544 |
| Saxo2 | 0.14028 | -0.79695848 |
| Maf | 0.18495 | 0.28844633 |
| Tcp1 | 0.44272 | -0.2444096 |
| Tcp1 | 0.58147 | 0.18636978 |
| Cdc123 | 0.07215 | 0.48641243 |
| Cdc123 | 0.00172 | 1.21811779 |
| Cep350 | 0.11631 | -0.41489929 |
| Amn1 | 0.05207 | 0.80942421 |
| Smc1a | 0.00224 | -1.21985214 |
| Trrap | 0.04107 | -1.34504069 |
| Zwilch | 0.0963 | -1.28502633 |
| Pitpna | 0.01383 | 1.20095728 |
| Arih1 | 0.11289 | 0.92921337 |
| Arih1 | 0.00544 | 0.65086894 |
| Slc25a21 | 0.00144 | -1.24613586 |
| Nhsl1 | 0.34962 | -0.56676613 |
| Nhsl1 | 0.14407 | -0.78343786 |
| Dennd1b | 0.00575 | -1.0542001 |
| Phospho2 | 0.51242 | -0.14887701 |
| Napg | 0.00144 | -1.30017714 |
| Napg | 0.00229 | -0.84580107 |
| Napg | 0.40422 | -0.49689394 |
| Acot12 | 0.26185 | -0.35637582 |
| Mettl10 | 0.00144 | 1.35009115 |
| Mettl10 | 0.00229 | 1.10753401 |
| Dcx | 0.17847 | -0.77697229 |
| Ei24 | 0.81927 | -0.04686792 |
| Ei24 | 0.28381 | 0.34303989 |
| Atp5g3 | 0.06668 | 0.42378603 |
| Plaa | 0.00843 | -1.23313049 |
| Plaa | 0.00144 | 3.39415113 |
| Manba | 0.38613 | 0.42546045 |
| Fam188a | 0.01605 | 0.74671491 |
| Hdc | 0.22981 | 0.43541282 |
| Paf1 | 0.47867 | 0.15513517 |
| Zcchc4 | 0.81091 | 0.11670912 |
| Pten | 0.02291 | 0.4486274 |
| Fam206a | 0.14687 | -0.2814633 |
| Ttc3 | 0.00144 | 1.29749484 |
| Plcb4 | 0.21759 | 0.22679937 |
| Plcb4 | 0.03149 | 1.97176218 |
| Ift81 | 0.37581 | 0.13778557 |
| Akr1c14 | 0.72836 | 0.20357391 |
| Rmnd1 | 0.0361 | 0.51602866 |
| Rmnd1 | 0.69023 | 0.19672684 |
| Nfyb | 0.24929 | -0.23268505 |
| Nfyb | 0.7496 | -0.06678871 |
| Lamb1 | 0.71903 | -0.17683824 |
| Slc38a4 | 0.00653 | -0.70761743 |
| Lrrc56 | 0.01663 | 0.90100276 |
| Brap | 0.04784 | -0.84803444 |
| Espn | 0.00144 | -1.27165604 |
| BC028528 | 0.52787 | -0.21871161 |
| Zfp709 | 0.00144 | 1.48427345 |
| Slc26a5 | 0.00144 | -1.31495282 |
| Blmh | 0.44725 | 0.41194986 |
| Phf3 | 0.40241 | 0.14823503 |
| Phf3 | 0.64501 | 0.11684518 |
| Phf3 | 0.00144 | 1.69661932 |
| Dpy19l4 | 0.07116 | -0.42344589 |
| Dnajb4 | 0.00144 | -1.27446889 |
| Sfpq | 0.02805 | 0.59303875 |
| Dgkd | 0.76623 | -0.08163956 |
| Cox18 | 0.26151 | 0.61797572 |
| Col6a5 | 0.00144 | -1.29049509 |
| Mterf3 | 0.00156 | 1.21425501 |
| Fbxw2 | 0.00293 | 0.76636729 |
| Lztr1 | 0.03887 | -0.43584564 |
| Nebl | 0.1867 | -0.62238034 |
| Abca3 | 0.30237 | 0.27692921 |
| Mkln1 | 0.12496 | 0.34371863 |
| Mkln1 | 0.25273 | -0.34288002 |
| E130311K13Rik | 0.00145 | -1.09170241 |
| Edrf1 | 0.03144 | 1.6360393 |
| Kynu | 0.00144 | -1.29864962 |
| Tbx15 | 0.00144 | -1.27165604 |
| Nceh1 | 0.21224 | 0.57664113 |
| Slc25a36 | 0.0541 | 0.58830914 |
| Cand1 | 0.87684 | -0.17029728 |
| Cct4 | 0.00438 | 1.14859065 |
| Cct4 | 0.01333 | 0.71062866 |
| Mrvi1 | 0.46647 | -0.39380688 |
| Tmem56 | 0.00145 | -1.22376446 |
| Nbr1 | 0.50369 | -0.35276315 |
| Nbr1 | 0.08913 | -0.38086238 |
| Smg1 | 0.09079 | 0.98232966 |
| Dusp22 | 0.00144 | -1.24900642 |
| Strc | 0.04937 | -1.34715888 |
| Cask | 0.9683 | 0.0224156 |
| Mapt | 0.0039 | -1.24028248 |
| Fam114a2 | 0.21207 | 0.29150848 |
| Zranb3 | 0.11125 | -0.75987879 |
| Ccne2 | 0.01826 | -0.84957969 |
| Il20rb | 0.00202 | -1.37517696 |
| Slc18a1 | 0.34575 | -0.89930715 |
| Nkiras1 | 0.75963 | 0.15141362 |
| Zkscan3 | 0.54168 | -0.24911596 |
| Stam2 | 0.08635 | -0.61674377 |
| Zfp300 | 0.00425 | -1.52681567 |
| Farsb | 0.71627 | -0.08139091 |
| Ptpn12 | 0.00144 | 1.51154361 |
| Sub1 | 0.09344 | -1.01784861 |
| Lins | 0.3332 | 0.55867372 |
| Cp | 0.58563 | 0.1152655 |
| Naa25 | 0.01347 | -1.01042984 |
| Gatm | 0.11985 | 0.60382368 |
| Loxhd1 | 0.0475 | -0.92312902 |
| Ppp1r8 | 0.93324 | -0.03077153 |
| Exoc6b | 0.00527 | 1.30093534 |
| Bfar | 0.01167 | 0.63837161 |
| Aff1 | 0.37573 | 0.34236989 |
| Stxbp4 | 0.96024 | -0.02009473 |
| Fam175b | 0.00309 | -0.79252292 |
| Asb1 | 0.00335 | -0.76661693 |
| Trhr | 0.00149 | -1.22582153 |
| Ago1 | 0.48944 | 0.59371699 |
| Cnot2 | 0.0878 | -0.3945723 |
| Cog4 | 0.06668 | 0.83585093 |
| Eif2s3x | 0.00266 | -0.89421218 |
| Nrxn3 | 0.0093 | -1.36709445 |
| Slc38a11 | 0.00609 | -1.5334902 |
| Sulf1 | 0.1668 | 1.0242059 |
| Rlf | 0.27502 | 0.22448168 |
| Gmeb2 | 0.33468 | -0.25305203 |
| Cers6 | 0.00144 | -1.20281695 |
| Rnasel | 0.39067 | 0.21856285 |
| Azi2 | 0.02006 | 0.51007737 |
| Idh3g | 0.03614 | 0.63753131 |
| Osbpl6 | 0.5566 | 0.24260363 |
| Sclt1 | 0.00144 | 2.23985847 |
| BC029214 | 0.95648 | -0.0143473 |
| Ms4a6b | 0.62901 | 0.11620779 |
| Sepsecs | 0.03616 | -0.84235323 |
| Zfp286 | 0.02271 | -1.09650221 |
| Slc43a3 | 0.40534 | 0.27616189 |
| Nckap1 | 0.00853 | -0.68980818 |
| Soat1 | 0.00144 | -1.20555712 |
| Soat1 | 0.00233 | -0.8722919 |
| Zmym1 | 0.54315 | -0.22924207 |
| Elmod2 | 0.00144 | 2.19940243 |
| Pnpla8 | 0.93339 | -0.01413877 |
| Cdc23 | 0.76623 | 0.12934265 |
| Casp8ap2 | 0.37072 | 0.3021765 |
| Mettl13 | 0.246 | -0.7967607 |
| Ifrd1 | 0.00144 | 1.16734373 |
| Lpin2 | 0.0578 | -0.76345841 |
| Slc26a2 | 0.11937 | -1.71268797 |
| Tnfrsf11a | 0.02473 | -1.02376733 |
| Cers2 | 0.04401 | -0.49528445 |
| Galnt7 | 0.88185 | 0.04086188 |
| Csmd1 | 0.00144 | -1.25927895 |
| Tfdp2 | 0.003 | -0.81543304 |
| Tfdp2 | 0.01967 | -0.65597439 |
| Lrrc18 | 0.03608 | -2.31764119 |
| Slc9a7 | 0.00144 | -1.26301121 |
| Cap1 | 0.75691 | 0.09499122 |
| Recql | 0.74745 | 0.0769398 |
| Lepr | 0.00929 | -1.0433256 |
| Lepr | 0.00144 | -1.26153594 |
| Ublcp1 | 0.5767 | 0.53555252 |
| D430041D05Rik | 0.00144 | -1.30253725 |
| Brd7 | 0.00191 | -1.2492394 |
| Cald1 | 0.20096 | -0.30621193 |
| Rbbp6 | 0.15071 | -0.26540813 |
| Sidt2 | 0.21735 | -0.41971026 |
| Cdc42bpa | 0.02054 | 0.93011073 |
| Mospd2 | 0.00144 | -1.31884045 |
| Zfand1 | 0.09828 | -0.32054509 |
| Rps9 | 0.68269 | 0.21797888 |
| Agl | 0.6566 | -0.10011508 |
| Slbp | 0.04913 | 0.70355497 |
| Slc40a1 | 0.19719 | -0.74444472 |
| Heyl | 0.00144 | -1.42919588 |
| Tmem234 | 0.60564 | 0.30407991 |
| Slc7a9 | 0.08277 | -0.99549506 |
| Ubtd2 | 0.0377 | -0.50306131 |
| Crebrf | 0.02109 | -0.89782899 |
| Nfya | 0.00144 | -1.27165604 |
| Slc35a2 | 0.01515 | -0.57529996 |
| Ezh2 | 0.1648 | 0.27693983 |
| Ezh2 | 0.11953 | -0.30697981 |
| Ezh2 | 0.03368 | 0.50911964 |
| Calcr | 0.00158 | -1.29082685 |
| Jph4 | 0.01566 | -0.98182861 |
| Vamp4 | 0.22282 | 0.45576288 |
| Fbxw10 | 0.00144 | -1.48564159 |
| Fam92a | 0.31713 | 0.16673393 |
| Postn | 0.00144 | 1.80592516 |
| Mga | 0.72688 | 0.10686206 |
| Mga | 0.13501 | 0.43428442 |
| Ppp1cb | 0.17806 | 0.43483149 |
| Rc3h2 | 0.00827 | -0.76520574 |
| Mknk1 | 0.27955 | -0.4695949 |
| Stau2 | 0.00612 | -0.68649811 |
| Dzip3 | 0.0184 | 0.61268622 |
| Rars2 | 0.03526 | -0.75555777 |
| Aldh1l2 | 0.67593 | -0.1533138 |
| Tmod4 | 0.07208 | 0.99671506 |
| Rnf145 | 0.14405 | -0.4913104 |
| Trnt1 | 0.16447 | -0.70676474 |
| Nck1 | 0.00144 | 1.25458431 |
| Mettl16 | 0.02808 | -0.65005199 |
| Mettl16 | 0.10214 | -1.09513407 |
| Xrn1 | 0.0582 | -1.0035899 |
| 2310035C23Rik | 0.89616 | -0.03298673 |
| Slco5a1 | 0.07321 | -1.2208022 |
| Mbd2 | 0.00646 | 0.68888629 |
| Tbx22 | 0.00145 | -1.26219879 |
| Top2b | 0.00144 | 1.64838257 |
| Mdm4 | 0.16012 | -0.43990748 |
| Mdm4 | 0.04763 | -0.54620415 |
| Sdha | 0.20726 | -0.32494989 |
| Hp1bp3 | 0.00147 | -1.37448719 |
| Tiam1 | 0.01123 | -0.82392073 |
| Hspa5 | 0.3384 | 0.1795435 |
| Hspa5 | 0.10283 | -0.37855328 |
| Cdc16 | 0.04311 | 0.50192852 |
| Gadd45a | 0.00144 | -1.31495282 |
| Lsm3 | 0.35165 | -0.85933135 |
| Insig2 | 0.12582 | 0.55651934 |
| Dhrs7c | 0.47647 | -0.46448365 |
| Sik2 | 0.00144 | -1.27789725 |
| Papolg | 0.08813 | 0.45397247 |
| Chek1 | 0.32672 | -0.36688373 |
| 2810474O19Rik | 0.00144 | 2.91270411 |
| Msh5 | 0.18206 | -0.8860457 |
| Immt | 0.94874 | -0.0293416 |
| Ccdc39 | 0.00144 | -1.29051721 |
| Pigk | 0.00145 | -1.25646236 |
| Efr3a | 0.00179 | -1.13056285 |
| Setd2 | 0.12125 | -0.76578889 |
| Cobll1 | 0.00144 | -1.60732145 |
| Esp24 | 0.00144 | -1.28898079 |
| Tbc1d14 | 0.14997 | -0.73570468 |
| Tmem110 | 0.00753 | -0.58898116 |
| Zfand5 | 0.02479 | -0.43649718 |
| Thoc6 | 0.00544 | -0.94135724 |
| Vta1 | 0.68136 | -0.06557718 |
| Il33 | 0.02448 | -0.8493692 |
| Thsd7a | 0.274 | -0.21973159 |
| Adgrb3 | 0.34637 | -0.40391506 |
| Asic5 | 0.07203 | -1.0560271 |
| Scmh1 | 0.02327 | -0.63149799 |
| Hddc2 | 0.42648 | 0.29843421 |
| Pdcd10 | 0.45501 | -0.12812317 |
| Megf11 | 0.00144 | -1.27165604 |
| Megf11 | 0.00144 | -1.29049509 |
| Tmem62 | 0.21817 | 0.31402825 |
| Gtdc1 | 0.4725 | -0.25032114 |
| Pum2 | 0.0257 | -0.46503201 |
| Pum2 | 0.00484 | -0.88333056 |
| Drg2 | 0.01474 | -0.77677312 |
| Cldn18 | 0.01036 | -1.63069312 |
| Erlec1 | 0.10676 | 0.84590341 |
| Fam175a | 0.02841 | 1.24013242 |
| Ddx19a | 0.60264 | 0.08724558 |
| Adprm | 0.01362 | 1.01869745 |
| Csnk1d | 0.07382 | -0.86014901 |
| Mtm1 | 0.03125 | 2.03942176 |
| Myo9a | 0.32393 | 0.2274291 |
| Prc1 | 0.06793 | -0.72407839 |
| Znrf2 | 0.41401 | -0.27060486 |
| Spata6 | 0.00202 | 1.25156446 |
| Gcsam | 0.00144 | -1.28116048 |
| Dynlt3 | 0.13046 | -0.94822182 |
| Plk4 | 0.0063 | 0.97653689 |
| Adam17 | 0.0129 | -0.6586821 |
| Adam17 | 0.10703 | 0.45316837 |
| Syncrip | 0.04276 | 0.72432787 |
| Pfkfb1 | 0.0187 | 0.57309715 |
| Prrc2c | 0.95057 | 0.02011189 |
| Setd6 | 0.11828 | 0.26414657 |
| Golga1 | 0.93782 | 0.01624442 |
| Rnf8 | 0.00144 | -1.41937871 |
| Nup62cl | 0.00144 | -1.26560808 |
| Acad9 | 0.31879 | -0.16118851 |
| Pikfyve | 0.01322 | 1.17996992 |
| Zcchc11 | 0.0723 | 0.40247032 |
| Zcchc11 | 0.00316 | 1.03791546 |
| Mdga2 | 0.00144 | -1.29864962 |
| Glud1 | 0.04 | 0.59256662 |
| Hnrnpl | 0.51816 | -0.24686798 |
| Zfp322a | 0.95726 | 0.0397133 |
| Col1a2 | 0.00144 | -1.29085005 |
| Col1a2 | 0.1195 | -0.74631914 |
| Tbc1d10b | 0.00144 | -1.29746368 |
| Trpm7 | 0.56775 | -0.15042362 |
| Eml6 | 0.78959 | 0.08418849 |
| Ncald | 0.0841 | -0.37853645 |
| Mfn1 | 0.98703 | -0.00469522 |
| Ptprd | 0.04871 | -1.42760943 |
| Ptprd | 0.00227 | -1.12522109 |
| Ddx58 | 0.28431 | -0.21348943 |
| Smpx | 0.26879 | -0.20605579 |
| Smpx | 0.22779 | -0.23215989 |
| Gm4894 | 0.00144 | -1.31495282 |
| Rars | 0.03678 | 0.49732609 |
| Skil | 0.04453 | 0.43059533 |
| Prrc2b | 0.00144 | -1.48834485 |
| Swt1 | 0.04786 | 0.66941936 |
| Med7 | 0.24811 | 0.21705021 |
| Cacfd1 | 0.93846 | -0.03331948 |
| Ranbp17 | 0.00314 | -1.14703327 |
| Tro | 0.04198 | -1.03175467 |
| Phf20 | 0.37886 | 0.23992827 |
| Cul1 | 0.5772 | 0.35234058 |
| Cul1 | 0.87421 | -0.03638021 |
| Nrd1 | 0.01388 | 0.66084053 |
| Trmt1 | 0.37695 | 0.22900989 |
| 1700016L04Rik | 0.00197 | -1.28998455 |
| Kbtbd2 | 0.8362 | 0.03943628 |
| Kbtbd2 | 0.02435 | -0.54405288 |
| Chchd3 | 0.21674 | 0.32657945 |
| Usp6nl | 0.47818 | 0.23699551 |
| Slc8b1 | 0.87854 | 0.08368725 |
| Hspa4l | 0.03019 | 0.62256193 |
| Dixdc1 | 0.01819 | -1.35378788 |
| Zc3h7a | 0.01288 | 0.91494862 |
| Gpd2 | 0.07852 | 0.45466572 |
| Naalad2 | 0.36893 | -0.57017489 |
| Dcun1d1 | 0.88039 | -0.08099225 |
| Mdm2 | 0.03067 | -0.53087921 |
| Csnk1g1 | 0.94193 | 0.05287606 |
| Hspa4 | 0.2638 | 0.24162689 |
| Adh5 | 0.0484 | 0.65654176 |
| Herc1 | 0.0231 | 1.81226344 |
| Camsap1 | 0.00144 | -1.56939375 |
| Dlat | 0.25402 | 0.38066224 |
| Itga4 | 0.01911 | -0.97469996 |
| Sycp2 | 0.26357 | -0.55965333 |
| 5430419D17Rik | 0.00144 | -1.28116048 |
| Rbm6 | 0.00158 | 2.31966534 |
| Ndufc1 | 0.00609 | -0.78701108 |
| Hiat1 | 0.00144 | -1.29085005 |
| Hiat1 | 0.0096 | -0.97150126 |
| Cdh13 | 0.26958 | -0.77971246 |
| Clasp2 | 0.03608 | -0.51956977 |
| Osbpl10 | 0.45745 | -0.23488068 |
| Hgd | 0.00149 | -1.22582153 |
| Plxnc1 | 0.00145 | -1.2388121 |
| Bbs9 | 0.22844 | -0.40097017 |
| Kdm5a | 0.43539 | -0.24748371 |
| Eps15 | 0.12303 | -0.30638621 |
| Eps15 | 0.00819 | 2.19278121 |
| Pi4ka | 0.0243 | -0.55768451 |
| Rab14 | 0.01751 | -0.7396941 |
| Hrasls | 0.09862 | -2.20502706 |
| Naa35 | 0.82131 | 0.04378532 |
| St3gal6 | 0.1214 | 0.2777079 |
| St3gal6 | 0.71356 | 0.0728454 |
| Fbxo11 | 0.65124 | -0.18974071 |
| Gfm2 | 0.02878 | -0.53996893 |
| Tnpo3 | 0.00144 | -1.20677601 |
| Tnpo3 | 0.01049 | 1.35471364 |
| Ect2 | 0.4903 | -0.30070585 |
| Usp29 | 0.02338 | -2.19367074 |
| Rps29 | 0.71898 | 0.19191888 |
| Tlr4 | 0.27655 | 0.42701504 |
| Zfp346 | 0.16597 | -0.41726069 |
| Kcnn2 | 0.20282 | 0.23239613 |
| BC049635 | 0.01809 | -0.95081427 |
| Ctc1 | 0.03998 | 0.7998334 |
| Fyttd1 | 0.11167 | 0.26354803 |
| Alox12e | 0.00144 | -1.26428512 |
| Rbl1 | 0.59112 | -0.1443198 |
| Nup43 | 0.05943 | -0.96587093 |
| Nup43 | 0.26357 | -0.33333333 |
| Nup43 | 0.01273 | 0.72926884 |
| Fzd3 | 0.35987 | -0.2711749 |
| Zfp936 | 0.00144 | -1.28116048 |
| Trmu | 0.86107 | -0.08009005 |
| Cep295 | 0.00144 | 1.53564542 |
| Cep295 | 0.491 | -0.70908795 |
| Ppp2r2d | 0.01643 | 0.73430232 |
| Htt | 0.07661 | 0.42665709 |
| Ubr4 | 0.17483 | -0.38505108 |
| Dscr3 | 0.41869 | -0.4691117 |
| Cops8 | 0.00144 | -1.28898079 |
| Sema6a | 0.5321 | 0.38453507 |
| Accsl | 0.00193 | -1.16824959 |
| Nsf | 0.01458 | -1.00464763 |
| Pqlc1 | 0.01215 | -0.99618761 |
| Pqlc1 | 0.63764 | -0.17481474 |
| Dnajc8 | 0.00745 | -0.88530624 |
| Tox4 | 0.00195 | -1.35679644 |
| Orc4 | 0.00677 | -0.61241947 |
| Orc4 | 0.00144 | 1.13477257 |
| Lactb2 | 0.01956 | 0.65495576 |
| Msh3 | 0.02238 | -0.55074873 |
| Cyp7a1 | 0.01882 | -1.09849823 |
| Zcchc7 | 0.87041 | -0.04910178 |
| Rbm28 | 0.02531 | -0.54029779 |
| Nanp | 0.36169 | -0.2850346 |
| Hibch | 0.00144 | 2.06339088 |
| Cfap44 | 0.00144 | -1.26560808 |
| Dpy19l1 | 0.00194 | -1.08169839 |
| Aass | 0.69985 | -0.20565763 |
| Arhgap29 | 0.15824 | 0.56188772 |
| Pknox2 | 0.0049 | -1.11996532 |
| Polr2b | 0.03557 | -0.93227475 |
| Col24a1 | 0.00144 | -1.29864962 |
| Dnase1l1 | 0.00456 | 1.68109041 |
| Dnase1l1 | 0.41159 | -0.24158188 |
| Ndufaf6 | 0.08093 | -0.53251008 |
| Ndufaf6 | 0.0033 | -0.79073286 |
| Sirt1 | 0.51071 | -0.4325847 |
| Mtus1 | 0.20719 | 0.35525845 |
| Zfp454 | 0.04166 | -0.71716998 |
| Hc | 0.01903 | -1.16519364 |
| Hps5 | 0.02374 | -0.60619472 |
| Hps5 | 0.00145 | -1.26219879 |
| Arr3 | 0.00144 | -1.28116048 |
| Arr3 | 0.11839 | -2.40179533 |
| Hdlbp | 0.24989 | 0.51077057 |
| Ogg1 | 0.02785 | -0.99241763 |
| Hars2 | 0.00447 | -0.89476041 |
| Zfp37 | 0.05902 | 0.58063315 |
| Aldh1a3 | 0.00325 | -1.15314387 |
| Arhgef18 | 0.53805 | 0.29783719 |
| Rpgr | 0.14225 | -0.540377 |
| Phka2 | 0.00144 | -1.0288548 |
| Cipc | 0.55271 | -0.1838852 |
| Racgap1 | 0.00144 | -1.52591528 |
| Ipmk | 0.06113 | -2.16176754 |
| Efemp1 | 0.89482 | 0.08753696 |
| Fignl1 | 0.00144 | -1.29085005 |
| Atp6v0d2 | 0.00144 | -1.20555712 |
| Car14 | 0.76463 | 0.11100688 |
| Pfkp | 0.00631 | 1.0071035 |
| Cep57 | 0.92012 | -0.03389265 |
| Vmp1 | 0.05449 | 0.77248568 |
| Abcc5 | 0.01864 | -0.7412152 |
| Abcc5 | 0.2446 | 0.30034099 |
| Phactr1 | 0.00189 | -0.83872029 |
| Whsc1l1 | 0.99994 | -0.00003949 |
| Alkbh3 | 0.03418 | -0.43724279 |
| Clcc1 | 0.0133 | 1.37155967 |
| Mkks | 0.1313 | 0.3772573 |
| Uqcrb | 0.02412 | 1.60353914 |
| Galnt1 | 0.28742 | -0.25015439 |
| Wdr3 | 0.08778 | -0.70463592 |
| Rufy2 | 0.69237 | -0.10313727 |
| Dcaf11 | 0.82127 | 0.10281629 |
| Amot | 0.00569 | -0.8690368 |
| Pir | 0.17012 | 0.47240498 |
| Ankrd34b | 0.00144 | -1.26560808 |
| Kbtbd4 | 0.9277 | -0.01956458 |
| Tsga10 | 0.03662 | -0.63765971 |
| Clec9a | 0.26357 | -0.56946548 |
| Asrgl1 | 0.72849 | -0.20072393 |
| Syn3 | 0.01155 | -1.07332189 |
| Tlk1 | 0.03895 | -0.40242176 |
| Oprk1 | 0.66092 | -0.39449855 |
| Tsc1 | 0.16247 | -0.42045186 |
| Shcbp1l | 0.01696 | -1.19646947 |
| Dio1 | 0.00151 | 1.82146102 |
| Ccdc62 | 0.66972 | 0.20316767 |
| Zbtb33 | 0.12734 | 0.37113628 |
| 4930447C04Rik | 0.00631 | -1.58700642 |
| Kdm5d | 0.64981 | 0.28767738 |
| Gm3259 | 0.00144 | -1.29085005 |
| Gpbp1 | 0.33995 | -0.25441986 |
| Pdk1 | 0.09223 | 1.16200877 |
| Pdk1 | 0.01277 | -0.84709722 |
| Tax1bp1 | 0.31605 | 0.1964302 |
| Map2k4 | 0.00144 | -1.23017224 |
| Cdkal1 | 0.08852 | -0.44145118 |
| Wdr4 | 0.00144 | -1.28116048 |
| Samsn1 | 0.00144 | -1.27165604 |
| Samsn1 | 0.31101 | -0.69963192 |
| Zfp949 | 0.53168 | -0.14273846 |
| D330045A20Rik | 0.02369 | -1.06863477 |
| Phykpl | 0.66085 | -0.2182849 |
| Rftn1 | 0.08603 | -0.71649875 |
| Tbc1d9 | 0.00144 | -1.26301121 |
| Aox3 | 0.00144 | -1.53889045 |
| Snx1 | 0.77049 | -0.05441629 |
| Man1b1 | 0.29128 | -0.64828615 |
| Bmpr1a | 0.59063 | -0.12649663 |
| Lingo2 | 0.00144 | -1.28116048 |
| Med23 | 0.01388 | -1.16456105 |
| Spink10 | 0.00144 | -1.20555712 |
| Spink10 | 0.00144 | -1.27730451 |
| Zmiz1 | 0.0313 | -0.80079515 |
| Secisbp2 | 0.00359 | 0.80421846 |
| Zeb1 | 0.01801 | 0.67460048 |
| Glce | 0.03274 | -0.88598411 |
| Srrm2 | 0.13373 | 0.34242576 |
| Pld1 | 0.86678 | -0.05255617 |
| Zbtb25 | 0.3006 | 0.24534738 |
| Mllt10 | 0.86389 | -0.0318153 |
| Mllt10 | 0.19394 | -0.27638986 |
| Mllt10 | 0.61295 | -0.08633332 |
| Oxnad1 | 0.14814 | 0.56506186 |
| Acad8 | 0.0049 | -1.37741422 |
| Lrrc9 | 0.33907 | 0.35229653 |
| Epha7 | 0.05152 | -1.26977126 |
| Tubgcp3 | 0.00144 | -1.28898079 |
| Orc3 | 0.85555 | -0.04575827 |
| Gm13093 | 0.00144 | -1.28898079 |
| Ube4bos3 | 0.00314 | -1.14703327 |
| Rbbp4 | 0.06252 | -0.34424229 |
| LOC102641089 | 0.35351 | 0.46462604 |
| Gm36547 | 0.00144 | -1.26428512 |
| Gm33039 | 0.02853 | -2.49704683 |
| Chm | 0.21879 | -1.01447765 |
| 5330434G04Rik | 0.00144 | -1.30017714 |
| Gm30993 | 0.01308 | -1.61579653 |
| Gm35107 | 0.0047 | -0.95809404 |
| Gm19956 | 0.01664 | -1.91302266 |
| Gm33951 | 0.30421 | 0.57112484 |
| Rab11fip2 | 0.00795 | 1.04626991 |
| Atrnl1 | 0.68252 | 0.11091734 |
| Gm22219 | 0.00144 | -1.27730451 |
| Gm4146 | 0.00144 | -1.30713006 |
| Gm31706 | 0.00144 | -1.28116048 |
| Gm31018 | 0.00144 | -1.29746368 |
| LOC105246372 | 0.05427 | 0.5245092 |
| Gm36232 | 0.00144 | -1.29085005 |
| Gm32465 | 0.00144 | -1.29085005 |
| Gm31704 | 0.04536 | -0.60034176 |
| Gm34265 | 0.00144 | -1.37964577 |
| Gm36542 | 0.03396 | -0.83557911 |
| Gm29938 | 0.00158 | -1.2446827 |
| Camkmt | 0.88096 | 0.13314299 |
| Gm31495 | 0.00144 | -1.26560808 |
| Gm31431 | 0.00144 | -1.25118919 |
| Gm30636 | 0.15555 | -0.43895829 |
| Gm36903 | 0.00852 | -1.47397797 |
| LOC105242494 | 0.00144 | -1.31884045 |
| Gm36902 | 0.01404 | -1.23984951 |
| Efcab1 | 0.0156 | -1.08617297 |
| Dzip3 | 0.07006 | -0.65449137 |
| Gm32677 | 0.00144 | -1.30713006 |
| Gm32192 | 0.00144 | -1.86165417 |
| Gm30609 | 0.10905 | -2.72705791 |
| 1700025B11Rik | 0.02871 | -1.07159308 |
| Gm34562 | 0.00482 | -0.95647196 |
| Gm34503 | 0.00144 | -1.26560808 |
| Gm34336 | 0.00144 | -1.30017714 |
| Hoxc13 | 0.00145 | -1.26219879 |
| Smc1b | 0.00145 | -1.2388121 |
| 4930570B17Rik | 0.0029 | -1.98093908 |
| Ubr5 | 0.00342 | -0.98368073 |
| Gm30439 | 0.00144 | -1.29085005 |
| Gm34361 | 0.01992 | -1.77880922 |
| Gm32913 | 0.00144 | -1.28898079 |
| Gm32729 | 0.00144 | -1.33059316 |
| Gm32498 | 0.01161 | -1.29135307 |
| Gm32498 | 0.00144 | -1.22098834 |
| Gm31980 | 0.01578 | -0.88590964 |
| LOC102640957 | 0.00145 | -1.2388121 |
| Gm35678 | 0.41204 | -0.2325834 |
| Gm16573 | 0.04096 | -0.99198802 |
| 4930538L07Rik | 0.00158 | -1.2446827 |
| Gm34360 | 0.00144 | -2.41805106 |
| Gm35164 | 0.00145 | -1.24191372 |
| Gm32645 | 0.01904 | -0.91186145 |
| Naa16 | 0.00689 | 1.30410096 |
| Ankrd28 | 0.03204 | -0.88925806 |
| 1700062C10Rik | 0.37057 | -0.51765977 |
| Gm31222 | 0.00144 | -1.53131709 |
| Gm2977 | 0.08648 | 0.83288281 |
| Gm15327 | 0.44094 | 0.12528969 |
| Gm32450 | 0.00144 | -1.20555712 |
| Gm36921 | 0.00144 | -1.29746368 |
| Slc30a5 | 0.30814 | -0.22615123 |
| Gm35245 | 0.00145 | -1.28488688 |
| Gm34196 | 0.00144 | -1.31014844 |
| Gm33274 | 0.00403 | -1.59861976 |
| Gm33073 | 0.00144 | -1.24613586 |
| Gm3772 | 0.0023 | -0.98517932 |
| Gm33787 | 0.02281 | -1.41869388 |
| Gm34558 | 0.00144 | -1.26934956 |
| Gm34354 | 0.00144 | -1.31884045 |
| Tmem161b | 0.95196 | -0.03646115 |
| Gm34991 | 0.00144 | -1.33059316 |
| Gm30240 | 0.77704 | -0.13995501 |
| Gm31482 | 0.04833 | 0.84803262 |
| Gm34667 | 0.10575 | -2.75464996 |
| Gm31884 | 0.02612 | -1.90598351 |
| Gm35989 | 0.45967 | 0.20227669 |
| Gm29530 | 0.00144 | -1.29085005 |
| Gm34666 | 0.02728 | -0.8983632 |
| Gm34552 | 0.00184 | 2.03957236 |
| Gm32151 | 0.00149 | -1.22582153 |
| Gm31447 | 0.30787 | -0.55785214 |
| Gm34745 | 0.00144 | -1.31495282 |
| Gm34581 | 0.31449 | -0.68392751 |
| Ccdc176 | 0.14976 | 0.61868306 |
| Smek1 | 0.09959 | 0.77723106 |
| Gm36886 | 0.00144 | -1.28116048 |
| Gm34489 | 0.41842 | -0.97056841 |
| Gm35828 | 0.02203 | -0.972494 |
| Gm35828 | 0.01754 | -1.48964623 |
| Gm11494 | 0.00654 | -1.20692419 |
| Gm34726 | 0.02426 | -0.80638962 |
| 9230020A06Rik | 0.00144 | -1.33632959 |
| Gm32708 | 0.00144 | -1.28116048 |
| Arhgef15 | 0.00145 | -1.22376446 |
| Tom1l2 | 0.30459 | -1.36825357 |
| Gm35282 | 0.00489 | -0.99928809 |
| A230004M16Rik | 0.02024 | -1.48111429 |
| Aatf | 0.00144 | -1.25281511 |
| Gm34185 | 0.59498 | 0.26648324 |
| Gm34075 | 0.00152 | -1.25878908 |
| Gm31505 | 0.00144 | -1.25118919 |
| Gm30423 | 0.00144 | -1.27730451 |
| Mtif2 | 0.00144 | 2.75776897 |
| Gm33544 | 0.00144 | -1.41534266 |
| Gm36719 | 0.00144 | -1.25118919 |
| Gm15723 | 0.00144 | -1.22925777 |
| LOC105245279 | 0.00144 | -1.26560808 |
| Jmjd1c | 0.02388 | -1.00299626 |
| Gm30618 | 0.00145 | -1.2388121 |
| Gm36313 | 0.87729 | -0.05685581 |
| Gm33945 | 0.56079 | -0.2529973 |
| Gm32926 | 0.6444 | -0.19869745 |
| Gm31329 | 0.00145 | -1.22518835 |
| Rtn4ip1 | 0.00197 | -0.89005378 |
| Gm36367 | 0.00144 | -1.26560808 |
| Gm31233 | 0.03505 | -0.83757347 |
| Gm31587 | 0.08177 | -2.47056965 |
| Clpx | 0.04922 | 0.55375778 |
| Xrn1 | 0.20638 | 0.36784687 |
| Gm32359 | 0.00144 | -1.29864962 |
| Gm16118 | 0.80825 | 0.14300666 |
| Gm35145 | 0.31899 | -0.54534556 |
| Gm36670 | 0.00299 | 1.07627929 |
| Gm36243 | 0.00144 | -1.43031347 |
| Gm34846 | 0.00144 | -1.27165604 |
| Gm35368 | 0.89191 | -0.07783004 |
| Gm34533 | 0.00145 | -1.2388121 |
| Gm33281 | 0.00144 | -1.27165604 |
| Gm32735 | 0.06872 | -1.33701184 |
| Gm7562 | 0.00144 | -1.26560808 |
| Gm32315 | 0.00144 | -1.26560808 |
| Gm32707 | 0.00641 | -1.45195848 |
| LOC102638993 | 0.00144 | -1.31495282 |
| Gm31740 | 0.67898 | -0.18840876 |
| Gm35209 | 0.00144 | -1.26560808 |
| Gm32963 | 0.00232 | -1.15063496 |
| Gm33190 | 0.00145 | -1.24191372 |
| Gm33989 | 0.11843 | -0.62762709 |
| Gm33067 | 0.00144 | -1.31884045 |
| Gm35768 | 0.00156 | -1.59761845 |
| Gm34416 | 0.00537 | -1.5030208 |
| Gm35824 | 0.00144 | -1.28898079 |
| Gm31661 | 0.02233 | -1.74494446 |
| 4930533I22Rik | 0.00144 | -1.61432628 |
| Gm31103 | 0.05131 | 0.64272657 |
| Gm35674 | 0.00144 | -1.28116048 |
| Gm33788 | 0.00589 | -1.11030563 |
| Gm35929 | 0.00144 | -1.28898079 |
| Gm36841 | 0.00144 | -1.29085005 |
| Gm13790 | 0.00144 | -1.42082928 |
| Gm36481 | 0.00362 | -1.41766025 |
| Mthfd2l | 0.00144 | -1.20555712 |
| Gm36814 | 0.00144 | -1.31518407 |
| Gm35762 | 0.00144 | -1.28898079 |
| Gm36665 | 0.00591 | -1.60036531 |
| Gm35848 | 0.96718 | -0.072637 |
| Gm31752 | 0.03396 | -0.9889904 |
| Gm31752 | 0.00144 | -1.30713006 |
| 1700003C15Rik | 0.00145 | -1.2388121 |
| Gm32807 | 0.00144 | -1.27165604 |
| Gm32117 | 0.68317 | 0.16416149 |
| Gm31682 | 0.00149 | -1.32850014 |
| Gm35137 | 0.00144 | -1.29085005 |
| Mup-ps16 | 0.00144 | -1.26428512 |
| Gm35578 | 0.01709 | -0.99496437 |
| Gm32955 | 0.05873 | -2.30737044 |
| Gm11820 | 0.00144 | -1.22925777 |
| Gm33759 | 0.00144 | -1.26934956 |
| Gm29836 | 0.00144 | -1.21743532 |
| Gm35555 | 0.00144 | 3.14892579 |
| Gm35555 | 0.00144 | 2.59882328 |
| 1700006H20Rik | 0.00149 | -1.22582153 |
| Gm30147 | 0.00144 | -1.27165604 |
| Gm35787 | 0.00415 | -1.06195552 |
| Gm35787 | 0.00144 | -1.26934956 |
| Gm35237 | 0.00144 | -1.26934956 |
| Gm36916 | 0.00144 | -1.30713006 |
| 4930459I23Rik | 0.00145 | -1.24191372 |
| Gm36010 | 0.00144 | -1.20555712 |
| Gm35699 | 0.00144 | -1.28116048 |
| Gm31879 | 0.00144 | -1.27699683 |
| Gm36069 | 0.26516 | -0.46955944 |
| Gm34048 | 0.00145 | -1.2388121 |
| Gm31415 | 0.00144 | -1.28116048 |
| Gm29959 | 0.00163 | -1.11663244 |
| Gm35425 | 0.00144 | -1.30713006 |
| Gm30340 | 0.00144 | -1.24613586 |
| LOC102636393 | 0.00144 | -1.27165604 |
| Gm29732 | 0.99197 | 0.00582198 |
| Gm30017 | 0.00218 | -1.18878227 |
| Gm29859 | 0.00145 | -1.22518835 |
| Gm36254 | 0.00144 | -1.28898079 |
| Gm35719 | 0.00144 | -1.31495282 |
| Gm34344 | 0.00144 | -1.26560808 |
| 4930442J19Rik | 0.01105 | -1.04591093 |
| Gm33648 | 0.14617 | -2.5237189 |
| Gm31993 | 0.00145 | -1.2388121 |
| Gm35981 | 0.11931 | -0.48768813 |
| Chd6 | 0.26138 | -0.44641081 |
| Rmdn3 | 0.22908 | -0.22528489 |
| 6430550D23Rik | 0.2002 | 0.32930906 |
| Gm32232 | 0.40719 | -0.15003534 |
| Gm13470 | 0.00149 | -1.19348234 |
| Gm30848 | 0.00158 | -1.54560964 |
| Gm13562 | 0.03437 | -0.47888436 |
| Gm34769 | 0.00144 | -1.31884045 |
| Lrp1b | 0.00144 | -1.33632959 |
| Gm13383 | 0.0849 | -0.88130859 |
| Gm32334 | 0.08597 | -1.9038387 |
| Gm36561 | 0.00166 | -0.95616974 |
| Gm36360 | 0.1907 | -1.6092223 |
| Gm36196 | 0.00144 | -1.33632959 |
| Gm35473 | 0.00741 | -2.70230897 |
| Gm34421 | 0.00144 | -1.31495282 |
| Hnrnpu | 0.00234 | 3.24715779 |
| Gm30521 | 0.00315 | -1.43219214 |
| Gm34306 | 0.00144 | -1.28898079 |
| Gm35562 | 0.00151 | -1.17792995 |
| Gm35243 | 0.0027 | -1.41114265 |
| Gm34584 | 0.05277 | -0.96916139 |
| Gm28382 | 0.05949 | -0.98728829 |
| Gm32879 | 0.00144 | -1.28898079 |
| Usp40 | 0.01038 | -0.7330336 |
| Gm33152 | 0.00144 | -1.38646478 |
| Gm31254 | 0.03947 | -0.97449752 |
| Gm31137 | 0.26996 | -2.13031721 |
| LOC105247484 | 0.16526 | -0.79972114 |
| Gm36678 | 0.01081 | -1.74293956 |
| Gm36678 | 0.01072 | -1.05977457 |
| Gm35935 | 0.00144 | -1.52265203 |
| Gm34596 | 0.07874 | -0.83038016 |
| 4930532G15Rik | 0.40931 | -0.60991472 |
| Gm31841 | 0.00242 | -2.55480918 |
| Gm34786 | 0.29003 | -0.99727924 |
| Gm34786 | 0.36604 | -0.90291174 |
| Gm34786 | 0.26388 | -1.04589379 |
| Ptcd3 | 0.00398 | 1.12993713 |
| LOC102641953 | 0.00144 | -1.28116048 |
| Gm15230 | 0.00144 | -1.26560808 |
| 4930555B12Rik | 0.00144 | -1.29085005 |
| Gm34464 | 0.00144 | -1.20555712 |
| Gm32262 | 0.6608 | -0.17428026 |
| Gm14902 | 0.79026 | -0.09450442 |
| Gm35587 | 0.14197 | -0.94252271 |
| Gm38438 | 0.00144 | -1.28116048 |
| Gm34665 | 0.84872 | -0.17421549 |
| Gm33339 | 0.00149 | -1.25408 |
| Gm33733 | 0.116 | -2.91695213 |
| LOC102641533 | 0.80806 | -0.12210601 |
| Gm32960 | 0.00155 | -1.27627822 |
| Gm31417 | 0.14171 | -0.80293518 |
| Gm29946 | 0.00145 | -1.22193674 |
| Gm36774 | 0.12743 | -3.07503299 |
| Gm35268 | 0.04964 | 0.9429529 |
| Gm38581 | 0.00145 | -1.26219879 |
| Gm33950 | 0.00763 | 1.15554884 |
| Gm31441 | 0.00144 | -1.28898079 |
| Gm31331 | 0.00144 | -1.28898079 |
| 1700095A13Rik | 0.00145 | -1.22376446 |
| LOC102634716 | 0.9366 | 0.02379488 |
| Gm32022 | 0.02964 | -0.93601623 |
| Gm30190 | 0.00158 | -1.22364055 |
| Gm36598 | 0.01355 | -1.646833 |
| Gm36368 | 0.1837 | -0.53006638 |
| Gm35778 | 0.00144 | -1.26560808 |
| Gm35778 | 0.20533 | -0.24154128 |
| LOC102641109 | 0.00144 | -1.36658832 |
| Gm34345 | 0.00654 | -1.29746368 |
| Gm32339 | 0.56526 | -0.32575997 |
| Gm32288 | 0.00368 | -1.10379914 |
| Gm31907 | 0.00144 | -1.20555712 |
| Gm30961 | 0.00144 | -1.29051721 |
| Gm36204 | 0.50623 | 0.28967253 |
| Gm36091 | 0.00208 | -1.42519669 |
| Gm34182 | 0.01241 | -1.74165546 |
| Gm33674 | 0.00182 | -1.61224351 |
| Gm33674 | 0.00144 | -1.30713006 |
| Gm33437 | 0.13402 | -0.43399099 |
| Gm33228 | 0.00144 | -1.29085005 |
| Gm36802 | 0.00144 | -1.25118919 |
| Gm32512 | 0.00144 | -1.31518407 |
| Gm34211 | 0.00144 | -1.30713006 |
| Gm33373 | 0.00144 | -1.26560808 |
| Gm31818 | 0.00193 | -1.24713717 |
| Gm31084 | 0.00328 | -1.10633687 |
| Gm36594 | 0.00145 | -1.27532353 |
| Gm36252 | 0.00167 | -1.22948903 |
| Gm35550 | 0.00145 | -1.22518835 |
| Gm30958 | 0.00144 | -1.24466058 |
| LOC102642231 | 0.18167 | -0.70192983 |
| Gm33802 | 0.00144 | -1.70851029 |
| Gm36199 | 0.69524 | -0.10692824 |
| Gm35716 | 0.01236 | -1.27794628 |
| Gm38551 | 0.02362 | -1.468939 |
| Gm34208 | 0.00149 | -1.22582153 |
| Gm31175 | 0.02551 | -1.41140623 |
| Gm29880 | 0.01306 | -0.83121578 |
| A730009L09Rik | 0.0156 | -1.08617297 |
| 4930570E03Rik | 0.00144 | -1.29746368 |
| Gm31322 | 0.00144 | 2.12833371 |
| Gm30569 | 0.01942 | -1.77208326 |
| Gm41475 | 0.00144 | -1.41443942 |
| Gm4633 | 0.00144 | -1.30017714 |
| LOC102641321 | 0.00149 | -1.2703832 |
| Gm33696 | 0.00144 | -1.30713006 |
| Gm32817 | 0.50707 | -0.14622435 |
| Gm15738 | 0.10656 | 0.69199003 |
| LOC105242421 | 0.00317 | -1.11935153 |
| Gm36824 | 0.04482 | -2.18737173 |
| Gm36195 | 0.18132 | -0.75456021 |
| Gm34447 | 0.01497 | -0.51867854 |
| Gm35933 | 0.00144 | -1.29049509 |
| Gm28068 | 0.21776 | -0.25771137 |
| Gm33174 | 0.29628 | -0.6456673 |
| Gm36677 | 0.03937 | -1.11262321 |
| Gm34678 | 0.00144 | -1.20555712 |
| 1100001I12Rik | 0.00152 | -1.25878908 |
| Gm34724 | 0.00145 | -1.2388121 |
| Gm38594 | 0.92705 | 0.06421503 |
| Gm32011 | 0.00145 | -1.26219879 |
| Gm31953 | 0.01527 | -1.95332318 |
| LOC102642852 | 0.00819 | -1.71250117 |
| Gm31133 | 0.00145 | -1.24191372 |
| LOC102632231 | 0.15199 | -0.50022822 |
| 4930404K13Rik | 0.00554 | -1.25162917 |
| Gm30216 | 0.01079 | -0.97080959 |
| D130079A08Rik | 0.00504 | -1.53524214 |
| Gm35362 | 0.00145 | -1.2388121 |
| Gm35116 | 0.00144 | -1.30017714 |
| Gm33880 | 0.00144 | -1.27165604 |
| Gm33450 | 0.00144 | -1.31518407 |
| LOC102641927 | 0.00144 | -1.40363226 |
| Gm32860 | 0.00176 | -1.24266807 |
| Gm31840 | 0.86115 | 0.17330777 |
| LOC328448 | 0.00144 | -1.38400103 |
| Gm36583 | 0.00144 | -1.30017714 |
| Gm36271 | 0.00171 | -1.13622611 |
| Gm31896 | 0.00144 | -1.20555712 |
| Gm35307 | 0.00144 | -1.29085005 |
| Gm34997 | 0.00974 | -1.13751021 |
| Gm33472 | 0.00144 | -1.31495282 |
| Gm32092 | 0.6176 | -0.12147854 |
| Gm7247 | 0.00144 | -1.28898079 |
| Gm36270 | 0.77881 | 0.52827176 |
| LOC102639645 | 0.00144 | -1.30017714 |
| Gm31393 | 0.00427 | -1.4705884 |
| Gm31131 | 0.00771 | -1.21963655 |
| LOC102642850 | 0.00144 | -1.29049509 |
| Gm36022 | 0.18316 | 0.23578638 |
| Gm34223 | 0.00144 | 1.77869266 |
| 4930467J12Rik | 0.07883 | -2.45539167 |
| Gm36703 | 0.00534 | 1.22820135 |
| Gm34995 | 0.06808 | -1.70585111 |
| Gm29680 | 0.01174 | -1.11750547 |
| Gm34526 | 0.00144 | -1.24811894 |
| Gm20362 | 0.02887 | 1.24527829 |
| Gm32067 | 0.00149 | -1.25408 |
| Gm30867 | 0.00144 | -1.30713006 |
| Gm38580 | 0.00158 | -1.2446827 |
| Gm36267 | 0.00144 | -1.27165604 |
| Gm36216 | 0.00144 | -1.31884045 |
| Gm30709 | 0.00145 | -1.3246192 |
| LOC102642927 | 0.00277 | 1.11361056 |
| Gm33820 | 0.75439 | -0.16337338 |
| 2010203P06Rik | 0.00144 | -1.28116048 |
| Gm34439 | 0.00144 | -1.48396829 |
| Gm33041 | 0.00144 | -1.41216421 |
| Gm34084 | 0.01236 | -0.67563847 |
| Gm32448 | 0.00145 | -1.2388121 |
| Gm38636 | 0.24903 | -0.67742893 |
| Gm34639 | 0.03766 | -1.6992191 |
| Gm34019 | 0.00144 | -1.28116048 |
| Gm32420 | 0.05723 | -0.91020214 |
| Gm31711 | 0.01892 | -1.76526189 |
| Gm20043 | 0.00144 | -1.37833222 |
| Gm30893 | 0.05279 | -0.46259151 |
| Gm36525 | 0.01555 | -1.13351264 |
| Gm36136 | 0.10492 | -1.43580951 |
| Gm30238 | 0.01782 | -1.23661281 |
| Gm35463 | 0.28234 | -0.4111456 |
| 4930559C10Rik | 0.00144 | -1.25118919 |
| Gm16876 | 0.16358 | -1.09671512 |
| Gm34717 | 0.02156 | -1.78813441 |
| Gm34613 | 0.28087 | -0.62218151 |
| Gm34189 | 0.78102 | 0.07795941 |
| Gm32664 | 0.01175 | -1.21376075 |
| Gm32608 | 0.36859 | 0.22500524 |
| Gm30687 | 0.00798 | -1.22437304 |
| Gm30001 | 0.00767 | -1.02946836 |
| Gm33811 | 0.00144 | -1.28898079 |
| Gm33680 | 0.00145 | -1.2388121 |
| Gm4166 | 0.00145 | -1.22376446 |
| 4933409F18Rik | 0.00144 | -1.31627578 |
| Gm36235 | 0.99455 | -0.00279614 |
| Gm36007 | 0.00145 | -1.25646236 |
| 9530020I12Rik | 0.00163 | -1.44174357 |
| Gm32442 | 0.00144 | -1.28116048 |
| Gm32398 | 0.00492 | -1.13895867 |
| Gm11682 | 0.00145 | -1.22376446 |
| LOC102641234 | 0.00144 | -1.28783973 |
| Gm33528 | 0.00144 | -1.29049509 |
| Gm12320 | 0.00145 | -1.2388121 |
| Gm30305 | 0.00185 | -1.10110414 |
| Gm12279 | 0.34905 | -0.32080112 |
| Gm33350 | 0.00144 | -1.28898079 |
| Gm31688 | 0.00158 | -1.2446827 |
| Gm30525 | 0.00982 | -1.03188337 |
| LOC102641267 | 0.0096 | -1.32137818 |
| Gm33277 | 0.4541 | -0.52883489 |
| Gm29887 | 0.00144 | -1.26934956 |
| 4930469K13Rik | 0.16922 | -0.76252921 |
| Gm33440 | 0.00439 | -1.19584548 |
| LOC102643017 | 0.98157 | 0.01380542 |
| Gm30770 | 0.18923 | -0.44076834 |
| Gm11967 | 0.04333 | -0.64037773 |
| Gm33983 | 0.00144 | -1.25118919 |
| Gm31504 | 0.02199 | -1.46433706 |
| Gm30456 | 0.00144 | -1.29746368 |
| Gm36284 | 0.03802 | -1.29347279 |
| Gm32552 | 0.0287 | -1.44524463 |
| LOC105245295 | 0.00306 | 1.95861026 |
| Gm26579 | 0.00144 | -1.28898079 |
| Gm30095 | 0.29644 | -0.38670855 |
| Gm29832 | 0.00144 | -1.29085005 |
| Gm34346 | 0.00144 | -1.27165604 |
| Gm33981 | 0.80189 | -0.36248761 |
| Gm34515 | 0.00144 | -1.28116048 |
| Gm30400 | 0.00149 | -1.2703832 |
| Gm35721 | 0.00144 | -1.27165604 |
| Gm34137 | 0.00144 | -1.30713006 |
| Gm30644 | 0.00144 | -1.31884045 |
| Gm17059 | 0.00144 | -1.27165604 |
| Gm36318 | 0.01325 | -0.8956441 |
| Gm36158 | 0.00149 | -1.25408 |
| Gm33058 | 0.0049 | -0.97481632 |
| Gm32411 | 0.02731 | -0.93945247 |
| Gm32284 | 0.00467 | -1.24333761 |
| Gm36908 | 0.00144 | -1.29085005 |
| Gm29571 | 0.00144 | -1.29746368 |
| Gm32543 | 0.00149 | -1.25408 |
| LOC102643113 | 0.58069 | 0.18505391 |
| Gm28905 | 0.01855 | 0.92312902 |
| LOC102633239 | 0.00144 | -1.30713006 |
| Gm30959 | 0.13798 | -3.12564995 |
| Gm29987 | 0.00145 | -1.22376446 |
| Gm35916 | 0.00145 | -1.24191372 |
| Gm34159 | 0.00144 | -1.5593573 |
| Gm35006 | 0.07626 | -1.55048979 |
| Gm34037 | 0.00149 | -1.25408 |
| Gm31701 | 0.15067 | -0.75511486 |
| LOC102637419 | 0.00144 | -1.26560808 |
| Gm33302 | 0.00145 | -1.2388121 |
| Gm32133 | 0.00144 | -1.27454685 |
| Gm32018 | 0.00144 | -1.20555712 |
| Gm31588 | 0.00145 | 1.31962522 |
| Gm31531 | 0.02929 | 0.80039945 |
| Gm31409 | 0.00144 | -1.30713006 |
| Gm36767 | 0.78203 | 0.08126791 |
| Gm38553 | 0.00145 | -1.2388121 |
| Gm34887 | 0.00144 | -1.28116048 |
| Gm32074 | 0.00144 | -1.35619208 |
| Gm31872 | 0.00314 | -1.08719927 |
| Gm35574 | 0.00257 | -1.1333102 |
| Gm33914 | 0.00159 | -1.26431393 |
| Gm33210 | 0.0017 | -1.24950944 |
| 2310003N18Rik | 0.00144 | -1.54795206 |
| Gm32016 | 0.00144 | -1.26911831 |
| LOC102633321 | 0.00353 | -1.06721129 |
| Gm16322 | 0.01909 | -0.70337569 |
| Gm35657 | 0.00145 | -1.2388121 |
| Gm31928 | 0.00144 | -1.26560808 |
| Gm34263 | 0.00144 | -1.3348153 |
| Gm31069 | 0.00744 | -1.28851018 |
| Gm35995 | 0.00144 | -1.20579466 |
| Gm35622 | 0.00591 | -1.44510092 |
| Gm34413 | 0.00144 | -1.28116048 |
| LOC102642471 | 0.00144 | -1.29051721 |
| Gm29896 | 0.00144 | -1.33632959 |
| Gm34197 | 0.02649 | -1.14119984 |
| Gm32642 | 0.06546 | -1.93609714 |
| Gm32642 | 0.00158 | -1.2446827 |
| LOC102641616 | 0.00186 | -1.38908362 |
| Gm30968 | 0.01369 | 0.60814281 |
| Gm35593 | 0.00317 | -1.12815152 |
| Gm2716 | 0.00144 | -1.26934956 |
| Gm32623 | 0.00329 | -1.44524463 |
| Gm35472 | 0.00399 | -0.9359682 |
| Gm34623 | 0.02221 | -1.64149562 |
| Gm30635 | 0.00144 | -1.20555712 |
| Gm40490 | 0.06483 | -0.88148784 |
| Gm33638 | 0.00144 | -1.28116048 |
| Gm38572 | 0.00144 | -2.68549656 |
| Gm30845 | 0.00749 | -1.05513651 |
| LOC102641681 | 0.00144 | -1.28116048 |
| B020031H02Rik | 0.20485 | -0.61498368 |
| Gm32485 | 0.00144 | -1.28898079 |
| Gm17215 | 0.00144 | -1.25118919 |
| Gm29978 | 0.00964 | 1.05945443 |
| Gm34908 | 0.00144 | -1.27165604 |
| Gm34645 | 0.00144 | -1.33059316 |
| Gm33027 | 0.00145 | -1.22376446 |
| Gm36449 | 0.28209 | 0.40676527 |
| LOC102638993 | 0.10085 | -0.70030275 |
| Gm33452 | 0.03428 | -0.62542331 |
| Gm32227 | 0.00144 | -1.28898079 |
| Gm26708 | 0.00144 | -1.31884045 |
| Gm29796 | 0.00144 | -1.28116048 |
| Gm20083 | 0.00144 | -1.30017714 |
| Gm36212 | 0.02122 | -0.96350362 |
| LOC102643257 | 0.00144 | -1.24613586 |
| Gm33990 | 0.01566 | -1.24333761 |
| Gtf2h1 | 0.02364 | -0.71799055 |
| Gm31882 | 0.00144 | -1.33632959 |
| 2310016G11Rik | 0.93474 | 0.02068396 |
| Gm34052 | 0.00144 | -1.30713006 |
| Gm30544 | 0.00144 | -1.29051721 |
| Gm38635 | 0.00384 | -1.6816109 |
| LOC102642512 | 0.00144 | -1.28898079 |
| Gm19733 | 0.37289 | 0.28733006 |
| Gm31152 | 0.0017 | -1.19401501 |
| Gm32069 | 0.00144 | -1.25118919 |
| Gm36269 | 0.05314 | -2.83953008 |
| Gm34617 | 0.00147 | -1.15996015 |
| Gm36788 | 0.07126 | -0.50037304 |
| Gm35649 | 0.00144 | -1.20555712 |
| Gm35467 | 0.00144 | -1.26428512 |
| Gm32705 | 0.00144 | -1.30017714 |
| Gm32481 | 0.00144 | -1.31884045 |
| Gm32191 | 0.00688 | -0.73531629 |
| Gm32425 | 0.01738 | -1.21541015 |
| Gm32378 | 0.93134 | 0.06852804 |
| Gm30782 | 0.00144 | -1.26042915 |
| Gm36408 | 0.00145 | -1.2388121 |
| Gm38539 | 0.00144 | -1.24811894 |
| Gm33047 | 0.69066 | 0.25197828 |
| LOC102642825 | 0.10606 | -0.95924197 |
| Gm31686 | 0.42559 | -0.76621944 |
| Gm35163 | 0.00144 | -1.29085005 |
| Gm35113 | 0.67482 | 0.32413586 |
| Gm33429 | 0.00198 | -1.60245164 |
| Gm33046 | 0.46537 | -0.36288034 |
| Gm30896 | 0.04953 | -2.17256898 |
| Gm35961 | 0.02745 | -2.1275663 |
| LOC102641712 | 0.55894 | -0.70459433 |
| Gm20714 | 0.00144 | -1.28116048 |
| Gm36187 | 0.00144 | -1.29746368 |
| Gm35704 | 0.08623 | -0.90336077 |
| Gm36667 | 0.00144 | -1.33632959 |
| Gm35628 | 0.00144 | -1.34578684 |
| Gm26779 | 0.03833 | -0.52681567 |
| LOC102641211 | 0.40622 | 0.41945724 |
| Gm33474 | 0.00145 | -1.24191372 |
| Gm32973 | 0.00208 | -1.15498993 |
| Gm35313 | 0.36406 | 0.18404958 |
| 1700031L13Rik | 0.59133 | -0.23461444 |
| Gm32780 | 0.00144 | -1.28116048 |
| Gm32558 | 0.00144 | -1.28116048 |
| Gm32064 | 0.00144 | -1.29085005 |
| Gm30601 | 0.03747 | -1.28752119 |
| Gm30178 | 0.00144 | -1.27699683 |
| Gm34193 | 0.00158 | -1.2446827 |
| Gm33993 | 0.00158 | -1.2446827 |
| Gm34600 | 0.00605 | -1.08150631 |
| Gm31831 | 0.00145 | -1.26219879 |
| Gm30962 | 0.00144 | -1.28116048 |
| Gm36157 | 0.00144 | -1.30017714 |
| Gm33710 | 0.05193 | -1.5923046 |
| Gm12829 | 0.00144 | -1.29049509 |
| Gm34784 | 0.00769 | -1.87642623 |
| Junos | 0.0534 | -1.2834123 |
| LOC102642989 | 0.01076 | -1.59273619 |
| Gm32209 | 0.01072 | -1.05977457 |
| Gm30764 | 0.50508 | -0.35722923 |
| Gm35402 | 0.00144 | -1.65855868 |
| Gm35344 | 0.00145 | -1.34348037 |
| Gm34628 | 0.00145 | -1.2388121 |
| Gm33729 | 0.01876 | 0.7049086 |
| Gm33159 | 0.00144 | -1.3348153 |
| Gm32135 | 0.00144 | -1.28116048 |
| Gm31145 | 0.00144 | -1.27730451 |
| Gm31085 | 0.00145 | -1.22376446 |
| Gm11884 | 0.00144 | -1.28898079 |
| Gm38450 | 0.00152 | -1.25878908 |
| Gm30092 | 0.00975 | 0.80623932 |
| Gm35059 | 0.0208 | 0.91819162 |
| Gm35008 | 0.00144 | -1.26560808 |
| Gm38586 | 0.00876 | 0.85217378 |
| Gm32823 | 0.00144 | -1.29049509 |
| Gm15577 | 0.00144 | -1.31884045 |
| Gm35555 | 0.00284 | 3.19707897 |
| Gm35555 | 0.00144 | 3.49854757 |
| LOC102641915 | 0.13778 | 0.52931194 |
| Gm35352 | 0.13645 | -3.36497333 |
| Gm26691 | 0.03733 | -1.06721129 |
| Gm29949 | 0.07157 | -0.59084672 |
| Gm38548 | 0.2258 | -0.39300726 |
| Gm32344 | 0.00144 | -1.29085005 |
| Gm31429 | 0.00144 | -1.28116048 |
| Gm35786 | 0.02255 | -1.43092725 |
| Gm35786 | 0.00778 | -1.06398377 |
| Gm30044 | 0.00144 | -1.30017714 |
| Gm35699 | 0.00145 | -1.22376446 |
| LOC105244486 | 0.0019 | -1.17487561 |
| Gm36396 | 0.00144 | -1.33632959 |
| Gm36288 | 0.00149 | -1.22582153 |
| Gm35236 | 0.06006 | -1.41097436 |
| Gm34780 | 0.37884 | 0.39503637 |
| Gm32237 | 0.10564 | -0.71150656 |
| Gm31796 | 0.00149 | -1.22582153 |
| Gm30063 | 0.67691 | -0.21445973 |
| Gm32843 | 0.02238 | -1.11355215 |
| Gm32795 | 0.01926 | -0.98294245 |
| Scd4 | 0.52295 | -0.10917943 |
| Gm30789 | 0.08035 | -2.44794749 |
| Gm33874 | 0.00144 | -1.82672492 |
| Gm30797 | 0.07879 | -1.22930105 |
| Gm30591 | 0.03151 | 0.95492107 |
| Gm35481 | 0.00144 | -1.32313902 |
| Gm30908 | 0.02792 | -0.91509798 |
| Gm31475 | 0.00144 | -1.27992547 |
| LOC100125594 | 0.00144 | -1.3348153 |
| Gm34570 | 0.00144 | -1.27730451 |
| LOC102643024 | 0.00144 | -1.6282881 |
| Gm14061 | 0.00145 | -1.2388121 |
| Gm14210 | 0.00144 | -1.24613586 |
| Gm35029 | 0.00145 | -1.40260632 |
| Gm33412 | 0.00144 | -1.26934956 |
| Gm31413 | 0.00144 | -1.31495282 |
| Gm31351 | 0.00144 | -1.24466058 |
| Gm13974 | 0.00242 | -1.40516868 |
| LOC102642119 | 0.03351 | -1.23404734 |
| Gm33780 | 0.00149 | -1.25408 |
| Gm35813 | 0.00144 | -1.27165604 |
| Gm35266 | 0.0137 | -0.91151295 |
| Gm31731 | 0.00144 | -1.27165604 |
| Gm31534 | 0.34492 | -0.44672858 |
| Gm38603 | 0.00144 | -1.30017714 |
| Gm31264 | 0.00144 | -1.25118919 |
| Gm8541 | 0.00144 | -1.28116048 |
| Gm33594 | 0.00144 | 1.74255926 |
| LOC102642365 | 0.0177 | -1.36653438 |
| Gm30374 | 0.07631 | -1.02423813 |
| LOC102642915 | 0.06979 | -2.01986102 |
| Gm33126 | 0.12296 | -0.83608965 |
| Gm31729 | 0.00144 | -1.26560808 |
| Gm13211 | 0.00145 | -1.25646236 |
| Gm13179 | 0.0017 | -1.19401501 |
| Gm32201 | 0.29603 | -0.38629533 |
| Gm31753 | 0.00144 | -1.25118919 |
| Gm33612 | 0.14459 | -0.53912799 |
| Gm36904 | 0.00144 | -1.28116048 |
| Gm35686 | 0.00144 | -1.29746368 |
| Gm32864 | 0.00144 | -1.48296698 |
| Gm31925 | 0.00145 | -1.24191372 |
| Pappa2 | 0.00144 | -1.28116048 |
| Glrx2 | 0.00144 | -1.26560808 |
| Gm32559 | 0.00149 | -1.22582153 |
| LOC102641701 | 0.02613 | -0.99601094 |
| Gm32810 | 0.00145 | -1.54633014 |
| Gm31973 | 0.00145 | -1.26219879 |
| Gm34616 | 0.04712 | -0.78454565 |
| Gm38609 | 0.00144 | -1.30713006 |
| Gm32700 | 0.00283 | -1.19015431 |
| Gm38631 | 0.6113 | -0.42815113 |
| Gm32793 | 0.59911 | 0.1285151 |
| Gm28818 | 0.00615 | -1.04993521 |
| Gm30308 | 0.00224 | -1.21636428 |
| Gm29822 | 0.00144 | -1.28898079 |
| Gm33774 | 0.00144 | -1.28116048 |
| Gm33724 | 0.00144 | -1.29049509 |
| Gm32792 | 0.00144 | -1.30017714 |
| Gm29155 | 0.01421 | 1.26048166 |
| LOC102641428 | 0.18016 | 0.90439099 |
| Gm31581 | 0.00144 | -1.31884045 |
| Gm31076 | 0.00336 | -1.20864476 |
| Gm30442 | 0.00144 | -1.25118919 |
| Gm31982 | 0.00144 | -1.3348153 |
| Gm30898 | 0.00144 | -1.28898079 |
| Gnb1l | 0.05036 | -0.71425718 |
| 2510039O18Rik | 0.01535 | -1.06652382 |
| Hsf5 | 0.36011 | -0.46066957 |
| 4932438A13Rik | 0.61956 | -0.74442052 |
| Sly | 0.00165 | 1.64173047 |
| Ofd1 | 0.00637 | -1.02877696 |
| Gm34521 | 0.00144 | -1.28898079 |
| Zfp280c | 0.03404 | 1.35370741 |
| Zdhhc6 | 0.223 | -0.24334798 |
| Asah2 | 0.08754 | -0.79468327 |
| Neto1 | 0.00144 | -1.27165604 |
| Gm35751 | 0.00144 | -1.28898079 |
| Dym | 0.75091 | -0.20836219 |
| Dtwd2 | 0.00144 | -1.30713006 |
| Gm10103 | 0.00144 | -1.30713006 |
| Hsf2bp | 0.6865 | -0.16572499 |
| Hnrnpll | 0.01032 | 0.77232877 |
| Opn5 | 0.70466 | -0.37281382 |
| Ttc7 | 0.21435 | -0.58275669 |
| Lhcgr | 0.00144 | -1.28116048 |
| Atp6v0e | 0.05757 | -1.06354197 |
| Efcab1 | 0.00144 | -1.31884045 |
| Olfr209 | 0.0247 | -1.17812235 |
| Tatdn1 | 0.89817 | 0.03420126 |
| Gm32970 | 0.00144 | -1.32599123 |
| Lmbrd2 | 0.00987 | -1.2020604 |
| Gm31606 | 0.00144 | -1.27165604 |
| Pcca | 0.00594 | 1.59720571 |
| Cdca2 | 0.09199 | -1.00063318 |
| Adamts16 | 0.09732 | -0.80886674 |
| Adamts16 | 0.00149 | -1.19696847 |
| Ttc37 | 0.0618 | -0.55796139 |
| Ift43 | 0.00144 | -1.60371653 |
| 4930447C04Rik | 0.11686 | -0.48019086 |
| Slc38a6 | 0.88794 | 0.05149291 |
| Mdga2 | 0.00144 | -1.25118919 |
| Hbp1 | 0.00144 | -1.28177426 |
| Nt5c1b | 0.00144 | -1.27165604 |
| Ttc19 | 0.18338 | -0.52054333 |
| Krt26 | 0.00144 | -1.26751264 |
| 4930505A04Rik | 0.00144 | -1.30713006 |
| Ranbp17 | 0.07046 | -0.87763815 |
| Anks1b | 0.00144 | -1.25118919 |
| Cep83 | 0.00144 | -1.29864962 |
| Nr2c1 | 0.66662 | 0.14633038 |
| Zfc3h1 | 0.00397 | 1.28500579 |
| Jmjd1c | 0.18788 | 0.38926608 |
| Ttc41 | 0.00144 | 1.35077048 |
| Hint3 | 0.093 | -0.42671695 |
| Aig1 | 0.67419 | 0.09807826 |
| Oprm1 | 0.15645 | -0.98117297 |
| Trank1 | 0.00144 | -1.29085005 |
| Elovl5 | 0.27167 | -1.92127398 |
| Cep70 | 0.1034 | 1.12382355 |
| Fat3 | 0.00152 | -1.39396588 |
| Mgat4d | 0.00337 | -1.43713248 |
| Zfp827 | 0.84943 | 0.10867919 |
| Nfatc3 | 0.00144 | -1.28898079 |
| Aktip | 0.00145 | -1.26219879 |
| Phkb | 0.27447 | 0.33038948 |
| Tmem221 | 0.00733 | -1.14281464 |
| Gm40503 | 0.35641 | -0.46049498 |
| Cdc16 | 0.48232 | -0.27867475 |
| Tmem255b | 0.00144 | -1.20009358 |
| Vwa3a | 0.68548 | 0.30798192 |
| Btbd1 | 0.00144 | 2.95171687 |
| Fam169b | 0.01766 | -1.70457192 |
| Adamts17 | 0.00208 | -1.15498993 |
| Gm33626 | 0.14493 | -0.74575259 |
| 2900092C05Rik | 0.00313 | -1.7572958 |
| Zfp329 | 0.00144 | 2.31783418 |
| C030039L03Rik | 0.13964 | -0.90779323 |
| Asun | 0.00144 | 1.30625934 |
| Gm10209 | 0.1625 | -2.00198491 |
| Agbl3 | 0.03831 | -2.99993596 |
| Slc25a26 | 0.40099 | -0.40845364 |
| Tnip3 | 0.00428 | 1.33948522 |
| Clec4b2 | 0.00144 | -1.38400103 |
| Dnah6 | 0.00149 | -1.19348234 |
| Dera | 0.16375 | -0.52679611 |
| Rxfp2 | 0.00916 | -1.06669723 |
| Zdhhc4 | 0.00205 | -0.90055155 |
| Snx8 | 0.0083 | -1.2924622 |
| Kntc1 | 0.00144 | -1.29878664 |
| Hscb | 0.25505 | 0.43222286 |
| Gm35871 | 0.65145 | 0.19861694 |
| Cfap69 | 0.00144 | -1.29085005 |
| Pdik1l | 0.65972 | -0.52657705 |
| Sfpq | 0.00181 | 1.58907799 |
| Zmym4 | 0.81186 | -0.19731131 |
| Rragc | 0.20375 | 0.205996 |
| Trmt10b | 0.1956 | 0.25516522 |
| Txndc8 | 0.0434 | -0.93768952 |
| Ccnc | 0.00556 | 1.64267935 |
| Ggh | 0.00144 | -1.27165604 |
| Gm38445 | 0.00144 | -1.28116048 |
| Lce6a | 0.00908 | -0.82441247 |
| Rpf1 | 0.16161 | 0.66545839 |
| Vmn2r1 | 0.00144 | -1.28116048 |
| Sh3glb1 | 0.44055 | 0.13153006 |
| Ankub1 | 0.01226 | -2.76829202 |
| Rorc | 0.00498 | -1.47816261 |
| Abca4 | 0.00784 | -1.02492676 |
| 3110057O12Rik | 0.01405 | 0.60283441 |
| Cyp7b1 | 0.13014 | 0.45070944 |
| Tti1 | 0.38528 | 0.22480283 |
| Katnbl1 | 0.69091 | 0.20792435 |
| Kif16b | 0.26351 | 0.20218383 |
| Arhgap15 | 0.05935 | -2.20827301 |
| Pip5kl1 | 0.0992 | -1.1443198 |
| Abl1 | 0.35155 | -1.47990231 |
| Axdnd1 | 0.00144 | -1.25118919 |
| 1700016C15Rik | 0.77273 | 0.07985223 |
| Suco | 0.00642 | 0.73303573 |
| Adss | 0.11515 | 0.91779525 |
| Pask | 0.01566 | -0.82099648 |
| R3hdm1 | 0.00209 | 2.23944288 |
| Tgfbrap1 | 0.01049 | -1.22130963 |
| Plcl1 | 0.00146 | 1.6408551 |
| Kansl3 | 0.00822 | -0.78496708 |
| Kansl3 | 0.18472 | -0.57543945 |
| Mcmdc2 | 0.01134 | -1.02600084 |
| Gm38717 | 0.0093 | -1.06147486 |
| LOC102641975 | 0.01771 | -2.3934719 |
| Prssly | 0.87881 | -0.07188046 |
| LOC102641312 | 0.0243 | -1.26177621 |
| Scd3 | 0.00145 | -1.26219879 |
| Gm36049 | 0.00144 | -1.26428512 |
| Gm35060 | 0.01569 | -0.96315785 |
| Nudt12os | 0.00145 | -1.22376446 |
| Zfp654 | 0.04174 | -0.69378729 |
| LOC102638754 | 0.00776 | -1.05664167 |
| Gm6657 | 0.16255 | -0.84350491 |
| Gm35095 | 0.00341 | -1.44701231 |
| Gm1096 | 0.00145 | -1.18741414 |
| Gm3189 | 0.00929 | -1.0433256 |
| Gm12963 | 0.00144 | -1.29746368 |
| Nsun4 | 0.01702 | -1.00335122 |
| 4930421P05Rik | 0.00144 | -1.27165604 |
| LOC102642345 | 0.00144 | -1.27699683 |
| Gm30732 | 0.01344 | 1.10460768 |
| Gm15133 | 0.60174 | -0.3774858 |
| 1700125H03Rik | 0.00144 | -1.29746368 |
| 4930596I21Rik | 0.2784 | -0.22750594 |
| 1700125H03Rik | 0.00144 | -1.26934956 |
| Gm15179 | 0.23511 | -0.85422193 |
| Uckl1os | 0.92063 | 0.05308075 |
| 4933406J10Rik | 0.00144 | -1.28116048 |
| Gm15679 | 0.00145 | -1.2388121 |
| Ncf1 | 0.23932 | -0.51140307 |
| l7Rn6 | 0.04057 | 1.75042584 |
| 4930448H16Rik | 0.00144 | -1.24811894 |
| St8sia3os | 0.00144 | -1.34578684 |
| Rubie | 0.00144 | -1.25118919 |
| Gm16336 | 0.00145 | -1.22376446 |
| Gm16325 | 0.47961 | -0.23805189 |
| Pantr2 | 0.00144 | -1.27165604 |
| Halr1 | 0.9629 | 0.03668878 |
| 4930486I03Rik | 0.00225 | -1.18527205 |
| Gm16291 | 0.00866 | 1.6221023 |
| Gm15880 | 0.15139 | -0.79051962 |
| Gm15850 | 0.00493 | -0.68153367 |
| Nell1os | 0.00144 | -1.40395392 |
| Rmst | 0.00144 | -1.26934956 |
| Gm38509 | 0.00182 | -1.19015431 |
| Airn | 0.64704 | 0.07734478 |
| 4930483O08Rik | 0.23017 | -0.30091145 |
| Gm20759 | 0.0097 | -1.20526003 |
| 4930413M19Rik | 0.00621 | -1.70507192 |
| Gm19710 | 0.0444 | -0.67450486 |
| Gm19299 | 0.00144 | -1.27699683 |
| Gm16617 | 0.72539 | -0.21521073 |
| 4930471L23Rik | 0.00144 | -1.26560808 |
| Platr21 | 0.00144 | -1.28116048 |
| 4930515L19Rik | 0.00144 | -1.30017714 |
| 2010010A06Rik | 0.02401 | -0.92329341 |
| 1700064J06Rik | 0.02805 | -0.83459957 |
| Gm14827 | 0.00144 | 2.27299195 |
| 4930449E18Rik | 0.83308 | -0.03317856 |
| 4930593A02Rik | 0.00144 | -1.25927895 |
| A830019L24Rik | 0.00298 | -1.39967264 |
| 1700003L19Rik | 0.00149 | -1.19696847 |
| 1700080N15Rik | 0.01001 | -0.66611932 |
| 4930568G15Rik | 0.00144 | -1.33632959 |
| D030024E09Rik | 0.00743 | -1.01204217 |
| 2810454H06Rik | 0.70537 | -0.25786608 |
| 2900060B14Rik | 0.05007 | -0.74147071 |
| 2700069I18Rik | 0.00144 | -1.34414049 |
| LOC100505025 | 0.17406 | -0.50673075 |
| Gm21283 | 0.00144 | -1.30017714 |
| Dreh | 0.00144 | -1.30713006 |
| Gm12596 | 0.00144 | -1.20555712 |
| 1700124L16Rik | 0.72976 | 0.29501615 |
| 6430562O15Rik | 0.22476 | -0.33807726 |
| A730017L22Rik | 0.1555 | -0.27760957 |
| E330017L17Rik | 0.08218 | -0.65795092 |
| C130036L24Rik | 0.92408 | -0.08218742 |
| 9330111N05Rik | 0.02331 | -1.87071863 |
| 1500004A13Rik | 0.56357 | 0.25557098 |
| 4930513N10Rik | 0.05182 | 0.46694297 |
| A530053G22Rik | 0.00145 | -1.2388121 |
| A930015D03Rik | 0.89083 | -0.08012729 |
| 1700120C14Rik | 0.01321 | -0.79932123 |
| Gm6300 | 0.00144 | -1.28898079 |
| Gm17769 | 0.45218 | 0.31886106 |
| Gm19424 | 0.00144 | -1.28116048 |
| AA536875 | 0.03876 | -0.51892549 |
| Gm17830 | 0.16796 | -0.68972786 |
| 1700047L14Rik | 0.00145 | -1.22376446 |
| D630041G03Rik | 0.08802 | 0.75537939 |
| 4933405D12Rik | 0.57391 | -0.10260751 |
| Scpep1os | 0.00259 | -1.45799871 |
| 1700066N21Rik | 0.00144 | -1.30017714 |
| 1700001D01Rik | 0.83225 | 0.13950522 |
| G630093K05Rik | 0.05478 | -0.98578313 |
| E130008D07Rik | 0.00144 | -1.28116048 |
| Gm20741 | 0.00144 | -1.4406427 |
| 1700126H18Rik | 0.40795 | -0.25353226 |
| Gm12718 | 0.00144 | -1.36831552 |
| D830005E20Rik | 0.00144 | 1.77779691 |
| 1700125G02Rik | 0.59464 | 0.16135576 |
| 9230105E05Rik | 0.00144 | -1.29051721 |
| 4930590L20Rik | 0.00144 | -1.25118919 |
| Snora81 | 0.06536 | 0.49838562 |
| Olfr1077-ps1 | 0.02044 | -1.32766452 |
| Snora23 | 0.01309 | 0.89964642 |
| Scarna6 | 0.06539 | -1.19805465 |
| Snord17 | 0.00144 | -1.25342998 |
| 4930433N12Rik | 0.00144 | -1.30017714 |
| 9530036O11Rik | 0.00145 | -1.26219879 |
| 4632427E13Rik | 0.40787 | 0.19798922 |
| Gm12633 | 0.00145 | -1.36286464 |
| Cdv3 | 0.32494 | -0.1678826 |
| Snhg9 | 0.09676 | -0.4574611 |
| C230004F18Rik | 0.97734 | -0.06654395 |
| Gm16159 | 0.18917 | -0.52136524 |
| 4930571O06Rik | 0.00317 | -1.11935153 |
| 4933407K13Rik | 0.00144 | 2.7451123 |
| Gm14005 | 0.0025 | -0.99428852 |
| Gm11213 | 0.22918 | -0.77416505 |
| 9330020H09Rik | 0.0017 | -1.24950944 |
| 1700109K24Rik | 0.00144 | -1.27454685 |
| Gm16712 | 0.00144 | -1.27558215 |
| AA465934 | 0.00496 | -0.80628392 |
| 4833422C13Rik | 0.02303 | 1.65911807 |
| Gm29805 | 0.02913 | -1.30451811 |
| Gm15908 | 0.00145 | -1.22376446 |
| 1010001N08Rik | 0.17403 | 0.23461349 |
| C4bp-ps1 | 0.04466 | 0.67957379 |
| Atad3aos | 0.03102 | -1.42632532 |
| Zp4-ps | 0.00166 | -1.37728565 |
| C130046K22Rik | 0.07891 | -0.35045927 |
| 5830444B04Rik | 0.04489 | -0.76812756 |
| AI839979 | 0.15984 | 0.342291 |
| AI314278 | 0.00144 | -1.29085005 |
| Gm10619 | 0.00145 | -1.33480237 |
| Vax2os | 0.13116 | -1.25409129 |
| AA543186 | 0.53608 | 0.16008837 |
| H2-Q7 | 0.0018 | -1.02269074 |
| Gm16973 | 0.00215 | 2.18686458 |
| C630031E19Rik | 0.08656 | -0.78914758 |
| 1700120G07Rik | 0.0113 | -0.79766044 |
| AI463170 | 0.10053 | -0.89388033 |
| 9530052E02Rik | 0.48524 | -0.45546182 |
| AU021063 | 0.00144 | -1.39713491 |
| A730085K08Rik | 0.07512 | -0.66873302 |
| 4930401O12Rik | 0.00144 | -1.26428512 |
| B020014A21Rik | 0.00152 | -1.25878908 |
| Gm15008 | 0.00144 | -1.28898079 |
| 9530027J09Rik | 0.00158 | -1.2446827 |
| Gm17746 | 0.00144 | -1.48564159 |
| 1700021N21Rik | 0.00144 | -1.24811894 |
| Gm16596 | 0.11448 | -1.33249771 |
| 1700030F04Rik | 0.00144 | -1.27992547 |
| E130310I04Rik | 0.0069 | -1.61720061 |
| E130310I04Rik | 0.00144 | -1.30017714 |
| 1700007L15Rik | 0.09645 | -0.61868454 |
| I730028E13Rik | 0.00225 | -1.18527205 |
| Fam120aos | 0.18582 | -0.23141421 |
| 4930442L01Rik | 0.02363 | -1.31343206 |
| BC024386 | 0.00144 | -1.25927895 |
| 5430402O13Rik | 0.00293 | 1.0762729 |
| 2210015D19Rik | 0.49851 | -0.16815483 |
| 4732471J01Rik | 0.00145 | -1.22376446 |
| 4930511M06Rik | 0.15683 | -0.63865976 |
| B430010I23Rik | 0.0705 | 0.98597981 |
| 3010001F23Rik | 0.34621 | 0.42921818 |
| 1700071M16Rik | 0.00406 | -1.20200164 |
| 4930513D17Rik | 0.04873 | -0.42266128 |
| C030018K13Rik | 0.00144 | -1.46410581 |
| 4930527G23Rik | 0.00144 | -1.36353418 |
| 2610016A17Rik | 0.00144 | -1.24811894 |
| Gm13539 | 0.00442 | -1.01701239 |
| Gm19705 | 0.57232 | 0.27986308 |
| Gm19557 | 0.01347 | -0.80683671 |
| 1700048M11Rik | 0.00144 | -1.29878664 |
| 2900009J06Rik | 0.50837 | 0.36669367 |
| 1700064M15Rik | 0.01072 | -1.05977457 |
| 4932702P03Rik | 0.0019 | -1.34515366 |
| 4933427I22Rik | 0.00145 | -1.20627532 |
| 1110028F11Rik | 0.00397 | -0.70834793 |
| Gm16677 | 0.54336 | -0.10369579 |
| Gm2721 | 0.24909 | -0.72477109 |
| AI197445 | 0.08067 | -1.44203557 |
| Gm16576 | 0.45256 | -0.22062726 |
| Gm11351 | 0.15422 | -0.68049221 |
| 3110056K07Rik | 0.42956 | -0.23367295 |
| Gm15987 | 0.14448 | -0.55785214 |
| Gm20756 | 0.03381 | -0.99625852 |
| B230323A14Rik | 0.02203 | -0.972494 |
| LOC100503496 | 0.00308 | -1.21363744 |
| Frmpd1os | 0.00601 | -1.09465341 |
| Arhgap15os | 0.00145 | -1.24191372 |
| Gm1653 | 0.00144 | -1.20555712 |
| 4930567J20Rik | 0.00144 | -1.26934956 |
| Gm10560 | 0.01005 | -1.36035573 |
| Gm20356 | 0.52503 | -0.46941966 |
| Gm10007 | 0.00144 | -1.31495282 |
| Gm13003 | 0.00144 | -1.25118919 |
| AV051173 | 0.33326 | -0.23174635 |
| Gm16023 | 0.93391 | -0.02257781 |
| Gm15471 | 0.03734 | -1.06568406 |
| 4930570G19Rik | 0.00144 | -1.38316541 |
| Gm13293 | 0.12364 | -1.18851502 |
| Gm16796 | 0.3094 | 0.37501 |
| Gm13544 | 0.91518 | -0.07658174 |
| 9430091E24Rik | 0.05018 | 1.48221444 |
| D330023K18Rik | 0.0138 | -0.94127216 |
| A230077H06Rik | 0.00144 | -1.27165604 |
| 1700017J07Rik | 0.1688 | -0.39413865 |
| Platr14 | 0.08825 | -2.64477545 |
| 2310069G16Rik | 0.79914 | -0.07955292 |
| Gm20743 | 0.0031 | -1.09550759 |
| Gm19757 | 0.02706 | -1.18627336 |
| Gm12130 | 0.86037 | 0.27415277 |
| 3632454L22Rik | 0.00144 | -1.31495282 |
| Gm3143 | 0.00974 | -1.36944865 |
| 1700001G11Rik | 0.3281 | -0.32115804 |
| C030037D09Rik | 0.45005 | 0.27098997 |
| 4930444F02Rik | 0.00144 | -1.29746368 |
| Gm15910 | 0.00144 | -1.30713006 |
| 4930486F22Rik | 0.00144 | -1.27730451 |
| Gm16998 | 0.00583 | -1.04768057 |
| 4930558J18Rik | 0.00144 | -1.29051721 |
| B230216N24Rik | 0.0234 | 0.80195019 |
| Platr22 | 0.02298 | -1.79260595 |
| Gm41 | 0.21958 | -0.4476452 |
| A730018C14Rik | 0.00144 | -1.29085005 |
| Miat | 0.15705 | -1.53851694 |
| Cyp2c53-ps | 0.00702 | -1.56335487 |
| Gm10125 | 0.18463 | -0.53073944 |
| Gm10510 | 0.00144 | -1.31884045 |
| Gm5089 | 0.00149 | -1.63417072 |
| Ppp1r12b | 0.00827 | 1.44299857 |
| Gm7788 | 0.1685 | -0.69023529 |
| 5730412P04Rik | 0.00144 | -1.27165604 |
| 4930433B08Rik | 0.02344 | -1.79829712 |
| 2010320M18Rik | 0.00916 | -0.81883586 |
| Mira | 0.05897 | -1.17049547 |
| F630206G17Rik | 0.19436 | -0.62090372 |
| A630066F11Rik | 0.26303 | -0.81158264 |
| 4930545E07Rik | 0.00145 | -1.22193674 |
| Gm19782 | 0.04115 | 0.82474007 |
| B020018J22Rik | 0.00144 | -1.24466058 |
| Gm5833 | 0.10956 | -0.89118547 |
| Gm20139 | 0.00144 | -1.26428512 |
| Gm14204 | 0.00154 | 0.94118903 |
| Gm11762 | 0.04554 | -2.04485991 |
| A930004D18Rik | 0.03634 | -1.9895684 |
| AI506816 | 0.00144 | -1.77456843 |
| 1600002D24Rik | 0.00144 | -1.29746368 |
| 4930558G05Rik | 0.00144 | -1.25118919 |
| 5730408K05Rik | 0.01127 | -0.85334376 |
| 1700063O14Rik | 0.00158 | -1.2446827 |
| Gm13483 | 0.13487 | -0.81630032 |
| 1700020M21Rik | 0.0105 | -1.1138881 |
| Mir143hg | 0.0154 | 1.41447012 |
| Gm17801 | 0.00144 | -1.29085005 |
| 5031426D15Rik | 0.00269 | 1.7353073 |
| Gm17762 | 0.00144 | -1.31495282 |
| Gm10033 | 0.491 | -0.26717685 |
| Vaultrc5 | 0.0097 | -1.08350696 |
| Gm10814 | 0.31514 | -0.28761825 |
| 5930403L14Rik | 0.58787 | -0.12052613 |
| Gm19583 | 0.11664 | -1.41047821 |
| Gm19589 | 0.46473 | -0.51126328 |
| Gm16675 | 0.06551 | -1.08842762 |
| Gm10400 | 0.15142 | -0.36335845 |
| Gm15446 | 0.0041 | 1.48848937 |
| 0610038B21Rik | 0.00305 | -0.97746836 |
| 2010310C07Rik | 0.03584 | -0.65498321 |
| 1700123O21Rik | 0.00286 | -1.1443198 |
| Gm13752 | 0.78017 | 0.19798098 |
| Rprl2 | 0.33791 | -0.26820576 |
| Rpph1 | 0.02285 | -1.22366937 |
| Gm15694 | 0.87289 | 0.060342 |
| Bambi-ps1 | 0.00144 | -1.10424287 |
| AA413626 | 0.25819 | 0.45543639 |
| Snora78 | 0.00144 | -1.32394819 |
| Stmn1-rs1 | 0.09437 | -0.39258895 |
| Gm6623 | 0.00185 | -1.42584466 |
| Rprl1 | 0.56763 | -0.19668532 |
| Ywhaq | 0.00786 | 0.65580654 |
| Rprl3 | 0.76978 | -0.08833071 |
| Ppp1r2-ps9 | 0.00144 | -1.46698089 |
| Snora73a | 0.00144 | -1.10849934 |
| Gm10416 | 0.12965 | 0.52694756 |
| Rps19 | 0.00265 | -0.94762821 |
| Gm9159 | 0.01183 | 1.21644249 |
| Scarna13 | 0.00144 | -1.46681442 |
| Marcksl1-ps4 | 0.00145 | -0.9683623 |
| Gm10012 | 0.0052 | -0.90645692 |
| 1700084J12Rik | 0.44117 | 0.25049092 |
| 1700028B04Rik | 0.10868 | -0.5503127 |
| Cfap43 | 0.50639 | -0.1087002 |
| Uqcc1 | 0.57207 | 0.13818887 |
| Izumo1r | 0.00767 | -1.13052618 |
| Mterf3 | 0.00199 | 0.88521041 |
| Zfyve19 | 0.00813 | -0.84732399 |
| Cpped1 | 0.22354 | 0.2005967 |
| Wbscr22 | 0.03484 | -0.51502016 |
| Taf1 | 0.01162 | 1.03201946 |
| Esp6Esp5 | 0.26412 | -0.86833342 |
| Col11a2 | 0.01709 | -0.574036 |
| Chd6 | 0.00421 | 1.33942698 |
| Gyk | 0.00144 | 1.81691447 |
| Lmntd2 | 0.20056 | -0.57821848 |
| Wnt11 | 0.14936 | -1.06028001 |
| Tpm3 | 0.13103 | -0.32270827 |
| Fam206a | 0.578 | 0.08637687 |
| Cflar | 0.13408 | 0.38259728 |
| Nme7 | 0.07766 | -0.84372505 |
| Clasp1 | 0.31359 | -0.17614574 |
| Zfp799 | 0.37886 | -0.19120088 |
| Osgep | 0.02632 | -0.67856206 |
| Hgf | 0.00144 | -1.27165604 |
| Lck | 0.09012 | -0.96819348 |
| Fgfr1 | 0.04646 | -0.54459841 |
| Eef2k | 0.32561 | 0.20163131 |
| Nf2 | 0.00483 | -0.80686796 |
| Cacna1a | 0.17172 | -0.28548092 |
| Arrb2 | 0.86501 | -0.08462465 |
| Ghrl | 0.17029 | 0.59105348 |
| Cops7a | 0.10357 | -0.50629127 |
| Prkaa2 | 0.01682 | 0.56310266 |
| Aasdh | 0.54309 | 0.10221589 |
| Hes5 | 0.40099 | -0.25757224 |
| Fbxw7 | 0.88513 | 0.02768294 |
| Mapk8 | 0.08233 | 0.3983567 |
| Ripk1 | 0.01304 | 0.74341213 |
| Foxo3 | 0.0088 | -0.65858201 |
| Pdcd4 | 0.00815 | 1.03343889 |
| Ppt1 | 0.04302 | -0.37688871 |
| Cirbp | 0.1368 | 0.80806624 |
| Ly96 | 0.20892 | 0.40361431 |
| Use1 | 0.00144 | -1.08943075 |
| Ube2d2a | 0.01095 | 0.62936188 |
| Lepr | 0.00751 | 2.24406824 |
| Nrf1 | 0.48029 | -0.14221202 |
| Mad2l2 | 0.24713 | -0.39404629 |
| Figla | 0.00145 | -1.27532353 |
| Tfpi | 0.00144 | 1.25707094 |
| Grin2a | 0.25894 | -1.88898867 |
| Pla2g7 | 0.6964 | 0.08537961 |
| Eftud2 | 0.00941 | -0.64434699 |
| Pot1a | 0.77732 | -0.0486514 |
| Ercc3 | 0.86772 | -0.03161381 |
| Grn | 0.10951 | -0.53615405 |
| Trpc5os | 0.00144 | -1.26560808 |
| Dclk1 | 0.07439 | 1.14825499 |
| Nnmt | 0.00188 | -1.16080741 |
| Arhgef12 | 0.03691 | 0.43135292 |
| Erc1 | 0.05149 | -0.57335277 |
| Ptpn6 | 0.00398 | -0.98022875 |
| Bmi1 | 0.00144 | 1.462409 |
| Ano5 | 0.00194 | -1.92204526 |
| Muc2 | 0.00144 | -1.31495282 |
| Wfdc11 | 0.00144 | -1.29085005 |
| 1700074P13Rik | 0.50318 | -0.37206434 |
| Fcrl1 | 0.28049 | -0.68777986 |
| Utp14b | 0.15601 | 0.55658374 |
| Arhgap21 | 0.00144 | 1.38478909 |
| Cobll1 | 0.42435 | 0.13720198 |
| Slc13a1 | 0.05105 | -0.92035376 |
| Wrn | 0.34007 | 0.30392075 |
| Zfr | 0.24607 | 0.23321334 |
| Armc2 | 0.59294 | 0.08459948 |
| Epb4.1l5 | 0.00477 | 1.03104005 |
| Fggy | 0.07123 | 0.36201693 |
| Col5a1 | 0.10707 | -0.44397839 |
| Synj2 | 0.89267 | -0.02994382 |
| Cyth1 | 0.81305 | 0.0449515 |
| Lats1 | 0.00173 | 1.26111221 |
| Adat2 | 0.81046 | -0.07188827 |
| Asah1 | 0.32469 | 0.18835336 |
| Adamts9 | 0.10956 | 0.40498859 |
| Apbb1ip | 0.05908 | -0.39959734 |
| Srgap2 | 0.00734 | -0.91840937 |
| Drc7 | 0.00453 | -1.09544188 |
| Fbxl18 | 0.00292 | -0.83961998 |
| Dock6 | 0.57481 | 0.09324067 |
| Lrriq4 | 0.00144 | -1.3169359 |
| Luzp1 | 0.07102 | -0.3131566 |
| Pctp | 0.30557 | -0.20452457 |
| Ikzf3 | 0.18551 | -0.2780637 |
| Arrdc4 | 0.00187 | -1.07218116 |
| Psmc3ip | 0.57975 | -0.12027931 |
| Mapt | 0.33484 | 0.26722618 |
| Bcl2l11 | 0.35493 | -0.17555291 |
| Gmnn | 0.3357 | 0.36864392 |
| Amelx | 0.00144 | -1.30713006 |
| Sort1 | 0.9496 | 0.01692123 |
| Igsf9 | 0.04808 | -0.45357665 |
| Elf4 | 0.38219 | -0.16910858 |
| Pmp22 | 0.08702 | -0.28809012 |
| Jazf1 | 0.00144 | 1.98173153 |
| Mdk | 0.03612 | -0.39048561 |
| Gnrh1 | 0.01578 | 1.71977611 |
| Acvr2a | 0.00144 | 1.97613979 |
| Postn | 0.00144 | 1.97616861 |
| Mnx1 | 0.00144 | -1.45144986 |
| Cdh8 | 0.00145 | -1.2388121 |
| Lrp4 | 0.98328 | 0.00731182 |
| Wdr78 | 0.01847 | 1.85483291 |
| Zyx | 0.18896 | -0.29576052 |
| Rbfox2 | 0.81977 | -0.03877794 |
| Trpc6 | 0.01881 | 0.88980052 |
| Bhlhe41 | 0.77147 | 0.06289176 |
| Kat5 | 0.83396 | 0.06924165 |
| Ptger1 | 0.65498 | -0.43682867 |
| Lmo2 | 0.07547 | -0.31649092 |
| Pou3f2 | 0.00144 | -1.45078974 |
| Prlr | 0.75686 | 0.05536776 |
| Cd82 | 0.00144 | -1.05189307 |
| Angpt2 | 0.01929 | -0.79098544 |
| Tmem97 | 0.00582 | -0.70142254 |
| Plcxd1 | 0.46995 | -0.43709652 |
| Tbx18 | 0.75951 | 0.1340569 |
| Cdo1 | 0.27445 | -1.43859874 |
| Trpc4 | 0.00145 | -1.26219879 |
| Tnni3 | 0.00746 | -0.72971427 |
| Spo11 | 0.00144 | -1.27165604 |
| Dthd1 | 0.26494 | -1.29910547 |
| Dsc3 | 0.29458 | -0.16590552 |
| Sema3d | 0.00273 | 2.37596003 |
| Romo1 | 0.00875 | -0.90245205 |
| Pomgnt1 | 0.9777 | 0.01626202 |
| Glo1 | 0.024 | -0.5314027 |
| Rnasek | 0.29887 | -0.44047369 |
| Suv420h1 | 0.05008 | 0.40190423 |
| Slc17a9 | 0.05067 | 0.73098078 |
| Atp5a1 | 0.04554 | -0.42732985 |
| Med1 | 0.00991 | 0.59394796 |
| Pnkd | 0.01657 | -0.69706724 |
| a | 0.008 | -0.97005524 |
| Smarca2 | 0.031 | 0.68179873 |
| Adh1 | 0.15421 | 0.48742291 |
| Epm2a | 0.00144 | -1.21306706 |
| Rsrp1 | 0.0024 | 0.81837901 |
| Prmt10 | 0.5431 | -0.09580337 |
| Mrps33 | 0.75885 | -0.05278424 |
| Ndufb5 | 0.08612 | -0.45002754 |
| Hmgn1 | 0.90084 | -0.02147523 |
| Smc4 | 0.0128 | 1.58904029 |
| Rabl2 | 0.25233 | 0.21443899 |
| Smn1 | 0.02569 | -0.47944293 |
| Tfip11 | 0.00698 | -0.77350596 |
| 4930563E22Rik | 0.0017 | 1.18730903 |
| Tiam1 | 0.69058 | -0.08906016 |
| Setd4 | 0.05408 | 0.48588483 |
| Ikzf2 | 0.00144 | 1.30974675 |
| 1700011H14Rik | 0.16506 | -2.51916166 |
| Catip | 0.03107 | 0.94605499 |
| H2-L | 0.00145 | -1.12918781 |
| Vcan | 0.00222 | 1.30866637 |
| Agtpbp1 | 0.46748 | 0.14875204 |
| Dnm3 | 0.04912 | 0.72627078 |
| Whrn | 0.29422 | -0.715923 |
| Trim72 | 0.02106 | -0.67728491 |
| Slc9a5 | 0.082 | 0.56806971 |
| Lrrk1 | 0.00328 | -0.74818031 |
| Polb | 0.6819 | -0.06450776 |
| Gm10267 | 0.01761 | -1.71461918 |
| Csnk1e | 0.01809 | -0.66410974 |
| Ptprt | 0.00338 | -1.36162963 |
| Inpp4a | 0.34505 | 0.16475901 |
| Awat2 | 0.35851 | 0.23336607 |
| Ildr1 | 0.00144 | -1.30713006 |
| Rbm46 | 0.71527 | 0.10540481 |
| Lrrtm4 | 0.01818 | -1.64175637 |
| Mroh6 | 0.00144 | -1.29746368 |
| Pet117 | 0.00324 | -0.97144332 |
| D630023F18Rik | 0.00571 | -1.59771307 |
| Meaf6 | 0.32531 | -0.18321115 |
| Milr1 | 0.10592 | -0.76701096 |
| Shisa5 | 0.00463 | -0.80706311 |
| Gabpb1 | 0.59554 | 0.12644311 |
| Upp2 | 0.00144 | -1.28116048 |
| Ankra2 | 0.29319 | 0.69327901 |
| Hist1h1c | 0.01344 | -0.71940915 |
| Dtna | 0.02265 | 0.58949035 |
| Rhox5 | 0.00144 | -1.11775236 |
| Sh3glb1 | 0.00293 | 0.99548481 |
| Hist3h2ba | 0.00144 | -1.1002443 |
| Klf10 | 0.36912 | 0.34304878 |
| Kcnj2 | 0.06606 | 0.33438289 |
| Tnk1 | 0.15157 | -0.80293518 |
| Amot | 0.00144 | -1.31495282 |
| Hist1h2ai | 0.00185 | -1.11390758 |
| Hist1h2bb | 0.89194 | 0.04945399 |
| Steap2 | 0.00144 | -1.28116048 |
| Ghr | 0.53785 | 0.12078905 |
| Tecta | 0.01709 | -0.99496437 |
| Bcl2l1 | 0.00686 | -0.70183845 |
| Ppp2r1a | 0.00639 | -0.79485295 |
| Crk | 0.002 | 1.835902 |
| Snrpa1 | 0.83098 | -0.03517521 |
| Brd7 | 0.08805 | 0.32940442 |
| Rnf145 | 0.39813 | 0.40776017 |
| Insig2 | 0.01285 | 0.81357728 |
| Clic1 | 0.00965 | -0.69441216 |
| Pou2f1 | 0.5761 | 0.10606172 |
| Snx25 | 0.00354 | 1.03253886 |
| Coasy | 0.5428 | -0.25889553 |
| Ddc | 0.25184 | 0.46754856 |
| Ptms | 0.27381 | -0.23691045 |
| Cadps2 | 0.01072 | -1.54660501 |
| Blm | 0.805 | -0.12161667 |
| Atxn1 | 0.0139 | 0.64568757 |
| Unc80 | 0.5333 | -0.35471011 |
| Dcaf17 | 0.00245 | 1.12759416 |
| 4932438A13Rik | 0.00144 | 1.33591086 |
| Pfkfb2 | 0.0237 | 0.71244206 |
| Ak2 | 0.32029 | -0.1807843 |
| Myo1c | 0.00404 | -0.82165567 |
| Akr1a1 | 0.02268 | -0.56205748 |
| Zfp407 | 0.61429 | -0.11647554 |
| Arr3 | 0.26797 | -1.29083974 |
| Slc25a11 | 0.71083 | 0.27990807 |
| Mcm3 | 0.03308 | -0.45293022 |
| Cadps2 | 0.15725 | 0.92005625 |
| 0610010K14Rik | 0.0261 | -0.65230035 |
| Jmjd6 | 0.00863 | -0.77560027 |
| Psmd14 | 0.01026 | 0.62971186 |
| Cadps2 | 0.00945 | -0.73054002 |
| Bpnt1 | 0.61966 | 0.09282397 |
| Glrx3 | 0.0674 | 0.49137497 |
| Rbm39 | 0.00144 | 1.48178906 |
| Rsg1 | 0.46634 | -0.22066668 |
| Fam210b | 0.94652 | -0.0139974 |
| Timm44 | 0.05273 | 1.07794932 |
| Wwox | 0.00904 | -0.70107036 |
| Myl1 | 0.2313 | -0.55978247 |
| Pla2g5 | 0.36859 | -0.36034366 |
| Trim28 | 0.00743 | -0.69747052 |
| Psma6 | 0.23764 | -0.19503431 |
| Zak | 0.19148 | 0.52392787 |
| H2afy | 0.05555 | -0.43480326 |
| Mecr | 0.51674 | -0.22044249 |
| Uroc1 | 0.00144 | -1.27165604 |
| Bcl9l | 0.00368 | -0.84805267 |
| Pikfyve | 0.23459 | 0.4690638 |
| Mrpl48 | 0.04596 | -0.47347921 |
| Lrrc48 | 0.49835 | -0.11962073 |
| Tmigd1 | 0.19088 | 0.27113922 |
| Zranb2 | 0.00144 | 1.83095445 |
| Riiad1 | 0.01565 | -0.60729318 |
| Tomm40l | 0.43315 | -0.24237279 |
| Tmsb15a | 0.37126 | -0.45028026 |
| Efcab5 | 0.07487 | -0.73306264 |
| Lars | 0.32732 | 0.2064684 |
| Idh3g | 0.05298 | -0.42420981 |
| Dvl1 | 0.48136 | -0.14795604 |
| Irgm1 | 0.01206 | 0.72643387 |
| Ola1 | 0.2461 | 0.27003804 |
| Scai | 0.00144 | 3.38124082 |
| Mff | 0.10868 | 0.33282593 |
| Cox17 | 0.00334 | -0.84270287 |
| Mcoln1 | 0.03714 | -0.5948531 |
| Cyb5rl | 0.89232 | -0.0846865 |
| Ypel1 | 0.67748 | -0.11514032 |
| Nsfl1c | 0.77323 | -0.05257805 |
| Gpcpd1 | 0.00144 | 1.11126225 |
| Pdxdc1 | 0.05847 | 0.68909408 |
| Ppp1r42 | 0.15934 | -0.61748094 |
| Tsga10 | 0.03954 | -0.85473465 |
| Osbpl6 | 0.1389 | -0.7084769 |
| Zfp451 | 0.82652 | 0.17281824 |
| Suv39h1 | 0.94591 | -0.01505761 |
| Hist1h2ad | 0.00144 | -1.24811894 |
| Fer1l5 | 0.00144 | -1.29746368 |
| Rnf32 | 0.29749 | 0.41791886 |
| Mmadhc | 0.16376 | 0.71372945 |
| Rpain | 0.12172 | -0.32624449 |
| Nadk2 | 0.00145 | -1.21148542 |
| Rnaseh1 | 0.18656 | 0.52690965 |
| Ndufb8 | 0.0021 | -1.10028984 |
| Rangrf | 0.00202 | -1.05257614 |
| Rps6ka6 | 0.11404 | 0.45449522 |
| Sun3 | 0.00144 | 2.06968787 |
| Fubp3 | 0.01646 | 0.79354295 |
| Fnbp1 | 0.09158 | 0.41702724 |
| Rsu1 | 0.32986 | 0.1638237 |
| Cdc7 | 0.00144 | 1.45516303 |
| Hdac9 | 0.70575 | 0.13558886 |
| Nup98 | 0.41605 | 0.20752555 |
| Azi2 | 0.033 | 0.48150777 |
| Dnajc19 | 0.00144 | 3.83859124 |
| Ifi27l2a | 0.49444 | 0.33914236 |
| Atp6v0a2 | 0.00581 | -0.65100295 |
| Rcn2 | 0.04392 | -1.06431775 |
| Osbpl9 | 0.0034 | 0.90542771 |
| Kdm4a | 0.23618 | -0.21092273 |
| Slc4a10 | 0.00144 | -1.30713006 |
| Lias | 0.2295 | 0.24554119 |
| Strn4 | 0.09168 | -0.35145611 |
| Prkag3 | 0.77205 | 0.05660779 |
| Metap1d | 0.00144 | 1.1754851 |
| Slc9b2 | 0.00144 | -1.30017714 |
| Musk | 0.00144 | -1.29085005 |
| Msra | 0.18378 | -0.37012522 |
| Gcat | 0.00144 | -1.53315402 |
| Mrpl9 | 0.71326 | 0.09950384 |
| Pnma5 | 0.07355 | -2.51478466 |
| Nfu1 | 0.09652 | -0.35349245 |
| Noto | 0.02442 | -0.94424971 |
| Mrpl37 | 0.01077 | -0.77693282 |
| Mto1 | 0.69642 | -0.10550272 |
| Mipep | 0.3115 | 0.54134761 |
| Fhit | 0.09034 | -0.56782654 |
| Rbp7 | 0.01765 | -0.80428472 |
| Pigx | 0.32453 | -0.28561345 |
| Prmt2 | 0.40328 | -0.2959139 |
| Zfp934 | 0.00144 | 2.84354279 |
| Esp23 | 0.00144 | -1.29085005 |
| Adam15 | 0.00144 | -1.38138233 |
| Slc38a6 | 0.01442 | 1.84564512 |
| Btg1-ps2 | 0.00149 | -1.2703832 |
| Ccdc24 | 0.61035 | -0.42754978 |
| Acsl6 | 0.01253 | 1.51464177 |
| Mageb4 | 0.00152 | -1.25878908 |
| Pusl1 | 0.05645 | -0.61856011 |
| Fam47e | 0.13556 | -0.5723857 |
| Zbtb2 | 0.1406 | 0.34316376 |
| Gal3st4 | 0.26318 | -0.38362752 |
| Rbm44 | 0.00759 | 1.48402274 |
| A630095E13Rik | 0.00145 | -1.22376446 |
| Espnl | 0.06537 | -0.70787712 |
| C78339 | 0.2792 | -0.23602099 |
| Ephx3 | 0.00284 | -0.83155141 |
| Dnajb14 | 0.89895 | -0.02333021 |
| Gm166 | 0.03322 | -0.58091199 |
| Gemin8 | 0.39744 | -0.14592634 |
| Rilpl1 | 0.48788 | 0.31442478 |
| Mmp19 | 0.50889 | -0.15923191 |
| Actr1b | 0.12482 | -0.41380402 |
| Skiv2l | 0.07724 | -0.31193167 |
| Ctage5 | 0.00144 | 1.54460723 |
| Wdr61 | 0.00755 | 0.69270445 |
| Tmem183a | 0.30646 | -0.16147366 |
| Tbl1x | 0.02094 | -0.52977706 |
| Wipi1 | 0.74367 | 0.05490887 |
| Pitpnc1 | 0.01429 | 0.67871166 |
| Cpa2 | 0.00144 | -1.28898079 |
| Fam149b | 0.03568 | 0.47232337 |
| Agfg2 | 0.00164 | -1.52972134 |
| Slc14a1 | 0.34409 | 0.26924913 |
| Mgst1 | 0.20458 | 0.31853546 |
| Degs2 | 0.17056 | -0.41520235 |
| Arid3c | 0.00144 | -1.25927895 |
| Yipf1 | 0.00465 | -0.8595644 |
| Zmynd12 | 0.45955 | -0.30865647 |
| Oxnad1 | 0.08546 | 1.01055058 |
| Ncs1 | 0.0073 | -0.8978381 |
| Tprkb | 0.93459 | 0.01428392 |
| Arpc1a | 0.01396 | -0.49469715 |
| Tmprss4 | 0.25561 | -0.69710506 |
| Ube4a | 0.1124 | 0.50510742 |
| Trip4 | 0.01204 | 0.64690498 |
| Tgfbrap1 | 0.04913 | -0.378241 |
| Ankrd55 | 0.0057 | -1.19115562 |
| Net1 | 0.00449 | 0.97131337 |
| Mtbp | 0.03781 | 0.88552308 |
| Lgi2 | 0.00548 | -1.37622101 |
| Xkr4 | 0.00145 | -1.25646236 |
| Ankrd10 | 0.74352 | -0.08665234 |
| Ppp1r3e | 0.11875 | -0.79792947 |
| Cyp2c50 | 0.00144 | -1.39243484 |
| Nagk | 0.27701 | -0.37605275 |
| Zfp568 | 0.13028 | -0.31214654 |
| AI846148 | 0.29094 | -0.57995906 |
| Aarsd1 | 0.38317 | -0.29148535 |
| Ccdc132 | 0.00144 | -1.30713006 |
| Gm17365 | 0.00144 | -1.25118919 |
| Gm17677 | 0.00144 | -1.28116048 |
| Cabp4 | 0.33238 | 0.5076136 |
| Dclre1a | 0.01435 | 0.50779033 |
| Paf1 | 0.00328 | 0.98027393 |
| Lekr1 | 0.00144 | -1.34578684 |
| Hps1 | 0.03998 | -0.41452236 |
| Ntsr1 | 0.57334 | 0.24716414 |
| Nudt5 | 0.40192 | 0.18846696 |
| Rgs3 | 0.00144 | -0.92399718 |
| Mtmr1 | 0.0088 | -0.75707614 |
| Lxn | 0.38735 | -0.14554207 |
| Abca6 | 0.04566 | 0.74154634 |
| Desi1 | 0.01199 | -0.59191091 |
| Atg9b | 0.37057 | -0.36986645 |
| Pex12 | 0.43004 | 0.1485224 |
| Erv3 | 0.56526 | -0.41606185 |
| Slc4a5 | 0.00145 | -1.22376446 |
| Slc35d2 | 0.65651 | 0.08651134 |
| Acox1 | 0.04211 | -0.40263724 |
| Slc33a1 | 0.00172 | 0.89679505 |
| Gpn1 | 0.10407 | -0.33041256 |
| Ucma | 0.00365 | -2.73535992 |
| Prr3 | 0.02027 | 0.58917951 |
| Thsd7a | 0.07973 | 0.65317693 |
| Yipf6 | 0.00144 | 0.99778056 |
| Ntng2 | 0.15736 | -1.0356384 |
| Pign | 0.01434 | 0.99445049 |
| Atp13a4 | 0.00144 | -1.29049509 |
| Maf1 | 0.05702 | -0.72348477 |
| Pqlc1 | 0.53576 | -0.18902274 |
| Gm4846 | 0.68516 | 0.19364901 |
| Nme5 | 0.02588 | 0.6388655 |
| Hps3 | 0.04011 | 0.63020888 |
| Jtb | 0.13361 | 0.51688753 |
| Lmo7 | 0.00897 | 0.75498862 |
| 1110038F14Rik | 0.01506 | -0.65614542 |
| Plcz1 | 0.00144 | -1.26301121 |
| Tbc1d24 | 0.03407 | 0.45949808 |
| Hapln1 | 0.63442 | -0.10170116 |
| Tepp | 0.00922 | -0.99645092 |
| C9 | 0.23349 | -0.88212102 |
| Atp1b2 | 0.01122 | -0.61102413 |
| Apoh | 0.25333 | -0.73101857 |
| Gm17660 | 0.00144 | -1.28116048 |
| Bri3 | 0.02906 | -0.6986641 |
| Psmb3 | 0.00197 | -1.10344452 |
| Psma4 | 0.10477 | -0.31255469 |
| Orc5 | 0.04059 | 0.77027986 |
| Adam33 | 0.20181 | 0.65532972 |
| E230008N13Rik | 0.78552 | -0.1736848 |
| Dlg5 | 0.12946 | -0.28271999 |
| Acss3 | 0.00144 | -1.27165604 |
| Fez2 | 0.05128 | -1.36067691 |
| Tbck | 0.44066 | -1.2564861 |
| Eci2 | 0.98128 | -0.00851725 |
| Phactr1 | 0.06733 | -0.55513182 |
| Gmnc | 0.00145 | -1.2388121 |
| Fxyd4 | 0.64858 | -0.38450004 |
| Enkd1 | 0.12627 | 0.66728281 |
| Tubgcp6 | 0.07805 | -0.36562046 |
| Usp2 | 0.00188 | -0.89042496 |
| Zfp804b | 0.00144 | -1.26560808 |
| Slc6a20b | 0.24736 | -0.65974715 |
| Klhl32 | 0.68199 | 0.190649 |
| Aim1l | 0.00671 | -1.43903703 |
| D630045J12Rik | 0.20386 | -0.76589352 |
| Dpp7 | 0.01229 | -0.66501105 |
| Rasl12 | 0.01681 | -0.60243327 |
| Ttk | 0.17147 | -0.51571014 |
| Nsmce4a | 0.31142 | 0.24732678 |
| Theg | 0.49435 | -0.32688995 |
| Pex11b | 0.65244 | 0.26179618 |
| Il1rapl2 | 0.00144 | -1.31495282 |
| Defb14 | 0.01064 | -1.24450462 |
| Cmtm1 | 0.00144 | -1.30017714 |
| Sdr16c5 | 0.00144 | -1.26560808 |
| Trappc12 | 0.24856 | 0.43659255 |
| Optc | 0.27997 | -0.56324494 |
| Rrbp1 | 0.40466 | 0.16190351 |
| Syt15 | 0.38214 | 0.31034387 |
| Usp54 | 0.00583 | 0.79748508 |
| Pcdhac2 | 0.09454 | -2.26543529 |
| Zfp687 | 0.01542 | -0.55871627 |
| Plb1 | 0.04908 | -0.90497976 |
| Dnajc21 | 0.00144 | 1.31327002 |
| Ralgps2 | 0.01196 | 0.87343985 |
| Fbxl5 | 0.25112 | 0.20661075 |
| Ccdc150 | 0.0077 | 0.7455122 |
| Kctd10 | 0.00458 | -0.71272964 |
| Dnajb2 | 0.14281 | -0.37896272 |
| Rps6kc1 | 0.60364 | -0.11410234 |
| Apex2 | 0.98535 | -0.00363941 |
| Ptpn3 | 0.18267 | 0.34455477 |
| 9530003J23Rik | 0.03932 | -1.38847538 |
| Ppm1l | 0.02911 | -0.50615106 |
| Zfp385b | 0.04822 | -0.36116102 |
| Fgf12 | 0.44727 | -0.31633769 |
| H13 | 0.45009 | -0.19463325 |
| H13 | 0.0204 | -0.63424502 |
| Bcas1 | 0.94007 | 0.01555794 |
| Arfip2 | 0.60029 | 0.10846467 |
| Papola | 0.01959 | 0.62990846 |
| Pfdn2 | 0.99815 | 0.00051159 |
| Pcdh11x | 0.00144 | -1.29746368 |
| Gins2 | 0.00684 | -0.6974845 |
| Sh3rf2 | 0.04632 | -0.39164898 |
| Tbce | 0.00381 | 1.01108896 |
| Casc5 | 0.58285 | 0.40370152 |
| Lyrm1 | 0.84475 | -0.04460015 |
| Abca13 | 0.00149 | -1.2703832 |
| Emc8 | 0.71031 | -0.07257982 |
| 1700065D16Rik | 0.90936 | 0.02262138 |
| Slc6a5 | 0.00144 | -1.41401027 |
| Nfe2l3 | 0.00144 | -1.28116048 |
| Edrf1 | 0.0507 | 0.52414101 |
| Stard3nl | 0.56967 | 0.13314033 |
| Yeats2 | 0.00149 | -1.08902743 |
| Arhgef40 | 0.02048 | -0.85773161 |
| Ipo4 | 0.02387 | -0.59191499 |
| Egfem1 | 0.1279 | -0.54564559 |
| Dennd2c | 0.25563 | 0.36372155 |
| Cd46 | 0.01302 | 1.36316067 |
| Ccdc60 | 0.00784 | -1.03698725 |
| Phpt1 | 0.47009 | -0.27242934 |
| Gm3259 | 0.00144 | -1.28116048 |
| Zfp955b | 0.78444 | 0.12295475 |
| Dpf3 | 0.02299 | -0.93367577 |
| Ccdc146 | 0.0057 | 1.59278863 |
| Cep57l1 | 0.00144 | 1.57293813 |
| Uhmk1 | 0.07409 | 0.59899097 |
| Spdya | 0.82912 | -0.11696905 |
| Lrrc74b | 0.02251 | 1.94140958 |
| Lvrn | 0.37331 | -0.39695028 |
| Gm933 | 0.00377 | -1.69992165 |
| Btn2a2 | 0.0019 | -1.13608632 |
| Rapgef5 | 0.79994 | 0.04860468 |
| Hivep2 | 0.05587 | 0.40981466 |
| Hira | 0.00429 | -0.69544165 |
| Heph | 0.06637 | 0.86256182 |
| Uba7 | 0.01917 | -0.56444414 |
| Slc35f5 | 0.05337 | 0.92953558 |
| Cstf3 | 0.00144 | 2.68564965 |
| A630076J17Rik | 0.00252 | -1.31286649 |
| Samd12 | 0.0692 | -0.39579315 |
| Cbx6 | 0.04622 | -0.38504828 |
| Snrnp200 | 0.02098 | -0.50521483 |
| 9430015G10Rik | 0.87012 | -0.05054378 |
| 5830411N06Rik | 0.00204 | -1.31326841 |
| Tmtc4 | 0.0925 | 0.45541662 |
| A630001G21Rik | 0.12897 | 0.67019453 |
| Rslcan18 | 0.02827 | 1.74681301 |
| BC035044 | 0.00317 | -1.11935153 |
| Nwd1 | 0.16958 | -0.5264674 |
| Snhg11 | 0.03143 | 1.67768245 |
| Zfp493 | 0.09561 | 0.84704011 |
| Pou6f1 | 0.06234 | -0.41779534 |
| Setd5 | 0.86282 | -0.03201141 |
| Zbtb38 | 0.27074 | -0.29159622 |
| Patz1 | 0.00702 | -0.67025758 |
| Pdzd11 | 0.3016 | -0.22544769 |
| Zcchc11 | 0.00415 | 1.00986413 |
| 1700112E06Rik | 0.01635 | -0.62139176 |
| H2-M5 | 0.00144 | -1.25118919 |
| U2surp | 0.00165 | 1.26954577 |
| Fam163b | 0.8831 | -0.04655371 |
| Plekhb2 | 0.15915 | -0.72568929 |
| Tbc1d7 | 0.39108 | -0.22930294 |
| Scn3b | 0.51287 | -0.20974485 |
| Ccpg1 | 0.02143 | 0.60562064 |
| Slc37a3 | 0.15337 | -0.25465462 |
| Cmas | 0.31033 | 0.1982582 |
| Cyp2c55 | 0.08227 | -0.76663393 |
| Cd84 | 0.00261 | 0.99877165 |
| Cyp4f18 | 0.36181 | -0.26695003 |
| Cd247 | 0.00144 | -1.87089815 |
| Kif27 | 0.00853 | -1.19218073 |
| Cd7 | 0.00582 | -1.5007705 |
| Arhgap26 | 0.02514 | 0.52667805 |
| Eif2b3 | 0.96125 | 0.00913594 |
| Mdfic | 0.03636 | 0.75723955 |
| Lrwd1 | 0.00305 | -0.85754317 |
| Glyctk | 0.08754 | -0.39460053 |
| Gm38425 | 0.02632 | -1.27592915 |
| Mcm9 | 0.99727 | 0.00221378 |
| Ap3s1 | 0.0049 | 0.68396432 |
| Cul5 | 0.27383 | 0.20009882 |
| Rcbtb1 | 0.00144 | 1.26426934 |
| Noxred1 | 0.00581 | 2.11197373 |
| Spata24 | 0.01293 | -0.54645129 |
| Adam3 | 0.00144 | -1.26560808 |
| Csnk1g1 | 0.77709 | 0.0599852 |
| Zfp61 | 0.57072 | 0.13601377 |
| Cnbd2 | 0.07616 | -0.82901203 |
| Akr1cl | 0.00176 | -1.13860941 |
| Yes1 | 0.00144 | 1.81080097 |
| Ggnbp1 | 0.00341 | 1.44183151 |
| Nrsn1 | 0.00144 | -1.27165604 |
| Fam124a | 0.71167 | -0.07735419 |
| Ube2b | 0.18099 | 0.25921338 |
| Ube2e3 | 0.33861 | 0.28728135 |
| Cbfa2t2 | 0.83759 | 0.04280137 |
| Serpinb13 | 0.00144 | -1.27165604 |
| Ube2cbp | 0.21023 | 0.72866423 |
| Pigw | 0.0242 | 0.68649682 |
| Ly6g6e | 0.00144 | -1.41401027 |
| Btbd7 | 0.17257 | 0.26873906 |
| Ankrd60 | 0.03132 | -0.97953315 |
| Kctd13 | 0.50212 | 0.55552697 |
| Naa25 | 0.04986 | 0.38354047 |
| Nabp2 | 0.85463 | 0.07937744 |
| Med6 | 0.06909 | 0.33914864 |
| Ssr4 | 0.01514 | -0.77871373 |
| A630095N17Rik | 0.01769 | -2.2307897 |
| Siah1a | 0.51559 | -0.10329938 |
| 2300009A05Rik | 0.08376 | -0.6136755 |
| Dnajc8 | 0.59425 | 0.08364139 |
| Bcl11a | 0.00933 | -0.75686392 |
| Galnt18 | 0.01248 | -0.60567399 |
| 2210018M11Rik | 0.00144 | 1.08241188 |
| Tmem258 | 0.00334 | -1.06066759 |
| Pxmp2 | 0.79177 | -0.06002748 |
| Thap7 | 0.03583 | -0.86972949 |
| Wdr53 | 0.05089 | 0.53767634 |
| Ptges3l | 0.35111 | -0.21979261 |
| Zfp644 | 0.00144 | 1.12006591 |
| Ofcc1 | 0.00144 | -1.48026729 |
| Serhl | 0.6962 | -0.17059085 |
| Uckl1 | 0.08437 | -0.55262358 |
| Anxa9 | 0.72519 | -0.13213218 |
| Zfp655 | 0.23566 | 0.23060666 |
| Slc35a2 | 0.03481 | -0.5083559 |
| Dennd1c | 0.07155 | 1.15112826 |
| Gabarapl2 | 0.55246 | -0.13188328 |
| Pde6d | 0.97199 | -0.01382405 |
| Agpat4 | 0.0104 | -0.68850642 |
| Tmbim4 | 0.58787 | -0.17690439 |
| Rbm45 | 0.04079 | -1.12747294 |
| Fam135a | 0.03011 | 0.88745633 |
| Cgnl1 | 0.02476 | 0.62080387 |
| Gpn3 | 0.83654 | 0.04755331 |
| Rapgef4 | 0.00269 | 1.52029282 |
| Akirin1 | 0.14406 | 0.26859891 |
| Pxt1 | 0.00688 | -1.61145093 |
| Gm7030 | 0.00149 | -1.22582153 |
| Gtpbp10 | 0.1809 | 0.24031924 |
| Gm17252 | 0.00144 | -1.29323526 |
| Myo16 | 0.03297 | -0.98564376 |
| AW551984 | 0.89059 | -0.03731565 |
| Nupl2 | 0.19855 | 0.28398752 |
| Wdr83 | 0.01548 | -0.52516066 |
| Zkscan2 | 0.1912 | 0.35978357 |
| Asmt | 0.16243 | -0.62713896 |
| Ldhb | 0.12671 | -0.31180952 |
| Lrch3 | 0.00289 | 0.9223898 |
| BC049352 | 0.42575 | 0.22532359 |
| Hcfc2 | 0.15162 | 0.27612574 |
| 4930430A15Rik | 0.03228 | -1.69749437 |
| Dhdds | 0.00336 | -0.9705353 |
| Gucy2g | 0.00159 | -1.26431393 |
| Cylc1 | 0.28776 | 0.42799353 |
| Phactr2 | 0.00144 | 2.07952842 |
| Efcab3 | 0.00144 | -1.29049509 |
| Tmco5 | 0.01877 | -1.04950201 |
| Cwc27 | 0.00212 | 1.5876229 |
| H2-Bl | 0.00478 | -1.77159571 |
| Guk1 | 0.02031 | -0.78633693 |
| Ddx43 | 0.00145 | -1.22376446 |
| Eif4e2 | 0.00466 | -0.83635045 |
| Rnaseh2b | 0.00404 | 0.82424153 |
| Brdt | 0.00312 | -0.95682423 |
| Dnajb11 | 0.64067 | 0.07536279 |
| Cand2 | 0.80377 | -0.09958525 |
| Fut1 | 0.00145 | -1.26219879 |
| Upf3a | 0.00249 | 0.76203488 |
| Gimap8 | 0.00144 | 2.91451492 |
| Abi1 | 0.00144 | 1.4177123 |
| Smarcd3 | 0.31063 | -0.32148859 |
| Fgf3 | 0.01266 | -1.38269311 |
| Agbl4 | 0.31058 | -0.49377024 |
| Cdca7 | 0.9705 | 0.01211395 |
| Wfdc18 | 0.06569 | -0.48566807 |
| Gpm6b | 0.02667 | 1.06228448 |
| Rpgr | 0.00144 | -1.60245164 |
| Rpgr | 0.00373 | -1.08684252 |
| Eps15 | 0.00607 | 1.23512352 |
| Lztr1 | 0.08769 | -0.35722515 |
| Lrrc72 | 0.80676 | -0.12603 |
| Dpagt1 | 0.81311 | 0.10591048 |
| Map2k7 | 0.0891 | -0.42499813 |
| Nlrp1b | 0.00679 | 0.99361749 |
| Cyp2b19 | 0.00144 | -1.27165604 |
| Gemin2 | 0.53037 | 0.11090703 |
| Aste1 | 0.37411 | 0.16926095 |
| Pdzd9 | 0.00145 | -1.2388121 |
| Rnf181 | 0.36929 | -0.19627365 |
| Pstk | 0.40228 | 0.22442719 |
| Piezo2 | 0.01867 | 1.37240223 |
| Psmd6 | 0.41093 | 0.16283062 |
| Ergic3 | 0.24484 | -0.37405955 |
| Esp8 | 0.00631 | -2.07119431 |
| Ece2 | 0.02855 | -0.7308946 |
| Asb1 | 0.01926 | -0.5329257 |
| Zfp964 | 0.00144 | -1.24353547 |
| Gm7861 | 0.11783 | -1.86866063 |
| Vta1 | 0.02077 | 0.62401118 |
| EU599041 | 0.00144 | 2.17486143 |
| Negr1 | 0.03107 | 1.06866284 |
| Gngt2 | 0.00144 | -0.98409338 |
| Marveld2 | 0.11967 | 0.67704929 |
| Gnpda2 | 0.00144 | 1.20943955 |
| Cnot2 | 0.00144 | 1.3580712 |
| Spn | 0.30627 | 0.3259433 |
| Eif4e1b | 0.00267 | -1.38470183 |
| Prmt6 | 0.89483 | -0.03370955 |
| Rasgrf2 | 0.09668 | 0.58420216 |
| Pcgf5 | 0.4666 | -0.13933626 |
| Scmh1 | 0.82487 | -0.08934102 |
| Tdpoz2 | 0.00144 | -1.20555712 |
| Zfp82 | 0.1199 | -0.75029278 |
| Nrf1 | 0.0427 | -1.99774049 |
| Rnf223 | 0.95258 | 0.0445124 |
| Rp1 | 0.04897 | -2.06655745 |
| 6820408C15Rik | 0.47354 | -0.2744736 |
| Naip7 | 0.00144 | -1.30253725 |
| L3mbtl2 | 0.13122 | -0.36973873 |
| Ntn5 | 0.82068 | -0.16855786 |
| Kynu | 0.03115 | -0.9700101 |
| Gm15881 | 0.00144 | -1.30017714 |
| Esp38 | 0.00144 | -1.20555712 |
| Esp18 | 0.00144 | -1.29746368 |
| Esp34 | 0.00144 | -1.28116048 |
| Esp4 | 0.0047 | -1.48780017 |
| Esp3 | 0.98701 | -0.01804875 |
| Esp15 | 0.60467 | -0.58628547 |
| Esp36 | 0.00145 | -1.34759674 |
| Esp16 | 0.00589 | -1.11030563 |
| Esp31 | 0.00144 | -1.30017714 |
| Ascl5 | 0.36856 | -0.57160439 |
| Gm14692 | 0.00149 | -1.2703832 |
| Vmn1r187 | 0.00159 | -1.26431393 |
| Gm17689 | 0.00144 | -1.3199115 |
| 4930432M17Rik | 0.02827 | -1.85682098 |
| Olfr1414 | 0.16665 | -0.56978714 |
| Teddm2 | 0.00144 | -0.94758258 |
| Gm14478 | 0.91946 | 0.03576097 |
| H2-L | 0.00185 | -1.08330699 |
| Hist1h2bq | 0.00145 | 1.35283096 |
| Olfr976 | 0.00144 | -1.27699683 |
| Vmn1r61 | 0.07291 | -1.02209275 |
| Vmn1r88 | 0.00299 | -1.53359264 |
| Gm20594 | 0.00501 | -0.78238848 |
| Gm14477 | 0.00566 | 0.78813945 |
| Gm11562 | 0.00144 | -1.27454685 |
| Krtap27-1 | 0.00144 | -1.27165604 |
| A530032D15Rik | 0.88289 | 0.05469076 |
| Snap23 | 0.00144 | 1.63610761 |
| 4930449E01Rik | 0.0124 | -1.40874825 |
| 5031434C07Rik | 0.00451 | 1.51398049 |
| 4930579D07Rik | 0.00145 | -1.22376446 |
| 9530082P21Rik | 0.04556 | -0.68757077 |
| Srp54a | 0.68528 | 0.09038335 |
| Zfp3 | 0.34278 | -0.57818103 |
| B3galt6 | 0.16883 | -0.4476095 |
| Ttc21b | 0.72965 | -0.11422889 |
| 4930402H24Rik | 0.01978 | -0.63447151 |
| Zmym5 | 0.01547 | 1.05127572 |
| Zfp398 | 0.40932 | -0.1495335 |
| Taf1 | 0.01592 | -0.78383408 |
| Nsf | 0.01729 | -0.47559702 |
| Fam118b | 0.16973 | -0.27439408 |
| Guf1 | 0.00144 | 1.51596647 |
| Hykk | 0.09655 | -1.23272556 |
| Wdfy3 | 0.04345 | -0.65406985 |
| G2e3 | 0.35493 | 0.26805749 |
| Klhl20 | 0.00225 | 0.92115823 |
| Amn1 | 0.89211 | 0.02465209 |
| Alms1 | 0.23278 | 0.53267885 |
| Gmeb1 | 0.66817 | 0.09448432 |
| Mgea5 | 0.00803 | -0.94130422 |
| Smim8 | 0.35564 | 0.20212225 |
| N4bp2 | 0.01166 | -0.71626742 |
| Gls | 0.06211 | 0.617704 |
| Mdn1 | 0.26782 | -0.3678017 |
| Asxl2 | 0.04517 | -0.58930703 |
| Pcdhga5 | 0.008 | 0.90465623 |
| Samd8 | 0.01755 | -0.57166796 |
| Pcdhga1 | 0.00394 | 1.0392085 |
| Gabpb2 | 0.03577 | 0.48193146 |
| Wdhd1 | 0.00676 | -0.78557152 |
| Usp24 | 0.06567 | 0.67664549 |
| R74862 | 0.07689 | 0.76644139 |
| Ifnar1 | 0.59566 | -0.14037799 |
| 8430431K14Rik | 0.32282 | 0.62812431 |
| Xpo7 | 0.00144 | 2.41591963 |
| Etl4 | 0.26287 | 0.38156881 |
| Cep295 | 0.00144 | 1.51845201 |
| Tcf12 | 0.01562 | -0.619123 |
| Bloc1s6 | 0.45637 | 0.11662823 |
| Prr14l | 0.33519 | 0.17323374 |
| Srpk2 | 0.01978 | -0.6499925 |
| Dido1 | 0.01909 | -0.80028642 |
| Zbtb8b | 0.00144 | -1.30017714 |
| Fam35a | 0.00807 | -0.89804642 |
| Timmdc1 | 0.47409 | -0.27515878 |
| Atad2 | 0.72966 | 0.09892965 |
| 2610301B20Rik | 0.06615 | 0.55292538 |
| Lrrc2 | 0.0034 | 1.43664049 |
| Retn | 0.3622 | -0.81692331 |
| Unc13c | 0.00145 | -1.22376446 |
| Agl | 0.10234 | 0.34152951 |
| 3110068a07rik | 0.11397 | -0.58331225 |
| 4921525O09Rik | 0.03447 | -1.01182033 |
| C430002N11Rik | 0.19858 | -0.52883489 |
| D530017H19Rik | 0.66916 | 0.21691441 |
| 1500004A13Rik | 0.06554 | 0.77224167 |
| Prrc2c | 0.03093 | 1.24249401 |
| A830080H07Rik | 0.00144 | -1.25927895 |
| C530043K16Rik | 0.12099 | 0.65032074 |
| D030036P13Rik | 0.6578 | -0.11498046 |
| D030036P13Rik | 0.69984 | -0.08720226 |
| 1700017I07Rik | 0.0985 | -2.67315011 |
| 4932443L11Rik | 0.00879 | -1.19143842 |
| D030063E12 | 0.00299 | 1.2959064 |
| Nhsl2 | 0.0736 | 0.53404812 |
| C78228 | 0.75399 | 0.2618008 |
| 1300014J16Rik | 0.20063 | -0.49679836 |
| 4933436F18Rik | 0.23232 | -2.26814359 |
| Atg9a | 0.12969 | -0.525342 |
| AA763515 | 0.00144 | -1.30713006 |
| 4930543D07Rik | 0.00144 | -1.27165604 |
| Nrg3os | 0.41893 | -0.45510257 |
| Ttn | 0.24248 | 0.19044626 |
| 2900057C01Rik | 0.00679 | 2.11137251 |
| Eya1 | 0.02746 | -0.52125887 |
| B230369F24Rik | 0.63182 | -0.11951275 |
| B230369F24Rik | 0.21616 | 0.25945636 |
| 4932416J16Rik | 0.01462 | -1.67969507 |
| AI464196 | 0.17026 | 0.37535205 |
| 4921523P09Rik | 0.00144 | -1.29085005 |
| 2900072G11Rik | 0.01328 | -1.00087525 |
| Fezf1 | 0.99252 | -0.00531919 |
| BC025933 | 0.94032 | 0.04875915 |
| 4930517G19Rik | 0.0168 | 1.07129126 |
| AW050000 | 0.05629 | -1.01017398 |
| 4933408J17Rik | 0.0101 | -0.98680942 |
| B630006N21Rik | 0.0236 | -1.48541675 |
| 6330575P09Rik | 0.49496 | -0.1758534 |
| 2900016J10Rik | 0.83843 | 0.06132389 |
| Igip | 0.01023 | 0.86921757 |
| C030014L02 | 0.00144 | -1.5371449 |
| Srek1 | 0.01024 | 2.07089553 |
| Abhd11os | 0.00144 | -1.53333327 |
| D17Ertd648e | 0.00145 | -1.2388121 |
| Ptpn4 | 0.12133 | 0.98191977 |
| 4932412D23Rik | 0.00144 | -1.55087898 |
| 4933435F18Rik | 0.00144 | -1.28116048 |
| Ccny | 0.0916 | -0.31578677 |
| 1110028F11Rik | 0.00144 | -1.29085005 |
| 4732460I02Rik | 0.00857 | 2.70658079 |
| 1110006E14Rik | 0.00158 | 1.95116203 |
| 1110006E14Rik | 0.04564 | 0.70952738 |
| Snhg9 | 0.00144 | -1.26560808 |
| 8430419K02Rik | 0.0049 | -1.08617297 |
| 9330188P03Rik | 0.00622 | 0.76186473 |
| 4930527F14Rik | 0.00144 | -1.28898079 |
| D230034L24Rik | 0.11833 | 0.52371212 |
| AU021884 | 0.00144 | -1.31495282 |
| C76472 | 0.00145 | -1.24191372 |
| LOC102640811 | 0.32311 | -0.31357075 |
| BC031361 | 0.0614 | 0.7555852 |
| AW125324 | 0.00828 | 0.87957889 |
| Prrx2 | 0.00193 | -1.17569177 |
| E130215H24Rik | 0.19531 | 0.25817678 |
| I830077J02Rik | 0.45441 | 0.37268858 |
| AU024581 | 0.01673 | -0.5109127 |
| Tmem175 | 0.00144 | 1.87501614 |
| 1700084F23Rik | 0.00145 | -1.2388121 |
| 1700099I09Rik | 0.00144 | -1.28898079 |
| 5033428I22Rik | 0.00149 | -1.22582153 |
| Adgrl3 | 0.00145 | -1.26219879 |
| 4930517J16Rik | 0.78206 | 0.15250536 |
| Gm2379 | 0.00717 | -1.0591794 |
| Grip1os1 | 0.20845 | -0.58519376 |
| 4930592A05Rik | 0.01099 | -1.65235141 |
| Olfr29-ps1 | 0.01426 | -1.27302807 |
| Mta1 | 0.42292 | -0.25072832 |
| Luc7l | 0.05897 | 0.55723876 |
| 4933411B09Rik | 0.14298 | -0.72330833 |
| 4732463B04Rik | 0.00144 | -1.24811894 |
| C630004M23Rik | 0.00144 | -1.29051721 |
| 9030613N10Rik | 0.00182 | -1.65709301 |
| LOC102634333 | 0.23878 | -0.22228887 |
| AW552393 | 0.00836 | -1.3083897 |
| 1700109G14Rik | 0.00144 | -1.27165604 |
| Vwa2 | 0.00144 | -1.22925777 |
| 1700048M11Rik | 0.08027 | 1.31695635 |
| Gm10404 | 0.99667 | -0.00141475 |
| C130090I23Rik | 0.36452 | -0.26973445 |
| 4933427I22Rik | 0.00144 | -1.25118919 |
| 5830468K08Rik | 0.03781 | 0.94724681 |
| Tro | 0.00144 | -1.31884045 |
| Tcrb-J | 0.07093 | -0.9597901 |
| AW111846 | 0.00938 | -1.612976 |
| 4930581F22Rik | 0.96642 | -0.0114821 |
| A830052D11Rik | 0.30116 | -0.76234552 |
| B230213L16Rik | 0.12508 | 0.51032336 |
| AI596198 | 0.00144 | 2.01486592 |
| 4930540E01Rik | 0.00144 | -1.3826329 |
| C030037D09Rik | 0.92926 | -0.02627337 |
| LOC105242755 | 0.0049 | -1.11996532 |
| 4930434J08Rik | 0.00144 | -1.27454685 |
| A430061O12Rik | 0.09963 | 0.96632326 |
| 9330156P08Rik | 0.00252 | 1.90980511 |
| Glb1l | 0.10886 | -0.52234686 |
| 2610028D06Rik | 0.46239 | -0.30426644 |
| Gm20556 | 0.00149 | -1.22582153 |
| R3hdm1 | 0.08134 | 0.83349865 |
| 9130230N09Rik | 0.0418 | -0.40423662 |
| Luc7l2 | 0.96692 | 0.01167904 |
| 4932429P19Rik | 0.27341 | -0.46664354 |
| 9030624J02Rik | 0.06063 | -0.53068225 |
| Gm16508 | 0.01347 | -1.01042984 |
| BB163080 | 0.00163 | 2.29197172 |
| 7630403G23Rik | 0.00145 | -1.2388121 |
| 4933406K04Rik | 0.07233 | -1.2232838 |
| Mtx3 | 0.0156 | -0.89368237 |
| 4933439C10Rik | 0.15186 | -0.47182521 |
| 4930556C24Rik | 0.00144 | -1.28116048 |
| Kcnq1ot1 | 0.08233 | 0.93773903 |
| BB166591 | 0.00144 | 2.56868815 |
| 4930412F09Rik | 0.00144 | -1.29049509 |
| 4930431P03Rik | 0.46356 | -0.33450361 |
| Gm12945 | 0.00144 | -1.31518407 |
| F630042J09Rik | 0.0028 | -1.16569658 |
| AI449212 | 0.00149 | -2.06510713 |
| Tomm20l | 0.33335 | 0.79299439 |
| 9130001E16Rik | 0.10023 | -0.85867646 |
| Fam92a | 0.11852 | 0.57823418 |
| Toporsos | 0.00158 | -1.2446827 |
| AA416453 | 0.51532 | 0.66819789 |
| Lix1 | 0.90562 | -0.02346268 |
| AU024546 | 0.00144 | -1.25118919 |
| Mboat2 | 0.44739 | -0.23443167 |
| E230008O15Rik | 0.00144 | 3.04878492 |
| M5C1000I18Rik | 0.92748 | 0.06428788 |
| Platr4 | 0.00144 | -1.26301121 |
| 1700061N14Rik | 0.16927 | -0.49559404 |
| Iqch | 0.00145 | -1.27532353 |
| 2210406H18Rik | 0.2221 | -0.5910307 |
| LOC552909 | 0.0035 | 1.22902585 |
| Atcayos | 0.1143 | 0.31156924 |
| 1700001L05Rik | 0.00144 | 2.17892457 |
| 4930435N07Rik | 0.01247 | -1.37797036 |
| 4930545L23Rik | 0.12847 | -2.31163725 |
| 4933430N04Rik | 0.0051 | -1.02851644 |
| D230014I24Rik | 0.30666 | -0.38165208 |
| 1700092C02Rik | 0.91803 | -0.04589027 |
| 1700039O17Rik | 0.00144 | -1.51183838 |
| Gm10389 | 0.05249 | -0.82512166 |
| BC037032 | 0.91505 | -0.01962631 |
| AI451458 | 0.03716 | 0.98151771 |
| 4930548J01Rik | 0.00144 | -1.20555712 |
| B430203I24Rik | 0.02895 | 1.85211477 |
| Pam | 0.01545 | 0.95272001 |
| A330049N07Rik | 0.00145 | -1.25646236 |
| Gm15850 | 0.00149 | -1.28183002 |
| A530083I20Rik | 0.00145 | -1.2388121 |
| D030062O11Rik | 0.00144 | -1.31495282 |
| Mad2l2 | 0.37333 | 0.33682949 |
| 4930432J09Rik | 0.00144 | -1.30713006 |
| Hnrnpr | 0.00752 | 2.53872081 |
| AU015680 | 0.71722 | 0.15546146 |
| BE949265 | 0.45913 | -0.2468597 |
| Rel | 0.45718 | 0.15094909 |
| 4930422I22Rik | 0.08172 | -2.0891601 |
| 4930558N01Rik | 0.0122 | -0.73536524 |
| B230110C06Rik | 0.04314 | 0.91897458 |
| B230110C06Rik | 0.58034 | -0.23048594 |
| 6430500D05Rik | 0.00144 | -1.26153594 |
| Arhgap21 | 0.00564 | 0.7901944 |
| Lrch2 | 0.00715 | 1.27516199 |
| D7Ertd661e | 0.07382 | -0.84757432 |
| Fgf12 | 0.00145 | -1.22376446 |
| 4930402F11Rik | 0.13993 | -1.14535237 |
| 5930409G06Rik | 0.0054 | -1.1593491 |
| B930025P03Rik | 0.32275 | 0.43810497 |
| 4930512B01Rik | 0.85918 | -0.0718911 |
| Lelp1 | 0.11885 | -0.67335787 |
| D2Ertd640e | 0.20291 | 0.62854585 |
| Rnf112 | 0.03206 | -1.31286649 |
| 4833428L15Rik | 0.00144 | -1.26560808 |
| 4930505O19Rik | 0.0057 | -1.19115562 |
| Astn2 | 0.00147 | -1.53332605 |
| 5033423O07Rik | 0.00299 | 2.1358476 |
| B130024M06Rik | 0.03374 | -0.85417504 |
| 4930465M20Rik | 0.00144 | -1.30784197 |
| Car10 | 0.00149 | -1.2703832 |
| AI428406 | 0.59896 | -0.69432178 |
| B930025B16Rik | 0.38011 | 0.3760056 |
| AW987390 | 0.01496 | -1.98967077 |
| D130046C19Rik | 0.01786 | 2.68015054 |
| B430316J06Rik | 0.00316 | 2.70660576 |
| Skida1 | 0.14083 | -0.88511728 |
| 4930480K15Rik | 0.00144 | -1.26560808 |
| 1810073O08Rik | 0.21366 | -0.26564455 |
| A730056I06Rik | 0.01591 | -0.90417085 |
| 4930473H19Rik | 0.00776 | 0.99760112 |
| D2Ertd295e | 0.056 | 0.8278669 |
| 2810427A07Rik | 0.04842 | -2.95271187 |
| AU017263 | 0.00144 | -1.28116048 |
| 1700015C15Rik | 0.00144 | -1.29049509 |
| AU022702 | 0.00149 | -1.25408 |
| AA408954 | 0.00144 | -1.32313902 |
| 4930429F11Rik | 0.01242 | -0.98748982 |
| 4930488N15Rik | 0.00149 | -1.22582153 |
| Gm38458 | 0.00144 | -1.34945434 |
| 1700023A20Rik | 0.05116 | -0.84810684 |
| Slc16a7 | 0.05405 | 0.65785325 |
| 1700120O09Rik | 0.00144 | -1.29085005 |
| 4933431M02Rik | 0.00144 | -1.28116048 |
| 4933431M02Rik | 0.00145 | -1.25646236 |
| 6430584L05Rik | 0.39577 | -0.17295756 |
| A430106G13Rik | 0.03206 | 1.53349531 |
| K230015D01Rik | 0.00415 | -0.90487314 |
| 9330159F19Rik | 0.6477 | -0.21926598 |
| BC055308 | 0.00253 | 1.43700834 |
| D430047D06Rik | 0.00144 | -1.30017714 |
| Gm12108 | 0.00144 | -1.29085005 |
| 4931407E12Rik | 0.40178 | 0.38466115 |
| AI449595 | 0.00207 | -1.12931932 |
| 4933415J04Rik | 0.72 | 0.15667297 |
| Ankle2 | 0.01015 | -0.65837879 |
| AI195381 | 0.00177 | -1.35765442 |
| Cep128 | 0.13505 | 0.53478943 |
| C230014O12Rik | 0.52833 | -0.27274985 |
| A330015K06Rik | 0.00969 | -1.02369437 |
| 4933430L12Rik | 0.69211 | -0.22988663 |
| 4933406J10Rik | 0.36649 | -0.62871068 |
| 2310009A05Rik | 0.72119 | 0.24495482 |
| 1810064F22Rik | 0.28337 | -0.59273619 |
| 4930448A20Rik | 0.00144 | -1.29085005 |
| 4930551I15Rik | 0.00149 | -1.22582153 |
| 5330411O13Rik | 0.1253 | -1.20907166 |
| A830035O19Rik | 0.13769 | -0.62631038 |
| 9430014N10Rik | 0.00144 | -1.30713006 |
| AA517446 | 0.07608 | -2.20850553 |
| D130061D10Rik | 0.04151 | 1.07844125 |
| 5430437J10Rik | 0.00144 | -1.28116048 |
| Gm15688 | 0.00144 | -1.28898079 |
| C430019N01Rik | 0.00876 | -1.11958238 |
| 1700003G18Rik | 0.81817 | 0.06630136 |
| Gm26808 | 0.00144 | -0.98193853 |
| Gm5089 | 0.00145 | -1.22376446 |
| 4731419I09Rik | 0.00144 | -1.28116048 |
| Gm10804 | 0.00144 | -1.26934956 |
| 4933412L11Rik | 0.00144 | -1.28116048 |
| 9130221J18Rik | 0.04721 | -2.09859523 |
| 9130208D14Rik | 0.13672 | -0.668753 |
| 1700041M19Rik | 0.00144 | -1.26560808 |
| 2700005E23Rik | 0.00144 | -1.3723916 |
| LOC102640241 | 0.2537 | -0.79468327 |
| 4930426I24Rik | 0.05139 | -1.34604504 |
| 4930471M09Rik | 0.57367 | -0.71579129 |
| 2310011E23Rik | 0.00144 | -1.20555712 |
| 2900024J01Rik | 0.46488 | -0.34054273 |
| AU023639 | 0.00717 | -1.0591794 |
| Gm9958 | 0.10759 | -1.36056372 |
| Cep63 | 0.22722 | 0.65262131 |
| Mettl25 | 0.00144 | -1.27165604 |
| D130062J10Rik | 0.13118 | 0.64114357 |
| Ceacam2 | 0.01867 | 0.81008226 |
| 9630039A02Rik | 0.00144 | -1.27585605 |
| 0610009K14Rik | 0.10956 | -1.26779338 |
| G370062D05Rik | 0.00144 | -1.30713006 |
| 4933431K23Rik | 0.48797 | 0.22530432 |
| A130014A01Rik | 0.01482 | 0.56302587 |
| 6720482D04 | 0.09396 | -0.66728123 |
| 4930518C09Rik | 0.00145 | -1.22376446 |
| Ankrd33b | 0.38622 | 0.20637525 |
| 4930425O10Rik | 0.97164 | 0.0100546 |
| 4933438B17Rik | 0.2913 | -0.50384004 |
| C81489 | 0.05474 | -0.83674251 |
| 8030453O22Rik | 0.47859 | -0.37897797 |
| 8030453O22Rik | 0.14788 | -0.49710113 |
| 2810040C05Rik | 0.0178 | 1.43648419 |
| 1700123I01Rik | 0.00144 | -1.29746368 |
| Gdap5 | 0.00959 | -1.14324664 |
| 2310001H17Rik | 0.52282 | -0.2987313 |
| 6820445E23Rik | 0.5445 | 0.35891073 |
| 5430402P08Rik | 0.00144 | -1.28898079 |
| AI314604 | 0.05331 | 1.24315248 |
| C030017G13Rik | 0.59357 | -0.35296456 |
| AI845619 | 0.15268 | -0.61836363 |
| 4933435E02Rik | 0.82275 | -0.13526589 |
| BC035044 | 0.35393 | -0.50418467 |
| 4930406D14Rik | 0.55061 | 0.17462988 |
| 6430706H07Rik | 0.43102 | 0.28371595 |
| 9630013K17Rik | 0.00144 | -1.29051721 |
| LOC105243204 | 0.00286 | 2.58689312 |
| A330106F07Rik | 0.00245 | 2.76177228 |
| Lias | 0.90519 | 0.03993674 |
| Nalcn | 0.33911 | -0.79743914 |
| Gm2115 | 0.54516 | -0.28082588 |
| 5430440L12Rik | 0.00236 | -0.96031125 |
| Gm4221 | 0.00144 | -1.31495282 |
| AI844869 | 0.29955 | 0.25874627 |
| LOC102633312 | 0.00406 | -1.08384626 |
| D130050E23Rik | 0.30246 | 0.75121402 |
| BC037438 | 0.24076 | -0.44212925 |
| Gm29684 | 0.00151 | -1.17792995 |
| Tspy-ps | 0.14512 | -3.52931385 |
| LOC553096 | 0.76767 | 0.10323328 |
| 4930509J09Rik | 0.00144 | -1.52957155 |
| Kcnma1 | 0.011 | -1.6570291 |
| 4933439N14Rik | 0.00144 | -1.28898079 |
| 2310068J16Rik | 0.40634 | -0.20664684 |
| Olfr1300-ps1 | 0.00144 | -1.42249344 |
| 4930435M08Rik | 0.0437 | -1.01963123 |
| AI428301 | 0.00286 | -1.71624359 |
| 4933409L14Rik | 0.62764 | 0.87104813 |
| G630030J09Rik | 0.00261 | -1.11284335 |
| 4930502C17Rik | 0.00144 | -1.30713006 |
| Igkv4-72 | 0.00144 | -1.21455352 |
| 4930432F04Rik | 0.33878 | 0.39066909 |
| 4930521G14Rik | 0.03645 | -1.78034564 |
| 1810044D09Rik | 0.47773 | 0.34112247 |
| Gm12018 | 0.13543 | -1.07989287 |
| 1110035E04Rik | 0.23684 | 0.6902319 |
| 2610027H17Rik | 0.00144 | -1.20030135 |
| A930007I19Rik | 0.00285 | -1.33107382 |
| 4921533I20Rik | 0.0475 | -0.92312902 |
| P2rx3 | 0.71083 | 0.21915218 |
| AI225934 | 0.07798 | 0.815218 |
| F730016J06Rik | 0.00144 | -1.25118919 |
| 8430428J23Rik | 0.21778 | -0.44503367 |
| D9Ertd788e | 0.02045 | -1.29931916 |
| A230001M10Rik | 0.00144 | -1.26934956 |
| A130071D04Rik | 0.65925 | -0.18369968 |
| 9530085L11Rik | 0.94133 | 0.04695257 |
| Spag17os | 0.01952 | -1.06874756 |
| 4930542N06Rik | 0.00144 | -1.28898079 |
| A730009E18Rik | 0.39146 | 0.50757082 |
| Tmem147os | 0.78593 | 0.11915452 |
| 2310058N22Rik | 0.01039 | 1.33446866 |
| B430319H21Rik | 0.00402 | -1.17452065 |
| A330072L02Rik | 0.01094 | -1.11366036 |
| Pacrgl | 0.01216 | 1.54127604 |
| Lhx4 | 0.49338 | 0.36952417 |
| 9030607L02Rik | 0.49985 | 0.32827901 |
| Zim3 | 0.11397 | -0.5012814 |
| A930001A20Rik | 0.00535 | -1.93967261 |
| Nfat5 | 0.37466 | 0.6142046 |
| 4833441D16Rik | 0.10897 | -1.87919698 |
| Gcap8 | 0.1456 | -0.81407411 |
| A430046D13Rik | 0.48549 | 0.13868595 |
| AA419673 | 0.53236 | -0.47237494 |
| 4930413E15Rik | 0.32732 | -0.51282929 |
| 9030601B04Rik | 0.00662 | 0.91954552 |
| C80171 | 0.04904 | -0.90199263 |
| Kcnip3 | 0.24851 | -0.49907374 |
| Sox2ot | 0.01311 | -1.60728483 |
| C030009J22Rik | 0.00144 | -1.29085005 |
| D4Ertd335e | 0.00145 | -1.21051401 |
| AA409749 | 0.00144 | -1.29051721 |
| D1Ertd704e | 0.26436 | 0.46170732 |
| 8430406P12Rik | 0.00653 | 0.70587863 |
| Syn3 | 0.65512 | -0.33816761 |
| 2810429I04Rik | 0.00145 | -1.15845772 |
| 1700025K04Rik | 0.00144 | -1.22925777 |
| Gm10623 | 0.00144 | -1.27699683 |
| C130009A20Rik | 0.01205 | 1.53543985 |
| Ano5 | 0.01685 | -1.08368725 |
| 4930502C15Rik | 0.00144 | -1.28116048 |
| Gm5919 | 0.12454 | -1.30765707 |
| 9530018H14Rik | 0.03653 | -0.61976819 |
| C430049E01Rik | 0.00254 | -1.22203309 |
| Nr1h4 | 0.69703 | -0.08946615 |
| 1700066O22Rik | 0.03911 | -2.11978786 |
| Gm17739 | 0.08834 | -0.49536242 |
| C230037E05Rik | 0.05309 | 1.24172574 |
| Gm10741 | 0.03604 | -0.74446497 |
| C330018A13Rik | 0.00144 | -1.30713006 |
| 2010320M18Rik | 0.04306 | -0.93281651 |
| 2610319H10Rik | 0.17356 | 0.61435758 |
| Spaca6 | 0.01625 | 1.3000423 |
| Cracr2b | 0.00587 | -0.76496879 |
| Acer2 | 0.64391 | 0.13711561 |
| A630026N12Rik | 0.01247 | 1.64052629 |
| 4933403J19Rik | 0.00144 | -1.30404241 |
| 4833421G17Rik | 0.15387 | 0.98591891 |
| 4933425D22Rik | 0.00415 | -1.06195552 |
| A430088P11Rik | 0.05765 | -0.35808171 |
| Amn1 | 0.00476 | 2.19877763 |
| Frs3os | 0.00544 | -1.10518964 |
| C130033p17rik | 0.09524 | -1.00968452 |
| Dut | 0.58147 | -0.40172827 |
| B130046B21Rik | 0.2889 | -0.38721913 |
| LOC433198 | 0.85918 | 0.0567613 |
| 4930540M05Rik | 0.03111 | 0.7575053 |
| 8030402F09Rik | 0.02808 | -1.44399392 |
| 4930461C15Rik | 0.05631 | -0.70180777 |
| 9430037G07Rik | 0.03202 | 0.89596126 |
| AA060545 | 0.0483 | -0.80948131 |
| D030002E05Rik | 0.09906 | 0.73884366 |
| Nhsl2 | 0.2954 | 0.24943648 |
| 2810428J06Rik | 0.00144 | 1.91939057 |
| 2610020P09Rik | 0.10385 | 0.51046751 |
| Ccnl2 | 0.17729 | -2.28762987 |
| 9530004P13Rik | 0.41439 | 0.38040447 |
| Zfp867 | 0.04617 | 0.75546663 |
| 4930406D18Rik | 0.00144 | -1.29049509 |
| 1700007J24Rik | 0.00144 | 1.31787149 |
| Cmtm4 | 0.0032 | -0.98105296 |
| AI836737 | 0.00144 | -1.27165604 |
| 4930471E19Rik | 0.00144 | -1.44803386 |
| AI853106 | 0.92964 | 0.02835406 |
| 4930568G15Rik | 0.00144 | -1.29746368 |
| Slc9a3 | 0.00382 | 1.11205045 |
| D4Ertd681e | 0.26132 | 0.55956523 |
| E130112N10Rik | 0.65013 | 0.23215106 |
| 4921516I12Rik | 0.10095 | -0.62126521 |
| AU015584 | 0.33047 | -0.72833938 |
| Far2os2 | 0.07936 | -0.90858039 |
| 4930480G23Rik | 0.00821 | -1.17200505 |
| AW047481 | 0.06748 | -0.43260188 |
| C230085N15Rik | 0.67618 | 0.31456774 |
| 2610201A13Rik | 0.5656 | -0.14783435 |
| AI451250 | 0.01982 | -1.05114563 |
| 4930598N05Rik | 0.40057 | -0.56466999 |
| BC005561 | 0.00144 | 2.7746759 |
| 1700086P04Rik | 0.00144 | -1.28898079 |
| 4933401D09Rik | 0.00144 | -1.31495282 |
| 9530056K15Rik | 0.1025 | -2.81569596 |
| 4833411C07Rik | 0.76153 | 0.11797582 |
| D5Ertd798e | 0.00253 | 1.44579755 |
| 2610035F20Rik | 0.00144 | -1.30713006 |
| Lncbate1 | 0.02864 | -1.83919189 |
| Dnajb14 | 0.05224 | 0.42746777 |
| AI646519 | 0.02745 | -0.43508532 |
| C230073G13Rik | 0.46961 | -0.34499219 |
| A930026B05Rik | 0.66097 | 0.16499807 |
| A230005M16Rik | 0.03334 | -1.47676176 |
| 4933426B08Rik | 0.00145 | -1.2388121 |
| Trim56 | 0.00556 | 1.31366817 |
| 1700111N16Rik | 0.33285 | -0.5157877 |
| AU021128 | 0.00218 | -1.18878227 |
| A930006I01Rik | 0.00144 | -1.29049509 |
| 1810006J02Rik | 0.07888 | -2.45001648 |
| Atxn7l1 | 0.76307 | 0.07755568 |
| 4930552N02Rik | 0.00144 | -1.27992547 |
| B930042K01Rik | 0.06841 | 0.97560817 |
| Slfn5os | 0.81173 | 0.0634738 |
| Taco1os | 0.00866 | -1.67832297 |
| 1600020E01Rik | 0.02091 | 0.65623018 |
| Anks1b | 0.00144 | -1.28116048 |
| 4933431I19Rik | 0.00144 | -1.26301121 |
| A130019P10Rik | 0.95497 | 0.01984653 |
| C1qtnf5 | 0.01374 | -0.75541595 |
| 4930435F05Rik | 0.00144 | -1.29746368 |
| 1700011B04Rik | 0.00144 | -1.31495282 |
| 2610029K11Rik | 0.00508 | -0.68385931 |
| 1700028E10Rik | 0.10733 | -0.82044372 |
| AI481207 | 0.22488 | -0.74549754 |
| Gm2447 | 0.00144 | -1.29085005 |
| 9530036O11Rik | 0.26388 | 0.88672855 |
| 4921509O07Rik | 0.00144 | -1.30713006 |
| AU042950 | 0.00144 | -1.29085005 |
| C130030K03Rik | 0.00144 | -1.28116048 |
| LOC102631735 | 0.03338 | 1.04555699 |
| 4833419O12Rik | 0.00879 | -1.05401892 |
| Pitpnm2os1 | 0.00144 | -1.42439278 |
| Mon2 | 0.01666 | -0.73878533 |
| Hsp90aa1 | 0.11966 | -0.9119901 |
| Gm29677 | 0.00144 | -1.30626366 |
| 4930545E07Rik | 0.00145 | -1.22376446 |
| Unc45bos | 0.62459 | -0.17573127 |
| AU014795 | 0.00144 | -1.27165604 |
| 1700129O19Rik | 0.39145 | -0.6785413 |
| 9030203C11Rik | 0.00145 | 2.41607668 |
| 1700112J16Rik | 0.73201 | -0.08122083 |
| Tmem150cos | 0.04749 | -1.92948002 |
| 4933404M09Rik | 0.00144 | -1.28898079 |
| 4930403D09Rik | 0.28106 | -0.70472537 |
| 3300002P13Rik | 0.08278 | -1.25438672 |
| Snora47 | 0.89341 | -0.06351104 |
| DXErtd223e | 0.43132 | -0.40623579 |
| Slc37a2 | 0.06448 | -0.57206584 |
| 4933405D12Rik | 0.01041 | 1.68503688 |
| Gm14858 | 0.00355 | 1.97414163 |
| D10Ertd447e | 0.00372 | -1.14963365 |
| A530072M11Rik | 0.29245 | 0.8193806 |
| Akap6 | 0.75862 | 0.05126928 |
| 8030494B02Rik | 0.22413 | -0.35740267 |
| AU021880 | 0.00144 | -1.28375735 |
| Ptk2 | 0.34279 | -0.17650218 |
| Gm10776 | 0.00145 | -1.22376446 |
| D330022H12Rik | 0.04371 | -0.76658677 |
| 5830408B19Rik | 0.01718 | 0.96278709 |
| 4933413C19Rik | 0.00144 | -1.27165604 |
| B130052P14Rik | 0.0354 | 0.69975187 |
| LOC105247671 | 0.02673 | -0.73264207 |
| D6Ertd160e | 0.34169 | -0.23508558 |
| D430022A14Rik | 0.13399 | 0.45326517 |
| 1700003I16Rik | 0.09822 | -0.73387795 |
| 4933417O13Rik | 0.00144 | -1.31495282 |
| B230303A05Rik | 0.00145 | -1.31323225 |
| AU040972 | 0.01118 | -0.58323744 |
| 3010003L21Rik | 0.00721 | -0.98623191 |
| 1110013I04Rik | 0.02236 | -1.03547402 |
| AI662501 | 0.01018 | 1.60861108 |
| C79798 | 0.00644 | -1.3137021 |
| C630007K24Rik | 0.41605 | 0.1904514 |
| A430105D02Rik | 0.10577 | 1.1670379 |
| A330058E17Rik | 0.49789 | 0.28710372 |
| C730037M02Rik | 0.00144 | -1.30713006 |
| Sec14l3 | 0.00145 | -1.22376446 |
| 8030498J20Rik | 0.00144 | 2.02498004 |
| Gm29674 | 0.00144 | -1.28116048 |
| 3010001F23Rik | 0.00145 | -1.22193674 |
| 4930458K08Rik | 0.00144 | -1.29049509 |
| 3830612M24 | 0.13483 | 0.39318378 |
| 1700013M08Rik | 0.00144 | -1.28898079 |
| N4bp2l1 | 0.00182 | 1.59928567 |
| D030025E07Rik | 0.00144 | -1.29746368 |
| Tcrg-V1 | 0.09077 | -0.62482304 |
| Gm15328 | 0.10733 | -0.82044372 |
| Sat2 | 0.92408 | 0.02807017 |
| 1700003P14Rik | 0.0017 | -1.24950944 |
| LOC552890 | 0.00901 | 2.54233612 |
| C030011L09Rik | 0.25358 | 0.41639595 |
| 1700109K24Rik | 0.9333 | 0.03050056 |
| Mttp | 0.25569 | -1.67235746 |
| C230014O12Rik | 0.041 | -0.9136939 |
| A930009L07Rik | 0.00144 | -1.31884045 |
| D7Wsu130e | 0.12842 | -0.30619888 |
| E330037I15Rik | 0.23952 | -0.37361371 |
| LOC553089 | 0.00144 | 2.87722428 |
| 1700012E03Rik | 0.00272 | -1.09759392 |
| 4930556H04Rik | 0.13124 | -0.37999395 |
| 4930511A02Rik | 0.00144 | -1.28116048 |
| 4930532I03Rik | 0.00727 | -1.13382426 |
| A230009B12Rik | 0.05821 | -1.34661642 |
| F930015N05Rik | 0.07892 | -0.58588355 |
| 1700120C14Rik | 0.05458 | -0.79019759 |
| 9530026P05Rik | 0.02729 | -0.62849054 |
| 4930443G03Rik | 0.00157 | -1.19247859 |
| A230061C15Rik | 0.00144 | -1.28898079 |
| Akap1 | 0.1934 | -0.40793391 |
| D0Kist5 | 0.00317 | -1.11935153 |
| Gm33093 | 0.00144 | -1.3348153 |
| Sorbs2os | 0.00733 | -1.10984709 |
| 1700125G22Rik | 0.14419 | -0.87431012 |
| 2610008G14Rik | 0.47621 | -0.2675718 |
| 1700101I11Rik | 0.00145 | 1.49761771 |
| Fcor | 0.00145 | -1.22193674 |
| Rbms3 | 0.05442 | 0.70291892 |
| Cdrt4os1 | 0.29466 | -0.70041676 |
| 4930529I22Rik | 0.18325 | -0.29978126 |
| A230004M16Rik | 0.00145 | -1.24191372 |
| Chn1os3 | 0.00145 | -1.26219879 |
| Igkv4-53 | 0.00144 | -1.79219685 |
| C630001G18Rik | 0.63086 | -0.28239928 |
| 4930452L12Rik | 0.0021 | -1.16531916 |
| 4930554N03Rik | 0.0017 | -1.24950944 |
| AI849538 | 0.00144 | 1.46698535 |
| B230110G15Rik | 0.00144 | -1.29085005 |
| 4930404H11Rik | 0.00144 | -1.29049509 |
| B230114P17Rik | 0.04466 | 1.13204588 |
| Junos | 0.00144 | -1.27165604 |
| Ppp1r12b | 0.00647 | 2.42240237 |
| E230006M18Rik | 0.91348 | -0.01927947 |
| LOC102643247 | 0.76868 | 0.05566839 |
| C230006B20 | 0.02469 | 1.2968685 |
| AW124847 | 0.00145 | -1.22376446 |
| Hnrnpl | 0.02149 | -0.4804843 |
| A930027P06Rik | 0.21839 | -0.7907259 |
| LOC105243207 | 0.0293 | -0.76945634 |
| Glb1l2 | 0.30989 | -0.60643066 |
| Snhg12 | 0.02454 | -1.19163628 |
| F830115B05Rik | 0.03728 | -0.8681977 |
| Gm10125 | 0.00144 | -1.31495282 |
| Gm31214 | 0.62095 | 0.28598627 |
| 9530052C20Rik | 0.00144 | -1.29085005 |
| C030010L15Rik | 0.16941 | 0.24853273 |
| 4933434C23Rik | 0.00149 | -1.25408 |
| BC003331 | 0.01611 | 0.51017927 |
| 4930557J02Rik | 0.00534 | -1.45950387 |
| Mid1 | 0.03777 | -1.0065176 |
| Dph6 | 0.00435 | 0.98816328 |
| 6330564D18Rik | 0.26905 | -0.90860835 |
| Mirg | 0.13103 | -0.54169775 |
| 0610033M10Rik | 0.00144 | -1.42082928 |
| Nlgn3 | 0.00299 | -0.97083335 |
| 2900009J06Rik | 0.00286 | -1.17314862 |
| D11Ertd712e | 0.04956 | -0.7256038 |
| 1110059G02Rik | 0.00145 | -1.22193674 |
| 4921531C22Rik | 0.00281 | -0.86770864 |
| 4930500F04Rik | 0.00149 | -1.58243492 |
| Gm2824 | 0.00144 | -1.26560808 |
| 9.13E+15 | 0.02931 | 1.4470922 |
| D9Ertd115e | 0.974 | -0.03251844 |
| D630024D03Rik | 0.76177 | -0.09106472 |
| AI327134 | 0.20155 | -0.40849671 |
| AU022934 | 0.00144 | -1.26428512 |
| 4933428C20Rik | 0.11587 | -1.2173589 |
| 4930401C15Rik | 0.00144 | -1.31884045 |
| BC020402 | 0.80751 | -0.05952573 |
| Mgea5 | 0.57809 | 0.17365837 |
| A230101C19Rik | 0.18202 | -0.56306786 |
| 4930453H23Rik | 0.03243 | -1.35014862 |
| D130062J21Rik | 0.00767 | 1.267342 |
| Gm15998 | 0.51884 | 0.15584717 |
| 4933404K08Rik | 0.00144 | -1.28116048 |
| D030004A10Rik | 0.00145 | -1.25646236 |
| D630028G08Rik | 0.04247 | 1.41543707 |
| A930033C23Rik | 0.02939 | -0.81772421 |
| AU020951 | 0.00144 | -1.28898079 |
| Olfr947-ps1 | 0.00794 | -1.4104604 |
| Epb4.1l5 | 0.07289 | 0.66121821 |
| D1Ertd83e | 0.00144 | -1.44399392 |
| 4930554C24Rik | 0.69799 | -0.24214498 |
| 1700120E14Rik | 0.00149 | -1.22582153 |
| Zfp81 | 0.02512 | 0.49306325 |
| 9630041G16Rik | 0.39825 | -0.27119563 |
| A730090N16Rik | 0.09356 | -1.07150653 |
| Lmbr1 | 0.06018 | 0.60072361 |
| 4930474G06Rik | 0.00144 | 1.73078091 |
| D130007C19Rik | 0.00144 | 2.03949361 |
| Dbhos | 0.00144 | -1.31495282 |
| 1700030L20Rik | 0.76154 | 0.34003678 |
| Camkk2 | 0.14586 | -0.27634156 |
| BB086117 | 0.00144 | -1.31233397 |
| Dyrk2 | 0.42814 | 0.14214194 |
| Catsperd | 0.58414 | -0.15478316 |
| C530014P21Rik | 0.40213 | 0.50008332 |
| Ccdc148 | 0.04629 | -0.81215216 |
| D130017N08Rik | 0.04551 | -0.60674101 |
| LOC552877 | 0.72317 | 0.09098276 |
| 6030451C04Rik | 0.0446 | 0.85602228 |
| 4930567K12Rik | 0.20835 | -0.87483029 |
| Gm7265 | 0.83783 | 0.15100178 |
| AI606473 | 0.00145 | -1.26219879 |
| Aste1 | 0.16068 | -0.4727128 |
| 4931431B13Rik | 0.75681 | -0.0775145 |
| Il13ra2 | 0.32124 | -0.30973752 |
| Arhgef38 | 0.00144 | -1.29746368 |
| 1500032P08Rik | 0.06853 | 0.76487007 |
| 5031425F14Rik | 0.02662 | -1.20325065 |
| LOC433611 | 0.00144 | -1.20228444 |
| Ano6 | 0.00892 | -0.90642389 |
| 9430085M18Rik | 0.00144 | -1.06907495 |
| 5730408K05Rik | 0.98202 | -0.01043498 |
| Ube2i | 0.00423 | -1.08454176 |
| A630057N01Rik | 0.02229 | 1.25765487 |
| 4930525G20Rik | 0.97748 | -0.00698647 |
| 2810405F17Rik | 0.028 | 1.33811362 |
| 9330175M20Rik | 0.26694 | -0.18461637 |
| Tnfrsf11a | 0.52316 | -0.1038032 |
| Gm38405 | 0.1375 | -0.8483381 |
| Arhgap1 | 0.03817 | -0.41887747 |
| 2010002M09Rik | 0.32854 | 0.22847367 |
| Cers3 | 0.22337 | -0.7319734 |
| BB085087 | 0.68956 | 0.16840468 |
| 1700060J05Rik | 0.39902 | 0.47222416 |
| 5033406O09Rik | 0.02706 | -0.79659031 |
| 0610039H22Rik | 0.7589 | 0.1346179 |
| Dmrt1 | 0.30847 | -0.28667074 |
| C030014I23Rik | 0.00144 | -1.28898079 |
| 5830403F22Rik | 0.16086 | -0.71757475 |
| 6230400D17Rik | 0.91481 | -0.01859568 |
| AA960618 | 0.04845 | -1.27391416 |
| AI463170 | 0.00144 | -1.26560808 |
| 2700078F05Rik | 0.00145 | -1.25200268 |
| C78859 | 0.01198 | 0.9733045 |
| 4930556L07Rik | 0.00447 | -0.92949863 |
| 4633401B06Rik | 0.71099 | 0.23575202 |
| BC051537 | 0.00144 | -1.26934956 |
| 5930433N17Rik | 0.49392 | 0.33811028 |
| A130042O14Rik | 0.23829 | -0.24428707 |
| D1Ertd622e | 0.00235 | -1.61732194 |
| 1700092E19Rik | 0.00177 | -1.15847607 |
| C530042K13Rik | 0.01584 | 1.86951813 |
| Gm5617 | 0.02504 | -0.99910862 |
| Gm16853 | 0.02118 | -0.68702225 |
| F830005D05Rik | 0.01509 | 1.24288653 |
| D030051J21Rik | 0.48851 | 0.67158778 |
| AI314760 | 0.3931 | -0.540363 |
| 4933404I11Rik | 0.00145 | -1.22376446 |
| 4930453O03Rik | 0.0849 | -0.81183568 |
| Gm26604 | 0.00145 | -1.26219879 |
| D11Ertd726e | 0.00472 | 1.55382846 |
| 4930488B22Rik | 0.0042 | -1.72814152 |
| D230040A04Rik | 0.0418 | 0.5818712 |
| Zfp882 | 0.11488 | 0.43017805 |
| B930053N02Rik | 0.0083 | 1.37617779 |
| C130083M11Rik | 0.00144 | -1.30017714 |
| B230207O21Rik | 0.00149 | -1.22582153 |
| 4930500H12Rik | 0.00144 | -1.26560808 |
| D15Ertd509e | 0.0035 | 2.05150462 |
| Hmga2-ps1 | 0.01069 | -1.04957001 |
| 4833415N18Rik | 0.01862 | -0.91784103 |
| 4930414F18Rik | 0.04384 | -1.23187423 |
| Pou2f3 | 0.18029 | -1.1388367 |
| 9430076C15Rik | 0.31738 | 0.32665528 |
| Plekha3 | 0.05036 | 0.40688873 |
| AW011956 | 0.25565 | -0.54848387 |
| BB557941 | 0.00144 | -1.7774367 |
| 4930417H01Rik | 0.00144 | -1.29085005 |
| 1200007C13Rik | 0.47534 | 0.20821571 |
| Aqp8 | 0.00374 | -1.69770971 |
| Amy2a4 | 0.00144 | -1.30017714 |
| BB236558 | 0.15779 | -0.61111671 |
| 2310067P03Rik | 0.01799 | -1.9596296 |
| E2f1 | 0.00144 | -1.29390089 |
| Gm11747 | 0.01514 | -0.95990192 |
| 1700051A21Rik | 0.00144 | -1.35366536 |
| A530076I17Rik | 0.89549 | 0.23490058 |
| E130104P22Rik | 0.14428 | 0.55882309 |
| AI594674 | 0.00168 | -1.22518835 |
| C030013C21Rik | 0.01668 | -1.1786843 |
| E330018M18Rik | 0.57848 | -0.59251039 |
| 9430063H18Rik | 0.0028 | -1.05910102 |
| Fgf11 | 0.02999 | -0.61381971 |
| Gfm2 | 0.01567 | 0.86462873 |
| BC050972 | 0.27985 | -0.46816765 |
| 5330431K02Rik | 0.07373 | 0.45204873 |
| 4930590A17Rik | 0.02942 | -1.05312216 |
| Gripap1 | 0.29229 | -0.17466446 |
| D630022N01Rik | 0.00145 | -1.22376446 |
| Ttll8 | 0.00346 | -1.42218106 |
| 4930458D05Rik | 0.83462 | 0.13747706 |
| LOC102632594 | 0.00145 | -1.22376446 |
| 4931403G20Rik | 0.00743 | -1.15015382 |
| 5430405H02Rik | 0.00144 | -1.32766452 |
| Secisbp2l | 0.68272 | -0.48319155 |
| BC065403 | 0.02218 | -0.74611132 |
| Hnf4aos | 0.23438 | -0.56409184 |
| AU024245 | 0.00144 | -1.22925777 |
| LOC328066 | 0.20479 | -0.26680028 |
| 4930509E22Rik | 0.127 | -1.60491148 |
| Zbed6 | 0.54516 | 0.17115669 |
| D10Bwg1070e | 0.70553 | -0.14530555 |
| 1700040D17Rik | 0.19684 | -0.66250302 |
| D030029J20Rik | 0.00519 | 1.19308744 |
| AU046084 | 0.00157 | 1.45882772 |
| BB070754 | 0.02686 | 1.50710976 |
| AU023617 | 0.00149 | -1.2703832 |
| B230398E01Rik | 0.00144 | -1.72157622 |
| A530028O18 | 0.04197 | -1.16079366 |
| 4930554H23Rik | 0.00144 | -1.29085005 |
| E330024J20Rik | 0.15349 | -0.62482304 |
| AU015723 | 0.0784 | -1.73438704 |
| 2410012E07Rik | 0.00144 | -1.28116048 |
| C130045F17Rik | 0.04249 | 0.81629388 |
| Kantr | 0.03523 | 1.03341737 |
| Ky | 0.11138 | -0.60646379 |
| 1810007D17Rik | 0.11358 | -0.88869975 |
| E130110O22Rik | 0.00144 | 1.57822939 |
| Vmn2r-ps129 | 0.00145 | -1.2388121 |
| A430106A12Rik | 0.04394 | 0.72911553 |
| AI851613 | 0.00144 | -1.20555712 |
| Gm16062 | 0.12483 | -0.56661517 |
| Ccr9 | 0.00144 | -1.30017714 |
| 4930528D03Rik | 0.00144 | -1.27165604 |
| LOC100036540 | 0.00144 | -1.27454685 |
| 5730526G10Rik | 0.00911 | 2.20142874 |
| R74862 | 0.72156 | -0.08034244 |
| E530011L22Rik | 0.1104 | 0.64792471 |
| D130051D11Rik | 0.00582 | 2.47158769 |
| 4930555B11Rik | 0.59816 | -0.43580951 |
| AU015558 | 0.01088 | -1.18049462 |
| 4933408A14Rik | 0.38284 | -0.33679401 |
| 6330571C24Rik | 0.00145 | -1.22376446 |
| Kcnmb4os2 | 0.00434 | -1.0948178 |
| AA617406 | 0.15309 | -0.97316354 |
| 4930512H18Rik | 0.41842 | -0.34987692 |
| C81189 | 0.52705 | -0.25259482 |
| 4933411E02Rik | 0.79584 | 0.108178 |
| D9Wsu90e | 0.74043 | 0.19093682 |
| Ush1c | 0.59842 | 0.29382953 |
| 6720427H10Rik | 0.00144 | 1.67593072 |
| Ptpn21 | 0.00144 | -1.40303356 |
| 1520401A03Rik | 0.00498 | -1.19502277 |
| Gm20754 | 0.00144 | -1.44608204 |
| 4921515L22Rik | 0.6744 | -0.32030006 |
| A730089K16Rik | 0.00208 | -1.15498993 |
| A430105J06Rik | 0.0146 | -1.18792946 |
| Ros1 | 0.00144 | -1.44554351 |
| 4921515E04Rik | 0.0266 | -1.83635413 |
| G630022F23Rik | 0.00144 | -1.25118919 |
| C530044C16Rik | 0.31135 | -0.58083459 |
| Noxa1 | 0.60597 | -0.16287098 |
| Hoxaas3 | 0.00144 | -1.54415904 |
| Hoxa3 | 0.01541 | -1.73508602 |
| C230079E05Rik | 0.02888 | -1.44315161 |
| AU015230 | 0.03223 | -1.46019789 |
| D830044D21Rik | 0.53829 | -0.24281803 |
| 5033421B08Rik | 0.54446 | 0.28342306 |
| 9530020I12Rik | 0.03315 | -1.18304346 |
| AW413774 | 0.06619 | 0.41000786 |
| Man2c1os | 0.05067 | -0.69930881 |
| Ccnb3 | 0.00149 | -1.22582153 |
| 1700048F04Rik | 0.00144 | -1.27165604 |
| Kdm5d | 0.0327 | -1.39699554 |
| AU042410 | 0.00145 | -1.25646236 |
| 9430065F17Rik | 0.06932 | -0.91089797 |
| C80120 | 0.00372 | -1.14963365 |
| AI426330 | 0.11686 | 0.26300814 |
| 3300002A11Rik | 0.57127 | -0.19123172 |
| Tarbp2 | 0.0038 | -1.02682347 |
| AU017834 | 0.00144 | -1.29085005 |
| 0610040F04Rik | 0.26618 | -1.59806985 |
| Pcdh10 | 0.26741 | -2.20327906 |
| A530030E21Rik | 0.23217 | 0.30642159 |
| 9430062P05Rik | 0.0045 | 2.22751706 |
| Pet100 | 0.01661 | -1.9258358 |
| 9930024M15Rik | 0.3206 | 0.38068755 |
| C78653 | 0.00144 | -1.66163818 |
| D030046N08Rik | 0.00286 | -1.1443198 |
| D130095D21Rik | 0.01602 | 1.36928201 |
| AW050198 | 0.09948 | -1.11163201 |
| AI843588 | 0.00144 | -1.26428512 |
| Tmprss11b | 0.19232 | -0.31291896 |
| 4833412C15Rik | 0.69352 | 0.13485289 |
| C79122 | 0.00144 | -1.31884045 |
| Scube1 | 0.0021 | -1.1065604 |
| 4930588A03Rik | 0.00464 | -1.18731051 |
| 4930412L05Rik | 0.02024 | -0.94109882 |
| Papolg | 0.07412 | 0.67772722 |
| 4930554I06Rik | 0.02632 | -1.07029893 |
| Trmt10a | 0.27524 | 0.8845624 |
| Gm30871 | 0.09746 | -2.52155639 |
| 4921534H16Rik | 0.54267 | -0.37884234 |
| Whsc1l1 | 0.30947 | -0.22603485 |
| 4930566D17Rik | 0.01044 | -0.92449895 |
| C85181 | 0.41629 | 0.4089754 |
| 2010310C07Rik | 0.48218 | -0.5804705 |
| Zfp264 | 0.00144 | -1.56449565 |
| A730056A06Rik | 0.02687 | -0.77326093 |
| Gm13629 | 0.00153 | -1.23808184 |
| 4930512B01Rik | 0.00144 | -1.29051721 |
| LOC102634887 | 0.00144 | -1.27165604 |
| 4833420D23Rik | 0.41295 | 0.17682535 |
| 9630010G10Rik | 0.9638 | -0.01718845 |
| Sarm1 | 0.00996 | -1.65932456 |
| 5830427D02Rik | 0.02261 | 1.22293961 |
| Elmo3 | 0.168 | -0.44723045 |
| AI594671 | 0.00144 | -1.26560808 |
| A930007D18Rik | 0.00144 | 2.64669205 |
| 4933433G15Rik | 0.00144 | -1.26934956 |
| D9Ertd720e | 0.0064 | -0.96858549 |
| Mapk1ip1l | 0.09419 | -0.80202537 |
| 2600002B07Rik | 0.9694 | 0.01195323 |
| 4930432L08Rik | 0.0502 | -1.00873723 |
| AU067648 | 0.04836 | -0.91880445 |
| Ube4bos1 | 0.00816 | -0.86014901 |
| Tanc2 | 0.99013 | 0.01006454 |
| C030006F08Rik | 0.0108 | -1.08231701 |
| Tmem2 | 0.32655 | -0.18923006 |
| 4930557K07Rik | 0.04294 | 0.95490705 |
| 1700047E10Rik | 0.00145 | -1.2388121 |
| AA536887 | 0.20408 | 0.53126343 |
| 2900035I09Rik | 0.5688 | -0.26663219 |
| 2410024F20Rik | 0.00145 | -1.60870824 |
| Pura | 0.55603 | -0.21694627 |
| Tbc1d5 | 0.54607 | 0.18287882 |
| 5830419E12Rik | 0.12578 | -0.98520196 |
| 1700001C19Rik | 0.00165 | -1.3119945 |
| Slc2a4rg-ps | 0.51797 | 0.22305583 |
| 4933406G16Rik | 0.00144 | -1.31884045 |
| AI662175 | 0.00144 | -1.27165604 |
| Gm20757 | 0.00144 | -1.28116048 |
| D430004P15Rik | 0.01167 | 1.45619339 |
| 2310002F09Rik | 0.00705 | -0.67268611 |
| LOC102639982 | 0.14685 | 0.82229479 |
| 9130015A21Rik | 0.01224 | -0.99902751 |
| Slc8a1 | 0.23178 | -0.23302845 |
| D230018H15Rik | 0.34479 | -0.42798675 |
| 4933421D24Rik | 0.18523 | -0.62769066 |
| 2810403G07Rik | 0.1094 | -2.80306737 |
| 4833447P13Rik | 0.04933 | -0.40115749 |
| 5830437K03Rik | 0.1857 | -0.84350491 |
| 2610100L16Rik | 0.11657 | -0.72429914 |
| C86371 | 0.00144 | -1.25118919 |
| A830009L08Rik | 0.00144 | -1.28898079 |
| Htr1b | 0.00149 | -1.22582153 |
| AI853502 | 0.00144 | -1.26301121 |
| 1700001D01Rik | 0.78057 | -0.07069761 |
| Cd163 | 0.59443 | 0.14992235 |
| Fryl | 0.01895 | -0.49859392 |
| Scamp1 | 0.47715 | -0.36607191 |
| Cacnb4 | 0.06538 | 0.38691128 |
| 8430437L04Rik | 0.00144 | -1.27992547 |
| BC046401 | 0.00145 | -1.22376446 |
| AI429363 | 0.70646 | 0.07704937 |
| B230308N11Rik | 0.50841 | 0.11842123 |
| Ceacam2 | 0.00293 | -1.30945677 |
| AI852580 | 0.61193 | -0.22119488 |
| 1700066J03Rik | 0.62393 | -0.49950561 |
| LOC497255 | 0.45406 | -0.34912037 |
| Adgrg4 | 0.00144 | -1.28898079 |
| 2310007J06Rik | 0.17887 | 0.49812501 |
| Gucy1a2 | 0.00219 | 1.61814197 |
| 1700006F04Rik | 0.00221 | -1.10308723 |
| Igkv6-15 | 0.25887 | -0.72298284 |
| A930037O16Rik | 0.06471 | 0.63291212 |
| A530058O07Rik | 0.00145 | -1.26219879 |
| Tbc1d22bos | 0.00144 | -1.40179533 |
| AI848615 | 0.00144 | -1.30713006 |
| 4930519N06Rik | 0.96943 | -0.04184363 |
| AI415730 | 0.01488 | 1.18845623 |
| Snap23 | 0.12837 | 0.53287872 |
| 4933407C09Rik | 0.00144 | -1.26560808 |
| 4930473O22Rik | 0.03132 | -0.97953315 |
| 9430099O15Rik | 0.1837 | 0.43909785 |
| 6720462K09Rik | 0.15721 | 0.65811706 |
| A930001C03Rik | 0.37244 | -0.18228177 |
| 4933405E24Rik | 0.60835 | -0.19794369 |
| Srrm4os | 0.00144 | -1.29085005 |
| 4932441J04Rik | 0.05781 | -2.17144728 |
| A630038E17Rik | 0.00144 | -1.31495282 |
| 5730437C12Rik | 0.00489 | -1.78355103 |
| Rptor | 0.86926 | -0.09193782 |
| 1700094J05Rik | 0.00585 | -1.07062058 |
| Smim18 | 0.00144 | -1.2069806 |
| D230044P21Rik | 0.00328 | -1.40855338 |
| A330032B11Rik | 0.00144 | -1.26560808 |
| 4930444A19Rik | 0.00145 | -1.2388121 |
| A430085C19 | 0.00328 | -1.15105754 |
| 4933433G08Rik | 0.00144 | -1.26560808 |
| 4930579K21 | 0.12774 | -0.64608244 |
| Gm10732 | 0.00144 | -1.27165604 |
| Gm10739 | 0.0066 | -1.59951652 |
| 2810455B08Rik | 0.89262 | -0.04401849 |
| LOC102633880 | 0.53615 | 0.29501143 |
| A530020G20Rik | 0.0037 | -1.11214255 |
| 4930478P22Rik | 0.00145 | -1.26219879 |
| 4930505M18Rik | 0.01266 | -1.35248342 |
| 4930447N08Rik | 0.2906 | -0.42816853 |
| 2210011K15Rik | 0.00145 | -1.22193674 |
| Smyd4 | 0.00144 | -1.29085005 |
| 2810455O05Rik | 0.00144 | -1.27165604 |
| 4933432I03Rik | 0.18403 | -0.62359469 |
| 4933425M03Rik | 0.10401 | -0.82107543 |
| 4930484H19Rik | 0.00158 | -1.17515383 |
| Gm28856 | 0.00144 | -1.29049509 |
| 2610034E01Rik | 0.21189 | 0.51124988 |
| 2610002M06Rik | 0.16205 | 0.49151284 |
| Gm3822 | 0.00145 | -1.22376446 |
| 4930553P18Rik | 0.12053 | 0.7554608 |
| 5730405A17Rik | 0.00144 | -1.28898079 |
| Zfp697 | 0.01982 | -0.97999278 |
| 4930570B17Rik | 0.00774 | -0.65735665 |
| D2Bwg0886e | 0.27699 | -0.43012937 |
| 5730414N17Rik | 0.08349 | -0.44755278 |
| 5930430L01Rik | 0.06393 | 1.18987867 |
| AU019202 | 0.41489 | -0.57060626 |
| AW492981 | 0.02566 | -0.79723457 |
| 2810404M03Rik | 0.05051 | -1.63976826 |
| AW125646 | 0.20404 | -0.88887182 |
| 2900076A07Rik | 0.00324 | -0.84553468 |
| 5430400D12Rik | 0.87324 | 0.05212956 |
| C79015 | 0.49998 | 0.41940228 |
| 4933438K21Rik | 0.00292 | -1.14532111 |
| D8Ertd503e | 0.00144 | -1.31884045 |
| 4930516K23Rik | 0.00145 | -1.27532353 |
| F830014O18Rik | 0.39063 | 0.37271168 |
| 9030419F21Rik | 0.0076 | -1.08211872 |
| 5430420F09Rik | 0.18016 | 0.68031208 |
| 9630028H03Rik | 0.04454 | -0.87156107 |
| 9630028H03Rik | 0.11176 | -0.90165173 |
| Olfr714 | 0.00212 | -1.42249344 |
| 3830408C21Rik | 0.55063 | -0.28261215 |
| A1cf | 0.00144 | -1.20555712 |
| A330009N23Rik | 0.00159 | -1.08442195 |
| 1700037F03Rik | 0.21268 | 0.72551936 |
| C920008N22Rik | 0.0044 | 1.87158258 |
| AI480461 | 0.00704 | 0.9158736 |
| Dnah1 | 0.10451 | -2.02570383 |
| 4933407O12Rik | 0.23038 | 0.29538364 |
| Nudt5 | 0.59365 | 0.30411501 |
| Gm16523 | 0.00478 | 2.14878645 |
| 4921518B13Rik | 0.00144 | -1.30017714 |
| 9330179D12Rik | 0.14003 | -0.44792125 |
| Arf4os | 0.00145 | -1.26219879 |
| C030013G03Rik | 0.86772 | 0.06915185 |
| 4930562D21Rik | 0.00144 | -1.27165604 |
| Gm10134 | 0.84712 | 0.04525611 |
| AA522020 | 0.16549 | 0.38269152 |
| AI415467 | 0.00144 | -1.30713006 |
| C030034L19Rik | 0.00144 | -1.29878664 |
| 1700064N11Rik | 0.06305 | -0.59646641 |
| D630014O11Rik | 0.00144 | -1.28116048 |
| 4931439C15Rik | 0.01855 | -0.83607133 |
| 9330175E14Rik | 0.18098 | 0.74189195 |
| Ppifos | 0.00145 | -1.2388121 |
| C78197 | 0.0163 | -2.57522951 |
| 9630023C09Rik | 0.00405 | 2.14129422 |
| Hoxb5os | 0.00144 | -1.31884045 |
| 9330177L23Rik | 0.51778 | 0.2467863 |
| A230055C15 | 0.00144 | -1.24353547 |
| 1700025F24Rik | 0.00192 | -1.57602389 |
| Mid2 | 0.45083 | -0.21402681 |
| Th | 0.21751 | -0.63104526 |
| Nrap | 0.01157 | -0.58326736 |
| G630055G22Rik | 0.00144 | -1.3230041 |
| 9530047P18Rik | 0.11915 | -0.40508345 |
| A830029E22Rik | 0.00144 | -1.28898079 |
| 9530051G07Rik | 0.00144 | -1.29049509 |
| 2900060N18Rik | 0.00144 | -1.28116048 |
| AW549542 | 0.00392 | 1.81711402 |
| A630023A22Rik | 0.00158 | -1.2446827 |
| Agap3 | 0.00176 | -0.91307598 |
| G930009F23Rik | 0.00144 | -1.29049509 |
| Gm20558 | 0.33763 | -0.41704073 |
| A330044H09 | 0.0178 | 0.61859853 |
| AW495222 | 0.00149 | -1.22582153 |
| D7Ertd128e | 0.00144 | -1.24811894 |
| C79296 | 0.0643 | -1.48318712 |
| Tdrd5 | 0.10539 | -2.99922449 |
| C87102 | 0.00144 | -1.27165604 |
| 4933416O17Rik | 0.00144 | -1.28116048 |
| AA914427 | 0.59562 | 0.38127981 |
| 9130001E16Rik | 0.03732 | 1.90812185 |
| Lrrfip2 | 0.0373 | 0.60291201 |
| AW107722 | 0.00145 | -1.22376446 |
| C230037L09 | 0.01317 | -0.78549733 |
| Cyb5d1 | 0.00175 | -0.97646189 |
| Dcdc2c | 0.00144 | -1.27165604 |
| Dep1 | 0.06837 | 1.10102302 |
| Gm10441 | 0.00144 | -1.28116048 |
| C230096K16Rik | 0.01157 | 0.71094597 |
| 5930438M14Rik | 0.01678 | -1.70878851 |
| 4833417C18Rik | 0.27162 | -1.51545611 |
| 4933411E08Rik | 0.00144 | -1.29878664 |
| Ccdc163 | 0.0774 | 0.77545618 |
| Pdp1 | 0.11083 | 0.33665364 |
| Prl8a6 | 0.00144 | -1.27165604 |
| BB116930 | 0.07344 | 0.83155925 |
| Gm4211 | 0.00972 | 0.92999381 |
| Dgkg | 0.26725 | -0.62097442 |
| A330093E20Rik | 0.00144 | -1.24811894 |
| Gm17244 | 0.00157 | -1.19247859 |
| 4930445G23Rik | 0.056 | -2.25367309 |
| 9330199G10Rik | 0.00701 | -0.71614429 |
| 5830462I19Rik | 0.00144 | -1.27699683 |
| 4632411P08Rik | 0.32557 | -0.62382842 |
| 4930471G03Rik | 0.00145 | -1.64381461 |
| 5830426K05Rik | 0.07456 | -2.13451395 |
| Ahcyl2 | 0.00144 | 1.51840242 |
| B930095M22Rik | 0.62328 | -0.13097358 |
| Mettl21c | 0.0204 | 1.19190019 |
| 6330522J23Rik | 0.00416 | -0.81007817 |
| Il2rb | 0.01385 | -1.32152804 |
| Gm13032 | 0.06829 | -1.02171756 |
| AI465300 | 0.02197 | -0.99788027 |
| 1700057D03Rik | 0.0111 | -1.02598223 |
| 4933407K13Rik | 0.01517 | 0.7368176 |
| Kif26b | 0.63707 | -0.16075805 |
| B230334L07Rik | 0.00144 | 2.01886215 |
| 6330526H18Rik | 0.00464 | 1.08276169 |
| 2210408K08Rik | 0.01911 | -0.99981781 |
| AU045094 | 0.00144 | -1.28116048 |
| 1700029M20Rik | 0.54591 | -0.17449291 |
| A930019D19Rik | 0.60228 | -0.12128828 |
| C80406 | 0.19238 | 0.3960589 |
| A130078K24Rik | 0.00144 | 3.36846864 |
| Hal | 0.10112 | 0.9834542 |
| Defa10 | 0.00144 | -1.29746368 |
| 1700026H06Rik | 0.00749 | -1.06678871 |
| 9530053J19Rik | 0.02548 | 1.34645569 |
| LOC102639385 | 0.00144 | -1.26560808 |
| 4930429F24Rik | 0.00144 | 1.87775384 |
| Gm14230 | 0.45933 | 0.15234611 |
| 2310007O11Rik | 0.00144 | -1.28438799 |
| Tbck | 0.00149 | 1.19452991 |
| 5830487J09Rik | 0.00144 | -1.55701652 |
| 4930563F15Rik | 0.16179 | -0.37840585 |
| D230040J21Rik | 0.00818 | 1.91980138 |
| Adam3 | 0.00144 | -1.26560808 |
| 1700028N14Rik | 0.04502 | -1.51256341 |
| AI661323 | 0.00144 | -1.30713006 |
| 6330418K02Rik | 0.28633 | -0.41003676 |
| Ctps2 | 0.31713 | 0.27982309 |
| Gm17753 | 0.08435 | 0.87231339 |
| A130009E19Rik | 0.00145 | -1.22376446 |
| LOC102632493 | 0.00144 | -1.54014117 |
| AK155734 | 0.06639 | -1.10616505 |
| D130012P04Rik | 0.39521 | 0.38693725 |
| 4933425B07Rik | 0.0423 | -0.76217425 |
| AI835086 | 0.02231 | -1.33270016 |
| D7Ertd143e | 0.09552 | 0.55882704 |
| 9330184L24Rik | 0.0017 | -1.19401501 |
| AU044856 | 0.00144 | -1.30017714 |
| 5230400M03Rik | 0.0229 | 0.9482641 |
| LOC433461 | 0.31962 | 0.25417162 |
| D930043N17Rik | 0.22879 | 0.62155014 |
| Xkr6 | 0.00144 | 1.73728725 |
| C030009H01Rik | 0.00144 | -1.26353294 |
| 1810008I18Rik | 0.00144 | 1.6725543 |
| 8030425K09Rik | 0.96914 | 0.02014181 |
| E330032C10Rik | 0.55977 | 0.10840965 |
| Gm7111 | 0.00224 | 1.65051789 |
| Mc5r | 0.00144 | -1.20052149 |
| E330011O21Rik | 0.12945 | 0.56908887 |
| 2410004I01Rik | 0.07002 | -0.88534458 |
| 4930402D18Rik | 0.46515 | -0.39593772 |
| 4930455D15Rik | 0.00743 | -0.72454106 |
| C130078N14 | 0.25696 | -0.27898024 |
| AU022434 | 0.01203 | -1.62614601 |
| Gm11783 | 0.00164 | 2.85784003 |
| 1110028F18Rik | 0.00144 | -1.28898079 |
| AU045717 | 0.00144 | -1.28116048 |
| Syncrip | 0.02976 | 0.88880027 |
| 1700030L22Rik | 0.08705 | -2.50377833 |
| 4930467D21Rik | 0.00144 | -1.28116048 |
| 4930506C21Rik | 0.00982 | 1.79867076 |
| LOC100049077 | 0.04089 | -0.67598802 |
| LOC102638465 | 0.00144 | -1.29051721 |
| 9130002K18Rik | 0.00144 | 2.45796836 |
| 9430013L14Rik | 0.06794 | 0.72618451 |
| AA763521 | 0.00144 | -1.28116048 |
| 1700007L15Rik | 0.88964 | -0.18925603 |
| F630048H11Rik | 0.04908 | 0.83772172 |
| Insl5 | 0.84847 | 0.08106381 |
| A730021G18Rik | 0.4005 | 0.58989981 |
| D7Bwg0826e | 0.06205 | -0.88151665 |
| D7Bwg0826e | 0.00144 | -1.30713006 |
| 2610020C07Rik | 0.00159 | -1.26431393 |
| BB212172 | 0.00621 | 0.6983817 |
| AU020094 | 0.01445 | -0.6860157 |
| 4931402G19Rik | 0.0179 | -0.90769781 |
| Armt1 | 0.03586 | -0.4983812 |
| Luc7l3 | 0.02176 | 1.21177648 |
| 9330198I05Rik | 0.05148 | 1.25895015 |
| Galnt7 | 0.00199 | -0.92165173 |
| 4930567H12Rik | 0.00334 | -1.65217379 |
| 1700025L06Rik | 0.70855 | -0.12408638 |
| B230104C14Rik | 0.37601 | 0.26444935 |
| B230344G16Rik | 0.00144 | -1.28898079 |
| 4930481B07Rik | 0.61513 | 0.19799292 |
| Olfr1372-ps1 | 0.02228 | 0.85449083 |
| Stamos | 0.01818 | -1.08200933 |
| Mpp2 | 0.08411 | -0.64509241 |
| A930038B10Rik | 0.00144 | -1.29049509 |
| 4930556N13Rik | 0.13014 | -1.82183431 |
| 2900076A13Rik | 0.03132 | -0.88365573 |
| A230098N10Rik | 0.0031 | -1.70284149 |
| B230214O09Rik | 0.00577 | 1.64192832 |
| Qk | 0.06177 | 0.49775562 |
| Gm11335 | 0.0719 | -1.03768349 |
| 4933416A02Rik | 0.53135 | 0.31510619 |
| Sycp1-ps1 | 0.00144 | -1.28898079 |
| 9530029O12Rik | 0.56378 | 0.22158013 |
| Nbea | 0.00637 | 0.6724711 |
| 6330566A10Rik | 0.54942 | 0.15327982 |
| Taok1 | 0.56298 | -0.11819586 |
| Pcdhgc5 | 0.0582 | -0.8050125 |
| Ppp1r18os | 0.20953 | -0.57238508 |
| Gm13205 | 0.14974 | -0.50112707 |
| 6330531I01Rik | 0.00178 | 1.00547629 |
| Sema6a | 0.08388 | 0.56391875 |
| BB031773 | 0.00145 | -1.2551153 |
| A630081D01Rik | 0.0093 | 1.07894151 |
| Nucks1 | 0.1947 | -0.28985896 |
| Gm16982 | 0.01071 | 1.2827486 |
| A930016O22Rik | 0.00145 | -1.2388121 |
| A430102J17Rik | 0.00327 | 1.86889041 |
| 9630025H16Rik | 0.04731 | -0.73167199 |
| Hcn1 | 0.35509 | -0.40508572 |
| Aak1 | 0.13037 | -0.24576956 |
| 6530402F18Rik | 0.92733 | 0.03061754 |
| D030045P18Rik | 0.00144 | -1.8460297 |
| 2700068H02Rik | 0.02107 | -1.69444692 |
| C430003N24Rik | 0.02608 | 1.49809331 |
| 2310043M15Rik | 0.55727 | 0.3318068 |
| 4930423M02Rik | 0.00166 | -1.21062785 |
| AI662245 | 0.1641 | -0.33755444 |
| Gpatch2 | 0.09406 | 0.79083893 |
| 4931428A05Rik | 0.02485 | -0.57170606 |
| 1700027A07Rik | 0.8481 | -0.22835278 |
| AI854308 | 0.00145 | -1.25646236 |
| Edaradd | 0.0231 | -0.88816906 |
| Rab26os | 0.00144 | -1.98380104 |
| BC028777 | 0.02104 | -0.69978605 |
| 4930511E03Rik | 0.00144 | -1.29049509 |
| AU041480 | 0.00144 | -1.27730451 |
| 1700023D09Rik | 0.00159 | -1.26431393 |
| 5430434I15Rik | 0.02108 | -1.20581678 |
| Nom1 | 0.84722 | -0.03667258 |
| Trav14d-3-dv8 | 0.12017 | -0.89782899 |
| 4833422M21Rik | 0.00145 | -1.26219879 |
| 9330159M07Rik | 0.02714 | 0.51961722 |
| 5830407E08Rik | 0.56254 | 0.15228436 |
| 4933409F18Rik | 0.00144 | -1.73313323 |
| D7Ertd495e | 0.11721 | -0.5947571 |
| Fam172a | 0.01125 | 1.64109544 |
| Lrrc8a | 0.32934 | -0.18542196 |
| 9530003O04Rik | 0.00405 | 2.04343795 |
| Tspan32 | 0.00616 | -1.3292702 |
| 8430422H06Rik | 0.00144 | -1.27165604 |
| 4930452A19Rik | 0.00144 | -1.28898079 |
| 1700061E17Rik | 0.00144 | -1.26560808 |
| 4930588J15Rik | 0.00144 | -1.31495282 |
| 4930404O17Rik | 0.00144 | -1.69155799 |
| Dazap1 | 0.00144 | -1.29085005 |
| Zgrf1 | 0.49391 | 0.13906403 |
| Znrf2 | 0.05889 | -2.20283908 |
| Klf13 | 0.38034 | -0.15611007 |
| Amotl1 | 0.03185 | -0.63743857 |
| 9330118I20Rik | 0.00144 | -1.37115268 |
| Dcst1 | 0.00299 | -0.95416549 |
| B230117O15Rik | 0.00849 | 1.26548925 |
| LOC552901 | 0.05389 | 1.19358766 |
| D930030O05Rik | 0.16377 | 0.46999058 |
| 9030405F24Rik | 0.0142 | -2.41112909 |
| 4932430A15Rik | 0.16989 | -0.67465774 |
| 2010308F09Rik | 0.42197 | -0.3756424 |
| 9530097N15Rik | 0.65718 | 0.21447525 |
| AU015621 | 0.00589 | -1.48548259 |
| Gm31087 | 0.00144 | -1.5623083 |
| D2Ertd127e | 0.78882 | 0.15298487 |
| AW061096 | 0.14327 | -0.69664738 |
| 4930542D17Rik | 0.0027 | -1.11012596 |
| 4930434J06Rik | 0.03692 | -1.46016009 |
| AU016102 | 0.00144 | -1.20555712 |
| 4931413K12Rik | 0.1786 | -1.7978294 |
| Gm10494 | 0.00144 | -1.45290105 |
| 5830432E09Rik | 0.70477 | -0.23624431 |
| 9330133O14Rik | 0.10854 | -0.41711437 |
| 5330439K02Rik | 0.42299 | 0.46797399 |
| Gm38660 | 0.01685 | -1.12758811 |
| 1190001M18Rik | 0.00144 | -1.26560808 |
| A730032A03Rik | 0.00293 | 1.56953128 |
| Glra4 | 0.00144 | -1.39594897 |
| Epb4.1 | 0.05639 | -0.47702138 |
| Gm20740 | 0.16267 | -0.51868864 |
| Rita1 | 0.01478 | -0.57981815 |
| 1110019D14Rik | 0.0082 | 0.88950197 |
| Gm20555 | 0.29258 | -0.32227771 |
| 2310005E17Rik | 0.77777 | 0.17945708 |
| Zbtb11os1 | 0.0017 | -1.24950944 |
| 4931430N09Rik | 0.00144 | -1.30713006 |
| A630052K13Rik | 0.00144 | -1.30017714 |
| Gpatch8 | 0.00906 | 1.68573922 |
| Tmevpg1 | 0.00144 | -1.30017714 |
| 5031415H12Rik | 0.19709 | -0.4986525 |
| A830021K08Rik | 0.00145 | -1.27532353 |
| 6330407A03Rik | 0.01591 | 0.91311921 |
| 1700081H04Rik | 0.01325 | -0.80677727 |
| A830054O07Rik | 0.00144 | 2.71340064 |
| A730091E23Rik | 0.02478 | 1.0441501 |
| A730028G07Rik | 0.00144 | -1.7630873 |
| 4930419G24Rik | 0.09441 | -1.29537735 |
| Gm13999 | 0.00317 | -1.11935153 |
| Smarca1 | 0.78462 | 0.1110648 |
| Gm29686 | 0.00144 | 1.7699129 |
| A930015G24Rik | 0.00144 | -1.27165604 |
| LOC552873 | 0.00144 | -1.30017714 |
| C130023C23Rik | 0.00903 | 2.17261777 |
| A430103D13Rik | 0.00298 | 1.03812398 |
| AU014756 | 0.00312 | -1.7129791 |
| 4930444M15Rik | 0.00144 | -1.28375735 |
| 4930404K06Rik | 0.00145 | -1.2388121 |
| D8Ertd158e | 0.6838 | -0.14257382 |
| Ppap2a | 0.72194 | 0.05911527 |
| Syt16 | 0.03557 | -0.93227475 |
| C130068B02Rik | 0.00676 | 1.41395402 |
| 4930519E07Rik | 0.01069 | -1.16460487 |
| Cacna1c | 0.00653 | 0.61684046 |
| Gm9856 | 0.02824 | 0.8860844 |
| 0610009E02Rik | 0.68169 | -0.07309224 |
| Gm10278 | 0.02547 | -1.86165417 |
| 1700069L16Rik | 0.22182 | -0.80836372 |
| Fam222b | 0.08442 | 0.73626012 |
| Dpy19l2 | 0.06084 | -0.88061585 |
| Gm33989 | 0.00144 | -1.56432267 |
| 3110027N22Rik | 0.80971 | -0.11999871 |
| AI987986 | 0.18657 | -0.66283479 |
| Spice1 | 0.00144 | 1.42102808 |
| Sgcd | 0.00307 | 1.23507309 |
| BB187676 | 0.00144 | -1.27992547 |
| B930036N10Rik | 0.00746 | -1.57369717 |
| B930036N10Rik | 0.00144 | -1.3393073 |
| 9330195I24Rik | 0.02065 | 0.83287721 |
| Rnf150 | 0.24375 | -0.22010559 |
| AI662168 | 0.59507 | -0.35957889 |
| 9130214F15Rik | 0.00145 | -1.25646236 |
| 2700022O18Rik | 0.04935 | 0.64126369 |
| D930023I05Rik | 0.77406 | 0.18267249 |
| 4930572J10Rik | 0.12696 | -2.0333534 |
| Tmem260 | 0.41292 | 0.24543112 |
| Scube2 | 0.06271 | -0.41738563 |
| A730094K22Rik | 0.02181 | -1.15637139 |
| 5930403N24Rik | 0.00194 | -1.3230041 |
| Smarcal1 | 0.67778 | -0.07244529 |
| AY512915 | 0.00144 | -1.31495282 |
| 2900057B20Rik | 0.00577 | -1.05313144 |
| AI195470 | 0.37497 | -0.71464326 |
| Vwc2l | 0.00145 | -1.25646236 |
| D930019F10Rik | 0.01515 | -1.05631617 |
| AU022793 | 0.03396 | -0.9889904 |
| AI448005 | 0.11019 | 0.88163039 |
| 4921513H07Rik | 0.05149 | -1.14963365 |
| LOC102643243 | 0.00727 | -1.13364756 |
| Gm13372 | 0.35805 | 0.18914011 |
| D930050J11 | 0.07533 | -0.66083078 |
| C030017B01Rik | 0.00145 | -1.24191372 |
| 4930533K18Rik | 0.00144 | -1.31884045 |
| Pigm | 0.00468 | 1.25630577 |
| A330069K06Rik | 0.00499 | -1.49245118 |
| C80998 | 0.29926 | -0.65198538 |
| 4931402H11Rik | 0.00144 | -1.68245717 |
| A730046J19Rik | 0.03686 | 1.00364191 |
| Gm10452 | 0.06774 | 0.69548191 |
| E430022K19Rik | 0.00144 | -1.30017714 |
| Tmem116 | 0.73559 | 0.0785658 |
| Zfp708 | 0.63256 | -0.12288495 |
| Platr10 | 0.00145 | -1.22376446 |
| 4921529L05Rik | 0.42514 | -0.19388984 |
| 4933440J02Rik | 0.00144 | -1.26560808 |
| D630002J18Rik | 0.33 | -0.61058076 |
| Nudt7 | 0.78036 | -0.06789051 |
| Gabrg3 | 0.00257 | -1.21868187 |
| Zbtb38 | 0.00144 | 2.19951891 |
| 4932442G11Rik | 0.04895 | -1.96084026 |
| A130072N09Rik | 0.37963 | -0.24423131 |
| Ptpro | 0.68367 | -0.07542451 |
| 1700123M08Rik | 0.82715 | -0.08796827 |
| AI317158 | 0.56836 | -0.21891509 |
| A530041M06Rik | 0.06365 | 1.06302839 |
| B930078G14Rik | 0.9721 | 0.02305595 |
| 5033418A18Rik | 0.00249 | 1.79209958 |
| 1700095A13Rik | 0.00153 | -1.79714635 |
| 4933433G19Rik | 0.35932 | 0.1666912 |
| 1700007J10Rik | 0.00145 | -1.18741414 |
| 6330415G19Rik | 0.25973 | 0.61864909 |
| A230092J17Rik | 0.00144 | 2.47655178 |
| AW555355 | 0.00315 | 1.87847075 |
| 4831440D22Rik | 0.00144 | 2.39282942 |
| 9530036M11Rik | 0.00144 | -1.26560808 |
| Mettl4 | 0.00197 | 1.45291834 |
| Mettl4 | 0.35497 | -0.87515755 |
| D230044B12Rik | 0.22012 | 0.55152975 |
| 6330412A17Rik | 0.10447 | -0.50254792 |
| 2700054A10Rik | 0.17879 | -0.83995582 |
| A730062M13Rik | 0.01381 | -1.03043839 |
| 4930465K12Rik | 0.00144 | -1.28116048 |
| B130019D13Rik | 0.82754 | 0.25103753 |
| Erc1 | 0.01753 | -0.84536284 |
| 1600022D10Rik | 0.08692 | -0.91617611 |
| D430040D24Rik | 0.28765 | 0.30758812 |
| AA408650 | 0.21878 | -0.54049613 |
| 4631422I05Rik | 0.00198 | -1.13811458 |
| AW493563 | 0.4601 | -0.61429841 |
| AI605517 | 0.15935 | 0.80122218 |
| F420014N23Rik | 0.00545 | -0.93763104 |
| Wdr20 | 0.59094 | 0.179762 |
| AU024180 | 0.73135 | -0.14671726 |
| A930030B08Rik | 0.83098 | 0.07709112 |
| 9230106L01Rik | 0.73135 | 0.47165557 |
| 4930434B07Rik | 0.10831 | -2.67790101 |
| 4833419A21Rik | 0.00325 | -1.15314387 |
| Rabgap1l | 0.02264 | 2.69940575 |
| 4932416H05Rik | 0.07233 | 0.3444592 |
| D230035N22Rik | 0.50218 | 0.35808145 |
| 4930526H09Rik | 0.00144 | -1.28116048 |
| Hoxa4 | 0.42778 | -0.33387892 |
| AW061147 | 0.00158 | -1.05867538 |
| Wnt5a | 0.06748 | -0.63132976 |
| AI413759 | 0.01752 | -1.4980559 |
| Tmf1 | 0.01515 | 1.52214427 |
| C76332 | 0.02715 | -1.76185022 |
| 4921509A06Rik | 0.08965 | 0.37194944 |
| 4930568E12Rik | 0.00144 | -1.31884045 |
| D3Ertd711e | 0.00159 | -1.26431393 |
| 2010013B24Rik | 0.00145 | -1.26219879 |
| Lmnb2 | 0.00554 | -0.89605708 |
| C77488 | 0.02275 | -2.55017539 |
| 4922502B01Rik | 0.00144 | -1.25118919 |
| 9430011C21Rik | 0.11284 | 1.32610483 |
| 4930453L07Rik | 0.00144 | -1.49567869 |
| 2900054C01Rik | 0.51734 | 0.14842482 |
| 4930567K20Rik | 0.10956 | -2.58326049 |
| 4930588G17Rik | 0.00145 | -1.2388121 |
| D10Ertd755e | 0.07537 | -2.23254374 |
| D030047H15Rik | 0.00144 | -1.31495282 |
| 4921515G04Rik | 0.35015 | 0.33994048 |
| LOC545198 | 0.01962 | -0.72930141 |
| Tmem87a | 0.32664 | -0.25358711 |
| A830021M18Rik | 0.21471 | -0.52444176 |
| D13Ertd608e | 0.95216 | 0.08414766 |
| 4930551O13Rik | 0.03432 | -0.96933153 |
| 2500002B13Rik | 0.00495 | 0.96289429 |
| 2610209C05Rik | 0.1784 | -0.81616877 |
| 2610209C05Rik | 0.15215 | -0.46101495 |
| C80425 | 0.06 | -1.18949419 |
| 9630055L06Rik | 0.0629 | 1.4041471 |
| Ephb2 | 0.1798 | 0.42541547 |
| Gm15612 | 0.54195 | -0.29806845 |
| 1700102J08Rik | 0.88864 | -0.06688747 |
| 4933436E23Rik | 0.00144 | -1.28116048 |
| D230017M19Rik | 0.09993 | -2.65506903 |
| Snhg20 | 0.84085 | -0.10771847 |
| LOC552907 | 0.00144 | 1.65697358 |
| 4930519A11Rik | 0.00241 | -1.23495262 |
| 1700058M13Rik | 0.01778 | -0.98704802 |
| Il9r | 0.00144 | -1.31884045 |
| Phtf1os | 0.04273 | 1.25215392 |
| 4930469K13Rik | 0.62292 | 0.31939327 |
| 1700034G24Rik | 0.00144 | -1.27165604 |
| Igh-VJ558 | 0.00144 | -1.30713006 |
| 4932439E07Rik | 0.14159 | -0.46130052 |
| 4933416M07Rik | 0.01623 | -1.58890431 |
| 4933412E12Rik | 0.08663 | -0.34236186 |
| Ccdc42os | 0.00144 | -1.31884045 |
| D7Ertd183e | 0.20389 | -0.37705889 |
| Hnrnpr | 0.00144 | 2.27964709 |
| 4933417E11Rik | 0.00145 | -1.26219879 |
| 5330421C15Rik | 0.00145 | 2.82051457 |
| Olfml3 | 0.66802 | 0.19173183 |
| AU023525 | 0.00144 | -2.25799878 |
| Cd247 | 0.89281 | 0.07444318 |
| Pon2 | 0.83037 | 0.07558275 |
| C80012 | 0.71251 | -0.10924073 |
| Gm3143 | 0.00144 | -1.29049509 |
| A230107N01Rik | 0.06807 | 0.46187011 |
| Apba2 | 0.00396 | -1.12762982 |
| 5730457N03Rik | 0.44646 | -0.70787712 |
| C030005K06Rik | 0.05331 | 0.77902409 |
| LOC548102 | 0.01544 | 1.35735976 |
| 4933435G04Rik | 0.00144 | -1.28116048 |
| 4930539J05Rik | 0.01133 | 1.03706575 |
| 2310034O05Rik | 0.00144 | -1.28116048 |
| Utrn | 0.1107 | 0.49319527 |
| 2410087M07Rik | 0.63175 | 0.18136348 |
| Smr2 | 0.62613 | 0.20976953 |
| C330022C24Rik | 0.02578 | -3.90993047 |
| Snhg8 | 0.00144 | -1.26560808 |
| E130101M22 | 0.18797 | 0.79006728 |
| 6720474J12Rik | 0.0093 | 1.75837174 |
| 1700097M23Rik | 0.00144 | -1.30713006 |
| 9430018G01Rik | 0.00144 | -1.27165604 |
| Map3k4 | 0.01825 | -0.58355657 |
| 5330406M23Rik | 0.01756 | 1.16515381 |
| Trcg1 | 0.55405 | -1.01039451 |
| A830073O21Rik | 0.45063 | 0.2126478 |
| Gm12992 | 0.00144 | -1.22952292 |
| 4732423E21Rik | 0.00545 | 1.62328809 |
| Mroh1 | 0.08333 | -0.54385465 |
| Pou2f1 | 0.3885 | -0.1857177 |
| 9530083O12Rik | 0.04031 | -0.39515978 |
| 1700064E03Rik | 0.71094 | -0.11366264 |
| B430105A11Rik | 0.0088 | -1.08115135 |
| 2900001G08Rik | 0.00144 | -1.39166333 |
| B830008H07Rik | 0.55877 | 0.10131272 |
| 4833419E13Rik | 0.73662 | -0.15205873 |
| C78283 | 0.00158 | -1.17515383 |
| 4930432N10Rik | 0.08985 | -0.88732951 |
| Gm30211 | 0.00144 | -1.17683551 |
| B230334C09Rik | 0.10675 | 0.74731914 |
| D7Ertd807e | 0.15966 | -0.76914284 |
| Onecut2 | 0.35067 | -0.45077081 |
| 6820426E19Rik | 0.15411 | 0.46801557 |
| 9330109K16Rik | 0.2929 | -0.87436588 |
| Gad1os | 0.00144 | -1.24466058 |
| Zfp950 | 0.01322 | -1.07441361 |
| D230019N24Rik | 0.05021 | 0.93077792 |
| Nbeal1 | 0.00144 | 1.78037581 |
| Snhg14 | 0.00144 | -1.27730451 |
| AA414993 | 0.01916 | -0.97612344 |
| 8430437O03Rik | 0.00706 | 2.21315534 |
| F530104D19Rik | 0.00144 | -1.30713006 |
| 1810059H22Rik | 0.00519 | -0.93918912 |
| Gm40828 | 0.50484 | 0.34767768 |
| Gm10419 | 0.69071 | -0.15437537 |
| 6430710M23Rik | 0.04505 | -0.99600912 |
| Grip1os2 | 0.00775 | -1.01080716 |
| 1810062O18Rik | 0.02091 | -0.95992547 |
| 9130403I23Rik | 0.35306 | -0.27250791 |
| BC049715 | 0.24792 | -1.1857469 |
| AI835735 | 0.03643 | -1.32803515 |
| Gm38527 | 0.00144 | -1.29746368 |
| 4933422A05Rik | 0.00144 | -1.30564809 |
| C88045 | 0.35481 | -0.87959656 |
| Polk | 0.684 | 0.13644688 |
| 2610507I01Rik | 0.77727 | -0.04908839 |
| Hnf1a | 0.04888 | -1.29217232 |
| C330026H20Rik | 0.17112 | -1.02346311 |
| 2700079J08Rik | 0.02256 | -0.97953315 |
| 4930442P07Rik | 0.01278 | -0.72279472 |
| 6430519N07Rik | 0.00987 | 1.77505409 |
| Aamdc | 0.9132 | -0.06300932 |
| AU019990 | 0.11362 | 0.52746285 |
| 6030458E02Rik | 0.0849 | 1.11605922 |
| B530045E10Rik | 0.00144 | -1.30017714 |
| 5430416N02Rik | 0.81977 | -0.24616925 |
| Prdm10 | 0.00488 | 0.83489273 |
| 2900011F02Rik | 0.1143 | -0.3328509 |
| Gt(ROSA)26Sor | 0.00745 | 1.50313148 |
| BC028789 | 0.05602 | -0.49245764 |
| 9330162012Rik | 0.18765 | 0.77375053 |
| Carlr | 0.00149 | -1.19696847 |
| 0710001A04Rik | 0.01019 | -1.64927964 |
| 4933400F21Rik | 0.00144 | -1.33632959 |
| C430045I18Rik | 0.47889 | -0.24812865 |
| 2310047N11Rik | 0.002 | -1.22920044 |
| 4930519D14Rik | 0.00216 | -1.10905491 |
| Naf1 | 0.14224 | 0.32293129 |
| 4930571K23Rik | 0.51229 | -0.29200376 |
| Cyp2r1 | 0.00762 | -1.08702071 |
| 2310079F09Rik | 0.9721 | 0.01124073 |
| Gm11381 | 0.00144 | -1.55051004 |
| 2410017I17Rik | 0.00144 | -1.29864962 |
| Gm15760 | 0.12683 | 0.51742124 |
| AI790442 | 0.13052 | 0.97036084 |
| AI844685 | 0.43213 | -0.3821975 |
| Igkv4-55 | 0.00144 | -1.28898079 |
| Zfp389 | 0.01485 | 1.03981174 |
| 1700100L14Rik | 0.00144 | -1.25118919 |
| 4733401D01Rik | 0.23697 | -0.30693788 |
| LOC552902 | 0.4828 | -0.21790311 |
| 8030456M14Rik | 0.17263 | -0.52699452 |
| 4930423D22Rik | 0.42666 | 0.30090093 |
| A630014C17Rik | 0.01514 | -1.66440716 |
| F630201L12Rik | 0.00328 | -1.10633687 |
| Gm11266 | 0.02378 | -2.92795211 |
| Dleu2 | 0.0019 | -1.10945145 |
| Dleu2 | 0.00298 | 3.55711094 |
| AU043392 | 0.33882 | -0.67074135 |
| 5830443J22Rik | 0.02596 | 1.02339898 |
| Dlgap2 | 0.00626 | -1.06520311 |
| 9330102E08Rik | 0.95921 | 0.01186664 |
| 2810002D19Rik | 0.07282 | 0.73495291 |
| Tfap2a | 0.02671 | -1.00277612 |
| Plxna4os1 | 0.00144 | -1.31552497 |
| Gm11201 | 0.12108 | -0.75414236 |
| Gm10635 | 0.71778 | 0.12306845 |
| Il22 | 0.04617 | -1.23836216 |
| Bend4 | 0.32784 | 0.24811997 |
| LOC102635358 | 0.21227 | -0.41616472 |
| 4930415C11Rik | 0.32465 | -0.29077755 |
| Gm10768 | 0.01363 | -1.70421622 |
| 5830420C07Rik | 0.02772 | 0.95734219 |
| 4930506A18Rik | 0.59431 | 0.08836108 |
| D4Ertd58e | 0.00778 | -1.04962113 |
| 2010004M13Rik | 0.75765 | 0.12934764 |
| Srgap1 | 0.08364 | -0.38911219 |
| Lhx1os | 0.00213 | -1.29142283 |
| 3110001N23Rik | 0.88459 | -0.04946067 |
| 1700048B10Rik | 0.0023 | -1.24900821 |
| 1110046J04Rik | 0.01157 | -0.60724844 |
| C80360 | 0.54985 | -0.33531431 |
| Tnfsf13os | 0.02326 | -0.6014136 |
| A930018M24Rik | 0.15226 | 0.65662525 |
| 9430069I07Rik | 0.04869 | -0.7826411 |
| LOC105245193 | 0.30326 | -0.53469044 |
| 4930412C18Rik | 0.78451 | 0.08903469 |
| Srrm3os | 0.00144 | -1.30017714 |
| A630031M23Rik | 0.39713 | 0.45660016 |
| Slc9a7 | 0.03732 | 0.62646737 |
| 6330509M05Rik | 0.00145 | -1.22376446 |
| E130218I03Rik | 0.00309 | -1.44547589 |
| 4930532M18Rik | 0.00144 | -1.29085005 |
| 9030407P20Rik | 0.9981 | 0.00130192 |
| Rabepk | 0.89712 | -0.0543085 |
| 4930458B22Rik | 0.00732 | -1.04583451 |
| 1700016K05Rik | 0.01802 | -0.84407541 |
| 4933424G05Rik | 0.18656 | 0.29237898 |
| Gm11823 | 0.00144 | -1.30713006 |
| Dbn1 | 0.11935 | -0.40201565 |
| 1700066D14Rik | 0.00202 | -1.44458078 |
| 1700126A01Rik | 0.04188 | -2.39562502 |
| A230056J06Rik | 0.92899 | -0.05085168 |
| Platr14 | 0.00144 | -1.28898079 |
| E130106K03Rik | 0.05255 | 1.79399409 |
| C030014A21Rik | 0.00144 | -1.26301121 |
| C87882 | 0.00144 | -1.32313902 |
| 9530062K07Rik | 0.17841 | 1.05519536 |
| Pard3bos1 | 0.00562 | -1.15214925 |
| Cd226 | 0.02389 | 0.89661527 |
| AA986695 | 0.00575 | -1.62197294 |
| 6720420G18Rik | 0.1315 | 0.79849883 |
| 4731420N21 | 0.72365 | 0.10326479 |
| A330032P22Rik | 0.37033 | 0.17360912 |
| 6720473M08Rik | 0.71426 | 0.11142388 |
| BB157357 | 0.1966 | 0.46704795 |
| 5830469G19Rik | 0.0447 | 1.23752287 |
| E330017L17Rik | 0.05791 | -0.52868147 |
| AI854905 | 0.29739 | -0.45419003 |
| H2bfm | 0.00785 | -1.20570782 |
| A330023F24Rik | 0.69703 | 0.16598665 |
| AW556556 | 0.06659 | 0.8123546 |
| 6330418B08Rik | 0.65608 | 0.34178585 |
| 4931440J10Rik | 0.05442 | -0.54398146 |
| Gm10428 | 0.00144 | -1.12878666 |
| Cdsn | 0.85562 | 0.11590794 |
| D15Ertd55e | 0.00144 | -1.29051721 |
| F830212C03Rik | 0.22865 | 0.68303814 |
| 4930579H20Rik | 0.00144 | -1.29864962 |
| Fggy | 0.7816 | -0.05834483 |
| 9130007G19Rik | 0.00145 | -1.2388121 |
| 2010110K18Rik | 0.973 | -0.04410314 |
| A730011C13Rik | 0.09023 | 0.37021828 |
| A630073K07Rik | 0.38583 | 0.30357359 |
| 3021401L19Rik | 0.00145 | -1.2388121 |
| D8Wsu26e | 0.00144 | -1.34971296 |
| 4933417C20Rik | 0.05378 | 0.70753128 |
| LOC106740 | 0.07381 | 0.46380072 |
| 9030425P06Rik | 0.00144 | 2.32964044 |
| 5830426C09Rik | 0.00827 | -0.66838986 |
| C030034E14Rik | 0.14251 | -0.93792706 |
| 4921507G05Rik | 0.02509 | -3.48915872 |
| 7120432I05Rik | 0.00144 | -1.27730451 |
| Uhrf1bp1l | 0.04469 | 0.45255874 |
| 4930405D01Rik | 0.35654 | -0.64076738 |
| C530008M17Rik | 0.00144 | -1.48564159 |
| 2510003B16Rik | 0.02715 | -1.02338883 |
| Cep55 | 0.29668 | 0.17300513 |
| 4930560O18Rik | 0.10945 | 0.31007278 |
| 4930505N22Rik | 0.0072 | -1.5232852 |
| 5430402O13Rik | 0.03035 | 0.73008506 |
| Pop1 | 0.4502 | -0.12120965 |
| 1700096J18Rik | 0.00144 | -1.28898079 |
| Olfr426 | 0.00145 | -1.22376446 |
| Gm4884 | 0.05801 | -1.02918768 |
| Sv2c | 0.00144 | -1.3348153 |
| Pdcd1lg2 | 0.46886 | -0.36062832 |
| Gpr137b-ps | 0.80825 | -0.04948136 |
| Sncg | 0.00208 | -1.10140164 |
| Tpm1 | 0.05761 | -0.38404741 |
| 2410141K09Rik | 0.00144 | -1.25927895 |
| 2300003K06Rik | 0.00144 | -1.30017714 |
| Olfr905 | 0.33489 | -0.59828786 |
| Cfap126 | 0.28131 | -0.20128033 |
| Pdik1l | 0.07533 | 0.65911852 |
| Gm5414 | 0.00159 | -1.26431393 |
| Acp1 | 0.53877 | 0.1444483 |
| Cacnb2 | 0.01185 | -1.65795253 |
| Shmt1 | 0.01266 | -1.07708845 |
| Hars2 | 0.02825 | 0.43603011 |
| Gm32296 | 0.01227 | -1.85867646 |
| Nlrp1c-ps | 0.24346 | -0.4042874 |
| Duoxa1 | 0.00144 | -1.30713006 |
| Gm1976 | 0.00144 | 1.68672414 |
| Emx2os | 0.00144 | -1.29746368 |
| Phc1 | 0.21529 | -0.2456709 |
| Ttc12 | 0.06247 | 0.3341195 |
| Chrna9 | 0.00305 | -1.14487517 |
| Gm9866 | 0.10601 | -0.99849011 |
| Agxt2 | 0.00144 | -1.61814106 |
| Gm3317 | 0.01611 | 0.99856324 |
| Oprd1 | 0.02668 | -1.35250213 |
| F13a1 | 0.48671 | -0.24407353 |
| R3hdm2 | 0.00322 | -0.99877122 |
| Atp11c | 0.00144 | 2.451698 |
| Kmt2a | 0.04754 | 0.73363994 |
| Gm6878 | 0.02359 | -2.88018444 |
| Mbtps2 | 0.05802 | 0.40148522 |
| Nrg4 | 0.3797 | -0.1777992 |
| Nrg4 | 0.93402 | -0.04201181 |
| Cfh | 0.04147 | 1.11713815 |
| Gm7546 | 0.00145 | -1.25646236 |
| Cyp3a13 | 0.00144 | -1.28116048 |
| Wnk1 | 0.00833 | 0.79602348 |
| Stxbp4 | 0.00144 | -1.28116048 |
| Stoml3 | 0.00377 | -1.47542158 |
| Skp1a | 0.28317 | -0.63525288 |
| Cln3 | 0.01137 | -0.64634843 |
| Cd48 | 0.74681 | -0.0600795 |
| Ascl3 | 0.03319 | -0.97588305 |
| Cdk8 | 0.00144 | 1.53210549 |
| Tle2 | 0.57561 | -0.09905376 |
| 1700016P03Rik | 0.00174 | -1.22657361 |
| Masp2 | 0.01128 | 0.95197698 |
| Rpl9 | 0.02143 | -0.47310957 |
| Copz2 | 0.31348 | -0.26499006 |
| Sirt4 | 0.99548 | -0.00335264 |
| Slc25a18 | 0.76074 | 0.14850905 |
| Umps | 0.00396 | -0.69973359 |
| Snrnp40 | 0.0665 | -0.42385789 |
| Mettl7a3 | 0.61313 | -0.0940827 |
| Fkbp6 | 0.00144 | -1.28898079 |
| Cage1 | 0.83765 | 0.07910844 |
| Gm3002 | 0.00144 | 1.42943511 |
| Sppl2c | 0.21062 | 0.4858192 |
| Mageb16 | 0.00144 | -1.24613586 |
| Rbm19 | 0.01668 | -1.02054344 |
| Tmem185b | 0.21222 | -0.27232006 |
| LOC102642742 | 0.00145 | -1.26219879 |
| Olfr828 | 0.0091 | -0.83593155 |
| Eif2s1 | 0.11344 | 0.3502329 |
| Pcdhb18 | 0.04934 | 1.39812538 |
| Katnal1 | 0.00195 | -0.83996872 |
| Tesk2 | 0.02316 | -0.91460333 |
| Btbd10 | 0.07622 | -0.65567632 |
| Itgal | 0.07585 | -0.68282606 |
| Il1rapl1 | 0.00144 | -1.29864962 |
| Gm3161 | 0.31524 | 0.37602446 |
| BC043934 | 0.40886 | -0.20304832 |
| Cdadc1 | 0.0201 | 0.64702223 |
| Esco1 | 0.04953 | 0.54892747 |
| Sp100 | 0.63941 | -0.37543042 |
| Gm10387 | 0.06431 | 0.42305064 |
| Nfatc3 | 0.03764 | 0.85814563 |
| Mdga1 | 0.14997 | -0.45577231 |
| Defb42 | 0.00687 | -2.00121042 |
| Pih1h3b | 0.14546 | -0.93219348 |
| LOC102640520 | 0.00144 | -1.44399392 |
| Rps26 | 0.00351 | -0.92381225 |
| Yy2 | 0.00144 | 2.14862435 |
| Ndufv3 | 0.00144 | -1.1468259 |
| Ndufa3 | 0.00144 | -1.23150984 |
| Atp1a4 | 0.00145 | -1.2388121 |
| Aurkaip1 | 0.00258 | -1.22132233 |
| Gm10589 | 0.00144 | -1.20555712 |
| Dnah7a | 0.5717 | -0.47492849 |
| Rnd2 | 0.02563 | -0.62444383 |
| Pak7 | 0.01704 | -1.20529812 |
| Olfr97 | 0.15741 | -0.50229249 |
| Olfr77 | 0.00144 | -1.28116048 |
| Olfr47 | 0.07234 | -0.88281418 |
| Vmn2r17 | 0.00144 | -1.28116048 |
| Gm7120 | 0.01717 | 2.37234262 |
| Gm5617 | 0.00813 | -0.90652059 |
| Mc4r | 0.00144 | -1.27730451 |
| Gm5296 | 0.00144 | -0.92830238 |
| 9130401M01Rik | 0.88115 | -0.04713003 |
| Ncoa3 | 0.13531 | -0.30708556 |
| Pisd | 0.08666 | 0.35047678 |
| Mknk1 | 0.08972 | -0.33474941 |
| Rgs19 | 0.93753 | 0.01402364 |
| Gm4566 | 0.08762 | -0.82746032 |
| Pebp1 | 0.00144 | -1.02923806 |
| Vwa3b | 0.00144 | -1.56342466 |
| Tmem201 | 0.25114 | -0.37993706 |
| Tbc1d2 | 0.00456 | 1.3888317 |
| Tbc1d2 | 0.00144 | -1.50283373 |
| Gli1 | 0.05122 | 0.65072564 |
| Gm10614 | 0.4987 | 0.25093292 |
| Hist1h1d | 0.0093 | -1.06147486 |
| Cldn3 | 0.00144 | -1.50259821 |
| Cldn4 | 0.01526 | -2.3464238 |
| Runx1 | 0.00725 | -0.99045622 |
| Rab30 | 0.11868 | -0.33239109 |
| 4933439G19Rik | 0.00676 | -0.91090486 |
| Pds5b | 0.03546 | 0.57036914 |
| Snx11 | 0.05471 | -0.40527163 |
| Snx11 | 0.00257 | -0.84618263 |
| Marveld3 | 0.01546 | -2.45013165 |
| Cryzl1 | 0.0056 | 0.66796697 |
| Rdh13 | 0.68396 | -0.1666912 |
| Prl2c1 | 0.00248 | -1.10620201 |
| Rdx | 0.18306 | 0.38322039 |
| Fmr1 | 0.01993 | 0.91121588 |
| Exoc7 | 0.00144 | -1.3124439 |
| Zfp787 | 0.01385 | -0.66997152 |
| P2rx4 | 0.01638 | -0.53447525 |
| P2rx7 | 0.0384 | -0.87810487 |
| P2rx2 | 0.00822 | -1.05102626 |
| Aqp6 | 0.33798 | -0.60065379 |
| Sim2 | 0.00803 | -0.65106059 |
| LOC102638083 | 0.03531 | -1.14181089 |
| AF067061 | 0.00144 | -1.23155796 |
| Chrnb4 | 0.0034 | -1.32248171 |
| Arl16 | 0.0091 | 0.62368533 |
| B4galt7 | 0.00928 | -0.68034334 |
| Limd2 | 0.00477 | -0.65858623 |
| Prkab1 | 0.6982 | 0.16512437 |
| Orai3 | 0.00144 | -1.03448158 |
| Gm3045 | 0.04108 | -1.12545863 |
| Esrp1 | 0.04698 | -0.59668429 |
| Wars2 | 0.91414 | 0.02710183 |
| Gm10835 | 0.08726 | -0.32558278 |
| Kcnh4 | 0.0483 | -0.47813661 |
| Tnfsf15 | 0.09672 | 0.84955139 |
| Tbc1d1 | 0.89973 | -0.04963488 |
| Adat3 | 0.00477 | -0.96935713 |
| Smg9 | 0.78814 | 0.09161995 |
| Gm33651 | 0.00149 | -1.22582153 |
| Enah | 0.0172 | -0.52820788 |
| Bin2 | 0.5641 | 0.26490581 |
| Mnx1 | 0.64858 | -0.3960432 |
| 4833439L19Rik | 0.00419 | -0.70755038 |
| Rpl39 | 0.00538 | -0.7938655 |
| Zfp352 | 0.19859 | -0.83414817 |
| Tmem252 | 0.26775 | -0.68219318 |
| Faf2 | 0.0149 | -0.57050645 |
| Cacng6 | 0.89194 | -0.10022512 |
| 1700008O03Rik | 0.00144 | -1.56214007 |
| Gm8369 | 0.37828 | 1.29862937 |
| Cd164l2 | 0.6221 | -0.16155033 |
| Krtap5-1 | 0.22619 | -0.70700513 |
| Efna3 | 0.00144 | -1.28116048 |
| Bdh1 | 0.00659 | 1.56579954 |
| Apol7a | 0.2308 | -0.54129939 |
| Lyz1 | 0.28055 | -0.20790663 |
| LOC102642271 | 0.00284 | -2.24142882 |
| Dennd1b | 0.04426 | 0.38500186 |
| Gm4129 | 0.54952 | -0.32414809 |
| Spef2 | 0.04879 | -2.03279869 |
| Calb1 | 0.00144 | -1.24613586 |
| Nnmt | 0.07636 | -0.68778445 |
| Sult1d1 | 0.00144 | -1.28116048 |
| Fndc9 | 0.92039 | -0.03445925 |
| Gm10653 | 0.02078 | -0.75656738 |
| Gm5782 | 0.07265 | 0.53475435 |
| Dnah9 | 0.00144 | -1.40395392 |
| Adm2 | 0.04302 | -1.47902127 |
| Dfnb59 | 0.00144 | -1.27778338 |
| Ecel1 | 0.00144 | -1.30713006 |
| Myo16 | 0.00144 | -1.30017714 |
| Stpg2 | 0.00587 | -1.06500533 |
| Nhsl2 | 0.02665 | 0.77184934 |
| A430105I19Rik | 0.24562 | -0.18300992 |
| Spink14 | 0.03309 | -2.02077201 |
| Btg1 | 0.13206 | -0.24890266 |
| Nipal3 | 0.08032 | -0.64979067 |
| Ccdc112 | 0.9216 | -0.04926361 |
| Olfr1157 | 0.16215 | -0.96976633 |
| Rims3 | 0.00158 | -1.2446827 |
| 4930572G02Rik | 0.00184 | -1.19286777 |
| Vmn2r105 | 0.09098 | -2.57676206 |
| Lcp2 | 0.00145 | -1.42178152 |
| Gm8273 | 0.00318 | -0.85036388 |
| Optc | 0.00373 | -1.2220099 |
| Aldh3a2 | 0.23717 | -0.24103952 |
| Ocel1 | 0.00145 | -1.35335508 |
| Tbc1d21 | 0.00633 | -1.20190496 |
| Mrgprg | 0.45384 | -0.34152987 |
| AA684185 | 0.11243 | -0.66381657 |
| Wdsub1 | 0.69476 | 0.06518853 |
| Spryd3 | 0.07337 | -0.50247933 |
| Prr22 | 0.66928 | 0.14342115 |
| Pex3 | 0.0281 | 0.73867594 |
| Olfr1449 | 0.00144 | -1.31495282 |
| Gls | 0.31738 | 0.17614958 |
| Zfp740 | 0.3151 | -0.32580863 |
| Zfp740 | 0.00144 | -1.19856923 |
| Olfr649 | 0.79567 | 0.14898423 |
| Olfr449 | 0.00144 | -1.36101858 |
| Fam60a | 0.34046 | 0.19407696 |
| Gsdma3 | 0.10229 | -1.10103474 |
| 4933413G19Rik | 0.08566 | -2.72509475 |
| 1810020O05Rik | 0.02805 | -1.28668533 |
| Gm33700 | 0.0411 | -1.06706903 |
| Gm10493 | 0.13548 | -0.92970107 |
| Clcn5 | 0.25356 | -0.90875818 |
| Mrpl10 | 0.92743 | 0.04263038 |
| Ccdc32 | 0.1014 | -0.51021831 |
| 0610006L08Rik | 0.00515 | -1.20518361 |
| Gm4201 | 0.01962 | -1.98380043 |
| Vmn1r219 | 0.79257 | -0.13048356 |
| Gm15772 | 0.00208 | -0.8718176 |
| Lrrc28 | 0.87213 | 0.08422311 |
| Lrrc28 | 0.02127 | -0.47575177 |
| Zfand5 | 0.01056 | 0.62389202 |
| 1500004F05Rik | 0.00144 | -1.59671371 |
| Syn3 | 0.0031 | -1.13721313 |
| Gm10637 | 0.00166 | -1.21062785 |
| Spsb1 | 0.02808 | -0.41052324 |
| Prickle4 | 0.01364 | -0.78012689 |
| Vmn1r118 | 0.08811 | -2.64285185 |
| Vmn1r101 | 0.00145 | -1.2388121 |
| LOC105245869 | 0.00144 | -1.41443942 |
| Gatad2a | 0.00253 | -0.77876033 |
| Glyatl3 | 0.00144 | -1.27992547 |
| Syngr4 | 0.59039 | -0.62740849 |
| 6720473M11Rik | 0.09867 | -0.58031929 |
| Prss33 | 0.00144 | -2.58070858 |
| Slc22a15 | 0.13056 | -0.84876068 |
| Vash1 | 0.27364 | -0.33666559 |
| Gbp8 | 0.00144 | -1.27165604 |
| Celsr3 | 0.27164 | -0.47296327 |
| Slc18a3 | 0.05558 | -0.5596875 |
| Ppp1r8 | 0.00614 | -0.97588758 |
| C230013L11Rik | 0.00406 | -1.30360365 |
| Wdr31 | 0.51783 | -0.14413809 |
| Lig3 | 0.1313 | -1.13264548 |
| Alg1 | 0.00144 | -1.06550145 |
| Zkscan8 | 0.11554 | 0.44584484 |
| Mapk14 | 0.01228 | -0.65004507 |
| Usp33 | 0.00144 | 1.39326442 |
| Macrod2 | 0.07881 | 1.33039343 |
| A630033H20Rik | 0.00232 | 1.18417632 |
| Ceacam13 | 0.00615 | -1.04993521 |
| Ccdc30 | 0.04096 | -0.80313031 |
| Man1a2 | 0.10826 | 0.47797232 |
| Gm10370 | 0.01043 | -1.78128398 |
| Gm3510 | 0.00331 | 1.1678315 |
| Ostf1 | 0.01113 | -0.54696803 |
| 9030404E10Rik | 0.00216 | -1.54444184 |
| A430027H14Rik | 0.06748 | -1.2125324 |
| Ipcef1 | 0.00442 | -1.1333102 |
| 1110020C17Rik | 0.03228 | -1.90631374 |
| Itk | 0.12096 | -1.0405903 |
| Zbtb5 | 0.00627 | -0.78560915 |
| Ugt1a10 | 0.00145 | -1.25592773 |
| Gja5 | 0.47509 | -0.51803385 |
| Commd7 | 0.00145 | -1.25646236 |
| Tbc1d1 | 0.73484 | -0.097928 |
| Dtl | 0.00145 | -1.25646236 |
| P2rx5 | 0.01812 | -0.65979231 |
| Olfr1094 | 0.00144 | -1.28898079 |
| Tmem80 | 0.0239 | -0.75205203 |
| Calhm1 | 0.57908 | -0.39720689 |
| Rbbp9 | 0.01215 | 0.64714541 |
| Tmprss4 | 0.01001 | -1.19982069 |
| Apoa2 | 0.11657 | -0.3171008 |
| Nacc2 | 0.15634 | -0.71210357 |
| Gm13274 | 0.5061 | -0.20702967 |
| Rpl26 | 0.00388 | -0.69877822 |
| Mc3r | 0.76582 | 0.27527447 |
| Fam53b | 0.00468 | -0.97933179 |
| Fam53b | 0.03887 | -0.77314895 |
| Fam53b | 0.02465 | -1.0669183 |
| Gkn1 | 0.00144 | -1.29746368 |
| Ywhaq | 0.02443 | -0.52382832 |
| Rap1gds1 | 0.49883 | -0.10593228 |
| Tll1 | 0.00698 | -0.85271125 |
| Teyorf1 | 0.00144 | -1.29085005 |
| Gm10846 | 0.00152 | -1.25878908 |
| Fam20b | 0.00144 | -1.14009733 |
| Derl2 | 0.61883 | 0.19745594 |
| Wdr45b | 0.25061 | -0.19138333 |
| Ccsap | 0.14346 | -0.25153128 |
| Dnmt3a | 0.1395 | -0.25075157 |
| Inppl1 | 0.01807 | -0.71571444 |
| Adad1 | 0.00172 | -1.10915543 |
| Tmprss13 | 0.01594 | -0.81644603 |
| Lmf1 | 0.55682 | -0.17661938 |
| Tmem208 | 0.00484 | 0.94756779 |
| Tmem208 | 0.04237 | -0.62991397 |
| Lepr | 0.02059 | 1.31028461 |
| Lepr | 0.00144 | -1.82672595 |
| Crem | 0.00144 | -1.28898079 |
| Dact1 | 0.00699 | -1.05651233 |
| Dact3 | 0.00144 | -1.556191 |
| Tmem29 | 0.00308 | -1.39602038 |
| LOC102638515 | 0.01771 | -0.63806464 |
| Ccdc24 | 0.05835 | -0.63811645 |
| Zfyve28 | 0.04168 | -0.64733441 |
| Srsf3 | 0.84161 | -0.03267393 |
| Atp6v0a1 | 0.09043 | -1.18124554 |
| Gm4981 | 0.01554 | -1.6637225 |
| Chrna2 | 0.00869 | -1.18766327 |
| BC048594 | 0.00144 | -1.29051721 |
| B130024G19Rik | 0.00527 | 2.04704448 |
| Gm2814 | 0.00144 | -1.26560808 |
| Cradd | 0.40806 | -0.24818442 |
| Gm3086 | 0.47574 | 0.356244 |
| Gabra2 | 0.00144 | -1.28116048 |
| Gm10657 | 0.10223 | 0.36767928 |
| Lipi | 0.11431 | -0.67785085 |
| Htr7 | 0.00156 | -1.77040536 |
| Krt77 | 0.31865 | -0.74401557 |
| DXBay18 | 0.07013 | -1.04589842 |
| Evc2 | 0.00144 | -1.37174076 |
| Abhd5 | 0.76 | -0.10590687 |
| Gnl1 | 0.00969 | -0.71598635 |
| Hist1h2bm | 0.89243 | -0.05441384 |
| Nkx3-2 | 0.12695 | -0.46344222 |
| Prrg2 | 0.00393 | -0.77170657 |
| Npsr1 | 0.00144 | -1.29085005 |
| Stx8 | 0.00144 | -1.20555712 |
| Hsph1 | 0.08218 | 0.62213522 |
| Gm4858 | 0.01287 | 0.92693065 |
| Mitf | 0.01175 | 0.55148636 |
| Sh3gl3 | 0.69665 | -0.09839879 |
| Prdx6 | 0.54763 | -0.21494267 |
| D830039M14Rik | 0.04363 | -0.54244831 |
| Gm10683 | 0.00383 | -1.13029182 |
| Donson | 0.00175 | 1.6630975 |
| Cyp2j5 | 0.15431 | -0.8025309 |
| Ldah | 0.44402 | -0.20181569 |
| D130079A08Rik | 0.00144 | -1.26934956 |
| Gm10873 | 0.00144 | -1.31884045 |
| Gm10730 | 0.00171 | -1.25646236 |
| 0610010K14Rik | 0.06946 | -0.53674909 |
| St6galnac4 | 0.21825 | -0.37446542 |
| Aire | 0.01183 | -1.04972215 |
| Rffl | 0.01431 | -1.05590685 |
| Slc33a1 | 0.2472 | 0.22461644 |
| Gucy2e | 0.061 | -1.69064995 |
| Pdxk | 0.04477 | -0.63052464 |
| Pcdh15 | 0.00144 | -1.55637959 |
| Pcdh15 | 0.51229 | 0.73494743 |
| Pcdh15 | 0.08913 | -1.08049503 |
| Pcdh15 | 0.00623 | -1.48374382 |
| Pdzd9 | 0.04679 | -1.27251805 |
| Rgs17 | 0.40236 | 0.21892451 |
| Cox11 | 0.27386 | -0.4333006 |
| Lrrc7 | 0.34383 | -0.619327 |
| Lrrc9 | 0.00144 | -1.25118919 |
| Ralgapa2 | 0.67995 | -0.14297215 |
| Epha10 | 0.38634 | 0.24600678 |
| Ms4a2 | 0.00259 | -1.45078974 |
| Gm3604 | 0.5574 | 0.25005639 |
| Vmn2r35 | 0.00144 | 1.37120687 |
| Vmn2r35 | 0.00158 | -1.15468698 |
| Ano9 | 0.00144 | -1.27165604 |
| Olfr1111 | 0.00144 | -1.39713491 |
| Mgst1 | 0.08227 | 0.48519938 |
| Ccl26 | 0.00144 | -1.31495282 |
| Ythdf2 | 0.13875 | -0.32358183 |
| Rangrf | 0.0042 | -0.87813356 |
| 1700023E05Rik | 0.00144 | -1.29878664 |
| Gm5486 | 0.00144 | -1.25162917 |
| Olfr1466 | 0.33902 | -0.39025848 |
| Tbxas1 | 0.00144 | -1.28116048 |
| Gm20492 | 0.00262 | -0.92524159 |
| Sfxn5 | 0.00634 | -1.7873795 |
| Gm10375 | 0.00577 | -1.11999871 |
| Chd2 | 0.48563 | -0.19474238 |
| Mettl13 | 0.43988 | -0.24163092 |
| Uros | 0.65112 | -0.24863608 |
| LOC100043315 | 0.00158 | -1.2446827 |
| Gm38682 | 0.00145 | -1.22376446 |
| Tet3 | 0.01266 | -0.53433601 |
| Tusc3 | 0.02206 | 0.58884354 |
| Rbbp6 | 0.01778 | 0.77955214 |
| Cep250 | 0.06279 | 0.52010744 |
| Gm2379 | 0.05334 | -1.04047549 |
| Gm11536 | 0.00144 | -1.30713006 |
| Olfr982 | 0.0019 | -1.15423558 |
| LOC102638466 | 0.00144 | -1.26934956 |
| A730067D02Rik | 0.00144 | -1.28116048 |
| Six6 | 0.06908 | -2.1304601 |
| Ppapdc1b | 0.10171 | 0.36770336 |
| Mansc4 | 0.00152 | -1.25878908 |
| Ftx | 0.38045 | -0.28240991 |
| Creb3l3 | 0.00956 | -1.29529628 |
| Cngb1 | 0.11677 | 0.66450823 |
| Cngb1 | 0.15949 | -0.35346979 |
| Elac2 | 0.33361 | -0.21468192 |
| Akr1b3 | 0.77657 | 0.2028807 |
| Gm34542 | 0.00145 | -1.22376446 |
| Mn1 | 0.0187 | -0.74150871 |
| Cuta | 0.00219 | -0.99718169 |
| Kri1 | 0.46048 | -0.42884092 |
| Olfr866 | 0.00296 | -1.49566245 |
| Zfp605 | 0.59478 | 0.45818181 |
| Olfr766 | 0.00144 | -1.29085005 |
| Olfr566 | 0.00144 | -1.28749883 |
| Rab8a | 0.00256 | -1.28076576 |
| Zfp266 | 0.92926 | -0.01873425 |
| Akap7 | 0.00144 | 2.64848799 |
| Tmsb15l | 0.00144 | -1.27165604 |
| Smr3a | 0.07515 | -2.41265501 |
| Gm4311 | 0.00144 | -1.31884045 |
| Plekha7 | 0.16042 | 0.36017998 |
| Svopl | 0.02721 | -2.1397227 |
| Stx17 | 0.96394 | 0.00973229 |
| Stx17 | 0.00528 | 1.04043292 |
| Cd37 | 0.05353 | -0.9072762 |
| Ccdc42b | 0.22535 | -0.4003653 |
| 9430038I01Rik | 0.16279 | 0.62287608 |
| Stam2 | 0.1483 | -0.35584033 |
| Pgpep1l | 0.013 | -1.31949526 |
| Olfr1045 | 0.00144 | -1.25118919 |
| Gm2102 | 0.00144 | -2.44608204 |
| Snap29 | 0.00726 | -0.7427629 |
| Slco5a1 | 0.41958 | -0.17608383 |
| Slco5a1 | 0.00276 | -1.81175118 |
| Pin4 | 0.43345 | 0.15207173 |
| Olfr728 | 0.00945 | -1.20864476 |
| Lims2 | 0.00153 | -1.0381563 |
| Sprr1b | 0.00145 | -1.26219879 |
| Frat2 | 0.00184 | -1.46838792 |
| Brd4 | 0.00144 | -1.12627178 |
| Rrm2 | 0.1641 | -0.36950715 |
| Ctdspl | 0.00176 | -0.87188968 |
| Myh6 | 0.00181 | -0.85291762 |
| Zar1l | 0.00144 | -1.26934956 |
| Insr | 0.03463 | -0.50976878 |
| Pla2g2d | 0.13648 | -0.66039046 |
| Tox2 | 0.00873 | -0.76776818 |
| Pkhd1 | 0.00144 | -1.51704382 |
| Hspa4l | 0.00485 | 1.03754513 |
| Ebpl | 0.00144 | -1.03465086 |
| Ptprz1 | 0.04564 | -1.13077247 |
| Gm626 | 0.03326 | -0.48505761 |
| Samd3 | 0.00145 | -1.2388121 |
| Samd3 | 0.00173 | -1.99025039 |
| Samd3 | 0.00144 | -1.29746368 |
| Samd1 | 0.00546 | -0.97058987 |
| 6330562C20Rik | 0.77095 | 0.18067118 |
| B130034C11Rik | 0.00156 | -1.01937225 |
| A230070E04Rik | 0.0792 | -0.65702515 |
| Arsi | 0.04591 | -0.84847805 |
| Zfp62 | 0.73229 | -0.12553413 |
| Slc16a7 | 0.01067 | -1.05476252 |
| Plcb4 | 0.00144 | 1.46293428 |
| Gm20939 | 0.26416 | -1.31384558 |
| Plcb1 | 0.00159 | 2.52683668 |
| Plcb2 | 0.01685 | -2.00500147 |
| Pcdhb1 | 0.23134 | -0.59584881 |
| Vmn2r4 | 0.67618 | -0.23683113 |
| Commd1 | 0.01893 | -0.7247408 |
| Prdm15 | 0.10962 | -0.85524553 |
| Zfp931 | 0.00303 | 2.65418843 |
| Nfasc | 0.9117 | -0.05883879 |
| Dnase2a | 0.91962 | -0.03079719 |
| Ptpn23 | 0.03731 | -0.42976647 |
| Plb1 | 0.00145 | -1.2388121 |
| Cnih2 | 0.02072 | 0.53961976 |
| Dgkb | 0.09214 | -0.71434724 |
| Mettl6 | 0.74403 | -0.11029302 |
| Syk | 0.17022 | -0.42513938 |
| Hacd4 | 0.63868 | -0.12358949 |
| Mettl7a1 | 0.8018 | -0.11258876 |
| Mfn2 | 0.53483 | -0.21595047 |
| Mfn2 | 0.12567 | 0.40547854 |
| Srbd1 | 0.01565 | -1.01936982 |
| Tm4sf19 | 0.00176 | -1.24266807 |
| LOC102643177 | 0.05024 | -0.84490899 |
| Gm1123 | 0.40891 | 0.18517924 |
| Fam174a | 0.60164 | 0.19068125 |
| Mageb3 | 0.00229 | -1.21636428 |
| Gmnn | 0.00177 | -1.03713654 |
| Btn2a2 | 0.00144 | -1.27165604 |
| Sult2a6 | 0.00144 | -1.43903703 |
| 1700061I17Rik | 0.52995 | -0.42774921 |
| Nrgn | 0.01017 | -1.00825597 |
| Scarf2 | 0.00266 | -0.87093291 |
| Zxdb | 0.00927 | 0.87022717 |
| Krit1 | 0.00144 | 2.31718451 |
| Zfp384 | 0.53161 | -0.14008552 |
| Nup155 | 0.08122 | 0.36480539 |
| Scamp4 | 0.00181 | -1.15487447 |
| Smc2 | 0.0037 | 1.33879579 |
| LOC102642864 | 0.00221 | -0.95741382 |
| Zfp57 | 0.51628 | 0.36083717 |
| Thnsl1 | 0.05986 | 0.65584517 |
| Mc1r | 0.00144 | -1.29746368 |
| Gm10640 | 0.00396 | -1.23373852 |
| Trim55 | 0.97189 | 0.00894428 |
| Trim65 | 0.08566 | -1.83922577 |
| B4galt1 | 0.00277 | -0.83119448 |
| Mrgpra2a | 0.1026 | -0.88228623 |
| Mrgbp | 0.07532 | 0.41068477 |
| Rps11 | 0.00144 | -1.02983159 |
| Tnfaip8l3 | 0.00462 | -1.38699729 |
| Notch1 | 0.01063 | 1.73988169 |
| 4921517D16Rik | 0.00144 | -1.27730451 |
| Zfp786 | 0.62397 | -0.16552211 |
| Dnmt3aos | 0.20044 | 0.89541038 |
| Gm26806 | 0.00144 | -1.40758963 |
| Spag11b | 0.59456 | 0.15744455 |
| Mrps33 | 0.81332 | 0.06474123 |
| Stx3 | 0.99819 | -0.0006183 |
| Ppp1r7 | 0.07604 | 0.35788626 |
| Mkrn1 | 0.03993 | -0.49897304 |
| Mkrn3 | 0.00304 | 1.09196053 |
| 3110082I17Rik | 0.36102 | -0.18561656 |
| Wipf1 | 0.4252 | 0.2963 |
| BC030867 | 0.02191 | -0.83275592 |
| Lta4h | 0.00362 | -0.72633668 |
| Nptn | 0.01288 | 0.65425715 |
| Nptn | 0.34501 | 0.16172459 |
| Tm7sf2 | 0.38525 | 0.31584069 |
| Tceb1 | 0.98832 | 0.00319508 |
| Grm2 | 0.04443 | -0.38020163 |
| Gm10588 | 0.10758 | -0.93205857 |
| Mier3 | 0.00144 | 1.72852465 |
| Gm13180 | 0.05757 | -0.90041701 |
| Vsig10l | 0.1997 | -0.60310386 |
| Matn1 | 0.00144 | -1.30713006 |
| Matn4 | 0.00999 | -0.89123088 |
| Ttll13 | 0.00145 | -1.26219879 |
| Gm36769 | 0.95178 | -0.02657248 |
| Hnrnpc | 0.11894 | 0.26418196 |
| Olfr427 | 0.00144 | -1.31884045 |
| Lgals3 | 0.00144 | -2.09881121 |
| Prcp | 0.10531 | -0.58243253 |
| Sec22c | 0.00219 | -0.91310192 |
| Gm2483 | 0.00144 | -1.30713006 |
| Kcnk18 | 0.05858 | -1.92737031 |
| Gm35772 | 0.00833 | -0.59693023 |
| Ankrd13d | 0.02867 | -0.59926168 |
| Gspt1 | 0.15977 | 0.22960541 |
| Gm10366 | 0.8179 | 0.05955091 |
| 4930515L03Rik | 0.00144 | -1.27165604 |
| Fbxo27 | 0.50696 | -0.21677278 |
| Gm14149 | 0.00145 | -1.51999521 |
| Myo18a | 0.00144 | -1.63107747 |
| 1700022N22Rik | 0.14769 | -0.91232186 |
| AI847159 | 0.00144 | -2.91494461 |
| Pkig | 0.01581 | -0.80654192 |
| Braf | 0.10849 | 0.68144399 |
| Igf2bp2 | 0.19046 | -0.39769946 |
| Cdca8 | 0.40123 | -0.19497244 |
| Htr3b | 0.00184 | -1.27646041 |
| Fam19a4 | 0.00728 | -1.50211756 |
| Ggps1 | 0.87638 | -0.0389638 |
| Zfp949 | 0.00342 | 0.84783904 |
| Tas2r114 | 0.03161 | -1.89531338 |
| 4933406I18Rik | 0.05575 | -2.25767149 |
| Yipf4 | 0.04616 | 0.47778492 |
| Yipf2 | 0.02102 | -0.52042691 |
| Gsta2 | 0.08045 | -0.37637575 |
| Zfp180 | 0.87951 | -0.03028557 |
| Dock3 | 0.00158 | -1.15468698 |
| Gm2518 | 0.06098 | -0.71842608 |
| Klra22 | 0.00353 | -1.06721129 |
| Klra22 | 0.30762 | 0.72293835 |
| Gm6729 | 0.0015 | -0.94766026 |
| Ppfibp2 | 0.23248 | 0.38640992 |
| Gm5115 | 0.00194 | 3.18762979 |
| Ap1s3 | 0.73139 | -0.19432738 |
| Klk5 | 0.00144 | -1.52145939 |
| Eif2b4 | 0.00298 | -0.83664888 |
| Bivm | 0.53182 | 0.14850675 |
| Lrrc27 | 0.11638 | -0.30847286 |
| LOC105247169 | 0.02077 | 0.56292427 |
| Catsperd | 0.00144 | -1.32599123 |
| Gm7854 | 0.28077 | -0.41409195 |
| a | 0.59713 | 0.18065514 |
| Lactb2 | 0.49458 | 0.11273686 |
| Cep128 | 0.02072 | -1.14633247 |
| Gm13152 | 0.01705 | 1.28449962 |
| Olfr773 | 0.00144 | -1.29085005 |
| Ostm1 | 0.18267 | -0.5745336 |
| Gm10823 | 0.00477 | -1.53396486 |
| Rufy3 | 0.21141 | 0.36818419 |
| 3632451O06Rik | 0.27028 | -0.27823005 |
| Nanos3 | 0.04658 | -0.44799556 |
| Ano3 | 0.00144 | -1.2383131 |
| Rps17 | 0.00828 | -0.61345164 |
| Csf1 | 0.01761 | -0.56061456 |
| Vwf | 0.00494 | -1.25791981 |
| Gm36315 | 0.17519 | -2.28679707 |
| 2410127L17Rik | 0.00324 | 0.92341671 |
| Fgf23 | 0.1817 | 0.38924139 |
| Fgf20 | 0.00144 | -1.26560808 |
| Mfsd4 | 0.53701 | -0.26022368 |
| Mfsd6 | 0.00571 | 0.96728222 |
| Mfsd6 | 0.57228 | 0.2266409 |
| Gm10760 | 0.00261 | -1.36811222 |
| Cgn | 0.24739 | -0.63917928 |
| Atp6v1a | 0.00539 | 0.75521265 |
| Gm1966 | 0.00163 | 1.76434138 |
| 4732491K20Rik | 0.06486 | -0.49467363 |
| Gm15056 | 0.13017 | -0.78800402 |
| Olfr547 | 0.05772 | -2.1439486 |
| Pycr1 | 0.00767 | -1.02946836 |
| Ccdc184 | 0.00189 | -1.19673561 |
| Ccdc13 | 0.01235 | -0.85350315 |
| Ccdc53 | 0.44325 | 0.34457745 |
| Slc22a30 | 0.0345 | -0.8556802 |
| Gm10405 | 0.04058 | -0.45164713 |
| Olfr1474 | 0.00146 | -1.27088387 |
| Strada | 0.70592 | -0.08533172 |
| Olfr458 | 0.2434 | -1.03910697 |
| Ndfip1 | 0.01417 | -0.59997106 |
| Olfr157 | 0.00144 | -1.29085005 |
| Tmsb10 | 0.00303 | -0.95357085 |
| 2610020H08Rik | 0.21802 | 0.40162786 |
| Dok6 | 0.00144 | -1.25118919 |
| Nlrp4e | 0.09798 | -0.92809044 |
| 4732440D04Rik | 0.41546 | 1.17901266 |
| Sf1 | 0.00506 | -0.68666383 |
| Relb | 0.00158 | -1.01933567 |
| Frmd8os | 0.06107 | -0.53017015 |
| Cep131 | 0.00311 | -0.73916445 |
| Rbpms2 | 0.32395 | -0.26504425 |
| Khk | 0.39967 | -0.38685336 |
| Sac3d1 | 0.00811 | -0.93381564 |
| Cdk9 | 0.21378 | -0.31681331 |
| Scube3 | 0.00144 | -1.27165604 |
| Ap5b1 | 0.00786 | -0.5779355 |
| Pik3c2b | 0.08798 | -0.55317528 |
| Nxpe5 | 0.00144 | -1.29049509 |
| A930017K11Rik | 0.53055 | -0.17369896 |
| Amer1 | 0.85502 | -0.0403517 |
| Pced1b | 0.65359 | -0.10420705 |
| Myo18b | 0.00621 | -0.63396714 |
| Slc46a1 | 0.04164 | -0.62664902 |
| Grcc10 | 0.15675 | -0.88123723 |
| 6430553K19Rik | 0.00144 | -1.27165604 |
| Top3a | 0.02082 | -0.73665322 |
| 4921539H07Rik | 0.97991 | 0.03668878 |
| Tex11 | 0.009 | -1.1398278 |
| Nudcd1 | 0.2766 | 0.50259821 |
| Sema3g | 0.06818 | -0.49815201 |
| Prmt7 | 0.83732 | 0.11093365 |
| Mmd | 0.29517 | -0.20348142 |
| A230006K03Rik | 0.00981 | -0.96989034 |
| Dmd | 0.01584 | 2.27741832 |
| Dmd | 0.02093 | 1.6668335 |
| Ermard | 0.06218 | 0.35151805 |
| Nat9 | 0.33742 | -0.43032456 |
| Gm9777 | 0.04163 | -1.5914047 |
| Gm14151 | 0.10108 | 0.4200845 |
| 4930431F10Rik | 0.00144 | -1.27165604 |
| Rabgef1 | 0.0083 | -0.64278738 |
| Gm6093 | 0.00167 | -1.37548212 |
| U2af2 | 0.46296 | -0.14534022 |
| Klra12 | 0.00144 | -1.26560808 |
| Fcho1 | 0.03993 | -0.37613486 |
| Gm6936 | 0.02993 | -0.62831958 |
| 6330416G13Rik | 0.78045 | -0.09728223 |
| Gm10723 | 0.00144 | -1.31884045 |
| Clec18a | 0.23228 | -0.23562137 |
| Scube3 | 0.00144 | -1.51184487 |
| Scube3 | 0.00149 | -1.21097148 |
| Acsf2 | 0.00621 | -0.8982377 |
| Scgb1b24 | 0.00144 | -1.34813137 |
| Resp18 | 0.00144 | -1.26934956 |
| Homer3 | 0.07819 | -0.56510228 |
| Cnnm2 | 0.0029 | -0.84736268 |
| Cnnm3 | 0.00144 | -1.41371683 |
| Zfp944 | 0.00144 | 1.68439335 |
| Cnot3 | 0.02394 | -0.92006307 |
| Cort | 0.62365 | -0.11699007 |
| Gm4466 | 0.77581 | -0.17806973 |
| 4931429P17Rik | 0.00145 | -1.2388121 |
| Pcsk4 | 0.67979 | -0.07072436 |
| Atp1b4 | 0.00145 | -1.27532353 |
| 4921504E06Rik | 0.09865 | -1.48746529 |
| Gm32464 | 0.04377 | -2.1159968 |
| Hdhd2 | 0.00148 | -0.91652506 |
| Cyp4b1-ps2 | 0.05934 | -1.08684252 |
| Cdk15 | 0.60448 | 0.40699481 |
| BC049730 | 0.00144 | -1.26153594 |
| Kif5a | 0.00364 | -1.6356302 |
| Paip2 | 0.00144 | 1.14134691 |
| Zfand1 | 0.01167 | 1.55580439 |
| Pvrl2 | 0.00648 | -0.7181356 |
| Atp5sl | 0.02784 | -0.67687758 |
| Ppcs | 0.0391 | -0.44200577 |
| Dedd2 | 0.00209 | -1.06165686 |
| 2310061N02Rik | 0.00794 | -1.70910791 |
| 1700028M03Rik | 0.00144 | -1.28898079 |
| Gm5481 | 0.0297 | -0.42251686 |
| Ms4a14 | 0.00144 | 1.52990772 |
| Sp3os | 0.05745 | -0.59430496 |
| Gm13446 | 0.00787 | -1.01334489 |
| Timm10b | 0.47037 | -0.54149601 |
| Timm10b | 0.02295 | -0.73748818 |
| Timm10b | 0.02197 | -1.2349942 |
| Dnhd1 | 0.01475 | -1.09499705 |
| Gm5129 | 0.07565 | -0.96198825 |
| Cdk4 | 0.41571 | -0.24183223 |
| Srrm2 | 0.95479 | -0.02006774 |
| Pam16 | 0.02091 | -0.74916469 |
| Foxi3 | 0.00272 | -2.4867876 |
| Foxi1 | 0.00981 | 0.71756856 |
| Gpr6 | 0.68172 | 0.10631629 |
| Cd44 | 0.00473 | -0.79556335 |
| Cd44 | 0.22888 | -0.50211756 |
| Timm10b | 0.01288 | -0.62526143 |
| Rpusd3 | 0.12362 | -0.44626187 |
| Stk11 | 0.10173 | -0.52445586 |
| Cnot1 | 0.1046 | -0.30464192 |
| LOC102636406 | 0.00144 | -1.28898079 |
| Rnf215 | 0.01187 | -0.53728331 |
| H2-M1 | 0.00144 | -1.29085005 |
| Dspp | 0.07147 | -1.07502219 |
| Atp9a | 0.09031 | -0.43544272 |
| Gm3948 | 0.00144 | -1.26301121 |
| 2310079G19Rik | 0.84741 | 0.06789792 |
| Vmn1r65 | 0.07206 | 0.81530996 |
| Vmn1r63 | 0.44848 | -0.31475574 |
| Ifna13 | 0.11772 | -1.73022785 |
| Tmem128 | 0.04696 | -0.426893 |
| Mtx3 | 0.40176 | -0.3362147 |
| Olfr8 | 0.04741 | -0.75767283 |
| Olfr9 | 0.0713 | -0.38634556 |
| Ncor1 | 0.77903 | 0.06988524 |
| Olfr3 | 0.05362 | -1.06828626 |
| Ankrd42 | 0.2171 | -0.68448404 |
| Cebpb | 0.08691 | -0.57079061 |
| Gemin7 | 0.1292 | -0.50935387 |
| Cebpe | 0.64614 | -0.08384626 |
| Olfr1502 | 0.00144 | -1.27165604 |
| Cenpo | 0.92853 | 0.04257417 |
| Jakmip1 | 0.00144 | -1.29864962 |
| Gm10779 | 0.00158 | -1.2446827 |
| Plekhg5 | 0.0096 | -0.55076706 |
| Aebp2 | 0.0149 | 0.56904465 |
| Aebp2 | 0.12387 | -0.39758095 |
| Mettl11b | 0.03313 | -0.63248887 |
| Gm5486 | 0.22647 | -0.54096767 |
| Krt36 | 0.00367 | -1.16778291 |
| Serpina1a | 0.29186 | -1.08136963 |
| Athl1 | 0.44159 | -0.52725336 |
| Gpc3 | 0.00144 | -1.31495282 |
| Nde1 | 0.54346 | -0.09263188 |
| Acsl3 | 0.57302 | 0.36253776 |
| Mybpc1 | 0.46316 | -0.19327689 |
| Atrn | 0.85788 | -0.06257456 |
| Papln | 0.3352 | -0.16863996 |
| 2300005B03Rik | 0.92252 | -0.01594885 |
| 4930467J12Rik | 0.0031 | -1.09550759 |
| Lrrtm4 | 0.81599 | 0.09168716 |
| Gm7420 | 0.04043 | 1.52083351 |
| Trav3-4 | 0.00184 | -1.79198148 |
| Arhgef1 | 0.01373 | -0.67621299 |
| Acnat2 | 0.15328 | -0.77046581 |
| Nrxn3 | 0.15172 | -0.51896912 |
| Vmn2r24 | 0.00144 | -1.32766452 |
| Lce3e | 0.00144 | -1.28116048 |
| Fut10 | 0.50188 | -0.19082449 |
| Scrt1 | 0.75845 | 0.12839238 |
| Tcf24 | 0.00427 | -1.00578904 |
| Gsdmc4 | 0.14579 | -2.41729881 |
| Gm12128 | 0.13119 | -0.91948177 |
| Ptdss1 | 0.37324 | -0.18081087 |
| Vmn2r73 | 0.00144 | -1.32766452 |
| Muc20 | 0.00449 | -1.49614359 |
| Gnaq | 0.01371 | 0.74870213 |
| Chrd | 0.49229 | -0.50119546 |
| Olfr1408 | 0.00145 | -1.26219879 |
| Tlx2 | 0.00144 | -1.28898079 |
| Tas2r123 | 0.00149 | -1.22582153 |
| Ptn | 0.00758 | -1.34104157 |
| LOC102631952 | 0.00313 | -0.75592557 |
| Gm960 | 0.77527 | -0.15189316 |
| Gm5724 | 0.00144 | -1.33632959 |
| Dtl | 0.1725 | 0.55144266 |
| Zfp872 | 0.23722 | -0.41779845 |
| Samhd1 | 0.00145 | -1.24191372 |
| Gm36299 | 0.09325 | 0.48044643 |
| Cd27 | 0.00379 | -1.12694059 |
| Asb16 | 0.01801 | -0.50864682 |
| Hoxa10 | 0.00144 | -1.26560808 |
| Tas2r117 | 0.0156 | -1.08617297 |
| Prl7c1 | 0.00144 | -1.28898079 |
| Prl7b1 | 0.41426 | -0.63336975 |
| Cfap74 | 0.02685 | 1.12859153 |
| Nploc4 | 0.00144 | -0.97807547 |
| Gm10804 | 0.00145 | -1.66534991 |
| Grip1 | 0.09679 | 0.71002067 |
| Efcab12 | 0.85376 | 0.06410935 |
| Kcnj5 | 0.96887 | 0.00900456 |
| Vmn1r157 | 0.00158 | -1.2446827 |
| Trim12c | 0.00585 | 3.09873766 |
| Med14 | 0.06557 | 0.377708 |
| Cd22 | 0.01607 | -1.62083216 |
| Crip1 | 0.00144 | -1.37572945 |
| Tvp23a | 0.00418 | -1.08459353 |
| LOC102642943 | 0.1814 | 0.23526691 |
| Rspo3 | 0.32014 | -1.08166783 |
| Rspo4 | 0.0091 | -1.53027053 |
| Syce1l | 0.00144 | -1.28898079 |
| Rps27 | 0.00145 | -1.09727846 |
| Gm29992 | 0.06127 | -0.89697361 |
| Adamts3 | 0.9676 | -0.01349125 |
| Cfap44 | 0.08133 | -1.95647196 |
| Tgm3 | 0.14222 | -0.67523157 |
| Olfr963 | 0.00144 | -1.30713006 |
| Olfr463 | 0.00144 | -1.30783085 |
| Actn2 | 0.77115 | -0.15838822 |
| Foxm1 | 0.00144 | -1.43713248 |
| Olfr611 | 0.14902 | -1.34522238 |
| Clec2f | 0.00144 | -1.26560808 |
| Sox3 | 0.00145 | -1.2388121 |
| Zbtb8os | 0.81209 | -0.06054577 |
| Pglyrp3 | 0.29728 | -0.70515907 |
| Fktn | 0.00144 | -1.34635255 |
| Prok1 | 0.00144 | -1.26428512 |
| Cdk4 | 0.00841 | -0.77238193 |
| Tm4sf5 | 0.0247 | -0.74029056 |
| 0610042G04Rik | 0.00144 | -1.26049461 |
| Akr1c18 | 0.50742 | -0.34834483 |
| Nxf7 | 0.00273 | -1.41952492 |
| Gm10220 | 0.05031 | -0.80649447 |
| Hoxb13 | 0.00144 | -1.20555712 |
| Olfr1253 | 0.00144 | -1.28898079 |
| Mapkapk5 | 0.47763 | -0.14205387 |
| Tmem179 | 0.00764 | -0.64788098 |
| Nit1 | 0.00321 | -0.84752355 |
| Ntn1 | 0.09117 | -0.46020971 |
| Rtn1 | 0.02311 | -0.62969667 |
| Zfp780b | 0.00494 | 1.00499671 |
| Cyp3a57 | 0.00144 | -1.2383131 |
| Olfr208 | 0.01134 | -1.02600084 |
| Cnksr2 | 0.00144 | -1.28116048 |
| Smco3 | 0.04706 | -0.94383626 |
| Olfr1015 | 0.05277 | -0.5520152 |
| C1s1 | 0.00299 | -1.2018878 |
| Dnajc24 | 0.63636 | 0.14501607 |
| Asic4 | 0.06395 | -1.44389862 |
| Prpf40b | 0.02391 | -0.50157266 |
| Dnajc10 | 0.9494 | 0.016766 |
| Cyp2b23 | 0.04258 | -1.34973697 |
| E530001F21Rik | 0.00144 | -1.29864962 |
| Lman2 | 0.00237 | -1.05255027 |
| Art3 | 0.00844 | 0.74635063 |
| Ing4 | 0.3932 | -0.19187119 |
| Pex6 | 0.00896 | -0.75118928 |
| Tbx20 | 0.00598 | -0.7394704 |
| Mpdu1 | 0.00144 | -0.96452059 |
| D630029K05Rik | 0.00305 | -1.39343567 |
| Gm13021 | 0.10998 | -0.41753177 |
| Actbl2 | 0.00144 | -1.26153594 |
| Cacfd1 | 0.83458 | -0.07786121 |
| Lrsam1 | 0.46582 | -0.26461055 |
| Bmx | 0.84808 | 0.03854398 |
| Bri3 | 0.14526 | -0.55641648 |
| 4931406P16Rik | 0.01899 | -1.19825122 |
| LOC432842 | 0.04819 | -1.98502173 |
| Anp32a | 0.3533 | 0.15609404 |
| Hmgn2 | 0.11404 | -0.27164608 |
| Gm9867 | 0.06358 | -2.15048697 |
| AU017193 | 0.00144 | 1.26025304 |
| 2410141K09Rik | 0.00144 | 2.40393962 |
| Rabgap1 | 0.20508 | -0.54968947 |
| Oxt | 0.51229 | 0.36077306 |
| Pdlim7 | 0.38331 | -0.47751274 |
| Hmgb1-rs17 | 0.09618 | -0.37772182 |
| Prr3 | 0.01408 | -0.84479545 |
| Mpp3 | 0.44085 | 0.24346766 |
| Nrip2 | 0.02078 | -1.2226244 |
| Tomm40 | 0.95851 | 0.0288795 |
| Zfp133-ps | 0.04588 | -0.37643924 |
| LOC102642173 | 0.00144 | -1.34484129 |
| Gm13088 | 0.00149 | -1.2703832 |
| Diap2 | 0.00144 | 2.05573434 |
| Hpca | 0.9093 | -0.03159746 |
| Coq10a | 0.04159 | -0.59478554 |
| Ddx21 | 0.18806 | 0.21722939 |
| Pvrl4 | 0.00144 | -1.20555712 |
| Gm10516 | 0.00315 | 0.84555252 |
| Serpina3a | 0.06334 | -0.72282768 |
| Serpina3a | 0.00144 | -1.27165604 |
| Serpina3b | 0.16623 | -0.39176289 |
| Galr1 | 0.00144 | -1.23532597 |
| Gm6370 | 0.05116 | -0.76881734 |
| Kcnmb4 | 0.08768 | -1.62243249 |
| Acmsd | 0.00144 | -1.27165604 |
| Pkib | 0.08292 | 0.5375578 |
| Pkib | 0.00145 | -1.2388121 |
| Spata2l | 0.23647 | -0.79986915 |
| Thsd7a | 0.10066 | -0.45485331 |
| Zfp94 | 0.419 | 0.45170761 |
| Rgs6 | 0.22034 | 0.37373518 |
| Gm2511 | 0.00597 | -1.77732725 |
| Cldn34b3 | 0.00144 | -1.25118919 |
| Trp63 | 0.40326 | -0.15256501 |
| Slc24a2 | 0.03075 | -0.79871176 |
| Slc24a2 | 0.06321 | -0.93147196 |
| 4933402N03Rik | 0.03809 | -0.84374529 |
| Faim2 | 0.01839 | -1.55400519 |
| Vmn2r90 | 0.11479 | -3.0619968 |
| Gja8 | 0.00432 | -1.20547544 |
| Xdh | 0.0235 | 0.73502595 |
| Foxd4 | 0.28678 | -0.68667141 |
| Foxd2 | 0.25209 | -0.42906845 |
| Gm10677 | 0.00144 | -1.34390295 |
| Zcchc2 | 0.05087 | -0.50801035 |
| Col25a1 | 0.23653 | -0.47856566 |
| Tfcp2 | 0.1166 | 0.25681693 |
| Tfcp2 | 0.07011 | -0.35661254 |
| Arf5 | 0.00144 | -1.66797791 |
| Tsc22d1 | 0.15573 | 0.42159462 |
| Ret | 0.59359 | 0.10566761 |
| Gm10854 | 0.0057 | -1.19115562 |
| Emd | 0.04003 | -0.50275123 |
| Hoxd12 | 0.00144 | -1.25118919 |
| Kalrn | 0.10014 | 0.71833399 |
| Tcp10b | 0.5031 | -0.36358867 |
| Slc8a3 | 0.1168 | -0.51845857 |
| Defb40 | 0.00144 | -1.26934956 |
| Stk26 | 0.01004 | -1.26576709 |
| Xylt2 | 0.01061 | -0.75402329 |
| Cxcl11 | 0.18737 | -0.34516726 |
| Slc35e2 | 0.44117 | 0.15614548 |
| Bicd2 | 0.02024 | 0.51259358 |
| Anapc15 | 0.26623 | -0.43914957 |
| Olfm1 | 0.00271 | -0.78478973 |
| Gm5602 | 0.33046 | -0.24906805 |
| LOC105246057 | 0.2098 | -0.33730092 |
| Ntpcr | 0.58855 | -0.11470814 |
| Ccdc28b | 0.00454 | -0.77151587 |
| A330069E16Rik | 0.59242 | -0.08829936 |
| Atf1 | 0.02134 | 0.50910478 |
| Vmn2r27 | 0.00144 | -1.30713006 |
| Polr1e | 0.24604 | 0.2917753 |
| Zfp52 | 0.01326 | 1.74627598 |
| Zbtb7a | 0.12293 | 0.41720621 |
| Zfp59 | 0.06646 | 0.55522283 |
| Yaf2 | 0.0758 | 0.41758862 |
| Rfc4 | 0.18836 | -0.25707325 |
| Rfc4 | 0.0182 | -0.49961061 |
| Serpinb3d | 0.00144 | -1.30713006 |
| Mob1a | 0.37689 | 0.41873464 |
| Mfsd2b | 0.3474 | -0.21304194 |
| Gm5591 | 0.34291 | -0.57465612 |
| Acvr1 | 0.25473 | -0.18168695 |
| Acvr1 | 0.18831 | -0.58664225 |
| Kcnj3 | 0.29804 | -0.27480261 |
| Zfp534 | 0.0164 | 1.08843666 |
| Pm20d1 | 0.58093 | -0.12761476 |
| Reep6 | 0.00675 | -0.91026561 |
| Tas2r131 | 0.50378 | -0.19623821 |
| Adamts4 | 0.44969 | 0.2104968 |
| Col14a1 | 0.45386 | 0.18428197 |
| Plekha4 | 0.00735 | -1.87430955 |
| Gm3458 | 0.00144 | -1.27992547 |
| Fam160a1 | 0.15249 | -0.27606 |
| Sbf1 | 0.01873 | -0.58076265 |
| Fndc4 | 0.02379 | -0.63721357 |
| Pfn2 | 0.07365 | -0.91315748 |
| Ap5s1 | 0.59316 | -0.3036771 |
| Lifr | 0.02459 | 0.54397153 |
| Lifr | 0.01254 | 1.17815332 |
| Gm5771 | 0.00144 | -1.43031347 |
| Tatdn2 | 0.0187 | -0.79554201 |
| Decr1 | 0.00144 | -1.04314648 |
| Nsg2 | 0.03649 | -1.26682285 |
| Olfr984 | 0.00144 | -1.20555712 |
| Olfr684 | 0.00776 | -0.95716175 |
| Olfr284 | 0.67816 | -0.3570462 |
| P3h1 | 0.01519 | -0.54855572 |
| Taar8a | 0.00838 | -1.57523359 |
| Rpl22l1 | 0.87426 | 0.02922442 |
| Jun | 0.00144 | -1.42557806 |
| Dixdc1 | 0.00647 | -1.79102518 |
| Lama2 | 0.34228 | 0.16565397 |
| Ppid | 0.00144 | 1.66465435 |
| Nme6 | 0.01229 | -0.87290337 |
| Prob1 | 0.14374 | -0.36331839 |
| Nos2 | 0.10207 | -0.4111768 |
| Hist1h4d | 0.00208 | -1.24969096 |
| Hist1h4i | 0.01064 | -0.75668229 |
| Tmem211 | 0.00144 | -1.27165604 |
| Cldn7 | 0.0027 | -1.32982311 |
| Cldn7 | 0.01253 | -0.6048987 |
| Sirpb1b | 0.38174 | -0.42685995 |
| Nphp4 | 0.0037 | -1.11214255 |
| Gm31907 | 0.07105 | -0.43701326 |
| Pbx1 | 0.00144 | -1.40876853 |
| Btk | 0.18285 | 0.28503651 |
| Nkiras2 | 0.37472 | -0.32828081 |
| Nkiras2 | 0.01484 | -0.80061096 |
| 1700028P14Rik | 0.0017 | -1.24950944 |
| Iqcc | 0.79099 | -0.04691767 |
| Gdf3 | 0.09527 | -0.42763535 |
| Odf3 | 0.42279 | -0.21132622 |
| Aanat | 0.0336 | -0.42674345 |
| Rpl18a | 0.00144 | -1.09595005 |
| 7330403C04Rik | 0.12263 | -1.89991532 |
| Olfr39 | 0.33993 | -0.53307049 |
| Clca3b | 0.00144 | -1.31495282 |
| Ddx31 | 0.39902 | -0.15052499 |
| Krtap4-16 | 0.01665 | -0.56649984 |
| Glrx3 | 0.36424 | 0.21143733 |
| Rnf39 | 0.88678 | -0.03756604 |
| Knstrn | 0.94175 | 0.02823239 |
| Gm14243 | 0.22684 | -0.64979131 |
| Cntn4 | 0.09597 | -0.93195309 |
| Gm10755 | 0.18567 | -0.91465454 |
| Tmem241 | 0.03844 | -0.41448063 |
| Ndufaf1 | 0.13615 | 0.26719925 |
| Igsf10 | 0.4663 | -0.18376483 |
| Srsf5 | 0.00144 | 1.29066699 |
| Nlrp4d | 0.07123 | -0.85982117 |
| Gm10087 | 0.10085 | 0.60845381 |
| Ccl1 | 0.06556 | -0.38629649 |
| Gmds | 0.53451 | 0.10726447 |
| Myo7b | 0.01262 | -1.06655745 |
| Nyap2 | 0.00365 | -1.12123021 |
| Neu2 | 0.00442 | -0.99891824 |
| Gm7534 | 0.04131 | -2.07810448 |
| Stk35 | 0.01996 | -0.73845802 |
| Gpr158 | 0.00145 | -1.25646236 |
| Tsc1 | 0.29602 | 0.19816372 |
| Wdr34 | 0.44003 | 0.17448076 |
| Gm17783 | 0.05419 | -1.54287027 |
| Gm10734 | 0.00239 | -0.92545401 |
| Rpusd3 | 0.04171 | -0.68940425 |
| Gm5460 | 0.07009 | -1.77294443 |
| Gm14410 | 0.00144 | 2.71283364 |
| Gm10866 | 0.09843 | -0.44250136 |
| Fam20c | 0.86706 | -0.05875493 |
| Gm10864 | 0.37256 | -0.41020431 |
| Abr | 0.01228 | -0.67461675 |
| Gm40835 | 0.03147 | -0.44575133 |
| Skint9 | 0.02395 | -0.49692056 |
| Xlr5b | 0.00144 | -1.28116048 |
| Xlr5a | 0.21886 | -0.97242965 |
| Gm5741 | 0.21645 | 0.59746866 |
| Tagap1 | 0.18182 | 0.26115689 |
| Gfap | 0.12171 | -1.16018154 |
| 9330160F10Rik | 0.02611 | -0.94534324 |
| LOC102638475 | 0.07236 | -0.53683253 |
| Cdx1 | 0.03786 | -0.77101772 |
| Gm34006 | 0.0079 | -0.90427852 |
| Prss38 | 0.00144 | -1.29051721 |
| Bcl2l14 | 0.01165 | -1.53220847 |
| Nalcn | 0.00144 | -1.20555712 |
| Cdhr2 | 0.005 | -0.85769924 |
| Mllt3 | 0.72 | -0.25432027 |
| Bace2 | 0.00145 | -1.00012508 |
| Ear10 | 0.00314 | -1.34828127 |
| Cyp11b1 | 0.00145 | -1.26219879 |
| 9430007A20Rik | 0.17606 | -0.32841412 |
| Sytl3 | 0.96579 | 0.05912947 |
| Sox5 | 0.34982 | -0.30347596 |
| Gm10459 | 0.00144 | -1.32599123 |
| Tmem39b | 0.01505 | -0.52235861 |
| Aox2 | 0.00144 | -1.26911831 |
| Car13 | 0.30162 | 0.44708441 |
| Pms2 | 0.05464 | 1.18883375 |
| Pom121l2 | 0.01095 | -1.36097654 |
| Pom121l2 | 0.00144 | -1.30017714 |
| Ptx4 | 0.30724 | -0.324306 |
| Stx4a | 0.78541 | 0.04725962 |
| H2-Eb1 | 0.00407 | -1.14116401 |
| Gpr1 | 0.19216 | -0.49516436 |
| Il17a | 0.3268 | -1.77203196 |
| Cdh9 | 0.34561 | -0.49305595 |
| Adgrg5 | 0.07519 | -0.52617874 |
| Gm9869 | 0.15272 | -1.48462948 |
| Ifnk | 0.00144 | -1.34324911 |
| Cdk5r1 | 0.00514 | -0.69897676 |
| Gm3146 | 0.14277 | 0.53966535 |
| Nt5c3b | 0.86124 | 0.08128621 |
| Nt5c3b | 0.2224 | -0.28249915 |
| Upk1a | 0.92595 | 0.02699865 |
| Rpl26 | 0.06183 | -0.44268087 |
| Ccdc136 | 0.00144 | -1.28116048 |
| Btbd16 | 0.36646 | 0.1563569 |
| BB014433 | 0.31803 | 0.34483273 |
| Gm4129 | 0.25464 | -0.47893997 |
| Defb44-ps | 0.03366 | -1.89185502 |
| Kcnj16 | 0.00144 | -1.27992547 |
| Pcyox1 | 0.38506 | 0.1686036 |
| Gm42151 | 0.41443 | -0.15191476 |
| Plekha2 | 0.22077 | -0.20201642 |
| Bpifb3 | 0.75417 | -0.10281311 |
| Olfr509 | 0.01229 | -1.09550759 |
| Cd3e | 0.00144 | -1.54655203 |
| Gm10549 | 0.00208 | -1.15498993 |
| BC028528 | 0.67235 | 0.0917895 |
| LOC102634852 | 0.00144 | -1.30017714 |
| Gadl1 | 0.00144 | -1.30713006 |
| Tcerg1l | 0.0017 | -1.24950944 |
| Sema6c | 0.00144 | -1.49161358 |
| Skp1a | 0.68962 | -0.07317211 |
| Fam132b | 0.01815 | -1.87318776 |
| Recql4 | 0.04289 | -0.52999394 |
| Kcnk15 | 0.01098 | -0.82857281 |
| Sumo2 | 0.55547 | -0.10288805 |
| Hcrtr1 | 0.0148 | -1.36796504 |
| Hace1 | 0.4739 | 0.16987862 |
| Chmp3 | 0.49308 | 0.1147272 |
| Snhg10 | 0.02265 | -0.47090747 |
| Gm16501 | 0.40219 | -0.294899 |
| Klc1 | 0.91978 | 0.06095314 |
| Olfr243 | 0.04597 | -2.13983693 |
| Gm12253 | 0.00144 | -1.29049509 |
| Rplp1 | 0.00144 | -1.39037243 |
| Rbbp8 | 0.00741 | -1.10980439 |
| Aknad1 | 0.02193 | -1.00899623 |
| Spink13 | 0.00325 | -1.27185848 |
| Clec3a | 0.00145 | -1.22376446 |
| Mafa | 0.80794 | -0.04168463 |
| Mef2c | 0.01107 | 0.79018959 |
| Nlrc3 | 0.78072 | -0.05262373 |
| Lck | 0.003 | -1.18341381 |
| Rps27 | 0.00144 | -1.26850792 |
| Sec61g | 0.00469 | -0.6876651 |
| Bsph2 | 0.0699 | -1.02742313 |
| Gm12695 | 0.82524 | 0.34876455 |
| Dytn | 0.00144 | -1.30017714 |
| Grk4 | 0.00144 | 2.38139647 |
| Dock3 | 0.07476 | -0.34251069 |
| Ksr2 | 0.01751 | -1.05175941 |
| Ankrd34a | 0.02712 | -0.93852144 |
| 1700080O16Rik | 0.00144 | -1.3421574 |
| Tnfrsf22 | 0.1009 | -2.12400143 |
| Klrc3 | 0.00144 | -1.30713006 |
| Rtp2 | 0.13921 | -0.38585933 |
| Gm5086 | 0.78561 | -0.25269748 |
| Naa40 | 0.00724 | -0.82420518 |
| Ssbp2 | 0.18049 | -0.24496105 |
| Krtap28-13 | 0.03956 | -0.79997687 |
| Rpap3 | 0.01093 | 0.57027432 |
| Cd200r4 | 0.00736 | -1.57316072 |
| Acap1 | 0.07814 | -0.67303048 |
| Ccdc28a | 0.70075 | 0.0839021 |
| Morn3 | 0.8285 | 0.09857952 |
| 2810006K23Rik | 0.57737 | -0.18478109 |
| 2810006K23Rik | 0.32909 | -0.16275824 |
| Gm41795 | 0.01033 | -0.79701792 |
| Shisa6 | 0.00144 | 1.20018751 |
| Mettl4 | 0.82735 | 0.03976959 |
| Klhl9 | 0.78227 | 0.0600229 |
| Smarca4 | 0.42306 | -0.21472882 |
| Smarca4 | 0.22973 | -0.20942585 |
| Gm11159 | 0.00144 | -1.32766452 |
| Slco1a5 | 0.00145 | -1.22518835 |
| Mrpl39 | 0.3212 | 0.2621335 |
| Pofut1 | 0.13921 | -0.47831807 |
| Cdhr4 | 0.41535 | -0.18729296 |
| Ctxn2 | 0.00144 | -1.36353418 |
| Xkr6 | 0.03322 | 0.94980633 |
| Cd320 | 0.01646 | -0.78010554 |
| Car15 | 0.35204 | 0.14668218 |
| Bmp4 | 0.06334 | 0.64893245 |
| Gm32204 | 0.00928 | -1.08541862 |
| Peak1 | 0.96381 | 0.00785473 |
| Cbx1 | 0.71755 | 0.13237261 |
| Gfpt2 | 0.63246 | 0.20916246 |
| Gfpt1 | 0.0047 | 0.88135174 |
| Rps29 | 0.00144 | -1.13871054 |
| 1700008J07Rik | 0.05612 | -1.56986869 |
| Ncapd3 | 0.00227 | 1.14454524 |
| Zc3hav1 | 0.01332 | 0.96714271 |
| Olfr612 | 0.90753 | -0.08209221 |
| Gm5526 | 0.00358 | -0.80520119 |
| Gm3230 | 0.11396 | -1.65063175 |
| 6030498E09Rik | 0.13496 | -0.6570291 |
| Tuba1b | 0.00144 | -1.12676312 |
| Gcm1 | 0.00144 | 4.00694246 |
| Myo18a | 0.24263 | -0.33045817 |
| Myo18a | 0.16625 | 0.52972453 |
| Ptgis | 0.00144 | -1.44264154 |
| Tldc2 | 0.24 | -0.20416695 |
| Utrn | 0.00236 | -1.13100373 |
| Slc17a5 | 0.92077 | -0.01938062 |
| LOC102642045 | 0.36517 | 0.13709039 |
| Irak1 | 0.54172 | 0.17280274 |
| Fgfr1op | 0.09762 | 0.37687378 |
| Fmo1 | 0.19332 | 0.34237464 |
| Muc3a | 0.05234 | 1.34685467 |
| 5830411N06Rik | 0.00706 | -1.68778525 |
| LOC102631757 | 0.00144 | -1.28898079 |
| Ccdc127 | 0.11427 | -0.30183924 |
| Ahnak | 0.02683 | -0.58717394 |
| Ahnak | 0.00499 | -1.2761659 |
| Zfp655 | 0.25251 | 0.67802068 |
| Zkscan3 | 0.15652 | 0.53535402 |
| Zkscan3 | 0.60674 | 0.08935142 |
| Enho | 0.0018 | -1.19263154 |
| Cecr2 | 0.00793 | -0.6138103 |
| Tmem217 | 0.00985 | -1.59449576 |
| Palm2 | 0.32311 | 0.53610568 |
| Ermap | 0.01229 | -1.37627344 |
| Ermap | 0.28424 | 0.25088293 |
| C530008M17Rik | 0.92498 | 0.09780027 |
| Cngb3 | 0.37107 | 0.30763061 |
| Tubb4b | 0.01835 | -0.58058998 |
| Ldhc | 0.00144 | -1.7470027 |
| Zap70 | 0.02922 | -0.99865602 |
| Tsix | 0.00158 | -1.2446827 |
| Dtnb | 0.00144 | -1.28116048 |
| 2610028H24Rik | 0.06787 | -0.41436266 |
| Ttn | 0.1639 | 0.2784392 |
| Ccdc64 | 0.00144 | -1.33059316 |
| Ccdc54 | 0.00144 | -1.26560808 |
| Mtr | 0.75258 | -0.10337524 |
| Plekhm3 | 0.98682 | 0.00277027 |
| Scaf8 | 0.00177 | -1.10721314 |
| Nol4 | 0.00144 | -1.27699683 |
| Mybbp1a | 0.0155 | -0.69007161 |
| 2900040C04Rik | 0.15057 | -0.38271592 |
| Gm1141 | 0.04616 | -0.88410199 |
| Lyrm1 | 0.06663 | 0.78519829 |
| Fgfrl1 | 0.05145 | -0.50363758 |
| Ints10 | 0.15638 | 0.7416187 |
| Tom1l2 | 0.01046 | -0.74000069 |
| Lhfpl2 | 0.51165 | -0.1844239 |
| Lhfpl2 | 0.06085 | -0.84459662 |
| Adam20 | 0.00145 | -1.25646236 |
| Gm34066 | 0.077 | -0.75587467 |
| Zic4 | 0.00286 | -1.42054161 |
| Cyp4a12b | 0.00541 | -0.99902553 |
| Agpat3 | 0.00952 | -1.02429982 |
| Plekha5 | 0.00251 | 0.82136025 |
| Gapdhs | 0.00144 | -1.4750072 |
| Lce3a | 0.00144 | -1.27992547 |
| Lce3f | 0.01704 | -1.06449324 |
| Mitf | 0.01206 | -0.68389017 |
| Zwilch | 0.00571 | 0.72072808 |
| Runx2os1 | 0.70755 | -0.26314434 |
| Atp5h | 0.00654 | -0.75423921 |
| Atp5h | 0.0016 | -0.99471723 |
| Rpl19 | 0.00297 | -0.93817092 |
| Ihh | 0.03364 | -0.67601151 |
| Scgb1b2 | 0.00144 | -1.66099786 |
| LOC102640292 | 0.01117 | -1.03256097 |
| Ctps2 | 0.00176 | -1.24266807 |
| Xrcc3 | 0.21308 | -1.25836147 |
| Tesc | 0.00144 | -1.58389376 |
| Gngt2 | 0.00145 | -0.97793577 |
| Gm6570 | 0.01332 | -0.511588 |
| Olfr1406 | 0.00316 | -1.34462115 |
| Sval3 | 0.00144 | -1.28898079 |
| Mest | 0.13101 | 0.76934752 |
| Plxnd1 | 0.00144 | -1.09020253 |
| Chdh | 0.00327 | -1.13251802 |
| Dis3l2 | 0.00257 | -0.88838577 |
| Matr3 | 0.00144 | 2.63816485 |
| Olfr11 | 0.01036 | -1.91313078 |
| Traf6 | 0.86704 | -0.15312583 |
| Pira11 | 0.05851 | -0.48746977 |
| Gm5294 | 0.00554 | -1.04681273 |
| Klra15 | 0.00144 | -1.33632959 |
| Sorl1 | 0.01314 | -0.58724696 |
| Samd9l | 0.00144 | 1.78978709 |
| Pde3b | 0.03336 | -1.11245883 |
| C8g | 0.05125 | -0.57851454 |
| Adarb1 | 0.14428 | 0.29773017 |
| Mfap3 | 0.53351 | -0.3381028 |
| Casc3 | 0.05637 | -0.36453326 |
| Fut9 | 0.00248 | -2.00060681 |
| Hemt1 | 0.75129 | -0.11604599 |
| Gml | 0.00157 | -1.19247859 |
| Tmem116 | 0.75785 | -0.15195576 |
| Tmem116 | 0.28457 | 0.67634418 |
| Pwwp2a | 0.90149 | 0.13529775 |
| Olfr772 | 0.00147 | -1.15996015 |
| Olfr372 | 0.00144 | -2.10367506 |
| Olfr172 | 0.00145 | -1.2388121 |
| Elovl1 | 0.00144 | -1.01164718 |
| Krtap19-2 | 0.73723 | -0.2812936 |
| Krtap19-4 | 0.00144 | -1.76241291 |
| Dynlt1c | 0.10447 | -0.44051937 |
| Dynlt1f | 0.57975 | -0.17097455 |
| Micu1 | 0.00688 | -0.65970952 |
| Jakmip2 | 0.09524 | -2.09899833 |
| Phactr4 | 0.37865 | 0.17585502 |
| Cox7a2l | 0.00159 | -0.89841656 |
| 2210411A11Rik | 0.26716 | -0.41889174 |
| Rarg | 0.00394 | -0.95283437 |
| Ccdc50 | 0.00747 | 2.59321939 |
| C1qtnf7 | 0.47683 | -0.25997354 |
| C1qtnf7 | 0.11041 | -0.27267534 |
| Npas1 | 0.00272 | -1.15217138 |
| Cpne7 | 0.01669 | -0.72633249 |
| Als2cr12 | 0.00144 | -1.30017714 |
| Gm7073 | 0.00144 | -1.27165604 |
| Pla2g3 | 0.00144 | -1.20555712 |
| Cdh4 | 0.31889 | -0.28162628 |
| 6330405D24Rik | 0.45134 | 0.16691329 |
| Ccdc85c | 0.04502 | -1.21535801 |
| Mul1 | 0.00511 | -0.98572573 |
| Mul1 | 0.18791 | -0.72298439 |
| Gzmm | 0.01408 | -0.57439046 |
| Exoc3l | 0.01462 | -0.95056639 |
| Eomes | 0.03346 | -0.90748867 |
| Cpne4 | 0.08985 | -0.88732951 |
| Socs6 | 0.00144 | -1.27165604 |
| Gadd45gip1 | 0.00144 | -1.05759666 |
| Mmp28 | 0.00144 | -1.27165604 |
| Gm10831 | 0.0475 | -1.02554043 |
| Camk2b | 0.02769 | -0.85411887 |
| Lalba | 0.00346 | -0.97740423 |
| Cstl1 | 0.00346 | -1.22679402 |
| Ptprd | 0.00144 | -1.28116048 |
| Fgfr3 | 0.00257 | -1.09100616 |
| Piezo1 | 0.00226 | -0.89876743 |
| E030030I06Rik | 0.00177 | -0.87496204 |
| E030030I06Rik | 0.20095 | 0.36314609 |
| E030030I06Rik | 0.02682 | -0.43070176 |
| Gm4787 | 0.2624 | -0.61448468 |
| Olfr126 | 0.00158 | -1.22853957 |
| Ucn | 0.02133 | -3.23483251 |
| C1s2 | 0.13104 | 0.42144314 |
| 5730522E02Rik | 0.00144 | -1.30626366 |
| Gm10641 | 0.00216 | -1.10905491 |
| Pgm2l1 | 0.00302 | 1.20501316 |
| Zfp593 | 0.02275 | -0.54532261 |
| Cdkl2 | 0.25208 | -0.24647694 |
| Otub1 | 0.00289 | -0.95200809 |
| Klra12 | 0.00144 | -1.29864962 |
| Gnat3 | 0.02979 | -1.09398387 |
| Ssty1 | 0.00145 | -1.22376446 |
| Prx | 0.0034 | -0.79907959 |
| Ninj2 | 0.41431 | -0.31584419 |
| Ninj2 | 0.01013 | 1.34933944 |
| Lrrc75a | 0.00837 | -0.88646673 |
| Poln | 0.00144 | -1.28898079 |
| 1700047A11Rik | 0.00229 | -0.93730352 |
| Srsf11 | 0.18855 | -0.44214894 |
| Srsf11 | 0.00292 | 1.75117812 |
| Abhd15 | 0.72345 | 0.13022256 |
| Fcnb | 0.03684 | -1.32746027 |
| Vmn1r19 | 0.03118 | -1.50960097 |
| Vmn1r26 | 0.00144 | -1.27165604 |
| Atp5k | 0.00144 | -0.91025407 |
| Ptchd4 | 0.03065 | -0.76293762 |
| Susd1 | 0.00144 | -1.28116048 |
| Ngfrap1 | 0.45275 | -0.165984 |
| Rsrc2 | 0.88077 | 0.05364541 |
| Oas1h | 0.00144 | -1.30017714 |
| Ehmt1 | 0.81328 | 0.03977502 |
| Wasf3 | 0.19581 | -0.74946254 |
| Agfg2 | 0.07793 | -0.50286598 |
| Sh3bp5l | 0.00145 | -0.98039404 |
| Aar2 | 0.00197 | -0.95703409 |
| Camk2a | 0.00144 | -1.64266456 |
| Farp2 | 0.94611 | -0.01420753 |
| BC055324 | 0.90084 | 0.07159308 |
| Fpr-rs6 | 0.0256 | -0.93578497 |
| Tubgcp5 | 0.06706 | -1.36126408 |
| Hif1a | 0.00316 | 1.34285638 |
| F830208F22Rik | 0.34747 | -0.61347435 |
| Gm13124 | 0.00144 | -1.26560808 |
| A730017C20Rik | 0.00144 | -1.28116048 |
| S100a16 | 0.00441 | -0.81736787 |
| Gm4489 | 0.02273 | -0.90257945 |
| Tsr3 | 0.63657 | 0.10456848 |
| Zan | 0.25305 | -0.58003328 |
| Dclk1 | 0.80285 | 0.04311374 |
| Gm32916 | 0.00144 | -1.28898079 |
| Nipal3 | 0.00819 | -0.69806805 |
| Gm14762 | 0.06834 | 0.87577813 |
| Gm14762 | 0.00405 | 1.4949864 |
| Paqr6 | 0.01127 | -1.09930078 |
| Rab4a | 0.00157 | -0.94276714 |
| Gpr61 | 0.09682 | -2.53171 |
| LOC102633497 | 0.00144 | -1.28898079 |
| A430060F13Rik | 0.00144 | -1.31495282 |
| Nr1d2 | 0.00915 | 1.32735542 |
| Pithd1 | 0.15155 | -0.41846954 |
| Teddm2 | 0.00332 | -0.87772564 |
| Pax2 | 0.00244 | -1.16878422 |
| Krt2 | 0.00397 | -1.26895874 |
| Klhl25 | 0.00144 | -1.34458633 |
| Foxred2 | 0.05861 | 0.57726796 |
| Vmn2r82 | 0.06425 | -1.56946548 |
| Gm14347 | 0.00283 | -1.6312286 |
| Hand1 | 0.36377 | -0.46485738 |
| 2810021J22Rik | 0.05898 | 0.52499539 |
| Gm15401 | 0.07122 | -0.87939581 |
| Tmem79 | 0.43839 | -0.13229769 |
| Gm20822 | 0.00144 | -1.25118919 |
| Xxylt1 | 0.91509 | 0.0231268 |
| G530011O06Rik | 0.00215 | -1.40039174 |
| Wdr12 | 0.01789 | -0.499594 |
| Gm10572 | 0.08414 | -0.49904778 |
| Ptgdr | 0.00144 | -1.55701652 |
| Lzts1 | 0.95265 | 0.02530004 |
| Gm7133 | 0.00144 | -1.28116048 |
| Ncmap | 0.90327 | 0.03497828 |
| Tsr2 | 0.17504 | 0.26974785 |
| BC039966 | 0.00144 | -1.24811894 |
| 2610005L07Rik | 0.01982 | 1.25429691 |
| Fam71a | 0.00144 | -1.51912556 |
| Fam196b | 0.00145 | -1.26219879 |
| Fam118a | 0.82068 | -0.06806127 |
| Abhd17a | 0.00709 | -1.04815111 |
| Npcd | 0.00282 | -0.88232688 |
| Gimap4 | 0.03074 | 0.73020641 |
| Fam129c | 0.18994 | 0.71543685 |
| Gm13103 | 0.00144 | -1.28375735 |
| Dpysl4 | 0.05113 | -0.50158608 |
| Ctla4 | 0.40868 | -1.50359728 |
| Vmn2r56 | 0.00147 | -1.15512485 |
| Prickle2 | 0.23698 | -0.51470776 |
| Gm29692 | 0.00144 | -1.28116048 |
| Cstf1 | 0.0447 | -0.49284125 |
| Gm36696 | 0.00144 | -1.591907 |
| Med27 | 0.25278 | -0.42381449 |
| Dnajc11 | 0.75084 | 0.09073114 |
| Cyth3 | 0.00158 | -1.12355313 |
| Gm6410 | 0.00144 | -1.30017714 |
| Gm6116 | 0.00666 | -1.00718272 |
| Nt5dc1 | 0.00156 | -1.78390286 |
| Robo3 | 0.00852 | -1.47397797 |
| Sh2d4b | 0.02375 | -1.60415193 |
| Caprin2 | 0.00492 | 0.79200008 |
| Kcnh8 | 0.05566 | -0.87890304 |
| Ttc37 | 0.50218 | 0.32259194 |
| A430005L14Rik | 0.02046 | -0.65335175 |
| Ctdspl2 | 0.02497 | 0.58705645 |
| Spata2 | 0.0039 | -0.69164681 |
| Zfp712 | 0.00388 | 0.86939851 |
| Ercc5 | 0.08628 | 0.94280168 |
| Sytl4 | 0.40569 | -0.84456955 |
| Sytl4 | 0.17903 | 0.52150143 |
| Tnfaip6 | 0.4111 | -0.30267055 |
| Dmc1 | 0.00752 | -1.52429656 |
| Tmc1 | 0.00145 | -1.22376446 |
| Wdr55 | 0.19675 | 0.3212864 |
| Olfr1389 | 0.04978 | -2.11683242 |
| Ggt5 | 0.61958 | -0.19038557 |
| Ggt5 | 0.34845 | -0.4535674 |
| Map2k5 | 0.4886 | -0.13764303 |
| Olfr681 | 0.01436 | -0.59433199 |
| Olfr681 | 0.21425 | -0.7987559 |
| Olfr481 | 0.00144 | -2.03430875 |
| Gm4466 | 0.00144 | -1.28116048 |
| Wfdc8 | 0.10258 | -0.73571488 |
| Rps13 | 0.01453 | -0.65856424 |
| Ppp1r1c | 0.0073 | -1.88199731 |
| Zfp354a | 0.0076 | 1.37334003 |
| Zcchc9 | 0.02069 | -0.55067528 |
| 4731417B20Rik | 0.02771 | -1.81749295 |
| Gm36107 | 0.00144 | -1.26428512 |
| LOC105243544 | 0.00144 | -1.30713006 |
| Trim7 | 0.40892 | -0.30515821 |
| Gm4663 | 0.41483 | -0.30878278 |
| Gm13570 | 0.31555 | 0.51081021 |
| Timm22 | 0.00144 | -1.40550151 |
| H2-Ab1 | 0.00501 | -1.09533712 |
| Slc2a12 | 0.24399 | 0.19665346 |
| Cnr2 | 0.78543 | 0.09368062 |
| Camta2 | 0.0081 | -0.64095101 |
| Carns1 | 0.31214 | -0.19335059 |
| Tmem102 | 0.96658 | -0.02568922 |
| Ccdc107 | 0.42104 | 0.34067999 |
| Gm10554 | 0.12616 | 0.49591729 |
| Zmynd8 | 0.00214 | -1.14723775 |
| Gm13178 | 0.36236 | -0.37412093 |
| Syngap1 | 0.34537 | -0.44404088 |
| Ginm1 | 0.48726 | 0.25473568 |
| Filip1 | 0.00814 | 0.79898675 |
| Nmnat1 | 0.01145 | -0.57861263 |
| Lmx1a | 0.76964 | -0.31942667 |
| Dnajc6 | 0.00336 | -1.20864476 |
| Lmx1b | 0.04054 | -0.40954182 |
| Nop9 | 0.05831 | -0.88481247 |
| Ifitm1 | 0.11405 | -0.52355182 |
| Gm11691 | 0.00144 | -1.56157582 |
| Rs1 | 0.93888 | -0.04499007 |
| Cerk | 0.01461 | -0.57891416 |
| Cox20 | 0.12391 | 0.53763908 |
| Gla | 0.60164 | -0.12231955 |
| Zfp763 | 0.30906 | -1.09976397 |
| Olfr247 | 0.00759 | -1.38098265 |
| Pank4 | 0.03198 | -0.51443088 |
| Fbxw15 | 0.00144 | -1.29085005 |
| Myh9 | 0.0789 | -0.48952838 |
| Phb | 0.00849 | -0.80118375 |
| Tpt1 | 0.01227 | -0.52466113 |
| Srsf1 | 0.00492 | -1.10113754 |
| Maged2 | 0.40469 | -1.62562601 |
| Dmpk | 0.31409 | -0.20922284 |
| Dmpk | 0.31171 | -0.1746644 |
| Aen | 0.02596 | -0.46210057 |
| Pcf11 | 0.03036 | -0.5062594 |
| Atp6v1g3 | 0.00144 | -1.27165604 |
| Nlrp9a | 0.00144 | -1.26560808 |
| Tox3 | 0.19213 | 0.23440752 |
| Tox3 | 0.52284 | 0.33571826 |
| P3h4 | 0.07111 | -0.48828362 |
| Hlcs | 0.69996 | 0.06797814 |
| A930023M06Rik | 0.51711 | -0.38328235 |
| Krt82 | 0.00592 | -1.86480943 |
| Krt80 | 0.20271 | -0.68090237 |
| Mpst | 0.02302 | -0.79891318 |
| Stk38l | 0.02136 | -0.67473538 |
| Gm36012 | 0.78225 | -0.27778338 |
| Flt3l | 0.71997 | -0.12574103 |
| Wdr45 | 0.89786 | -0.03249856 |
| Gm10415 | 0.00144 | -1.31495282 |
| LOC102640328 | 0.02137 | -1.6044105 |
| D5Ertd577e | 0.00144 | -1.30713006 |
| 4930478M13Rik | 0.00144 | -1.46134994 |
| Pde1a | 0.00325 | -0.77037143 |
| Fastk | 0.21087 | -0.2474729 |
| 4933406F09Rik | 0.56185 | -0.16797531 |
| Srd5a2 | 0.04597 | -0.88511728 |
| Pex5l | 0.39652 | -0.20836654 |
| Six3os1 | 0.00144 | -1.28898079 |
| Cpn2 | 0.50196 | -0.52211561 |
| Btnl9 | 0.90748 | -0.04575832 |
| Gbp5 | 0.53669 | -0.14365325 |
| Swt1 | 0.00144 | 1.09229941 |
| Gm826 | 0.35675 | 0.56508927 |
| Lrrc74a | 0.00749 | -1.05513651 |
| Recql5 | 0.00144 | -1.15919597 |
| Ccdc90b | 0.00488 | -0.70052332 |
| Gfer | 0.3203 | -0.25942597 |
| Erich5 | 0.14798 | -0.3879446 |
| Pcdh11x | 0.02156 | -1.20181442 |
| Ptges3l | 0.00145 | -1.2388121 |
| Prelp | 0.09001 | -2.08460051 |
| Hecw2 | 0.01576 | 1.09784544 |
| 4930579K19Rik | 0.00694 | -1.13077247 |
| Dmbt1 | 0.00145 | -1.22376446 |
| Prpsap1 | 0.02251 | -0.47989531 |
| Sdk1 | 0.00345 | -1.12155526 |
| Ccdc163 | 0.40313 | 0.14274592 |
| 9030025P20Rik | 0.00974 | 0.59841445 |
| Gm10869 | 0.00941 | -0.97527059 |
| Stard7 | 0.00728 | 0.73420963 |
| Flrt3 | 0.30701 | 0.60426385 |
| Flrt3 | 0.00144 | -1.34749171 |
| Fam65b | 0.38685 | 0.35057459 |
| Suv420h1 | 0.05389 | 0.41643055 |
| Suv420h1 | 0.36009 | 0.15292732 |
| Suv420h1 | 0.02891 | 1.27667535 |
| Gm4319 | 0.00145 | -1.16044081 |
| Gm11123 | 0.77042 | 0.07872054 |
| Gm4354 | 0.7239 | 0.16657687 |
| Platr17 | 0.8571 | 0.10101156 |
| Gm26919 | 0.00317 | -1.11935153 |
| Nnt | 0.25578 | 0.25940671 |
| F830045P16Rik | 0.00144 | -1.27165604 |
| Usp17lc | 0.02743 | -0.97379672 |
| Olfr518 | 0.00144 | -1.38400103 |
| Gm10386 | 0.00144 | -1.25118919 |
| Mink1 | 0.90034 | -0.02463727 |
| Gm2800 | 0.11698 | -0.78800402 |
| Efcab1 | 0.00145 | -1.22376446 |
| Gtdc1 | 0.00144 | -1.29049509 |
| Gps1 | 0.01338 | -0.81798886 |
| Pank2 | 0.16105 | -0.34164178 |
| Slc13a2 | 0.12596 | -0.65442886 |
| Slc17a2 | 0.00144 | -1.33632959 |
| Defa-ps13 | 0.00144 | -1.27558215 |
| Etl4 | 0.7988 | 0.11267752 |
| Etl4 | 0.01447 | 0.63015322 |
| Skint5 | 0.25989 | 0.37345645 |
| Gm996 | 0.80348 | 0.0745296 |
| Ugt1a6b | 0.00144 | -1.27165604 |
| Tcf7l2 | 0.00243 | -1.13269597 |
| Sytl1 | 0.5186 | -0.18197082 |
| Ntm | 0.00271 | 0.98775769 |
| Hdx | 0.01423 | 0.74417082 |
| Gm5784 | 0.44881 | -0.3012575 |
| Vmn2r48 | 0.00144 | -1.26560808 |
| 5830453K13Rik | 0.00144 | -1.28898079 |
| 2700046A07Rik | 0.07841 | -0.82833624 |
| Plscr5 | 0.00144 | 1.63964972 |
| Fstl3 | 0.01392 | -0.67780543 |
| Fstl1 | 0.895 | -0.03039335 |
| Pgbd5 | 0.55424 | -0.2959268 |
| Doc2b | 0.347 | 0.40190041 |
| Cyp4f37 | 0.04258 | -0.92360968 |
| Gja10 | 0.88042 | 0.08260456 |
| Rpp21 | 0.0035 | -1.10667575 |
| Rpp21 | 0.12568 | -0.58365956 |
| Dyrk1b | 0.082 | -0.57725026 |
| Rest | 0.25974 | 0.33710461 |
| Nkd2 | 0.01104 | -0.78168043 |
| Nkd2 | 0.5258 | -0.19716892 |
| Rims3 | 0.38769 | 0.3533437 |
| Rasgrp2 | 0.01164 | -0.60126902 |
| Rasgrp2 | 0.00951 | -0.58938241 |
| Axin1 | 0.4265 | -0.29636861 |
| Dyrk1a | 0.93543 | -0.02496827 |
| Hrh4 | 0.42543 | -0.36783888 |
| 1700008F21Rik | 0.00144 | -1.24811894 |
| Qk | 0.30051 | 0.16385315 |
| Myadml2 | 0.0121 | -0.96958931 |
| Rwdd3 | 0.00144 | -1.28898079 |
| Ddx39b | 0.0049 | -0.82526537 |
| Gm10280 | 0.00144 | -1.27165604 |
| LOC102634533 | 0.02775 | 0.96337319 |
| Ear7 | 0.00144 | -1.29878664 |
| Sema3c | 0.00254 | 1.1712325 |
| Sema6c | 0.00144 | -1.12257093 |
| Gm9465 | 0.19471 | -0.33928044 |
| Pifo | 0.00251 | -2.39458474 |
| Gm3404 | 0.0526 | -1.40800056 |
| LOC105247474 | 0.00144 | -1.23532597 |
| Cadm2 | 0.29299 | -0.60554425 |
| K230010J24Rik | 0.2092 | -0.40244632 |
| Mink1 | 0.83987 | -0.03674901 |
| Parp9 | 0.01693 | 0.74480974 |
| Parp9 | 0.00144 | -1.55661713 |
| Ninj1 | 0.015 | -1.02303984 |
| AI463229 | 0.00144 | -1.29085005 |
| Slc9a7 | 0.10491 | -0.30563778 |
| Lrrc48 | 0.40156 | 0.4406427 |
| Lrrc48 | 0.00696 | -1.52481061 |
| Lrrc48 | 0.00288 | -0.79277495 |
| Gm4651 | 0.00457 | -1.22536112 |
| Mark3 | 0.52689 | -0.09726113 |
| Mark4 | 0.00455 | -0.88631158 |
| Mark4 | 0.01078 | -0.70159497 |
| Tdrd5 | 0.00144 | -1.26560808 |
| Gm10389 | 0.00144 | -1.29777136 |
| Gm3853 | 0.68612 | 0.09798417 |
| Echdc2 | 0.06905 | 0.62092565 |
| Cacna1a | 0.04422 | -0.40041422 |
| Hcrt | 0.02077 | -0.92383109 |
| Zkscan7 | 0.00327 | -1.42092784 |
| Zfp831 | 0.00144 | -1.29746368 |
| Hist2h3c2 | 0.04334 | -0.87801637 |
| Serpina3i | 0.77307 | -0.13299722 |
| Serpina3f | 0.15375 | -0.51976789 |
| Serpina3h | 0.97235 | -0.0155482 |
| 4732471J01Rik | 0.02009 | -1.04198494 |
| Olfr1229 | 0.00253 | -0.98664921 |
| Mapk1ip1 | 0.00503 | 0.79472618 |
| Glul | 0.02658 | -0.44936256 |
| Gm33441 | 0.37445 | 0.40703255 |
| Synj2 | 0.50029 | 0.11245068 |
| Synj2 | 0.0158 | -0.68084524 |
| Clip4 | 0.04962 | 0.42163351 |
| Gm4847 | 0.00144 | -1.28116048 |
| Ube2l6 | 0.01237 | -0.76471765 |
| Gm35953 | 0.00423 | -1.28971466 |
| Olfr884 | 0.00149 | -1.22582153 |
| Tagln2 | 0.00953 | -0.79795511 |
| Ldha | 0.01118 | -0.70653663 |
| Aen | 0.03646 | -0.51369135 |
| Gm39976 | 0.00144 | -1.27165604 |
| Pard6g | 0.03928 | -0.5998243 |
| Cdhr4 | 0.79166 | -0.21257753 |
| Snrnp27 | 0.01189 | 1.01698519 |
| Gm13275 | 0.13607 | -0.31411581 |
| Efcab9 | 0.00144 | -1.30844149 |
| Mrgpra8 | 0.00144 | -1.27165604 |
| Rnaseh2a | 0.9359 | -0.13224518 |
| Magi2 | 0.68381 | -0.53866702 |
| Cacng3 | 0.00363 | -1.23873568 |
| Park7 | 0.00825 | -0.8652812 |
| Ly6g6c | 0.59993 | -0.25112245 |
| AU022252 | 0.03663 | -0.7770206 |
| Synj2bp | 0.01015 | 1.56334529 |
| Erlin1 | 0.12018 | 0.31433924 |
| Tmem69 | 0.23923 | 0.36690837 |
| Mb21d1 | 0.67366 | 0.16532371 |
| Zbtb49 | 0.08075 | 0.49459941 |
| Zbtb49 | 0.00145 | -1.26219879 |
| 4930577H14Rik | 0.00248 | -1.09321212 |
| Eya2 | 0.01051 | -0.71147149 |
| Tenm4 | 0.06885 | -0.57749578 |
| Tenm4 | 0.03905 | -0.66072296 |
| Gm11128 | 0.00144 | -1.3230041 |
| Siglech | 0.84873 | 0.05602401 |
| Siglech | 0.08847 | -1.08036104 |
| Emx1 | 0.02213 | -1.43580951 |
| Wnt8b | 0.17061 | -1.38509274 |
| Vax2os | 0.00261 | -1.11284335 |
| Gm5941 | 0.00144 | -1.59026227 |
| Gm6034 | 0.13962 | -1.19270877 |
| Vmn1r124 | 0.26671 | -0.53363468 |
| Sprr2a1 | 0.00145 | -1.22376446 |
| Scfd2 | 0.0111 | -1.02598223 |
| Ssu72 | 0.0832 | -2.41941094 |
| Polr3gl | 0.74211 | 0.06074509 |
| Plcd4 | 0.12454 | -1.48687448 |
| Gm6658 | 0.23318 | 0.25885677 |
| Creg2 | 0.02466 | -1.22935729 |
| Creg1 | 0.00144 | -1.3622748 |
| Krtap24-1 | 0.43845 | -0.58682717 |
| Olfr1457 | 0.00144 | -1.22925777 |
| Pex13 | 0.00491 | 0.73550392 |
| Myh6 | 0.00229 | -1.44750274 |
| Defb48 | 0.00149 | -1.22582153 |
| Mis18a | 0.2024 | 0.20717328 |
| Olfr1181 | 0.00144 | -1.27165604 |
| Phka2 | 0.22566 | -0.19964202 |
| Gm6083 | 0.00272 | -1.81324387 |
| Wap | 0.00144 | -1.28116048 |
| 1700102H20Rik | 0.29791 | 0.37158232 |
| Dupd1 | 0.03594 | -0.89429631 |
| Gm15517 | 0.00814 | -0.7930931 |
| Gm14725 | 0.00145 | -1.22376446 |
| Flcn | 0.07733 | -0.5294481 |
| Gm5129 | 0.04905 | -1.30608989 |
| Qpct | 0.10833 | 0.29473209 |
| Cyp21a1 | 0.00587 | -2.10656939 |
| Olfr937 | 0.68169 | -0.37916784 |
| Krt32 | 0.02757 | -2.14240445 |
| Krt31 | 0.02277 | -0.53392426 |
| Krt36 | 0.00144 | -1.27165604 |
| Krt39 | 0.00144 | -1.30713006 |
| Tnfaip8 | 0.52821 | -0.12046168 |
| Mrps18b | 0.00872 | -0.7446306 |
| Ssb | 0.01495 | 1.05133147 |
| Tsr3 | 0.01241 | -0.56537022 |
| Sft2d1 | 0.01954 | -1.7264313 |
| Fgf13 | 0.00413 | -1.03541034 |
| Fgf13 | 0.00144 | -1.38524278 |
| Trim56 | 0.79643 | 0.0753608 |
| Jade2 | 0.05166 | -0.42747786 |
| Phf14 | 0.00169 | 2.06015777 |
| Fbxo18 | 0.78397 | 0.07520112 |
| Gtf2a2 | 0.24538 | 0.44521864 |
| Acbd4 | 0.06193 | -0.3919985 |
| Olfr596 | 0.04782 | -1.09650221 |
| B3gnt3 | 0.01809 | -0.60932809 |
| Gm7444 | 0.13932 | -0.57520191 |
| Alk | 0.00145 | -1.2388121 |
| Ilk | 0.985 | -0.00542418 |
| Odf2l | 0.13343 | 1.5061047 |
| Ces2h | 0.00144 | -1.27165604 |
| Myl12b | 0.00699 | -0.66295847 |
| Amy2a5 | 0.00688 | 2.59859629 |
| Gm12070 | 0.03287 | -0.55234226 |
| Cdhr3 | 0.00169 | 2.30201884 |
| Tbpl2 | 0.00144 | -1.59362757 |
| Olfr1156 | 0.00145 | -1.25646236 |
| Rps6ka1 | 0.00149 | -1.05954766 |
| Pramef17 | 0.08925 | -3.32203027 |
| 4930594M22Rik | 0.00144 | -1.29049509 |
| LOC102641662 | 0.00144 | -1.25118919 |
| Hmgcll1 | 0.00144 | -1.28898079 |
| Olfr1036 | 0.12433 | 0.65850185 |
| Cdc26 | 0.03894 | -0.63120229 |
| Rab6a | 0.03751 | 0.71675479 |
| Obox8 | 0.8426 | 0.22546909 |
| Slc22a28 | 0.00328 | -0.80827598 |
| Slc22a28 | 0.01606 | -1.65947063 |
| Piezo2 | 0.04662 | -1.18802243 |
| 4833439L19Rik | 0.64206 | -0.10089226 |
| Plec | 0.07836 | -0.49995336 |
| Hivep3 | 0.98978 | -0.00328548 |
| Lcn6 | 0.05243 | -1.03892787 |
| Nab2 | 0.58329 | 0.28597917 |
| Tpk1 | 0.0105 | 0.58972143 |
| Tpk1 | 0.09454 | -0.60041831 |
| Lrfn1 | 0.05527 | -0.49754692 |
| Lrfn1 | 0.00144 | -1.74727509 |
| Lrfn4 | 0.00439 | -1.02758388 |
| Gm1698 | 0.00308 | -1.36719057 |
| Fdx1 | 0.38634 | -0.27086443 |
| Scn1a | 0.04962 | -1.01908132 |
| Scn1a | 0.2854 | -0.66415775 |
| Gm14483 | 0.04736 | -1.46990239 |
| Sgcd | 0.07195 | -0.32476931 |
| Cyb5rl | 0.47328 | 0.229471 |
| Cc2d1a | 0.00846 | -0.64519631 |
| Mrpl40 | 0.19838 | -0.32046533 |
| Olfr823 | 0.02676 | -1.06093691 |
| Olfr923 | 0.00145 | -1.22376446 |
| Olfr723 | 0.06949 | -2.75437131 |
| Obox1 | 0.05383 | -2.07405566 |
| Xpo4 | 0.30812 | 0.17441783 |
| Ndufaf5 | 0.01708 | -0.47522839 |
| Cnr1 | 0.00144 | -1.35112763 |
| Arl14epl | 0.00149 | -1.22582153 |
| Ube2d2a | 0.72619 | -0.09080781 |
| Gigyf1 | 0.01595 | -0.55278869 |
| Efcab5 | 0.38237 | -0.4786997 |
| Pias2 | 0.00144 | 1.09008181 |
| Atxn7l3 | 0.1888 | -0.25547598 |
| U2af1l4 | 0.02793 | -0.76205196 |
| Ppm1m | 0.00144 | -1.27165604 |
| Stau2 | 0.43 | 0.42306345 |
| Fxyd1 | 0.00765 | -0.86393719 |
| Cd82 | 0.46228 | -0.231611 |
| Mpdz | 0.38581 | -0.14063982 |
| Mettl20 | 0.00144 | 1.35846092 |
| Gprc5b | 0.00761 | -1.06183985 |
| Rad50 | 0.01133 | 0.99353566 |
| Srgap3 | 0.02938 | -0.83024038 |
| Gm13247 | 0.65691 | -0.08737545 |
| Cga | 0.00159 | -1.26431393 |
| Nphp4 | 0.00355 | -0.99737929 |
| Nphp3 | 0.00144 | 2.08660004 |
| Gm996 | 0.99299 | 0.00165576 |
| Gm996 | 0.00158 | -1.2446827 |
| Olfr1289 | 0.0021 | -1.42358515 |
| Fam183b | 0.04592 | -0.97117444 |
| Nmral1 | 0.0216 | -0.50722359 |
| Mpv17 | 0.53245 | -0.1502646 |
| Kcnu1 | 0.21473 | -0.73387795 |
| Rpl14 | 0.00144 | -1.12708257 |
| Vmn1r207-ps | 0.05083 | -0.83269502 |
| Rtel1 | 0.86989 | 0.04006051 |
| Bax | 0.00144 | -1.32299684 |
| Dip2c | 0.26242 | 0.21431609 |
| Kctd21 | 0.0624 | -0.55481475 |
| Osbp2 | 0.6318 | 0.3887621 |
| B4galt3 | 0.17934 | -0.36654425 |
| Tas2r129 | 0.00293 | -1.19010524 |
| Sp140 | 0.19843 | -0.44936904 |
| AA467197 | 0.0614 | -0.54657919 |
| Chrna7 | 0.03105 | -1.46547394 |
| Hhipl1 | 0.00762 | 1.14484551 |
| Gm11213 | 0.07341 | -0.59419357 |
| Wbp1l | 0.06604 | -0.4793924 |
| Vpreb3 | 0.24381 | -0.35597658 |
| Cisd3 | 0.084 | -0.44975327 |
| Arhgap33os | 0.6743 | -0.77442836 |
| Ppp1r15a | 0.04886 | -0.61027516 |
| Dpy30 | 0.00884 | -0.5786164 |
| Gm6981 | 0.05228 | -0.54776969 |
| Coro2b | 0.0138 | -0.55867802 |
| Rab10os | 0.00361 | -1.22849007 |
| Zkscan1 | 0.12996 | -0.28796308 |
| Sult1b1 | 0.00144 | -1.30713006 |
| Uqcr10 | 0.08194 | -0.69456422 |
| Tomm34 | 0.00145 | -0.91182439 |
| Ermn | 0.00144 | 4.17332486 |
| D930016D06Rik | 0.08422 | 0.71919099 |
| Cd99 | 0.00144 | -1.12678075 |
| Zfp120 | 0.00144 | 1.874964 |
| 8030462N17Rik | 0.02064 | -0.54600825 |
| Crnn | 0.01281 | -2.08690059 |
| Cdc42se2 | 0.02994 | -0.41481673 |
| Gm10680 | 0.02896 | -0.82323332 |
| Pkn2 | 0.98963 | 0.00240912 |
| BC055111 | 0.00144 | -1.22925777 |
| Kmt2d | 0.0023 | -1.11792485 |
| Tmem128 | 0.36394 | -0.14367872 |
| Tsfm | 0.27888 | -0.75051292 |
| Prss57 | 0.01211 | -1.05620169 |
| Rps3a1 | 0.39764 | 0.16104285 |
| Cep250 | 0.64004 | -0.25534269 |
| Gm8675 | 0.02928 | -0.48484622 |
| Ripk4 | 0.01797 | -0.7692592 |
| Sva | 0.01358 | -1.69582095 |
| Hdgfrp3 | 0.24679 | 0.81419395 |
| Heca | 0.01823 | 0.50739705 |
| Cnn3 | 0.71052 | 0.10778839 |
| Gm33045 | 0.00144 | -1.49514228 |
| Uba2 | 0.19388 | 0.272164 |
| Ms4a4c | 0.02962 | -0.51084112 |
| Gm10752 | 0.00144 | -1.28116048 |
| Fancl | 0.59581 | -0.1083032 |
| Gm13023 | 0.00144 | -1.28116048 |
| Gucy2f | 0.22676 | -0.80682282 |
| Gm35438 | 0.00144 | -1.70872646 |
| Zfp526 | 0.21945 | -0.52989801 |
| Gm9912 | 0.03853 | 0.81594788 |
| Gm34843 | 0.48331 | -0.36609571 |
| BC024978 | 0.08448 | -0.33388261 |
| Slco1a5 | 0.00144 | -1.27165604 |
| Rps18 | 0.00402 | -0.77230017 |
| Rps18 | 0.01046 | -0.76996265 |
| Rpgrip1 | 0.06422 | 0.36168825 |
| Dnase1l2 | 0.71663 | -0.12355729 |
| Gm10616 | 0.00415 | -1.06195552 |
| Cnpy4 | 0.00144 | -1.24613586 |
| Gzmc | 0.0507 | -0.88511728 |
| Gm14703 | 0.31973 | -0.33574253 |
| Sh2d7 | 0.23891 | 0.67401062 |
| Gm5907 | 0.00144 | -1.27165604 |
| Srsf12 | 0.23478 | -0.7433352 |
| Pkd1l2 | 0.00144 | -1.25118919 |
| Evl | 0.0044 | -0.72284155 |
| Nol3 | 0.91084 | 0.02629072 |
| Gm7276 | 0.11345 | -1.91440819 |
| Mcoln3 | 0.15428 | -0.71731073 |
| Zfp358 | 0.02603 | -0.77766736 |
| Gm31025 | 0.09858 | -2.65995129 |
| Slfn10-ps | 0.15993 | 1.57336098 |
| Lcn12 | 0.00786 | -1.34987692 |
| Lce1c | 0.64617 | 0.12156371 |
| Vmn2r19 | 0.00144 | -1.29051721 |
| H2-M5 | 0.05527 | -1.54479862 |
| H2-M2 | 0.34156 | -0.71420147 |
| Hyi | 0.00185 | -0.92031295 |
| Hist1h4m | 0.00562 | -2.30170067 |
| Golgb1 | 0.0866 | 0.45121232 |
| Krtap10-4 | 0.16234 | -0.36107318 |
| Cinp | 0.45621 | -0.2016561 |
| Cinp | 0.00363 | -0.73699491 |
| Qsox1 | 0.00145 | -1.00794817 |
| A130077B15Rik | 0.00144 | 2.08912951 |
| Fam227a | 0.29462 | -0.65449137 |
| Med12l | 0.81162 | -0.12090875 |
| BC049715 | 0.37276 | -0.41551543 |
| Fam71e1 | 0.00772 | -0.81166413 |
| Gm10545 | 0.00144 | -1.29049509 |
| Ms4a13 | 0.01367 | -0.896971 |
| Kctd8 | 0.87038 | -0.15337513 |
| Olfr556 | 0.00144 | -1.30713006 |
| Olfr544 | 0.00144 | -1.32775172 |
| Msl3 | 0.5826 | 0.0953833 |
| Olfr1110 | 0.46746 | -0.40926173 |
| Gm10517 | 0.00144 | -2.37968801 |
| Hmgb1 | 0.56956 | -0.11314163 |
| Foxa2 | 0.00144 | -1.31884045 |
| Wdr31 | 0.09512 | 1.02435449 |
| Gm36543 | 0.00144 | -1.26560808 |
| Whrn | 0.1597 | 0.7814209 |
| 1700121N20Rik | 0.13207 | -2.72982222 |
| Mzt2 | 0.00524 | -1.05977989 |
| Spanxn4 | 0.00144 | -1.30017714 |
| Brsk2 | 0.77432 | 0.08856309 |
| Tex22 | 0.02584 | -1.35296456 |
| Gm6867 | 0.00679 | -1.11765636 |
| Oxa1l | 0.11752 | -0.44808101 |
| Gm10792 | 0.02553 | 0.60204494 |
| Gm436 | 0.0249 | -0.61462872 |
| Slc38a8 | 0.09249 | -2.96860857 |
| Olfr1465 | 0.00145 | -1.2551153 |
| Tas2r115 | 0.10681 | -2.62505524 |
| Ttll10 | 0.11397 | -0.31659488 |
| Hnrnph2 | 0.02713 | 0.66811806 |
| Eid2b | 0.5688 | 0.09073071 |
| Sipa1 | 0.01682 | -0.57022073 |
| 1700023F06Rik | 0.91628 | 0.05227548 |
| 6430531B16Rik | 0.07097 | -1.83887367 |
| Nos1 | 0.03869 | -0.92436403 |
| Dppa1 | 0.00282 | -1.84880582 |
| Usp36 | 0.01004 | -0.74478799 |
| Olfr1197 | 0.00144 | -1.28898079 |
| Tax1bp3 | 0.00416 | -0.723092 |
| Cep162 | 0.00244 | 1.62115081 |
| Cep162 | 0.24776 | -0.54606603 |
| Etfa | 0.00151 | 1.39634765 |
| Sipa1l2 | 0.03106 | -0.57802988 |
| Ndufaf5 | 0.00564 | -1.17300636 |
| Nbr1 | 0.00768 | 0.5883414 |
| Gapvd1 | 0.02448 | 0.44689863 |
| Sbk2 | 0.45235 | -0.57343358 |
| Gm7173 | 0.00144 | -1.27165604 |
| Prss48 | 0.02498 | -0.8172328 |
| Vkorc1 | 0.00756 | -0.6669266 |
| Gm39115 | 0.24586 | -0.48363698 |
| Dmkn | 0.04052 | -0.95081427 |
| Gm4610 | 0.00144 | -1.24811894 |
| 1110017D15Rik | 0.18293 | -0.49099802 |
| Steap2 | 0.93189 | 0.04210338 |
| Cxx1c | 0.01635 | -0.68381139 |
| Mad2l1 | 0.25429 | 0.17929685 |
| Mmgt2 | 0.03425 | -0.45069839 |
| Arhgef39 | 0.98144 | -0.00733315 |
| Ino80e | 0.02237 | -1.2026609 |
| Gm4951 | 0.31499 | 0.42102188 |
| Wwc1 | 0.85407 | -0.0780753 |
| Rin3 | 0.88702 | 0.0508072 |
| Tgds | 0.21413 | -0.20693805 |
| Ube2v1 | 0.93223 | -0.03446357 |
| Kirrel3 | 0.37717 | -0.17029781 |
| Cntfr | 0.79631 | -0.1030951 |
| Cntfr | 0.01268 | -0.91494615 |
| Bhlhe41 | 0.00144 | -1.31703101 |
| Cypt10 | 0.00144 | -1.25118919 |
| LOC102631585 | 0.13446 | -0.54728252 |
| Gm29776 | 0.17425 | -0.28834762 |
| Adal | 0.59932 | -0.08669516 |
| Mageb1 | 0.01344 | -0.66098766 |
| Anxa4 | 0.25464 | -0.54363293 |
| 1600023N17Rik | 0.07767 | -0.73942934 |
| Sdcbp | 0.12628 | -0.28996063 |
| Sdcbp | 0.09489 | 1.34867101 |
| Sumo2 | 0.08681 | 0.4726905 |
| Rps16 | 0.00145 | -0.98362522 |
| Vmn1r178 | 0.00144 | -1.29049509 |
| Hsd3b2 | 0.00144 | 1.99442378 |
| Zfp367 | 0.00144 | -1.72292113 |
| Cacna1g | 0.34965 | -0.34534904 |
| Tmem248 | 0.77903 | 0.09284858 |
| LOC100041708 | 0.02434 | 0.52006954 |
| LOC102634333 | 0.00174 | -0.8594149 |
| Gm2447 | 0.00211 | -2.38916609 |
| Cd48 | 0.65229 | -0.37447945 |
| Gmppa | 0.81143 | 0.05849506 |
| Ngef | 0.03876 | -0.91663488 |
| Bcl2l2 | 0.084 | -0.29160497 |
| Pkn1 | 0.00411 | -0.88603835 |
| Crb1 | 0.00145 | -1.21051401 |
| Prpf4b | 0.00383 | 1.29373391 |
| Nfx1 | 0.17119 | 0.23995129 |
| Pbdc1 | 0.5276 | 0.12441121 |
| Luzp1 | 0.01036 | -0.61998311 |
| Mthfr | 0.00489 | -1.62753453 |
| Fastkd3 | 0.39998 | 0.22628289 |
| Speer5-ps1 | 0.91971 | -0.04985879 |
| Gm10538 | 0.00144 | -1.29085005 |
| Gm14486 | 0.04038 | -2.23551476 |
| ND3 | 0.0063 | -0.74768158 |
| Gm5532 | 0.0205 | 0.62545958 |
| Gm553 | 0.00597 | -1.81778229 |
| Tekt1 | 0.04124 | 0.63818139 |
| Det1 | 0.25202 | -0.44835777 |
| Pdia2 | 0.0111 | -1.02598223 |
| Gm2590 | 0.01543 | 1.57104206 |
| Prrx1 | 0.00144 | 1.35045868 |
| Poldip3 | 0.00173 | -0.90059383 |
| Krtap10-10 | 0.00488 | -0.77645627 |
| A530006G24Rik | 0.03077 | -0.47024074 |
| Gm10636 | 0.00144 | -1.29864962 |
| LOC100045026 | 0.00144 | -1.29746368 |
| Lpcat1 | 0.00144 | -1.14486607 |
| Mical2 | 0.0055 | 2.33354164 |
| Vstm2b | 0.00144 | -1.26911831 |
| Trim40 | 0.33293 | -0.42269588 |
| Wdr83 | 0.0736 | 0.34212674 |
| Trnau1ap | 0.11533 | -0.45375359 |
| Arfrp1 | 0.0308 | -0.62717861 |
| Vash1 | 0.34561 | -0.28272188 |
| Tamm41 | 0.49867 | -0.2950688 |
| Hmgb1 | 0.01361 | -1.01876051 |
| Rmnd5a | 0.0143 | -0.53217787 |
| Otogl | 0.13837 | -0.74746769 |
| Hist2h2aa2 | 0.00144 | -1.39497892 |
| Mettl10 | 0.00194 | 1.05275278 |
| Loxl2 | 0.3323 | -0.20632346 |
| Gm10785 | 0.16947 | -1.95084872 |
| Ank2 | 0.00799 | 0.77153547 |
| Ank2 | 0.43905 | 0.57221319 |
| Zfp663 | 0.40027 | 0.23987096 |
| Ttc39a | 0.00144 | -1.29746368 |
| Ttc39a | 0.44193 | -0.47459704 |
| Echdc3 | 0.42699 | -0.30223331 |
| Gm13083 | 0.03823 | -0.98294245 |
| Helq | 0.11397 | 0.34383204 |
| Vwa5a | 0.06184 | 0.44304174 |
| 9530053H05Rik | 0.00623 | 1.77713555 |
| D830050J10Rik | 0.0017 | -1.19401501 |
| Parp10 | 0.00317 | -0.85874748 |
| Nhp2 | 0.00145 | -1.08266025 |
| Parva | 0.0626 | -0.44943252 |
| Mas1 | 0.09617 | 0.68892296 |
| Parl | 0.71134 | -0.08543846 |
| Nap1l1 | 0.00144 | 1.4238397 |
| Gm4200 | 0.00625 | -0.91933169 |
| Armc4 | 0.00153 | -1.24063982 |
| Armc1 | 0.75005 | -0.05633061 |
| Tmc3 | 0.00144 | -1.27165604 |
| Tmc3 | 0.00906 | -1.02209498 |
| Smc3 | 0.00144 | 1.498656 |
| Musk | 0.00144 | -1.28898079 |
| Jakmip1 | 0.23895 | -0.967839 |
| Prdm11 | 0.20097 | -1.03467582 |
| Arl14ep | 0.10683 | 0.77046581 |
| Sdr16c6 | 0.00145 | -1.26219879 |
| 6530409C15Rik | 0.16567 | 0.68266226 |
| Tmem51os1 | 0.7842 | -0.09443327 |
| Nav1 | 0.83862 | -0.08229479 |
| Rilp | 0.18326 | -0.42697003 |
| Pnpla1 | 0.00366 | 1.08949861 |
| Dcaf11 | 0.0592 | -0.49825462 |
| Nup93 | 0.00144 | -1.27165604 |
| Nlrp5-ps | 0.70631 | -0.12339442 |
| Olfr1135 | 0.82178 | -0.26077082 |
| Polr2l | 0.07577 | -0.48201873 |
| Sh3gl2 | 0.10426 | -1.16641088 |
| Sh3gl2 | 0.01163 | -1.59404557 |
| Sh3gl2 | 0.03045 | -0.79513267 |
| Zbbx | 0.00144 | -1.5961653 |
| Fn1 | 0.01406 | -0.55376302 |
| BC006965 | 0.00144 | -1.31884045 |
| Cdh13 | 0.96567 | -0.01032649 |
| 5830477G23Rik | 0.00144 | -1.30713006 |
| AY761184 | 0.00653 | -2.01625649 |
| Esp6 | 0.00337 | -1.80883312 |
| Rabgef1 | 0.00776 | -0.59010205 |
| Chit1 | 0.00144 | -1.33632959 |
| Gm9295 | 0.09702 | 0.55314721 |
| Chd7 | 0.34473 | -0.18784753 |
| Gm14812 | 0.00144 | -1.31495282 |
| Mtfmt | 0.58729 | 0.09696696 |
| Slc38a10 | 0.00144 | -1.42306908 |
| Prrt1 | 0.92899 | -0.04085992 |
| Prrt1 | 0.05611 | -0.41588157 |
| Prrt3 | 0.00583 | -1.89800175 |
| Srsf4 | 0.09027 | -0.34139185 |
| Smad9 | 0.15795 | -0.60093735 |
| AB124611 | 0.00373 | -1.08087259 |
| Pkig | 0.75763 | 0.19705223 |
| H2-M10.3 | 0.00158 | -1.2446827 |
| Akp3 | 0.13969 | -0.74212044 |
| Nrp | 0.16481 | -0.65301723 |
| Cnpy1 | 0.00144 | -1.26934956 |
| Gm10860 | 0.53516 | 0.31274823 |
| Gm9519 | 0.02435 | 1.08279969 |
| Trerf1 | 0.88289 | -0.06008712 |
| Gm5712 | 0.0263 | -0.57861121 |
| Spata3 | 0.00144 | -1.26301121 |
| Wfdc10 | 0.10497 | -0.63243235 |
| Sult3a1 | 0.00144 | -1.28116048 |
| Gm10030 | 0.50472 | -0.30077171 |
| Ppap2c | 0.01097 | -0.88746628 |
| 4932431P20Rik | 0.00144 | 4.60549247 |
| Gm9736 | 0.00144 | -1.24865146 |
| Capn11 | 0.00144 | -1.56968216 |
| Palb2 | 0.11844 | -0.66163104 |
| Gm3807 | 0.14647 | -0.63471005 |
| Ntng2 | 0.27063 | -0.72708411 |
| Pilra | 0.32041 | -0.33455492 |
| Pilra | 0.75796 | -0.15892281 |
| 2210016L21Rik | 0.02912 | -0.51301989 |
| Gm38485 | 0.75987 | -0.06293598 |
| Dclre1c | 0.1075 | 0.66395654 |
| Zc3hc1 | 0.04178 | -0.62587544 |
| Tead2 | 0.11369 | -0.31328955 |
| Arhgap36 | 0.00144 | -1.30713006 |
| Calm2 | 0.05487 | 0.41815174 |
| Rab40b | 0.81392 | -0.06029685 |
| 1700084C01Rik | 0.01448 | -0.82682714 |
| Cdc42bpa | 0.07104 | 0.44435602 |
| Gm5464 | 0.01244 | -1.07815508 |
| Tlr7 | 0.85303 | -0.09873517 |
| Tlr7 | 0.00734 | -1.08892885 |
| Cyyr1 | 0.27919 | 0.32020588 |
| Wbscr27 | 0.02355 | -0.53660465 |
| Ccdc152 | 0.02728 | -0.90199263 |
| Cc2d2a | 0.0134 | -0.8072969 |
| Cep85 | 0.02366 | -0.82670069 |
| Ccnb1 | 0.00487 | -1.05523347 |
| Gm10855 | 0.0272 | 0.54259578 |
| 3830408C21Rik | 0.00149 | -1.2703832 |
| Gm4349 | 0.01803 | -0.78761311 |
| Nr1h5 | 0.00144 | -1.29864962 |
| Klk6 | 0.00144 | -1.28898079 |
| Dclre1b | 0.01655 | 0.94364725 |
| Pfn2 | 0.15719 | 0.37550098 |
| Mgl2 | 0.68168 | -0.08357821 |
| Rap1a | 0.0891 | 0.40276763 |
| Dnhd1 | 0.01726 | 1.13569149 |
| Sfpq | 0.06176 | 0.3800374 |
| Gm10673 | 0.00353 | -1.08965911 |
| Ahrr | 0.994 | 0.01122334 |
| Ubap2l | 0.71615 | -0.10058997 |
| Nr2f2 | 0.06778 | 0.63966796 |
| Phf21a | 0.87467 | 0.04273576 |
| Phf21a | 0.1378 | -0.30463601 |
| Rpl18 | 0.00327 | -0.97740004 |
| Trappc13 | 0.02769 | 0.58918088 |
| Col6a1 | 0.05399 | -0.38938013 |
| D830031N03Rik | 0.00521 | 1.66011179 |
| Il19 | 0.0823 | -0.63586146 |
| 5830411N06Rik | 0.02757 | -1.52726507 |
| LOC102641654 | 0.66589 | 0.16368873 |
| Klk12 | 0.00144 | -1.24613586 |
| Hsf2 | 0.66134 | 0.07659609 |
| Zfp36l3 | 0.00144 | -1.69538251 |
| Arhgef19 | 0.01063 | -0.9957843 |
| Palm3 | 0.37743 | 0.32660737 |
| Spata17 | 0.11575 | -2.66182274 |
| Spata17 | 0.00706 | -1.18479139 |
| Bcas3 | 0.07888 | 0.90718475 |
| Bcas3 | 0.00144 | -1.4261749 |
| Fbxo17 | 0.00381 | -1.71778612 |
| Racgap1 | 0.06351 | -0.56431906 |
| Pira2 | 0.01333 | -0.78378818 |
| Timm50 | 0.2939 | -0.45952491 |
| 4933434E20Rik | 0.41854 | -0.15971616 |
| 4933434E20Rik | 0.15171 | 0.59300937 |
| Acsbg1 | 0.55569 | 0.27763 |
| Smcr8 | 0.12282 | -0.5493329 |
| Smcr8 | 0.26414 | 0.51488226 |
| Abracl | 0.00322 | 0.79428702 |
| Trim35 | 0.10873 | -0.36768402 |
| Cped1 | 0.35985 | 0.58078776 |
| Kri1 | 0.04922 | -0.3661856 |
| Olfr1023 | 0.04956 | -1.54065722 |
| Spsb2 | 0.00144 | -1.52464871 |
| Spsb1 | 0.99026 | -0.00703542 |
| Tpsb2 | 0.74998 | -0.17896728 |
| Phc3 | 0.44174 | 0.20053925 |
| 4931422A03Rik | 0.00144 | -1.29085005 |
| Il24 | 0.00367 | -1.33983982 |
| Amigo1 | 0.00846 | -0.61988589 |
| Zfp628 | 0.00144 | -1.10728111 |
| Hist1h3d | 0.00689 | -0.67074479 |
| Trmt112 | 0.01348 | -0.78654362 |
| Olfr632 | 0.01801 | -1.29400531 |
| Olfr432 | 0.00926 | -1.25888604 |
| Gm6406 | 0.00144 | -1.25118919 |
| 4930553J12Rik | 0.00144 | -1.28116048 |
| Nup54 | 0.00144 | 1.0481477 |
| Olfr720 | 0.0041 | -1.61256367 |
| Zfp467 | 0.00752 | -0.80639857 |
| Zfp467 | 0.00574 | -0.81548318 |
| 1700066B19Rik | 0.93399 | -0.02528534 |
| Msantd3 | 0.00744 | -0.72322497 |
| Safb2 | 0.00372 | -1.19361937 |
| Nsf | 0.19958 | 0.52501935 |
| Nudt11 | 0.01185 | 0.54927007 |
| Gm5800 | 0.89194 | 0.04370262 |
| Wdr81 | 0.00144 | -1.10774224 |
| Pgam5 | 0.36136 | -0.24281617 |
| Fam219b | 0.00557 | -0.9828931 |
| Cav1 | 0.35898 | 0.23041169 |
| Nav3 | 0.04724 | -0.77582209 |
| Gm42192 | 0.62315 | 0.08212671 |
| Tll2 | 0.0029 | -1.06147486 |
| Gm41515 | 0.0249 | -0.89267786 |
| Tmem25 | 0.25741 | -0.36737608 |
| Ssmem1 | 0.31215 | -0.54419753 |
| Gm5858 | 0.86791 | 0.05451536 |
| Zdhhc13 | 0.06078 | 0.38604113 |
| Fam53c | 0.02706 | -0.57586454 |
| Trhr | 0.00144 | -1.29085005 |
| Atxn7l3b | 0.0059 | -0.67248477 |
| Scn2a1 | 0.09257 | 0.51219983 |
| Rpl7 | 0.24317 | -0.20680714 |
| 1810032O08Rik | 0.17665 | -0.337115 |
| Speer4a | 0.00681 | -1.08745708 |
| Dhodh | 0.56057 | -0.12679305 |
| Atad5 | 0.00144 | 1.9631073 |
| Wnt10b | 0.01186 | -1.24291503 |
| Cyp2a12 | 0.00144 | -1.30713006 |
| Iqck | 0.00144 | 2.29842245 |
| 1600014K23Rik | 0.64661 | -0.15189316 |
| Lgr5 | 0.00198 | -1.11811652 |
| Mamstr | 0.24387 | -0.61232046 |
| Usp16 | 0.00144 | 1.31254245 |
| Mrps21 | 0.02033 | -0.66262851 |
| Lrrc4b | 0.58787 | -0.18025612 |
| Adam26b | 0.00663 | -0.99120751 |
| Cct6a | 0.00853 | 0.75235971 |
| Cog1 | 0.2245 | -0.36628405 |
| Gm14440 | 0.03557 | 0.48699919 |
| Esam | 0.00282 | -0.81492855 |
| Gm3740 | 0.29974 | 0.37625194 |
| Lrrc8e | 0.01954 | -0.7436333 |
| Sec14l1 | 0.83871 | 0.05990183 |
| Ola1 | 0.00274 | 0.91027511 |
| Ola1 | 0.0406 | 0.61687252 |
| Tas2r119 | 0.00144 | -1.22925777 |
| Camkk2 | 0.04397 | -0.5884286 |
| Trank1 | 0.00446 | -1.87975012 |
| Rab11fip4os1 | 0.01073 | -1.90619814 |
| Acbd5 | 0.67876 | 0.1127526 |
| Fam71f2 | 0.47143 | 0.40908693 |
| Lrrc58 | 0.00144 | 1.38701753 |
| Mrps36 | 0.14534 | -0.48637678 |
| Mmadhc | 0.94137 | 0.01360648 |
| Tmem210 | 0.14214 | -0.49064628 |
| Evx1 | 0.00144 | -1.26560808 |
| Gm10731 | 0.64767 | 0.19442166 |
| Tnfrsf4 | 0.36456 | -0.27441725 |
| Gal3st3 | 0.82487 | -0.15917166 |
| Cacna1d | 0.87 | -0.0758035 |
| Rab3b | 0.09196 | -0.69536116 |
| Celf2 | 0.00144 | -1.29085005 |
| Brd1 | 0.80891 | -0.07916237 |
| Arhgef2 | 0.07734 | -0.34649959 |
| Arid5a | 0.01098 | -0.92404876 |
| Mterf1b | 0.88725 | 0.03978638 |
| Ext2 | 0.98005 | -0.00412533 |
| Gm5476 | 0.00144 | -1.23532597 |
| Tulp1 | 0.48889 | 0.17141152 |
| Gm13128 | 0.34823 | -1.70125404 |
| Pramef17 | 0.00144 | -1.26560808 |
| Gm11127 | 0.00157 | -1.19011363 |
| Hist1h4j | 0.00206 | -1.24108882 |
| Brinp1 | 0.08058 | 0.97959998 |
| Gm10436 | 0.00144 | -1.28116048 |
| Olfr121 | 0.00875 | -1.79249457 |
| Cyp2j13 | 0.06004 | -2.20737502 |
| Ces2c | 0.18303 | -0.82279767 |
| Tas2r102 | 0.00687 | -1.7976704 |
| Gxylt2 | 0.03948 | -1.19831553 |
| Smurf2 | 0.00145 | -1.00514924 |
| Xlr4a | 0.94796 | 0.0723697 |
| Xlr4a | 0.20301 | -1.14741906 |
| Cep41 | 0.03227 | -0.9173515 |
| Cep41 | 0.00776 | -0.86702868 |
| Capns1 | 0.00435 | -0.86954126 |
| Il17rb | 0.24704 | -0.59164448 |
| Olfr220 | 0.00624 | -1.55067777 |
| Zfp661 | 0.02256 | 0.61718567 |
| Gm29776 | 0.02417 | -0.69397252 |
| Ak4 | 0.00302 | -1.10952158 |
| Ivns1abp | 0.00633 | 1.02424204 |
| Olfr1318 | 0.99819 | -0.00184606 |
| Vmn1r231 | 0.00144 | -1.52432995 |
| Vmn1r228 | 0.00144 | -1.31495282 |
| Vsig8 | 0.00686 | -0.87596532 |
| Uaca | 0.02292 | 0.46142692 |
| Rusc2 | 0.00144 | -1.01627815 |
| Gm10344 | 0.13932 | -0.85751533 |
| Ywhae | 0.00274 | 0.86087681 |
| Invs | 0.02666 | 0.64723898 |
| Ttc7 | 0.00145 | -1.06441493 |
| Gm6981 | 0.29772 | -0.40093952 |
| Ambra1 | 0.0689 | -0.31891046 |
| Tceanc | 0.07269 | -0.71041484 |
| Synj2 | 0.53381 | -0.1519807 |
| Arid3b | 0.00144 | -1.28898079 |
| Minos1 | 0.02256 | -0.5910275 |
| Acvr1b | 0.20234 | 0.9270843 |
| Lmbrd1 | 0.23427 | 0.24131886 |
| Tmc5 | 0.04028 | -1.58449218 |
| D2hgdh | 0.72053 | 0.11152717 |
| Tmem200b | 0.37973 | 0.26842453 |
| Nhlh2 | 0.00144 | -1.26934956 |
| Zfp616 | 0.00144 | -1.29864962 |
| Wnk3 | 0.00144 | -1.30713006 |
| Zfhx4 | 0.04172 | -0.90881164 |
| Thap6 | 0.00966 | 0.92721934 |
| Nfe2l3 | 0.1936 | -0.67491186 |
| Rbbp7 | 0.02777 | 0.54958045 |
| Zp1 | 0.00145 | -1.21845061 |
| Gm5127 | 0.04824 | -0.86696801 |
| Vmn2r16 | 0.18928 | -0.73707107 |
| Sipa1l2 | 0.00144 | -1.22730863 |
| Olfr819 | 0.00144 | -1.29746368 |
| Jam2 | 0.49152 | 0.23189118 |
| B3gnt5 | 0.98802 | -0.00767608 |
| Gm11627 | 0.48636 | -0.14544435 |
| Kpna2 | 0.1104 | 0.44243718 |
| 4930594M22Rik | 0.00144 | -1.31495282 |
| Fga | 0.00144 | -2.07748203 |
| Cog2 | 0.26259 | -0.21208383 |
| Kcna6 | 0.00144 | -1.26560808 |
| Olfr1321 | 0.85303 | -0.20519391 |
| Akirin1 | 0.79176 | 0.09471703 |
| Prr5l | 0.00989 | -1.44513012 |
| Zic5 | 0.00144 | -1.45718629 |
| Dos | 0.03514 | -0.60224814 |
| Cbfa2t3 | 0.13679 | -1.40957542 |
| Tet1 | 0.29818 | -0.36336563 |
| Tet1 | 0.05899 | -0.39305319 |
| Dlx3 | 0.01156 | 0.71466832 |
| Olfr741 | 0.00144 | -1.30713006 |
| Olfr541 | 0.00144 | -1.26428512 |
| Wdyhv1 | 0.81371 | 0.12271422 |
| Rnf25 | 0.00213 | -0.83327704 |
| Pla2g4e | 0.12761 | -0.67549074 |
| Galnt5 | 0.02893 | -1.8351382 |
| Pcbp3 | 0.00351 | -1.61867509 |
| Zfp185 | 0.00144 | 1.96637433 |
| Homer1 | 0.23188 | -0.18753163 |
| Arhgef3 | 0.02446 | 0.65090868 |
| Zmym3 | 0.46212 | -0.1450623 |
| Gm40313 | 0.05588 | -0.97550887 |
| Dnah3 | 0.0024 | -1.20214741 |
| Dtymk | 0.00262 | -1.21222136 |
| Airn | 0.26656 | 0.53452427 |
| Asb11 | 0.51068 | 0.13848752 |
| Asb11 | 0.72457 | -0.06531118 |
| Vmn2r104 | 0.00329 | -1.31427976 |
| Cbr2 | 0.04848 | -0.67469154 |
| Csnk1e | 0.31661 | -0.25025302 |
| Csnk1e | 0.27051 | -0.21069974 |
| Edil3 | 0.00144 | -1.29085005 |
| Arl1 | 0.00329 | 0.98676087 |
| Arl1 | 0.00523 | 0.67559934 |
| Zfp14 | 0.00289 | 0.76930092 |
| 9230020A06Rik | 0.03557 | -0.90858039 |
| Nfic | 0.00144 | -1.62975372 |
| Emc1 | 0.01786 | -0.78429935 |
| Cir1 | 0.41819 | 0.24450833 |
| Top3a | 0.02592 | -1.63354301 |
| Kcnk4 | 0.00787 | -0.74580403 |
| 1700019M22Rik | 0.76898 | -0.29999039 |
| Adgrl3 | 0.00144 | -1.30017714 |
| Olfr907 | 0.02587 | -1.09163189 |
| Erich3 | 0.02143 | 0.88282181 |
| Olfr507 | 0.00144 | -1.28375735 |
| Olfr207 | 0.03855 | -1.20630328 |
| Olfr307 | 0.00145 | -1.22376446 |
| Ppp2r3d | 0.00499 | -0.96505204 |
| Olfr135 | 0.00144 | -1.26560808 |
| Gm30827 | 0.00159 | -0.97525527 |
| Plcb4 | 0.09707 | 0.3870184 |
| Psmc3 | 0.07201 | -0.42315756 |
| Gck | 0.18687 | -0.61834424 |
| Cnot6l | 0.2811 | 0.16821354 |
| Wiz | 0.01077 | -0.64597133 |
| Adi1 | 0.51469 | 0.29452545 |
| Ier5l | 0.00605 | -1.0374811 |
| Rps3a1 | 0.06038 | -0.35847968 |
| Dgcr2 | 0.5582 | -0.21072256 |
| Sp9 | 0.00144 | -1.30017714 |
| Gm10764 | 0.00145 | -1.26219879 |
| Gm9513 | 0.00165 | -1.35933417 |
| E530001K10Rik | 0.03418 | -0.9383227 |
| Alox12b | 0.07978 | -0.67870845 |
| Gprin2 | 0.00144 | -1.28116048 |
| Tox4 | 0.82081 | -0.28953111 |
| Sox4 | 0.0092 | -0.60234231 |
| Sox4 | 0.42925 | 0.15254833 |
| 2810047C21Rik1 | 0.18247 | -0.8303531 |
| Ankfy1 | 0.10816 | 0.63576816 |
| Rps20 | 0.00144 | -1.15926451 |
| Hist1h3i | 0.00251 | -1.0748749 |
| Ankfn1 | 0.00144 | -1.27699683 |
| AI849053 | 0.00144 | -1.26560808 |
| Bbs9 | 0.34395 | -0.20488063 |
| Fam43a | 0.00145 | -1.02776767 |
| Gm10751 | 0.07452 | 0.47361126 |
| Gm29720 | 0.0045 | -0.96401858 |
| Tpm2 | 0.02762 | -0.67142329 |
| Acaca | 0.14767 | -0.62113732 |
| Trp53i13 | 0.00144 | -1.13798682 |
| Cdk5 | 0.03615 | -0.50758704 |
| Nudcd3 | 0.00212 | -0.82976033 |
| Gm4907 | 0.35581 | -0.23074819 |
| Syt6 | 0.03821 | -1.17854493 |
| Clcc1 | 0.02903 | 0.60564167 |
| Nuggc | 0.77002 | 0.5320426 |
| Omp | 0.2496 | 0.55442735 |
| Efcc1 | 0.56444 | 0.33179573 |
| Gm31854 | 0.68447 | 0.14971616 |
| Znf41-ps | 0.04426 | 0.92608195 |
| Tbc1d22b | 0.02611 | -0.46128167 |
| Tbc1d22b | 0.01703 | -0.54161931 |
| Fbn2 | 0.48608 | -0.32263685 |
| 5730488B01Rik | 0.00144 | -1.25085248 |
| Cul2 | 0.00144 | 1.13470344 |
| Cul2 | 0.00351 | 1.92543017 |
| Gm10825 | 0.04089 | -0.96919479 |
| 4930413G21Rik | 0.00144 | -1.27165604 |
| Mefv | 0.34054 | 0.23288289 |
| Gm35550 | 0.18009 | -0.58061918 |
| Cldn24 | 0.00603 | -1.50434376 |
| Cracr2a | 0.53877 | -0.23859199 |
| Gm7120 | 0.00837 | 1.21405377 |
| Dctn4 | 0.37466 | -0.16351535 |
| Gm10537 | 0.00156 | -0.93732091 |
| Olfr75-ps1 | 0.00144 | -1.31495282 |
| Ssh3 | 0.4446 | -0.11986104 |
| Olfr1085 | 0.05758 | -0.95353231 |
| Wdtc1 | 0.1234 | -0.61236727 |
| Fanca | 0.04277 | -1.03849241 |
| Snx16 | 0.00235 | 0.91314389 |
| Skint7 | 0.03429 | -0.49959057 |
| Cib2 | 0.00448 | -0.79275822 |
| Pdcd7 | 0.02344 | -0.60835665 |
| Zfp457 | 0.02369 | -0.60662265 |
| Avpi1 | 0.00332 | -1.06231 |
| Rad51b | 0.00144 | -1.26560808 |
| Myh8 | 0.28434 | -0.6035074 |
| Zmynd11 | 0.91705 | -0.01641243 |
| Gm2984 | 0.02663 | -1.17152439 |
| Fgfr4 | 0.71711 | -0.2649943 |
| Gm7596 | 0.00144 | -1.26560808 |
| Baiap2 | 0.01623 | -0.57726201 |
| Gm5893 | 0.14686 | -1.12365051 |
| Bdh2 | 0.0047 | -1.01738989 |
| 1110034G24Rik | 0.18522 | -0.44322741 |
| Mlxipl | 0.64661 | 0.08358286 |
| Ikzf4 | 0.07982 | -0.39330644 |
| Dnah11 | 0.00454 | -1.04100801 |
| Erbb3 | 0.32921 | -0.42178152 |
| Pnisr | 0.06764 | 0.50450442 |
| Cd2bp2 | 0.02158 | -0.59410107 |
| D6Ertd474e | 0.00257 | -1.1333102 |
| Plekhj1 | 0.00933 | -0.70266841 |
| Scn3a | 0.01822 | 1.90936577 |
| Gm10228 | 0.65848 | 0.15790605 |
| AA414992 | 0.00144 | -1.27165604 |
| Defb43 | 0.00144 | -1.35112763 |
| Gnal | 0.28506 | 0.58283493 |
| Prss55 | 0.48154 | -0.1783195 |
| 4933409K07Rik | 0.00608 | -1.84379382 |
| Gm13298 | 0.16434 | -1.0328236 |
| Gstcd | 0.42292 | -0.3458249 |
| Ndufv2 | 0.07653 | -0.37174554 |
| Slc25a44 | 0.02935 | -0.95492833 |
| Vma21 | 0.23643 | -0.45313651 |
| Vma21 | 0.0402 | -0.75595047 |
| Usp10 | 0.00523 | -0.68163586 |
| Zeb2 | 0.00144 | -1.3230041 |
| 1700066M21Rik | 0.21195 | -0.47232383 |
| Pfkfb4 | 0.00144 | -1.28116048 |
| Cspp1 | 0.01654 | 1.68621721 |
| Grip2 | 0.93782 | -0.02963735 |
| Gm33222 | 0.00144 | -1.29051721 |
| Serpine1 | 0.07995 | 0.80168262 |
| Srrm3 | 0.00144 | -1.29085005 |
| Six2 | 0.00371 | -1.38485247 |
| Olfr96 | 0.31757 | 0.31852439 |
| Olfr16 | 0.49331 | -0.36983029 |
| Set | 0.18132 | 0.22114593 |
| Gm36913 | 0.00144 | -1.30713006 |
| Yipf1 | 0.00345 | -0.8825399 |
| Osbpl9 | 0.03537 | -0.62894491 |
| Foxe3 | 0.0096 | -1.46807948 |
| Foxe1 | 0.01185 | -1.18330208 |
| Foxe1 | 0.00148 | -1.15350668 |
| Foxj3 | 0.01385 | 0.5353077 |
| Mgat4c | 0.00592 | -1.07650415 |
| Ifltd1 | 0.07765 | -1.34594708 |
| Zfp882 | 0.0112 | 1.34526811 |
| 1700054O19Rik | 0.63187 | -0.1996283 |
| AY512931 | 0.00145 | -1.26219879 |
| 4930423O20Rik | 0.07927 | -0.71229873 |
| Platr28 | 0.00144 | -1.24613586 |
| 2310057N15Rik | 0.95805 | 0.05157136 |
| Otop2 | 0.00145 | -1.22518835 |
| Rcor2 | 0.0136 | -0.7594551 |
| Ninl | 0.19538 | 0.89306616 |
| Fam228b | 0.00236 | 0.91608672 |
| Idh2 | 0.00551 | -0.69149087 |
| Tnip1 | 0.00608 | -0.95442733 |
| Taar8b | 0.01082 | -1.63959699 |
| Taar7b | 0.00203 | -1.2841476 |
| Lasp1 | 0.01448 | -1.48342466 |
| Lasp1 | 0.1391 | -0.53675465 |
| LOC101056056 | 0.12322 | -0.8703416 |
| St6galnac1 | 0.83472 | -0.12581026 |
| St6galnac1 | 0.9488 | -0.02966834 |
| Cd37 | 0.01491 | -0.56894602 |
| H60c | 0.21241 | -0.78752336 |
| Col1a1 | 0.002 | -0.98299964 |
| B430212C06Rik | 0.00216 | -1.43268243 |
| Mx1 | 0.01347 | 1.4343974 |
| H2-T3 | 0.55285 | -0.47017401 |
| Lrp6 | 0.00378 | -0.91355759 |
| C230012O17Rik | 0.24926 | -0.77380727 |
| Gm38700 | 0.04747 | -0.92886545 |
| Chpf | 0.00144 | -1.11861454 |
| Gm7271 | 0.00145 | -1.22376446 |
| Igf2 | 0.85666 | -0.05729427 |
| LOC102641619 | 0.00603 | -1.4182713 |
| Zfp61 | 0.69718 | 0.18163642 |
| Zfp68 | 0.00163 | 1.17636094 |
| Gm10426 | 0.00144 | -1.28116048 |
| Gm12171 | 0.00144 | -1.27165604 |
| Pdzd7 | 0.01369 | -1.59448555 |
| 4930404H24Rik | 0.01626 | -0.64418646 |
| Leng8 | 0.12168 | -1.38316552 |
| 1700036A12Rik | 0.36231 | -0.59761845 |
| Cep63 | 0.02194 | -2.66927661 |
| Cyp4b1 | 0.00397 | -0.77450429 |
| Tas2r130 | 0.00149 | -1.22582153 |
| Vmn1r222 | 0.00144 | -1.26428512 |
| Lhx9 | 0.00144 | -1.40363226 |
| 5830415F09Rik | 0.02024 | -0.86490099 |
| Pigu | 0.46316 | 0.17879709 |
| Gm3474 | 0.00144 | -1.28898079 |
| Azi2 | 0.00144 | -1.24811894 |
| 4732440D04Rik | 0.00349 | -1.17027173 |
| Gm14092 | 0.81839 | -0.25118919 |
| Vangl2 | 0.09805 | -0.4678342 |
| Gm5925 | 0.00145 | -1.15845772 |
| Nck2 | 0.80592 | 0.03975427 |
| Pak4 | 0.03212 | -0.50589649 |
| Lppr3 | 0.72038 | -0.19437293 |
| Rnf185 | 0.03727 | -0.43246896 |
| Snhg1 | 0.18566 | 0.22264447 |
| Pak7 | 0.22288 | -0.40286221 |
| Rmnd1 | 0.26729 | 0.19021421 |
| Slfn8 | 0.00373 | 1.49810148 |
| Tnfrsf13c | 0.07281 | -1.09513407 |
| Olfr1082 | 0.00145 | -1.22518835 |
| Ppih | 0.90844 | 0.02749237 |
| Siglec15 | 0.00144 | -1.29746368 |
| Rbm3 | 0.16195 | 0.34355167 |
| Cftr | 0.00144 | -1.26560808 |
| Gm38710 | 0.01909 | 0.99076111 |
| AI481877 | 0.00145 | -1.22518835 |
| Gm6559 | 0.00196 | -1.65633744 |
| Syt5 | 0.01859 | -0.56930946 |
| Gm10257 | 0.42435 | 0.13828113 |
| Cacng7 | 0.00148 | -1.04104601 |
| A530017D24Rik | 0.67474 | 0.29197393 |
| Ntan1 | 0.27392 | 0.18693772 |
| Kcnt1 | 0.00144 | -1.21153109 |
| Enam | 0.00144 | -1.24811894 |
| Npm1 | 0.00349 | 1.07084675 |
| Lhfpl5 | 0.97664 | -0.00991666 |
| Mtx1 | 0.00442 | -0.74236749 |
| Olfr1243 | 0.00144 | -1.26934956 |
| Zmym1 | 0.00461 | 1.09587338 |
| Apoc3 | 0.07875 | -2.50940993 |
| Zfp937 | 0.01051 | 2.31370908 |
| Efhd1 | 0.14448 | -0.35944971 |
| Ccnj | 0.00581 | 1.92301576 |
| BC107364 | 0.00144 | -1.40054462 |
| Mtmr1 | 0.01485 | 0.62817191 |
| Hnrnpdl | 0.00335 | 1.37967349 |
| Neb | 0.01843 | 2.22877547 |
| Neb | 0.26833 | -0.39176345 |
| Dbi | 0.0024 | -0.94377934 |
| Gm10371 | 0.49593 | -0.13436075 |
| Vax1 | 0.06497 | -0.94950804 |
| A130010J15Rik | 0.39141 | -0.25047893 |
| Brpf1 | 0.03766 | -0.41566787 |
| Odf4 | 0.29788 | -0.65287907 |
| Xirp1 | 0.04237 | -0.56973954 |
| 3425401B19Rik | 0.70006 | 0.16595955 |
| Olfr1057 | 0.00144 | -1.28116048 |
| Tuba1b | 0.03112 | -0.65820473 |
| Gpr143 | 0.00144 | -1.37665893 |
| Gpr183 | 0.1021 | -1.86394732 |
| Gfm1 | 0.02691 | -0.74647003 |
| Tnfrsf25 | 0.4542 | 0.21297048 |
| Cyp2c38 | 0.10676 | -0.64446616 |
| D8Ertd82e | 0.00148 | -1.09576329 |
| Ap2a1 | 0.04942 | -0.40821077 |
| Gsc2 | 0.04117 | -2.00648344 |
| Xkr7 | 0.01652 | -1.42614982 |
| AY074887 | 0.01138 | -0.98574584 |
| Sms | 0.10617 | 0.46593196 |
| Xlr5c | 0.00144 | -1.31495282 |
| Gm14325 | 0.00306 | 1.26927466 |
| Olfr479 | 0.11391 | -2.93095178 |
| Raet1e | 0.35307 | -0.78613351 |
| Dsg1c | 0.23284 | 0.4554078 |
| Dsg1b | 0.00554 | -1.58979569 |
| 1700012D14Rik | 0.58203 | 0.12530126 |
| Inpp5j | 0.01776 | -0.92362558 |
| Gm10836 | 0.03389 | 1.00563883 |
| Acnat1 | 0.02272 | -1.88995047 |
| Rbmy | 0.02668 | -0.84947887 |
| Olfr1247 | 0.0119 | -1.06460776 |
| 5930422O12Rik | 0.0119 | 2.06508773 |
| Gm17026 | 0.0017 | -1.24950944 |
| Ikbip | 0.00144 | 1.3224458 |
| Slc20a1 | 0.6192 | 0.25285988 |
| Egfl8 | 0.00193 | -0.8872531 |
| Pot1a | 0.00303 | -1.87320288 |
| Gm35208 | 0.03504 | -1.95716175 |
| Nrg4 | 0.14298 | 1.1942693 |
| Ap3s1 | 0.00753 | 0.70539126 |
| Gm13202 | 0.00999 | -0.84785374 |
| LOC102642717 | 0.03843 | 2.15979458 |
| Fer | 0.01122 | 0.65665602 |
| Bckdhb | 0.06888 | 0.51693449 |
| Fap | 0.0278 | 0.72664772 |
| Gm11437 | 0.00145 | -1.2388121 |
| Sike1 | 0.62657 | 0.13246253 |
| Pdhx | 0.03014 | 0.43019765 |
| Pxylp1 | 0.11686 | -0.35575795 |
| 4632428N05Rik | 0.00377 | -0.96015836 |
| Rnaseh2a | 0.05871 | -0.5091983 |
| Tuba1a | 0.00186 | -0.97441857 |
| Neurl3 | 0.29466 | -0.52794351 |
| Zfp808 | 0.67611 | -0.20978554 |
| Fastkd1 | 0.05149 | 0.39889197 |
| Lonrf2 | 0.31489 | 0.22864493 |
| Exosc10 | 0.48927 | -0.12982918 |
| Btnl1 | 0.15084 | -1.0410572 |
| Usp18 | 0.30011 | -0.20012124 |
| Irf9 | 0.08179 | -0.3494355 |
| Nek3 | 0.43711 | 0.14759281 |
| Gdap2 | 0.96663 | -0.00828414 |
| Tceal3 | 0.00734 | -1.08892885 |
| Gtf2e2 | 0.00385 | 0.82706908 |
| Arntl2 | 0.02291 | 0.78948115 |
| Greb1 | 0.89531 | 0.04599941 |
| Gm7168 | 0.10717 | -0.6165873 |
| Mospd4 | 0.06792 | -0.68060673 |
| Olfr816 | 0.00144 | -1.28898079 |
| Gcm1 | 0.00144 | 3.53649871 |
| Zbtb14 | 0.6785 | 0.09835012 |
| Zfp191 | 0.15263 | 0.40933861 |
| Gm3924 | 0.04137 | -1.98271119 |
| Vwde | 0.00144 | -1.28898079 |
| Gm8801 | 0.15227 | -0.45842088 |
| Icmt | 0.00273 | -0.94312708 |
| Scaper | 0.00144 | -1.30713006 |
| Pvalb | 0.00385 | -1.81218484 |
| Creb1 | 0.20058 | 0.48833659 |
| Notum | 0.01495 | -0.49156237 |
| Ccdc162 | 0.00165 | -1.64577724 |
| Tbc1d31 | 0.37286 | 0.48382094 |
| Gm10872 | 0.2052 | -0.75604691 |
| Krt40 | 0.00158 | -1.2446827 |
| Mrpl24 | 0.02352 | -0.4437151 |
| 1700047I17Rik2 | 0.02766 | -0.53757953 |
| Zcchc7 | 0.00218 | 2.25129682 |
| Gm5105 | 0.14746 | 0.81123224 |
| Garnl3 | 0.83773 | 0.0334777 |
| Cadm1 | 0.15629 | -0.51876855 |
| Cadm4 | 0.02779 | -0.63260412 |
| Acrbp | 0.41243 | 0.30365726 |
| Hyls1 | 0.03869 | -0.92436403 |
| Ankra2 | 0.041 | 0.853205 |
| Mospd2 | 0.00216 | 0.95018387 |
| St6gal2 | 0.19025 | -0.59743667 |
| Nuf2 | 0.00144 | -1.28116048 |
| Zfp583 | 0.67934 | 0.16180894 |
| Serpina1e | 0.1858 | -1.2615879 |
| Tbc1d1 | 0.0062 | -0.68920396 |
| Yif1b | 0.13795 | -0.66931439 |
| Ubash3a | 0.03678 | -1.4155171 |
| Pecr | 0.99387 | -0.00270889 |
| Spib | 0.94015 | -0.04529709 |
| Nup205 | 0.65164 | 0.08548939 |
| Raph1 | 0.02408 | 0.76590129 |
| Raph1 | 0.03315 | 0.4449787 |
| Eml1 | 0.6493 | -0.07883898 |
| Sumo2 | 0.00144 | -1.29085005 |
| Bzw1 | 0.0019 | 0.85338369 |
| Ikzf3 | 0.66329 | 0.16127008 |
| Nudt3 | 0.4655 | 0.15304216 |
| Whsc1 | 0.09707 | 0.3560771 |
| Wash1 | 0.01462 | -0.71367568 |
| Foxp2 | 0.00144 | -1.26560808 |
| Zfp523 | 0.00229 | -0.76862994 |
| Tmem87a | 0.4927 | 0.35643946 |
| LOC100125594 | 0.09237 | -0.94346196 |
| Sik3 | 0.44084 | -0.22484519 |
| Parp11 | 0.26793 | -0.45732293 |
| Cyth4 | 0.00286 | -0.82296372 |
| LOC102638913 | 0.04316 | -0.48112595 |
| Mal | 0.03561 | 0.52661856 |
| Usp17lb | 0.00252 | -1.51614554 |
| Tmem132a | 0.01286 | -0.80124686 |
| Rem1 | 0.00294 | -1.14475146 |
| Ago4 | 0.11114 | -0.42788894 |
| Zkscan5 | 0.44565 | -0.12497986 |
| Fosb | 0.44155 | -0.39769782 |
| Foxo4 | 0.02161 | -0.87297327 |
| Npy6r | 0.63893 | -0.42919767 |
| Ddx18 | 0.32777 | 0.15857081 |
| Slc4a3 | 0.00144 | -1.02774935 |
| Klk1b22 | 0.18493 | 0.86869889 |
| Gm6288 | 0.00145 | -1.24191372 |
| Diap1 | 0.00215 | -1.03956057 |
| Vmn1r84 | 0.00298 | -1.42363422 |
| Olfr1033 | 0.10778 | -0.66008166 |
| C030034I22Rik | 0.04668 | 0.74399262 |
| Pate2 | 0.00144 | -1.30713006 |
| Impdh2 | 0.00557 | -0.64547851 |
| Spata45 | 0.00145 | -1.31323225 |
| Adh6a | 0.00144 | -1.27730451 |
| Vmn2r101 | 0.00144 | -1.24811894 |
| Gabrr3 | 0.0046 | -1.12385295 |
| Iigp1b | 0.00144 | -1.24613586 |
| Actb | 0.00354 | -0.91696684 |
| Sipa1l1 | 0.02218 | 0.55152246 |
| Habp2 | 0.00187 | -1.29995233 |
| Smad4 | 0.12569 | 0.42106116 |
| Gucy2d | 0.06011 | -0.44807354 |
| Vmn2r71 | 0.00144 | -1.28898079 |
| Tbr1 | 0.00144 | -1.28116048 |
| Ern1 | 0.62511 | 0.20933184 |
| Ern1 | 0.01123 | -1.04967308 |
| Ern1 | 0.05823 | -0.92245948 |
| Kcnmb2 | 0.00144 | -1.28116048 |
| Kcnv1 | 0.00144 | -1.28116048 |
| Rwdd3 | 0.00144 | 1.49954788 |
| Nudt16l1 | 0.06555 | -0.39977917 |
| Cldn22 | 0.01388 | -3.18559877 |
| Tbx4 | 0.00283 | -1.70999226 |
| Gm26691 | 0.00149 | -1.2703832 |
| BC051628 | 0.07521 | -1.15063496 |
| Olfr1008 | 0.0072 | 0.81489434 |
| Arhgap17 | 0.76727 | -0.05230342 |
| Anapc7 | 0.00337 | -0.82764599 |
| 6330444E15Rik | 0.55669 | -0.47503039 |
| Hmg20b | 0.0025 | -0.85757854 |
| Gm6471 | 0.00144 | -1.27454685 |
| Ccdc13 | 0.01597 | 1.404288 |
| Cdk5rap2 | 0.47912 | -0.14800642 |
| Cdk5rap2 | 0.36624 | 0.17659329 |
| Pcyt2 | 0.11378 | 0.38965813 |
| 4930564C03Rik | 0.31048 | -0.39327088 |
| Nhsl1 | 0.90967 | -0.01993677 |
| Crtc2 | 0.12632 | -0.33978042 |
| Crtc1 | 0.035 | -0.54618511 |
| Tmbim7 | 0.09337 | -2.81854026 |
| Tnk2 | 0.00145 | -0.91215837 |
| Tnk2 | 0.0248 | -0.54883136 |
| Ube2b | 0.31468 | -0.17323383 |
| Vwa8 | 0.31413 | -0.17418763 |
| Prss35 | 0.00232 | -1.15063496 |
| Hsd3b5 | 0.00158 | -1.2446827 |
| Dnajc7 | 0.05172 | 0.77891314 |
| Noxo1 | 0.89841 | -0.03070899 |
| Taf12 | 0.42128 | 0.37679118 |
| Rogdi | 0.87686 | 0.06003624 |
| Gale | 0.00157 | -0.96402845 |
| 1700001C02Rik | 0.00586 | -1.06337941 |
| Gem | 0.35987 | -0.22817114 |
| Klhl13 | 0.02745 | 0.58984857 |
| Rhox2c | 0.62095 | -0.19847536 |
| Rhox2f | 0.00144 | -1.27699683 |
| Npcd | 0.13774 | -1.09185749 |
| Spint2 | 0.00144 | -1.51842295 |
| Vmn2r13 | 0.00144 | -1.24811894 |
| Gabpb1 | 0.51661 | 0.18051496 |
| Itga10 | 0.14681 | 0.47522446 |
| E230008N13Rik | 0.0061 | -1.49504241 |
| Higd1b | 0.00158 | -0.8814936 |
| 9230113P08Rik | 0.22477 | -0.42390179 |
| Polq | 0.00144 | -1.38239536 |
| Polq | 0.25296 | 0.2817804 |
| Trpv3 | 0.00145 | -1.22376446 |
| Trpv6 | 0.03219 | -1.42556172 |
| Gpsm1 | 0.41685 | -0.26540857 |
| Btf3l4 | 0.06995 | 0.6444742 |
| Eny2 | 0.1155 | 0.29558118 |
| Pcp2 | 0.00649 | -1.10326729 |
| Efcc1 | 0.00156 | -1.62225687 |
| Ctnnd2 | 0.02621 | 1.02569468 |
| Egfl7 | 0.00144 | -1.15222244 |
| Klhl32 | 0.26151 | 0.49128496 |
| Klhl32 | 0.00582 | -0.67019222 |
| Cp | 0.00699 | 0.91777977 |
| 3110009E18Rik | 0.13555 | -0.38363789 |
| 3110009E18Rik | 0.04339 | -0.52793146 |
| Cdc73 | 0.67454 | 0.0835033 |
| Lhx9 | 0.00144 | -1.27853773 |
| 9130209A04Rik | 0.06797 | -2.2742009 |
| Mecom | 0.02993 | 1.08651154 |
| Rgs6 | 0.30809 | 0.38487065 |
| Ccdc105 | 0.00968 | -1.79523571 |
| Olfr550 | 0.43549 | 0.2821967 |
| Olfr250 | 0.57174 | -0.46587348 |
| Gpr153 | 0.00425 | -0.80345802 |
| Atp6ap1 | 0.36924 | 0.40480389 |
| Flrt1 | 0.03931 | -0.77751561 |
| Flrt1 | 0.01398 | -0.76705425 |
| 2700046G09Rik | 0.05319 | -0.55649238 |
| Olfr1090 | 0.24915 | -1.06995316 |
| Rnf7 | 0.8293 | 0.07231792 |
| B430306N03Rik | 0.01662 | -0.90820096 |
| Mettl7a2 | 0.09102 | 0.61683007 |
| Pttg1 | 0.01753 | 0.66264988 |
| Adgrb2 | 0.98316 | 0.00864704 |
| Anxa2 | 0.00248 | -0.83671738 |
| Hhat | 0.00144 | -1.28116048 |
| BC107364 | 0.00266 | 1.81226576 |
| Krtap4-9 | 0.2045 | -0.40136881 |
| Slc37a2 | 0.5909 | -0.24833119 |
| 4933431J24Rik | 0.00144 | -1.28898079 |
| Proca1 | 0.60287 | 0.11631522 |
| Chrnd | 0.00145 | -1.22376446 |
| Slfn9 | 0.00144 | 2.11665838 |
| Olfr763 | 0.10557 | -2.67884817 |
| Gm10253 | 0.14728 | -0.64776933 |
| Omt2a | 0.00149 | -1.22582153 |
| 3830406C13Rik | 0.14595 | -0.35529335 |
| Skint4 | 0.01379 | -1.44302143 |
| Olfr916 | 0.00222 | -1.1481805 |
| Zfp637 | 0.66799 | -0.17245412 |
| Eppk1 | 0.38214 | -0.40414799 |
| Gm13030 | 0.49087 | -0.31559846 |
| Olfr804 | 0.00144 | -1.27165604 |
| Olfr904 | 0.12066 | -3.15539282 |
| 1700039E15Rik | 0.00175 | -1.30132437 |
| Hcrt | 0.00975 | -1.36238398 |
| Tmem254a | 0.03947 | -0.57326265 |
| Rd3l | 0.2204 | -0.77120736 |
| Trip12 | 0.06878 | 0.42455909 |
| Ckap5 | 0.16773 | 0.2330531 |
| Tm2d3 | 0.00164 | -0.96846871 |
| Rasgrp1 | 0.54866 | -0.2477387 |
| Cisd3 | 0.0051 | -0.8320781 |
| Akap7 | 0.19921 | 0.31067216 |
| Gm7936 | 0.00249 | -0.87526111 |
| Tmem5 | 0.03983 | -1.29741461 |
| Dear1 | 0.37546 | 0.26724835 |
| Mpg | 0.26357 | -0.31798006 |
| Rps15a | 0.00497 | -0.70513603 |
| Tmem63b | 0.00144 | -1.25214776 |
| Myo19 | 0.44719 | -0.35018212 |
| LOC105243194 | 0.31173 | 0.19013527 |
| Gm4718 | 0.00144 | -1.27165604 |
| Rpl23a | 0.22844 | -0.30265131 |
| Rpl23a | 0.15397 | -0.34636345 |
| Rgs2 | 0.01238 | 0.60431989 |
| Vmn1r9 | 0.00144 | -1.30737044 |
| Vmn1r6 | 0.00149 | -1.25408 |
| Tank | 0.00144 | 1.71376688 |
| Psg17 | 0.00144 | -1.26560808 |
| Olfr1065 | 0.00144 | -1.34484129 |
| Nans | 0.00144 | -0.96282074 |
| Kif20b | 0.00144 | 2.65853076 |
| Shb | 0.34008 | -0.62360828 |
| Shb | 0.00676 | -0.92106904 |
| LOC102639950 | 0.08182 | 0.57415062 |
| Tcf7 | 0.00145 | -1.22193674 |
| Zdhhc21 | 0.0381 | 0.84274177 |
| Ahr | 0.01353 | 1.3911226 |
| Filip1l | 0.0511 | 1.09747529 |
| Slc1a4 | 0.03857 | -0.58543929 |
| Plekha7 | 0.89059 | -0.09071357 |
| Sprr2e | 0.00144 | -1.29049509 |
| Trim6 | 0.16054 | -0.39346286 |
| Trnt1 | 0.05004 | 0.40156162 |
| Milr1 | 0.00824 | -0.67630347 |
| Milr1 | 0.38164 | -0.19714781 |
| Zfp941 | 0.71764 | -0.29878664 |
| Irf4 | 0.90551 | -0.0507451 |
| Rfng | 0.00398 | -0.77643146 |
| Tas2r104 | 0.0093 | -0.80890014 |
| Gm8505 | 0.00166 | -1.34462115 |
| Rnase12 | 0.00144 | -1.27165604 |
| Scn10a | 0.83234 | -0.09964073 |
| Scn11a | 0.7439 | -0.11455096 |
| Adora2a | 0.00144 | -1.61956248 |
| Camk2b | 0.00959 | -0.69896499 |
| Dot1l | 0.1461 | -0.30035459 |
| Dot1l | 0.2151 | -0.21878236 |
| Gba2 | 0.0163 | -0.50768508 |
| Ache | 0.05267 | -0.75965928 |
| Ache | 0.08326 | -0.82440596 |
| Ppp1r14b | 0.00455 | -1.06468839 |
| Nipal3 | 0.00761 | -0.62709294 |
| Rps3 | 0.00177 | -1.06949291 |
| Csmd3 | 0.00144 | -1.27699683 |
| Hist1h2bg | 0.30762 | -0.52174537 |
| Hist1h2be | 0.70403 | -0.07642753 |
| Hist1h2bc | 0.10558 | -0.47147729 |
| BC048644 | 0.00144 | -1.20579466 |
| Gm9951 | 0.00144 | -1.29049509 |
| Vash1 | 0.00509 | -0.75784436 |
| Hmgcs2 | 0.00144 | -1.59562102 |
| Gm4117 | 0.03679 | -0.6551336 |
| Armcx6 | 0.2051 | 0.29484093 |
| Kcnip3 | 0.04589 | -0.50019397 |
| A430078G23Rik | 0.56786 | -0.14029756 |
| Gjc3 | 0.02612 | -0.91585234 |
| Ccr6 | 0.14787 | -1.05140304 |
| Hps5 | 0.02818 | 0.4525973 |
| Dao | 0.01579 | -1.10278386 |
| AV356131 | 0.00402 | -0.9362 |
| Gm7160 | 0.68189 | 0.15237692 |
| Tuba1c | 0.01219 | -0.65289756 |
| Adamts3 | 0.77765 | 0.24428707 |
| Epb4.1l4b | 0.19464 | -0.27314272 |
| Ubfd1 | 0.0052 | -0.66995759 |
| Nck1 | 0.00144 | 1.3835228 |
| Gm5936 | 0.08013 | -0.75281895 |
| Gm8709 | 0.68395 | -0.23631447 |
| Mtch2 | 0.476 | -0.16502687 |
| Tlcd1 | 0.97569 | 0.00949145 |
| Phkg2 | 0.00617 | -0.62770899 |
| LOC102639117 | 0.0017 | -1.24950944 |
| D830030K20Rik | 0.01175 | -0.62829175 |
| Ephb6 | 0.59494 | -0.32245499 |
| Lgmn | 0.27654 | -0.29927381 |
| Art4 | 0.01368 | -0.56001595 |
| Vmn1r-ps79 | 0.00145 | -1.29082685 |
| Gnptab | 0.04842 | 0.43462348 |
| Hbb-b1 | 0.31501 | -0.39686879 |
| Arcn1 | 0.01599 | 0.56466903 |
| Vmn2r40 | 0.03669 | -0.94779588 |
| Dgcr8 | 0.00978 | 0.54585571 |
| Dgcr2 | 0.00865 | -0.83441299 |
| Tmem255b | 0.00144 | -1.29020301 |
| Fam78b | 0.04423 | 0.45103522 |
| Slc22a13b-ps | 0.04108 | 0.9724037 |
| Smad5 | 0.06646 | 0.34046695 |
| Gm1110 | 0.00144 | -1.28438799 |
| H2-M10.2 | 0.00144 | -1.31495282 |
| Cnbd1 | 0.00144 | -1.25118919 |
| Hnrnpk | 0.00205 | 0.90610178 |
| Fam81b | 0.04784 | -2.07463506 |
| Col28a1 | 0.83845 | -0.08865063 |
| Vmn2r66 | 0.00144 | -1.28116048 |
| Mrgpra6 | 0.00145 | -1.27532353 |
| Nol9 | 0.4279 | -0.42114638 |
| Ttc41 | 0.41494 | -0.41566342 |
| Ttc41 | 0.0072 | 1.12679146 |
| Pde4b | 0.31929 | 0.55294413 |
| Fat1 | 0.0951 | -0.2932766 |
| Rab5b | 0.00368 | -1.20273397 |
| Slc18a1 | 0.51652 | 0.18431737 |
| Kif21a | 0.01765 | 0.58151957 |
| Gm9855 | 0.00371 | -0.81859642 |
| Tdg | 0.05278 | -0.3512334 |
| Samd10 | 0.20626 | 0.37295027 |
| Vmn2r96 | 0.0017 | -1.24950944 |
| Pbx3 | 0.54521 | 0.15563093 |
| Gm15421 | 0.96305 | 0.00964105 |
| Aurkaip1 | 0.00248 | -1.2051546 |
| Tssk5 | 0.02754 | -0.66163255 |
| Ggn | 0.01345 | -0.81379798 |
| Tssk6 | 0.00282 | -1.26150133 |
| Sult2a6 | 0.0611 | -0.7586473 |
| Gpr137b | 0.00341 | -0.79099409 |
| Gm6484 | 0.00749 | -0.67491819 |
| Gm12886 | 0.00144 | -1.28116048 |
| 1810013D15Rik | 0.32097 | 0.60007829 |
| Rasd2 | 0.10698 | -0.29555677 |
| Olfr623 | 0.00145 | -1.2388121 |
| Olfr323 | 0.00935 | -1.25832135 |
| Kansl2 | 0.02133 | 0.49335946 |
| Kansl2 | 0.16509 | -1.25601728 |
| Cabyr | 0.33286 | 0.3446128 |
| Myl4 | 0.13487 | -2.59201969 |
| Mcmbp | 0.15237 | -0.27050714 |
| Flad1 | 0.05444 | -0.36414837 |
| Gm10690 | 0.00144 | -1.28116048 |
| Gm5523 | 0.00415 | -0.72953433 |
| Kcna2 | 0.83002 | -0.08000115 |
| Kcna3 | 0.0044 | -2.03440393 |
| Arfgap3 | 0.02366 | -0.44604965 |
| Kcna1 | 0.79589 | 0.14648241 |
| Wnt1 | 0.0278 | -0.49849502 |
| Nkain4 | 0.00176 | -1.58895771 |
| Gm35287 | 0.09372 | -0.3249624 |
| Ndufs3 | 0.03087 | -0.58830679 |
| Bglap2 | 0.08706 | -1.14416262 |
| Bglap | 0.3123 | -0.32091777 |
| Bglap | 0.00144 | -1.28898079 |
| LOC102633794 | 0.00222 | -1.08458553 |
| Slc9a2 | 0.09059 | 0.81297756 |
| Trim63 | 0.64739 | 0.19758669 |
| Taar3 | 0.10208 | -2.70356371 |
| Taar5 | 0.00167 | -1.09376204 |
| Vmn2r59 | 0.27833 | -0.72282329 |
| Tal1 | 0.58223 | -0.13824277 |
| Adcy9 | 0.00299 | -0.84036577 |
| Tcf24 | 0.50357 | -0.17882133 |
| Gm12349 | 0.00774 | -0.97229754 |
| Gm2042 | 0.00749 | -1.27796736 |
| Pcdha8 | 0.0467 | 0.90748867 |
| Hist1h4d | 0.00144 | -1.39709013 |
| Olfr1264 | 0.00144 | -1.28116048 |
| Nmnat3 | 0.34278 | -0.33974775 |
| Scn2a1 | 0.03976 | -0.6613489 |
| Skor1 | 0.37413 | 0.44869672 |
| Ankrd52 | 0.02696 | -0.5702411 |
| Zfhx3 | 0.00593 | 1.24670216 |
| N28178 | 0.39714 | 0.59093019 |
| Zfp114 | 0.02252 | 0.85090538 |
| Herc2 | 0.47016 | 0.22060213 |
| Gapdh | 0.00265 | -0.79944856 |
| Knstrn | 0.00144 | -1.89993188 |
| Grxcr1 | 0.02197 | -0.99788027 |
| Arid4b | 0.28472 | 0.57397838 |
| Sstr3 | 0.01892 | -1.76526189 |
| Gm15023 | 0.00158 | -1.2446827 |
| Mbnl3 | 0.00144 | 2.53009082 |
| Trappc6a | 0.00183 | -1.00379302 |
| 4930432K21Rik | 0.00144 | -0.9969156 |
| Syngr2 | 0.00474 | -0.81610153 |
| Zfp410 | 0.02017 | 0.61152575 |
| Vmn1r175 | 0.00149 | -1.2703832 |
| Dnah2 | 0.74188 | -0.29228148 |
| Gch1 | 0.01239 | -0.85216564 |
| Cenpk | 0.00504 | 1.83698731 |
| Il21 | 0.00144 | -1.29085005 |
| C5ar2 | 0.05273 | -0.41173919 |
| Glrx2 | 0.01619 | -0.63099308 |
| Wfikkn1 | 0.8779 | -0.07746353 |
| Ndufs2 | 0.02144 | -0.60696318 |
| Gm11110 | 0.97868 | 0.01068421 |
| Map3k6 | 0.0105 | 0.80394615 |
| Stxbp5l | 0.00769 | -1.92360968 |
| Stxbp5l | 0.00144 | -1.30713006 |
| Stxbp5l | 0.02324 | -0.96462022 |
| Trpa1 | 0.11118 | -2.82977539 |
| Olfr224 | 0.86219 | -0.05043644 |
| Lrrc18 | 0.93078 | -0.03658242 |
| Ccr10 | 0.18597 | -1.99610592 |
| Ppp2r5c | 0.13877 | 0.25135509 |
| Atf5 | 0.00144 | -1.26054465 |
| Mbd1 | 0.11617 | 0.55636367 |
| Tbc1d31 | 0.0316 | 0.59611253 |
| Tbc1d31 | 0.17963 | -0.51557983 |
| Wdr62 | 0.02108 | -0.61813422 |
| Klrb1-ps1 | 0.02665 | -1.21078685 |
| 2310007L24Rik | 0.00848 | -0.69335269 |
| Adrb3 | 0.46961 | 0.17308283 |
| Lhx4 | 0.01427 | -0.97407952 |
| Mmp1b | 0.08493 | -1.03231216 |
| Ralgapa2 | 0.07652 | 0.6699432 |
| 1700104L18Rik | 0.00415 | -1.06195552 |
| Pde2a | 0.01175 | -0.63415967 |
| Frem3 | 0.07364 | 0.47429957 |
| Nop56 | 0.42246 | -0.13534508 |
| Zfp7 | 0.11493 | 0.89809184 |
| Foxp1 | 0.26358 | 0.25638355 |
| Foxp1 | 0.00145 | -1.2388121 |
| Foxp1 | 0.07171 | -0.5906184 |
| Nrxn3 | 0.16692 | 0.78913676 |
| Nrxn3 | 0.03948 | -0.98863362 |
| Tmed5 | 0.01276 | 1.08179526 |
| Tmed5 | 0.07183 | -1.73548796 |
| Tmed5 | 0.82998 | 0.07522673 |
| Acin1 | 0.00383 | -1.30274408 |
| Pdlim7 | 0.70269 | -0.08696843 |
| Arhgap39 | 0.00891 | -0.62011086 |
| Defb47 | 0.00962 | -1.84263399 |
| Hscb | 0.38556 | -0.21504777 |
| Nlrp4c | 0.00293 | -1.4329383 |
| Ercc8 | 0.04439 | -2.12157278 |
| Mrap2 | 0.00144 | -1.29085005 |
| Tfb2m | 0.48251 | 0.1379699 |
| Zfp335os | 0.84328 | -0.06971546 |
| Asic1 | 0.29788 | -0.72802128 |
| Edc4 | 0.00289 | -0.81493217 |
| Serpina3f | 0.9721 | -0.02180474 |
| B020011L13Rik | 0.04657 | -0.98967077 |
| Rpl30 | 0.00936 | -0.59180013 |
| Ppp1r1c | 0.01663 | -1.64619982 |
| Ppp1r1c | 0.00145 | -1.63409916 |
| Nphp1 | 0.00284 | -1.75774026 |
| Pisd-ps2 | 0.0046 | -1.02483124 |
| Col5a2 | 0.01059 | 1.06407705 |
| LOC102640586 | 0.13027 | -0.47154253 |
| Rsl24d1 | 0.30702 | 0.23855752 |
| P2rx4 | 0.00227 | -0.94949741 |
| Mrpl27 | 0.98998 | 0.00455554 |
| Utf1 | 0.21872 | 0.47579757 |
| Gm10408 | 0.75492 | -0.09249271 |
| D830030K20Rik | 0.71944 | 0.32895353 |
| Gm3317 | 0.00144 | 2.30121471 |
| Ttc33 | 0.45658 | 0.17251232 |
| Kctd9 | 0.65756 | 0.08379593 |
| C87436 | 0.70477 | 0.06129444 |
| Casc1 | 0.10511 | 0.31715128 |
| Olfr1391 | 0.00144 | -1.28116048 |
| Pld1 | 0.12249 | 0.35044682 |
| Inadl | 0.01312 | -0.89789868 |
| Inadl | 0.31383 | 0.4152166 |
| Gm10081 | 0.00144 | -1.4803666 |
| Gm12302 | 0.00808 | -1.10247618 |
| Gm6420 | 0.02909 | -1.3490905 |
| Olfr66 | 0.00182 | -1.19015431 |
| Plin5 | 0.1048 | -0.75537474 |
| Hoxc13 | 0.00788 | -1.56198005 |
| Gm17727 | 0.03132 | -0.97953315 |
| Olfr448 | 0.00144 | -1.30713006 |
| Defa23 | 0.03565 | -0.87705171 |
| Gm5767 | 0.00587 | -1.49868863 |
| Bhmt | 0.00257 | -1.1333102 |
| Bhmt | 0.17115 | -0.82474921 |
| Mrpl30 | 0.85139 | -0.0561704 |
| Clec4a4 | 0.08035 | -2.43517941 |
| Gm3258 | 0.20673 | -0.3146449 |
| Gtf2ird1 | 0.39308 | -0.19574012 |
| Spata18 | 0.65229 | -0.26972077 |
| Hgs | 0.00983 | -0.58420827 |
| Gbp4 | 0.00383 | 2.08009663 |
| Gm6653 | 0.0024 | -1.33528303 |
| D630003M21Rik | 0.00427 | -2.32815847 |
| Cdh12 | 0.78933 | 0.11441287 |
| Gm5593 | 0.16923 | -1.37510006 |
| Isg15 | 0.01428 | -0.61319089 |
| Zfp316 | 0.0021 | -1.01460246 |
| Zfp366 | 0.97975 | -0.0090039 |
| Arid1b | 0.00254 | -0.82123402 |
| Slc27a4 | 0.19011 | -0.26190998 |
| Gm831 | 0.53811 | -0.14027476 |
| Slc30a4 | 0.00243 | 1.02396717 |
| Prdx6 | 0.06828 | -0.51021915 |
| Mrpl9 | 0.20132 | -1.9276506 |
| Irf2bpl | 0.00144 | -1.64544466 |
| Fam170b | 0.5091 | -0.35734014 |
| Npy1r | 0.38741 | 0.49882652 |
| Gm9876 | 0.28988 | -0.33699251 |
| Psmc3 | 0.00282 | -0.79130322 |
| Oosp3 | 0.00144 | -1.22925777 |
| Golt1a | 0.02676 | -0.84784546 |
| Cox15 | 0.1115 | -0.40488391 |
| Mknk2 | 0.00145 | -1.05495283 |
| Twf2 | 0.31188 | -0.49219304 |
| Cxcr1 | 0.00144 | -1.32313902 |
| Slc25a18 | 0.12364 | 0.34159168 |
| Olfr49 | 0.00144 | -1.49973687 |
| Ppp1r2 | 0.5462 | -0.11786283 |
| Tor1aip2 | 0.04227 | 0.57969025 |
| Ccr8 | 0.84578 | -0.06053499 |
| Mkx | 0.16275 | -2.67649299 |
| Sema4d | 0.00414 | -0.80472729 |
| Gm4311 | 0.00144 | -1.27454685 |
| LOC102633321 | 0.0902 | -1.51486708 |
| Olfr351 | 0.00411 | -1.11066059 |
| Pnrc1 | 0.61425 | 0.1688 |
| Fbxl4 | 0.00667 | 0.73588383 |
| Zfp28 | 0.08268 | 0.47542699 |
| Inca1 | 0.12279 | 0.48685022 |
| Gm13201 | 0.34337 | -0.57964237 |
| Rpl36a | 0.0115 | -0.55162034 |
| Abca8a | 0.00151 | -1.30227649 |
| Mme | 0.29863 | 0.35056355 |
| Acvr1 | 0.09863 | 0.35379017 |
| Eaf2 | 0.61676 | 0.10793379 |
| Olfr130 | 0.00145 | -1.2388121 |
| Prorsd1 | 0.05969 | 0.40056286 |
| Olfml3 | 0.65164 | -0.1041201 |
| Lrrtm4 | 0.00144 | -1.28898079 |
| Eif1 | 0.55596 | -0.15491011 |
| Nxf7 | 0.00144 | -1.31014844 |
| B3gntl1 | 0.18697 | -0.88167463 |
| Armc9 | 0.12727 | -1.77484608 |
| Spag9 | 0.00144 | 1.4367745 |
| Spag7 | 0.03433 | -0.46185948 |
| Stambpl1 | 0.09875 | -0.28877792 |
| Zfp423 | 0.01496 | -0.7093557 |
| Yif1b | 0.01499 | -0.93209052 |
| Nprl3 | 0.20138 | -0.36733475 |
| Rgs20 | 0.09021 | -0.42386353 |
| Rgs20 | 0.6002 | -0.35693778 |
| Rdh12 | 0.08773 | -0.36390393 |
| Lamp2 | 0.20541 | 0.21930999 |
| Gm10639 | 0.36856 | -0.19214702 |
| Cc2d1b | 0.06049 | -0.38668654 |
| Olfr1107 | 0.00216 | -1.37827373 |
| Apitd1 | 0.0063 | -1.1502822 |
| Zfp112 | 0.65452 | -0.28797948 |
| Eva1c | 0.26438 | -0.32155234 |
| Rpl10a | 0.01019 | -0.63566825 |
| Dgat2l6 | 0.00158 | -1.2446827 |
| Adora1 | 0.02162 | -0.58559389 |
| Dscc1 | 0.10905 | -0.39550899 |
| Olfr155 | 0.75613 | -0.32361788 |
| Gnat2 | 0.10534 | 0.44996542 |
| Vamp5 | 0.4003 | 0.26549123 |
| Gm10682 | 0.01456 | -1.03105883 |
| Vta1 | 0.03071 | 0.85949401 |
| Cd55 | 0.00208 | 2.07608722 |
| Zhx3 | 0.48026 | -0.15640258 |
| Zhx3 | 0.92683 | -0.05978089 |
| Zhx3 | 0.27649 | -0.19014602 |
| H2afb2 | 0.01687 | -1.47832646 |
| Olfr328 | 0.00968 | -1.13227942 |
| Vmn2r7 | 0.05855 | -0.75626675 |
| L1td1 | 0.14742 | 0.72687939 |
| Fgf8 | 0.07423 | -0.83557911 |
| Stxbp3-ps | 0.64663 | 0.0967239 |
| Gpbar1 | 0.13884 | -0.29130166 |
| Mrps25 | 0.00247 | -0.80127554 |
| Gm10403 | 0.08621 | 0.96483067 |
| LOC102642456 | 0.26174 | -0.69895385 |
| Ppfia3 | 0.07818 | -1.25790184 |
| Aftph | 0.06177 | 0.62024109 |
| Ramp3 | 0.00144 | -1.26541537 |
| Ramp2 | 0.00184 | -0.95446915 |
| Try4 | 0.00715 | -1.53617241 |
| LOC102641266 | 0.0263 | -0.83541749 |
| Aldh7a1 | 0.62281 | -0.16747793 |
| Aldh3a1 | 0.02455 | -0.5232435 |
| Pcmt1 | 0.43471 | 0.1868699 |
| Tomm7 | 0.00589 | -0.7569244 |
| Ptchd3 | 0.85488 | 0.06193635 |
| LOC102634401 | 0.00144 | -1.25118919 |
| Slc5a11 | 0.51007 | -0.20482935 |
| Olfr785 | 0.58758 | -0.44547589 |
| LOC102642191 | 0.02312 | -0.48611559 |
| 1700019J19Rik | 0.01082 | -1.21582786 |
| Alpk1 | 0.0024 | 1.30612874 |
| Lao1 | 0.83765 | -0.04967173 |
| Smad3 | 0.04722 | -0.55165991 |
| Olfr1219 | 0.00144 | -1.27454685 |
| Dnah7b | 0.01584 | 1.25982622 |
| Ccdc158 | 0.00144 | -1.31518407 |
| Ptgds | 0.96404 | -0.02528152 |
| Arnt | 0.50759 | -0.10492676 |
| Fjx1 | 0.11325 | -0.56842815 |
| Tma7 | 0.22463 | -0.19740397 |
| Sprtn | 0.04523 | -0.96818714 |
| Sprtn | 0.01215 | -0.68266559 |
| Gm19410 | 0.00144 | -1.30017714 |
| Nif3l1 | 0.33046 | -0.47881883 |
| Olfr1402 | 0.01357 | -1.12920153 |
| Usp48 | 0.15298 | 0.35098555 |
| Cngb1 | 0.02186 | -0.73426002 |
| Auh | 0.0548 | -1.41904437 |
| Auh | 0.45013 | -0.30262655 |
| Pygo2 | 0.00229 | -0.87791197 |
| Dnm2 | 0.23263 | -0.2906352 |
| Gpha2 | 0.01945 | -0.87511821 |
| Gm12216 | 0.00262 | -0.84859515 |
| Gm12216 | 0.67542 | 0.15579414 |
| Cass4 | 0.01727 | 0.7221055 |
| Cass4 | 0.00564 | -1.16073293 |
| L1cam | 0.00382 | -1.3145374 |
| Mtif2 | 0.07745 | 0.6031864 |
| Gm10419 | 0.00144 | -1.27165604 |
| Epha8 | 0.00332 | -1.12694059 |
| Gas2l3 | 0.03651 | -1.87494705 |
| Epha5 | 0.00144 | -1.29049509 |
| Asb12 | 0.60295 | -0.12133783 |
| Gm32539 | 0.00144 | -1.27165604 |
| Tcp11l1 | 0.22634 | -0.41148842 |
| Lrba | 0.79163 | -0.06454714 |
| Dnmbp | 0.45879 | -0.23529281 |
| Defb30 | 0.00773 | -1.58180512 |
| Defb30 | 0.12429 | -0.76148446 |
| Defb30 | 0.00907 | -1.06952887 |
| Gm5433 | 0.30859 | 0.31510344 |
| Kif2c | 0.11864 | -0.78861507 |
| LOC102634459 | 0.00144 | -1.27165604 |
| Mapt | 0.00144 | -1.03202771 |
| Nme1 | 0.05096 | -0.57426761 |
| Zfp148 | 0.96843 | 0.01071373 |
| Htr7 | 0.00914 | 1.48090906 |
| Cyp2ab1 | 0.00144 | -1.44721599 |
| Mdga2 | 0.34288 | -0.30566102 |
| Zscan22 | 0.57294 | -0.15245185 |
| Gm10696 | 0.00144 | -1.31518407 |
| Cul9 | 0.03103 | -0.57967164 |
| Hist1h2ag | 0.00407 | -1.26562896 |
| Car1 | 0.00144 | -1.27165604 |
| Cers5 | 0.08899 | -0.31708026 |
| F730043M19Rik | 0.05628 | -0.91440819 |
| Atxn7 | 0.032 | -0.47901552 |
| Gm10461 | 0.20071 | -0.76346078 |
| Vmn2r85 | 0.00179 | -1.14160071 |
| Gm20783 | 0.00144 | -1.30713006 |
| Usp15 | 0.00144 | 1.6007895 |
| 4933400A11Rik | 0.00206 | 1.21141447 |
| Ccl19 | 0.00144 | -1.30352622 |
| Klc4 | 0.01369 | -0.72125659 |
| Msantd4 | 0.02253 | 0.50283743 |
| Lrp2 | 0.00144 | -1.29746368 |
| Gpr37 | 0.00144 | -1.28116048 |
| Gm36851 | 0.00144 | -1.27165604 |
| Slc24a3 | 0.04383 | -0.49926015 |
| Hmx2 | 0.02961 | -0.94782714 |
| Agbl1 | 0.00145 | -1.2388121 |
| Ect2 | 0.00144 | -1.22925777 |
| Scd3 | 0.02051 | -1.50567835 |
| Rarb | 0.00144 | -1.22098834 |
| Nol9 | 0.00675 | -0.65337645 |
| Apobec1 | 0.09636 | 0.65187322 |
| Gm4151 | 0.04552 | 1.10456144 |
| Slc12a2 | 0.0178 | 0.54816707 |
| Neil2 | 0.93592 | -0.02289713 |
| Gm12633 | 0.22327 | 0.43094683 |
| Stac2 | 0.78135 | -0.1698177 |
| Cars | 0.07268 | -0.32700311 |
| Wdr78 | 0.00144 | -1.28116048 |
| Tmem29 | 0.8803 | 0.05374722 |
| Ift80 | 0.0052 | 0.88750198 |
| Fgd4 | 0.06763 | -0.59330394 |
| Bdp1 | 0.00188 | 1.34286138 |
| 1700021F02Rik | 0.00144 | -1.22925777 |
| Gm8127 | 0.03958 | -1.1467723 |
| Mettl21b | 0.08834 | -0.68576538 |
| Irak1 | 0.03534 | 0.59566476 |
| Cd200r2 | 0.42511 | -0.61831656 |
| Olfr175-ps1 | 0.00144 | -1.29746368 |
| Cfhr2 | 0.20858 | -0.23069948 |
| C87414 | 0.00144 | -1.56519132 |
| Gm14085 | 0.00826 | 0.97409656 |
| 1700110I01Rik | 0.01638 | 0.93267111 |
| Sh2d3c | 0.02287 | -0.46682234 |
| Wfdc5 | 0.00144 | -1.37964577 |
| Lrrcc1 | 0.02148 | 1.60734319 |
| Galnt10 | 0.00144 | -1.24978056 |
| Asic5 | 0.03979 | -1.29553072 |
| LOC102632901 | 0.00601 | -1.09465341 |
| Pp2d1 | 0.00156 | -1.21312198 |
| Dnajc2 | 0.00144 | -1.25118919 |
| Adamtsl3 | 0.09356 | -1.4537796 |
| Gm32031 | 0.00237 | -1.12440287 |
| Slc12a7 | 0.01047 | -1.79970012 |
| Ttc21b | 0.00145 | 1.14583347 |
| Tnfaip2 | 0.95169 | 0.02756096 |
| Ube2d2b | 0.00145 | -1.26219879 |
| Gm10252 | 0.00144 | -1.63862646 |
| Spag16 | 0.01201 | -1.58979569 |
| Spag16 | 0.00588 | -1.30174695 |
| 2610318N02Rik | 0.57652 | -0.29903489 |
| Gm10466 | 0.00145 | -1.25646236 |
| Slc39a1 | 0.00516 | -0.97153256 |
| Itgb3bp | 0.05272 | 0.46394349 |
| Krt40 | 0.00144 | -1.27699683 |
| Izumo1 | 0.00195 | -1.59685509 |
| Olfr630 | 0.06351 | -2.25459848 |
| Shf | 0.00308 | -0.93339863 |
| Chmp6 | 0.62994 | -0.20116075 |
| C1rl | 0.49421 | -0.57795887 |
| 4931406P16Rik | 0.11028 | 0.31169372 |
| Tpbg | 0.21858 | -0.70162777 |
| Olfr401 | 0.00144 | -1.30229687 |
| Tmed2 | 0.896 | -0.02684767 |
| Col26a1 | 0.04076 | -0.61709185 |
| Arhgef9 | 0.00925 | 0.55869306 |
| Trim25 | 0.02711 | -0.51363728 |
| Trim25 | 0.11902 | 0.36973437 |
| Etos1 | 0.20527 | -0.40959053 |
| 1500017E21Rik | 0.04095 | 0.6040462 |
| 9030622O22Rik | 0.00519 | -1.02346311 |
| 9030622O22Rik | 0.00158 | -1.2446827 |
| Nkx1-1 | 0.0879 | -0.54671185 |
| L2hgdh | 0.07935 | -0.33914486 |
| Gm13769 | 0.00472 | -1.16801417 |
| Gmfg | 0.00668 | -0.73620283 |
| Olfr122 | 0.64307 | -0.65658488 |
| H2-T18 | 0.02191 | -1.8729064 |
| Slc44a5 | 0.00144 | -1.29049509 |
| Msh4 | 0.01662 | -1.02474722 |
| Gtpbp4 | 0.01691 | 1.29403675 |
| Arl4a | 0.32883 | -0.17517072 |
| Gm32762 | 0.00144 | -1.27730451 |
| LOC102643134 | 0.02074 | -1.082323 |
| Rabif | 0.01194 | 0.75938977 |
| C77080 | 0.00144 | -1.09506386 |
| Olfr1287 | 0.06902 | -1.04376346 |
| Zdhhc22 | 0.1766 | -0.5032093 |
| Mcc | 0.08991 | 0.50859083 |
| Gm6713 | 0.79442 | -0.11982324 |
| Ccdc34 | 0.04026 | -1.32346451 |
| Gda | 0.05343 | 0.76317269 |
| Creb5 | 0.0235 | -0.54167405 |
| Gm6219 | 0.89524 | -0.03553656 |
| Rbpms | 0.44949 | -0.15015153 |
| Strm | 0.05985 | -0.97100745 |
| Hs6st1 | 0.2346 | 0.47460257 |
| Cypt14 | 0.00144 | -1.26560808 |
| Gm5796 | 0.00144 | 2.74920101 |
| Tctex1d4 | 0.30892 | -0.51542734 |
| Camkv | 0.06305 | -1.28865822 |
| Gm5334 | 0.00144 | -1.39528885 |
| Zufsp | 0.00152 | 1.55860748 |
| Bptf | 0.95353 | 0.01206103 |
| Chaf1b | 0.07766 | -0.84372505 |
| Camk4 | 0.56007 | -0.29792866 |
| Gm11554 | 0.0319 | -0.86518464 |
| Idua | 0.19127 | 0.38817753 |
| Ifitm7 | 0.02846 | -0.51108857 |
| Tulp1 | 0.19373 | -0.63471242 |
| Cnep1r1 | 0.02895 | 0.59172219 |
| Cnep1r1 | 0.05207 | 0.91241448 |
| Mrc2 | 0.79344 | 0.10756075 |
| Eid1 | 0.02153 | -0.50926193 |
| Gjb1 | 0.03258 | -0.54435694 |
| Ereg | 0.2453 | -0.69422369 |
| Cntln | 0.03963 | 0.65166778 |
| Mreg | 0.3797 | -0.26052606 |
| Steap3 | 0.00335 | -0.88996856 |
| Gap43 | 0.91429 | -0.03650068 |
| Cd200r3 | 0.00144 | -1.30713006 |
| Fbxl19 | 0.04182 | -0.39594665 |
| Polr2c | 0.24221 | -0.1983984 |
| Nlrp1b | 0.96088 | -0.02349107 |
| Tprn | 0.00144 | -1.11367408 |
| Arhgdig | 0.00305 | -1.15876195 |
| Noto | 0.00144 | -1.20052149 |
| Elmod3 | 0.02415 | -0.65298436 |
| Gm13420 | 0.00144 | -1.57624836 |
| Calcoco2 | 0.03649 | -1.04055661 |
| Ssxb9 | 0.48165 | 0.21759933 |
| Tsc22d4 | 0.8399 | -0.1357107 |
| 4932429P05Rik | 0.00144 | -1.26560808 |
| Styk1 | 0.00144 | -1.29864962 |
| R3hdml | 0.00144 | -1.29049509 |
| Gm10706 | 0.06098 | -0.91531813 |
| Bcs1l | 0.14363 | -0.41840327 |
| Bcs1l | 0.13765 | -0.50613548 |
| Tmem240 | 0.01448 | -1.00503563 |
| AW554918 | 0.01031 | 1.70993074 |
| Inhbb | 0.27056 | -0.26328508 |
| Cdc42bpa | 0.0925 | 0.35942561 |
| Plce1 | 0.00144 | -1.5848881 |
| LOC102632770 | 0.64149 | -0.13305447 |
| Ank2 | 0.00621 | -0.9321927 |
| Gm34484 | 0.00801 | -1.74203917 |
| Rnf170 | 0.26773 | 0.17904457 |
| Rc3h1 | 0.24788 | 0.23816456 |
| Sftpb | 0.00144 | -1.33864718 |
| Myl6 | 0.00144 | -0.99844349 |
| Kdm1b | 0.01903 | 0.85629093 |
| Rfx8 | 0.12092 | 0.55265595 |
| Hax1 | 0.31797 | -0.22743843 |
| 5830473C10Rik | 0.00417 | -1.29746368 |
| Gnao1 | 0.82264 | 0.06775648 |
| Cyp20a1 | 0.09238 | 0.66450163 |
| Myo5b | 0.78442 | -0.08720021 |
| Myo5b | 0.36069 | -0.25328066 |
| Gm8256 | 0.00202 | -0.87345214 |
| Nanos2 | 0.78007 | -0.20131075 |
| Acot11 | 0.42644 | 0.21287111 |
| Neurog3 | 0.04379 | -1.15720701 |
| Zranb1 | 0.00385 | 0.77445433 |
| Lamtor3 | 0.03767 | 0.82154822 |
| 2310057B04Rik | 0.00594 | 1.91061792 |
| Pou6f2 | 0.00145 | -1.2388121 |
| Kalrn | 0.05824 | -0.80469325 |
| Kalrn | 0.04108 | -0.60365505 |
| Mrgpra5 | 0.01671 | -1.06195552 |
| Fbrsl1 | 0.02188 | -0.6475281 |
| Pml | 0.00767 | -0.6344092 |
| Gnrh1 | 0.00144 | 1.87487842 |
| Rgr | 0.00144 | -1.28898079 |
| Pgr | 0.00144 | -1.24811894 |
| Ccdc8 | 0.01513 | -1.04598882 |
| Ccdc65 | 0.9314 | -0.04262494 |
| Tcp10b | 0.28572 | 0.30789687 |
| Spata5l1 | 0.56194 | -0.13474807 |
| Slc8a2 | 0.01635 | -1.47211635 |
| 1600027J07Rik | 0.00144 | -1.27165604 |
| Try5 | 0.00144 | -1.28116048 |
| Pvrl3 | 0.05 | 0.79567437 |
| 1700028K03Rik | 0.00411 | -1.51500476 |
| 4930430A15Rik | 0.03689 | -2.81731076 |
| Dhx37 | 0.00725 | -0.6978884 |
| 1700012I11Rik | 0.61843 | -0.1835524 |
| Meaf6 | 0.02279 | -0.48150937 |
| Rara | 0.00794 | -0.65579665 |
| Rara | 0.00144 | -1.37860262 |
| AI182371 | 0.00145 | -1.22376446 |
| Ly6g | 0.023 | -0.71661858 |
| LOC105243282 | 0.32445 | -0.97384264 |
| Pkn3 | 0.058 | -0.47254289 |
| Ppp1r16a | 0.04182 | -0.77433237 |
| Ell3 | 0.65013 | 0.09100616 |
| Dusp22 | 0.01478 | -0.56772129 |
| Fdxacb1 | 0.05353 | -0.42556097 |
| Usp5 | 0.00783 | -0.83758253 |
| Pdf | 0.42571 | -0.1467447 |
| Synpo2 | 0.01416 | 1.09423307 |
| Gucy1a2 | 0.26342 | 0.55673831 |
| Abca4 | 0.71028 | 0.1564012 |
| Gm30556 | 0.01704 | -0.88285956 |
| Krtap6-1 | 0.00144 | -1.26153594 |
| Ncmap | 0.00396 | -0.81747203 |
| Ubash3b | 0.02695 | -0.9318359 |
| Atp1a3 | 0.02599 | -0.81635641 |
| Hist2h3b | 0.00316 | -0.98285582 |
| Grm1 | 0.00248 | 2.4396158 |
| Trp53tg5 | 0.00461 | -1.70389303 |
| Fat1 | 0.00221 | -1.10308723 |
| Srgn | 0.37627 | 0.23520302 |
| Ocm | 0.00272 | -1.09759392 |
| Phyhd1 | 0.12862 | -0.36164066 |
| Fgfr2 | 0.00377 | -1.06735844 |
| Ly75 | 0.04575 | -0.82849797 |
| Rabep1 | 0.09833 | 0.4149854 |
| Pgbd1 | 0.11765 | -0.77857796 |
| Slco1c1 | 0.02498 | 1.04721804 |
| Olfr62 | 0.00191 | -1.28116048 |
| Pcdhga10 | 0.47364 | 0.14739816 |
| Olfr263 | 0.00144 | -1.31286649 |
| Sox13 | 0.04498 | -0.42064118 |
| Sox11 | 0.08738 | -2.15443381 |
| Smim10l1 | 0.03087 | -0.85478238 |
| Kcnq1 | 0.26154 | -0.38844821 |
| Prdm12 | 0.00144 | -1.29085005 |
| Ralgps1 | 0.00837 | -0.65057933 |
| Ralgps1 | 0.06412 | -0.49380595 |
| Lrch1 | 0.00646 | 0.7954143 |
| Gm10367 | 0.00144 | -1.27165604 |
| Rapgef6 | 0.00144 | 1.39529157 |
| Gm14005 | 0.03774 | 0.69489568 |
| Fam171a1 | 0.17812 | -0.29185835 |
| Tmem150b | 0.0075 | -1.17039274 |
| Tmem150b | 0.00244 | -1.1876454 |
| Gm14135 | 0.04242 | -0.99315307 |
| Zeb2os | 0.02719 | -0.81891879 |
| Ms4a10 | 0.00144 | -1.36300167 |
| Ssbp1 | 0.00439 | -1.12780333 |
| Scgb2b15 | 0.01566 | -0.72700172 |
| Scgb2b27 | 0.00144 | -1.29085005 |
| Erp29 | 0.48527 | -0.24616224 |
| Zfp523 | 0.50043 | -0.29433622 |
| Olfr986 | 0.02749 | -1.70492782 |
| Olfr486 | 0.00144 | -1.26560808 |
| Olfr286 | 0.00144 | -1.29746368 |
| Erp27 | 0.61393 | -0.1073453 |
| Gm5447 | 0.06614 | 0.50615799 |
| Gm9694 | 0.0021 | -1.33841592 |
| Zfp644 | 0.00386 | -0.74223481 |
| Ulk4 | 0.06228 | -0.48629501 |
| Dnase1l3 | 0.50503 | -1.39533861 |
| Lhx6 | 0.04891 | -0.83038016 |
| Mroh5 | 0.10313 | -0.83930489 |
| Gzma | 0.00254 | -1.45322521 |
| Gm15800 | 0.12106 | -0.63251758 |
| Gm15800 | 0.14671 | -0.36148149 |
| Nrg2 | 0.3797 | -0.48985919 |
| Slc17a4 | 0.11234 | -0.77572158 |
| Maged2 | 0.17199 | -0.24733875 |
| Zfp428 | 0.09158 | -0.63900166 |
| Zfp408 | 0.1371 | -0.32214427 |
| Zscan25 | 0.94591 | 0.03661287 |
| Arhgef19 | 0.24587 | -0.46283992 |
| Pbxip1 | 0.00331 | -0.72288261 |
| 4930402F06Rik | 0.00144 | -1.27165604 |
| Nol4 | 0.43395 | -0.36049568 |
| Usp3 | 0.82531 | -0.04074006 |
| Pip4k2a | 0.45545 | 0.21389636 |
| Chl1 | 0.55165 | 0.28968027 |
| Eif2s3x | 0.00144 | -1.26560808 |
| Meig1 | 0.19961 | 0.39315013 |
| Gm5092 | 0.00144 | -1.47907491 |
| Pold3 | 0.1095 | 0.35570448 |
| Zfp599 | 0.08177 | -0.66652477 |
| Ddi2 | 0.13891 | -0.27392703 |
| Dmd | 0.00885 | -0.89981169 |
| Dennd4a | 0.06507 | 0.5847162 |
| Mmd | 0.33736 | -0.17897454 |
| Hfm1 | 0.19118 | -1.33765637 |
| Ufm1 | 0.04217 | -0.46842245 |
| Hapln3 | 0.69831 | 0.28090087 |
| Rps8 | 0.00414 | -0.69783794 |
| Gm5984 | 0.00144 | -1.31495282 |
| Gm2022 | 0.00144 | -1.30713006 |
| Mex3d | 0.05515 | -0.46381327 |
| Kcnk12 | 0.0015 | -1.20535468 |
| Zmpste24 | 0.25043 | 0.20758928 |
| Ghsr | 0.00145 | -1.26219879 |
| Htr3a | 0.00815 | -1.04929957 |
| Coil | 0.44519 | 0.16537984 |
| Apod | 0.00651 | -0.8296159 |
| Zc3h4 | 0.00913 | -0.75810914 |
| Myh7 | 0.24176 | -0.37736148 |
| Rnf111 | 0.02648 | 0.4544731 |
| Rnf151 | 0.03057 | -0.80142101 |
| LOC101055727 | 0.01816 | -0.70372982 |
| Rell2 | 0.12986 | -0.84963108 |
| Zbtb14 | 0.01124 | 0.60124341 |
| AL117821 | 0.07229 | 1.03479457 |
| D330045A20Rik | 0.02964 | -0.93601623 |
| Traf3 | 0.33924 | -0.15134434 |
| Traf2 | 0.00144 | -1.06341168 |
| Traf5 | 0.17639 | -0.27398552 |
| Rassf1 | 0.83894 | -0.04399811 |
| Ddb2 | 0.00293 | 1.0668348 |
| LOC102641663 | 0.84775 | 0.11738401 |
| Anxa9 | 0.4598 | 0.31308832 |
| Anxa4 | 0.06816 | -0.38192658 |
| Anxa1 | 0.06823 | 0.41890092 |
| Apoo | 0.05277 | 0.80215992 |
| Rhbdd3 | 0.00362 | -0.91256611 |
| Car9 | 0.76218 | 0.10730936 |
| Trim43b | 0.018 | 1.42461748 |
| Nppc | 0.00145 | -1.26219879 |
| Nup210 | 0.01346 | -0.58604038 |
| Mptx1 | 0.00144 | -1.38646478 |
| Tbc1d25 | 0.0031 | -0.90924169 |
| Slc45a3 | 0.00174 | -0.9172137 |
| Gm3858 | 0.11782 | -1.03833239 |
| Tgif1 | 0.22134 | -0.23694434 |
| Il18r1 | 0.00229 | -1.65700698 |
| Ccdc160 | 0.23314 | 0.71754341 |
| Ctbs | 0.02356 | 0.60641095 |
| Ermard | 0.00291 | 0.84474363 |
| BC053393 | 0.00144 | -1.25118919 |
| Adrm1 | 0.03875 | -0.48991361 |
| Fbxo2 | 0.00144 | -1.06873864 |
| Frrs1l | 0.1001 | -2.04089622 |
| Sec61a2 | 0.08388 | -0.66013988 |
| Serpina9 | 0.8254 | 0.07135454 |
| Camkmt | 0.44184 | 0.24911696 |
| Tmsb10 | 0.0028 | -0.92863879 |
| Taf11 | 0.5582 | -0.09030467 |
| Cyp4a10 | 0.0187 | -1.04692622 |
| Gpr157 | 0.17367 | -0.36046145 |
| Gpr137 | 0.01435 | -0.73248444 |
| Gm10375 | 0.00907 | -1.03958412 |
| BC061237 | 0.02506 | -1.03780074 |
| Raet1b | 0.05247 | -0.41900963 |
| Gm8005 | 0.11634 | -0.9169238 |
| 2210015D19Rik | 0.01134 | -0.86730553 |
| Gm10548 | 0.54628 | -0.23984951 |
| Hipk2 | 0.16054 | -0.35340832 |
| Hipk2 | 0.00866 | -0.61510218 |
| Gm4151 | 0.06037 | 0.77259262 |
| Nxnl1 | 0.00144 | -1.49418788 |
| Gm11716 | 0.00542 | -0.8610397 |
| Lamp3 | 0.00144 | 1.40484916 |
| Rassf2 | 0.61986 | 0.10626576 |
| Nudt3 | 0.062 | -0.52331719 |
| Nudt1 | 0.18395 | -0.40214689 |
| Fas | 0.00394 | 0.97195053 |
| Psmg1 | 0.61581 | -0.13418801 |
| Coa4 | 0.26114 | -0.44379283 |
| Krt222 | 0.26067 | 0.34665029 |
| 4933407I18Rik | 0.00144 | -1.28116048 |
| Bnipl | 0.39266 | -0.52097873 |
| Arhgap9 | 0.17028 | -0.36051431 |
| Vmn1r1 | 0.00144 | -1.27165604 |
| Srp54c | 0.00144 | 1.77334577 |
| Dppa3 | 0.00144 | -1.39629016 |
| Bnip3 | 0.10097 | 0.36274011 |
| Dio3 | 0.08502 | -1.19556008 |
| Casp4 | 0.00672 | 1.33868198 |
| Trim33 | 0.17906 | 0.33983636 |
| 4933402D24Rik | 0.00144 | -1.28116048 |
| Cep164 | 0.62373 | -0.08700367 |
| Cep164 | 0.00144 | -1.43195441 |
| ND4L | 0.04695 | -0.46860591 |
| Vmn1r71 | 0.00144 | -1.27699683 |
| Olfr871 | 0.00778 | -1.06398377 |
| 2610318M16Rik | 0.09581 | 0.37180425 |
| Ublcp1 | 0.39966 | 0.34901601 |
| Gm28905 | 0.00144 | -1.29051721 |
| Rpl6 | 0.02614 | -0.59163663 |
| Thoc2 | 0.40775 | 0.46249724 |
| Hrct1 | 0.00144 | -1.39566645 |
| Slc28a2 | 0.05045 | 0.64169007 |
| C330011M18Rik | 0.39437 | -0.16079308 |
| BC051142 | 0.44717 | 0.22008899 |
| BC051142 | 0.00144 | -1.26560808 |
| Hist1h4k | 0.00159 | -1.26740712 |
| Hist1h4k | 0.00144 | -1.34330021 |
| Kbtbd12 | 0.14758 | 0.40269106 |
| Kbtbd12 | 0.16379 | 0.22455806 |
| Gm6209 | 0.01241 | -0.63089787 |
| Ebna1bp2 | 0.88169 | -0.02802303 |
| Ebna1bp2 | 0.0568 | 0.36853482 |
| Larp4 | 0.02904 | 0.45585795 |
| Tpst2 | 0.00391 | -0.82177887 |
| Fshb | 0.05134 | -2.00116327 |
| Stk40 | 0.0294 | -0.54891011 |
| Map3k19 | 0.00144 | -1.31884045 |
| Rad21l | 0.00149 | -1.22582153 |
| Macrod2 | 0.84167 | -0.11356223 |
| Macrod2 | 0.08837 | -2.5744351 |
| Macrod2 | 0.04461 | 0.6890434 |
| Mtus1 | 0.04414 | 0.42325669 |
| Mtus1 | 0.05179 | 0.89614287 |
| Gm10576 | 0.0421 | -0.44472475 |
| Olfr1459 | 0.00144 | -1.25118919 |
| Pop5 | 0.00144 | -1.14261496 |
| Gm30378 | 0.00144 | -1.32766452 |
| Gm19410 | 0.04841 | 0.7576016 |
| Ptp4a1 | 0.00514 | -0.67292424 |
| Clca4c-ps | 0.00144 | -1.30017714 |
| Pilra | 0.15171 | -0.55623562 |
| Abat | 0.00145 | -1.2388121 |
| Thrap3 | 0.31127 | -0.38679476 |
| Ppdpf | 0.05062 | -0.53911202 |
| BC028471 | 0.00879 | -0.7453708 |
| Gm29455 | 0.00144 | -1.25118919 |
| Nat9 | 0.8329 | -0.09761824 |
| Nat9 | 0.22609 | -0.41342978 |
| H13 | 0.00814 | -0.77268249 |
| Vmn1r68 | 0.00144 | -1.28116048 |
| Plcd4 | 0.69642 | -0.09100082 |
| Plcd4 | 0.00176 | -1.42846741 |
| Kcnmb4 | 0.09543 | -0.8584298 |
| 1700034E13Rik | 0.00144 | -1.20281695 |
| Vmn1r34 | 0.00144 | -1.26560808 |
| Gm4340 | 0.00144 | -1.25118919 |
| Nwd1 | 0.00679 | -2.10229398 |
| Sntg2 | 0.00144 | -1.25118919 |
| Mrpl48 | 0.00424 | -0.72713072 |
| Cyb5r2 | 0.00667 | -1.02883272 |
| Tpm3 | 0.73466 | -0.07467712 |
| Tpm3 | 0.07088 | -0.47909159 |
| Kcna7 | 0.82118 | 0.05625428 |
| Nek10 | 0.00144 | -1.31495282 |
| Myh14 | 0.00265 | -0.82906185 |
| Mab21l3 | 0.00144 | -1.33632959 |
| Phrf1 | 0.0447 | -0.40956167 |
| Ilkap | 0.97528 | -0.01889871 |
| Mest | 0.26027 | 0.27740094 |
| Gm6634 | 0.00158 | -1.2446827 |
| Myo3a | 0.10222 | -0.58979569 |
| Rhox7a | 0.93532 | 0.03279741 |
| Xpo1 | 0.00285 | 0.93934766 |
| Adk | 0.07411 | 0.56150463 |
| Atf7 | 0.5642 | 0.15318507 |
| Atp6v1g2 | 0.00542 | -0.87173029 |
| Mtfr1l | 0.51356 | 0.37582836 |
| Ednrb | 0.00144 | 1.15994356 |
| Eif2s2 | 0.007 | 1.03936805 |
| Tmed5 | 0.78797 | -0.16995916 |
| Tbx22 | 0.34473 | -0.57773521 |
| Fkbp10 | 0.00181 | -1.19629164 |
| Col10a1 | 0.00145 | -1.2388121 |
| Cyp4f40 | 0.00144 | -1.25118919 |
| Sypl | 0.00579 | 0.73292259 |
| 1700020A23Rik | 0.01971 | -1.02659601 |
| Ncoa5 | 0.05279 | -0.40991524 |
| Wfdc13 | 0.26124 | -0.38781971 |
| 2900097C17Rik | 0.4448 | 0.12145661 |
| Olfr1440 | 0.01153 | -1.070189 |
| Rnf121 | 0.02656 | -0.52140716 |
| Gm6890 | 0.00144 | -1.26428512 |
| Vmn1r14 | 0.00144 | -1.29085005 |
| Atg4c | 0.00144 | 1.35059856 |
| Fam76a | 0.00812 | -0.62170758 |
| Ak4 | 0.233 | -0.24949521 |
| Rgs7 | 0.00189 | -1.12781694 |
| 9830147E19Rik | 0.00144 | 2.45742502 |
| 2900073C17Rik | 0.42066 | -0.41617828 |
| Msmp | 0.00848 | -1.25100272 |
| 4933431K14Rik | 0.72599 | -0.08222306 |
| Pole | 0.33555 | -0.32180659 |
| Slc23a2 | 0.19525 | -0.60093735 |
| Slc35g2 | 0.06575 | 0.39697332 |
| Tmem52 | 0.00208 | -1.13549041 |
| Plscr1 | 0.00688 | 0.63117108 |
| Vstm2b | 0.00245 | -1.46238524 |
| Olfr1388 | 0.07993 | -0.67368721 |
| Gapdh | 0.46327 | -0.30853064 |
| Ttll6 | 0.90651 | 0.17441963 |
| Sox5 | 0.00217 | -1.1219484 |
| Krtap4-1 | 0.67981 | 0.19089036 |
| Mtif3 | 0.32425 | 0.40687256 |
| Pcnp | 0.7327 | 0.16236794 |
| Usp14 | 0.16909 | 0.26868264 |
| Sgsm1 | 0.79206 | 0.06974902 |
| Sgsm1 | 0.00144 | -1.41551543 |
| BC094435 | 0.02651 | 0.5852991 |
| Ahcyl2 | 0.87076 | 0.05078068 |
| Ap3s2 | 0.09383 | -0.38168282 |
| Fbln5 | 0.01127 | -0.55460199 |
| Snrpd2 | 0.00144 | -1.12889242 |
| Htt | 0.06708 | -0.59567649 |
| Myh10 | 0.04107 | 0.48071876 |
| 4932414N04Rik | 0.00144 | -1.34334725 |
| Arrdc2 | 0.06986 | 0.63945102 |
| Tcn2 | 0.00309 | -0.83034742 |
| Lamtor5 | 0.06474 | -0.378626 |
| H2-T9 | 0.0089 | -0.91098232 |
| Tnnt1 | 0.03068 | 0.46628241 |
| Tnnt3 | 0.7866 | -0.13526906 |
| Olfr1312 | 0.83778 | 0.09472945 |
| Vmn1r87 | 0.00144 | -2.23495565 |
| Golga3 | 0.94007 | -0.01253026 |
| Olfr782 | 0.09694 | -2.6959388 |
| Cyp27b1 | 0.07713 | 0.58114772 |
| Synpr | 0.00145 | -1.22376446 |
| Uqcrb | 0.61691 | 0.11806104 |
| Hcn2 | 0.00144 | -1.2727898 |
| Ash2l | 0.28059 | -0.17247274 |
| Elk4 | 0.38903 | -0.31447216 |
| Gm6904 | 0.00144 | -1.38759431 |
| Mtss1 | 0.74865 | 0.06112225 |
| Fhod3 | 0.24477 | 0.38276201 |
| Cdc42ep4 | 0.01164 | -0.67805772 |
| Tenm1 | 0.00144 | -1.28898079 |
| Lypd6 | 0.00606 | -1.23130807 |
| Dnaja1 | 0.00344 | 1.35983057 |
| Otud3 | 0.16597 | -0.33105048 |
| Mthfsd | 0.34389 | -0.2216845 |
| Mroh1 | 0.31622 | -0.4275083 |
| Patz1 | 0.34637 | -0.30019262 |
| Tinf2 | 0.58854 | -0.12835084 |
| Ncoa2 | 0.00465 | 0.93485764 |
| Gm9963 | 0.27111 | 0.2295397 |
| Dnaja3 | 0.84437 | -0.05113441 |
| Myeov2 | 0.00165 | -1.21877022 |
| Zfp874a | 0.01539 | 1.19242541 |
| Hpcal4 | 0.0019 | -1.13608632 |
| Abhd12 | 0.98084 | 0.0128959 |
| Grhpr | 0.95438 | -0.04318987 |
| LOC102642448 | 0.44936 | -0.39943225 |
| Il4i1 | 0.00144 | -1.51991685 |
| Cp | 0.00144 | 2.29767471 |
| Gm8300 | 0.00145 | -1.22376446 |
| Rps11 | 0.00396 | -0.86127018 |
| Olfr373 | 0.17925 | 0.21698541 |
| Eno1b | 0.45629 | -0.31116583 |
| Clpsl2 | 0.00144 | -1.31202167 |
| Gm10498 | 0.07596 | -0.98313485 |
| D930015E06Rik | 0.09406 | -0.36190675 |
| Oprm1 | 0.15674 | -0.54791549 |
| Oprm1 | 0.00144 | -1.41803376 |
| Mapre3 | 0.01453 | -0.70715421 |
| Tex22 | 0.00383 | -1.0441501 |
| Unc93a | 0.00256 | -1.4220123 |
| Gps1 | 0.02638 | -0.67777238 |
| Gm6251 | 0.0043 | -0.80302045 |
| LOC102641600 | 0.03147 | -0.61382637 |
| Htr5a | 0.00144 | -1.31884045 |
| Gm4022 | 0.112 | -0.83236325 |
| Vmn2r3 | 0.1329 | -0.47895477 |
| Vmn2r3 | 0.00145 | -1.26219879 |
| Mipep | 0.32177 | 0.44514927 |
| 9530077C14Rik | 0.00144 | -1.14855125 |
| Ier3ip1 | 0.01043 | -0.5900422 |
| Car4 | 0.23984 | -0.32821729 |
| Gm35522 | 0.16483 | -0.45958348 |
| Dnhd1 | 0.27901 | 0.36772812 |
| Dnhd1 | 0.23834 | -0.58761474 |
| Ly6i | 0.01285 | -0.65287648 |
| Pecam1 | 0.31753 | 0.20115616 |
| Slc25a10 | 0.41266 | -0.37526397 |
| Tmem240 | 0.02913 | -0.56615001 |
| Dsg3 | 0.00144 | -1.27165604 |
| Atxn2 | 0.27958 | -0.22323406 |
| Col23a1 | 0.5001 | -0.23079253 |
| Cntf | 0.15227 | 0.58454251 |
| Ccdc121 | 0.00144 | -1.33632959 |
| Rgl3 | 0.12262 | -0.44888909 |
| Zfp651 | 0.05199 | -0.7230264 |
| Sp140 | 0.18413 | 0.29404515 |
| Sp140 | 0.15632 | 0.411397 |
| Olfr362 | 0.06075 | -0.42066031 |
| Uevld | 0.01925 | 0.65874209 |
| 1700062C10Rik | 0.01226 | -1.44496975 |
| Fam3a | 0.004 | -1.01619783 |
| Nfib | 0.10531 | 0.27563381 |
| Nfib | 0.2003 | 0.34148026 |
| Snrk | 0.03481 | -0.5802086 |
| Rad54l | 0.86052 | 0.07395507 |
| Gpr137c | 0.01981 | -1.15363697 |
| Nlrp1c-ps | 0.00144 | -1.27891018 |
| Bglap3 | 0.01343 | -1.72413476 |
| Wscd2 | 0.86436 | -0.08886408 |
| Gm10385 | 0.17834 | -1.07959131 |
| Oasl1 | 0.00351 | -1.11949552 |
| Recql4 | 0.80828 | -0.0804814 |
| Kif14 | 0.35411 | 0.30203866 |
| Gm12542 | 0.13011 | -2.66506728 |
| BC051408 | 0.00144 | -1.36780146 |
| Phldb3 | 0.11755 | -0.62095162 |
| Phldb3 | 0.1207 | -3.95027993 |
| Gm7609 | 0.66405 | -0.23588167 |
| Gm8580 | 0.00476 | -0.75497753 |
| Pan3 | 0.02247 | 0.8981872 |
| Kcnq2 | 0.00158 | -1.2446827 |
| Kcnq2 | 0.00144 | -1.27992547 |
| Kcnq2 | 0.00144 | -1.30017714 |
| Kcnq3 | 0.00144 | -1.52681567 |
| Kcnq1 | 0.31354 | -0.36063927 |
| Kcnq4 | 0.08301 | -0.34675314 |
| Olfr1124 | 0.01178 | -1.40363226 |
| Bcdin3d | 0.00383 | -0.69423244 |
| Uhmk1 | 0.62876 | 0.09811359 |
| Cyp39a1 | 0.39752 | 0.24619913 |
| Gm2061 | 0.50113 | 0.27257201 |
| Ube2h | 0.00461 | -0.69753552 |
| Zfat | 0.01965 | -0.48018468 |
| Gm13119 | 0.00144 | -1.29746368 |
| Igf1 | 0.01578 | -0.52462322 |
| Dnajb11 | 0.00144 | -1.30017714 |
| Dot1l | 0.40713 | -0.17928499 |
| Lamtor2 | 0.0277 | -0.45717402 |
| Anpep | 0.02924 | -0.54841253 |
| Fam83a | 0.97193 | 0.01303327 |
| 5330417C22Rik | 0.61673 | 0.09352857 |
| 1700037H04Rik | 0.12536 | -0.51996321 |
| BC029214 | 0.00246 | -0.8463849 |
| Gm561 | 0.15212 | -0.43481624 |
| Gm6287 | 0.17184 | -0.68980931 |
| Calr3 | 0.00184 | -1.26516318 |
| Olfr1261 | 0.00552 | -1.14048792 |
| Mapk8ip3 | 0.39552 | 0.18986083 |
| Stk38 | 0.01245 | 1.60732516 |
| Stk38 | 0.00144 | 1.70342692 |
| Meaf6 | 0.10053 | -0.33064421 |
| Lmbr1l | 0.03591 | -0.56616438 |
| Nfix | 0.13868 | -0.54907977 |
| Dennd4b | 0.00965 | -0.83902141 |
| Dnaja4 | 0.02422 | -0.50565759 |
| Pidd1 | 0.28053 | -0.30791137 |
| 5530601H04Rik | 0.17481 | 0.70476989 |
| Acsm4 | 0.00144 | -1.27165604 |
| Acsm1 | 0.16014 | -0.95997452 |
| Ankle1 | 0.96566 | -0.06196935 |
| Traf7 | 0.00625 | -1.20486733 |
| Pbsn | 0.04971 | -1.24191324 |
| E230008N13Rik | 0.00144 | -1.27699683 |
| Vmn1r33 | 0.00144 | -1.63681614 |
| Abhd12b | 0.86098 | -0.07414656 |
| Mia | 0.02993 | -0.76649778 |
| Fbxo44 | 0.73559 | 0.23356778 |
| Prdm10 | 0.80608 | -0.09601309 |
| Inf2 | 0.10337 | -0.33378692 |
| 2010007E15Rik | 0.01434 | 0.59712406 |
| Supt6 | 0.03184 | -0.86985317 |
| Chaf1b | 0.02071 | -0.61947383 |
| Gm4461 | 0.50717 | -0.25817077 |
| Ccdc40 | 0.00144 | -1.27699683 |
| Fam103a1 | 0.96562 | -0.00801744 |
| Gm34808 | 0.00144 | -1.31884045 |
| Gsx1 | 0.00144 | -1.26560808 |
| Vgf | 0.00149 | -1.22582153 |
| Vsx1 | 0.00547 | -1.18195423 |
| 4933416I08Rik | 0.00144 | -1.35019362 |
| Klk12 | 0.04825 | -0.73497447 |
| Klk13 | 0.00222 | -2.25658021 |
| Klk14 | 0.75781 | -0.26455824 |
| Slc8b1 | 0.88963 | 0.04167486 |
| 1700021P04Rik | 0.00144 | -1.30017714 |
| Anxa11 | 0.03623 | -1.25468296 |
| Sec31a | 0.39538 | 0.32441565 |
| Aff3 | 0.50355 | 0.16844064 |
| Zkscan6 | 0.1624 | -0.23194068 |
| Asb3 | 0.02724 | 1.13038905 |
| Acaca | 0.26445 | -0.51312442 |
| Hist1h2ac | 0.04722 | 0.70869168 |
| Hist1h2af | 0.00144 | -1.35938065 |
| Hist1h2ah | 0.00144 | -1.36837827 |
| Hist1h2ak | 0.00144 | -1.36095679 |
| Csrnp3 | 0.00242 | -1.8117455 |
| Hist1h2an | 0.0025 | -1.32299399 |
| Rab5b | 0.18677 | -0.27710036 |
| Fam78a | 0.19368 | -1.04024127 |
| B4galt6 | 0.00224 | 1.10412423 |
| Gm10814 | 0.30312 | -0.36736606 |
| Zbtb40 | 0.00693 | -0.6562042 |
| Ssbp3 | 0.00148 | -0.86029201 |
| Trim65 | 0.05702 | -0.65142863 |
| Tma16 | 0.31425 | -1.68837801 |
| Lrrtm2 | 0.10682 | -0.43886129 |
| Pigp | 0.52756 | -0.24965106 |
| Polr3f | 0.01239 | -1.1675496 |
| Nid1 | 0.04308 | -0.40398543 |
| Macrod2os2 | 0.20134 | -0.52620111 |
| Azin2 | 0.02059 | -1.04107898 |
| Klhl34 | 0.2632 | -0.48420856 |
| Gm8314 | 0.00144 | -1.20555712 |
| Rbm12b2 | 0.005 | 1.62444051 |
| Dus4l | 0.84534 | -0.0649135 |
| Rnf135 | 0.90957 | 0.02911677 |
| Olfr667 | 0.00144 | -1.28116048 |
| Olfr467 | 0.03181 | -1.04318857 |
| Rad1 | 0.32332 | 0.53054754 |
| Phgr1 | 0.90873 | -0.11973844 |
| Scfd1 | 0.13399 | 0.53012844 |
| Irf3 | 0.01926 | -0.50273467 |
| Robo2 | 0.8771 | -0.11214255 |
| BC023829 | 0.42666 | -0.19085685 |
| Vsig2 | 0.00729 | 1.17556814 |
| Atp13a1 | 0.00144 | -0.94622273 |
| Gm10781 | 0.06546 | -1.21336802 |
| 1700085B13Rik | 0.00144 | -1.20681653 |
| Zzz3 | 0.01796 | 0.54665024 |
| Tmem159 | 0.86652 | 0.05336303 |
| 2810410L24Rik | 0.70832 | -0.07377926 |
| Rbm12 | 0.18749 | 0.39486318 |
| Adam33 | 0.51143 | -0.18968044 |
| Adam33 | 0.46417 | 0.13094077 |
| Rps6kc1 | 0.22968 | 0.45841555 |
| Alas1 | 0.41483 | -0.36261225 |
| Dkk3 | 0.08582 | -1.06030623 |
| Hrk | 0.83133 | -0.11514978 |
| Fam179a | 0.02302 | 0.77786997 |
| Fam159a | 0.02673 | -1.01770746 |
| Lpin1 | 0.16466 | 0.86566416 |
| Lpin3 | 0.08209 | 0.43243026 |
| Gm10844 | 0.52639 | -0.38462579 |
| Pkp4 | 0.40985 | -0.14063577 |
| Cyp4f18 | 0.02666 | -0.54579706 |
| Radil | 0.14862 | -0.30167896 |
| Gm5477 | 0.0093 | -1.1233389 |
| Snf8 | 0.00144 | -0.97985991 |
| Gm2044 | 0.97507 | 0.02996899 |
| Hs3st1 | 0.01017 | -0.85674758 |
| E230025N22Rik | 0.24808 | -0.74728006 |
| Olfr1062 | 0.23653 | 0.36074036 |
| Mtus2 | 0.00256 | -0.85143099 |
| Mup-ps16 | 0.00144 | -1.30761071 |
| Gm10024 | 0.04762 | -2.15063496 |
| Gm10142 | 0.41482 | 0.34858421 |
| Gm10272 | 0.32771 | 0.30753867 |
| Necab3 | 0.14899 | -0.3484905 |
| 1700001J03Rik | 0.77906 | 0.32516043 |
| Sec16a | 0.03132 | -0.46775731 |
| Olfr1278 | 0.00164 | -1.63587059 |
| Vill | 0.06675 | -0.45541101 |
| Nkx2-4 | 0.01721 | -0.95370499 |
| Tatdn3 | 0.00144 | 0.9542373 |
| Haus7 | 0.21673 | 0.36704776 |
| Hps1 | 0.01383 | -0.93518796 |
| Hmgb1 | 0.01606 | -0.67647152 |
| Irak2 | 0.00144 | -1.30655127 |
| Irak2 | 0.73723 | -0.07842507 |
| Hsfy2 | 0.00494 | -1.04898329 |
| Fnbp1l | 0.00144 | 2.19169395 |
| Acly | 0.02167 | -0.82446568 |
| Wars | 0.83405 | -0.06128739 |
| Ezh1 | 0.05812 | -0.34397321 |
| Fam160b1 | 0.31301 | 0.19514665 |
| Rpl21 | 0.3942 | -0.15513294 |
| Cybb | 0.30159 | 0.42161732 |
| Olfr1193 | 0.00144 | -1.36377172 |
| Pwp2 | 0.00377 | -0.7996031 |
| Gm10258 | 0.00144 | -1.28898079 |
| Ak1 | 0.00767 | -1.01386246 |
| Aph1b | 0.04172 | -0.76640267 |
| Gm2270 | 0.04413 | -1.03617482 |
| Gm31105 | 0.00366 | -0.83812315 |
| Zfp711 | 0.02157 | 1.00594758 |
| Zfp791 | 0.01994 | -0.58375298 |
| Ndor1 | 0.4409 | -0.20781881 |
| Vmn1r237 | 0.12749 | -2.29114033 |
| Vmn1r234 | 0.06611 | -0.86038026 |
| Sav1 | 0.0033 | 0.94783709 |
| Olfr283 | 0.0015 | -1.91578254 |
| Kidins220 | 0.01559 | 0.67966269 |
| AU019990 | 0.00144 | -1.27165604 |
| Npepps | 0.64493 | -0.0700012 |
| D2Wsu81e | 0.00649 | -0.66640761 |
| Btbd11 | 0.7933 | 0.04053315 |
| 2610206C17Rik | 0.02955 | 0.80277238 |
| Six3 | 0.48143 | -0.61985966 |
| Six3 | 0.01679 | -0.5642189 |
| Crybb2 | 0.00144 | -1.59314691 |
| Ppt1 | 0.86321 | 0.03171469 |
| BC094916 | 0.00521 | -1.05862264 |
| Gm35144 | 0.43764 | 0.28253282 |
| Ttbk1 | 0.09439 | -0.40857977 |
| Tnfrsf9 | 0.25695 | -0.60288216 |
| 4930512J16Rik | 0.57087 | -0.81057208 |
| Vps13a | 0.88204 | 0.05695102 |
| Cdc20b | 0.00502 | -1.63417072 |
| Vwa5b1 | 0.00158 | -1.2446827 |
| B430319F04Rik | 0.00966 | -0.64883086 |
| Slc22a20 | 0.00144 | -1.20555712 |
| 9430015G10Rik | 0.44802 | 0.3629201 |
| Pramel5 | 0.00144 | -1.28116048 |
| 9330158H04Rik | 0.00144 | -1.28898079 |
| Prn | 0.17011 | -0.30393659 |
| Rps4x | 0.00508 | -0.66308388 |
| Slc44a1 | 0.21477 | -0.45176653 |
| Slc45a1 | 0.587 | -0.27746668 |
| Asb7 | 0.09274 | 0.54905282 |
| Gm9558 | 0.00144 | -1.5686593 |
| Olfr877 | 0.00144 | -1.28116048 |
| Olfr477 | 0.00441 | -0.98413508 |
| Spata17 | 0.06517 | -0.86145442 |
| Gpa33 | 0.00192 | -1.00153245 |
| Timeless | 0.32955 | 0.24251178 |
| Olfr1320 | 0.00144 | -1.27165604 |
| Dennd5b | 0.00144 | 1.36377405 |
| Raver1 | 0.07947 | -0.31274372 |
| Phf20 | 0.07346 | 0.46663677 |
| Phf23 | 0.34312 | -0.26247101 |
| Sh3bgrl3 | 0.00316 | -0.81038072 |
| Pglyrp1 | 0.18727 | -0.39569179 |
| Atp5g2 | 0.02865 | -0.90910267 |
| Fgd1 | 0.84541 | -0.10218602 |
| Vmn1r86 | 0.00144 | -1.29085005 |
| Rltpr | 0.02543 | -0.53888228 |
| BC052688 | 0.43156 | -0.14955356 |
| Mdm4 | 0.78863 | 0.08167317 |
| Gm13043 | 0.00144 | -1.30017714 |
| Glis1 | 0.00531 | -0.76724161 |
| Gnas | 0.04144 | 0.78320563 |
| Gnas | 0.17939 | -0.41753309 |
| Gnas | 0.88042 | -0.03787553 |
| Tet2 | 0.00145 | 1.03357769 |
| Dlx4 | 0.00144 | -1.27992547 |
| Gabbr2 | 0.34611 | -0.30803853 |
| Sptbn1 | 0.02956 | 0.73311058 |
| Slc28a1 | 0.01762 | -0.74465141 |
| Srcin1 | 0.30774 | -0.29212288 |
| Nxn | 0.01016 | -0.82864643 |
| C330024D21Rik | 0.00144 | -1.27165604 |
| Kcnj13 | 0.00144 | 2.71076614 |
| Gpr26 | 0.40612 | 0.37683794 |
| Gpr25 | 0.00273 | 1.57709311 |
| Gpr20 | 0.01384 | -0.95469532 |
| Tgif2lx1 | 0.89125 | -0.03368031 |
| Rbm41 | 0.67346 | 0.09915465 |
| Chka | 0.15194 | -0.41050249 |
| 4732456N10Rik | 0.00144 | -1.26560808 |
| Rpl26 | 0.00721 | -0.6787557 |
| Olfr827 | 0.04597 | -0.90881164 |
| Taf9 | 0.01031 | 0.61197968 |
| Zfp609 | 0.00144 | -1.20052149 |
| Qdpr | 0.66272 | 0.16176367 |
| Slc8a1 | 0.02283 | -0.7962303 |
| Olfr704 | 0.03925 | -0.95060786 |
| Rhox3g | 0.04121 | -2.14445472 |
| Cstf2 | 0.1073 | 0.30220287 |
| Gli1 | 0.0774 | -0.98902846 |
| Zbtb21 | 0.69892 | 0.25375571 |
| 5430416O09Rik | 0.4317 | 0.30461625 |
| Spata31d1b | 0.0027 | -0.94694483 |
| Poln | 0.6228 | -0.14263758 |
| 6430710C18Rik | 0.56956 | 0.63793667 |
| Gm31135 | 0.0017 | -1.60918938 |
| 4930570E03Rik | 0.00144 | -1.28898079 |
| Ttc30a2 | 0.88967 | 0.13338348 |
| Gm10674 | 0.31013 | -1.03592617 |
| Gm1971 | 0.02657 | -1.11664749 |
| Cyp2j8 | 0.0032 | 0.94216285 |
| Cyp2b19 | 0.00824 | -1.00868743 |
| Platr8 | 0.00144 | -1.28116048 |
| Dgkk | 0.02774 | -1.15602615 |
| Carhsp1 | 0.00144 | -1.23976282 |
| Hspa2 | 0.09085 | 0.2896347 |
| Aim2 | 0.33519 | -0.40776017 |
| Dcx | 0.00144 | -1.43461203 |
| 5730403I07Rik | 0.00144 | -1.31495282 |
| Ahnak2 | 0.00334 | -0.89496738 |
| Papss2 | 0.09018 | 0.51451943 |
| Gm10649 | 0.05778 | -0.76417947 |
| Gipc3 | 0.03326 | -1.46155462 |
| Henmt1 | 0.00144 | -1.28898079 |
| Henmt1 | 0.00144 | -1.27165604 |
| Ccdc68 | 0.01258 | 1.00580996 |
| Gm29243 | 0.98995 | 0.01705755 |
| Asap3 | 0.01926 | -0.64955347 |
| Prl6a1 | 0.00149 | -1.19696847 |
| Olfr1532-ps1 | 0.94954 | 0.08446004 |
| Olfr508 | 0.00144 | -1.28898079 |
| Olfr108 | 0.02189 | -1.79458904 |
| Prss50 | 0.12732 | -1.10715643 |
| Ggps1 | 0.61924 | -0.08906466 |
| Myo1h | 0.39079 | 0.35902256 |
| Gramd1c | 0.00257 | 0.86131504 |
| B3gat2 | 0.07278 | 0.45799692 |
| Dnase2b | 0.00144 | -1.28116048 |
| Clec4b2 | 0.00262 | -1.40516868 |
| LOC102641648 | 0.01337 | -0.49841751 |
| Pira7 | 0.85918 | -0.05770977 |
| Gm10555 | 0.05081 | -2.10695301 |
| Gm10839 | 0.00309 | -1.13602825 |
| Mpp1 | 0.01776 | -0.80512634 |
| Ankrd33b | 0.49349 | -0.28174669 |
| LOC102642334 | 0.00144 | -1.28116048 |
| Gm14340 | 0.03018 | -0.86953797 |
| 4930579G24Rik | 0.00144 | -1.27165604 |
| 4930579G24Rik | 0.84444 | -0.04479175 |
| Snora74a | 0.0186 | -0.89042956 |
| Scgb1b20 | 0.00144 | -1.31518407 |
| Zfp382 | 1 | 0 |
| Sephs2 | 0.87986 | -0.06707809 |
| Aars | 0.00144 | -1.28116048 |
| Kars | 0.01271 | -0.51273376 |
| Dnm1 | 0.6797 | -0.08067103 |
| Dnm1 | 0.5465 | -0.13475029 |
| Zc2hc1b | 0.00144 | -1.27730451 |
| Fam124b | 0.99455 | -0.00207531 |
| Pan2 | 0.03027 | 0.55934934 |
| Cd19 | 0.06779 | 0.63763151 |
| Cacna1i | 0.22981 | -0.28465225 |
| Ppnr | 0.96343 | -0.02258048 |
| Ndufv1 | 0.00511 | -0.81871303 |
| Snhg7os | 0.00332 | -0.79068806 |
| Lrrc31 | 0.19116 | -0.31515534 |
| Cmtm1 | 0.08269 | -2.3344966 |
| Olfr55 | 0.00144 | -1.25622482 |
| Etv4 | 0.12323 | -0.38955059 |
| Kmt2e | 0.07688 | 0.32663659 |
| Kmt2e | 0.2009 | 0.45671273 |
| Ankrd13b | 0.15617 | -1.261126 |
| Morc4 | 0.02487 | 0.49490825 |
| Fbxl13 | 0.00149 | -1.22582153 |
| Tpm1 | 0.07888 | -0.36286334 |
| Cbs | 0.01811 | -0.75857719 |
| Pramel7 | 0.00307 | -1.87793418 |
| Mettl5 | 0.05873 | 1.17642695 |
| Mettl5 | 0.0021 | 1.29293474 |
| Gm10469 | 0.00144 | -1.27454685 |
| Cib3 | 0.814 | -0.05675345 |
| Fam50a | 0.21449 | 0.20147733 |
| 1700008F21Rik | 0.16669 | -0.80719985 |
| Nrl | 0.00144 | -1.48010564 |
| Mcoln2 | 0.87 | -0.09654536 |
| Tstd1 | 0.76141 | -0.08204748 |
| Rgs19 | 0.12887 | -0.26847051 |
| Gm3264 | 0.01098 | 1.69550206 |
| Rgs13 | 0.00144 | -1.26560808 |
| Fndc7 | 0.01689 | -1.24426767 |
| Ctag2 | 0.06792 | -3.01749304 |
| H3f3a | 0.83151 | 0.03420575 |
| Gm10183 | 0.10531 | -0.37001721 |
| Ppp1r10 | 0.03276 | -1.03772473 |
| Ppp1r10 | 0.99273 | 0.00359934 |
| Cdca2 | 0.00144 | -1.31884045 |
| Prr5 | 0.18463 | -0.23846644 |
| Otop1 | 0.00563 | -1.38003246 |
| Rora | 0.46077 | -0.1474622 |
| Rps6kc1 | 0.01895 | -1.14915299 |
| Rps6kc1 | 0.03455 | -0.6903945 |
| Pqlc2 | 0.00177 | -1.15847607 |
| Pqlc2 | 0.02181 | -0.6864008 |
| Cacna1b | 0.01134 | -1.02600084 |
| Vps8 | 0.30079 | 0.40261633 |
| Klra5 | 0.00144 | -1.31884045 |
| Klrb1 | 0.00144 | -1.24613586 |
| Naaladl2 | 0.04182 | -0.85113592 |
| Tpgs2 | 0.67355 | -0.07479602 |
| Cd2 | 0.00144 | -1.12999368 |
| Kat6b | 0.24174 | 0.41426655 |
| Kat6b | 0.13505 | 0.2690755 |
| Deaf1 | 0.17161 | -0.26739717 |
| Nktr | 0.46581 | 0.23745996 |
| Ppp1cc | 0.17092 | 0.30912735 |
| Hmgxb3 | 0.00631 | -0.75434358 |
| Tubb2a-ps2 | 0.0736 | -0.48265706 |
| Slc38a11 | 0.01998 | -1.07815975 |
| Vmn2r5 | 0.00144 | -1.24613586 |
| Prkag3 | 0.69501 | 0.17621703 |
| Porcn | 0.01997 | -0.50338254 |
| Dcxr | 0.41945 | -0.28631296 |
| Calr4 | 0.18904 | -2.02022144 |
| Sod3 | 0.00144 | -1.29381979 |
| C330021F23Rik | 0.03591 | -0.43809571 |
| Git1 | 0.00466 | -0.70861443 |
| Zfp513 | 0.0041 | -0.73984028 |
| Arhgef4 | 0.25402 | -1.41582541 |
| Zfp583 | 0.98468 | 0.01593535 |
| BC005624 | 0.00144 | 3.44696767 |
| Adarb2 | 0.05945 | -2.18074573 |
| Vmn1r167 | 0.10081 | -3.33853591 |
| Mmd | 0.00968 | -0.99308048 |
| Ceacam12 | 0.00158 | -1.2446827 |
| Ahdc1 | 0.0127 | -0.62610627 |
| Cenpk | 0.00959 | 1.48965114 |
| Cenpl | 0.43374 | 0.19022355 |
| Cenpb | 0.01131 | -0.87069823 |
| Stxbp1 | 0.38094 | 0.16225023 |
| Mro | 0.00144 | -1.28116048 |
| Slc27a2 | 0.00144 | -1.31884045 |
| Mcidas | 0.22468 | 0.36507677 |
| Oxsr1 | 0.04416 | -0.40581132 |
| Pof1b | 0.01521 | -1.23534458 |
| Tmem132e | 0.34504 | -0.27994652 |
| Rab29 | 0.1424 | -0.34780369 |
| Malt1 | 0.00227 | 2.55124439 |
| Itih4 | 0.0144 | -1.19780167 |
| Itih5 | 0.00303 | 1.13008096 |
| Acap3 | 0.00256 | -0.83527924 |
| Csn1s2b | 0.00206 | -1.67344955 |
| Defa-rs10 | 0.03292 | -0.59302516 |
| Pkd1l2 | 0.00236 | -0.8369157 |
| Farp1 | 0.0026 | -1.30132103 |
| Lrr1 | 0.1115 | -0.46227254 |
| Ints10 | 0.05515 | 0.38595508 |
| A230065H16Rik | 0.00144 | -1.26934956 |
| Ehd2 | 0.08868 | -0.41397943 |
| A330050F15Rik | 0.00546 | -0.79634743 |
| Aadacl3 | 0.00144 | -1.28116048 |
| Lef1 | 0.81605 | -0.08221223 |
| Megf6 | 0.0019 | -0.97394859 |
| Sema6d | 0.30128 | -0.22497189 |
| 9430076C15Rik | 0.00913 | 0.66729252 |
| Creb5 | 0.00144 | -1.28116048 |
| Ddx5 | 0.94081 | -0.03163066 |
| Ddx5 | 0.01257 | -0.76911078 |
| Anxa6 | 0.36883 | -0.23212499 |
| Synpo | 0.00144 | -1.09812809 |
| Mdfi | 0.00654 | -1.21554223 |
| Hspa5 | 0.01049 | 0.74908664 |
| Znfx1 | 0.00353 | -0.75457285 |
| Wfdc6a | 0.00999 | 1.44425003 |
| Sort1 | 0.00144 | -1.22738855 |
| Vps25 | 0.60489 | -0.18943227 |
| Gypc | 0.13554 | -0.28985247 |
| Plekho2 | 0.36667 | -0.98000682 |
| Tdpoz4 | 0.00149 | -1.2703832 |
| Acaa1a | 0.00756 | -0.74365812 |
| Nr5a1 | 0.01305 | -0.55912799 |
| Hsd17b13 | 0.29743 | 0.39643593 |
| Mpzl3 | 0.03125 | -0.70942246 |
| Pmvk | 0.00627 | -0.77163682 |
| 1700028I16Rik | 0.00144 | -1.39611437 |
| Rac1 | 0.05631 | -0.42323139 |
| Gm14322 | 0.00451 | 1.53907992 |
| Lnx1 | 0.01107 | 0.66598009 |
| Dmtf1 | 0.92967 | -0.02356647 |
| Bpifb1 | 0.01246 | -0.70949532 |
| Gm9502 | 0.00144 | -1.31495282 |
| Prokr2 | 0.00201 | -1.11996532 |
| Prokr2 | 0.00144 | -1.25118919 |
| Mup17 | 0.0173 | -2.39480819 |
| Ccdc169 | 0.9516 | -0.04790873 |
| Zfp850 | 0.40126 | 0.51859327 |
| Hmga1 | 0.00175 | -1.69702083 |
| Tas2r137 | 0.87821 | 0.10085426 |
| Cacna1f | 0.25443 | 0.62742372 |
| Spag6l | 0.00144 | -1.29051721 |
| Prss51 | 0.06593 | -1.92350444 |
| Rbm12 | 0.08002 | 0.37419733 |
| Sptbn4 | 0.40011 | 0.18347391 |
| Arhgap40 | 0.04105 | -1.07764103 |
| Vmn1r12 | 0.59918 | -0.43631564 |
| Tnni3k | 0.28255 | -0.21986761 |
| Tti2 | 0.02567 | -0.51524343 |
| Ube2n | 0.02017 | 0.64882552 |
| Gm32126 | 0.56708 | -0.43072692 |
| Ctnna2 | 0.00144 | -1.27699683 |
| Prr23a | 0.00144 | -1.26301121 |
| Asphd1 | 0.77469 | -0.09248373 |
| Gm904 | 0.27358 | -0.44849886 |
| Trpm3 | 0.00158 | -1.2446827 |
| Trpm3 | 0.84596 | 0.21917322 |
| C130036L24Rik | 0.014 | 1.07651075 |
| 5330426L24Rik | 0.81362 | -0.1517422 |
| Slc20a2 | 0.0653 | -0.45230948 |
| Apc2 | 0.01377 | -0.69950445 |
| Gm13286 | 0.38585 | -0.33094863 |
| Clstn1 | 0.00565 | -0.70225651 |
| Gpatch2 | 0.21934 | 0.31337455 |
| Smim23 | 0.00145 | -1.2388121 |
| Tnfsf18 | 0.01118 | -0.94175894 |
| Ube2dnl1 | 0.00144 | -1.53332231 |
| 5430405H02Rik | 0.08101 | 0.36007363 |
| Ttll9 | 0.00144 | -1.24353547 |
| Ibsp | 0.09347 | -2.44733688 |
| Baalc | 0.69379 | 0.13436177 |
| Dtnb | 0.59302 | 0.29455825 |
| Ttll3 | 0.66085 | 0.19016137 |
| Ttll3 | 0.81424 | 0.14244593 |
| Ttll4 | 0.99932 | -0.00023969 |
| Gm29796 | 0.00446 | -1.46067242 |
| Gm8994 | 0.14512 | -0.31200027 |
| Ankrd16 | 0.80812 | 0.1369777 |
| Zfp619 | 0.03167 | 1.16666844 |
| Atp8a1 | 0.79742 | -0.06297282 |
| Rab11fip4 | 0.01002 | -1.33053696 |
| Tmem216 | 0.93102 | -0.02730585 |
| Acnat2 | 0.00144 | -1.24811894 |
| LOC105247651 | 0.43444 | 0.49412342 |
| Muc3 | 0.13225 | -0.63946208 |
| Gm10501 | 0.20867 | 0.26881995 |
| Ulk3 | 0.14092 | -0.27525222 |
| BC051226 | 0.00727 | -1.1139838 |
| Kcnab2 | 0.00405 | -1.75764491 |
| Kbtbd11 | 0.34653 | -1.31003313 |
| Tor3a | 0.00199 | -0.86414567 |
| Cd302 | 0.74631 | -0.05544786 |
| Has2os | 0.00528 | -1.47197047 |
| Npm3-ps1 | 0.00144 | -1.29085005 |
| Sirt3 | 0.0522 | -0.63727933 |
| Pax2 | 0.22075 | -0.85757348 |
| Pax2 | 0.93153 | 0.05907484 |
| Bcl9 | 0.82934 | -0.0418995 |
| Gm7257 | 0.00265 | -1.80368281 |
| Fat2 | 0.01948 | -1.94040875 |
| S1pr5 | 0.34906 | 0.37846954 |
| Gm5862 | 0.00144 | -1.27165604 |
| Gm10471 | 0.00145 | -1.23753193 |
| Atic | 0.46308 | -0.1493879 |
| 4932411E22Rik | 0.00177 | -1.19683356 |
| Eif4e1b | 0.31597 | -0.70217194 |
| Eif4e1b | 0.00144 | -1.24613586 |
| Tpt1 | 0.10717 | -0.30477701 |
| Gm6297 | 0.60264 | -0.29212288 |
| Gm7932 | 0.39374 | -0.52457688 |
| Ppp1r13l | 0.02445 | -0.69165589 |
| Ube4b | 0.02158 | -0.53702499 |
| 2310016D23Rik | 0.72615 | -0.09672538 |
| Gm4081 | 0.13386 | -0.81581966 |
| Gm35335 | 0.00144 | -1.28200278 |
| 4933433C11Rik | 0.00144 | -1.31884045 |
| Mapk8ip1 | 0.00144 | -1.25853398 |
| Fam168a | 0.0477 | 0.36917421 |
| Zfp609 | 0.09927 | -0.30079519 |
| Exosc7 | 0.02875 | -0.44878238 |
| Ptgis | 0.90823 | -0.02959165 |
| Prkar1b | 0.01978 | -0.66484772 |
| Gm2516 | 0.00144 | -1.27165604 |
| Fh1 | 0.31523 | -0.25972192 |
| Olfr1056 | 0.00819 | -1.59064986 |
| Ephx4 | 0.00144 | 1.63253759 |
| Hoxc4 | 0.77559 | 0.08147291 |
| Gm3831 | 0.22908 | -0.60205891 |
| Padi2 | 0.00145 | -1.13321342 |
| Adap1 | 0.02231 | -0.62192378 |
| Ppm1b | 0.81605 | 0.04450615 |
| Kcp | 0.38752 | -0.18637706 |
| Gtf2f1 | 0.0521 | -0.37850348 |
| Gtf2a1 | 0.39628 | 0.14610102 |
| Ralyl | 0.00144 | -1.29085005 |
| Ralyl | 0.04908 | -0.90497976 |
| Ldoc1 | 0.01869 | -1.90992111 |
| Lin28a | 0.13359 | -0.60364885 |
| Ngf | 0.04892 | -0.4716517 |
| Neu4 | 0.00144 | -1.31884045 |
| Ap2b1 | 0.49006 | -0.15023979 |
| Fbxo6 | 0.00537 | -1.04689032 |
| Nr2f1 | 0.01486 | -0.89268077 |
| Zfp184 | 0.92279 | 0.04327653 |
| Dlgap4 | 0.02095 | -0.58850952 |
| Zbtb24 | 0.02576 | 0.48473575 |
| Cpt2 | 0.39165 | 0.8076488 |
| Tmprss11e | 0.00144 | -1.28898079 |
| Gm21404 | 0.04919 | -2.18354068 |
| Tet2 | 0.15364 | 0.51719312 |
| Haao | 0.03904 | -0.72101617 |
| Tctn1 | 0.0198 | -0.85731028 |
| Mycbp | 0.08857 | 0.4414956 |
| BC049715 | 0.09931 | -0.68529659 |
| Eif2b3 | 0.01195 | 0.60664591 |
| Abcc5 | 0.04782 | 0.59452837 |
| Nos1 | 0.08711 | -1.11070478 |
| Mina | 0.04741 | 0.80290758 |
| Bbs5 | 0.00144 | 1.05503534 |
| Mrpl27 | 0.14493 | -0.36374585 |
| Ocm | 0.43845 | -0.32782709 |
| AU015836 | 0.14157 | -1.48943311 |
| Mecr | 0.34264 | -0.29045603 |
| Adam23 | 0.00144 | -1.30017714 |
| Tfdp2 | 0.96682 | 0.00718494 |
| Gm266 | 0.02678 | -1.20821276 |
| Grm4 | 0.45305 | 0.21105256 |
| Elp2 | 0.02425 | 0.450563 |
| 1600014C10Rik | 0.17243 | -0.45783262 |
| Fgf6 | 0.00149 | -1.65023753 |
| Fgf6 | 0.05654 | -1.57009445 |
| Gm10650 | 0.03512 | -0.90014657 |
| Lrp2bp | 0.01455 | -1.01991403 |
| Ankef1 | 0.00152 | -1.63418876 |
| Epb4.1l4b | 0.4003 | 0.3660193 |
| Slc27a5 | 0.7881 | -0.06825182 |
| A930003A15Rik | 0.00152 | -1.25878908 |
| Foxr2 | 0.00145 | -1.22376446 |
| H2-Ke6 | 0.02078 | -0.60193113 |
| 2610528J11Rik | 0.25026 | -1.33015088 |
| Gtf2i | 0.00737 | -0.61812459 |
| Chd6 | 0.65484 | -0.24516902 |
| Glp2r | 0.0027 | -1.11012596 |
| Olfr183 | 0.00144 | -1.25118919 |
| Wnt8a | 0.10673 | -0.74797519 |
| B020004J07Rik | 0.00144 | -1.28116048 |
| Syn1 | 0.02066 | -0.66177314 |
| Bin1 | 0.01391 | -0.56239068 |
| Hadhb | 0.52084 | -0.11957853 |
| Gm11468 | 0.00145 | -1.14157963 |
| Vmn2r34 | 0.02256 | -0.97953315 |
| Slc25a34 | 0.12637 | -0.67443206 |
| Daf2 | 0.74144 | 0.10836512 |
| Olfr251 | 0.00144 | -1.26301121 |
| Cenpp | 0.21028 | 0.42084394 |
| Manbal | 0.19081 | -0.67277589 |
| Gm438 | 0.00464 | -1.08005237 |
| Trex1 | 0.00284 | -1.0287142 |
| Trex2 | 0.18226 | -0.53593014 |
| Gm4841 | 0.53019 | 0.3974677 |
| Lin37 | 0.01962 | -0.51565671 |
| Gm4832 | 0.25452 | -0.18562331 |
| Sptan1 | 0.02087 | -0.52882885 |
| Vsig10 | 0.27749 | 0.38368605 |
| Lpin2 | 0.88521 | 0.05610525 |
| Lypd5 | 0.41148 | -0.52168799 |
| Cd3g | 0.63717 | -0.27221091 |
| LOC102641253 | 0.00149 | -1.34303097 |
| Etv3l | 0.14769 | -0.8580351 |
| Chsy1 | 0.19694 | -0.26218875 |
| Olfr1132 | 0.71134 | -0.35244439 |
| Cyp2d37-ps | 0.00807 | -1.65679785 |
| Prex1 | 0.00144 | -1.05048902 |
| Ckmt1 | 0.01299 | -0.70797897 |
| Prr5l | 0.7398 | 0.13529405 |
| Sh2d1b2 | 0.05042 | -2.05114563 |
| Olfr747 | 0.14079 | -2.26981911 |
| Socs2 | 0.22655 | 0.4898479 |
| Pcdhb12 | 0.13201 | 0.29641567 |
| Ncoa6 | 0.45997 | -0.38590832 |
| Sin3b | 0.04107 | -0.64307109 |
| Pcdhb16 | 0.00186 | 2.23400373 |
| Gm10648 | 0.00144 | 1.14860122 |
| Gm8672 | 0.96299 | -0.03591873 |
| Gm29750 | 0.00227 | -1.12522109 |
| Sirt6 | 0.06611 | -0.38250779 |
| Atp13a4 | 0.00144 | -1.38239536 |
| Suv39h2 | 0.06546 | -0.42162157 |
| Msn | 0.0447 | -0.40321807 |
| Scn8a | 0.44661 | -0.47874606 |
| Gm10216 | 0.00144 | -1.5146942 |
| Erbb2ip | 0.00777 | -0.8276154 |
| Dnaaf3 | 0.10873 | -0.45142759 |
| Gm10578 | 0.04374 | -2.08876985 |
| Zcchc12 | 0.0789 | -0.65401072 |
| Cdk9 | 0.00144 | -0.94843053 |
| Olfr616 | 0.21783 | -0.70402954 |
| Haglr | 0.82543 | -0.0422312 |
| Vpreb2 | 0.01826 | -1.33886732 |
| Pisd-ps3 | 0.07351 | 0.54679404 |
| AW011738 | 0.52853 | -0.33495868 |
| Dpcr1 | 0.00144 | -1.27165604 |
| Cyp4a30b | 0.00144 | -1.30713006 |
| Thy1 | 0.00447 | -0.76924858 |
| Ctsc | 0.00384 | -1.41420816 |
| Pcmtd1 | 0.8326 | -0.16008478 |
| Gm4544 | 0.05466 | 1.04185428 |
| Pick1 | 0.90339 | 0.04170078 |
| Nav2 | 0.01702 | -0.90605962 |
| Wdr92 | 0.0103 | 1.6059193 |
| Mrgprb1 | 0.0119 | -1.11518788 |
| Olfr1432 | 0.32168 | -0.90072423 |
| Slc45a1 | 0.09615 | -0.96013946 |
| Ncoa3 | 0.81338 | -0.06567185 |
| Mgmt | 0.00144 | -1.18256627 |
| Slbp | 0.63945 | -0.36950258 |
| Slbp | 0.00389 | 1.1111226 |
| 1700020N01Rik | 0.09051 | -0.90040519 |
| Rasgef1c | 0.07105 | -0.51900165 |
| Klre1 | 0.0097 | -2.40841736 |
| Kank3 | 0.17237 | -0.30764023 |
| Lemd1 | 0.06145 | -0.88410199 |
| Kcnma1 | 0.00144 | -1.30713006 |
| Ppara | 0.75432 | -0.06936232 |
| Mettl14 | 0.02429 | -1.02583207 |
| Papln | 0.01638 | -0.5621879 |
| Suv39h1 | 0.00144 | -1.52746817 |
| Spag17 | 0.01548 | -1.40428704 |
| Atf1 | 0.01211 | 1.05853565 |
| Ncf1 | 0.32214 | -0.20045761 |
| Kcnip1 | 0.00462 | -1.14485232 |
| Pde3a | 0.0048 | -0.91804804 |
| Crh | 0.00144 | -1.28116048 |
| Gm15762 | 0.0044 | -1.25333528 |
| Fam179a | 0.81362 | -0.06937543 |
| Trh | 0.00144 | -1.43144742 |
| Anapc1 | 0.68311 | -0.08783747 |
| Rrh | 0.00144 | -1.33059316 |
| Rhog | 0.01028 | -0.56760014 |
| Rpl36 | 0.00144 | -1.18996177 |
| Rnf126 | 0.00317 | -0.80616318 |
| Gm35028 | 0.03669 | -0.94779588 |
| Tmc4 | 0.12469 | 0.71555605 |
| 1700020N15Rik | 0.02219 | -1.16347252 |
| Abracl | 0.00144 | 0.96381208 |
| Pcnx | 0.06218 | -0.41412482 |
| Trappc11 | 0.08251 | 0.40219915 |
| Aqp5 | 0.03132 | -0.97953315 |
| Il1f5 | 0.00144 | -1.27165604 |
| Prss8 | 0.26609 | -0.45071819 |
| Olfr1152 | 0.00144 | -1.51667806 |
| Vmn2r55 | 0.09885 | -2.59090759 |
| Gm10757 | 0.14932 | -0.57192923 |
| Asap1 | 0.02108 | 0.55086777 |
| Tnfrsf23 | 0.77987 | -0.04949362 |
| Olfr1032 | 0.00449 | -1.11367897 |
| Vmn2r23 | 0.00144 | -1.27165604 |
| Adrm1 | 0.01605 | -0.62787564 |
| Cyb561d1 | 0.01439 | -0.5347852 |
| 2310002D06Rik | 0.88381 | 0.06660416 |
| Fer1l4 | 0.00145 | -1.22376446 |
| Nab2 | 0.83419 | -0.1233544 |
| Vmn2r100 | 0.05029 | -1.4256134 |
| Prr27 | 0.00176 | -1.24266807 |
| Aldoc | 0.00334 | -0.99175111 |
| Krtap20-2 | 0.29984 | -0.51496996 |
| Gm34478 | 0.96069 | -0.01435624 |
| Rpl14 | 0.00144 | -1.12495996 |
| Lcn2 | 0.20837 | -0.32085371 |
| Qk | 0.05678 | -2.25408167 |
| Cdh17 | 0.00144 | -1.22925777 |
| Gm29690 | 0.00439 | -0.84388187 |
| Nudt5 | 0.05561 | -0.42290976 |
| Ino80d | 0.00144 | 1.19852408 |
| Ehf | 0.05009 | -0.98169174 |
| Tyw1 | 0.01784 | -0.53608265 |
| Tyw1 | 0.96513 | 0.02748433 |
| Mrps14 | 0.21944 | 0.64700907 |
| Dus1l | 0.04458 | -0.46106932 |
| Rapgef1 | 0.01936 | -0.46935924 |
| Wdfy1 | 0.0298 | -0.61775466 |
| Taf7l | 0.32501 | -0.31985859 |
| Emc10 | 0.08131 | -0.69208215 |
| Zfp59 | 0.16834 | 0.61804498 |
| Cxxc4 | 0.00192 | -1.78563736 |
| Pkd1l3 | 0.00963 | -1.05225272 |
| Ccl27a | 0.01421 | -0.89468198 |
| 1700037C18Rik | 0.08723 | -0.41840794 |
| Vmn2r60 | 0.02615 | -0.64066583 |
| Vmn2r59 | 0.66768 | -0.21910445 |
| Gm40331 | 0.22043 | -1.71740592 |
| Sp6 | 0.29508 | -0.4243395 |
| Coro7 | 0.16336 | 0.64481799 |
| 4930402H24Rik | 0.14218 | -0.27772158 |
| Hs6st3 | 0.00144 | -1.29085005 |
| Ccdc155 | 0.3127 | -0.50681087 |
| Nlrp1a | 0.14099 | -0.85782229 |
| Csf3r | 0.19463 | 0.42179385 |
| Rps2 | 0.01654 | -0.89040924 |
| 5830416P10Rik | 0.28191 | -1.2856619 |
| Olfr281 | 0.06022 | -0.78914758 |
| Ankrd36 | 0.3116 | -0.70541769 |
| Gm3696 | 0.42186 | 0.57491025 |
| BC051628 | 0.00434 | -1.0948178 |
| D330025C20Rik | 0.19064 | -0.32914496 |
| Mfn1 | 0.00579 | 0.78522897 |
| Gm10621 | 0.96637 | 0.05452496 |
| Pqbp1 | 0.96396 | -0.0286132 |
| Preb | 0.06559 | -0.38743466 |
| Preb | 0.81646 | 0.09488846 |
| Gm4241 | 0.00144 | -1.29746368 |
| Speer2 | 0.00144 | -1.22098834 |
| Ing4 | 0.09027 | -0.36936981 |
| Lgals9 | 0.01236 | -0.57414817 |
| Itpkb | 0.00372 | -0.70447933 |
| Senp3 | 0.00174 | -0.84722611 |
| Golga7b | 0.01691 | -1.22563501 |
| Zfyve9 | 0.82166 | 0.04541262 |
| Smco2 | 0.70233 | 0.10878792 |
| Dynlrb1 | 0.02994 | -0.53746328 |
| Fxyd2 | 0.00206 | -0.996403 |
| Mucl1 | 0.00144 | -1.29864962 |
| Uba52 | 0.00144 | -1.11351976 |
| Trim44 | 0.02451 | 0.59926804 |
| Esp1 | 0.00144 | -1.27165604 |
| Tnpo3 | 0.03874 | 0.42040637 |
| Plp1 | 0.00144 | 2.98195164 |
| D5Ertd579e | 0.02874 | -0.53692073 |
| Prpsap1 | 0.02771 | -0.45107006 |
| Mapk10 | 0.7365 | 0.06457745 |
| Iba57 | 0.0687 | -0.98336503 |
| Kiss1 | 0.03433 | -1.35289493 |
| Tymp | 0.0675 | -0.74138473 |
| Csta1 | 0.00144 | -1.29085005 |
| Hspa8 | 0.90026 | 0.02261251 |
| Sqstm1 | 0.47683 | -0.27093151 |
| Zfp316 | 0.06661 | -0.69791812 |
| Olfr846 | 0.01227 | -1.3007917 |
| Olfr746 | 0.00144 | -1.22465584 |
| Olfr346 | 0.00144 | -1.5520152 |
| Cemip | 0.03036 | -2.03695825 |
| Gm15386 | 0.00145 | -1.26219879 |
| C1ql4 | 0.12744 | -1.18827374 |
| Tspan17 | 0.09362 | -0.37690237 |
| Pitpnm3 | 0.45397 | 0.13534041 |
| 1700028E10Rik | 0.73576 | 0.13247043 |
| Brix1 | 0.00237 | 0.94372048 |
| Zmym6 | 0.00144 | 1.92311609 |
| 1110017D15Rik | 0.86026 | 0.04249983 |
| Nt5c1b | 0.00213 | -1.08099404 |
| Nt5c1b | 0.01946 | -1.02877696 |
| Ttll3 | 0.11015 | 0.62342636 |
| Als2cr11 | 0.00144 | -1.27699683 |
| Fxr1 | 0.12892 | 0.51114789 |
| LOC102642045 | 0.06654 | 0.39855871 |
| Gm30551 | 0.01034 | -1.12164699 |
| Psph | 0.22852 | -0.31206927 |
| Anapc15 | 0.32425 | 0.22565771 |
| Ptpn18 | 0.00144 | -1.51182018 |
| Prr19 | 0.09744 | -0.75051292 |
| Prr18 | 0.59382 | 0.26747487 |
| Ddx39b | 0.01089 | -0.58949796 |
| AI429214 | 0.38057 | -1.494403 |
| Prr16 | 0.00144 | -1.59212241 |
| Prr11 | 0.01495 | 0.80713678 |
| Tas2r125 | 0.09369 | -0.85473465 |
| Vmn2r67 | 0.02033 | -0.85810509 |
| Sacs | 0.25495 | 0.33098105 |
| 2810032G03Rik | 0.00144 | -1.30713006 |
| A330070K13Rik | 0.03855 | -1.94298416 |
| Pidd1 | 0.02967 | -0.59642602 |
| LOC102641578 | 0.15283 | -0.33192059 |
| Maged2 | 0.0022 | -1.33816652 |
| Armcx3 | 0.01344 | 0.77754879 |
| Vmn2r52 | 0.22981 | -0.63379055 |
| Vmn1r203 | 0.00144 | -1.32599123 |
| Ago3 | 0.6405 | 0.16522197 |
| Il6ra | 0.16552 | 0.28611278 |
| Pear1 | 0.72253 | 0.25461238 |
| Ibtk | 0.15172 | 0.36933389 |
| Gm21637 | 0.12717 | -2.9119479 |
| Pigv | 0.0105 | -0.7316076 |
| Nudc | 0.73832 | -0.06479867 |
| Cdk20 | 0.00211 | -1.01410029 |
| Pkm | 0.00921 | -0.69545164 |
| Farp2 | 0.23311 | -0.50361602 |
| Ccl27a | 0.0607 | -0.50764017 |
| Gm20878 | 0.01006 | -0.92929675 |
| Gm20878 | 0.00144 | -1.41798464 |
| Ccl27a | 0.02672 | -0.75560671 |
| Gm6280 | 0.02602 | -1.24610027 |
| Bpifb4 | 0.07707 | -0.64960912 |
| Rnf19b | 0.59393 | -0.11369887 |
| 9430025E04Rik | 0.00816 | -1.19674833 |
| Gm10428 | 0.11708 | -0.59296673 |
| Defa1 | 0.11416 | -0.36438509 |
| LOC102635467 | 0.19679 | -0.22160248 |
| 9030624G23Rik | 0.46718 | 0.13326533 |
| 2410018L13Rik | 0.29497 | -0.23835117 |
| LOC105244782 | 0.10617 | 0.29434527 |
| Gm9292 | 0.0671 | -0.35397616 |
| 1700016K19Rik | 0.01304 | -0.64228109 |
| Cryzl1 | 0.0884 | 0.30561189 |
| 5430401F13Rik | 0.02442 | -0.73993521 |
| Gm17019 | 0.0017 | -1.2839886 |
| Gm17019 | 0.00256 | -1.94100048 |
| Polr1b | 0.00168 | -1.22579271 |
| LOC102641328 | 0.26235 | 0.41671739 |
| Slc17a2 | 0.0178 | -1.01654359 |
| LOC101055749 | 0.00144 | -1.20555712 |
| Mif | 0.00149 | -1.0519617 |
| Hist1h3g | 0.08107 | -0.64967737 |
| Phactr1 | 0.00244 | -0.81169337 |
| Cdc5l | 0.00658 | 0.72434355 |
| Nufip2 | 0.00985 | 0.63172657 |
| Anapc15 | 0.01949 | -0.63029446 |
| Sun5 | 0.10142 | -0.28377464 |
| Rap1gap | 0.62858 | -0.19573483 |
| Rapgefl1 | 0.02799 | 0.65906644 |
| Lzts1 | 0.00144 | -1.31495282 |
| Rora | 0.00158 | 1.47720554 |
| Prss54 | 0.00144 | -1.27165604 |
| Cplx2 | 0.43479 | -0.2480487 |
| Krba1 | 0.285 | -0.20193531 |
| Ccdc38 | 0.00341 | -1.45865201 |
| Vmn1r181 | 0.00144 | -1.26934956 |
| Vmn1r59 | 0.00195 | -1.41597584 |
| 2410089E03Rik | 0.01404 | 0.53526248 |
| 2410089E03Rik | 0.19943 | -0.25879741 |
| Psg27 | 0.0498 | 1.13459348 |
| Psg26 | 0.01312 | -1.65214684 |
| Psg21 | 0.72194 | -0.23592088 |
| Mxra7 | 0.02057 | -0.77085315 |
| Camsap3 | 0.01063 | -0.63442635 |
| Pcdhgb1 | 0.02156 | 1.3633384 |
| Pcdhgb1 | 0.05601 | -1.10838041 |
| Mep1a | 0.00144 | -1.28898079 |
| Tas2r113 | 0.97441 | -0.01841653 |
| Adam28 | 0.01107 | -1.00198097 |
| Gm5136 | 0.00186 | 0.97099003 |
| Ush1c | 0.04588 | 1.15374246 |
| Ush1g | 0.93887 | -0.01996486 |
| Clmp | 0.36813 | 0.15665794 |
| Cd68 | 0.3672 | -0.54623149 |
| Il1rl2 | 0.00144 | -1.29049509 |
| Slc13a3 | 0.65166 | -0.21096911 |
| Slc10a3 | 0.01987 | -0.67078694 |
| Cdh6 | 0.00144 | -1.28883288 |
| 1700110I01Rik | 0.02601 | -0.56040349 |
| Arf5 | 0.00144 | -1.63545479 |
| Def8 | 0.00145 | -1.22193674 |
| Dpf1 | 0.43864 | -0.2721078 |
| Zfp827 | 0.37893 | -0.23106787 |
| Zfp827 | 0.05723 | -0.91020214 |
| Dpep2 | 0.25125 | -0.28303908 |
| Prune2 | 0.00546 | 1.44308622 |
| LOC102642370 | 0.09051 | -0.48388418 |
| Spata19 | 0.02462 | -0.89467554 |
| Iqsec3 | 0.00144 | -2.0486235 |
| Usp21 | 0.00765 | -0.72424731 |
| Usp26 | 0.12592 | -0.79997687 |
| Fau | 0.00144 | -1.2053761 |
| Dclre1c | 0.00145 | 1.80263809 |
| Fam179b | 0.01845 | -0.59947041 |
| Fam179b | 0.00243 | 1.48245168 |
| 8430429K09Rik | 0.06506 | -0.79468327 |
| Agxt2 | 0.03481 | -0.6351948 |
| Phykpl | 0.32187 | 0.20908026 |
| Phykpl | 0.3311 | 0.68321686 |
| Sh3glb1 | 0.02012 | 0.47047031 |
| Galt | 0.87102 | 0.0334148 |
| Hexim2 | 0.01157 | -0.57316282 |
| Sp110 | 0.03526 | -0.47977283 |
| AB124611 | 0.35372 | -0.38051332 |
| Agbl5 | 0.33776 | -0.20066999 |
| Serpinb6a | 0.8198 | 0.03928241 |
| Trim27 | 0.03942 | -0.38074943 |
| Trim47 | 0.00144 | -1.15550996 |
| Vps13a | 0.00144 | 1.50424379 |
| Lcp1 | 0.00158 | -1.49478293 |
| Oprl1 | 0.47889 | -0.19025992 |
| Ifi204 | 0.00327 | 1.50149005 |
| Mcu | 0.03286 | -0.56298136 |
| Endou | 0.65855 | 0.09711045 |
| Oacyl | 0.19126 | 0.40886348 |
| Baiap2l2 | 0.02135 | -0.47042451 |
| Orai2 | 0.0804 | -0.56853456 |
| Akt2 | 0.44123 | -0.27371678 |
| Akt2 | 0.00494 | -0.92493771 |
| Mpo | 0.00324 | -1.41218282 |
| Crtc3 | 0.00494 | -0.78815577 |
| Noa1 | 0.27107 | -0.30481624 |
| Enah | 0.67291 | -0.07116872 |
| Scgb2b7 | 0.00144 | -1.28375735 |
| Scgb2b21 | 0.00144 | -1.28116048 |
| Gm10115 | 0.0068 | 2.1474664 |
| Myh3 | 0.00144 | -1.29746368 |
| Dffa | 0.02539 | -0.80237851 |
| Gabpb1 | 0.00348 | 0.79549329 |
| Fcho2 | 0.00144 | 1.93645362 |
| 1700010L13Rik | 0.00144 | -1.30017714 |
| Aak1 | 0.86658 | -0.06827098 |
| Hck | 0.01925 | -0.58205384 |
| Gm3598 | 0.00144 | -1.59027635 |
| Gm3455 | 0.11812 | -0.90282563 |
| Hid1 | 0.00926 | -0.87157795 |
| Bcl3 | 0.02887 | -0.66896694 |
| Hnrnpa1 | 0.01098 | 0.95380764 |
| Crocc | 0.02618 | -0.54623769 |
| S100a11 | 0.01241 | -0.71405425 |
| Setd3 | 0.37799 | -0.14584697 |
| Ctdp1 | 0.13465 | -0.25099711 |
| Gm24474 | 0.5127 | 0.30592152 |
| Akap8l | 0.8833 | -0.02937671 |
| Macrod2 | 0.38019 | -1.3474745 |
| Macrod2 | 0.16068 | -0.72299303 |
| Sebox | 0.00144 | -1.28116048 |
| D430042O09Rik | 0.11775 | 0.30023857 |
| Cygb | 0.00144 | -1.23290229 |
| 9230110C19Rik | 0.01457 | 1.24648123 |
| Smtn | 0.10142 | 0.32942805 |
| Gm6432 | 0.00144 | -1.29085005 |
| Naa50 | 0.07226 | 0.36204362 |
| Krtap19-5 | 0.00144 | -1.61626714 |
| Medag | 0.03506 | -0.50399339 |
| Snx29 | 0.63274 | -0.1815542 |
| Snx29 | 0.71217 | -0.24385548 |
| Ccnb1 | 0.49331 | -0.45235308 |
| Zfp109 | 0.05084 | -1.06616472 |
| Tcra | 0.00503 | -1.3395994 |
| Zfp189 | 0.00249 | 1.25240233 |
| Hook3 | 0.36138 | -0.24046681 |
| Hook2 | 0.87162 | 0.03413845 |
| Rps15a-ps6 | 0.6888 | 0.14224936 |
| Gm15417 | 0.01124 | -0.87144403 |
| 6430503K07Rik | 0.00144 | -1.31627578 |
| Chac2 | 0.00185 | 1.1064564 |
| Dppa4 | 0.03272 | -0.88809498 |
| Mok | 0.19914 | -0.24363347 |
| Kdm5b | 0.01617 | -0.55372477 |
| Sstr2 | 0.00158 | -1.2446827 |
| Brwd1 | 0.82934 | -0.06498424 |
| Tdrkh | 0.07542 | 1.06019075 |
| Siva1 | 0.02218 | -0.80323377 |
| Gm14308 | 0.05949 | 0.91512133 |
| Rpl27a | 0.00265 | -0.94492713 |
| 2310067B10Rik | 0.00144 | -1.90807018 |
| Olfr1500 | 0.0763 | -1.35112964 |
| Scn3a | 0.01306 | 1.41541917 |
| Scn3a | 0.39093 | -0.43466873 |
| Pgr15l | 0.00145 | -1.26219879 |
| Gm38388 | 0.01588 | -0.99849484 |
| Krtap9-5 | 0.01261 | -1.96695178 |
| Kdm2b | 0.28472 | -0.29408082 |
| Kdm2b | 0.56239 | -0.1215948 |
| Gm5849 | 0.00243 | -1.61302126 |
| Gm11559 | 0.02296 | -0.46083196 |
| Hdac9 | 0.27698 | -0.52247236 |
| Capzb | 0.92816 | 0.03082841 |
| 4930528A17Rik | 0.00287 | 1.16563405 |
| Gm10624 | 0.00144 | 1.04307599 |
| Nebl | 0.00283 | 1.32622018 |
| 1500002C15Rik | 0.09249 | -1.13466011 |
| Defa3 | 0.00144 | -1.24613586 |
| Gm8580 | 0.03637 | -0.4667721 |
| Olfr1213 | 0.00158 | -1.2446827 |
| Fam89b | 0.00144 | -1.21756305 |
| Ica1 | 0.27477 | 0.53136726 |
| Mpp4 | 0.09089 | -0.76017882 |
| Mpp4 | 0.12024 | -0.63834366 |
| Prss53 | 0.00569 | -0.81938892 |
| Gm10561 | 0.75873 | -0.13303199 |
| Arpc3 | 0.01763 | -0.52168273 |
| 4930412O13Rik | 0.10788 | -0.99256328 |
| Ly6c1 | 0.01062 | -0.65445076 |
| Nmb | 0.00154 | -1.14992475 |
| Npbwr1 | 0.00144 | -1.27165604 |
| 4921534A09Rik | 0.2364 | -0.79441651 |
| Lrrc31 | 0.00144 | -1.27165604 |
| Lrrc61 | 0.00579 | -0.76405704 |
| Gas2l3 | 0.21182 | 0.36545601 |
| Trim12a | 0.00595 | 1.55241841 |
| Trim12c | 0.59219 | -0.123287 |
| Cnot6 | 0.45367 | 0.13789164 |
| Prkci | 0.03287 | 0.48240904 |
| Gm33402 | 0.00144 | -1.29878664 |
| Pcmtd2 | 0.00145 | 1.97358042 |
| Trim58 | 0.44667 | -0.54728252 |
| Cdc27 | 0.79764 | 0.04345774 |
| Gng7 | 0.00145 | -1.25646236 |
| Pced1a | 0.00416 | -0.70069666 |
| Pabpn1 | 0.20866 | -0.26327501 |
| Olfr1040 | 0.00144 | -1.29085005 |
| Gm4098 | 0.0181 | -0.96319758 |
| Vipr1 | 0.02973 | -0.65808339 |
| Lama5 | 0.00715 | -0.84834984 |
| Vmn2r116 | 0.01133 | -1.90474851 |
| Msrb1 | 0.44111 | -0.28692512 |
| Olfr1255 | 0.00144 | -1.25118919 |
| Dnaic2 | 0.50599 | -0.36599718 |
| Rhot2 | 0.89367 | -0.07781586 |
| Hemk1 | 0.03763 | -0.4189589 |
| 1700027F06Rik | 0.00144 | -1.29049509 |
| Gm10754 | 0.00144 | -1.27165604 |
| Hormad1 | 0.00144 | -1.28116048 |
| Vps37d | 0.00172 | -1.24013383 |
| Casp14 | 0.099 | 0.46177227 |
| Fam122c | 0.00144 | -1.31495282 |
| Rapgef2 | 0.09537 | 0.3597045 |
| Gpatch8 | 0.11633 | 0.25581988 |
| Rps5 | 0.00144 | -1.25924357 |
| Clec18a | 0.65795 | 0.17716606 |
| Clec18a | 0.17631 | -0.81220581 |
| Ube2l3 | 0.19125 | -0.2605723 |
| Ank1 | 0.1802 | -0.27944888 |
| LOC102642272 | 0.00144 | -1.29049509 |
| Naip6 | 0.00144 | 2.57468206 |
| Mup20 | 0.00526 | -1.5196067 |
| Krcc1 | 0.00144 | 1.80470633 |
| Matk | 0.1186 | -0.30978658 |
| Tnk2os | 0.01372 | -0.93139367 |
| Tomm5 | 0.0818 | -0.67553728 |
| Gm5801 | 0.0193 | -0.63907302 |
| Tomm6 | 0.1487 | 0.70549525 |
| Gm10473 | 0.00145 | -1.27532353 |
| Dph3 | 0.01947 | -0.51735566 |
| Thbs3 | 0.01049 | -0.66264456 |
| Ntng1 | 0.23464 | -0.69012978 |
| Csmd1 | 0.00144 | -1.28116048 |
| Rgp1 | 0.03137 | -0.54006533 |
| Gm13271 | 0.03284 | -0.49707552 |
| Zfand2a | 0.70994 | -0.0663605 |
| Cfap58 | 0.00144 | -1.28116048 |
| Tmem74b | 0.00347 | -1.11584304 |
| Me2 | 0.00144 | -1.25118919 |
| 4930427A07Rik | 0.0866 | -0.42675272 |
| Mgll | 0.00389 | -0.8863579 |
| Mgll | 0.00144 | -1.26279151 |
| Gm4763 | 0.00766 | -1.12466538 |
| 2210010C04Rik | 0.92099 | -0.06952887 |
| Lgals4 | 0.0464 | 1.34059123 |
| Dusp5 | 0.35403 | -0.40601295 |
| Dusp14 | 0.0282 | -0.95800248 |
| C230099D08Rik | 0.00166 | -1.21062785 |
| Ogfod1 | 0.01448 | -0.56919295 |
| Spata9 | 0.00144 | -1.4333006 |
| Inadl | 0.32423 | 0.22380815 |
| Inadl | 0.98349 | -0.00573643 |
| Phc2 | 0.18491 | -0.74317082 |
| Phc2 | 0.04469 | 0.93222403 |
| Tmprss11b | 0.00144 | -1.28116048 |
| Tmprss11a | 0.05995 | -0.8689511 |
| Abca9 | 0.05687 | 0.5713431 |
| Ppp3cc | 0.00144 | 1.57717956 |
| Amh | 0.00144 | -1.30713006 |
| Abca3 | 0.00802 | -0.58919614 |
| D430041D05Rik | 0.17536 | -0.2767124 |
| Epha5 | 0.00594 | -1.29258142 |
| Atf2 | 0.00386 | 0.88873951 |
| Ttf2 | 0.16182 | 0.23086303 |
| Tomm5 | 0.30562 | -0.45495414 |
| Mfap2 | 0.00144 | -1.19069884 |
| Mfap3 | 0.0334 | 0.47054823 |
| Mfap5 | 0.0404 | -0.43095793 |
| Mag | 0.11845 | -0.76085211 |
| Elovl2 | 0.00144 | -1.28116048 |
| Mthfsl | 0.11873 | 0.49385947 |
| Msi1 | 0.00144 | -1.28116048 |
| Magt1 | 0.11196 | -0.27754937 |
| Smc5 | 0.09397 | -0.52933954 |
| Tmem238 | 0.00172 | -1.13797618 |
| BC030499 | 0.10665 | 1.18536748 |
| 4932438A13Rik | 0.02704 | 1.37840877 |
| E2f7 | 0.47908 | 0.25580611 |
| Mtnr1a | 0.00144 | -1.29746368 |
| C1qtnf3 | 0.15662 | -0.5325855 |
| Gm10433 | 0.00149 | -1.2703832 |
| Gm6524 | 0.04455 | 0.52321703 |
| 1700027A15Rik | 0.76948 | -0.11562497 |
| Pdzrn3 | 0.15883 | 0.42346213 |
| Smyd1 | 0.00144 | -1.17574354 |
| Vmn2r81 | 0.00218 | -1.63746178 |
| Col12a1 | 0.00459 | -0.81225791 |
| Col12a1 | 0.00144 | -1.30713006 |
| Pde6a | 0.04064 | -2.51337542 |
| Begain | 0.54677 | -0.17189471 |
| Atp6v0a1 | 0.02548 | -0.51444452 |
| Gm14920 | 0.00144 | -1.26560808 |
| Plxdc1 | 0.15566 | -0.50557381 |
| 2900041M22Rik | 0.03113 | -0.81969673 |
| Mettl8 | 0.44188 | 0.29494765 |
| Olfr850 | 0.00428 | -1.34813137 |
| Rad23b | 0.54069 | -0.3997696 |
| Gm20654 | 0.01745 | -0.96204401 |
| Aspscr1 | 0.05947 | -0.54261135 |
| Syne3 | 0.09201 | -0.37182967 |
| Tgtp2 | 0.04593 | 1.01190268 |
| Tmem41a | 0.09511 | -0.53791085 |
| Gstm1 | 0.00659 | -0.76174619 |
| Shank1 | 0.3986 | -0.56681324 |
| Etv3 | 0.01523 | 0.56278172 |
| Gm7714 | 0.00144 | -1.29746368 |
| Dbndd2 | 0.00482 | -0.83308466 |
| Gcsam | 0.00145 | -1.2388121 |
| Tsen34 | 0.00308 | -1.23030436 |
| Tcp11 | 0.00201 | -2.3518233 |
| 9530057J20Rik | 0.00324 | 1.0631645 |
| Pnldc1 | 0.99844 | -0.0005276 |
| Slc7a12 | 0.00144 | -1.31495282 |
| Olfr222 | 0.00144 | -1.31978601 |
| Gm4303 | 0.00144 | -1.20555712 |
| Opn4 | 0.27372 | -0.33307695 |
| Cdh24 | 0.00144 | -1.29085005 |
| 1700122H20Rik | 0.03896 | -0.82593788 |
| Zfp456 | 0.0072 | 1.48358158 |
| Tmsb4x | 0.60731 | 0.21643405 |
| Tmsb4x | 0.08453 | -0.40775333 |
| Ak9 | 0.00144 | -1.28898079 |
| Zfhx4 | 0.02725 | -1.04486201 |
| Olfr455 | 0.00145 | -1.55947114 |
| ND6 | 0.00144 | -3.02278111 |
| Nlgn1 | 0.52211 | -0.48530292 |
| Chst1 | 0.02755 | -0.48271717 |
| Psmd1 | 0.03628 | 0.41975263 |
| Zfp229 | 0.00144 | 1.73534056 |
| Pdxdc1 | 0.0823 | -0.63586146 |
| Pnldc1 | 0.57047 | -0.16805696 |
| Mybl2 | 0.00149 | -1.2703832 |
| Nfyc | 0.38414 | 0.19898903 |
| Scgb2b2 | 0.06129 | -0.95010236 |
| Fads6 | 0.00272 | -1.89530675 |
| Rhobtb3 | 0.01946 | 0.48124305 |
| Chd3 | 0.5844 | 0.1086624 |
| Ghitm | 0.16508 | -0.24538388 |
| Ift74 | 0.00317 | 1.33570679 |
| Mthfs | 0.52029 | 0.17005359 |
| Sik1 | 0.01302 | -0.69620284 |
| Pou4f2 | 0.00144 | -1.25118919 |
| Ces3a | 0.0017 | -1.24950944 |
| Gm31048 | 0.01292 | -1.14580177 |
| Wfikkn2 | 0.02869 | -0.94196568 |
| Gm3219 | 0.16873 | 0.31173132 |
| Set | 0.62342 | 0.09528085 |
| Rpl29 | 0.14829 | -0.34943925 |
| Fam83e | 0.17855 | -1.2755212 |
| Smg6 | 0.00237 | -0.77753493 |
| Lppr3 | 0.03085 | -1.09185762 |
| Mov10 | 0.00144 | -1.06663172 |
| Gm5627 | 0.00144 | -1.28116048 |
| Unc5a | 0.01227 | -0.59545897 |
| Gm3238 | 0.72888 | -0.12531839 |
| LOC101056219 | 0.10883 | -0.82601577 |
| Gm30942 | 0.00145 | -1.2388121 |
| Gm13807 | 0.17631 | -0.6168114 |
| Esco1 | 0.00793 | 0.74293524 |
| Trmt10b | 0.03931 | 0.43681495 |
| Tdpoz3 | 0.00144 | -1.30017714 |
| Uso1 | 0.00145 | 1.10129532 |
| Il15ra | 0.87818 | -0.02696666 |
| Olfr1423 | 0.00183 | -1.16801417 |
| Acsf2 | 0.49768 | -0.17082212 |
| Hoxa3 | 0.28824 | -0.75191572 |
| Krt9 | 0.09427 | -1.8209788 |
| Spidr | 0.11166 | 0.33958558 |
| H2-M10.6 | 0.00145 | -1.2388121 |
| Gm5797 | 0.00144 | 2.83130998 |
| Hoxa1 | 0.33052 | -1.3942581 |
| Ddx39 | 0.11169 | -0.38753356 |
| Samt3 | 0.00144 | -1.30713006 |
| Mtl5 | 0.20071 | -0.37454378 |
| Zfp563 | 0.2521 | 0.5141741 |
| Gm13582 | 0.00144 | -1.26560808 |
| Whsc1l1 | 0.55428 | -0.17262475 |
| Apopt1 | 0.10829 | -0.39877303 |
| 1700020D12Rik | 0.00145 | -1.26219879 |
| Scube2 | 0.01789 | -1.01729162 |
| Pus3 | 0.00973 | 0.70282669 |
| Grwd1 | 0.49742 | -0.24786849 |
| Col15a1 | 0.00225 | -1.4801611 |
| Zfp352 | 0.16454 | -0.29093426 |
| 3110062M04Rik | 0.003 | -0.81507203 |
| 3110062M04Rik | 0.0776 | -1.07046721 |
| Fam45a | 0.14927 | 0.32174847 |
| Gm12657 | 0.26658 | 0.1862105 |
| Catsperb | 0.0017 | -1.24950944 |
| Itgbl1 | 0.27239 | 0.33999079 |
| Gm10503 | 0.09342 | -0.79009704 |
| Mcf2l | 0.52711 | -0.1408735 |
| Mcf2l | 0.28704 | 0.3292688 |
| Hira | 0.2091 | -0.21910359 |
| Lamtor3 | 0.53578 | -0.13830379 |
| 1700049E17Rik1 | 0.12891 | -2.13347669 |
| ATP8 | 0.26477 | -0.20504006 |
| Tprgl | 0.00317 | -0.71706798 |
| Prdm5 | 0.13888 | 0.25645949 |
| Rabep1 | 0.14792 | 0.33171141 |
| Ulk2 | 0.70661 | 0.06085892 |
| 4930433I11Rik | 0.00145 | -1.25646236 |
| Chst14 | 0.10405 | -0.4030528 |
| Gm9934 | 0.00144 | -1.30713006 |
| Zfp383 | 0.0995 | 0.54302399 |
| Fdx1 | 0.11787 | -0.39469005 |
| Igsf1 | 0.24427 | -0.57363095 |
| Foxf1 | 0.86843 | -0.02891847 |
| Dppa2 | 0.03528 | -1.04228543 |
| Gm10440 | 0.06595 | -3.65611172 |
| Rcc1 | 0.00652 | -0.70772081 |
| Atpaf2 | 0.46921 | -0.21932364 |
| Abhd14b | 0.07106 | -0.3796978 |
| Stat2 | 0.916 | -0.02431822 |
| Fmo3 | 0.14792 | -1.0763072 |
| Tas2r122 | 0.00144 | -1.28898079 |
| LOC102641064 | 0.00183 | -0.88522762 |
| AU022751 | 0.00144 | -1.29085005 |
| Arl6 | 0.00229 | 1.21686784 |
| Myo7a | 0.16496 | -0.28242935 |
| Ormdl3 | 0.01048 | -0.74381213 |
| Lamp1 | 0.06018 | -0.3273346 |
| Fastk | 0.00152 | -1.07002814 |
| Gm13315 | 0.63281 | 0.30944261 |
| Map2 | 0.34696 | -1.68431704 |
| Gm5177 | 0.05504 | -2.14153722 |
| Slc48a1 | 0.00144 | -1.09493603 |
| Hsf3 | 0.00144 | -1.29085005 |
| H3f3b | 0.55527 | 0.09541655 |
| Dtx3l | 0.63744 | 0.0852438 |
| Gm38448 | 0.22609 | -0.80133933 |
| Skap1 | 0.03872 | -1.2300286 |
| Gm9920 | 0.05857 | 0.60700332 |
| Fignl2 | 0.35133 | -0.50747857 |
| Tagap | 0.38585 | -0.13828024 |
| Zfp457 | 0.0189 | 0.9035661 |
| Zfp943 | 0.00284 | 1.33302408 |
| Gm13403 | 0.70147 | 0.20486733 |
| Prss40 | 0.00584 | -1.13460435 |
| U2af1 | 0.12689 | -0.25926427 |
| Mapk8ip3 | 0.11324 | -0.32002818 |
| Rbp1 | 0.00406 | -1.05903286 |
| Adcy10 | 0.28195 | -0.41883052 |
| Vamp4 | 0.31914 | 0.17735096 |
| Syt1 | 0.00974 | -1.12233866 |
| Rpl13a | 0.00144 | -1.01840745 |
| Adgrf3 | 0.0197 | -0.94464244 |
| Kif1b | 0.08998 | 0.34126808 |
| Gphb5 | 0.00232 | -0.93484667 |
| Sgpp1 | 0.11661 | 0.31477167 |
| Idi2 | 0.66677 | -0.26200034 |
| Fktn | 0.1455 | -0.39351831 |
| Otud5 | 0.04332 | -0.38715185 |
| Mvk | 0.01105 | -0.79201187 |
| Fancf | 0.02499 | -0.70497755 |
| Fancc | 0.03231 | 0.8210127 |
| Cmip | 0.06246 | -0.42164892 |
| Magea8 | 0.00354 | -1.16139591 |
| Hilpda | 0.05659 | -0.57545142 |
| G6pc2 | 0.00144 | -1.25118919 |
| Mterf1a | 0.14743 | 0.51340708 |
| Hap1 | 0.39198 | -0.27116783 |
| Ephb4 | 0.00144 | -1.34061518 |
| Lrrc55 | 0.04633 | -0.91948177 |
| Gm10817 | 0.7666 | -0.30462381 |
| Zfyve28 | 0.12063 | -0.5133682 |
| Hnrnpa3 | 0.00144 | 1.97742494 |
| C130079G13Rik | 0.00144 | -1.26428512 |
| Mylpf | 0.52788 | -0.21420147 |
| Dnajb5 | 0.11878 | -0.60896395 |
| Tdgf1 | 0.81162 | -0.23593975 |
| Gm20867 | 0.00144 | -1.27730451 |
| Tmem50a | 0.10517 | -0.31766627 |
| Gm4419 | 0.15389 | -2.61143767 |
| Olfr1480 | 0.00145 | -1.22518835 |
| Gm6277 | 0.08823 | -0.61623023 |
| Cyp2c68 | 0.00717 | -1.0591794 |
| H2afy | 0.23897 | -0.32005673 |
| Prdx4 | 0.35469 | 0.37946159 |
| Insm2 | 0.00144 | -1.28116048 |
| Cluh | 0.98703 | 0.00887867 |
| LOC101056056 | 0.13966 | -0.72786665 |
| 4930412E21Rik | 0.13433 | -0.8613224 |
| Ipo5 | 0.17494 | 0.26495202 |
| Cd46 | 0.55026 | -1.17331277 |
| Scgb2b20 | 0.14769 | 0.49053681 |
| Fsip2 | 0.00144 | -1.26219879 |
| Gm12887 | 0.00144 | -1.28116048 |
| Smek2 | 0.0103 | 0.86515866 |
| Pclo | 0.1598 | -0.22355034 |
| Pde4dip | 0.04153 | -0.4075151 |
| Pde4dip | 0.26381 | 0.52022674 |
| Slc35d1 | 0.00144 | -1.89563678 |
| Pigyl | 0.0016 | -1.22789763 |
| Mif4gd | 0.28697 | -0.3834491 |
| Tbc1d19 | 0.00144 | 1.28138446 |
| 1700003M02Rik | 0.00144 | -1.25118919 |
| 1700049G17Rik | 0.26556 | 0.45996245 |
| Gm12250 | 0.00234 | 1.00187669 |
| H2-Ob | 0.00494 | -1.7828001 |
| Pex5 | 0.02546 | -0.63872241 |
| Cetn2 | 0.66648 | -0.07500729 |
| 9130011E15Rik | 0.31543 | -0.29950339 |
| Gm6654 | 0.25349 | 0.52035548 |
| 1700116B05Rik | 0.00144 | -1.27699683 |
| Olfr843 | 0.09595 | -0.98421697 |
| Olfr543 | 0.01282 | -0.95969948 |
| Clrn2 | 0.013 | -1.06171525 |
| 6330403A02Rik | 0.32848 | -0.53780099 |
| Fgfr1 | 0.00247 | -0.78923271 |
| Olfr971 | 0.00144 | -1.31884045 |
| Olfr371 | 0.00733 | -1.25878908 |
| Cdc42ep5 | 0.00144 | -1.04828482 |
| Ocel1 | 0.08535 | -0.29104606 |
| Arx | 0.00144 | -1.27730451 |
| Ugt1a6a | 0.3134 | -0.85306196 |
| Zfp12 | 0.06947 | 1.00942815 |
| Gas7 | 0.05505 | -0.98350779 |
| Arl4d | 0.00144 | -1.22909108 |
| Ppm1b | 0.749 | 0.05841422 |
| Mall | 0.00523 | -0.74256194 |
| Ccdc134 | 0.52706 | -0.11042733 |
| Gm6566 | 0.04185 | 0.85188393 |
| Lipn | 0.09687 | -0.80699347 |
| Sox5it | 0.00145 | -1.22376446 |
| Kansl3 | 0.63084 | 0.07371431 |
| Magi1 | 0.02715 | 0.5261106 |
| Etohi1 | 0.00735 | 2.08978158 |
| Gm3902 | 0.08883 | -2.15906877 |
| LOC105242475 | 0.00486 | -1.16364063 |
| Scn8a | 0.01042 | -0.66120194 |
| Mid1 | 0.0025 | -1.18018946 |
| Sft2d3 | 0.00179 | -0.86569711 |
| Magix | 0.20449 | 0.27195139 |
| Slmo1 | 0.00145 | 1.6818584 |
| Gcgr | 0.0285 | -1.01165528 |
| Lyrm7 | 0.22467 | 0.25823637 |
| Cul9 | 0.01968 | -1.18783144 |
| Olfr437 | 0.00365 | -1.52074747 |
| Mtss1l | 0.03956 | -0.42419659 |
| Kif2a | 0.8798 | 0.02763359 |
| Akap13 | 0.43897 | -0.1971924 |
| Olfr525 | 0.17233 | -0.7718563 |
| A330008L17Rik | 0.00144 | -1.28116048 |
| Cmtm2a | 0.00336 | -1.20864476 |
| Cmtm2a | 0.00145 | -1.2388121 |
| Hsd17b14 | 0.2406 | -0.25344038 |
| Col16a1 | 0.00145 | -1.22376446 |
| Col16a1 | 0.00376 | -0.77167502 |
| Col16a1 | 0.89088 | -0.07264884 |
| Igfbp2 | 0.17243 | -0.57350695 |
| AF067061 | 0.00144 | -1.20555712 |
| Bsnd | 0.00144 | -1.32766452 |
| Lrrk1 | 0.00442 | -0.79986746 |
| Bcar1 | 0.00584 | -0.74758971 |
| Gm5891 | 0.00144 | -1.28898079 |
| LOC102640985 | 0.00145 | -1.26219879 |
| Nin | 0.00145 | 1.72267999 |
| Tk1 | 0.09393 | -0.80017931 |
| Tk1 | 0.11535 | -0.47299823 |
| Tk1 | 0.07799 | -0.80604716 |
| Slc4a9 | 0.00144 | -1.24811894 |
| Phkg1 | 0.71383 | -0.07099581 |
| Akna | 0.03172 | -1.26017723 |
| Coq6 | 0.01192 | -0.99380805 |
| Sacs | 0.00144 | -1.24466058 |
| Mrps21 | 0.0372 | -0.58059517 |
| Gm9992 | 0.13153 | -0.524265 |
| Mgst3 | 0.01627 | -1.24291392 |
| Olfr1275 | 0.00145 | -1.31323225 |
| Anxa3 | 0.03887 | 0.42135885 |
| Tbca | 0.21588 | -0.23609121 |
| Sf3b3 | 0.08841 | -0.30577833 |
| Sf3b5 | 0.00232 | -1.07399155 |
| Wdr89 | 0.0232 | -0.51942595 |
| Gm21319 | 0.02093 | -1.84517172 |
| Pdlim5 | 0.0795 | 0.34483506 |
| Gpr3 | 0.96861 | -0.01003684 |
| Fpr3 | 0.0059 | -1.10169949 |
| Siva1 | 0.00483 | -0.998986 |
| Flad1 | 0.00314 | -0.83688924 |
| Myt1l | 0.1613 | -0.84021482 |
| Pus1 | 0.01296 | -0.76460313 |
| Hfe | 0.18019 | -0.26416657 |
| Zfp114 | 0.00589 | -0.89394135 |
| Traf7 | 0.0107 | -0.59055141 |
| Cd209d | 0.00241 | -1.42784012 |
| Ppip5k1 | 0.02823 | 0.89576107 |
| LOC102639013 | 0.70367 | 0.28051068 |
| Kcnd3os | 0.82463 | -0.08852009 |
| Josd2 | 0.3525 | -0.39289455 |
| Dvl2 | 0.01342 | -0.53935054 |
| Ppara | 0.0748 | -0.58572142 |
| Adam15 | 0.00144 | -1.3716835 |
| Rab15 | 0.57137 | -0.33519368 |
| Gm5878 | 0.00144 | 2.64718068 |
| Adtrp | 0.00144 | -1.27165604 |
| Vmn1r52 | 0.0747 | -1.19276846 |
| Vmn1r48 | 0.00412 | -1.16485373 |
| Atp5o | 0.0081 | -0.69807084 |
| 2810407A14Rik | 0.0088 | -1.08115135 |
| Enpp2 | 0.11072 | -0.4163107 |
| Enpp4 | 0.0045 | 0.77899676 |
| Sdsl | 0.00457 | -0.71695801 |
| Cabp5 | 0.00206 | -1.40263095 |
| Parp3 | 0.04469 | -0.38656571 |
| Scaf11 | 0.00974 | 0.8571471 |
| Snapin | 0.61155 | -0.10485194 |
| Arid4a | 0.10043 | 0.4019526 |
| Rps7 | 0.13819 | -0.33754226 |
| Slc15a4 | 0.01281 | -0.76967551 |
| 4932430I15Rik | 0.52933 | -0.34699059 |
| Vmn1r224 | 0.00144 | -1.27699683 |
| Park2 | 0.02806 | -0.43705254 |
| Srrm2 | 0.54652 | -0.77488769 |
| Chrm2 | 0.71435 | -0.07870359 |
| Chrm1 | 0.00144 | -1.29085005 |
| Sdf4 | 0.43187 | -0.20427937 |
| Ldlrad2 | 0.00144 | -1.27165604 |
| Abcf1 | 0.1559 | 0.37014292 |
| Airn | 0.68973 | 0.11947149 |
| Kcng1 | 0.00144 | -1.29085005 |
| Gm2016 | 0.12547 | 0.36665264 |
| Hormad2 | 0.0959 | -2.66540847 |
| Slc7a14 | 0.00144 | -1.28116048 |
| Prss44 | 0.61429 | -0.18278271 |
| Agpat1 | 0.00144 | -1.36093822 |
| Prkar2b | 0.35452 | 0.30599681 |
| 4930547M16Rik | 0.00144 | -1.30784197 |
| Uqcc2 | 0.00145 | -1.07705441 |
| Fam19a1 | 0.00144 | -1.20052149 |
| Lrrc69 | 0.75637 | -0.10144087 |
| Lrrc49 | 0.03655 | -0.90547877 |
| Fam136a | 0.93513 | 0.01803093 |
| Olfr406 | 0.2947 | -0.43366742 |
| Rnd1 | 0.00151 | 1.34216858 |
| Trappc1 | 0.33798 | -0.28616688 |
| Trappc1 | 0.8053 | 0.14396922 |
| Ube2v2 | 0.00144 | 1.47908569 |
| Rxfp1 | 0.00144 | -1.25118919 |
| Maml1 | 0.70625 | -0.06563286 |
| Sh3bgrl2 | 0.78151 | 0.12739096 |
| Polr3c | 0.98876 | 0.00484963 |
| Ldlrad3 | 0.02973 | -0.5145776 |
| Abcb5 | 0.00144 | -1.26428512 |
| Olfr688 | 0.00144 | -1.27454685 |
| Gm694 | 0.00144 | -1.61405477 |
| Tmbim7 | 0.00144 | -1.28116048 |
| Nrxn3 | 0.00144 | -1.27165604 |
| Nrxn1 | 0.47271 | 0.4147617 |
| Krt78 | 0.31922 | -0.47890385 |
| Slc31a2 | 0.00578 | 0.96741362 |
| Gm11497 | 0.00145 | -1.28488688 |
| Adprm | 0.27383 | 0.20849186 |
| Cd84 | 0.34869 | 0.55812086 |
| Ttpal | 0.13252 | 0.40395899 |
| Ttpal | 0.00689 | 0.8576485 |
| Tcf4 | 0.37318 | 0.24350027 |
| Pira2 | 0.64314 | 0.46431049 |
| Defa-ps1 | 0.0212 | -1.1799076 |
| Gm15299 | 0.01686 | -1.7620697 |
| Rbm6 | 0.00159 | 1.37982501 |
| Fbxo44 | 0.15866 | -0.38586737 |
| 2810408I11Rik | 0.30319 | -0.86161176 |
| Ephb3 | 0.01852 | -0.61251843 |
| M6pr | 0.0416 | -0.40564113 |
| Alg8 | 0.59126 | -0.08398179 |
| Fam178b | 0.00145 | -1.2388121 |
| Fam178b | 0.01388 | -0.53296749 |
| Med8 | 0.26045 | -0.20595057 |
| Sly | 0.00144 | -1.31495282 |
| E330014E10Rik | 0.00144 | -1.29878664 |
| Gm3285 | 0.56263 | -0.48420863 |
| Hspa4 | 0.01305 | 0.70148558 |
| Tulp2 | 0.00275 | -0.86254688 |
| Gm9495 | 0.162 | -0.74168885 |
| Chst9 | 0.00144 | -1.27730451 |
| Chst2 | 0.07621 | -0.53862738 |
| Chst5 | 0.00381 | -1.09251133 |
| 2610042L04Rik | 0.00144 | 1.98581949 |
| Bdkrb2 | 0.00144 | -1.27165604 |
| Olfr1368 | 0.37874 | 0.16924125 |
| Foxa1 | 0.00145 | -1.2388121 |
| Rab17 | 0.42355 | -0.23143378 |
| Scamp3 | 0.02531 | -0.73132592 |
| Tnfaip3 | 0.01905 | 0.67551018 |
| Iqcf4 | 0.00144 | -1.29051721 |
| Iqcf6 | 0.00144 | -1.30713006 |
| Ppp2r3a | 0.03241 | 0.49272647 |
| Pglyrp4 | 0.00144 | -1.26301121 |
| A730020M07Rik | 0.10663 | 0.34918637 |
| Gm10632 | 0.00493 | -1.5493202 |
| Ms4a15 | 0.07291 | -2.25595485 |
| Cela3a | 0.03349 | -0.93578497 |
| Alpk3 | 0.28303 | -0.40849002 |
| Mast4 | 0.03113 | -0.48948399 |
| Rundc3b | 0.00144 | -1.28375735 |
| Cacfd1 | 0.00144 | -0.94716844 |
| Poli | 0.04267 | 0.49825703 |
| Pdia6 | 0.65177 | 0.08723807 |
| Timd2 | 0.00145 | -1.22376446 |
| Gm6548 | 0.49398 | -0.12818953 |
| Ivl | 0.00525 | -1.2005433 |
| Olfr5 | 0.00144 | -1.20555712 |
| Pirb | 0.70502 | -0.15189316 |
| Pirb | 0.04485 | -0.49054678 |
| Olfr1099 | 0.00144 | -1.25118919 |
| Zfr2 | 0.57701 | 0.09649322 |
| Rgs21 | 0.24982 | -0.29921732 |
| Gm5415 | 0.03432 | -0.83293529 |
| St6galnac6 | 0.03247 | -1.03754295 |
| St6galnac6 | 0.00686 | -0.7538651 |
| 4930415L06Rik | 0.00144 | -1.26934956 |
| Poc1a | 0.00581 | -0.85052076 |
| Gabrg3 | 0.20957 | -0.51555482 |
| Gng4 | 0.1885 | -0.3124702 |
| Kdm4c | 0.00144 | 1.27591629 |
| Itsn2 | 0.00144 | 1.63126339 |
| Nefh | 0.00144 | -1.20555712 |
| Fam20b | 0.11776 | -0.27688482 |
| Zc3h3 | 0.02352 | -0.50588957 |
| Foxg1 | 0.00548 | -1.41875196 |
| Myo9a | 0.36166 | -0.3885991 |
| Chuk | 0.00145 | 1.33034606 |
| Ppp2r1b | 0.00144 | -1.28898079 |
| Ppp2r1b | 0.16356 | 0.58189084 |
| Xkr8 | 0.00297 | -0.95492635 |
| Fmr1os | 0.10961 | -0.88697657 |
| Ctbp1 | 0.0048 | -0.75533433 |
| Gm6983 | 0.00696 | -0.65924514 |
| Dyrk4 | 0.03396 | -0.9889904 |
| Sepw1 | 0.00327 | -0.76448139 |
| Morf4l1-ps1 | 0.11009 | 1.11329012 |
| Xiap | 0.00144 | 1.1001197 |
| Olfr1303 | 0.11539 | -0.84733679 |
| Ankrd23 | 0.99149 | -0.00615014 |
| Fam73a | 0.00667 | 0.91804397 |
| 0610009B22Rik | 0.19226 | 0.53020251 |
| Lrrc14b | 0.06454 | 0.33809673 |
| Cask | 0.00175 | 1.64012049 |
| Cask | 0.09218 | 0.38346365 |
| Morc2a | 0.06728 | -0.31391266 |
| Scd3 | 0.42217 | 0.35636529 |
| Pcm1 | 0.01466 | 0.66110036 |
| Synm | 0.04634 | -0.72300598 |
| Rpl6 | 0.02004 | -0.487406 |
| Pgap2 | 0.00338 | -0.75337917 |
| A830080D01Rik | 0.00752 | 2.14688045 |
| Id1 | 0.0019 | -1.08776961 |
| Gm14393 | 0.00567 | 2.24077071 |
| Tmx2 | 0.56817 | -0.11954846 |
| Ushbp1 | 0.26778 | -0.24579454 |
| Olfr1130 | 0.00144 | -1.26552682 |
| Setd4 | 0.63764 | 0.11385378 |
| Iqsec1 | 0.02741 | -0.97366552 |
| Tia1 | 0.97823 | -0.00525577 |
| Olfr1382 | 0.0107 | -0.80720027 |
| Hyal4 | 0.00144 | -1.33632959 |
| Hyal6 | 0.36903 | -0.44567833 |
| Psat1 | 0.00533 | -0.71466545 |
| Rnf208 | 0.48472 | 0.33461011 |
| Zscan30 | 0.05815 | 1.21774908 |
| Zfp280c | 0.00267 | 1.11380105 |
| Gm362 | 0.00144 | -1.31884045 |
| Crlf1 | 0.00389 | -1.28761446 |
| Cacng4 | 0.00144 | -1.37833222 |
| Lamtor1 | 0.00672 | -0.96670692 |
| Gm6637 | 0.45055 | 0.22711784 |
| Akap13 | 0.00716 | -0.81346349 |
| Ly6g6d | 0.01283 | -0.72179737 |
| Tex21 | 0.00229 | -1.27300595 |
| BC049762 | 0.05774 | -1.77276855 |
| Eif1ad | 0.01834 | -0.55389095 |
| Gsx2 | 0.0073 | -1.17129313 |
| Cd200 | 0.07432 | 0.62669101 |
| A3galt2 | 0.67844 | 0.26568511 |
| Pih1d2 | 0.00813 | 1.23073735 |
| Nol4 | 0.00158 | -1.2446827 |
| Brsk1 | 0.09878 | -0.3246481 |
| Spink7 | 0.00144 | -1.29051721 |
| Olfr743 | 0.00145 | -1.2388121 |
| Sec61b | 0.02386 | -0.48853298 |
| Zfp518a | 0.86036 | -0.11356128 |
| Zfp568 | 0.1878 | -0.23656747 |
| Gm29707 | 0.04551 | -0.91232186 |
| Atp2c1 | 0.00489 | 0.93675268 |
| Bco2 | 0.96763 | 0.0163032 |
| Ccdc171 | 0.15718 | 0.53463204 |
| Psmc2 | 0.33649 | -0.16708551 |
| Golgb1 | 0.00155 | -1.08116757 |
| Golgb1 | 0.91776 | -0.0170214 |
| Snap25 | 0.15652 | 0.38722383 |
| Layn | 0.2346 | -0.24934085 |
| Gm128 | 0.05368 | -0.56451778 |
| Cbr4 | 0.12356 | 0.77571923 |
| Sult2b1 | 0.09672 | -0.8657173 |
| Lbx1 | 0.65935 | 0.2150807 |
| Lbx1 | 0.00403 | -1.35156863 |
| Rnf8 | 0.4987 | -0.10994675 |
| Psg22 | 0.33275 | -0.31447216 |
| Tbx1 | 0.02086 | -0.86691066 |
| Gm8479 | 0.08376 | 0.52509486 |
| Olfr1496 | 0.39681 | -0.42035763 |
| Gm31142 | 0.00144 | -1.4182713 |
| Gm4146 | 0.98596 | -0.00680942 |
| Mup20 | 0.00879 | -1.58012931 |
| Pgk2 | 0.88325 | 0.07873274 |
| Prl3d2 | 0.00144 | -1.25118919 |
| Ccdc88c | 0.05431 | -0.37915827 |
| AA066038 | 0.06833 | -0.71956438 |
| Pcdh11x | 0.00144 | -1.30017714 |
| Ppfia1 | 0.00758 | 0.70914283 |
| H2-K2 | 0.00503 | -0.94266899 |
| Gstk1 | 0.06072 | -0.51992862 |
| Tbcd | 0.16135 | -0.84823061 |
| Tbcd | 0.00144 | -1.29085005 |
| Gm2479 | 0.3208 | -0.44555287 |
| Tctex1d1 | 0.00589 | -1.11030563 |
| Sco1 | 0.91366 | 0.03829445 |
| Pvt1 | 0.87061 | -0.04961331 |
| Gpr142 | 0.01103 | -1.14573213 |
| Gpr132 | 0.61182 | -0.09336359 |
| 1700108F19Rik | 0.96709 | 0.05849046 |
| Upp2 | 0.00144 | -1.30713006 |
| Scaf1 | 0.00443 | -0.72627403 |
| Rorc | 0.09762 | -0.55303432 |
| Gm11634 | 0.76421 | -0.33404525 |
| Gm10787 | 0.00144 | -1.30713006 |
| Rps10 | 0.00144 | -1.28462557 |
| Gm11937 | 0.00424 | -0.81389226 |
| Serinc4 | 0.00767 | -0.6345995 |
| Nmu | 0.00144 | -1.28116048 |
| Tlr7 | 0.15968 | -0.40935602 |
| C920025E04Rik | 0.26953 | -0.53602826 |
| Catsper4 | 0.00144 | -1.30737044 |
| Plekhg2 | 0.95331 | 0.01225256 |
| Fcrlb | 0.02739 | -0.56157225 |
| D11Wsu47e | 0.08196 | -0.36567965 |
| Olfr669 | 0.00145 | -1.2388121 |
| Olfr769 | 0.00144 | -1.29085005 |
| Olfr569 | 0.00158 | -1.2446827 |
| Mtg2 | 0.0076 | -0.7223548 |
| Nup35 | 0.13671 | 0.31772629 |
| Cd209c | 0.00257 | -1.1333102 |
| 4930557B15Rik | 0.07046 | -0.87763815 |
| Fcrl5 | 0.04673 | -3.12467677 |
| Fcrl5 | 0.09819 | -0.30220337 |
| Nfkbil1 | 0.00153 | -0.8613994 |
| Zbed4 | 0.59274 | 0.08300962 |
| Gm10457 | 0.6192 | 0.2341942 |
| Ikbkg | 0.06938 | -0.5480935 |
| Cyp4a31 | 0.37413 | -1.04241097 |
| Ncam1 | 0.04251 | -0.66396167 |
| Olfr913 | 0.05629 | -1.01017398 |
| Olfr213 | 0.08324 | -2.11816266 |
| Pgbd1 | 0.00301 | 0.96626261 |
| Abcc12 | 0.04105 | -0.94116377 |
| Hemt1 | 0.03732 | -1.95737781 |
| Dlgap5 | 0.03031 | -1.02137678 |
| LOC105244782 | 0.1325 | 0.5371523 |
| Cdh22 | 0.10469 | -0.79037748 |
| Igf1os | 0.25072 | 0.51211989 |
| Prpmp5 | 0.09595 | -0.32485256 |
| Cct6b | 0.07147 | 0.54973463 |
| Nfkbid | 0.60205 | 0.10857389 |
| l7Rn6 | 0.27403 | 0.33661212 |
| Ankrd27 | 0.95265 | -0.010173 |
| ND5 | 0.11269 | 0.39336324 |
| Vmn1r197 | 0.0019 | 1.3209123 |
| Fance | 0.04294 | -0.61821087 |
| Agps | 0.00199 | 1.92620573 |
| P2rx3 | 0.01574 | -2.98405515 |
| Rpl36al | 0.04214 | -0.39525938 |
| Scml2 | 0.24095 | -0.39606467 |
| Slc9a1 | 0.00733 | -0.70832915 |
| Vmn2r69 | 0.00144 | -1.26560808 |
| Araf | 0.00346 | 1.18358487 |
| LOC102640359 | 0.26226 | -0.3307863 |
| Lmo3 | 0.00144 | -1.22925777 |
| Gm10629 | 0.4613 | 0.49967295 |
| Pamr1 | 0.02785 | -0.56800551 |
| Agrp | 0.00858 | -1.42955407 |
| Efna2 | 0.00522 | -0.99189161 |
| Stox1 | 0.62625 | 0.18386284 |
| H2-K1 | 0.0028 | -1.0535285 |
| Prickle4 | 0.02785 | -1.32759156 |
| Kat2a | 0.23328 | -0.29882467 |
| Apbb3 | 0.92661 | -0.01545562 |
| Aggf1 | 0.05382 | 0.37033223 |
| Pld2 | 0.02839 | -0.44092569 |
| Pld2 | 0.05158 | 0.84650174 |
| Rab22a | 0.37303 | -0.20821018 |
| Rasl10b | 0.7136 | 0.21339014 |
| Sh3bp5 | 0.53278 | 0.11617648 |
| Sh3bp5 | 0.25945 | 0.1854063 |
| Phtf1 | 0.07973 | 0.52209398 |
| Phtf2 | 0.06833 | 0.59970114 |
| Zfp26 | 0.00314 | -1.14703327 |
| Erp29 | 0.01195 | -0.63797318 |
| Pde3a | 0.32655 | -0.17614829 |
| Olfr1097 | 0.08649 | -0.95081427 |
| Olfr1097 | 0.00144 | -1.30017714 |
| Gm4755 | 0.0249 | -1.55887664 |
| Ppih | 0.06628 | -0.78428665 |
| Ly6h | 0.01065 | -0.75872396 |
| Mettl6 | 0.15447 | 0.27098176 |
| Gm3373 | 0.84905 | 0.04024521 |
| Allc | 0.07382 | -0.86014901 |
| Oxgr1 | 0.00145 | -1.33072139 |
| Pisd-ps1 | 0.01429 | 0.54032962 |
| Ifna16 | 0.16024 | -1.05324138 |
| Tm6sf2 | 0.00517 | -0.72350335 |
| Atat1 | 0.12848 | -0.51648089 |
| Map3k7 | 0.00144 | 0.99085868 |
| Fam160a2 | 0.02252 | -0.87613356 |
| Otx2 | 0.03414 | -0.83601401 |
| Rbmy | 0.02549 | -1.65297958 |
| 4930515G16Rik | 0.00144 | -1.31495282 |
| Cbfb | 0.0059 | 0.84236086 |
| Themis2 | 0.238 | -0.29341063 |
| Sprr2a2 | 0.03534 | -0.97392494 |
| Sprr2a2 | 0.00144 | -1.26934956 |
| Ppil2 | 0.28303 | -0.18090806 |
| 4930588K23Rik | 0.05023 | -1.20633438 |
| Cd82 | 0.35558 | -0.19119575 |
| Cd82 | 0.00144 | -1.12799209 |
| Zfp454 | 0.46469 | -0.45346741 |
| Zfp454 | 0.00199 | 1.38041977 |
| Mgarp | 0.547 | -0.19116379 |
| Eif2ak3 | 0.27063 | 0.24254722 |
| Gm4786 | 0.05685 | -0.54147841 |
| Fgf2 | 0.00145 | -1.25646236 |
| Rpl27 | 0.00865 | -0.88212828 |
| Rpl27a | 0.00226 | -0.94177185 |
| Dennd4a | 0.03452 | -0.5167772 |
| Spock1 | 0.20431 | -0.69377703 |
| Hp | 0.11576 | -1.2588309 |
| Scgb1b30 | 0.04461 | -2.03123602 |
| Cdk2ap1 | 0.02237 | -0.6635538 |
| Smn1 | 0.37497 | 0.22417185 |
| Hbb-bh2 | 0.00368 | -1.10379914 |
| Ankrd55 | 0.00346 | -1.22679402 |
| LOC102637192 | 0.08294 | 1.00796262 |
| 4931440P22Rik | 0.01421 | -0.62675052 |
| D17Zt10e | 0.15138 | 0.78715215 |
| Olfml1 | 0.02164 | -0.62744832 |
| Fam166b | 0.00144 | -1.27165604 |
| Rps2 | 0.20393 | -0.60086755 |
| Syt3 | 0.51948 | -0.20134613 |
| Hsp90ab1 | 0.01195 | -0.5821045 |
| Gm29258 | 0.00144 | -1.27165604 |
| Ggact | 0.10082 | -2.46710207 |
| Apool | 0.06261 | 0.40865216 |
| Suv420h1 | 0.00479 | 0.77196054 |
| Umodl1 | 0.00424 | -1.45284633 |
| Fggy | 0.29989 | 0.30743477 |
| Tlr11 | 0.00144 | -1.28266564 |
| Tlr13 | 0.40054 | -0.16408493 |
| Golph3l | 0.03019 | 0.45922213 |
| Ganab | 0.00561 | -0.63659094 |
| Wscd1 | 0.01298 | -1.24924692 |
| Msantd3 | 0.18183 | -0.35010036 |
| Slc29a4 | 0.00144 | -1.00827376 |
| Tmem97 | 0.00493 | -0.72320599 |
| Mzf1 | 0.00737 | -0.90655034 |
| Klhl14 | 0.00149 | -1.22582153 |
| Slc44a1 | 0.06358 | -0.37695621 |
| Zfx | 0.01328 | 0.69028274 |
| Gm6578 | 0.25386 | 0.30249533 |
| Larp4b | 0.7727 | 0.06363175 |
| Olfr1173 | 0.26746 | -0.5593573 |
| Ankrd23 | 0.65546 | -0.22910034 |
| Osbpl7 | 0.21424 | -0.91061128 |
| Bfar | 0.26619 | 0.35638666 |
| Gjc2 | 0.00191 | -1.04165971 |
| Ccdc83 | 0.00144 | -1.27165604 |
| Clcn3 | 0.00389 | -0.71615602 |
| Clcn3 | 0.30902 | 0.21182347 |
| Spatc1l | 0.05701 | -0.95567832 |
| 4933416C03Rik | 0.00144 | -1.27992547 |
| Snhg11 | 0.01588 | -0.99849484 |
| Katnal1 | 0.02102 | -0.50456854 |
| Krtap4-8 | 0.00314 | -0.74885921 |
| Serpinb6c | 0.67066 | 0.06902723 |
| 4930519F24Rik | 0.00144 | -1.29746368 |
| Mafg | 0.03308 | -0.40203367 |
| Trav12-1 | 0.00145 | -1.22376446 |
| Pop7 | 0.00396 | -1.09824757 |
| Srr | 0.75963 | -0.04989438 |
| Coq2 | 0.61904 | -0.16555526 |
| Cubn | 0.51893 | 0.18519792 |
| Cutal | 0.00192 | -1.0130454 |
| Zfp809 | 0.00581 | 0.77178683 |
| Arhgdia | 0.00144 | -1.03872852 |
| Rps9 | 0.00172 | -1.02346092 |
| Ccp110 | 0.00379 | 2.13000949 |
| Gm6600 | 0.00144 | -1.29085005 |
| Oog4 | 0.00145 | -1.2388121 |
| Ypel3 | 0.82965 | -0.06323024 |
| Ufd1l | 0.62206 | 0.08183469 |
| LOC102636563 | 0.00144 | -1.28116048 |
| Calm1 | 0.60328 | -0.08603583 |
| Fcho1 | 0.03792 | -0.61142933 |
| Ppih | 0.05691 | -0.39181228 |
| Gm4868 | 0.00144 | -1.29085005 |
| Capn5 | 0.37799 | -0.99438879 |
| Methig1 | 0.16774 | -0.62334107 |
| Mbtps1 | 0.00517 | -0.66350516 |
| Pdgfb | 0.00144 | -1.16181381 |
| Adcy5 | 0.03698 | -0.64580097 |
| Ankrd40 | 0.75046 | 0.1389751 |
| Lcn6 | 0.97703 | -0.02046299 |
| Tspan7 | 0.02651 | -0.42871516 |
| Uxs1 | 0.05899 | 0.37327438 |
| Slc37a4 | 0.65798 | -0.12410142 |
| 1700029I15Rik | 0.00144 | -1.28898079 |
| Fgf1 | 0.74683 | -0.05291301 |
| Meis2 | 0.02425 | -0.54531838 |
| Unc13a | 0.00207 | -1.15463903 |
| Gm30810 | 0.03429 | -0.89782899 |
| Rell2 | 0.10066 | -0.38734593 |
| Pla2g1b | 0.012 | -1.58714596 |
| Mylk2 | 0.00671 | -1.2138988 |
| Sel1l2 | 0.13423 | -0.77803917 |
| Slfn5 | 0.00144 | -1.14180884 |
| Mmp27 | 0.00145 | -1.2388121 |
| Adpgk | 0.10951 | -0.61720272 |
| Wdr83os | 0.00642 | -0.81313975 |
| Vmn2r15 | 0.00144 | -1.25118919 |
| 9430060I03Rik | 0.00144 | -1.48336017 |
| Gata5 | 0.00258 | -1.18734797 |
| Gata1 | 0.00144 | -1.50593498 |
| Tmem253 | 0.8514 | -0.04330264 |
| Sparc | 0.00145 | -0.98027579 |
| Nbas | 0.01349 | -0.83902207 |
| Eogt | 0.01438 | 0.62090303 |
| Mpped1 | 0.03508 | -0.82781308 |
| Mpped1 | 0.06374 | -0.59138276 |
| Vmn2r42 | 0.00317 | -1.11935153 |
| Piwil4 | 0.22589 | -0.4536554 |
| Cspg5 | 0.08509 | -0.64166573 |
| Tvp23b | 0.00413 | 0.90819143 |
| Higd1c | 0.00144 | -1.26560808 |
| Tnfrsf14 | 0.00558 | -1.65287051 |
| Dio1 | 0.83055 | 0.25622482 |
| Vstm2b | 0.00144 | -1.29085005 |
| 1700030C10Rik | 0.80995 | 0.07906467 |
| Cym | 0.63673 | 0.48432533 |
| Olfr1311 | 0.79178 | -0.16515478 |
| Amot | 0.01288 | 0.64285331 |
| 1700055D18Rik | 0.13451 | 0.56877868 |
| Vmn1r70 | 0.08148 | -0.31389623 |
| Olfr560 | 0.15207 | -0.48647721 |
| Aarsd1 | 0.00173 | -0.8631942 |
| Tmem235 | 0.05186 | -0.88534853 |
| Duoxa1 | 0.64362 | -0.13321657 |
| Lcn3 | 0.00159 | -1.26431393 |
| Ucn3 | 0.00144 | -1.30713006 |
| Eif4a1 | 0.23676 | -0.18887155 |
| Rcn3 | 0.00145 | -0.96918943 |
| Eif4g1 | 0.00959 | -0.57640961 |
| Mapkap1 | 0.52597 | 0.151819 |
| Itpripl2 | 0.69695 | -0.09318102 |
| Mapk14 | 0.02761 | -0.4473154 |
| Gm6225 | 0.26761 | 0.53015405 |
| H2-M10.1 | 0.11842 | -2.98416957 |
| Tmem239 | 0.02861 | -0.50780659 |
| Hes1 | 0.22812 | -0.30498687 |
| Slc25a25 | 0.17768 | -0.33788996 |
| Ccdc90b | 0.00446 | 0.96485467 |
| Kremen1 | 0.50525 | -0.19874907 |
| Gm10693 | 0.19643 | -0.66064624 |
| Xlr4b | 0.5562 | 0.59744095 |
| Dcun1d4 | 0.42466 | 0.21169241 |
| Tmem44 | 0.60052 | -0.1253846 |
| Il22ra2 | 0.00144 | -1.30017714 |
| Arhgap42 | 0.45569 | -0.33771791 |
| Gm805 | 0.00144 | -1.37877024 |
| Gm8165 | 0.0018 | -1.21153109 |
| Mysm1 | 0.00144 | 2.17128796 |
| Enthd2 | 0.01798 | -0.52683963 |
| Csmd2os | 0.11429 | -2.71813463 |
| Btnl10 | 0.00145 | -1.2388121 |
| Btnl10 | 0.31171 | 0.35386255 |
| Gdnf | 0.17104 | -0.52864478 |
| 2900008C10Rik | 0.07873 | -0.65880391 |
| LOC102643033 | 0.49059 | 0.16233869 |
| Sigmar1 | 0.00983 | -0.65218652 |
| Pnma3 | 0.21881 | -2.17279184 |
| Gmfb | 0.03197 | 0.79346574 |
| Rnf224 | 0.08828 | -0.45605212 |
| Plcxd2 | 0.83591 | 0.10837684 |
| Grk1 | 0.00325 | -1.31322145 |
| Spag6l | 0.0785 | -2.83353792 |
| Olfr1425 | 0.00145 | -1.2388121 |
| Sirt7 | 0.23739 | -0.1933068 |
| Sirt7 | 0.01165 | 0.54110625 |
| Rassf5 | 0.15998 | -0.29182514 |
| Nme6 | 0.30442 | -0.45359753 |
| Stmn4 | 0.15272 | -0.38907965 |
| Setdb2 | 0.00144 | 1.73544519 |
| Ano7 | 0.00144 | -1.29746368 |
| Gpkow | 0.00773 | 0.71471767 |
| Olfr901 | 0.42214 | -0.73668029 |
| Rsg1 | 0.3196 | -0.27296588 |
| Rbms3 | 0.11514 | -0.68211071 |
| Hmgxb4 | 0.06882 | 0.47201744 |
| Fam83b | 0.00158 | -1.17515383 |
| Olfr506 | 0.00144 | -1.31495282 |
| Hipk4 | 0.00151 | -0.88368484 |
| Ppp4r1 | 0.47502 | 0.1125248 |
| G630025P09Rik | 0.01634 | -0.77380532 |
| Gpc5 | 0.06345 | -1.45981352 |
| Gm10845 | 0.87717 | 0.02551969 |
| Ehd1 | 0.01566 | -0.64911992 |
| 4921531P14Rik | 0.15227 | 0.40970161 |
| Defa-rs12 | 0.18303 | -0.78338184 |
| Chp1 | 0.63525 | -0.08641075 |
| Mlxipl | 0.09303 | 0.68279155 |
| 5830410O09Rik | 0.00144 | -1.25118919 |
| Scgb2b3 | 0.00585 | -1.56044901 |
| Tmem242 | 0.77709 | -0.14095656 |
| Stk32a | 0.5554 | -0.16183257 |
| Hjurp | 0.02368 | 0.95121675 |
| Rpl34-ps1 | 0.00154 | -0.90804203 |
| Eif5a | 0.69072 | -0.20021362 |
| Zfp953 | 0.1123 | 0.60783576 |
| Mgrn1 | 0.04266 | -0.70855507 |
| Hrh1 | 0.57673 | -0.36539627 |
| Gm14025 | 0.13912 | -0.54877927 |
| Zfp74 | 0.00145 | 1.24459998 |
| Zfp74 | 0.41024 | 0.23478934 |
| Vmn1r4 | 0.21983 | -0.74270202 |
| Lst1 | 0.00237 | -0.94538549 |
| Gpr84 | 0.03067 | -1.10929245 |
| Gpr82 | 0.07162 | -0.88282181 |
| Prdm13 | 0.00244 | -1.16878422 |
| Rpl17-ps8 | 0.02328 | -0.45786442 |
| 9130019O22Rik | 0.63772 | 0.10843956 |
| Gm10796 | 0.2601 | 0.41997095 |
| Aoc2 | 0.02642 | 1.07067649 |
| Zfp869 | 0.98416 | -0.00501186 |
| Ccdc171 | 0.04611 | -1.29577298 |
| Asb10 | 0.22976 | -0.49420759 |
| Gm10532 | 0.86772 | -0.04702081 |
| Cyp2b10 | 0.52244 | -0.28826598 |
| Olfr1058 | 0.00144 | -1.29051721 |
| Hsf4 | 0.23593 | -0.2656375 |
| Siah3 | 0.00577 | -1.10847967 |
| 1810037I17Rik | 0.20475 | -0.362392 |
| Sorcs1 | 0.02007 | -1.05815398 |
| Iglon5 | 0.01448 | -0.85924477 |
| Dusp15 | 0.10275 | -0.40883618 |
| Gm8210 | 0.00254 | -0.84006932 |
| Tespa1 | 0.00144 | -1.27165604 |
| 2810416G20Rik | 0.00144 | -1.20682437 |
| 4930555G01Rik | 0.00144 | -1.26560808 |
| Stard9 | 0.02531 | 0.8599388 |
| Magi2 | 0.29922 | -0.32014275 |
| Gm3961 | 0.00144 | -1.20555712 |
| Padi6 | 0.22576 | -0.39688824 |
| Pramef8 | 0.25064 | -0.1962975 |
| 3110039I08Rik | 0.27782 | -0.36370208 |
| Cxcl11 | 0.30496 | 0.33543723 |
| Kif6 | 0.0039 | -0.90038923 |
| Fgfbp3 | 0.24398 | 0.20448779 |
| Gm5460 | 0.00276 | -1.15746563 |
| Mms22l | 0.02641 | -0.52535989 |
| D4Ertd617e | 0.00596 | 1.64159962 |
| Ccdc7 | 0.00144 | -1.30017714 |
| Fam96a | 0.28897 | 0.28077023 |
| Mpp6 | 0.00167 | 0.87069143 |
| Znf512b | 0.0282 | -0.47887574 |
| Slc13a5 | 0.6643 | -0.25394675 |
| Slc10a5 | 0.00161 | -1.73810637 |
| 9930021J03Rik | 0.00449 | 1.27925852 |
| Slc15a5 | 0.00144 | -1.26560808 |
| Bphl | 0.70143 | 0.18499381 |
| Gm572 | 0.09301 | -0.72415789 |
| Loxl4 | 0.00144 | 1.33071263 |
| Klc1 | 0.19997 | -0.240142 |
| Rpl28 | 0.00144 | -1.00781857 |
| Fgfr1op | 0.15243 | 0.34221338 |
| 1700001G01Rik | 0.00144 | -1.27730451 |
| Tdg | 0.00163 | -1.23019144 |
| AA465934 | 0.00264 | -1.05992277 |
| Cops7a | 0.96716 | -0.02116081 |
| Mtm1 | 0.00317 | 1.09507069 |
| Mtm1 | 0.00495 | -0.74027806 |
| Luzp4 | 0.00144 | -1.30713006 |
| Gm10071 | 0.00395 | -1.67198051 |
| Tmem8b | 0.01758 | -0.61398294 |
| 2300003K06Rik | 0.00277 | -1.52311644 |
| LOC102642069 | 0.00149 | -1.2703832 |
| Stmn1 | 0.48695 | -0.18043931 |
| Ankmy1 | 0.22231 | -1.39807612 |
| Cmtr1 | 0.01562 | -0.61820679 |
| Mrto4 | 0.00224 | -0.90349911 |
| Gm27230 | 0.00144 | -1.29051721 |
| Trpm1 | 0.00147 | 2.95401322 |
| Trpm1 | 0.00772 | -1.51731123 |
| Tfg | 0.03138 | 0.41904272 |
| Tmc1 | 0.00144 | -1.29085005 |
| Ubac1 | 0.10423 | -0.46225411 |
| Zfp617 | 0.00288 | 0.94737224 |
| Zfp607 | 0.01325 | 1.18162244 |
| Zfp677 | 0.00144 | 3.08836383 |
| Fam89a | 0.01402 | -0.55966696 |
| Grhpr | 0.01423 | -0.94111235 |
| 4930480E11Rik | 0.37089 | -0.1785684 |
| Slc12a4 | 0.00663 | -0.68195235 |
| Hnmt | 0.00144 | 2.43295088 |
| Msx2 | 0.16976 | -0.43981433 |
| Atmin | 0.14365 | -0.27733017 |
| Llgl2 | 0.01612 | -0.93471273 |
| Gm9258 | 0.00423 | -0.71594435 |
| Apol9a | 0.00144 | -1.07810739 |
| Colq | 0.01163 | -0.89127358 |
| Rnf26 | 0.03418 | -0.6694206 |
| Bag1 | 0.01678 | -0.49085966 |
| Rag1 | 0.00144 | -1.29049509 |
| Cdc42se1 | 0.00607 | -0.93923911 |
| Rpe65 | 0.673 | -0.38721913 |
| Magea1 | 0.00144 | -1.27699683 |
| Gm6647 | 0.07263 | -0.78079231 |
| Exosc9 | 0.10597 | 0.34763711 |
| Gimap6 | 0.84548 | -0.05252335 |
| Lilra6 | 0.61349 | -0.20833239 |
| Akr1e1 | 0.2835 | 0.18422907 |
| Gm3045 | 0.00145 | -1.25646236 |
| Arsg | 0.75857 | 0.07329031 |
| Ccdc138 | 0.02495 | -0.52323514 |
| Exoc4 | 0.93626 | 0.02450905 |
| Cyp4a12a | 0.04376 | -1.3543366 |
| Vmn2r68 | 0.00144 | -1.28116048 |
| Hs3st6 | 0.00144 | -1.42170513 |
| Pla2g3 | 0.04994 | 0.61384562 |
| 2610020H08Rik | 0.0051 | 1.51362537 |
| Vmn2r76 | 0.90596 | -0.13602448 |
| A930007A09Rik | 0.65821 | 0.17142092 |
| Psmg2 | 0.55424 | -0.08904827 |
| Psmg2 | 0.98617 | 0.00465715 |
| Arl6ip6 | 0.02364 | -0.54725505 |
| Aoah | 0.32805 | 0.40119408 |
| Nynrin | 0.08483 | -0.70767954 |
| 1010001N08Rik | 0.76799 | -0.15661847 |
| Rad50 | 0.017 | 0.62224624 |
| Acd | 0.03941 | -0.43011221 |
| Zcchc4 | 0.01453 | 0.72226913 |
| Sgk2 | 0.00145 | -1.25646236 |
| Trim37 | 0.93748 | 0.01638952 |
| Mbtd1 | 0.99826 | -0.00174314 |
| Mbtd1 | 0.0183 | -0.85878675 |
| Aoc2 | 0.82725 | -0.0563668 |
| Gm5409 | 0.91765 | 0.1516619 |
| Disc1 | 0.22561 | 0.50003771 |
| Nkx2-9 | 0.20798 | -0.58117588 |
| Sh3bp2 | 0.0667 | -0.5273923 |
| Gbp11 | 0.27645 | -1.5786115 |
| 2310075C17Rik | 0.07224 | -0.62967257 |
| Pde5a | 0.01115 | -0.82047193 |
| Tmem27 | 0.01588 | -0.99849484 |
| Glrp1 | 0.04793 | 0.53736788 |
| Tmem196 | 0.88796 | 0.0605356 |
| Pigq | 0.00418 | -0.85897664 |
| Hsd17b10 | 0.05575 | -0.65472331 |
| Gm15506 | 0.01978 | -0.51083169 |
| Mpl | 0.00904 | -1.60687672 |
| Spats1 | 0.7586 | 0.10158937 |
| Gm12576 | 0.32966 | 0.26977167 |
| C1s1 | 0.05148 | 0.51505105 |
| Pls1 | 0.6236 | -0.20599252 |
| LOC102641028 | 0.00234 | 1.60207797 |
| Polm | 0.00413 | -0.96389876 |
| Wfdc8 | 0.00144 | -1.26560808 |
| Wfdc8 | 0.00144 | -1.27165604 |
| Mccc2 | 0.8921 | 0.05967649 |
| Rfwd2 | 0.0058 | 0.63812489 |
| Adig | 0.00353 | -1.36445646 |
| Dpysl3 | 0.00144 | -1.04644446 |
| Vgll2 | 0.00218 | -1.88044309 |
| Clrn3 | 0.00144 | -1.29864962 |
| Rftn1 | 0.07565 | -0.3576139 |
| Gzmk | 0.00144 | -1.28116048 |
| Cyp2d40 | 0.94456 | 0.02046685 |
| Pbrm1 | 0.0578 | 0.38511649 |
| Pbrm1 | 0.04411 | 0.44367022 |
| Nlrp2 | 0.06559 | -0.86188542 |
| Fam161a | 0.03112 | 0.68021399 |
| Pigo | 0.01193 | -0.89037081 |
| Bzw2 | 0.02417 | 0.82488562 |
| Kif6 | 0.01014 | -0.66799736 |
| Krtap26-1 | 0.00144 | -1.28116048 |
| Aatk | 0.00144 | -1.34767375 |
| Sorcs2 | 0.00176 | -1.58440181 |
| Fech | 0.18839 | -0.26117752 |
| Prune2 | 0.08372 | -0.42623184 |
| Prune2 | 0.00144 | 3.56131405 |
| Nrtn | 0.00267 | -1.08702633 |
| Dkk4 | 0.00144 | -1.29878664 |
| Slc25a3 | 0.45377 | -0.22367747 |
| 5430427O19Rik | 0.00144 | -1.34945434 |
| Dnajc5b | 0.34928 | -0.35912486 |
| Bmp2 | 0.39304 | -0.27767847 |
| LOC102636777 | 0.0036 | -1.18318572 |
| LOC102636777 | 0.00144 | -1.29085005 |
| Ssmem1 | 0.00144 | -1.28116048 |
| Ptafr | 0.32388 | -0.56211317 |
| Nde1 | 0.31874 | 0.24301028 |
| Hyal1 | 0.89925 | -0.03055193 |
| Gm10002 | 0.0653 | -0.97873706 |
| Gna12 | 0.73868 | 0.18208204 |
| Scand1 | 0.00144 | -1.2620182 |
| Il15ra | 0.00144 | 1.15730555 |
| Gm595 | 0.00144 | -1.30017714 |
| Gm590 | 0.00144 | -1.30017714 |
| Mid1ip1 | 0.32676 | -0.37437621 |
| Gas2 | 0.00947 | -2.31786796 |
| Gas2 | 0.00965 | 1.01951149 |
| Hoxa11os | 0.01264 | 0.69145308 |
| Ttc38 | 0.09874 | -0.4352074 |
| 4930503E14Rik | 0.01468 | -1.67724289 |
| Ceacam12 | 0.00144 | -1.30017714 |
| Rdh1 | 0.03112 | 0.55282719 |
| Olfr855 | 0.09274 | -1.21616142 |
| Olfr655 | 0.0046 | -1.48550471 |
| Olfr555 | 0.00224 | -2.54181839 |
| Olfr255 | 0.01214 | -1.41135178 |
| Eya4 | 0.30804 | 0.459623 |
| Doxl2 | 0.03884 | -0.71231593 |
| 4930550L24Rik | 0.08881 | -2.53706976 |
| 1110004F10Rik | 0.18029 | 0.35483312 |
| LOC102635661 | 0.45491 | -0.42392413 |
| Xlr3a | 0.0025 | -1.14220008 |
| Il1rap | 0.00144 | -1.30713006 |
| 1700008O03Rik | 0.01729 | -1.04563207 |
| Ezh2 | 0.00237 | 1.26946844 |
| Gm3693 | 0.00159 | -1.26431393 |
| Lrp6 | 0.07874 | -0.44241354 |
| Cyp2d34 | 0.70127 | -0.18881414 |
| Cep350 | 0.5839 | 0.20807036 |
| Evx2 | 0.00144 | -1.0493253 |
| Gm3970 | 0.00793 | -1.20266358 |
| Arhgef1 | 0.00586 | -0.65965319 |
| Utp6 | 0.35173 | -0.25462666 |
| Nav2 | 0.93216 | -0.03758562 |
| Vmn1r76 | 0.00447 | -1.5268794 |
| Gm10354 | 0.82217 | 0.09539583 |
| Gm9758 | 0.66081 | -0.18550331 |
| Rn4.5s | 0.87288 | -0.05147337 |
| Six4 | 0.1447 | 0.57358927 |
| Tyw5 | 0.00149 | -1.28183002 |
| Ccnd3 | 0.00144 | -1.06019438 |
| Rbp3 | 0.85769 | 0.04146294 |
| Zmym5 | 0.00862 | 0.72434153 |
| Csnk1g2 | 0.00146 | -1.01670952 |
| Tomm20 | 0.41387 | -0.15521437 |
| Slc2a13 | 0.00227 | 1.66366386 |
| Klf7 | 0.0065 | 0.73884683 |
| Tom1 | 0.0187 | -0.72330477 |
| Eng | 0.00144 | -1.25082366 |
| Gtf2i | 0.00151 | -1.08412238 |
| Dhx15 | 0.00144 | 1.37872482 |
| Ung | 0.02322 | -0.5419137 |
| Gm867 | 0.00436 | -1.25788577 |
| Cnn3 | 0.8952 | -0.02976531 |
| Sun3 | 0.38992 | 0.23226407 |
| Gm14548 | 0.60019 | -0.13746212 |
| Lipo1 | 0.00144 | 1.57057938 |
| Sec62 | 0.00823 | 1.12049883 |
| Gm3234 | 0.00144 | -1.24865146 |
| Dnali1 | 0.03277 | -1.92245597 |
| Susd2 | 0.02063 | -0.79206022 |
| Gm10472 | 0.03303 | -1.16611144 |
| Gm10824 | 0.13802 | -0.70981809 |
| Gid8 | 0.09702 | -0.50976891 |
| Klhdc8b | 0.88069 | -0.10222045 |
| Pabpc5 | 0.00144 | -1.31495282 |
| Pabpc5 | 0.00175 | -1.6844835 |
| Map6d1 | 0.00386 | -0.94100212 |
| Phox2b | 0.00144 | -1.28116048 |
| Btbd9 | 0.8844 | -0.0249969 |
| Btbd9 | 0.97414 | -0.01995249 |
| Mtfr1 | 0.71818 | 0.09392337 |
| Ms4a4a | 0.00715 | 2.14883904 |
| Dio3os | 0.00338 | -1.85453221 |
| Ano10 | 0.93818 | -0.02723983 |
| Guf1 | 0.01941 | 1.27020578 |
| Pradc1 | 0.33981 | -0.27948644 |
| Rnaset2a | 0.89533 | -0.03158578 |
| Bola2 | 0.00144 | -1.03048868 |
| Gm7337 | 0.01911 | 0.94073114 |
| Ptp4a1 | 0.56343 | -0.10724982 |
| Dhps | 0.39848 | -0.34011595 |
| Acot5 | 0.01347 | -0.87522463 |
| Gm10033 | 0.31509 | 0.41455197 |
| Piezo2 | 0.19375 | 0.56413269 |
| Olfr118 | 0.00144 | -1.28116048 |
| Pmp22 | 0.0027 | -0.91916356 |
| Cdv3 | 0.02605 | -0.44257819 |
| Cdv3 | 0.01654 | 0.63145693 |
| Qser1 | 0.18407 | 0.36162083 |
| Fn3k | 0.0564 | 0.88909914 |
| Gm6880 | 0.05262 | -0.66183348 |
| Eddm3b | 0.00404 | -1.24750126 |
| 2410004P03Rik | 0.01183 | -0.775458 |
| Swi5 | 0.07294 | -0.37001692 |
| Swi5 | 0.05552 | -0.39932471 |
| Itga4 | 0.01119 | 0.58592163 |
| Itga7 | 0.00969 | -0.95535953 |
| Grik2 | 0.238 | -0.44706548 |
| Grik2 | 0.00144 | -1.29085005 |
| Wsb2 | 0.01626 | -0.61421426 |
| Gp1bb | 0.00144 | -1.24613586 |
| Olfr678 | 0.04099 | -1.0209542 |
| Olfr378 | 0.00144 | -1.29864962 |
| Gm4858 | 0.01983 | -0.9330192 |
| Olfr1154 | 0.00144 | -1.30713006 |
| Gigyf2 | 0.77727 | -0.05110078 |
| S100a2 | 0.01217 | -1.52097873 |
| Camp | 0.81449 | 0.05220138 |
| Clca3a1 | 0.48851 | -0.16270909 |
| Gpr34 | 0.46456 | 0.27799668 |
| Gpr35 | 0.00144 | -1.24613586 |
| Ift88 | 0.00255 | 0.9391111 |
| Clec4a2 | 0.00592 | -1.07650415 |
| Eed | 0.09098 | 0.36916911 |
| Adrbk2 | 0.06628 | 0.87132014 |
| Arhgap23 | 0.00185 | -0.83339518 |
| Pak6 | 0.00229 | -0.96315844 |
| 1700003M07Rik | 0.00144 | -1.3826329 |
| Pax8 | 0.11322 | -2.89501261 |
| Gm10724 | 0.00144 | -1.25118919 |
| 1700019P21Rik | 0.00214 | -1.28964092 |
| Timmdc1 | 0.00144 | -1.20304353 |
| Gm5088 | 0.00144 | -1.57314696 |
| Cmss1 | 0.15129 | -0.32551207 |
| Ppp1ca | 0.00435 | -0.84621184 |
| Ilkap | 0.00398 | -0.7344877 |
| Dio1 | 0.00229 | -1.21636428 |
| D930007P13Rik | 0.03298 | -1.65311934 |
| Tnfsf11 | 0.00144 | -1.28116048 |
| Bpifa6 | 0.00144 | -1.28116048 |
| Zfp345 | 0.00144 | -1.26560808 |
| D730002M21Rik | 0.00144 | -1.28116048 |
| Hspb6 | 0.06962 | -0.60824641 |
| Hspb1 | 0.01085 | -0.92101956 |
| Tnfaip8 | 0.07701 | 1.20120834 |
| Mboat2 | 0.80351 | -0.04296478 |
| Mat2b | 0.2178 | 0.29397549 |
| Pydc4 | 0.01217 | 1.0540293 |
| Rap1gap | 0.56787 | -0.22855192 |
| Rprd2 | 0.05936 | -0.49914369 |
| Gfra4 | 0.00144 | -1.59236045 |
| Gfra4 | 0.0849 | -0.44874145 |
| Zswim8 | 0.00144 | -1.00196412 |
| ND2 | 0.00561 | -0.65382561 |
| Rbck1 | 0.00661 | -0.62742538 |
| Zfp605 | 0.14512 | 0.79622925 |
| Nfkbiz | 0.00253 | 1.00439023 |
| Zfp664 | 0.00144 | -1.32697761 |
| Speer6-ps1 | 0.0017 | -1.24950944 |
| Psmb9 | 0.85656 | 0.25058678 |
| Mcat | 0.00516 | -0.98868024 |
| Gareml | 0.24528 | -0.61031391 |
| Jph4 | 0.00144 | -1.29085005 |
| Fam49a | 0.29852 | 0.25213444 |
| Fblim1 | 0.10743 | -0.34818596 |
| Olfr18 | 0.05455 | -0.4423503 |
| Ano1 | 0.66958 | 0.08872639 |
| Slc29a1 | 0.00409 | -0.93115581 |
| Gm626 | 0.00144 | -1.29085005 |
| Zfp235 | 0.56592 | 0.09690794 |
| Tmco1 | 0.14353 | 0.33874801 |
| Hbb-b2 | 0.34763 | -0.38607818 |
| Gm6999 | 0.00144 | -1.29085005 |
| C3 | 0.00903 | -0.77548927 |
| Gm15411 | 0.0475 | -0.76652009 |
| Rexo2 | 0.94318 | 0.01486286 |
| 4933427D06Rik | 0.02765 | -0.55194391 |
| Adamts17 | 0.90362 | 0.03780802 |
| Prkd3 | 0.00589 | -1.11030563 |
| Acot6 | 0.02357 | 0.9322627 |
| Acot5 | 0.2019 | -0.55242583 |
| Acot3 | 0.80242 | -0.08084151 |
| Rpl31 | 0.00708 | -0.65238895 |
| Rpl31-ps12 | 0.00254 | -0.75889749 |
| Phf11b | 0.58405 | 0.12074939 |
| Tyro3 | 0.00166 | -1.0013448 |
| Tchhl1 | 0.3631 | -0.66099786 |
| Atp13a4 | 0.03041 | -0.93095178 |
| Zfp687 | 0.1333 | -0.320429 |
| BC051019 | 0.59675 | 0.10802914 |
| Insc | 0.03767 | -1.07131853 |
| Nt5c | 0.00174 | -0.91673262 |
| Ptprj | 0.2042 | -0.32614554 |
| Tpmt | 0.21451 | 0.27184836 |
| Tmem104 | 0.00356 | -0.78229548 |
| Tmem104 | 0.49348 | 0.21324268 |
| Cst8 | 0.00144 | -1.20555712 |
| Dock11 | 0.00144 | 2.32717495 |
| Frmd3 | 0.00152 | -1.25878908 |
| Fbxo47 | 0.00717 | -1.0591794 |
| Rpsa | 0.00144 | -1.34433257 |
| Trpm8 | 0.01049 | -2.05881277 |
| Csrp1 | 0.00144 | -1.23585681 |
| Trpm1 | 0.00144 | -1.29085005 |
| Abo | 0.00144 | -1.28116048 |
| Ino80 | 0.39426 | 0.16556913 |
| Svs2 | 0.00144 | -1.29049509 |
| Bmp10 | 0.00591 | -1.01612101 |
| Palmd | 0.01289 | 2.19019292 |
| 1700019O17Rik | 0.6785 | -0.06762497 |
| BC040756 | 0.16656 | 0.40194547 |
| Gm10727 | 0.00166 | 1.75051676 |
| Tyw1 | 0.03951 | -0.41655466 |
| Dtna | 0.01668 | -2.18897937 |
| Gm4047 | 0.00144 | -1.27165604 |
| Foxl2 | 0.00144 | -1.13922277 |
| Gm10638 | 0.86731 | 0.05228021 |
| Gm10638 | 0.00174 | -1.1409142 |
| Gm9733 | 0.15324 | -0.75987879 |
| Gm1968 | 0.00327 | -1.36949191 |
| Cttn | 0.33985 | -0.15071522 |
| Gm10427 | 0.03653 | -0.38767492 |
| Elmsan1 | 0.11402 | -0.40572839 |
| Acp5 | 0.00534 | -0.88188997 |
| Trf | 0.09771 | -0.36966745 |
| Trf | 0.00766 | -0.62963811 |
| Sec14l5 | 0.00147 | -1.48940061 |
| Acsf3 | 0.05004 | -0.61776115 |
| Pld3 | 0.0027 | -0.92859732 |
| Kifap3 | 0.03852 | 0.57148764 |
| Etv5 | 0.00387 | -0.93610693 |
| Depdc1a | 0.0075 | -1.08536055 |
| Gapdh | 0.46399 | -0.34485821 |
| Hk1 | 0.0182 | -0.47945678 |
| Rph3al | 0.03474 | -1.62751804 |
| H2-Q10 | 0.5323 | -0.43823715 |
| Plcd3 | 0.003 | -0.94456643 |
| Ppp1r12b | 0.01972 | -0.78266147 |
| Ppp1r12b | 0.01343 | -0.56844021 |
| Sp110 | 0.06388 | -0.45520185 |
| Klhdc10 | 0.23295 | -0.25849515 |
| Alkbh2 | 0.01038 | -0.56708192 |
| Esyt1 | 0.41836 | 0.2427167 |
| Pja1 | 0.58107 | 0.10371204 |
| Igfbpl1 | 0.35773 | 0.61183159 |
| Jsrp1 | 0.00144 | -1.39353727 |
| Tmem106a | 0.01674 | -0.56423002 |
| Ran | 0.17503 | -0.32348302 |
| Exoc2 | 0.01364 | 0.67574775 |
| 2610305D13Rik | 0.00144 | 3.17163006 |
| Zfp600 | 0.85485 | 0.08905287 |
| 2610305D13Rik | 0.12323 | 0.81595304 |
| Rgs22 | 0.00144 | -1.26428512 |
| H2-M10.4 | 0.06495 | -0.92433994 |
| Zic3 | 0.00144 | -1.31884045 |
| Mrps14 | 0.19851 | -0.35114634 |
| Rangrf | 0.003 | -0.97711698 |
| Med29 | 0.00261 | -0.87032331 |
| Hist2h2bb | 0.00144 | -1.60258533 |
| Hist3h2ba | 0.00144 | -1.38943975 |
| Gm16462 | 0.00953 | -1.85408081 |
| Zfhx2os | 0.08827 | -0.94312708 |
| Pxylp1 | 0.00158 | -1.22727468 |
| Stk36 | 0.00545 | -1.10044792 |
| Ddx52 | 0.01282 | 0.98302345 |
| Cpsf3l | 0.41129 | -0.18060672 |
| Ssrp1 | 0.64314 | -0.08439774 |
| Pydc3 | 0.45809 | -0.53197093 |
| Lrif1 | 0.00144 | -1.34986068 |
| Lrif1 | 0.00144 | 1.73124705 |
| Fhl5 | 0.90068 | -0.03848891 |
| Gm13547 | 0.95275 | 0.03114499 |
| Gm13547 | 0.04218 | -0.54257872 |
| Vav1 | 0.00744 | -0.85005106 |
| Fbxl12os | 0.00232 | 1.41030683 |
| Hsd3b2 | 0.01208 | -1.27668055 |
| Nek10 | 0.12239 | -2.11036252 |
| Mbd2 | 0.01225 | 0.54784091 |
| Dis3 | 0.0228 | 0.60295102 |
| Gm16495 | 0.00158 | -1.2446827 |
| Klhl10 | 0.80002 | 0.07441223 |
| Ifna11 | 0.01398 | -1.01895817 |
| Synb | 0.49669 | -0.17582046 |
| Med24 | 0.74084 | -0.16834652 |
| LOC105242925 | 0.7922 | -0.10768639 |
| Magi1 | 0.0582 | 0.91078962 |
| Slc26a6 | 0.70247 | -0.23512367 |
| Zfp92 | 0.00144 | -1.26428512 |
| Ipcef1 | 0.00144 | -1.26560808 |
| Oc90 | 0.00144 | -1.22925777 |
| Vmn2r70 | 0.00144 | -1.28116048 |
| Bdnf | 0.86316 | 0.02995575 |
| Gm10432 | 0.2474 | -0.89858943 |
| Psen2 | 0.00222 | -0.95417704 |
| AV320801 | 0.00601 | -1.09465341 |
| Slc22a12 | 0.01395 | -1.01228913 |
| Chadl | 0.0039 | -1.09201413 |
| Mcf2l | 0.16984 | 0.33152965 |
| Dusp13 | 0.89135 | -0.05383708 |
| Gm2027 | 0.00144 | -1.37434134 |
| Gm3764 | 0.04698 | -0.91567309 |
| Gm10615 | 0.57214 | -0.19039905 |
| Spin2d | 0.00159 | -1.26431393 |
| Gm2837 | 0.00144 | -1.31495282 |
| Fam63a | 0.01547 | -0.63394252 |
| Mark3 | 0.52444 | -0.10136672 |
| Hipk3 | 0.2277 | 0.22203409 |
| Gm6307 | 0.27577 | -0.26895288 |
| Haus8 | 0.01367 | -0.67098615 |
| Prkcsh | 0.00997 | -0.76719687 |
| Mical3 | 0.00214 | -0.87259285 |
| Smarcd2 | 0.31141 | 0.5068865 |
| Gm1043 | 0.16098 | -0.76142342 |
| Fzd8 | 0.28939 | -0.26044562 |
| Pla2g10 | 0.00144 | -1.30017714 |
| Gm8884 | 0.8927 | 0.0576122 |
| Reg3d | 0.02483 | -1.23190553 |
| Gyk | 0.25365 | 0.23750379 |
| Aebp1 | 0.00144 | -0.94189007 |
| Psmb6 | 0.00313 | -0.88667163 |
| Cep89 | 0.18746 | -0.33176957 |
| Tmbim1 | 0.01027 | -0.62440663 |
| Rbm15b | 0.39543 | -0.22010663 |
| Gm36117 | 0.3036 | -0.22561455 |
| Strip1 | 0.00515 | -0.70663852 |
| Zfp879 | 0.31631 | -0.17986781 |
| D3Ertd751e | 0.00546 | 1.71267944 |
| Kif9 | 0.68487 | 0.1998416 |
| Kif9 | 0.00144 | -1.28116048 |
| Nt5c3 | 0.11598 | 0.29254623 |
| Zbtb20 | 0.00186 | 0.97005506 |
| Gucd1 | 0.97112 | -0.00712969 |
| Olfr199 | 0.00144 | -1.29049509 |
| Ripk4 | 0.03148 | -1.44298857 |
| Vmn2r12 | 0.00144 | -1.30713006 |
| Fbxw14 | 0.00145 | -1.24191372 |
| LOC105247324 | 0.09857 | -0.89108496 |
| D5Ertd605e | 0.04425 | -0.58846997 |
| Nfil3 | 0.02525 | -0.90996502 |
| Rasgef1b | 0.68234 | 0.09573624 |
| 3110099E03Rik | 0.24175 | -0.55691638 |
| Rhot1 | 0.00246 | 0.84881653 |
| Uhrf1 | 0.04308 | -0.83638102 |
| Elf4 | 0.09162 | -1.32660737 |
| Trpm7 | 0.00145 | 2.23765121 |
| Skint6 | 0.00345 | -1.66666667 |
| Cyb5d1 | 0.11202 | -0.98371999 |
| Nifk | 0.45791 | 0.11708407 |
| Pcdh9 | 0.18018 | -0.82498108 |
| Pcdh1 | 0.00481 | -0.78248978 |
| Btnl5-ps | 0.00149 | -1.19348234 |
| Itln1 | 0.00144 | -1.30713006 |
| Arhgef15 | 0.04253 | -0.43493342 |
| Il1f10 | 0.00144 | -1.54666795 |
| Gm2436 | 0.00675 | 0.7124911 |
| Zfp653 | 0.11812 | -0.26486104 |
| Clec4n | 0.14232 | -0.47236589 |
| Vmn2r49 | 0.00144 | -1.26560808 |
| Ciita | 0.01946 | -1.26909909 |
| Tjp1 | 0.00145 | 0.99443121 |
| Vav2 | 0.32458 | 0.35982836 |
| Sh3rf3 | 0.11856 | -0.83073512 |
| Mbd5 | 0.51007 | -0.3077505 |
| BC147527 | 0.04033 | -0.64365887 |
| Socs7 | 0.0137 | -0.49521981 |
| Siglecg | 0.07714 | -0.59253586 |
| Gm5868 | 0.04611 | -0.77397603 |
| Tubb2b | 0.02017 | -0.64922803 |
| Pias4 | 0.01157 | -0.73285972 |
| Exosc2 | 0.01479 | -0.61004219 |
| Rasal2 | 0.82167 | -0.04377463 |
| Cfap36 | 0.00855 | 1.22154182 |
| Clvs1 | 0.63077 | 0.38732743 |
| Gm20619 | 0.00144 | -1.28116048 |
| Fam227b | 0.08469 | -0.87477891 |
| Sema6a | 0.00675 | 0.89043112 |
| Pdcd5 | 0.10492 | 0.29334762 |
| 1700013F07Rik | 0.59565 | -0.14786888 |
| Abcc12 | 0.04551 | -0.91232186 |
| Sds | 0.00145 | -1.2388121 |
| Serpinb7 | 0.93753 | -0.0739154 |
| Ankhd1 | 0.00624 | 1.10087759 |
| Zfp69 | 0.05253 | 0.7101947 |
| Eif4g3 | 0.05733 | -0.62656153 |
| Eif4g3 | 0.03369 | 0.56263627 |
| Terf2 | 0.50774 | -0.14260965 |
| Terf2 | 0.00852 | -0.71743877 |
| Qrfpr | 0.00523 | -1.36541807 |
| Cage1 | 0.23507 | 0.54155254 |
| Tmem40 | 0.00721 | -1.5208417 |
| Arfgap2 | 0.01323 | -0.71851612 |
| Zscan26 | 0.00144 | 2.12275486 |
| Meox1 | 0.01575 | -0.5893165 |
| Sprr3 | 0.00144 | -1.30783085 |
| Elavl1 | 0.06606 | -0.31905435 |
| Myom3 | 0.1253 | 0.30973108 |
| Il12rb1 | 0.29904 | -0.24321602 |
| Nos3 | 0.00764 | -0.62742074 |
| Ddo | 0.3523 | -0.23924741 |
| Gm35339 | 0.39187 | 0.37295217 |
| Rnf212b | 0.98802 | 0.00977352 |
| Sltm | 0.00144 | 1.48424832 |
| Tsga8 | 0.11249 | -0.30790294 |
| Fut10 | 0.02029 | -0.83437943 |
| Gm12253 | 0.18657 | 0.28483557 |
| Plekha8 | 0.31362 | -0.19942473 |
| Psmc1 | 0.09262 | -0.2807341 |
| Sphkap | 0.00145 | -1.22376446 |
| Olfr403 | 0.00158 | -1.2446827 |
| Olfr203 | 0.00144 | -1.29878664 |
| A230051N06Rik | 0.09604 | -0.92355315 |
| Fabp6 | 0.04636 | 0.52753441 |
| Fabp3 | 0.00234 | -0.81934557 |
| Esrp1 | 0.09192 | -0.65363339 |
| Gm8179 | 0.0029 | -1.20702967 |
| Olfr731 | 0.00144 | -1.26428512 |
| Ascl2 | 0.25583 | -0.36497205 |
| Olfr131 | 0.00144 | -1.20555712 |
| Pax3 | 0.83117 | -0.08209121 |
| Vipr2 | 0.1175 | -0.72654504 |
| LOC102642592 | 0.00144 | -1.23575345 |
| 9430038I01Rik | 0.04231 | -0.41015988 |
| Mmab | 0.00144 | -1.07693656 |
| 4930505N22Rik | 0.05764 | -1.56914678 |
| Med6 | 0.01305 | 0.62788889 |
| Ppm1m | 0.01713 | -0.51952562 |
| Ltbp4 | 0.00398 | -0.72362728 |
| Ltbp4 | 0.00158 | -0.95056676 |
| Ece2 | 0.64735 | -0.16882948 |
| Gm13298 | 0.0506 | -0.92910588 |
| Rps23 | 0.02979 | -0.42797023 |
| Stra6l | 0.00144 | -1.29495476 |
| Sppl2a | 0.11862 | 0.26709855 |
| Med4 | 0.90203 | -0.04022106 |
| Meg3 | 0.00205 | 1.4936844 |
| 1810041L15Rik | 0.02815 | -0.61375653 |
| Lgi4 | 0.00144 | -1.23609378 |
| Six2 | 0.38266 | -0.34054743 |
| Cept1 | 0.0733 | 0.46959707 |
| Speer5-ps1 | 0.00318 | 1.56836418 |
| Treh | 0.00144 | -1.28116048 |
| Prdx5 | 0.00436 | -0.90835432 |
| Fdft1 | 0.67717 | 0.20043711 |
| Cabp5 | 0.87743 | -0.09672221 |
| Pomgnt1 | 0.0242 | -0.80185313 |
| Gm3830 | 0.00145 | -1.26219879 |
| Srxn1 | 0.08469 | -0.32284761 |
| Crisp3 | 0.00369 | -1.47467543 |
| Cdcp1 | 0.73585 | -0.16917281 |
| Olfr1215 | 0.87808 | -0.24297368 |
| Clcn1 | 0.02254 | 0.95395544 |
| Pdc | 0.01339 | -1.12694059 |
| Hmgcs1 | 0.07717 | 0.50531641 |
| Brap | 0.00781 | -0.58105151 |
| Pabpn1l | 0.01875 | -1.00497514 |
| Olfr23 | 0.00144 | -1.72513992 |
| Slc6a20a | 0.02298 | -0.72061164 |
| Gm6588 | 0.06639 | -2.20858179 |
| Pip5k1c | 0.02883 | -0.65753107 |
| Pip5k1c | 0.01356 | -1.11035991 |
| Gm32062 | 0.00764 | -1.11787821 |
| Trim2 | 0.24434 | 0.55089157 |
| Rnase13 | 0.10513 | -0.88511728 |
| Gm10450 | 0.00144 | -1.30017714 |
| Inpp5e | 0.14514 | 0.5640826 |
| Ctif | 0.00469 | -0.70811139 |
| Cpt1c | 0.05564 | -0.41490969 |
| Iars2 | 0.00216 | -1.10833901 |
| Gm7903 | 0.00144 | -1.28116048 |
| Dnajc10 | 0.00145 | -0.93121538 |
| Klra9 | 0.00153 | 1.67739134 |
| Fhit | 0.49529 | -0.25823923 |
| Foxp3 | 0.00144 | -1.58553387 |
| Gm4814 | 0.32287 | 0.41726855 |
| Zcchc11 | 0.06117 | 0.3882203 |
| Tox | 0.94216 | -0.03849644 |
| Kif5b | 0.07363 | -2.37373657 |
| Ajap1 | 0.00299 | -1.143921 |
| LOC101055907 | 0.02849 | 1.08925313 |
| Rps24 | 0.04022 | -0.3761879 |
| Vmn2r118 | 0.00144 | -1.28898079 |
| 9430024F10Rik | 0.01092 | -0.81107003 |
| Slc4a5 | 0.24878 | -0.7610065 |
| Cblc | 0.00553 | -0.79617081 |
| Pde3b | 0.04024 | 1.08126687 |
| Slc5a4a | 0.36338 | -0.4211851 |
| Ubap1l | 0.00151 | -1.17792995 |
| Sfxn5 | 0.52598 | -0.19892826 |
| Gm6934 | 0.11446 | -0.28894308 |
| Slc25a37 | 0.02534 | -0.78184956 |
| Gm8378 | 0.11968 | -1.32778823 |
| C030044M21Rik | 0.01709 | -0.99496437 |
| 4933423P22Rik | 0.00144 | -1.28116048 |
| 4930522L14Rik | 0.31886 | -0.20633567 |
| B230307C23Rik | 0.66884 | 0.13575338 |
| Zfp932 | 0.63097 | 0.11352543 |
| Habp2 | 0.02299 | -1.50540501 |
| Gm10767 | 0.00297 | 0.9166896 |
| 1700125H20Rik | 0.03188 | -0.64270288 |
| Pdlim1 | 0.48251 | -0.17048425 |
| Rgs9 | 0.18101 | 0.36660328 |
| Tm9sf2 | 0.20688 | 0.30055966 |
| Gm12666 | 0.09613 | 0.64356422 |
| Ifi202b | 0.2508 | -0.89670575 |
| Wnt9a | 0.11482 | -0.554699 |
| Dnase1l1 | 0.79258 | 0.05021633 |
| Zfp715 | 0.03397 | 0.83423705 |
| Dbp | 0.01339 | -0.74605952 |
| Tbp | 0.7962 | -0.04027312 |
| Uggt2 | 0.0306 | 0.91877239 |
| Cc2d2b | 0.0407 | -0.84884611 |
| 9030617O03Rik | 0.71192 | -0.10505608 |
| Fbxo39 | 0.9366 | 0.02832844 |
| Bend6 | 0.27882 | 0.48793996 |
| Aqp4 | 0.00182 | 1.71637905 |
| Cdh7 | 0.00144 | -1.4333006 |
| Cdh7 | 0.00144 | -1.31884045 |
| Gm17026 | 0.00144 | -1.31495282 |
| Gm17175 | 0.00144 | -1.27165604 |
| Plekhh3 | 0.00144 | -1.13776881 |
| Gm4278 | 0.00144 | -1.51017157 |
| Gpr85 | 0.0378 | -0.93613993 |
| Fuk | 0.00262 | -0.77439541 |
| Npas3 | 0.00149 | -1.22582153 |
| Npas1 | 0.00144 | -1.80931378 |
| Gstm6 | 0.45456 | -0.21424849 |
| Gstp1 | 0.2085 | -0.53505223 |
| 2210008F06Rik | 0.09538 | -0.70419224 |
| Gm3032 | 0.20859 | -0.59123812 |
| Vmn2r121 | 0.35575 | -1.10477164 |
| 1700029H14Rik | 0.00144 | -1.28116048 |
| Hnrnpa3 | 0.00144 | 1.39000451 |
| Rufy3 | 0.14867 | 0.76666295 |
| Rufy4 | 0.00778 | 1.07929084 |
| Lrrc32 | 0.00413 | -1.14836216 |
| Pithd1 | 0.26633 | -0.3183665 |
| Ly6e | 0.00152 | -1.01247065 |
| Smim24 | 0.01759 | -0.61512873 |
| 9230009I02Rik | 0.00144 | -1.66099786 |
| Brd1 | 0.11645 | -0.71098908 |
| Ppp3r2 | 0.00144 | -1.20579466 |
| Uxs1 | 0.55867 | 0.32910491 |
| Zfp597 | 0.11474 | -0.48910543 |
| Lhx3 | 0.32053 | -0.35800019 |
| Sun1 | 0.01011 | -0.66600159 |
| Vps52 | 0.00701 | -0.78339341 |
| Ggnbp2 | 0.00144 | 1.66302347 |
| Gtdc1 | 0.00874 | 0.65514108 |
| Efna1 | 0.00144 | -1.27152684 |
| Efna3 | 0.04174 | -0.43994322 |
| Tmem220 | 0.00147 | 1.2135391 |
| Ube2l6 | 0.0112 | -0.69182153 |
| Dhps | 0.01164 | -0.74838516 |
| Gm4737 | 0.18618 | -0.35810819 |
| Gm33969 | 0.00544 | -1.22542589 |
| Mc2r | 0.00145 | -1.22376446 |
| Stat5b | 0.04057 | -0.5954922 |
| Bex1 | 0.53608 | 0.26439718 |
| S100pbp | 0.71622 | -0.06863149 |
| Olfr325 | 0.00257 | -1.1333102 |
| 9830107B12Rik | 0.06461 | -2.64876408 |
| 2610301B20Rik | 0.3392 | 0.32479357 |
| Gm16524 | 0.10835 | 0.48537282 |
| Trim59 | 0.00935 | 1.44883791 |
| Rimbp2 | 0.00144 | -1.28116048 |
| Esr1 | 0.00145 | -1.2388121 |
| 4933416I08Rik | 0.91037 | 0.0728911 |
| Actr10 | 0.01178 | -0.55540482 |
| Rpl3 | 0.13143 | -0.37667127 |
| Fnbp1 | 0.01082 | -1.01248548 |
| Nedd4l | 0.02481 | 0.66971267 |
| Scmh1 | 0.90862 | -0.04516985 |
| Ankle1 | 0.05601 | 1.1562847 |
| Gm1527 | 0.00144 | -1.31884045 |
| Gm1587 | 0.00144 | -1.27454685 |
| Eda | 0.00144 | -1.39887133 |
| Eda | 0.14387 | -0.38245026 |
| Dusp13 | 0.57216 | 0.15369204 |
| Dusp23 | 0.01438 | -0.68537326 |
| 4922502B01Rik | 0.00144 | -1.24613586 |
| Gm5932 | 0.00144 | -1.29051721 |
| 2010002M12Rik | 0.00583 | 1.34360955 |
| A430033K04Rik | 0.04614 | -0.94653086 |
| Ppp2r4 | 0.00144 | -1.05202817 |
| Gm5089 | 0.00144 | -1.55888744 |
| Srsf2 | 0.35594 | -0.42499215 |
| Gpi1 | 0.0028 | -0.87262634 |
| Sirt1 | 0.00334 | 0.91357512 |
| Frmd4a | 0.00144 | 1.18327992 |
| Frmd4a | 0.01282 | -0.51382238 |
| Atp1a2 | 0.75131 | 0.0609228 |
| Tm7sf3 | 0.04639 | -0.62851329 |
| Iltifb | 0.00144 | -1.28116048 |
| Mcf2 | 0.00144 | -1.29049509 |
| Trem1 | 0.91053 | 0.03729983 |
| Gm3383 | 0.00144 | 2.71078854 |
| Dnah6 | 0.27075 | 0.32264033 |
| Dnah6 | 0.00144 | -1.26934956 |
| Fan1 | 0.33587 | -1.55460447 |
| Fan1 | 0.09092 | -0.60206615 |
| Fan1 | 0.66863 | 0.20550091 |
| Supt6 | 0.00492 | -0.6709062 |
| Ifna5 | 0.87992 | -0.06656846 |
| Ifna7 | 0.19557 | -0.44105427 |
| Ifna1 | 0.0039 | 1.18191041 |
| Ifna2 | 0.799 | -0.16118209 |
| Tcf7l2 | 0.00197 | -1.98194723 |
| Rbm39 | 0.0262 | 1.34685305 |
| Tfpt | 0.13097 | -0.38962264 |
| Tfpt | 0.0303 | -0.74317541 |
| Gm7697 | 0.00145 | -1.26219879 |
| Ifnab | 0.14098 | 0.55717968 |
| Barx1 | 0.00145 | -1.26219879 |
| Adssl1 | 0.0723 | -0.66559468 |
| C130026L21Rik | 0.00544 | -1.10518964 |
| Olfr1151 | 0.00144 | -1.31884045 |
| Syndig1 | 0.00144 | -1.26560808 |
| Ptprs | 0.15451 | -0.64052555 |
| Gab3 | 0.00144 | 3.87838108 |
| Cfap46 | 0.00145 | -1.2388121 |
| LOC102641693 | 0.11152 | -1.99741247 |
| Gm38396 | 0.00158 | 2.104423 |
| Gm13219 | 0.00144 | -1.29049509 |
| Gm13219 | 0.00144 | -1.3348153 |
| Peg10 | 0.00144 | -1.24613586 |
| Ndufs5 | 0.00167 | -0.99114178 |
| C730027H18Rik | 0.00144 | -1.27165604 |
| Cdh2 | 0.37587 | 0.36522356 |
| Fry | 0.0107 | 0.7914566 |
| Etfa | 0.57523 | -0.11752136 |
| Gm9510 | 0.42447 | 0.21816478 |
| Glt6d1 | 0.11039 | -0.74962154 |
| Sry | 0.92123 | -0.13609912 |
| Mpdu1 | 0.24798 | -0.31077477 |
| Ppp1r3f | 0.04455 | -0.557841 |
| Dhx35 | 0.07025 | -0.39298174 |
| Muc4 | 0.00144 | -1.31884045 |
| Dus2 | 0.0153 | -0.6093944 |
| Nt5c1a | 0.11942 | -0.4972885 |
| Adrb2 | 0.93224 | -0.0270946 |
| Dtd1 | 0.322 | -0.25500077 |
| C130040N14Rik | 0.11803 | -1.03701044 |
| Wnk2 | 0.94588 | 0.03206852 |
| Osbpl1a | 0.45093 | -0.13697455 |
| Gm5544 | 0.00144 | -1.29085005 |
| Btbd8 | 0.10967 | -1.93953559 |
| 2610318N02Rik | 0.41631 | 0.4652538 |
| Cd70 | 0.00144 | -1.31518407 |
| Smad1 | 0.15269 | -0.42221522 |
| 4930556A20Rik | 0.0019 | -1.17487561 |
| Btn1a1 | 0.03658 | -0.46905484 |
| Dopey2 | 0.06359 | -0.46070532 |
| Pde4a | 0.00144 | -1.25272588 |
| Pde4a | 0.00144 | -1.52571102 |
| Atr | 0.00144 | 2.89764993 |
| Snap47 | 0.83094 | 0.12836904 |
| Zfp287 | 0.81521 | 0.09678149 |
| Pex11g | 0.63375 | 0.07370846 |
| Pex11g | 0.22092 | 0.62961044 |
| Bmf | 0.00747 | -0.65591637 |
| Lcorl | 0.92037 | 0.03824193 |
| A930029G22Rik | 0.06464 | -1.56472691 |
| Fgd3 | 0.14547 | -0.34660945 |
| Bpifb5 | 0.00144 | -1.26560808 |
| Tcf20 | 0.62687 | -0.097398 |
| Gm12568 | 0.40034 | -0.54074716 |
| Idi1 | 0.00352 | 1.238806 |
| Gm21944 | 0.05145 | -2.50869413 |
| Ncam1 | 0.95269 | -0.01588987 |
| Prl2b1 | 0.00186 | -1.38908362 |
| Gata4 | 0.78218 | -0.11963386 |
| Rnft2 | 0.44519 | 0.13194169 |
| Dsg4 | 0.00145 | -1.2388121 |
| Dsg4 | 0.00144 | -1.28898079 |
| Olfr1250 | 0.01729 | -1.04563207 |
| Cntnap3 | 0.00144 | -1.40179533 |
| Nqo2 | 0.19303 | -0.26557614 |
| Slc39a11 | 0.00935 | -0.61299705 |
| Slc39a11 | 0.17829 | -1.05468637 |
| Slc22a17 | 0.00924 | -0.55681101 |
| Klra16 | 0.00174 | 1.22727468 |
| G630018N14Rik | 0.00149 | -1.22582153 |
| C87499 | 0.00144 | -1.22465584 |
| Dusp12 | 0.36629 | 0.23184897 |
| Slc31a1 | 0.64174 | 0.08563291 |
| Cfap46 | 0.09522 | -0.68880866 |
| Ptprm | 0.09826 | -0.94992628 |
| Sgsh | 0.01618 | -0.66363937 |
| Becn2 | 0.00144 | -1.26560808 |
| Stac | 0.17906 | -1.88655721 |
| Gm14957 | 0.02125 | -0.72597128 |
| Gm10165 | 0.00145 | -1.2388121 |
| Adam22 | 0.00292 | 1.2168935 |
| Adam22 | 0.00144 | -1.30713006 |
| Adam22 | 0.35333 | -0.42397999 |
| Hacd2 | 0.00434 | 1.03423486 |
| 1810013A23Rik | 0.99748 | -0.00188219 |
| Atg13 | 0.00384 | -0.85572656 |
| Gm6590 | 0.00144 | -1.30713006 |
| Vmn2r110 | 0.00149 | -1.19696847 |
| Gm3134 | 0.00144 | -1.28898079 |
| Lrrc36 | 0.58177 | -0.13660596 |
| Htr6 | 0.88551 | 0.07444729 |
| Ccser2 | 0.03146 | 0.47329918 |
| Coro6 | 0.00241 | 1.16683291 |
| Ankrd7 | 0.04696 | -1.29878664 |
| Ppm1d | 0.04458 | 0.42815103 |
| Nhlrc4 | 0.04909 | -0.84536667 |
| Pik3cd | 0.02999 | -0.55017102 |
| Pik3cd | 0.03433 | -0.70552044 |
| Rhbdf2 | 0.00145 | -0.99760154 |
| Dnajc19 | 0.24096 | -0.31494556 |
| Mgat1 | 0.00331 | -0.78337413 |
| Fhl3 | 0.02098 | -0.69095702 |
| Gnrhr | 0.01491 | -1.01447254 |
| Prph2 | 0.00145 | -1.26219879 |
| Gm10933 | 0.0802 | 0.75685073 |
| Kdm4b | 0.01862 | -0.5494522 |
| Incenp | 0.05916 | -0.38395012 |
| Cyp3a59 | 0.00144 | -1.28116048 |
| Olfr957 | 0.00144 | -1.29085005 |
| Pawr | 0.04632 | 0.60331348 |
| Tas2r124 | 0.17147 | -1.80042639 |
| Hk1 | 0.29444 | -0.18838105 |
| Rpl10l | 0.00144 | -1.68501379 |
| Myl6 | 0.00353 | -0.79370807 |
| Kir3dl2 | 0.00144 | -1.28116048 |
| Clvs1 | 0.00144 | -1.47614798 |
| Nsa2 | 0.00492 | -0.96475414 |
| Gabra2 | 0.67943 | 0.75301934 |
| Ccl25 | 0.91822 | -0.03502462 |
| Tnfrsf10b | 0.71848 | -0.09437426 |
| Cryba4 | 0.00202 | -1.36969723 |
| Ccdc101 | 0.01872 | -0.56399809 |
| Rpl41 | 0.00144 | -1.06575339 |
| Arxes2 | 0.15024 | 0.84897718 |
| Baiap2 | 0.10998 | -0.41730611 |
| Zfp260 | 0.01988 | 0.70890978 |
| Gm11735 | 0.18109 | -0.86231677 |
| Il1rl1 | 0.18463 | 0.46667548 |
| Il1rl1 | 0.00144 | -1.31884045 |
| Ndufa5 | 0.00589 | -0.66070333 |
| Neto1 | 0.04596 | -1.22929445 |
| Rps28 | 0.00144 | -1.12611748 |
| Skint10 | 0.096 | 0.44050292 |
| Scgb3a2 | 0.79636 | -0.26892475 |
| Taf3 | 0.45487 | -0.16749369 |
| Fsd1l | 0.09514 | 0.28669502 |
| Adck5 | 0.00144 | -0.99660524 |
| Pxk | 0.38366 | 0.13876967 |
| Il1f8 | 0.05044 | -2.41605226 |
| Bri3 | 0.00538 | -1.015066 |
| Fam163a | 0.00144 | -1.33632959 |
| Gpr146 | 0.54822 | -0.13520351 |
| Gpr156 | 0.12653 | -0.77481165 |
| Gm32934 | 0.00144 | -1.44178182 |
| Casc5 | 0.01126 | -1.15703151 |
| Cdc34 | 0.00144 | -1.47570801 |
| Gm8630 | 0.03655 | -0.62890365 |
| Gas2l1 | 0.00377 | -0.75109306 |
| Gm9902 | 0.04827 | -0.99433119 |
| Engase | 0.00144 | -1.12561078 |
| Krtap20-2 | 0.07378 | 0.73315346 |
| Gm35257 | 0.09648 | -0.44182353 |
| Rph3al | 0.48322 | -0.14772244 |
| Ssh1 | 0.40685 | -0.17356388 |
| Mrps18c | 0.58796 | 0.08971446 |
| Hist1h3f | 0.0083 | -0.68385169 |
| Triml2 | 0.00144 | -1.47490668 |
| Rgs3 | 0.01546 | -0.7441927 |
| Rgs3 | 0.56772 | -0.32010249 |
| Rgs3 | 0.00151 | -0.95672244 |
| Kcnj10 | 0.02688 | 0.59425098 |
| Shc2 | 0.50539 | 0.15691232 |
| Phc2 | 0.00205 | -1.00511906 |
| Olfr729 | 0.0017 | -1.24950944 |
| Olfr329-ps | 0.00144 | -1.25118919 |
| Serpinb8 | 0.08582 | -0.35640239 |
| Tdrp | 0.04739 | -0.54247632 |
| C530030P08Rik | 0.30288 | 0.64375954 |
| Vmn1r169 | 0.00144 | -1.29746368 |
| Tigit | 0.00727 | -1.07804057 |
| Med10 | 0.92886 | 0.01577642 |
| Gm21292 | 0.00144 | -1.30713006 |
| Trim32 | 0.11278 | 0.41412721 |
| Vprbp | 0.00209 | 0.8563464 |
| Vprbp | 0.00177 | 1.24911697 |
| Gm10558 | 0.00158 | -1.15468698 |
| Rgs4 | 0.3931 | 0.19591578 |
| Mylk4 | 0.04406 | -0.79546835 |
| 4930445E18Rik | 0.79487 | 0.12375194 |
| Gm11564 | 0.35806 | 0.22082015 |
| Asnsd1 | 0.03723 | 0.45245255 |
| Pilrb1 | 0.15597 | 0.51832259 |
| Zyg11a | 0.00144 | -1.29878664 |
| Fam189a1 | 0.00144 | -1.33632959 |
| Ush2a | 0.00144 | -1.44071912 |
| Cdkn1b | 0.07826 | 0.38846805 |
| Gal3st2 | 0.23102 | -0.35018019 |
| Erc2 | 0.54319 | -0.10193703 |
| Erc2 | 0.00158 | 1.85760055 |
| Stab2 | 0.0242 | -1.03617482 |
| Stab2 | 0.03566 | -0.95635284 |
| Fabp12 | 0.00144 | -1.20555712 |
| Tfam | 0.00425 | 1.46412926 |
| Ceacam-ps1 | 0.00144 | -1.31495282 |
| Foxb1 | 0.00144 | -1.30713006 |
| Fbxw20 | 0.02717 | -0.79981249 |
| Syne1 | 0.00144 | 1.62917509 |
| Ppp1r1b | 0.37081 | -0.53261802 |
| St6galnac4 | 0.00966 | -0.82759733 |
| Igsf8 | 0.00158 | -0.87089379 |
| Ccdc172 | 0.01483 | -1.43528444 |
| Nxpe5 | 0.00425 | -1.12545863 |
| Dlec1 | 0.03401 | -0.4899902 |
| Rpap1 | 0.01373 | -0.80667883 |
| Mroh2a | 0.89983 | -0.16690738 |
| Usp30 | 0.01233 | -0.70673629 |
| Mc5r | 0.07216 | -0.54845911 |
| Anks1b | 0.00144 | -1.32599123 |
| Ruvbl2 | 0.32043 | -0.19618494 |
| Acly | 0.15297 | -0.27386232 |
| Sms | 0.18527 | -0.27386186 |
| Fam71e2 | 0.01552 | -0.97598014 |
| Ccdc169 | 0.61443 | -0.19378934 |
| Zfp932 | 0.70401 | 0.19276626 |
| Hnrnpul1 | 0.01989 | -0.49314268 |
| Heatr3 | 0.10627 | 0.44473022 |
| Spred2 | 0.00852 | -0.79722267 |
| Plcb4 | 0.04341 | 0.51945794 |
| Fanci | 0.09709 | -1.60481478 |
| Skint1 | 0.97797 | 0.03875102 |
| Adam8 | 0.61904 | 0.15847425 |
| Adam9 | 0.00144 | 1.69819349 |
| Adam5 | 0.02158 | -1.08169189 |
| Adam7 | 0.00144 | -1.27165604 |
| Adam3 | 0.19653 | -1.84009993 |
| Immt | 0.05734 | 0.97476064 |
| C330007P06Rik | 0.00637 | -1.12344346 |
| Slc5a7 | 0.0018 | -1.21153109 |
| Muc4 | 0.03563 | 1.09901933 |
| Dnah8 | 0.39476 | -0.33489271 |
| Gm13238 | 0.00293 | 0.98218014 |
| Psme2b | 0.14655 | -0.35302127 |
| Lsm6 | 0.00213 | -0.82163081 |
| Gcnt2 | 0.49503 | -1.12831642 |
| Zfp780b | 0.35987 | -0.37823055 |
| Ano4 | 0.0161 | 1.15585407 |
| Arhgap6 | 0.20595 | -1.5350638 |
| Arhgap6 | 0.39414 | -0.49338746 |
| Pnpla3 | 0.78955 | 0.20109084 |
| Tnfrsf18 | 0.00647 | -1.89368963 |
| Rfesd | 0.00144 | 2.01224718 |
| Brpf3 | 0.33844 | 0.17339512 |
| Ttc12 | 0.00453 | 1.31999767 |
| Gm29797 | 0.00144 | -1.5864962 |
| Oprm1 | 0.21263 | 0.42659656 |
| C230004F18Rik | 0.00144 | -1.27165604 |
| Loxhd1 | 0.00144 | -1.41224981 |
| Nkain1 | 0.00144 | -1.1822713 |
| Ccnl2 | 0.76972 | -0.06088977 |
| Mup21 | 0.13842 | 0.49949791 |
| Tacr2 | 0.18676 | 0.39335229 |
| Gm13090 | 0.03547 | 1.2352255 |
| Pqlc3 | 0.16042 | 0.50128501 |
| Hrasls5 | 0.68675 | -0.36546151 |
| Rps27a | 0.25885 | -0.6075725 |
| Hmgb1 | 0.012 | -1.53982404 |
| Tlk1 | 0.07278 | 0.35077999 |
| Proca1 | 0.19074 | 0.35123845 |
| Gins3 | 0.49005 | -0.11892188 |
| Csf1r | 0.02247 | -0.70193565 |
| Csf1r | 0.16485 | -0.26891531 |
| Cpe | 0.57697 | -0.13579043 |
| Cep55 | 0.00145 | -1.2388121 |
| Klk1b26 | 0.55539 | 0.23884491 |
| Hoxb4 | 0.59546 | -0.37294849 |
| Hoxb8 | 0.00144 | -1.29878664 |
| Dnah17 | 0.18763 | -0.29741311 |
| Dnah17 | 0.01456 | -0.92301519 |
| Hhipl1 | 0.00144 | -1.31495282 |
| Lrp3 | 0.30389 | -0.21713757 |
| Psg18 | 0.00182 | -1.65709301 |
| Fars2 | 0.00401 | -0.93052146 |
| Gstt1 | 0.00367 | -0.87501116 |
| Abl1 | 0.21213 | -0.28074668 |
| Mmp12 | 0.00233 | -1.09985343 |
| Rbl1 | 0.02719 | -1.20410433 |
| Hspb11 | 0.00144 | -1.32766452 |
| Gm10765 | 0.29838 | -0.16995816 |
| Myo6 | 0.00144 | 1.30285341 |
| Rpe | 0.00144 | 2.22172105 |
| Eral1 | 0.00992 | -0.82206981 |
| Eral1 | 0.83887 | 0.08578721 |
| Obox3 | 0.00145 | -1.22518835 |
| Zfp658 | 0.69489 | 0.11481378 |
| Atxn7l1 | 0.25912 | -0.30276851 |
| Atxn7l1 | 0.00958 | 0.60540365 |
| Dtnbp1 | 0.04308 | -0.69273703 |
| Sec24c | 0.15082 | -0.2997122 |
| Ppap2a | 0.51891 | -0.10264915 |
| Vmn2r102 | 0.00144 | -1.30713006 |
| Ank2 | 0.00354 | 1.47717406 |
| Tbkbp1 | 0.02861 | -0.73199439 |
| Tbkbp1 | 0.00144 | -1.14788001 |
| Gm15412 | 0.00144 | -1.27165604 |
| Gaa | 0.81605 | 0.14520208 |
| Gaa | 0.01028 | -0.93971626 |
| Gaa | 0.0073 | -0.95421538 |
| Napepld | 0.03072 | 0.53257628 |
| Raver2 | 0.85242 | 0.04296835 |
| Rpl34 | 0.00483 | -0.79668882 |
| Tmem33 | 0.00768 | 0.86061695 |
| Suv420h2 | 0.00631 | -0.62828198 |
| Gm4221 | 0.60514 | -0.27969174 |
| Spin1 | 0.30025 | 0.19560968 |
| Gm9973 | 0.00144 | -1.46410581 |
| Fam149a | 0.00144 | 1.96511315 |
| Olfr1288 | 0.00144 | -1.30713006 |
| Ctnnbl1 | 0.00449 | -0.7392551 |
| Zbtb16 | 0.20756 | 0.42217651 |
| Slco6b1 | 0.00144 | -1.29085005 |
| Vmn1r62 | 0.29674 | -0.6728156 |
| Tas2r136 | 0.00144 | -1.26934956 |
| Gm10229 | 0.00495 | -1.43514666 |
| Med1 | 0.01408 | 0.61907429 |
| Prkacb | 0.98028 | 0.00599463 |
| Slc16a5 | 0.04144 | -1.30198583 |
| Slc16a5 | 0.46201 | -0.47232859 |
| Cyp4a31 | 0.3185 | -2.13140447 |
| Rpl5 | 0.15999 | 0.27788692 |
| Vmn1r209 | 0.00158 | -1.2446827 |
| Znf660 | 0.45181 | -0.43620515 |
| Ccdc77 | 0.02702 | 0.52451577 |
| Impg1 | 0.06894 | -0.47400268 |
| Ptpre | 0.54692 | 0.24358663 |
| Mfap1a | 0.14011 | 0.44148169 |
| Cep112 | 0.02557 | 0.68132318 |
| Fam107b | 0.00222 | 1.02272958 |
| Mfsd10 | 0.00245 | -0.79293068 |
| Limch1 | 0.44952 | -0.12035375 |
| Kcnq5 | 0.00145 | -1.24191372 |
| Zfp386 | 0.00144 | 2.1499259 |
| Apex2 | 0.82312 | -0.08774083 |
| 9030624J02Rik | 0.03341 | -0.49548524 |
| Prg4 | 0.05148 | -0.81307185 |
| Vmn2r77 | 0.00144 | -1.29085005 |
| Kiss1r | 0.25084 | -0.55357252 |
| Ikzf1 | 0.82484 | 0.1315182 |
| Rgsl1 | 0.00144 | -1.24811894 |
| Txn2 | 0.09911 | -0.48780017 |
| Txn2 | 0.98235 | -0.0080938 |
| Cd300c | 0.00144 | -1.40512625 |
| Ccr7 | 0.0109 | -0.87945615 |
| Aloxe3 | 0.40456 | 0.35543825 |
| Trip10 | 0.03798 | -0.43658344 |
| Gm10687 | 0.09872 | -0.37950005 |
| Grhl3 | 0.02724 | -1.63792566 |
| Fam24a | 0.00144 | -1.23532597 |
| Gm20877 | 0.02134 | -0.92142873 |
| Ect2l | 0.01645 | -0.86531955 |
| Casz1 | 0.27983 | -0.17375886 |
| Pcdha9 | 0.00398 | -1.69109175 |
| A630075F10Rik | 0.00144 | -1.30017714 |
| Thada | 0.01446 | 0.60922615 |
| Miox | 0.24626 | -0.23606534 |
| Lsm2 | 0.01672 | -0.84478901 |
| Lsm2 | 0.00151 | -0.97631465 |
| LOC102642701 | 0.01924 | -1.78985677 |
| Oog2 | 0.11575 | -0.46782517 |
| Gm13084 | 0.00149 | -1.25408 |
| Gm13084 | 0.04243 | -2.13306794 |
| Nlrp1a | 0.24669 | 0.59559554 |
| Rnf214 | 0.32583 | 0.31806785 |
| Prss28 | 0.255 | -0.33931084 |
| Hsd3b2 | 0.10842 | -2.94632105 |
| Pes1 | 0.7881 | 0.09142185 |
| Dnajb2 | 0.98239 | 0.01227938 |
| Gm4759 | 0.14686 | -2.81692271 |
| Gm6961 | 0.00144 | -1.29864962 |
| 4930449I24Rik | 0.38725 | -0.58145228 |
| Psmd2 | 0.03057 | -0.42073065 |
| Gm17175 | 0.00144 | -1.20555712 |
| Tspan9 | 0.30684 | -0.39768014 |
| Nsa2 | 0.01119 | -0.57483773 |
| Slc12a8 | 0.30253 | -0.22160379 |
| Zfp866 | 0.06554 | 0.54037662 |
| Amph | 0.01552 | -0.97598014 |
| Elovl1 | 0.01014 | -0.57932101 |
| Csnk2a1 | 0.15157 | -0.24132708 |
| Csnk2a1 | 0.99656 | 0.0008479 |
| Cyp4f17 | 0.18106 | 0.32992396 |
| 1700021K19Rik | 0.0229 | -0.46713097 |
| Ssxb10 | 0.03044 | -1.50583356 |
| Trpc1 | 0.0034 | -1.10155641 |
| Tenm3 | 0.1315 | 0.49233 |
| Tenm3 | 0.15157 | -2.2377955 |
| LOC105245882 | 0.01099 | 1.60078506 |
| Tcerg1 | 0.00144 | 1.76999791 |
| Cdk11b | 0.05201 | -0.47728745 |
| Ndufv3 | 0.09415 | -0.38010177 |
| Oxct2b | 0.00384 | 1.94265284 |
| Pira6 | 0.46573 | -0.17629563 |
| Mgat5b | 0.11488 | -0.36420785 |
| Pdss2 | 0.64808 | 0.08016944 |
| Rxfp2 | 0.20525 | -0.84225836 |
| Gpi1 | 0.00236 | -0.89174358 |
| D030018L15Rik | 0.00144 | -1.30737044 |
| D030018L15Rik | 0.11648 | -2.69401742 |
| Gtf2h5 | 0.00641 | -0.64595525 |
| Ghrl | 0.13515 | 0.72874227 |
| Lbh | 0.01056 | -0.81042018 |
| Nxn | 0.00144 | -1.25801729 |
| Tmem209 | 0.04705 | 0.43120929 |
| Dhcr7 | 0.08605 | -0.61564465 |
| Slc38a6 | 0.01886 | 1.09248379 |
| Zfp579 | 0.45577 | 0.1159595 |
| Scgb1b7 | 0.39399 | -1.5415914 |
| Nfe2l1 | 0.00144 | -1.16516333 |
| Cdkn2d | 0.01695 | -0.69289439 |
| Uncx | 0.00343 | -0.94415191 |
| Gm826 | 0.3414 | -0.32456793 |
| Plxdc2 | 0.11732 | -0.44393112 |
| Arvcf | 0.04659 | -0.49494154 |
| Arvcf | 0.01223 | -0.76792381 |
| Sly | 0.00144 | -1.26560808 |
| Gc | 0.00144 | -1.30713006 |
| Pura | 0.16356 | 0.32708061 |
| Cntnap1 | 0.80873 | 0.06479603 |
| Spata22 | 0.59801 | -0.43713248 |
| Rfwd2 | 0.00172 | 1.25469163 |
| Mta3 | 0.88804 | -0.03957327 |
| Klf13 | 0.04293 | -0.40424125 |
| Klf10 | 0.01743 | 0.70105939 |
| Olfr412 | 0.02385 | -1.82947692 |
| Olfr312 | 0.00144 | -1.26560808 |
| Zmym4 | 0.40712 | -0.17286091 |
| Ctsw | 0.00757 | -1.14412538 |
| Olfr700 | 0.59887 | -0.49666652 |
| Olfr1231 | 0.00144 | -1.26911831 |
| Fkbp1a | 0.00144 | -1.40974481 |
| Fkbp1a | 0.00144 | -1.20237225 |
| Ccdc114 | 0.18132 | 0.39645394 |
| Mrpl2 | 0.01035 | -0.9835826 |
| Prmt1 | 0.00144 | -1.12055517 |
| Dyrk1b | 0.00506 | -0.88678157 |
| Slc9b1 | 0.01205 | -1.29746368 |
| BC039771 | 0.00144 | -1.29085005 |
| Dcc | 0.02065 | -1.3170094 |
| Gm7467 | 0.05735 | -0.97671778 |
| Enpp5 | 0.018 | 0.87654108 |
| A630077J23Rik | 0.0624 | 1.55809662 |
| Chrdl2 | 0.32758 | -1.16134453 |
| Abcb8 | 0.01677 | -0.88752511 |
| Abcb7 | 0.06391 | -0.37458475 |
| C87414 | 0.00219 | -1.38514181 |
| Ntf3 | 0.07625 | -0.50649629 |
| Tbrg1 | 0.01317 | -0.53837125 |
| Ptcd1 | 0.84158 | -0.0794098 |
| Myocd | 0.10023 | -0.38481866 |
| LOC105242500 | 0.01433 | -0.70374899 |
| Adra2a | 0.01699 | -1.11804231 |
| Gm16386 | 0.17545 | 0.29110064 |
| Phldb2 | 0.00284 | 1.49953493 |
| Gm1096 | 0.03565 | -0.86198882 |
| Morn3 | 0.49441 | -0.22816606 |
| Celf4 | 0.00392 | -0.98023434 |
| Celf4 | 0.45442 | -0.14156797 |
| Vmn2r65 | 0.01682 | -1.72330833 |
| Wapal | 0.00144 | 1.58529564 |
| Klhl15 | 0.70446 | 0.1941134 |
| LOC102636349 | 0.00163 | -1.01715634 |
| Iqca | 0.01295 | -1.11776409 |
| Hpse | 0.27441 | 0.44654 |
| Nxnl1 | 0.08331 | -0.91134937 |
| Zfp248 | 0.00152 | 1.97885508 |
| Crlf3 | 0.17171 | 0.24919815 |
| Crlf3 | 0.56894 | -0.35458648 |
| Cltb | 0.07139 | -0.47076604 |
| BB287469 | 0.02233 | -1.7572958 |
| Sun2 | 0.01458 | -0.79199522 |
| Olfr613 | 0.10226 | -2.70606619 |
| Tmem181a | 0.65708 | -0.07180931 |
| E130308A19Rik | 0.15451 | 0.270514 |
| Ugp2 | 0.00261 | 1.1682216 |
| Tigd3 | 0.3163 | -0.39294272 |
| Tigd4 | 0.13174 | -1.01940487 |
| Sfi1 | 0.60382 | -0.22326636 |
| Gm10190 | 0.06395 | -1.27461162 |
| Trim42 | 0.26541 | -0.6580016 |
| Pla2g2f | 0.42019 | -0.47788499 |
| Trip11 | 0.00291 | -0.84477156 |
| B020004C17Rik | 0.11344 | -1.62063976 |
| Rps6ka6 | 0.00144 | -1.29878664 |
| Parp16 | 0.83696 | -0.03899414 |
| E2f7 | 0.18499 | -0.42850837 |
| Tmem234 | 0.00464 | -0.6956394 |
| Vmn1r32 | 0.00523 | -1.55785214 |
| Man1b1 | 0.00542 | -0.65430432 |
| Prkar1a | 0.10221 | -0.39258725 |
| Prkar1a | 0.25268 | -0.57954131 |
| Atoh7 | 0.00144 | -1.59421439 |
| Abcc10 | 0.35231 | -0.15588393 |
| Brs3 | 0.00145 | -1.23347991 |
| Gm16505 | 0.00465 | 2.14219974 |
| LOC102633724 | 0.03071 | -3.29536846 |
| Pacsin1 | 0.00269 | -0.81782282 |
| Trim3 | 0.28694 | -0.23055449 |
| Pcolce | 0.00458 | -0.81268081 |
| Psme3 | 0.04156 | -0.52922391 |
| Iscu | 0.01985 | -0.4811419 |
| Iscu | 0.68052 | -0.19422448 |
| BC089491 | 0.11639 | -0.44392054 |
| Dcaf12l1 | 0.16955 | 0.42632763 |
| Cyp2c50 | 0.00144 | -1.24811894 |
| Rufy2 | 0.69137 | -0.12821752 |
| Hmgn2 | 0.09865 | -0.43620248 |
| Vmn1r31 | 0.0276 | -1.92503148 |
| Hnrnpa3 | 0.00235 | 1.62210505 |
| Elavl2 | 0.00145 | -1.22376446 |
| Slc22a26 | 0.0027 | -1.11012596 |
| Mapk8 | 0.24796 | -0.30239849 |
| Gm8765 | 0.00144 | -1.46547784 |
| Gm11543 | 0.11796 | -0.47292988 |
| Lzts3 | 0.69893 | -0.2183342 |
| Camk2g | 0.60729 | -0.11209906 |
| Scml4 | 0.06561 | 0.89595522 |
| Camk1g | 0.97758 | 0.00783913 |
| Skint2 | 0.06106 | -3.10633687 |
| Aasdh | 0.01117 | 0.7805873 |
| Wdr76 | 0.00258 | -1.18828104 |
| Sik2 | 0.01957 | -0.47852564 |
| Usp50 | 0.00149 | -1.02751818 |
| Smok4a | 0.0054 | -1.81265336 |
| Sfrp5 | 0.00144 | -2.1115107 |
| Snx6 | 0.05086 | 0.82271287 |
| Cycs | 0.78809 | -0.08267871 |
| Zfp239 | 0.0019 | -1.13608632 |
| Slc39a3 | 0.01322 | -0.70383506 |
| I830134H01Rik | 0.13218 | -3.02371845 |
| Dhrs3 | 0.01291 | -0.73593921 |
| Mum1l1 | 0.00225 | 1.30886263 |
| 6720475J19Rik | 0.81039 | 0.04205981 |
| Adra1d | 0.40051 | -0.41685133 |
| Spopl | 0.00144 | 2.19595489 |
| Gm12942 | 0.21165 | -0.22147048 |
| D730005E14Rik | 0.92033 | -0.05876252 |
| Rnf20 | 0.37831 | 0.17556886 |
| Olfr1360 | 0.00144 | -1.56603655 |
| Lrrtm1 | 0.04437 | -0.87222379 |
| Gxylt1 | 0.03033 | 1.2204147 |
| Paox | 0.003 | -0.95776154 |
| Oas2 | 0.05875 | 0.41599078 |
| Samhd1 | 0.31386 | -0.19128134 |
| 1700061G19Rik | 0.02851 | -1.85677191 |
| Slc43a1 | 0.46746 | -0.14211488 |
| Bspry | 0.05567 | -1.2356839 |
| Nps | 0.00144 | -1.28116048 |
| Col19a1 | 0.80401 | -0.14703327 |
| Vmn2r94 | 0.00144 | -1.27730451 |
| Neil1 | 0.08291 | -0.54620337 |
| Csprs | 0.50277 | -0.15344657 |
| 1500011B03Rik | 0.6236 | -0.15732243 |
| Vmn2r62 | 0.01525 | -1.99465422 |
| Vmn2r62 | 0.01638 | -1.81061526 |
| Mrgpra2b | 0.8779 | 0.03819638 |
| Cnga1 | 0.0048 | -1.10366211 |
| Cnga4 | 0.02817 | -1.76054706 |
| Zscan4c | 0.00144 | -1.41242282 |
| Cma2 | 0.0027 | -1.00123071 |
| Mocs2 | 0.18656 | 0.22448919 |
| Ndrg4 | 0.04338 | 0.64971589 |
| Tnks1bp1 | 0.01534 | -0.56691672 |
| Eno2 | 0.03373 | -0.54203359 |
| Ano2 | 0.00186 | -1.16706471 |
| Htr1d | 0.02406 | -0.83545089 |
| Htr1f | 0.04739 | -1.00895677 |
| Mtch1 | 0.00205 | -1.03858892 |
| Olfr1118 | 0.00149 | -1.19348234 |
| Deptor | 0.02065 | -1.04678864 |
| Gm17026 | 0.38019 | -0.28174966 |
| Txnrd2 | 0.00144 | -1.2383131 |
| Per1 | 0.42693 | 0.28848537 |
| Pcdh15 | 0.00228 | -1.11808857 |
| Aim1l | 0.04823 | -0.63302686 |
| Gm8220 | 0.00144 | -1.26560808 |
| Trpc4ap | 0.01702 | -0.60922535 |
| Gsto2 | 0.10532 | -0.52829329 |
| Satl1 | 0.00144 | -1.24811894 |
| Fmn1 | 0.40219 | -0.17406212 |
| Fmn1 | 0.00144 | -1.47614798 |
| AA414768 | 0.00192 | -0.92593133 |
| Apol7b | 0.20542 | 0.45076273 |
| Rps6kb1 | 0.09637 | 0.48703018 |
| Flii | 0.00526 | -0.75698235 |
| Acaca | 0.31018 | -0.25104348 |
| Acacb | 0.00144 | -1.25944325 |
| Tmem120b | 0.03798 | -0.4758413 |
| Ncoa7 | 0.22771 | 0.40537453 |
| Fbxo7 | 0.00397 | -0.69228596 |
| Snx27 | 0.28465 | -0.24559387 |
| Spns2 | 0.00338 | -0.90051838 |
| Spns1 | 0.00144 | -1.17612058 |
| Kbtbd6 | 0.2759 | -0.51805009 |
| Defa-rs4 | 0.00144 | -1.31495282 |
| Defa-rs7 | 0.00144 | -1.27165604 |
| Defa-rs7 | 0.58532 | -0.16845298 |
| Vmn2r28 | 0.00144 | -1.28375735 |
| Trim30e-ps1 | 0.00145 | -1.24191372 |
| Kxd1 | 0.04612 | -0.6016468 |
| Ldhd | 0.56251 | -0.18503513 |
| Olfr1362 | 0.07334 | -0.95678824 |
| LOC105242515 | 0.00144 | -1.28116048 |
| Asb15 | 0.14695 | 0.91593803 |
| Thrsp | 0.9185 | 0.01693005 |
| Cfap74 | 0.0069 | -1.28976089 |
| 9530077C05Rik | 0.14271 | -0.98167524 |
| 4930590L14Rik | 0.09005 | -0.87777517 |
| Morf4l1 | 0.00144 | 1.40390585 |
| Gcsh | 0.51803 | -0.14891227 |
| Gorasp2 | 0.03603 | -0.71399289 |
| 2410127L17Rik | 0.0794 | 0.36534723 |
| Hdac7 | 0.00144 | -1.23267415 |
| Tas2r121 | 0.00144 | -1.28116048 |
| Tbpl1 | 0.47171 | 0.3561426 |
| 6430628N08Rik | 0.19425 | -0.60643351 |
| Osbpl3 | 0.89841 | 0.03429828 |
| Sycp3 | 0.02142 | -0.92309563 |
| Vmn2r66 | 0.111 | -2.9091872 |
| Lmo4 | 0.88864 | -0.02665849 |
| Tbx10 | 0.00738 | -0.92342601 |
| Sgip1 | 0.13413 | -2.03545329 |
| Sgip1 | 0.00184 | -1.45290105 |
| Gm35185 | 0.00158 | -1.2446827 |
| A730008H23Rik | 0.00144 | 1.00250734 |
| Pabpc4l | 0.17625 | -0.91763571 |
| Mettl7a1 | 0.00294 | -1.4803666 |
| Arap1 | 0.01483 | -0.58873596 |
| 1700010L04Rik | 0.00144 | -1.29281267 |
| Cd209a | 0.00144 | -1.50295106 |
| Vps4b | 0.02285 | 1.23062229 |
| Prpf31 | 0.09403 | -0.63425308 |
| Tnfsf10 | 0.06538 | 0.80357198 |
| Abcg4 | 0.04816 | -0.72165447 |
| Abcg4 | 0.25329 | -0.32978317 |
| Pde8b | 0.74737 | -0.16320075 |
| Pde8b | 0.35041 | 0.20179177 |
| Gm525 | 0.08399 | -0.80576654 |
| Slc7a5 | 0.74283 | -0.13530511 |
| Unc13a | 0.00144 | -1.35019362 |
| Lamc3 | 0.23469 | -0.29237711 |
| Clu | 0.01989 | -0.68333204 |
| A630089N07Rik | 0.06576 | 0.67961698 |
| Csf1 | 0.05367 | -0.39485679 |
| Syt2 | 0.00144 | -1.25118919 |
| 2810417H13Rik | 0.0743 | 0.59049216 |
| Olfr1160 | 0.47943 | -0.38195711 |
| LOC102641427 | 0.00144 | -1.19270867 |
| Ehbp1 | 0.88077 | 0.03998942 |
| Myb | 0.00144 | -1.28898079 |
| AY761185 | 0.00144 | -1.27165604 |
| B020031M17Rik | 0.00144 | 4.47420253 |
| Cep89 | 0.14473 | -0.26676776 |
| Evi2a | 0.2627 | -0.62245907 |
| Aldh16a1 | 0.63445 | -0.261509 |
| Cdc45 | 0.0776 | -0.5245133 |
| Gm2138 | 0.14392 | 0.41150571 |
| 1700003F12Rik | 0.03726 | -0.64369303 |
| Kat5 | 0.00541 | -0.67343403 |
| Sgol2b | 0.17184 | 0.50211756 |
| Gprc5c | 0.00144 | -1.32685116 |
| Sly | 0.00144 | -1.27730451 |
| Tap2 | 0.00495 | -0.7709874 |
| Crmp1 | 0.28696 | -0.24377521 |
| Exog | 0.57761 | -0.09515935 |
| Spaca6 | 0.01431 | -1.3926034 |
| Cldn34c1 | 0.30806 | 0.26821547 |
| Disp2 | 0.00144 | -1.22925777 |
| Rbm43 | 0.18141 | 0.29672461 |
| Nfyb | 0.19764 | -0.47642803 |
| Gm7168 | 0.00144 | -1.27585605 |
| Tdp1 | 0.36195 | 0.63926545 |
| Gm4788 | 0.00144 | 1.72598769 |
| Gm11529 | 0.00144 | -1.26560808 |
| Olfr424 | 0.00144 | -1.29878664 |
| Ina | 0.05568 | -0.37031541 |
| Prr36 | 0.74624 | -0.11862514 |
| Krtap11-1 | 0.87252 | 0.04430347 |
| Olfr767 | 0.00144 | -1.25118919 |
| Olfr767 | 0.00605 | -1.08150631 |
| Fam46d | 0.00144 | -1.31884045 |
| Eapp | 0.17953 | 0.25898603 |
| Olfr815 | 0.40359 | -0.53600803 |
| Olfr615 | 0.00149 | -1.28183002 |
| Olfr715 | 0.00144 | -1.20555712 |
| Olfr215 | 0.20232 | -0.57321489 |
| Nup188 | 0.02489 | -0.51876025 |
| Serpinb12 | 0.02477 | -3.87021413 |
| Vmn2r89 | 0.00144 | -1.24900642 |
| Vmn2r88 | 0.18727 | -0.82376857 |
| Ezh1 | 0.78859 | -0.09608383 |
| Hnrnpa2b1 | 0.00144 | 2.26650858 |
| Zfp54 | 0.00144 | 1.98299955 |
| Pou1f1 | 0.41616 | -0.5686593 |
| Cbx3 | 0.02206 | -0.62835227 |
| Zfp729a | 0.00321 | 1.34735222 |
| Gm10563 | 0.00144 | -1.20555712 |
| Gm8213 | 0.00144 | -1.14056395 |
| Nme2 | 0.01915 | -0.70741745 |
| Gm5607 | 0.00144 | -1.30713006 |
| Arpp21 | 0.45577 | -0.23832044 |
| Ppfia4 | 0.02303 | -0.66485054 |
| Sdccag3 | 0.18102 | -0.23888394 |
| Nlrp4c | 0.00145 | -1.22376446 |
| Lce1a2 | 0.42359 | -0.17316006 |
| Me1 | 0.00463 | -0.74670015 |
| Inpp4b | 0.01682 | 0.56854679 |
| Rbm12b2 | 0.01302 | 0.77157161 |
| Slc30a2 | 0.985 | 0.00877619 |
| Msl1 | 0.16452 | 0.24485486 |
| 1700039I01Rik | 0.00847 | -1.14366197 |
| Alkbh7 | 0.00482 | -1.08847843 |
| Gstm7 | 0.47849 | 0.44932164 |
| Gstm7 | 0.31862 | 0.28669586 |
| Neurl1a | 0.00575 | -0.83385125 |
| Gm10829 | 0.00144 | -1.27992547 |
| Cry2 | 0.01304 | -0.5907667 |
| Lrrc49 | 0.04596 | 0.72739096 |
| Lrrc69 | 0.00144 | -1.27454685 |
| Tmem131 | 0.70746 | -0.06933174 |
| Ddx39b | 0.56512 | -0.17732636 |
| Cxcl10 | 0.884 | -0.10885039 |
| Cxcl10 | 0.00144 | -1.28116048 |
| Nova1 | 0.01931 | 0.90366533 |
| Eif3k | 0.00282 | -0.96916198 |
| Mctp1 | 0.88018 | -0.03270022 |
| Rhox3f | 0.24048 | 0.25114427 |
| Eif3c | 0.06792 | -0.34154049 |
| Eif3d | 0.00144 | -0.95908907 |
| Xrn2 | 0.00149 | 1.03566689 |
| Fbxo40 | 0.01778 | -0.77850738 |
| Isl2 | 0.01882 | -1.07465098 |
| Ctnnal1 | 0.00144 | -1.25512862 |
| Ssc4d | 0.13366 | -0.90553493 |
| Ssc4d | 0.00144 | -1.28116048 |
| Mrpl47 | 0.02447 | 0.61790867 |
| Aaed1 | 0.00144 | 1.37749529 |
| Olfr586 | 0.7062 | 0.1457097 |
| Slc1a7 | 0.17966 | -0.27709478 |
| Atp6ap1l | 0.10665 | -0.70615611 |
| Sgcb | 0.5678 | 0.15280921 |
| Fasl | 0.00144 | -1.26153594 |
| Errfi1 | 0.03839 | 1.28456261 |
| Gpr31b | 0.00383 | -1.16044081 |
| Erich4 | 0.31914 | -0.2611497 |
| Adamtsl1 | 0.19695 | -1.57419299 |
| Nlrc5 | 0.78192 | 0.13259294 |
| Kif3c | 0.01052 | -0.71879721 |
| Cpsf4l | 0.00144 | -1.25118919 |
| Cd22 | 0.33451 | 0.35799111 |
| Fgf9 | 0.34551 | 0.19021 |
| BC003331 | 0.00144 | 3.21004567 |
| Rif1 | 0.00247 | 1.13047739 |
| Cpa5 | 0.32659 | -0.67653953 |
| Zfp961 | 0.04471 | 0.92703685 |
| Zfp961 | 0.90717 | -0.04596066 |
| Wwox | 0.79677 | 0.06523876 |
| Clps | 0.14937 | -0.67437049 |
| Plcg1 | 0.45305 | 0.16260618 |
| Gm18762 | 0.00144 | -1.25118919 |
| Mtmr3 | 0.21467 | -0.20666982 |
| Stmnd1 | 0.00145 | -1.18741414 |
| Cggbp1 | 0.07646 | 0.4492428 |
| Rab4a | 0.03837 | -0.5310244 |
| Evi5l | 0.44723 | -0.20389136 |
| Phf11d | 0.00883 | 1.05804335 |
| Itga6 | 0.18587 | 0.31671581 |
| Slc5a9 | 0.20977 | -0.51120521 |
| Gm5766 | 0.02633 | -0.56436497 |
| Olfr1012 | 0.03002 | -1.93227475 |
| Foxp4 | 0.02691 | -0.55228427 |
| LOC102642270 | 0.13798 | -0.67684065 |
| Hif3a | 0.00866 | 0.76129762 |
| Plekha1 | 0.17252 | 0.27211146 |
| Abat | 0.01218 | 1.38647251 |
| Cfap45 | 0.26696 | 0.45893327 |
| 0610010K14Rik | 0.03217 | -0.69051409 |
| 9930111J21Rik1 | 0.5316 | -0.32616831 |
| 9930111J21Rik1 | 0.0253 | 0.8237821 |
| Gm10535 | 0.00893 | -1.61510759 |
| Efcab7 | 0.2105 | -0.27956162 |
| Ltc4s | 0.00479 | -1.00923549 |
| Cyld | 0.80844 | 0.05131641 |
| Pde11a | 0.00653 | -1.79934369 |
| Ggnbp2os | 0.69401 | -0.06380924 |
| Glt1d1 | 0.00145 | -1.25646236 |
| Nap1l1 | 0.01004 | 0.74026255 |
| LOC102643183 | 0.00144 | -1.28116048 |
| Hhat | 0.07784 | -1.11683587 |
| Frmpd4 | 0.09162 | -2.46372013 |
| Atg5 | 0.18942 | 0.29207974 |
| LOC102632416 | 0.00492 | 0.88647979 |
| Gm10856 | 0.32425 | 0.33035886 |
| Zfp46 | 0.40221 | -0.14862109 |
| Amz2 | 0.39653 | 0.22969914 |
| Gatsl2 | 0.00177 | -0.90899747 |
| Dvl3 | 0.00144 | -1.76302786 |
| LOC102632231 | 0.00144 | -1.24900642 |
| Zfp341 | 0.13138 | -0.42099337 |
| Dhrs9 | 0.00144 | -1.29746368 |
| Vmn1r44 | 0.00144 | -1.28898079 |
| Vmn1r41 | 0.00144 | -1.28898079 |
| Olfr1281 | 0.5779 | -0.516843 |
| Defb25 | 0.00212 | -1.17524536 |
| Stk-ps1 | 0.0083 | -1.72402943 |
| Pclo | 0.09622 | 0.76398897 |
| Pclo | 0.05027 | -0.91989204 |
| Naip5 | 0.38441 | 0.22798646 |
| Taok3 | 0.42935 | -0.13391121 |
| Olfr800 | 0.0049 | -1.08617297 |
| Vmn2r97 | 0.00158 | -1.2446827 |
| Knop1 | 0.04709 | 0.3547703 |
| Ccdc82 | 0.00144 | 1.79674334 |
| Megf11 | 0.00144 | -1.40870584 |
| Zfp961 | 0.00216 | 0.94690762 |
| Ces5a | 0.00144 | -1.24811894 |
| Lrp8 | 0.00144 | -1.29085005 |
| BC068281 | 0.83597 | 0.07255112 |
| Hes7 | 0.01373 | -0.53063475 |
| Zfp787 | 0.01231 | -0.944813 |
| Amd2 | 0.00857 | -0.99564452 |
| Amd2 | 0.28142 | 0.30104461 |
| N28178 | 0.53101 | -0.17643216 |
| B430105G09Rik | 0.00607 | -0.85943724 |
| Il17re | 0.6989 | -0.152211 |
| LOC102643057 | 0.08805 | -1.94961052 |
| Olfr94 | 0.16416 | -0.92531742 |
| Olfr54 | 0.08174 | -2.43092725 |
| Pdpk1 | 0.74389 | -0.05592865 |
| Pdpk1 | 0.04822 | -0.54648402 |
| Mbtps2 | 0.37072 | -0.17844391 |
| Bach1 | 0.00195 | 0.98995426 |
| Cntln | 0.00232 | 1.60368831 |
| Gm33698 | 0.00168 | 1.7139711 |
| Armcx6 | 0.05082 | -0.37517356 |
| Kank1 | 0.87221 | -0.03455036 |
| Serpina11 | 0.00144 | -1.38278225 |
| Zfp771 | 0.0081 | -0.79188239 |
| Zfp82 | 0.53346 | 0.11021028 |
| Sema4a | 0.04824 | -0.59044865 |
| Sema5a | 0.04377 | 0.64422496 |
| Scyl2 | 0.02988 | 0.50963452 |
| 9130004C02Rik | 0.00532 | -1.51726521 |
| Ppa2 | 0.21231 | -0.25839636 |
| Cacna1c | 0.33401 | 0.20624002 |
| Olfr830 | 0.0149 | -1.39879907 |
| Gjb3 | 0.00145 | -1.25646236 |
| Atp5j | 0.05714 | 0.38160875 |
| Gm7120 | 0.89304 | -0.09655861 |
| Ercc2 | 0.00423 | -0.68324222 |
| 1700092M07Rik | 0.75584 | 0.15482494 |
| Rab14 | 0.03062 | 0.50186369 |
| Gm7285 | 0.20375 | 0.33204435 |
| Gm29795 | 0.74451 | 0.23097461 |
| Cntn3 | 0.00149 | -1.22582153 |
| Cntn4 | 0.01347 | -1.01042984 |
| Cntn4 | 0.00144 | -1.30737044 |
| Gm3718 | 0.3212 | 0.29265751 |
| 1700028J19Rik | 0.15146 | -0.29180564 |
| Krt42 | 0.00144 | -1.83006326 |
| Gal3st1 | 0.04129 | -1.5534481 |
| Zfp654 | 0.52944 | -0.09542241 |
| Raly | 0.01758 | -0.65573704 |
| Slc39a14 | 0.18462 | 0.28175279 |
| Sult5a1 | 0.07337 | -0.86419189 |
| Tsga10 | 0.28096 | 0.65046433 |
| Tsga10 | 0.85472 | -0.150388 |
| Islr2 | 0.00207 | -1.14521696 |
| Gm5346 | 0.00149 | -1.28183002 |
| Napa | 0.04783 | -0.50243651 |
| Apoc3 | 0.26938 | -0.68413366 |
| LOC101055915 | 0.00183 | -0.81093308 |
| Mybpc2 | 0.17532 | -0.46361892 |
| Cd4 | 0.00582 | -1.00659697 |
| Tubg2 | 0.00202 | -0.92870041 |
| Ldb2 | 0.46995 | 0.17150059 |
| Agbl4 | 0.00163 | -1.21015905 |
| Agbl4 | 0.67976 | -0.12306301 |
| Slc25a19 | 0.11903 | -0.5138467 |
| Gm14743 | 0.00145 | -1.2388121 |
| Dmwd | 0.02151 | -0.57765045 |
| Gm4402 | 0.00277 | -0.90638562 |
| Ttc29 | 0.18362 | -0.73113778 |
| Renbp | 0.00877 | -0.63398777 |
| Renbp | 0.89351 | -0.05162923 |
| Melk | 0.78674 | -0.13572513 |
| Gm9573 | 0.00145 | -1.22376446 |
| Ankrd53 | 0.003 | -1.33816652 |
| Prkag2 | 0.01237 | -0.54010326 |
| 2410125D13Rik | 0.02792 | 0.79408844 |
| S100z | 0.00144 | -1.29864962 |
| Oxtr | 0.03505 | -1.02384043 |
| Cdh8 | 0.00144 | -1.28116048 |
| Dnajb6 | 0.05178 | -0.38515591 |
| Atp2b3 | 0.00144 | -1.29051721 |
| Brat1 | 0.05733 | -0.46699893 |
| Esrp2 | 0.77844 | 0.13035884 |
| Bak1 | 0.00159 | -1.01926938 |
| Arpc4 | 0.00312 | -1.06076363 |
| Thoc5 | 0.08617 | -0.33467149 |
| Shb | 0.02886 | -0.89006259 |
| Serpinb6b | 0.00815 | 0.8237791 |
| LOC102632383 | 0.61462 | -0.14941281 |
| Lpar1 | 0.23226 | -0.2991095 |
| Ank3 | 0.0974 | -0.53057792 |
| Ldha | 0.02377 | -0.64341753 |
| Sdha | 0.07658 | 0.35979959 |
| Arid5b | 0.54847 | -0.43571899 |
| Sbspon | 0.00144 | -1.85535806 |
| Cfap221 | 0.01299 | -0.90441091 |
| Dock10 | 0.59338 | 0.19270215 |
| Sec61a2 | 0.1725 | 0.34246476 |
| Acvr2b | 0.00155 | -1.03097083 |
| Rtkn | 0.01634 | -0.49398587 |
| Nmbr | 0.00159 | -1.34329818 |
| Impa1 | 0.18754 | 0.27663488 |
| Rnf167 | 0.00144 | -1.06115793 |
| Rnf167 | 0.00463 | -0.79103679 |
| Ccdc64 | 0.03422 | -0.57442732 |
| B930041F14Rik | 0.00294 | -1.07873266 |
| Kcnh2 | 0.02432 | -0.77393515 |
| Kcnh6 | 0.00145 | -1.2388121 |
| Kcnh7 | 0.00144 | -1.32766452 |
| Stk16 | 0.88577 | -0.05036567 |
| Gm10536 | 0.87412 | 0.05757193 |
| Olfr1333 | 0.00715 | -1.17660698 |
| Cacnb1 | 0.2827 | -0.29453151 |
| Gm10847 | 0.00396 | 1.3673225 |
| Twf1 | 0.00688 | 0.84846697 |
| Cdca7 | 0.2096 | -0.2660599 |
| Sfxn3 | 0.02694 | -0.45712716 |
| Smim14 | 0.00365 | -1.3896922 |
| Gopc | 0.00144 | 1.50133853 |
| Pabpc6 | 0.00474 | -1.10247618 |
| Vmn1r121 | 0.00144 | -1.27699683 |
| Ctso | 0.0264 | 1.79892111 |
| Armcx2 | 0.68509 | -0.0805252 |
| Ooep | 0.17581 | -0.40149645 |
| Gm6444 | 0.00244 | -1.10802194 |
| Eps15l1 | 0.44728 | 0.14590505 |
| Gm31005 | 0.00144 | -1.30017714 |
| Spata6 | 0.00475 | 1.06720404 |
| Veph1 | 0.03231 | -1.97450864 |
| Vmn2r10 | 0.00144 | -1.60371653 |
| Bmp8a | 0.51801 | 0.25015607 |
| Sh2d1a | 0.00144 | -1.26934956 |
| Sox1 | 0.27198 | -0.29808058 |
| Gigyf1 | 0.00188 | -0.97093373 |
| Tceal7 | 0.52757 | -0.72295944 |
| St7 | 0.3538 | 0.15210083 |
| Ebf4 | 0.05421 | -0.43535775 |
| Akirin2 | 0.9585 | -0.0093823 |
| Gramd1a | 0.04842 | -0.37321564 |
| Gramd1a | 0.43068 | 0.14556768 |
| Fignl1 | 0.00297 | 1.20140289 |
| Ecm2 | 0.02144 | 0.75107132 |
| Kif13a | 0.7364 | -0.06014146 |
| Scd4 | 0.08619 | 0.85745647 |
| Kif19a | 0.64132 | 0.07886991 |
| Cdk2 | 0.91155 | 0.03362595 |
| Pdzrn4 | 0.32309 | -0.38995015 |
| Syna | 0.04501 | -1.18481727 |
| Pdk2 | 0.0188 | -0.64604494 |
| Gm5493 | 0.02925 | -1.55415291 |
| Lsg1 | 0.32664 | -0.18103248 |
| Gm6938 | 0.02253 | -1.71242648 |
| Neurl1b | 0.02363 | -1.08920263 |
| Gm3364 | 0.00568 | 1.83575418 |
| 2610005L07Rik | 0.09582 | 0.65332857 |
| Pdzk1ip1 | 0.04113 | -0.82374063 |
| Rpl17 | 0.96915 | 0.0069593 |
| Gpr141 | 0.00144 | -1.24900642 |
| Lyst | 0.06225 | -0.77546258 |
| Gpr101 | 0.0962 | -1.30942903 |
| Adgrf2 | 0.00144 | -1.27165604 |
| F2rl3 | 0.00491 | -0.92538409 |
| Trav6-3 | 0.46647 | 0.6481688 |
| 1600010M07Rik | 0.47799 | -0.12219926 |
| 1810009J06Rik | 0.00145 | -1.22376446 |
| Apol7d | 0.00879 | -1.40845069 |
| Rnf214 | 0.10044 | 0.34721063 |
| Ralgapb | 0.45937 | -0.13381703 |
| Gm11223 | 0.0945 | -0.40271706 |
| Atp10b | 0.00144 | -1.27165604 |
| Pdzk1 | 0.28962 | -0.54013935 |
| Tead1 | 0.24632 | 0.5223156 |
| Tifa | 0.89751 | -0.04381795 |
| Acvrl1 | 0.00144 | -1.23496763 |
| Cyb561a3 | 0.18418 | -0.85992578 |
| Gm6598 | 0.26523 | -0.28785852 |
| Fggy | 0.20505 | -0.25865062 |
| Hopx | 0.00474 | -0.77829409 |
| Rpl13 | 0.0017 | -0.91300088 |
| LOC102641347 | 0.11999 | -0.63608216 |
| Lce1j | 0.01297 | -0.61514844 |
| Kncn | 0.00144 | -1.27165604 |
| Tmprss3 | 0.01578 | -0.56433552 |
| Reep1 | 0.00549 | 0.8691745 |
| Armc10 | 0.51102 | -0.12417826 |
| Meiob | 0.11166 | -0.45320718 |
| Junb | 0.08272 | 0.52066113 |
| Gsdmc3 | 0.00144 | -1.31884045 |
| Gsdma2 | 0.00144 | -1.47295486 |
| Gsdma2 | 0.00479 | -1.73535992 |
| Gm1604b | 0.07343 | -0.66938471 |
| Gnptg | 0.21696 | -0.21997438 |
| Tspan12 | 0.00689 | 0.87093806 |
| Abca16 | 0.02673 | -1.21977358 |
| Cdk5rap1 | 0.29743 | 1.20534198 |
| Gm10509 | 0.00144 | 1.89336347 |
| Zfp688 | 0.00765 | -0.69085229 |
| Surf6 | 0.04886 | 0.37157818 |
| Gm4971 | 0.31077 | 0.28628638 |
| Fbxo17 | 0.0524 | -0.4816471 |
| Jak3 | 0.01078 | -0.63274297 |
| Daam2 | 0.18976 | -0.22457283 |
| Mrps23 | 0.06971 | -0.49759647 |
| Olfr59 | 0.05196 | -0.99089022 |
| Il11ra1 | 0.00152 | -1.1298469 |
| Dpm3 | 0.00144 | -1.27733755 |
| BC037034 | 0.05628 | -0.39828504 |
| Gm2164 | 0.00913 | -2.07247988 |
| Kif17 | 0.00144 | -1.28116048 |
| Sec16b | 0.28579 | 0.31231262 |
| Tdpoz2 | 0.00149 | -1.19696847 |
| Gm6252 | 0.00269 | 2.14398935 |
| 1700029M03Rik | 0.00472 | -1.02583646 |
| Dlx1as | 0.00191 | -1.4076954 |
| Unk | 0.05595 | -0.37914679 |
| Pde4d | 0.0972 | -0.53750165 |
| Pde4d | 0.00674 | -1.71477793 |
| Tspan32 | 0.01559 | -0.99341325 |
| Ewsr1 | 0.55821 | 0.09410715 |
| Ewsr1 | 0.34775 | 0.38312306 |
| Serpina1a | 0.00166 | -1.30175691 |
| Serpina1c | 0.18746 | -1.05930962 |
| Serpina1d | 0.12274 | -1.16654348 |
| Serpina1f | 0.47147 | -0.27340931 |
| Gabarap | 0.01027 | -0.78321626 |
| Serpina10 | 0.02041 | -1.16501851 |
| Fastkd5 | 0.22063 | 0.21027221 |
| Sell | 0.0065 | 0.94207659 |
| Rab27b | 0.90038 | -0.06332539 |
| Zxdc | 0.00187 | -0.84479218 |
| Mup2 | 0.00144 | -2.12264266 |
| Fam73b | 0.00713 | -0.73626726 |
| Capn3 | 0.46028 | -0.17384639 |
| Capn3 | 0.10339 | -0.8216566 |
| Tnfsf9 | 0.62721 | -0.13094886 |
| Zfp113 | 0.50196 | 0.22023884 |
| Trib3 | 0.00145 | -1.25825171 |
| Ttc3 | 0.14746 | 0.3417417 |
| Cd300lg | 0.03494 | -1.42198991 |
| Me1 | 0.68266 | 0.06985394 |
| Spen | 0.18586 | -0.24127854 |
| Gm5113 | 0.09213 | -0.33860207 |
| Olfr781 | 0.00144 | -1.31495282 |
| Pld5 | 0.57015 | -0.29292565 |
| Gm5751 | 0.65002 | -0.12854933 |
| Ssxb8 | 0.00144 | -1.31495282 |
| Mapkapk3 | 0.04066 | -1.06270987 |
| Mapkapk3 | 0.17762 | -0.48327152 |
| Prr9 | 0.00425 | -0.72672013 |
| Zmiz1 | 0.82029 | -0.15694176 |
| Zmiz1 | 0.04557 | -0.37713876 |
| Ppp1r14d | 0.00144 | -1.29051721 |
| S100pbp | 0.05806 | 1.35789415 |
| S100pbp | 0.12002 | 0.47373939 |
| Rpl13 | 0.00174 | -0.91391325 |
| Rnu12 | 0.00571 | -0.86476733 |
| Ptbp3 | 0.85362 | -0.17470982 |
| Ptbp3 | 0.07216 | 0.56393012 |
| Ccdc88a | 0.40054 | -0.18046769 |
| Ocrl | 0.01413 | 0.53558073 |
| Ccdc28a | 0.15741 | -0.33654905 |
| Gm21996 | 0.82863 | 0.1118482 |
| 9530068E07Rik | 0.05568 | -0.35052224 |
| Pnma2 | 0.00144 | 4.42058074 |
| Nrsn2 | 0.02145 | -0.59336041 |
| Gm10487 | 0.00145 | -1.26219879 |
| Ovch2 | 0.04003 | -0.45544587 |
| LOC102642717 | 0.00503 | -1.17452065 |
| 4932414J04Rik | 0.02729 | -1.23952598 |
| Cd34 | 0.29772 | -0.17404951 |
| Rnu3b1 | 0.19882 | -0.30373214 |
| Pde7a | 0.72399 | 0.07077041 |
| Sumo2 | 0.52284 | 0.1336495 |
| Gm5441 | 0.00756 | -0.72178557 |
| Otp | 0.00773 | -1.04351692 |
| Agrn | 0.04982 | -0.79474382 |
| Agrn | 0.07088 | -0.40240304 |
| F830002L21Rik | 0.08107 | 0.56144363 |
| Mad1l1 | 0.00981 | -0.61065039 |
| Sybu | 0.09057 | 0.42811497 |
| Ccrn4l | 0.06731 | 0.39769483 |
| Cfap74 | 0.38966 | 0.17865298 |
| Gprc5d | 0.17135 | -0.34241177 |
| Gm10034 | 0.72539 | 0.15208348 |
| Syp | 0.0479 | -0.40226384 |
| Nnt | 0.7187 | -0.30009205 |
| Slc17a3 | 0.00144 | -1.28116048 |
| Actg1 | 0.02606 | -0.53794485 |
| Tmsb15b1 | 0.52756 | 0.15597505 |
| Cltc | 0.62993 | 0.10043275 |
| Cltc | 0.00144 | 1.08966532 |
| Gm15520 | 0.0046 | -1.12385295 |
| Gm34466 | 0.00144 | -1.29049509 |
| Cdc123 | 0.10231 | 0.35021675 |
| Cox17 | 0.91616 | 0.05638749 |
| Nlk | 0.08669 | 0.43865994 |
| Ktn1 | 0.01286 | 0.92886254 |
| Qdpr | 0.69675 | -0.10823225 |
| Gm6268 | 0.55879 | -0.31430778 |
| Pcdhb10 | 0.01156 | -0.74683775 |
| Lancl2 | 0.04043 | 0.43563372 |
| Rxrb | 0.0018 | -1.03300647 |
| Pcx | 0.27738 | -0.27116096 |
| Kras | 0.74993 | -0.13775402 |
| Gm35189 | 0.00144 | -1.24811894 |
| Chmp4b | 0.04642 | -0.38365518 |
| B4galt7 | 0.0133 | -0.66058983 |
| Gm10468 | 0.00144 | -1.28898079 |
| Wtap | 0.36062 | 0.16600622 |
| Mybpc1 | 0.00144 | -1.28116048 |
| Cdkn2a | 0.00462 | -0.98260954 |
| 5830416I19Rik | 0.58312 | 0.5921809 |
| Mpc1 | 0.45366 | 0.23268052 |
| Mnd1 | 0.0274 | 0.63616493 |
| Coq9 | 0.45313 | -0.33309432 |
| Acadsb | 0.00463 | 1.09064927 |
| Prelid1 | 0.00276 | -0.83850555 |
| Prelid1 | 0.00302 | -0.83237068 |
| Eno1b | 0.0508 | -0.39651197 |
| 1700016L21Rik | 0.00144 | -1.27165604 |
| Larp1b | 0.00176 | 0.95999854 |
| Gdf7 | 0.00547 | -2.0353998 |
| Esr2 | 0.08841 | 0.6383409 |
| Zfp952 | 0.54157 | 0.26073846 |
| Il3ra | 0.00158 | -1.13268659 |
| Zscan12 | 0.00186 | 0.95388463 |
| Gm10857 | 0.00144 | -1.43144742 |
| Rad51 | 0.6263 | -0.8049356 |
| 9630001P10Rik | 0.00145 | -1.22376446 |
| Otud6b | 0.82928 | 0.13256246 |
| Gm12863 | 0.00144 | -1.29051721 |
| Vmn2r91 | 0.00298 | -1.34453613 |
| Rhoa | 0.00299 | -0.72239557 |
| Prkcd | 0.09425 | 0.28421548 |
| Dlg4 | 0.09509 | -0.70410331 |
| Gm15348 | 0.19154 | 0.27682079 |
| Ydjc | 0.00144 | -2.07733611 |
| Srpx2 | 0.00501 | -0.7234511 |
| Armc3 | 0.0871 | -1.01224685 |
| Tuba3b | 0.00192 | -1.24094212 |
| Zscan4f | 0.00144 | -1.39552011 |
| Fmn2 | 0.07754 | 0.34791134 |
| Tmem215 | 0.00472 | -0.71573384 |
| Cx3cr1 | 0.66135 | 0.18827342 |
| Prkce | 0.00511 | -1.10576476 |
| Prss51 | 0.69219 | -0.35542831 |
| Ptpdc1 | 0.72599 | 0.06634882 |
| Ccdc172 | 0.00144 | 1.57021102 |
| Slc12a6 | 0.02248 | -0.52950117 |
| Smarca2 | 0.06673 | 0.5360337 |
| 1700101I19Rik | 0.68717 | -0.32801303 |
| Catsper2 | 0.00144 | 1.53208815 |
| Gm8709 | 0.19046 | -0.48961155 |
| Krt16 | 0.02958 | -0.49684421 |
| Epn1 | 0.00203 | -0.98530106 |
| Nanog | 0.0419 | -0.72937653 |
| Nanog | 0.02658 | -0.48752721 |
| Gm4120 | 0.38583 | -0.14206946 |
| Xntrpc | 0.01039 | -0.65251781 |
| Lrsam1 | 0.01535 | -0.53831561 |
| Alkbh8 | 0.00144 | 2.40576034 |
| Cryaa | 0.18928 | -0.73707107 |
| Cryaa | 0.00144 | -1.24811894 |
| Sez6l2 | 0.00541 | -0.78471731 |
| Sftpd | 0.01519 | -1.01056962 |
| Trmt61b | 0.52927 | 0.12414723 |
| Scgb1b3 | 0.01879 | -0.4909741 |
| Edf1 | 0.00674 | -0.84388521 |
| Pef1 | 0.02143 | -0.80203491 |
| Tmem151b | 0.00144 | -1.27165604 |
| Pdgfa | 0.01053 | -0.6604497 |
| Muc5b | 0.03741 | -1.26205357 |
| Agps | 0.00927 | 0.67266068 |
| Vbp1 | 0.22793 | -0.22354407 |
| Sgca | 0.6772 | -0.14701819 |
| 1700010L04Rik | 0.0266 | -1.3511998 |
| Elf2 | 0.00573 | 0.66483586 |
| Cyp2t4 | 0.26007 | 0.24405266 |
| Mepce | 0.01223 | -0.6045866 |
| Cyp2a4 | 0.00144 | -1.54497436 |
| Pole | 0.90948 | -0.02307775 |
| Spdef | 0.31757 | -0.44527005 |
| Kcnc1 | 0.57214 | 0.51430717 |
| Aldoa | 0.00332 | -0.92110664 |
| Olfr1052 | 0.29783 | -0.4642186 |
| Vmn2r25 | 0.00144 | -1.30713006 |
| Slc5a2 | 0.77861 | -0.2417383 |
| Dnajc21 | 0.24167 | 0.35873439 |
| Rnf128 | 0.61521 | -0.09565169 |
| Vmn1r194 | 0.00167 | -1.68329629 |
| Gm9112 | 0.01295 | -0.73902556 |
| Adgre5 | 0.00885 | -0.62887829 |
| Zscan4a | 0.11318 | -0.85661854 |
| Frmd4b | 0.52853 | 0.17542284 |
| Ctnnbip1 | 0.00144 | -0.98382701 |
| Parp10 | 0.02743 | -0.79850468 |
| Mageb5 | 0.13125 | -2.66517995 |
| Sfmbt1 | 0.00294 | 1.14308388 |
| Pik3c3 | 0.02951 | 0.59666975 |
| Armcx3 | 0.00144 | 1.24875254 |
| Cdk15 | 0.00144 | -1.30361983 |
| Dgkb | 0.08849 | 0.66969446 |
| Olfr1158 | 0.00144 | -1.27165604 |
| Xkr4 | 0.00145 | -1.25200268 |
| Gm26919 | 0.00145 | -1.21051401 |
| Ntrk3 | 0.87989 | -0.05327123 |
| Ntrk2 | 0.00146 | -1.19479945 |
| Bzw1 | 0.15692 | 0.41890032 |
| Mfsd12 | 0.01167 | -0.77016259 |
| Mfsd12 | 0.24176 | -0.61048541 |
| Bmp1 | 0.01224 | -0.54653227 |
| Bmp1 | 0.83393 | -0.03889843 |
| Bmp1 | 0.01354 | -0.62560299 |
| Pstpip2 | 0.30225 | -0.29044787 |
| Olfr1238 | 0.00145 | -1.22376446 |
| LOC102642957 | 0.04498 | 1.41286045 |
| Pi4kb | 0.00144 | -1.21720445 |
| Gm14431 | 0.00367 | 2.33403142 |
| Map4k5 | 0.00247 | 1.12875801 |
| Pcdha12 | 0.00144 | -1.31884045 |
| Pcdhac1 | 0.03504 | -0.90748867 |
| Rbbp9 | 0.00144 | 1.27361612 |
| Eif4e2 | 0.08797 | -0.37034399 |
| Gm1553 | 0.00667 | -1.45727232 |
| Adamtsl4 | 0.0382 | -0.42983807 |
| Eif4a2 | 0.69227 | 0.08034029 |
| Esrrg | 0.00144 | 1.06441691 |
| Apol10b | 0.74171 | -0.12430992 |
| 3300002I08Rik | 0.28806 | 0.377563 |
| Serf2 | 0.00264 | -0.98282312 |
| Olfr482 | 0.40142 | 0.46785463 |
| Olfr282 | 0.23329 | -0.75332952 |
| Olfr382 | 0.00144 | -1.29085005 |
| Emd | 0.03669 | -0.52829662 |
| Defb18 | 0.00434 | -1.0948178 |
| Defb28 | 0.00144 | -1.29085005 |
| Uba52 | 0.00144 | -1.07203246 |
| Ptprg | 0.01265 | 0.59000033 |
| Krt12 | 0.00144 | -1.20052149 |
| Krt13 | 0.04953 | -0.90332903 |
| Ogdh | 0.00144 | -0.97218366 |
| Ogdh | 0.02635 | -0.56843532 |
| Olfr1446 | 0.00283 | -1.6312286 |
| Krt15 | 0.00144 | -1.33632959 |
| Txnip | 0.01434 | 0.52641762 |
| Myo5a | 0.0089 | 1.1765861 |
| Olfr129 | 0.00144 | -1.28898079 |
| Ugdh | 0.05578 | 0.55079891 |
| Fgf22 | 0.00144 | -1.29864962 |
| Lrrc74b | 0.00292 | -0.78757347 |
| Gm11710 | 0.13693 | -1.16664699 |
| Tmc6 | 0.07433 | -0.4673138 |
| Nebl | 0.34971 | 0.3462724 |
| Gm7897 | 0.00144 | -1.28116048 |
| Galnt15 | 0.00145 | -1.4870936 |
| Slc4a2 | 0.01088 | -0.58516124 |
| Lyzl6 | 0.60256 | -0.39486486 |
| Serpinb1a | 0.1284 | -0.26656047 |
| LOC102642485 | 0.00144 | -1.31884045 |
| Wfdc15b | 0.00196 | -1.29222898 |
| Stim1 | 0.00588 | -0.69420522 |
| Egfbp2 | 0.7674 | 0.12654131 |
| Lin9 | 0.03884 | 0.92934115 |
| Lhx6 | 0.24156 | 0.36628449 |
| Atf7ip2 | 0.00144 | -1.31884045 |
| Foxr1 | 0.06922 | -0.87181469 |
| Zfp280d | 0.64004 | 0.23061863 |
| Fn3k | 0.41215 | -0.4315943 |
| Nacc2 | 0.5715 | -0.12654207 |
| Ticrr | 0.0692 | -0.84400391 |
| Adamts8 | 0.01212 | -0.58803271 |
| Hbb-bh1 | 0.36878 | -0.63149388 |
| 3110057O12Rik | 0.09846 | 0.36320245 |
| 3110057O12Rik | 0.12349 | 0.30400128 |
| Gm9271 | 0.00144 | -1.27165604 |
| Uap1 | 0.10073 | 0.29697135 |
| 1700123K08Rik | 0.00716 | -1.021171 |
| Gm6408 | 0.00144 | -1.26560808 |
| Manbal | 0.00563 | -0.71186584 |
| Ubxn11 | 0.05459 | -0.42641213 |
| Trpm2 | 0.06138 | 0.45616627 |
| Gm4251 | 0.00144 | -1.27165604 |
| Gm10651 | 0.5566 | 0.16264263 |
| Ipo11 | 0.45 | 0.34037484 |
| Ipo11 | 0.29229 | 0.1772006 |
| Acyp2 | 0.61432 | 0.12167169 |
| Ntsr1 | 0.06819 | -1.16063361 |
| Olfr676 | 0.58758 | -0.44547589 |
| Olfr476 | 0.00144 | -1.51110598 |
| Olfr576 | 0.6409 | -0.42089103 |
| Olfr376 | 0.00144 | -1.28898079 |
| Trps1 | 0.59675 | -0.19825226 |
| Celf2 | 0.31704 | -0.18126221 |
| Arhgef10l | 0.00625 | -0.92547838 |
| Tnn | 0.00144 | -1.30713006 |
| Krtap12-1 | 0.00583 | -1.04768057 |
| Olfr392 | 0.67426 | 0.18997647 |
| Sult1a1 | 0.0145 | 0.74747571 |
| Olfr1310 | 0.00144 | -1.35870099 |
| Dennd5a | 0.04739 | -0.36993845 |
| Glra3 | 0.00144 | -1.29085005 |
| Epb4.1 | 0.02934 | -0.62042008 |
| Egln2 | 0.00641 | -0.80106533 |
| Dcstamp | 0.02016 | -1.33973889 |
| Zfp672 | 0.01187 | -0.58685265 |
| Gm9753 | 0.22347 | -0.46693238 |
| Ranbp3 | 0.01225 | -0.69677836 |
| Klhl22 | 0.03274 | -0.54100884 |
| Col9a1 | 0.10636 | -1.15041973 |
| Meis3 | 0.26311 | -0.19044901 |
| LOC102639700 | 0.00144 | 2.82209274 |
| 1700023G09Rik | 0.00144 | -1.29746368 |
| Herc4 | 0.0028 | 0.89827687 |
| Fam129a | 0.00144 | -1.32982311 |
| Vmn1r8 | 0.00144 | -1.29051721 |
| Tardbp | 0.0327 | 0.71444129 |
| Rpl10 | 0.01037 | -0.6144063 |
| Gm5621 | 0.00314 | -0.8281202 |
| Ddx43 | 0.14057 | -1.11589195 |
| 1700091H14Rik | 0.00144 | -1.2456552 |
| Dcps | 0.32682 | -0.52140131 |
| Cdk5rap1 | 0.4183 | 0.22341583 |
| 9130017K11Rik | 0.02211 | 0.73357477 |
| Hddc2 | 0.93604 | 0.0157419 |
| Dnajc25 | 0.11907 | 0.3551993 |
| 4930407I19Rik | 0.00159 | -1.26431393 |
| LOC102636873 | 0.00145 | -1.22376446 |
| Olfr1417 | 0.17689 | -0.63178509 |
| Hexim2 | 0.28818 | 0.31426126 |
| Tacc3 | 0.01566 | 0.58611938 |
| Tacc2 | 0.00192 | -0.92350181 |
| Ass1 | 0.00253 | -0.8154817 |
| Micall2 | 0.00292 | -0.85710502 |
| Gm5591 | 0.0029 | -1.20702967 |
| H2-Eb2 | 0.00144 | -1.26560808 |
| Prl2c5 | 0.02277 | -0.97931301 |
| Xkrx | 0.03126 | 0.4211675 |
| Ttc39a | 0.16875 | 0.59149336 |
| Gm10353 | 0.00144 | -1.60378294 |
| Gm766 | 0.02086 | -0.75458023 |
| Cep95 | 0.00144 | 2.05949616 |
| Mrgpra7 | 0.00145 | -1.2388121 |
| Klrc1 | 0.00144 | -1.28116048 |
| Tas2r144 | 0.00144 | -1.30713006 |
| LOC102639603 | 0.00325 | -1.15314387 |
| Nabp2 | 0.06734 | -0.39430443 |
| Nphs1os | 0.00144 | -1.28116048 |
| Pitpnm2 | 0.01048 | -0.60071429 |
| Klra2 | 0.09834 | 0.78737814 |
| Klra1 | 0.43464 | -0.41749073 |
| Klra6 | 0.00145 | -1.24191372 |
| Klra7 | 0.00145 | -1.2388121 |
| Klra4 | 0.41679 | -0.34681601 |
| Pianp | 0.02531 | -0.80629436 |
| Klra8 | 0.11886 | 1.42259367 |
| Cd300lf | 0.44163 | -0.41814319 |
| Prrg1 | 0.08744 | -0.43432797 |
| Tceal8 | 0.10909 | -0.4509798 |
| Pvt1 | 0.07899 | -2.5134996 |
| Ear6 | 0.36754 | -0.23141538 |
| Tmem14a | 0.65664 | 0.13493612 |
| Rpl24 | 0.00719 | -0.67478836 |
| BC025920 | 0.17102 | 0.30132616 |
| Apcdd1 | 0.08156 | -0.40895318 |
| Emc6 | 0.04917 | -0.69380359 |
| Gprasp2 | 0.35721 | 0.38138844 |
| Defa-rs2 | 0.00189 | -1.92523391 |
| Gm6460 | 0.00162 | -1.65002452 |
| Gm34028 | 0.01078 | -0.82112285 |
| Chil6 | 0.00144 | -1.33442504 |
| Gm6402 | 0.52754 | 0.36343665 |
| Gnai2 | 0.00144 | -1.22740109 |
| Gm853 | 0.00144 | -1.26042915 |
| LOC102635992 | 0.00144 | -1.05723882 |
| Iqce | 0.05102 | -0.51251196 |
| Ap4e1 | 0.00145 | -1.22376446 |
| 3110040M04Rik | 0.01299 | -1.78101924 |
| Ctrc | 0.06691 | -1.2121242 |
| Crispld2 | 0.00144 | -1.32254214 |
| LOC102639543 | 0.1253 | 0.71552691 |
| H2-T24 | 0.16763 | 0.4615531 |
| H2-T24 | 0.17074 | -0.34998651 |
| Tmem192 | 0.0017 | -0.97392993 |
| Mrgprx2 | 0.12895 | -2.50227859 |
| Anp32b | 0.32696 | 0.31937151 |
| Bfsp1 | 0.25057 | -2.00438526 |
| Gm9909 | 0.00361 | 1.26733568 |
| 4930451G09Rik | 0.23846 | -0.22106173 |
| Asgr1 | 0.02996 | -1.42275798 |
| Scml2 | 0.00144 | 2.57225316 |
| Tspan3 | 0.71878 | 0.08730654 |
| Taf4a | 0.00635 | 0.79621656 |
| Sh2d5 | 0.9049 | -0.10846293 |
| Serpinb3a | 0.80194 | 0.4313407 |
| Zscan4b | 0.00144 | -1.30017714 |
| Arhgef7 | 0.02823 | -1.61729536 |
| Zic5 | 0.00144 | -1.25118919 |
| Tmem143 | 0.00383 | -1.08535827 |
| Gm10129 | 0.00145 | -1.02330412 |
| Tstd2 | 0.0704 | -0.37169564 |
| Stxbp6 | 0.00187 | 1.76059721 |
| 4930480K23Rik | 0.00267 | -1.12657613 |
| Slc2a7 | 0.00144 | -1.29746368 |
| Nudt12os | 0.00176 | -1.24266807 |
| Unkl | 0.06551 | -0.96608491 |
| Scnm1 | 0.03477 | -0.45835668 |
| Nrcam | 0.35625 | -0.28071594 |
| Arl9 | 0.04162 | -1.12319191 |
| Olfr631 | 0.00144 | -1.28116048 |
| Hsph1 | 0.13877 | 0.49797815 |
| Pfn1 | 0.04528 | -0.8606043 |
| Lsm7 | 0.00363 | -0.78472219 |
| Kng1 | 0.05644 | -1.83483984 |
| Kng1 | 0.00144 | -1.26451637 |
| Rmdn1 | 0.0023 | 1.21062915 |
| LOC102637409 | 0.0171 | -1.40320086 |
| Kcns2 | 0.01267 | -1.65082388 |
| Fkbp8 | 0.01214 | -0.67639383 |
| Fkbp8 | 0.5241 | -0.36554686 |
| Gm9257 | 0.28923 | -0.17385092 |
| Gm156 | 0.00144 | -1.27165604 |
| Fam107a | 0.0763 | 0.49972054 |
| Magel2 | 0.35724 | -0.25394464 |
| Asxl2 | 0.00316 | 1.21811433 |
| Gimap3 | 0.21955 | 0.28222731 |
| Igsf5 | 0.41662 | -0.36546151 |
| Igsf5 | 0.00453 | -1.00184606 |
| Setd1b | 0.10263 | -0.71131953 |
| Zc2hc1c | 0.11841 | -0.4444692 |
| Phf8 | 0.01412 | -0.75048047 |
| Ngrn | 0.88011 | -0.03795798 |
| Tmem194 | 0.00166 | -1.20466732 |
| Jkamp | 0.06098 | -0.32859746 |
| Rnf7 | 0.88085 | 0.04668045 |
| 1500012K07Rik | 0.00144 | -1.27165604 |
| Pla2g6 | 0.32125 | -0.32311161 |
| Sbpl | 0.16369 | -0.64619982 |
| Pcdh1 | 0.02347 | -1.3510618 |
| E130202H07Rik | 0.00144 | -1.24466058 |
| Disc1 | 0.03104 | -0.83131665 |
| P4ha3 | 0.36916 | -0.98669176 |
| Capza1 | 0.39519 | -0.14474512 |
| Olah | 0.12161 | -0.95191946 |
| Usp17ld | 0.72467 | -0.21380448 |
| Mrgprb4 | 0.00194 | -2.50837157 |
| Coro1a | 0.0084 | -0.93947141 |
| Klk1b5 | 0.46913 | -0.41033209 |
| Klc2 | 0.01113 | -0.81318681 |
| Tas2r138 | 0.00144 | -1.22925777 |
| Smoc2 | 0.73541 | -0.06513386 |
| Pfkfb1 | 0.00359 | 0.83215711 |
| Scgb2b15 | 0.00144 | -1.21868187 |
| Aifm1 | 0.00842 | 0.77170696 |
| Aifm2 | 0.15328 | -0.41477247 |
| Cystm1 | 0.00151 | -1.27463674 |
| Rabgap1l | 0.8623 | 0.05566987 |
| Usp17lc | 0.00229 | -1.21636428 |
| Gm33397 | 0.00144 | -1.25118919 |
| Vps26a | 0.00974 | 0.64288709 |
| 1810053B23Rik | 0.20626 | -1.33480857 |
| 1810053B23Rik | 0.00267 | -0.82926394 |
| Tmem74b | 0.17481 | -0.48439037 |
| Blnk | 0.00596 | -0.66779353 |
| Inpp4a | 0.79868 | 0.05069264 |
| Inpp5a | 0.06565 | -0.4828359 |
| Mllt10 | 0.24305 | -0.26163413 |
| Ddx18 | 0.69005 | 0.08200784 |
| Trim26 | 0.97677 | -0.00480408 |
| Ccdc67 | 0.97379 | 0.01147567 |
| Rap1b | 0.29435 | 0.2225595 |
| Zfp619 | 0.02268 | 1.11383274 |
| Atp5l | 0.00292 | -0.84492296 |
| Gm8702 | 0.00144 | -1.30713006 |
| Ephx1 | 0.00159 | -1.111872 |
| Gm9234 | 0.07638 | -0.33555814 |
| Sec11a | 0.15666 | 0.31797966 |
| Stx16 | 0.02113 | 1.29767989 |
| Gsdmcl2 | 0.00144 | -1.35619208 |
| Chst10 | 0.00251 | -1.42863179 |
| Tmem265 | 0.85274 | 0.04853938 |
| Zfp747 | 0.13746 | 0.28043457 |
| Setd1b | 0.00144 | -1.51898311 |
| Gm3509 | 0.00144 | -1.33632959 |
| Rps6kb2 | 0.07214 | 0.42483138 |
| Srm | 0.13185 | -0.40531934 |
| Lats2 | 0.45644 | 0.15931457 |
| Gm11054 | 0.00144 | -1.28116048 |
| Ap5s1 | 0.00225 | -1.17914854 |
| Zmiz2 | 0.00264 | -0.95525786 |
| Pip5kl1 | 0.0972 | 0.34331852 |
| Msx3 | 0.03419 | -1.40472397 |
| Msx3 | 0.37605 | -0.29872383 |
| Dzip1 | 0.26119 | 0.71551985 |
| Arfip1 | 0.16861 | 0.22810131 |
| Bre | 0.04101 | -0.51635763 |
| Bre | 0.00495 | 1.74208702 |
| Sumo3 | 0.08088 | -0.36876418 |
| Vmn2r-ps54 | 0.00605 | -1.08150631 |
| Rgs12 | 0.24776 | -0.22240325 |
| Rgs12 | 0.00396 | -0.80477609 |
| Kbtbd4 | 0.51356 | 0.14471264 |
| Rpl36 | 0.00144 | -1.37503734 |
| Hmha1 | 0.33038 | -0.22906401 |
| Gm10510 | 0.17489 | -0.98178825 |
| Phf8 | 0.47177 | -0.29333577 |
| Phf8 | 0.64471 | -0.14271413 |
| Wdr88 | 0.08347 | -2.52570889 |
| Wdr89 | 0.15058 | 0.46613801 |
| Gm8232 | 0.00144 | -1.25118919 |
| Rhox12 | 0.00144 | -1.24613586 |
| 6330408A02Rik | 0.10342 | 0.316345 |
| 6330408A02Rik | 0.25102 | -0.46888705 |
| Cab39l | 0.00379 | -0.89065029 |
| Flna | 0.16 | -0.28305167 |
| Smim4 | 0.0097 | -0.80111618 |
| Prps1 | 0.46607 | 0.12076568 |
| Uros | 0.53735 | -0.2308946 |
| Gm36718 | 0.37243 | -0.35830908 |
| Myt1l | 0.01553 | -1.77310405 |
| Copg2 | 0.031 | 0.56128832 |
| Pex1 | 0.01583 | 0.569969 |
| Blvra | 0.30275 | -0.22425391 |
| Grid1 | 0.00144 | -1.27165604 |
| Atp5g1 | 0.00943 | -0.80445844 |
| Klhl3 | 0.35249 | -0.23903771 |
| Rnf180 | 0.00612 | -0.76565949 |
| Tspan18 | 0.31603 | 0.24631756 |
| Zfp128 | 0.31214 | -0.19206499 |
| Igdcc4 | 0.75432 | 0.09649362 |
| Pde6h | 0.00144 | -1.21284878 |
| Gm21158 | 0.00145 | -1.22376446 |
| Il19 | 0.19844 | -0.67891797 |
| Zscan29 | 0.3193 | -0.53818965 |
| Olfr297 | 0.18261 | 0.49924691 |
| Gm5478 | 0.0207 | -0.83784505 |
| Rpl7a | 0.00439 | -0.88510479 |
| Gpr31b | 0.17016 | -0.44363411 |
| Olfr285 | 0.00144 | -1.28116048 |
| Cpz | 0.07068 | -0.79617948 |
| Luc7l3 | 0.00507 | 1.68020823 |
| Pydc3 | 0.0073 | 0.725493 |
| Mpz | 0.04194 | -1.47557218 |
| Cd80 | 0.07892 | 0.5084695 |
| Dnm2 | 0.0042 | -0.74399929 |
| Phf6 | 0.00144 | 1.49369779 |
| 2310015D24Rik | 0.12039 | 0.7764046 |
| Oser1 | 0.61113 | -0.07968678 |
| Rpl31-ps12 | 0.0019 | -0.81559242 |
| Ehmt2 | 0.00144 | -1.00458031 |
| Mmel1 | 0.00778 | -1.30588071 |
| Arid2 | 0.10347 | 0.77005935 |
| Fcf1 | 0.07035 | -0.33375664 |
| Rom1 | 0.00424 | -0.892514 |
| Lrrc19 | 0.22424 | -0.31925666 |
| Syt14 | 0.00144 | -1.29746368 |
| Ppp2r3c | 0.03113 | 0.43277314 |
| Akr1c21 | 0.00144 | -1.31884045 |
| Nrip3 | 0.39419 | -0.44968531 |
| Zfp58 | 0.03698 | 1.45210616 |
| Mvb12b | 0.0115 | -0.97339166 |
| E2f8 | 0.34972 | -0.55581933 |
| Rbm39 | 0.00144 | 1.38257287 |
| Cab39l | 0.66647 | -0.07975692 |
| Ccdc144b | 0.00803 | 1.23220061 |
| Kif7 | 0.27404 | -0.31882112 |
| Olfr1469 | 0.00159 | -1.26431393 |
| Clcn6 | 0.01417 | -0.72320089 |
| Myef2 | 0.0368 | 0.92244988 |
| Gabarap | 0.99541 | 0.00259869 |
| Gm7457 | 0.00144 | -1.29049509 |
| Gm6958 | 0.00144 | -1.29085005 |
| Eci2 | 0.2106 | -0.34035498 |
| Gm15645 | 0.26193 | 0.26438782 |
| Gnb5 | 0.05658 | -0.41627807 |
| Stag2 | 0.00905 | 0.77478312 |
| Slc6a1 | 0.1716 | -0.63737575 |
| Slc50a1 | 0.0077 | -0.59103269 |
| 8030411F24Rik | 0.60982 | -0.16128981 |
| Lrrc30 | 0.32301 | -0.4163114 |
| Slc6a8 | 0.23945 | -0.33638787 |
| Cd209f | 0.29004 | -0.55710121 |
| Lonrf1 | 0.10179 | 0.28537933 |
| Peli1 | 0.00144 | 1.14244185 |
| Tspan18 | 0.0019 | -1.13608632 |
| Tspan18 | 0.00806 | -1.24322971 |
| Ptprz1 | 0.0418 | 0.60285499 |
| LOC100862466 | 0.00731 | 1.1675189 |
| Rhox3e | 0.04959 | -1.15978305 |
| Rab44 | 0.03482 | 0.98102431 |
| Rhox4a | 0.06554 | -0.92412888 |
| Arpc5 | 0.20371 | 0.27617615 |
| Pcnxl3 | 0.00192 | -0.84422738 |
| Rgs11 | 0.02993 | 0.54004249 |
| Terf1 | 0.00144 | 1.91556977 |
| Mab21l2 | 0.84317 | -0.04216734 |
| Phox2a | 0.06368 | -0.84602304 |
| Mthfd1l | 0.94414 | 0.02161713 |
| Slc36a1 | 0.00195 | -1.93198609 |
| Snx29 | 0.27673 | -0.30761945 |
| Cd63 | 0.01635 | -0.5199588 |
| Ubap2 | 0.05307 | -0.4522819 |
| Gm16497 | 0.01515 | -1.05631617 |
| Rhoj | 0.44217 | -1.09228007 |
| Rhoj | 0.01467 | -1.11221018 |
| Slc5a6 | 0.02389 | -0.72312791 |
| 5031439G07Rik | 0.00268 | -1.00639106 |
| Pik3r3 | 0.63558 | 0.11986857 |
| Dnaic1 | 0.06306 | -0.63679089 |
| Ptpru | 0.01163 | -1.09698013 |
| Ptpru | 0.01553 | -0.93990201 |
| Tgfb1i1 | 0.00541 | -0.75055978 |
| Mettl21a | 0.12582 | -0.26124392 |
| Arf1 | 0.11626 | -0.39790792 |
| Nrf1 | 0.13214 | -0.26465078 |
| Irf1 | 0.10535 | -0.42528654 |
| 9930104L06Rik | 0.00151 | 1.03488296 |
| Prf1 | 0.17449 | -0.32497201 |
| Ccdc151 | 0.09597 | -1.03976631 |
| Gm27197 | 0.17532 | -1.00194637 |
| AU041133 | 0.00144 | 2.36183086 |
| Podn | 0.04285 | -0.74845396 |
| Pik3c2g | 0.00145 | -1.48268419 |
| Pip5k1a | 0.28934 | 0.26221235 |
| Pip5k1a | 0.01235 | 1.05366764 |
| Gm14499 | 0.00145 | -1.2388121 |
| Sec24b | 0.00814 | 0.5923364 |
| Rad51ap2 | 0.00144 | -1.29746368 |
| Tchp | 0.58459 | -0.09046545 |
| Nutf2 | 0.69412 | -0.0846866 |
| Cox7c | 0.10463 | -0.55185734 |
| Tenm1 | 0.04919 | -0.79168531 |
| Thoc7 | 0.00801 | 0.67961399 |
| LOC105246531 | 0.023 | -0.91567488 |
| Pkdcc | 0.00566 | -0.8538441 |
| Lrrc51 | 0.02169 | -0.50561404 |
| Cntrl | 0.00725 | 2.30572021 |
| Enpp7 | 0.00145 | -1.22376446 |
| Plin2 | 0.00553 | -0.82032611 |
| Gm15455 | 0.86358 | -0.0289059 |
| Cit | 0.05064 | -0.72326 |
| Gm10631 | 0.92051 | 0.02711303 |
| Josd1 | 0.05905 | -0.55866566 |
| Pram1 | 0.00145 | 1.17139653 |
| Ccdc62 | 0.00144 | 1.81805198 |
| Ccdc42 | 0.04484 | -0.40897057 |
| Ndst4 | 0.00144 | -1.24613586 |
| Gm7207 | 0.01746 | 0.91232186 |
| Aurkc | 0.15925 | 0.38191294 |
| Dsc3 | 0.03613 | -2.02831866 |
| Ppp1r26 | 0.93754 | 0.03423432 |
| Zfp781 | 0.1158 | 0.85463363 |
| Vmn1r7 | 0.00144 | -1.29746368 |
| Ino80e | 0.13872 | -0.32065102 |
| Ptch2 | 0.33628 | -0.20351448 |
| 1500009C09Rik | 0.00144 | -1.28898079 |
| Ccdc186 | 0.31436 | 0.22385287 |
| Spopl | 0.02862 | -1.03550528 |
| Krt85 | 0.16483 | -0.26064236 |
| Dip2a | 0.92799 | -0.04014611 |
| Inpp5b | 0.20132 | 0.33778557 |
| Inpp5b | 0.92705 | 0.0207544 |
| Nudt6 | 0.38475 | -0.22251181 |
| Dhx33 | 0.16025 | -0.25975849 |
| Zfp668 | 0.00566 | -0.81927887 |
| Cyp2e1 | 0.13346 | -0.95959212 |
| Gpbp1 | 0.00233 | 1.26781561 |
| Ovol2 | 0.00326 | -0.87347512 |
| Ovol3 | 0.00144 | -1.26911831 |
| Adrbk1 | 0.0059 | -0.72802154 |
| Gm10318 | 0.14315 | -0.27991494 |
| Gm2696 | 0.01111 | -0.5714489 |
| Defb45 | 0.3358 | -0.44246895 |
| Vmn1r217 | 0.00144 | -1.8553137 |
| Cdk12 | 0.11724 | 0.94526917 |
| Nat10 | 0.1315 | -0.29800014 |
| Defa1 | 0.0105 | -1.35242007 |
| Defa1 | 0.00144 | -1.51500476 |
| Pi16 | 0.00144 | -1.1240078 |
| Clec14a | 0.01783 | -0.68729946 |
| Gm10325 | 0.0588 | -1.04038813 |
| Cps1 | 0.00619 | -1.73774563 |
| Speg | 0.59765 | -0.13177964 |
| Oas1b | 0.15542 | 0.23058447 |
| Trim16 | 0.62812 | -0.08558028 |
| Oas1a | 0.00206 | -0.93915271 |
| H2-M11 | 0.00239 | 1.47175072 |
| Tmem154 | 0.13236 | -0.83666181 |
| Txnrd2 | 0.00144 | -1.1894698 |
| Gm7538 | 0.06862 | -0.70472367 |
| Prim2 | 0.00624 | 0.8078193 |
| 1700122E12Rik | 0.07288 | -0.33200208 |
| Zfand5 | 0.95215 | 0.01347899 |
| Zcchc16 | 0.00144 | -1.30713006 |
| Adck4 | 0.0173 | -0.52499203 |
| Brwd1 | 0.69692 | 0.10846728 |
| Gm2030 | 0.00539 | 1.48519652 |
| Gm2030 | 0.00144 | -1.26560808 |
| Gm8154 | 0.91776 | -0.03829455 |
| Gm3985 | 0.37538 | -0.53860782 |
| Entpd5 | 0.00402 | -0.8047843 |
| Gm10176 | 0.00145 | -1.22376446 |
| Capg | 0.00144 | -1.18409825 |
| 9630028I04Rik | 0.68032 | -0.1441867 |
| Nupl2 | 0.99394 | 0.00209269 |
| Fam43b | 0.04946 | -1.90946118 |
| Ncdn | 0.04294 | -0.6088309 |
| Ncdn | 0.0535 | -0.51620494 |
| C1qtnf1 | 0.00149 | -0.94796745 |
| S100a9 | 0.05199 | 1.45129938 |
| Birc7 | 0.94954 | -0.02487055 |
| Sema3f | 0.00144 | -1.17997682 |
| Sema4f | 0.00144 | -1.27992547 |
| Ifi47 | 0.01438 | -0.77720393 |
| Chchd3 | 0.62148 | 0.10447164 |
| Chchd3 | 0.00762 | 0.8038758 |
| Eif2ak4 | 0.00557 | 0.83917603 |
| Eif2ak4 | 0.0054 | 1.20015281 |
| Eif2ak4 | 0.23006 | 0.41112609 |
| Wsb2 | 0.06627 | -0.46936408 |
| Traf6 | 0.92192 | -0.03262318 |
| Ccser1 | 0.69543 | 0.10556888 |
| Ccser1 | 0.00721 | -0.86402751 |
| Itgb8 | 0.00767 | -0.97701755 |
| Snrpn | 0.07088 | 0.44327161 |
| Cdk4 | 0.03557 | -0.59880603 |
| Cdk4 | 0.01386 | -0.72674951 |
| Spef2 | 0.02009 | -2.28551376 |
| Rab28 | 0.03494 | 0.86041228 |
| Deaf1 | 0.03564 | -0.41397799 |
| Klkb1 | 0.00509 | -1.31836896 |
| Gm20877 | 0.00144 | -1.30713006 |
| Gm9992 | 0.00958 | -1.91567309 |
| Trmt1 | 0.00169 | -0.85516078 |
| Gm20806 | 0.00897 | -1.15797706 |
| Klra23 | 0.18222 | -2.23679902 |
| Klra23 | 0.00243 | -1.12283767 |
| AI314180 | 0.05891 | -0.51240742 |
| Rab2b | 0.02165 | 0.54822435 |
| Htatsf1 | 0.52097 | 0.13115728 |
| Smek2 | 0.00144 | 1.23448119 |
| Hsd17b7 | 0.58194 | 0.28828672 |
| Map9 | 0.03218 | -0.41894057 |
| 1700025C18Rik | 0.00145 | -1.28599367 |
| Nox1 | 0.00144 | -1.90197341 |
| Tfeb | 0.00144 | -1.14814443 |
| Vmn2r109 | 0.00144 | -1.26428512 |
| Osbp2 | 0.24626 | -0.306693 |
| Dbndd1 | 0.01523 | -1.18955748 |
| Nat8l | 0.02093 | -0.69715968 |
| Il6 | 0.00144 | -1.25118919 |
| Prpf8 | 0.09179 | -0.37147445 |
| Cox16 | 0.13969 | -0.3015871 |
| Slc9a8 | 0.00182 | -0.84438925 |
| Plbd1 | 0.02355 | -0.52619308 |
| Smim15 | 0.00161 | 1.50116778 |
| Espn | 0.00726 | -0.9935659 |
| Gm9693 | 0.00816 | -0.81962061 |
| Pla2g4a | 0.03031 | 0.52914951 |
| Slc6a19 | 0.00193 | -1.24713717 |
| Gm4850 | 0.50026 | 0.13684067 |
| Kcnn3 | 0.03403 | -0.79341231 |
| Gm4871 | 0.24747 | -0.4300668 |
| Arpp21 | 0.34817 | -0.50766558 |
| Zbtb32 | 0.2918 | -0.81149594 |
| Gm10778 | 0.00166 | 2.45236679 |
| Usp35 | 0.05457 | -1.08493797 |
| Nhp2l1 | 0.15292 | -0.44312133 |
| Cwc22 | 0.00393 | 1.48054215 |
| Cwc22 | 0.04054 | -0.67692101 |
| Gm5416 | 0.00149 | -1.28183002 |
| Exoc6b | 0.61924 | 0.08645572 |
| Exoc6b | 0.08038 | 1.11781466 |
| Cpd | 0.7077 | 0.12758856 |
| Mylk | 0.89738 | -0.03285978 |
| Ccdc142 | 0.41175 | -0.22873349 |
| Vps9d1 | 0.05898 | -0.6791851 |
| Zim1 | 0.01187 | -1.00553399 |
| Fam57a | 0.68633 | -0.20882557 |
| Mtfr2 | 0.02966 | -0.50422119 |
| Fam53a | 0.00688 | -0.99095241 |
| Fam135a | 0.00144 | 1.57585026 |
| Xpnpep1 | 0.06562 | -0.50327313 |
| LOC630751 | 0.00144 | -1.26560808 |
| Agap2 | 0.00465 | -1.03164225 |
| Pou4f3 | 0.21575 | -0.71712487 |
| Gm4211 | 0.00144 | 1.53784388 |
| Kdm5c | 0.14339 | -0.3622918 |
| Klri1 | 0.00144 | -1.50566664 |
| Nlrx1 | 0.0093 | -0.83604557 |
| Mybl2 | 0.2557 | -0.35249445 |
| Tmprss12 | 0.01064 | -1.23515844 |
| Gyltl1b | 0.16041 | -1.29079296 |
| Bod1 | 0.90948 | 0.0432801 |
| Faxc | 0.00149 | -1.22582153 |
| Tgm7 | 0.00144 | -1.16750011 |
| Gm33101 | 0.00144 | -1.39487581 |
| Polr2a | 0.00872 | -0.62435352 |
| Papd5 | 0.00896 | 0.61936602 |
| Btg1-ps2 | 0.18528 | 0.47669728 |
| Gm10848 | 0.00144 | -1.28898079 |
| Mtcl1 | 0.96209 | -0.01147589 |
| Cdc20 | 0.01394 | -1.13047067 |
| Adgrl1 | 0.1343 | -0.4835538 |
| Rasa3 | 0.04807 | -0.50018415 |
| Zpbp2 | 0.00668 | -1.52511679 |
| Polr2k | 0.56775 | 0.11941782 |
| Psme2b | 0.00257 | -0.83744991 |
| Baz2b | 0.00144 | 1.20768971 |
| LOC102634429 | 0.45632 | -0.51336942 |
| 4930469G21Rik | 0.00144 | -1.29746368 |
| Rbm14 | 0.45116 | -0.27252878 |
| Taar7a | 0.00144 | -1.29085005 |
| Cachd1 | 0.26728 | -0.27630514 |
| Olfr1443 | 0.01448 | 1.17878793 |
| Grm8 | 0.0247 | -0.96581009 |
| Gm16381 | 0.02175 | -0.66181503 |
| Itih1 | 0.10572 | -0.55146767 |
| D030056L22Rik | 0.92835 | -0.01838461 |
| Polr2j | 0.00305 | -0.8488772 |
| Dnajb12 | 0.05308 | -0.44126097 |
| Tmco5 | 0.00144 | -1.30017714 |
| Ppdpf | 0.41234 | -0.23369748 |
| Fgfr3 | 0.14564 | -0.30749501 |
| Ttc13 | 0.76941 | 0.05218894 |
| Ttc17 | 0.0731 | -0.37594575 |
| Vmn1r5 | 0.00145 | -1.25646236 |
| Arhgap42 | 0.00369 | 1.26229559 |
| Rpl29 | 0.00514 | -0.71339244 |
| Abcg2 | 0.13487 | 0.37095079 |
| Nphs2 | 0.01241 | -1.25463654 |
| Nphs1 | 0.00674 | -1.24377301 |
| Srrm1 | 0.00589 | 0.65431629 |
| Rps11-ps1 | 0.00144 | -1.01900218 |
| 5033417F24Rik | 0.18325 | -0.51590904 |
| Gm2366 | 0.03567 | -1.24202576 |
| Ifna14 | 0.0826 | -0.69491156 |
| Dcst2 | 0.00312 | -1.08747569 |
| Wdr38 | 0.00242 | -1.53661011 |
| Amy2a5 | 0.05427 | -2.86644532 |
| Pofut1 | 0.14596 | -0.27264017 |
| 5730422E09Rik | 0.59853 | -0.20170543 |
| Tlr8 | 0.0946 | -0.87892225 |
| 9430078G10Rik | 0.1152 | -0.58040631 |
| Sf3b4 | 0.04258 | -0.51217928 |
| Zbtb2 | 0.04749 | 0.38531971 |
| Olfr770 | 0.00144 | -1.24811894 |
| Vmn2r80 | 0.00144 | -1.28898079 |
| Gm14326 | 0.00172 | 2.29894908 |
| Gm14325 | 0.00145 | 2.69230796 |
| Zfp936 | 0.00144 | 2.82957893 |
| Zfp931 | 0.00152 | 2.35908136 |
| Gm13212 | 0.01753 | 1.45967169 |
| Gm14432 | 0.00653 | 1.95740407 |
| Gm13251 | 0.00414 | 1.97879458 |
| Gm14322 | 0.00144 | 2.75674047 |
| Gm14431 | 0.0015 | 2.63536925 |
| Gm14305 | 0.00628 | 1.92947575 |
| Gm14288 | 0.20385 | 0.20998216 |
| Thumpd1 | 0.04968 | 0.42306015 |
| Zfp605 | 0.84136 | 0.07157962 |
| Rexo4 | 0.69189 | -0.0626529 |
| Pomp | 0.01723 | -0.50606922 |
| Olfr209 | 0.12828 | -1.16710254 |
| Mcpt9 | 0.08528 | -1.06459058 |
| Meis1 | 0.14223 | 0.25032681 |
| Tmem139 | 0.08232 | -1.12029708 |
| Aqp11 | 0.0943 | -0.46700715 |
| Gm5643 | 0.07397 | 0.36191623 |
| Atad3a | 0.35789 | -0.27801814 |
| Nxf2 | 0.01943 | -1.0340189 |
| Btnl2 | 0.87461 | -0.11162309 |
| Btnl6 | 0.00144 | -1.30713006 |
| Btnl5-ps | 0.00144 | -1.29085005 |
| Cobl | 0.15559 | 0.53039815 |
| Top2a | 0.68375 | 0.4820525 |
| Sult4a1 | 0.00144 | -1.45144986 |
| Odc1 | 0.11174 | -0.30995121 |
| Mdc1 | 0.25546 | 0.21467543 |
| Casz1 | 0.69255 | -0.10436053 |
| Rplp1 | 0.00144 | -1.44588476 |
| Btbd19 | 0.04513 | -0.9853158 |
| H2-Q10 | 0.0021 | -1.00916658 |
| Ubald2 | 0.00282 | -1.01099411 |
| Prr7 | 0.04256 | -0.97700775 |
| Zc3hav1 | 0.86591 | 0.06846555 |
| Gm21498 | 0.00144 | -1.31884045 |
| Plekhm2 | 0.00294 | -1.09545747 |
| Arhgap11a | 0.10115 | -1.04556243 |
| Celrr | 0.06106 | -1.03386835 |
| Vmn2r54 | 0.00144 | -1.26911831 |
| Mif | 0.00144 | -1.27200016 |
| Hmgb2 | 0.01689 | 0.65786395 |
| Rab3il1 | 0.0043 | -1.05670244 |
| Wdr5 | 0.16976 | -0.33044575 |
| Ppp2cb | 0.99061 | 0.00197692 |
| Senp2 | 0.99061 | -0.00210077 |
| Etfb | 0.01584 | -0.91481985 |
| Ccdc174 | 0.10481 | 0.46978647 |
| Vmn2r20 | 0.50434 | 0.61798706 |
| Olfr1434 | 0.00144 | -1.28375735 |
| Taf15 | 0.00896 | 1.20117218 |
| 2010001E11Rik | 0.00145 | -1.22518835 |
| Vamp1 | 0.20867 | -0.58305167 |
| Gpr174 | 0.00144 | -1.28375735 |
| Khdrbs1 | 0.03671 | 0.76786817 |
| Gm5141 | 0.13113 | 0.41477931 |
| Strip2 | 0.82027 | 0.06100555 |
| Tti1 | 0.67283 | 0.09543365 |
| Eef1d | 0.00144 | -1.01177088 |
| Dpp6 | 0.00145 | -1.22376446 |
| Itsn1 | 0.31928 | -0.23709069 |
| Itsn1 | 0.82448 | 0.0748132 |
| Itsn1 | 0.02395 | -0.53160138 |
| Itsn1 | 0.08266 | -0.34789452 |
| Shank1 | 0.28201 | -0.29842471 |
| Shank1 | 0.13523 | -0.43744244 |
| Cldn12 | 0.18754 | 0.25985606 |
| Tpd52l2 | 0.07982 | -0.4162668 |
| Sptbn5 | 0.38433 | -0.8093215 |
| Usp31 | 0.45357 | 0.13555538 |
| Prrc2c | 0.00159 | 0.89066007 |
| LOC105245236 | 0.58709 | -0.44701231 |
| Avpr2 | 0.00145 | -1.22376446 |
| Hoxd3 | 0.00144 | -1.29864962 |
| Teddm3 | 0.03875 | -1.18993653 |
| Gm10697 | 0.14338 | -0.56617504 |
| B3gnt6 | 0.06175 | -2.02106348 |
| Gm5916 | 0.00144 | -1.45144986 |
| Ptges3 | 0.38481 | -0.1733052 |
| Golim4 | 0.00144 | 2.05031193 |
| Dhrs7c | 0.00208 | -0.86025057 |
| Ptgir | 0.91361 | 0.02014264 |
| Gm10791 | 0.00877 | 1.59716256 |
| Catsper4 | 0.15906 | 0.97587343 |
| Gar1 | 0.5323 | -0.11681384 |
| Far1 | 0.06731 | 0.81109102 |
| Gapdh | 0.83879 | 0.14687546 |
| Hrc | 0.01371 | -0.55629174 |
| Ythdf1 | 0.10243 | -0.27854425 |
| Ythdc1 | 0.00329 | 0.95942979 |
| Heph | 0.03701 | 0.89413369 |
| Heph | 0.96793 | -0.01811526 |
| Ppil3 | 0.863 | -0.04598736 |
| Zar1 | 0.12434 | -0.87369633 |
| Ccdc154 | 0.35952 | -0.65163737 |
| Tmem184a | 0.00191 | -1.20954856 |
| Tmem184a | 0.10513 | -1.80701208 |
| Ifnl3 | 0.08029 | -0.58689991 |
| Sema5b | 0.94255 | 0.0343264 |
| Sema5b | 0.594 | -0.24562754 |
| Asxl1 | 0.00838 | -0.59335989 |
| Zbp1 | 0.00144 | -1.53081161 |
| Xbp1 | 0.08913 | -0.38702942 |
| Adra2b | 0.01106 | -0.77707997 |
| Ufm1 | 0.1772 | 0.46772772 |
| Snx16 | 0.92168 | -0.02249711 |
| Hemk1 | 0.89696 | -0.0736374 |
| Aimp2 | 0.0027 | -0.95228834 |
| Nutm2 | 0.00149 | -1.22582153 |
| Pltp | 0.00175 | -0.92242238 |
| Sh3bgrl3 | 0.00222 | -0.84841982 |
| Taz | 0.06363 | -0.36178313 |
| 4930562C15Rik | 0.00938 | -1.47676176 |
| Rps24 | 0.13355 | -0.26396492 |
| LOC102641930 | 0.11747 | -0.2619374 |
| Sh2b3 | 0.00295 | -0.7963028 |
| Amelx | 0.0049 | -1.11996532 |
| Gm3264 | 0.9849 | -0.00415839 |
| Tmem132c | 0.01134 | -1.25959526 |
| Itpkb | 0.00334 | -0.82583849 |
| Kdm1a | 0.73046 | -0.06064901 |
| Nudt10 | 0.00957 | 1.20032414 |
| Zfp947 | 0.00274 | 1.41588777 |
| Hmgn2 | 0.30156 | 0.25668381 |
| Rbfox3 | 0.2406 | -1.64608216 |
| Gm2824 | 0.31127 | -0.25831035 |
| Tmem116 | 0.01731 | -0.63703391 |
| Acsbg2 | 0.21337 | -0.69162923 |
| Gm6994 | 0.01412 | -0.59750003 |
| Slx4ip | 0.15511 | 0.32792294 |
| Oaz2 | 0.00174 | -0.92727636 |
| Dcp1b | 0.56432 | -0.10372733 |
| Polr2h | 0.50597 | 0.11996181 |
| Gm29073 | 0.80967 | -0.2585424 |
| Adam1b | 0.11968 | -1.95014121 |
| D5Ertd579e | 0.14546 | -0.36954352 |
| Tbc1d14 | 0.73375 | -0.06863605 |
| D5Ertd579e | 0.44743 | -0.13007114 |
| Efr3a | 0.00219 | 0.87286445 |
| Gm5168 | 0.66323 | 0.23431519 |
| Gm1993 | 0.89002 | -0.03264393 |
| LOC101055939 | 0.25973 | -0.64778492 |
| Ftsj1 | 0.0693 | -0.37622372 |
| Vmn1r170 | 0.00145 | -1.26219879 |
| Cyp2c54 | 0.00144 | -1.28898079 |
| Gm5570 | 0.0024 | -1.57606513 |
| Gm16026 | 0.93563 | -0.02411695 |
| Slc44a5 | 0.00144 | 1.77237127 |
| Olfr873 | 0.0047 | -1.17385111 |
| Ndrg3 | 0.15552 | -0.25497826 |
| Olfr173 | 0.00144 | -1.27165604 |
| Vmn1r104 | 0.03397 | -0.89030722 |
| Olfr761 | 0.00144 | -1.27165604 |
| Olfr161 | 0.00144 | -1.26560808 |
| Speer4c | 0.73317 | 0.0867076 |
| Smtnl1 | 0.08359 | -1.07767989 |
| Ascl4 | 0.00144 | -1.33632959 |
| Gas8 | 0.30873 | -0.17067778 |
| Gm8232 | 0.20239 | -0.61569441 |
| Gm4583 | 0.8134 | 0.11693623 |
| Psip1 | 0.13242 | 0.7039052 |
| Gm10822 | 0.00145 | -1.2388121 |
| F830005K03Rik | 0.85393 | 0.15948458 |
| Pfkfb4 | 0.03368 | -0.4902986 |
| Ddr2 | 0.02619 | 0.50140285 |
| Gyg | 0.12454 | -0.26916894 |
| LOC102636664 | 0.00145 | -1.22518835 |
| Serping1 | 0.00448 | -0.67273431 |
| Tas2r135 | 0.04657 | -0.90571904 |
| Adrb3 | 0.00144 | -1.2841476 |
| Socs2 | 0.09061 | 0.40386833 |
| Shq1 | 0.75885 | -0.19275595 |
| Nudc | 0.8779 | 0.02524779 |
| Rtl1 | 0.00286 | -1.1443198 |
| Kdelr1 | 0.29737 | -0.37897745 |
| Epb4.1l1 | 0.04124 | -1.05003011 |
| Epb4.1l1 | 0.02849 | -0.98862697 |
| Dync1i2 | 0.00916 | 0.77752219 |
| Olfr1115 | 0.00144 | -1.30713006 |
| Olfr1115 | 0.07306 | -0.45522067 |
| Igfbp3 | 0.00289 | -1.30782904 |
| Ubtf | 0.03711 | -0.46515336 |
| Hdac6 | 0.00374 | -0.72306574 |
| Bloc1s1 | 0.00232 | -0.90183251 |
| Il5ra | 0.07233 | -1.13674644 |
| Gm41468 | 0.67745 | -0.15152183 |
| Gys2 | 0.00144 | -1.28898079 |
| Pmpca | 0.14755 | -0.40780814 |
| Olfr727 | 0.00144 | -1.28116048 |
| Entpd4 | 0.05548 | -0.4881095 |
| Olfr764-ps1 | 0.00144 | -1.28898079 |
| Gpc4 | 0.02143 | -0.72262793 |
| Scg2 | 0.00145 | -1.26219879 |
| Vdac2 | 0.69822 | -0.08773306 |
| Vdac1 | 0.08347 | -0.47101652 |
| Hmgb3 | 0.00672 | 0.8305587 |
| Gm3414 | 0.02061 | 0.87068376 |
| Gm11567 | 0.00627 | -1.01128782 |
| Simc1 | 0.55894 | 0.15094857 |
| Gm9884 | 0.00144 | -1.30935626 |
| AY358078 | 0.00144 | -1.28116048 |
| 1700049E17Rik1 | 0.00144 | -1.31518407 |
| Gm5622 | 0.00144 | -1.30017714 |
| Gmpr | 0.95979 | 0.03537734 |
| Rbbp8nl | 0.76277 | 0.22773207 |
| Col4a3bp | 0.02254 | -0.73986311 |
| 1700025D23Rik | 0.00144 | -1.29085005 |
| Rergl | 0.92073 | -0.04899816 |
| Slx | 0.55198 | 0.50596175 |
| 2610203C22Rik | 0.69357 | 0.14727253 |
| Chchd2 | 0.00396 | -0.94660615 |
| Psg20 | 0.00749 | -1.05513651 |
| Mboat7 | 0.34061 | -0.42218106 |
| Tars2 | 0.01537 | -0.7503512 |
| Tars2 | 0.65977 | -0.22452813 |
| Dnaja1 | 0.39445 | 0.30740114 |
| Ptprj | 0.29567 | -0.24204289 |
| Ptprg | 0.00568 | 1.39117349 |
| Ptprf | 0.02977 | -0.43203039 |
| Ptpra | 0.99243 | -0.00179568 |
| Armc10 | 0.2275 | 0.22951634 |
| Gucy1a3 | 0.14296 | 0.59544835 |
| Msi2 | 0.01753 | 0.52074707 |
| Msi2 | 0.3548 | 0.48059537 |
| Epm2a | 0.05026 | -0.73274255 |
| Dydc2 | 0.00144 | -1.29417808 |
| Ptpru | 0.10232 | -0.29062983 |
| Iqsec2 | 0.0147 | -0.52276234 |
| Nwd1 | 0.01139 | -0.86469094 |
| Scgb1b19 | 0.05696 | -2.22630219 |
| LOC102641638 | 0.00987 | -1.34544111 |
| Acadvl | 0.00149 | -1.000909 |
| Gm5155 | 0.00144 | -1.25927895 |
| Slc34a1 | 0.10727 | -1.02773535 |
| Dcpp3 | 0.4739 | -0.32982311 |
| Speg | 0.1773 | -0.30833539 |
| Slc22a27 | 0.00151 | -1.17792995 |
| Nrg2 | 0.40771 | -0.3512308 |
| Gm5431 | 0.07269 | 0.94317037 |
| Slc25a46 | 0.05486 | 0.59171331 |
| Fam219a | 0.03223 | -0.52693421 |
| Pdlim4 | 0.02834 | -0.72062249 |
| Mtcp1 | 0.00807 | 1.11272567 |
| Mtcp1 | 0.12468 | 0.91687784 |
| Zfp956 | 0.83617 | -0.04260723 |
| Alkbh3 | 0.48232 | 0.1827666 |
| Hmmr | 0.00371 | 1.07607258 |
| Samt2 | 0.10426 | -2.85002446 |
| Rad51ap1 | 0.0262 | 0.8287642 |
| Gm10276 | 0.00144 | -1.27730451 |
| A830010M20Rik | 0.01519 | -1.01056962 |
| Ppp1r16a | 0.00553 | -0.87845732 |
| Blvrb | 0.00343 | -0.69775 |
| Lilra5 | 0.30674 | -0.26229017 |
| Mrgprx1 | 0.00208 | -1.15498993 |
| Atraid | 0.01662 | -0.65601532 |
| E4f1 | 0.08422 | -0.32778378 |
| Obfc1 | 0.15177 | -0.26097709 |
| Olfr803 | 0.00144 | -1.29746368 |
| Zscan21 | 0.04446 | 0.36062791 |
| Olfr303 | 0.00144 | -1.29049509 |
| Fbxo34 | 0.22797 | 0.23388974 |
| Fbxo24 | 0.65062 | -0.57897085 |
| Adam34 | 0.00144 | -1.25118919 |
| Npvf | 0.01416 | -1.11139486 |
| Mettl5 | 0.04691 | -0.92781888 |
| Zfp407 | 0.14499 | -0.34376317 |
| Chrna10 | 0.35331 | -0.6283312 |
| 9930021J03Rik | 0.0586 | -0.86282997 |
| Stat3 | 0.78116 | -0.05273338 |
| Nos1ap | 0.78282 | 0.09489088 |
| Mlc1 | 0.26959 | -0.77857796 |
| Rpl13 | 0.00144 | -1.11042717 |
| Tom1l2 | 0.01837 | -0.63613022 |
| D10Jhu81e | 0.08636 | -0.61542977 |
| Gm10910 | 0.0288 | -0.54520056 |
| Lmf1 | 0.01871 | -0.78058919 |
| Tmf1 | 0.05126 | 0.64022431 |
| Gm3643 | 0.006 | 0.96251695 |
| Dclk2 | 0.04814 | -0.89185502 |
| Cnga3 | 0.01356 | -0.96209206 |
| Lman1l | 0.21425 | -0.46635727 |
| Casp9 | 0.0483 | -0.52434664 |
| Osbpl11 | 0.21281 | 0.23434343 |
| Casp1 | 0.11289 | 0.61437188 |
| Msrb1 | 0.01731 | -0.83016135 |
| Psmd4 | 0.00276 | -0.76226765 |
| Mylk3 | 0.28011 | 0.3383656 |
| Rpl6 | 0.01861 | -0.48500155 |
| Dclre1b | 0.40813 | 0.43612206 |
| Gm20877 | 0.02331 | -2.02536766 |
| Gm29142 | 0.00144 | -1.30713006 |
| Ssty1 | 0.00929 | -1.0433256 |
| Tcp10b | 0.26438 | 0.42569944 |
| 4932412D23Rik | 0.08464 | -0.38896198 |
| Zranb3 | 0.07023 | 0.51264532 |
| Ptp4a1 | 0.10926 | 0.40091176 |
| Ppp3r1 | 0.67487 | -0.07878337 |
| Gm17782 | 0.68973 | 0.11353703 |
| Slc16a7 | 0.09012 | -0.33755547 |
| 1700101G07Rik | 0.06436 | -1.03772473 |
| Gm5820 | 0.03287 | 0.73741525 |
| Ddx5 | 0.00214 | 1.44941898 |
| Comt | 0.00144 | -1.11523589 |
| Grm6 | 0.80368 | -0.27165604 |
| Oosp3 | 0.00144 | -1.25927895 |
| Ube2j1 | 0.03782 | -0.51167083 |
| Ube2j1 | 0.55269 | 0.18357276 |
| Olfr1448 | 0.00144 | -1.29085005 |
| Snrnp70 | 0.67514 | 0.07246885 |
| Adgrb1 | 0.99267 | 0.00541298 |
| Tbc1d22a | 0.00144 | -1.12584903 |
| Ube2v1 | 0.00222 | -0.94790248 |
| Masp2 | 0.22642 | 0.51986692 |
| Stat5a | 0.00454 | -0.89143721 |
| Gas2l1 | 0.013 | -1.15916515 |
| Rpl15 | 0.08593 | -0.52177335 |
| R3hdm1 | 0.01702 | 0.54052648 |
| Htr4 | 0.00144 | -1.28116048 |
| Vmn2r48 | 0.00532 | -0.97805118 |
| Add1 | 0.0374 | -0.50262953 |
| Ergic3 | 0.01553 | -0.64333801 |
| Elavl4 | 0.39612 | 0.3206617 |
| Rplp2 | 0.00144 | -1.09612971 |
| Fthl17 | 0.00144 | -1.31495282 |
| Tbx22 | 0.00145 | -1.22376446 |
| Fnta | 0.31392 | -0.17074733 |
| LOC102637854 | 0.39543 | -0.64329428 |
| Scpep1 | 0.29133 | -0.16402167 |
| Gm7849 | 0.00461 | -1.08200933 |
| Ccdc177 | 0.00144 | -1.29085005 |
| Dolpp1 | 0.02277 | -0.65732565 |
| Aqp5 | 0.00144 | -1.27165604 |
| Mks1 | 0.18974 | -0.24125158 |
| Mylk3 | 0.31295 | 0.21806686 |
| Nxt2 | 0.01046 | 0.74061268 |
| Cxcl5 | 0.03386 | -0.9299365 |
| Cd200r3 | 0.00144 | -1.26934956 |
| Tcte1 | 0.06932 | -0.35630307 |
| Fam149b | 0.50474 | 0.1366745 |
| Fam129b | 0.00254 | -0.90976168 |
| Gm3561 | 0.9494 | -0.02107537 |
| Eml2 | 0.10085 | -0.37525432 |
| Lin54 | 0.00221 | 1.21667176 |
| Ddb1 | 0.00144 | -1.01381367 |
| Smarce1 | 0.35238 | -0.19733964 |
| Vmn2r57 | 0.09958 | -0.72744423 |
| Thrap3 | 0.06117 | -0.66397116 |
| Tm6sf1 | 0.00413 | 0.84687433 |
| Gcm1 | 0.97407 | 0.01551654 |
| Chml | 0.01254 | 0.64022585 |
| Sqrdl | 0.35381 | -0.22442494 |
| Gm3952 | 0.02173 | -0.81570629 |
| Bag6 | 0.02588 | -0.76899685 |
| C2cd2l | 0.00238 | -0.91399461 |
| Vmn2r120 | 0.05401 | -2.51464038 |
| Slco2a1 | 0.00151 | -0.95480641 |
| Slco3a1 | 0.12671 | -0.44214571 |
| Slco1a1 | 0.01337 | -1.27891681 |
| Slco4a1 | 0.05643 | -0.86585418 |
| Olfr70 | 0.00145 | -1.22518835 |
| Olfr50 | 0.0022 | -1.34813137 |
| Olfr10 | 0.03732 | -2.45208501 |
| Spdyb | 0.00144 | -1.30017714 |
| Mynn | 0.00144 | 1.25855074 |
| Tmem175 | 0.15129 | -0.36286305 |
| Cklf | 0.35148 | -0.24453418 |
| Cklf | 0.03414 | 1.44129809 |
| Fndc1 | 0.16896 | -1.17476483 |
| Fndc1 | 0.00144 | -1.29548728 |
| 2010016I18Rik | 0.02606 | -0.88233197 |
| Tmem164 | 0.91185 | -0.03364866 |
| Chrna3 | 0.00144 | -1.52681567 |
| Gm6639 | 0.62892 | -0.19707032 |
| Nenf | 0.0226 | -0.76142404 |
| Elf1 | 0.16747 | 0.74958161 |
| Rnf181 | 0.82985 | 0.08020775 |
| Tdrd9 | 0.0185 | -0.79848571 |
| Vmn1r2 | 0.00145 | -1.24191372 |
| Rps12 | 0.00144 | -1.07966206 |
| Prkd3 | 0.14662 | 0.3219669 |
| Tnxb | 0.00144 | -0.992306 |
| Romo1 | 0.01105 | -0.89484657 |
| Chfr | 0.91686 | 0.01814249 |
| Gm16080 | 0.00145 | -1.2388121 |
| Olfr1508 | 0.5575 | -0.32450926 |
| 5430419D17Rik | 0.70641 | 0.12850073 |
| Olfr994 | 0.00144 | -1.46134994 |
| Olfr694 | 0.00144 | -1.31884045 |
| Olfr794 | 0.00462 | -0.84409625 |
| Olfr394 | 0.00144 | -1.48661212 |
| Olfr194 | 0.00144 | -1.43181863 |
| Vmn1r77 | 0.00144 | -1.20579466 |
| Rps17 | 0.00357 | -0.76999119 |
| Hmgn1 | 0.27403 | 0.21603947 |
| Oas1d | 0.1738 | -0.6741226 |
| Tsc22d3 | 0.05377 | -0.36225863 |
| Tm9sf1 | 0.02086 | -0.70961067 |
| Eif6 | 0.03211 | -0.64018792 |
| Olfr1265 | 0.00144 | -1.28898079 |
| Vmn1r151 | 0.00144 | -1.27730451 |
| Ms4a4b | 0.12351 | 0.42520292 |
| Mkks | 0.12133 | 0.32614961 |
| Gm36283 | 0.01431 | -1.13142631 |
| Cdkal1 | 0.45195 | -0.71598709 |
| Eml4 | 0.21627 | 0.26482037 |
| Bcl11a | 0.19526 | -0.2210034 |
| Klrg1 | 0.00144 | -1.29049509 |
| Acss2 | 0.66338 | 0.14365052 |
| Slc17a5 | 0.19977 | 0.38746955 |
| Vmn2r112 | 0.00145 | -1.21051401 |
| Sestd1 | 0.00239 | 0.95940168 |
| Fbxl19 | 0.00272 | -0.80483281 |
| Gm5953 | 0.05615 | -0.95464615 |
| Psors1c2 | 0.06758 | 0.84716129 |
| Gstz1 | 0.07778 | -0.30780256 |
| Olfr1205 | 0.00144 | -1.27699683 |
| Cacna2d1 | 0.01716 | 0.68803869 |
| Rbpjl | 0.04043 | -0.60844422 |
| Map3k14 | 0.00144 | -1.2517234 |
| 4930558N11Rik | 0.01278 | 1.12224968 |
| Myh11 | 0.5184 | 0.36156896 |
| Naa20 | 0.23146 | 0.31276868 |
| Gnl2 | 0.00145 | -1.24191372 |
| D830024N08Rik | 0.58107 | 0.1847999 |
| Tpi1 | 0.95697 | -0.03720045 |
| Gm8541 | 0.00144 | -1.20555712 |
| Gm2347 | 0.87852 | -0.11360562 |
| Slc7a3 | 0.17347 | 0.65089988 |
| Cenpu | 0.63392 | 0.31592335 |
| Tex24 | 0.0031 | -1.36138936 |
| Lrrc47 | 0.02838 | -0.52110149 |
| Dennd4c | 0.96964 | 0.07489871 |
| Rnf103 | 0.00284 | 0.78804646 |
| Vmn2r106 | 0.00144 | -1.31884045 |
| Aspm | 0.00145 | 1.87670862 |
| Cdkn2b | 0.14083 | -0.51698587 |
| Proser3 | 0.36093 | 0.26130586 |
| Esd | 0.01356 | -0.58894044 |
| Tox | 0.28314 | 0.56391832 |
| Trp73 | 0.23008 | -0.55326591 |
| Cyth2 | 0.56697 | 0.1041415 |
| Mrc1 | 0.01562 | 1.0846581 |
| Gm13089 | 0.0109 | -1.21301306 |
| Prc1 | 0.57683 | 0.26740126 |
| Eef1g | 0.06861 | -0.60696356 |
| Tyw3 | 0.00421 | 1.80186172 |
| Ssh1 | 0.06122 | -0.77702456 |
| Gng7 | 0.59231 | 0.11730473 |
| Gm10406 | 0.00144 | 2.93646807 |
| LOC102637882 | 0.00144 | -1.29085005 |
| Atg2b | 0.31158 | 0.26919586 |
| Zfpm1 | 0.00538 | -0.97496808 |
| Plrg1 | 0.30211 | 0.18670367 |
| Glipr1l3 | 0.99826 | 0.00145948 |
| Gm5622 | 0.00144 | -1.28116048 |
| Gm5938 | 0.27892 | -0.51126328 |
| Rai1 | 0.59964 | -0.09196363 |
| Vmn1r122 | 0.00144 | -1.29085005 |
| Map4k4 | 0.73984 | -0.0515986 |
| Chpf2 | 0.00225 | -0.85169562 |
| Chpf2 | 0.19939 | 0.3421555 |
| Skint4 | 0.10838 | -2.87224858 |
| Erbb2ip | 0.00144 | 1.77074862 |
| Skint8 | 0.00144 | -1.28898079 |
| Gm35091 | 0.02264 | 0.82492653 |
| Rps6ka5 | 0.25016 | 0.46522515 |
| Psenen | 0.00234 | -0.99701747 |
| Fam213a | 0.00341 | 0.88372852 |
| Gria4 | 0.05134 | -0.62805056 |
| Epb4.1l2 | 0.07101 | -0.53742047 |
| Ptprd | 0.1294 | -3.19641305 |
| Ptprd | 0.88298 | -0.05639073 |
| Tmem161b | 0.28341 | -0.18885715 |
| Fam186a | 0.00744 | -1.03871255 |
| 1700030F18Rik | 0.01764 | -1.65843555 |
| Nav3 | 0.03022 | 1.23998557 |
| Trmt112 | 0.00456 | -0.81156668 |
| LOC101055915 | 0.00195 | -0.81339205 |
| Ccnc | 0.36678 | 0.48965342 |
| Gabrr2 | 0.0184 | -0.50661777 |
| Pgm2 | 0.01151 | -0.79257987 |
| Pigt | 0.01983 | -0.56655481 |
| Ep400 | 0.48599 | -0.13566601 |
| Fam196a | 0.02446 | 1.24845789 |
| Fam196a | 0.5184 | -0.1618647 |
| Uba1y | 0.00144 | -1.29085005 |
| Nbeal1 | 0.80029 | -0.08517151 |
| Nbeal1 | 0.95681 | 0.01880796 |
| LOC100048884 | 0.02036 | -2.89475026 |
| Tbl1xr1 | 0.39111 | 0.21842807 |
| Defa-ps12 | 0.00145 | -1.26219879 |
| Olfr110 | 0.0303 | -1.32679072 |
| Prl3d1 | 0.01019 | 0.55428796 |
| Prl3a1 | 0.02045 | -1.10014643 |
| Eny2 | 0.02177 | 0.48894488 |
| 1700045I11Rik | 0.05053 | -0.91375781 |
| Lppr5 | 0.11054 | -1.38300641 |
| Rnu1b6 | 0.01466 | -0.81129756 |
| Olfr1383 | 0.01445 | 1.01915009 |
| Gm10652 | 0.00144 | -1.26560808 |
| Olfr1257 | 0.25043 | -0.71819235 |
| Gnb2 | 0.00144 | -1.1284624 |
| Fam214b | 0.0632 | -0.34643655 |
| Ywhaz | 0.01351 | -0.75744801 |
| Fuom | 0.01285 | -0.99392369 |
| Fuom | 0.18326 | -0.43552655 |
| Fuom | 0.02776 | -0.78542937 |
| Gm3448 | 0.05048 | -0.45644185 |
| Ttll9 | 0.00145 | -1.26219879 |
| Adgrf4 | 0.00187 | 1.31764206 |
| Lsm14b | 0.73872 | -0.10734302 |
| Gm4559 | 0.82473 | 0.14710974 |
| Kifc5b | 0.04323 | -0.83666181 |
| Gm10372 | 0.69514 | -0.30582734 |
| Enoph1 | 0.12068 | -0.26706834 |
| Gne | 0.00521 | -0.79198549 |
| Brms1l | 0.03428 | 0.49871453 |
| Dll1 | 0.05416 | -0.69864147 |
| Gm9914 | 0.01462 | -1.43531104 |
| Mrgprd | 0.00215 | -1.16778291 |
| Tulp4 | 0.11167 | 0.2927515 |
| Gm8075 | 0.86695 | 0.04837584 |
| Aldh3b3 | 0.00144 | -1.71553316 |
| Aldh3b3 | 0.00144 | -1.29746368 |
| Olfr619 | 0.00144 | -1.29878664 |
| Olfr319 | 0.18099 | -0.56856261 |
| Podxl2 | 0.17345 | -0.61003209 |
| 3110070M22Rik | 0.09099 | -0.84276776 |
| Cacna1h | 0.00602 | -1.24833909 |
| 4921524L21Rik | 0.00144 | -1.20555712 |
| Gm19402 | 0.39455 | -0.38605762 |
| Gm19668 | 0.00303 | 0.7591862 |
| Mcam | 0.0247 | -0.55001897 |
| G6b | 0.00589 | -1.4490675 |
| Cdh26 | 0.36114 | 0.17619931 |
| Gtf2ird1 | 0.37147 | -0.26474368 |
| Galnt13 | 0.02349 | -1.21742281 |
| Obp2b | 0.00144 | -1.33944222 |
| Pde4d | 0.00144 | 2.636272 |
| G6bos | 0.00144 | -1.86756596 |
| Tcp11 | 0.0209 | -0.75684658 |
| Pnpo | 0.09121 | -0.81498259 |
| Senp3 | 0.29548 | -0.21704915 |
| Tbc1d32 | 0.06651 | -0.7070618 |
| Tbc1d32 | 0.00144 | 3.15791295 |
| Gm4956 | 0.94079 | -0.0220836 |
| Ltv1 | 0.00144 | 1.15133268 |
| Tagln2 | 0.00294 | -0.96503429 |
| Cdkn1a | 0.04326 | -0.68019914 |
| Tmem41b | 0.53245 | 0.11939873 |
| Gm5934 | 0.00144 | -1.31884045 |
| Gm3596 | 0.00144 | -1.32343611 |
| LOC102638521 | 0.05505 | -0.39414348 |
| Fut1 | 0.00144 | -1.30017714 |
| Tubgcp4 | 0.15073 | -0.79232315 |
| Plekhg5 | 0.10868 | -0.6780454 |
| Ccnb1ip1 | 0.00385 | -1.13547254 |
| Def8 | 0.19496 | -0.51926958 |
| LOC102641574 | 0.00208 | -0.87023717 |
| Gdpgp1 | 0.70832 | -0.10549481 |
| Klhdc4 | 0.00272 | -0.76446745 |
| Sccpdh | 0.6395 | 0.076827 |
| Prr14 | 0.02666 | -0.51477648 |
| Pcnx | 0.05564 | -0.44887502 |
| Fip1l1 | 0.01258 | -0.6343415 |
| Slc35f4 | 0.00145 | -1.25646236 |
| Il3 | 0.09146 | -2.66814864 |
| Actg1 | 0.37127 | -0.19757329 |
| Speer1 | 0.0341 | -0.80275299 |
| Xpnpep2 | 0.00636 | -1.28765494 |
| 9130015G15Rik | 0.00329 | -1.10120227 |
| Pgr | 0.00144 | -1.30017714 |
| Nkx2-2 | 0.00144 | -1.20555712 |
| Fastkd1 | 0.04267 | 0.71547742 |
| Dnase1 | 0.80873 | -0.14725848 |
| Ranbp9 | 0.00234 | 1.06245943 |
| Dock6 | 0.0116 | -1.65280697 |
| Dock6 | 0.04779 | -0.39931171 |
| Trhr2 | 0.042 | -1.90897064 |
| Ttyh2 | 0.01334 | -0.58590621 |
| Psmd9 | 0.13035 | -0.46931876 |
| Tcl1b3 | 0.00145 | -1.20627532 |
| Gm7562 | 0.52912 | -0.47366014 |
| Mcoln1 | 0.67577 | 0.52125609 |
| Zbtb37 | 0.0065 | -1.08084356 |
| Lrp1 | 0.90284 | -0.05092009 |
| Abcc3 | 0.03788 | -0.71730112 |
| Gm4230 | 0.00145 | -1.361652 |
| Cxx1a | 0.21162 | -0.32301565 |
| Tomm6 | 0.01514 | -0.58109191 |
| Gatad1 | 0.47841 | -0.20843985 |
| Kcnk10 | 0.00144 | -1.31495282 |
| Shc3 | 0.58812 | -0.1257862 |
| BC028528 | 0.02097 | -0.58819603 |
| Dlg3 | 0.06177 | 0.61676769 |
| Gm9839 | 0.38922 | -0.36458795 |
| Gm12271 | 0.84555 | -0.03040116 |
| Susd3 | 0.90948 | 0.06813823 |
| Susd3 | 0.02975 | -1.06045047 |
| Nrg1 | 0.12184 | -0.53539932 |
| Atg16l2 | 0.04247 | -0.47610283 |
| Reps2 | 0.11656 | 0.59650779 |
| Kcnk15 | 0.00144 | -1.25118919 |
| Xylt1 | 0.02928 | -0.57572737 |
| Man1c1 | 0.30618 | 0.26512496 |
| Slc35f2 | 0.04656 | -1.30987689 |
| LOC102637278 | 0.13423 | -0.77803917 |
| Tmem241 | 0.02169 | 0.49642191 |
| Kif14 | 0.10785 | -2.92840984 |
| Krtap5-3 | 0.00281 | -0.7821883 |
| Krtap5-2 | 0.94771 | -0.01223684 |
| Lmcd1 | 0.27093 | -0.34544082 |
| Atp2b4 | 0.38677 | -0.2512727 |
| C2cd3 | 0.00587 | -0.86555786 |
| Necab1 | 0.00145 | -1.2388121 |
| Olfr1234 | 0.98835 | -0.00665755 |
| Gimap1os | 0.91888 | -0.04953374 |
| Olfr658 | 0.01901 | -0.8944554 |
| Pogz | 0.32545 | 0.40974319 |
| Hs3st4 | 0.00592 | -1.07650415 |
| Fam195b | 0.00144 | -1.25126567 |
| Robo1 | 0.00144 | -1.29746368 |
| Pard3 | 0.15024 | -0.35425515 |
| Pard3 | 0.00198 | -0.80651336 |
| Pard3 | 0.03196 | -0.80254746 |
| Cirbp | 0.10296 | -0.4088463 |
| Gm31649 | 0.00144 | -1.27165604 |
| Wnt5b | 0.15344 | -0.53629776 |
| LOC102636514 | 0.20184 | 0.44635643 |
| Olfr20 | 0.06819 | -2.25696303 |
| Zc3h18 | 0.02539 | -0.44797785 |
| Wdr41 | 0.00158 | -1.1474389 |
| Hcst | 0.00144 | -1.25591252 |
| 1700024P16Rik | 0.00144 | -1.28116048 |
| Gmcl1 | 0.01634 | 1.15951758 |
| Zbtb1 | 0.04 | 0.55918977 |
| Polr2i | 0.04879 | -0.6995762 |
| Polr2i | 0.01084 | -0.59724554 |
| Fgl1 | 0.63695 | 0.08925387 |
| Bloc1s2 | 0.15598 | -0.25788445 |
| Snph | 0.00145 | -1.25646236 |
| Retn | 0.40681 | -0.82991942 |
| Fam220a | 0.31879 | -0.27938016 |
| Klk1b16 | 0.9629 | 0.00971545 |
| Pappa2 | 0.00144 | -1.41729881 |
| Sgpl1 | 0.60489 | 0.09552734 |
| Ip6k3 | 0.09413 | -0.56903089 |
| Abhd6 | 0.64482 | 0.07957454 |
| Gm5434 | 0.37906 | -0.6356302 |
| Ube2f | 0.47171 | 0.11945845 |
| Parp4 | 0.02864 | 0.49964758 |
| Smarcc1 | 0.99841 | 0.00069708 |
| Smarcc1 | 0.15722 | -0.63648695 |
| Stxbp5 | 0.12684 | 0.57376261 |
| Cpne2 | 0.00613 | -0.68520471 |
| Gm1305 | 0.14322 | 0.49448216 |
| Adra2c | 0.04609 | -0.95155295 |
| Usp44 | 0.00144 | -1.27165604 |
| Gm9948 | 0.19832 | -0.71642827 |
| Lrrk2 | 0.00144 | 2.01740401 |
| Shank2 | 0.0057 | -1.19115562 |
| Depdc5 | 0.01939 | -0.83619474 |
| Depdc5 | 0.06411 | -0.40055817 |
| Vmn2r99 | 0.00144 | -1.29051721 |
| Cdadc1 | 0.00144 | 1.12337276 |
| Rhox3h | 0.44443 | 0.26774958 |
| Rhox2h | 0.03237 | -0.73262014 |
| BC042761 | 0.00145 | -1.26219879 |
| Trim43c | 0.23166 | -0.78329405 |
| Trim43c | 0.11105 | 0.26725914 |
| Adgrf5 | 0.00258 | 1.791905 |
| Efcab14 | 0.11895 | 0.28057948 |
| Gapdh | 0.19789 | -0.46897948 |
| Pdcl2 | 0.00144 | -1.24811894 |
| Lincpint | 0.52544 | -0.25257963 |
| Lincpint | 0.40241 | -0.2828361 |
| Naip2 | 0.05176 | 0.7624817 |
| Gfral | 0.28825 | -0.66947452 |
| Gabre | 0.00144 | -1.29085005 |
| Pde4dip | 0.04487 | -0.41856577 |
| Pde4dip | 0.09581 | 0.5079972 |
| Slc44a2 | 0.02329 | -0.49954955 |
| 1600029O15Rik | 0.64537 | -0.08304168 |
| Cfap61 | 0.2244 | 0.37656284 |
| Cfap61 | 0.42063 | -0.16377451 |
| Nup62 | 0.02068 | -0.84332841 |
| Gfra3 | 0.00151 | -1.4939917 |
| Pdcd4 | 0.1723 | 0.41820348 |
| Litaf | 0.17853 | -0.26476054 |
| Rsph3b | 0.31082 | -0.26562886 |
| Desi1 | 0.25767 | -0.2656155 |
| Desi1 | 0.57923 | -0.20696993 |
| Neurl4 | 0.62199 | -0.18854884 |
| Slc8b1 | 0.14169 | -1.15590828 |
| Ftl1 | 0.00492 | -0.93590416 |
| Tmem74 | 0.00144 | -1.29051721 |
| Crybb3 | 0.06079 | 0.43303405 |
| Thap4 | 0.47327 | -0.24342547 |
| Scel | 0.00145 | -1.2388121 |
| Srsf7 | 0.18329 | 0.2390136 |
| Birc5 | 0.18578 | -0.64156715 |
| Sdhc | 0.05588 | -0.58295311 |
| Pcp4l1 | 0.28387 | -0.28603864 |
| Aldh1l1 | 0.39764 | -0.14954969 |
| Insrr | 0.74479 | -0.19176447 |
| 1700012B07Rik | 0.00144 | -1.28116048 |
| Rgag4 | 0.04186 | -0.42295001 |
| Gm6377 | 0.12874 | -0.79809298 |
| Vmn1r45 | 0.00144 | -1.20579466 |
| Mapk6 | 0.04918 | 0.48335876 |
| Tmed1 | 0.00201 | -1.21672065 |
| AF529169 | 0.00144 | -1.31495282 |
| Tmed6 | 0.00149 | -1.2703832 |
| Tmed6 | 0.17619 | -3.44193713 |
| Cd300ld | 0.0878 | 0.43136029 |
| Lsr | 0.00144 | -1.04497501 |
| Cflar | 0.00488 | 0.65882758 |
| Dux | 0.00238 | -1.95751671 |
| Abhd14a | 0.00606 | -0.85285253 |
| Amy1 | 0.00406 | 1.55606903 |
| Hhla1 | 0.71664 | -0.10878005 |
| Prss52 | 0.00144 | -1.29085005 |
| Ampd3 | 0.37292 | -0.18669594 |
| Sh3d21 | 0.13613 | -0.23896202 |
| C86187 | 0.01828 | -1.73947451 |
| D7Ertd443e | 0.02195 | -0.60344162 |
| 1700026J12Rik | 0.00144 | -1.20579466 |
| Gm5897 | 0.80282 | -0.26380446 |
| Crat | 0.13825 | -0.615222 |
| LOC102643251 | 0.45219 | 0.18878284 |
| Flot2 | 0.11397 | -0.40009971 |
| 4930444P10Rik | 0.00226 | 1.18839975 |
| Pstpip2 | 0.00159 | -1.00472149 |
| Rps29 | 0.00144 | -1.17458136 |
| Pxmp4 | 0.00319 | -1.12640025 |
| Adam19 | 0.0569 | -0.46541981 |
| Adam39 | 0.40165 | -0.25088707 |
| Uhrf1bp1 | 0.46241 | -0.16477282 |
| Plch1 | 0.00353 | -1.06721129 |
| Gm5150 | 0.00144 | -1.30017714 |
| Tdrd3 | 0.00959 | 0.93242838 |
| Gm3556 | 0.88771 | 0.03438283 |
| Hdac5 | 0.00144 | -1.1411857 |
| Ramp1 | 0.10805 | -0.49868908 |
| Spata3 | 0.17162 | -0.68522055 |
| 1810043G02Rik | 0.12768 | -0.61248756 |
| Otol1 | 0.00149 | -1.22582153 |
| Gm32742 | 0.00145 | -1.22376446 |
| Bzrap1 | 0.00144 | -1.27165604 |
| Tceal3 | 0.22671 | -0.54517483 |
| Klk9 | 0.48537 | -0.26322404 |
| Psmb11 | 0.91664 | 0.14040983 |
| Gys1 | 0.04317 | -0.62472318 |
| Naa10 | 0.09437 | -0.6370184 |
| Tmem9b | 0.23402 | -0.25914372 |
| Hsd3b7 | 0.51281 | -0.12962195 |
| Slc8a3 | 0.23951 | 0.40033793 |
| Dnajc5 | 0.0755 | -0.49668574 |
| Dnajc5 | 0.39744 | -0.33787589 |
| Dnajb5 | 0.00632 | -1.02372452 |
| Rtfdc1 | 0.0049 | -0.68004898 |
| Gm16442 | 0.00144 | -1.30626366 |
| Cib1 | 0.00144 | -1.08910124 |
| Gm20767 | 0.0044 | -1.14548549 |
| Tma16 | 0.02737 | 0.46642205 |
| Rad51d | 0.02254 | -0.67808685 |
| Kdm3a | 0.00354 | 0.82909173 |
| Rhbdf1 | 0.00553 | -0.69096015 |
| Gm26795 | 0.01527 | -0.95910692 |
| Elovl4 | 0.30736 | -0.60858265 |
| Cyhr1 | 0.77041 | -0.13293122 |
| Gm14496 | 0.47145 | -0.29148971 |
| Gldn | 0.02477 | -1.01647779 |
| 1700012L04Rik | 0.20799 | 0.57815221 |
| Gm9937 | 0.13939 | -1.29530941 |
| Gm4432 | 0.0855 | -0.75941838 |
| Eps8 | 0.00192 | 0.97405854 |
| Hmgn5 | 0.00451 | 2.33182082 |
| Gm32391 | 0.51727 | 0.15392015 |
| Gpm6b | 0.17613 | 0.77532149 |
| Scara5 | 0.00165 | -1.97386367 |
| Vmn1r204 | 0.06891 | -0.96943506 |
| Llph | 0.00144 | 1.277132 |
| Ndst4 | 0.00162 | -1.69066619 |
| Clca3a1 | 0.00144 | 1.80480776 |
| Ctrb1 | 0.01534 | -0.60048534 |
| Zfp708 | 0.95692 | -0.01221949 |
| Ap3b2 | 0.08172 | -1.34794077 |
| Sgsm2 | 0.00148 | -0.88041645 |
| H2afz | 0.08354 | -0.36809216 |
| Spsb4 | 0.70768 | 0.24631527 |
| Nelfcd | 0.01332 | -0.76574275 |
| Homer1 | 0.15945 | 0.34967296 |
| Drap1 | 0.00315 | -0.93155373 |
| Icam2 | 0.00144 | -1.37050124 |
| Gm29735 | 0.07271 | -0.4258364 |
| BC117090 | 0.08631 | -0.87682572 |
| Dcdc2c | 0.00144 | -1.22925777 |
| Tnfrsf13b | 0.00856 | -0.64796425 |
| Tnfrsf13b | 0.02556 | -0.59871558 |
| Trafd1 | 0.01499 | -0.60287976 |
| Scg5 | 0.58593 | 0.27998159 |
| Ptprt | 0.00144 | -1.21977358 |
| Ercc6l2 | 0.00427 | -0.70117882 |
| Ercc6l2 | 0.00144 | 1.38432855 |
| Ccm2l | 0.81374 | 0.05348663 |
| Rhox8 | 0.00144 | 3.43930158 |
| Myo3b | 0.00144 | -1.03574715 |
| Gp1ba | 0.00144 | -1.27454685 |
| Ccdc101 | 0.33883 | -0.31478916 |
| Rrp8 | 0.43088 | 0.21520903 |
| Vmn2r88 | 0.70207 | 0.20706628 |
| Donson | 0.00308 | 1.36660765 |
| Zcchc6 | 0.00423 | 1.0570309 |
| Nr2c2 | 0.13178 | 0.28390656 |
| Nr2c1 | 0.00544 | 0.77554382 |
| Rreb1 | 0.72731 | -0.10921217 |
| Rreb1 | 0.15529 | 0.35986811 |
| Rreb1 | 0.42839 | 0.38753607 |
| Ccdc113 | 0.11269 | -0.63490044 |
| Syt8 | 0.03035 | -0.78031828 |
| Atic | 0.07027 | -0.31463284 |
| Scgb2b12 | 0.00144 | -1.26560808 |
| Mecom | 0.1914 | -0.58614844 |
| Olfr670 | 0.00144 | -1.24613586 |
| Ccdc125 | 0.00176 | 1.20904777 |
| Olfr470 | 0.09028 | -1.07345775 |
| Olfr270 | 0.00254 | -1.1839779 |
| Park7 | 0.07151 | -0.71560249 |
| Gm5615 | 0.00144 | -1.29864962 |
| Cfap70 | 0.02213 | -0.84233455 |
| Rpl10a | 0.00935 | -0.63679533 |
| H2-Bl | 0.0021 | -1.03443005 |
| Irgm1 | 0.00252 | 0.79066013 |
| Olfr1046 | 0.00144 | -1.31014844 |
| 5430410E06Rik | 0.01384 | -0.89747573 |
| Dtx2 | 0.00144 | -1.25281511 |
| Dnah12 | 0.00144 | -1.28116048 |
| Gsk3b | 0.0077 | -0.63156621 |
| Gsk3a | 0.00995 | -0.61532696 |
| Lamc1 | 0.74 | 0.05071405 |
| Lamc2 | 0.07271 | -0.37517193 |
| Gm21637 | 0.11175 | -0.684874 |
| Vmn2r114 | 0.00144 | -1.28898079 |
| Sdccag8 | 0.27954 | 0.33010555 |
| Grhl2 | 0.03039 | -0.62724179 |
| Olfr1290 | 0.0017 | -1.24950944 |
| Hdgfrp3 | 0.00676 | 0.91300878 |
| Nup88 | 0.1632 | 0.25175424 |
| Ankrd33 | 0.00144 | -1.48564159 |
| Gm2582 | 0.01098 | -1.66775838 |
| Fkbp14 | 0.03506 | 0.4752645 |
| Il24 | 0.89594 | 0.05761873 |
| Aatf | 0.0475 | -0.37647582 |
| Xrcc4 | 0.00206 | 1.17273311 |
| Mup-ps12 | 0.00144 | -1.25118919 |
| Drd3 | 0.18864 | 0.58541604 |
| C130026I21Rik | 0.01458 | -1.28797675 |
| Phldb1 | 0.00945 | -0.76301281 |
| Trub2 | 0.03538 | -0.97810926 |
| Trub2 | 0.11041 | -0.27806184 |
| Ap1b1 | 0.01759 | -0.76290503 |
| Olfr1458 | 0.00144 | -1.31495282 |
| Oosp2 | 0.84614 | -0.23613215 |
| Slc35c2 | 0.00599 | -0.86734479 |
| Lca5l | 0.00878 | -0.62797988 |
| Apba3 | 0.00249 | -1.13210213 |
| Osbp | 0.00839 | -0.82882071 |
| 1110012L19Rik | 0.01332 | -0.60218158 |
| 4921504E06Rik | 0.03198 | -1.93977585 |
| Ephb6 | 0.00431 | -0.94355326 |
| Gm6313 | 0.00428 | -1.75556429 |
| Trav7n-5 | 0.00144 | -1.29051721 |
| Arpc2 | 0.06907 | -0.38975727 |
| Kcnrg | 0.00673 | 0.92241825 |
| Kcnrg | 0.68011 | -0.28464482 |
| Il2ra | 0.17587 | -0.25068088 |
| Fam83c | 0.01241 | -2.63672191 |
| Olfr536 | 0.0254 | -1.82367123 |
| 1520401A03Rik | 0.13286 | 0.35166308 |
| Dram2 | 0.00197 | 1.10917408 |
| Olfr824 | 0.0085 | -1.1190958 |
| Olfr724 | 0.00957 | -1.54749165 |
| 1700007K09Rik | 0.00477 | -0.686434 |
| Ubr5 | 0.29204 | 0.19208364 |
| Trmt2b | 0.21645 | 0.27616897 |
| Leprot | 0.09147 | -0.31847875 |
| Gm4316 | 0.16256 | 0.62119295 |
| Ube2i | 0.42179 | 0.23178496 |
| Cetn4 | 0.12425 | 0.48049885 |
| Pramel4 | 0.00145 | -1.26219879 |
| Ccdc85a | 0.73187 | 0.13314587 |
| Gm13274 | 0.62908 | -0.19652802 |
| Grin2a | 0.00144 | -1.28116048 |
| 2900079G21Rik | 0.00144 | -1.27699683 |
| Sbno1 | 0.41869 | 0.13155742 |
| Zkscan1 | 0.11169 | -0.29406414 |
| Cbx7 | 0.02092 | -0.63525204 |
| Pcbp3 | 0.0138 | -0.55736664 |
| Pcbp2 | 0.12405 | -0.27709972 |
| Stap1 | 0.0763 | -1.12515525 |
| Birc3 | 0.00892 | 0.679394 |
| Cxx1b | 0.02082 | -0.67816844 |
| Acad11 | 0.01167 | 0.78562526 |
| Adamts12 | 0.00144 | -1.27165604 |
| Tardbp | 0.10216 | 0.49601989 |
| Gm8098 | 0.00144 | 1.74873123 |
| Prdm12 | 0.31854 | -0.68806557 |
| Srsf3 | 0.00329 | 0.81728876 |
| Gm5901 | 0.10388 | -0.51348393 |
| Gm8359 | 0.00144 | -1.29049509 |
| Gm4312 | 0.00644 | -1.05441012 |
| Gm10334 | 0.37293 | -0.4822858 |
| Tmem189 | 0.42098 | -0.24737092 |
| Il17d | 0.00144 | -1.04615689 |
| Tbc1d9b | 0.00775 | -0.66092341 |
| Rsl1d1 | 0.091 | 0.35977201 |
| 1700026L06Rik | 0.00658 | -1.72141296 |
| Crcp | 0.7034 | -0.06061884 |
| Gm35578 | 0.39174 | 0.40731967 |
| Vps52 | 0.91078 | -0.06657364 |
| Mup1 | 0.04884 | -2.05645947 |
| Tef | 0.02975 | -0.46879053 |
| Gm7167 | 0.76979 | -0.44459655 |
| Cited1 | 0.28216 | -0.30327217 |
| Srp54b | 0.01589 | -1.07690166 |
| Abcc8 | 0.09983 | -0.34454567 |
| 6430411K18Rik | 0.00144 | -1.31884045 |
| Atf4 | 0.00879 | -0.69877213 |
| Klk11 | 0.81364 | -0.16944707 |
| Ubap1 | 0.42361 | -0.13924902 |
| Spop | 0.88413 | 0.02715352 |
| Rhoc | 0.00162 | -0.899279 |
| Ikzf4 | 0.09223 | -0.35569217 |
| Nsl1 | 0.01941 | -0.95924808 |
| S100a1 | 0.00205 | -1.14495999 |
| Sepn1 | 0.00377 | -0.72784342 |
| Shroom2 | 0.06797 | 0.50689296 |
| Bpifb9b | 0.04341 | -2.01588074 |
| Wdr62 | 0.80023 | 0.13863594 |
| Pitrm1 | 0.00147 | 1.09645939 |
| Rp1 | 0.12744 | -0.76501493 |
| Gm13490 | 0.11227 | -0.94598108 |
| Hras | 0.01241 | -1.29914342 |
| Clic3 | 0.00749 | -1.02349059 |
| Cd28 | 0.17269 | 0.39200537 |
| Malrd1 | 0.00144 | -1.31884045 |
| Gm9954 | 0.0469 | -1.61943063 |
| Prdx5 | 0.01288 | -0.79907028 |
| Prdx2 | 0.00341 | -1.04994126 |
| Pom121l2 | 0.00144 | -1.26560808 |
| Tigar | 0.29013 | -0.2295158 |
| Palm | 0.00262 | -0.81914583 |
| Cyba | 0.00144 | -1.05223454 |
| 1810062G17Rik | 0.93631 | -0.04781216 |
| Dapp1 | 0.15419 | -0.29944806 |
| Gle1 | 0.52445 | -0.13221125 |
| Lrit3 | 0.34945 | -0.59136626 |
| Rab1 | 0.00351 | 0.90384662 |
| Glod4 | 0.15722 | -0.2492835 |
| Cep192 | 0.0955 | 0.9461021 |
| Abtb1 | 0.00144 | -0.93613381 |
| Tbata | 0.06252 | -0.69066192 |
| Tbata | 0.82476 | -0.11858196 |
| Tbata | 0.0103 | -0.61173352 |
| Klrb1c | 0.04881 | -0.84729793 |
| E330009J07Rik | 0.00418 | -0.91063746 |
| Naip1 | 0.00144 | -1.4628551 |
| Ublcp1 | 0.00744 | 0.68503392 |
| Shc1 | 0.00507 | -0.66554568 |
| Atp6v0c | 0.15645 | -0.4987699 |
| Tmem65 | 0.5586 | 0.1491675 |
| Zfp648 | 0.15975 | -0.40511425 |
| Gm14851 | 0.00243 | -1.12283767 |
| Defa-rs1 | 0.00144 | -1.28898079 |
| Rp2h | 0.10268 | 0.34128558 |
| Slc9a3r2 | 0.00318 | -0.78454876 |
| Slc7a15 | 0.0219 | -1.78034564 |
| Srgap1 | 0.95292 | 0.03374248 |
| Skint3 | 0.14261 | 0.7968417 |
| Skint3 | 0.15687 | -0.81215216 |
| Kmt2b | 0.00522 | -0.69326661 |
| Vmn1r183 | 0.00861 | -1.93011617 |
| Camk2d | 0.33754 | -0.20165207 |
| Gm10556 | 0.00145 | -1.25646236 |
| Srsf10 | 0.83985 | 0.12584884 |
| Pja2 | 0.98498 | 0.0032819 |
| Zfp64 | 0.93904 | 0.01618198 |
| Zfp606 | 0.18924 | 0.5533044 |
| Gm26806 | 0.00144 | -1.28116048 |
| Abcb4 | 0.0188 | -0.7626673 |
| Mospd3 | 0.12651 | -0.26733324 |
| Sybu | 0.39235 | -0.15065211 |
| Wasf3 | 0.00487 | -1.44422578 |
| Wasf1 | 0.01838 | -0.64484664 |
| Gm5166 | 0.08286 | -1.04626337 |
| Grcc10 | 0.00161 | -1.32054374 |
| Rpl17 | 0.59398 | -0.09536641 |
| Trit1 | 0.14069 | 0.30289294 |
| Dtx2 | 0.00386 | -0.8680066 |
| Otx2 | 0.00144 | -1.28116048 |
| Gm10439 | 0.0051 | -1.06784446 |
| Gbp10 | 0.64285 | 0.09789803 |
| H2-Q7 | 0.00313 | -0.98546859 |
| H2-Q8 | 0.0026 | -1.12236646 |
| H2-Q1 | 0.00258 | -1.49768 |
| Nr1h2 | 0.00212 | -0.84222997 |
| Pnma5 | 0.00144 | -1.25118919 |
| H2-Q5 | 0.0084 | -1.3259566 |
| Ttc1 | 0.33014 | -0.16680888 |
| Kif3a | 0.49122 | 0.15153522 |
| Bcl2a1d | 0.09824 | -0.54685619 |
| Chd5 | 0.28608 | -1.41186397 |
| Panx2 | 0.03166 | -0.92187831 |
| Fbxo8 | 0.00697 | 0.76857063 |
| Adamts14 | 0.31182 | -0.24825089 |
| Erh | 0.20897 | -0.23775252 |
| Saxo1 | 0.00761 | -1.08938024 |
| Gm13078 | 0.01368 | -1.68466871 |
| 4930519F16Rik | 0.00144 | -1.28116048 |
| Artn | 0.66941 | -0.24021765 |
| Mrps7 | 0.07132 | -0.51512083 |
| Park2 | 0.96607 | 0.00742978 |
| C2cd4c | 0.00286 | -1.26584838 |
| Abhd17c | 0.00339 | -0.78112536 |
| Zfp945 | 0.07754 | 1.19446409 |
| Rpl35 | 0.00144 | -1.16801419 |
| LOC102633705 | 0.11519 | -0.74145114 |
| Tnip2 | 0.4283 | 0.13376628 |
| Vmn2r78 | 0.14343 | -3.4260543 |
| Mutyh | 0.8504 | -0.0350116 |
| 1700080N15Rik | 0.05273 | -0.92312902 |
| Dcaf17 | 0.00144 | 1.22103876 |
| Dcaf17 | 0.00145 | -1.22376446 |
| Arvcf | 0.06836 | -1.3223628 |
| Aven | 0.27097 | -0.24680885 |
| Sez6 | 0.01436 | -1.04604364 |
| Gm5795 | 0.00144 | -1.26560808 |
| Obp1a | 0.04951 | -0.4040806 |
| Pigp | 0.2221 | 0.44543296 |
| Epb4.1 | 0.0972 | -0.29085929 |
| Insm1 | 0.75675 | -0.19348234 |
| Fcgbp | 0.00145 | -1.2388121 |
| Itgam | 0.16553 | 0.39313029 |
| Itgam | 0.28678 | -0.43945351 |
| C430049B03Rik | 0.89841 | 0.04796608 |
| Gm5833 | 0.34755 | 0.26037957 |
| Arglu1 | 0.84709 | -0.04348647 |
| Adgrg4 | 0.00144 | -1.22925777 |
| Sall1 | 0.00158 | -1.2446827 |
| Cd244 | 0.09636 | 0.6332507 |
| Izumo1r | 0.07137 | 0.64427871 |
| Folr1 | 0.79731 | -0.14527252 |
| Cfap57 | 0.00144 | -1.28898079 |
| Rs5-8s1 | 0.00144 | -1.12400479 |
| Ttc14 | 0.01566 | 1.82564514 |
| Picalm | 0.00376 | 1.11080938 |
| Znrf4 | 0.46696 | -0.63450588 |
| Npy2r | 0.00144 | -1.32599123 |
| Palld | 0.03481 | -0.4633832 |
| Hoxd8 | 0.28364 | 0.46109076 |
| Irf2bpl | 0.00144 | -0.97103813 |
| Epc2 | 0.19149 | -0.22744813 |
| Galk2 | 0.0117 | 0.72019919 |
| Eno3 | 0.01257 | -0.70258129 |
| Htr2c | 0.2406 | -1.85970277 |
| Htr2b | 0.08452 | 0.73576002 |
| Zfp629 | 0.01686 | -0.52992606 |
| Dab2ip | 0.00145 | -0.93512962 |
| 4930570G19Rik | 0.12465 | -1.00087357 |
| Cck | 0.0022 | -1.13659127 |
| Oscar | 0.40903 | -0.16950525 |
| Rps20 | 0.00144 | -1.15995917 |
| Ugt2b34 | 0.11911 | -2.86005478 |
| 1700060C20Rik | 0.00336 | -1.20864476 |
| Olfr891 | 0.00144 | -1.48581436 |
| Ptprn2 | 0.02739 | -0.88969602 |
| Ogfod2 | 0.01443 | -0.58348319 |
| Olfr191 | 0.06985 | -0.98550712 |
| Zbtb1 | 0.011 | 1.90695547 |
| Ulk1 | 0.33861 | -0.29340734 |
| Pde11a | 0.02369 | -1.20348633 |
| Zbtb9 | 0.01373 | -0.54465425 |
| Gm6614 | 0.00798 | -1.03220606 |
| Slc22a13 | 0.65855 | -0.08705309 |
| Gm9916 | 0.00144 | -1.28898079 |
| Ferd3l | 0.00551 | -1.40791784 |
| Gltscr1 | 0.0447 | -0.74161115 |
| Gm29142 | 0.19386 | -0.73947343 |
| Susd6 | 0.08293 | -0.34390042 |
| Elk4 | 0.18626 | -0.25265158 |
| Fam110a | 0.08968 | -0.29345548 |
| 1700034J04Rik | 0.00144 | -1.24613586 |
| Gfi1 | 0.00144 | -1.26934956 |
| Zfp707 | 0.01002 | -0.70459789 |
| Tmprss9 | 0.04376 | -0.61178977 |
| Tmprss9 | 0.00144 | -1.31884045 |
| Unc13d | 0.02102 | -0.54181823 |
| Unc13d | 0.01411 | -0.78930254 |
| Tgfbr3l | 0.24985 | -0.2005841 |
| Pnp2 | 0.17922 | -0.42045618 |
| LOC102636203 | 0.00213 | -1.21132865 |
| Hsdl2 | 0.09172 | -0.32945421 |
| Fbxl20 | 0.10459 | 0.51656172 |
| D130009I18Rik | 0.00144 | -1.26934956 |
| Olfr150 | 0.00145 | -1.2388121 |
| Polr3e | 0.01577 | 0.64845678 |
| Gm36028 | 0.00313 | 0.82165857 |
| Cacna2d2 | 0.008 | -1.24043998 |
| Gm3871 | 0.03941 | -0.81203304 |
| Gm36340 | 0.0134 | -1.90417361 |
| Snrnp35 | 0.02962 | -0.4684483 |
| Snrnp35 | 0.01292 | -0.50869955 |
| Olfr46 | 0.10549 | -1.97012735 |
| Gm14625 | 0.00149 | -1.46382301 |
| Olfr657 | 0.90675 | 0.0513417 |
| Tmod2 | 0.18272 | 0.54316308 |
| Tmod4 | 0.17519 | -0.23393122 |
| Olfr645 | 0.59887 | -0.3396996 |
| Clcnka | 0.36414 | -0.78410058 |
| Wdr72 | 0.47143 | -0.60633928 |
| Wdr72 | 0.00158 | -1.2446827 |
| Ccdc176 | 0.24122 | 0.48579597 |
| Lman2l | 0.02356 | -0.56285087 |
| Tgm4 | 0.00144 | -1.30713006 |
| 3110053B16Rik | 0.12777 | -0.26672494 |
| Olfr1120 | 0.00144 | -1.28116048 |
| Gimap4 | 0.06619 | 0.50738508 |
| Gpcpd1 | 0.00374 | -0.93575498 |
| Tbx20 | 0.16347 | 0.32070386 |
| Prkcg | 0.00144 | 2.14881327 |
| Prkcb | 0.22095 | 0.51007104 |
| Ckb | 0.00499 | -0.79653275 |
| Arf5 | 0.00144 | -1.09933527 |
| Cdkn1a | 0.75778 | -0.10481604 |
| Lmtk3 | 0.50738 | -0.36039869 |
| Vrk2 | 0.09762 | -0.3345916 |
| Gm10609 | 0.18811 | -0.79537494 |
| Ect2 | 0.05269 | 0.61870283 |
| C130074G19Rik | 0.91749 | -0.02640368 |
| Rps27a | 0.00231 | -0.7865036 |
| Slc39a9 | 0.86344 | -0.05999562 |
| Fads3 | 0.01434 | -0.55757523 |
| Gm12059 | 0.23813 | -0.32452107 |
| Olfr1475 | 0.00144 | -1.28116048 |
| A430089I19Rik | 0.00144 | -1.32313902 |
| Cnih4 | 0.02302 | -0.60325724 |
| Psme4 | 0.09779 | 0.27860212 |
| Wdr63 | 0.00144 | -1.31884045 |
| Syt3 | 0.41979 | -0.30445108 |
| LOC102642641 | 0.12031 | -0.8451782 |
| Olfr629 | 0.00869 | -0.71496758 |
| Itlnb | 0.00144 | -1.35320495 |
| Dnmt3b | 0.09703 | -0.50758882 |
| Bcl2l15 | 0.00144 | -1.25118919 |
| Zfp873 | 0.00618 | 1.9114531 |
| 1810009N02Rik | 0.00164 | -1.20803357 |
| Gm6880 | 0.00148 | -1.63079701 |
| Srd5a3 | 0.73179 | -0.06118587 |
| Sipa1l3 | 0.01741 | -0.55787868 |
| Skint7 | 0.00144 | -1.28116048 |
| Tor1aip1 | 0.6323 | 0.1194058 |
| Snx18 | 0.41604 | -0.13735376 |
| Bicc1 | 0.02109 | -0.59960107 |
| Rhox2e | 0.00432 | -0.9065518 |
| Prps1l3 | 0.59184 | 0.08897256 |
| Rhox4g | 0.91348 | -0.06467478 |
| Rhox4e | 0.07459 | -0.82155609 |
| Hemgn | 0.02767 | -0.55751576 |
| Zfp217 | 0.12079 | -0.33543996 |
| Ttc34 | 0.41671 | -0.23171514 |
| Ifi204 | 0.00161 | 1.83983236 |
| Gm20736 | 0.24463 | 0.5601476 |
| Nutf2 | 0.96671 | -0.01364717 |
| H6pd | 0.00307 | -0.88450506 |
| Gm20865 | 0.15332 | -0.76914284 |
| Olfr1128 | 0.00144 | -1.24466058 |
| Ptger3 | 0.00144 | -1.28116048 |
| Mdm2 | 0.00448 | 0.96914987 |
| Nfyb | 0.00543 | 0.74905278 |
| St3gal3 | 0.00787 | -0.86515574 |
| 2210417A02Rik | 0.07033 | -0.86389263 |
| Dhx40 | 0.00144 | 1.56633476 |
| Cpped1 | 0.99628 | 0.00091961 |
| Flnc | 0.00236 | -1.08726139 |
| Ighmbp2 | 0.01403 | -0.59842284 |
| Dctn1 | 0.0059 | -0.82741309 |
| Oraov1 | 0.30735 | -0.63665019 |
| C030010C08Rik | 0.03833 | -0.78004423 |
| Gm10745 | 0.0156 | -1.08617297 |
| Tmem259 | 0.00144 | -1.0578906 |
| Tgif2lx2 | 0.17803 | -0.58410452 |
| Crygc | 0.03002 | -1.36016688 |
| Crygf | 0.14465 | -0.8050125 |
| Cryge | 0.0018 | -1.21153109 |
| Nwd2 | 0.00149 | -1.22582153 |
| Bicd1 | 0.01313 | 1.19076873 |
| Npas4 | 0.09594 | -0.6862979 |
| Fubp3 | 0.7453 | -0.09307038 |
| Sqle | 0.13745 | -0.57017685 |
| Acyp1 | 0.07531 | -0.58192204 |
| Agtr1b | 0.00144 | -1.29746368 |
| Mageb18 | 0.00144 | -1.27165604 |
| Galnt6 | 0.02898 | -0.91326254 |
| Acvr1c | 0.0132 | -0.89971117 |
| Timmdc1 | 0.00672 | -0.74630176 |
| Ugt2b5 | 0.00144 | -1.64745053 |
| Ssxb2 | 0.00149 | -1.22582153 |
| Amt | 0.31411 | -0.2843862 |
| Sgcg | 0.93113 | 0.02127298 |
| Rad18 | 0.03506 | 1.47801481 |
| Ufc1 | 0.00589 | -0.88197732 |
| Sprr2a2 | 0.03084 | -0.40123606 |
| Plekhg3 | 0.01131 | -0.67152857 |
| Tnr | 0.03649 | -0.68564236 |
| Tmem232 | 0.26222 | -0.53315402 |
| Olfr668 | 0.00144 | -1.30017714 |
| Olfr768 | 0.04353 | -1.9195854 |
| Ndufab1 | 0.05131 | -0.61229375 |
| Bcl2l1 | 0.21257 | -0.262623 |
| Hormad1 | 0.00144 | -1.28248344 |
| Zfp868 | 0.00154 | 1.13445788 |
| Pde1a | 0.55704 | -0.12937236 |
| Pde1c | 0.08055 | 0.45086569 |
| Pde1b | 0.0421 | -0.45707465 |
| A930006D01Rik | 0.08942 | -1.21568621 |
| Slc24a4 | 0.60911 | -0.33801935 |
| Cfl1 | 0.08464 | -0.35659524 |
| Tsnax | 0.02044 | 0.45232834 |
| Duox2 | 0.01569 | -1.45976249 |
| Nsun4 | 0.65328 | -0.08232864 |
| Sft2d1 | 0.68906 | -0.09924209 |
| Entpd2 | 0.00144 | -1.18884507 |
| Gm14625 | 0.00145 | 2.34787082 |
| BC037156 | 0.03895 | -0.94383626 |
| Trappc9 | 0.00264 | -0.84302769 |
| C030039L03Rik | 0.00841 | 1.00079663 |
| Map2k7 | 0.62377 | -0.18005582 |
| Vangl1 | 0.00319 | -0.98298723 |
| Eif4h | 0.53064 | -0.13910718 |
| Nfam1 | 0.00573 | -0.9267536 |
| Plch2 | 0.57232 | -0.30428268 |
| A530099J19Rik | 0.13103 | -0.70188915 |
| Atp8b5 | 0.08331 | -0.91134937 |
| Msh3 | 0.00726 | 1.06701704 |
| Ndufb6 | 0.04906 | -0.65785824 |
| Lime1 | 0.96428 | -0.01269617 |
| Sumf2 | 0.00241 | -0.92329542 |
| Kat2a | 0.00418 | -0.8189267 |
| Cdk14 | 0.01412 | -0.56259411 |
| Defb26 | 0.0759 | 0.45579036 |
| Fabp3 | 0.00342 | -0.7662371 |
| Hp1bp3 | 0.99165 | 0.00181272 |
| Olfr1038-ps | 0.00144 | -1.28375735 |
| Rela | 0.00208 | -0.88597145 |
| Ndufs7 | 0.01246 | -0.67793013 |
| Dbh | 0.00484 | -1.46166022 |
| Dtd2 | 0.40931 | 0.19064113 |
| Kif4-ps | 0.00144 | -1.48661212 |
| Fam172a | 0.00612 | 0.86352276 |
| Ptges | 0.15807 | 0.23253136 |
| Ptges | 0.05888 | -0.9677146 |
| Olfr204 | 0.00144 | -1.29049509 |
| Ict1 | 0.00227 | -1.01248647 |
| Gjd2 | 0.09393 | -1.03091905 |
| Mup19 | 0.14424 | -1.91095373 |
| Gm2545 | 0.98522 | 0.01900737 |
| Gm7715 | 0.0046 | -1.12385295 |
| Stab2 | 0.15782 | -0.54346072 |
| Dapk1 | 0.65915 | -0.08335576 |
| Tnfrsf23 | 0.22401 | -0.49287388 |
| Gm6756 | 0.00144 | -1.53591432 |
| Ppp1r13b | 0.01698 | -0.51850645 |
| Krt74 | 0.01152 | -1.15943925 |
| Krt75 | 0.00276 | -1.14169705 |
| Krt79 | 0.65356 | 0.08589801 |
| Ipo13 | 0.00511 | -0.78942224 |
| Trp53inp1 | 0.0071 | 0.65290875 |
| Lcor | 0.00144 | -1.20555712 |
| Vmn2r86 | 0.36009 | -0.58176465 |
| Socs4 | 0.14709 | -0.26579492 |
| Gm31992 | 0.03116 | -1.34838753 |
| Sema3b | 0.41564 | -0.19288769 |
| Slc6a9 | 0.00144 | -1.25945688 |
| Hbb-bt | 0.15184 | -0.57792632 |
| Tex9 | 0.00448 | 1.15799081 |
| Pde4c | 0.97841 | -0.00737648 |
| Unc5b | 0.0028 | -0.87511477 |
| Gm4454 | 0.00144 | -1.30017714 |
| Etfdh | 0.0354 | 0.6035451 |
| Htra2 | 0.23699 | -0.35518668 |
| Gm15482 | 0.00144 | -1.30713006 |
| Raf1 | 0.07408 | -0.45681679 |
| Dlx1 | 0.00158 | -1.2446827 |
| D630008O14Rik | 0.15131 | -1.32235068 |
| Spn | 0.00927 | -1.58895338 |
| 5730559C18Rik | 0.00144 | -1.26560808 |
| Bbs12 | 0.06702 | 1.12336015 |
| Fam216b | 0.09843 | -1.31394704 |
| Rnf7 | 0.71416 | 0.12065731 |
| Duxbl2 | 0.00346 | -1.22679402 |
| Duxbl1 | 0.00144 | -1.28116048 |
| Ceacam16 | 0.01954 | 0.511218 |
| Ybx2 | 0.00708 | 0.59926367 |
| Tex14 | 0.00144 | -1.25927895 |
| Gm15433 | 0.74062 | 0.10577306 |
| Tada2a | 0.84506 | 0.03797597 |
| Vstm2a | 0.00711 | -3.45914948 |
| Gm42035 | 0.69763 | 0.07309427 |
| Mapk9 | 0.82267 | 0.03739592 |
| Spint5 | 0.58078 | -0.76526938 |
| Ksr1 | 0.0337 | -0.60259194 |
| Trim34b | 0.40578 | 0.46245415 |
| Tax1bp3 | 0.56047 | -0.13246601 |
| Cldn17 | 0.44615 | -0.33753335 |
| Gm2240 | 0.00623 | -1.33966906 |
| Gm2238 | 0.00742 | 0.79451574 |
| Haus5 | 0.64273 | 0.075226 |
| Bricd5 | 0.66584 | -0.16944999 |
| Gm10753 | 0.05729 | -0.89604186 |
| AI507597 | 0.61549 | -0.25313433 |
| Vmn1r29 | 0.00719 | -1.83065656 |
| Cttnbp2nl | 0.05392 | 0.40711508 |
| Ltbp1 | 0.15613 | -0.79725591 |
| Ltbp1 | 0.04443 | -0.42841276 |
| Gm10417 | 0.00158 | -1.2446827 |
| H2-DMb1 | 0.00144 | -1.31495282 |
| Celf5 | 0.87837 | -0.12362946 |
| Celf5 | 0.00144 | -1.28116048 |
| Gm35410 | 0.02491 | -0.52296351 |
| Cab39 | 0.40057 | 0.20925425 |
| Ankrd65 | 0.02576 | -1.8941726 |
| Ifit3b | 0.08013 | 0.53771933 |
| Gm21498 | 0.10376 | -2.63133643 |
| Unkl | 0.99267 | -0.00385184 |
| Mcrs1 | 0.00212 | -0.92436883 |
| Klf3 | 0.11974 | 0.32566296 |
| Rps28 | 0.00144 | -1.08510897 |
| Stil | 0.13498 | -0.47820632 |
| Gsg1 | 0.15396 | -1.27412361 |
| 0610037L13Rik | 0.01809 | 0.93966052 |
| 0610037L13Rik | 0.07944 | -0.31220886 |
| Pmm1 | 0.00145 | -0.94358068 |
| Pmm1 | 0.01346 | -0.96547203 |
| Spinkl | 0.00144 | -1.27165604 |
| Rars2 | 0.00674 | 0.66537062 |
| Wnk1 | 0.00772 | 0.84326079 |
| Smok2b | 0.00145 | -1.20627532 |
| Smok3b | 0.04588 | -0.47644985 |
| Tada3 | 0.01752 | -0.71503877 |
| Gm15340 | 0.0158 | -1.54651916 |
| Mtcp1 | 0.03241 | 0.63002702 |
| Vwa5b2 | 0.00144 | -1.27165604 |
| Adora3 | 0.46767 | -0.21971045 |
| Tgif2 | 0.02375 | -0.80444953 |
| Tmeff1 | 0.01019 | 0.8575367 |
| Zfp92 | 0.66744 | -0.18706478 |
| Zfp960 | 0.00205 | 1.72376196 |
| Eftud1 | 0.76896 | 0.04987783 |
| Gm11544 | 0.0682 | -0.99236187 |
| Gm42372 | 0.0055 | -1.26149799 |
| Psg16 | 0.07297 | -0.42139438 |
| Cmtm1 | 0.00144 | -1.76800206 |
| Themis | 0.00144 | -1.27165604 |
| Gjb4 | 0.00229 | -1.15758681 |
| Mrgprb8 | 0.00149 | -1.25408 |
| Wars | 0.00145 | -0.94643694 |
| Sars | 0.00184 | -0.82121109 |
| Slc12a6 | 0.06747 | -0.87009624 |
| Phka1 | 0.7136 | 0.05978619 |
| Slc25a14 | 0.50632 | 0.11422525 |
| Siah1b | 0.19973 | -0.21239623 |
| Cacng2 | 0.00466 | -0.92549187 |
| Vmn1r61 | 0.00144 | -1.25118919 |
| Espnl | 0.00148 | -1.0306067 |
| Arhgap27 | 0.16556 | -0.56505754 |
| Ccndbp1 | 0.51774 | 0.20708573 |
| Acsm3 | 0.00144 | -1.29085005 |
| Capn10 | 0.01013 | -0.7104808 |
| Hist1h4c | 0.00144 | -1.42908685 |
| Gm324 | 0.24965 | -0.37877382 |
| Kdf1 | 0.00144 | -1.26042915 |
| Uba1 | 0.08571 | -0.41705441 |
| Tmem176b | 0.05987 | -0.43920012 |
| Tmem176b | 0.65385 | -0.12429286 |
| Ift46 | 0.00865 | -0.64874205 |
| Cd86 | 0.09138 | 0.65937631 |
| Defa21 | 0.00434 | -3.37686229 |
| Pla2r1 | 0.07282 | 1.21804437 |
| F3 | 0.01357 | 1.11599132 |
| Gm5922 | 0.00144 | -1.28116048 |
| Rbm45 | 0.49837 | -0.13313205 |
| Gm6042 | 0.00144 | -1.27165604 |
| Vcpkmt | 0.47139 | -0.13215881 |
| Sh3glb2 | 0.00147 | -0.9834477 |
| Muc13 | 0.02985 | 0.99471244 |
| Muc15 | 0.00145 | -1.26219879 |
| Fsip1 | 0.33398 | 0.52884538 |
| Fsip1 | 0.00144 | -1.28116048 |
| Gm10057 | 0.00144 | -1.28116048 |
| Cr2 | 0.89861 | 0.08226473 |
| Gm3287 | 0.00144 | -1.31884045 |
| Ttc19 | 0.52402 | -0.11979967 |
| Fam206a | 0.79552 | 0.04728647 |
| Bcan | 0.05388 | -2.08648616 |
| Bcan | 0.01878 | -1.75735193 |
| Pqbp1 | 0.00402 | -0.74288254 |
| Acox3 | 0.02407 | -0.68633369 |
| Acox3 | 0.00144 | -1.37833939 |
| Gm33819 | 0.00144 | -1.20555712 |
| Slxl1 | 0.06178 | -0.86447076 |
| Kif13b | 0.12522 | -0.59090506 |
| Kif16b | 0.00415 | 0.95987379 |
| Exoc1 | 0.08466 | 0.35459103 |
| Gm4425 | 0.83875 | 0.03572374 |
| Snrpd1 | 0.29537 | -0.18211238 |
| Serpinb9c | 0.00144 | -1.29746368 |
| Serpinb9d | 0.00145 | -1.24191372 |
| Serpinb9e | 0.00152 | -1.35733286 |
| Serpinb9f | 0.00144 | -1.20579466 |
| Serpinb9g | 0.00198 | -1.13811458 |
| Nlrp12 | 0.23708 | 0.41914055 |
| Ciz1 | 0.57704 | -0.11790734 |
| Mta3 | 0.56968 | -0.12389675 |
| Lage3 | 0.01705 | -0.70749962 |
| Lonp1 | 0.02414 | -0.53075572 |
| Olfr1436 | 0.06084 | -0.88061585 |
| Kdm2a | 0.48912 | 0.14033122 |
| 6530403M18Rik | 0.03376 | -1.38063688 |
| 1700022I11Rik | 0.00459 | -1.99052192 |
| Eya3 | 0.03195 | -0.51758297 |
| Ndufa4l2 | 0.00158 | -0.86934481 |
| Mup3 | 0.40466 | -0.4479848 |
| Slc25a36 | 0.46418 | 0.11631379 |
| Slc4a1ap | 0.26127 | 0.19064301 |
| Timm23 | 0.55596 | 0.20941886 |
| Gm34983 | 0.00144 | -1.31495282 |
| Cd1d2 | 0.01556 | 0.5663153 |
| Gm5469 | 0.01631 | 1.03191617 |
| 9530002B09Rik | 0.00144 | -1.59764727 |
| Atp11a | 0.00231 | 0.91625195 |
| Paqr6 | 0.00144 | -1.0953062 |
| Gzme | 0.13028 | -1.51517305 |
| Urah | 0.13158 | -0.65287818 |
| 4930520O04Rik | 0.07808 | -0.95123685 |
| Pknox2 | 0.9093 | 0.03674913 |
| Hnf1a | 0.00149 | -1.25940286 |
| Stard13 | 0.00902 | 0.60083398 |
| Lin28b | 0.04666 | -0.84548799 |
| Gramd2 | 0.00144 | -1.34945434 |
| Olfr889 | 0.00144 | -1.30713006 |
| Vmn2r16 | 0.87222 | -0.18320785 |
| Olfr589 | 0.00537 | -1.5030208 |
| Gm5779 | 0.05424 | -0.77641561 |
| Rplp0 | 0.00479 | -0.98087538 |
| Pemt | 0.1094 | -0.29739855 |
| Pemt | 0.24803 | -0.47198221 |
| Nr2c2 | 0.37979 | 0.15007453 |
| Nubpl | 0.01949 | -1.23347991 |
| Adamts5 | 0.00144 | -1.09836803 |
| Agpat2 | 0.09895 | -0.55944812 |
| Hdac1 | 0.90867 | -0.0178941 |
| Pilrb2 | 0.93565 | 0.01944602 |
| Hdac9 | 0.00361 | 0.8104989 |
| Mllt6 | 0.00985 | -0.57313384 |
| Eif3j1 | 0.11865 | 0.28045289 |
| Sh3yl1 | 0.02761 | 0.51033049 |
| Scp2 | 0.1448 | -0.30514529 |
| Kdm3b | 0.00817 | -0.77489519 |
| Kdm3b | 0.30781 | 0.21292191 |
| Dgkh | 0.00447 | 0.67822547 |
| Unc45a | 0.00162 | -0.88825836 |
| Cdh8 | 0.01316 | -1.8007937 |
| Cdh8 | 0.0072 | -1.13787598 |
| LOC102642169 | 0.00341 | -1.48050714 |
| Rdh8 | 0.06449 | -1.46095236 |
| Bcl7b | 0.00144 | -2.07369097 |
| Defb42 | 0.01224 | -0.99902751 |
| Il12rb2 | 0.00157 | -1.34742246 |
| Tmem67 | 0.00144 | 1.27957479 |
| Usp35 | 0.00144 | -1.20555712 |
| 2010107G12Rik | 0.06588 | -0.93077 |
| Fam221a | 0.63957 | 0.246018 |
| Zim2 | 0.00144 | -1.27992547 |
| Teddm1a | 0.0046 | -1.12385295 |
| Gm10696 | 0.00276 | 1.46835567 |
| Arhgap44 | 0.03669 | -0.5025433 |
| Olfr1061 | 0.00144 | -1.42400773 |
| Mttp | 0.01125 | -0.66280214 |
| Gm9125 | 0.00158 | -1.22727468 |
| Zscan10 | 0.00144 | -1.28898079 |
| Dclk1 | 0.00144 | -1.30017714 |
| Dclk2 | 0.13327 | 0.4322014 |
| Alg14 | 0.93874 | -0.07493644 |
| Cdk3-ps | 0.00145 | -1.16044081 |
| Rnf11 | 0.00584 | -0.81999671 |
| Rnf11 | 0.01228 | 0.84468543 |
| Rnf10 | 0.36011 | -0.25228913 |
| Rnf17 | 0.00144 | -1.28116048 |
| Slc16a14 | 0.21472 | 0.39894166 |
| Heatr5b | 0.17969 | -0.34739577 |
| Ltbp2 | 0.00156 | -0.95186494 |
| Tex28 | 0.01737 | -0.7571703 |
| Grin2c | 0.31158 | 0.44467926 |
| Rpl35a | 0.00223 | -0.79857048 |
| Idi2 | 0.00145 | -1.22376446 |
| Hoxa13 | 0.00144 | -1.27165604 |
| Igsf9b | 0.07141 | -0.67411614 |
| Igsf9b | 0.01641 | -1.33921835 |
| Per1 | 0.00903 | -0.5937294 |
| Zer1 | 0.00863 | -0.78784884 |
| Sh3kbp1 | 0.21381 | -0.5311836 |
| Myo15 | 0.37497 | -0.55580822 |
| Defa25 | 0.0896 | -2.45409368 |
| Tcf25 | 0.00144 | -0.98997404 |
| Abcc6 | 0.00144 | -1.27165604 |
| Morf4l2 | 0.27765 | 0.19405606 |
| Morf4l2 | 0.07313 | 0.5655413 |
| Zap70 | 0.00592 | -1.08412076 |
| Ap2m1 | 0.01107 | -0.57840436 |
| Tmem68 | 0.80383 | 0.04289436 |
| Lrpprc | 0.00316 | 0.7538273 |
| Ptpmt1 | 0.05931 | -0.5249686 |
| LOC105243338 | 0.36549 | -0.61788943 |
| Pdhb | 0.18291 | -0.29838692 |
| Prb1 | 0.01578 | -0.61959076 |
| Stim1 | 0.00144 | -0.94907549 |
| Pitpnm1 | 0.00797 | -0.5717935 |
| Olfr1380 | 0.00933 | -0.90180771 |
| 5430402E10Rik | 0.56958 | -0.29806845 |
| Hsd3b4 | 0.00144 | -1.58851158 |
| Pex26 | 0.19137 | -0.27111826 |
| Rasgrp1 | 0.77205 | -0.12164699 |
| Sdcbp | 0.19084 | 0.3081899 |
| Hnrnpr | 0.00217 | 1.16747894 |
| Pnck | 0.12133 | -0.44160315 |
| Gm10701 | 0.00158 | -1.2446827 |
| Atp9b | 0.01542 | -0.60404291 |
| Pin4 | 0.67577 | 0.07489617 |
| Zfp146 | 0.34438 | 0.20168248 |
| Sumo2 | 0.37136 | 0.18890082 |
| Ttc23l | 0.98375 | -0.01053271 |
| Hivep3 | 0.19837 | -0.7714383 |
| Abcb1a | 0.00144 | 2.60485362 |
| Sntg1 | 0.00144 | -1.29085005 |
| Tmem97 | 0.00144 | -1.03608552 |
| Rpl39l | 0.39865 | 0.77889677 |
| Col4a5 | 0.00231 | 0.91431168 |
| Trappc2 | 0.05895 | 0.41058724 |
| Brip1 | 0.00144 | -1.24811894 |
| Gramd1b | 0.03346 | -0.48793158 |
| Hhip | 0.00144 | -1.25927895 |
| Fxr2 | 0.01571 | -0.56706422 |
| Pam | 0.1686 | -0.38672501 |
| Olfr533 | 0.00144 | -1.27730451 |
| 5730585A16Rik | 0.00144 | -1.30017714 |
| Epha7 | 0.28247 | -0.23197296 |
| Cops6 | 0.01364 | -0.60950951 |
| Rgs9 | 0.99832 | -0.00141948 |
| Rgs9 | 0.44667 | -0.54728252 |
| Tbx3 | 0.47935 | -0.15260737 |
| Tmub2 | 0.02903 | -0.48569407 |
| Mrto4 | 0.00353 | -0.84606565 |
| Crct1 | 0.00479 | -1.06284689 |
| Pbx3 | 0.0758 | 0.52524015 |
| Mycs | 0.19914 | -0.81544234 |
| Def6 | 0.00722 | -0.62914147 |
| Cbln1 | 0.00539 | -1.07389054 |
| AA645497 | 0.45121 | -0.16195589 |
| Prph | 0.38093 | -0.41995571 |
| Klrb1 | 0.00144 | -1.50896782 |
| Synpo2l | 0.04273 | -0.48715055 |
| Cadps | 0.24153 | -0.57184887 |
| Rpgr | 0.00144 | 2.48262083 |
| Rpgr | 0.31535 | 0.50887119 |
| Wipi1 | 0.54 | -0.17465251 |
| Krtap1-5 | 0.04838 | -1.23215694 |
| Rasa3 | 0.04066 | -0.37613981 |
| Shc4 | 0.78502 | -0.17309131 |
| Nfkb1 | 0.55271 | -0.09112483 |
| Ccrn4l | 0.02443 | 1.00705596 |
| Gfra2 | 0.00677 | -0.66972078 |
| Anks1b | 0.01042 | -1.37182574 |
| Anks1b | 0.00145 | -1.70065414 |
| Gpatch2l | 0.21553 | 0.21738275 |
| Vmn1r173 | 0.80873 | -0.09450916 |
| Gm9325 | 0.00144 | -1.29049509 |
| Ppm1k | 0.01229 | -0.64134238 |
| Olfr1505 | 0.00856 | -1.83931018 |
| Btbd9 | 0.00145 | -0.9918757 |
| Telo2 | 0.00149 | -0.92496943 |
| Gtpbp6 | 0.00404 | -0.71721455 |
| Kat6a | 0.00914 | 0.71471219 |
| Agpat9 | 0.99517 | -0.0017715 |
| Cradd | 0.96019 | -0.02310539 |
| Cast | 0.00144 | 1.3032518 |
| Tle1 | 0.03667 | -0.4600766 |
| Plekhh1 | 0.68145 | -0.14667447 |
| Agbl3 | 0.00163 | 1.78714263 |
| Agbl3 | 0.00383 | -1.13029182 |
| Gm10568 | 0.00144 | -1.30017714 |
| Aes | 0.00144 | -1.05369525 |
| Cyp2j13 | 0.04959 | -0.7949793 |
| Cbr4 | 0.43353 | 0.15140781 |
| Rab33b | 0.60791 | -0.09649075 |
| Cyp2d13 | 0.16804 | -0.56161429 |
| Fhit | 0.56513 | -0.24137294 |
| Btf3 | 0.03199 | -0.40820036 |
| Smagp | 0.00144 | -1.15295444 |
| Vmn1r139 | 0.00144 | -1.43671944 |
| Vmn1r132 | 0.03165 | -1.51721302 |
| Pcgf2 | 0.61666 | -0.16580421 |
| Pla2g2c | 0.00144 | -1.49514228 |
| Olfr1280 | 0.0247 | -0.96581009 |
| Dpp10 | 0.00144 | -1.29051721 |
| Jmjd1c | 0.00144 | 1.16389425 |
| Gm33125 | 0.01866 | -0.95010236 |
| Lbhd1 | 0.01092 | -0.62201822 |
| Tdgf1 | 0.59622 | -0.28189576 |
| Nfatc2 | 0.49051 | -0.13014138 |
| Srfbp1 | 0.00176 | 1.21356205 |
| Srfbp1 | 0.50436 | 0.19411562 |
| Nr3c1 | 0.54157 | 0.12224859 |
| Kctd20 | 0.06513 | -0.43671408 |
| Kctd10 | 0.22693 | -0.30562185 |
| Tmem198 | 0.00978 | -0.58354869 |
| Klc3 | 0.95771 | 0.02051822 |
| Foxc1 | 0.23803 | 0.41471368 |
| Tmem151a | 0.18744 | -0.30873114 |
| Nkd1 | 0.0022 | -1.07062996 |
| Polr2i | 0.00144 | -1.13055991 |
| Adamts13 | 0.03055 | -1.01865874 |
| Bex4 | 0.34584 | -0.69671815 |
| Tas2r109 | 0.79746 | -0.07173767 |
| Flcn | 0.06854 | -1.45525557 |
| Flcn | 0.11788 | -0.47884071 |
| Plb1 | 0.00158 | -1.04785894 |
| Sf3b3 | 0.84327 | -0.0381046 |
| Med16 | 0.01185 | -0.63514006 |
| Ccnk | 0.16534 | -0.30269116 |
| Olfr993 | 0.00144 | -1.27165604 |
| Tmem254c | 0.01499 | -0.60553547 |
| 1700123K08Rik | 0.39593 | -0.46613706 |
| Hunk | 0.51078 | 0.15098386 |
| Gm5069 | 0.88956 | 0.09269917 |
| Dnah7b | 0.39097 | -1.43646242 |
| Nell1 | 0.00547 | -1.10562496 |
| Zmynd15 | 0.08051 | -0.64062216 |
| Hbb-y | 0.00144 | -1.31495282 |
| Efcab1 | 0.10894 | -2.81380241 |
| Antxr1 | 0.00144 | -1.25118919 |
| Ugt2b35 | 0.00144 | -1.27165604 |
| Gm5069 | 0.2631 | -0.39115353 |
| Fam19a5 | 0.24985 | -0.27177434 |
| Ppp1r9b | 0.00149 | -0.9340852 |
| Gm10437 | 0.00145 | -1.26219879 |
| Zfp85 | 0.02837 | -1.42373611 |
| Ncaph | 0.79352 | -0.0629638 |
| Plxnb2 | 0.0647 | -0.44474955 |
| Stbd1 | 0.08663 | 0.37823329 |
| Adam10 | 0.01084 | 0.88918162 |
| Lamb3 | 0.54412 | 0.17582709 |
| Gm4632 | 0.04596 | -0.83560893 |
| Gm5486 | 0.00144 | -1.28116048 |
| Ston1 | 0.05253 | 0.44678014 |
| Irx3 | 0.07719 | -0.34258819 |
| LOC102637947 | 0.00144 | -1.56304308 |
| Stra13 | 0.21043 | -0.31550687 |
| Ppp1r3b | 0.24446 | -0.2533843 |
| Ankef1 | 0.24239 | -0.59100254 |
| Trim39 | 0.08613 | -0.5232246 |
| Adgrb2 | 0.01693 | -0.77935631 |
| Rpl11 | 0.002 | -0.87562808 |
| Lpar6 | 0.04052 | 0.77224836 |
| Thoc7 | 0.05033 | -1.09460317 |
| Gprin1 | 0.0037 | -1.11214255 |
| Arhgdib | 0.0125 | -0.60251695 |
| Limk1 | 0.02111 | -0.61472698 |
| Gsr | 0.01837 | -0.54204595 |
| Sntb1 | 0.6084 | -0.13024924 |
| Mef2d | 0.41837 | -0.29194962 |
| Zfp40 | 0.00376 | 2.12072285 |
| Rnaseh2a | 0.00248 | -0.84513617 |
| Rtbdn | 0.23108 | -0.52993316 |
| Mad2l2 | 0.00964 | -0.74865264 |
| Prss32 | 0.11474 | -0.65565707 |
| Xaf1 | 0.4536 | 0.29116967 |
| Xaf1 | 0.61911 | -0.09234253 |
| LOC102643255 | 0.00198 | -1.11811652 |
| Fdps | 0.00848 | -0.87172802 |
| Serpina3i | 0.02759 | -1.1047111 |
| Cyp2s1 | 0.1726 | -0.89615923 |
| Cyp2u1 | 0.03237 | -0.49118762 |
| 9930105H17Rik | 0.52878 | 0.12098093 |
| Iqcc | 0.10039 | 0.3690402 |
| Kbtbd13 | 0.11635 | -0.67201528 |
| Cdc14b | 0.30849 | -0.1982125 |
| LOC547349 | 0.00201 | -1.10986124 |
| Glmn | 0.00819 | 1.91637297 |
| Trpc6 | 0.06474 | -1.8829346 |
| Vmn2r75 | 0.00144 | -1.31495282 |
| Actr3b | 0.95362 | -0.01758238 |
| Ighv1-33 | 0.00144 | -1.39736617 |
| Bpifc | 0.29947 | -0.20456283 |
| Bpifc | 0.04244 | -0.43721202 |
| Gemin4 | 0.7866 | -0.04719092 |
| Vmn2r39 | 0.00145 | -1.2388121 |
| Vmn2r43 | 0.27971 | 0.28935917 |
| Ctu2 | 0.00197 | -0.87970314 |
| Hoxc5 | 0.10023 | -2.58056029 |
| Hoxc6 | 0.00144 | -1.27558215 |
| Krt83 | 0.00145 | -1.26219879 |
| Krt81 | 0.00854 | -2.10580637 |
| Gstm1 | 0.23983 | -0.36639123 |
| Scrn2 | 0.00144 | -1.16243833 |
| Olfr1328 | 0.00144 | -1.28116048 |
| 4930502E18Rik | 0.00145 | -1.2388121 |
| Srek1 | 0.05611 | -0.3833659 |
| Itgb2l | 0.00144 | -1.26934956 |
| Polr2h | 0.03353 | -0.49735123 |
| Eif3h | 0.00897 | -0.59791975 |
| Ren1 | 0.02787 | -0.83021526 |
| Gm9839 | 0.4455 | -0.15446684 |
| Pdzd4 | 0.00282 | -0.86543413 |
| Pdzd7 | 0.00144 | -1.48224879 |
| Podxl | 0.00145 | -1.30176224 |
| Fez2 | 0.09885 | -0.29274971 |
| Cxcl12 | 0.07551 | -0.506861 |
| Plek | 0.07646 | 0.43690621 |
| Il4ra | 0.06683 | -0.77376651 |
| Ppdpf | 0.05174 | -0.53558068 |
| Wfdc6b | 0.00325 | 1.64311315 |
| Slfn14 | 0.0039 | -1.10754975 |
| Prl3c1 | 0.00144 | -1.28116048 |
| Erh | 0.14366 | -0.282774 |
| Epn2 | 0.01529 | -0.48405885 |
| Nme5 | 0.06354 | 0.40308894 |
| Apoa2 | 0.00438 | -1.18852843 |
| Pkd1l1 | 0.00144 | -1.59314691 |
| Zfp329 | 0.00537 | 1.37597606 |
| Ccl25 | 0.07108 | -2.24243389 |
| Kynu | 0.52719 | -0.7233144 |
| Cldn34c1 | 0.20099 | -2.42631406 |
| Rwdd1 | 0.14978 | -0.23334386 |
| Gm9999 | 0.00144 | -1.28116048 |
| Hs6st3 | 0.00145 | -1.2388121 |
| Opn1mw | 0.20058 | -0.43975677 |
| Zfp939 | 0.72567 | 0.07511431 |
| Sp2 | 0.01585 | -0.73136053 |
| Crebbp | 0.50151 | -0.12521912 |
| Ubb | 0.01313 | -0.68076734 |
| Uprt | 0.00465 | -0.70972488 |
| D3Ertd751e | 0.02233 | -0.51401039 |
| Atp2a3 | 0.00579 | -1.05528324 |
| Eme2 | 0.01218 | -0.80422586 |
| Abca17 | 0.97086 | 0.02631532 |
| Gapdh | 0.94614 | -0.04544643 |
| D930030D11Rik | 0.00144 | -1.30713006 |
| Gabrg2 | 0.00144 | -1.30017714 |
| Alg2 | 0.8159 | 0.03760556 |
| AA792892 | 0.00144 | -2.24215599 |
| Setdb1 | 0.0089 | 0.66761332 |
| Hcfc1 | 0.11 | -0.35818945 |
| Bud31 | 0.02761 | -0.42021914 |
| 1700019G17Rik | 0.17236 | 0.48300114 |
| Eif4e | 0.31177 | -0.80039745 |
| Gm15085 | 0.00144 | -1.3348153 |
| Orc3 | 0.01368 | 0.53629046 |
| Ndufb4 | 0.01992 | -0.89224217 |
| Gm6525 | 0.00699 | -0.62692108 |
| 4933424M12Rik | 0.05813 | -0.74744057 |
| Trnau1ap | 0.00472 | -0.76351045 |
| Kcnd3 | 0.00144 | -1.36454935 |
| Kcnd1 | 0.01208 | -0.66164413 |
| Atp9a | 0.00144 | -1.11887727 |
| Klhl18 | 0.26908 | -0.21113113 |
| Serpinb6e | 0.09526 | -0.90933474 |
| 9430091E24Rik | 0.07423 | -0.65765833 |
| Naa60 | 0.36809 | -0.26277722 |
| Mctp2 | 0.00144 | -1.29085005 |
| Ccdc66 | 0.00144 | 1.78269072 |
| Gm5640 | 0.14642 | -0.676561 |
| Wdr17 | 0.00144 | -1.3230041 |
| Dmrtc1a | 0.00144 | -1.25118919 |
| Olfr554 | 0.03888 | -4.10058327 |
| Sh3tc1 | 0.00144 | -1.25133839 |
| Syt6 | 0.69132 | -0.098059 |
| Fbxl5 | 0.05555 | -0.7775065 |
| Fbxl5 | 0.01175 | 1.00130304 |
| Fbxl2 | 0.06617 | 0.59139142 |
| Spc25 | 0.53636 | -0.23570572 |
| Pnkp | 0.01141 | -0.80396207 |
| Taar7d | 0.00462 | -1.14485232 |
| Spsb3 | 0.00352 | -1.03541723 |
| Xlr3b | 0.99192 | 0.01936014 |
| Map7d1 | 0.01246 | -0.77106633 |
| Slc47a2 | 0.44985 | -0.33915307 |
| Slc5a12 | 0.01606 | -1.62216614 |
| Gm3763 | 0.00236 | -1.13100373 |
| 6530402F18Rik | 0.0371 | -0.85024893 |
| Ddx58 | 0.13937 | 0.37428064 |
| Gm1673 | 0.00144 | -1.11379964 |
| Il33 | 0.05942 | 0.80160781 |
| Gm3716 | 0.00144 | -1.27165604 |
| Gm30454 | 0.00149 | -1.25408 |
| Gm3014 | 0.04649 | 1.54387708 |
| Art2b | 0.00144 | -1.35320495 |
| Gm5549 | 0.00144 | -1.29746368 |
| Ndufs6 | 0.00144 | -1.08306698 |
| Tfap2a | 0.02203 | -0.972494 |
| Rpa1 | 0.03747 | -0.41135708 |
| Slc35e4 | 0.00204 | -0.9915688 |
| Slc23a3 | 0.68973 | 0.13290112 |
| Adgrb3 | 0.009 | -1.1398278 |
| Krt28 | 0.62498 | 0.1247985 |
| Fus | 0.00144 | 1.58951162 |
| Btla | 0.00554 | 0.89068714 |
| LOC102633627 | 0.01832 | 0.78448779 |
| Dnttip1 | 0.21142 | -0.33636237 |
| Dnttip1 | 0.51288 | -0.13000221 |
| Hsd17b7 | 0.19145 | -0.517104 |
| 4932411N23Rik | 0.7763 | 0.14228764 |
| Arhgef6 | 0.01802 | 0.54291125 |
| Megf10 | 0.54684 | 0.34904558 |
| Smarca5-ps | 0.13501 | 0.5834787 |
| Lcn12 | 0.32277 | -0.20417503 |
| Cd164l2 | 0.04052 | -0.88235287 |
| Olfr854 | 0.1153 | -0.8941726 |
| Cryab | 0.01551 | -0.77449905 |
| Gm9731 | 0.09545 | -0.45803418 |
| Kctd14 | 0.03727 | -0.57441785 |
| Cnot8 | 0.84428 | -0.03519136 |
| Gdpd2 | 0.02403 | -0.86785939 |
| Ppwd1 | 0.00375 | -0.86503486 |
| Klrb1b | 0.00942 | -1.07292168 |
| 1110051M20Rik | 0.38545 | 0.59114793 |
| Pla2g4b | 0.40422 | 0.17448478 |
| Mlf2 | 0.0654 | -0.48540038 |
| Mlf2 | 0.03086 | -0.41269257 |
| Gm16401 | 0.00144 | -1.28116048 |
| Sbk3 | 0.17074 | -0.74169797 |
| Apol7a | 0.00144 | -1.50188002 |
| Olfr631 | 0.53596 | -0.25473084 |
| 4932418E24Rik | 0.00144 | -1.26560808 |
| Tnks | 0.39798 | -0.17367318 |
| Elac1 | 0.32642 | 0.26255465 |
| Tprkb | 0.74638 | -0.197989 |
| Dysf | 0.00498 | -0.76700928 |
| 1700001G17Rik | 0.00509 | -2.12002539 |
| Pign | 0.09668 | 0.35522213 |
| B230118H07Rik | 0.0121 | 0.77321541 |
| Rangrf | 0.01818 | -0.73578847 |
| Dnajb6 | 0.1121 | -0.26736283 |
| Golga4 | 0.00409 | 0.88653793 |
| Gm3519 | 0.06618 | -0.92904723 |
| Slc14a2 | 0.9338 | -0.03286368 |
| 1700015G11Rik | 0.00613 | -1.73387795 |
| LOC102634968 | 0.27978 | -0.68376017 |
| Lrrc16a | 0.01318 | -0.69466839 |
| LOC102642410 | 0.92902 | 0.02834537 |
| Scimp | 0.04474 | -0.82443142 |
| Guca1b | 0.12816 | -0.48747359 |
| Mib2 | 0.00906 | -0.6456335 |
| Nrxn3 | 0.23739 | -0.66666667 |
| Ndfip1 | 0.4126 | 0.14791289 |
| Plekho2 | 0.00587 | -0.80462792 |
| 1110057P08Rik | 0.00938 | -1.01588975 |
| Rps6 | 0.2936 | -0.19230653 |
| Rhox7a | 0.00144 | -1.28898079 |
| Gtf3c6 | 0.60789 | 0.15177045 |
| Npr2 | 0.69179 | -0.08036745 |
| Odam | 0.00144 | -1.27454685 |
| 5530400C23Rik | 0.00144 | -1.25118919 |
| Aktip | 0.75177 | 0.07472032 |
| Lcn5 | 0.22556 | -0.25769737 |
| Gm30784 | 0.00144 | -1.33632959 |
| Tsen54 | 0.0017 | -0.9602089 |
| Sycn | 0.00157 | -1.42492896 |
| Yae1d1 | 0.01116 | 0.59658437 |
| Hcfc1 | 0.00588 | -1.3356398 |
| Mbd5 | 0.18935 | 0.49244662 |
| Mbd5 | 0.00163 | 1.65515419 |
| Hes3 | 0.00144 | -1.32766452 |
| Gm11595 | 0.00594 | -0.86604434 |
| Gm11595 | 0.54357 | -0.68493171 |
| Zfp689 | 0.00569 | -0.874402 |
| Vmn1r174 | 0.00149 | -1.22582153 |
| Vmn1r168 | 0.68597 | 0.19995611 |
| Abcb10 | 0.47612 | -0.14649604 |
| LOC100503338 | 0.00618 | -1.90531304 |
| 4933404G15Rik | 0.02498 | -1.17015626 |
| Ptma | 0.01882 | -0.99384211 |
| Vps16 | 0.03793 | -0.41087829 |
| Gabra3 | 0.12267 | 0.61213804 |
| Cenpq | 0.00458 | -0.79560857 |
| Gm40787 | 0.00144 | -1.27165604 |
| Mthfs | 0.00144 | -1.30626366 |
| Klhdc7a | 0.35791 | 0.15622189 |
| Klhdc7a | 0.10322 | -1.009302 |
| Nxpe3 | 0.51159 | -0.15645128 |
| Upf3b | 0.00144 | 2.04655296 |
| E430025E21Rik | 0.96926 | -0.00695472 |
| G3bp1 | 0.0627 | 0.36844537 |
| Brwd3 | 0.01128 | -1.15648064 |
| Vsig10 | 0.52575 | 0.12323155 |
| Klhdc7b | 0.00322 | -0.93467497 |
| Myoz3 | 0.33208 | -0.27565208 |
| Olfr1396 | 0.21704 | 0.528841 |
| Clec12b | 0.00434 | 1.14083039 |
| Gm36864 | 0.28824 | -0.87431012 |
| Chchd7 | 0.0387 | 0.576578 |
| Pgap1 | 0.02095 | 1.37894077 |
| Lctl | 0.02739 | 0.77023341 |
| Folh1 | 0.00144 | -1.39594897 |
| Creg1 | 0.00342 | -1.01730351 |
| Mtmr14 | 0.0354 | -0.43407555 |
| Prh1 | 0.00198 | -0.88553614 |
| Gm6109 | 0.01304 | -0.50794715 |
| Rps16 | 0.00144 | -1.17728384 |
| Prl3c1 | 0.07296 | -1.03222143 |
| Gm4861 | 0.05601 | 0.79626335 |
| Oxr1 | 0.00144 | 1.38217527 |
| Ampd2 | 0.0016 | -1.05471114 |
| Stfa1 | 0.00144 | -1.29085005 |
| Slc26a7 | 0.10973 | -2.73948072 |
| Ppfia4 | 0.00763 | -0.78888729 |
| Sub1 | 0.00144 | -1.32766452 |
| Nckap1 | 0.0373 | 0.57758342 |
| Stx5a | 0.99 | -0.00218862 |
| Syk | 0.99295 | 0.00189458 |
| Trem2 | 0.00144 | -1.42353083 |
| Snapc3 | 0.08264 | 0.57565211 |
| Ifi205 | 0.00222 | 1.9004617 |
| Als2cl | 0.91942 | -0.02421792 |
| Fam170a | 0.00144 | -1.26560808 |
| Fam170a | 0.00792 | -1.16599495 |
| Adam11 | 0.01722 | -0.48806497 |
| Gpr179 | 0.00144 | -1.27165604 |
| Gm904 | 0.01806 | -1.68796923 |
| 6030469F06Rik | 0.01018 | -1.11996532 |
| Cyfip2 | 0.11194 | 0.36249359 |
| Fam131c | 0.00144 | -1.30017714 |
| Ttll2 | 0.00144 | -1.31495282 |
| H2-Ab1 | 0.00369 | -1.10846896 |
| Sec23b | 0.62942 | 0.08378748 |
| Kdm7a | 0.64746 | 0.10113668 |
| Cyp2c67 | 0.05929 | -0.78745627 |
| Olfr652 | 0.0141 | -1.25096905 |
| Wapal | 0.00177 | -1.71124756 |
| Rnf26 | 0.00144 | -1.12233384 |
| Gcnt7 | 0.00144 | -1.30713006 |
| Chil5 | 0.1065 | -0.72578778 |
| Cplx2 | 0.21991 | 0.51614657 |
| Gm34396 | 0.06008 | -0.34424192 |
| Bbx | 0.07367 | -0.77738533 |
| Btnl4 | 0.04588 | -1.13834583 |
| Zic2 | 0.00167 | -1.69949026 |
| Fam19a2 | 0.14838 | -0.37620753 |
| a | 0.00144 | -1.7055637 |
| Gpbp1l1 | 0.74487 | -0.0572093 |
| Gm14409 | 0.00321 | 1.93657685 |
| Rpl21 | 0.04975 | -0.35591095 |
| Ugt1a6b | 0.37424 | -0.19612288 |
| Il1rn | 0.01371 | -2.27356464 |
| BC055111 | 0.80892 | -0.34784151 |
| Trim21 | 0.93604 | -0.02501376 |
| Trim41 | 0.03126 | -0.42656137 |
| Trim61 | 0.00144 | -1.29864962 |
| Plac8 | 0.00327 | -1.52621959 |
| Hba-a1 | 0.09199 | -0.64221714 |
| Hba-a2 | 0.00594 | -1.10452308 |
| Klhl24 | 0.01986 | 0.60843209 |
| Plac1 | 0.00144 | -1.28116048 |
| Ccnt1 | 0.11464 | 0.35574079 |
| Bad | 0.00144 | -1.12497998 |
| Prima1 | 0.04544 | -1.54532578 |
| Cxcr6 | 0.91703 | 0.04206686 |
| Rab8a | 0.20799 | -0.30124574 |
| Dnah10 | 0.00144 | -1.28898079 |
| Mff | 0.05163 | 0.6208834 |
| Vmn2r103 | 0.00149 | -1.28183002 |
| Gm4925 | 0.02022 | -1.01610076 |
| Anxa7 | 0.84136 | 0.03217258 |
| Cryl1 | 0.02967 | 0.50851675 |
| Uqcr11 | 0.00144 | -1.2482731 |
| Plagl1 | 0.00144 | 2.05949867 |
| Plagl1 | 0.29947 | 0.19630924 |
| Cd68 | 0.02469 | -0.54133039 |
| Otop3 | 0.01585 | -0.89846216 |
| Erich6 | 0.13487 | -0.3836762 |
| Pygm | 0.24448 | -0.28722802 |
| Gm15217 | 0.22311 | -1.34138147 |
| Fbxw11 | 0.40831 | 0.16549191 |
| Twistnb | 0.06203 | -0.49272765 |
| Akt3 | 0.10128 | 0.46208725 |
| LOC100862444 | 0.0016 | -1.0338228 |
| Pcdhga9 | 0.0249 | -0.43971876 |
| Zfand3 | 0.00202 | -0.88879258 |
| Adra1d | 0.76409 | -0.17220511 |
| Dcc | 0.00144 | 3.50376738 |
| Rcan2 | 0.32612 | 0.21067535 |
| Kcnt1 | 0.88771 | -0.07012474 |
| Ush2a | 0.00144 | -1.33632959 |
| Tmem182 | 0.05168 | -0.37478484 |
| Vax2 | 0.79595 | -0.0989757 |
| Ypel4 | 0.24024 | -0.69143126 |
| Ypel3 | 0.00722 | -0.66714319 |
| D030028A08Rik | 0.00145 | -1.22376446 |
| A930028N01Rik | 0.16434 | 0.42019041 |
| Tmem194 | 0.04799 | 0.36645273 |
| Ly6c1 | 0.00152 | -0.93838884 |
| Svs3a | 0.00149 | -1.2703832 |
| Kcnj11 | 0.94427 | 0.03754466 |
| Dct | 0.0086 | -1.0296515 |
| Bnip3l | 0.20498 | 0.19983035 |
| Plcb2 | 0.06868 | -0.52172698 |
| Prkca | 0.00705 | 0.63996093 |
| Vmn1r131 | 0.023 | -1.36068408 |
| Gsta3 | 0.09895 | 0.48283213 |
| Defa3 | 0.02253 | -0.85939466 |
| Defa2 | 0.01158 | -1.69619797 |
| Defa2 | 0.07653 | -1.05096911 |
| Gm17778 | 0.01351 | -0.87889929 |
| Gm12472 | 0.02787 | 1.32954954 |
| Ctsk | 0.10101 | -0.27642696 |
| Tes | 0.11974 | -0.83260352 |
| Tes | 0.06293 | 0.53380971 |
| Gm11974 | 0.14839 | -0.34308831 |
| Cd72 | 0.01482 | -0.83776327 |
| LOC102642579 | 0.01486 | -1.28554784 |
| Vps53 | 0.46095 | -0.20361371 |
| Ppp1cc | 0.57822 | -0.08736785 |
| Gm32139 | 0.01593 | -0.7716251 |
| Gm6981 | 0.2385 | -0.40402078 |
| 4930429B21Rik | 0.1382 | -0.67487695 |
| Olfr575 | 0.00144 | -1.27165604 |
| Pou3f1 | 0.30519 | -0.36103021 |
| Mettl3 | 0.93216 | 0.01525635 |
| Atg4d | 0.00882 | -0.92305668 |
| Map3k1 | 0.03102 | 0.45472263 |
| Usp16 | 0.00144 | 1.16429779 |
| Cep295 | 0.41872 | -0.21127084 |
| Pnpla2 | 0.01082 | -0.79295423 |
| Gm13272 | 0.02912 | -1.12195511 |
| Selenbp1 | 0.03961 | -0.71079988 |
| Pigr | 0.00144 | 3.01546226 |
| Nlgn2 | 0.03166 | -0.40956067 |
| Lrrc59 | 0.18222 | -0.24509345 |
| Zdhhc9 | 0.00331 | -0.83845124 |
| Gm10789 | 0.05981 | -2.57218046 |
| Egln2 | 0.00144 | -1.02905373 |
| Tgfbr1 | 0.11767 | -0.60790527 |
| Tgfbr1 | 0.08527 | 0.55130601 |
| Cabp1 | 0.08833 | 0.50151297 |
| Olfr639 | 0.00482 | -1.56793291 |
| Sorbs2 | 0.39075 | -0.20437256 |
| Gm6579 | 0.67911 | -0.08859079 |
| Slc16a13 | 0.06258 | -0.35048952 |
| Gm11565 | 0.79261 | -0.32997682 |
| Rps15a | 0.02378 | -0.52628556 |
| Kif9 | 0.53231 | -0.32764216 |
| Arl13b | 0.00144 | 2.21199298 |
| Styxl1 | 0.55229 | -0.43072692 |
| Gm4470 | 0.09595 | 0.54827559 |
| Gdf15 | 0.00144 | -2.56914622 |
| Pih1d2 | 0.01919 | 0.99595031 |
| Cd300lh | 0.06704 | -2.10583525 |
| Tmem220 | 0.36856 | 0.29418847 |
| Fer1l6 | 0.43767 | -0.21012111 |
| Tenm4 | 0.00144 | -1.21758364 |
| Ube2w | 0.00451 | -0.73670507 |
| Trpt1 | 0.02396 | -0.8354053 |
| Gm20764 | 0.09596 | -0.28329308 |
| Dnajc17 | 0.23798 | -0.28148984 |
| Olfr1013 | 0.05474 | -0.78406451 |
| Kdm5b | 0.57826 | 0.28960537 |
| Kdm5b | 0.58935 | -0.0860474 |
| Ybx3 | 0.07418 | -0.33294683 |
| Acox1 | 0.03566 | -0.4010127 |
| Gm14221 | 0.00149 | -1.28183002 |
| Polg | 0.57533 | -0.19453462 |
| Larp1 | 0.58338 | -0.10064413 |
| Thsd7a | 0.02161 | -0.87297327 |
| Ubn1 | 0.64067 | -0.07470017 |
| Lrrc23 | 0.05198 | -0.46959152 |
| Lrrc43 | 0.00144 | -1.31495282 |
| Ccl1 | 0.00144 | -1.27165604 |
| Cd209e | 0.00206 | -1.19897838 |
| Cntn1 | 0.55031 | 0.31999477 |
| Srbd1 | 0.00811 | 0.62701955 |
| Dnase1l1 | 0.07912 | -0.32314024 |
| Tmem80 | 0.23831 | 0.21721157 |
| Tjp3 | 0.01408 | -0.81586541 |
| Med7 | 0.00144 | 1.12090494 |
| Slc2a6 | 0.00978 | -1.28010492 |
| Slc2a6 | 0.06919 | -0.62845248 |
| Txn2 | 0.02933 | -0.603563 |
| Tas2r120 | 0.00145 | -1.2388121 |
| Vcan | 0.61944 | -0.08652673 |
| P2ry14 | 0.10671 | 0.41377848 |
| Myot | 0.00145 | 1.53425428 |
| Bcat2 | 0.78325 | -0.07398062 |
| Pet2 | 0.00704 | -1.19266991 |
| Epb4.1l3 | 0.03434 | 0.45737258 |
| Fam217a | 0.57141 | -0.2361926 |
| Pafah1b3 | 0.00164 | -0.8653786 |
| Hvcn1 | 0.00305 | -0.79277814 |
| 2810408A11Rik | 0.01886 | -0.51585751 |
| Prune | 0.20698 | 0.58549113 |
| Trmt5 | 0.02374 | 0.50334262 |
| Fbxw25 | 0.00291 | -1.34723098 |
| Unc5cl | 0.03096 | -0.87106872 |
| Dcaf12l2 | 0.77651 | 0.16016688 |
| Helz2 | 0.82985 | 0.04646747 |
| Pde7b | 0.06076 | -0.62193035 |
| Atp4b | 0.07503 | -0.7158292 |
| Map7 | 0.68469 | 0.09298968 |
| Cst10 | 0.10722 | -0.78312176 |
| Gm2933 | 0.42139 | -0.69797862 |
| Slc35g1 | 0.9002 | -0.03973351 |
| Gm10697 | 0.00144 | -1.27165604 |
| Mef2b | 0.05641 | -1.55443392 |
| Dach1 | 0.00144 | 1.60053397 |
| Top3b | 0.62988 | -0.17163949 |
| Asns | 0.00484 | -0.90808488 |
| Dlgap1 | 0.23592 | -1.97476519 |
| Zfp646 | 0.00436 | -0.83642477 |
| Hspg2 | 0.00417 | -0.79063766 |
| Hnf4a | 0.00144 | -1.29085005 |
| Vmn2r18 | 0.00145 | -1.22376446 |
| Tom1l1 | 0.0494 | 0.66184579 |
| Tmem222 | 0.00447 | -0.88653646 |
| Fmo5 | 0.34812 | 0.15295203 |
| Gm9886 | 0.00144 | -1.30713006 |
| 5031425E22Rik | 0.1292 | 0.64615644 |
| Rltpr | 0.01691 | -0.69178308 |
| Wbscr25 | 0.50843 | 0.35579377 |
| Etv4 | 0.73007 | -0.16583322 |
| Cep135 | 0.00153 | 1.23037383 |
| 4933406J08Rik | 0.022 | -1.05452194 |
| Ces2e | 0.00144 | -1.37100983 |
| Sbno2 | 0.0019 | -0.86365687 |
| Lrrfip1 | 0.00144 | 1.98063763 |
| Lrrfip1 | 0.00144 | 1.0673694 |
| Chat | 0.72073 | 0.12539552 |
| Slc16a3 | 0.12695 | -0.45983474 |
| Hhat | 0.00513 | -0.75004786 |
| BC080695 | 0.18405 | -0.33731564 |
| Olfr898 | 0.67587 | 0.14284725 |
| Olfr998 | 0.00144 | -1.80255849 |
| Olfr598 | 0.00144 | -1.20555712 |
| Ins1 | 0.02362 | -1.3205202 |
| Rbm42 | 0.00237 | -0.82589527 |
| Rbm47 | 0.02106 | -0.49487066 |
| Vmn2r11 | 0.00145 | -1.24191372 |
| Dnpep | 0.00456 | -0.95048341 |
| Nagk | 0.0465 | -0.54887175 |
| Slc4a4 | 0.44022 | 0.30534035 |
| Usp51 | 0.00144 | -1.26934956 |
| Gm6899 | 0.01822 | -0.94912986 |
| Gm10324 | 0.00144 | 1.07716281 |
| 2410141K09Rik | 0.00446 | 1.40170481 |
| Gm13119 | 0.02888 | -1.1520689 |
| Cd164l2 | 0.05606 | -1.9674093 |
| LOC102642966 | 0.00144 | -1.26560808 |
| Ren2 | 0.00464 | -0.91590965 |
| Cdkl5 | 0.09374 | -1.17111094 |
| Clec2e | 0.0352 | 1.46577029 |
| Oas1b | 0.46399 | 0.2108476 |
| Pom121 | 0.00258 | -0.84506694 |
| Tcaf3 | 0.00145 | -1.2388121 |
| Cox16 | 0.90786 | -0.0558218 |
| Gm32474 | 0.04583 | -0.71890283 |
| Imp4 | 0.15121 | -0.30304799 |
| Spata7 | 0.23105 | 0.28091465 |
| Gm10531 | 0.00144 | -1.56060801 |
| Gm5538 | 0.00145 | -1.26219879 |
| 4930589L23Rik | 0.22736 | -0.38774345 |
| Ankrd29 | 0.00437 | 1.41186397 |
| Olfr1347 | 0.26938 | -0.20983237 |
| Col17a1 | 0.00276 | -1.23532597 |
| DXBay18 | 0.60256 | -0.29521486 |
| 4833422C13Rik | 0.02117 | 0.84857244 |
| Higd1a | 0.01252 | -0.62976839 |
| Nek6 | 0.05342 | -0.35715198 |
| Lrp8os2 | 0.27564 | -0.65497251 |
| Itga6 | 0.38966 | -0.1337025 |
| Adam12 | 0.03311 | -0.50413206 |
| Stoml2 | 0.02961 | -0.54745956 |
| Mobp | 0.00149 | -1.22582153 |
| Olfr965 | 0.53971 | -0.28998353 |
| Nsmaf | 0.24329 | 0.45867306 |
| Muc19 | 0.38217 | -0.24058174 |
| Muc19 | 0.25629 | -0.65960193 |
| Rbm33 | 0.18919 | 0.30072517 |
| Brd2 | 0.00625 | -0.6337651 |
| Arf2 | 0.03632 | -0.41177621 |
| Ncln | 0.00144 | -1.14670382 |
| Tas2r106 | 0.00144 | -1.30205933 |
| Slc27a3 | 0.00903 | -0.94098561 |
| Ar | 0.88968 | 0.0290221 |
| Gm7550 | 0.00613 | -1.05687292 |
| Map6 | 0.00336 | -0.84527051 |
| Hr | 0.04202 | -0.73941227 |
| Parp6 | 0.65712 | -0.07101455 |
| LOC102637808 | 0.00144 | -1.30761071 |
| Tgm5 | 0.00948 | -0.73855777 |
| Gm10729 | 0.00144 | -1.27165604 |
| LOC545005 | 0.00144 | 2.62614335 |
| Nbeal2 | 0.00479 | -0.70917013 |
| Gm9802 | 0.00267 | -1.24518035 |
| Got1 | 0.54852 | -0.15266376 |
| Slit1 | 0.00732 | -0.78567553 |
| Zdhhc17 | 0.15328 | -0.90054661 |
| Rps29 | 0.00144 | -1.1277205 |
| Nipsnap3b | 0.92355 | -0.01837662 |
| Harbi1 | 0.09721 | -0.60912718 |
| Gm3081 | 0.00144 | 2.37031448 |
| Vmn2r113 | 0.81894 | 0.08562739 |
| Mospd1 | 0.03741 | -0.46923117 |
| Thrap3 | 0.37119 | 0.14504417 |
| Arrdc1 | 0.61317 | -0.16946327 |
| Arrdc1 | 0.14529 | -0.41725378 |
| Bcl11a | 0.01483 | -0.87111017 |
| Bcl11a | 0.01542 | -1.4913104 |
| Tcof1 | 0.00144 | -1.21988456 |
| Ccr4 | 0.00144 | -1.3826329 |
| Ephb1 | 0.15638 | -0.34818625 |
| P2rx1 | 0.10313 | -0.7093949 |
| Ms4a5 | 0.1497 | -0.73900572 |
| Ms4a5 | 0.00144 | -1.25927895 |
| Ms4a7 | 0.05773 | -0.55727639 |
| Sh2d1a | 0.00145 | -1.25646236 |
| B9d1 | 0.00149 | -1.03696671 |
| Immp2l | 0.16986 | -0.56473724 |
| Zfp692 | 0.00144 | -1.41186687 |
| Eno1 | 0.07117 | -0.38088574 |
| Itsn1 | 0.00717 | -1.0591794 |
| Aoc3 | 0.34501 | -0.29989369 |
| Ddx24 | 0.00439 | -0.77685769 |
| Gm15743 | 0.38249 | 0.58774641 |
| Tmem30c | 0.00144 | -1.28898079 |
| Dhx34 | 0.00276 | -0.78179969 |
| Rsu1 | 0.00841 | 0.57125243 |
| Peg3os | 0.83195 | 0.37324963 |
| Nkain2 | 0.13847 | -0.42063534 |
| Arrdc2 | 0.0754 | 0.61400138 |
| Asap2 | 0.01119 | 0.5518403 |
| Tom1 | 0.00176 | -1.13725828 |
| Mrpl49 | 0.02375 | -0.65672731 |
| Dlg1 | 0.1309 | -0.28300679 |
| Hn1l | 0.09144 | -0.45071128 |
| LOC105247581 | 0.1628 | 0.45919429 |
| Snx9 | 0.1723 | -0.24964782 |
| Tnfaip1 | 0.05249 | -0.34726503 |
| Tmub1 | 0.00149 | -1.14733608 |
| Olfr902 | 0.00144 | -1.26301121 |
| Bpifb1 | 0.12962 | -0.47984272 |
| Olfr702 | 0.00144 | -1.31884045 |
| Olfr202 | 0.00144 | -1.22098834 |
| Inip | 0.04532 | 0.40526292 |
| Guca2b | 0.16749 | 0.39153154 |
| Cysrt1 | 0.00145 | -1.3170949 |
| Defb9 | 0.00144 | -1.22925777 |
| Olfr630 | 0.00144 | -1.29864962 |
| Ttll11 | 0.02219 | -0.63926347 |
| Olfr330 | 0.15595 | -0.36558022 |
| Defb5 | 0.00144 | -1.31495282 |
| AY702102 | 0.00637 | -1.02877696 |
| Gpsm3 | 0.00327 | -0.85071486 |
| Ak2 | 0.0507 | -0.5404151 |
| Ino80b | 0.00144 | -2.01222491 |
| Gps2 | 0.75915 | 0.05622281 |
| Cypt9 | 0.10202 | -0.60245164 |
| Cypt2 | 0.00144 | -1.39297126 |
| Cypt3 | 0.00166 | -1.21062785 |
| Cypt1 | 0.00144 | -1.29051721 |
| Cypt7 | 0.63896 | 1.07429192 |
| Cypt8 | 0.00158 | -1.33166064 |
| Cypt4 | 0.47488 | -0.31394146 |
| Gm2927 | 0.02024 | -1.09396448 |
| Gm14374 | 0.00144 | -1.38400103 |
| Ggnbp1 | 0.01951 | -0.65194616 |
| She | 0.74924 | 0.07345908 |
| Lilrb4 | 0.00183 | 1.7967117 |
| Best3 | 0.01783 | -0.57501227 |
| Gm35116 | 0.00144 | -1.45426645 |
| Ensa | 0.61519 | 0.08597032 |
| Lsp1 | 0.20906 | -0.23174386 |
| Gm4567 | 0.04405 | -1.04069639 |
| Unc119 | 0.00179 | -1.05676338 |
| Gm3428 | 0.00144 | -1.30713006 |
| Art5 | 0.01363 | -0.63764065 |
| Krr1 | 0.00192 | 1.11126882 |
| Rpl39 | 0.00159 | -1.0582045 |
| Obfc1 | 0.08991 | -0.29576635 |
| Obfc1 | 0.95901 | -0.01475354 |
| Vmn2r115 | 0.10957 | -0.69577828 |
| Sgk3 | 0.82086 | 0.09578866 |
| Las1l | 0.00144 | 1.46614673 |
| Ptma | 0.29283 | -0.27762231 |
| 3930401B19Rik | 0.06502 | 0.90195209 |
| 4932443I19Rik | 0.00144 | -1.27730451 |
| Rpl37a | 0.00144 | -1.30126744 |
| Apob | 0.02862 | -1.03550528 |
| Gm15104 | 0.04551 | -0.91232186 |
| Gm34059 | 0.00144 | -1.30713006 |
| 4930547H16Rik | 0.00188 | -1.39467507 |
| D14Ertd670e | 0.45683 | -0.21760865 |
| 1700034P13Rik | 0.01104 | -0.63498909 |
| Tns4 | 0.03758 | -0.84478818 |
| Vmn2r46 | 0.00635 | -1.11902425 |
| Mtfr1l | 0.12151 | -0.46022276 |
| Fam53b | 0.00212 | -0.94551549 |
| Gareml | 0.05122 | -0.86805242 |
| Adgrb1 | 0.10868 | -0.55987088 |
| Gstp2 | 0.00144 | -1.15289324 |
| Kat7 | 0.21934 | -0.23682192 |
| Caskin2 | 0.00159 | -0.85207179 |
| Timm10 | 0.28317 | -0.36814765 |
| Tln1 | 0.00155 | -1.26745198 |
| Tln1 | 0.00185 | -0.86876061 |
| Rpl27 | 0.00144 | -1.15498214 |
| Prss29 | 0.09824 | -0.83510342 |
| Defb46 | 0.00145 | -1.26219879 |
| Cpt1b | 0.00144 | -1.3083854 |
| Timm17a | 0.86211 | 0.06865296 |
| Nolc1 | 0.72956 | -0.06446512 |
| Ogfr | 0.0042 | -0.76766402 |
| Gm4832 | 0.12989 | -0.26431022 |
| Fam83g | 0.00226 | -1.49887581 |
| Gm14391 | 0.00162 | 2.41219635 |
| Smpx | 0.98781 | 0.0067004 |
| 1700101E01Rik | 0.03089 | -0.6383213 |
| Sgms1 | 0.802 | -0.07205189 |
| Hsf2bp | 0.02584 | -0.89223204 |
| Rab38 | 0.08159 | -1.03849241 |
| Med28 | 0.00295 | -0.99473776 |
| Rab37 | 0.0366 | -0.41813072 |
| Rab34 | 0.00471 | -0.7226463 |
| Cyfip1 | 0.05013 | 0.38837565 |
| Fhad1 | 0.07714 | -1.36585177 |
| Sfmbt2 | 0.00967 | -0.85716675 |
| Sfmbt2 | 0.10101 | 0.48471244 |
| Gm14431 | 0.00144 | 1.58927862 |
| Olfr102 | 0.00144 | -1.27699683 |
| Plekha4 | 0.19473 | -0.30711146 |
| Dcaf8 | 0.08129 | -0.34991457 |
| Rab3c | 0.05769 | -2.26431393 |
| Rab3a | 0.02985 | -0.6161419 |
| Slc6a5 | 0.00144 | -1.28898079 |
| Gpr52 | 0.06462 | -0.98558627 |
| Dach2 | 0.00144 | -1.27165604 |
| Dach2 | 0.00144 | -1.27165604 |
| Polr2l | 0.00156 | -1.10576992 |
| Rhox4g | 0.06614 | -1.29124124 |
| Hnrnph1 | 0.00914 | 0.96733539 |
| Serpinf2 | 0.15966 | -0.58955815 |
| Gm6980 | 0.20139 | 0.49583638 |
| Dmtn | 0.0532 | -0.41798764 |
| Stam | 0.59883 | 0.08391331 |
| 6030458C11Rik | 0.01851 | 0.52733647 |
| Efr3b | 0.42247 | -0.17536705 |
| Smco1 | 0.01035 | -0.60635904 |
| Bach2 | 0.14498 | -0.99991592 |
| H2-Q4 | 0.01204 | 1.06242135 |
| Zfp60 | 0.00879 | 1.80124507 |
| Chka | 0.02652 | 0.6606104 |
| Gm10094 | 0.39 | -0.26676005 |
| Git2 | 0.12151 | -0.2735694 |
| Gm8909 | 0.00144 | -1.26560808 |
| B4galnt2 | 0.00145 | -1.14181089 |
| Ctcfl | 0.90135 | 0.04736943 |
| Gpr68 | 0.02286 | -0.66499398 |
| Atp5g1 | 0.44214 | 0.18770374 |
| Pbld1 | 0.03571 | -0.51693906 |
| Nlrp1a | 0.92541 | -0.01866435 |
| Afg3l2 | 0.19987 | 0.32815604 |
| Cbfa2t2 | 0.91456 | -0.02435349 |
| Gm8437 | 0.55053 | 0.22961725 |
| Slc25a42 | 0.53428 | 0.15784189 |
| Aqp7 | 0.00245 | -1.23532872 |
| Rad51c | 0.0453 | -0.39746039 |
| Nlrp4f | 0.04377 | -1.23094218 |
| Ap5z1 | 0.06119 | 0.68733352 |
| Ctsa | 0.00508 | -0.85710722 |
| Gm13304 | 0.00626 | -0.8360246 |
| 2810021J22Rik | 0.10027 | 0.61611337 |
| Vmn2r108 | 0.00144 | -1.28898079 |
| Fopnl | 0.0845 | -0.36865361 |
| Adamts4 | 0.51127 | -0.41382837 |
| Ptpn5 | 0.00144 | -1.26560808 |
| Ptpn5 | 0.00144 | -1.42961246 |
| Med15 | 0.01376 | -0.60036033 |
| Hccs | 0.8381 | -0.05786314 |
| Gm6710 | 0.34561 | 0.37401551 |
| Spg7 | 0.00964 | -0.7530893 |
| Gm6146 | 0.03134 | 0.88459055 |
| Alg6 | 0.00462 | 0.87870512 |
| Chrm3 | 0.08117 | -0.88933941 |
| Ctla2a | 0.81202 | -0.07620194 |
| Trp53i11 | 0.07325 | -0.60481164 |
| Trp53i11 | 0.05907 | -0.55790534 |
| Nup93 | 0.26239 | -0.18277898 |
| Ccm2 | 0.00838 | -0.73187918 |
| Extl2 | 0.03048 | 0.49063893 |
| Extl3 | 0.02829 | -0.53074803 |
| Tfr2 | 0.10654 | -0.71587465 |
| Kcnj14 | 0.03876 | -1.27652649 |
| Synj1 | 0.00335 | 0.85220319 |
| Fam208b | 0.00232 | 1.40559338 |
| Bbs1 | 0.79678 | -0.11973891 |
| Grb10 | 0.00144 | -1.50830696 |
| Cmtm5 | 0.00144 | -1.46562577 |
| Gm1715 | 0.8295 | 0.06495029 |
| Atad3a | 0.01129 | -0.68530989 |
| Rgma | 0.00177 | 1.03991544 |
| Crygc | 0.44166 | -0.58888861 |
| Zfp820 | 0.00144 | 1.95286034 |
| Gm4969 | 0.01254 | -0.9021214 |
| Rps24 | 0.0867 | -0.29000168 |
| Rps6 | 0.62377 | -0.10293653 |
| Rabac1 | 0.00144 | -1.14015074 |
| Gm10204 | 0.37829 | -0.33832248 |
| Spata21 | 0.00144 | -1.28116048 |
| Crxos | 0.47811 | 0.24022893 |
| Camk2d | 0.21105 | 0.53135383 |
| 4933415F23Rik | 0.70835 | -0.32980286 |
| Camk1d | 0.31094 | 0.42549373 |
| Gm5475 | 0.00144 | -1.26934956 |
| Fam69c | 0.16009 | -0.85682098 |
| Fam65a | 0.00221 | -0.84496337 |
| Mx2 | 0.00144 | -1.28898079 |
| Gm11237 | 0.37263 | 0.33826705 |
| Igflr1 | 0.16822 | 0.44652231 |
| Spink12 | 0.00145 | -1.25646236 |
| Myo1g | 0.00821 | -0.76095831 |
| Trpv1 | 0.00144 | -1.57116529 |
| Slc10a1 | 0.2635 | -0.29295899 |
| Elk1 | 0.04496 | -0.51662524 |
| Npm1 | 0.0216 | 0.64450424 |
| Fbxl13 | 0.00144 | -1.29051721 |
| Lyg1 | 0.00144 | -1.31495282 |
| Endov | 0.24015 | -0.26461519 |
| Fbxw5 | 0.01313 | -0.77591127 |
| Fbxw2 | 0.12888 | 0.26310729 |
| Rpl12 | 0.00145 | -0.98708302 |
| Rpl17 | 0.93795 | -0.01332005 |
| Pars2 | 0.05975 | -0.52454434 |
| Ramp3 | 0.00144 | -1.31200743 |
| D1Ertd622e | 0.00462 | 0.7043041 |
| Defa26 | 0.00144 | -1.23532597 |
| Gm15292 | 0.00144 | -1.8553457 |
| Gm12616 | 0.01597 | -0.56377058 |
| Prickle1 | 0.00144 | -1.10868499 |
| Cyb561 | 0.85608 | -0.0317666 |
| LOC545966 | 0.00158 | -1.2446827 |
| Tac1 | 0.97938 | 0.0144306 |
| B230206H07Rik | 0.0051 | -1.06784446 |
| Ccdc63 | 0.00144 | -1.28898079 |
| Siglec1 | 0.03864 | -0.51639268 |
| Gm23952 | 0.09543 | 0.73327842 |
| Ssh2 | 0.29046 | -0.18196662 |
| Gm15800 | 0.25147 | -0.25886679 |
| Zranb3 | 0.00145 | -1.24191372 |
| Zfp446 | 0.20835 | -0.23533417 |
| Btbd10 | 0.01933 | 0.5659375 |
| Gm4297 | 0.00144 | -1.29085005 |
| Mapt | 0.00145 | -0.9327978 |
| Gm5072 | 0.00144 | -1.32766452 |
| Myh14 | 0.12908 | -0.41865549 |
| Gm7967 | 0.05041 | 0.85575902 |
| Gm10475 | 0.00144 | -1.27165604 |
| Susd4 | 0.00232 | -1.21789579 |
| Vmn2r72 | 0.00145 | -1.2388121 |
| Nrn1 | 0.00144 | -1.27165604 |
| Ttc28 | 0.03173 | -0.417784 |
| Ttc23 | 0.09939 | -0.31991335 |
| Ttc24 | 0.00144 | -1.27165604 |
| Murc | 0.048 | 0.50304377 |
| Esrra | 0.13491 | -0.48974675 |
| Esrrb | 0.07511 | -0.63199472 |
| Emid1 | 0.03374 | -0.48749048 |
| Emid1 | 0.27707 | -0.383539 |
| Zfp672 | 0.53526 | -0.15307594 |
| Zfp652 | 0.09945 | -0.41217618 |
| Il1rapl2 | 0.00158 | -1.17515383 |
| Lrtm2 | 0.71244 | 0.32168234 |
| Olfr1018 | 0.00144 | -1.25118919 |
| Emp1 | 0.01644 | 0.59832434 |
| Snrpd2 | 0.00201 | -1.01118282 |
| C1ql3 | 0.20361 | -0.73065044 |
| C1ql1 | 0.00144 | -1.29085005 |
| Wdr43 | 0.00335 | 0.99958502 |
| C8b | 0.00145 | -1.22518835 |
| Ccdc115 | 0.95068 | 0.01198492 |
| Nrxn2 | 0.00704 | -0.84839072 |
| Nrxn2 | 0.0015 | -1.08857924 |
| Jakmip1 | 0.00144 | -1.22035516 |
| Scarb1 | 0.00182 | -0.87017672 |
| Pbx1 | 0.97647 | -0.00711025 |
| Nme9 | 0.00793 | -1.16097332 |
| Gm11033 | 0.02382 | 0.87778627 |
| Pdhb | 0.51118 | -0.15155692 |
| Cdkl3 | 0.1173 | -0.99670992 |
| Cdkl3 | 0.67563 | 0.13050352 |
| Cdkl3 | 0.23387 | 0.24493986 |
| Tex35 | 0.00144 | -1.25118919 |
| Dcdc2b | 0.09129 | -0.31756136 |
| Me3 | 0.05269 | -0.56810957 |
| Mccc1 | 0.08758 | 0.38739353 |
| Mccc2 | 0.74061 | -0.05287267 |
| Dpm1 | 0.02634 | 0.5080955 |
| Skor2 | 0.00158 | -1.2446827 |
| 1700041C23Rik | 0.00144 | -1.28898079 |
| Sema4c | 0.03055 | -0.52767032 |
| Vstm2l | 0.01581 | -0.83486434 |
| Stk39 | 0.00525 | 1.05122789 |
| Abca8b | 0.8623 | 0.04071584 |
| Pld5 | 0.64572 | -0.22774348 |
| 4930566N20Rik | 0.01629 | -1.68726843 |
| Btk | 0.01212 | -0.99768241 |
| Fam71d | 0.02214 | 0.67331018 |
| Pla2g16 | 0.43817 | -0.1353739 |
| Fam98c | 0.00242 | -0.92800349 |
| Itk | 0.9001 | -0.05229403 |
| Serinc2 | 0.02628 | -1.06524408 |
| Cnr1 | 0.00144 | -1.28116048 |
| Bop1 | 0.05952 | -0.54202705 |
| Cry2 | 0.2474 | -0.35411381 |
| Zc3h12b | 0.00144 | -1.28116048 |
| Top1 | 0.00303 | 1.12784104 |
| Nsmaf | 0.0715 | 0.36113463 |
| Stk4 | 0.12307 | -0.73954676 |
| Vmn2r33 | 0.73262 | -0.20716279 |
| Zdhhc18 | 0.00144 | -1.10382058 |
| Nkx6-1 | 0.27348 | -0.6285891 |
| Olfr1262 | 0.15082 | -0.76538382 |
| Top3a | 0.02045 | -0.45396284 |
| Tyrp1 | 0.00144 | -1.30713006 |
| Brd2 | 0.00405 | -0.76807002 |
| Spon1 | 0.14794 | -0.40806116 |
| Lrrc8b | 0.01305 | -0.80316828 |
| Copz1 | 0.58338 | -0.11425326 |
| Yap1 | 0.95468 | 0.00979575 |
| Osbpl6 | 0.22098 | 0.39190861 |
| Lemd3 | 0.00776 | 0.59641953 |
| Rab13 | 0.53962 | 0.10746108 |
| Rab12 | 0.10681 | -0.26369528 |
| Efcab3 | 0.00144 | -1.2963229 |
| Mlana | 0.22671 | -0.86649574 |
| Rbfox1 | 0.92344 | 0.02382713 |
| Fabp5 | 0.00144 | -1.10976367 |
| Luc7l2 | 0.13476 | 0.64144008 |
| Luc7l2 | 0.02717 | -0.53903574 |
| Triobp | 0.20182 | 0.33007347 |
| Triobp | 0.00145 | -1.22376446 |
| Wfdc3 | 0.00525 | -1.08389866 |
| Sord | 0.00149 | -0.88597176 |
| Vmn1r176 | 0.00144 | -1.27165604 |
| Maneal | 0.98793 | -0.00511604 |
| Ctnnd1 | 0.45442 | 0.27895212 |
| Ctnnd1 | 0.49438 | -0.10243555 |
| Mafk | 0.16119 | -0.31041956 |
| Epb4.2 | 0.9926 | -0.01228913 |
| Map2k5 | 0.01112 | -0.63028452 |
| Gm14484 | 0.0671 | 0.40269527 |
| Olfr1301 | 0.00144 | -1.25118919 |
| Uchl3 | 0.75259 | 0.16562624 |
| Mettl18 | 0.26104 | 0.2787251 |
| Olfr1450 | 0.00144 | -1.30713006 |
| Gm9567 | 0.00144 | -1.29049509 |
| Wt1 | 0.00706 | 0.85061903 |
| Olfr221 | 0.00144 | -1.26301121 |
| Pabpc4 | 0.00145 | -1.05415993 |
| Hspe1 | 0.95458 | -0.02030857 |
| E130311K13Rik | 0.01369 | 0.61512839 |
| Ttll7 | 0.02385 | -0.96358491 |
| Tnfsf12 | 0.00353 | -0.69170895 |
| Ccser1 | 0.00144 | -1.27165604 |
| Ccser1 | 0.00144 | -1.28116048 |
| Myl6b | 0.00229 | -0.93504393 |
| Rps6ka3 | 0.26659 | 0.3681963 |
| Rps6ka3 | 0.17069 | 0.28808261 |
| Olfr851 | 0.05499 | -2.08607875 |
| Olfr651 | 0.00144 | -1.28116048 |
| Olfr551 | 0.23242 | -0.66451208 |
| Cic | 0.003 | -0.7834031 |
| Olfr151 | 0.01019 | -1.01479804 |
| Gm833 | 0.00144 | -1.29085005 |
| Cyp4f18 | 0.01023 | -0.67891458 |
| Snrpf | 0.00933 | -0.61401303 |
| Timm8a1 | 0.21481 | 0.20266653 |
| Kctd2 | 0.00144 | -1.00049708 |
| Rpl12 | 0.00601 | -0.75397822 |
| Gm38521 | 0.01831 | -1.29381006 |
| Gtf2a2 | 0.79315 | 0.05957143 |
| LOC105242408 | 0.00826 | -1.92242163 |
| Gm3772 | 0.00144 | -1.27165604 |
| Kctd18 | 0.00145 | 1.7066445 |
| Dpys | 0.03401 | -0.89712819 |
| Ear2 | 0.00603 | -1.87250911 |
| Gm21951 | 0.01214 | -1.72975665 |
| Ces1a | 0.00144 | -1.36353418 |
| Rrp7a | 0.00144 | -0.99827514 |
| Sult2a1 | 0.00144 | -1.54415904 |
| Sult2a2 | 0.00144 | -1.27165604 |
| Sult2a4 | 0.00144 | -1.28116048 |
| Mapk7 | 0.01136 | -0.53525749 |
| Pbp2 | 0.00144 | -1.28116048 |
| Gm4719 | 0.03347 | -1.99066187 |
| Rpl30 | 0.01317 | -0.5111767 |
| Dyx1c1 | 0.05855 | 1.05836488 |
| Tm2d1 | 0.39159 | 0.25966577 |
| Odf2 | 0.00218 | 0.89067668 |
| Odf2 | 0.50375 | -0.15241935 |
| Cphx1 | 0.00268 | -1.38302854 |
| Fkbp5 | 0.02657 | 0.79747199 |
| Anapc5 | 0.01847 | -0.48507953 |
| D630023F18Rik | 0.65389 | -0.22352909 |
| Olfr1415 | 0.49734 | -0.36165176 |
| C030016D13Rik | 0.05209 | 1.15195629 |
| Xylt2 | 0.01549 | -0.60919271 |
| Ccnb1 | 0.07112 | -0.71115412 |
| Slitrk2 | 0.00144 | -1.27165604 |
| Gm8883 | 0.10094 | -1.93197334 |
| Pnpo | 0.00561 | -0.75787564 |
| Nkx3-1 | 0.00145 | -1.26219879 |
| Vmn2r111 | 0.00472 | -1.02583646 |
| Arid3a | 0.02383 | -0.62291429 |
| Psma3 | 0.15251 | 0.24945232 |
| LOC102641351 | 0.09972 | -0.5727456 |
| Car10 | 0.00158 | -1.2446827 |
| Car10 | 0.00385 | -1.73728725 |
| Car10 | 0.0051 | -1.86188489 |
| Npb | 0.37547 | -0.16738006 |
| Npb | 0.03881 | 0.46053838 |
| Trp63 | 0.89707 | 0.13684091 |
| Olfr417 | 0.02134 | -0.87819776 |
| Olfr317 | 0.1437 | -0.7465339 |
| R3hdm2 | 0.02183 | -0.58225457 |
| Cxcl16 | 0.2891 | -0.19468936 |
| Pik3cd | 0.00327 | -1.20817336 |
| Cx3cr1 | 0.18029 | 0.65580803 |
| Dpy19l3 | 0.20673 | -0.33227932 |
| Slc35b4 | 0.05161 | -0.49974132 |
| A630072L19Rik | 0.00264 | 1.36465872 |
| Mastl | 0.00758 | -1.5074314 |
| Gm10199 | 0.00144 | -1.27165604 |
| Pdk3 | 0.61267 | -0.15659592 |
| Gm19337 | 0.00144 | 2.03511906 |
| Ippk | 0.32618 | -0.16084206 |
| 4933434M16Rik | 0.09244 | -1.06528354 |
| Scrn3 | 0.42048 | 0.18311628 |
| Itm2b | 0.40283 | -0.15851678 |
| Tm9sf1 | 0.13819 | 0.32164515 |
| Mgat4c | 0.00144 | -1.27165604 |
| Mef2b | 0.00144 | -1.3348153 |
| Ang4 | 0.57077 | -0.11676718 |
| H2-M10.3 | 0.00144 | -1.29085005 |
| Gm37915 | 0.00144 | -1.30713006 |
| Arhgef15 | 0.33758 | 0.24777259 |
| Klra8 | 0.06548 | 1.0190966 |
| Btrc | 0.90668 | 0.02047813 |
| Il3 | 0.05375 | -2.2096489 |
| Sowahc | 0.00873 | 1.48647816 |
| Slc6a2 | 0.00145 | -1.26219879 |
| Pla2g3 | 0.00144 | -1.21868187 |
| Olfr1196 | 0.00144 | -1.31495282 |
| Hira | 0.11314 | -0.2940444 |
| Zfp874a | 0.00144 | 2.33247382 |
| Dcxr | 0.00152 | -0.9829518 |
| Gm29684 | 0.04413 | -1.03617482 |
| Nhs | 0.03972 | 0.77277228 |
| Gm5127 | 0.00144 | -1.28116048 |
| Gm26653 | 0.00144 | -1.28898079 |
| Sap30bp | 0.95853 | 0.0257423 |
| Gm29571 | 0.0033 | -1.46517328 |
| Gm29674 | 0.00144 | -1.26560808 |
| Depdc5 | 0.05555 | 0.48406499 |
| Ccdc15 | 0.46515 | -0.43836085 |
| Gm29968 | 0.08686 | -1.0906744 |
| Gm732 | 0.00144 | -1.25118919 |
| B4galnt4 | 0.00144 | -1.30713006 |
| 1700025K24Rik | 0.00144 | -1.28898079 |
| C2cd3 | 0.00206 | -1.518327 |
| H2-Eb2 | 0.09244 | -2.68108838 |
| D430019H16Rik | 0.52738 | -0.18479648 |
| Adam18 | 0.62475 | -0.43481489 |
| Parp14 | 0.84697 | -0.07065661 |
| Atad2b | 0.61198 | 0.09186323 |
| Inpp5k | 0.95282 | -0.01568488 |
| Egflam | 0.00144 | -1.27165604 |
| Aplnr | 0.00194 | -1.47836481 |
| Gm4371 | 0.61689 | -0.16021001 |
| Ago1 | 0.00488 | -0.95581663 |
| Tubgcp5 | 0.00144 | 1.7064488 |
| Pard3b | 0.02051 | -0.91531813 |
| Polr2e | 0.0124 | -0.81526431 |
| Hoxb9 | 0.00144 | -1.26934956 |
| Fzr1 | 0.19504 | -0.4237507 |
| Synj1 | 0.00483 | -2.06916939 |
| Gm41230 | 0.00144 | -1.29746368 |
| Trpm3 | 0.2811 | -0.22994723 |
| Reps1 | 0.01743 | -0.90212755 |
| Gm6260 | 0.18927 | -0.63686169 |
| C730036E19Rik | 0.00144 | -1.33059316 |
| LOC105246851 | 0.00145 | -1.22376446 |
| 4930507D05Rik | 0.00145 | -1.25646236 |
| Gm35215 | 0.00145 | -1.2388121 |
| Tmprss11a | 0.00145 | -1.26219879 |
| Slc25a12 | 0.00347 | 1.3541333 |
| Aph1a | 0.29058 | -0.37083821 |
| 1700024I08Rik | 0.00144 | -1.30713006 |
| Smlr1 | 0.00144 | -1.33632959 |
| Wasl | 0.04332 | -0.41085132 |
| Pgp | 0.88218 | -0.0785025 |
| Ttc30b | 0.00208 | 1.55260638 |
| Hmmr | 0.39906 | -1.036808 |
| Vkorc1l1 | 0.08681 | 0.44285295 |
| Gm38596 | 0.00144 | -1.44608204 |
| Fam65c | 0.18793 | -0.81917677 |
| Srek1 | 0.00144 | 1.64892446 |
| Sf1 | 0.02068 | -0.51366907 |
| Scn9a | 0.02122 | -1.79743914 |
| Gm39244 | 0.00144 | -1.23984951 |
| Spire1 | 0.23455 | -0.66019418 |
| 1700128A07Rik | 0.00144 | -1.33632959 |
| 5430440L12Rik | 0.15325 | 0.46943797 |
| 1700029N11Rik | 0.00144 | -1.28116048 |
| Dsg2 | 0.00678 | -1.23116232 |
| Oog3 | 0.00144 | -1.30713006 |
| Tbx19 | 0.00151 | -1.17792995 |
| Ugcg | 0.00867 | 0.78480603 |
| Sh3rf3 | 0.09727 | 0.70656624 |
| Smim19 | 0.69402 | 0.15252016 |
| 4933400C23Rik | 0.00144 | -1.26560808 |
| Ndnf | 0.82231 | 0.0958081 |
| Ankrd12 | 0.00144 | 1.51468722 |
| C430019N01Rik | 0.64453 | -0.29785597 |
| Skint2 | 0.00158 | -1.20978554 |
| Gm35412 | 0.00144 | -1.30713006 |
| Scarf1 | 0.04434 | -0.56262744 |
| Cyp2a22 | 0.00144 | -1.30017714 |
| Tcerg1l | 0.00144 | -1.29085005 |
| Clca4b | 0.00144 | -1.26560808 |
| 1700025L06Rik | 0.00144 | -1.31627578 |
| Pms2 | 0.03022 | -1.60794495 |
| Tgfb1i1 | 0.05012 | -0.51818651 |
| Gsdma | 0.09304 | -0.71834496 |
| Zfp160 | 0.03174 | 0.75805503 |
| Nicn1 | 0.3057 | 0.54958594 |
| Galnt16 | 0.0055 | -0.9804196 |
| Mnat1 | 0.00144 | -1.31495282 |
| C77370 | 0.00415 | -1.16878422 |
| Gm31343 | 0.09323 | -1.26154495 |
| 4930445N08Rik | 0.00144 | -1.28116048 |
| 2010007H06Rik | 0.00144 | 1.5494924 |
| Slc22a19 | 0.00741 | -1.03531684 |
| 2510009E07Rik | 0.97885 | -0.01027737 |
| Xndc1 | 0.00512 | 0.79376589 |
| Rtn4rl1 | 0.08416 | -0.35500077 |
| 4932441J04Rik | 0.29441 | -0.21417027 |
| Ddx24 | 0.00144 | -1.29983008 |
| Gm8096 | 0.00144 | -1.35523776 |
| C78532 | 0.00145 | -1.22376446 |
| Id2 | 0.00693 | -1.94289878 |
| LOC382202 | 0.20094 | -0.7272063 |
| Spam1 | 0.00144 | -1.24811894 |
| Gm8369 | 0.08976 | -1.11780688 |
| Copg2os2 | 0.18393 | 0.61987944 |
| Gm26519 | 0.00144 | -1.27992547 |
| Itgb6 | 0.15608 | -0.41314143 |
| Gm19744 | 0.00144 | -1.31495282 |
| Nrp2 | 0.06478 | 0.45873304 |
| Alg5 | 0.23702 | -0.19150127 |
| Sec61b | 0.00377 | -0.74926198 |
| Nacc1 | 0.49636 | -0.2678247 |
| Spcs2 | 0.00182 | -0.86951612 |
| Ddx23 | 0.0019 | -0.94455821 |
| Cabin1 | 0.40778 | -0.49144619 |
| Gxylt2 | 0.01012 | -0.86334164 |
| Cep162 | 0.11471 | 0.38611791 |
| Mycl | 0.1352 | -1.60980395 |
| Gm10941 | 0.25561 | -0.77036218 |
| Gm31739 | 0.0766 | -2.39341909 |
| Gm17102 | 0.02719 | -0.96413034 |
| Gm11549 | 0.00144 | -1.23532597 |
| 1810013L24Rik | 0.00356 | 1.20565624 |
| Klhl42 | 0.29975 | -0.22698888 |
| 4631422I05Rik | 0.00971 | -1.62754768 |
| Zfp236 | 0.80143 | -0.22261622 |
| Fam172a | 0.04175 | -0.50020915 |
| Abcb1b | 0.04504 | -0.73372858 |
| Rgs2 | 0.00735 | 0.8949721 |
| Kdm2a | 0.06827 | -0.82689682 |
| Supt20 | 0.00144 | 1.20588657 |
| Gm31007 | 0.00144 | -1.28116048 |
| C130057D09Rik | 0.06409 | 0.42306724 |
| B4galnt3 | 0.00298 | -1.42363422 |
| Rab5b | 0.72474 | 0.13446687 |
| Gm26512 | 0.00144 | -1.30017714 |
| LOC102643203 | 0.00144 | -1.30713006 |
| Dnajb12 | 0.01084 | -0.65900765 |
| A930041C12Rik | 0.00775 | -1.30437418 |
| Klhdc2 | 0.62444 | 0.21724117 |
| Ermard | 0.00144 | 2.29151021 |
| Drp2 | 0.84961 | 0.25470178 |
| Ankrd33b | 0.89564 | -0.0382254 |
| Slc38a11 | 0.00144 | -1.25118919 |
| Rbm24 | 0.45694 | 0.17913697 |
| Ppp4r1l-ps | 0.01031 | 1.04972882 |
| Bcorl1 | 0.01463 | -0.71741673 |
| Slitrk4 | 0.01382 | 1.44126054 |
| A930001C03Rik | 0.82552 | -0.06123732 |
| Zbtb34 | 0.49761 | 0.78651413 |
| Ptpro | 0.00144 | -1.28116048 |
| Gm11264 | 0.00144 | -1.26560808 |
| 4930406M16Rik | 0.00144 | -1.28116048 |
| 4933434P08Rik | 0.22388 | -0.47357245 |
| Zcchc17 | 0.03276 | -0.43845414 |
| Mier1 | 0.84678 | 0.06505573 |
| Kif15 | 0.00144 | -1.25927895 |
| Ubr7 | 0.37024 | -0.34192537 |
| Yars2 | 0.15336 | 0.35495038 |
| 1700018G05Rik | 0.00225 | -1.18527205 |
| Ccni | 0.73417 | 0.05991084 |
| Tprg | 0.16982 | -0.78018126 |
| Olfr1386 | 0.00144 | -1.31495282 |
| Pros1 | 0.02986 | 0.54294893 |
| Gm26878 | 0.00144 | -1.27165604 |
| Lrrc15 | 0.00144 | -1.25118919 |
| Gm12381 | 0.00178 | 1.59940476 |
| BB217526 | 0.41052 | 0.18418638 |
| Gabra3 | 0.00875 | -1.62591475 |
| Gm35288 | 0.00144 | -1.28116048 |
| Gm37896 | 0.00186 | -1.71166556 |
| 1700101C01Rik | 0.00144 | -1.29085005 |
| Klhl31 | 0.03576 | -0.54513003 |
| 2310069B03Rik | 0.00144 | -1.27165604 |
| Olfr435 | 0.00144 | -1.25927895 |
| Ppp3r1 | 0.19298 | -0.23743868 |
| Ifi27l2a | 0.00144 | -1.42945582 |
| Mgat1 | 0.29178 | -0.30996341 |
| Oaz2 | 0.89575 | -0.05194657 |
| Ucp2 | 0.01385 | -0.72413959 |
| Prep | 0.24448 | -0.28606574 |
| Atxn2 | 0.43303 | 0.28689259 |
| LOC105244195 | 0.00145 | -1.22376446 |
| E030004H24Rik | 0.78703 | 0.12136105 |
| Gm16838 | 0.05179 | -1.00969764 |
| Gm6213 | 0.00144 | -1.28116048 |
| Arfgef3 | 0.00144 | -1.27454685 |
| Trpc5 | 0.00144 | -1.29049509 |
| Aco2 | 0.02499 | -0.61282114 |
| Fndc3c1 | 0.00144 | -1.27165604 |
| Mcur1 | 0.12998 | 0.31660197 |
| 2310021N16Rik | 0.01735 | -0.8645468 |
| Pmp2 | 0.00144 | -1.30713006 |
| Topaz1 | 0.00144 | -1.28898079 |
| Dym | 0.951 | -0.04408442 |
| Bcl9 | 0.00813 | -0.59321297 |
| Pcnt | 0.02885 | -0.4190699 |
| Prdx2 | 0.35024 | -0.50156078 |
| Tmie | 0.02758 | -0.67688808 |
| Frmd5 | 0.00502 | -1.46570614 |
| Cbx8 | 0.00218 | -0.93353976 |
| Gatsl2 | 0.11077 | -0.38291803 |
| Gm12289 | 0.23507 | -0.64733147 |
| A030007N12Rik | 0.3047 | 0.76200681 |
| Vps16 | 0.00144 | -1.24428707 |
| Cdr2l | 0.00243 | -0.85747087 |
| Ltn1 | 0.99923 | 0.00025802 |
| Gpr174 | 0.00144 | -1.30713006 |
| Dusp18 | 0.57683 | 0.13052951 |
| Dlg2 | 0.06451 | 0.60061377 |
| Tmem251 | 0.0974 | 0.55027661 |
| Dock5 | 0.28531 | 0.25129442 |
| Ubfd1 | 0.21423 | 0.53723086 |
| Ctsq | 0.00145 | -1.2551153 |
| Vps37a | 0.00185 | 1.19989415 |
| Zfp933 | 0.02658 | 0.71077948 |
| Tor2a | 0.00147 | -1.03764068 |
| 1700109G14Rik | 0.00153 | -1.24063982 |
| Senp7 | 0.00144 | 2.07062322 |
| Dgka | 0.00144 | 1.11497955 |
| Cyp24a1 | 0.14744 | -2.76066909 |
| Pofut2 | 0.00144 | -0.99388716 |
| Pxylp1 | 0.01151 | -2.35536306 |
| Gm36839 | 0.86241 | -0.03204767 |
| A730011C13Rik | 0.12923 | -0.57144857 |
| Epha5 | 0.02568 | -1.45738376 |
| Gm37065 | 0.00144 | -1.25118919 |
| Slc30a10 | 0.00145 | -1.22193674 |
| Gm20149 | 0.00144 | -1.45121232 |
| Gm26544 | 0.00144 | -1.27165604 |
| E130202H07Rik | 0.00144 | -1.29878664 |
| Dsg2 | 0.00357 | -1.00273706 |
| Chrm4 | 0.80431 | -0.39491329 |
| Ampd1 | 0.00145 | -1.26219879 |
| Slc6a7 | 0.48852 | -0.41559194 |
| Pla2g12a | 0.00214 | -0.94187369 |
| Kat2b | 0.00345 | -0.96352056 |
| Ccdc15 | 0.00149 | -1.28183002 |
| Fam107a | 0.00144 | 1.3721451 |
| Fam175b | 0.00592 | 0.90234424 |
| Aldh1a3 | 0.073 | -0.47290875 |
| Gm30827 | 0.99416 | 0.00466442 |
| Cfap61 | 0.46737 | -0.33004325 |
| Mfsd4 | 0.81332 | -0.400003 |
| Olfr635 | 0.91925 | 0.06566352 |
| Olfr860 | 0.00145 | -1.22193674 |
| Il24 | 0.00144 | -1.27165604 |
| LOC102634683 | 0.95103 | 0.03043697 |
| Timp1 | 0.02605 | -0.77119789 |
| Scgb1b27 | 0.95767 | 0.08094416 |
| 4930595D18Rik | 0.00144 | -1.31884045 |
| Gm30225 | 0.0627 | -1.2232838 |
| A530010F05Rik | 0.00158 | -1.2446827 |
| Crocc2 | 0.0018 | -1.21153109 |
| Gm7607 | 0.00176 | -1.24266807 |
| Gm12436 | 0.03384 | -4.49467242 |
| Gm36011 | 0.00144 | -1.29049509 |
| Gm36487 | 0.00144 | -1.24811894 |
| Gm2136 | 0.00144 | -1.30713006 |
| Hbq1b | 0.04915 | -0.65526001 |
| Cox8c | 0.00144 | -1.25927895 |
| Vezt | 0.43727 | 0.38973316 |
| 1700019E08Rik | 0.00144 | -1.29864962 |
| Tcf12 | 0.01459 | 1.45876055 |
| Odf2l | 0.38072 | -0.38045195 |
| Dcun1d5 | 0.04526 | 0.66109657 |
| Sez6 | 0.00145 | -1.22518835 |
| Thoc3 | 0.87067 | 0.06769403 |
| Mief1 | 0.03105 | -0.43962281 |
| Gm4665 | 0.00144 | -1.26560808 |
| Mthfd2l | 0.04112 | -0.61021797 |
| Rabl6 | 0.01471 | 0.99243654 |
| Kansl1l | 0.00144 | -1.33632959 |
| Kcnc1 | 0.00144 | -1.29864962 |
| Arl4a | 0.60905 | 0.77263292 |
| Ifi27 | 0.00991 | -0.58369665 |
| D030068K23Rik | 0.00144 | -1.29085005 |
| 1700016F12Rik | 0.00144 | -1.28898079 |
| Pex12 | 0.31581 | -0.54149695 |
| Cacng5 | 0.00159 | -1.26431393 |
| Haus6 | 0.97568 | 0.00691952 |
| Mtx2 | 0.14549 | 0.55684647 |
| Klhl28 | 0.00336 | -1.20864476 |
| Serpinb1b | 0.00144 | -1.24811894 |
| Mrpl1 | 0.00145 | 1.20827688 |
| Fam35a | 0.29548 | -0.61956739 |
| Ddx25 | 0.00144 | -1.27165604 |
| Mier1 | 0.10232 | 0.92857597 |
| Mrpl45 | 0.0137 | -0.65096207 |
| Gm29683 | 0.00144 | -1.30017714 |
| A230059L01Rik | 0.00145 | -1.22518835 |
| Tcf12 | 0.01054 | 1.43099094 |
| Trim66 | 0.01481 | -0.99746227 |
| Chrna9 | 0.13958 | -0.85682098 |
| Mb21d2 | 0.6772 | -0.07943071 |
| Cant1 | 0.33755 | -0.21831221 |
| Eif6 | 0.01008 | -0.72289234 |
| Pag1 | 0.00144 | -1.31495282 |
| Chrm1 | 0.00144 | -1.00790463 |
| Olfr146 | 0.00144 | -1.28116048 |
| Teddm2 | 0.00144 | -1.20579466 |
| Angptl2 | 0.44407 | 0.27888097 |
| Vwc2l | 0.00144 | -1.25118919 |
| Stk38 | 0.44625 | -0.25052187 |
| Mrps9 | 0.05611 | 0.50646458 |
| Gm6288 | 0.00144 | -1.29746368 |
| Clcn4-2 | 0.09235 | 0.31067045 |
| Tlr3 | 0.00144 | 1.56058026 |
| Acad11 | 0.01661 | 1.78757045 |
| Metap2 | 0.03078 | -0.87097478 |
| Grip1os2 | 0.00144 | -1.29746368 |
| 5730480H06Rik | 0.00144 | 2.40180662 |
| Pak3 | 0.85165 | 0.06171759 |
| Slc44a1 | 0.88063 | 0.02749655 |
| Zscan10 | 0.20029 | -0.41890416 |
| Tpsab1 | 0.00144 | -1.29746368 |
| Ankrd6 | 0.56884 | 0.11834215 |
| Ddrgk1 | 0.04342 | -0.41704271 |
| Tbca | 0.00144 | -1.30713006 |
| Gm9804 | 0.00144 | -1.30017714 |
| Otud3 | 0.47961 | -0.14408927 |
| Cmss1 | 0.00799 | 0.91104023 |
| Dnal1 | 0.29744 | -0.60457803 |
| Homez | 0.34326 | 0.20528049 |
| 4931419H13Rik | 0.14385 | -0.84559124 |
| Ric1 | 0.0018 | 1.09462261 |
| Rgs17 | 0.00144 | 1.55001918 |
| Gm13480 | 0.27209 | -0.70593766 |
| LOC545785 | 0.01268 | -1.05366396 |
| Gm35172 | 0.11653 | -2.70775292 |
| 4930532M18Rik | 0.13413 | -3.10353666 |
| Ophn1 | 0.00158 | -1.2446827 |
| Pgd | 0.03494 | -0.62851621 |
| Socs2 | 0.02213 | 0.81057481 |
| Cyp7a1 | 0.00144 | -1.28116048 |
| Taf7 | 0.91776 | -0.03637783 |
| 4932416K20Rik | 0.00144 | -1.27699683 |
| Gm35216 | 0.00144 | -1.26911831 |
| B230354K17Rik | 0.09575 | -0.49563019 |
| Tmem260 | 0.01897 | 0.57497436 |
| Vps29 | 0.03249 | 1.50567434 |
| Txk | 0.01566 | -0.98182861 |
| Rapgef6 | 0.00579 | 0.68640321 |
| Adh1 | 0.00144 | -1.29746368 |
| Gm3134 | 0.66316 | 0.13432156 |
| Gm14023 | 0.05893 | 0.72841889 |
| Taf5 | 0.00149 | -1.25408 |
| 4930552P06Rik | 0.73451 | -0.31286649 |
| 4930428G15Rik | 0.00145 | -1.33072139 |
| 3021401N23Rik | 0.24578 | -0.88782149 |
| Ncbp2 | 0.60054 | 0.11183362 |
| Hpn | 0.35858 | 0.39652034 |
| Tmem178 | 0.99847 | 0.00069276 |
| Xpa | 0.74493 | 0.06171068 |
| Mroh2a | 0.01183 | -1.04972215 |
| Kcnq5 | 0.00144 | -1.26560808 |
| Iba57 | 0.34751 | -0.28591685 |
| Olfm4 | 0.13028 | -0.6578426 |
| Hspa1a | 0.1453 | -0.99427932 |
| Lurap1 | 0.0279 | 0.78114134 |
| Cxadr | 0.0208 | -1.06728525 |
| Ctcflos | 0.0017 | -1.24950944 |
| Bcl11a | 0.06938 | 1.11246058 |
| Slc9a3 | 0.00144 | -1.27165604 |
| 4933432K03Rik | 0.01206 | -1.14950855 |
| Ascl4 | 0.00144 | -1.30017714 |
| Mcm9 | 0.0213 | 0.71858514 |
| B3galt1 | 0.7565 | -0.19649259 |
| Nmt1 | 0.98559 | 0.00461013 |
| Cluap1 | 0.10901 | -0.30697448 |
| Lypla1 | 0.00144 | 1.58305763 |
| Cycs | 0.00511 | 0.80555978 |
| Wnk1 | 0.36156 | -0.14744276 |
| Hypk | 0.12365 | -0.26762457 |
| Fli1 | 0.03773 | 0.7533244 |
| Pitpnc1 | 0.27314 | 0.42110798 |
| Cxcl15 | 0.00144 | -1.27165604 |
| Gm30667 | 0.04425 | -0.92374632 |
| Micu1 | 0.07496 | -0.46456234 |
| Gm31548 | 0.55593 | -0.31925349 |
| Slc17a6 | 0.00144 | -1.30713006 |
| D030044L04Rik | 0.00144 | -1.41443942 |
| 9530006C21Rik | 0.01791 | 0.71270622 |
| 1700065O20Rik | 0.24489 | -0.74642781 |
| 4930579H20Rik | 0.00144 | -1.28116048 |
| 4930565D16Rik | 0.00145 | -1.22376446 |
| Klf12 | 0.00144 | 1.35739579 |
| Smim22 | 0.00158 | -1.50139888 |
| Gm6815 | 0.0219 | -0.60776549 |
| LOC100502592 | 0.93754 | 0.02369437 |
| 2810001G20Rik | 0.53387 | -0.18411564 |
| Trhde | 0.00144 | -1.25927895 |
| Ttc26 | 0.01177 | 1.40715729 |
| Psma4 | 0.54298 | 0.10607289 |
| Zfhx4 | 0.04211 | -3.55080493 |
| B230311B06Rik | 0.01226 | -0.87933658 |
| BC064078 | 0.11253 | -2.82784463 |
| Wwp2 | 0.02915 | -1.27217175 |
| Ogfrl1 | 0.03164 | -0.81484716 |
| Gm33378 | 0.10154 | -1.87803607 |
| Gm9932 | 0.00144 | -1.63351047 |
| Ostc | 0.00145 | -1.2388121 |
| Gm3830 | 0.00144 | -1.29049509 |
| Amn1 | 0.01991 | 2.0087392 |
| LOC102633151 | 0.82624 | -0.10080288 |
| Ipmk | 0.05824 | -0.53372773 |
| Wnk3 | 0.00144 | -1.30713006 |
| Rbm33 | 0.67118 | -0.21656019 |
| Plscr3 | 0.27375 | -0.40316433 |
| 2010320O07Rik | 0.04198 | -0.70284149 |
| Syncrip | 0.0032 | 1.04898044 |
| 4930503B20Rik | 0.00144 | -1.27730451 |
| Pole3 | 0.05958 | -0.35817506 |
| Sphk2 | 0.01285 | -0.94602986 |
| Nbas | 0.92571 | 0.02929348 |
| Nrg3 | 0.0015 | -1.15488942 |
| Dpm2 | 0.03999 | -0.50915021 |
| Dnajb7 | 0.06124 | -2.24524175 |
| Camk1d | 0.28303 | 0.34346783 |
| Mplkip | 0.00144 | 2.44052427 |
| LOC101056001 | 0.00144 | -1.26934956 |
| Ythdc1 | 0.00144 | 2.01170868 |
| AU023762 | 0.00144 | -1.28116048 |
| A1300008O04Rik | 0.00629 | 0.92445448 |
| 1700092C02Rik | 0.04472 | -1.01963123 |
| Gpr139 | 0.21356 | -0.74915713 |
| Tmem171 | 0.07473 | -0.98249171 |
| Ndst1 | 0.20697 | -0.36045835 |
| Zfp691 | 0.00461 | -0.86240733 |
| Gm9871 | 0.00733 | -1.14281464 |
| Cfhr2 | 0.00234 | 1.47983282 |
| Vat1 | 0.00144 | -1.09411516 |
| Gm20276 | 0.00144 | -1.31495282 |
| Nckap1 | 0.00144 | 1.76201932 |
| LOC102639568 | 0.00144 | -1.20555712 |
| 1700049E15Rik | 0.00159 | -1.26431393 |
| Gad1os | 0.00144 | -1.28116048 |
| Pidd1 | 0.03739 | -0.61104947 |
| Ankrd12 | 0.08514 | -0.60312583 |
| Gm29692 | 0.00315 | -1.08432691 |
| Ythdc2 | 0.00144 | 2.1451066 |
| Phf12 | 0.78866 | -0.08195215 |
| Zdhhc15 | 0.70192 | -0.29670031 |
| Chchd7 | 0.00929 | -1.0433256 |
| St6galnac3 | 0.82516 | -0.19149697 |
| Gm2861 | 0.16427 | -0.84223835 |
| Chpt1 | 0.00211 | 1.73989465 |
| LOC102641840 | 0.00144 | -1.5371449 |
| Zfp758 | 0.04338 | -0.97279541 |
| Lama4 | 0.00757 | 1.3900899 |
| Gm9861 | 0.00144 | -1.24811894 |
| Cntn5 | 0.01463 | 0.64139595 |
| Rnf217 | 0.01184 | 1.0493486 |
| Sorbs3 | 0.0099 | -0.57476879 |
| Lrrc29 | 0.93872 | 0.04435594 |
| Acsl4 | 0.01052 | 0.90348351 |
| Rnf43 | 0.00144 | -1.35112763 |
| Dcaf12 | 0.00851 | -0.60117774 |
| Rps4x | 0.0015 | -0.96846517 |
| Limd2 | 0.00144 | -0.97819407 |
| 4933402N03Rik | 0.00333 | -1.13565473 |
| Gm5420 | 0.06567 | -1.51316783 |
| Zfand5 | 0.08237 | 0.54985799 |
| Gm15731 | 0.07192 | -1.17935384 |
| LOC105246502 | 0.00144 | -1.32313902 |
| Pogz | 0.0202 | 0.84701945 |
| Gbas | 0.00812 | 0.68530601 |
| Gm33298 | 0.51038 | -0.16448524 |
| Vgll3 | 0.34784 | 0.56973349 |
| Stx16 | 0.00212 | 0.89425345 |
| 7420700N18Rik | 0.00329 | -0.99496437 |
| Nt5c3 | 0.00276 | 1.38002311 |
| Hs6st1 | 0.01615 | -0.68609926 |
| Npn2 | 0.67066 | -0.10379914 |
| Fam189b | 0.07102 | -0.78642868 |
| Gm20274 | 0.01832 | 1.14992005 |
| Snx8 | 0.00368 | -0.89046874 |
| Rnf212 | 0.912 | -0.05148671 |
| Lrrc20 | 0.05608 | -0.71451849 |
| Gm31019 | 0.00144 | -1.24811894 |
| Syt10 | 0.1605 | -0.64770849 |
| Zfp652os | 0.01751 | -0.79907456 |
| Gm13814 | 0.25958 | -0.75631813 |
| 1600014C10Rik | 0.69136 | 0.18090098 |
| Gm9876 | 0.01904 | -1.27563591 |
| Nalcn | 0.00144 | -1.28116048 |
| LOC100047044 | 0.19441 | 0.4840138 |
| Cdrt4os1 | 0.00144 | -1.30017714 |
| 4933408A14Rik | 0.00144 | -1.28116048 |
| 1700029E06Rik | 0.00144 | -1.29746368 |
| Wdhd1 | 0.00144 | 1.35195377 |
| LOC102641345 | 0.01037 | 1.59503847 |
| Gm8995 | 0.00144 | 5.38422107 |
| Sfpq | 0.36156 | 0.30050349 |
| Gm4285 | 0.07297 | -0.34407119 |
| Gm36793 | 0.00144 | -1.29085005 |
| Zc3h12c | 0.00193 | 0.85992064 |
| 2210408I21Rik | 0.41257 | 0.35422362 |
| Gdf7 | 0.05769 | -2.26431393 |
| Gm36027 | 0.0195 | -0.92886545 |
| Mthfs | 0.09108 | 0.69633614 |
| Gm11872 | 0.00144 | -1.29864962 |
| 4930477O03Rik | 0.92141 | 0.14357939 |
| Lrrn1 | 0.00144 | -1.28898079 |
| Pvt1 | 0.00158 | -1.2446827 |
| 1700016D18Rik | 0.00144 | -1.29085005 |
| Gm6787 | 0.03782 | -0.56294788 |
| Akap10 | 0.02751 | -0.53253423 |
| Lgr6 | 0.08031 | -0.39946105 |
| Gm3255 | 0.45763 | 0.27253739 |
| Aftph | 0.64815 | -0.08255469 |
| Arrdc3 | 0.03648 | 1.14992906 |
| Apex2 | 0.0512 | 1.14463121 |
| Fbxl18 | 0.01584 | -0.58692784 |
| Gm10266 | 0.00144 | -1.29746368 |
| Gdpd1 | 0.98166 | 0.00495485 |
| 4933432G23Rik | 0.11202 | -3.01928546 |
| B230219D22Rik | 0.00144 | -1.25283868 |
| Spdl1 | 0.28683 | -0.30783085 |
| Poc1b | 0.71506 | 0.07511164 |
| Lyrm2 | 0.0667 | -0.33625284 |
| 9930012K11Rik | 0.85488 | 0.05805933 |
| Cfl1 | 0.03601 | -0.44417364 |
| H1fx | 0.00144 | -1.70135606 |
| 9130204L05Rik | 0.45092 | -0.56282236 |
| Gm33989 | 0.85332 | -0.06217566 |
| Gm11412 | 0.00149 | -1.25408 |
| A330084C13Rik | 0.08287 | -0.81730163 |
| Polr3a | 0.00145 | -1.2388121 |
| 4930500G05Rik | 0.00144 | -1.26560808 |
| Ccdc65 | 0.01663 | -1.05114836 |
| Il5ra | 0.00144 | -1.28898079 |
| Gm9734 | 0.09368 | -0.49178933 |
| Acox3 | 0.37074 | -0.19549997 |
| D930028M14Rik | 0.48403 | -0.36082072 |
| Olfr176 | 0.00144 | -1.29746368 |
| Fam117b | 0.04393 | 0.81467283 |
| Gm12927 | 0.00144 | -1.22925777 |
| Rnf133 | 0.18335 | -1.58026244 |
| Snx29 | 0.46924 | 0.37847343 |
| Mtnr1a | 0.00144 | -1.28116048 |
| Dock3 | 0.00252 | -1.036808 |
| 4930522O17Rik | 0.05486 | 0.63451595 |
| Dnajc27 | 0.24626 | 0.28970668 |
| Serpinb10 | 0.95357 | -0.07376589 |
| A1bg | 0.00144 | -1.29085005 |
| Wdr61 | 0.00755 | -1.27353532 |
| Fam78a | 0.2572 | -0.42474054 |
| Lrrc73 | 0.03054 | -1.22696922 |
| Cyb5r4 | 0.01896 | 0.70521561 |
| Adam17 | 0.71048 | -0.06975484 |
| Nfatc2 | 0.84182 | -0.14374826 |
| Fas | 0.67118 | -0.07921517 |
| Acad9 | 0.97734 | -0.00709765 |
| Epb4.2 | 0.00207 | -1.12931932 |
| A130015J22Rik | 0.00144 | -1.30017714 |
| Rapgef4 | 0.05027 | 1.5761316 |
| LOC105243111 | 0.00367 | 0.9373853 |
| Map4k5 | 0.22904 | 0.67253677 |
| Unc80 | 0.05736 | -0.56358572 |
| Olfr847 | 0.03581 | -1.70938368 |
| Sec23a | 0.00824 | 1.58744006 |
| Ocrl | 0.01964 | 1.9030073 |
| Trabd2b | 0.06754 | -0.44870713 |
| Slc25a17 | 0.04392 | 0.39887317 |
| Prkcq | 0.03517 | 0.6047254 |
| Acbd3 | 0.00191 | 0.94884256 |
| Ndufa12 | 0.0087 | -0.77319592 |
| Fto | 0.00403 | -1.44125961 |
| Khk | 0.00207 | -0.98792962 |
| Pou6f2 | 0.00145 | -1.26219879 |
| Lin52 | 0.09419 | -0.80202537 |
| Creb1 | 0.70882 | 0.15464903 |
| Fsip2 | 0.00144 | -1.26560808 |
| Defb10 | 0.00144 | -1.31495282 |
| A430033K04Rik | 0.1263 | 0.58200415 |
| BC043934 | 0.85569 | 0.07924361 |
| Olfr1140 | 0.00144 | -1.25118919 |
| Olfr1410 | 0.048 | 0.91993074 |
| Ears2 | 0.00144 | 1.38285673 |
| Gm19501 | 0.00144 | -1.29746368 |
| Gm34907 | 0.00317 | -1.11935153 |
| Raver2 | 0.01051 | -1.07183535 |
| Slc25a26 | 0.6711 | 0.16823589 |
| Wdr5 | 0.02895 | -0.42473893 |
| Gm4668 | 0.36181 | -0.5045115 |
| Wdr12 | 0.17046 | -0.34745828 |
| Gucd1 | 0.29207 | -0.34975585 |
| 4930488N15Rik | 0.22079 | -0.72068738 |
| 1700046C09Rik | 0.00144 | -1.29746368 |
| LOC102635707 | 0.04505 | -1.27246953 |
| Hnrnph3 | 0.00144 | 1.36651933 |
| Sertad3 | 0.00144 | -1.40935055 |
| Gm35878 | 0.00144 | -1.29746368 |
| Khdc1c | 0.17676 | -1.08422443 |
| Gm5547 | 0.00144 | -1.55648401 |
| Tbc1d9b | 0.86128 | 0.07792556 |
| Cxcr5 | 0.16516 | -0.43100485 |
| Ptgfr | 0.86128 | 0.06373389 |
| Gm9752 | 0.13653 | -2.39916507 |
| Tmem212 | 0.00144 | -1.30017714 |
| 4930539C22Rik | 0.03473 | -0.92545574 |
| Tsc22d2 | 0.00166 | 2.84136322 |
| Mex3a | 0.32668 | -0.22697342 |
| Zfand5 | 0.32492 | -0.55264314 |
| Nek1 | 0.75414 | 0.17788071 |
| Stk36 | 0.19689 | -0.29437684 |
| Map2k7 | 0.61292 | -0.41591576 |
| Stat1 | 0.14441 | 0.44640597 |
| Adamts5 | 0.00144 | 2.42147188 |
| Yipf6 | 0.00144 | -1.26560808 |
| Chd9 | 0.48414 | 0.30190252 |
| Cox16 | 0.48176 | 0.14928499 |
| Wfdc17 | 0.02677 | -0.50511685 |
| Gm19692 | 0.01578 | -1.86775832 |
| Oprm1 | 0.16914 | -0.85980811 |
| Tesc | 0.0031 | -1.28133816 |
| Gm17751 | 0.00144 | -1.29864962 |
| Helb | 0.19879 | 0.4368049 |
| Gm16725 | 0.00144 | -1.25118919 |
| Gm9928 | 0.00144 | -1.46134994 |
| Lhx1os | 0.00144 | -1.29049509 |
| Mterf4 | 0.79822 | -0.04596083 |
| Ergic1 | 0.03188 | -0.57718174 |
| Ndufa3 | 0.00603 | -1.07401276 |
| Hnf4a | 0.00144 | -1.2383131 |
| Kmt2c | 0.05515 | -0.56092039 |
| Shb | 0.3229 | -0.46214826 |
| Gm8221 | 0.01709 | -2.05749478 |
| Paf1 | 0.08138 | 0.46338413 |
| Cic | 0.0192 | -0.54625833 |
| Tfb2m | 0.07701 | 0.53653566 |
| Itgb1 | 0.16689 | -0.32458223 |
| Rimkla | 0.02739 | 0.50835784 |
| Gm30963 | 0.00144 | -1.30017714 |
| Cerkl | 0.00145 | -1.26219879 |
| Nkap | 0.00314 | 0.93171108 |
| Slc24a5 | 0.00255 | 1.75767283 |
| Vmn1r23 | 0.00144 | -1.27165604 |
| Ifng | 0.00184 | -1.19286777 |
| Defb14 | 0.00145 | -1.27532353 |
| Frmd4b | 0.02712 | -0.68153839 |
| Lyve1 | 0.00571 | 1.261728 |
| Gdf11 | 0.68982 | -0.14317902 |
| Nfam1 | 0.61289 | -0.37583981 |
| Ttll4 | 0.14551 | 0.38745863 |
| Pip | 0.00144 | -1.28116048 |
| Olfr366 | 0.00144 | -1.25118919 |
| Hhipl1 | 0.29441 | 0.3360484 |
| Plb1 | 0.0078 | -0.92501938 |
| Prrc2b | 0.32203 | -0.18958889 |
| Phf2 | 0.80556 | -0.08359307 |
| Mettl7b | 0.02656 | -1.43545555 |
| Srsf11 | 0.00144 | 1.68340385 |
| Ppig | 0.17237 | 0.35449252 |
| Tmem245 | 0.00144 | 1.28643277 |
| Cct3 | 0.02265 | 0.78512049 |
| 4833405L11Rik | 0.00144 | -1.29085005 |
| Myo10 | 0.36079 | 0.15022369 |
| Prrg1 | 0.00144 | -1.28116048 |
| Gm5087 | 0.00144 | -1.24811894 |
| Senp6 | 0.0239 | 0.94887371 |
| 4930539J05Rik | 0.01763 | 1.57576677 |
| Ptprd | 0.00144 | -1.26560808 |
| Srek1 | 0.00144 | 1.40877247 |
| Tapbp | 0.00552 | -0.84228338 |
| Aars | 0.00158 | -1.15468698 |
| Sltm | 0.00597 | 1.56693087 |
| Ptk2b | 0.00144 | -1.52015662 |
| Ankmy2 | 0.27066 | 0.30897885 |
| 4932435O22Rik | 0.81251 | -0.20875297 |
| Slit2 | 0.00144 | -1.26560808 |
| Ptdss2 | 0.74997 | 0.15954034 |
| Gpx1 | 0.02912 | -0.59300205 |
| 4933432I09Rik | 0.01507 | -0.87183465 |
| Dpf3 | 0.02601 | -0.74989672 |
| 2510002D24Rik | 0.57701 | -0.09371917 |
| Atoh7 | 0.43158 | -0.46433706 |
| 1110014L15Rik | 0.00512 | -1.06802371 |
| Snupn | 0.12391 | -0.26433002 |
| Arcn1 | 0.09376 | -0.37285394 |
| H2-Ab1 | 0.00446 | -1.05317671 |
| 4921517D22Rik | 0.00158 | -1.2446827 |
| Capn5 | 0.00676 | -0.73913543 |
| Esyt2 | 0.00144 | -1.26934956 |
| Scrib | 0.01552 | -0.69768556 |
| Fam13b | 0.01082 | 0.95640123 |
| Taok3 | 0.09045 | -0.4695786 |
| Cdip1 | 0.02392 | -0.5245039 |
| Cdh11 | 0.01802 | 0.82849519 |
| Mccc2 | 0.21281 | 0.39290824 |
| Dlgap2 | 0.00145 | -1.18741414 |
| Timm50 | 0.03563 | -0.82790772 |
| Mfsd9 | 0.09738 | -0.64718458 |
| Xk | 0.0037 | 1.75502741 |
| Icos | 0.00595 | 0.96415283 |
| Dock4 | 0.00144 | 1.300555 |
| Ncapd2 | 0.45257 | 0.39162946 |
| Mcrs1 | 0.17115 | -0.52065101 |
| Uimc1 | 0.02192 | 1.03265839 |
| Scd1 | 0.73232 | 0.10825282 |
| Tmem44 | 0.02124 | -0.77065821 |
| Rnf213 | 0.02297 | -0.63709653 |
| Psd3 | 0.03198 | 0.47135908 |
| 4933421O10Rik | 0.31562 | 0.54317784 |
| Gria3 | 0.04294 | 0.737267 |
| Arid4b | 0.00144 | 2.22591032 |
| Fam149b | 0.25279 | 0.31285928 |
| Rprd2 | 0.07977 | 0.31871054 |
| Gpr107 | 0.02691 | -0.800738 |
| Eif2ak1 | 0.71722 | -0.21805381 |
| Cybrd1 | 0.0198 | -1.00128411 |
| Naaladl2 | 0.35722 | 0.32656719 |
| Rftn2 | 0.13395 | -0.30669106 |
| G0s2 | 0.02294 | -0.89740802 |
| Plaur | 0.35816 | -0.33943662 |
| Cnot4 | 0.00144 | 2.14861265 |
| Ghr | 0.1056 | 0.51390825 |
| Ctxn1 | 0.86401 | -0.03662109 |
| Enpp6 | 0.0446 | -1.02147729 |
| Srpk2 | 0.6687 | -0.07387155 |
| Fam69c | 0.0094 | -1.09535421 |
| Tdrd6 | 0.00145 | -1.24082201 |
| Kpna3 | 0.00398 | 1.2304093 |
| Olfr1076 | 0.06483 | -0.88148784 |
| Gm15672 | 0.00954 | 1.10780234 |
| Pdcd10 | 0.00165 | 1.14827576 |
| Rab11fip1 | 0.0789 | -0.86363514 |
| Bap1 | 0.00407 | -0.70819742 |
| Ace | 0.00618 | -0.70773509 |
| Cdk16 | 0.55729 | -0.23583233 |
| Slmap | 0.58835 | -0.12897113 |
| Eif4a3 | 0.36213 | -0.25396612 |
| Fmo6 | 0.00158 | -1.2446827 |
| Lcmt2 | 0.03925 | -0.6996399 |
| Rbm22 | 0.75613 | -0.06373871 |
| Cat | 0.07299 | 0.67340463 |
| 4930538K18Rik | 0.90499 | -0.02118424 |
| 5031434C07Rik | 0.02761 | -1.06935338 |
| Ss18 | 0.00144 | 1.18243127 |
| Slc35f6 | 0.066 | -0.38090444 |
| Clk1 | 0.00144 | 2.72936821 |
| Prss23 | 0.8516 | 0.04713262 |
| Tjp2 | 0.05551 | -0.35592776 |
| Nav2 | 0.86638 | -0.13849382 |
| LOC102635912 | 0.768 | 0.15160746 |
| Dynlt3 | 0.00144 | 1.46844869 |
| Rttn | 0.00149 | -1.25408 |
| Ctsm | 0.40065 | -0.30498797 |
| Rft1 | 0.0075 | -1.08536055 |
| Slc22a14 | 0.00144 | -1.28116048 |
| Xpr1 | 0.01755 | 0.76534043 |
| Pex11g | 0.034 | -0.68013131 |
| Itsn2 | 0.00144 | 2.37930916 |
| Trmt44 | 0.22908 | -0.33090081 |
| Tmem5 | 0.02082 | 0.85734024 |
| Cdh8 | 0.00144 | -1.31495282 |
| Senp7 | 0.41443 | -0.17029125 |
| Gm12830 | 0.89596 | -0.16593685 |
| Ttll3 | 0.42229 | -0.94765733 |
| Pkn1 | 0.09924 | -0.64593339 |
| Prmt5 | 0.33093 | -0.25376447 |
| Ptprc | 0.00878 | 1.20790784 |
| Tirap | 0.85697 | -0.02996322 |
| Tacr3 | 0.00144 | -1.25118919 |
| Bnc1 | 0.00144 | -1.31495282 |
| Gigyf1 | 0.00258 | -0.90231059 |
| Gpr37 | 0.87592 | 0.26935077 |
| Stra6l | 0.00144 | -1.28116048 |
| Trpm2 | 0.52738 | 0.35508072 |
| Zfp518a | 0.00357 | 0.93675747 |
| Fzd8 | 0.00596 | 0.83598492 |
| B830004H01Rik | 0.09928 | -2.59642888 |
| Mtor | 0.00245 | -1.03529907 |
| Cdc37l1 | 0.00144 | -1.1796145 |
| Sc5d | 0.00144 | 1.70339476 |
| Itpripl1 | 0.1857 | -0.37539834 |
| Dzip1l | 0.00602 | -1.12831388 |
| Tmem126b | 0.10239 | 0.83463519 |
| Klra5 | 0.00145 | -1.2388121 |
| Cers4 | 0.00144 | 2.21600567 |
| Agbl3 | 0.11397 | 0.32533804 |
| Crb1 | 0.03513 | 0.56635349 |
| Ghrl | 0.017 | 1.06678926 |
| Fat4 | 0.52893 | 0.21737886 |
| Zfp97 | 0.77641 | -0.07351504 |
| Fhad1 | 0.04415 | -2.02369437 |
| Azi2 | 0.10133 | 0.5854786 |
| Chrdl1 | 0.31698 | -2.1009675 |
| Pnpla6 | 0.10675 | -0.39687924 |
| Btbd3 | 0.00241 | 1.05446856 |
| Blmh | 0.11821 | 0.37224193 |
| Arih1 | 0.18992 | 0.31449364 |
| Mkln1 | 0.00144 | 1.12394651 |
| Thtpa | 0.90699 | -0.03296158 |
| 9130230L23Rik | 0.00144 | -1.30017714 |
| Egfr | 0.77889 | 0.27068253 |
| 3222401L13Rik | 0.01271 | 0.658728 |
| Mocs2 | 0.00376 | 0.72199083 |
| A830010M09Rik | 0.00145 | -1.2551153 |
| Kif17 | 0.00144 | -1.30713006 |
| Rsbn1l | 0.00379 | 1.29715962 |
| Rbfox3 | 0.32965 | -0.5383764 |
| Nsmaf | 0.09822 | -0.61624603 |
| Glce | 0.06712 | -0.75222507 |
| Stag1 | 0.6504 | 0.14545587 |
| Cnot10 | 0.20557 | 0.47688729 |
| Csmd1 | 0.00158 | -1.2446827 |
| Cdkn1b | 0.02615 | 0.50468547 |
| 5330439M10Rik | 0.00144 | -1.3230041 |
| Mrvi1 | 0.0065 | -1.5619132 |
| Nsa2 | 0.10911 | 0.81476363 |
| Shmt1 | 0.92376 | 0.03651321 |
| Reep3 | 0.43427 | -0.27400571 |
| 2610044O15Rik8 | 0.00158 | 0.86183455 |
| Maz | 0.0212 | -0.75065975 |
| Tph2 | 0.00144 | -1.28898079 |
| Jakmip1 | 0.00381 | -1.09251133 |
| 4930515G01Rik | 0.15922 | -0.58318136 |
| Gpr45 | 0.00144 | -1.28116048 |
| Olfr974 | 0.08319 | -0.89043112 |
| Aamdc | 0.06407 | -0.52556564 |
| G630025P09Rik | 0.00149 | -1.2703832 |
| Prpf3 | 0.00254 | 0.95716413 |
| Ascc1 | 0.00416 | -0.7492349 |
| Zmat4 | 0.00257 | -1.86561706 |
| Exoc5 | 0.01551 | 0.89155965 |
| 2900052N01Rik | 0.00258 | -1.61971066 |
| Stt3b | 0.01301 | 0.60145951 |
| Phka2 | 0.9684 | 0.00653063 |
| Adamts10 | 0.9938 | -0.00218601 |
| Arhgap18 | 0.00144 | 2.69053672 |
| Acad12 | 0.00693 | -0.84513493 |
| Rasgrf1 | 0.00144 | -1.28116048 |
| Trim45 | 0.13166 | -0.5184816 |
| Pomc | 0.01471 | -0.72215563 |
| Abhd8 | 0.00144 | -1.33854563 |
| Zmat1 | 0.00144 | 2.86017844 |
| 9930021J03Rik | 0.68832 | -0.10919545 |
| B3galt2 | 0.00144 | -1.26301121 |
| Gtf2b | 0.03382 | -0.98711279 |
| Eif4e | 0.00605 | -0.68552417 |
| Zmynd19 | 0.13238 | -0.34410244 |
| Pdcl | 0.00146 | -1.09165805 |
| Nme4 | 0.88217 | -0.08375153 |
| Ebag9 | 0.00144 | 1.42851492 |
| Snhg18 | 0.00583 | -0.6940272 |
| 1700030J22Rik | 0.39475 | 0.26437353 |
| 2210409E12Rik | 0.22504 | -0.48689754 |
| Meis1 | 0.11649 | -0.67048638 |
| Kif14 | 0.03869 | -0.92436403 |
| Tfpi | 0.00691 | 1.05663922 |
| Kpnb1 | 0.00604 | 0.72710661 |
| Ccdc42 | 0.00144 | -1.28898079 |
| 1700025H01Rik | 0.00145 | -1.25200268 |
| Rsad2 | 0.01216 | 1.16848476 |
| Cchcr1 | 0.54149 | -0.12156498 |
| Hamp | 0.60259 | -1.12708657 |
| LOC102637912 | 0.00144 | -1.29085005 |
| Slc5a1 | 0.00601 | -1.09465341 |
| Col25a1 | 0.00144 | -1.28116048 |
| Lrat | 0.88407 | 0.21267823 |
| Tmem243 | 0.86652 | -0.03376584 |
| Trappc12 | 0.28818 | -0.33494079 |
| D930015E06Rik | 0.6278 | -0.20087258 |
| 9330154J02Rik | 0.00144 | -1.26560808 |
| A530088E08Rik | 0.00144 | -1.26934956 |
| Igf1r | 0.75896 | 0.05677027 |
| Dph6 | 0.43054 | 0.30330425 |
| Megf11 | 0.02327 | -1.24021765 |
| LOC105243401 | 0.12105 | -2.62496366 |
| Git2 | 0.86735 | -0.11371934 |
| Cpa5 | 0.09489 | -2.59058452 |
| 4933400F21Rik | 0.90917 | -0.12385295 |
| Ncam1 | 0.00145 | -1.22465584 |
| Ccpg1 | 0.1581 | -0.29225788 |
| Asb1 | 0.01236 | -0.89593506 |
| Mrpl44 | 0.28821 | 0.8328279 |
| Tcte2 | 0.00145 | -1.12217966 |
| Hspa4l | 0.00144 | 1.4035558 |
| Pcdhga9 | 0.00144 | -2.01847325 |
| Inadl | 0.02645 | -0.97091751 |
| Fyco1 | 0.0017 | 1.82564436 |
| Cers6 | 0.25273 | -0.29172725 |
| Ttc19 | 0.06757 | 0.50730599 |
| Tpt1 | 0.44933 | -0.15263634 |
| 6720468P15Rik | 0.00144 | -1.26934956 |
| Thra | 0.28382 | -0.37365418 |
| Ksr1 | 0.05576 | -0.55516665 |
| Ptpn6 | 0.55908 | -0.20443778 |
| 4930519H02Rik | 0.1957 | 0.44075101 |
| Ttl | 0.01747 | -1.28277862 |
| Ildr1 | 0.00145 | -1.22376446 |
| Dmrtb1 | 0.00144 | -1.43497389 |
| Gck | 0.41026 | -0.4003978 |
| Cox18 | 0.78252 | -0.0957284 |
| Tssk1 | 0.00144 | -1.26560808 |
| Abca1 | 0.0027 | 1.37872867 |
| Adarb1 | 0.10122 | -0.7108955 |
| Dnajc10 | 0.18011 | -0.25309998 |
| Ube2m | 0.06345 | -0.71397034 |
| Fam207a | 0.02692 | -0.62475587 |
| Med13 | 0.00144 | 1.69494306 |
| Ric3 | 0.00144 | -1.30713006 |
| Ptpdc1 | 0.00216 | -1.10092326 |
| Gm4827 | 0.00144 | -1.24613586 |
| Elavl2 | 0.751 | 0.11891928 |
| Wiz | 0.0113 | -1.16395103 |
| Ppp1r1b | 0.09477 | -0.72944084 |
| Csf2rb | 0.24461 | -0.41923201 |
| Spef2 | 0.00144 | -1.29864962 |
| Zfp472 | 0.00144 | 1.8164423 |
| Zfp191 | 0.03567 | 0.45086277 |
| Abhd2 | 0.03758 | -0.72445013 |
| Slu7 | 0.47895 | 0.21307797 |
| Zfp286 | 0.12385 | 1.1606637 |
| Upf2 | 0.25819 | 0.66168741 |
| Zfp560 | 0.22443 | -1.28997336 |
| Nedd1 | 0.00145 | -1.0026092 |
| Tnik | 0.00144 | 1.18018859 |
| Ganc | 0.04223 | 0.42579243 |
| Nlrx1 | 0.59708 | -0.28943926 |
| Podnl1 | 0.15202 | -1.51329382 |
| Pak1 | 0.00671 | 0.98748411 |
| Cntnap4 | 0.03713 | -0.75160463 |
| Gad1 | 0.00144 | -1.20555712 |
| Nmrk1 | 0.00215 | 1.49135275 |
| P4ha2 | 0.07439 | -0.34763953 |
| Jagn1 | 0.81887 | 0.09961598 |
| 2010109A12Rik | 0.55863 | 0.22027891 |
| Wdr60 | 0.13531 | 0.54557287 |
| Angptl2 | 0.36027 | -0.49600026 |
| Zfp367 | 0.06769 | -0.79412132 |
| Psmf1 | 0.75668 | -0.14237587 |
| Dgke | 0.01067 | -1.13630513 |
| Stat5b | 0.03279 | -0.69622117 |
| 1700083H02Rik | 0.77448 | -0.15266321 |
| 9430021M05Rik | 0.00145 | -1.24191372 |
| Spaca1 | 0.00144 | -1.28898079 |
| Olfr701 | 0.00258 | -1.16351105 |
| Anxa13 | 0.09508 | 0.85653749 |
| Cdhr3 | 0.03641 | 0.49000891 |
| Slc25a47 | 0.2083 | -0.29928221 |
| BC061212 | 0.00144 | -1.28116048 |
| Obsl1 | 0.01634 | -0.59443669 |
| Ccdc141 | 0.0487 | -0.46187443 |
| Gen1 | 0.06938 | -1.1137284 |
| Gm136 | 0.0221 | -1.21356835 |
| Snhg3 | 0.15897 | -0.27113717 |
| Ccr3 | 0.22981 | -0.52148735 |
| Tmem220 | 0.01368 | 1.03718703 |
| Plxna4 | 0.18145 | 0.36027565 |
| Ctsf | 0.20375 | -0.49724596 |
| Eif3a | 0.49649 | -0.27437408 |
| Dsel | 0.00144 | 1.47920576 |
| Il7 | 0.00144 | -1.22925777 |
| Mip | 0.00144 | -1.20555712 |
| Trmt6 | 0.00144 | 1.11375882 |
| 4932441J04Rik | 0.03711 | 1.30255979 |
| Isy1 | 0.73542 | 0.0579175 |
| Ano6 | 0.16452 | 0.28406272 |
| 1810034E14Rik | 0.8304 | 0.1321484 |
| Glb1 | 0.14101 | -0.33016317 |
| Bclaf1 | 0.01761 | 0.88345965 |
| Rgs9bp | 0.00144 | -1.25118919 |
| Spsb4 | 0.04517 | -0.62859005 |
| Man2a2 | 0.51909 | -0.25379349 |
| Ppat | 0.04051 | 0.55591891 |
| Cyp4a10 | 0.01765 | -1.02766312 |
| Fnip1 | 0.00303 | 1.0224839 |
| Lhb | 0.00144 | -1.31495282 |
| Mfsd7a | 0.03435 | -0.50281517 |
| Ogt | 0.01044 | -1.0238672 |
| C130030K03Rik | 0.00145 | -1.22518835 |
| Ets2 | 0.09358 | -0.31891989 |
| Trappc8 | 0.00144 | -1.26560808 |
| Tspyl1 | 0.77812 | -0.04349688 |
| Phka2 | 0.64482 | -0.19791434 |
| Zcchc8 | 0.14646 | -2.53526926 |
| Zbtb20 | 0.09851 | -0.39443421 |
| Mapk8 | 0.0142 | 1.65810445 |
| Cep152 | 0.00455 | 1.25475375 |
| Asxl3 | 0.00145 | -1.2388121 |
| Mctp1 | 0.28996 | -0.41672968 |
| Fxr1 | 0.00684 | 0.81389253 |
| Gsdmcl1 | 0.44327 | -0.32722624 |
| Psmc5 | 0.03527 | -0.39282377 |
| 4933406B17Rik | 0.00144 | -1.26934956 |
| Tnni1 | 0.07278 | -1.45518077 |
| Tmem160 | 0.0024 | -1.20857512 |
| Trim39 | 0.00186 | 1.06395013 |
| Cdc40 | 0.37472 | 0.14660542 |
| Eme2 | 0.71217 | -0.06138073 |
| Mfsd11 | 0.29331 | -0.37152399 |
| Fam98b | 0.01337 | 0.76838994 |
| Slc25a30 | 0.55633 | -0.31082491 |
| Mtnr1a | 0.00144 | -1.20555712 |
| Ctf2 | 0.01251 | -0.84225749 |
| Map3k5 | 0.02438 | -0.59361452 |
| Actn3 | 0.00225 | -1.40363226 |
| Slc22a5 | 0.02826 | -0.54396143 |
| Pex6 | 0.00512 | -0.9772239 |
| Ogt | 0.02963 | 0.56996608 |
| Met | 0.16869 | 0.98111089 |
| Sirt2 | 0.99333 | 0.00343175 |
| BC030336 | 0.00235 | 0.98729612 |
| Wdhd1 | 0.14839 | 0.36881305 |
| Ank1 | 0.66138 | 0.16853639 |
| Rpe | 0.01283 | 1.03001031 |
| Chpt1 | 0.32318 | 0.33465743 |
| Egfl6 | 0.00144 | -1.33632959 |
| Smarcal1 | 0.43625 | 0.25672629 |
| Alk | 0.00144 | -1.28898079 |
| Klhl20 | 0.00144 | -1.29746368 |
| Zfp462 | 0.75637 | -0.16870135 |
| Mas1 | 0.12343 | 0.34711609 |
| Bora | 0.02244 | 0.65858564 |
| Rhot1 | 0.38582 | 0.48011298 |
| LOC102631757 | 0.00255 | -1.40334014 |
| Nlrc4 | 0.01713 | 1.26860969 |
| Grik2 | 0.1146 | -2.81786459 |
| Tbc1d17 | 0.01609 | -0.62217408 |
| Cxxc4 | 0.00797 | 1.49300511 |
| Eif3j2 | 0.02011 | -0.99696012 |
| Iqgap2 | 0.03732 | -0.59967552 |
| Iqgap2 | 0.00144 | -1.30626366 |
| Trub1 | 0.01903 | -0.65937254 |
| 1500004A13Rik | 0.05675 | -0.93227475 |
| Trim69 | 0.00144 | 2.0190876 |
| Pisd-ps3 | 0.24108 | -0.46492992 |
| Znhit3 | 0.76976 | 0.12841181 |
| Cmss1 | 0.0105 | -0.67718175 |
| Dmbx1 | 0.00158 | -1.2446827 |
| Mypopos | 0.00453 | -1.78654245 |
| Trim25 | 0.14665 | -0.31136205 |
| Oaz2 | 0.59759 | -0.16842424 |
| Utrn | 0.00149 | -1.25408 |
| Msto1 | 0.0018 | -0.9804803 |
| Tex261 | 0.76962 | -0.16446643 |
| Atf7ip | 0.29618 | -0.22065583 |
| Thap2 | 0.00171 | 1.64955558 |
| Olfr262 | 0.00144 | -1.27165604 |
| Keg1 | 0.15332 | -0.7789147 |
| Rab7 | 0.00639 | -0.73913816 |
| Eprs | 0.13075 | 0.36955717 |
| Atp1a3 | 0.02488 | -1.11967074 |
| Fbxw14 | 0.00201 | -1.11996532 |
| Lrrc39 | 0.0062 | 0.82072778 |
| Ltbp3 | 0.02662 | -0.54262661 |
| Akip1 | 0.07929 | -0.35343817 |
| Zbtb46 | 0.97414 | -0.02540727 |
| Lrp1b | 0.00144 | -1.37833222 |
| Thnsl1 | 0.00799 | 1.47242883 |
| Hps1 | 0.55179 | -0.44135461 |
| Pcm1 | 0.02951 | 1.36122603 |
| Lman1 | 0.17161 | 0.3047946 |
| AI314180 | 0.01685 | 1.21822941 |
| Zfp541 | 0.1456 | -3.34348131 |
| Lhx1 | 0.00144 | -1.41392798 |
| Mapre2 | 0.21477 | -0.23392158 |
| Trim24 | 0.00144 | 1.99227364 |
| Snrpa1 | 0.00144 | 1.7295754 |
| Psmd14 | 0.00498 | 0.80626015 |
| Sar1a | 0.92454 | -0.01752143 |
| Brd3 | 0.02169 | -0.54371299 |
| Pet100 | 0.0671 | -0.763918 |
| Cyp2c65 | 0.17984 | -0.99516681 |
| 4933427I18Rik | 0.00144 | -1.30017714 |
| Minpp1 | 0.80251 | 0.40438926 |
| Fam229b | 0.43711 | 0.29209892 |
| Rpl23 | 0.64514 | -0.07701048 |
| Soat1 | 0.14927 | -0.45689932 |
| C630004L07Rik | 0.01673 | -0.83267562 |
| Epha10 | 0.00162 | -1.30069119 |
| Alg8 | 0.04352 | 0.76457391 |
| Zdhhc2 | 0.0076 | 1.32797508 |
| Gm6994 | 0.00945 | -0.8752015 |
| Tmx1 | 0.00144 | 1.91546336 |
| Mrc2 | 0.72253 | 0.0969608 |
| Dyrk1a | 0.05948 | 0.41674678 |
| Myo1d | 0.11891 | -0.31697464 |
| Barhl1 | 0.00158 | -1.2446827 |
| Coq10b | 0.02578 | 0.76518072 |
| Nlrp3 | 0.5017 | 0.33821559 |
| Prox2 | 0.04205 | -1.17660698 |
| Slc39a13 | 0.0086 | -0.80467474 |
| Spink4 | 0.00497 | -1.16767019 |
| Ccnjl | 0.80014 | -0.05605566 |
| Dnah12 | 0.00144 | -1.30713006 |
| Slc35e1 | 0.51117 | -0.21169378 |
| Mrps5 | 0.5833 | 0.18259674 |
| Nipal2 | 0.91729 | -0.03817613 |
| Uri1 | 1 | -0.00000275 |
| Dock10 | 0.00144 | 1.80942181 |
| Vps29 | 0.47286 | 0.40546684 |
| Il23a | 0.00144 | -1.27165604 |
| Zfp91 | 0.1738 | 0.25848076 |
| Hecw1 | 0.00145 | -1.24191372 |
| Aipl1 | 0.00144 | -1.31884045 |
| Heg1 | 0.05234 | -0.43952333 |
| 3110052M02Rik | 0.09864 | 1.29364911 |
| Sdf4 | 0.09039 | -0.40107798 |
| Cdc26 | 0.28848 | -0.18005233 |
| Xrcc6bp1 | 0.55667 | 0.33920045 |
| Elac2 | 0.12004 | -0.33686906 |
| A230050P20Rik | 0.0265 | -1.30951526 |
| Insr | 0.21839 | -0.24961796 |
| Brpf3 | 0.46456 | -0.9793088 |
| St6galnac2 | 0.01911 | -1.6343057 |
| Cep250 | 0.44878 | -0.28329271 |
| Zc3h12d | 0.94936 | -0.03043945 |
| Dusp9 | 0.00144 | -1.24865146 |
| Gorab | 0.00145 | 1.85781596 |
| Tex13 | 0.00144 | -1.27165604 |
| Pigs | 0.01506 | -0.86301516 |
| Vmn2r29 | 0.00204 | -1.76104648 |
| Klhdc4 | 0.00144 | -1.28116048 |
| Gna12 | 0.09749 | -0.57650946 |
| Klhl41 | 0.01107 | -0.71866858 |
| Gen1 | 0.13311 | -0.42666492 |
| Ubr4 | 0.00547 | -0.68225147 |
| Hsf1 | 0.02193 | -0.58258982 |
| Uncx | 0.86263 | 0.05236751 |
| Gspt2 | 0.04536 | -2.27293173 |
| Clip4 | 0.00157 | -0.89194879 |
| Svep1 | 0.0843 | -1.22916586 |
| Foxa3 | 0.0551 | -1.6031247 |
| Ankrd52 | 0.52695 | -0.39445918 |
| D16Ertd472e | 0.77303 | 0.0681553 |
| Grpr | 0.00144 | -1.72128008 |
| Invs | 0.78959 | 0.10556382 |
| Camk2n1 | 0.14569 | -0.29295841 |
| Car12 | 0.00144 | -1.31495282 |
| Samsn1 | 0.17515 | 0.38018669 |
| Fbxl22 | 0.02604 | -0.97769414 |
| Smoc2 | 0.01006 | -0.69069866 |
| Tex30 | 0.00227 | 1.02418907 |
| Commd7 | 0.00711 | -0.76146955 |
| Klhl28 | 0.66422 | 0.08184189 |
| 9030624J02Rik | 0.03509 | -0.54130112 |
| Olfr128 | 0.00267 | -1.01564035 |
| Gtpbp10 | 0.2811 | 0.90769153 |
| Clnk | 0.02899 | -0.67908402 |
| Plekha5 | 0.07203 | -0.46052955 |
| Rps13 | 0.14695 | 0.60486537 |
| Zfp609 | 0.01893 | -1.58427037 |
| Nfatc2ip | 0.92274 | -0.01587536 |
| Irx3os | 0.1299 | -0.77272532 |
| Agtr1a | 0.06653 | 0.64360283 |
| Stbd1 | 0.01622 | 0.80856847 |
| Olfr523 | 0.72491 | -0.18164261 |
| D5Ertd615e | 0.01685 | -1.31586668 |
| Senp8 | 0.00659 | 1.57862725 |
| Apobec3 | 0.07172 | 0.89663245 |
| Adipor1 | 0.55032 | -0.10507948 |
| Herc6 | 0.00302 | 1.45618309 |
| Sec24a | 0.00144 | 1.81549745 |
| Mzt1 | 0.00144 | 0.99985505 |
| Zbtb39 | 0.96886 | 0.00715462 |
| Exoc4 | 0.00144 | -1.28116048 |
| Proser1 | 0.02497 | -0.4671545 |
| Wdr33 | 0.14987 | -0.78389181 |
| Gpc5 | 0.00144 | -1.27165604 |
| Zzef1 | 0.03802 | -0.40289882 |
| Flt3l | 0.40303 | -0.22665208 |
| Trappc6b | 0.00144 | 1.66828523 |
| Sult1b1 | 0.00258 | -1.07351702 |
| Prl8a1 | 0.00144 | -1.29085005 |
| Dmd | 0.1119 | 0.79174797 |
| Dhrsx | 0.50834 | -0.1678406 |
| Dock9 | 0.0065 | 0.81954235 |
| Zfp106 | 0.00144 | 1.52700981 |
| Acta2 | 0.00144 | -1.31518407 |
| Cul9 | 0.00144 | -0.97837555 |
| 4930524O08Rik | 0.00144 | -1.29085005 |
| 1700018L02Rik | 0.08556 | -0.61083182 |
| Fem1c | 0.03876 | 0.59627299 |
| 1700028E10Rik | 0.00144 | -1.29746368 |
| Mob1b | 0.00221 | -1.06220763 |
| Snord33 | 0.77841 | -0.06254412 |
| Crybb1 | 0.02536 | -1.04302558 |
| Mogat1 | 0.11553 | -0.33653428 |
| Sparcl1 | 0.02708 | 0.77248674 |
| Scyl2 | 0.11535 | -0.37804143 |
| Ankle2 | 0.05562 | 0.37754071 |
| Samd4 | 0.03404 | 0.57180859 |
| Olfr315 | 0.00144 | -1.31495282 |
| Snora52 | 0.00773 | -0.73173033 |
| Lats1 | 0.47968 | 0.17295526 |
| Efhd1 | 0.55161 | 0.24246892 |
| Rbm20 | 0.00317 | -0.81885561 |
| Kcnk1 | 0.25507 | -0.70613157 |
| Obscn | 0.51288 | -0.19699632 |
| Gm9059 | 0.10141 | -2.80016858 |
| Mkrn1 | 0.34197 | -0.33384159 |
| Mapk10 | 0.01778 | -1.38911702 |
| Soat1 | 0.10082 | -0.63174397 |
| Prpsap1 | 0.2384 | -0.25893349 |
| Casc1 | 0.00144 | -1.31884045 |
| BC006965 | 0.00144 | -1.28116048 |
| Camta1 | 0.02414 | 0.54767433 |
| Nhlrc3 | 0.00591 | -0.91321223 |
| Hacd3 | 0.58485 | -0.08403668 |
| Khnyn | 0.02646 | -0.45789617 |
| Simc1 | 0.00155 | 1.40835552 |
| Ggct | 0.0595 | 0.35366869 |
| Sh2d1a | 0.00145 | -1.26219879 |
| Themis | 0.00314 | -1.14703327 |
| Nfia | 0.0194 | 0.5490353 |
| Zc3h7a | 0.31449 | 0.34664795 |
| Wars2 | 0.2873 | 0.25599951 |
| Pdilt | 0.00144 | -1.28116048 |
| Galk2 | 0.06708 | 0.3486328 |
| 1700029J07Rik | 0.00371 | 2.44623657 |
| Sox14 | 0.84428 | 0.38841413 |
| Olfr774 | 0.00144 | -1.26560808 |
| Dcdc2a | 0.23137 | -0.64335754 |
| Gdpd4 | 0.34856 | -0.54818332 |
| Gnb4 | 0.44091 | 0.14150859 |
| Tpr | 0.00514 | 0.73947852 |
| Atp5h | 0.00781 | -0.69834504 |
| Olfr68 | 0.51428 | -0.54097678 |
| Fmnl2 | 0.84182 | -0.07388417 |
| Slc22a18 | 0.11576 | -0.62780305 |
| Stam | 0.00144 | 1.77203517 |
| Ing4 | 0.00144 | -1.44333429 |
| Gart | 0.09511 | 0.81285798 |
| Btbd16 | 0.05729 | -0.89604186 |
| Akap14 | 0.00144 | -1.30017714 |
| Gm4907 | 0.10319 | -0.84326464 |
| Ate1 | 0.00218 | 0.98816011 |
| Mff | 0.64797 | -0.15883504 |
| Mms19 | 0.00144 | -1.28898079 |
| Kcnk3 | 0.42199 | -0.42312232 |
| 4930570G19Rik | 0.03604 | -1.89592444 |
| Fgd4 | 0.01787 | 0.59010324 |
| Bche | 0.00145 | -0.9478779 |
| Slc9a8 | 0.84151 | -0.10536468 |
| Usp15 | 0.00144 | 2.18408202 |
| Lrrc14 | 0.05361 | -0.80731808 |
| Ddc | 0.39232 | 0.86358058 |
| Adra1b | 0.41284 | -0.45174829 |
| Glt8d2 | 0.0017 | -1.24950944 |
| Myt1l | 0.3537 | -0.40746414 |
| Tmem57 | 0.27069 | -0.18782289 |
| Vegfa | 0.00144 | 1.63982929 |
| Xpo5 | 0.0026 | -1.0935788 |
| Mipol1 | 0.1904 | -0.34013512 |
| Stk31 | 0.00221 | -1.10308723 |
| Phf14 | 0.00144 | 2.16282012 |
| Ap1m2 | 0.00144 | -1.27165604 |
| S100a7a | 0.00144 | -1.28898079 |
| Cfap74 | 0.00144 | -1.31014844 |
| Zbtb11os1 | 0.17876 | -0.35646895 |
| Fam110b | 0.0017 | -1.24950944 |
| Ccl5 | 0.00144 | -1.82558151 |
| Scimp | 0.3375 | -0.63408709 |
| Fam71f1 | 0.02469 | -0.82741985 |
| Gm13583 | 0.00395 | -1.19099662 |
| Slc1a1 | 0.56108 | 0.17304263 |
| Entpd6 | 0.79173 | 0.12672822 |
| Ubl4 | 0.00144 | -1.02137028 |
| Spred1 | 0.15514 | 0.33087947 |
| Eif4g3 | 0.00272 | 0.77196559 |
| Wdr25 | 0.26995 | -0.29221957 |
| Ctsa | 0.28504 | -0.30487027 |
| Zbtb43 | 0.5843 | 0.1348132 |
| Wipf1 | 0.01731 | 0.72272311 |
| Osr1 | 0.00546 | -1.63518399 |
| AU018823 | 0.83672 | -0.06517267 |
| Chordc1 | 0.02846 | -0.69986776 |
| Pyroxd2 | 0.00461 | 0.74394881 |
| Fam49b | 0.00144 | 1.24734916 |
| Pdgfd | 0.00587 | 1.76241677 |
| Acer1 | 0.00144 | -1.27165604 |
| Rnf122 | 0.01985 | -0.61447301 |
| Csmd1 | 0.09604 | -2.64725419 |
| Zfp652 | 0.00144 | -1.75960741 |
| Pglyrp2 | 0.01809 | -0.95081427 |
| Olfr1155 | 0.00144 | -1.27165604 |
| Wipi2 | 0.0626 | -0.43275696 |
| Olfr98 | 0.00144 | -1.26560808 |
| Golph3 | 0.01038 | 0.75867923 |
| Snca | 0.00144 | -1.28116048 |
| Gpatch4 | 0.03536 | 0.66176668 |
| Anapc11 | 0.03211 | -0.64945362 |
| Plk4 | 0.00144 | 1.26475549 |
| Myo5a | 0.04565 | 0.66988845 |
| Krtap4-6 | 0.00144 | -1.32540776 |
| Pcbp2 | 0.02779 | -0.55831171 |
| Rgs8 | 0.00144 | -1.4750072 |
| Fes | 0.00244 | -0.9071619 |
| Ece1 | 0.00442 | -0.79529154 |
| Btbd6 | 0.43721 | -0.29212245 |
| Rras | 0.00286 | -0.82777536 |
| Pigb | 0.01196 | 0.66116042 |
| Lrp4 | 0.08653 | 0.37270737 |
| Gpr39 | 0.00244 | -1.16878422 |
| Ap4m1 | 0.13851 | -0.25529808 |
| Nap1l4 | 0.00624 | -0.79054127 |
| Mis18bp1 | 0.00621 | -0.90040948 |
| Lgals4 | 0.03189 | 1.50215586 |
| Eif2d | 0.00325 | -0.88285291 |
| Sox5 | 0.00144 | -1.29746368 |
| Ttf2 | 0.74408 | 0.07331838 |
| Rexo1 | 0.00144 | -1.12733144 |
| Jag1 | 0.69867 | 0.10787812 |
| Gpd1l | 0.40205 | 0.27808652 |
| Zbtb6 | 0.47023 | -0.30812908 |
| Gm11696 | 0.22996 | -0.27010742 |
| Gm38549 | 0.24076 | -0.70987843 |
| Apc | 0.33769 | 0.33109363 |
| Troap | 0.06981 | -0.93831698 |
| Amtn | 0.03197 | -0.81054649 |
| Zfpm2 | 0.06571 | 0.64843952 |
| Nodal | 0.04195 | -0.45763853 |
| Ghr | 0.01533 | 1.57613917 |
| C1qtnf4 | 0.00164 | -1.02729897 |
| Ciart | 0.71406 | 0.08247953 |
| Gabarapl1 | 0.08515 | -0.35374068 |
| Prosc | 0.59695 | 0.1362013 |
| Morc1 | 0.53452 | -0.42367996 |
| Dcaf17 | 0.06301 | 0.60844422 |
| Mut | 0.00344 | 0.90457917 |
| Aasdhppt | 0.03373 | 0.44334148 |
| Zfp395 | 0.02691 | -0.69622851 |
| Zfp811 | 0.09833 | -0.3814633 |
| Palm2 | 0.32128 | 0.91228984 |
| Olfr1316 | 0.00188 | -1.1975031 |
| Gtf3c3 | 0.01214 | 0.72794397 |
| Csnk1g1 | 0.06801 | 0.9371338 |
| Tfpi | 0.00621 | -0.89979991 |
| Mast3 | 0.13577 | -0.24446766 |
| Cytl1 | 0.00296 | -1.16721486 |
| Atp5f1 | 0.30756 | -0.22665804 |
| Exoc6 | 0.05353 | 0.43354345 |
| E230012J19Rik | 0.04123 | -2.52548457 |
| Cobll1 | 0.02571 | -0.66341834 |
| Rerg | 0.00144 | -1.45950387 |
| Frmd4a | 0.48797 | -0.490875 |
| Tkt | 0.42462 | -0.40746414 |
| Vps13a | 0.01157 | 0.78307455 |
| Myh10 | 0.00144 | -1.31495282 |
| Cnot3 | 0.00246 | -1.20482022 |
| Cdh18 | 0.00144 | -1.28116048 |
| Abcc4 | 0.20153 | 0.2759806 |
| Sec22a | 0.30892 | -0.58880149 |
| Tac1 | 0.00544 | -1.10518964 |
| 5930436O19Rik | 0.00727 | 1.6206711 |
| 4930412F12Rik | 0.73932 | -0.1669364 |
| Gpr62 | 0.15746 | -0.57032346 |
| Rcbtb2 | 0.17302 | 0.36358254 |
| Epg5 | 0.00767 | 0.80939099 |
| Ept1 | 0.66824 | -0.19956011 |
| Ppp1r7 | 0.00457 | 0.68013944 |
| Dnajb6 | 0.03709 | -0.48859606 |
| Lce1g | 0.00719 | -1.089501 |
| Wisp3 | 0.00144 | -1.28898079 |
| Lsp1 | 0.88482 | 0.04327366 |
| Bet1l | 0.00144 | -1.14704239 |
| Gm2716 | 0.01097 | -1.10038984 |
| BC106179 | 0.6984 | 0.078964 |
| Adh1 | 0.20097 | 0.4643759 |
| Zfp42 | 0.01423 | -0.96858621 |
| Notumos | 0.00511 | 0.80466234 |
| Olfr1314 | 0.02167 | -2.17294994 |
| Zranb3 | 0.12381 | -0.76003597 |
| Bend6 | 0.7364 | -0.34492181 |
| Cdk5rap2 | 0.00158 | -1.2446827 |
| Ghdc | 0.03369 | -1.52090852 |
| Syt4 | 0.00144 | -1.20555712 |
| Col27a1 | 0.35396 | -0.41178667 |
| Oxsr1 | 0.55837 | -0.13117631 |
| Cntn2 | 0.04336 | -1.10480902 |
| 1810013L24Rik | 0.00215 | 1.49784812 |
| Ggt7 | 0.88384 | -0.03035383 |
| D830026I12Rik | 0.21888 | 0.69771884 |
| Ewsr1 | 0.07672 | 0.45375215 |
| Far2 | 0.13089 | -0.76374377 |
| Tctn3 | 0.01809 | 1.06802316 |
| Foxred2 | 0.91527 | 0.04124128 |
| Esrrb | 0.06083 | -1.16850934 |
| Nfatc2 | 0.02361 | -1.02454652 |
| Zfp592 | 0.00368 | 0.70085316 |
| Fam229a | 0.00916 | -1.06669723 |
| Abcc5 | 0.26716 | 0.31902864 |
| Cdca3 | 0.02763 | -1.25965689 |
| Pex3 | 0.05494 | 1.31155142 |
| Ssbp3 | 0.00144 | -0.96456276 |
| Tgm1 | 0.01286 | -1.73411549 |
| A230057D06Rik | 0.00149 | -1.22582153 |
| Grin2a | 0.00144 | -1.31495282 |
| Elk3 | 0.02504 | -0.52990909 |
| Nav3 | 0.00853 | 1.02796263 |
| Mtf2 | 0.22987 | 0.26616635 |
| B230319C09Rik | 0.0615 | -0.77022964 |
| Ces2b | 0.00144 | -1.20766347 |
| E330037G11Rik | 0.12132 | 0.87070945 |
| Disp2 | 0.00144 | -1.27165604 |
| Trnt1 | 0.00144 | 2.12640793 |
| Ube2g1 | 0.35785 | 0.21084785 |
| Gm9895 | 0.44035 | 0.26915881 |
| Cdk14 | 0.00179 | -1.40595898 |
| Klrb1f | 0.00144 | -1.28898079 |
| Sf3b1 | 0.02657 | 1.46454548 |
| Itch | 0.2341 | -0.96210209 |
| Pxmp4 | 0.06697 | -0.49461357 |
| Lysmd4 | 0.05163 | 1.0270751 |
| Trim9 | 0.00144 | -1.32766452 |
| Atp9b | 0.24205 | -0.21448242 |
| Aqp4 | 0.66481 | -0.15114636 |
| Ipo11 | 0.06573 | 1.01560238 |
| Samd7 | 0.00969 | -0.99774049 |
| Cabp4 | 0.02871 | -1.10379914 |
| Sclt1 | 0.00144 | 2.83482787 |
| Accs | 0.04294 | -0.4631001 |
| 1700024G13Rik | 0.78307 | 0.14655612 |
| Sorcs3 | 0.01967 | -2.11811652 |
| Asph | 0.23848 | 0.2269475 |
| Nudt16 | 0.16176 | -0.2868571 |
| Ssu2 | 0.04072 | -0.499673 |
| Ssu2 | 0.34361 | -0.25212789 |
| Efr3a | 0.03599 | -0.94101771 |
| 4930451E10Rik | 0.00144 | -1.26934956 |
| Syn2 | 0.42542 | 0.16343663 |
| Clec1a | 0.00222 | 2.8157021 |
| Opalin | 0.112 | -0.83236325 |
| Aamp | 0.20788 | -0.4488478 |
| Tug1 | 0.00557 | 0.94688603 |
| Atad2 | 0.00144 | 1.94796671 |
| Gm5039 | 0.00144 | -1.24466058 |
| Capn7 | 0.00144 | 1.55797797 |
| Fem1b | 0.04767 | 0.38221233 |
| Hic2 | 0.00283 | -0.80965156 |
| Boc | 0.00145 | -1.20627532 |
| Ccdc141 | 0.62144 | 0.11494518 |
| Sgcd | 0.00144 | -1.27454685 |
| Ophn1 | 0.00144 | -1.27165604 |
| R3hdm4 | 0.1104 | -0.65908193 |
| Nbeal1 | 0.00144 | 1.3357206 |
| Slc25a37 | 0.02731 | -0.93945247 |
| Zic3 | 0.00144 | -1.29085005 |
| 2900041H08Rik | 0.00727 | -1.49287441 |
| Adal | 0.02306 | -0.52462456 |
| 4930548F15Rik | 0.03655 | -1.94524508 |
| Pdcl3 | 0.01092 | 0.54508044 |
| Nop16 | 0.65082 | -0.18084162 |
| Tnnt3 | 0.9298 | 0.05163912 |
| 6030419C18Rik | 0.01698 | -0.70235692 |
| Gm4890 | 0.06702 | -1.30763391 |
| Sgpl1 | 0.95473 | 0.01458293 |
| Abcc3 | 0.00676 | -1.23532597 |
| Pth2 | 0.00144 | -1.30202163 |
| Apaf1 | 0.46878 | -0.14476714 |
| Tbpl2 | 0.00144 | -1.45722203 |
| Shoc2 | 0.00144 | 1.66351791 |
| Olfr101 | 0.00144 | -1.29085005 |
| Clrn1 | 0.027 | -0.87572167 |
| Dnajb4 | 0.47453 | -0.13142629 |
| Ifnlr1 | 0.74697 | -0.08773569 |
| Tcf20 | 0.79779 | -0.06112686 |
| 4930447C04Rik | 0.00144 | -1.30713006 |
| Gpr37 | 0.04467 | -1.20888515 |
| Nmt1 | 0.01695 | -0.54644628 |
| Ajap1 | 0.10985 | -0.5364127 |
| Armc9 | 0.63772 | -0.16357724 |
| Eif2a | 0.04512 | 1.01316463 |
| Ncl | 0.61006 | -0.20685274 |
| Adgrl3 | 0.00144 | -1.26934956 |
| 4931428F04Rik | 0.05755 | -1.04151663 |
| Mospd3 | 0.05935 | -0.62120978 |
| Gpr182 | 0.01285 | -0.90977933 |
| A830082K12Rik | 0.00623 | -1.57036479 |
| Mab21l1 | 0.61894 | -0.30784372 |
| Rmdn1 | 0.68035 | -0.16994719 |
| A530079E22Rik | 0.00144 | -1.28116048 |
| Gm41810 | 0.86897 | -0.06421503 |
| Umad1 | 0.00639 | 1.52193815 |
| Dact2 | 0.00623 | -1.0185076 |
| Tmem216 | 0.58403 | 0.18996369 |
| 4930518F22Rik | 0.00144 | -1.56603655 |
| LOC105242417 | 0.03567 | -1.1434953 |
| Mical3 | 0.01954 | -0.95882478 |
| Nudt12 | 0.06356 | -0.49316367 |
| Ubqlnl | 0.80333 | 0.32310107 |
| Rundc3b | 0.01643 | 0.61672047 |
| Atp8b5 | 0.23785 | 0.25753022 |
| Olfr1487 | 0.00145 | -1.22376446 |
| Lcn9 | 0.00144 | -1.30713006 |
| Nono | 0.07708 | 0.37505561 |
| Crk | 0.39224 | -0.42190074 |
| Ermp1 | 0.00495 | 0.99424594 |
| Wnt2 | 0.03979 | -0.70788523 |
| Ikzf1 | 0.44625 | -0.27779051 |
| Slc35a5 | 0.00281 | -1.15316677 |
| Rps15a | 0.01247 | -0.60699376 |
| BC028777 | 0.00144 | -1.29085005 |
| Mrs2 | 0.45842 | -0.16740157 |
| Manea | 0.00544 | 1.54343111 |
| Fam221b | 0.15791 | -0.81877524 |
| Micall1 | 0.04867 | -2.66806499 |
| H3f3b | 0.00693 | -0.621175 |
| A930012O16Rik | 0.25099 | 0.32788914 |
| Cinp | 0.07345 | 0.78688354 |
| P4ha2 | 0.85425 | -0.108319 |
| Klf12 | 0.00144 | 1.56735319 |
| Fbrs | 0.23717 | -0.55007205 |
| Huwe1 | 0.0925 | 1.12459243 |
| Sel1l3 | 0.06277 | -2.21677792 |
| 4632427E13Rik | 0.02396 | 0.74189263 |
| Psmc6 | 0.00144 | 1.14813535 |
| Galntl6 | 0.00144 | -1.28898079 |
| 4930519F16Rik | 0.00145 | -1.22376446 |
| Ube2q2 | 0.00203 | -1.23098019 |
| 9930111J21Rik2 | 0.6888 | -0.26038847 |
| Cd59b | 0.00873 | -0.80742248 |
| Rusc2 | 0.05369 | -0.58158735 |
| Lgr6 | 0.24178 | -0.38454895 |
| Sowahd | 0.00144 | -1.29049509 |
| Scn10a | 0.85477 | -0.23229859 |
| Bard1 | 0.00221 | -1.10308723 |
| Sbno2 | 0.01476 | -0.57021136 |
| Ccr1 | 0.00216 | -1.10905491 |
| Atp10d | 0.00709 | 1.83106359 |
| 3110057O12Rik | 0.00144 | 2.3981772 |
| Uba1 | 0.00144 | -1.3417423 |
| Qk | 0.23144 | 0.2674645 |
| Cdc25b | 0.01691 | -1.18846071 |
| Adgrf2 | 0.00144 | -1.31495282 |
| Vmn2r83 | 0.12882 | -0.58533953 |
| Cntnap5a | 0.13817 | -0.69250312 |
| Tpra1 | 0.5225 | -0.24635835 |
| Hist3h2a | 0.00895 | -0.68814281 |
| Rnaseh2b | 0.00572 | 0.70570601 |
| A630052C17Rik | 0.927 | -0.05230223 |
| Adcyap1r1 | 0.02716 | -0.94135081 |
| Nsun3 | 0.30575 | -0.49549471 |
| Wnt16 | 0.00144 | -1.26301121 |
| Acox1 | 0.00144 | -1.30422036 |
| Gm15800 | 0.8349 | -0.10072353 |
| Rsbn1l | 0.22521 | 0.26370128 |
| Vmn2r29 | 0.73979 | -0.35319582 |
| Sucnr1 | 0.00583 | -1.04768057 |
| Hbs1l | 0.099 | -0.54228314 |
| Ube4b | 0.55837 | -0.11129226 |
| Rhobtb1 | 0.0085 | 0.78605595 |
| Rpusd1 | 0.02978 | -0.76409587 |
| 3000002C10Rik | 0.46188 | -0.34952617 |
| Pld3 | 0.05896 | -0.66017 |
| 0610037L13Rik | 0.31624 | -0.31928034 |
| Tph1 | 0.0266 | -1.81552903 |
| Defb39 | 0.00171 | -1.13622611 |
| Zbtb7a | 0.00827 | -0.57287841 |
| Dlec1 | 0.07947 | -0.87680132 |
| Elmo1 | 0.69908 | 0.13741427 |
| Zfp3 | 0.16376 | 0.34342504 |
| Clec4b1 | 0.0044 | -1.01967147 |
| 4833420G17Rik | 0.00321 | 0.88932957 |
| Cenpo | 0.65642 | -0.12027386 |
| Ncam1 | 0.61598 | -0.1318133 |
| Icam1 | 0.06439 | 0.53629277 |
| Zfp715 | 0.00188 | 0.87296451 |
| Pkdrej | 0.41629 | 0.50052475 |
| Ccng1 | 0.00144 | 2.21654036 |
| Leng8 | 0.00625 | 1.09293683 |
| Brcc3 | 0.09685 | 0.62542272 |
| Zfp697 | 0.00151 | -1.17792995 |
| Ss18l1 | 0.00583 | 1.5160371 |
| Htr1a | 0.00144 | -1.20555712 |
| Abhd16b | 0.16135 | -0.7405572 |
| A930038B10Rik | 0.00144 | -1.28898079 |
| Runx1t1 | 0.05122 | 0.99692935 |
| Prepl | 0.92056 | 0.01753953 |
| Atp2c1 | 0.01152 | -0.5783824 |
| Arntl2 | 0.28005 | -0.9102132 |
| Smad4 | 0.32025 | -0.1785108 |
| Slc47a1 | 0.66079 | 0.16379301 |
| Fam104a | 0.0059 | -0.78886801 |
| Cacna1g | 0.10028 | -0.55367545 |
| Rab2a | 0.03128 | -0.45763795 |
| Wwox | 0.66042 | 0.23675691 |
| Dnajc28 | 0.00951 | 0.85796283 |
| 4931440F15Rik | 0.89148 | -0.10931843 |
| Slc28a3 | 0.04206 | -1.12265448 |
| Usp47 | 0.01404 | 1.60157208 |
| Car9 | 0.11706 | 0.76813282 |
| Naalad2 | 0.01923 | 0.58823608 |
| Hexdc | 0.99759 | -0.00093499 |
| Evc | 0.14319 | -1.13062963 |
| Calm1 | 0.93514 | 0.03786461 |
| Abl2 | 0.03867 | -0.82799674 |
| Trak1 | 0.88269 | 0.10456801 |
| Eln | 0.44487 | -0.29079032 |
| Ehf | 0.00144 | -1.28116048 |
| Timd4 | 0.27535 | 0.76390531 |
| Wipi2 | 0.06826 | -0.63934714 |
| Abca5 | 0.00144 | -1.29049509 |
| Klhdc8b | 0.38962 | -0.18416555 |
| Serinc3 | 0.01139 | 0.72352545 |
| Fam84b | 0.73109 | 0.12500273 |
| Eef1g | 0.02939 | -0.6246749 |
| Pdxp | 0.01902 | -0.86334437 |
| Nol7 | 0.00164 | 0.87175406 |
| Cenpw | 0.01095 | -0.65089846 |
| Vcpkmt | 0.86197 | -0.02976703 |
| Aars2 | 0.04003 | -0.67566853 |
| Nfat5 | 0.00144 | 2.05925364 |
| Cntnap1 | 0.10172 | -0.34349702 |
| Maff | 0.74271 | -0.07556152 |
| Pitpnb | 0.8716 | -0.03193433 |
| Pim2 | 0.02088 | 1.17606695 |
| Eya4 | 0.00218 | -1.229352 |
| Snx32 | 0.21613 | 0.226437 |
| 1110002L01Rik | 0.00144 | -1.28116048 |
| Ppm1e | 0.01115 | 0.7730307 |
| Ppap2c | 0.19083 | -0.57254669 |
| 4921513I03Rik | 0.00144 | -1.31884045 |
| Golim4 | 0.05114 | -0.62244539 |
| Usp15 | 0.0037 | -0.95180509 |
| Atp13a5 | 0.79466 | -0.29447683 |
| Msr1 | 0.00884 | 1.03950285 |
| E2f7 | 0.40147 | 0.54810507 |
| Cylc2 | 0.01134 | -1.04486201 |
| C530042K13Rik | 0.00144 | 2.46086027 |
| Lin54 | 0.32557 | 0.73832573 |
| Tmem241 | 0.00144 | -1.27699683 |
| Calhm2 | 0.00969 | -0.99774049 |
| Pigh | 0.08323 | 0.77614449 |
| Tceb3 | 0.1073 | 0.77243219 |
| 1700003H04Rik | 0.00144 | -1.28898079 |
| Mrpl3 | 0.59238 | 0.13533802 |
| Lclat1 | 0.00144 | -1.15650272 |
| Ptrh1 | 0.02439 | -0.68819123 |
| Olfr107 | 0.09356 | -1.19068682 |
| Olfr739 | 0.00144 | -1.27992547 |
| Nxnl2 | 0.26403 | 0.32898128 |
| Sdccag3 | 0.20355 | -0.57511941 |
| Mmp24 | 0.02039 | -1.44780261 |
| Eef2 | 0.00159 | -0.99134251 |
| Tsg101 | 0.06957 | 0.90616107 |
| Pigx | 0.98728 | 0.00672353 |
| Pcdhga12 | 0.29181 | -0.54795206 |
| Ndor1 | 0.14179 | -0.4657383 |
| Il17rb | 0.02351 | -1.0906744 |
| Fbxl2 | 0.00144 | -1.31495282 |
| 4732416N19Rik | 0.08043 | 0.5885567 |
| Csrnp1 | 0.02998 | -0.55224669 |
| S1pr2 | 0.0148 | -0.53058925 |
| Mrpl19 | 0.45443 | 0.21508611 |
| Zfp319 | 0.00874 | -0.65538736 |
| Vps37a | 0.00144 | 1.78912369 |
| Gm21276 | 0.00144 | -1.28898079 |
| Mbp | 0.00552 | -0.79905786 |
| Ptprd | 0.00144 | -1.27454685 |
| Adamts6 | 0.17209 | -1.85960573 |
| Cdc37l1 | 0.02894 | 0.74041556 |
| Prps1l3 | 0.35233 | 0.37909363 |
| Jtb | 0.1295 | 0.43990685 |
| Ero1lb | 0.39016 | 0.20594656 |
| 4933435F18Rik | 0.02525 | -2.47616659 |
| Otub1 | 0.70249 | -0.17747717 |
| Cpq | 0.04051 | -0.73531924 |
| Aig1 | 0.00144 | -0.99553211 |
| Stx8 | 0.92964 | -0.03106882 |
| Greb1l | 0.00205 | 1.1925556 |
| Gm166 | 0.84838 | -0.12464217 |
| Scd4 | 0.04251 | -1.12575436 |
| Sufu | 0.02225 | -0.60419084 |
| Nlrc3 | 0.01051 | 1.44033888 |
| Olig1 | 0.0046 | -1.19134025 |
| Ccdc27 | 0.00596 | -1.03427027 |
| Nkrf | 0.01241 | 1.22131262 |
| Glycam1 | 0.00164 | -1.34390295 |
| Srpk2 | 0.71892 | -0.05641742 |
| Serpinb6d | 0.19221 | -0.47788439 |
| Hmox1 | 0.12885 | -0.77300354 |
| Zdhhc3 | 0.41736 | -0.52150235 |
| Sned1 | 0.12262 | 0.67810177 |
| Otud7b | 0.00144 | 1.9241965 |
| Syne2 | 0.11215 | 0.37037466 |
| C4bp | 0.00166 | -1.21062785 |
| Lemd2 | 0.0041 | -0.78235397 |
| 6030458C11Rik | 0.07888 | -2.51198972 |
| Zfp668 | 0.00144 | -1.2622024 |
| Astn2 | 0.00144 | -1.28898079 |
| Trim27 | 0.00395 | -1.3027367 |
| Lilra6 | 0.33326 | -0.24717926 |
| Zfp326 | 0.02183 | 0.92216847 |
| 6230424C14Rik | 0.01134 | 1.0930407 |
| Atp6v0c | 0.61862 | -0.25205736 |
| Mro | 0.07002 | -2.36801901 |
| 4930455J16Rik | 0.11427 | -1.05634202 |
| 1700031L13Rik | 0.00144 | -1.28898079 |
| Mtss1 | 0.01947 | 0.55093568 |
| Prkcz | 0.63742 | -0.11351705 |
| Src | 0.0046 | -0.90319702 |
| Zfp39 | 0.24282 | 0.45030786 |
| Gm38410 | 0.0149 | -0.84340856 |
| Rgs8 | 0.00144 | -1.29878664 |
| Thumpd3 | 0.15873 | -0.23315191 |
| Qrich2 | 0.00251 | -1.56681324 |
| Arhgef10l | 0.20525 | -0.38695708 |
| Fam174a | 0.2612 | -0.46078798 |
| Cald1 | 0.05931 | 0.67182087 |
| 4930596D02Rik | 0.15327 | -0.77732725 |
| Rragb | 0.07132 | 0.49981577 |
| Klrb1c | 0.50244 | 0.65696903 |
| Fam181a | 0.00145 | -1.25646236 |
| Cpeb2 | 0.00422 | 1.3724164 |
| Hs6st2 | 0.80643 | -0.08428224 |
| Mdfic | 0.16693 | 0.31229252 |
| 1700112J05Rik | 0.0405 | -1.76385148 |
| Tmem243 | 0.01505 | 1.94303601 |
| Atg14 | 0.95651 | -0.00919178 |
| Bfar | 0.01863 | 1.0774025 |
| Erc1 | 0.12222 | -0.33967439 |
| Gm23952 | 0.00144 | -1.29746368 |
| Baz2b | 0.09263 | 0.57430861 |
| Afg3l1 | 0.09129 | -0.51274646 |
| 1190007I07Rik | 0.29757 | -0.37102242 |
| 1700025F24Rik | 0.00464 | -1.39528885 |
| Ttll4 | 0.00449 | -1.21125457 |
| Rbm20 | 0.02183 | 1.00582843 |
| Chchd3 | 0.30668 | -0.17988484 |
| Klhl22 | 0.45132 | -0.21064255 |
| 1500012F01Rik | 0.02105 | -0.47122613 |
| Gtf2f2 | 0.06892 | 0.41862006 |
| Clpb | 0.0116 | 0.82863214 |
| Bola2 | 0.00152 | -0.96949361 |
| Lgals8 | 0.08689 | 0.3930064 |
| Ing1 | 0.40767 | -0.12917986 |
| Wasf2 | 0.00244 | -1.00080889 |
| Robo2 | 0.31788 | -0.34522788 |
| Gm9725 | 0.00144 | -1.29878664 |
| Gprc5a | 0.06049 | -0.41880179 |
| Ccdc84 | 0.05741 | -0.42091287 |
| Polr3f | 0.26244 | 0.58229347 |
| Fzd1 | 0.80404 | 0.04329695 |
| Acsl1 | 0.53688 | 0.24688298 |
| Olfr444 | 0.00144 | -1.26934956 |
| Ncald | 0.61591 | 0.15608957 |
| Inpp5e | 0.75396 | 0.09332742 |
| Cp | 0.00211 | 1.2536858 |
| Hrsp12 | 0.00144 | 1.56069977 |
| Pnma2 | 0.00144 | -1.20555712 |
| Rassf9 | 0.04342 | 0.89082131 |
| Plk4 | 0.00144 | -1.28116048 |
| Hectd2 | 0.73262 | -0.09231619 |
| Ccdc64 | 0.00144 | -1.20555712 |
| 5031414D18Rik | 0.00145 | -1.2388121 |
| Cckbr | 0.00144 | -1.28898079 |
| Rnf25 | 0.90026 | 0.03526254 |
| Fam204a | 0.01142 | 0.59419961 |
| Sfxn5 | 0.02269 | -1.63893082 |
| Wdr33 | 0.04788 | 0.66053477 |
| 2310067B10Rik | 0.01606 | -0.76653058 |
| Med23 | 0.03017 | -0.59398435 |
| Zeb2 | 0.25968 | 0.27295384 |
| Nelfa | 0.00583 | -0.6884008 |
| Cyp1a2 | 0.00145 | -1.2388121 |
| Pigc | 0.22034 | -0.22797915 |
| Tmbim6 | 0.2076 | -0.34913884 |
| Smug1 | 0.3733 | 0.27399396 |
| Ints12 | 0.00416 | -1.37305723 |
| Snx21 | 0.1104 | -0.42469001 |
| Gm25380 | 0.0071 | -0.73125495 |
| Vps35 | 0.02492 | 0.45701659 |
| Hsd17b13 | 0.07049 | -0.77588058 |
| Vps72 | 0.8673 | 0.076048 |
| Olfr91 | 0.07686 | -3.16632413 |
| Pip4k2c | 0.04975 | -0.47728621 |
| Olfr980 | 0.00144 | -1.27699683 |
| Aprt | 0.00262 | -0.98035932 |
| Asb4 | 0.4902 | 0.19426517 |
| Tmem259 | 0.50648 | -0.28988078 |
| Speer2 | 0.00144 | -1.25118919 |
| Isca2 | 0.66563 | -0.14880978 |
| Nupl1 | 0.0018 | 1.28032107 |
| Msc | 0.22288 | -0.68661939 |
| Lrba | 0.31085 | 0.29551731 |
| Wdfy2 | 0.0088 | 0.60930526 |
| Piezo1 | 0.40145 | -0.5593573 |
| Cyp21a1 | 0.0461 | -1.92003642 |
| Actr5 | 0.0015 | -1.01442557 |
| A830018L16Rik | 0.01039 | -1.82488412 |
| Kbtbd3 | 0.00229 | 1.00567275 |
| 4933402P03Rik | 0.07538 | -0.91306114 |
| Fam188a | 0.00364 | 1.5241636 |
| Ptgdr2 | 0.02772 | -0.98161948 |
| Arap2 | 0.90041 | 0.05258279 |
| Pax9 | 0.01588 | -0.99849484 |
| Astn1 | 0.15363 | -0.90793808 |
| Lyrm7 | 0.03928 | -0.9493323 |
| Arcn1 | 0.08273 | 0.76557992 |
| Lsm6 | 0.00992 | -0.61351343 |
| Rilpl1 | 0.00144 | -1.27165604 |
| 4931406B18Rik | 0.00144 | -1.28116048 |
| Ptbp2 | 0.29781 | -0.3374594 |
| Acsl5 | 0.09113 | 0.47825976 |
| Pibf1 | 0.00144 | 1.78086654 |
| Ostc | 0.9343 | 0.02040788 |
| Dgat2 | 0.30631 | -0.29727144 |
| Taf9b | 0.09694 | 0.86702599 |
| Sncaip | 0.05685 | 0.54332915 |
| Rhoj | 0.01213 | -0.57774519 |
| Klk1b7-ps | 0.00144 | -1.39528885 |
| Kcnc1 | 0.00144 | -1.29746368 |
| Zswim3 | 0.06932 | -0.48055249 |
| Klre1 | 0.00145 | -1.25646236 |
| Zkscan8 | 0.00177 | -1.52257906 |
| Brf1 | 0.98772 | -0.00282066 |
| Cst6 | 0.00544 | -0.77655936 |
| Ergic2 | 0.23934 | 0.4089553 |
| Hoxd13 | 0.05901 | -1.44443572 |
| Ms4a7 | 0.13046 | -0.38920468 |
| Brms1 | 0.02793 | -0.5008657 |
| Dpysl2 | 0.23329 | -0.25097686 |
| Pld4 | 0.42571 | -0.24256124 |
| Cntn4 | 0.00144 | -1.29049509 |
| E230016K23Rik | 0.00144 | -1.27699683 |
| Nbea | 0.00327 | -1.43580951 |
| Dnm3os | 0.15534 | -0.80154196 |
| Timm23 | 0.00367 | 1.92691783 |
| Fam13c | 0.00144 | 2.40869957 |
| 2900092D14Rik | 0.00144 | -1.28116048 |
| Slc25a35 | 0.1961 | 0.23698643 |
| Tle3 | 0.01353 | -0.54091463 |
| 2810403D21Rik | 0.32699 | -0.41358097 |
| Tmem161a | 0.03044 | -0.66178684 |
| Fbln2 | 0.01001 | -0.64046951 |
| 4933407L21Rik | 0.00173 | -1.28323428 |
| Ccnl1 | 0.06292 | -0.6066854 |
| Rab10 | 0.28992 | 0.20156056 |
| Kazn | 0.15933 | -0.52399626 |
| Cwf19l2 | 0.00309 | 2.27370452 |
| 1700025G04Rik | 0.01173 | 0.66175827 |
| Vars2 | 0.00331 | -0.83802871 |
| Txn2 | 0.13311 | -0.45967179 |
| Saal1 | 0.38603 | -0.19194746 |
| Gpr137c | 0.21394 | -1.02477299 |
| Pde4d | 0.62511 | -0.07971749 |
| Polr3a | 0.46079 | 0.17429047 |
| Zfp846 | 0.02958 | 1.11557687 |
| Polg2 | 0.66101 | -0.14537404 |
| Foxo6 | 0.08975 | -0.77560296 |
| Scaper | 0.05575 | -0.48566487 |
| Doc2a | 0.16184 | -0.68909615 |
| Smg7 | 0.00682 | -0.81488869 |
| 2010111I01Rik | 0.99847 | -0.00045646 |
| Rhno1 | 0.70576 | 0.14406068 |
| Gcn1l1 | 0.68003 | 0.24099609 |
| Mbtps1 | 0.67578 | 0.08869787 |
| Zfp7 | 0.75845 | 0.12359723 |
| Iqgap3 | 0.33981 | -0.5704546 |
| Ntn5 | 0.46253 | -0.46700648 |
| C1qtnf3 | 0.00234 | -1.43684968 |
| Zfp386 | 0.00144 | -1.25118919 |
| Snapin | 0.30471 | 0.26346803 |
| Fbln1 | 0.00144 | -1.38347855 |
| LOC102633345 | 0.00144 | -1.28116048 |
| Cacna1h | 0.00144 | -1.20555712 |
| Ppapdc1a | 0.00184 | -1.19286777 |
| Ubxn2b | 0.95439 | -0.0213518 |
| Zfp11 | 0.0787 | 0.33577144 |
| Zeb2 | 0.00144 | 1.50412574 |
| Ddx56 | 0.01466 | -0.75275817 |
| P2ry2 | 0.04713 | -0.61313899 |
| Phf3 | 0.00184 | 0.81283799 |
| Ddx19a | 0.1092 | 0.52365872 |
| 2310022A10Rik | 0.81178 | 0.15063474 |
| Ptp4a1 | 0.0024 | -0.86884822 |
| Oas2 | 0.90668 | -0.04102461 |
| Plekha5 | 0.31362 | 0.3585705 |
| A430090L17Rik | 0.35024 | -0.40054462 |
| Pdyn | 0.00144 | -1.41773667 |
| Gng4 | 0.00144 | -1.29085005 |
| Vmn1r188 | 0.00144 | -1.28116048 |
| Six1 | 0.51662 | -0.32458348 |
| Eef2k | 0.00559 | -1.30785486 |
| Adcy7 | 0.18567 | 0.21920619 |
| Hk3 | 0.02651 | -0.85003654 |
| Lipo1 | 0.00651 | 0.77875362 |
| Pla2g4f | 0.00144 | -1.29746368 |
| Bap1 | 0.0601 | -0.46340056 |
| Scgb2b26 | 0.05737 | -0.59737818 |
| Gns | 0.80963 | -0.07843286 |
| Pex3 | 0.09534 | -0.63184358 |
| Ppp6r2 | 0.06522 | -1.06134173 |
| Ciao1 | 0.18837 | 0.33851775 |
| Fndc3b | 0.01871 | 0.49746283 |
| Oxr1 | 0.01579 | 1.12432448 |
| Egfr | 0.3325 | -0.49952606 |
| Ispd | 0.00144 | 1.87256457 |
| Neu1 | 0.01112 | -0.7172753 |
| Ccpg1 | 0.00144 | 1.1277471 |
| Tmem67 | 0.00144 | -1.35619208 |
| Stxbp4 | 0.00144 | -1.30713006 |
| Cxcr4 | 0.02351 | -0.61387133 |
| Fkbp4 | 0.2934 | -0.37719431 |
| Las1l | 0.75271 | -0.10539079 |
| Armc2 | 0.24982 | 0.38809549 |
| Cnep1r1 | 0.5202 | 0.10452308 |
| Srpk2 | 0.00144 | -1.28898079 |
| Tusc5 | 0.00608 | -1.25549476 |
| Prkar2a | 0.00183 | -1.35990906 |
| Tmem199 | 0.30975 | 0.39441908 |
| Ube3a | 0.00249 | 1.32721945 |
| Cars2 | 0.00281 | -0.77348075 |
| Folr1 | 0.00144 | -1.32575997 |
| Pnlip | 0.00144 | -1.33632959 |
| Fpr-rs4 | 0.00244 | -1.16878422 |
| Frat1 | 0.10165 | -0.59640252 |
| Lrpap1 | 0.57015 | -0.14973669 |
| Tlx1 | 0.22671 | -0.99321985 |
| Tom1l1 | 0.00539 | 0.83915568 |
| Zbtb6 | 0.00144 | 1.58834624 |
| Plcb2 | 0.20728 | -0.51980914 |
| Ykt6 | 0.54298 | 0.22116008 |
| C2cd4a | 0.32382 | -1.76397149 |
| Gpatch11 | 0.02278 | 1.5128111 |
| Arvcf | 0.00809 | -0.95282466 |
| Nfkb1 | 0.05574 | 0.94025137 |
| Entpd4 | 0.0116 | -0.8767401 |
| Dgkd | 0.12814 | -0.25078048 |
| Kcna5 | 0.23939 | -0.2628896 |
| Rab22a | 0.27713 | -0.16922077 |
| Myh2 | 0.00632 | -1.24781287 |
| Fam73b | 0.24477 | -0.30259206 |
| Mrps6 | 0.33817 | -0.33032829 |
| Ust | 0.69815 | -0.19564672 |
| Rbm48 | 0.37261 | 0.34306311 |
| 9130221H12Rik | 0.53904 | -0.14347949 |
| Mrps11 | 0.38258 | -0.4598454 |
| Epsti1 | 0.14732 | -1.43592334 |
| 4930469G21Rik | 0.45012 | -0.5550168 |
| 1700034H15Rik | 0.02671 | -1.00277612 |
| Nub1 | 0.01141 | -0.55120823 |
| Ccdc18 | 0.06219 | -1.03573653 |
| Gosr2 | 0.28916 | -0.1745373 |
| Hcfc1r1 | 0.03754 | -0.60344232 |
| Suclg2 | 0.01958 | 1.18585909 |
| Nrg2 | 0.00473 | -1.83335787 |
| Aldh1a2 | 0.01347 | -0.59884015 |
| Wasf2 | 0.02074 | -0.57667349 |
| Kcnt1 | 0.02114 | -1.21932572 |
| Hcfc2 | 0.00968 | 0.83742967 |
| Adrbk1 | 0.5401 | -0.24131804 |
| Kansl1 | 0.39391 | 0.40397719 |
| Zfp781 | 0.00144 | 2.88051534 |
| Gm13791 | 0.05027 | 0.40230144 |
| Rbm46 | 0.00144 | -1.28116048 |
| Usp34 | 0.08144 | 0.34483817 |
| Nppa | 0.05481 | -1.04462143 |
| Spata33 | 0.551 | -0.12883404 |
| Rnf8 | 0.40379 | 0.16724763 |
| Gatad2a | 0.01354 | -0.52570805 |
| Arpc5 | 0.97426 | -0.00588998 |
| Zfp146 | 0.84615 | 0.05519921 |
| Mtcl1 | 0.02537 | -0.60434222 |
| Suds3 | 0.20464 | -0.45626184 |
| 4930452A19Rik | 0.02161 | -1.33227556 |
| 4930564B18Rik | 0.96788 | -0.01152642 |
| Mrps33 | 0.93707 | -0.02088392 |
| Rrs1 | 0.03573 | 0.61895901 |
| Klrb1 | 0.01162 | -1.95058301 |
| Pdlim3 | 0.11461 | -0.34348598 |
| Cox10 | 0.88066 | -0.10021841 |
| Oscp1 | 0.48323 | 0.12284827 |
| Uap1l1 | 0.10921 | -0.49414145 |
| Zfp532 | 0.90155 | -0.08311982 |
| Muc4 | 0.04259 | -0.99433119 |
| Pigb | 0.00232 | -1.15063496 |
| Dok3 | 0.43186 | -0.15929359 |
| Ccr5 | 0.0657 | -1.93643062 |
| Arhgap32 | 0.77853 | 0.0559186 |
| Igsf11 | 0.54791 | -0.25356988 |
| Slc41a1 | 0.09411 | 0.76852015 |
| Selp | 0.00144 | -1.20555712 |
| Sec63 | 0.04502 | 0.70994653 |
| Tank | 0.0797 | -0.54382939 |
| Nup214 | 0.72146 | 0.20220946 |
| St6galnac2 | 0.62984 | 0.30417326 |
| Lpxn | 0.36874 | -0.42337699 |
| Rnf169 | 0.17802 | 0.25602127 |
| Ednrb | 0.00144 | 1.85374469 |
| Vat1l | 0.00144 | -1.27730451 |
| Epdr1 | 0.74621 | 0.13075827 |
| Il18bp | 0.43832 | 0.15657623 |
| Il18bp | 0.90511 | -0.03065568 |
| Lrig2 | 0.03106 | -0.4318258 |
| C730002L08Rik | 0.03219 | -1.46773966 |
| Gucy2e | 0.00262 | -1.4650987 |
| Scfd2 | 0.05402 | 0.46226437 |
| Arhgef3 | 0.95635 | -0.01029257 |
| Galnt13 | 0.00244 | -1.41003405 |
| 1520401A03Rik | 0.00144 | -1.25281511 |
| Srd5a1 | 0.71121 | -0.19719746 |
| Dcbld2 | 0.87862 | 0.04054695 |
| Smurf2 | 0.97267 | 0.00791329 |
| Cep44 | 0.00293 | 1.25699657 |
| Calm4 | 0.00144 | -1.31884045 |
| Zfp944 | 0.06758 | 0.42988235 |
| Dap | 0.03158 | -0.42765182 |
| Dcst2 | 0.00144 | -1.31014844 |
| Adgrl3 | 0.61695 | 0.14351992 |
| Ppl | 0.92686 | 0.03606752 |
| Acss3 | 0.05731 | -0.82450882 |
| BC048671 | 0.5316 | -0.48598203 |
| Thap1 | 0.14792 | -0.29390925 |
| Sh2b1 | 0.32823 | -0.25295729 |
| Inhbc | 0.00905 | -1.10278386 |
| Rgl1 | 0.13494 | -0.33823269 |
| Acvr2a | 0.11598 | 0.48409652 |
| Zscan20 | 0.35871 | -0.45788491 |
| Mtfr1 | 0.04547 | -0.44947069 |
| Meis3 | 0.20892 | 0.33621667 |
| Sin3b | 0.00391 | -1.02103674 |
| Mylk | 0.12259 | 0.35304437 |
| Cdon | 0.13037 | -0.31809634 |
| Gria2 | 0.00145 | -1.2388121 |
| Elavl3 | 0.00144 | -1.28898079 |
| Babam1 | 0.00534 | -0.84665222 |
| Pappa2 | 0.06373 | -0.78049363 |
| Mesdc2 | 0.13698 | 0.53777711 |
| 1500026H17Rik | 0.35996 | -0.20409872 |
| Hus1 | 0.92094 | -0.01879161 |
| Rbpjl | 0.57155 | -0.2607331 |
| Oit3 | 0.06438 | -0.6770965 |
| Khdrbs1 | 0.0559 | 0.39518277 |
| Med14 | 0.00144 | 1.58609645 |
| Nt5e | 0.00211 | 1.10158323 |
| Bid | 0.0502 | -0.51926329 |
| D6Wsu163e | 0.59709 | -0.48123561 |
| Chic2 | 0.40011 | -0.19072464 |
| Spatc1 | 0.00144 | -1.20555712 |
| Rrp1b | 0.00787 | -0.59233541 |
| Psmd12 | 0.0103 | 0.57249619 |
| Sox5os3 | 0.00262 | -1.4650987 |
| Arl4c | 0.00144 | -1.29085005 |
| Trove2 | 0.02104 | 0.49975445 |
| Pde4c | 0.72236 | 0.08620018 |
| 6030498E09Rik | 0.00144 | -1.28898079 |
| Ddx6 | 0.08626 | -0.33409272 |
| Prol1 | 0.00144 | -1.34263533 |
| Hnrnpul1 | 0.16011 | 0.42106799 |
| Sbsn | 0.29758 | -0.17549241 |
| Cdc42ep1 | 0.00144 | -1.16932388 |
| N4bp2 | 0.0182 | 0.99901202 |
| Fam19a3 | 0.01281 | 0.80180103 |
| Cand1 | 0.33525 | -0.15213654 |
| Ptpn14 | 0.01609 | -0.66718624 |
| Atp1b3 | 0.01548 | -0.5769964 |
| Dtna | 0.00653 | 0.87875585 |
| Gxylt1 | 0.01033 | 0.80714746 |
| Fcrl6 | 0.00158 | -1.2446827 |
| Dhrs9 | 0.17483 | -1.15575983 |
| Agpat5 | 0.0275 | 0.53748038 |
| Ctcflos | 0.00144 | -1.27165604 |
| Laptm4a | 0.43061 | 0.27985339 |
| Lin9 | 0.54677 | -0.28827384 |
| Atp1a3 | 0.00158 | -1.2446827 |
| D130043K22Rik | 0.0017 | -1.19401501 |
| Rbm4b | 0.23608 | -1.33205264 |
| Hspa4 | 0.379 | 0.57453083 |
| Arv1 | 0.00993 | 0.60798056 |
| Hist2h2aa1 | 0.04716 | 1.368854 |
| Zfp626 | 0.00537 | 1.0441431 |
| Pcsk5 | 0.00151 | -1.17792995 |
| Troap | 0.04795 | -2.09114319 |
| Fam169a | 0.00144 | 2.73394653 |
| Rrp15 | 0.18941 | -0.56633258 |
| Nhlrc2 | 0.07622 | -0.44854471 |
| Myo1a | 0.38533 | -0.21212726 |
| Ash2l | 0.08065 | 0.62400628 |
| Minos1 | 0.36181 | -0.22967655 |
| Tmem144 | 0.00595 | -0.75600017 |
| Pate2 | 0.00969 | -0.99774049 |
| Ptms | 0.00144 | -2.51819619 |
| Klhl5 | 0.04425 | -1.02289157 |
| Dock6 | 0.92958 | -0.03373742 |
| Slc22a22 | 0.00144 | -1.31884045 |
| Scgb2b24 | 0.04632 | -0.77354063 |
| Flywch1 | 0.00144 | -1.4764456 |
| Map4k5 | 0.20968 | -0.32353873 |
| Sdk2 | 0.42139 | -0.367237 |
| Olfr1344 | 0.47775 | -0.48760626 |
| Dync2h1 | 0.01313 | -0.8723243 |
| Syncrip | 0.42101 | 0.22877713 |
| Psmb10 | 0.00144 | -1.43836628 |
| Kbtbd11 | 0.22093 | -0.41065878 |
| Arl14ep | 0.00422 | 1.01149018 |
| BB114814 | 0.33117 | -0.2317955 |
| Mroh2b | 0.00144 | -1.30626366 |
| Gm4758 | 0.00254 | -1.1839779 |
| Mphosph9 | 0.02149 | 1.58094381 |
| Actr1b | 0.92969 | -0.0348771 |
| Usp47 | 0.00169 | 1.05999289 |
| D030004A10Rik | 0.174 | -0.68481593 |
| Gramd1b | 0.0264 | -0.73225682 |
| Lig4 | 0.00144 | 2.42073926 |
| Stat3 | 0.36138 | -0.24559943 |
| Etl4 | 0.01862 | 0.66079396 |
| Dcc | 0.00144 | -1.53983991 |
| Fgf5 | 0.00144 | -1.27165604 |
| Dbt | 0.00346 | 1.2158493 |
| Masp2 | 0.00144 | -1.28116048 |
| Nwd1 | 0.00144 | -1.27165604 |
| Ccdc88a | 0.00644 | 1.52933219 |
| Wdpcp | 0.00642 | 0.75624935 |
| Gm20740 | 0.00144 | -1.27585605 |
| Sphkap | 0.00144 | -1.26911831 |
| Atp2a2 | 0.33825 | -0.15582641 |
| 1700029F12Rik | 0.01577 | -0.96101038 |
| Kcnab1 | 0.03799 | 1.33384965 |
| Ubxn1 | 0.02914 | -0.46834616 |
| Blzf1 | 0.09584 | -0.35192816 |
| Fstl5 | 0.00144 | -1.27699683 |
| Enah | 0.19437 | 0.23048252 |
| Usmg2 | 0.00144 | -1.29878664 |
| Slc25a40 | 0.01029 | -1.60281818 |
| Asah2 | 0.65417 | 0.16576391 |
| Akap9 | 0.00144 | 1.38351514 |
| Cux1 | 0.7067 | 0.13298207 |
| Lcp1 | 0.63128 | 0.11357349 |
| Tmem132b | 0.00791 | -1.91460197 |
| Cyp2w1 | 0.31211 | -1.24390974 |
| Stambpl1 | 0.04308 | 0.80099003 |
| Gm38606 | 0.00378 | -1.15821745 |
| Pard3bos1 | 0.13872 | -0.6073339 |
| Antxrl | 0.00145 | -1.22376446 |
| Msi1 | 0.00144 | -1.30017714 |
| Bcl10 | 0.06521 | -0.61291249 |
| Adgrf1 | 0.28989 | -0.62904245 |
| Sla2 | 0.38152 | -0.42573211 |
| Tcf7l2 | 0.56871 | -0.08802989 |
| Gpm6a | 0.00367 | 1.32712811 |
| Emc3 | 0.09259 | 0.57522058 |
| Ntan1 | 0.21427 | 0.45298217 |
| Rnf122 | 0.4011 | -0.25981187 |
| Zfyve26 | 0.28131 | 0.25387079 |
| Chia1 | 0.47742 | -0.3931079 |
| Map3k11 | 0.00505 | -0.68909491 |
| Ccdc37 | 0.00144 | -1.20281695 |
| Slc16a14 | 0.00144 | -1.26911831 |
| Mon1b | 0.01332 | 0.5235553 |
| Taok2 | 0.01738 | -0.65284901 |
| Nsd1 | 0.72264 | -0.07516143 |
| Muc2 | 0.00233 | -1.24652876 |
| AU018091 | 0.77313 | -0.2272363 |
| Arhgap20 | 0.06133 | -0.42860601 |
| Pax6 | 0.00144 | -1.25118919 |
| Prl8a1 | 0.00144 | -1.22925777 |
| 1700010H22Rik | 0.00243 | -1.12283767 |
| Cecr5 | 0.82407 | -0.05760444 |
| Sae1 | 0.01417 | -0.56945572 |
| Cpne3 | 0.00851 | -0.74231514 |
| Specc1 | 0.00942 | -0.71566747 |
| C2cd3 | 0.0102 | 0.6756521 |
| Rps9 | 0.97613 | 0.00688647 |
| Zfp558 | 0.05279 | -3.79051722 |
| Egfr | 0.11395 | -0.43520855 |
| Ube2b | 0.51562 | 0.28109343 |
| Kank1 | 0.00144 | -1.55334825 |
| Fabp4 | 0.20093 | -0.37479388 |
| A530064D06Rik | 0.02197 | -2.20345013 |
| Pik3c2g | 0.19861 | -0.66434908 |
| Prkg1 | 0.00144 | -1.30713006 |
| Nf1 | 0.02513 | -0.76199852 |
| Etl4 | 0.00214 | 1.46110955 |
| Zfp251 | 0.02915 | -0.45255615 |
| Aunip | 0.16575 | -0.52164158 |
| Fbxw5 | 0.50244 | -0.30719519 |
| Psmg4 | 0.01174 | -0.64269493 |
| B3galt1 | 0.22488 | 0.45056304 |
| Fam162a | 0.10283 | 0.95420242 |
| Ssbp4 | 0.01533 | -0.53537722 |
| Map1lc3b | 0.90408 | 0.05327438 |
| Mrps15 | 0.86134 | -0.09353659 |
| Brix1 | 0.00744 | 0.94237164 |
| Lgals2 | 0.03505 | -1.32000064 |
| Col11a2 | 0.0843 | -1.74924486 |
| Pianp | 0.01992 | -1.77880922 |
| 4930520O04Rik | 0.02023 | 1.26400267 |
| Ank1 | 0.68332 | -0.08716868 |
| Sult4a1 | 0.55285 | 0.19462258 |
| Pkhd1l1 | 0.00727 | -1.07804057 |
| Srd5a1 | 0.11894 | -0.85616086 |
| Tpgs1 | 0.00144 | -1.14226723 |
| Sync | 0.00144 | -1.00685738 |
| Far1 | 0.00659 | 1.22475451 |
| Ick | 0.00173 | 1.54928347 |
| Sptssa | 0.57954 | -0.13616159 |
| Ncor1 | 0.07806 | -0.34113215 |
| Med13 | 0.01113 | 1.40829702 |
| Cntnap3 | 0.00144 | -1.27165604 |
| Arhgap6 | 0.24588 | 0.46105691 |
| Ccnyl1 | 0.00144 | 1.02091804 |
| BC034090 | 0.13224 | -0.42431587 |
| Vwa1 | 0.00144 | -1.13498991 |
| Fam160b1 | 0.01485 | -0.59202551 |
| Tmem135 | 0.42798 | -0.1727751 |
| 4930402H24Rik | 0.75192 | 0.10450413 |
| 4932438H23Rik | 0.00949 | -1.04161237 |
| Stard4 | 0.6039 | -0.30866647 |
| Gm38654 | 0.05499 | -2.08607875 |
| Tox | 0.00179 | -2.0433256 |
| Slc25a32 | 0.06061 | 0.39696982 |
| Gm26782 | 0.65288 | -0.13071299 |
| Pawr | 0.01281 | -1.21075607 |
| Akap8 | 0.00995 | 1.14760624 |
| Rnf141 | 0.31674 | -0.21289089 |
| Slc25a39 | 0.01972 | -0.88850705 |
| Serhl | 0.05687 | -0.44695939 |
| Pibf1 | 0.01183 | 0.61111143 |
| Abhd11 | 0.11512 | -0.59404373 |
| Gm22 | 0.00907 | -0.62532106 |
| Dab2 | 0.00145 | -1.22376446 |
| Cxcr2 | 0.9974 | 0.00232362 |
| Fbln7 | 0.22947 | -0.35140164 |
| Znhit6 | 0.65868 | 0.13236447 |
| BC026585 | 0.492 | -0.18170252 |
| Cercam | 0.00267 | -1.05113396 |
| Prdm6 | 0.15325 | -1.03090722 |
| Aim1l | 0.00144 | -1.30713006 |
| Mapk8 | 0.00144 | -1.28898079 |
| Il15ra | 0.00144 | 1.18340294 |
| Gpr155 | 0.04707 | 0.93985267 |
| Rab3gap1 | 0.1349 | 0.29865063 |
| Rras2 | 0.76495 | -0.07793891 |
| Necab1 | 0.17396 | 0.96982261 |
| Runx2 | 0.00145 | -1.26219879 |
| Mat2a | 0.05928 | -0.45917786 |
| Smek1 | 0.00324 | 1.39794388 |
| Tmem260 | 0.12357 | 0.81464051 |
| Clec16a | 0.31966 | -0.24609627 |
| Zfp263 | 0.29777 | 0.26035231 |
| Il16 | 0.08928 | 0.85081287 |
| Cav2 | 0.39717 | 0.27471689 |
| Zdhhc1 | 0.002 | -0.87021188 |
| Rnmt | 0.00144 | -1.29049509 |
| Tor1aip2 | 0.01661 | 0.5824115 |
| Secisbp2 | 0.95489 | 0.01216142 |
| Poc5 | 0.08807 | 0.30200054 |
| Ccdc59 | 0.12336 | -0.33773097 |
| Fbxo6 | 0.3223 | -0.49874612 |
| 4930455C13Rik | 0.27329 | -0.4929643 |
| Tbxa2r | 0.00144 | -1.65719381 |
| Ccdc36 | 0.00277 | -1.47835842 |
| Gm5084 | 0.00144 | -1.33059316 |
| Slmo1 | 0.00305 | -1.78391247 |
| Tmprss2 | 0.02119 | -1.44978843 |
| Ugt3a2 | 0.02317 | -1.91192703 |
| Adamts10 | 0.86995 | -0.05705512 |
| Olfr968 | 0.00144 | -1.30017714 |
| Olfr996 | 0.00144 | -1.30713006 |
| Slc12a5 | 0.19021 | 0.23108869 |
| Ssbp2 | 0.00415 | 1.82740463 |
| Ntrk3 | 0.15166 | -1.0900471 |
| Dlx6 | 0.00421 | -2.3370175 |
| Fam50b | 0.00372 | -1.50348569 |
| Fancd2 | 0.00144 | -1.27165604 |
| Calb2 | 0.03306 | -0.91062157 |
| Ralgapa2 | 0.40895 | 0.1622944 |
| LOC102641241 | 0.02211 | 0.83545089 |
| Txlng | 0.00786 | 1.06225156 |
| G3bp2 | 0.0103 | 0.69770421 |
| Vapb | 0.10295 | -0.29149966 |
| Iars2 | 0.00144 | 1.65431598 |
| Oip5 | 0.31988 | 0.4634232 |
| Trmt2b | 0.11885 | 0.28326752 |
| Kmt2c | 0.14388 | -0.42428783 |
| Zc3h15 | 0.04672 | -1.30229687 |
| Nf2 | 0.81048 | -0.06899265 |
| Sntg1 | 0.00144 | -1.23984951 |
| Wt1os | 0.00144 | -1.27165604 |
| Ywhag | 0.97784 | -0.01128791 |
| Trappc12 | 0.27051 | -0.22988494 |
| Vrk1 | 0.0949 | 0.57965199 |
| B3gnt5 | 0.00144 | -1.35320495 |
| Zfp862-ps | 0.00736 | -0.91857389 |
| Apbb2 | 0.68391 | -0.10368839 |
| Snhg20 | 0.20358 | -0.27540248 |
| Klhdc10 | 0.01672 | -0.5787612 |
| Ube2d2b | 0.02115 | -1.8306177 |
| Krr1 | 0.01634 | 0.79056391 |
| Rgs19 | 0.15212 | 0.23343963 |
| Gm9758 | 0.02743 | -0.97379672 |
| Fam133b | 0.76716 | -0.13795377 |
| Hipk2 | 0.02299 | -0.5009284 |
| BC051076 | 0.00144 | -1.24613586 |
| Dnm1l | 0.04498 | 1.81507171 |
| Gprin3 | 0.00968 | -0.99308048 |
| Gm9725 | 0.25872 | -0.34214506 |
| Calu | 0.00532 | -0.66464075 |
| Gzmd | 0.00144 | -1.28898079 |
| Tfap2e | 0.00144 | -1.30713006 |
| Ier2 | 0.14171 | -0.39248518 |
| Tnfrsf26 | 0.47519 | -0.39303731 |
| Dnajc11 | 0.2356 | -0.38022703 |
| Olfr1138 | 0.06844 | -1.01933913 |
| 1300017J02Rik | 0.01132 | -2.57596203 |
| Rwdd2a | 0.37549 | -1.31839123 |
| Ilf3 | 0.42086 | -0.13897745 |
| Rabl2 | 0.99202 | -0.00696893 |
| Ccdc66 | 0.77758 | -0.36462589 |
| Itgb3 | 0.89287 | -0.02347133 |
| Atp5b | 0.87354 | 0.06469077 |
| Ttc17 | 0.04135 | -0.47776447 |
| 9030617O03Rik | 0.15408 | 0.41684312 |
| Marf1 | 0.0951 | 0.59252774 |
| Snrk | 0.01863 | 0.54558967 |
| Kdm5a | 0.77841 | -0.1315835 |
| Rsrc1 | 0.68015 | 0.10730936 |
| Prkg1 | 0.0478 | 0.37678297 |
| Cadm3 | 0.00973 | -1.61096334 |
| Tceanc | 0.05225 | 0.60012737 |
| Cracr2b | 0.00332 | -0.96016121 |
| 1700102J08Rik | 0.00267 | -1.20283908 |
| Ndufa4 | 0.00466 | -0.79765713 |
| Fzd7 | 0.24123 | 0.4862323 |
| 4930578I06Rik | 0.24282 | -0.73580755 |
| Agtrap | 0.06392 | -0.5128841 |
| Tjap1 | 0.0271 | -0.71966643 |
| Adat1 | 0.61016 | 0.10286367 |
| Rbsn | 0.08557 | 0.2969705 |
| Ffar1 | 0.00144 | -1.24811894 |
| Magea6 | 0.00144 | -1.26934956 |
| BC005537 | 0.12108 | -0.32916763 |
| Celf1 | 0.01183 | -0.80641115 |
| Ccdc69 | 0.29562 | -0.68183822 |
| Nav2 | 0.27304 | 0.48022577 |
| Olfr849 | 0.00144 | -1.28116048 |
| Raph1 | 0.00687 | 0.65000819 |
| Carf | 0.80026 | 0.05513651 |
| Ndc1 | 0.01848 | -0.48914933 |
| Smim1 | 0.00144 | -1.33620293 |
| Wdr7 | 0.04551 | -1.22380712 |
| H1f0 | 0.00736 | 1.04306802 |
| Ifi44 | 0.00144 | 1.99824911 |
| Lyrm5 | 0.00773 | 0.87513906 |
| Cdk14 | 0.07126 | -2.29878664 |
| Orc5 | 0.29841 | -0.54486442 |
| Nsmce2 | 0.01371 | 0.96524956 |
| Rictor | 0.39576 | 0.49047332 |
| Iars2 | 0.93853 | 0.02288839 |
| Tubgcp4 | 0.34278 | 0.25298382 |
| Rasgrf1 | 0.00144 | -1.28116048 |
| Ank2 | 0.22988 | 0.74952431 |
| Apobec1 | 0.01691 | -0.81563858 |
| Tmem100 | 0.19675 | 0.48049764 |
| Trmt12 | 0.73414 | 0.09681079 |
| Urm1 | 0.00391 | -0.92341746 |
| Slamf1 | 0.92944 | -0.05614513 |
| Sned1 | 0.00144 | 2.34550102 |
| 1700112J16Rik | 0.11131 | -0.72247272 |
| 4933414I15Rik | 0.00847 | -1.43436468 |
| Nfya | 0.00251 | 0.92751953 |
| Fam32a | 0.76052 | -0.0602372 |
| Erich2 | 0.0269 | -1.16589444 |
| Ost4 | 0.03218 | -0.88538178 |
| 5730455P16Rik | 0.81328 | -0.06506135 |
| 5730455P16Rik | 0.59191 | -0.14956717 |
| 2210019I11Rik | 0.08655 | -1.19047888 |
| 1600002D24Rik | 0.00144 | -1.28898079 |
| Trim52 | 0.63876 | -0.44969803 |
| Itgae | 0.03494 | -0.81883805 |
| Cldn8 | 0.00144 | -1.33632959 |
| Adgrg2 | 0.00144 | 1.68790853 |
| Arsj | 0.10204 | -0.52404164 |
| Olfr736 | 0.00144 | -1.29051721 |
| Frem2 | 0.06714 | 0.55389033 |
| Cgnl1 | 0.36062 | -0.17947017 |
| Hsd3b4 | 0.0124 | -1.1412272 |
| Lrrk1 | 0.08504 | 0.68309009 |
| Ubald1 | 0.04853 | -0.57729403 |
| Madd | 0.32764 | 0.31184472 |
| Shank2 | 0.00603 | -1.50434376 |
| Cntn5 | 0.07845 | -1.21684494 |
| Trim80 | 0.00144 | -1.22925777 |
| Mrpl38 | 0.84777 | -0.11293171 |
| Gm10030 | 0.03229 | 0.88685506 |
| Tmem175 | 0.5901 | -0.22488947 |
| Chd1 | 0.82084 | 0.04120349 |
| Opa1 | 0.00669 | 0.61252726 |
| Usp8 | 0.20569 | 0.5880008 |
| Aven | 0.00144 | -1.2500235 |
| LOC102634481 | 0.00144 | -1.28898079 |
| P2ry1 | 0.00144 | 1.54508038 |
| Spag1 | 0.01603 | 1.03078659 |
| Lurap1 | 0.02612 | -0.86396832 |
| Caprin1 | 0.00417 | 0.87047185 |
| Mrpl15 | 0.66973 | 0.18203865 |
| Lce1f | 0.00144 | -1.29049509 |
| Sirt2 | 0.70222 | 0.16351558 |
| Zfp560 | 0.74743 | -0.06561422 |
| Tomm70a | 0.00331 | 1.29158223 |
| Tab2 | 0.10105 | 0.36775659 |
| Csnka2ip | 0.00144 | -1.54014117 |
| 1700029I15Rik | 0.40541 | -0.16092116 |
| Map2 | 0.00953 | -1.21413759 |
| Pam | 0.82524 | 0.06544956 |
| Pelp1 | 0.01821 | -0.55308434 |
| Npy4r | 0.00144 | -1.43238607 |
| Ube2d1 | 0.65268 | -0.09557983 |
| Nr1h3 | 0.00553 | -0.75238733 |
| Hacd1 | 0.00144 | -1.29049509 |
| Mettl3 | 0.3375 | 0.24702315 |
| Glis3 | 0.00144 | -1.26560808 |
| 2810408B13Rik | 0.23214 | -2.11470053 |
| Ssr4 | 0.01998 | -0.74805172 |
| Eml1 | 0.29743 | -0.59003949 |
| Atf7ip | 0.00253 | -1.26718232 |
| Tssc1 | 0.88042 | -0.05908405 |
| Lrrfip1 | 0.00144 | 1.73248849 |
| Fcgr2b | 0.11845 | 0.41296963 |
| Deaf1 | 0.00144 | -1.29085005 |
| Rptor | 0.44153 | -0.19017563 |
| Syt14 | 0.00149 | -1.22582153 |
| Itga4 | 0.00144 | -1.29051721 |
| Caln1 | 0.24543 | -1.23914871 |
| Slc39a11 | 0.01946 | -0.60924339 |
| Gemin2 | 0.05444 | 0.43464397 |
| Klf5 | 0.13145 | -1.60309729 |
| 4931406C07Rik | 0.00651 | 0.78518379 |
| Slc22a15 | 0.10787 | -0.61472507 |
| Terc | 0.0091 | -1.07116381 |
| Pcsk5 | 0.95781 | 0.01561727 |
| A430057M04Rik | 0.10249 | -2.87978024 |
| Cep164 | 0.00926 | -1.35004116 |
| Qrich1 | 0.79064 | -0.07782329 |
| Klk1b26 | 0.52995 | 0.47411893 |
| Pibf1 | 0.66941 | -0.24482049 |
| Pbx1 | 0.00145 | -1.06228196 |
| Tmem260 | 0.0018 | 1.29462605 |
| Dhtkd1 | 0.16818 | -0.26116138 |
| Utp14a | 0.01664 | -0.85433614 |
| Cacna1h | 0.02823 | -1.42068811 |
| Rbm27 | 0.36656 | 0.30937255 |
| Hist1h2al | 0.00144 | -1.36658832 |
| Masp1 | 0.98178 | 0.01040114 |
| 2700029M09Rik | 0.04534 | 1.05895137 |
| Ephb4 | 0.00144 | -1.04845445 |
| Tmem56 | 0.00674 | 1.06335613 |
| Bhlhe23 | 0.00144 | -1.27730451 |
| Lmf2 | 0.00656 | -0.79773772 |
| Rb1cc1 | 0.00144 | 2.01293202 |
| Nsmf | 0.39764 | -0.19331359 |
| Gm26787 | 0.22288 | -1.07697149 |
| Exosc1 | 0.93248 | 0.01346405 |
| Mrps23 | 0.49904 | 0.40722062 |
| 1700020N18Rik | 0.0377 | -1.00148197 |
| Vps37b | 0.0274 | -0.74809574 |
| Axin2 | 0.00144 | -1.28116048 |
| Zbtb20 | 0.0034 | 3.05133938 |
| Cd244 | 0.7309 | 0.37829004 |
| Crebl2 | 0.00144 | -1.24863231 |
| Marveld1 | 0.00989 | -0.66978248 |
| Rgs18 | 0.0023 | 1.19628642 |
| Zfp850 | 0.13538 | -3.0765319 |
| Ccdc55 | 0.73857 | 0.16521865 |
| Ppp1cc | 0.05934 | 0.59584187 |
| Timp2 | 0.00144 | -1.05525328 |
| Lmo7 | 0.32795 | 0.70807485 |
| Pibf1 | 0.01002 | 1.47476407 |
| Cspg4 | 0.31462 | -0.61543702 |
| Ap5m1 | 0.01861 | -2.06772311 |
| Setd3 | 0.12551 | 0.51204102 |
| AV039307 | 0.34588 | -0.42693195 |
| Lbh | 0.35226 | 0.38368629 |
| Gtf2h3 | 0.42707 | -0.21542724 |
| 1810022K09Rik | 0.03084 | 0.50906623 |
| 2610002J02Rik | 0.11379 | -0.40029359 |
| Saxo2 | 0.05588 | -0.42773102 |
| Serp2 | 0.00814 | -0.62307676 |
| Adam30 | 0.0111 | -1.02598223 |
| Rdh11 | 0.30703 | -0.18749393 |
| Kirrel2 | 0.57313 | 0.13003038 |
| Ccnd2 | 0.21718 | -0.33563383 |
| Tada3 | 0.01084 | -0.6285247 |
| Fgf1 | 0.0063 | -1.21076099 |
| R3hdm1 | 0.03646 | 1.6737008 |
| Ssh2 | 0.0087 | -0.59317649 |
| Klk7 | 0.69513 | -0.13314144 |
| Kir3dl1 | 0.00191 | -1.43580951 |
| Bloc1s5 | 0.00793 | -0.57540115 |
| Olfr811 | 0.02627 | -0.9926579 |
| Olfr165 | 0.00144 | -1.27699683 |
| Abt1 | 0.39174 | -0.2950812 |
| Pcdhb2 | 0.00145 | -1.2388121 |
| Rpl5 | 0.13618 | 0.30475595 |
| Cdc14a | 0.94936 | -0.02621348 |
| Asb13 | 0.21915 | -0.70247653 |
| Tsga10 | 0.01162 | 1.3073684 |
| Fam120b | 0.00376 | 0.95921173 |
| Nemf | 0.00637 | 0.75340822 |
| Wdr72 | 0.00221 | 1.2819872 |
| Trap1a | 0.05628 | -0.91440819 |
| Cenpt | 0.95362 | -0.01103163 |
| Smpd3 | 0.11532 | -0.30313881 |
| Ak7 | 0.01185 | -1.65795253 |
| Zfp330 | 0.77009 | 0.05032175 |
| Mapk10 | 0.33445 | 0.26442503 |
| Dimt1 | 0.60963 | -0.08080024 |
| Pdgfrl | 0.00552 | -1.33679839 |
| Frmd5 | 0.00158 | -1.2446827 |
| Khdrbs2 | 0.00144 | -1.28116048 |
| Zdhhc24 | 0.23649 | -0.53810873 |
| Ube4a | 0.29826 | 0.48679665 |
| Rad54l | 0.03611 | 1.13443454 |
| Pik3r4 | 0.26427 | 0.45932599 |
| Rcsd1 | 0.28053 | 0.47784664 |
| Per2 | 0.53017 | -0.13764142 |
| Hpgds | 0.08974 | -0.74079747 |
| Hpcal1 | 0.13068 | -0.27081721 |
| Asap2 | 0.00464 | -0.72449519 |
| Mogs | 0.007 | -0.77320231 |
| Apol8 | 0.00144 | -1.28116048 |
| Prpf4 | 0.20805 | -0.2115807 |
| Rasgrf1 | 0.00331 | -1.20149398 |
| Prmt7 | 0.69 | 0.2315617 |
| Sde2 | 0.00221 | 0.96905667 |
| Faim2 | 0.12337 | -0.69131088 |
| Mri1 | 0.78716 | -0.16305549 |
| Hook1 | 0.00261 | 0.81294264 |
| Gvin1 | 0.00144 | 2.65458177 |
| Abca16 | 0.00144 | -1.31884045 |
| Rhot2 | 0.00304 | -0.81443579 |
| Syf2 | 0.00167 | -1.04103718 |
| Cpeb3 | 0.04019 | -0.57933526 |
| Sgol1 | 0.10208 | -2.73592288 |
| Bcl2 | 0.00144 | 1.62632167 |
| Atp8a1 | 0.32519 | -0.18995538 |
| Gm597 | 0.00784 | -1.03698725 |
| Npas2 | 0.11739 | -1.19389826 |
| Gm10101 | 0.20831 | 0.35070529 |
| Oxnad1 | 0.34763 | 0.18432206 |
| Mpp1 | 0.11916 | 0.84205772 |
| Kcnq1ot1 | 0.00454 | -1.12501865 |
| Ahsp | 0.12073 | -1.64759781 |
| Frmd6 | 0.00144 | -1.61679946 |
| Iqce | 0.07838 | -0.9930555 |
| Yipf6 | 0.14465 | -0.82853137 |
| Orc3 | 0.00516 | 0.73210658 |
| Anxa7 | 0.98928 | 0.00759759 |
| Tnik | 0.30228 | -0.44706697 |
| Rbms3 | 0.03521 | 1.1864841 |
| Srms | 0.00957 | -0.80103485 |
| Pias2 | 0.06031 | 0.91326338 |
| Magi3 | 0.00144 | 2.00221725 |
| Pih1d3 | 0.00144 | -1.28116048 |
| Angptl2 | 0.9985 | -0.00077686 |
| 4933427E13Rik | 0.00144 | -1.26560808 |
| Cyp4f16 | 0.42666 | 0.28708534 |
| Barx2 | 0.13368 | -1.01479804 |
| 1110037F02Rik | 0.1634 | 0.24506134 |
| Hsd17b14 | 0.00144 | -1.29049509 |
| Ino80 | 0.53101 | -0.17501062 |
| Chrna1 | 0.00144 | -1.28116048 |
| Eif5 | 0.31101 | -0.18140807 |
| Prm1 | 0.73776 | 0.24528275 |
| Tmem45a2 | 0.70467 | -0.4050043 |
| Spata31d1d | 0.09725 | -1.03672339 |
| 1700041G16Rik | 0.15801 | -0.74293956 |
| Cd300lb | 0.00948 | -1.39355808 |
| Dnajc2 | 0.06393 | 1.27533351 |
| Ablim1 | 0.00144 | -1.3914662 |
| Ddit3 | 0.46117 | -0.13077535 |
| Mixl1 | 0.00144 | -1.26560808 |
| 1110006O24Rik | 0.00144 | -1.43569228 |
| Dnajc16 | 0.8567 | 0.06353526 |
| Olfr805 | 0.00144 | -1.25118919 |
| Olfr237-ps1 | 0.00315 | -1.07029893 |
| C87436 | 0.00414 | 0.82855349 |
| Thrb | 0.98934 | 0.00225445 |
| Lin28b | 0.00144 | -1.28116048 |
| Marveld2 | 0.48971 | -0.49388618 |
| Slc25a43 | 0.19905 | -0.60851984 |
| S1pr1 | 0.99899 | 0.00038577 |
| Zfyve27 | 0.07109 | 0.36287723 |
| Tbk1 | 0.00144 | 2.01004432 |
| Rgmb | 0.00144 | 1.65255111 |
| Ttc9c | 0.00145 | 1.04093947 |
| Etnppl | 0.00144 | -1.28116048 |
| Gcnt3 | 0.00144 | -1.47491297 |
| 6330411D24Rik | 0.00144 | -1.28898079 |
| Vps53 | 0.00678 | -0.78455727 |
| Fkbp3 | 0.00323 | 1.00549308 |
| Hemt1 | 0.00144 | -1.36658832 |
| Man2a2 | 0.01949 | -0.80302848 |
| Cd2bp2 | 0.0073 | -0.6527236 |
| Capn8 | 0.00144 | -1.27165604 |
| Rhbdl3 | 0.04501 | -0.50672683 |
| Bfsp2 | 0.6823 | -0.18817785 |
| Unc13c | 0.00213 | -1.02335948 |
| Ppip5k1 | 0.97235 | -0.00630767 |
| Lrrc52 | 0.82086 | 0.16079332 |
| Drd2 | 0.26751 | -1.14416303 |
| Nsl1 | 0.70861 | 0.11113458 |
| Wdr77 | 0.49427 | 0.19181899 |
| Erich3 | 0.00157 | -1.19247859 |
| Enox1 | 0.98591 | -0.00666856 |
| Rab11fip5 | 0.0086 | -0.84871243 |
| Pcdhb4 | 0.41459 | 0.2336823 |
| Usp11 | 0.00144 | -1.25118919 |
| Fam151a | 0.0044 | -1.75188727 |
| Dmtf1 | 0.00144 | 2.32810199 |
| Paip2b | 0.00771 | -1.27161396 |
| Eps15l1 | 0.8004 | 0.04941881 |
| Zfp287 | 0.04834 | -0.85084701 |
| Mrpl57 | 0.04929 | -0.73958545 |
| Ttc27 | 0.11289 | 0.48970343 |
| Hhex | 0.23683 | -0.27339562 |
| Rabggta | 0.3474 | -0.34884839 |
| Slc48a1 | 0.45119 | -0.34558625 |
| Phospho2 | 0.00236 | -1.42385836 |
| Cpxm2 | 0.59879 | -0.2895178 |
| Mfsd3 | 0.00405 | -1.04937622 |
| Fam107b | 0.74479 | -0.21323041 |
| Fndc1 | 0.00144 | -1.63728248 |
| Ano8 | 0.01533 | -0.73249784 |
| Slc16a10 | 0.00211 | -0.94234458 |
| Rrp12 | 0.00788 | -0.86559117 |
| Reps1 | 0.05022 | 0.52573007 |
| Emilin2 | 0.16661 | -0.40219141 |
| Olfr934 | 0.00144 | -1.28116048 |
| Helb | 0.13294 | 0.3200498 |
| D15Ertd621e | 0.00159 | 1.3297495 |
| Adgb | 0.00144 | -1.33340976 |
| Gm5800 | 0.10563 | -0.78454565 |
| 3110043O21Rik | 0.0687 | 0.78455782 |
| Arhgap12 | 0.00516 | 1.72391328 |
| Ccdc158 | 0.00144 | -1.29085005 |
| Itga4 | 0.31088 | -0.646833 |
| A430093F15Rik | 0.52288 | -0.22153851 |
| Atg16l1 | 0.3684 | -0.43916073 |
| Pde1a | 0.127 | 0.42790127 |
| Atrnl1 | 0.6971 | -0.07916657 |
| Atl1 | 0.00298 | 1.28374481 |
| Zeb2 | 0.30989 | 0.61607685 |
| Wdr73 | 0.00558 | -0.6540239 |
| Csf2rb | 0.17231 | -0.68510423 |
| Arhgap15 | 0.11603 | 0.41926592 |
| 4930584F24Rik | 0.02768 | -1.81078403 |
| 4930523O13Rik | 0.00144 | -1.29746368 |
| Nebl | 0.0126 | -0.53475259 |
| Olfr519 | 0.00144 | -1.31495282 |
| 1700040F17Rik | 0.00157 | -1.19247859 |
| Gja5 | 0.00537 | -0.83443733 |
| Rhobtb2 | 0.00144 | -1.30017714 |
| Trim65 | 0.33849 | 0.15658968 |
| C130073F10Rik | 0.00144 | -1.30017714 |
| Col1a1 | 0.00175 | -0.96298856 |
| Vamp4 | 0.00167 | 1.54969669 |
| Epb4.1l1 | 0.15319 | -0.28382736 |
| Olfr149 | 0.05462 | -0.87662679 |
| Wbscr17 | 0.63329 | -0.27025384 |
| Atg2b | 0.80525 | 0.18086013 |
| Ddx58 | 0.01853 | 0.54976917 |
| Ceacam9 | 0.09441 | -2.49768 |
| Tbx5 | 0.93901 | -0.02375734 |
| Tceal3 | 0.00144 | -1.30713006 |
| Agpat3 | 0.01958 | -0.91749791 |
| Hapln2 | 0.99026 | -0.0160609 |
| Rfx1 | 0.18815 | -0.24005271 |
| Cdhr5 | 0.68029 | -0.23002154 |
| Dtnb | 0.03935 | -0.54327311 |
| 4930447C04Rik | 0.02506 | -1.70639057 |
| Psapl1 | 0.39322 | -0.19301854 |
| Tmem147 | 0.08816 | -0.58980999 |
| Snrpd3 | 0.40434 | -0.14674632 |
| Sh2d6 | 0.01107 | -0.96322961 |
| Rptn | 0.00144 | -1.28898079 |
| Gfap | 0.00144 | -1.28116048 |
| Zfp442 | 0.00144 | 2.42603003 |
| Zfp846 | 0.01989 | 0.68856684 |
| Alx4 | 0.67543 | -0.16166957 |
| D17Wsu92e | 0.00519 | -0.8770961 |
| Hsh2d | 0.00208 | -1.15498993 |
| Zak | 0.77727 | 0.07055676 |
| Aard | 0.00144 | -1.28898079 |
| Pitx1 | 0.00144 | -1.25118919 |
| Snhg5 | 0.10249 | -0.92586919 |
| Snhg5 | 0.13288 | 0.62450994 |
| Adipor1 | 0.00144 | -1.01860437 |
| Hddc3 | 0.34187 | -0.31718943 |
| Dph3 | 0.02006 | -0.5762566 |
| Myo9a | 0.09437 | 0.8587643 |
| Ppp1r36 | 0.04517 | -1.18509027 |
| Trio | 0.00144 | 1.03374183 |
| Chd1l | 0.03988 | -0.41969413 |
| Leo1 | 0.0049 | 0.75844445 |
| Ppm1d | 0.91251 | -0.07222626 |
| Lyst | 0.00391 | 1.04537953 |
| Tcf4 | 0.2033 | 0.3823683 |
| Serpinb8 | 0.0415 | -0.8304433 |
| BC029214 | 0.04716 | -0.63676033 |
| Taf7 | 0.53135 | -0.16874912 |
| Gm19466 | 0.00144 | -1.26428512 |
| Rbm28 | 0.00619 | -0.64667849 |
| Hspa9 | 0.01941 | -0.69198371 |
| Ankrd22 | 0.00144 | -1.30713006 |
| 1700084E18Rik | 0.00489 | -0.72281697 |
| Vcam1 | 0.41929 | -0.19778073 |
| Kif18b | 0.05555 | -1.30836506 |
| Timm8b | 0.77129 | -0.10642898 |
| Heg1 | 0.00392 | -0.79976655 |
| Rimbp2 | 0.36231 | -0.59761845 |
| Siglec1 | 0.04985 | -2.90330707 |
| Fads6 | 0.00341 | -1.09295312 |
| Kcnc3 | 0.85106 | 0.32378287 |
| Ltk | 0.00144 | -1.61127571 |
| Dock8 | 0.00342 | -1.27019565 |
| Uba6 | 0.00516 | 0.77195847 |
| D430022A14Rik | 0.59053 | 0.20703117 |
| Gm11938 | 0.00144 | -1.50305172 |
| Gm20337 | 0.0179 | -1.6327068 |
| Epha2 | 0.00144 | -1.11709235 |
| Itga1 | 0.84126 | -0.06181256 |
| Mmd | 0.19778 | -0.32529083 |
| Adam23 | 0.00455 | -1.3124439 |
| Tmem194b | 0.52756 | -0.29281267 |
| Hmbox1 | 0.07744 | -0.49244578 |
| Pdlim4 | 0.14704 | -0.74592034 |
| Tmem255a | 0.77005 | -0.17559381 |
| Ndrg3 | 0.21065 | 0.25431367 |
| Helz | 0.52354 | 0.16950396 |
| Magi3 | 0.06784 | 0.42751807 |
| Galns | 0.16115 | -0.40813244 |
| A230107N01Rik | 0.73198 | -0.19200979 |
| Nudt17 | 0.56899 | -0.31187011 |
| Tmem50b | 0.00243 | -1.1228689 |
| Usp50 | 0.00144 | -1.29085005 |
| 1700019J19Rik | 0.0096 | -1.63414365 |
| Lemd1 | 0.00144 | -1.28116048 |
| Man2c1 | 0.13664 | -0.33289918 |
| Sec1 | 0.05709 | -0.86363514 |
| Nrd1 | 0.03565 | 0.48902729 |
| Creb1 | 0.00494 | 0.98450002 |
| Cep290 | 0.03106 | 1.53308414 |
| Klhl23 | 0.15353 | 0.29323422 |
| Necab1 | 0.22581 | -0.81717313 |
| Trmt44 | 0.48165 | 0.34285638 |
| Akap1 | 0.93053 | 0.02879045 |
| Iqsec3 | 0.88081 | -0.03563836 |
| Trim63 | 0.5534 | 0.31998191 |
| Wdtc1 | 0.20097 | -0.47212337 |
| Ctdspl2 | 0.23647 | -0.76638697 |
| Dlc1 | 0.25308 | 0.73802115 |
| Rpf1 | 0.00144 | 1.27486202 |
| Spata3 | 0.00144 | -1.30713006 |
| Clcn1 | 0.05459 | -0.75001912 |
| Abca5 | 0.35797 | -0.24017875 |
| Adipor2 | 0.30877 | -0.28665035 |
| Col4a6 | 0.081 | -0.44069704 |
| Leng9 | 0.00211 | -1.01890264 |
| Pla2g15 | 0.00633 | -0.97000825 |
| Tia1 | 0.00144 | 1.12443425 |
| Ptpn1 | 0.0965 | -0.35442559 |
| Creb5 | 0.78726 | -0.086849 |
| 4930503L19Rik | 0.12532 | 0.55674111 |
| Vsnl1 | 0.00144 | -1.30253725 |
| Rgmb | 0.00974 | -0.97653689 |
| Reln | 0.00144 | -1.30017714 |
| Pcdh12 | 0.00292 | -0.81508682 |
| Myom1 | 0.01511 | 1.52289005 |
| Arhgap10 | 0.00144 | -1.37386711 |
| Gpr83 | 0.00145 | -1.22193674 |
| 5033421B08Rik | 0.00147 | -1.15512485 |
| Slc18a2 | 0.01194 | 2.10180434 |
| Pard3 | 0.70816 | -0.07175324 |
| Arih1 | 0.00514 | 0.82701422 |
| Slc9b1 | 0.00144 | -1.30017714 |
| Atp6v0a1 | 0.37826 | 0.44539105 |
| 1110051M20Rik | 0.62829 | -0.15164718 |
| Arhgdib | 0.00916 | -0.70403113 |
| Lrriq1 | 0.03356 | -1.96273903 |
| Brip1os | 0.01225 | -0.98016633 |
| Sugt1 | 0.10092 | 0.3370664 |
| 1700031A10Rik | 0.82457 | -0.10646587 |
| Rprd2 | 0.66039 | 0.14522115 |
| Slc25a24 | 0.01381 | 0.72674794 |
| Htatip2 | 0.51735 | 0.2516858 |
| 4930470H14Rik | 0.01326 | -1.72535225 |
| Klra15 | 0.87426 | -0.04789993 |
| Kcnk5 | 0.0207 | -0.97025956 |
| Slc35a3 | 0.51211 | 0.22210022 |
| Dennd1c | 0.00364 | 0.85720175 |
| Olfr318 | 0.15149 | -0.78412307 |
| Prkcb | 0.71286 | 0.10648967 |
| Dzip3 | 0.07647 | 0.65201431 |
| Arfgef1 | 0.75247 | -0.07265048 |
| Ugt8a | 0.2889 | -0.89577828 |
| Brca2 | 0.0132 | 1.03333193 |
| Zswim5 | 0.13946 | -1.49023035 |
| Sla | 0.01182 | 0.89108395 |
| Larp4b | 0.00388 | -0.89208666 |
| Cald1 | 0.00864 | 0.95479355 |
| Dna2 | 0.00144 | 1.5755185 |
| 9530052C20Rik | 0.00144 | -1.22925777 |
| Ptgr2 | 0.00144 | -1.30017714 |
| Cops7b | 0.15898 | -0.39528617 |
| Kif13a | 0.23273 | 0.25453881 |
| Hnrnpll | 0.14847 | 0.39021727 |
| Shisa5 | 0.00144 | -1.23173183 |
| Ccny | 0.00456 | 0.9142419 |
| Setd8 | 0.14537 | -0.36434509 |
| Cdc7 | 0.04135 | -0.80406695 |
| Mkrn2os | 0.0391 | -0.46183724 |
| Zfp219 | 0.52008 | -0.18697487 |
| Hes6 | 0.79996 | 0.17675828 |
| 4930512B01Rik | 0.00145 | -1.18741414 |
| Sp110 | 0.05564 | 0.49197446 |
| Polr1d | 0.63754 | -0.09847044 |
| Iqub | 0.04888 | -1.50994032 |
| Vit | 0.00144 | -1.26560808 |
| Tesk1 | 0.00515 | -0.79742818 |
| Mlec | 0.55341 | 0.18426829 |
| Bcl2l1 | 0.14365 | -0.29720456 |
| Ctsh | 0.0779 | -0.37810709 |
| Tbl3 | 0.04422 | -0.67405177 |
| Chtop | 0.00846 | 0.64552897 |
| E030003E18Rik | 0.33316 | 0.51184305 |
| 9130227L01Rik | 0.00144 | -1.28898079 |
| Adamts2 | 0.7832 | -0.096285 |
| Dhx33 | 0.04114 | -1.99591278 |
| Cdc40 | 0.00171 | 1.41542194 |
| Fastkd2 | 0.09005 | 0.52940774 |
| Tfg | 0.00261 | -1.11284335 |
| Ralgapa1 | 0.00144 | 2.08842017 |
| Gna15 | 0.26257 | -0.21736498 |
| Traf6 | 0.02004 | 0.97135096 |
| 9930014A18Rik | 0.56993 | -0.39236901 |
| Sh3gl2 | 0.00144 | -1.63862646 |
| Nudt15 | 0.85712 | 0.05008251 |
| A430046D13Rik | 0.32019 | -0.29947471 |
| Ift172 | 0.2954 | -0.25067431 |
| Spint1 | 0.00144 | -1.23532597 |
| Eva1c | 0.24205 | 0.54818812 |
| Nup160 | 0.00399 | 1.35068779 |
| Zwint | 0.02904 | 0.454235 |
| Gabpb2 | 0.01903 | 0.81059115 |
| Fto | 0.40353 | -0.21486369 |
| Zfp318 | 0.00213 | 0.98034579 |
| Fmo1 | 0.03064 | 0.77385142 |
| Abca6 | 0.01187 | 0.79664729 |
| Grn | 0.54677 | 1.61800395 |
| Zfp951 | 0.0016 | 1.70180591 |
| 4921528I07Rik | 0.00144 | -1.22465584 |
| 1810026B05Rik | 0.01837 | 0.54876572 |
| Dkk1 | 0.00256 | -1.65502389 |
| Gm7104 | 0.09003 | 0.43201649 |
| Rmi2 | 0.00144 | -1.28116048 |
| E2f3 | 0.72977 | 0.10355279 |
| Psap | 0.10371 | -0.62176297 |
| Poglut1 | 0.00144 | -1.25162917 |
| Cwc27 | 0.00507 | 1.59231716 |
| Col11a1 | 0.06633 | -0.90970992 |
| Ikbkg | 0.49203 | -0.22130071 |
| Chm | 0.41842 | -0.46269761 |
| Lpar1 | 0.00298 | 1.14452184 |
| Nrep | 0.00144 | -1.28116048 |
| Cadps2 | 0.70075 | -0.14088356 |
| Gabpb1 | 0.31663 | 0.2768932 |
| Slc25a37 | 0.80953 | 0.14452324 |
| Plxnc1 | 0.05896 | 0.45580338 |
| Unc93b1 | 0.00496 | -0.82573464 |
| Mrps24 | 0.0294 | -0.82074238 |
| Mmp9 | 0.00144 | -1.24865146 |
| Usp12 | 0.18459 | -0.7363994 |
| Plet1 | 0.00144 | -1.28116048 |
| Glyat | 0.23426 | -0.6665482 |
| Plcb1 | 0.18917 | 0.8095849 |
| Rabgap1l | 0.04396 | 2.54621164 |
| Arhgap27 | 0.88077 | -0.06728388 |
| Mcmdc2 | 0.00144 | -1.27165604 |
| Olfr441 | 0.08378 | -0.37966476 |
| Rhod | 0.70075 | -0.15531677 |
| Gigyf2 | 0.08831 | -0.68941007 |
| Cyb5r4 | 0.00216 | -1.08693479 |
| Phf21a | 0.00144 | -1.29085005 |
| Prkab1 | 0.97847 | 0.01284397 |
| Nlrp12 | 0.11011 | -1.18875059 |
| Ubxn10 | 0.88804 | 0.11256126 |
| Stt3a | 0.30602 | -0.22980399 |
| Ntn3 | 0.0163 | -0.78655803 |
| Fsd2 | 0.284 | 0.2282162 |
| Jrk | 0.11586 | -1.01826568 |
| Psmd13 | 0.88514 | -0.04923664 |
| Lcor | 0.00607 | 0.66997201 |
| Dstyk | 0.00144 | -1.29049509 |
| Trpc4ap | 0.46347 | 0.23962365 |
| Cdh13 | 0.00234 | 1.12140669 |
| Epha7 | 0.00767 | 0.67915369 |
| Rnf123 | 0.31818 | -0.35241116 |
| Cdyl | 0.17678 | 0.2362395 |
| Zfp865 | 0.03374 | -0.50694402 |
| Zfp60 | 0.03047 | -0.66892597 |
| 1700052I22Rik | 0.00144 | -2.18423158 |
| Adprh | 0.02148 | -0.7704383 |
| Ccnt2 | 0.00667 | 1.3738877 |
| Rnf138 | 0.00144 | 1.23928445 |
| Coa7 | 0.65567 | -0.18979433 |
| Ankib1 | 0.00144 | 1.69408642 |
| Pmpca | 0.10983 | 0.55838066 |
| B3galnt1 | 0.29149 | 0.35216844 |
| Cdk8 | 0.10776 | -0.55308095 |
| Prickle1 | 0.42606 | -0.40314222 |
| Col5a3 | 0.01328 | -1.1300332 |
| Ffar4 | 0.00144 | -1.27165604 |
| Kcnj2 | 0.71153 | -0.06935231 |
| Slc4a1ap | 0.09425 | 0.49103041 |
| Dcun1d3 | 0.68614 | -0.11474609 |
| Erlin2 | 0.07814 | 0.34596621 |
| Arhgdig | 0.23296 | -0.64390236 |
| Foxj2 | 0.00144 | -1.02109831 |
| Zfp957 | 0.13931 | -0.34186418 |
| Mbtd1 | 0.11102 | 0.44465573 |
| Aldh7a1 | 0.04117 | -0.50229343 |
| Ube2k | 0.00818 | 1.10595295 |
| Bend7 | 0.02099 | -0.55700749 |
| Mgat5 | 0.36268 | -0.70704126 |
| Ampd3 | 0.01985 | 1.53815054 |
| Dpep2 | 0.00145 | -1.36498085 |
| 4921511C20Rik | 0.07429 | -0.93601623 |
| S1pr3 | 0.07852 | -0.37949815 |
| Scai | 0.35756 | 0.18877209 |
| Fam173b | 0.47274 | 0.42717614 |
| Mir99ahg | 0.00144 | 4.21993536 |
| Epc2 | 0.5399 | 0.1280637 |
| 2810429I04Rik | 0.00144 | -1.25118919 |
| Hectd2 | 0.00144 | 2.74716367 |
| Olfr1427 | 0.00144 | -1.38646478 |
| Abr | 0.78712 | -0.06189303 |
| Naa35 | 0.54292 | -0.11411637 |
| Dnajc19 | 0.33512 | -0.28967546 |
| Med12l | 0.01197 | 1.32567987 |
| Ccdc173 | 0.55452 | -0.21027185 |
| Slc6a14 | 0.00144 | -1.29864962 |
| Snrnp70 | 0.15587 | 0.60588037 |
| Ccdc127 | 0.2871 | 0.76901344 |
| Stamos | 0.08097 | -0.85903142 |
| Ptprn2 | 0.02907 | -3.72619418 |
| Gm7072 | 0.00582 | 1.40861244 |
| Fam171a2 | 0.00144 | -1.23998599 |
| BC002059 | 0.00144 | 2.49412844 |
| Pcsk1 | 0.00307 | -1.0397239 |
| Srsf5 | 0.01843 | -0.62031454 |
| Acsm2 | 0.00203 | -1.17111094 |
| 8430419L09Rik | 0.47222 | -0.3301269 |
| Lars | 0.67578 | 0.06613466 |
| Atrn | 0.00188 | 1.00258352 |
| Taar2 | 0.17234 | -0.98415721 |
| Zfp551 | 0.91628 | -0.01687755 |
| LOC102636989 | 0.00144 | -1.30017714 |
| Nfil3 | 0.02244 | -0.97070987 |
| Tspyl5 | 0.02766 | 0.55455687 |
| Pias3 | 0.17226 | -0.29600353 |
| Smkr-ps | 0.01124 | -0.84142486 |
| Ipo11 | 0.5805 | -0.10508527 |
| Kcnj1 | 0.00145 | -1.18741414 |
| Gstz1 | 0.18584 | 0.55701843 |
| Scmh1 | 0.00144 | -1.29085005 |
| Scfd1 | 0.63468 | 0.83217176 |
| Cdk17 | 0.62316 | 0.16989843 |
| 4930513N10Rik | 0.00144 | -1.29085005 |
| Zmym3 | 0.6052 | -0.13259493 |
| 4930412C18Rik | 0.07695 | -0.61449381 |
| Tex101 | 0.41836 | -0.62201028 |
| Synj2 | 0.08221 | 0.47184428 |
| Tmx1 | 0.09928 | -0.39802936 |
| 9130008F23Rik | 0.43909 | 0.20566715 |
| Zfp512 | 0.0321 | 0.96571929 |
| 0610039H22Rik | 0.00251 | 1.5030661 |
| Wrn | 0.40541 | -0.60832471 |
| 4933409G03Rik | 0.0017 | -1.19401501 |
| Lyrm4 | 0.72968 | -0.14127636 |
| Cdkn2b | 0.32541 | -0.30894837 |
| Aldh1l2 | 0.00144 | 3.03169224 |
| Olfr385 | 0.00144 | -1.28116048 |
| Ccdc94 | 0.10459 | -0.34066252 |
| G6pc2 | 0.00601 | -1.09465341 |
| Spi1 | 0.00737 | -1.10659321 |
| A630001G21Rik | 0.00156 | 0.91627372 |
| Mipol1 | 0.0017 | 1.82168755 |
| Aanat | 0.2296 | -0.44419178 |
| Psmd1 | 0.04392 | 0.46881977 |
| Camkv | 0.00158 | -1.2446827 |
| Prrg1 | 0.00144 | 2.10901503 |
| A730090H04Rik | 0.073 | -0.8842013 |
| Nlgn1 | 0.00144 | -1.30935626 |
| Vps52 | 0.53707 | -0.26556676 |
| Supt20 | 0.63996 | 0.21706947 |
| Acsbg1 | 0.07588 | -0.69245296 |
| Mgat4a | 0.00273 | 1.05115841 |
| 2310011J03Rik | 0.2306 | -0.51466377 |
| Siae | 0.90533 | 0.04451857 |
| Papolg | 0.00144 | -1.5298028 |
| Vangl2 | 0.11787 | -0.37382799 |
| Tmprss11f | 0.00144 | -1.26934956 |
| Rasd1 | 0.74758 | -0.07797144 |
| Leprotl1 | 0.49597 | 0.12558395 |
| 2700081O15Rik | 0.78927 | 0.0504959 |
| Psd3 | 0.10503 | -0.38934013 |
| Tmem239 | 0.45846 | 0.22408156 |
| Ttll10 | 0.12927 | -3.19440652 |
| 1700001O22Rik | 0.83304 | -0.05280979 |
| Pms2 | 0.01893 | 0.78398995 |
| Exosc8 | 0.29235 | 0.31116143 |
| Pcsk1n | 0.06098 | -0.49985569 |
| Spats1 | 0.14038 | -0.70230507 |
| Fth1 | 0.03433 | -0.44020094 |
| Mafb | 0.612 | 0.11305797 |
| Kcnmb3 | 0.00902 | -1.08033603 |
| Efnb3 | 0.15073 | -0.77488822 |
| Zbtb10 | 0.0062 | 0.70262851 |
| Peo1 | 0.76102 | -0.10540151 |
| Tomm40 | 0.87471 | 0.09038835 |
| Nln | 0.18162 | 0.31301052 |
| Rrnad1 | 0.02317 | 0.64134411 |
| Zwint | 0.02756 | 0.65278498 |
| Heca | 0.00144 | 1.44706344 |
| 9130401M01Rik | 0.05634 | -0.42719113 |
| Fnip2 | 0.93076 | 0.0153894 |
| Gm12185 | 0.00929 | -1.0433256 |
| Kansl1l | 0.00174 | 1.12969463 |
| Nr4a2 | 0.48866 | -0.25964608 |
| Ppp2r1b | 0.19832 | -1.60864892 |
| Fbxl16 | 0.32668 | 0.27445315 |
| E330034G19Rik | 0.0028 | -1.70976101 |
| Dgkq | 0.41184 | 0.16993285 |
| Pam | 0.17233 | 0.32444812 |
| Itpr2 | 0.00149 | 1.63787719 |
| Prr18 | 0.27193 | -0.47551105 |
| Usp46 | 0.03219 | 0.46271737 |
| Gatad2b | 0.19084 | -0.22329187 |
| Zdhhc2 | 0.00144 | -1.20052149 |
| Il1bos | 0.0019 | -1.39477479 |
| Gucy1a3 | 0.009 | 1.44295497 |
| Cyp2d22 | 0.01987 | 0.72357011 |
| 2810403D21Rik | 0.06582 | 0.55223123 |
| Srpk1 | 0.83381 | 0.0495671 |
| 2310034G01Rik | 0.30912 | -0.51420798 |
| Kif1a | 0.43834 | -0.27077794 |
| Wdr13 | 0.20223 | -0.40748618 |
| Taf5l | 0.42618 | 0.26666441 |
| Fgfr1op2 | 0.00144 | -0.95027095 |
| Fam171a1 | 0.05323 | -0.68780326 |
| Igll1 | 0.01523 | -1.0894429 |
| Lnx1 | 0.02113 | 0.55165818 |
| Serpinb1c | 0.14833 | -2.53666377 |
| Trav9n-2 | 0.00144 | -1.29085005 |
| Chmp3 | 0.60977 | -0.08352788 |
| Zfp866 | 0.18424 | 0.70090397 |
| Ift140 | 0.42079 | -0.59302321 |
| Fmr1 | 0.0081 | 1.58161963 |
| Ncan | 0.0017 | -1.19401501 |
| Zdhhc6 | 0.00144 | -1.43031347 |
| Adam29 | 0.00144 | -1.29085005 |
| D19Bwg1357e | 0.05646 | 0.70937775 |
| Ttc7 | 0.31949 | -0.5070155 |
| Erbb2 | 0.00248 | -0.9038028 |
| Hif1an | 0.00182 | -0.93050492 |
| Cd300a | 0.27469 | -0.39703254 |
| Ahcyl1 | 0.23697 | 0.48886874 |
| D830046C22Rik | 0.00144 | -1.42226218 |
| Stag1 | 0.02082 | 0.63767326 |
| Trp53 | 0.01407 | -0.75756943 |
| Mbtps2 | 0.54993 | -0.30495407 |
| 9630041I06Rik | 0.01037 | -1.93179409 |
| 1700017B05Rik | 0.11104 | -0.38313872 |
| Nedd9 | 0.00753 | 0.66330395 |
| Abcc1 | 0.00159 | -0.94637023 |
| Msh3 | 0.0516 | -0.92212233 |
| Rps15a | 0.90928 | 0.05886391 |
| Sipa1l1 | 0.51935 | -0.44831483 |
| Tet3 | 0.0649 | -0.50925637 |
| Wars2 | 0.00144 | -1.31495282 |
| Zfp287 | 0.0017 | 1.62435435 |
| Nek8 | 0.2917 | -0.20279912 |
| Crhr1 | 0.63571 | -0.51245592 |
| Srrm4 | 0.00144 | -1.31903845 |
| Nop10 | 0.0332 | -0.55825675 |
| Zfp26 | 0.06641 | 0.39221267 |
| Arfgef3 | 0.00144 | -1.30017714 |
| Pbrm1 | 0.00243 | 0.88673518 |
| Proser3 | 0.26267 | 0.48633126 |
| Nup98 | 0.42272 | 0.13583324 |
| Ermard | 0.00396 | 0.74812307 |
| Xcr1 | 0.00144 | -1.28116048 |
| Olfr810 | 0.00286 | -1.1443198 |
| Sptb | 0.00144 | -1.31592062 |
| Gse1 | 0.00309 | -0.784502 |
| Sec61a1 | 0.00352 | -0.77138338 |
| Tsc22d1 | 0.0224 | 0.80277415 |
| Npr2 | 0.99953 | 0.00012874 |
| Adamts1 | 0.06476 | 0.50268088 |
| Pde4a | 0.95132 | -0.02675302 |
| 4732465J04Rik | 0.11638 | -0.66773702 |
| Nol6 | 0.03659 | -0.45629136 |
| Hgf | 0.10517 | -0.75965865 |
| Xpo4 | 0.05253 | -0.59460676 |
| 2700097O09Rik | 0.00881 | -1.73322411 |
| D330013E07Rik | 0.00144 | 2.5188943 |
| Tnfsf11 | 0.02024 | -0.94109882 |
| Zbtb26 | 0.00144 | 1.00416926 |
| Dgkb | 0.00144 | -1.28898079 |
| Shprh | 0.04339 | -0.83038016 |
| 5830415F09Rik | 0.04318 | -0.67415379 |
| Susd2 | 0.02707 | -0.78405655 |
| Ap1s2 | 0.07685 | 0.3146496 |
| Elmo1 | 0.02915 | 1.09545001 |
| Sox2ot | 0.00149 | -1.28183002 |
| Gtsf1l | 0.00144 | -1.28116048 |
| Plek2 | 0.13653 | -0.92184492 |
| Sec14l2 | 0.89741 | 0.03820036 |
| Tmem110 | 0.00835 | -0.74242852 |
| Ndel1 | 0.00564 | 0.75557016 |
| Get4 | 0.00741 | -0.81240296 |
| Krtap19-1 | 0.12352 | -0.41136759 |
| BC100530 | 0.20686 | 0.4062782 |
| Rassf8 | 0.01959 | -0.58749912 |
| Lin7a | 0.16852 | 0.75466275 |
| Fam105a | 0.67281 | -0.32411803 |
| Thnsl2 | 0.01012 | -0.72874969 |
| Nags | 0.00144 | -1.28116048 |
| Olfr390 | 0.04953 | -2.1797196 |
| Ppp4r4 | 0.29982 | -0.53083995 |
| Ubl3 | 0.01256 | -0.55320695 |
| Rdh9 | 0.14014 | 0.48402274 |
| Smg7 | 0.35803 | -0.14196383 |
| Srp72 | 0.29007 | 0.1912008 |
| Spata20 | 0.7112 | -0.17688884 |
| G3bp1 | 0.32376 | -0.26808677 |
| R3hcc1l | 0.00875 | 0.89995364 |
| Fzr1 | 0.0776 | -0.616845 |
| Popdc3 | 0.6408 | 0.13869821 |
| Prelp | 0.00659 | -0.74082114 |
| Polr2b | 0.02963 | 0.64275121 |
| Zfp207 | 0.02918 | 0.91791645 |
| E230015B07Rik | 0.00144 | -1.26934956 |
| Coq4 | 0.04066 | -0.70353276 |
| Scap | 0.00599 | -0.91788507 |
| Tmem168 | 0.76805 | -0.14352483 |
| Slc39a8 | 0.03595 | 0.97390371 |
| Dnah5 | 0.00144 | -1.30017714 |
| Grid2 | 0.00485 | -1.33590701 |
| Zdhhc17 | 0.00144 | 1.82087666 |
| Ppid | 0.00144 | 1.38091684 |
| Slc38a4 | 0.00144 | 1.59635103 |
| 4930543E12Rik | 0.00144 | -1.28116048 |
| 1700120B22Rik | 0.00144 | -1.28898079 |
| Tef | 0.96984 | 0.00745768 |
| Alg14 | 0.00651 | 0.73663286 |
| Rnf41 | 0.08706 | -0.29279185 |
| Ube2k | 0.0034 | 0.83096781 |
| Crxos | 0.00179 | -1.40595898 |
| Wdr27 | 0.01729 | -0.98762227 |
| Abca2 | 0.37689 | -0.31447507 |
| Palld | 0.04877 | -0.50288013 |
| Nnat | 0.01236 | -1.26451637 |
| Rfxank | 0.01514 | -0.80263106 |
| Prss40 | 0.01589 | -1.6980083 |
| Vmn1r226 | 0.00144 | -1.26934956 |
| Lrrc40 | 0.17504 | 0.31811828 |
| Zfp27 | 0.01535 | 0.65246625 |
| Erp44 | 0.84444 | 0.03100538 |
| Dbndd1 | 0.00145 | -1.16432455 |
| Urb2 | 0.02206 | 0.65642946 |
| Ube2v1 | 0.00828 | -0.78332612 |
| Rnf41 | 0.03732 | -0.58363656 |
| C1ql2 | 0.00152 | -1.25878908 |
| Cfap44 | 0.00144 | -1.29746368 |
| Cpsf4 | 0.13822 | -0.79997687 |
| Pydc4 | 0.0044 | -1.14548549 |
| Dcun1d4 | 0.00144 | -1.5634206 |
| Tmprss15 | 0.00144 | -1.28898079 |
| Kcnk10 | 0.00144 | -1.53823661 |
| Aars | 0.5953 | 0.2852642 |
| Mios | 0.06372 | 0.48597393 |
| Lamp2 | 0.11349 | 0.34826328 |
| Utp23 | 0.75164 | 0.07388846 |
| 4930519G04Rik | 0.19675 | -0.62406869 |
| Cd164 | 0.00377 | 1.0328358 |
| Serbp1 | 0.00166 | 1.03659693 |
| Areg | 0.01067 | -1.06721129 |
| Tmco2 | 0.00144 | -1.28898079 |
| Hrnr | 0.00356 | -1.08631 |
| Spg11 | 0.77581 | 0.07384303 |
| Arhgef17 | 0.06529 | -0.59634907 |
| Ifna2 | 0.04616 | -0.88410199 |
| Gjb6 | 0.00144 | -1.30017714 |
| Tsen2 | 0.37529 | -0.17336398 |
| Tdrd1 | 0.08989 | -2.44781353 |
| Tra2a | 0.00216 | 1.78319884 |
| Rad23a | 0.00481 | -0.68858781 |
| Pnma3 | 0.00144 | -1.25118919 |
| Olfr1254 | 0.00144 | -1.31552497 |
| Grem1 | 0.00144 | -1.30713006 |
| Cep78 | 0.06652 | 0.49188794 |
| Wtip | 0.00413 | -0.7436973 |
| Fkbp5 | 0.24575 | 0.41545776 |
| Wdr93 | 0.72317 | 0.19282907 |
| Drc1 | 0.03544 | -0.82135365 |
| Tnpo1 | 0.32276 | -0.16151549 |
| Gm9947 | 0.0068 | -1.13602825 |
| Edaradd | 0.32125 | -0.39641671 |
| Prrx1 | 0.40165 | -0.44575869 |
| Kat6b | 0.00144 | -1.30017714 |
| Tnpo2 | 0.07491 | -0.42235992 |
| Parp3 | 0.14746 | 0.37531107 |
| Avl9 | 0.01013 | 2.42306817 |
| Dut | 0.0654 | -0.61805695 |
| Loxl2 | 0.62858 | -0.10839449 |
| Sppl3 | 0.15616 | -0.5737643 |
| Atat1 | 0.98005 | -0.01096936 |
| Plxna1 | 0.32583 | -0.25682595 |
| Ptges3l | 0.36947 | 0.49575876 |
| Col27a1 | 0.07005 | 0.95221102 |
| BC023105 | 0.40601 | 0.32712811 |
| Gulp1 | 0.00144 | 1.80651596 |
| Npepps | 0.91491 | 0.01759002 |
| Gfpt1 | 0.64627 | -0.12586509 |
| Olfr1370 | 0.00144 | -1.30713006 |
| Sdc1 | 0.00162 | -1.15015443 |
| Col6a3 | 0.91681 | 0.02011094 |
| Cnot8 | 0.04949 | 0.798588 |
| Ckap5 | 0.98701 | 0.01712061 |
| Amdhd2 | 0.02134 | -0.70317842 |
| Gcn1l1 | 0.18582 | -0.42142936 |
| Gm9885 | 0.00789 | -1.24517406 |
| Dleu7 | 0.00314 | -1.14703327 |
| Samm50 | 0.41609 | -0.33696676 |
| Kdr | 0.21425 | -0.60633928 |
| Dlc1 | 0.00267 | 2.41079821 |
| Sv2b | 0.05522 | -0.69538555 |
| P2rx6 | 0.00144 | -1.26560808 |
| Smpd3 | 0.17023 | -0.53351723 |
| Capza2 | 0.00969 | 1.86100974 |
| Hmox2 | 0.24629 | -0.26855046 |
| Slc17a4 | 0.00145 | -1.22376446 |
| Dek | 0.01014 | 1.38369378 |
| 1810058I24Rik | 0.00875 | -0.91167905 |
| Crtac1 | 0.00144 | -1.20579466 |
| Mnt | 0.00515 | -0.75925271 |
| Csnk1d | 0.64958 | -0.10203828 |
| Ncoa7 | 0.00347 | 1.28163801 |
| Chd6 | 0.0814 | 0.59965519 |
| Rfx4 | 0.00144 | -1.32575997 |
| Rhpn1 | 0.12239 | -0.81581966 |
| Ubash3b | 0.66225 | -0.10381698 |
| Fam110c | 0.00144 | -1.32599123 |
| Far1 | 0.19315 | 0.56804473 |
| Ahi1 | 0.00157 | 1.55976325 |
| Tenm2 | 0.40287 | -0.35245253 |
| Tpcn1 | 0.00455 | -0.72258403 |
| Defa23 | 0.00158 | -1.2446827 |
| Cul3 | 0.00597 | 0.94664227 |
| Evc | 0.00372 | -0.85677011 |
| Dnpep | 0.30532 | -0.43957521 |
| Gm3604 | 0.5453 | 0.26816362 |
| Nxf3 | 0.00144 | -1.30017714 |
| Nol4l | 0.43105 | 0.18413624 |
| Irf6 | 0.00205 | -1.90897064 |
| Nol10 | 0.04381 | -1.07522817 |
| Nupl2 | 0.4639 | 0.21135091 |
| Jrkl | 0.00145 | -1.2388121 |
| Nr2e1 | 0.00155 | -1.41135178 |
| Dnajb4 | 0.58361 | 0.14793584 |
| Smchd1 | 0.46836 | -0.54486442 |
| Rbm48 | 0.00346 | -1.19576403 |
| Ablim2 | 0.22462 | -0.57899366 |
| Sox6 | 0.00455 | 1.57001109 |
| Dsg1a | 0.00144 | -1.31495282 |
| Arsk | 0.00664 | 0.83918519 |
| Etaa1 | 0.00144 | 2.28623032 |
| Fsip1 | 0.02892 | -1.21069326 |
| Chd1 | 0.00626 | 1.27575511 |
| Col20a1 | 0.22827 | -0.33359195 |
| 4921506M07Rik | 0.01622 | -1.42782775 |
| Denr | 0.00144 | 1.31863255 |
| Lrrc16a | 0.0352 | 0.43677592 |
| 0610025J13Rik | 0.56475 | -0.14032751 |
| Lancl2 | 0.18081 | 0.24411694 |
| Bag4 | 0.00144 | 1.95481919 |
| Onecut3 | 0.00144 | -1.27699683 |
| Slc7a9 | 0.07971 | -1.2422705 |
| Capn6 | 0.23201 | -0.68804345 |
| Cpsf4 | 0.01945 | -0.53354575 |
| Kdelr3 | 0.75421 | -0.07911661 |
| Ctdspl | 0.02909 | -0.46585913 |
| Dmrtc1b | 0.09794 | -0.96298956 |
| Lrrc71 | 0.49959 | 0.18777836 |
| Atxn7l1 | 0.23044 | -0.41274187 |
| Cldnd1 | 0.33817 | 0.39091807 |
| Stag2 | 0.84885 | 0.14445604 |
| Zfp236 | 0.01807 | -1.44693979 |
| Otos | 0.07552 | -1.41777476 |
| Utp6 | 0.51464 | 0.11096588 |
| BC021785 | 0.33395 | -0.476079 |
| Malt1 | 0.0283 | 2.03795093 |
| Ecd | 0.98849 | 0.0044748 |
| Kdelc1 | 0.00186 | -1.06484863 |
| Rrbp1 | 0.50624 | -0.22390187 |
| Gmps | 0.0021 | 1.16710295 |
| Sacs | 0.13649 | 1.14091644 |
| Cyp2b10 | 0.00144 | -1.30713006 |
| Ctnnb1 | 0.03222 | -0.66418121 |
| Slpi | 0.05711 | 0.92955563 |
| Plxnd1 | 0.00446 | -0.67971486 |
| Fxyd6 | 0.02257 | -0.67899792 |
| Dmap1 | 0.90084 | -0.06418173 |
| Sgsh | 0.02527 | -1.21615515 |
| Plcl2 | 0.00144 | -1.27992547 |
| Ccdc116 | 0.04014 | -1.36992119 |
| Dcaf13 | 0.50638 | -0.40453967 |
| Ifngr2 | 0.67232 | -0.17561293 |
| Gm16897 | 0.00326 | 1.12772559 |
| Ercc8 | 0.17396 | -0.60243577 |
| Pusl1 | 0.01898 | -0.54795624 |
| Ctage5 | 0.17396 | 0.39484079 |
| Taf1d | 0.00144 | 1.68955982 |
| Mipol1 | 0.00711 | 1.05851022 |
| Rnf4 | 0.53841 | 0.19755835 |
| Cse1l | 0.0823 | 0.7606951 |
| Cntnap5c | 0.00144 | -1.29878664 |
| Gnb1 | 0.87753 | -0.02861945 |
| Tmem186 | 0.41958 | 0.15433534 |
| Pias2 | 0.00278 | 0.88487893 |
| Fam161b | 0.44856 | 0.2802518 |
| Cyfip2 | 0.0128 | 1.01267165 |
| Kank1 | 0.00144 | -1.72414556 |
| Zc3h6 | 0.35281 | -0.28287286 |
| Rfwd3 | 0.18744 | 0.24870381 |
| Calcr | 0.00144 | -1.26934956 |
| Rnf38 | 0.01677 | -0.98034558 |
| 2210018M11Rik | 0.00412 | 0.82806336 |
| Cda | 0.00976 | -0.68694892 |
| Toe1 | 0.32529 | 0.23472358 |
| Comtd1 | 0.00144 | -1.34857497 |
| Espn | 0.29908 | -0.79434237 |
| Pax6 | 0.92206 | 0.1581753 |
| Hcrtr2 | 0.00173 | -1.6426896 |
| Zfp106 | 0.90462 | -0.01999961 |
| Trim44 | 0.00422 | 1.00672009 |
| 4933409G03Rik | 0.60164 | -0.44534973 |
| Olfr450 | 0.00144 | -1.28898079 |
| Uqcc1 | 0.21111 | -0.38111744 |
| Pdxk-ps | 0.47913 | -0.23824876 |
| Tchh | 0.27442 | 0.39223451 |
| Setd1b | 0.00317 | -0.80340474 |
| Aldob | 0.0909 | 1.15928253 |
| Abcb7 | 0.13958 | -0.85682098 |
| Fam76b | 0.34533 | -0.39868924 |
| Urad | 0.55609 | -0.17077004 |
| Gm33948 | 0.00144 | -1.28116048 |
| Nsg1 | 0.06037 | -0.41893453 |
| Sptbn1 | 0.07009 | -0.39415664 |
| Ankrd63 | 0.49993 | -0.40008318 |
| Med12l | 0.1162 | -0.40160859 |
| Git2 | 0.50989 | -0.10184451 |
| R3hdm1 | 0.06431 | 1.24399959 |
| Gm38764 | 0.00402 | -1.40387265 |
| Slc35d3 | 0.87578 | 0.05633651 |
| Alox12 | 0.01679 | -0.94175351 |
| Cd99l2 | 0.01231 | 1.99027203 |
| Dffb | 0.00589 | 1.25776311 |
| 9330168O09Rik | 0.01456 | -1.03105883 |
| Slc15a3 | 0.00364 | -0.76863261 |
| Mtf1 | 0.05922 | 0.43497726 |
| Il1rap | 0.08749 | 0.45321732 |
| D030028M11Rik | 0.00144 | -1.27165604 |
| Snd1 | 0.0032 | -0.97998215 |
| Golga4 | 0.1997 | 0.44526537 |
| Wdr48 | 0.02575 | -0.48125016 |
| Synj2bp | 0.55734 | -0.2422407 |
| Hnrnpd | 0.00209 | 0.81390124 |
| Bmyc | 0.87804 | -0.04284667 |
| Rngtt | 0.22526 | 0.64501388 |
| Hax1 | 0.4153 | -0.20615504 |
| Fuom | 0.00826 | -1.00509349 |
| Speer9-ps1 | 0.00144 | -1.28898079 |
| Prkg2 | 0.12695 | -3.09051102 |
| Cd22 | 0.09715 | -3.23581043 |
| Fanca | 0.00144 | -1.48248633 |
| Rarres2 | 0.00253 | -0.85366555 |
| A330041J22Rik | 0.03874 | -0.99900538 |
| Samhd1 | 0.19699 | 0.28288677 |
| Slc9a1 | 0.17237 | -0.31695429 |
| Efcab2 | 0.81884 | -0.0963355 |
| Cplx1 | 0.90158 | 0.03999757 |
| Dmrt3 | 0.00144 | -1.26428512 |
| Olfr275 | 0.03843 | -1.34100682 |
| Gramd3 | 0.02785 | -0.50192073 |
| A630072M18Rik | 0.77945 | -0.06397632 |
| Pwwp2a | 0.04965 | 0.88733657 |
| Fam178a | 0.63731 | 0.20687951 |
| Dpp6 | 0.00521 | -1.05862264 |
| Naa25 | 0.5471 | 0.09740666 |
| Ctdspl2 | 0.04056 | 0.72446606 |
| Gm5150 | 0.00144 | -1.45950387 |
| Sema5a | 0.05067 | -1.09267033 |
| Ppp2r2b | 0.56467 | -0.31836253 |
| Dlx1 | 0.13215 | -0.48088065 |
| Hcrtr2 | 0.00145 | -1.2388121 |
| Mast4 | 0.00144 | -1.21868187 |
| 4930525G20Rik | 0.04698 | -0.91567309 |
| Phf20l1 | 0.00144 | 1.24446866 |
| Nme7 | 0.047 | 0.65759735 |
| 9630050P21Rik | 0.96737 | -0.02046685 |
| Crim1 | 0.15163 | 0.33119376 |
| Trp53bp1 | 0.15791 | 0.30431855 |
| Mpp7 | 0.48431 | -0.37002749 |
| 1700009N14Rik | 0.00144 | -1.31495282 |
| Grk6 | 0.01128 | -0.57549745 |
| AK010878 | 0.01166 | 0.6479957 |
| Uvssa | 0.75781 | 0.07920268 |
| Hdac8 | 0.21263 | 0.65697664 |
| Tmem140 | 0.00849 | -0.75622104 |
| Krtap16-3 | 0.10782 | -0.44192572 |
| Tmco4 | 0.01125 | -0.89918596 |
| Cd200r1 | 0.01809 | -1.85661854 |
| Twf1 | 0.02685 | 0.71257919 |
| Gjd3 | 0.00175 | -1.76298291 |
| Olfr1141 | 0.00521 | -1.05862264 |
| Gmds | 0.26612 | 0.24444044 |
| Ankrd33b | 0.0179 | -0.77365085 |
| Cdca8 | 0.79249 | -0.26947327 |
| Olfr309 | 0.00144 | -1.31495282 |
| Cdc37 | 0.00215 | -0.89982555 |
| Rnf19a | 0.91127 | 0.06207622 |
| Obox7 | 0.0352 | -0.94397117 |
| Morn1 | 0.03255 | -0.71795441 |
| Gm11545 | 0.0297 | -1.35601513 |
| Mtmr14 | 0.82487 | 0.11855298 |
| C77370 | 0.27067 | -0.43337234 |
| Lrp1b | 0.00145 | -1.2388121 |
| Ipo8 | 0.00152 | -1.25423771 |
| Dr1 | 0.38994 | -0.19381523 |
| Cpeb1 | 0.11036 | 0.53588372 |
| Lrrc8d | 0.59706 | -0.45296102 |
| Ppp1r1c | 0.00144 | -1.27165604 |
| Abl1 | 0.15689 | -0.73568149 |
| Csde1 | 0.11636 | 0.48550471 |
| Smarca5-ps | 0.94501 | 0.01254166 |
| Pigo | 0.21023 | -0.46631682 |
| Slc7a6 | 0.00162 | 1.56250333 |
| Gm17473 | 0.93313 | -0.04646769 |
| Rcbtb1 | 0.18496 | -0.42623552 |
| Tmbim1 | 0.40622 | -0.23797152 |
| Upk3a | 0.10151 | -0.92497781 |
| Tfe3 | 0.0015 | -1.29511914 |
| Oaz1 | 0.66176 | -0.24569663 |
| Crem | 0.2505 | 0.23230083 |
| BC048507 | 0.04831 | -0.92199673 |
| Mvp | 0.18214 | -0.41161042 |
| Lrrn4 | 0.0073 | -1.15314387 |
| A930104D05Rik | 0.00145 | -1.17894292 |
| Ppy | 0.00144 | -1.31884045 |
| Crhbp | 0.92503 | 0.05411572 |
| A830018L16Rik | 0.00144 | -1.28116048 |
| Gabrb2 | 0.00144 | -1.29085005 |
| Nxf7 | 0.00149 | -1.22582153 |
| Runx2 | 0.00149 | -1.49231123 |
| Lpar2 | 0.00144 | -1.27165604 |
| Eif4g2 | 0.00144 | 1.15095995 |
| Nrbp1 | 0.00144 | -1.23768878 |
| Itga1 | 0.01068 | 0.74946644 |
| Hsd3b3 | 0.00144 | -1.49245118 |
| Pmm1 | 0.0425 | -0.64004461 |
| Itga2b | 0.0574 | -0.75480521 |
| Ccdc174 | 0.78385 | 0.10380023 |
| Mkln1 | 0.00144 | 4.00690799 |
| Itgb4 | 0.00144 | -1.25927895 |
| St14 | 0.00798 | -1.03220606 |
| Lama2 | 0.13403 | -0.68017424 |
| 4930402H24Rik | 0.00336 | 0.75847719 |
| Kctd20 | 0.09005 | -0.35438936 |
| Themis | 0.00144 | -1.29085005 |
| Celf6 | 0.00144 | -1.25118919 |
| 2900011O08Rik | 0.00144 | -1.25118919 |
| Slc25a16 | 0.00334 | 1.54915964 |
| LOC105244061 | 0.00144 | -1.32766452 |
| C530008M17Rik | 0.00279 | -0.97691669 |
| Sdha | 0.1648 | -0.41042028 |
| Birc2 | 0.13911 | 0.31305386 |
| Ttll5 | 0.46435 | -0.21257772 |
| Gorasp1 | 0.83308 | -0.07172066 |
| Prlr | 0.28559 | 0.40714022 |
| Gm4763 | 0.02043 | -0.96631129 |
| 4930419G24Rik | 0.00144 | -1.20555712 |
| Rrp1b | 0.34144 | 0.20141107 |
| AI467606 | 0.17375 | -0.31417383 |
| Trmt10a | 0.00616 | 2.03121965 |
| Olfr734 | 0.08867 | -2.56863382 |
| Ptrf | 0.05691 | -0.66591734 |
| Fbxw21 | 0.31282 | -0.35916915 |
| Ubl5 | 0.01503 | -0.72211453 |
| Trim35 | 0.00276 | -1.55379677 |
| Gm1965 | 0.06007 | -1.9883894 |
| 2810429I04Rik | 0.0063 | -1.05452194 |
| Tada2a | 0.41535 | 0.20922426 |
| Sybu | 0.00144 | -1.33632959 |
| Rapgef4 | 0.42358 | 0.76512987 |
| Cyp2s1 | 0.08125 | -1.38944037 |
| Gm16794 | 0.18029 | -0.47537117 |
| Aph1b | 0.551 | -0.09484887 |
| Gm3500 | 0.00536 | 2.04171441 |
| Ccnc | 0.09897 | 1.17683824 |
| Phf3 | 0.03279 | -0.93739244 |
| Plekhm3 | 0.06194 | -0.32617892 |
| Abcd1 | 0.93088 | -0.04231294 |
| Dmtf1 | 0.00144 | 2.74672792 |
| 1810013L24Rik | 0.2032 | 0.28155778 |
| Ccdc81 | 0.00144 | -1.31884045 |
| Anxa4 | 0.50788 | -0.1367352 |
| 5330426P16Rik | 0.00144 | 3.0325109 |
| Limch1 | 0.00697 | -1.04397027 |
| Myg1 | 0.19557 | -0.79582329 |
| Zfp943 | 0.00151 | -1.17792995 |
| Cct8 | 0.01757 | 0.69724741 |
| Tmem167 | 0.8905 | 0.0263741 |
| Exoc4 | 0.00144 | -1.30017714 |
| 2900079G21Rik | 0.01266 | -0.88981344 |
| Sh3d21 | 0.28901 | -0.27821758 |
| Emilin3 | 0.19697 | -0.27948975 |
| Fchsd1 | 0.02323 | -0.45889914 |
| Dgat1 | 0.46564 | -0.2262651 |
| Commd2 | 0.20839 | -0.25938413 |
| Kif1bp | 0.00693 | -0.66624098 |
| Pfdn1 | 0.00218 | -0.78517708 |
| Skida1 | 0.52119 | -0.16383565 |
| Slamf6 | 0.00145 | -1.22376446 |
| Ric8b | 0.03765 | 0.56477779 |
| Glra2 | 0.00144 | -1.27165604 |
| Itpa | 0.50224 | -0.19286502 |
| Pramel6 | 0.00145 | -1.22376446 |
| A2m | 0.0928 | -0.94387656 |
| Dzank1 | 0.00144 | -1.28116048 |
| Crebzf | 0.04189 | -0.84696728 |
| Eif3c | 0.01508 | 0.64463661 |
| Pi4ka | 0.20053 | -0.23559372 |
| Fgf11 | 0.07676 | 0.42477052 |
| 6430548M08Rik | 0.01572 | -1.21424849 |
| Srgap1 | 0.01235 | -0.57345694 |
| Arid3b | 0.10513 | -0.29182547 |
| Hars | 0.00701 | 1.08859031 |
| Olfml2b | 0.29155 | -0.33895268 |
| Hecw1 | 0.00144 | -1.29049509 |
| Pomt1 | 0.11007 | -0.66093024 |
| Myt1l | 0.00145 | -1.26219879 |
| LOC102635786 | 0.00144 | -1.76023631 |
| Foxo1 | 0.04205 | -0.39145508 |
| Tgfbr1 | 0.00144 | 1.37739784 |
| Whsc1l1 | 0.00144 | 1.18225784 |
| Bcas3os2 | 0.00144 | -1.28116048 |
| 1500011K16Rik | 0.00686 | -0.98895596 |
| Arl6ip6 | 0.94255 | 0.01765367 |
| Med19 | 0.2614 | -0.20751006 |
| Afg3l1 | 0.15062 | -0.45884026 |
| Dnase2a | 0.46388 | -0.44987493 |
| 2310031A07Rik | 0.12434 | -0.87369633 |
| Lrrn4cl | 0.03792 | 0.50571883 |
| Poln | 0.19824 | -0.49329348 |
| Cftr | 0.91068 | -0.10137938 |
| Pdcd6ip | 0.01028 | 0.71682131 |
| Dao | 0.00247 | -1.12736318 |
| Atf3 | 0.09398 | -0.83421046 |
| Prom2 | 0.00145 | -1.25646236 |
| Olfr730 | 0.00145 | -1.2388121 |
| Kirrel | 0.04262 | -0.98782471 |
| Zfp263 | 0.06464 | 0.38581067 |
| Stk35 | 0.04414 | -0.45978474 |
| 1700042G07Rik | 0.00144 | -1.29746368 |
| Tmod2 | 0.1683 | -0.26124622 |
| Fbxl3 | 0.00144 | 1.25117814 |
| Xpr1 | 0.00726 | 0.71809189 |
| Ehmt1 | 0.00144 | -1.27165604 |
| St6galnac3 | 0.943 | 0.01944306 |
| Hnrnpr | 0.08296 | 0.92812923 |
| Tnfrsf21 | 0.06841 | 0.44856452 |
| Ccdc150 | 0.02168 | -1.01056962 |
| Paqr9 | 0.34512 | -0.32261892 |
| Cdpf1 | 0.02881 | -0.63597925 |
| Dpm1 | 0.11529 | 0.35890719 |
| Ndrg3 | 0.01062 | -0.79357021 |
| Plxna4os2 | 0.08683 | -2.51029355 |
| Hdhd2 | 0.34858 | -0.20844511 |
| Fgf2os | 0.44519 | -0.43526948 |
| Tex18 | 0.01466 | -1.67676643 |
| Spata1 | 0.0378 | -0.93613993 |
| Mtmr4 | 0.05815 | -0.34406152 |
| Ranbp6 | 0.00279 | 2.50016054 |
| Paics | 0.00846 | -0.66921825 |
| Ankrd13c | 0.02933 | 0.52041233 |
| Vangl1 | 0.14844 | 0.2629575 |
| Atp11b | 0.21435 | -0.47564401 |
| V1rd19 | 0.00144 | -1.3348153 |
| Fmr1nb | 0.7884 | 0.18081474 |
| Ppp2r5e | 0.06043 | -0.40381493 |
| A730011C13Rik | 0.00147 | 2.06824305 |
| Rab26 | 0.21733 | -0.53668449 |
| 1700096K18Rik | 0.12829 | -0.37823109 |
| A330040F15Rik | 0.02506 | -1.03780074 |
| Cacul1 | 0.00647 | 0.8770321 |
| Rbm25 | 0.00523 | 2.47676823 |
| Tbc1d23 | 0.00802 | 0.99568401 |
| Ddo | 0.78135 | 0.06157383 |
| Setd5 | 0.43043 | -0.16025262 |
| Rpgrip1 | 0.0079 | -1.16902461 |
| Cops7a | 0.15629 | -0.41104993 |
| Nit2 | 0.67002 | 0.11456559 |
| Gm29738 | 0.36043 | 1.55873096 |
| Epcam | 0.00144 | -1.50541481 |
| Ppp2r5a | 0.03744 | -0.40557772 |
| 5330417C22Rik | 0.00318 | -1.16999514 |
| Prepl | 0.09255 | -0.36205404 |
| Prss43 | 0.00144 | -1.29746368 |
| Clic6 | 0.32703 | -0.65778829 |
| A730035I17Rik | 0.00144 | -1.26428512 |
| Kctd20 | 0.02396 | -0.78375445 |
| Bivm | 0.11567 | -0.51017157 |
| Ccdc78 | 0.17829 | -0.59968898 |
| Rhbdl2 | 0.0746 | -1.02494508 |
| Cep170b | 0.00149 | -0.99852508 |
| Ly6g6f | 0.00145 | -1.21051401 |
| Lgi2 | 0.42969 | -0.42662115 |
| Thumpd1 | 0.23018 | -0.2652955 |
| Commd6 | 0.768 | 0.09896345 |
| Ptprk | 0.22441 | 0.40971133 |
| Chsy3 | 0.0017 | -1.24950944 |
| Ubn2 | 0.00144 | 3.06932522 |
| Ccdc108 | 0.00144 | -1.32155392 |
| Abcf2 | 0.70384 | 0.16102958 |
| Kcnj3 | 0.00382 | 0.90658733 |
| Gm16702 | 0.00144 | -1.29878664 |
| 2610002M06Rik | 0.03358 | 0.48137408 |
| Ccnh | 0.0112 | 0.55546391 |
| 4930558K02Rik | 0.01134 | -1.02600084 |
| Agtpbp1 | 0.65277 | 0.08856861 |
| Clk4 | 0.00462 | 1.47935499 |
| Dnajc25 | 0.01998 | -0.52747757 |
| Krt20 | 0.39116 | -0.45699788 |
| Dapk1 | 0.57537 | 0.37148624 |
| Ier3ip1 | 0.02319 | -0.45328733 |
| Cwc15 | 0.25453 | 0.1755096 |
| Plp1 | 0.73431 | 0.07692674 |
| Rprml | 0.25949 | -0.71378528 |
| Yme1l1 | 0.00144 | 1.51808245 |
| Myb | 0.00145 | -1.2388121 |
| Cars | 0.61144 | 0.11369558 |
| N4bp2l2 | 0.64857 | 0.18806263 |
| B430209F14Rik | 0.00158 | -1.15468698 |
| Cybb | 0.95258 | 0.01933471 |
| Dlgap1 | 0.00144 | -1.28898079 |
| Gabrg2 | 0.00144 | -1.30713006 |
| Pfn3 | 0.59932 | -0.4274321 |
| Cfap70 | 0.00144 | -1.35679644 |
| Dnlz | 0.05861 | -0.54862542 |
| Ppfibp1 | 0.28813 | -0.32701224 |
| Aen | 0.60872 | 0.12371788 |
| 4932438A13Rik | 0.38418 | -0.44969803 |
| B930095G15Rik | 0.83558 | 0.0611771 |
| Ncoa4 | 0.00631 | 0.78484396 |
| Cd8a | 0.06768 | -0.71931927 |
| Usp43 | 0.51123 | -0.25899925 |
| Naa35 | 0.97319 | -0.00793262 |
| Epha5 | 0.00144 | -1.21868187 |
| Pdzd11 | 0.01348 | -1.68401917 |
| Mief1 | 0.04208 | -1.12179417 |
| Ndnf | 0.35435 | 0.58739679 |
| Cux1 | 0.45101 | -0.20694353 |
| Gtf2i | 0.0619 | -0.39271119 |
| Trim30a | 0.8213 | 0.04235653 |
| Il12a | 0.06893 | -0.97264118 |
| Mllt6 | 0.06149 | -0.66202975 |
| Bsn | 0.0475 | -0.89918478 |
| Magi1 | 0.04482 | 0.45597922 |
| Evc2 | 0.0229 | -0.46736355 |
| 4932442L08Rik | 0.00144 | -1.28898079 |
| Map3k1 | 0.55094 | -0.149704 |
| Chl1 | 0.12658 | 0.62577697 |
| Col23a1 | 0.43546 | 0.14415876 |
| Naa25 | 0.00231 | 1.11890702 |
| Egr3 | 0.00858 | 2.70540593 |
| Atf7ip | 0.50208 | 0.13921539 |
| Ryr2 | 0.00734 | 1.59389765 |
| Nek1 | 0.00284 | 1.23812306 |
| Rassf10 | 0.11809 | -0.68903807 |
| Polr1a | 0.18027 | 0.49000634 |
| Has1 | 0.00144 | -1.28116048 |
| LOC102631977 | 0.18685 | -0.25245192 |
| 4930451G09Rik | 0.01389 | -0.80580311 |
| Rab43 | 0.71702 | 0.0918249 |
| 2410017I17Rik | 0.01758 | 1.28281742 |
| Hspbap1 | 0.01654 | -0.72806923 |
| Ccdc130 | 0.6157 | -0.08505605 |
| 4930447F04Rik | 0.00175 | -1.05305018 |
| Plekhh1 | 0.39326 | -0.53865006 |
| Sema3e | 0.00144 | -1.26560808 |
| Ugt8a | 0.00149 | -1.22582153 |
| Olfr1019 | 0.00145 | -1.2388121 |
| Prkrir | 0.02013 | 0.69411907 |
| Rabep1 | 0.53815 | 0.26809523 |
| Stk24 | 0.29809 | -0.27333268 |
| Rrbp1 | 0.00286 | 0.90286159 |
| Olfr524 | 0.00527 | -1.5623651 |
| Abcf3 | 0.83809 | -0.0691478 |
| Spag11a | 0.02961 | -0.94782714 |
| Olfr1102 | 0.00149 | -1.22582153 |
| Rabl6 | 0.09276 | 0.81465666 |
| Bcl2 | 0.0057 | 2.58736018 |
| Cebpg | 0.00484 | 0.70451526 |
| Lpar4 | 0.00144 | 2.36593685 |
| Mib1 | 0.00419 | 1.39999722 |
| 2810474O19Rik | 0.00144 | 2.44751157 |
| Fam13c | 0.00144 | -1.70197953 |
| Gm17767 | 0.00144 | -1.27165604 |
| Fbxo28 | 0.17476 | 0.36150584 |
| Ccdc172 | 0.00144 | -1.37665893 |
| Timm17b | 0.01482 | -0.92633221 |
| Arhgap27 | 0.15206 | -0.32160999 |
| 5031439G07Rik | 0.97645 | 0.00507856 |
| Rbx1 | 0.0041 | -0.84701877 |
| Rassf4 | 0.00837 | -1.3606212 |
| Gltp | 0.84489 | 0.04595173 |
| Asah2 | 0.00144 | 2.63264666 |
| Vegfb | 0.28323 | -0.41218906 |
| Rpl18 | 0.01937 | -0.84980161 |
| Wbscr17 | 0.83027 | -0.09454364 |
| Cdh20 | 0.01216 | 1.24000782 |
| Sytl5 | 0.06095 | -0.32696373 |
| Ddx59 | 0.22261 | 0.52529706 |
| Slc25a35 | 0.44924 | 0.32923601 |
| Odc1 | 0.37714 | 0.27849618 |
| Arcn1 | 0.02103 | 0.59128082 |
| Zfp651 | 0.32119 | -0.46807101 |
| Npepl1 | 0.1586 | -0.46182376 |
| Amz2 | 0.18728 | 0.60153757 |
| Prr15l | 0.00144 | -1.28116048 |
| Bud13 | 0.00492 | -1.98161948 |
| Lrig2 | 0.00597 | -1.11645509 |
| Ifi205 | 0.44878 | 0.2242323 |
| Ubqln1 | 0.48044 | 0.17374157 |
| Coa4 | 0.28402 | -0.41366171 |
| Gnpda2 | 0.00679 | 0.63809288 |
| Daw1 | 0.00328 | -1.41692424 |
| Camta1 | 0.92249 | -0.03262155 |
| Anxa6 | 0.31127 | -0.32597341 |
| Ccdc178 | 0.00144 | -1.26560808 |
| Pop1 | 0.35661 | -0.42128495 |
| Lnx1 | 0.78683 | 0.07562586 |
| Birc3 | 0.00209 | 1.69513999 |
| Lsm2 | 0.07728 | -0.41179704 |
| Tctn3 | 0.41101 | 0.20079419 |
| Vsx1 | 0.00438 | -1.69113109 |
| Gm3764 | 0.01741 | -0.55227387 |
| Esyt2 | 0.84674 | -0.03634325 |
| Pcdh19 | 0.02913 | 0.52378011 |
| Erbb3 | 0.77653 | 0.07188979 |
| Rab3d | 0.01775 | -0.56785481 |
| Ddx11 | 0.56493 | 0.17068732 |
| Crisp2 | 0.00144 | -1.20555712 |
| Mrpl50 | 0.47796 | 0.25539113 |
| Gm14410 | 0.00144 | 2.3121426 |
| Icosl | 0.23465 | -0.99304713 |
| Arid5b | 0.47145 | -0.14808905 |
| Chdh | 0.00144 | -1.27165604 |
| Cpeb3 | 0.56773 | -0.11120969 |
| Cbln4 | 0.00144 | -1.28116048 |
| Yipf6 | 0.12884 | -0.2864537 |
| Plxna2 | 0.02351 | 0.58898836 |
| Hmgxb3 | 0.06372 | -0.43280696 |
| Rad54l | 0.00144 | -1.28116048 |
| Pknox1 | 0.44194 | -0.12207211 |
| Ptcd3 | 0.02164 | 0.5470248 |
| 2010111I01Rik | 0.30021 | -0.19562894 |
| Slc4a10 | 0.48959 | -0.57534128 |
| 1700008A07Rik | 0.11285 | -0.95635284 |
| Baat | 0.01023 | -1.01602953 |
| Rbm11 | 0.00144 | -1.28116048 |
| Thpo | 0.35713 | 0.50940649 |
| Il1rn | 0.00144 | -1.27165604 |
| Foxn2 | 0.12371 | 0.34562246 |
| Tob2 | 0.0026 | -1.71518955 |
| Zfyve26 | 0.95442 | 0.02684875 |
| Krt13 | 0.5273 | 0.68738713 |
| Sult2a5 | 0.00229 | -1.40472397 |
| Rabggtb | 0.11553 | 0.50443786 |
| Fam186b | 0.93078 | 0.1245831 |
| Dnaaf5 | 0.02922 | -0.57859837 |
| Rbm15 | 0.14412 | -0.27967749 |
| Nacc1 | 0.04466 | -0.68154887 |
| Rai14 | 0.06797 | 0.51600577 |
| Plekha6 | 0.63524 | 0.1382109 |
| Dip2b | 0.38691 | 0.24003915 |
| C230057H02Rik | 0.00144 | -1.5686593 |
| Tbx20 | 0.06076 | -0.41548227 |
| Pax5 | 0.1296 | -0.89061332 |
| Cnst | 0.18403 | 0.59268002 |
| Tmem115 | 0.10141 | -0.48662538 |
| Dnajc24 | 0.00998 | -0.5760656 |
| Ngp | 0.11133 | -1.62753621 |
| Ccdc69 | 0.09376 | 0.45481884 |
| Efhd2 | 0.00969 | -0.6595325 |
| Ppp6r2 | 0.03857 | -0.43696477 |
| Synj2bp | 0.00144 | 3.86129045 |
| Gm5108 | 0.01356 | -1.10879133 |
| Capzb | 0.4399 | -0.39089806 |
| Lrp2bp | 0.6098 | -0.16529859 |
| Jmjd6 | 0.75496 | -0.07418219 |
| Tdrkh | 0.15426 | -0.62871068 |
| Fgd1 | 0.12737 | -0.52359044 |
| H2-Q10 | 0.00382 | -1.06182007 |
| Atr | 0.00154 | 1.39432175 |
| Fibp | 0.05113 | -0.45031101 |
| Zscan18 | 0.04832 | 0.95104201 |
| Ankrd31 | 0.00144 | -1.28797948 |
| Zp3 | 0.24282 | -0.44514763 |
| Slc35b2 | 0.00163 | -1.01459896 |
| Ccdc167 | 0.96417 | -0.02308058 |
| Gpbp1l1 | 0.64709 | -0.09027865 |
| Sez6l | 0.03887 | -1.32257561 |
| Anapc1 | 0.00942 | 1.67379339 |
| Ltn1 | 0.00144 | 2.27982871 |
| Zfp202 | 0.48283 | 0.15352247 |
| Gtpbp3 | 0.2151 | -0.29836442 |
| Zdhhc19 | 0.00248 | -1.17452065 |
| Ldlr | 0.00144 | -1.39708866 |
| C230052I12Rik | 0.0071 | 1.08442881 |
| Cited4 | 0.08191 | -0.89293723 |
| Rab27a | 0.00673 | 1.34850533 |
| 1700037C18Rik | 0.13067 | -0.50073883 |
| 1700022A22Rik | 0.05164 | -1.27470585 |
| Lmnb2 | 0.1885 | -0.32474714 |
| Abhd14a | 0.1002 | -0.67528076 |
| Abcc12 | 0.01771 | -2.43903703 |
| Ippk | 0.20346 | -0.30810113 |
| Eif4e2 | 0.23621 | 0.74287698 |
| Lat2 | 0.40424 | -0.31447216 |
| Dnah8 | 0.00144 | -1.20555712 |
| Impdh2 | 0.3316 | -0.22053621 |
| 1700067K01Rik | 0.21779 | -0.929811 |
| L3mbtl4 | 0.00144 | -1.25118919 |
| Slfn1 | 0.00144 | -1.29049509 |
| Retnlg | 0.01025 | 2.4344807 |
| Depdc5 | 0.00144 | -1.26560808 |
| Wtap | 0.00144 | 1.4121874 |
| Afm | 0.00144 | -1.28116048 |
| Rnf168 | 0.00723 | 0.82370174 |
| Eif2b4 | 0.38643 | -0.25282785 |
| 4933406C10Rik | 0.12831 | -0.61429797 |
| Eif3d | 0.01187 | -1.05088605 |
| Xrra1 | 0.11843 | -0.90435578 |
| Itgax | 0.00177 | -1.15847607 |
| Crbn | 0.01326 | 1.14719792 |
| Mrc2 | 0.00144 | -1.08089698 |
| Txnl4b | 0.01515 | -0.58694902 |
| Clstn2 | 0.30907 | 0.42673471 |
| Gm34996 | 0.00144 | -1.31884045 |
| C230012O17Rik | 0.00144 | -1.24811894 |
| Phc2 | 0.0242 | -1.03617482 |
| Col4a4 | 0.04091 | 0.7868746 |
| Ctnnb1 | 0.00817 | 0.72206199 |
| Apool | 0.00144 | 1.99582509 |
| Bmx | 0.00595 | -1.16184234 |
| Sybu | 0.52952 | 0.11122652 |
| Gatc | 0.9809 | 0.00444328 |
| Adamts16 | 0.06294 | -2.35658496 |
| Lrrtm4 | 0.00144 | -1.27165604 |
| 1700073E17Rik | 0.00144 | -1.26934956 |
| Mapkbp1 | 0.12861 | 0.2841328 |
| Nvl | 0.16656 | 0.23601244 |
| Nt5c | 0.00348 | -0.87535862 |
| Cox8b | 0.00145 | -1.05315335 |
| Mt1 | 0.70408 | 0.07344722 |
| Tmem106c | 0.90287 | -0.04607253 |
| Cep126 | 0.07369 | 1.40807871 |
| Fbxl7 | 0.0174 | -0.66733805 |
| Mos | 0.00144 | -1.43514939 |
| Gm5094 | 0.02075 | -1.00628634 |
| Pde4b | 0.23251 | 0.41776857 |
| Pet2 | 0.00144 | -1.24811894 |
| Acaa1b | 0.11045 | -0.31075321 |
| Arglu1 | 0.00144 | 1.02363977 |
| Olfr900 | 0.00144 | -1.26560808 |
| Hspg2 | 0.00144 | -1.21019199 |
| Fgf10 | 0.85525 | 0.0802222 |
| Zfp362 | 0.03008 | -0.46677422 |
| Klrb1a | 0.0281 | -1.05344382 |
| Pisd | 0.00144 | 1.34741155 |
| Fars2 | 0.85409 | -0.12144184 |
| Dapp1 | 0.98329 | -0.0091648 |
| Armc8 | 0.03241 | 1.70214345 |
| Ptbp2 | 0.01061 | 2.38090514 |
| Mtss1 | 0.51232 | 0.28182017 |
| Svil | 0.07184 | -0.5788502 |
| Morc4 | 0.00144 | 1.45505506 |
| Hgf | 0.07973 | -0.62153296 |
| Gbf1 | 0.00152 | -1.25878908 |
| 9930012K11Rik | 0.57404 | 0.20103456 |
| Cyp2u1 | 0.00144 | -1.29299358 |
| Vps54 | 0.0036 | 1.32226648 |
| Chmp5 | 0.56803 | -0.43332851 |
| Cyp2b19 | 0.00144 | -1.30713006 |
| Cldn23 | 0.02495 | -1.27539845 |
| LOC105247143 | 0.00145 | -1.26219879 |
| Cd164 | 0.25443 | -0.56504847 |
| Rnf24 | 0.0397 | -1.20030135 |
| Clip1 | 0.00277 | 1.43130602 |
| Paqr5 | 0.01514 | -0.95990192 |
| Dzip1 | 0.45758 | -1.06738469 |
| Lipt2 | 0.63708 | -0.19728653 |
| Rere | 0.98052 | 0.00501966 |
| Taf2 | 0.12206 | -0.34438343 |
| Prokr1 | 0.00144 | -1.46410581 |
| Uts2b | 0.00144 | -1.29864962 |
| Klhl11 | 0.20127 | -0.32131126 |
| Platr26 | 0.02241 | -1.2062323 |
| Acta2 | 0.00144 | -1.45997707 |
| Cry1 | 0.08048 | -1.0633596 |
| Hist1h2ab | 0.0044 | -1.55760851 |
| Apeh | 0.01572 | -0.76373773 |
| Slc15a1 | 0.00145 | -1.25646236 |
| Chka | 0.01792 | 1.06943941 |
| Prpf6 | 0.2939 | 0.67969994 |
| Rnps1 | 0.03638 | -0.429452 |
| Srcap | 0.01541 | -0.54306401 |
| Epha6 | 0.00144 | -1.28898079 |
| Adora2b | 0.04894 | 0.67894401 |
| Lrrc39 | 0.02928 | 0.42951615 |
| Cyhr1 | 0.15685 | -0.50815612 |
| 4933431E20Rik | 0.51717 | -0.46179934 |
| Clk2 | 0.07052 | 0.41804855 |
| Sec22b | 0.72662 | 0.06534188 |
| Pmch | 0.00144 | -1.33632959 |
| Grk4 | 0.50293 | -0.41223698 |
| Gm17296 | 0.04743 | 0.40392208 |
| Rnf220 | 0.00144 | -1.03139151 |
| Ttpal | 0.03919 | 0.61755955 |
| Plp2 | 0.36207 | 0.34195073 |
| Wrb | 0.6724 | -0.12017595 |
| 1700007G11Rik | 0.05019 | -1.03232058 |
| Prrc1 | 0.00443 | -0.87961233 |
| Tinagl1 | 0.00609 | -0.70117172 |
| Akt1 | 0.00149 | -1.34293727 |
| Fmn1 | 0.00144 | -1.36353418 |
| Zfp868 | 0.3909 | 0.30535373 |
| 3830403N18Rik | 0.36492 | -0.98634962 |
| Il1r2 | 0.0211 | -1.76914284 |
| Gid4 | 0.90909 | 0.02238133 |
| Tbkbp1 | 0.01778 | -0.56635878 |
| Tmem164 | 0.00257 | 1.2317842 |
| Olfr1170 | 0.00144 | -1.28116048 |
| Tcea1 | 0.00153 | 1.26579406 |
| Pacs1 | 0.70976 | -0.14881632 |
| Gab1 | 0.02836 | 0.89958919 |
| Dpp4 | 0.00272 | -1.09759392 |
| B3gat3 | 0.0017 | -1.24950944 |
| D17H6S56E-5 | 0.0375 | 0.61979422 |
| Fam210a | 0.1467 | -0.31008268 |
| C230075M21Rik | 0.00195 | 2.59992324 |
| Eif4g2 | 0.00174 | -0.83547979 |
| Fgd4 | 0.42925 | 0.3497132 |
| Asah2 | 0.81616 | 0.18132566 |
| Cdh23 | 0.7384 | 0.05683293 |
| Cd226 | 0.00144 | -1.26560808 |
| Ctnnal1 | 0.23983 | -0.785677 |
| Mms19 | 0.07492 | 1.27620396 |
| Casr | 0.12968 | -2.54921467 |
| Wfdc3 | 0.00497 | -1.08799931 |
| 1700049G17Rik | 0.99994 | -0.00004943 |
| Cenpu | 0.39185 | 0.31592335 |
| Atp13a2 | 0.98522 | -0.01665121 |
| Rps12 | 0.00157 | 1.5931914 |
| Sdccag8 | 0.00144 | 1.9288597 |
| Tsn | 0.95054 | -0.01986415 |
| Stkld1 | 0.01534 | -0.88702183 |
| Polq | 0.00824 | 1.39326458 |
| Nkain2 | 0.0766 | -0.84327365 |
| Wnt6 | 0.00291 | -1.1068713 |
| Trem1 | 0.00144 | -1.29085005 |
| Olfr229 | 0.62935 | 0.21996494 |
| Taf1d | 0.00478 | 1.6349621 |
| Sugt1 | 0.02958 | 0.86325442 |
| Rcan2 | 0.00144 | 1.20747895 |
| Dcun1d1 | 0.00869 | 0.81115447 |
| Tmem30b | 0.71122 | -0.37747242 |
| Cgn | 0.16496 | -1.73596428 |
| Myo10 | 0.66602 | -0.09976711 |
| Mgl2 | 0.00449 | -0.94001579 |
| Ap5m1 | 0.01428 | 1.31109881 |
| Slc35a3 | 0.00277 | 0.77723234 |
| Gm16794 | 0.10599 | -0.47558893 |
| Rad18 | 0.00149 | -1.2703832 |
| Bmp6 | 0.53195 | -0.17184438 |
| Mical3 | 0.02738 | -0.52474172 |
| Usp22 | 0.55501 | 0.09435865 |
| Nrip1 | 0.00144 | 1.01108744 |
| 4732416N19Rik | 0.0017 | -1.24950944 |
| Zcchc7 | 0.00527 | 1.74663657 |
| Ldlrap1 | 0.00953 | -0.71259647 |
| Csn1s2b | 0.00144 | -1.27165604 |
| Sln | 0.3031 | -3.22626092 |
| Ppef2 | 0.04477 | -1.04546769 |
| Gpr173 | 0.00144 | -1.28898079 |
| Igbp1b | 0.00144 | -1.27699683 |
| Ip6k1 | 0.04335 | -0.43914121 |
| Itga7 | 0.69352 | -0.26778158 |
| Map3k5 | 0.23833 | 0.20920009 |
| Daam2 | 0.66453 | -0.38735225 |
| Ear1 | 0.00989 | -1.20160653 |
| 4930451I11Rik | 0.00144 | -1.20555712 |
| Fbxo3 | 0.08 | 0.36283399 |
| Mup5 | 0.00179 | -1.61463084 |
| Nox3 | 0.00144 | -1.28116048 |
| Sacs | 0.02143 | -1.64998644 |
| Fbxl21 | 0.01023 | -0.90199263 |
| Olfr788 | 0.00149 | -1.2703832 |
| Pcgf6 | 0.00178 | 1.33704256 |
| Med13l | 0.12989 | -0.27287122 |
| Tyro3 | 0.45445 | -0.47728274 |
| Ep300 | 0.66765 | -0.07417023 |
| A830019P07Rik | 0.04814 | -0.89185502 |
| Rnf20 | 0.00144 | -1.29085005 |
| Ptprr | 0.0263 | -0.95291538 |
| Ccdc66 | 0.05488 | 0.86042192 |
| 1700028K03Rik | 0.05892 | -2.395022 |
| Npc1 | 0.03132 | -1.7905777 |
| Sema7a | 0.08763 | -0.42288944 |
| 2900079G21Rik | 0.00546 | -1.27102286 |
| Krt7 | 0.18269 | -0.59661821 |
| Trp73 | 0.04247 | -0.64179814 |
| Rab31 | 0.01104 | -0.90534105 |
| Farsa | 0.02381 | -0.54224751 |
| 2310057J18Rik | 0.00172 | -1.3924275 |
| Gm5885 | 0.03584 | -0.96852355 |
| Olfr90 | 0.00144 | -1.28116048 |
| 4933427D14Rik | 0.00747 | 0.94954519 |
| Slc2a2 | 0.00519 | -1.81053513 |
| Ccnt2 | 0.01333 | 0.7966752 |
| Plat | 0.28922 | 0.210826 |
| Wdfy3 | 0.00145 | -1.02779823 |
| Serpinc1 | 0.49919 | -0.25455349 |
| Scamp5 | 0.37856 | -0.23464232 |
| Sec24a | 0.24375 | -0.27256798 |
| Wdr11 | 0.11041 | 1.1542427 |
| Cd40 | 0.09739 | -0.3476948 |
| Pdzd2 | 0.00144 | 1.19442221 |
| 5330426P16Rik | 0.01615 | -0.53163852 |
| Zbtb7c | 0.01797 | -0.50992874 |
| Auh | 0.97584 | 0.01608506 |
| Tuba4a | 0.9501 | 0.03265443 |
| Gnptab | 0.33069 | 0.16404967 |
| Brd1 | 0.53256 | 0.10277968 |
| 9530083O12Rik | 0.00384 | -1.00787433 |
| Fcgr3 | 0.32376 | 0.31993059 |
| Slc16a7 | 0.00149 | -1.22582153 |
| Sft2d2 | 0.33991 | -0.4235996 |
| Zfp157 | 0.00269 | -1.27443216 |
| Huwe1 | 0.01239 | -1.07107107 |
| Pdgfc | 0.00144 | -1.28898079 |
| Tbl2 | 0.1474 | 0.60543753 |
| St3gal2 | 0.00529 | -0.76985987 |
| Jazf1 | 0.0059 | 0.81831157 |
| Rab11fip4 | 0.35952 | -0.69538555 |
| Fmo1 | 0.60092 | 0.18636811 |
| Kcnh7 | 0.00176 | -1.24266807 |
| 9330174C13Rik | 0.05225 | 1.02390546 |
| Arhgef6 | 0.0742 | 1.06783169 |
| Rab8b | 0.64197 | 0.15875842 |
| Mpped2 | 0.42893 | -0.15903455 |
| Nfx1 | 0.23557 | 0.19114599 |
| 4930402H24Rik | 0.03273 | 0.98293226 |
| St3gal6 | 0.01258 | 0.71414813 |
| 5830410O09Rik | 0.11023 | -0.57674948 |
| Foxk2 | 0.54172 | 0.18621668 |
| Lsm12 | 0.01856 | -0.59690828 |
| Slc29a3 | 0.35789 | -0.49674974 |
| Mdh1 | 0.75798 | -0.08951474 |
| Gpcpd1 | 0.72656 | -0.08422885 |
| Pom121 | 0.08805 | -0.39261394 |
| Irx3os | 0.66496 | 0.10115557 |
| H2afj | 0.00144 | -1.39623165 |
| Pald1 | 0.45569 | -0.24684114 |
| Adamtsl5 | 0.12453 | -0.54089824 |
| Cct8l1 | 0.81159 | 0.070189 |
| Hoxd10 | 0.00144 | -1.31884045 |
| Sap130 | 0.17879 | 1.209092 |
| P2ry4 | 0.00144 | -1.28116048 |
| Abi3bp | 0.00735 | -1.65517008 |
| 4632415L05Rik | 0.04714 | -0.50368864 |
| Sepsecs | 0.77527 | 0.05945837 |
| Fut9 | 0.08415 | -0.92963551 |
| Dkc1 | 0.00144 | 2.37890482 |
| Mastl | 0.75602 | -0.13284979 |
| Mus81 | 0.26558 | -0.44395326 |
| Zfp106 | 0.1283 | -0.50879012 |
| Mbtps1 | 0.02156 | -0.69606828 |
| Med14 | 0.01338 | 0.68577645 |
| Spdyb | 0.00461 | -1.56114443 |
| Adamts20 | 0.10335 | -0.71479664 |
| Nacad | 0.00811 | -0.79142683 |
| Rbm41 | 0.00144 | -1.31495282 |
| Pbx3 | 0.00144 | -1.27165604 |
| Orai1 | 0.0764 | -0.69170387 |
| Rpl24 | 0.01561 | -0.65593841 |
| Zfp938 | 0.00144 | 2.52938225 |
| Ntm | 0.00144 | -1.27730451 |
| Zfp950 | 0.00172 | 2.2968241 |
| Plat | 0.16174 | -0.52832083 |
| Dcc | 0.27509 | -0.68230702 |
| 1700066J03Rik | 0.04302 | -0.82322183 |
| Vipr2 | 0.01818 | -1.08200933 |
| Piga | 0.08787 | -0.68755772 |
| Pm20d1 | 0.00144 | -1.66233002 |
| Pak1ip1 | 0.06483 | 0.9581235 |
| Tm7sf3 | 0.00627 | -0.71661023 |
| C1qtnf6 | 0.00521 | -0.77179271 |
| Flt1 | 0.01031 | 1.71277809 |
| Polr3g | 0.00279 | -1.12046735 |
| LOC102640779 | 0.43719 | -0.31591185 |
| Rps19 | 0.00144 | -1.21569918 |
| Map3k7cl | 0.00144 | -1.28116048 |
| Tmem62 | 0.0136 | 0.80295458 |
| Baz2a | 0.91913 | -0.017006 |
| Pcdhac2 | 0.00953 | -1.53400316 |
| Adgrv1 | 0.00144 | -1.28116048 |
| Tmem60 | 0.89191 | -0.03438723 |
| Cdc42bpb | 0.65012 | -0.07302565 |
| Saa4 | 0.09102 | -1.49519804 |
| Olfml2a | 0.095 | -0.93195309 |
| Olfr711 | 0.00144 | -1.3348153 |
| Slc24a1 | 0.00144 | -1.29085005 |
| Gnb2l1 | 0.05314 | -0.6887177 |
| Papola | 0.00217 | 1.13916487 |
| St6gal2 | 0.00513 | -0.68145203 |
| 4930402H24Rik | 0.21142 | -0.67995981 |
| Grb10 | 0.63479 | -0.24039821 |
| Ppp1r14d | 0.01046 | -1.57548443 |
| Tns3 | 0.05478 | 0.44026793 |
| Atp6v1h | 0.47364 | 0.27124661 |
| Aqp7 | 0.00176 | -1.34117581 |
| Ihh | 0.00492 | -1.13895867 |
| Svep1 | 0.11253 | -2.78213095 |
| 0610005C13Rik | 0.34346 | 0.28451881 |
| Myoz2 | 0.01463 | 1.48216184 |
| Wnt2b | 0.00168 | -1.4256134 |
| Sorbs2 | 0.00219 | 0.94502739 |
| Xrn1 | 0.00367 | 0.72040801 |
| 5730460C07Rik | 0.00144 | -1.27165604 |
| Ptpn4 | 0.00144 | 1.56432038 |
| Hgd | 0.00145 | -1.35266853 |
| Bbx | 0.06845 | 0.33405349 |
| Cdh4 | 0.32682 | 0.57849864 |
| Inpp1 | 0.3873 | 0.26276394 |
| Mpp5 | 0.00144 | 2.16578825 |
| Prom1 | 0.05799 | -0.82786183 |
| Kcnj11 | 0.4107 | -0.30483205 |
| Ninl | 0.91505 | 0.04614612 |
| Cd59a | 0.84112 | -0.04623406 |
| Tmem121 | 0.00298 | -1.14915096 |
| Med20 | 0.44213 | 0.13282033 |
| Mup11 | 0.00144 | -1.28116048 |
| Bpgm | 0.95594 | -0.01258329 |
| D930048N14Rik | 0.14946 | 0.44992017 |
| Tnpo2 | 0.11951 | -0.57524167 |
| Cd209d | 0.00144 | -1.30017714 |
| Thbs4 | 0.00291 | -1.03920125 |
| Inha | 0.04012 | 0.52363746 |
| Hnrnpk | 0.00191 | -1.05039785 |
| Arhgap31 | 0.01411 | 0.73459802 |
| Adamts13 | 0.01039 | -1.54007366 |
| AW209491 | 0.02404 | 0.47585464 |
| Appbp2os | 0.15457 | -0.93122786 |
| Atm | 0.01099 | 1.34392184 |
| Pirt | 0.40738 | -0.28216326 |
| Strip1 | 0.00926 | -1.25888604 |
| Ccs | 0.00724 | -0.656539 |
| Atp6v0a1 | 0.00144 | -1.20686706 |
| B4galt2 | 0.00145 | -0.94514684 |
| Dusp2 | 0.00596 | -0.90978649 |
| Pde3a | 0.00605 | -1.08150631 |
| Tprkb | 0.2236 | 0.45070164 |
| Rmnd1 | 0.00988 | 1.21627461 |
| Ckap2l | 0.84667 | -0.15763084 |
| 1700025O08Rik | 0.00149 | -1.22582153 |
| Rffl | 0.40942 | -0.17614329 |
| Tmem138 | 0.04737 | -1.12757267 |
| Mettl24 | 0.11929 | -0.52503686 |
| Ankrd34b | 0.00144 | -1.25118919 |
| Tmed2 | 0.07715 | -0.31038426 |
| Gfra1 | 0.11166 | -0.92118393 |
| Cytip | 0.00235 | 1.08972766 |
| Pde6g | 0.44606 | -0.19728757 |
| Pde4d | 0.18342 | -0.71065238 |
| Stfa2l1 | 0.00144 | -1.29085005 |
| Cep57l1 | 0.02708 | -0.48340965 |
| A830080D01Rik | 0.01532 | 1.55830832 |
| Nudt5 | 0.5787 | 0.12402865 |
| Ccdc157 | 0.72653 | 0.11968479 |
| Irx2 | 0.87986 | -0.07910716 |
| Uggt2 | 0.06355 | 0.71509805 |
| Map3k15 | 0.01408 | -0.76395243 |
| 2810403A07Rik | 0.01147 | 1.26577296 |
| Golga7 | 0.08315 | 0.76863787 |
| Gm3740 | 0.28131 | -0.48372133 |
| Pced1a | 0.23795 | 0.23207954 |
| Atp2c1 | 0.64321 | -0.13501451 |
| Rap1gap2 | 0.99391 | 0.0031932 |
| 5330416C01Rik | 0.00144 | -1.27165604 |
| Dbf4 | 0.00144 | -1.28116048 |
| Adgrg3 | 0.73225 | -0.13801113 |
| Smad2 | 0.04918 | 0.40537472 |
| Csk | 0.00147 | -0.98963416 |
| 4930448K20Rik | 0.00144 | -1.26934956 |
| Eml5 | 0.00655 | 2.36669161 |
| 2700094K13Rik | 0.03574 | -0.72128306 |
| Itsn1 | 0.06129 | -0.45595788 |
| Ric1 | 0.00145 | -1.22376446 |
| Cdh17 | 0.00673 | -1.46869873 |
| Tjp2 | 0.00861 | -1.01826866 |
| Ddx3x | 0.0054 | -0.89681245 |
| Zfp282 | 0.11882 | 0.66665949 |
| 4930412M03Rik | 0.11439 | -0.72318987 |
| Sf1 | 0.00381 | -1.84318572 |
| Lrrc55 | 0.17425 | 0.83914111 |
| Map2k6 | 0.28017 | -0.56403712 |
| Mbd6 | 0.02253 | -0.44881727 |
| Nipbl | 0.00735 | 2.21516362 |
| Ythdf1 | 0.11421 | -0.38776073 |
| Usp45 | 0.09427 | -0.31502631 |
| Txnrd2 | 0.00257 | -1.23520454 |
| Fam228a | 0.00291 | -1.01517155 |
| 1700016K19Rik | 0.00228 | -1.03165578 |
| Napg | 0.57542 | 0.28843596 |
| Abcd3 | 0.0553 | 0.37856608 |
| 1600012H06Rik | 0.0542 | 0.40807057 |
| Bcat2 | 0.44051 | -0.16495443 |
| Lypd4 | 0.01806 | -1.67895579 |
| Wdr41 | 0.00144 | -1.27165604 |
| Sez6 | 0.4598 | 0.27913517 |
| Clmp | 0.4131 | 0.22969171 |
| Chrm1 | 0.00144 | -1.31592062 |
| Olfr137 | 0.00461 | -1.50785399 |
| P2rx7 | 0.15454 | -0.29183225 |
| Snap23 | 0.00942 | -0.90317961 |
| Zscan12 | 0.00628 | -1.22992689 |
| Lyrm9 | 0.04115 | 0.94331372 |
| Qtrtd1 | 0.00144 | 2.48396477 |
| Acot9 | 0.45449 | 0.13460226 |
| Zfp641 | 0.59467 | 0.10249646 |
| Bmp2k | 0.20363 | 0.61434804 |
| Gin1 | 0.00948 | 0.68219007 |
| Hbp1 | 0.77918 | -0.0453212 |
| Bzw2 | 0.24457 | 0.68759107 |
| Rnd3 | 0.22227 | -0.33433893 |
| Ints10 | 0.01022 | 0.6430035 |
| Gm17634 | 0.00291 | -1.99443988 |
| Dab1 | 0.00144 | -1.26934956 |
| Ylpm1 | 0.00276 | 0.93399276 |
| Nell1 | 0.00144 | -1.27165604 |
| 1700094J05Rik | 0.00144 | -1.28116048 |
| Thoc7 | 0.00485 | -0.77855837 |
| Trnp1 | 0.06359 | -0.46626915 |
| Terf2 | 0.01568 | -0.50875986 |
| Ryr1 | 0.0489 | -1.08448857 |
| Hinfp | 0.95353 | -0.01137025 |
| Ppp1r12c | 0.02337 | -0.53312846 |
| Zfp952 | 0.00941 | 0.70842982 |
| LOC102639950 | 0.00145 | -1.26219879 |
| Jade3 | 0.00144 | -1.63862646 |
| Chst3 | 0.01114 | 0.58176281 |
| Trip4 | 0.30902 | 0.18349196 |
| Ccdc96 | 0.00144 | -1.28116048 |
| Olfr740 | 0.00358 | -2.32826344 |
| Golga3 | 0.24835 | -0.3682095 |
| Gal3st2 | 0.37827 | -0.43462046 |
| Arhgef12 | 0.00618 | -0.65863305 |
| Eif4b | 0.01143 | -0.63002181 |
| Tdrd5 | 0.00144 | -1.31884045 |
| Lrch1 | 0.02331 | 0.44563656 |
| E330024J20Rik | 0.97751 | -0.02194882 |
| Socs6 | 0.00474 | -1.10247618 |
| Glra3 | 0.00144 | -1.30713006 |
| Sap25 | 0.31121 | 0.29514693 |
| Pag1 | 0.93961 | -0.06490726 |
| Sox2ot | 0.00144 | -1.29746368 |
| Tcaf1 | 0.01764 | 0.88427077 |
| Tmem132b | 0.01166 | -1.92074667 |
| Pom121l12 | 0.05699 | -0.99863187 |
| Zfyve9 | 0.00975 | 0.68766615 |
| Olfr890 | 0.00144 | -1.24811894 |
| Olfr608 | 0.09936 | -0.50670379 |
| Mfsd6 | 0.00235 | 1.2381489 |
| Tlk1 | 0.32771 | -0.25520634 |
| Dctn5 | 0.30701 | -0.17000838 |
| Col6a5 | 0.00149 | -1.22582153 |
| Fbll1 | 0.43458 | 0.55955763 |
| Tcf7l1 | 0.01139 | -0.62672586 |
| C87977 | 0.00144 | -1.27699683 |
| Mup6 | 0.00144 | -1.30713006 |
| Slirp | 0.13438 | 0.73531965 |
| Dcc | 0.00144 | -1.33632959 |
| Lyrm7 | 0.03891 | 0.62825923 |
| Zfp324 | 0.08662 | -0.83903351 |
| Creg2 | 0.00561 | -1.57351568 |
| Egln3 | 0.53293 | 0.28506394 |
| Birc6 | 0.0244 | 0.93118304 |
| Trmu | 0.03205 | -0.54724523 |
| Pcgf3 | 0.00144 | -1.53929237 |
| Bmp15 | 0.00706 | -0.8216566 |
| Irak3 | 0.03587 | 0.73089462 |
| Gpa33 | 0.00596 | -1.15942552 |
| Ccdc12 | 0.01549 | -0.59515409 |
| Fermt3 | 0.34768 | -0.20642769 |
| Vmn1r196 | 0.00145 | -1.22376446 |
| Efcab13 | 0.00144 | -1.31495282 |
| Gm15879 | 0.00165 | 0.88455911 |
| Spock2 | 0.87263 | -0.04690747 |
| Pantr1 | 0.00144 | -1.28116048 |
| Galntl6 | 0.00145 | -1.2388121 |
| Rnpc3 | 0.00144 | 2.13769915 |
| Ckb | 0.12085 | -0.51329142 |
| Tal2 | 0.32774 | -0.85974962 |
| Plekha3 | 0.00382 | 0.76987656 |
| Hoxd9 | 0.00144 | -1.31495282 |
| C1ql3 | 0.00164 | -1.77268705 |
| Olfr826 | 0.00144 | -1.29085005 |
| Ppp1r3a | 0.00144 | 2.29233215 |
| Rprd2 | 0.01295 | -0.92080079 |
| Olfr537-ps1 | 0.07736 | -0.87130337 |
| Angel2 | 0.00256 | -0.87926016 |
| Harbi1 | 0.04213 | 0.61107846 |
| Bin1 | 0.00385 | -0.82030838 |
| Cnot10 | 0.33548 | -0.16920532 |
| Slc25a21 | 0.00144 | -1.45078974 |
| Cdh10 | 0.73352 | -0.28749883 |
| Tex11 | 0.00177 | -1.15847607 |
| Trim2 | 0.56735 | -0.20475936 |
| Stard4 | 0.7781 | 0.10702805 |
| Mib2 | 0.00342 | -0.7395522 |
| Rnf217 | 0.41148 | 0.22503637 |
| Lrfn5 | 0.0049 | -1.11996532 |
| Tsc22d4 | 0.00144 | -1.82595473 |
| Tmem198b | 0.13622 | -0.25796756 |
| LOC102642673 | 0.0147 | -0.60238015 |
| Yme1l1 | 0.52181 | 0.78450831 |
| Mysm1 | 0.00613 | 1.95791294 |
| Anapc7 | 0.00144 | -1.26560808 |
| Rxfp2 | 0.15025 | -0.75984084 |
| Gpcpd1 | 0.57784 | 0.1269092 |
| Gm11732 | 0.00144 | -1.44780261 |
| Agbl2 | 0.02329 | -1.47447468 |
| Rb1cc1 | 0.82734 | 0.04314628 |
| Gsg1 | 0.05465 | -0.48778455 |
| 4930445N18Rik | 0.00144 | -1.24900642 |
| Copg2os2 | 0.00144 | 3.23767096 |
| Cpne8 | 0.042 | -0.4091524 |
| Sp6 | 0.49195 | -0.22643324 |
| Mylpf | 0.55679 | 0.11215082 |
| Naa20 | 0.0078 | 1.22125455 |
| Rnd1 | 0.06056 | -0.59644734 |
| Cd4 | 0.90756 | 0.06159974 |
| Ell3 | 0.43539 | -0.23153021 |
| Trip6 | 0.01911 | -0.62467003 |
| Gjb2 | 0.00144 | -1.20555712 |
| Bloc1s3 | 0.00513 | -1.17740882 |
| 1700102P08Rik | 0.34812 | -0.45495978 |
| Ddb2 | 0.00144 | 1.26194417 |
| Cyp26a1 | 0.11524 | -0.6628766 |
| Arpp21 | 0.11231 | 0.44151627 |
| 4930579D07Rik | 0.08985 | -2.51212126 |
| Gpr55 | 0.00144 | -1.29746368 |
| Irf2bp1 | 0.03374 | -0.75900568 |
| Rasa3 | 0.13149 | 0.42329204 |
| Neto2 | 0.02976 | -1.43735262 |
| Aff1 | 0.70043 | 0.07251184 |
| Nbea | 0.28449 | -0.54499755 |
| Robo1 | 0.84516 | 0.16967122 |
| Btbd17 | 0.45613 | -0.49514228 |
| Spon2 | 0.00296 | -1.29473258 |
| Nxph1 | 0.00144 | -1.33632959 |
| Mcph1 | 0.03382 | -1.20678928 |
| Tbc1d4 | 0.03448 | -0.49845611 |
| Zfp207 | 0.00918 | 1.17003212 |
| Gins2 | 0.09349 | -0.45327507 |
| A530016L24Rik | 0.08634 | -0.62922867 |
| Nfe2l3 | 0.00145 | -1.50995226 |
| Spaca5 | 0.00144 | -1.33632959 |
| 4930573C08Rik | 0.14554 | -1.2593759 |
| Prkrir | 0.00144 | -1.14871674 |
| Ap2b1 | 0.62808 | 0.16595346 |
| Zfp748 | 0.01569 | 1.7390894 |
| Srbd1 | 0.00144 | 1.64854707 |
| Phf11d | 0.00465 | 1.19482291 |
| Gm5082 | 0.01402 | -1.57569845 |
| Papd7 | 0.4316 | 0.18985662 |
| Unc5c | 0.08886 | -0.82929092 |
| Podxl | 0.50196 | -0.13122209 |
| Gtpbp6 | 0.04272 | -0.59463765 |
| Arl4c | 0.30128 | 0.2130409 |
| Adam6a | 0.01913 | -2.90379843 |
| Tardbp | 0.01751 | -1.00916556 |
| Homer1 | 0.00289 | 2.00349558 |
| Exoc5 | 0.00144 | 2.15742574 |
| Tm9sf1 | 0.3081 | -0.21638787 |
| Timeless | 0.00271 | -1.18408685 |
| Gm5334 | 0.00144 | -1.29864962 |
| Rtn4 | 0.01281 | 0.6704439 |
| Fktn | 0.00144 | 2.13939597 |
| Enpp3 | 0.21735 | -0.30330829 |
| Hip1 | 0.80202 | 0.08698061 |
| Slc1a3 | 0.55609 | 0.19435474 |
| Tmem232 | 0.11532 | -0.88511728 |
| Gfm2 | 0.77005 | -0.21367431 |
| AU040320 | 0.0015 | -1.28965523 |
| A130082J08Rik | 0.27768 | 0.28465622 |
| Lpcat2b | 0.00658 | 1.10371795 |
| Ankrd28 | 0.01635 | 0.58225038 |
| Primpol | 0.37456 | 0.32812312 |
| Col12a1 | 0.00144 | -1.22925777 |
| Slco5a1 | 0.00144 | -1.26428512 |
| Slc9b1 | 0.00144 | -1.24811894 |
| Rtca | 0.08 | 0.57274868 |
| Cdc42se2 | 0.05048 | -0.57750511 |
| Lca5 | 0.0021 | 1.72132557 |
| Pkhd1 | 0.00144 | -1.26560808 |
| 9530004P13Rik | 0.00338 | 1.34389577 |
| Peg10 | 0.08919 | -1.42521148 |
| Fsd1 | 0.00144 | -1.29085005 |
| Ap1s3 | 0.03566 | -0.95635284 |
| Eps8l1 | 0.68269 | 0.26887993 |
| Gabra1 | 0.46044 | 0.42519082 |
| Olfr1122 | 0.00601 | -1.09465341 |
| Olfr1477 | 0.00144 | -1.28116048 |
| Disp1 | 0.26981 | -0.20979906 |
| Zbtb11 | 0.90591 | 0.02389678 |
| Galnt6 | 0.00571 | -1.22679402 |
| Slc16a10 | 0.64735 | -0.2319373 |
| Fkbp9 | 0.8239 | -0.03730725 |
| Fgf2 | 0.69673 | -0.1300663 |
| Clec16a | 0.00201 | -1.11996532 |
| Aatf | 0.18037 | -0.23481188 |
| Pcsk6 | 0.04578 | -0.56248242 |
| Orc4 | 0.0015 | 1.36035963 |
| Spag16 | 0.00144 | -1.26934956 |
| Kcnh7 | 0.00144 | -1.29746368 |
| Sdhaf3 | 0.03685 | 0.79489653 |
| Eea1 | 0.98166 | -0.02758399 |
| Cstf3 | 0.06297 | 0.34967414 |
| Lypd6 | 0.33957 | 0.44740469 |
| Slc30a9 | 0.00983 | 1.28275913 |
| E130114P18Rik | 0.00144 | -1.28898079 |
| Rims2 | 0.13863 | -0.93505273 |
| Pex11g | 0.7127 | -0.10380715 |
| Arf3 | 0.01774 | -0.48880452 |
| Srp9 | 0.92523 | 0.02731937 |
| 4930538E20Rik | 0.00144 | -1.26560808 |
| Ubn1 | 0.02612 | -0.4526822 |
| Catsperg2 | 0.84444 | -0.11465147 |
| Tmem183a | 0.91437 | 0.02311715 |
| Gtpbp1 | 0.29396 | -0.34819165 |
| 4933403O08Rik | 0.00144 | -1.27165604 |
| Lrrc46 | 0.19413 | -0.5801954 |
| Tfdp1 | 0.03949 | 0.49777501 |
| Prkce | 0.23657 | -0.29336937 |
| Maats1 | 0.00144 | -1.29085005 |
| Amica1 | 0.3939 | -0.25817679 |
| Scamp5 | 0.01543 | -1.00177573 |
| Hyou1 | 0.96289 | -0.02123452 |
| Ptk6 | 0.06801 | -0.79743914 |
| Zfp445 | 0.00351 | 1.4956796 |
| 9330175E14Rik | 0.32312 | -0.46898807 |
| Dpcd | 0.23254 | -0.37282551 |
| Mob3a | 0.66176 | -0.11244748 |
| Ostn | 0.02144 | -1.96359031 |
| Magea3 | 0.00871 | -1.85199448 |
| Arg2 | 0.00734 | -1.08892885 |
| Atp6v1g2 | 0.29687 | 0.32377375 |
| Dusp16 | 0.00195 | 2.89996612 |
| Olfr1305 | 0.18624 | -0.65729647 |
| Gm40040 | 0.00453 | -1.16434667 |
| Csmd2 | 0.03418 | -0.9383227 |
| Jakmip3 | 0.00743 | -1.229352 |
| Mex3c | 0.00144 | 1.60678257 |
| Zcchc2 | 0.01348 | 0.59067663 |
| Gpd2 | 0.00224 | 1.38031691 |
| Acvr1b | 0.68612 | -0.09960819 |
| Pcgf3 | 0.2183 | 0.55317976 |
| Zdhhc3 | 0.49883 | -0.14913715 |
| Mipep | 0.00145 | -1.22376446 |
| Jph4 | 0.03418 | -0.9383227 |
| Zfp174 | 0.01119 | 0.98822668 |
| A530058N18Rik | 0.00144 | -1.28116048 |
| Clasp2 | 0.03318 | 0.72540568 |
| Atp11a | 0.00144 | 1.32208534 |
| Scaper | 0.00383 | 1.53391076 |
| D730048I06Rik | 0.00144 | -1.29085005 |
| Zfp142 | 0.03004 | -0.85145985 |
| Cpvl | 0.00144 | -1.25118919 |
| Rhox11 | 0.00144 | -1.30713006 |
| Ctnnal1 | 0.04091 | 0.55178873 |
| Olfr1145 | 0.03258 | -1.32085312 |
| Zfp9 | 0.0075 | 0.84309791 |
| Eif6 | 0.0308 | -1.2259802 |
| Tgs1 | 0.67627 | 0.07389396 |
| Ppil3 | 0.96807 | 0.00739811 |
| Sbf2 | 0.19169 | -0.26837607 |
| Nrp1 | 0.00421 | 0.7319599 |
| Pard6b | 0.03954 | 0.81587928 |
| Sgms1 | 0.03944 | 0.47444983 |
| C130050O18Rik | 0.07378 | -0.53913857 |
| Tmc3 | 0.00144 | -1.29051721 |
| Cdc6 | 0.87536 | -0.11850612 |
| Gpr107 | 0.14145 | -0.40685908 |
| Iqcc | 0.02069 | -0.57395088 |
| Mthfr | 0.02824 | -1.06152012 |
| Bend7 | 0.86774 | 0.05768197 |
| Glcci1 | 0.00768 | 0.77613523 |
| Magi1 | 0.04462 | 0.85799191 |
| 1700084E18Rik | 0.8937 | -0.05993367 |
| Meaf6 | 0.24528 | -0.59501807 |
| Nop56 | 0.08019 | 0.71061836 |
| Uqcrq | 0.00144 | -1.13744055 |
| Msl1 | 0.0836 | -0.39163827 |
| Urb1 | 0.03016 | -0.79343043 |
| Riok3 | 0.00332 | 0.95260415 |
| Kcmf1 | 0.76609 | 0.05417691 |
| Mapk8ip2 | 0.00487 | -0.98492355 |
| Plbd2 | 0.00356 | -0.90869088 |
| 4933417A18Rik | 0.01753 | -0.93511097 |
| Crisp1 | 0.01239 | -1.02834537 |
| Jarid2 | 0.10308 | -0.39828168 |
| Vav3 | 0.94762 | 0.05814683 |
| Gm36581 | 0.31373 | -0.51144548 |
| G2e3 | 0.51692 | 0.31262622 |
| Otc | 0.00144 | -1.30713006 |
| Nrg1 | 0.1933 | -0.85988021 |
| Trim45 | 0.00144 | -1.75016984 |
| Olfr1279 | 0.00152 | -1.25878908 |
| Rcan2 | 0.00144 | -1.07014531 |
| Morn2 | 0.62477 | -0.1373677 |
| Fam189a2 | 0.46402 | -0.36617256 |
| Trpm5 | 0.1647 | -1.54445913 |
| Skap2 | 0.00793 | 1.0414463 |
| Kiz | 0.00514 | 0.82764971 |
| Nfu1 | 0.17904 | -0.3338584 |
| Spry2 | 0.41586 | -0.16109583 |
| Mid2 | 0.00558 | 0.76442489 |
| Zfp354a | 0.2587 | 0.42685995 |
| Clk4 | 0.20186 | 0.29527242 |
| Rap2b | 0.28011 | -0.26123378 |
| Sp3 | 0.12924 | -2.78843727 |
| Tmem100 | 0.00388 | 1.56850968 |
| Bcl7c | 0.24861 | -0.19252855 |
| Cbll1 | 0.0095 | 0.58626909 |
| Pus7l | 0.591 | -0.32712811 |
| Slc7a11 | 0.00144 | -1.33632959 |
| Cpsf6 | 0.70056 | -0.62420899 |
| Msh3 | 0.01376 | -0.68169203 |
| Zfp280c | 0.00144 | 1.54012261 |
| Mri1 | 0.02323 | -0.60018345 |
| Zfp821 | 0.02983 | -0.44271206 |
| Calml4 | 0.98935 | 0.00300625 |
| Sdhaf2 | 0.0732 | 1.07035141 |
| Acin1 | 0.02173 | -0.53999106 |
| Nexn | 0.00329 | 1.05811337 |
| A430072C10Rik | 0.00144 | -1.31884045 |
| Pola2 | 0.05576 | -0.98757276 |
| Fsd1l | 0.03733 | 0.76950612 |
| Dgkeos | 0.81599 | -0.11214113 |
| Atxn2l | 0.73629 | -0.0588894 |
| Wrap73 | 0.01363 | -0.81140335 |
| Trim34a | 0.77205 | 0.09481447 |
| Crem | 0.47592 | 0.4045797 |
| Grin2b | 0.0034 | -1.39480819 |
| A730020M07Rik | 0.00144 | -1.29864962 |
| Gna11 | 0.03019 | -0.60284464 |
| Cyp3a44 | 0.00144 | -1.29049509 |
| Cipc | 0.71026 | -0.11344586 |
| Drd1 | 0.05331 | -1.83203424 |
| Erp27 | 0.00317 | -1.11935153 |
| Pyy | 0.05401 | -1.10237566 |
| Zfp111 | 0.86772 | 0.03805195 |
| Cul4a | 0.69491 | -0.06428701 |
| Lrpprc | 0.75879 | 0.04934994 |
| Tmem55b | 0.51574 | -0.21175204 |
| Hdlbp | 0.00455 | -0.6671169 |
| Etl4 | 0.00144 | -1.29746368 |
| Nsun2 | 0.10926 | 0.33075185 |
| Cdc37l1 | 0.02802 | 0.88073794 |
| Senp6 | 0.44817 | 0.1602839 |
| Lrrc4c | 0.26918 | 0.47287571 |
| Tmem91 | 0.157 | -0.86272738 |
| Ralyl | 0.00145 | -1.2388121 |
| Plcb2 | 0.00957 | -1.03989694 |
| Lrrn2 | 0.00715 | -0.82668813 |
| Poc5 | 0.12561 | -1.38554686 |
| 4930583K01Rik | 0.00144 | -1.28116048 |
| Fes | 0.02032 | -0.5719276 |
| Zbtb22 | 0.02931 | -0.66365967 |
| 1700095B10Rik | 0.00144 | -1.30017714 |
| Bnip2 | 0.06261 | -0.3459555 |
| 2210015D19Rik | 0.1536 | -0.45397196 |
| Mcm3 | 0.48232 | -0.17137868 |
| Trp53bp1 | 0.24979 | -0.29297599 |
| Adgre1 | 0.65256 | 0.09160318 |
| Gabra3 | 0.38217 | -0.3662919 |
| Pik3c2a | 0.50685 | 0.31469405 |
| Itga5 | 0.36324 | -0.27845598 |
| Map3k14 | 0.05776 | -0.3848105 |
| Zscan4c | 0.00144 | -1.28898079 |
| Ybx1 | 0.16179 | -0.33007055 |
| Lrrc37a | 0.00144 | -1.30017714 |
| Tarsl2 | 0.00759 | -0.78363488 |
| Ccdc110 | 0.07621 | -2.4893241 |
| Gcnt2 | 0.08571 | -0.55113665 |
| Stim1 | 0.00409 | -1.08010478 |
| Nmnat2 | 0.00144 | -1.20555712 |
| Pmm2 | 0.00714 | -0.77375965 |
| Ncaph | 0.18934 | 0.83990105 |
| D830050J10Rik | 0.01196 | -0.8303873 |
| Pfkfb3 | 0.49879 | -0.36784549 |
| Zmym4 | 0.44092 | -0.27705235 |
| Fat3 | 0.55593 | -0.33585245 |
| Setx | 0.01247 | 1.14528059 |
| Col6a2 | 0.03249 | -0.72180479 |
| Nrxn1 | 0.01105 | -1.096785 |
| Dppa5a | 0.00359 | -0.79701653 |
| Upf2 | 0.00144 | 2.19481615 |
| Fam3c | 0.05993 | 0.37819364 |
| Vmn1r184 | 0.02961 | -0.94782714 |
| Polr2e | 0.37547 | -0.38903048 |
| Copg2 | 0.7065 | -0.08012488 |
| Zc2hc1a | 0.0229 | 0.47685517 |
| Vti1a | 0.08145 | -0.41301879 |
| Hspa14 | 0.02986 | 0.46595929 |
| Zfp804a | 0.00144 | -1.30713006 |
| Olfr1153 | 0.07842 | 0.5650607 |
| Rab10os | 0.2535 | 0.4210804 |
| Kbtbd12 | 0.09937 | -0.34107932 |
| Foxm1 | 0.00144 | -1.28898079 |
| Rbm5 | 0.08634 | -0.41340401 |
| Lrrc43 | 0.05995 | -0.8689511 |
| Kcp | 0.00145 | -0.89842015 |
| Celsr2 | 0.13398 | -0.28511847 |
| Rbm6 | 0.00751 | -1.02966834 |
| Zscan18 | 0.95641 | -0.04187506 |
| Map1b | 0.06527 | 0.46831901 |
| Osbpl3 | 0.01272 | 2.39330144 |
| Cacna1e | 0.12737 | -0.7976704 |
| Clip2 | 0.10529 | -0.38121502 |
| E330013P08Rik | 0.00463 | 2.18721262 |
| Raph1 | 0.70648 | 0.14824306 |
| Osbpl6 | 0.11397 | 0.39874547 |
| Ntf5 | 0.86609 | 0.05270283 |
| 4930447J18Rik | 0.00145 | -1.22376446 |
| Tmem132cos | 0.03188 | -1.73067292 |
| Luzp1 | 0.07376 | 0.58004255 |
| Dohh | 0.39558 | -0.17312645 |
| Zfp354b | 0.0035 | 1.74815528 |
| Pdxk | 0.01487 | -0.67882365 |
| Sybu | 0.02568 | -1.07143971 |
| Ptk2 | 0.00813 | 0.67583469 |
| 5830408C22Rik | 0.40224 | 0.30821051 |
| Fam214b | 0.52799 | -0.18047633 |
| Slc25a25 | 0.52055 | -0.26472868 |
| 4930599N23Rik | 0.83672 | 0.07166239 |
| Hmg20b | 0.00144 | -1.71105428 |
| Gm5114 | 0.00144 | -1.27165604 |
| D830024N08Rik | 0.37949 | -0.32684804 |
| Olfr266 | 0.00291 | -1.31319825 |
| Filip1 | 0.1325 | -0.29935891 |
| Ppp4r2 | 0.03197 | 0.42002613 |
| Mtdh | 0.0075 | 0.63363689 |
| Zfp326 | 0.56845 | 0.23424714 |
| Sod1 | 0.26898 | -0.26448539 |
| Fbxo10 | 0.05764 | -0.46285451 |
| Ppp2ca | 0.00704 | 0.99921257 |
| Ms4a12 | 0.00144 | -1.28898079 |
| Rad51ap2 | 0.00144 | -1.27454685 |
| Acsbg2 | 0.00144 | -1.31884045 |
| Gli2 | 0.19285 | -0.53437396 |
| Dync2h1 | 0.16906 | 1.02877037 |
| Neu3 | 0.0096 | 0.57204322 |
| Herc1 | 0.00144 | -1.29746368 |
| Nup155 | 0.02051 | 1.0669595 |
| Fgd4 | 0.52398 | 0.21042313 |
| Nfrkb | 0.88629 | -0.10357207 |
| Frk | 0.01511 | 1.3150729 |
| Fgd4 | 0.87823 | 0.08313196 |
| Mlst8 | 0.0127 | -0.69228415 |
| Tmco1 | 0.00257 | -1.1333102 |
| Madd | 0.30202 | -0.56269969 |
| Pkd2l2 | 0.00223 | 1.92233475 |
| Krr1 | 0.77633 | 0.15941301 |
| Cep97 | 0.34353 | -0.31555006 |
| Ccdc88a | 0.00811 | -1.19015431 |
| Selm | 0.00144 | -1.20872454 |
| Atp8b3 | 0.00927 | -1.58895338 |
| B230110C06Rik | 0.00144 | 1.86922753 |
| Ccdc91 | 0.01743 | -0.54831661 |
| Tmem39b | 0.0416 | -0.4222632 |
| Zfp326 | 0.00144 | 2.19138368 |
| Tpgs2 | 0.47773 | 0.16688057 |
| Pdzd8 | 0.00144 | 1.36361609 |
| Vmn1r57 | 0.00144 | -1.31884045 |
| Snapc3 | 0.00144 | 2.15664292 |
| Tmem95 | 0.71746 | -0.14037799 |
| Stard9 | 0.00991 | 0.96034555 |
| Ccdc85b | 0.00144 | -1.21291506 |
| Nostrin | 0.00243 | 1.7495217 |
| Npffr2 | 0.00144 | -1.26934956 |
| B3galt2 | 0.62412 | 0.25497502 |
| Agmo | 0.00144 | -1.29746368 |
| Proz | 0.00149 | -1.22582153 |
| Piezo2 | 0.00144 | -1.20555712 |
| Tfcp2 | 0.00144 | -1.28116048 |
| Myo1b | 0.60805 | -0.13259307 |
| Nqo2 | 0.41666 | -0.18891049 |
| Arhgef2 | 0.26872 | -0.32192308 |
| Gm7361 | 0.00145 | -1.2388121 |
| Trpc1 | 0.86671 | 0.07803173 |
| Dars | 0.00144 | -1.360947 |
| E230029C05Rik | 0.00324 | 2.04892886 |
| Naa30 | 0.73444 | 0.07960495 |
| Kif21b | 0.00144 | -1.26560808 |
| Gdap2 | 0.00212 | 1.0831935 |
| Tnc | 0.00144 | -1.28116048 |
| Ppp1r17 | 0.00145 | -1.26219879 |
| Dstyk | 0.02758 | 0.42057627 |
| Metrnl | 0.03009 | -0.91810798 |
| Rab40c | 0.01367 | -0.57273546 |
| Wdr4 | 0.56516 | 0.09768177 |
| Rbm8a | 0.00907 | 0.56894916 |
| Gm40091 | 0.12828 | -2.78490372 |
| Ric3 | 0.49286 | -0.42521148 |
| Eda2r | 0.01198 | 1.12818355 |
| Irf8 | 0.01032 | -0.60600079 |
| Gspt1 | 0.83341 | -0.08486974 |
| Slc2a3 | 0.0442 | 0.43440055 |
| Elovl7 | 0.47336 | -0.30799703 |
| Edem1 | 0.1671 | 0.25501478 |
| Nanp | 0.27599 | -0.4033548 |
| Snrpn | 0.17013 | 0.6905377 |
| 1190002F15Rik | 0.06018 | -1.03494608 |
| Ap2a2 | 0.01831 | -0.63471714 |
| Dab1 | 0.00144 | -1.48224879 |
| Lrrc28 | 0.56344 | -0.45129646 |
| Tatdn1 | 0.03055 | 0.72863068 |
| Rhebl1 | 0.69697 | -0.09658893 |
| Fam83a | 0.00198 | -1.02881939 |
| Drd5 | 0.00144 | -1.26934956 |
| Plagl2 | 0.16211 | -0.36377791 |
| Taar9 | 0.08569 | -1.05058795 |
| Fbxw13 | 0.00144 | -2.33995106 |
| Scn7a | 0.02599 | -0.91544118 |
| Gm2a | 0.02707 | -0.78572727 |
| Chkb | 0.58169 | -0.13178176 |
| Ccdc153 | 0.48364 | -0.248792 |
| 1700073E17Rik | 0.66786 | 0.08646305 |
| Cntnap5b | 0.00144 | -1.28898079 |
| Rspo2 | 0.00144 | -1.28116048 |
| Gm7265 | 0.01503 | 1.46920754 |
| Sox5 | 0.00704 | -1.19266991 |
| Smad3 | 0.68639 | 0.20306545 |
| Sgk1 | 0.00144 | -1.30626366 |
| Acsl6 | 0.00954 | 0.83815325 |
| 9430041J12Rik | 0.03287 | -0.8956869 |
| Ank3 | 0.41425 | -0.1434618 |
| Impad1 | 0.07983 | -0.30211639 |
| Zfp868 | 0.06113 | -0.46057492 |
| Ankrd24 | 0.64519 | 0.15535383 |
| Gcc2 | 0.00144 | 1.49562174 |
| Plcb1 | 0.01259 | 1.32646217 |
| Sgsm3 | 0.03182 | -0.5518775 |
| Fam185a | 0.58126 | 0.1962856 |
| Ank1 | 0.00144 | -1.26042915 |
| Phlda2 | 0.36717 | -0.61446255 |
| Chrna4 | 0.00144 | -1.27165604 |
| 1700084K02Rik | 0.02976 | -0.89897698 |
| Pygb | 0.63796 | 0.32128251 |
| Tm7sf2 | 0.80433 | 0.06021175 |
| Mrm1 | 0.01119 | -0.84550754 |
| Kctd17 | 0.00144 | -1.05815667 |
| Kcnb1 | 0.19419 | -0.31848672 |
| Mtmr11 | 0.82879 | -0.05685252 |
| Arid5a | 0.17039 | -0.48821675 |
| Serpinf1 | 0.26737 | -0.38588687 |
| Mcfd2 | 0.09164 | 0.44667633 |
| Gm5347 | 0.00145 | -1.2388121 |
| Timm8a2 | 0.31902 | 0.65873841 |
| Celsr2 | 0.01227 | -0.98778948 |
| Megf9 | 0.00144 | 2.71357759 |
| Eno1 | 0.10656 | -0.54264244 |
| Agap2 | 0.26558 | -0.74389008 |
| Otub2 | 0.12065 | -1.27500431 |
| Obscn | 0.55165 | -0.32474051 |
| Copg1 | 0.84932 | -0.0702663 |
| Dpysl5 | 0.01613 | -1.49384308 |
| Rnf4 | 0.7896 | -0.20382321 |
| Mapk8ip1 | 0.31202 | -0.36495816 |
| Rhno1 | 0.17575 | 0.60846414 |
| Ccdc92 | 0.04163 | -1.72402986 |
| B3gat1 | 0.07105 | -0.98516583 |
| Rprd1b | 0.00144 | -1.22007743 |
| Arhgap15 | 0.00144 | -1.31495282 |
| E2f6 | 0.08651 | 0.49619747 |
| Gria4 | 0.12808 | -0.77262898 |
| Sf3b6 | 0.87593 | -0.06815684 |
| Dtwd2 | 0.00144 | 1.99523499 |
| A930001A20Rik | 0.00144 | -1.27165604 |
| 1700122C19Rik | 0.00144 | -1.27165604 |
| C2cd2 | 0.62532 | 0.16134058 |
| Stxbp4 | 0.00144 | -1.29746368 |
| Rhobtb3 | 0.00144 | 1.8667099 |
| Abra | 0.31979 | -0.44966568 |
| Osm | 0.00144 | -1.22925777 |
| Speer4cos | 0.00144 | -1.20228444 |
| Klhl29 | 0.86511 | -0.04861561 |
| Bcl2l11 | 0.26981 | 0.27958024 |
| Wee2 | 0.02018 | -0.98288437 |
| Syt9 | 0.49091 | 0.75494555 |
| LOC102634835 | 0.00144 | -1.29746368 |
| Cyp2j13 | 0.10604 | -1.37550153 |
| Olfr1384 | 0.02707 | 0.58440289 |
| Sost | 0.00144 | -1.26560808 |
| Gm14420 | 0.00218 | 1.38149913 |
| Zfp758 | 0.02469 | 0.93523875 |
| Lrrc74b | 0.00144 | -1.27699683 |
| Cyp4a29 | 0.47938 | -0.52581192 |
| Ndufa7 | 0.00339 | -1.0617458 |
| Ttll5 | 0.05182 | 0.62592551 |
| Slit2 | 0.00727 | -1.07804057 |
| Ccdc155 | 0.00144 | -1.44320067 |
| Glra4 | 0.00145 | -1.2388121 |
| Pcsk5 | 0.30379 | -0.64116419 |
| Pten | 0.00145 | 1.61575858 |
| Hoxc9 | 0.00144 | -1.30017714 |
| Kdm6a | 0.00144 | -1.30017714 |
| Tmem131 | 0.26758 | -0.80430058 |
| Rttn | 0.06047 | 0.5456566 |
| Foxred1 | 0.22857 | 0.49026818 |
| 9630028B13Rik | 0.00144 | -1.29746368 |
| Hspa4 | 0.01773 | -0.91766968 |
| Tollip | 0.39036 | -0.20030716 |
| Crb1 | 0.21556 | -1.38211503 |
| Foxn2 | 0.02728 | 0.66589088 |
| Rtfdc1 | 0.00935 | -0.73153014 |
| Akr1c12 | 0.23676 | 0.27971136 |
| Trmt1l | 0.45275 | 0.14465695 |
| Map4 | 0.00475 | -0.97815128 |
| Usp17le | 0.01669 | -1.06721129 |
| Txndc9 | 0.01767 | 0.69521353 |
| Zfp640 | 0.00144 | -1.25118919 |
| Tmbim4 | 0.23311 | -0.87965713 |
| Sntn | 0.0708 | -2.3676036 |
| Bcl2l10 | 0.00144 | -1.32766452 |
| Rnase9 | 0.03518 | -1.22031528 |
| Ccdc37 | 0.80412 | 0.13011235 |
| Olfr350 | 0.00144 | -1.30713006 |
| Tfb1m | 0.06722 | 0.66439163 |
| Olfr564 | 0.00144 | -1.31495282 |
| Trim32 | 0.29161 | -0.51201763 |
| Tpcn1 | 0.74749 | -0.23044812 |
| Gbp9 | 0.91691 | 0.04594435 |
| Mark3 | 0.47436 | -0.13676034 |
| Lipg | 0.00144 | -1.31518407 |
| Eif4a2 | 0.02259 | -0.70577638 |
| Rprd2 | 0.29596 | -0.52799534 |
| F2r | 0.00144 | 1.4101616 |
| Pik3ip1 | 0.81885 | 0.08283446 |
| Gabrb3 | 0.00144 | -1.26560808 |
| Rnf6 | 0.00286 | -1.33258241 |
| Pde7b | 0.00654 | 0.8252125 |
| Ric3 | 0.00145 | -1.22376446 |
| Creb3l2 | 0.11234 | -0.46587475 |
| Malsu1 | 0.00616 | -0.99034031 |
| Ric3 | 0.00212 | 1.61788981 |
| Kpna4 | 0.00377 | 0.99795042 |
| Lcn10 | 0.59454 | -0.35176099 |
| Bpifa2 | 0.00144 | -1.31884045 |
| Rgp1 | 0.45179 | -0.13190583 |
| H2-Aa | 0.20346 | -0.43773242 |
| Gpx2-ps1 | 0.57334 | -0.30185688 |
| Them6 | 0.05131 | -0.60730971 |
| Prss33 | 0.80206 | 0.06082877 |
| Csrnp2 | 0.42048 | -0.19273272 |
| Olfr1051 | 0.08376 | -2.45439036 |
| Loxl4 | 0.00145 | -1.18144017 |
| Fblim1 | 0.00239 | -0.81739616 |
| Accsl | 0.00145 | -1.22193674 |
| Mier1 | 0.50185 | 0.280321 |
| Nfatc4 | 0.00177 | -0.87140442 |
| Zfp532 | 0.07624 | 0.48719346 |
| Mier1 | 0.0085 | 0.70384384 |
| Lzts1 | 0.00564 | -1.78479103 |
| C130013H08Rik | 0.00144 | -1.27165604 |
| Stk32b | 0.00152 | -1.25878908 |
| Gm7120 | 0.0355 | 1.30374056 |
| Pgm3 | 0.36804 | -0.1561532 |
| Dis3l2 | 0.00171 | -1.29004271 |
| Tbccd1 | 0.01455 | -1.74611368 |
| Med17 | 0.14755 | -0.25413961 |
| H13 | 0.064 | 0.64499902 |
| 4930404I05Rik | 0.26465 | -0.75805832 |
| D930020B18Rik | 0.00347 | -1.32353662 |
| Boll | 0.00144 | -1.25118919 |
| Ppp6r3 | 0.33619 | -0.35136646 |
| Sufu | 0.00453 | -0.77980525 |
| Bhlha15 | 0.17818 | -0.61804971 |
| Eml5 | 0.00144 | 1.51288375 |
| Sub1 | 0.01322 | 0.76055549 |
| Ccdc108 | 0.02698 | -1.05214967 |
| Pon2 | 0.00144 | 2.25353091 |
| Synj1 | 0.00145 | 1.04824543 |
| Rundc1 | 0.84446 | -0.05225849 |
| Slc6a6 | 0.1293 | -0.39889313 |
| Inpp5j | 0.49356 | -0.35707222 |
| Arsb | 0.49259 | -0.36280169 |
| Hdgf | 0.00144 | -0.97465222 |
| Cyb5r3 | 0.78381 | 0.07243919 |
| Csnk1a1 | 0.00145 | -1.04898266 |
| C1d | 0.01497 | -0.58659326 |
| Utp6 | 0.58732 | -0.90821987 |
| Pbx2 | 0.22646 | -0.92593449 |
| Nap1l4 | 0.72881 | 0.12195924 |
| Aspg | 0.41935 | 0.2370782 |
| D630045M09Rik | 0.00462 | 1.48345881 |
| Rnf19a | 0.00144 | 1.38804266 |
| Trp63 | 0.00144 | -1.27165604 |
| Ispd | 0.13934 | 0.61212386 |
| Pitpnm2 | 0.00637 | -1.23002015 |
| Ccdc186 | 0.0367 | 0.57689292 |
| C230035I16Rik | 0.12324 | 0.29461413 |
| Lrrc27 | 0.98619 | 0.00571105 |
| Dlst | 0.06124 | 0.82059232 |
| Rec114 | 0.00204 | -1.76555969 |
| Dtl | 0.00704 | -1.19266991 |
| Timm13 | 0.00414 | -1.10708143 |
| Mgat4c | 0.00144 | -1.26560808 |
| Ubd | 0.00145 | -1.2388121 |
| Naif1 | 0.04196 | -0.82376463 |
| Gskip | 0.9777 | -0.00670198 |
| Rcor3 | 0.00144 | -1.3235377 |
| Fndc3a | 0.51672 | 0.19453982 |
| Tnik | 0.61153 | -0.3642786 |
| Canx | 0.03888 | 0.55864566 |
| Snx19 | 0.80924 | -0.05079989 |
| Izumo4 | 0.03573 | 0.53261989 |
| Kcnc3 | 0.01844 | -0.58737921 |
| Heyl | 0.24565 | -0.33050115 |
| Isl1 | 0.00145 | -1.22193674 |
| Alyref2 | 0.00367 | -0.82374338 |
| Taz | 0.04239 | 0.4539995 |
| Bves | 0.33551 | -0.32916177 |
| Naa60 | 0.09489 | -0.69864033 |
| 9430020K01Rik | 0.03506 | 0.57870855 |
| Arrb2 | 0.01664 | -0.66989178 |
| Als2 | 0.19416 | 0.40391797 |
| Rap2a | 0.04198 | 0.37516927 |
| Gabra2 | 0.00144 | -1.28116048 |
| Zfp62 | 0.76298 | -0.45312057 |
| Suv420h1 | 0.03615 | -0.50426706 |
| Syne1 | 0.00187 | 0.94344165 |
| Luc7l2 | 0.00144 | 2.01767403 |
| Rfx5 | 0.0125 | 2.13285765 |
| Idnk | 0.00583 | -0.82058248 |
| Prima1 | 0.05879 | -3.45232933 |
| 5730458M16Rik | 0.40831 | 0.34765072 |
| C130021H21Rik | 0.02245 | 1.20501037 |
| Tiprl | 0.05699 | 0.56361567 |
| Pole4 | 0.75081 | 0.07357265 |
| Dscaml1 | 0.01836 | -1.6756369 |
| Gm15350 | 0.02531 | -1.10913788 |
| Cdkal1 | 0.29858 | 0.23504997 |
| Slc2a9 | 0.00144 | -1.30017714 |
| Mustn1 | 0.00488 | -1.24112528 |
| Taar4 | 0.3298 | -0.62807868 |
| Agap3 | 0.00273 | -0.8338634 |
| Clock | 0.12037 | 0.34419904 |
| Vdr | 0.72985 | 0.11818797 |
| Atp5c1-ps | 0.645 | 0.28285233 |
| Tas2r118 | 0.31692 | -0.61385397 |
| Rmdn3 | 0.00446 | -0.78645313 |
| Rtel1 | 0.01772 | 0.4648871 |
| Ppp1r42 | 0.10678 | -1.83036001 |
| Capn15 | 0.43327 | -0.12665161 |
| Tbck | 0.02162 | -0.95716175 |
| Adam33 | 0.04947 | 0.61418422 |
| 4933427D14Rik | 0.04824 | -0.86696801 |
| Invs | 0.00144 | -1.26934956 |
| Mcoln3 | 0.00144 | -1.20555712 |
| Uba6 | 0.00144 | -1.5523561 |
| Taco1os | 0.0135 | -0.84191849 |
| Gm35498 | 0.02716 | -0.6892179 |
| Atg10 | 0.00144 | 2.34831695 |
| Gm9798 | 0.21405 | -1.2535353 |
| Ptpn7 | 0.18226 | -0.67549074 |
| Apbb1ip | 0.00446 | -1.08422051 |
| Ddhd1 | 0.08467 | 0.53773043 |
| Shisa3 | 0.02098 | 0.860192 |
| Zp2 | 0.28878 | -0.39243484 |
| Hn1l | 0.11309 | -0.36964083 |
| Trp53rkb | 0.09834 | -0.57277715 |
| Nfasc | 0.06891 | -0.85824446 |
| Nfasc | 0.02148 | -1.1113364 |
| Olfr1325 | 0.00144 | -1.31495282 |
| Lmo1 | 0.00144 | -1.46243164 |
| Col4a3 | 0.00149 | -1.22582153 |
| Brd9 | 0.00175 | 0.92018466 |
| Sox5 | 0.29548 | 0.29662431 |
| Eya3 | 0.00144 | -1.27959248 |
| Cspp1 | 0.00144 | 3.00441174 |
| Lin9 | 0.00144 | 2.08271463 |
| Gm8579 | 0.04551 | -0.91232186 |
| Mtfr1l | 0.80369 | 0.12929734 |
| Tle1 | 0.72408 | 0.06657261 |
| Coa5 | 0.00803 | -1.11990237 |
| Farp1 | 0.39395 | -0.13066445 |
| Agbl2 | 0.00945 | 1.53441058 |
| Lmo4 | 0.6812 | -0.19011062 |
| Inpp4b | 0.59277 | -0.10731075 |
| Myo3b | 0.00144 | -1.29051721 |
| Fanca | 0.00967 | -0.65128366 |
| LOC105247322 | 0.00144 | -1.31495282 |
| Pms2 | 0.39342 | 0.22602397 |
| Mllt3 | 0.14622 | 0.3844764 |
| Prrg2 | 0.00727 | -0.66598688 |
| Ctbs | 0.32927 | 0.41290494 |
| Spice1 | 0.08031 | -0.58777306 |
| Lpp | 0.12874 | 0.41725534 |
| Mbp | 0.02787 | -3.1241035 |
| Calm5 | 0.17112 | -0.75283964 |
| Atp12a | 0.23008 | 0.71089221 |
| D130020L05Rik | 0.11382 | -0.74109449 |
| Olfr305 | 0.00144 | -1.28116048 |
| Rbm26 | 0.00741 | 1.68650157 |
| Cep70 | 0.01602 | 1.24163134 |
| Herc4 | 0.00846 | -0.80085595 |
| Nipa2 | 0.0589 | 0.36151024 |
| Prkcb | 0.2541 | -1.21347474 |
| AI256396 | 0.00757 | 1.57241648 |
| Wdfy1 | 0.01672 | 0.55456545 |
| Prps2 | 0.00144 | 1.87254295 |
| Lrrc16b | 0.02707 | -0.45023881 |
| Ryr3 | 0.06106 | 0.51147836 |
| Lgals1-ps2 | 0.53005 | 0.22985047 |
| Pkm | 0.20131 | -0.51606842 |
| Ankrd32 | 0.00145 | -1.22376446 |
| A930033H14Rik | 0.42212 | 0.29184817 |
| Atp5b | 0.00422 | -0.72719397 |
| Tle1 | 0.111 | -0.35420675 |
| Ppm1h | 0.85922 | -0.04321464 |
| 4930539J05Rik | 0.69379 | -0.17100422 |
| Scaf4 | 0.60472 | -0.36111558 |
| Cdadc1 | 0.09653 | 0.94421144 |
| Gm13219 | 0.1486 | -3.36352166 |
| Gbp5 | 0.02473 | 1.18186674 |
| Rab18 | 0.00144 | 1.4168927 |
| Gm9993 | 0.5173 | -0.19624192 |
| Plcd4 | 0.00405 | -1.83005154 |
| Atf1 | 0.76535 | -0.05881903 |
| Il7r | 0.00144 | -1.29746368 |
| Med7 | 0.00322 | 0.84164332 |
| Cds1 | 0.01617 | 0.88138548 |
| Tbc1d5 | 0.02455 | 0.62153176 |
| Bbs7 | 0.02922 | -0.92715459 |
| Mdm1 | 0.0028 | 1.06757736 |
| Hnf4aos | 0.00144 | -1.33632959 |
| Ndfip1 | 0.19681 | -0.30514511 |
| Nup214 | 0.00472 | -0.75505046 |
| Park2 | 0.00709 | -0.64348111 |
| Tbcel | 0.08993 | -1.08404935 |
| Hcfc2 | 0.00443 | 0.79364644 |
| Ypel3 | 0.00818 | -0.71216913 |
| Ccl25 | 0.01038 | 1.14841258 |
| Unc93a | 0.02058 | -1.03164931 |
| Fam167b | 0.00716 | -0.97272086 |
| Kcna4 | 0.00402 | 1.4298913 |
| Asb16 | 0.22058 | -0.77643978 |
| Erf | 0.00144 | -1.04176533 |
| Samd15 | 0.00144 | -1.29085005 |
| Cdc14a | 0.00144 | 1.56146524 |
| Yy1 | 0.03441 | 0.44239618 |
| Mgmt | 0.00748 | -1.32801678 |
| Snx5 | 0.0042 | 0.76538257 |
| 6530411M01Rik | 0.0093 | -1.1233389 |
| Rnf185 | 0.11039 | -0.59264282 |
| Edil3 | 0.00144 | -1.29085005 |
| Trim16 | 0.80761 | 0.0759837 |
| Palm | 0.00144 | -0.95604125 |
| Arhgap29 | 0.00186 | 1.51719307 |
| Ankzf1 | 0.30906 | 0.22382521 |
| Ttc39c | 0.00144 | -1.26934956 |
| Ntng1 | 0.00144 | -1.31884045 |
| Khdrbs2 | 0.00144 | -1.28116048 |
| Rgs6 | 0.5601 | 0.15468699 |
| Gramd1b | 0.80208 | 0.04042672 |
| Slx4ip | 0.03055 | 1.58131226 |
| Crb1 | 0.00144 | -1.28116048 |
| Ccdc58 | 0.01788 | 1.15388315 |
| Itga6 | 0.01206 | 0.6052248 |
| Aoah | 0.47357 | -0.34945434 |
| Tmed8 | 0.00428 | 1.17951855 |
| Nupr1l | 0.00495 | -1.0858935 |
| Chchd5 | 0.01572 | -0.8987656 |
| Trabd | 0.17164 | -0.49453471 |
| Pcf11 | 0.00944 | 1.52920208 |
| Farsa | 0.56079 | -0.20340777 |
| 4930511J24Rik | 0.0892 | -2.57092917 |
| Grk4 | 0.80201 | -0.09831253 |
| 4931431C16Rik | 0.92553 | -0.05172346 |
| Zfp830 | 0.00264 | 0.81197425 |
| Ifne | 0.20624 | -1.64199786 |
| Hmgn3 | 0.00518 | 0.84074777 |
| Zfp955a | 0.00552 | 1.05004742 |
| 1700054M17Rik | 0.00158 | -1.2446827 |
| Arhgef11 | 0.55825 | -0.15156717 |
| Tcp11l2 | 0.17124 | 0.32831765 |
| Gm30938 | 0.01259 | -0.97019503 |
| Cers6 | 0.81357 | 0.04299307 |
| Gm11992 | 0.02186 | 1.54793327 |
| Exoc8 | 0.10698 | -0.27620369 |
| Mplkip | 0.00296 | 1.93421919 |
| Myom2 | 0.97539 | 0.01109366 |
| Rfx3 | 0.00599 | 0.87988946 |
| 8030451A03Rik | 0.00144 | -1.29864962 |
| Mpzl2 | 0.1874 | 0.52945479 |
| Strn4 | 0.89841 | -0.04663653 |
| Adrb1 | 0.00278 | -0.73173128 |
| Ccdc88b | 0.03456 | -0.73183643 |
| Patl2 | 0.00803 | -1.30816821 |
| 4930470P17Rik | 0.31674 | -0.97618193 |
| Lin28b | 0.00144 | -1.26934956 |
| Krtap3-1 | 0.03087 | -3.98497416 |
| Ice2 | 0.01876 | -0.55466531 |
| Ipo7 | 0.00144 | 1.75293942 |
| 4930544M13Rik | 0.00144 | -1.27165604 |
| Mapre1 | 0.32478 | 0.16601403 |
| Mt3 | 0.00144 | -1.10222687 |
| Tra2b | 0.72835 | 0.10369576 |
| Crbn | 0.00229 | 1.25925802 |
| Mrgprb3 | 0.00144 | -1.26560808 |
| Inca1 | 0.17605 | 0.35359558 |
| Cpa2 | 0.08613 | -1.37672853 |
| Nhlrc3 | 0.27758 | 0.27740565 |
| Clec2h | 0.00149 | -1.22582153 |
| Kdm4c | 0.08089 | -0.33873187 |
| Dgki | 0.00144 | -1.27165604 |
| Map4k3 | 0.00144 | 1.5520923 |
| Syt15 | 0.13988 | -3.32550691 |
| 9330182L06Rik | 0.00144 | -1.28116048 |
| Cdc14a | 0.95636 | -0.02511002 |
| Copg1 | 0.05481 | -0.3490338 |
| Med12l | 0.23586 | -0.7108955 |
| Boc | 0.43696 | -0.1542389 |
| Taz | 0.01198 | -0.78951376 |
| Ankrd37 | 0.23203 | -0.21954781 |
| Nlrp5-ps | 0.33467 | 0.41953117 |
| Efcab11 | 0.01253 | -1.60680751 |
| Lrrc51 | 0.30621 | 0.29491248 |
| Vps13b | 0.01083 | 0.90227672 |
| Lrrc57 | 0.22036 | 0.43234334 |
| Ap3m1 | 0.00145 | 1.16352218 |
| Saa1 | 0.40969 | -0.15811499 |
| Cytip | 0.12272 | -0.75227977 |
| 4931429L15Rik | 0.00144 | -1.28116048 |
| Gm16938 | 0.00462 | -1.78734117 |
| Olfr1441 | 0.43186 | 0.54460149 |
| 1110038B12Rik | 0.03447 | -0.52655337 |
| Acaca | 0.13874 | -0.29805825 |
| Ybx1 | 0.07093 | -0.50590914 |
| Zfp948 | 0.00144 | 3.30880204 |
| Fbxo41 | 0.1496 | -0.55562905 |
| Car1 | 0.02761 | -0.92858739 |
| Heatr9 | 0.00721 | -1.35296456 |
| Pxn | 0.00966 | -0.94972503 |
| Adgrg1 | 0.02912 | -0.54636376 |
| Grip1 | 0.00144 | -1.30737044 |
| Setdb1 | 0.01946 | -1.02877696 |
| Edem2 | 0.4784 | -0.18532859 |
| Sh3bgrl2 | 0.62451 | 0.10317359 |
| B230209E15Rik | 0.00144 | -1.27165604 |
| Ttll5 | 0.08699 | -0.39931989 |
| Ift80 | 0.00213 | -1.2500235 |
| Dpf2 | 0.63437 | -0.32424566 |
| Ttc19 | 0.35354 | 0.22872486 |
| Ost4 | 0.08787 | -0.6710174 |
| Bet1 | 0.01952 | 0.84606088 |
| Pot1b | 0.00172 | 1.81033885 |
| St3gal4 | 0.61453 | -0.16710087 |
| Lamtor4 | 0.01099 | -0.79159753 |
| Olfr987 | 0.64177 | 0.4406427 |
| Ccdc88c | 0.19443 | 0.31440203 |
| D630045J12Rik | 0.00144 | -1.25927895 |
| Prx | 0.00144 | -1.86337011 |
| Cyp2j5 | 0.27913 | -0.68321026 |
| Gpr22 | 0.00144 | 2.23045627 |
| Btg2 | 0.16605 | -0.34212097 |
| Cyb5r1 | 0.27044 | -0.26128594 |
| Lats2 | 0.00297 | 0.86115712 |
| Rax | 0.00144 | -1.27165604 |
| Nktr | 0.03231 | 0.70423668 |
| Mrpl52 | 0.01222 | -0.70624005 |
| Pdx1 | 0.39724 | -0.58382172 |
| Zfp251 | 0.3793 | -0.23264681 |
| Tmx3 | 0.15298 | -2.48584089 |
| Mgat4c | 0.00144 | -1.20555712 |
| A930006K02Rik | 0.00536 | -1.08957845 |
| Abca9 | 0.00144 | -1.24811894 |
| 3110054G05Rik | 0.06979 | -1.32908172 |
| Pkia | 0.00144 | 1.35879332 |
| 1700007H22Rik | 0.00144 | -1.31884045 |
| 1700057H21Rik | 0.02286 | -0.93400357 |
| Ppm1a | 0.01621 | 1.06827702 |
| Rundc3a | 0.00149 | -1.22582153 |
| Rad54b | 0.00144 | -1.31495282 |
| Nf1 | 0.39884 | -0.14399471 |
| Slc8a1 | 0.79271 | -0.09907701 |
| Adgrd1 | 0.01086 | -0.77487734 |
| Yipf7 | 0.19218 | 0.48974727 |
| Gip | 0.18422 | -1.14807836 |
| 4932438A13Rik | 0.26558 | -0.43372318 |
| Oas1h | 0.22409 | -0.67723629 |
| Olfr469 | 0.26749 | -0.69092731 |
| Rtf1 | 0.2705 | -0.33064062 |
| Umod | 0.00144 | -1.29085005 |
| Rnmt | 0.03401 | 0.54903848 |
| 9230114K14Rik | 0.78183 | 0.07506988 |
| Scmh1 | 0.08737 | -0.43801976 |
| Ssr3 | 0.00144 | -1.20393706 |
| Zfp345 | 0.36182 | 0.71733436 |
| Kndc1 | 0.72513 | -0.22010367 |
| Sema3g | 0.0127 | -0.73738512 |
| 9330175E14Rik | 0.03143 | -0.59857082 |
| Mef2a | 0.00385 | 0.78033909 |
| C330018A13Rik | 0.02423 | -0.7833753 |
| Dcdc2a | 0.00144 | -1.28898079 |
| Plb1 | 0.00144 | -1.29051721 |
| Ibtk | 0.08609 | 0.41560618 |
| Luc7l | 0.74629 | 0.10312125 |
| 1700020G17Rik | 0.00144 | -1.47691607 |
| Hcn1 | 0.01147 | -1.06301127 |
| LOC102637542 | 0.00145 | -1.22376446 |
| Gfm1 | 0.00508 | 1.40493584 |
| Brd7 | 0.95272 | 0.00970873 |
| Gm9757 | 0.00144 | -1.37184153 |
| Gfra1 | 0.00865 | -1.21780262 |
| Eif4e | 0.04198 | 0.52341448 |
| Ston2 | 0.80434 | -0.06177585 |
| Nlrp4a | 0.00294 | -1.47167917 |
| Car6 | 0.0018 | -1.67183603 |
| Tas2r110 | 0.00144 | -1.29051721 |
| Wsb2 | 0.97018 | 0.01490317 |
| Bcl9 | 0.02018 | 1.11090459 |
| Cdkn3 | 0.97966 | 0.00707867 |
| Cdkn3 | 0.31967 | -0.3426705 |
| Fam208a | 0.00144 | 2.74225938 |
| Ulk4 | 0.00144 | -1.69669451 |
| Ttc23 | 0.11927 | -0.35297086 |
| Cmtr1 | 0.03806 | -0.38209628 |
| Cfh | 0.03835 | 0.93045591 |
| Jak2 | 0.23644 | 0.45155906 |
| Pde12 | 0.10152 | -0.74105567 |
| Cdk14 | 0.08093 | 0.79849882 |
| Srr | 0.39448 | 0.24466065 |
| Tktl2 | 0.04899 | -2.13183995 |
| St6galnac5 | 0.11908 | -2.94429667 |
| Chmp4c | 0.03287 | 0.43678856 |
| Polr3c | 0.61734 | -0.13430583 |
| Apobec3 | 0.00154 | 2.19286626 |
| Dnajb4 | 0.00158 | 1.43868697 |
| Skida1 | 0.00388 | -1.06308232 |
| Gm5176 | 0.00787 | -0.97668181 |
| Atxn3 | 0.09897 | -0.37502006 |
| Cpn1 | 0.00144 | -1.28116048 |
| Kptn | 0.0801 | -0.56013024 |
| Vps53 | 0.82858 | 0.06062354 |
| 1810011H11Rik | 0.50182 | -0.28494825 |
| Abca5 | 0.00243 | 1.75030859 |
| Pik3r1 | 0.20891 | -0.29763291 |
| Adgrg7 | 0.00144 | -1.28116048 |
| Pip4k2b | 0.31096 | -0.21081853 |
| Gm9946 | 0.06773 | -2.03826115 |
| Gnaz | 0.14235 | -0.73088169 |
| Slc12a9 | 0.50491 | -0.20038797 |
| Faxc | 0.00144 | -1.21743532 |
| Abca17 | 0.21088 | -0.30923705 |
| Ktn1 | 0.55061 | 0.30375562 |
| 2210408F21Rik | 0.50476 | -0.1837459 |
| Ak9 | 0.00145 | -1.22376446 |
| Mthfd2l | 0.12388 | 0.31768471 |
| Dmd | 0.03168 | 0.55346474 |
| Wdr7 | 0.94793 | 0.03926677 |
| Lnx2 | 0.0183 | -0.57062568 |
| Mterf3 | 0.00157 | -1.19247859 |
| Ppp3cb | 0.50518 | 0.15002325 |
| Prok1 | 0.04318 | -0.92675761 |
| Asph | 0.00175 | 1.32541343 |
| Igf1 | 0.01689 | -0.98325242 |
| 1700007K13Rik | 0.09276 | -1.83245227 |
| Akap2 | 0.01982 | 0.50066818 |
| Epha7 | 0.98957 | 0.00590067 |
| 1700061G19Rik | 0.00523 | -1.55785214 |
| Nuak1 | 0.29981 | -0.19178609 |
| 1700016D06Rik | 0.03132 | -0.97953315 |
| Ammecr1 | 0.12559 | -0.58832026 |
| Mllt4 | 0.04485 | -0.57454987 |
| Chic1 | 0.83079 | -0.08346862 |
| Yipf5 | 0.00149 | 0.88785208 |
| 2410002F23Rik | 0.54518 | 0.09520167 |
| Tmem9 | 0.06636 | -0.62365593 |
| Ntng1 | 0.02871 | -2.03215054 |
| Epb4.1l2 | 0.85619 | -0.06609905 |
| D330041H03Rik | 0.73295 | 0.6599803 |
| Acp2 | 0.37413 | 0.18178309 |
| Spata13 | 0.0871 | -0.35593478 |
| Igsf21 | 0.00145 | -1.74216951 |
| Ccdc32 | 0.38974 | -0.20504358 |
| Pias1 | 0.01114 | 0.71511441 |
| B330016D10Rik | 0.16056 | 0.37760993 |
| Ccser2 | 0.10451 | 0.30085263 |
| Gnb1 | 0.00583 | 0.64911616 |
| D630014O11Rik | 0.00144 | -1.20555712 |
| Crlf1 | 0.00144 | -1.52265203 |
| Ppp1r3c | 0.29196 | -0.37281359 |
| Trrap | 0.03728 | -0.53758037 |
| Isca1 | 0.05064 | -0.46154384 |
| Dnajb5 | 0.62168 | -0.19989479 |
| Mmp14 | 0.08897 | -0.30701074 |
| Card6 | 0.01232 | 0.54440947 |
| Nr1d2 | 0.00144 | 1.54139979 |
| Rpgrip1l | 0.00144 | -1.31884045 |
| Dopey1 | 0.00144 | -1.25927895 |
| Bank1 | 0.00144 | -1.28116048 |
| A1cf | 0.00145 | -1.2388121 |
| Rcbtb2 | 0.94271 | 0.04208699 |
| Grid2 | 0.00144 | -1.24466058 |
| Setd1b | 0.058 | -0.44111129 |
| Tmem163 | 0.30149 | -1.36923852 |
| Ccr9 | 0.41177 | -0.3560541 |
| Trpm4 | 0.25042 | -0.19703933 |
| Caap1 | 0.0201 | 1.25954331 |
| Btbd1 | 0.10442 | 0.38700931 |
| Slc15a5 | 0.00145 | -1.22376446 |
| Col8a2 | 0.03953 | -0.82130946 |
| Fah | 0.43096 | 0.33643916 |
| Kcng3 | 0.00158 | -1.3789654 |
| Cyp4v3 | 0.01336 | 0.7987598 |
| Kcnv1 | 0.08659 | -0.95548789 |
| Suz12 | 0.31327 | 0.22018462 |
| Gm31679 | 0.00144 | -1.25118919 |
| Il20rb | 0.45967 | 0.26477801 |
| Itga3 | 0.02706 | -0.58799502 |
| Gm4794 | 0.00144 | -1.54151844 |
| Map3k10 | 0.00144 | -1.22683191 |
| Tppp2 | 0.00144 | -1.34263533 |
| Robo1 | 0.87315 | -0.18237681 |
| Vamp2 | 0.13576 | -0.3754596 |
| Aaas | 0.4415 | -0.20427227 |
| Tmco3 | 0.06081 | 1.03819734 |
| Med1 | 0.28759 | 0.28692018 |
| Tob2 | 0.0019 | -0.97521298 |
| Pgm2l1 | 0.58611 | -0.22046821 |
| Tnrc6b | 0.44987 | -0.19466589 |
| Rccd1 | 0.00364 | 0.83285039 |
| Apbb1ip | 0.09163 | 0.55178394 |
| Lrba | 0.14895 | 0.69448565 |
| Vstm4 | 0.92408 | 0.02449624 |
| Gm38852 | 0.00144 | -1.28898079 |
| Hmga2 | 0.05084 | 0.96539984 |
| Nsun6 | 0.00182 | -1.03154884 |
| Gphn | 0.00619 | 0.99578931 |
| Tcf23 | 0.09228 | -0.4966957 |
| Atp4a | 0.04427 | -0.82662682 |
| Dcp1a | 0.11333 | 0.30287648 |
| Xist | 0.00145 | -1.27532353 |
| 1700019G17Rik | 0.41242 | -1.08937065 |
| Wwp1 | 0.27307 | 0.27995714 |
| Slc16a5 | 0.00144 | -1.30017714 |
| Kif5c | 0.04928 | 0.68830887 |
| Pag1 | 0.11685 | -0.62210471 |
| Prickle2 | 0.74146 | -0.0570327 |
| Sh3rf1 | 0.57836 | -0.12989853 |
| Plxnc1 | 0.01093 | -0.87875058 |
| Bcl2a1c | 0.65949 | 0.17615466 |
| Defb50 | 0.15309 | -3.40568445 |
| Tcfl5 | 0.00144 | -1.38447896 |
| Hist4h4 | 0.00144 | -1.40375446 |
| Cntrl | 0.00589 | 1.07072583 |
| Pkdcc | 0.00814 | -0.77288782 |
| Fam212b | 0.58857 | 0.14046157 |
| Gm2011 | 0.00145 | -1.24191372 |
| Lclat1 | 0.03932 | 0.92263333 |
| Tns3 | 0.08058 | -0.7268388 |
| Rrp1b | 0.79257 | -0.06589315 |
| Slc7a11 | 0.00144 | -1.27165604 |
| Akap7 | 0.00348 | 0.72577918 |
| Efr3a | 0.01292 | 0.70464749 |
| Nbn | 0.00304 | 0.73638883 |
| Numa1 | 0.01795 | -0.47830961 |
| Racgap1 | 0.40537 | -0.4494753 |
| Lgi1 | 0.00351 | 1.33936865 |
| Gm14964 | 0.70535 | 0.12834512 |
| Tdrd3 | 0.91374 | 0.08148059 |
| Peli1 | 0.28012 | -0.19703135 |
| Itgb1bp2 | 0.83932 | -0.06697032 |
| Wibg | 0.00232 | -1.52467716 |
| 9230112J17Rik | 0.00281 | 0.91636699 |
| Chd4 | 0.00261 | -0.84165497 |
| Kansl1 | 0.095 | -0.53300311 |
| Nsdhl | 0.04617 | 0.60087864 |
| Uggt2 | 0.01778 | 0.64715982 |
| Pdia5 | 0.06255 | 0.48912071 |
| BC048562 | 0.00749 | -1.05513651 |
| 0610030E20Rik | 0.00144 | 1.17956688 |
| Sugct | 0.8227 | -0.07466502 |
| Oprl1 | 0.00168 | -1.15098282 |
| Fbxo33 | 0.00776 | 0.70977571 |
| E230001N04Rik | 0.37117 | -0.2866577 |
| Matn3 | 0.39857 | -0.6311297 |
| Heatr5a | 0.00205 | 1.21510889 |
| Fam219b | 0.11284 | -0.28613543 |
| Itpk1 | 0.00744 | -0.68767982 |
| Slc9a8 | 0.48066 | -0.31490767 |
| Cnnm1 | 0.0775 | -0.69039911 |
| 3110045C21Rik | 0.55171 | 0.42728519 |
| Pofut2 | 0.15986 | -0.33468591 |
| Rab11b | 0.13108 | -0.45617174 |
| Dmd | 0.13477 | 0.74495409 |
| Gm19784 | 0.00144 | -1.28898079 |
| Elf1 | 0.77745 | -0.09489268 |
| Galnt18 | 0.01363 | -0.58196475 |
| Rab27a | 0.00144 | -1.34749171 |
| Cstf1 | 0.31702 | -0.15358716 |
| Ttpa | 0.64688 | 0.20169002 |
| Ptprk | 0.04753 | 0.83176713 |
| Hps3 | 0.68324 | -0.2042895 |
| Exosc9 | 0.00144 | -1.27165604 |
| Eya4 | 0.09033 | 0.4860168 |
| Robo2 | 0.00144 | -1.29085005 |
| BC022713 | 0.59879 | -0.15687644 |
| A830005F24Rik | 0.00359 | -1.18713134 |
| Cacnb2 | 0.00144 | 1.89926322 |
| Slc25a28 | 0.00598 | -0.73340913 |
| Zfp28 | 0.03487 | -1.04429809 |
| Nme8 | 0.00144 | -1.49155079 |
| Glb1l3 | 0.00145 | -1.2388121 |
| Cr1l | 0.02962 | -1.20555712 |
| Smim5 | 0.04019 | -0.4985805 |
| Snn | 0.03451 | 0.60061566 |
| Dcdc2c | 0.00144 | -1.28116048 |
| Rdh18-ps | 0.21047 | -0.58430238 |
| Utrn | 0.37042 | 0.24113583 |
| Ces2f | 0.00144 | -1.31884045 |
| Ndufaf2 | 0.04356 | 0.66411359 |
| Sbp | 0.00181 | -1.15960772 |
| Samt4 | 0.00144 | -1.28375735 |
| Odf1 | 0.00145 | -1.22376446 |
| 2310002L09Rik | 0.46539 | 0.264748 |
| Zfp41 | 0.07518 | -0.44537241 |
| D2hgdh | 0.49464 | -0.1559751 |
| Sult2a7 | 0.00145 | -1.6102572 |
| Lgals12 | 0.0017 | -1.24950944 |
| Tshz2 | 0.97844 | 0.04373023 |
| Tcam1 | 0.00144 | -1.28116048 |
| Nipbl | 0.00482 | 1.39692663 |
| 0610010F05Rik | 0.39085 | -0.37100018 |
| Dock4 | 0.01825 | 0.71202766 |
| Mesdc1 | 0.00493 | -0.74203612 |
| Cadm3 | 0.01383 | -1.03849241 |
| Prrg3 | 0.00144 | 1.31752834 |
| Napepld | 0.22732 | -0.24000614 |
| Exosc4 | 0.00564 | -1.02803532 |
| Wdr20rt | 0.02473 | -0.78791609 |
| Rapgef4 | 0.00302 | -0.90967961 |
| Lce1h | 0.01322 | -1.08346893 |
| Apoa5 | 0.00144 | -1.30017714 |
| Hcn4 | 0.00145 | -1.16608929 |
| Ldlrad1 | 0.00144 | -1.20555712 |
| Gtpbp2 | 0.22081 | 0.31286722 |
| Stau2 | 0.3198 | 0.17643751 |
| 4932414N04Rik | 0.0298 | -1.95225942 |
| Ankrd44 | 0.00671 | 1.2828375 |
| Gtf2h2 | 0.69923 | 0.07335723 |
| Gm5458 | 0.00561 | 1.90301561 |
| Tmc7 | 0.007 | -0.98782471 |
| Ccdc77 | 0.01114 | -0.86583679 |
| Nobox | 0.00229 | -1.13943754 |
| Fbxw18 | 0.00144 | -1.28898079 |
| Mamdc4 | 0.09863 | 0.6776413 |
| AW554918 | 0.17084 | -0.45122151 |
| BC016579 | 0.00144 | -1.26560808 |
| Ccnl1 | 0.24488 | 0.53103031 |
| Tstd2 | 0.01138 | -0.83058387 |
| Vti1a | 0.0095 | 1.82274449 |
| Tunar | 0.02729 | -1.25162917 |
| Usp19 | 0.9082 | -0.06752531 |
| Phip | 0.42002 | -1.86614504 |
| Robo2 | 0.00144 | -1.27454685 |
| Zfp182 | 0.00144 | 2.08025205 |
| Meis2 | 0.02596 | 1.68619302 |
| Firre | 0.00144 | 3.09627952 |
| Tbc1d14 | 0.58319 | -0.50025579 |
| Stbd1 | 0.62157 | -0.17417425 |
| 1700001J04Rik | 0.00144 | -1.28116048 |
| Memo1 | 0.00144 | 2.09667603 |
| 4930556A20Rik | 0.00144 | -1.28116048 |
| Stk3 | 0.00205 | 1.67804917 |
| Ssbp1 | 0.00531 | 0.93050417 |
| Gdf6 | 0.01942 | -0.84528631 |
| C730034F03Rik | 0.02899 | -0.93369864 |
| Myo1b | 0.0037 | 1.28659241 |
| Notch2 | 0.89367 | 0.02372736 |
| Cyp2b13 | 0.00144 | -1.29864962 |
| Tcl1b1 | 0.00144 | -1.28898079 |
| Ccdc186 | 0.49507 | -0.20830627 |
| Olfr99 | 0.00144 | -1.42082928 |
| Chmp4b | 0.5953 | 0.154804 |
| 1110051M20Rik | 0.116 | -1.13408129 |
| Mat2b | 0.00144 | -0.98380413 |
| Gm527 | 0.25523 | 0.24962814 |
| Rars | 0.04706 | 0.68618967 |
| Nrap | 0.00309 | -0.80280796 |
| Hif3a | 0.79019 | -0.11600184 |
| Tmem204 | 0.00462 | -0.77670374 |
| Fbln5 | 0.0209 | -1.5999918 |
| Isg20l2 | 0.01104 | -0.54657134 |
| Dcaf10 | 0.07022 | 0.37527679 |
| 9930017N22Rik | 0.0031 | 4.05896332 |
| Npr3 | 0.09631 | -2.79435208 |
| Stk32c | 0.21403 | -1.00786504 |
| Krba1 | 0.68754 | 0.08033979 |
| Scgn | 0.00144 | -1.47699301 |
| Armc6 | 0.72306 | 0.33976552 |
| Gm1661 | 0.00144 | -1.27165604 |
| 4930587E11Rik | 0.00144 | -1.40202659 |
| Man2a2 | 0.5805 | 0.10197443 |
| Ufm1 | 0.37648 | -0.23734397 |
| Phf20l1 | 0.00144 | 1.94559906 |
| Arih1 | 0.01751 | 0.51324601 |
| Slc23a2 | 0.04689 | 0.39440021 |
| Pvrl1 | 0.04174 | -0.43577633 |
| Otc | 0.00144 | -1.25118919 |
| Sh2d1b1 | 0.00144 | -1.26934956 |
| Dhx33 | 0.37053 | -0.14743781 |
| Olfr474 | 0.00144 | -1.56365858 |
| Pde6c | 0.00144 | -1.28116048 |
| Cisd2 | 0.2603 | 0.21939328 |
| Ccdc174 | 0.21911 | -0.37492184 |
| Obsl1 | 0.61433 | -0.20922754 |
| Arfgap2 | 0.8409 | 0.09164548 |
| Ncln | 0.23844 | -0.75942739 |
| Zmym2 | 0.43051 | -0.15933342 |
| Lrp2 | 0.00144 | -1.27165604 |
| Eif4enif1 | 0.00144 | -1.26560808 |
| Vash2 | 0.00144 | -1.31884045 |
| Arhgef38 | 0.00144 | -1.3348153 |
| Rtkn2 | 0.0017 | -1.19401501 |
| Pcif1 | 0.00187 | -0.9166433 |
| Gcn1l1 | 0.09398 | -0.49690695 |
| Sertad4 | 0.07639 | 0.72478653 |
| Sox6 | 0.00144 | -1.44470583 |
| Ubtd2 | 0.73797 | -0.08576763 |
| Prkcz | 0.7136 | 0.10113749 |
| Slco4c1 | 0.00144 | -1.27165604 |
| Rae1 | 0.01822 | -0.51058746 |
| Cdkn2aipnl | 0.29432 | -0.23496839 |
| Wrb | 0.10068 | -1.41791495 |
| Ankrd33b | 0.42315 | 0.14828254 |
| Smim20 | 0.19501 | -0.42756731 |
| Cttnbp2 | 0.80291 | 0.06966146 |
| Mfap2 | 0.2236 | -1.42877762 |
| Rps6ka3 | 0.0018 | 1.09679231 |
| Rcan3 | 0.59618 | -0.13942074 |
| Zc3h7b | 0.26729 | 0.26492127 |
| LOC102632031 | 0.1153 | -2.82250098 |
| Surf6 | 0.00509 | 1.0265762 |
| Slc5a3 | 0.30102 | 0.63554798 |
| Smarca1 | 0.00487 | 2.0399733 |
| Yeats2 | 0.0077 | 0.85224258 |
| Ces1b | 0.00145 | -1.24191372 |
| Spin2c | 0.01211 | -1.05620169 |
| Myd88 | 0.01909 | -0.52793554 |
| Sdc3 | 0.00144 | -1.03403585 |
| Frk | 0.02259 | -1.07888492 |
| Zbtb33 | 0.00272 | 0.9692662 |
| Kif21b | 0.20343 | -0.36253443 |
| D830012I24Rik | 0.00144 | 2.81216801 |
| Rell1 | 0.00144 | -1.29085005 |
| Dock8 | 0.00144 | -1.28116048 |
| Slc45a4 | 0.29316 | -0.2727159 |
| Zfp386 | 0.03257 | -0.7789344 |
| Col8a1 | 0.41526 | -0.15078311 |
| Rnf5 | 0.8426 | 0.11647841 |
| Enah | 0.9144 | -0.04085663 |
| Zdhhc2 | 0.24982 | -0.19391514 |
| Ppp6r3 | 0.01712 | 0.7001692 |
| Ubtf | 0.00412 | -0.71983692 |
| Tctex1d1 | 0.00144 | -1.31884045 |
| Golga1 | 0.36764 | -0.21142866 |
| Wdr5b | 0.0206 | 0.503186 |
| Igfbp5 | 0.01862 | -0.61916435 |
| Gmeb1 | 0.04744 | 0.44267621 |
| Klf13 | 0.93827 | -0.01715169 |
| Mep1b | 0.00144 | -1.28898079 |
| Slc45a4 | 0.0671 | -0.4632796 |
| Kank4 | 0.50957 | -0.32388784 |
| Olfr353 | 0.02082 | -0.90728623 |
| Wdr33 | 0.51369 | -0.13598581 |
| Defb34 | 0.00898 | -1.10356789 |
| Ciz1 | 0.08708 | -0.54672606 |
| Enthd1 | 0.00144 | -1.3348153 |
| CK137956 | 0.03779 | -0.84082387 |
| Sorcs2 | 0.26853 | -0.34163818 |
| Cbfa2t2 | 0.21513 | -1.21981756 |
| Cast | 0.46626 | 0.18974927 |
| Nyap1 | 0.68636 | 0.08639706 |
| Abca12 | 0.70101 | -0.09831956 |
| Fbxo40 | 0.24001 | 0.73158328 |
| Gria4 | 0.00144 | -1.31884045 |
| Cd96 | 0.00145 | -1.2388121 |
| Clock | 0.00144 | 2.51684455 |
| Zcchc11 | 0.00144 | 3.58710916 |
| Acvrl1 | 0.00144 | -1.07071334 |
| Srp54a | 0.00296 | -0.73355541 |
| Vamp8 | 0.03601 | -0.67512623 |
| 9130019P16Rik | 0.00144 | -1.29085005 |
| Trim34a | 0.00144 | 1.81044057 |
| Sgce | 0.02006 | 0.56433989 |
| Lgr4 | 0.01068 | 1.30180725 |
| Myc | 0.19447 | 0.56947941 |
| Eif4ebp3 | 0.00149 | 0.9137736 |
| Gcfc2 | 0.01709 | -0.99496437 |
| Bmp7 | 0.17842 | -0.41428256 |
| Vtcn1 | 0.00144 | -1.5520152 |
| Pear1 | 0.0103 | -0.63746552 |
| Olfr490 | 0.98564 | -0.02369437 |
| Mapkap1 | 0.01672 | 0.65126524 |
| Rad1 | 0.02972 | 1.00401244 |
| Klhl7 | 0.00647 | 1.13225359 |
| Padi4 | 0.00145 | -1.26219879 |
| Snrpb | 0.29524 | -0.35476172 |
| Ints7 | 0.28077 | -0.67915824 |
| Zmym5 | 0.00144 | 1.82229097 |
| Vmn2r107 | 0.00144 | -1.50450276 |
| 1700026N04Rik | 0.00144 | -1.29864962 |
| Gm2762 | 0.9179 | 0.16250257 |
| Daglb | 0.04877 | -0.44750075 |
| Rps6kb2 | 0.00907 | -0.89657827 |
| Syn3 | 0.2167 | -0.84917803 |
| Gtpbp8 | 0.00144 | 2.19600664 |
| Adam23 | 0.03751 | 0.69684208 |
| Ankrd17 | 0.06205 | 0.45236282 |
| C230088H06Rik | 0.00674 | 1.33786587 |
| Tmem198 | 0.01217 | -0.58751513 |
| Bank1 | 0.56079 | 0.18417436 |
| Gpr143 | 0.00145 | -1.25646236 |
| Epha3 | 0.02669 | -0.95942126 |
| 2810408A11Rik | 0.42768 | 0.36720572 |
| Erbb2ip | 0.95083 | 0.01397454 |
| Als2 | 0.01205 | 1.43175839 |
| Lgr4 | 0.02128 | 0.97171431 |
| 1700015E13Rik | 0.00144 | -1.20555712 |
| Ptp4a2 | 0.02018 | -0.6997787 |
| 4930471M09Rik | 0.00144 | -1.27165604 |
| Ralgps2 | 0.94373 | -0.02150974 |
| Rimklb | 0.00144 | -1.26301121 |
| Rccd1 | 0.00144 | -1.20579466 |
| Kif23 | 0.0127 | 1.55981771 |
| Cep128 | 0.23262 | -0.33059316 |
| Ccdc158 | 0.00144 | -1.28116048 |
| Stxbp3 | 0.17912 | 0.43654583 |
| Gpr135 | 0.50577 | 0.24975703 |
| Eras | 0.00144 | -1.30713006 |
| Kpnb1 | 0.91853 | -0.01789741 |
| Olfr1263 | 0.05209 | -0.6977726 |
| Ppp4c | 0.01516 | -0.72738951 |
| Osbpl7 | 0.02424 | -0.48446459 |
| Sppl3 | 0.33595 | 0.38218371 |
| Rbm39 | 0.02628 | 1.72144796 |
| Btg1-ps1 | 0.1203 | -0.86966581 |
| Cdc16 | 0.04378 | -0.42271575 |
| Gm6792 | 0.0144 | -0.66880876 |
| Bahcc1 | 0.01014 | -0.72290576 |
| Rtkn2 | 0.03486 | -0.87406985 |
| Snx14 | 0.00465 | 0.79361986 |
| Dcaf12 | 0.21365 | 0.37489002 |
| 2010001K21Rik | 0.93716 | 0.08189744 |
| Tspan18 | 0.44859 | -0.13554957 |
| Atf7 | 0.07878 | -0.53866376 |
| Cdc37l1 | 0.0093 | 1.25483271 |
| Maea | 0.46142 | -0.15181597 |
| C330024D21Rik | 0.00144 | -1.27165604 |
| Nabp1 | 0.99671 | 0.00295502 |
| Mrpl17 | 0.01351 | -0.782124 |
| Fmo2 | 0.00144 | 1.71700093 |
| Cdk14 | 0.34812 | -1.8691101 |
| Ccdc7 | 0.00144 | -1.28116048 |
| Abhd5 | 0.28916 | 0.4163377 |
| Ifitm5 | 0.27983 | 0.37034377 |
| Ms4a13 | 0.00144 | -1.31495282 |
| Wdr37 | 0.00485 | 1.40971147 |
| Zc3h14 | 0.0016 | 1.35529918 |
| Kcnc2 | 0.00229 | -1.21636428 |
| Scn2a1 | 0.00145 | -1.3643698 |
| Ppp2r5c | 0.02241 | 0.52296777 |
| Kif5b | 0.00145 | 1.11688975 |
| Sh3d19 | 0.02908 | -0.74736859 |
| Myl1 | 0.99847 | 0.00099286 |
| Taf5 | 0.00826 | 0.75441789 |
| Olfr1375 | 0.13384 | -1.1227577 |
| Cpsf1 | 0.34269 | -0.30120875 |
| Dhdds | 0.01606 | -0.69569255 |
| Copa | 0.08128 | -0.45358525 |
| Mapk9 | 0.00463 | -0.68736412 |
| Gria3 | 0.38515 | -1.44152875 |
| Itk | 0.00144 | -1.30713006 |
| Plec | 0.00144 | -1.31731137 |
| Plekhh2 | 0.65125 | 0.14372825 |
| Rab42 | 0.05643 | -0.61615331 |
| Panx3 | 0.00144 | -1.31627578 |
| Bsdc1 | 0.00987 | 1.03293177 |
| Cep120 | 0.00144 | -1.5074314 |
| 9530026P05Rik | 0.73314 | 0.07803112 |
| Lpp | 0.91801 | -0.15298805 |
| Ece2 | 0.31062 | -0.35769197 |
| C030015D19Rik | 0.00144 | -1.3348153 |
| Cd28 | 0.00149 | -1.25408 |
| A430108G06Rik | 0.00144 | -1.26560808 |
| Efr3a | 0.01635 | -0.6912996 |
| Igf2bp3 | 0.00152 | -1.25878908 |
| 1700001C19Rik | 0.00144 | -1.26934956 |
| Dazl | 0.00193 | -1.24713717 |
| Mtpap | 0.00388 | 0.75785328 |
| Cyth4 | 0.00407 | -0.87842829 |
| Cml5 | 0.82566 | 0.1367744 |
| Dnajc18 | 0.57301 | -0.09107741 |
| 0610040B10Rik | 0.69043 | -0.12671407 |
| Gm11149 | 0.00144 | -1.29085005 |
| Scgb2b27 | 0.00149 | -1.19348234 |
| Fam25c | 0.16467 | -0.91989616 |
| Sobp | 0.00144 | -1.31405094 |
| E2f6 | 0.34126 | 0.17908211 |
| Bbs5 | 0.07479 | 1.08401497 |
| Tspyl2 | 0.0126 | 0.81521662 |
| Uggt2 | 0.08791 | 0.58217166 |
| Sox8 | 0.00144 | -1.28116048 |
| Sox8 | 0.58319 | -0.39316024 |
| Ap1g1 | 0.00228 | 0.96114901 |
| Ergic2 | 0.01152 | 0.94014665 |
| Rdh13 | 0.15812 | -0.47731 |
| Pole | 0.00144 | -1.27730451 |
| Bmp3 | 0.00144 | -1.30713006 |
| Elp4 | 0.00176 | 1.67270667 |
| Nek9 | 0.02063 | 0.60593695 |
| Foxd2os | 0.00144 | 2.38088735 |
| Fam227a | 0.22629 | -0.55636539 |
| Ikzf2 | 0.026 | 1.00951074 |
| Wdr19 | 0.0257 | -2.02499415 |
| Helt | 0.59309 | 0.24602766 |
| Arpp21 | 0.11799 | -3.0428244 |
| Raver2 | 0.00144 | -1.29085005 |
| Fn3k | 0.32778 | 0.24155168 |
| Kcnh1 | 0.10606 | -2.71812542 |
| Hipk1 | 0.00979 | 0.64958897 |
| Mrps7 | 0.70612 | -0.15008754 |
| Trdn | 0.03916 | 0.62226565 |
| Rhno1 | 0.00776 | -0.63298369 |
| 4930449C09Rik | 0.03687 | -1.30290368 |
| Cul5 | 0.129 | -0.33830584 |
| Zfp507 | 0.23567 | 0.25604927 |
| Fbxl17 | 0.60233 | 0.15232592 |
| Ctdsp2 | 0.00984 | -0.73267031 |
| Platr25 | 0.00144 | 1.86510787 |
| Hace1 | 0.64585 | -0.28185214 |
| LOC101056014 | 0.00144 | -1.69030187 |
| Ces2g | 0.00144 | -1.26607405 |
| Cd209b | 0.00144 | -1.43903703 |
| Arntl | 0.1699 | 0.74686113 |
| Fam185a | 0.00144 | -1.31627578 |
| Ginm1 | 0.02727 | 0.78432621 |
| Olfr32 | 0.01715 | -1.36803012 |
| Stag1 | 0.01989 | 0.69627961 |
| Trim12a | 0.03016 | 0.84742377 |
| Fgf13 | 0.06354 | -0.47785759 |
| Ksr2 | 0.02075 | -0.74510042 |
| Rmrp | 0.00144 | -1.2915661 |
| Gm4916 | 0.00144 | -1.49537353 |
| Efcab14 | 0.00144 | -1.47883908 |
| Ntsr2 | 0.28297 | 0.35438589 |
| Trpc4ap | 0.30416 | -0.22255069 |
| Ide | 0.05094 | 0.52529642 |
| Ing2 | 0.01087 | 0.96560268 |
| Spock1 | 0.00317 | -1.45898982 |
| Akap17b | 0.02036 | 0.59507621 |
| Mpdz | 0.02865 | 1.15950591 |
| Mbnl1 | 0.0164 | 1.51249758 |
| Fam92b | 0.04025 | -1.53880953 |
| Exoc2 | 0.00144 | -1.27165604 |
| Uty | 0.01114 | -0.89209256 |
| Fgl2 | 0.00144 | 2.41114718 |
| Tagln3 | 0.93053 | 0.09760447 |
| Aldh9a1 | 0.00361 | 0.74325711 |
| Ccdc9 | 0.12247 | -0.26806566 |
| 9530068E07Rik | 0.24673 | -0.18765938 |
| Fyco1 | 0.9151 | 0.04232448 |
| 1810011O10Rik | 0.11295 | 0.54849732 |
| Vmo1 | 0.16292 | -0.62364404 |
| Exoc6b | 0.87412 | 0.03561083 |
| Igsf1 | 0.39795 | -0.26947624 |
| Adarb2 | 0.00144 | -1.27165604 |
| Sertm1 | 0.69347 | -0.2541669 |
| Pdgfd | 0.00495 | 1.06111343 |
| Pdlim2 | 0.18437 | -1.12618015 |
| Cyb5d1 | 0.01602 | 0.56502264 |
| Tma16 | 0.00237 | 1.1445257 |
| Agk | 0.06194 | 1.08550622 |
| Mroh8 | 0.03622 | -1.17423785 |
| Nf1 | 0.00144 | 1.81588073 |
| Cdc23 | 0.21068 | -0.35977533 |
| D430030G11Rik | 0.00165 | 1.19289573 |
| Cbx7 | 0.00234 | -0.78141328 |
| Mettl20 | 0.00193 | 1.05180363 |
| Hes2 | 0.03334 | -0.85094737 |
| Taf10 | 0.04054 | 0.91448197 |
| Arhgef7 | 0.00267 | 1.51913154 |
| 1700021K19Rik | 0.06851 | -1.12256885 |
| Upf3a | 0.57271 | -0.31113345 |
| Xndc1 | 0.00423 | 1.92427819 |
| 3110040N11Rik | 0.85432 | -0.08862042 |
| Tmem33 | 0.62894 | 0.1319747 |
| Negr1 | 0.85613 | -0.07357837 |
| Ahnak | 0.05893 | 0.47911614 |
| 1700001C02Rik | 0.00144 | -1.60169982 |
| 9130011E15Rik | 0.80637 | -0.15378311 |
| Mrpl38 | 0.00523 | -0.72949617 |
| Slc35f1 | 0.00144 | 2.67669054 |
| 2310081J21Rik | 0.00144 | -1.26560808 |
| Fkbp15 | 0.01286 | 1.0656836 |
| 4931406H21Rik | 0.90227 | -0.05217113 |
| Cenpb | 0.10251 | -0.62126563 |
| Atcay | 0.29926 | -0.65198538 |
| Olfr1501 | 0.00149 | -1.22582153 |
| Prdm15 | 0.68634 | -0.17800851 |
| Ccdc93 | 0.00478 | -1.6991375 |
| Rgs3 | 0.01716 | -0.58351438 |
| Ryr3 | 0.44542 | -0.51446386 |
| Rfx1 | 0.49307 | -0.27926661 |
| Cpsf3 | 0.08157 | 0.6884081 |
| Gabpb1 | 0.98328 | 0.00613787 |
| Rttn | 0.01254 | 1.0985324 |
| Slamf7 | 0.01261 | -1.19730632 |
| Il1rl2 | 0.03581 | 0.78193903 |
| Slc12a1 | 0.01631 | -0.80350734 |
| Pkn2 | 0.05858 | 0.34060011 |
| Tmem140 | 0.69957 | 0.09829052 |
| Lppr1 | 0.00144 | -1.27165604 |
| Impg2 | 0.02024 | 0.91532347 |
| Orc4 | 0.00606 | 0.96547056 |
| Fgd2 | 0.43244 | 0.28276008 |
| Ric3 | 0.00417 | 1.0322277 |
| 9530053A07Rik | 0.00854 | -0.98835722 |
| Epb4.1l2 | 0.03721 | 0.58017703 |
| Arhgap5 | 0.01806 | 0.9558293 |
| Olfr171 | 0.00145 | -1.26219879 |
| Zfp397 | 0.14327 | 0.45350515 |
| Card10 | 0.01635 | -0.58230642 |
| Dopey1 | 0.3499 | 0.32616046 |
| Zc3hav1l | 0.96796 | 0.05751682 |
| Gabrb2 | 0.0029 | -1.20702967 |
| Arnt2 | 0.00144 | -1.25118919 |
| Smek1 | 0.01455 | 2.87635479 |
| Cpne3 | 0.00144 | 1.60419512 |
| Fbxo43 | 0.00144 | -1.27165604 |
| Vil1 | 0.63106 | -0.2013542 |
| Pigh | 0.05904 | 0.80152851 |
| Olfr1387 | 0.91515 | 0.03637849 |
| Dennd4c | 0.00144 | 2.50422482 |
| Gpm6b | 0.00144 | -1.31518407 |
| Atcayos | 0.00489 | -1.16266385 |
| U2surp | 0.17086 | 0.5628065 |
| Man1a | 0.00585 | -1.00974955 |
| Mageb4 | 0.00144 | -1.27165604 |
| Upp2 | 0.00144 | -1.29049509 |
| Mia3 | 0.3387 | 0.22475916 |
| Rag2 | 0.00144 | -1.27165604 |
| Prkcq | 0.00158 | -1.2446827 |
| Osbpl8 | 0.00144 | -1.28898079 |
| Rbm25 | 0.00774 | 2.28801797 |
| Dsg3 | 0.01572 | -0.730448 |
| Fkbp15 | 0.04601 | -0.48668864 |
| Cdc42 | 0.07987 | 0.45989859 |
| Olfr1352 | 0.00144 | -1.30017714 |
| Cd180 | 0.05398 | -0.99091235 |
| Slc26a9 | 0.00145 | -1.22376446 |
| Nlgn1 | 0.06702 | -2.26367754 |
| C130023O10Rik | 0.00159 | -1.26431393 |
| Cul4a | 0.90948 | 0.05743626 |
| Sgpp1 | 0.15332 | -0.76914284 |
| Proser1 | 0.05136 | 0.53976663 |
| Tox | 0.00144 | -1.55432167 |
| Crtac1 | 0.34181 | -0.57049623 |
| Fbxo46 | 0.00144 | -1.29193766 |
| Prdm2 | 0.99937 | 0.00022204 |
| Wnt3 | 0.00176 | -1.34618441 |
| Sys1 | 0.00144 | -1.44112336 |
| Soga1 | 0.00144 | -1.20748431 |
| Lpl | 0.00776 | 0.98311109 |
| Cyp4a10 | 0.00144 | -1.29746368 |
| Reg4 | 0.29466 | -0.70041676 |
| Cnnm4 | 0.03187 | -0.54156509 |
| Fam161a | 0.14998 | -0.83412694 |
| Rwdd3 | 0.00333 | 1.76869321 |
| Gm12300 | 0.00314 | -1.13684067 |
| Esm1 | 0.01428 | 1.16576993 |
| Tgif1 | 0.19531 | -1.27034886 |
| 0610009O20Rik | 0.3133 | -0.30991066 |
| 1110065P20Rik | 0.03404 | -0.67845542 |
| Igfbp4 | 0.00162 | -0.8469611 |
| Prr12 | 0.06617 | -0.62970358 |
| Eps8l2 | 0.124 | -0.82090575 |
| Col28a1 | 0.03731 | -0.89043112 |
| Utrn | 0.131 | 0.33253994 |
| Akr1b10 | 0.72339 | 0.1779262 |
| Nudt6 | 0.92408 | 0.0448116 |
| Iqcj | 0.00144 | -1.27165604 |
| Mras | 0.00144 | -1.33521552 |
| Stk19 | 0.02534 | -0.52994231 |
| Zik1 | 0.27051 | 0.34031324 |
| Ccdc14 | 0.77829 | -0.09574786 |
| Rhbdl1 | 0.55712 | -0.09893879 |
| Dbndd2 | 0.14746 | -0.43162172 |
| Grip2 | 0.00296 | -1.00543469 |
| Rnf146 | 0.13494 | -0.46002692 |
| Wdsub1 | 0.32898 | 0.28902039 |
| 3110021N24Rik | 0.02652 | 0.78617951 |
| Erbb4 | 0.00145 | -1.2388121 |
| Abca3 | 0.00144 | -1.26560808 |
| Fam57a | 0.07386 | -0.42122714 |
| Dbr1 | 0.01748 | 0.60658579 |
| Cbx1 | 0.00158 | 1.00236273 |
| Slco2b1 | 0.01562 | -0.62161562 |
| 1810009N02Rik | 0.63647 | -0.12965056 |
| E430021H15Rik | 0.00144 | -1.36658832 |
| Smap2 | 0.52004 | -0.1680187 |
| Il18rap | 0.07372 | -3.0050523 |
| Sec22c | 0.12125 | 0.27633984 |
| Rxfp3 | 0.01074 | -1.04347137 |
| Krit1 | 0.00144 | -1.27454685 |
| Amer3 | 0.07019 | -1.81504699 |
| Zfpm2 | 0.00232 | 2.46554172 |
| Rnf103 | 0.00144 | -1.38451424 |
| Gm12296 | 0.00145 | -1.2388121 |
| Rnf170 | 0.03837 | 0.64569182 |
| Spata4 | 0.04078 | -1.34773377 |
| Cntn5 | 0.25525 | 0.49382655 |
| Slc11a2 | 0.04061 | -0.7237897 |
| Zfp52 | 0.00144 | 2.84482709 |
| Srcin1 | 0.00314 | -1.14703327 |
| Irgq | 0.53045 | -0.2248975 |
| Tas2r139 | 0.00158 | -1.2446827 |
| Cds2 | 0.01743 | -0.59863427 |
| Maz | 0.00144 | -1.18142392 |
| Zbtb45 | 0.04363 | -0.62774823 |
| Sf3b4 | 0.00145 | -1.2388121 |
| Hsd3b6 | 0.61775 | -0.56576286 |
| Snx11 | 0.04362 | -0.81517276 |
| 4930481A15Rik | 0.00198 | -0.91926123 |
| Ttc41 | 0.00144 | 2.30646416 |
| Ankrd46 | 0.05148 | 0.42809578 |
| Syt13 | 0.32664 | -0.52116092 |
| 2810047C21Rik1 | 0.81818 | -0.18968502 |
| Erap1 | 0.64627 | -0.23432514 |
| Ralb | 0.01815 | -0.51824642 |
| Fam177a | 0.00316 | 1.23077779 |
| Pcdhb19 | 0.0732 | -1.62876465 |
| Dsc2 | 0.00215 | 1.88068036 |
| 4930539J05Rik | 0.25709 | 0.46062355 |
| Rangap1 | 0.30902 | -0.28078281 |
| Mak | 0.28442 | -1.21279312 |
| Platr25 | 0.29806 | -0.48940585 |
| Ndufb8 | 0.00391 | 0.85513265 |
| Lppr1 | 0.00144 | -1.28116048 |
| 2700062C07Rik | 0.03655 | 0.42225953 |
| Trim14 | 0.58732 | -0.24699218 |
| Mapk12 | 0.83466 | -0.03441104 |
| Slc39a8 | 0.13838 | -0.83101334 |
| Prpf31 | 0.34277 | 0.3252192 |
| Lce1a1 | 0.00145 | -1.2388121 |
| Nfs1 | 0.48869 | -0.19679012 |
| 4930431F12Rik | 0.05128 | -2.11951299 |
| Prodh2 | 0.00145 | -1.25646236 |
| 6430548M08Rik | 0.01084 | -0.71998849 |
| Fam222a | 0.08415 | -0.85094414 |
| Arhgef40 | 0.00563 | -0.68368294 |
| Cldn2 | 0.00144 | -1.28898079 |
| Trappc8 | 0.52412 | -0.11557331 |
| Gm10158 | 0.01707 | -1.17084646 |
| Phf3 | 0.1592 | 1.04620423 |
| Tmem177 | 0.20798 | -0.33073712 |
| Srsf10 | 0.05874 | -0.38185833 |
| Sox6 | 0.10244 | -0.4012844 |
| Wnt5a | 0.01754 | 0.71418138 |
| Arl5b | 0.00859 | 0.7810824 |
| Iqck | 0.14502 | -0.58979569 |
| LOC102636907 | 0.15455 | -1.70117483 |
| 4930589P08Rik | 0.97059 | 0.00959909 |
| Dnajc13 | 0.03304 | 0.67574418 |
| Zfp106 | 0.04205 | -0.56798212 |
| Dhrs13os | 0.00144 | -2.05660752 |
| Dhx29 | 0.12519 | -0.30596518 |
| Klra17 | 0.00144 | -1.31884045 |
| Adam28 | 0.00144 | -1.28898079 |
| Ticrr | 0.00144 | -1.29746368 |
| Metrnl | 0.53953 | -0.35342981 |
| Zscan29 | 0.02378 | 0.62452724 |
| Agpat6 | 0.00202 | -0.82867814 |
| Rnase1 | 0.78481 | -0.0961386 |
| Tcap | 0.00163 | -0.87336638 |
| 0710001D07Rik | 0.42922 | 0.18870885 |
| 4833417J20Rik | 0.00656 | 0.63251847 |
| Rnf25 | 0.257 | -1.59225634 |
| Ccl12 | 0.06116 | -1.14336712 |
| Ado | 0.01642 | 0.91216533 |
| Vegfa | 0.00144 | -1.36384835 |
| Ubp1 | 0.74528 | 0.07495061 |
| Stau2 | 0.9023 | 0.03646116 |
| Lmbr1 | 0.00472 | -1.25734054 |
| Exoc4 | 0.00144 | 2.28537935 |
| Cerk | 0.0548 | -0.45785654 |
| Olfr583 | 0.00144 | -1.28116048 |
| Cdh5 | 0.06614 | -0.44590302 |
| Zfx | 0.97751 | -0.00635598 |
| Pten | 0.00503 | 1.08348106 |
| Pvrl4 | 0.06401 | 0.80213921 |
| Aven | 0.59896 | 0.21423858 |
| Chsy3 | 0.00144 | -1.31495282 |
| Scyl3 | 0.57517 | -0.14229257 |
| Parl | 0.34681 | 0.41771947 |
| 4930526F13Rik | 0.00625 | -1.77405246 |
| Pds5b | 0.00268 | 1.76986661 |
| Ino80c | 0.02661 | -0.55953748 |
| Prune | 0.00144 | -1.26282022 |
| 9330199G10Rik | 0.09811 | -2.8100463 |
| Dach2 | 0.00145 | -1.24191372 |
| B930025P03Rik | 0.75989 | -0.10693204 |
| Hook3 | 0.83901 | -0.08749936 |
| 0610040B10Rik | 0.01411 | -1.22287441 |
| Ddhd1 | 0.06457 | -0.37426255 |
| Hykk | 0.88956 | -0.08426842 |
| Sh2b3 | 0.57482 | 0.09110697 |
| Xpo4 | 0.22274 | 0.34619697 |
| Ccni | 0.60309 | -0.12044189 |
| Synpr | 0.00144 | -1.27165604 |
| Atf7ip2 | 0.00144 | -1.29085005 |
| Masp1 | 0.31669 | 0.20504646 |
| Rab36 | 0.01456 | -1.03105883 |
| 4930558C23Rik | 0.15164 | -0.52082994 |
| Mgat5 | 0.84775 | 0.06469079 |
| Ndst3 | 0.00145 | -1.7882412 |
| Chmp5 | 0.143 | 0.32821032 |
| Ghitm | 0.01144 | 0.87822814 |
| Ak7 | 0.00144 | -1.31627578 |
| Fam126a | 0.00785 | -0.84098757 |
| Gm33814 | 0.00144 | -1.32766452 |
| Ubxn7 | 0.00293 | 0.75858854 |
| Eif2ak4 | 0.00145 | -1.27532353 |
| H2-T3 | 0.00145 | -1.33072139 |
| Cyp2a5 | 0.22867 | -0.66211611 |
| Catsper3 | 0.57505 | 0.25290986 |
| Cbln3 | 0.00182 | -1.19015431 |
| Ptprk | 0.46449 | 0.13551488 |
| Mknk1 | 0.1747 | 0.2247454 |
| Lrrc40 | 0.09883 | 0.43790253 |
| Zdhhc20 | 0.00144 | 1.25932171 |
| Itga1 | 0.00255 | 2.54331302 |
| Sncb | 0.07627 | -0.86003225 |
| Ncoa6 | 0.00327 | -0.7159527 |
| AW554918 | 0.03824 | 0.48790706 |
| Spt1 | 0.36115 | -0.40698348 |
| Gm31858 | 0.00145 | -1.18741414 |
| Aicda | 0.00144 | -1.22925777 |
| Slc8a1 | 0.00144 | -1.25927895 |
| Ldlrad4 | 0.18114 | 0.46753721 |
| Ncor1 | 0.00351 | 1.59405631 |
| D630033O11Rik | 0.00176 | -1.21714118 |
| Gcnt2 | 0.9315 | 0.04423402 |
| Cyth4 | 0.0044 | -1.04539752 |
| Jarid2 | 0.13321 | 0.53810478 |
| Tcf7 | 0.49305 | -0.18669594 |
| Rab7b | 0.01387 | -0.63001776 |
| Map4 | 0.25342 | -0.19527017 |
| Gm38412 | 0.00626 | -1.53615028 |
| Rabgap1 | 0.16103 | 0.33050624 |
| Evc | 0.02716 | -2.22177864 |
| Nkg7 | 0.00163 | -1.54203001 |
| Sftpa1 | 0.07302 | -0.94335833 |
| 2900093K20Rik | 0.0028 | -0.76418659 |
| Man2c1os | 0.00842 | -0.93339805 |
| Dcx | 0.00773 | -1.04351692 |
| 4930511M06Rik | 0.00149 | -1.22582153 |
| Ankrd11 | 0.04987 | 0.54941476 |
| Cd24a | 0.57826 | -0.25432027 |
| Isoc1 | 0.60625 | -0.12472212 |
| Rcbtb2 | 0.37529 | -0.33740402 |
| Ube2g2 | 0.00579 | -0.7149411 |
| Diablo | 0.1099 | 1.31495282 |
| Cd80 | 0.58889 | 0.21739564 |
| 2610027F03Rik | 0.00144 | -1.30017714 |
| Lrriq3 | 0.00144 | -1.32313902 |
| Hsd17b12 | 0.03351 | -0.45799042 |
| Celf2 | 0.08827 | 0.39184991 |
| Rmdn2 | 0.00144 | 1.6262978 |
| Sema4f | 0.13948 | -0.70515907 |
| Mapk3 | 0.00144 | -1.05833251 |
| Rpgrip1l | 0.0493 | -0.45150388 |
| Lias | 0.00185 | -0.91943222 |
| Ppp6c | 0.04977 | -0.36582263 |
| Kctd3 | 0.25146 | -0.58379284 |
| Zc3h11a | 0.81599 | -0.05197388 |
| Thoc1 | 0.00144 | 1.81151898 |
| Sec11a | 0.27115 | 0.22452092 |
| Dcaf8 | 0.00194 | -0.82376083 |
| Cspp1 | 0.12847 | -0.56778573 |
| Gm8363 | 0.96692 | -0.02046685 |
| Phactr4 | 0.70403 | 0.17647773 |
| Adamts20 | 0.41976 | -0.25920253 |
| Fam149b | 0.00729 | 0.61385308 |
| 4930512H18Rik | 0.0765 | -0.85914525 |
| 6230400D17Rik | 0.00158 | 1.31612343 |
| Spon1 | 0.04437 | 0.56545657 |
| Diexf | 0.33742 | 0.15474309 |
| Tube1 | 0.00144 | -1.29864962 |
| C030034L19Rik | 0.58457 | -0.3348153 |
| Gm2824 | 0.00144 | -1.28898079 |
| Hccs | 0.00144 | -1.30713006 |
| Toporsos | 0.01245 | -0.7273703 |
| Pex16 | 0.03617 | -0.70045793 |
| Slc9a3r2 | 0.00144 | -0.93753843 |
| Pbld2 | 0.20305 | -0.43025461 |
| Mrpl51 | 0.20158 | -0.30630354 |
| Supt7l | 0.11721 | 0.48953555 |
| Zbtb46 | 0.64776 | -0.20937165 |
| Rcl1 | 0.53644 | -0.1342098 |
| Fer | 0.06476 | 0.67951956 |
| Rdh16 | 0.00144 | -1.29085005 |
| Aqp12 | 0.01136 | -2.07470296 |
| Ddx27 | 0.59641 | -0.3605349 |
| Dock3 | 0.02724 | -1.0081862 |
| Olfr633 | 0.00144 | -1.29746368 |
| Zfp317 | 0.00144 | 1.77321907 |
| Id2 | 0.04196 | -0.62132571 |
| Ap1ar | 0.02066 | -0.78273663 |
| Alms1 | 0.59538 | 0.40028853 |
| Cops5 | 0.04944 | 0.73019579 |
| Aimp2 | 0.91006 | 0.07016233 |
| Ppef1 | 0.0652 | -2.39594897 |
| Sdf4 | 0.5158 | 0.10374362 |
| 4921531C22Rik | 0.33591 | -0.34204109 |
| Ppp1r9a | 0.01145 | 0.76924434 |
| Smek1 | 0.00483 | 0.93825923 |
| Klf7 | 0.01372 | 0.85366166 |
| Ipo7 | 0.01395 | 0.89253451 |
| Vps11 | 0.15535 | -0.448209 |
| Pld5 | 0.00144 | -1.3348153 |
| Syngr1 | 0.6491 | 0.19385777 |
| AI597479 | 0.64482 | 0.0883063 |
| Apol10a | 0.00499 | -0.99788027 |
| Zfp148 | 0.00404 | -0.85743114 |
| 4930529K09Rik | 0.00144 | -1.28116048 |
| Zfp955b | 0.04938 | 0.78083794 |
| Fscb | 0.00144 | -1.31518407 |
| Dennd1b | 0.0093 | -0.63308714 |
| Smg8 | 0.00144 | -1.29085005 |
| Shisa9 | 0.03291 | -1.38913938 |
| Ppt2 | 0.0893 | -0.55710061 |
| Ubc | 0.105 | -0.56439202 |
| Sprn | 0.01245 | -0.90023958 |
| Gipr | 0.90797 | -0.11119539 |
| Odf3l1 | 0.15938 | -2.72507922 |
| Gm9975 | 0.97405 | 0.01570496 |
| Psmc3ip | 0.25904 | 0.34806654 |
| Lyz2 | 0.60296 | -0.10861571 |
| Olfr170 | 0.24798 | -0.63455096 |
| Olfr492 | 0.00144 | -1.26934956 |
| Olfr937 | 0.00144 | -1.24613586 |
| Slc19a1 | 0.56658 | 0.16292861 |
| Eif4g1 | 0.00534 | -0.73678392 |
| Nup54 | 0.00456 | 0.76230895 |
| Anapc4 | 0.00609 | 1.47240682 |
| Anapc2 | 0.36813 | -0.26327771 |
| Fn1 | 0.05107 | 0.55395499 |
| Kiss1 | 0.00875 | -1.33833481 |
| Tdgf1 | 0.30739 | -0.75520278 |
| Spty2d1 | 0.00737 | 1.04239371 |
| Dpy19l3 | 0.01074 | 0.69633204 |
| Esr1 | 0.8285 | -0.1362595 |
| Prmt6 | 0.43859 | 0.35504395 |
| Slc38a9 | 0.17711 | -0.32096168 |
| Setd3 | 0.43695 | -0.25884431 |
| B3gat2 | 0.00144 | -1.2383131 |
| Adgrf3 | 0.3909 | -0.56416944 |
| Sp7 | 0.00144 | -1.36353418 |
| Ripply3 | 0.00613 | -0.89365903 |
| 1810008I18Rik | 0.06067 | -1.01649833 |
| Cldn19 | 0.07052 | -1.07123164 |
| 4930431P19Rik | 0.10056 | 0.51585414 |
| Ngfr | 0.0478 | -1.07042188 |
| Cers4 | 0.89341 | -0.02487455 |
| Sumo1 | 0.00144 | -1.29051721 |
| Gast | 0.02253 | -1.19897838 |
| 1700034G24Rik | 0.05621 | -2.49652155 |
| Cct4 | 0.0221 | 1.35192539 |
| Sf3b3 | 0.00564 | -1.01303174 |
| Mtf1 | 0.25435 | -0.19309116 |
| Nwd2 | 0.00144 | -1.27165604 |
| Hipk1 | 0.01802 | 0.50984747 |
| Gm5134 | 0.10998 | -0.56310506 |
| Peg3 | 0.00144 | 1.91504718 |
| Gm839 | 0.11347 | -0.79997687 |
| 4933413J09Rik | 0.02749 | -0.67267552 |
| Ipo5 | 0.10742 | 0.38074208 |
| Usp38 | 0.00144 | 1.24725798 |
| BC030500 | 0.00145 | -1.2388121 |
| Ctif | 0.14597 | -0.35992691 |
| Pigl | 0.38515 | 0.55625329 |
| Fgf21 | 0.00144 | -1.27165604 |
| Pms1 | 0.00144 | 1.06975091 |
| Cnnm3 | 0.41222 | -0.38829427 |
| Dvl1 | 0.15238 | -0.27701605 |
| Mapk6 | 0.00144 | -1.29085005 |
| Pkd2 | 0.00182 | 1.37857556 |
| Ptbp1 | 0.0104 | -1.01859859 |
| Hic2 | 0.53109 | -0.11484654 |
| Tnrc6b | 0.18309 | 0.65610686 |
| Tenm3 | 0.00144 | 1.84887673 |
| Znrf3 | 0.00158 | 2.05948631 |
| Usp33 | 0.02329 | -0.52474264 |
| Depdc7 | 0.36959 | 0.32914475 |
| Cbln2 | 0.00144 | -1.27165604 |
| Olfr279 | 0.52165 | -0.10076701 |
| Ttll13 | 0.00144 | -1.27165604 |
| Trim15 | 0.04698 | -0.91567309 |
| Pphln1 | 0.35687 | 0.14763192 |
| Vwc2 | 0.61625 | 0.30534941 |
| 5730508B09Rik | 0.05583 | 0.41478379 |
| Clec2g | 0.61346 | -0.29894564 |
| Ets1 | 0.2067 | -0.30856129 |
| Cxcl3 | 0.00144 | -1.20555712 |
| Tbx15 | 0.18211 | -0.3227213 |
| Dhh | 0.00144 | -1.48474478 |
| Dyrk3 | 0.88175 | 0.100488 |
| Mup3 | 0.02283 | -0.9976892 |
| Nars2 | 0.00309 | 1.34357315 |
| Olfr1419 | 0.03669 | -0.77790443 |
| Hnrnpa1 | 0.01529 | 0.76732206 |
| Ift140 | 0.00719 | -1.56954191 |
| Ido2 | 0.57025 | 0.46194698 |
| Actl6a | 0.12877 | 0.41089898 |
| Creb3 | 0.49067 | -0.19693378 |
| Wnt7b | 0.02654 | -1.14982275 |
| Skiv2l2 | 0.08513 | -0.73449252 |
| Taf6l | 0.08081 | -0.63070347 |
| Oxsm | 0.47973 | -0.20106921 |
| Cped1 | 0.09292 | 0.86056699 |
| Polr3e | 0.00717 | 1.81227433 |
| Cyp2j6 | 0.00144 | 1.50309119 |
| Nfia | 0.13775 | 1.07028951 |
| Megf9 | 0.68245 | 0.11268351 |
| Snap47 | 0.70631 | -0.12119059 |
| Fcrls | 0.03313 | -0.66034086 |
| Carf | 0.78135 | -0.08667071 |
| Agap1 | 0.00434 | -0.75938899 |
| Dlx6os1 | 0.3073 | -0.47029953 |
| Tmem138 | 0.06288 | -1.06204701 |
| Pibf1 | 0.02237 | -0.72546677 |
| Gtf2h5 | 0.38889 | -0.21686765 |
| Fgf22 | 0.00144 | -1.30299766 |
| Rep15 | 0.56634 | 0.1895495 |
| Mxd4 | 0.01 | -0.55039676 |
| Taf11 | 0.20405 | 0.29263066 |
| 1700065D16Rik | 0.00144 | -1.25118919 |
| Setbp1 | 0.20226 | 0.37383341 |
| Fbxw15 | 0.01216 | -1.93227475 |
| Cbx8 | 0.63112 | -0.24329093 |
| Slc51a | 0.61921 | -0.1511873 |
| Slc22a1 | 0.0101 | -1.65839967 |
| Slc51b | 0.00144 | -1.24613586 |
| Pgm5 | 0.00154 | -0.96550503 |
| Defb37 | 0.31631 | -0.62220419 |
| Cdc42 | 0.80834 | 0.06474554 |
| Rptor | 0.05103 | -0.56090601 |
| Galnt4 | 0.15799 | 0.41183018 |
| Pnpt1 | 0.10553 | -0.29673162 |
| Tmeff2 | 0.80487 | 0.07047163 |
| Rpgrip1 | 0.31318 | -0.75471725 |
| Scn3b | 0.00144 | -1.27165604 |
| Ints3 | 0.173 | 0.32001084 |
| A930004D18Rik | 0.01189 | -0.57409233 |
| A230005M16Rik | 0.00144 | -1.30713006 |
| Tnfrsf1b | 0.00353 | -0.87390286 |
| Gpr176 | 0.11533 | -0.70895209 |
| Lrig2 | 0.20621 | 0.22332857 |
| Smu1 | 0.16648 | -0.26990066 |
| Spns3 | 0.84909 | -0.09010897 |
| Tfrc | 0.13853 | 0.96076202 |
| Cwf19l1 | 0.07026 | -0.57637671 |
| Tmem179b | 0.01706 | -0.61026917 |
| Ipmk | 0.02626 | -0.91997558 |
| Nkain4 | 0.00144 | -1.40018982 |
| Cystm1 | 0.91793 | 0.0483884 |
| Olfr1036 | 0.01378 | 1.13660028 |
| Zfp866 | 0.02026 | 0.55777699 |
| Adam28 | 0.29737 | 0.43202424 |
| Parp2 | 0.00144 | 1.40098967 |
| Kif15 | 0.00144 | -1.21743532 |
| Mllt1 | 0.0016 | -0.99776671 |
| Vipr1 | 0.03266 | -0.96332603 |
| Atp1a2 | 0.24282 | -0.39045012 |
| 4930520O04Rik | 0.75568 | -0.15043672 |
| Cflar | 0.06559 | 0.39958581 |
| Rogdi | 0.50683 | 0.29112294 |
| Enoph1 | 0.51756 | 0.21845159 |
| Thsd4 | 0.48858 | -0.15193109 |
| Smim14 | 0.02076 | 0.50393417 |
| F730043M19Rik | 0.00645 | 1.5065185 |
| Dido1 | 0.00614 | 1.04985582 |
| Olfr78 | 0.00552 | 1.02754682 |
| 1110018N20Rik | 0.37315 | -0.27640051 |
| Gucd1 | 0.00144 | -1.29085005 |
| Mnat1 | 0.99479 | 0.00210494 |
| 6330549D23Rik | 0.04189 | -2.18723872 |
| Csrnp3 | 0.00144 | -1.27699683 |
| Abhd15 | 0.02812 | -1.78240984 |
| Parva | 0.00271 | 0.80259406 |
| Bri3 | 0.1438 | -0.56888318 |
| Cfap61 | 0.04986 | -0.66918227 |
| A630031M04Rik | 0.00144 | -1.26560808 |
| Ubox5 | 0.98473 | -0.0041555 |
| Kctd12b | 0.00539 | 1.35255668 |
| Pard3 | 0.11314 | 0.58767974 |
| 6430548M08Rik | 0.01278 | -1.16329416 |
| Dhx58 | 0.00579 | -0.77617032 |
| Rsg1 | 0.00144 | -1.29051721 |
| Cnot1 | 0.00978 | 0.82428497 |
| Olig2 | 0.04584 | -2.21422847 |
| Dennd6b | 0.39352 | -0.13428343 |
| Brwd1 | 0.0754 | 0.38603312 |
| Firre | 0.00229 | 2.16030927 |
| Mrpl35 | 0.07007 | 0.70706216 |
| Mib1 | 0.54986 | 0.09795082 |
| Akap10 | 0.73649 | 0.12161726 |
| Pln | 0.00144 | 2.0015861 |
| Rptoros | 0.00144 | -1.34049324 |
| Hsd17b11 | 0.14298 | -0.65403096 |
| Abcb4 | 0.01304 | -0.72505033 |
| Synpo2 | 0.62079 | -0.12276075 |
| Pfkp | 0.42515 | 0.39387719 |
| Adck2 | 0.0755 | -0.38429896 |
| Lims1 | 0.12533 | 0.29075843 |
| Pigu | 0.01868 | -0.6530557 |
| Poteg | 0.00144 | -1.28116048 |
| Pum2 | 0.00202 | 1.1100078 |
| Tmem186 | 0.14792 | 0.37717377 |
| Hspb2 | 0.0021 | -0.99906848 |
| Cebpzos | 0.47328 | -0.16069319 |
| C530043A13Rik | 0.00879 | -1.41242282 |
| Vps13c | 0.19818 | 0.30223448 |
| Flnb | 0.64903 | 0.09024627 |
| Gm42102 | 0.08294 | -0.34773515 |
| Tmpo | 0.37387 | -0.39924414 |
| 4931428F04Rik | 0.30597 | 0.325918 |
| Ackr2 | 0.04137 | -0.42847931 |
| D830013H23Rik | 0.12348 | -0.99396601 |
| Chrnb2 | 0.00151 | -1.17792995 |
| Ensa | 0.00475 | -0.7365473 |
| Zfy1 | 0.00152 | -1.25694302 |
| Trim23 | 0.01529 | 1.02312979 |
| Kif12 | 0.00144 | 1.67327203 |
| Spock2 | 0.33894 | -0.40673741 |
| Gm5382 | 0.59339 | -0.34745832 |
| Gm17359 | 0.00144 | -1.28116048 |
| Serpina3j | 0.15662 | -0.88374524 |
| P4ha3 | 0.00359 | -1.49768899 |
| Tgm2 | 0.07141 | -0.41234521 |
| D030068K23Rik | 0.04472 | -1.01963123 |
| Trib2 | 0.24155 | 0.23238757 |
| Tmem229b | 0.00144 | -1.18061357 |
| Serpini1 | 0.00144 | 2.16775532 |
| Uhrf2 | 0.23978 | 0.19701976 |
| Ankle1 | 0.04719 | -0.5988222 |
| Gpr34 | 0.00453 | -1.16434667 |
| Sf3b1 | 0.77675 | 0.05350815 |
| Dkk3 | 0.06312 | -1.15285742 |
| 5830454E08Rik | 0.00297 | -1.14096884 |
| Eif3h | 0.03697 | -0.43566292 |
| Actl6a | 0.16269 | 0.40980143 |
| Pafah1b2 | 0.06824 | -0.65124852 |
| Spg21 | 0.63756 | -0.10362953 |
| Epc1 | 0.00442 | -0.79867392 |
| Cdan1 | 0.93955 | 0.017529 |
| Afmid | 0.5898 | 0.2736499 |
| Mamld1 | 0.71483 | -0.21421521 |
| Cdkl5 | 0.12829 | -2.20010971 |
| A930002H24Rik | 0.05912 | -0.74641901 |
| Tmprss11f | 0.02744 | -1.91554938 |
| Fbxw19 | 0.09095 | -1.01536681 |
| Ssx9 | 0.00145 | -1.15845772 |
| Vasp | 0.00625 | -0.7722188 |
| Frmd8 | 0.00176 | -0.81961117 |
| Dennd4a | 0.03655 | -0.90547877 |
| 3110009E18Rik | 0.68866 | -0.19733564 |
| Cyld | 0.00703 | -1.37580803 |
| Slit2 | 0.75696 | 0.18634562 |
| Sult2a3 | 0.00362 | -1.13214451 |
| Gm35752 | 0.00145 | -1.25646236 |
| Phf20l1 | 0.00325 | 2.32468476 |
| Jade3 | 0.25972 | 0.56470678 |
| Hnrnpc | 0.09745 | -0.32288312 |
| Kif26a | 0.00459 | -0.8368788 |
| Mkl1 | 0.58041 | 0.19958943 |
| A730063M14Rik | 0.04688 | -0.51355629 |
| Ilf2 | 0.01516 | -0.53981534 |
| Abcc8 | 0.22273 | -1.02088582 |
| Zdhhc5 | 0.05923 | -0.36091366 |
| Clec5a | 0.03262 | 1.99227998 |
| A930003O13Rik | 0.13352 | -0.4979382 |
| Wdr36 | 0.04893 | 0.4310301 |
| Ift46 | 0.24205 | -0.49633771 |
| Gm40826 | 0.00157 | -1.19247859 |
| Fzd6 | 0.04007 | 0.3835766 |
| Serpinb6a | 0.93706 | -0.04875318 |
| Ankrd32 | 0.03841 | 0.54192889 |
| Snapc3 | 0.02648 | 0.50965253 |
| 5031425E22Rik | 0.00154 | 2.10416058 |
| Htra3 | 0.00973 | -0.78623874 |
| Ppme1 | 0.17789 | -0.31072712 |
| Slc25a46 | 0.20747 | 0.46472486 |
| Esf1 | 0.00144 | 3.40772361 |
| Galr1 | 0.00144 | -1.34253482 |
| Rtp2 | 0.00145 | -1.26219879 |
| Klk8 | 0.00146 | -0.95471352 |
| Gpr18 | 0.00217 | -1.1219484 |
| Dmtn | 0.02172 | -0.68304985 |
| Pck2 | 0.00144 | -1.18931824 |
| Actr5 | 0.08453 | -1.36283672 |
| Olfr995 | 0.03535 | -1.4063569 |
| Klra19 | 0.00144 | -1.26934956 |
| Nkx2-1 | 0.00144 | -1.29864962 |
| Gm36402 | 0.00144 | -1.30713006 |
| Mllt4 | 0.39645 | -0.25278088 |
| Masp1 | 0.10332 | -0.94758675 |
| Limk2 | 0.00144 | -1.22925777 |
| Alg13 | 0.0027 | 1.32780308 |
| Usp33 | 0.66891 | -0.37858756 |
| F630003A18Rik | 0.00144 | -1.28898079 |
| Tmem209 | 0.00145 | -2.00335122 |
| C030006K11Rik | 0.34731 | -0.35018043 |
| Dph7 | 0.0101 | -0.58256857 |
| Gcnt1 | 0.01442 | 0.82277742 |
| Armc9 | 0.58761 | 0.16968103 |
| C130071C03Rik | 0.00145 | -1.25646236 |
| Ranbp10 | 0.52255 | -0.19836744 |
| 4930556M19Rik | 0.00144 | -1.33632959 |
| Ywhaq | 0.87801 | -0.08259554 |
| Opn3 | 0.36991 | 0.43687713 |
| Acot4 | 0.82006 | -0.1324677 |
| Klf12 | 0.43801 | -0.17234957 |
| Dusp11 | 0.12854 | 0.26161641 |
| Pik3r6 | 0.03988 | -0.39744355 |
| Ctnna1 | 0.03217 | 0.90641848 |
| Mms19 | 0.00144 | -1.30713006 |
| Stx6 | 0.04225 | -0.41690445 |
| Hamp2 | 0.26049 | -0.35217958 |
| Plrg1 | 0.30787 | 0.32779485 |
| Ankrd45 | 0.00833 | -2.30223738 |
| Pmis2 | 0.21257 | -0.73817863 |
| Lcor | 0.00144 | 1.65919052 |
| Rab9 | 0.00155 | 1.31927407 |
| Gm35749 | 0.47742 | -0.33674263 |
| 1700026D11Rik | 0.01295 | -2.04843615 |
| Gapvd1 | 0.06704 | 0.5457424 |
| Cflar | 0.02819 | -0.51383067 |
| Lysmd3 | 0.00736 | 1.39576004 |
| Ccdc47 | 0.00144 | 1.65485149 |
| Gm5148 | 0.00144 | -1.28116048 |
| Cst11 | 0.00144 | -1.30713006 |
| Gt(pU21)140Imeg | 0.62628 | 0.17595028 |
| Mthfr | 0.00145 | -1.22518835 |
| Raf1 | 0.98391 | 0.00442969 |
| Zbtb46 | 0.38072 | 0.34168679 |
| Tmc7 | 0.0255 | 0.67794782 |
| Olfr228 | 0.00145 | -1.22376446 |
| Tas2r107 | 0.00144 | -1.28375735 |
| Fndc5 | 0.02564 | -0.78503291 |
| Chrna5 | 0.00144 | -1.28898079 |
| Zfr | 0.64323 | -0.07941576 |
| Arhgap19 | 0.00144 | -1.31014844 |
| Cul9 | 0.92017 | 0.02815036 |
| Ppp4r2 | 0.00253 | 0.97040484 |
| Lrrk1 | 0.00144 | -1.08955768 |
| Stil | 0.00144 | -1.28797948 |
| Cep128 | 0.00144 | -1.29051721 |
| Rnf125 | 0.00144 | 2.25584893 |
| Mr1 | 0.20943 | 0.30091719 |
| Trmt13 | 0.00632 | 1.27224247 |
| Usp54 | 0.6157 | 0.13962677 |
| Slc18a1 | 0.00144 | -1.32599123 |
| Set | 0.00144 | -1.3348153 |
| Pomt2 | 0.06016 | -0.43009596 |
| Ms4a4d | 0.29452 | 0.17631633 |
| Rph3a | 0.00541 | -1.84952714 |
| Smarce1 | 0.00453 | -1.16434667 |
| Plekha7 | 0.83393 | 0.0874798 |
| Myo1d | 0.00823 | 1.31855766 |
| C230012O17Rik | 0.09122 | -2.56658198 |
| Cdca2 | 0.20412 | 0.43090265 |
| Gnat1 | 0.00144 | -1.32934245 |
| Pkd2l2 | 0.6929 | -0.12322884 |
| Tbx22 | 0.00144 | -1.30017714 |
| Celsr3 | 0.02708 | -1.30300878 |
| Scn3a | 0.00144 | -1.28898079 |
| Fam20b | 0.14214 | -0.29088716 |
| Vcpip1 | 0.07216 | 0.78084759 |
| Nabp1 | 0.08841 | 0.48263039 |
| E430024P14Rik | 0.50825 | 0.26749639 |
| Acta2 | 0.03475 | -0.83349341 |
| Rfx7 | 0.00144 | -1.28898079 |
| Trav3-4 | 0.49353 | 0.80478124 |
| Ttc9 | 0.02235 | -0.62526041 |
| C130071C03Rik | 0.00144 | -1.57547831 |
| Arpp21 | 0.0017 | -1.24950944 |
| Zfyve26 | 0.04645 | 0.45119437 |
| A030001D20Rik | 0.02098 | -0.47522825 |
| Cacnb2 | 0.00254 | -1.01780277 |
| Tmem88 | 0.00451 | -0.86299551 |
| Phf6 | 0.08795 | 0.73989565 |
| Snx12 | 0.09325 | -0.37456599 |
| 4921507G05Rik | 0.01229 | -0.62862724 |
| Mrpl41 | 0.07594 | -0.41700469 |
| Twist1 | 0.97278 | 0.02625611 |
| Mamdc4 | 0.19901 | -0.91956346 |
| Tfcp2l1 | 0.00144 | -1.20616817 |
| Cartpt | 0.6021 | -0.24319535 |
| Fancb | 0.01025 | 1.67128139 |
| Bms1 | 0.00144 | 1.52027533 |
| Khdc1a | 0.00144 | -1.28116048 |
| Madd | 0.24033 | -0.23020184 |
| Olfr1431 | 0.00318 | -1.62382842 |
| Gm10248 | 0.00144 | -1.31495282 |
| Pde1c | 0.00145 | 1.24682748 |
| Ptprm | 0.68359 | 0.52458412 |
| Gtf3c2 | 0.1295 | 0.48054795 |
| Mrps18b | 0.98535 | 0.01067548 |
| Dot1l | 0.52594 | 0.34184503 |
| Syt12 | 0.11817 | -0.7585943 |
| Zfc3h1 | 0.0022 | 1.74597595 |
| Csnk2a2 | 0.61684 | -0.09544038 |
| Edn3 | 0.00182 | -1.75267211 |
| Peak1 | 0.58147 | -0.26736822 |
| Ubap2l | 0.6263 | 0.08674907 |
| Zmym6 | 0.00621 | -1.7813672 |
| Il1rap | 0.14013 | -0.82901203 |
| Aspm | 0.00144 | -1.28116048 |
| Tmem246 | 0.14196 | -0.49349839 |
| Htra4 | 0.47502 | 0.34816985 |
| Bmp3 | 0.26922 | -0.555249 |
| Lca5 | 0.05689 | -1.26477499 |
| Plod3 | 0.13512 | -0.42779056 |
| Nhlrc1 | 0.65566 | -0.11257229 |
| Serpine1 | 0.04428 | 1.04607816 |
| Eda2r | 0.98181 | -0.01718929 |
| Rnf111 | 0.00426 | 0.98852549 |
| Plcxd3 | 0.00145 | -1.2388121 |
| Ergic1 | 0.66481 | -0.21150335 |
| Plch2 | 0.00144 | -1.23532597 |
| Mdga2 | 0.03353 | -0.40622477 |
| Parp16 | 0.28528 | -0.33881112 |
| Prpf18 | 0.00394 | 0.80893079 |
| Klf8 | 0.46954 | -0.21949718 |
| Cylc2 | 0.00207 | -1.12931932 |
| Adss | 0.01049 | 1.17045591 |
| Taf15 | 0.02642 | 0.80980612 |
| Gtf2ird2 | 0.14615 | -0.25777857 |
| Pop4 | 0.6154 | 0.07906613 |
| Cdk19os | 0.53008 | -0.5490593 |
| Tenm3 | 0.01078 | -0.94724597 |
| Rd3 | 0.71782 | -0.13572309 |
| Apitd1 | 0.00144 | -1.24811894 |
| Gen1 | 0.03756 | 0.96145077 |
| Tmc8 | 0.92733 | -0.04553835 |
| Vgll4 | 0.05239 | -0.49499539 |
| Lnp | 0.01131 | 0.98684243 |
| Myl10 | 0.1329 | -0.92014462 |
| Olfr288 | 0.00144 | -1.31495282 |
| Ccdc138 | 0.04411 | -1.27056245 |
| Ttf1 | 0.37745 | -0.25076504 |
| G6b | 0.00144 | -1.31495282 |
| Rap1gap | 0.95529 | -0.03228682 |
| Bag6 | 0.60422 | -0.26823028 |
| Adam11 | 0.33894 | -0.29195239 |
| Gnao1 | 0.11702 | -0.86354997 |
| Cdca2 | 0.00145 | -1.25646236 |
| 4930435E12Rik | 0.00144 | -1.29085005 |
| Pcdhb13 | 0.01702 | -0.94550042 |
| Map4k2 | 0.69237 | 0.06951516 |
| Nova1 | 0.56775 | 0.23069292 |
| Rnf222 | 0.31735 | -0.97316083 |
| Nfat5 | 0.56244 | -0.26283196 |
| Ppp6c | 0.15282 | 0.29814709 |
| Dedd2 | 0.64263 | -0.11073157 |
| D630039A03Rik | 0.55165 | -0.32177153 |
| Spry4 | 0.11559 | -0.84625406 |
| 4933412A08Rik | 0.00144 | -1.28116048 |
| Pabpc1 | 0.01479 | 1.0399946 |
| Pla2g4e | 0.10745 | 0.53404939 |
| 2310009A05Rik | 0.06242 | -0.50250555 |
| Mras | 0.01443 | -0.74103275 |
| D5Ertd579e | 0.90108 | -0.0386746 |
| Vmn2r61 | 0.00144 | -1.29878664 |
| St3gal3 | 0.8537 | -0.10341103 |
| Arl5c | 0.22366 | -0.38435892 |
| Aqr | 0.13381 | 0.31421169 |
| Fzd10 | 0.00144 | -1.28116048 |
| U90926 | 0.03963 | -2.33291988 |
| Ttc24 | 0.01709 | -0.99496437 |
| Proz | 0.00177 | -1.15847607 |
| Rapgef1 | 0.77711 | -0.07645292 |
| Kcnf1 | 0.00144 | -1.27165604 |
| Prmt10 | 0.70698 | -0.23125866 |
| Olfr967 | 0.03198 | -0.95037887 |
| Focad | 0.27911 | 0.27275146 |
| Ccbe1 | 0.22768 | -0.81435063 |
| Sv2a | 0.48232 | -0.57360262 |
| Lrp1 | 0.06691 | 0.86313256 |
| 9330162012Rik | 0.32771 | -0.25450519 |
| Trak2 | 0.01741 | 0.72738579 |
| Gyltl1b | 0.44184 | -0.2885663 |
| E330021A06Rik | 0.18756 | -0.56298674 |
| Rgs7bp | 0.793 | -0.0894429 |
| Nav1 | 0.79363 | -0.05574992 |
| Gabarapl2 | 0.00462 | 0.82833317 |
| Map7d2 | 0.84475 | 0.06051579 |
| Mtif2 | 0.67231 | -0.10456684 |
| Gm26555 | 0.0463 | -1.22513214 |
| Cep350 | 0.00833 | 1.16851658 |
| Nfatc2ip | 0.85902 | 0.10357085 |
| Xpo5 | 0.45353 | -0.11923521 |
| Scrn1 | 0.04833 | -0.51046656 |
| Iars | 0.00202 | 1.30493576 |
| C1qtnf9 | 0.92965 | -0.01686701 |
| Tcf12 | 0.00412 | 1.0111415 |
| Fut4 | 0.76074 | -0.09617713 |
| Olfr825 | 0.00145 | -1.22376446 |
| Aco2 | 0.14302 | -0.40320118 |
| Vrk2 | 0.24929 | 0.57122399 |
| Mocs1 | 0.77799 | 0.04670076 |
| Sptlc2 | 0.4139 | 0.14151952 |
| Rmnd5a | 0.20621 | -0.38709346 |
| Cyp2c40 | 0.00144 | -1.24466058 |
| Lrriq1 | 0.00149 | -1.22582153 |
| Cnbp | 0.00713 | -1.13431288 |
| Trim30d | 0.00144 | 1.30638124 |
| Wdfy4 | 0.00211 | -1.01675286 |
| Kpna6 | 0.21397 | 0.29925793 |
| 1700034J05Rik | 0.00144 | -1.28797948 |
| Csmd3 | 0.04819 | -1.57473903 |
| Psmb6 | 0.00321 | -0.90664249 |
| Cdk13 | 0.01873 | -0.54775694 |
| A830010M20Rik | 0.37913 | -0.2195068 |
| Ermp1 | 0.11466 | -0.7439392 |
| Urgcp | 0.1606 | -0.5374423 |
| Lin54 | 0.02375 | 0.78397338 |
| Tbx20 | 0.87471 | -0.10944647 |
| Rap1gds1 | 0.02203 | -0.972494 |
| Cd55 | 0.00455 | 1.03940716 |
| Tm9sf4 | 0.99977 | -0.00014036 |
| Esrra | 0.39637 | -0.29654515 |
| Samd5 | 0.04144 | 0.73050861 |
| Slc14a2 | 0.02862 | -1.03550528 |
| Gle1 | 0.68948 | 0.10719287 |
| Ccdc171 | 0.7666 | -0.20274409 |
| Cep70 | 0.04176 | 0.48624247 |
| 1810043G02Rik | 0.08386 | -0.72262847 |
| Cd247 | 0.06824 | -1.3935538 |
| Tmem30c | 0.00346 | -1.49902991 |
| Rasgrp3 | 0.00245 | 1.63338217 |
| Ube2u | 0.00144 | -1.27992547 |
| Gpr155 | 0.00152 | -1.25878908 |
| Fuz | 0.01084 | -0.64293362 |
| Mrpl44 | 0.47274 | -0.20431631 |
| Col9a3 | 0.40525 | -0.23532597 |
| Xrcc2 | 0.85479 | 0.06254835 |
| Olfr134 | 0.00145 | -1.24191372 |
| Arl14ep | 0.55873 | 0.2162607 |
| Hmgcll1 | 0.00144 | -1.32313902 |
| Med12 | 0.0346 | 0.41311091 |
| Umps | 0.98605 | -0.0028335 |
| Prkcd | 0.02956 | 0.54142275 |
| Tbc1d30 | 0.01648 | 1.24739558 |
| Wdr37 | 0.08504 | -0.35129916 |
| Bgn | 0.71022 | -0.10087033 |
| Cdk17 | 0.00196 | 1.0797313 |
| Trim66 | 0.00145 | -1.2388121 |
| Phka1 | 0.04583 | 0.68262016 |
| Slc6a18 | 0.00144 | -1.28116048 |
| Zfp169 | 0.50354 | 0.31850162 |
| Ndnl2 | 0.00145 | -1.26219879 |
| Tmc1 | 0.00144 | -1.22925777 |
| Slc11a2 | 0.32336 | 0.35175667 |
| Dzip1 | 0.03352 | 0.61776374 |
| Drd4 | 0.00144 | -1.28116048 |
| 9330154J02Rik | 0.00323 | -1.17111094 |
| Txndc16 | 0.08675 | 0.54943941 |
| Sh3gl2 | 0.09084 | -0.96361298 |
| Gm3120 | 0.00144 | -1.29085005 |
| Ttyh3 | 0.00144 | -1.17163362 |
| Btn1a1 | 0.00144 | -1.28116048 |
| Tcf25 | 0.02856 | -0.62048949 |
| Osbpl1a | 0.00144 | -0.98687223 |
| Tac1 | 0.00144 | -1.34253482 |
| Exosc6 | 0.01387 | -0.68983285 |
| Kcng4 | 0.00144 | -1.26934956 |
| Ctsc | 0.01261 | 0.67253971 |
| Atp5s | 0.17477 | 0.58161265 |
| Rbmx | 0.01727 | 1.71503612 |
| Olfr1309 | 0.23811 | -1.07559808 |
| AW551984 | 0.00144 | -1.29085005 |
| Zfp319 | 0.50934 | -0.14472745 |
| Fgd3 | 0.03449 | -0.8244346 |
| Xxylt1 | 0.13934 | 0.51987572 |
| Esyt3 | 0.77563 | -0.09187815 |
| Oosp1 | 0.00145 | -1.22376446 |
| Arap3 | 0.15856 | -0.30288529 |
| Zfp706 | 0.91978 | 0.04202588 |
| Osbpl9 | 0.05523 | 0.35760404 |
| Zranb1 | 0.00513 | 0.81861943 |
| Ogfod1 | 0.25378 | -0.304693 |
| Ercc6l2 | 0.02939 | -0.60208591 |
| Phkg2 | 0.31797 | 0.40060089 |
| Atp13a3 | 0.153 | -0.31336078 |
| Ets1 | 0.02607 | 0.67782043 |
| Ube2r2 | 0.2327 | 0.42206206 |
| Rps6kb1 | 0.01209 | 0.76705651 |
| Sulf2 | 0.27398 | -0.29478473 |
| Pkd2l1 | 0.15553 | -1.00160033 |
| Trp73 | 0.00145 | -1.2388121 |
| Slc25a30 | 0.83178 | 0.03618652 |
| E130102H24Rik | 0.77841 | -0.13084627 |
| Alkbh8 | 0.0169 | -1.02747293 |
| Chmp4c | 0.09273 | -0.29253016 |
| Afg3l1 | 0.54682 | -0.21824051 |
| 1600002H07Rik | 0.49053 | -0.35490282 |
| Trnau1ap | 0.06298 | -0.4992464 |
| Slc35d2 | 0.04511 | -0.80350734 |
| Txnrd2 | 0.89719 | 0.06089702 |
| Hadha | 0.09132 | -0.44656165 |
| Hfe | 0.04182 | 0.51737392 |
| Chd6 | 0.48902 | 0.34943126 |
| Adgrv1 | 0.00144 | -1.29085005 |
| Pyroxd1 | 0.00879 | 0.84052158 |
| Tnfrsf1a | 0.00145 | -1.00836304 |
| Lgsn | 0.00145 | -1.25646236 |
| Olfr1276 | 0.00449 | -1.04047549 |
| Nup210l | 0.00144 | -1.30713006 |
| Rtn3 | 0.89982 | -0.02281016 |
| Gm30730 | 0.00974 | -1.40286221 |
| Fam71b | 0.0015 | -1.15488942 |
| Sec24b | 0.00392 | 0.72902054 |
| Rnmt | 0.00429 | 1.39527192 |
| Drg1 | 0.48619 | -0.56075323 |
| Msi2 | 0.74865 | -0.13925932 |
| Ubfd1 | 0.64959 | -0.15490367 |
| Fndc8 | 0.0591 | -1.19927706 |
| 9530091C08Rik | 0.09002 | 0.56984096 |
| Adamts19 | 0.00144 | -1.31495282 |
| Baz2a | 0.30084 | -0.78112687 |
| Kcnj6 | 0.23676 | -0.62228531 |
| Eif2ak1 | 0.25685 | 0.44724264 |
| Exog | 0.11659 | 0.35301442 |
| Ankrd34c | 0.00144 | -1.24466058 |
| Ppp1r3fos | 0.06205 | -0.67427598 |
| Rnpc3 | 0.00144 | 1.70848551 |
| Mxd3 | 0.06189 | -1.09190856 |
| Gpatch8 | 0.00202 | 0.88254503 |
| Ifnar2 | 0.5981 | -0.08517738 |
| Gnas | 0.00144 | -1.28116048 |
| Clasrp | 0.85293 | -0.0374847 |
| Lrrc1 | 0.17124 | -0.38427887 |
| Dbnl | 0.19244 | -0.34549038 |
| D130060J02Rik | 0.00597 | -1.11645509 |
| Gbp6 | 0.00144 | 2.00218091 |
| 1700066O22Rik | 0.00145 | -1.18318572 |
| Ppa2 | 0.24537 | 0.54359819 |
| Camk2a | 0.07844 | -0.48150709 |
| Olfr559 | 0.00194 | -1.74957552 |
| Clvs1 | 0.0017 | -1.24950944 |
| Eif4enif1 | 0.88413 | -0.02551678 |
| Atp8a2 | 0.00308 | -2.56578669 |
| 4930404N11Rik | 0.09688 | -0.6419573 |
| Mtm1 | 0.03925 | 1.34749067 |
| Gm32569 | 0.23546 | -0.81895971 |
| Chid1 | 0.8568 | 0.11415675 |
| Thap1 | 0.00155 | 0.97615083 |
| Mga | 0.02424 | 1.05715196 |
| Gk5 | 0.04897 | -0.55657794 |
| Pdcd6ip | 0.1367 | -0.31197057 |
| Dnah9 | 0.00144 | -1.29746368 |
| Gm7056 | 0.00144 | -1.27992547 |
| Tmem67 | 0.07867 | -2.23631707 |
| Pvrl2 | 0.22839 | -0.50554416 |
| Ccdc149 | 0.24049 | 0.2251945 |
| Brcc3 | 0.57329 | -0.31988235 |
| 5430411C19Rik | 0.51459 | 0.1680651 |
| Prosc | 0.37531 | -0.22072005 |
| Lrrfip1 | 0.15913 | 0.29716075 |
| Caps2 | 0.10234 | -2.81327735 |
| Heatr5a | 0.05857 | 1.40274234 |
| Fam159b | 0.06358 | -2.52079602 |
| Camk2d | 0.23029 | -0.27717651 |
| Nwd2 | 0.03107 | -0.94121351 |
| Gne | 0.09557 | 0.35700557 |
| 4930471G03Rik | 0.2009 | -0.61026254 |
| Trat1 | 0.00145 | -1.2388121 |
| Ccar1 | 0.00144 | 2.13757194 |
| Lfng | 0.01509 | -0.78739514 |
| Slco1a6 | 0.00144 | -1.29049509 |
| Zfp335 | 0.03651 | -0.5078283 |
| Arhgap26 | 0.15113 | 0.58479915 |
| Stoml2 | 0.91962 | 0.05025587 |
| Cyb5rl | 0.00622 | -0.96372928 |
| Nubpl | 0.78332 | 0.13096812 |
| Hsbp1l1 | 0.32415 | 0.32118469 |
| Ccdc144b | 0.01836 | -1.11664749 |
| Ikzf3 | 0.01511 | -1.34141653 |
| C87499 | 0.00144 | -1.26934956 |
| Rspo4 | 0.002 | -1.59027635 |
| Nptx2 | 0.00144 | -1.30713006 |
| Osbpl1a | 0.12265 | -0.36851763 |
| Prdx6b | 0.00144 | -1.28898079 |
| Dennd6a | 0.57661 | 0.21927809 |
| LOC102642189 | 0.90767 | 0.03016128 |
| Fndc3a | 0.18738 | -0.56541872 |
| Fcrl5 | 0.0015 | -1.29210076 |
| Spn | 0.09878 | -0.41606678 |
| Crabp1 | 0.00144 | -1.41253772 |
| Trps1 | 0.01784 | 1.28524919 |
| Dnajb12 | 0.00144 | -1.06569203 |
| 1500026H17Rik | 0.0242 | -0.76691673 |
| Sbno2 | 0.04528 | -0.58818861 |
| Klhl17 | 0.15722 | -0.29202368 |
| Arpc4 | 0.00144 | -1.04294397 |
| AI314180 | 0.05576 | 0.39351331 |
| Gna13 | 0.0979 | -0.40241551 |
| Cetn3 | 0.00246 | 0.99511272 |
| Kcnip3 | 0.01618 | -0.76540919 |
| Slc26a3 | 0.00152 | -1.25878908 |
| Xbp1 | 0.29252 | 0.35111335 |
| 1110035H17Rik | 0.0018 | -1.18667933 |
| Adipor2 | 0.85769 | -0.03120597 |
| Lrrc3 | 0.80315 | 0.2159756 |
| Gm10336 | 0.00144 | 1.91853097 |
| Ctnnd1 | 0.37935 | 0.14647336 |
| Qrfp | 0.09044 | -1.49057659 |
| Actl6b | 0.00145 | -1.53030181 |
| Ncam2 | 0.00144 | -1.28898079 |
| Hist3h2a | 0.00232 | -0.96409228 |
| Trpm6 | 0.16469 | -0.86338086 |
| Mthfd2 | 0.01705 | -1.10724265 |
| Abr | 0.13297 | -0.92586919 |
| Adamts2 | 0.68883 | -0.2379389 |
| Dctd | 0.34398 | -0.41192382 |
| Tmem86b | 0.10309 | 1.07025526 |
| Cramp1l | 0.53713 | 0.39970137 |
| Hs3st5 | 0.01851 | 1.14958095 |
| Mei4 | 0.00144 | -1.27165604 |
| Adar | 0.45366 | 0.13723462 |
| Rnf146 | 0.00144 | 1.29953624 |
| Impdh1 | 0.01214 | -0.54823789 |
| Stx19 | 0.00544 | -1.10518964 |
| Cs | 0.14548 | -0.36246827 |
| Trip4 | 0.00307 | -1.10247618 |
| Ube2w | 0.99583 | 0.00089509 |
| Timm9 | 0.04949 | 0.96067251 |
| Rps2 | 0.13162 | -3.0518654 |
| Arhgef12 | 0.12434 | -0.37014987 |
| 4930556M19Rik | 0.16434 | -0.97445464 |
| Pira6 | 0.00149 | -1.18838467 |
| Rln1 | 0.02899 | -0.70450955 |
| Pdik1l | 0.00793 | 1.47105307 |
| Ganc | 0.89834 | 0.03236393 |
| Cdh4 | 0.13086 | -1.58684011 |
| Htr2b | 0.25135 | -0.80656027 |
| Eif5a2 | 0.11412 | 0.29462951 |
| Mfsd7b | 0.08649 | -0.5000076 |
| Nol12 | 0.3154 | 0.40996417 |
| Crkl | 0.09096 | 0.36568964 |
| Olfr136 | 0.01894 | -1.27314258 |
| Prss44 | 0.00144 | -1.30713006 |
| Setd8 | 0.00144 | -1.6113541 |
| Dpp10 | 0.05753 | 1.07628853 |
| Use1 | 0.03824 | -0.75762424 |
| Ccl24 | 0.0486 | -0.52629048 |
| Strada | 0.24994 | 0.53341263 |
| Defb8 | 0.0378 | -0.93613993 |
| Kcnq2 | 0.00145 | -1.22376446 |
| Gigyf2 | 0.14331 | -0.56040387 |
| Exoc3 | 0.03482 | 0.51923052 |
| Ndufaf7 | 0.26819 | 0.28395871 |
| Tmem206 | 0.00359 | 0.83902229 |
| Fam53a | 0.20566 | -0.54853709 |
| 2610020H08Rik | 0.00144 | -1.35320495 |
| Mrpl27 | 0.52342 | -0.47878267 |
| Txlng | 0.00144 | 1.86622191 |
| Uggt2 | 0.0765 | -0.85914525 |
| 2310061I04Rik | 0.33825 | -0.42813395 |
| Epb4.1l5 | 0.29795 | 0.44241698 |
| Atxn7l3b | 0.0204 | 0.79849786 |
| Bcas2 | 0.4172 | 0.17005064 |
| Mpeg1 | 0.02991 | -0.80052678 |
| Mrpl28 | 0.18849 | -0.66546111 |
| 9030407P20Rik | 0.93496 | 0.0387387 |
| E430024P14Rik | 0.42185 | -0.22479247 |
| 4930405A10Rik | 0.00202 | -1.42919588 |
| Ripk2 | 0.0054 | 0.95738576 |
| Txnl4a | 0.27923 | -0.37444026 |
| Adnp2 | 0.42334 | 0.13827382 |
| H2afy | 0.33969 | -0.18000826 |
| Zmym5 | 0.00144 | 2.73124748 |
| Defb38 | 0.00144 | -1.27992547 |
| Bend3 | 0.11342 | -0.27607234 |
| Gm5087 | 0.9541 | 0.02661777 |
| Gucy1b3 | 0.00744 | 1.15713633 |
| Nat6 | 0.94305 | -0.03671522 |
| Fam168b | 0.56977 | 0.08563549 |
| Obox5 | 0.00159 | -1.26431393 |
| Sbds | 0.66995 | 0.08193875 |
| Ndrg4 | 0.02429 | 0.95043236 |
| Arid4b | 0.00144 | 1.65793591 |
| Atp6v0e | 0.99303 | 0.00384947 |
| Slc25a40 | 0.89191 | -0.07783004 |
| Slc6a6 | 0.01095 | -0.64462549 |
| Rbm27 | 0.00589 | 0.98137475 |
| Fam179b | 0.97532 | -0.01223753 |
| Tril | 0.10229 | 0.56156509 |
| Hnf1b | 0.00327 | -1.43580951 |
| Socs6 | 0.00145 | 0.95454464 |
| Cwf19l2 | 0.02529 | 1.34579993 |
| Cdx2 | 0.00144 | -1.29864962 |
| Rab34 | 0.04253 | -2.32818264 |
| Intu | 0.00144 | 2.1996576 |
| Lncbate1 | 0.00144 | -1.27165604 |
| Cox19 | 0.07184 | -0.50884229 |
| Dlk1 | 0.0688 | 0.86272164 |
| Mtl5 | 0.19761 | 0.60284224 |
| Otud1 | 0.4344 | -0.32909546 |
| Il22 | 0.00144 | -1.26560808 |
| Pts | 0.04062 | 0.77732396 |
| Klhl31 | 0.77068 | 0.0530174 |
| Lcorl | 0.73724 | -0.07831323 |
| Taf9b | 0.00144 | 1.43143766 |
| Atat1 | 0.99639 | 0.00095707 |
| Arx | 0.00144 | -1.29746368 |
| Inadl | 0.36384 | 0.35304876 |
| Ahcy | 0.06106 | -0.57376397 |
| Olfr308 | 0.01619 | -1.46662492 |
| Map3k7 | 0.08729 | -0.38050414 |
| Chd7 | 0.65566 | 0.17505856 |
| Gja3 | 0.01011 | 0.88071841 |
| Irf1 | 0.83486 | 0.04007638 |
| Gm13023 | 0.23195 | -0.33461235 |
| A4gnt | 0.00998 | -1.48671094 |
| Zdhhc7 | 0.55069 | -0.20928056 |
| Tfcp2l1 | 0.21435 | -0.6351884 |
| Sf1 | 0.90253 | 0.06673433 |
| Dab2 | 0.01221 | -0.70456417 |
| Rictor | 0.95585 | 0.06045505 |
| Irs1 | 0.22257 | 0.27831195 |
| Lrit2 | 0.00144 | -1.30017714 |
| Usp48 | 0.00144 | 1.50011023 |
| Cd2ap | 0.00144 | 2.47959786 |
| Ranbp17 | 0.20587 | -0.95505236 |
| Cachd1 | 0.90091 | 0.0263958 |
| Ercc4 | 0.19807 | -0.25645249 |
| Ube2e3 | 0.02235 | 1.20444157 |
| Nrp1 | 0.25432 | -0.35295284 |
| Gja1 | 0.03197 | 0.91877123 |
| Gabrq | 0.00144 | -1.26934956 |
| Lrrcc1 | 0.00348 | 2.19575164 |
| Ripk1 | 0.00543 | 1.27855542 |
| Defb20 | 0.00827 | -1.20127384 |
| Gatc | 0.15839 | 0.54128018 |
| 4930458B22Rik | 0.00144 | -1.37159448 |
| Hdhd1a | 0.02057 | -1.58593531 |
| Cdh6 | 0.18396 | -0.95787901 |
| Galc | 0.00289 | 1.18397473 |
| Creb1 | 0.48092 | -0.19107054 |
| Pde4dip | 0.00244 | -0.78474901 |
| Ago4 | 0.45012 | 0.32829613 |
| BC048546 | 0.00144 | -1.28116048 |
| Crkl | 0.34813 | -0.7313419 |
| Cep104 | 0.87387 | -0.05417538 |
| Slc9c1 | 0.00144 | -1.29049509 |
| Myrip | 0.93156 | -0.02019035 |
| Ociad1 | 0.106 | 0.42502691 |
| Lrriq3 | 0.00145 | -1.22193674 |
| Cd247 | 0.025 | -0.73230517 |
| Thsd7a | 0.7151 | 0.06292484 |
| Usp3 | 0.73344 | -0.06606206 |
| Wscd2 | 0.01992 | -0.64007528 |
| Tef | 0.00425 | -0.81742459 |
| Dusp7 | 0.00145 | -0.95828227 |
| Srr | 0.00144 | 1.13602825 |
| Dffa | 0.36948 | 0.50011642 |
| Tm9sf4 | 0.01239 | -1.02834537 |
| Usp34 | 0.00858 | 1.19780546 |
| Fitm2 | 0.03366 | -0.7096913 |
| Rtn4ip1 | 0.74609 | 0.14598691 |
| Tmem150c | 0.12977 | -0.68146471 |
| Zfp629 | 0.0021 | -1.22752875 |
| Pdf | 0.48773 | -0.19343677 |
| Fgf12 | 0.00144 | -1.27165604 |
| Ankrd34c | 0.00144 | -1.30713006 |
| Asph | 0.02482 | 0.71860827 |
| Pla2g4c | 0.3764 | -0.37134508 |
| Ramp1 | 0.12624 | 0.93128355 |
| Med22 | 0.95372 | 0.06627842 |
| Smg1 | 0.00386 | 1.80613152 |
| 2310009B15Rik | 0.0488 | -0.67821211 |
| Olfr152 | 0.00144 | -1.31495282 |
| Ccdc33 | 0.33616 | -0.77406798 |
| BC048679 | 0.00595 | -1.05247312 |
| Znrf1 | 0.06996 | -0.38864045 |
| Tnn | 0.00425 | -0.97828661 |
| Sgsm1 | 0.47145 | -0.33445425 |
| Mpp7 | 0.67288 | 0.07776127 |
| 4933407I05Rik | 0.00144 | -1.31495282 |
| Ccdc106 | 0.0049 | -0.88594507 |
| Col15a1 | 0.69035 | -0.08266034 |
| Uck1 | 0.20718 | -0.39799414 |
| Sirpa | 0.00144 | -1.04418508 |
| Ppp1r10 | 0.42371 | -0.21062542 |
| Ubxn4 | 0.0024 | 0.80633692 |
| Rps6kb1 | 0.19229 | 0.39568583 |
| Hcst | 0.00237 | -0.96035408 |
| Atp6v1b1 | 0.07484 | -1.80084136 |
| Cops7b | 0.85857 | -0.27606281 |
| Apex2 | 0.9409 | 0.02471673 |
| E030046B03Rik | 0.05193 | 0.65548323 |
| Redrum | 0.00144 | -1.27165604 |
| Skint11 | 0.00144 | -1.25118919 |
| Camk2d | 0.01625 | -0.96191222 |
| Jakmip1 | 0.07584 | -0.97331558 |
| Mrrf | 0.86843 | 0.05307412 |
| Rdh14 | 0.00787 | 1.57430749 |
| Cdc14a | 0.34278 | -0.97849785 |
| Auts2 | 0.93261 | 0.03647216 |
| Dbil5 | 0.02651 | -0.59217137 |
| Dnmt3a | 0.89149 | 0.03261561 |
| Vcan | 0.01456 | -0.9300669 |
| Tmcc3 | 0.13276 | 0.29522058 |
| Rasal3 | 0.95507 | -0.02003998 |
| Synrg | 0.0187 | 1.03136041 |
| Fam169b | 0.00544 | -1.10518964 |
| Cebpa | 0.00342 | -0.96514214 |
| Fgf14 | 0.91456 | -0.03492744 |
| Zdhhc21 | 0.35058 | -0.28708084 |
| Slc1a2 | 0.0096 | -0.82507292 |
| Thada | 0.36377 | 0.42636288 |
| Mapk11 | 0.01435 | -0.80725349 |
| Alkbh8 | 0.01021 | 0.96269263 |
| Mia2 | 0.02779 | -0.94311806 |
| Spg20 | 0.97371 | -0.00560812 |
| Scrt1 | 0.03084 | -1.16563544 |
| Atp2a2 | 0.82766 | -0.04058333 |
| Klhl15 | 0.05113 | -2.13584605 |
| Gripap1 | 0.01991 | 0.6563185 |
| Efcab12 | 0.53877 | 0.33063418 |
| Aox3 | 0.02403 | -0.9340016 |
| Pkd1l1 | 0.00319 | -1.61998985 |
| Tnk1 | 0.44889 | -0.56791805 |
| Tmem8 | 0.32389 | -0.37776889 |
| Nol4 | 0.00144 | -1.52432995 |
| Add3 | 0.00461 | 1.05542194 |
| Recql5 | 0.07928 | -1.40734981 |
| Spast | 0.00448 | 0.88277251 |
| Thoc2 | 0.07363 | -1.26978576 |
| Ccdc64 | 0.00144 | -1.29049509 |
| Nudt13 | 0.09174 | 1.07131174 |
| 5330427O13Rik | 0.05845 | 1.02509157 |
| Gigyf2 | 0.00144 | 1.17315518 |
| Taok3 | 0.2792 | -0.27966909 |
| Vamp1 | 0.0037 | 0.97437983 |
| Tmem87b | 0.15376 | 0.24175544 |
| Arhgef6 | 0.00648 | -1.41398053 |
| Swt1 | 0.00144 | 2.35711399 |
| Art1 | 0.82885 | -0.06243173 |
| Tunar | 0.00144 | -1.22098834 |
| Zrsr2 | 0.0142 | 0.72775717 |
| Snap25 | 0.0073 | -1.17129313 |
| Mutyh | 0.00151 | -1.17792995 |
| Etnk2 | 0.41158 | 0.35530594 |
| BC107364 | 0.00144 | -1.29878664 |
| Cdc42ep5 | 0.00144 | -1.03772896 |
| Lsm14b | 0.15779 | -0.37013977 |
| Olfr360 | 0.41486 | -1.03481072 |
| Fancg | 0.91179 | -0.03836794 |
| Wwp1 | 0.00144 | -1.27165604 |
| Sec16a | 0.22863 | -0.2356651 |
| Bpifb4 | 0.0044 | -1.14548549 |
| Gm12250 | 0.0719 | -0.89340883 |
| Lct | 0.02395 | -0.76652009 |
| Kmt2e | 0.00432 | 1.84005808 |
| Nsun6 | 0.00947 | 1.02575294 |
| Hsd17b12 | 0.18656 | 0.61302126 |
| Itgb8 | 0.00144 | -1.28116048 |
| Htr2c | 0.00149 | -1.21097148 |
| Gpd1 | 0.07744 | -0.54153353 |
| Far1 | 0.0747 | -0.80118062 |
| Cln6 | 0.44306 | -0.32872832 |
| Poldip2 | 0.66742 | -0.18068599 |
| Trpd52l3 | 0.00144 | -1.30713006 |
| Ivns1abp | 0.02039 | -0.74581128 |
| Rab40c | 0.14066 | -0.58071722 |
| Tubb5 | 0.00144 | -1.26042466 |
| Atp6v0a2 | 0.00483 | -0.98994426 |
| Krtap3-2 | 0.77075 | -0.18495039 |
| Cfhr2 | 0.00316 | -1.34462115 |
| Chadl | 0.00149 | -1.21097148 |
| Tmem106b | 0.20212 | -0.215598 |
| Ptpn13 | 0.00144 | -1.04258253 |
| Naif1 | 0.01583 | -1.5198561 |
| Spg7 | 0.06923 | -0.46032126 |
| Pvr | 0.50184 | -0.12450684 |
| Ahcyl2 | 0.21377 | -0.20171118 |
| Dph3 | 0.02352 | -2.64958417 |
| Bgn | 0.12284 | -0.4525894 |
| Gabbr1 | 0.59429 | 0.47125205 |
| Dcaf5 | 0.16766 | -0.2765625 |
| Ydjc | 0.43665 | -0.27703772 |
| Mroh7 | 0.00144 | -1.31884045 |
| Snx12 | 0.17896 | -0.45304979 |
| Vps13d | 0.28135 | 0.38865727 |
| Nek7 | 0.00334 | -1.06134173 |
| 5031425F14Rik | 0.00144 | -1.84810684 |
| Ovca2 | 0.04622 | -0.38378802 |
| Cand1 | 0.17029 | 0.24478196 |
| Mcm9 | 0.57194 | 0.17118256 |
| St6gal1 | 0.46172 | 0.16371596 |
| Cdc26 | 0.03741 | 0.92800747 |
| Vti1a | 0.5175 | 0.12545863 |
| Bcl6 | 0.92786 | 0.02538278 |
| Clec4e | 0.32986 | -0.47673716 |
| Luzp2 | 0.03663 | -0.91478561 |
| Usp4 | 0.50446 | -0.26029424 |
| Cnpy4 | 0.41328 | 0.16648212 |
| Tcea3 | 0.00144 | -1.52147203 |
| Cpa1 | 0.85139 | 0.0818152 |
| 2610008E11Rik | 0.00144 | 1.38706708 |
| 4921511H03Rik | 0.00145 | -1.2388121 |
| Cad | 0.24309 | -0.24270693 |
| Akap10 | 0.02651 | 0.429424 |
| Sox11 | 0.00144 | -1.27992547 |
| Gusb | 0.02292 | -0.60944411 |
| Dhx16 | 0.79869 | -0.05070922 |
| Cacna2d1 | 0.02528 | 1.12730568 |
| Sfmbt1 | 0.00844 | -0.61411801 |
| Ptger3 | 0.00746 | -1.55264192 |
| Ubr3 | 0.97891 | -0.00622661 |
| Sh3glb2 | 0.01447 | -0.65954831 |
| Olfr384 | 0.4444 | -1.17640454 |
| Ceacam13 | 0.02671 | -0.93324725 |
| Wee1 | 0.00545 | -1.59648993 |
| Pcyt1b | 0.00144 | -1.33632959 |
| Ddx49 | 0.00511 | -0.73552347 |
| AU021092 | 0.00144 | -1.52177967 |
| Wdr70 | 0.84516 | 0.06217039 |
| Lrba | 0.13698 | -0.67454519 |
| Grhl1 | 0.4233 | -0.95587406 |
| Zfp276 | 0.0058 | -0.86459664 |
| Msh2 | 0.00144 | -1.22925777 |
| Cds2 | 0.82965 | -0.06665027 |
| Slamf1 | 0.89775 | -0.09452148 |
| Fgfr2 | 0.25894 | -0.56581241 |
| Son | 0.17381 | 0.70289831 |
| Nrg1 | 0.92634 | 0.0490042 |
| Hspa13 | 0.00144 | -1.26560808 |
| Prpf40a | 0.74041 | 0.05462191 |
| Slc10a7 | 0.19936 | 0.62640859 |
| Bcl2l11 | 0.07147 | 0.57925212 |
| Tshz2 | 0.01183 | 1.00186168 |
| 2810002D19Rik | 0.01721 | 1.1371367 |
| Qk | 0.00492 | -0.72634099 |
| Hs3st2 | 0.00145 | -1.22376446 |
| Arhgef2 | 0.0462 | -0.67846017 |
| Abhd1 | 0.26108 | -0.51516895 |
| Trim34a | 0.20076 | -0.58814328 |
| Abi1 | 0.0028 | 0.95578634 |
| Rnf157 | 0.3329 | -0.30542818 |
| Scgb1c1 | 0.08962 | 0.69600384 |
| Fkbp15 | 0.10223 | -0.69616737 |
| Wdr95 | 0.06931 | -1.09471958 |
| Ankrd39 | 0.03025 | -0.67206644 |
| Cmah | 0.06466 | 0.61007405 |
| Agtpbp1 | 0.00269 | 1.24358978 |
| Esr2 | 0.00144 | -1.29746368 |
| Fam19a1 | 0.1054 | -2.92107079 |
| Ednra | 0.39398 | -0.21426506 |
| 3830408C21Rik | 0.06175 | 0.88234116 |
| Acaa1a | 0.59656 | -0.22487957 |
| Abca14 | 0.00145 | -1.22193674 |
| 2010204K13Rik | 0.03339 | -0.97622452 |
| Elk4 | 0.00144 | 1.20555411 |
| Cdc42ep2 | 0.00315 | -0.75615801 |
| Zfp317 | 0.99566 | 0.00145948 |
| A430057O09 | 0.03869 | -0.92436403 |
| Ankib1 | 0.76904 | 0.06788608 |
| Pvrl3 | 0.1175 | 0.31567513 |
| Pvrl3 | 0.00211 | 1.29210886 |
| A730049H05Rik | 0.00144 | -1.31884045 |
| Ankrd49 | 0.00144 | 1.32558232 |
| Pla2g16 | 0.00561 | -0.76240406 |
| Wdr20 | 0.53615 | 0.20652084 |
| Nme6 | 0.78951 | 0.20079318 |
| Ecm1 | 0.01597 | -0.74249216 |
| Calca | 0.00145 | -1.22376446 |
| Blk | 0.00144 | -1.27165604 |
| Zcwpw1 | 0.83355 | -0.22231569 |
| Gm13219 | 0.00144 | -1.31769968 |
| Brip1 | 0.00154 | 1.24502461 |
| Ltf | 0.00746 | -1.80417688 |
| Mir99ahg | 0.02961 | 2.3358068 |
| Wnt9b | 0.01399 | -0.9400267 |
| Phldb2 | 0.00456 | 1.2098396 |
| Opa1 | 0.00144 | -1.28116048 |
| Crisp4 | 0.33344 | -0.67231517 |
| Arfrp1 | 0.85139 | -0.06340208 |
| Ttc39b | 0.71621 | -0.20326199 |
| Ackr4 | 0.10867 | -0.3706112 |
| Il31 | 0.0093 | -1.06147486 |
| 4732416N19Rik | 0.10415 | -0.71047292 |
| Sco1 | 0.01377 | -0.56547056 |
| Ptgfr | 0.23653 | -0.57720298 |
| Hoxb2 | 0.0017 | -1.06644157 |
| Ssr1 | 0.05654 | -0.34634106 |
| Gpatch1 | 0.1217 | 0.30294477 |
| Tmem17 | 0.27493 | 0.27077354 |
| Rock1 | 0.02234 | 1.25603116 |
| Gabpa | 0.00144 | 1.45904074 |
| Rpgrip1l | 0.00144 | 2.00970101 |
| Ctbp2 | 0.05925 | 0.58305044 |
| Fbxw24 | 0.00144 | -1.29085005 |
| Clcn5 | 0.69042 | -0.13748203 |
| Dcun1d2 | 0.16549 | 0.29155516 |
| Gm355 | 0.00144 | -1.20579466 |
| Gm5132 | 0.86691 | 0.04169026 |
| Tpd52 | 0.00929 | -0.80068531 |
| Ttc26 | 0.02904 | -1.15468698 |
| Tmem191c | 0.02084 | 1.11104217 |
| Catsperg1 | 0.01359 | -1.05298882 |
| Lrrc75b | 0.39434 | 0.41717959 |
| Tnfaip8l1 | 0.00564 | -0.69619591 |
| Mettl6 | 0.92916 | -0.01944485 |
| Fam150a | 0.00144 | -1.20555712 |
| Gm11494 | 0.00144 | -2.61619533 |
| Homer1 | 0.00144 | 1.68082749 |
| Atg13 | 0.44466 | -0.38174424 |
| Kcnab3 | 0.50318 | -0.44853771 |
| R3hdm1 | 0.00335 | -1.31205117 |
| Txndc11 | 0.7043 | 0.23074352 |
| Adamts12 | 0.00201 | 2.03556801 |
| Grap2 | 0.0156 | -1.08617297 |
| Cog5 | 0.17062 | 0.33958723 |
| Snrpd3 | 0.54069 | 0.18230466 |
| Usp39 | 0.18453 | 0.63365928 |
| Cd8a | 0.0759 | -3.19898459 |
| Ndufaf6 | 0.39744 | -0.22019313 |
| Tmem150cos | 0.00784 | -1.03698725 |
| Kif2a | 0.22248 | -0.30292973 |
| 2610027K06Rik | 0.04789 | -1.06655745 |
| Plcz1 | 0.00145 | -1.22376446 |
| Apom | 0.46388 | -0.34270669 |
| Irak1 | 0.70951 | 0.14126649 |
| Pde4b | 0.00419 | -1.15489314 |
| Rxfp4 | 0.47601 | -0.27295806 |
| Rnf2 | 0.07964 | 0.44914449 |
| Casc1 | 0.86447 | -0.05652218 |
| Slfnl1 | 0.00145 | -1.22376446 |
| Rab25 | 0.02596 | -0.86165417 |
| Tacr1 | 0.00144 | -1.30713006 |
| Tdp1 | 0.18348 | -0.73679101 |
| Pdlim5 | 0.05649 | 0.4843529 |
| 1110051M20Rik | 0.00144 | -1.17439985 |
| Armcx6 | 0.02707 | -0.43960305 |
| Zfp946 | 0.0102 | 1.18695707 |
| Vmn2r53 | 0.00144 | -1.35112763 |
| Gm1564 | 0.06819 | -0.89394135 |
| Plcg1 | 0.05778 | -0.35698016 |
| Mettl22 | 0.80914 | -0.12421345 |
| Trim24 | 0.83561 | 0.06498508 |
| D16Ertd519e | 0.00175 | -1.26015721 |
| Herc4 | 0.64854 | -0.33864718 |
| Hpse | 0.0033 | -1.19712959 |
| D030028A08Rik | 0.00244 | -1.39831793 |
| Prorsd1 | 0.0287 | 0.60556913 |
| 1600014C10Rik | 0.11364 | -0.52520553 |
| Stap1 | 0.06704 | -0.49818247 |
| Chd3 | 0.80963 | -0.06866402 |
| Rnf214 | 0.36198 | -0.16183843 |
| Mrps5 | 0.00144 | -1.01115777 |
| Syne4 | 0.98701 | -0.0081474 |
| Fgfr1op | 0.00181 | -1.13620351 |
| Evi5l | 0.5067 | -0.20785221 |
| Rwdd4a | 0.74997 | 0.08410608 |
| Zbtb8os | 0.07119 | 0.39842723 |
| Flnb | 0.27879 | -0.26333511 |
| Col13a1 | 0.04827 | -0.92782594 |
| Klra2 | 0.00144 | -1.31518407 |
| Cacna2d2 | 0.0343 | -1.10013164 |
| A230059G12Rik | 0.00144 | -1.20555712 |
| Pcdhb11 | 0.00144 | -1.31495282 |
| Scfd2 | 0.28341 | -0.39049053 |
| Tshb | 0.00144 | -1.27165604 |
| Speer4b | 0.03999 | -0.75227769 |
| Arhgef38 | 0.00145 | -1.22376446 |
| Atl3 | 0.02124 | 0.87159444 |
| Tcf7l2 | 0.20194 | -0.28499924 |
| Ccdc74a | 0.00406 | -1.45478658 |
| Dync1li2 | 0.0106 | 0.69071171 |
| Cd86 | 0.00774 | 1.31021382 |
| Vcpip1 | 0.00281 | 1.14056077 |
| 1700101I11Rik | 0.71094 | 0.2514912 |
| Ism1 | 0.00149 | -1.25408 |
| Tshr | 0.00144 | -1.29049509 |
| Prr5 | 0.35191 | -0.34135176 |
| Snhg14 | 0.00144 | 4.07843884 |
| Rictor | 0.01326 | 0.89109761 |
| Cpxm2 | 0.64664 | 0.42007483 |
| Stradb | 0.38425 | 0.29375453 |
| Zyg11b | 0.19891 | 0.39095187 |
| Tcp10c | 0.69478 | -0.23347991 |
| Shtn1 | 0.25251 | -0.98044883 |
| Taf1d | 0.31121 | 1.26019611 |
| Sdccag8 | 0.10947 | 0.61760148 |
| Gm11476 | 0.00144 | -1.29085005 |
| Nme6 | 0.6755 | -0.22122535 |
| Ppp1r1a | 0.06947 | -0.57964069 |
| Tceanc2 | 0.40585 | 0.1495955 |
| Nkx1-2 | 0.00144 | -1.29085005 |
| Srrm4 | 0.20773 | -0.38018124 |
| Atxn1 | 0.06604 | -0.34449704 |
| Fgfr4 | 0.00229 | -0.89459943 |
| Gad1 | 0.00145 | -1.2388121 |
| Tspyl3 | 0.00244 | -0.87692681 |
| Zbtb44 | 0.28804 | -0.25426409 |
| Fam72a | 0.76162 | 0.05200573 |
| Rbms1 | 0.00145 | 0.91738011 |
| BC049715 | 0.00145 | -1.12949857 |
| Gcc1 | 0.42623 | -1.2487478 |
| Rbpms | 0.37136 | -0.32358876 |
| Il17rc | 0.00416 | -0.70398652 |
| Glul | 0.89912 | -0.05162835 |
| Arhgef39 | 0.00497 | -1.44422612 |
| Tgoln1 | 0.38455 | 0.17622077 |
| Otud6a | 0.00144 | -1.41534266 |
| 1700013D24Rik | 0.87384 | -0.17792995 |
| Pcsk5 | 0.68705 | 0.50998763 |
| Kremen1 | 0.0481 | -0.50355996 |
| Brd4 | 0.08969 | -0.36870486 |
| Slc38a11 | 0.00424 | -1.97417356 |
| Tex2 | 0.05981 | -0.44267952 |
| Herc1 | 0.01908 | -0.60175161 |
| 2610015P09Rik | 0.01257 | 0.73922064 |
| Rps20 | 0.0037 | 0.89332342 |
| 1700031C06Rik | 0.00286 | -1.03387158 |
| Jph2 | 0.47613 | -0.37085148 |
| Dtna | 0.80688 | -0.2197337 |
| Gpr37l1 | 0.02551 | -1.3707051 |
| Tcf3 | 0.01395 | -0.53719489 |
| H2afy | 0.4232 | -0.12171084 |
| Grk5 | 0.18995 | -0.2570266 |
| Galp | 0.04558 | -2.18915969 |
| Olfr1202 | 0.01647 | -1.0478897 |
| Cdcp1 | 0.48136 | -0.42351083 |
| Acot10 | 0.08326 | 0.48143105 |
| Rhbdf1 | 0.24656 | -0.21973733 |
| Hnrnpf | 0.25427 | -0.33175245 |
| Csnk1g3 | 0.9378 | -0.01951211 |
| Slc26a10 | 0.65309 | 0.11485556 |
| Hspa13 | 0.00853 | 0.89919285 |
| Golga3 | 0.60364 | 0.08912734 |
| Gm29686 | 0.13726 | 0.82742491 |
| Scfd1 | 0.02313 | 1.2938661 |
| Hepacam2 | 0.00144 | -1.17452065 |
| Slmo1 | 0.02945 | -0.86832866 |
| Nipal1 | 0.00144 | -1.20555712 |
| Osbpl9 | 0.02616 | -1.81009509 |
| Phf21b | 0.75595 | -0.10205105 |
| Zc3h6 | 0.00144 | 2.02165215 |
| Adamtsl1 | 0.40269 | -1.47277793 |
| Ndufa1 | 0.28525 | 0.16715055 |
| Mdp1 | 0.03604 | 0.93429976 |
| Atf4 | 0.91217 | -0.03133119 |
| Olfr112 | 0.00144 | -1.26911831 |
| Ska1 | 0.46135 | 0.34669913 |
| Pfn4 | 0.00485 | -0.73859759 |
| Edem3 | 0.00144 | 2.31798468 |
| Fam150b | 0.85166 | 0.06674643 |
| Tmem221 | 0.03767 | -0.90782975 |
| Slc26a3 | 0.00144 | -1.20555712 |
| H2-DMb2 | 0.1508 | -0.69794067 |
| Ube2a | 0.79646 | 0.13718892 |
| Hook1 | 0.08038 | -0.56571346 |
| Slc32a1 | 0.08798 | -0.92187831 |
| B3gnt2 | 0.4929 | 0.11896888 |
| Dlgap3 | 0.22261 | -0.33422735 |
| Gpr63 | 0.02126 | -1.12383671 |
| Prkaca | 0.01016 | -0.85134305 |
| Sec61a1 | 0.07288 | -0.35048115 |
| Baiap2l1 | 0.1753 | -1.70481945 |
| Cep295 | 0.6003 | -0.156131 |
| 4930523C07Rik | 0.13646 | 0.70969508 |
| Pdpr | 0.33534 | 0.32563476 |
| Lmna | 0.00183 | -0.92260173 |
| Ank2 | 0.04727 | -0.68320402 |
| Pdzd4 | 0.00144 | -1.28375735 |
| Rttn | 0.00144 | 2.16937302 |
| Fam199x | 0.01759 | 1.23879304 |
| 1700065I16Rik | 0.00182 | -1.29746368 |
| Slc35d1 | 0.21799 | -0.39615968 |
| Cbx2 | 0.00396 | -0.78568034 |
| Vps4b | 0.00144 | -1.29051721 |
| AI662270 | 0.65168 | -0.09673975 |
| Prpf38a | 0.04353 | 0.58765028 |
| Zim2 | 0.00144 | -1.22925777 |
| Ift74 | 0.02732 | 1.54538676 |
| Nudt7 | 0.29777 | 0.26726683 |
| C920021L13Rik | 0.70056 | 0.14088356 |
| Rgs5 | 0.00373 | 0.99331178 |
| Gm10997 | 0.05573 | -0.85419823 |
| Dtnb | 0.36856 | -0.36160464 |
| Zfp775 | 0.00218 | -1.18878227 |
| Samhd1 | 0.00217 | 1.7949793 |
| Fgfr1 | 0.00173 | -0.96025791 |
| Cox7c | 0.0873 | -0.52954693 |
| Myo18a | 0.00144 | -1.42424195 |
| Creb3 | 0.07321 | -0.33083878 |
| Dopey1 | 0.02493 | 0.70038948 |
| Pikfyve | 0.40419 | -0.37948082 |
| Ndst1 | 0.01523 | -0.77633011 |
| Srrm1 | 0.19483 | 0.73125623 |
| Uck2 | 0.11356 | -0.44190712 |
| Marveld3 | 0.78233 | 0.08058592 |
| Gpr137 | 0.24406 | -0.56896725 |
| Setd5 | 0.02414 | 1.02463588 |
| Tmem128 | 0.53168 | 0.20963679 |
| Tpcn2 | 0.87017 | 0.04644673 |
| Chd8 | 0.07277 | 1.28133013 |
| Ppcdc | 0.07087 | -0.42114057 |
| Wdr20 | 0.01072 | 1.01454252 |
| Slc30a9 | 0.05457 | 0.45288291 |
| Zfp2 | 0.35892 | -0.29911894 |
| 9330182L06Rik | 0.00644 | -1.60967688 |
| Tgoln2 | 0.02247 | -0.53005663 |
| 2010110K18Rik | 0.00152 | -1.25878908 |
| Purg | 0.66802 | -0.13581847 |
| Lsm11 | 0.09493 | -0.49774462 |
| Ceacam10 | 0.00144 | -1.26911831 |
| Mphosph10 | 0.00144 | 2.12361047 |
| Pyurf | 0.94183 | 0.04044893 |
| Cyhr1 | 0.00708 | -0.72541256 |
| Osbpl5 | 0.03211 | -0.53076392 |
| Rtp3 | 0.13523 | -0.5608706 |
| Myct1 | 0.01442 | -0.81701268 |
| Npffr1 | 0.00144 | -1.27165604 |
| Gpr158 | 0.09094 | -2.19196453 |
| BC048502 | 0.00144 | -1.31495282 |
| Plekhm1 | 0.05408 | -0.4317072 |
| Olfr197 | 0.00145 | -1.22193674 |
| Napa | 0.97193 | -0.01992956 |
| Nol9 | 0.52543 | -0.16174794 |
| Zfp943 | 0.00885 | 1.83678878 |
| Acaca | 0.10653 | -0.72862218 |
| 1700086P04Rik | 0.18599 | -0.42681894 |
| Prune | 0.21434 | 0.70534018 |
| Atp9b | 0.58117 | 0.20797522 |
| Hsd17b11 | 0.77175 | -0.05410093 |
| Lrba | 0.04739 | -0.38210096 |
| A230006K03Rik | 0.00144 | -1.28898079 |
| Acap2 | 0.00144 | 1.79511336 |
| Spag5 | 0.00144 | -1.20555712 |
| Bysl | 0.00713 | -0.73101958 |
| Kirrel | 0.03441 | -0.54489125 |
| Rbm7 | 0.05703 | 0.36591683 |
| 4930567H12Rik | 0.00144 | -1.25927895 |
| Nup54 | 0.06446 | -0.70208093 |
| Srprb | 0.69625 | -0.16294745 |
| Ajuba | 0.09463 | -0.49941973 |
| Atg4b | 0.15699 | -0.4855318 |
| Ttc39b | 0.00144 | -1.0206223 |
| Snx9 | 0.05822 | 0.43205468 |
| Camk1d | 0.05634 | -0.92245948 |
| Aldh2 | 0.8071 | -0.04016281 |
| Krtap8-1 | 0.00385 | -1.14134209 |
| Cln3 | 0.16124 | -0.4297795 |
| B4galnt1 | 0.80637 | 0.07043168 |
| Pcdh7 | 0.44766 | -0.15847119 |
| Exph5 | 0.00163 | -1.21015905 |
| Kcna6 | 0.00921 | -1.10403846 |
| Ldoc1l | 0.32376 | -0.38915582 |
| Olfr549 | 0.04014 | -0.56591143 |
| Ankrd42 | 0.2776 | -0.76748357 |
| Atf2 | 0.00751 | 0.85353682 |
| D1Pas1 | 0.00614 | -1.48123622 |
| Ppip5k2 | 0.01158 | 0.96297151 |
| Alas1 | 0.95164 | -0.03398306 |
| Stradb | 0.09535 | 0.52005384 |
| Galm | 0.18387 | -0.66436248 |
| Fhl2 | 0.00144 | -1.31627578 |
| Kctd12b | 0.00144 | 1.33716889 |
| Wdpcp | 0.05229 | 0.51955087 |
| Phactr1 | 0.00993 | -1.06570613 |
| Pitx2 | 0.00144 | -1.51500476 |
| Polb | 0.07537 | 1.00287003 |
| Acap3 | 0.01229 | -0.53451421 |
| 4930548J01Rik | 0.00144 | -1.26911831 |
| Nhej1 | 0.1436 | -0.46602295 |
| Ogfod2 | 0.00817 | -0.90925893 |
| Fam134c | 0.70098 | -0.08555684 |
| Gpr4 | 0.00473 | -0.74679524 |
| Psmd4 | 0.62653 | 0.15412903 |
| Zfp385b | 0.00668 | -0.79789131 |
| Clk4 | 0.07793 | 0.88772767 |
| Slc5a7 | 0.00144 | -1.79695848 |
| Foxd1 | 0.00144 | -1.24466058 |
| Kazn | 0.04892 | -1.25990571 |
| Gm5069 | 0.04389 | -0.50700326 |
| Asb3 | 0.04164 | 0.58798912 |
| Tfec | 0.04253 | -1.00048649 |
| Dagla | 0.06554 | -0.66336246 |
| Elf4 | 0.79081 | 0.07416363 |
| Pigg | 0.0044 | -0.96037072 |
| Ypel1 | 0.0243 | -0.58521389 |
| Caprin1 | 0.00144 | 2.4256912 |
| Vmp1 | 0.49718 | 0.12380766 |
| AI182371 | 0.0478 | -0.88995047 |
| Zfand4 | 0.00144 | -1.29085005 |
| Drc7 | 0.00144 | -1.33632959 |
| Mms22l | 0.52166 | -0.38428383 |
| Gm9 | 0.00144 | -1.26560808 |
| Msr1 | 0.04164 | 0.94006825 |
| Sema5b | 0.04782 | -0.48480886 |
| Tnks | 0.9058 | -0.01906869 |
| Cramp1l | 0.04436 | 0.3728937 |
| Ggt5 | 0.39874 | 0.24683299 |
| Actrt3 | 0.00144 | -1.22925777 |
| Nell1 | 0.00157 | -1.19247859 |
| Ago3 | 0.00144 | 1.33510816 |
| LOC102637001 | 0.00144 | -1.28898079 |
| Prkab2 | 0.18703 | -0.21879527 |
| Bnipl | 0.00144 | -1.28116048 |
| Mtch1 | 0.00242 | -0.99850934 |
| Riok1 | 0.4788 | 0.18457428 |
| Mmrn1 | 0.00144 | -1.27165604 |
| 1700113A16Rik | 0.39576 | -0.66098161 |
| Cnot2 | 0.00144 | 1.36565053 |
| Mogat2 | 0.00144 | -1.26560808 |
| Msmb | 0.89431 | 0.1864758 |
| Rhod | 0.47945 | -0.25412657 |
| Reep2 | 0.04874 | -0.57607833 |
| Rbp2 | 0.00144 | -1.28898079 |
| Olfr123 | 0.00446 | -1.1715245 |
| Usp15 | 0.43711 | -0.16174218 |
| Smc1a | 0.01672 | 0.89640597 |
| Cybrd1 | 0.00447 | -1.07435095 |
| Gm31248 | 0.00149 | -1.22582153 |
| Hipk4 | 0.18245 | -0.84535097 |
| Ace3 | 0.00144 | -1.30713006 |
| Kcnd2 | 0.00433 | 1.24195153 |
| Ifrd1 | 0.00182 | 1.03062172 |
| Khnyn | 0.33798 | 0.15721365 |
| A530076I17Rik | 0.02173 | -1.32044613 |
| Wdr47 | 0.2758 | -0.40941673 |
| C030005K06Rik | 0.00144 | -1.27165604 |
| Zdhhc8 | 0.00208 | -1.26960839 |
| Slc25a36 | 0.21679 | 0.2727001 |
| Mtap7d3 | 0.97364 | 0.0256052 |
| Rnf41 | 0.01197 | -0.6612413 |
| E130304I02Rik | 0.00306 | -1.05733172 |
| A630023P12Rik | 0.00397 | -1.42953488 |
| 1700031F10Rik | 0.00144 | -1.29051721 |
| Tcf25 | 0.13651 | -0.2596963 |
| Dnaaf2 | 0.25071 | -0.23323509 |
| Ptrh2 | 0.70693 | -0.12750027 |
| Smok3b | 0.00144 | -1.28116048 |
| Asxl1 | 0.00196 | -1.3886586 |
| Aldh4a1 | 0.7671 | 0.10795465 |
| Ids | 0.00888 | 0.86041182 |
| Wdfy2 | 0.00416 | -0.84247259 |
| Arl2 | 0.43619 | -0.48735709 |
| Cep41 | 0.79222 | 0.09002944 |
| Dmrta1 | 0.02378 | -1.02865305 |
| Rasgrp4 | 0.80887 | -0.05668578 |
| Il17rd | 0.03288 | -0.5303107 |
| Rtel1 | 0.00801 | -1.31192431 |
| Tmem47 | 0.00501 | 2.98796659 |
| Cyp2j11 | 0.06575 | -1.19752523 |
| Prmt3 | 0.19616 | 0.3433481 |
| 4930504O13Rik | 0.00481 | -1.45193052 |
| Ttc16 | 0.20325 | 0.55194294 |
| 4921529L05Rik | 0.49655 | -0.30564809 |
| Rbm26 | 0.03525 | 1.43206702 |
| Strbp | 0.12344 | 0.47537441 |
| Pllp | 0.00146 | -2.02915428 |
| Msrb3 | 0.10713 | -0.41621041 |
| Kcnn2 | 0.23542 | -0.82934264 |
| Nrxn1 | 0.02363 | 2.00361814 |
| 2610001J05Rik | 0.16862 | -0.29408455 |
| Tmed7 | 0.00144 | -1.25927895 |
| Fbxo22 | 0.01491 | 0.67336916 |
| 4930546O09Rik | 0.00499 | -1.49245118 |
| Osgepl1 | 0.00864 | 0.86210655 |
| Apool | 0.00144 | 1.31461922 |
| Cnot7 | 0.00144 | -0.91975819 |
| Tectb | 0.00652 | 1.12943717 |
| Ankrd1 | 0.22093 | 0.30167926 |
| 4933406K04Rik | 0.00144 | -1.31884045 |
| 0610030E20Rik | 0.87103 | -0.02814775 |
| Rab27b | 0.4954 | -0.19429546 |
| Igfl3 | 0.00144 | -1.30017714 |
| Grap2 | 0.00144 | -1.28116048 |
| Vcp | 0.39184 | 0.22410493 |
| Zfp729a | 0.0065 | 1.53610276 |
| 6030452D12Rik | 0.01157 | -1.02536766 |
| Zfp449 | 0.03353 | 0.7876168 |
| Carm1 | 0.43721 | -0.26744965 |
| Slc35e2 | 0.00195 | 1.30322409 |
| Slc13a5 | 0.00144 | -1.26560808 |
| Gadd45b | 0.02628 | -0.55964239 |
| Gm36401 | 0.00392 | -1.45927262 |
| Slc25a12 | 0.07265 | 1.04395879 |
| Med14 | 0.00325 | -1.15314387 |
| Zhx1 | 0.99113 | -0.0043848 |
| Gm3805 | 0.00144 | -1.44135461 |
| Inpp1 | 0.00144 | -1.52090118 |
| 3110023J12Rik | 0.07364 | -2.5737567 |
| Prom1 | 0.00144 | 1.94548393 |
| Fbxw22 | 0.00819 | 1.15369261 |
| 4732419C18Rik | 0.22976 | -0.56610132 |
| LOC102632383 | 0.00144 | 2.95494638 |
| Gm5065 | 0.46618 | -0.16980154 |
| Med25 | 0.96039 | 0.00942744 |
| Hmgcs1 | 0.44358 | -0.24613586 |
| Ptprc | 0.02583 | -0.61355947 |
| 1700041M19Rik | 0.00144 | -1.41534266 |
| Hs3st4 | 0.20728 | -0.72015489 |
| Jakmip2 | 0.00158 | -1.2446827 |
| Trim5 | 0.00164 | 1.35296692 |
| Gm20110 | 0.1037 | -2.97424435 |
| 9630013D21Rik | 0.00149 | 1.06563492 |
| Senp5 | 0.38291 | 0.1725024 |
| Tnfsfm13 | 0.08438 | -0.35242951 |
| Mroh1 | 0.09645 | -0.71673821 |
| Kdsr | 0.00965 | 1.56280922 |
| Ipmk | 0.01023 | 0.82572286 |
| Bmp5 | 0.00144 | -1.31703013 |
| Opa3 | 0.14721 | -0.5127036 |
| 1700108J01Rik | 0.00144 | -1.28116048 |
| Chn1 | 0.0438 | 0.57424672 |
| Lss | 0.02516 | 1.63651956 |
| Slc6a16 | 0.04742 | -0.99382835 |
| Ddhd2 | 0.00144 | -1.14537619 |
| Gm2137 | 0.13246 | -0.49637443 |
| Rtn4 | 0.00431 | 1.49784913 |
| Gm35066 | 0.00144 | -1.29746368 |
| Acvr2a | 0.32883 | 0.3739973 |
| Selt | 0.04425 | -0.51696985 |
| Gm16294 | 0.00144 | -1.31495282 |
| Fam173b | 0.38511 | -0.25629515 |
| 1700001N15Rik | 0.00145 | -1.22376446 |
| 4930404H11Rik | 0.01123 | -1.04967308 |
| Pde7a | 0.12316 | 0.55100578 |
| Defb19 | 0.6947 | -0.13632386 |
| Casp3 | 0.60685 | -0.12984925 |
| Snx27 | 0.06177 | 0.54175174 |
| Dnajc16 | 0.46625 | 0.31363949 |
| Dnajc16 | 0.31246 | 0.32257453 |
| Anapc1 | 0.16796 | -2.07567247 |
| Tg | 0.60951 | -0.28931508 |
| Tlr6 | 0.5776 | -0.22414178 |
| Gja4 | 0.00144 | -1.12613261 |
| Rbl2 | 0.01823 | 0.54086406 |
| Fryl | 0.0809 | 0.57580953 |
| Klk15 | 0.05772 | -1.11952886 |
| Gm9944 | 0.01269 | -1.14134209 |
| Gm15787 | 0.08155 | -1.41274471 |
| Eml6 | 0.00352 | 1.38957929 |
| Gm12408 | 0.00144 | -1.31884045 |
| 4930528J18Rik | 0.03684 | -1.74814978 |
| Pou4f1 | 0.00158 | -1.2446827 |
| Saxo1os | 0.01579 | -1.1237107 |
| Gm11651 | 0.00144 | -1.28898079 |
| Safb2 | 0.491 | 0.17663219 |
| Zfp663 | 0.00144 | -1.62385054 |
| Pign | 0.26414 | -0.4123464 |
| 8430419L09Rik | 0.90285 | 0.03716773 |
| Slco2a1 | 0.17815 | 0.81462243 |
| Cep126 | 0.9341 | -0.05822227 |
| Gm534 | 0.00144 | -1.28116048 |
| Gm32693 | 0.00144 | -1.29049509 |
| A630010A05Rik | 0.00144 | -1.27165604 |
| Usp4 | 0.70646 | -0.09633128 |
| Gm30289 | 0.18982 | 0.77365004 |
| 4930433E05Rik | 0.00317 | -1.11935153 |
| 1700022E09Rik | 0.2467 | -0.73088169 |
| 4930480K23Rik | 0.77354 | 0.07035242 |
| Gm4117 | 0.00144 | -1.25118919 |
| Adhfe1 | 0.00777 | 1.91807025 |
| Atf2 | 0.22376 | 0.4418523 |
| Aff4 | 0.02991 | 0.59326976 |
| Gm2795 | 0.00144 | -1.28116048 |
| Dusp19 | 0.00614 | 0.98267524 |
| Aldh2 | 0.90285 | 0.04953138 |
| Coro2a | 0.01353 | -1.35815045 |
| Gm35558 | 0.9262 | -0.02137678 |
| Ralgapb | 0.02651 | 0.89012954 |
| 1110019B24Rik | 0.05368 | -0.82613028 |
| Gm31437 | 0.09529 | -0.57395748 |
| Acot3 | 0.55171 | 0.2427986 |
| Zfp458 | 0.00461 | -1.14915299 |
| Fbxo45 | 0.0032 | 1.36102013 |
| Ints12 | 0.00805 | 0.73789847 |
| Spem1 | 0.06247 | -0.82721545 |
| Pcif1 | 0.01103 | -1.04056467 |
| 1700018A04Rik | 0.00321 | -1.55534323 |
| A930006K02Rik | 0.04824 | 0.79033162 |
| Tcerg1l | 0.00144 | -1.28116048 |
| Gm14207 | 0.03935 | -0.56739335 |
| Ifitm10 | 0.01589 | -0.82889466 |
| Upk3b | 0.08412 | -0.76180074 |
| Gm10322 | 0.01472 | -1.71549744 |
| Gm31063 | 0.71748 | -0.33339858 |
| Gm20275 | 0.00145 | -1.25646236 |
| Hltf | 0.00458 | 1.44472647 |
| 4930563M21Rik | 0.12608 | -1.09411165 |
| Zkscan16 | 0.00144 | -1.27165604 |
| Casc5 | 0.00144 | -1.26911831 |
| Dcaf10 | 0.0247 | -0.96581009 |
| Fmo5 | 0.41841 | 0.34593781 |
| 1700069P05Rik | 0.05629 | -1.01017398 |
| Ptpn6 | 0.00477 | -0.88326115 |
| Elmo3 | 0.36107 | 0.16863442 |
| Olfr1467 | 0.00144 | -1.60245164 |
| Olfr1112 | 0.00144 | -1.30017714 |
| Stat6 | 0.30038 | 0.20830442 |
| Rab6b | 0.33824 | -0.18438382 |
| B130006D01Rik | 0.54769 | 0.31632649 |
| Armcx5 | 0.00149 | 1.34473247 |
| C1rb | 0.0805 | -0.34444624 |
| Gm31592 | 0.48797 | -0.52546683 |
| Uxs1 | 0.00733 | -1.14281464 |
| Gm4926 | 0.00144 | -1.29051721 |
| Lrrc24 | 0.0145 | -0.93025735 |
| Gm30759 | 0.02902 | -0.93967261 |
| Gm10914 | 0.62936 | -0.42897943 |
| Taf1a | 0.00149 | -1.22582153 |
| Gpam | 0.62999 | -0.18275789 |
| 4933407G14Rik | 0.00144 | -1.3230041 |
| Fmo2 | 0.63959 | -0.09881979 |
| Dsc1 | 0.00144 | -1.26560808 |
| 1600025M17Rik | 0.07547 | 0.69323518 |
| Agxt2 | 0.00158 | -1.15468698 |
| D830026I12Rik | 0.00839 | 1.73853982 |
| Vrk2 | 0.00295 | -1.39782658 |
| Sfxn1 | 0.61066 | 0.13540692 |
| Chml | 0.00366 | 1.85761115 |
| Ptpn13 | 0.21075 | -0.762531 |
| Scn5a | 0.0892 | -0.49918291 |
| Htt | 0.20294 | 0.51894077 |
| Gm684 | 0.00653 | 0.83689876 |
| Aldh1a3 | 0.00145 | -1.22376446 |
| Poldip3 | 0.00144 | -1.468939 |
| LOC102635566 | 0.03404 | -0.42802838 |
| Tenm1 | 0.00144 | -1.31495282 |
| Hecw1 | 0.00144 | -1.20555712 |
| Gm33509 | 0.00145 | -1.26219879 |
| Hivep3 | 0.39285 | 0.25097974 |
| Dnajc5 | 0.15456 | -0.2719569 |
| Pan2 | 0.30021 | -0.84279299 |
| Trim67 | 0.24011 | 0.41372012 |
| 1110013I04Rik | 0.00149 | -1.22582153 |
| Kctd4 | 0.3639 | -0.31764392 |
| Tex9 | 0.00144 | 2.0616065 |
| Syn3 | 0.87272 | 0.05254072 |
| BC023829 | 0.30134 | -0.29498589 |
| Far1 | 0.00144 | 1.69324668 |
| Commd8 | 0.00144 | 0.97199694 |
| Lman2 | 0.28992 | -0.31687714 |
| Ly6g6e | 0.0507 | -0.88511728 |
| Dda1 | 0.00265 | -0.88342901 |
| Dlx6os1 | 0.00144 | -1.33864445 |
| Gm16386 | 0.00951 | 1.31190971 |
| Gm13111 | 0.02083 | 0.61129106 |
| R3hdm1 | 0.00144 | -1.29085005 |
| Ccdc73 | 0.009 | -0.85948868 |
| 4930563E18Rik | 0.10376 | -2.75840248 |
| Spire1 | 0.03287 | -0.76243904 |
| Gnas | 0.5225 | 0.32726151 |
| Srgap2 | 0.08766 | 0.43998471 |
| Kl | 0.00144 | -1.25048839 |
| Tmem82 | 0.27505 | 0.31946829 |
| 1700065D16Rik | 0.00144 | -1.3348153 |
| 1810030O07Rik | 0.96598 | 0.02003582 |
| Naa50 | 0.22564 | 0.19826301 |
| Nxph2 | 0.06947 | -2.21901925 |
| Kdm2a | 0.43887 | 0.30522043 |
| Hip1 | 0.94362 | 0.01205479 |
| Gtf3c3 | 0.00254 | 0.99668142 |
| Xndc1 | 0.26388 | 0.63922188 |
| Sh3yl1 | 0.00145 | -1.22376446 |
| LOC102642641 | 0.47417 | 0.49144977 |
| Ppp2r5a | 0.00144 | -1.31495282 |
| Ino80e | 0.00166 | -0.92950892 |
| 2310041K03Rik | 0.00144 | -1.28116048 |
| 2410003L11Rik | 0.01787 | -1.80529072 |
| Wdr54 | 0.00144 | -1.29085005 |
| Pcdh17 | 0.00144 | 2.2043258 |
| Ttc23 | 0.00144 | -1.26428512 |
| Gm9796 | 0.00144 | -1.29085005 |
| LOC102637720 | 0.09454 | -2.58625207 |
| Gm35392 | 0.00144 | -1.31495282 |
| Ubap2l | 0.60832 | 0.14291046 |
| Cntnap4 | 0.00206 | -1.471793 |
| A730059M13Rik | 0.49447 | -1.05812207 |
| Pcdhb5 | 0.00144 | -1.29746368 |
| Tomm70a | 0.00144 | -1.44780261 |
| Hmg20a | 0.87804 | 0.14915299 |
| Ttk | 0.00145 | -1.26219879 |
| Phf12 | 0.0116 | 0.78987033 |
| Nelfe | 0.09485 | -0.45169778 |
| Wdr83 | 0.53179 | 0.25648039 |
| 4930505O20Rik | 0.11807 | -0.80610421 |
| Gm35774 | 0.41638 | -0.58460901 |
| Ndufaf3 | 0.33434 | -0.48237299 |
| 5730416F02Rik | 0.00468 | -0.93212862 |
| Rqcd1 | 0.70595 | -0.13799382 |
| Gm34240 | 0.00145 | -1.22376446 |
| Ackr1 | 0.02059 | 0.94330835 |
| 1700125G22Rik | 0.00144 | -1.42632532 |
| Lhx9 | 0.00144 | -1.29085005 |
| 1700016G22Rik | 0.00144 | -1.27730451 |
| Lpgat1 | 0.00334 | 1.52660027 |
| Sdha | 0.00198 | 1.24860629 |
| Gapt | 0.01239 | -1.02834537 |
| Nptx1 | 0.00145 | -1.22193674 |
| Pik3r5 | 0.0521 | -0.41438841 |
| Gpr152 | 0.01263 | -0.87695271 |
| Asb8 | 0.13105 | -0.32260348 |
| E2f5 | 0.00528 | 1.83609282 |
| Irx1 | 0.82419 | -0.13967131 |
| P3h2 | 0.63117 | -0.51186426 |
| Gm33100 | 0.6669 | -0.41950631 |
| LOC105247168 | 0.00144 | -1.30713006 |
| 1500032F14Rik | 0.02728 | -0.8983632 |
| 4930555K05Rik | 0.00158 | -1.2446827 |
| Dcaf7 | 0.04253 | 0.60227029 |
| Hipk3 | 0.05178 | 0.4851449 |
| Rbm44 | 0.00153 | -1.93240252 |
| St8sia1 | 0.3701 | 0.31397303 |
| Clip3 | 0.01178 | -0.56984861 |
| Catsper1 | 0.01381 | -1.03043839 |
| LOC102631912 | 0.00518 | -0.91568357 |
| Gm39334 | 0.36691 | 0.25264052 |
| Gm39559 | 0.12352 | -0.53363468 |
| Ift57 | 0.97216 | 0.05188026 |
| Glis2 | 0.00634 | -0.74598289 |
| Tenm1 | 0.00144 | -1.29746368 |
| Tspear | 0.00254 | -1.66506099 |
| Gm16124 | 0.71848 | -0.07094232 |
| Zeb1 | 0.00394 | 0.8727498 |
| Gm26697 | 0.00144 | -1.30783085 |
| Myc | 0.13355 | 0.68385943 |
| 1700011B04Rik | 0.00144 | -1.27699683 |
| Pde1c | 0.02052 | 1.46460098 |
| Mettl14 | 0.00144 | -1.29878664 |
| BC030336 | 0.18416 | -0.23477331 |
| D430042O09Rik | 0.99197 | 0.0017691 |
| B230369F24Rik | 0.04003 | 0.69829461 |
| Rab2b | 0.10682 | -0.43486942 |
| Lman1 | 0.08108 | 0.41973933 |
| LOC105244251 | 0.60425 | 0.21529358 |
| Exoc4 | 0.29261 | -1.85334153 |
| Gm39090 | 0.02078 | 1.33864718 |
| Rbm4b | 0.00144 | 1.67175635 |
| Gm38532 | 0.15167 | -0.49403235 |
| Ndufs5 | 0.07417 | -1.14308831 |
| Gm35438 | 0.02008 | 1.54870744 |
| Gm19303 | 0.00144 | -1.33632959 |
| Rsrc1 | 0.04586 | -0.63766952 |
| 4930507D10Rik | 0.24908 | -0.74422025 |
| 1700039M10Rik | 0.01428 | -1.83659167 |
| Sel1l | 0.6886 | -0.23530663 |
| Map6 | 0.2561 | -0.20567365 |
| Prpf39 | 0.00144 | 3.36093079 |
| Fam81a | 0.01033 | 0.62165191 |
| Gm11744 | 0.00237 | -0.92429227 |
| Dmxl1 | 0.04912 | 0.79237235 |
| Gm16010 | 0.00144 | -1.26560808 |
| Gm14902 | 0.53953 | -0.31035397 |
| Gm11217 | 0.03206 | -1.96426526 |
| Tnfrsf11a | 0.52642 | -0.31453639 |
| Hist1h1d | 0.00156 | -1.20538087 |
| Mtf2 | 0.02798 | 0.7489474 |
| Rbm18 | 0.10697 | 0.31571951 |
| Mkl2 | 0.13236 | 0.97587948 |
| Pmp22 | 0.13613 | -0.7860182 |
| 4930474M22Rik | 0.00777 | -1.05183729 |
| Gm13427 | 0.00144 | -1.27165604 |
| Ddx46 | 0.00457 | 0.88125064 |
| Tnfrsf11b | 0.1387 | -1.93821873 |
| Zfp352 | 0.00276 | -1.14169705 |
| Olfr1295 | 0.13544 | -0.78795495 |
| C79468 | 0.02978 | 0.57186849 |
| Jak1 | 0.0332 | 0.51794639 |
| AU019823 | 0.0447 | 0.45228934 |
| Cdc14b | 0.04155 | 0.58958656 |
| Egfr | 0.15678 | 0.57773129 |
| Gm11978 | 0.0331 | -1.98366888 |
| Pgk1 | 0.00432 | -1.35825174 |
| Baz1a | 0.00144 | 1.72745774 |
| Dpp7 | 0.04566 | -0.77073729 |
| Sh3gl2 | 0.00144 | -1.28116048 |
| Gm17739 | 0.00223 | 1.43644269 |
| 4930511A02Rik | 0.00144 | -2.32861398 |
| Gm26688 | 0.00368 | -1.10379914 |
| Fnbp1 | 0.03906 | -0.66022465 |
| Cdc42bpg | 0.50173 | -0.32792034 |
| B2m | 0.21285 | 0.46016153 |
| Asah2 | 0.01505 | 0.885507 |
| Mettl4 | 0.00145 | -1.25646236 |
| Dgcr8 | 0.20911 | -0.75657293 |
| 5830417I10Rik | 0.00144 | 1.15426394 |
| Gm13605 | 0.12119 | -0.7602697 |
| Gm39926 | 0.90418 | 0.0972534 |
| Gm29638 | 0.00832 | -1.17281396 |
| Chrac1 | 0.19765 | -0.72382946 |
| 6330403A02Rik | 0.72906 | 0.14183881 |
| Nudcd3 | 0.68577 | 0.16448124 |
| 4930473M17Rik | 0.00144 | -1.26934956 |
| 4930430J02Rik | 0.03775 | -0.93497606 |
| Frs2 | 0.01085 | 0.62472281 |
| Slc25a4 | 0.87018 | 0.05159094 |
| 5830428M24Rik | 0.00144 | -1.28116048 |
| Lrp8os1 | 0.81757 | -0.1204385 |
| Arl5b | 0.01241 | -0.71279313 |
| Diap3 | 0.34254 | 0.43544455 |
| Fmnl3 | 0.00203 | -0.87106075 |
| Rlim | 0.00959 | 0.76756219 |
| Fam114a2 | 0.07701 | -0.29873378 |
| Tmem52b | 0.53745 | 0.31544718 |
| Lipt1 | 0.82655 | 0.06166633 |
| Gm11237 | 0.44358 | -0.23200366 |
| Grin2b | 0.45825 | -0.1949875 |
| Gm36017 | 0.03736 | -2.04253344 |
| Gtdc1 | 0.91653 | -0.04256516 |
| Gm26604 | 0.00144 | -1.29746368 |
| Gm33120 | 0.00145 | -1.2551153 |
| Gm30307 | 0.19803 | 0.86509833 |
| Nkain2 | 0.00144 | -1.29049509 |
| Akap5 | 0.00385 | 2.0693489 |
| Shc2 | 0.03238 | -0.45530213 |
| Frem1 | 0.00144 | -1.24900642 |
| Htr1b | 0.00144 | -1.23532597 |
| Ints8 | 0.00639 | 1.15380044 |
| Olfr1361 | 0.00144 | -1.35320495 |
| Gm5142 | 0.00144 | -1.29085005 |
| Gm29601 | 0.00144 | -1.22925777 |
| Usp15 | 0.07937 | 0.87460646 |
| Btbd10 | 0.03733 | -0.60106197 |
| LOC102637129 | 0.00499 | -0.78665406 |
| Tip3 | 0.00144 | -1.27157815 |
| Alad | 0.87315 | 0.0821627 |
| Flg2 | 0.00144 | -1.25118919 |
| Dpy19l1 | 0.21802 | -0.33681579 |
| Fam124b | 0.00741 | -1.03531684 |
| Tfdp1 | 0.08816 | 0.43474388 |
| 4930518J21Rik | 0.00144 | -1.27730451 |
| 4930474H20Rik | 0.00144 | -1.26560808 |
| Rpl13 | 0.354 | 0.32458797 |
| 5430434G16Rik | 0.00144 | 2.35798373 |
| B4galt1 | 0.01394 | -0.57410034 |
| Zfp729b | 0.00491 | 1.62508152 |
| AU022840 | 0.00144 | -1.37434134 |
| Ncoa3 | 0.0592 | -0.38007068 |
| Lasp1 | 0.01652 | -0.53921268 |
| Gimap5 | 0.00589 | -0.80278983 |
| Itgav | 0.04166 | -1.76015383 |
| LOC102640619 | 0.00144 | -1.49155079 |
| Mageb16-ps1 | 0.00144 | -1.30017714 |
| Galnt10 | 0.03172 | -1.03062302 |
| LOC105242767 | 0.00741 | -1.10980439 |
| Dync2h1 | 0.02401 | 0.52862865 |
| Lmtk3 | 0.06958 | -1.25360411 |
| 4930440C22Rik | 0.00209 | -1.83026526 |
| D030025P21Rik | 0.00144 | -1.26560808 |
| Hsf5 | 0.00145 | -1.26219879 |
| Tbc1d8b | 0.00144 | 2.20860619 |
| Ecscr | 0.01813 | -0.5576646 |
| Mark3 | 0.00281 | 1.96686919 |
| Trmt1 | 0.00565 | -0.7066133 |
| Dars2 | 0.1135 | 0.41921516 |
| Psmc4 | 0.12072 | -0.38266864 |
| Dsg1c | 0.00266 | -1.34922308 |
| Ccdc151 | 0.00144 | -1.28116048 |
| Pcnx | 0.00999 | -0.98292561 |
| Ntm | 0.69031 | -0.20896642 |
| AW112010 | 0.32943 | -0.31467802 |
| Adam32 | 0.00144 | -1.28116048 |
| Ryr2 | 0.01963 | 0.60986393 |
| 1700049G17Rik | 0.87612 | 0.04710841 |
| Srrd | 0.05047 | -0.57108313 |
| Hils1 | 0.00946 | -1.87782424 |
| Tmem129 | 0.97232 | 0.01347998 |
| Panct2 | 0.00144 | -1.25118919 |
| Senp2 | 0.33346 | 0.22936138 |
| C030014O09Rik | 0.82502 | 0.08973007 |
| Sparc | 0.04014 | -0.43976981 |
| LOC102642336 | 0.00144 | -1.28898079 |
| Pstpip2 | 0.02143 | -1.42772699 |
| Cad | 0.16 | -0.93692675 |
| Evi5l | 0.05171 | -2.05812364 |
| Tmem130 | 0.19518 | -0.85261546 |
| Tnfrsf10b | 0.05468 | -0.81964301 |
| Panx1 | 0.6786 | 0.25422722 |
| Mtx2 | 0.82484 | -0.09100616 |
| Fbxl21 | 0.00229 | -1.13943754 |
| Gm19648 | 0.92399 | -0.05721656 |
| B230354K17Rik | 0.01776 | 1.06794742 |
| Ago2 | 0.09099 | -0.3387073 |
| Mylk4 | 0.00144 | 1.34846979 |
| Fgf14 | 0.1362 | -0.955745 |
| Kantr | 0.00144 | 2.94277174 |
| Il1a | 0.00144 | -1.57415534 |
| Celf2 | 0.40966 | 0.19492837 |
| Pou3f3 | 0.00144 | -1.26428512 |
| Oosp3 | 0.00144 | -1.30017714 |
| Rgs13 | 0.02228 | -0.67491555 |
| Unc13b | 0.33247 | 0.28837435 |
| 0610009O20Rik | 0.33187 | 0.38029972 |
| Epha5 | 0.00145 | -1.22376446 |
| BC065397 | 0.24599 | -0.39169805 |
| Gm2109 | 0.00144 | -1.28898079 |
| Gm29679 | 0.00449 | -1.11367897 |
| Tbx3os2 | 0.00145 | -1.27532353 |
| Gm36879 | 0.00144 | -1.31884045 |
| Akr1b3 | 0.12604 | -0.44066513 |
| Zbtb42 | 0.17633 | -0.33022843 |
| Asf1a | 0.00144 | 2.74788216 |
| BC017643 | 0.00144 | -1.29085005 |
| Cypt15 | 0.00145 | -1.25646236 |
| Zfp334 | 0.17115 | -0.58474236 |
| 5830473C10Rik | 0.0019 | -1.17487561 |
| 4930557B21Rik | 0.22918 | -0.53412652 |
| 4930526I15Rik | 0.00144 | -1.079012 |
| 5430427M07Rik | 0.00144 | -1.29878664 |
| Ftsj2 | 0.88788 | -0.08330901 |
| Abhd10 | 0.06283 | 0.7272954 |
| Krt12 | 0.40187 | -0.50630621 |
| Slc25a27 | 0.02159 | 1.12614504 |
| Tnpo3 | 0.18676 | -0.74898367 |
| Rps24 | 0.00164 | 2.27048022 |
| Nudt13 | 0.88923 | -0.03576617 |
| Olfr1512 | 0.00145 | -1.24191372 |
| Klhl13 | 0.0043 | 1.14720246 |
| Rassf7 | 0.28751 | -0.2434289 |
| Hif1an | 0.84109 | -0.05684048 |
| Fbxw27 | 0.0021 | -1.17763327 |
| Ncoa6 | 0.01354 | 0.51760746 |
| Cfap36 | 0.83279 | -0.0564894 |
| 2210406O10Rik | 0.00144 | -1.21977358 |
| Ly6k | 0.28857 | -0.66097966 |
| Fam63a | 0.00308 | -1.34571643 |
| Crtc2 | 0.0198 | -0.51770428 |
| Bcar3 | 0.01242 | 0.59113801 |
| Ccdc97 | 0.0164 | -0.65685561 |
| Slc4a3 | 0.16978 | -0.4850962 |
| Gm12060 | 0.94256 | -0.01413127 |
| Sfrp2 | 0.08461 | -0.49578493 |
| Nupl1 | 0.00476 | -1.10566328 |
| Ptger3 | 0.00144 | -1.28898079 |
| Shisa8 | 0.04198 | -2.09552995 |
| Dlgap1 | 0.00144 | -1.29085005 |
| Mslnl | 0.94032 | 0.03020085 |
| Pnlip | 0.00145 | -1.22376446 |
| Sox30 | 0.00144 | -1.26934956 |
| Rabl6 | 0.00144 | -1.24966775 |
| Atp1b3 | 0.0061 | 0.69839553 |
| Alox12 | 0.64828 | 0.21184581 |
| A030005K14Rik | 0.05982 | -1.01426341 |
| Gpr35 | 0.00158 | -1.2446827 |
| Cntn1 | 0.00144 | -1.28898079 |
| Ccpg1os | 0.23698 | 0.37982657 |
| Adprhl2 | 0.01493 | -0.69236405 |
| Gmeb1 | 0.00312 | -0.75731661 |
| Rsbn1l | 0.00144 | 1.31198432 |
| Cherp | 0.06663 | -0.38882779 |
| Dock6 | 0.05582 | -0.98177666 |
| Sult1c1 | 0.45967 | 0.15778599 |
| Rusc1 | 0.0068 | -0.81496752 |
| Mical3 | 0.00573 | -0.69489202 |
| Olfr661 | 0.13228 | 0.74503603 |
| Il17rc | 0.08495 | -0.59171958 |
| 1600002K03Rik | 0.00173 | -0.82148305 |
| Eqtn | 0.07625 | -2.26090105 |
| 4930524B15Rik | 0.02862 | -1.03550528 |
| P2rx5 | 0.7553 | 0.10478634 |
| Fam83f | 0.11078 | -0.32709776 |
| Ryr3 | 0.00144 | -1.28116048 |
| Il17f | 0.11799 | -1.19010371 |
| Ndufb7 | 0.00144 | -1.11439049 |
| Nupr1 | 0.57777 | -0.09259342 |
| Dzank1 | 0.01426 | 1.71867568 |
| D430041D05Rik | 0.00144 | -1.23155796 |
| Nutm1 | 0.00158 | -1.2446827 |
| Mkx | 0.00174 | -1.22592762 |
| Cdh7 | 0.00144 | -1.20228444 |
| Ccin | 0.01741 | 0.49904529 |
| Fev | 0.75112 | 0.40933202 |
| Acsl6 | 0.00215 | 1.57311644 |
| Kif14 | 0.00144 | -1.32599123 |
| Bmpr2 | 0.00171 | 1.57294112 |
| Hist1h3a | 0.0125 | -1.32064399 |
| Atp6v1c2 | 0.00144 | -1.28116048 |
| Olfr944 | 0.00744 | -1.03871255 |
| Dpy19l1 | 0.43669 | 0.20118216 |
| Atpif1 | 0.03698 | -0.41934716 |
| Rnf187 | 0.00485 | -0.83453683 |
| Rrp9 | 0.00891 | -0.64200393 |
| Tomm20l | 0.00145 | -1.22376446 |
| Actrt1 | 0.00144 | -1.31627578 |
| Dusp4 | 0.35851 | -0.35151475 |
| Slc36a3 | 0.00144 | -1.28898079 |
| Nfatc1 | 0.00148 | -0.96577716 |
| Glipr2 | 0.63563 | -0.09654742 |
| Thumpd2 | 0.80291 | 0.05500118 |
| Madcam1 | 0.10413 | -0.85340528 |
| Nos1ap | 0.00144 | -1.52889892 |
| Fgf11 | 0.15964 | -0.47676119 |
| Ly6f | 0.03506 | -0.9858148 |
| Fntb | 0.00338 | -1.10758075 |
| Rnf152 | 0.09854 | 0.73048181 |
| Plet1os | 0.67716 | 0.11515164 |
| 2010106E10Rik | 0.00585 | -1.43037719 |
| Tmem169 | 0.00144 | -1.31627578 |
| Cox8a | 0.00503 | -0.98550323 |
| Cd1d1 | 0.00413 | 0.75382093 |
| Cers2 | 0.93473 | 0.01553716 |
| Wbscr28 | 0.02019 | -1.0408731 |
| Thada | 0.1427 | 0.46181654 |
| Grb14 | 0.34962 | -0.14840384 |
| Pla2g16 | 0.0552 | 1.2037396 |
| Tmem43 | 0.05699 | -0.34706851 |
| Zfp930 | 0.00144 | 2.43630798 |
| Casp8ap2 | 0.00144 | 1.71228299 |
| Sh3gl1 | 0.02729 | -0.46190636 |
| Serpinf1 | 0.00452 | -0.71779306 |
| Gatsl3 | 0.26569 | -0.18619053 |
| BC024929 | 0.00144 | -1.28116048 |
| Tysnd1 | 0.0717 | -0.54444727 |
| D130040H23Rik | 0.00177 | -1.15847607 |
| Stk11ip | 0.22075 | -0.28995098 |
| Itm2a | 0.09763 | 0.33260007 |
| Rapgef5 | 0.00419 | 1.1685879 |
| Rbbp5 | 0.03482 | 0.52013225 |
| Igf2 | 0.11238 | -0.48505321 |
| Snx4 | 0.01571 | 0.56909084 |
| Ern1 | 0.00398 | -1.02366692 |
| Bmp5 | 0.96116 | -0.02953131 |
| Gata2 | 0.07594 | -0.45257044 |
| Ndufb10 | 0.00271 | -0.89720551 |
| Aprt | 0.00144 | -1.23532597 |
| Vps11 | 0.09566 | -0.35992517 |
| Kctd4 | 0.24679 | 0.44334293 |
| Cldn34d | 0.03166 | -0.92187831 |
| Trpc4 | 0.35022 | 0.71415591 |
| Hist1h1c | 0.18161 | -0.45426345 |
| Olfr1148 | 0.99963 | 0.00029489 |
| Dntt | 0.41632 | -0.68261287 |
| Olfr57 | 0.00145 | -1.22376446 |
| Sprr2g | 0.26635 | 0.26707514 |
| Galnt11 | 0.36265 | 0.14729832 |
| Ckap4 | 0.01684 | -0.72190586 |
| Fndc3b | 0.00204 | -1.2031737 |
| Zfp516 | 0.02286 | -1.75492246 |
| Pcdhb22 | 0.78097 | 0.05384703 |
| Qpctl | 0.0139 | -0.60830618 |
| Col3a1 | 0.02259 | -0.4948038 |
| Tmem200a | 0.09635 | -0.70551403 |
| Rbm17 | 0.00988 | 0.72477252 |
| Olfr516 | 0.00145 | -1.2388121 |
| Fkbpl | 0.00589 | -0.85381006 |
| Strip2 | 0.00543 | 0.6539634 |
| Zkscan17 | 0.0181 | -0.64965507 |
| Hs3st3a1 | 0.23786 | 0.84003419 |
| Tiparp | 0.00192 | 1.45281382 |
| 4933402J10Rik | 0.00319 | -1.64309152 |
| Ccar2 | 0.00208 | -0.90416445 |
| Olfr446 | 0.62619 | -0.48208567 |
| Parp14 | 0.00869 | 1.12100582 |
| Spc25 | 0.72159 | -0.61214346 |
| Rnf146 | 0.02018 | 0.53684629 |
| Zcchc10 | 0.03303 | 0.51697208 |
| Srp19 | 0.22323 | -0.24079191 |
| Olfr1346 | 0.00144 | -1.28116048 |
| Gm12504 | 0.11091 | 0.78980931 |
| Nr5a2 | 0.0092 | 1.96162384 |
| Gsk3b | 0.20897 | -0.25622975 |
| Slc2a5 | 0.89323 | -0.07673395 |
| Slc13a4 | 0.39589 | 0.26693985 |
| Add2 | 0.24398 | -0.35610665 |
| Pax4 | 0.05601 | -2.59072202 |
| Lrrc2 | 0.00144 | 1.92172738 |
| Prdm16 | 0.00321 | -0.89753276 |
| Mx2 | 0.08308 | 0.64278866 |
| Cma1 | 0.03055 | -1.07754225 |
| Spag4 | 0.38072 | -0.33429457 |
| Rsl24d1 | 0.06628 | 0.68450001 |
| Csde1 | 0.09039 | -0.4474938 |
| Ctcf | 0.01069 | 0.6577869 |
| Cdkn2c | 0.09084 | -0.80414796 |
| Cdkn2c | 0.0464 | -0.47517993 |
| Ankrd55 | 0.00613 | -1.42018892 |
| Nuf2 | 0.07283 | 0.96499293 |
| Slc4a8 | 0.01055 | 0.84781359 |
| Olfr497 | 0.04824 | -0.86696801 |
| Stx11 | 0.77085 | 0.38709056 |
| Spag5 | 0.42798 | 0.47885689 |
| Gosr1 | 0.06307 | -0.47503382 |
| Cfhr1 | 0.00144 | -1.31884045 |
| Pogz | 0.1755 | 0.23604308 |
| D630003M21Rik | 0.14498 | -0.77195265 |
| Azi2 | 0.00404 | 0.95641137 |
| Rxrg | 0.09381 | -0.48103491 |
| Zfp949 | 0.02644 | 0.45312274 |
| Tra2a | 0.00144 | 1.7719239 |
| Pnmt | 0.69058 | 0.124567 |
| Gins4 | 0.15688 | 0.35640571 |
| Rdh13 | 0.89584 | -0.04090787 |
| B630019K06Rik | 0.27206 | 0.5028541 |
| Trps1 | 0.0076 | 1.08102487 |
| Rad9b | 0.00976 | 0.91996273 |
| Ntng1 | 0.00733 | -1.14281464 |
| Entpd3 | 0.0934 | -0.75458023 |
| Ppil1 | 0.05417 | -0.46543757 |
| Cilp2 | 0.08422 | -1.58766467 |
| Hace1 | 0.00144 | 1.99914482 |
| Dera | 0.43062 | 0.21124502 |
| Rmdn1 | 0.01262 | 0.72168445 |
| 4930478L05Rik | 0.00194 | -1.68082505 |
| Syngr4 | 0.1809 | -0.37272668 |
| Cdc42se1 | 0.01897 | -0.54218845 |
| Zfp68 | 0.00144 | 1.71278435 |
| Sdr39u1 | 0.56516 | -0.252579 |
| Gm4788 | 0.01147 | -1.5191379 |
| Bpnt1 | 0.13575 | -0.30152061 |
| Vmn1r51 | 0.00353 | -1.06721129 |
| Kirrel2 | 0.00144 | -1.30713006 |
| Tet1 | 0.14983 | 0.23920071 |
| AF067063 | 0.00145 | -1.21424148 |
| 1600015I10Rik | 0.88716 | 0.03346604 |
| Rer1 | 0.40393 | -0.14532663 |
| Setd7 | 0.01591 | 0.72308484 |
| Mthfsd | 0.00407 | -0.68930802 |
| Gm3696 | 0.01102 | 1.2739576 |
| Snip1 | 0.16452 | 0.377897 |
| Ntpcr | 0.26387 | -0.32715414 |
| Ttc38 | 0.70512 | -0.16005336 |
| Mtag2 | 0.00145 | -1.26219879 |
| Morc2b | 0.17008 | 0.51311508 |
| Stk33 | 0.77917 | 0.11362637 |
| Ift57 | 0.00144 | 1.361137 |
| Lrrc72 | 0.00927 | -1.29323526 |
| D3Ertd254e | 0.16344 | 0.71586858 |
| Ranbp2 | 0.00172 | 1.64132819 |
| Pstpip1 | 0.15815 | -0.4322637 |
| Kcnj9 | 0.05487 | -1.22414178 |
| Fam46b | 0.02025 | 0.60003951 |
| Tmem82 | 0.00144 | -1.29051721 |
| 4930558J22Rik | 0.00144 | -1.27165604 |
| Abca14 | 0.44689 | -0.44572469 |
| Csgalnact2 | 0.00144 | 1.57087947 |
| Fzd9 | 0.0054 | -0.94556172 |
| Cwh43 | 0.06775 | -0.53912799 |
| Atp7b | 0.0137 | -0.73853742 |
| Ap2m1 | 0.86365 | -0.05703109 |
| Chchd7 | 0.71748 | 0.1343994 |
| Gm14295 | 0.00144 | 1.42080385 |
| Serpini2 | 0.00144 | -1.29746368 |
| Afp | 0.00144 | -1.30713006 |
| Adam12 | 0.00694 | -1.1634164 |
| Tmem50b | 0.1811 | -0.25443354 |
| Agmo | 0.00574 | 0.90285106 |
| Rita1 | 0.12039 | -0.6022739 |
| Isg20 | 0.11018 | -0.38023872 |
| Supt3 | 0.7124 | -0.15908533 |
| Meiob | 0.30163 | 0.24780374 |
| Bloc1s6 | 0.00457 | -0.99410426 |
| Slc25a15 | 0.12424 | -0.8430943 |
| Aldh1b1 | 0.16651 | 0.90081918 |
| Lrch3 | 0.0599 | -0.40714303 |
| Lcn2 | 0.71378 | -0.11721843 |
| Lce1d | 0.00144 | -1.27730451 |
| Prl8a9 | 0.00144 | -1.27699683 |
| Cox6a2 | 0.00144 | -0.9675508 |
| Plekho1 | 0.00144 | -1.09884288 |
| Slc5a10 | 0.11421 | -0.55688771 |
| Psmc6 | 0.00144 | 1.80777209 |
| A330033J07Rik | 0.02836 | -1.75777646 |
| Actr1a | 0.00878 | -0.69555082 |
| Gm21379 | 0.0614 | -0.74605325 |
| Uap1l1 | 0.00159 | -1.03739259 |
| Snca | 0.48029 | 0.27280957 |
| Dnmbp | 0.00356 | 1.42084652 |
| Tspan4 | 0.00144 | -1.02937023 |
| Ccl4 | 0.02017 | -1.85064711 |
| Scn1a | 0.03663 | -0.91478561 |
| Ralgds | 0.00169 | -1.0432102 |
| Tmlhe | 0.00144 | 2.95356621 |
| 5430440P10Rik | 0.01129 | 1.23729154 |
| Ppp1ca | 0.18608 | -0.46394178 |
| Uvssa | 0.09468 | 0.84464212 |
| Hoxa7 | 0.00874 | -1.20729862 |
| Rnf34 | 0.13769 | 0.4357296 |
| U2af1l4 | 0.20253 | -0.44216431 |
| Hrk | 0.14746 | -0.82089324 |
| Galr3 | 0.6197 | -0.19792494 |
| Asah1 | 0.00248 | 1.03331478 |
| Mrps21 | 0.48584 | -0.24990405 |
| Prr15 | 0.00581 | -0.97930687 |
| Kiss1r | 0.10085 | -0.4045689 |
| Tas1r1 | 0.26498 | -0.43800924 |
| Ep400 | 0.05018 | -0.83158823 |
| Kcne4 | 0.75487 | -0.08899455 |
| Best1 | 0.19277 | -0.24717944 |
| Mmp17 | 0.01525 | -0.86849442 |
| Zfp879 | 0.72942 | -0.13820295 |
| Tcf15 | 0.00802 | -0.89715818 |
| Sprr1b | 0.03669 | -0.94779588 |
| Olfr103 | 0.00144 | -2.0469606 |
| St13 | 0.03453 | 0.54433539 |
| Cdc16 | 0.12945 | 0.28237513 |
| Kcnn3 | 0.10015 | -0.88324069 |
| Ppm1n | 0.00144 | -1.28116048 |
| Dscr3 | 0.86652 | -0.03018107 |
| Cyp11b2 | 0.00145 | -1.26219879 |
| Cryga | 0.84377 | -0.08495155 |
| Trank1 | 0.9189 | -0.07347822 |
| Zfp142 | 0.22113 | -0.31041238 |
| Brf2 | 0.0056 | -0.69923422 |
| Atp13a2 | 0.00186 | -0.94621157 |
| Ttc8 | 0.0046 | 0.96492767 |
| Gcc1 | 0.50218 | 0.10312855 |
| Pik3cg | 0.49489 | 0.21560586 |
| F13a1 | 0.00421 | 1.18641619 |
| Tubgcp6 | 0.00587 | -0.90239692 |
| Sergef | 0.03423 | -0.66951256 |
| Sdr42e1 | 0.18433 | 0.24521982 |
| Clcn3 | 0.00144 | -1.30713006 |
| Neu4 | 0.00144 | -1.29085005 |
| Itpkc | 0.08036 | -0.43127226 |
| Gimap1 | 0.01588 | -0.72876673 |
| Arf3 | 0.83439 | -0.04208848 |
| Hmgcr | 0.35914 | 0.17452701 |
| Hdac1 | 0.06874 | 0.31599643 |
| Kcnc2 | 0.17762 | 0.54018375 |
| Tmx3 | 0.07975 | 0.32070112 |
| Klf5 | 0.27507 | -0.38330512 |
| Patl1 | 0.02607 | 0.55249194 |
| 1700087M22Rik | 0.01447 | -1.20042684 |
| Fosl2 | 0.01019 | -1.38896326 |
| 4930557K07Rik | 0.00144 | -1.30713006 |
| 2310022A10Rik | 0.03668 | -0.70057849 |
| Slc38a1 | 0.13361 | -0.3990906 |
| Bhlhe22 | 0.31373 | -0.23599551 |
| Nt5dc3 | 0.17028 | -0.38879994 |
| Cldn9 | 0.78667 | -0.13384576 |
| Mrps12 | 0.03081 | -0.73428379 |
| Ugt8a | 0.00145 | -1.22376446 |
| Tep1 | 0.02797 | -0.42276461 |
| Arhgap35 | 0.01286 | -0.65374228 |
| P2rx7 | 0.88053 | -0.14527138 |
| Tdrkh | 0.0029 | 1.62669666 |
| Fbxw7 | 0.49195 | 0.19782585 |
| Rpp38 | 0.03319 | 0.43351342 |
| Vmn1r83 | 0.00144 | -1.28116048 |
| Olfr1022 | 0.0028 | -1.16569658 |
| Edil3 | 0.00144 | -1.27165604 |
| Aldh3b1 | 0.00144 | -1.22636149 |
| Cyp2j6 | 0.0378 | 0.70996763 |
| Cdkn2aipnl | 0.01241 | -0.53259398 |
| Hrg | 0.76303 | 0.10491086 |
| Cst9 | 0.00145 | -1.2388121 |
| Mrpl55 | 0.0017 | -1.19488313 |
| Cckbr | 0.00144 | -1.52584318 |
| Zfp28 | 0.02093 | 0.68961133 |
| Msgn1 | 0.01096 | -1.88534853 |
| Mrpl47 | 0.00144 | -1.24811894 |
| Fbxo44 | 0.7773 | 0.1993629 |
| Prss45 | 0.21341 | -0.81156534 |
| Tcl1b2 | 0.18996 | -1.46508636 |
| Endod1 | 0.75213 | -0.06409054 |
| Tapbpl | 0.09045 | -0.37187514 |
| Inpp5k | 0.02003 | -0.45209493 |
| Il18r1 | 0.05373 | -1.33818964 |
| Elovl5 | 0.09824 | 0.38811354 |
| Bcl7b | 0.67012 | 0.20286038 |
| Mpnd | 0.00171 | -0.9383281 |
| Gkn1 | 0.11511 | -2.1823501 |
| Gtpbp8 | 0.14125 | 0.42872766 |
| Tcf19 | 0.06085 | -0.4972504 |
| Rsad2 | 0.40324 | -0.2343921 |
| Slfn9 | 0.1371 | 1.46043761 |
| Tff1 | 0.05888 | -1.42698912 |
| Cdk18 | 0.88725 | -0.04012368 |
| Stfa3 | 0.58477 | -0.51432433 |
| Olfr1248 | 0.00144 | -1.29085005 |
| Olfr1416 | 0.00144 | -1.26934956 |
| Mef2d | 0.01977 | -0.71386143 |
| Map4k3 | 0.00153 | 1.31622017 |
| Tm2d2 | 0.81895 | -0.08841089 |
| Derl1 | 0.06393 | -0.64481454 |
| Dll3 | 0.49431 | -0.51500476 |
| Rbm34 | 0.00144 | 1.4593701 |
| Sf3a1 | 0.0441 | -0.52775824 |
| C030047K22Rik | 0.00144 | -1.29085005 |
| Nrarp | 0.03173 | -0.58680279 |
| Entpd1 | 0.03506 | 0.43502203 |
| Prpf38b | 0.00144 | 1.37623767 |
| Olfr491 | 0.03099 | -1.96426526 |
| Magix | 0.00305 | -1.01019461 |
| 4933402E13Rik | 0.00144 | -1.26560808 |
| Atp11a | 0.60143 | -0.19811469 |
| Nuak2 | 0.92503 | -0.01960094 |
| Spa17 | 0.91298 | 0.02069201 |
| Gm35496 | 0.00144 | -1.57547831 |
| Bzrap1 | 0.02642 | -1.0338381 |
| Pycr1 | 0.34134 | 0.4236225 |
| Plekhg4 | 0.59222 | -0.56804473 |
| Bptf | 0.00204 | 1.08857312 |
| Edem1 | 0.10178 | 0.44549389 |
| Slitrk6 | 0.0132 | 1.71432975 |
| Lair1 | 0.00907 | -0.78625266 |
| Iglv1 | 0.21206 | -0.81895256 |
| Tex19.2 | 0.93217 | -0.04834094 |
| Elavl4 | 0.00144 | -1.28116048 |
| Gsta3 | 0.27209 | 0.33784937 |
| Zfp414 | 0.00466 | -0.69172765 |
| Arc | 0.16297 | -0.68147355 |
| M1ap | 0.06504 | -0.54427627 |
| C1galt1c1 | 0.00144 | 1.53384391 |
| Lrrc8c | 0.89375 | -0.02308312 |
| Iqcf5 | 0.00145 | -1.2388121 |
| Rbm15b | 0.00144 | -1.561436 |
| Wnt11 | 0.05227 | -0.51493417 |
| Nr2e1 | 0.0481 | -1.36593268 |
| Ift88 | 0.32456 | 0.2721162 |
| Ankrd24 | 0.01049 | -0.87401134 |
| Tmem63a | 0.00144 | -0.99078048 |
| Nfic | 0.00144 | -1.54463387 |
| Ncoa1 | 0.29235 | -0.17451009 |
| 2200002D01Rik | 0.00144 | -1.10540459 |
| Dgkh | 0.00144 | 2.99496079 |
| Ube2h | 0.28993 | 0.25521402 |
| Nxpe4 | 0.0041 | 1.158786 |
| Hdac10 | 0.02896 | -0.41278638 |
| Olfr875 | 0.00144 | -1.30713006 |
| Fam102b | 0.005 | 1.89258044 |
| Vwc2 | 0.00144 | -1.28898079 |
| Nucks1 | 0.50688 | 0.12863417 |
| Vwa5a | 0.148 | 0.24495915 |
| Dusp6 | 0.02578 | -0.48343893 |
| Wwtr1 | 0.05878 | -0.35234765 |
| Glce | 0.50935 | -0.45358468 |
| 9530002B09Rik | 0.00144 | -1.30017714 |
| Olfr1232 | 0.0141 | -1.65741559 |
| Cacna2d3 | 0.20461 | -0.39480819 |
| Acot13 | 0.14639 | -0.32841248 |
| Olfr938 | 0.03662 | -2.01691604 |
| Slc22a4 | 0.0023 | 0.95630557 |
| Plekhm3 | 0.0809 | -0.77119165 |
| Slc19a1 | 0.75099 | -0.13427736 |
| Mmp23 | 0.00274 | -0.89487656 |
| F11 | 0.01295 | -1.3801894 |
| Rrm1 | 0.03545 | 0.5089371 |
| Gtf2b | 0.26782 | 0.28634985 |
| Asb18 | 0.01889 | -0.98003419 |
| Krt26 | 0.00144 | -1.25118919 |
| Cyp26b1 | 0.13965 | -0.77312134 |
| Dnajb3 | 0.37901 | -1.40164897 |
| Smdt1 | 0.00688 | -0.77531736 |
| Hoxa2 | 0.13197 | -1.05449958 |
| Slc35f3 | 0.00144 | -1.29049509 |
| Rgmb | 0.04205 | 0.36556126 |
| Kdelc2 | 0.01271 | 0.90916428 |
| 1700120K04Rik | 0.06227 | 0.86160422 |
| Uxs1 | 0.21064 | 0.36401482 |
| G3bp1 | 0.53719 | 0.12732114 |
| Ms4a8a | 0.75602 | -0.14880722 |
| Tbx21 | 0.00981 | -1.72581848 |
| Natd1 | 0.00526 | -0.91266414 |
| Sphk1 | 0.40252 | -0.20982885 |
| 4930503H13Rik | 0.20826 | -0.32867516 |
| Cul5 | 0.30095 | 0.22300912 |
| Spta1 | 0.00858 | 0.89955187 |
| Nek2 | 0.20188 | -0.73423291 |
| Fbxl20 | 0.76731 | -0.05798638 |
| Ncr1 | 0.06631 | -0.98823246 |
| Rnf182 | 0.00144 | -1.29049509 |
| Creb3l2 | 0.25773 | -0.18263334 |
| Flywch2 | 0.01389 | -0.83169631 |
| Dnttip1 | 0.47147 | -0.19994194 |
| Mat2a | 0.17797 | 0.2715171 |
| Sox5 | 0.06462 | -0.7628565 |
| Slc6a19os | 0.00224 | -1.73680659 |
| Gm14446 | 0.00145 | 1.92119909 |
| Itpr2 | 0.02949 | 0.65735011 |
| Vldlr | 0.00354 | 1.10547704 |
| Sema6d | 0.00144 | 1.19511465 |
| Hmgb2 | 0.0097 | 0.65590992 |
| Tubg1 | 0.03601 | -0.58375941 |
| Ghr | 0.00144 | 1.47015117 |
| Dab1 | 0.00144 | -1.28898079 |
| Bst1 | 0.86095 | 0.0490472 |
| Asprv1 | 0.20358 | 0.45386608 |
| Spred2 | 0.23698 | -0.28748251 |
| Mlana | 0.12958 | -1.05303589 |
| Osr2 | 0.00144 | -1.24613586 |
| Ankrd10 | 0.00144 | 1.36246738 |
| Prkd2 | 0.0045 | -0.71352856 |
| 1700055D18Rik | 0.00145 | -1.2551153 |
| Olfr1354 | 0.00145 | -1.2388121 |
| Tram1 | 0.00415 | 0.84654476 |
| Cog3 | 0.00144 | 1.19339071 |
| Mettl1 | 0.03031 | -0.54539216 |
| Igsf1 | 0.00144 | -1.27165604 |
| Ube2o | 0.00185 | -0.89936677 |
| Fbp2 | 0.00602 | -0.93713598 |
| Degs1 | 0.15325 | -0.26603186 |
| Derl2 | 0.07029 | -0.38182571 |
| Apip | 0.68956 | 0.12461441 |
| Mycbp2 | 0.06327 | -0.66172165 |
| Dfna5 | 0.17249 | -0.44888107 |
| 1700012A03Rik | 0.1479 | -0.40983749 |
| P2rx2 | 0.06764 | -0.59270938 |
| Frmd3 | 0.65665 | -0.2780637 |
| Piezo2 | 0.00144 | -1.30017714 |
| Slc34a1 | 0.00145 | -1.26219879 |
| Krtap15 | 0.00144 | -1.23532597 |
| Cnpy3 | 0.00694 | -0.6619758 |
| Sbk1 | 0.75173 | -0.15851545 |
| Rasa1 | 0.01102 | 0.84703922 |
| Otoa | 0.00145 | -1.24191372 |
| Stk-ps2 | 0.00145 | -1.2388121 |
| Rnf141 | 0.00429 | 0.83763821 |
| Sat2 | 0.08178 | -0.30358241 |
| Mbd3l2 | 0.00144 | -1.27165604 |
| Scyl1 | 0.13476 | -0.35318332 |
| C2 | 0.00723 | -1.18963723 |
| Creb3l4 | 0.50649 | 0.31081256 |
| Hormad1 | 0.00144 | -1.62367905 |
| Il1rapl1 | 0.00144 | -1.29746368 |
| Gpat2 | 0.02252 | -1.11409225 |
| Olfr539 | 0.12881 | -2.64997812 |
| Wdr95 | 0.00144 | -1.38007777 |
| Apol7a | 0.00144 | -1.28116048 |
| Ntn4 | 0.51008 | -0.24514474 |
| Vps18 | 0.01456 | -0.71837321 |
| Gm41609 | 0.0031 | -1.36138936 |
| AI118078 | 0.79476 | -0.13350883 |
| Dedd | 0.20876 | -0.30511455 |
| Tonsl | 0.2239 | -0.25160259 |
| Lypd1 | 0.01698 | -1.04453848 |
| Olfr30 | 0.00144 | -1.87326898 |
| Spint2 | 0.01051 | -1.59966785 |
| Tcp11l2 | 0.18019 | 0.49491431 |
| Defa4 | 0.00144 | -1.28375735 |
| Ly9 | 0.03129 | 0.54230645 |
| Gm17296 | 0.07479 | -0.4578495 |
| Enho | 0.00144 | -1.20243124 |
| Ppp2r2d | 0.56964 | -0.08979075 |
| Prl8a2 | 0.00145 | -1.2388121 |
| Mpp3 | 0.45834 | 0.23202689 |
| Cfi | 0.00144 | -1.29085005 |
| Capza2 | 0.00592 | 1.1519301 |
| Ticrr | 0.10309 | -0.82601577 |
| Adat2 | 0.97886 | 0.0049675 |
| Akap9 | 0.00144 | 1.20108707 |
| Nop58 | 0.00144 | 2.08090119 |
| Nphs1 | 0.00144 | -1.468939 |
| Grid1 | 0.00144 | -1.28116048 |
| Dnmt3l | 0.00762 | -0.69108484 |
| Pomk | 0.58298 | -0.15446138 |
| Slit2 | 0.03439 | 1.1423178 |
| Sh2d1b1 | 0.00145 | -1.2388121 |
| Casd1 | 0.31161 | -0.18242227 |
| Acsl1 | 0.4732 | 0.16819591 |
| Med9 | 0.04003 | -0.62099807 |
| Ptk7 | 0.00144 | -1.62809551 |
| Rfx4 | 0.04709 | -0.85084701 |
| Slc6a4 | 0.14914 | -0.53611234 |
| Hoxc6 | 0.00144 | -1.31495282 |
| Gm30769 | 0.00211 | -1.31534572 |
| Agfg2 | 0.19018 | -0.2696781 |
| Armcx1 | 0.00415 | 1.06973762 |
| Faxc | 0.07322 | -0.97200899 |
| Apof | 0.00144 | -1.26301121 |
| Plin4 | 0.09207 | -0.34456618 |
| 1700049L16Rik | 0.02571 | -1.4300613 |
| Stmn1 | 0.2225 | -0.283483 |
| Tlr1 | 0.60869 | 0.16466415 |
| Tgm3 | 0.017 | -1.21247664 |
| Pea15a | 0.01522 | -0.54356806 |
| Stx4a | 0.02858 | 0.4600783 |
| Tmem55a | 0.00467 | 0.81007797 |
| Gad2 | 0.00762 | -0.99028455 |
| Vmn1r235 | 0.10617 | -0.92886545 |
| Ndufa4l2 | 0.00144 | -1.48581436 |
| Fhod1 | 0.6504 | -0.10131854 |
| Lor | 0.11403 | -0.52961968 |
| Traf6 | 0.00144 | 1.6916397 |
| Lat | 0.67323 | -0.15798924 |
| Gpx8 | 0.01872 | 0.53434764 |
| Slc24a4 | 0.00144 | -1.30017714 |
| Pbdc1 | 0.04447 | 0.46300429 |
| Ift22 | 0.2954 | -0.36308553 |
| Ckmt1 | 0.01289 | -1.17407794 |
| Cbx1 | 0.00841 | -0.74393384 |
| Runx3 | 0.29023 | -0.49243806 |
| Vmac | 0.00839 | -0.64098071 |
| Cdk17 | 0.00532 | -1.10537834 |
| Mfhas1 | 0.96826 | -0.006763 |
| Stag3 | 0.32919 | -1.680636 |
| Rcor1 | 0.01535 | -0.48023706 |
| Mael | 0.01131 | -1.62727591 |
| Grip1 | 0.01761 | 1.39626134 |
| Sumf1 | 0.46703 | 0.15446021 |
| Cotl1 | 0.00144 | -1.57357075 |
| Rtn3 | 0.34314 | 0.15412219 |
| Themis2 | 0.1299 | -0.45012259 |
| Abhd17b | 0.00212 | 1.12179971 |
| Snx30 | 0.02513 | -0.50750153 |
| Id4 | 0.49725 | -0.1901334 |
| Mbnl2 | 0.00436 | 0.96300861 |
| Olfr1313 | 0.00145 | -1.25646236 |
| Atp8b1 | 0.19463 | -1.74430219 |
| 9130024F11Rik | 0.00144 | -1.33632959 |
| Alpl | 0.01266 | -0.79504317 |
| Dgkg | 0.94921 | -0.03329345 |
| Scaf8 | 0.06759 | 0.4076877 |
| Moxd1 | 0.61587 | 0.19863031 |
| Izumo2 | 0.00145 | -1.71073154 |
| Gpt2 | 0.25372 | 0.2512992 |
| Katna1 | 0.05265 | 0.47748122 |
| Rab44 | 0.16216 | -1.18486295 |
| Vmn1r220 | 0.00158 | -1.2446827 |
| Fam58b | 0.00144 | -1.27165604 |
| Tfam | 0.00663 | 1.25267982 |
| 2810474C18Rik | 0.48797 | -0.45779763 |
| Cep350 | 0.00144 | 1.33523082 |
| Hs3st2 | 0.00144 | -1.30017714 |
| Sult1e1 | 0.03054 | -1.42668627 |
| Btaf1 | 0.00144 | 1.52851783 |
| Rnpep | 0.08464 | -0.46760495 |
| Kazald1 | 0.00579 | -0.68690893 |
| Kif22 | 0.10148 | -0.52400783 |
| Krtap28-10 | 0.00144 | -1.27165604 |
| Kmo | 0.01987 | -1.1443198 |
| Dapk3 | 0.31046 | -0.40585643 |
| Pex10 | 0.65122 | -0.20291167 |
| Adgra1 | 0.00144 | -1.4333006 |
| Slc16a9 | 0.77527 | -0.05453664 |
| Mettl15 | 0.1039 | 0.53566691 |
| Dclre1a | 0.1667 | 0.68115976 |
| Cmya5 | 0.54605 | 0.10590351 |
| 4933430I17Rik | 0.00144 | -1.51680878 |
| Cenph | 0.49883 | -1.23099547 |
| Scn9a | 0.00144 | -1.31884045 |
| Wdr75 | 0.00201 | 1.08926706 |
| Bbs10 | 0.00772 | 1.3500083 |
| Sardh | 0.0017 | -1.23723921 |
| Klf3 | 0.06478 | -0.43490238 |
| Fbxo42 | 0.10917 | -0.4164641 |
| Lppr4 | 0.20017 | -0.64666861 |
| Cstf2t | 0.68103 | 0.07497776 |
| Has1 | 0.83667 | 0.1369603 |
| Ldah | 0.79331 | 0.06014906 |
| Pmvk | 0.03066 | -0.55817191 |
| Tmem174 | 0.00413 | -0.73993708 |
| Aqp2 | 0.00947 | -0.79833807 |
| Spint4 | 0.00144 | -1.29746368 |
| Wdr61 | 0.00702 | 0.92020828 |
| Senp2 | 0.01763 | -0.83164044 |
| Ciapin1 | 0.04762 | -0.61339395 |
| Pole3 | 0.09266 | -0.36312174 |
| Fam3b | 0.07792 | -1.81311075 |
| Clcn6 | 0.05849 | -0.65100437 |
| Ripk3 | 0.58752 | -0.10142136 |
| Fbxw17 | 0.03805 | -0.48533729 |
| Rassf6 | 0.00144 | -1.30935626 |
| Trpc5 | 0.04934 | -2.16002777 |
| Olfr1358 | 0.00144 | -1.29085005 |
| Ppm1a | 0.09732 | 0.46671558 |
| Taf1b | 0.50067 | 0.13715989 |
| Sprr2j-ps | 0.00317 | -0.78531993 |
| Nfe2l1 | 0.00144 | -0.97377316 |
| Vgll2 | 0.00144 | -2.16395103 |
| Ly6g6e | 0.10626 | -0.7688693 |
| E030042N06Rik | 0.01521 | 1.58743182 |
| AW549877 | 0.00144 | 1.33957807 |
| Olfr979 | 0.00845 | -1.87047824 |
| Ddx47 | 0.80359 | 0.07288277 |
| Cst13 | 0.31497 | -0.80224943 |
| Csn1s1 | 0.00144 | -1.29049509 |
| Zfp606 | 0.01051 | 1.58452219 |
| Col4a2 | 0.02925 | -0.51935803 |
| Myo3b | 0.00144 | -1.30713006 |
| Suclg1 | 0.85831 | -0.06793837 |
| Gzf1 | 0.0022 | 1.08267994 |
| Cfap53 | 0.06914 | 0.69203433 |
| Ints9 | 0.00704 | -0.64353918 |
| Pgap3 | 0.00673 | -0.82649665 |
| Zfp784 | 0.5357 | 0.10271525 |
| Hapln4 | 0.3005 | -0.58148024 |
| Trim31 | 0.00145 | -1.22376446 |
| Me2 | 0.0892 | 0.31740511 |
| Mxd1 | 0.22918 | -0.26409964 |
| AI593442 | 0.00144 | 2.64914413 |
| Ccdc40 | 0.23906 | -0.69864147 |
| Trim68 | 0.00209 | 1.40469293 |
| Gm34643 | 0.00703 | 0.79232315 |
| Olfr1164 | 0.00145 | -1.22376446 |
| Bub1b | 0.75077 | -0.19444534 |
| Cidec | 0.78094 | -0.31913918 |
| Nudt8 | 0.0458 | -0.90340899 |
| 1110059E24Rik | 0.04362 | 0.474268 |
| Tmem190 | 0.12349 | -3.17971733 |
| Pzp | 0.01347 | 0.73036277 |
| Ifi30 | 0.04479 | -0.47619087 |
| Slx4 | 0.36982 | 0.17177152 |
| Fbxo4 | 0.84969 | 0.03485054 |
| Pop5 | 0.00978 | -0.77459188 |
| Nubp2 | 0.16117 | -0.46708964 |
| Spry1 | 0.24176 | 0.27069694 |
| Tubb2a | 0.00545 | -0.75191239 |
| Dst | 0.30564 | 0.44044736 |
| Rps21 | 0.00145 | -0.90481871 |
| Pax6 | 0.00145 | -1.24191372 |
| Ogg1 | 0.03848 | -0.54866378 |
| Susd3 | 0.01054 | -0.91891979 |
| Nfyc | 0.50275 | 0.16198509 |
| Cdk6 | 0.96692 | 0.01553051 |
| Slc16a11 | 0.03307 | -0.92593517 |
| Ctla2b | 0.84505 | 0.06103277 |
| Cdo1 | 0.72584 | -0.21458533 |
| Luc7l | 0.0209 | 0.87728639 |
| Kdm3a | 0.23481 | 0.54169331 |
| Cyp3a25 | 0.00144 | -1.29049509 |
| Becn1 | 0.19905 | 0.24402703 |
| Sri | 0.2152 | -0.30316547 |
| Casc5 | 0.16106 | 0.68079165 |
| Ncapg | 0.03448 | 1.78708568 |
| Eif3g | 0.02566 | -0.5501522 |
| Postn | 0.00144 | 2.28320372 |
| Tor1b | 0.00699 | -0.77546167 |
| Crot | 0.00193 | 1.08929902 |
| Sptb | 0.6442 | 0.29546263 |
| Wdr19 | 0.26741 | 0.24712867 |
| Plekha2 | 0.19041 | -0.69371225 |
| 9430007A20Rik | 0.00144 | -1.26560808 |
| Kcnt1 | 0.05778 | -0.97011213 |
| Oaf | 0.02225 | -0.63188727 |
| Gm16348 | 0.03237 | -0.89971117 |
| Cdk12 | 0.04446 | -0.49983278 |
| Krt28 | 0.00144 | -1.31884045 |
| Slc25a44 | 0.05947 | -0.52299766 |
| Hcar2 | 0.00144 | -1.28116048 |
| Zfp612 | 0.00421 | 1.33051514 |
| Zfand2b | 0.00145 | -1.00926683 |
| Gnb2l1 | 0.00144 | -1.16129971 |
| 3010026O09Rik | 0.12723 | -0.40536002 |
| 2310034C09Rik | 0.00498 | -1.8378361 |
| Crlf2 | 0.00144 | -1.14696011 |
| A130022F02Rik | 0.07511 | 1.04500346 |
| 4922502D21Rik | 0.00145 | -1.21051401 |
| Clasp1 | 0.89644 | 0.02982119 |
| Bmper | 0.99656 | 0.0014976 |
| Lrrtm3 | 0.08899 | 1.41835635 |
| Phf20l1 | 0.00152 | 1.79296573 |
| Sgol1 | 0.02022 | 1.10271734 |
| Dhrs13 | 0.09551 | -0.53545255 |
| Psmd7 | 0.59189 | 0.09162048 |
| Rinl | 0.00528 | -0.82423661 |
| 2600006K01Rik | 0.29701 | -1.42584098 |
| Cyp4f16 | 0.19421 | -0.24919554 |
| Fabp1 | 0.01738 | -1.74669541 |
| Elk4 | 0.02347 | -0.47421313 |
| Ifi44 | 0.00144 | 2.46951761 |
| H2afy3 | 0.64478 | 0.08601556 |
| Zkscan1 | 0.23978 | -0.49292453 |
| Pramef12 | 0.00144 | -1.28898079 |
| Slmap | 0.32198 | -0.40620588 |
| Cd7 | 0.01182 | -1.46613126 |
| Ccdc91 | 0.023 | 0.73025323 |
| Casp12 | 0.00144 | 2.06601685 |
| Mob1a | 0.00149 | 1.03218678 |
| Olfr167 | 0.00149 | -1.22582153 |
| Hpcal1 | 0.10075 | -0.33159528 |
| Pstk | 0.9391 | 0.01584133 |
| Vasn | 0.01414 | -0.81281752 |
| Yipf3 | 0.11304 | -0.50262694 |
| B9d2 | 0.00144 | -1.27126117 |
| Cntrl | 0.07867 | 1.00763635 |
| Megf11 | 0.00994 | -0.90177174 |
| Acsm3 | 0.23699 | -0.39603968 |
| Rangap1 | 0.09325 | -0.41215142 |
| Bud31 | 0.5647 | 0.19270843 |
| Wfdc12 | 0.24136 | -0.76377993 |
| Rhox6 | 0.00145 | -1.22376446 |
| Lim2 | 0.0017 | -1.60918938 |
| Kcnk2 | 0.45977 | 0.34958534 |
| Dmrtc2 | 0.00145 | -1.22376446 |
| 4933413G19Rik | 0.00145 | -1.27532353 |
| Mtg2 | 0.17873 | -0.48228806 |
| Gpx2 | 0.38676 | -0.22074173 |
| Tmem247 | 0.51308 | -0.3707365 |
| Ap3b2 | 0.08237 | -2.45856102 |
| 1700129C05Rik | 0.06015 | -1.00190455 |
| Akr1b10 | 0.54943 | 0.24052437 |
| Afg3l1 | 0.80325 | 0.1268111 |
| 4930556A17Rik | 0.00144 | -1.27165604 |
| Gm3715 | 0.00158 | -1.22727468 |
| Taar1 | 0.00144 | -1.28898079 |
| Grm3 | 0.00144 | -1.29049509 |
| Letmd1 | 0.52397 | -0.10664267 |
| A530017D24Rik | 0.9175 | -0.04131528 |
| Rab5c | 0.00894 | -0.81830841 |
| Defb29 | 0.00186 | -1.79168349 |
| Izumo1 | 0.00144 | -1.33632959 |
| Cldn34b2 | 0.00144 | -2.3544051 |
| Ccdc171 | 0.08434 | 0.47804877 |
| Clec3b | 0.00315 | -0.87606173 |
| Wnk4 | 0.53756 | 0.17631083 |
| Pabpc2 | 0.01084 | -1.07413081 |
| Ocln | 0.0247 | -0.96581009 |
| Aff3 | 0.00177 | -0.97624535 |
| Ift52 | 0.05225 | 0.51939752 |
| Zfp280b | 0.90729 | 0.02533735 |
| Gm28410 | 0.00144 | -1.30713006 |
| Rbm25 | 0.00236 | 1.5706962 |
| Mbnl3 | 0.00388 | -1.09636557 |
| Srsf1 | 0.00246 | 1.18608422 |
| BC027072 | 0.13125 | -1.3759065 |
| Eef1a2 | 0.48292 | -0.43678478 |
| Olfr1104 | 0.00144 | -1.33632959 |
| Pygb | 0.39395 | 0.54931933 |
| Cyp51 | 0.00145 | -0.98371722 |
| Nts | 0.48563 | 0.15231259 |
| 3110052M02Rik | 0.00144 | 1.28194199 |
| Tab3 | 0.00807 | 0.89621323 |
| Ctps2 | 0.09977 | 0.35252934 |
| Olfr291 | 0.13116 | -0.81574544 |
| Prrc2a | 0.07156 | -0.55836524 |
| Txlna | 0.01166 | -0.83987971 |
| Zscan22 | 0.42315 | -0.48349961 |
| Parn | 0.73721 | -0.05822544 |
| Herc3 | 0.22172 | 0.33550274 |
| Efcab7 | 0.00506 | 1.06084101 |
| Adipor1 | 0.63245 | 0.15922471 |
| Nrbp1 | 0.73495 | 0.09993286 |
| Glt6d1 | 0.05989 | -0.89966591 |
| 4921523L03Rik | 0.00144 | -1.29085005 |
| Hoxb1 | 0.00144 | -1.28116048 |
| Hgf | 0.07053 | -0.75035392 |
| Cblc | 0.06631 | -0.52115567 |
| Rabep2 | 0.02158 | -0.52493916 |
| Polrmt | 0.04 | -0.68028665 |
| Bcl2l11 | 0.25673 | -0.86879805 |
| Gamt | 0.0032 | -0.91903546 |
| Olfr340 | 0.00144 | -1.29051721 |
| Asgr2 | 0.01869 | -0.6764686 |
| Ppm1g | 0.91869 | -0.02748424 |
| Fam3a | 0.05323 | -0.42723758 |
| Saraf | 0.71322 | -0.11717537 |
| Olfr138 | 0.2453 | -0.69422369 |
| Synj1 | 0.83303 | -0.09116778 |
| Adcy3 | 0.07532 | 0.6932424 |
| Spata25 | 0.10095 | -0.53982307 |
| 1700011I03Rik | 0.00314 | -1.14703327 |
| Smc2 | 0.11253 | 0.59690416 |
| Wif1 | 0.0812 | -0.59045872 |
| Pmfbp1 | 0.00144 | -1.25048839 |
| Ap4e1 | 0.00521 | 1.56494929 |
| Uxt | 0.08309 | -0.46089442 |
| Ptpdc1 | 0.00229 | -1.39626628 |
| Tnfsf8 | 0.00144 | -1.29746368 |
| Pcca | 0.01523 | 1.3466995 |
| Lrrc56 | 0.07604 | -0.45900867 |
| Olfr38 | 0.00144 | -1.25118919 |
| Steap1 | 0.0096 | -0.97150126 |
| Matn2 | 0.10878 | 0.55021052 |
| F7 | 0.00967 | 1.147466 |
| AF251705 | 0.00463 | -0.97798988 |
| Stx2 | 0.11782 | -0.29223886 |
| Dctn1 | 0.07051 | -0.59709063 |
| Pcdhb9 | 0.44481 | -0.49177934 |
| Crygd | 0.41667 | -0.54127114 |
| Cby3 | 0.00144 | -1.82030991 |
| Trim3 | 0.22735 | -0.20262494 |
| Prtg | 0.00418 | -0.91063746 |
| 0610040J01Rik | 0.77769 | -0.1405705 |
| Aass | 0.57734 | 0.18709787 |
| Dock8 | 0.03472 | 0.96713456 |
| Abcb8 | 0.50186 | -0.33334546 |
| 4632404H12Rik | 0.00499 | 1.02080239 |
| St3gal5 | 0.10141 | 0.30622177 |
| Kif26b | 0.0213 | -1.43891203 |
| Tex36 | 0.62858 | 0.31246436 |
| Rnaseh1 | 0.13938 | 0.33900433 |
| Gm32358 | 0.0049 | -1.08617297 |
| Sdad1 | 0.0232 | 0.4551414 |
| Ptpn22 | 0.7718 | -0.12664457 |
| Mpv17l2 | 0.03352 | -0.77087589 |
| Prnp | 0.06388 | 0.50691487 |
| Pomt1 | 0.79498 | -0.11165843 |
| Impact | 0.00182 | 1.18724408 |
| Phxr4 | 0.00367 | 1.14700599 |
| Snx7 | 0.00385 | 0.95924656 |
| Gchfr | 0.1484 | -0.52299475 |
| Hmga1 | 0.00714 | -0.7883589 |
| Hdac3 | 0.46024 | -0.1864873 |
| Dicer1 | 0.10329 | -0.413948 |
| Arel1 | 0.06835 | -0.32781435 |
| Fpgs | 0.01026 | -0.6011274 |
| Pphln1 | 0.29161 | -0.16269537 |
| Vmn1r28 | 0.00715 | -1.4835538 |
| Chrne | 0.00457 | -0.75909181 |
| A530083I20Rik | 0.00149 | -1.22582153 |
| Dhcr24 | 0.00144 | -1.59529121 |
| Rps6ka4 | 0.00838 | -0.6447485 |
| Dusp21 | 0.00144 | -1.27165604 |
| LOC105246056 | 0.00144 | -1.12080446 |
| Vegfa | 0.01495 | 0.68048845 |
| Tpd52l1 | 0.08519 | 0.83501928 |
| Lins | 0.57652 | 0.10226497 |
| Ceacam15 | 0.00144 | -1.28116048 |
| Vmn1r210 | 0.00145 | -1.26219879 |
| Ppp4r1l-ps | 0.06858 | 1.39263666 |
| 2610036A22Rik | 0.61837 | 0.17126512 |
| Gm13157 | 0.55654 | 0.15985359 |
| Bmf | 0.76595 | -0.08129751 |
| Slc39a9 | 0.65277 | 0.10204673 |
| Mecom | 0.691 | 0.12630367 |
| Edn2 | 0.09767 | -0.9415379 |
| Tmem8b | 0.2221 | -1.52539869 |
| Cyp3a16 | 0.0384 | -2.01265595 |
| Epm2aip1 | 0.00144 | 1.3670803 |
| Rab3c | 0.06828 | -2.58994595 |
| Cdh15 | 0.3553 | -0.47093875 |
| Ccna2 | 0.22857 | 0.4837462 |
| Spcs3 | 0.01584 | 0.72230896 |
| Mapk1 | 0.0064 | 0.74609918 |
| Tcstv1 | 0.00145 | -1.26219879 |
| Sult1d1 | 0.00144 | -1.40363226 |
| Angptl3 | 0.43721 | 0.24982482 |
| Mbip | 0.00152 | 1.18603862 |
| Ddx42 | 0.13268 | 0.30412971 |
| Gm11767 | 0.00145 | -1.2388121 |
| Robo3 | 0.04319 | -2.99293526 |
| Dram1 | 0.00521 | 0.87486402 |
| Obox3 | 0.00144 | -1.26301121 |
| Kif11 | 0.06412 | 1.09483804 |
| Ugcg | 0.00659 | 0.77568686 |
| Ier2 | 0.19379 | -0.30033486 |
| Foxq1 | 0.00144 | -1.36610297 |
| Dopey2 | 0.1699 | -0.26093953 |
| Bace2 | 0.3054 | -0.26981222 |
| Ofd1 | 0.00158 | 1.67781733 |
| Hdac8 | 0.43871 | -0.49492659 |
| Cbr3 | 0.00144 | -1.5489067 |
| Opn1sw | 0.00356 | -1.47378786 |
| Glo1 | 0.37968 | -0.14123342 |
| Zfyve16 | 0.00144 | 1.24222171 |
| Thap11 | 0.32247 | -0.29936205 |
| Pga5 | 0.00588 | -1.45641127 |
| Snx4 | 0.00145 | -1.73755976 |
| Tff2 | 0.00144 | -1.28898079 |
| Emc8 | 0.01777 | -0.70895538 |
| Olfr430 | 0.68392 | 0.20696844 |
| Adgrl4 | 0.00144 | 2.19391655 |
| Usp24 | 0.19026 | 0.79389297 |
| F2 | 0.00144 | -1.30017714 |
| Casz1 | 0.00998 | -0.89962519 |
| Elf3 | 0.89056 | 0.11239196 |
| Nmral1 | 0.03432 | -0.45079126 |
| Dvl3 | 0.41526 | -0.34726218 |
| Pdcl | 0.06774 | -0.79684465 |
| Cryba2 | 0.0031 | -1.33456483 |
| Prelid2 | 0.0259 | -0.47694322 |
| Chad | 0.06849 | -0.95768442 |
| 4931408D14Rik | 0.67596 | -0.21748008 |
| Car11 | 0.69573 | -0.08258259 |
| Drap1 | 0.0044 | -0.88483501 |
| Pik3cb | 0.01731 | 0.81796487 |
| Mars | 0.69986 | -0.12198138 |
| Cpsf3 | 0.39652 | 0.21992779 |
| Fam184a | 0.00144 | -1.22925777 |
| Glb1l | 0.45927 | 0.19280947 |
| Lonrf3 | 0.35589 | 0.47326605 |
| Mapk6 | 0.00144 | -1.28275058 |
| Ampd2 | 0.00992 | -0.62166603 |
| Eif2s3y | 0.00145 | 2.07719729 |
| Obp2a | 0.00202 | -1.38883422 |
| Cdnf | 0.01995 | 0.66963369 |
| Obsl1 | 0.03342 | -0.59338651 |
| Gpr150 | 0.00144 | -1.31518407 |
| Itgb8 | 0.00495 | 1.25273595 |
| Cdk19 | 0.00787 | 0.84676583 |
| Rttn | 0.64482 | -0.73514153 |
| Hyal1 | 0.04326 | 0.50566316 |
| Adamts9 | 0.05974 | 0.64424461 |
| Klhdc9 | 0.45345 | -0.22025414 |
| Acss1 | 0.09248 | 0.27969438 |
| Nat8 | 0.01068 | -1.03528162 |
| Gm15708 | 0.12941 | 0.45763642 |
| Nosip | 0.00144 | -1.33114983 |
| Olfr771 | 0.00144 | -1.29746368 |
| Zcchc3 | 0.03724 | -0.50553428 |
| Tars | 0.20327 | 0.20809254 |
| Nif3l1 | 0.42015 | -0.19234611 |
| Psme4 | 0.00475 | 1.84853207 |
| N4bp2l1 | 0.00411 | 1.18777058 |
| Exoc6 | 0.00792 | 0.62084284 |
| Ppp6r1 | 0.05444 | -0.47995597 |
| Ces4a | 0.02058 | -1.03164931 |
| Clhc1 | 0.00144 | -1.28116048 |
| Samd10 | 0.00379 | -0.92001789 |
| Angptl1 | 0.03506 | -0.9858148 |
| Suclg2 | 0.45305 | 0.23894152 |
| Asb6 | 0.00507 | -0.70671758 |
| Nfix | 0.15191 | -0.53131045 |
| Ncoa2 | 0.19428 | -0.32279765 |
| 1700030F18Rik | 0.00144 | -1.30713006 |
| Adamtsl1 | 0.45671 | -1.14622435 |
| Olfr1026 | 0.69914 | 0.12373005 |
| Crb1 | 0.00144 | -1.30713006 |
| Unc45b | 0.05359 | -0.41399678 |
| Zfp639 | 0.1683 | 0.25539373 |
| Akt1s1 | 0.16255 | -0.6418777 |
| Nmd3 | 0.00144 | 1.57470621 |
| Epb4.1l5 | 0.00144 | -1.29051721 |
| Cadm1 | 0.29252 | -0.34511396 |
| Olfr948 | 0.02983 | -1.88898867 |
| Fbxw4 | 0.06907 | -0.48104999 |
| Kdm5c | 0.00888 | -0.59380721 |
| Klhl21 | 0.73661 | -0.14375209 |
| Rab3d | 0.00718 | -0.75331832 |
| 4932415M13Rik | 0.00144 | -1.29746368 |
| Prss12 | 0.79522 | -0.07210854 |
| Mcph1 | 0.01131 | 0.86460478 |
| Pitx3 | 0.0415 | -0.49078198 |
| 4933412E24Rik | 0.08161 | -0.97970592 |
| Lyzl4 | 0.01686 | -0.51622482 |
| Prr32 | 0.00144 | -1.26934956 |
| Grid2 | 0.00144 | -1.28116048 |
| Cnot10 | 0.9298 | -0.01869477 |
| Sec23ip | 0.02988 | 0.44005546 |
| Nfic | 0.03973 | -0.5405336 |
| Echdc2 | 0.13372 | 0.38108296 |
| 1810014B01Rik | 0.84712 | 0.13759148 |
| Olfr780 | 0.02122 | -0.96350362 |
| Gpr20 | 0.0857 | -1.16518253 |
| Pmaip1 | 0.13631 | 0.91483651 |
| Tlk2 | 0.00163 | 1.06285849 |
| Rnaset2a | 0.7226 | 0.11612099 |
| Coro1c | 0.00787 | -0.83786461 |
| Csmd3 | 0.00144 | -1.27165604 |
| Fance | 0.0049 | -0.73765021 |
| Olfr2 | 0.00145 | -1.16432455 |
| Neo1 | 0.88154 | -0.03255268 |
| Myl6b | 0.01237 | -0.61303019 |
| Skiv2l2 | 0.00548 | 0.73179495 |
| Klrb1c | 0.28102 | -0.6587919 |
| Pcdha11 | 0.00144 | -1.27992547 |
| Krtcap3 | 0.61924 | 0.16092931 |
| Inafm2 | 0.4533 | 0.2509874 |
| Lhb | 0.0169 | -0.68220483 |
| Zbtb38 | 0.13703 | -0.26926642 |
| Cyp2j13 | 0.0699 | 0.83118986 |
| Olfr456 | 0.345 | -0.54227279 |
| Lrp1b | 0.00144 | -1.43766499 |
| Zfp120 | 0.00263 | 3.15540486 |
| Fscn3 | 0.47064 | -0.23418946 |
| Spry3 | 0.0037 | -1.11214255 |
| Sox7 | 0.93116 | 0.01739541 |
| Abcf1 | 0.24323 | 0.43404988 |
| Thra | 0.00181 | -0.86996004 |
| Mfsd6l | 0.65723 | -0.30709378 |
| 4932438A13Rik | 0.18115 | 0.28359276 |
| Lrwd1 | 0.13264 | -0.31397292 |
| Nol4l | 0.00144 | -1.30713006 |
| Tax1bp1 | 0.00145 | -1.2388121 |
| Krtap2-4 | 0.00144 | -1.28116048 |
| BC052040 | 0.21776 | -0.23675277 |
| 4933435E02Rik | 0.01539 | -1.74317995 |
| Pcdh9 | 0.12803 | 0.83430732 |
| Cd209b | 0.43325 | -0.45268487 |
| Fahd2a | 0.02357 | -0.7476089 |
| Ghrhr | 0.2854 | -0.66415775 |
| Brca1 | 0.15323 | 0.58743151 |
| Ndufa8 | 0.00677 | -0.9602727 |
| Cdc42bpb | 0.01175 | -0.66180107 |
| Echdc3 | 0.16997 | -0.44870528 |
| Chrnb1 | 0.92402 | 0.0785635 |
| Zfp110 | 0.68298 | -0.10044004 |
| Moap1 | 0.00838 | 1.13745979 |
| Rnf44 | 0.98899 | -0.0029783 |
| Armc6 | 0.04461 | -0.69290712 |
| Duox1 | 0.08781 | -0.48258447 |
| Vbp1 | 0.00144 | 2.23384165 |
| Ucn2 | 0.00144 | -1.52534313 |
| Mcu | 0.60663 | -0.13188437 |
| Fbp1 | 0.09863 | -0.98446685 |
| Esr1 | 0.00144 | -1.31884045 |
| Kpna2 | 0.07361 | 0.55783111 |
| Cyp19a1 | 0.14322 | -1.2391376 |
| Unc50 | 0.64779 | 0.08848245 |
| Tac4 | 0.02521 | -1.39097631 |
| Utp11l | 0.0459 | 0.35958248 |
| Socs3 | 0.6158 | -0.20671241 |
| Rnaseh2b | 0.0057 | 0.72285115 |
| Ift27 | 0.00144 | -0.97795415 |
| Sun5 | 0.00144 | -1.55081145 |
| Dhx57 | 0.00495 | 0.81009629 |
| Atg7 | 0.0107 | -0.76413082 |
| Proser2 | 0.01751 | -0.52798163 |
| Awat2 | 0.09641 | -0.88914155 |
| Erv3 | 0.0179 | 1.01715333 |
| Rhox13 | 0.40169 | -0.50994032 |
| 3300002A11Rik | 0.00145 | -1.25646236 |
| Arhgef26 | 0.00441 | 1.31783905 |
| Slc22a15 | 0.93548 | -0.01934447 |
| Mycbp2 | 0.00478 | 0.92157766 |
| Il6st | 0.25287 | 0.26472116 |
| H2-DMa | 0.11515 | -0.59581205 |
| B230216G23Rik | 0.90163 | -0.07135469 |
| Apex1 | 0.67748 | -0.15841266 |
| Gm4737 | 0.25038 | -0.4053756 |
| Ppil2 | 0.10995 | -0.26834212 |
| D730045B01Rik | 0.00144 | -1.54166029 |
| Tenm4 | 0.0542 | -0.84967373 |
| Prss21 | 0.00145 | -1.16432455 |
| Tspan6 | 0.88912 | -0.0296749 |
| Dpyd | 0.00144 | 1.37476661 |
| Zyx | 0.01487 | -0.58078564 |
| Zfp426 | 0.0035 | 0.7227632 |
| Zbtb7b | 0.011 | -0.72475861 |
| Siae | 0.20158 | -0.25769646 |
| Traf5 | 0.20492 | -0.27187235 |
| 2810032G03Rik | 0.00144 | -1.27165604 |
| Btbd16 | 0.0496 | -0.49446828 |
| Olfr223 | 0.0062 | -0.929665 |
| Atp8b1 | 0.01025 | 1.35486055 |
| Slc3a2 | 0.04178 | -0.44524945 |
| Enkd1 | 0.05526 | -0.38800249 |
| Masp1 | 0.12766 | 0.60795576 |
| Oas3 | 0.00145 | -1.2388121 |
| Harbi1 | 0.30389 | 1.30555512 |
| Pdlim2 | 0.00144 | -1.38718528 |
| H2afv | 0.00309 | -0.97575843 |
| Zfp719 | 0.02153 | 1.70798916 |
| Capn1 | 0.00278 | -0.92600455 |
| Dync2li1 | 0.06728 | 0.5582221 |
| 4930426D05Rik | 0.05628 | -0.91440819 |
| Tldc1 | 0.78198 | -0.13403205 |
| Fbxo18 | 0.08328 | -0.32458832 |
| Igfbp7 | 0.02596 | -0.45232875 |
| Fat3 | 0.00144 | -1.29051721 |
| Sox18 | 0.00158 | -1.04970667 |
| Slc7a7 | 0.00776 | -0.73649639 |
| Sapcd2 | 0.01276 | -1.34106333 |
| 8430432A02Rik | 0.19943 | 0.4630141 |
| Serpina10 | 0.66581 | -0.1576493 |
| Ttc22 | 0.08565 | -0.92360968 |
| 4921507L20Rik | 0.0235 | -1.01876483 |
| Gpam | 0.04701 | -0.37132723 |
| Phf5a | 0.24675 | -0.30595611 |
| Iqcf3 | 0.01134 | -1.02600084 |
| Acbd7 | 0.64522 | 0.12966654 |
| Alox12e | 0.03667 | -0.79975673 |
| Actl7b | 0.56142 | -0.27625399 |
| Olfr1336 | 0.00144 | -1.27165604 |
| Stk25 | 0.01751 | -0.47753658 |
| Socs4 | 0.00144 | 2.17162719 |
| Fbxo22 | 0.1217 | 0.26148577 |
| Sult5a1 | 0.01633 | -0.80904391 |
| Map2k4 | 0.14076 | 0.26898218 |
| Ccdc64b | 0.00144 | -1.45718629 |
| Kcnb2 | 0.00144 | -1.6429723 |
| 2900055J20Rik | 0.06474 | -0.47743499 |
| Olfr304 | 0.00144 | -1.28116048 |
| Stra6 | 0.00144 | 4.02119968 |
| Olfr1372-ps1 | 0.51675 | 0.21531585 |
| Cyp2c66 | 0.00414 | -1.30978182 |
| Vmn1r24 | 0.01091 | -1.07276267 |
| Paip1 | 0.89964 | -0.0218676 |
| Tmem52 | 0.04805 | -0.88904921 |
| Cplx3 | 0.01684 | -0.87476445 |
| Dcst1 | 0.29124 | -0.50725611 |
| Cish | 0.18126 | -0.54311215 |
| Rnf225 | 0.01484 | -1.18901353 |
| 1700019B03Rik | 0.61764 | -0.29448536 |
| Gsdmcl-ps | 0.00931 | -1.94514546 |
| Zfp819 | 0.00592 | -1.07650415 |
| Setx | 0.00437 | 0.9591258 |
| 4930412O13Rik | 0.00144 | -1.51680878 |
| Sepp1 | 0.32098 | 0.1974139 |
| Cby1 | 0.19731 | -0.3641894 |
| Caskin1 | 0.01764 | -1.9383227 |
| Vmn2r7 | 0.00144 | -1.29347279 |
| Rasl2-9 | 0.9556 | -0.01589331 |
| Spryd3 | 0.00551 | -0.73527212 |
| Mob3b | 0.00144 | -1.26911831 |
| Pou3f4 | 0.00144 | -1.29085005 |
| Ak6 | 0.06247 | 0.32805609 |
| Spn-ps | 0.05448 | -1.00077374 |
| H2-M3 | 0.02246 | -0.63230101 |
| Srgap3 | 0.52336 | 0.39257604 |
| Agxt | 0.00144 | -1.27165604 |
| Olfr1324 | 0.00144 | -1.24466058 |
| Vmn1r46 | 0.09459 | 0.37798337 |
| Syt17 | 0.06374 | 0.46481109 |
| Stk38 | 0.00897 | 0.61137788 |
| Dkc1 | 0.41256 | 0.21990675 |
| Cldn13 | 0.00144 | -1.29746368 |
| Zfp62 | 0.00295 | 1.95079493 |
| Gm11149 | 0.0366 | -1.056783 |
| 8030474K03Rik | 0.00308 | -1.68023648 |
| Traf3 | 0.16275 | 0.26946702 |
| Fam83b | 0.00144 | -1.25118919 |
| Zfp131 | 0.00281 | 1.04409665 |
| Zfp647 | 0.55825 | 0.14628498 |
| Olfr692 | 0.0359 | -1.88210223 |
| Fam214a | 0.12485 | 0.84328987 |
| Mtmr4 | 0.63393 | -0.09779249 |
| Ehd1 | 0.00181 | -0.95039139 |
| Gm29825 | 0.00144 | -1.27165604 |
| Hmg20a | 0.67851 | 0.0746413 |
| Alkbh6 | 0.04377 | -0.50259048 |
| Spryd7 | 0.09851 | 0.43515468 |
| Smchd1 | 0.00144 | 1.60789815 |
| Treml4 | 0.01335 | -1.62533709 |
| Trp53rka | 0.29989 | -0.32435736 |
| Entpd8 | 0.00144 | -1.32766452 |
| Gdpd5 | 0.09926 | -0.41487676 |
| S100a3 | 0.75697 | -0.12844856 |
| Ice2 | 0.00621 | 1.35348353 |
| Arsb | 0.80481 | -0.06994504 |
| 4930595M18Rik | 0.00747 | -1.61449381 |
| Psmb7 | 0.38263 | 0.36302912 |
| 5530401A14Rik | 0.00144 | -1.52234686 |
| Polg | 0.39943 | -0.26805529 |
| Itpr2 | 0.69533 | 0.18204821 |
| S100b | 0.00567 | -0.9899085 |
| Phex | 0.00144 | -1.29049509 |
| Dennd4a | 0.02086 | 0.81997286 |
| Olfr1308 | 0.00144 | -1.25118919 |
| Serpind1 | 0.06765 | -0.82799423 |
| Gtf2h1 | 0.30262 | -0.33360257 |
| Trim39 | 0.00772 | 0.80412643 |
| Ggt1 | 0.00765 | -0.98980568 |
| Smyd1 | 0.3335 | -0.26993039 |
| Dnaaf1 | 0.97319 | 0.01017282 |
| Bcas2 | 0.26893 | 0.19279511 |
| Esrp1 | 0.00158 | -1.2446827 |
| Ank3 | 0.22605 | 0.28299282 |
| Cntd1 | 0.00144 | -1.30713006 |
| Ap3m2 | 0.03618 | -0.68709553 |
| Snx19 | 0.32433 | 0.21982182 |
| Hgsnat | 0.23546 | -0.27426973 |
| Ick | 0.98109 | -0.00542009 |
| Efcab10 | 0.00144 | -1.31495282 |
| Thoc5 | 0.073 | -0.31930236 |
| Ttc29 | 0.00144 | -1.29746368 |
| Olfr685 | 0.00455 | -1.1280166 |
| Tec | 0.0906 | 0.52329934 |
| Pif1 | 0.52719 | 0.12747352 |
| AI480624 | 0.00144 | -1.31495282 |
| Vmn1r89 | 0.19292 | -0.58973092 |
| Trim62 | 0.02413 | -2.63718615 |
| Mak | 0.00144 | -1.27992547 |
| Ceacam19 | 0.00145 | -1.26219879 |
| Polr3gl | 0.04107 | -0.41603588 |
| Fbn1 | 0.0586 | 0.39987774 |
| Dpp4 | 0.11421 | 1.08252977 |
| Cyp2b9 | 0.99392 | -0.0060682 |
| Olfr1233 | 0.00144 | -1.30713006 |
| Pgp | 0.00225 | -1.01838813 |
| Cacna1g | 0.42576 | -0.23742957 |
| Sbds | 0.00388 | 0.7156229 |
| Vstm2a | 0.00523 | -2.09834007 |
| Fam109a | 0.00144 | -1.08128099 |
| Tyk2 | 0.49428 | 0.17617919 |
| Ropn1 | 0.066 | -2.41359711 |
| Zfp646 | 0.33305 | -0.18044079 |
| Mau2 | 0.00611 | -0.78149074 |
| Olfr959 | 0.00144 | -1.20030135 |
| Slc16a7 | 0.00538 | 1.08298149 |
| Homer3 | 0.01218 | -0.59671228 |
| Pappa2 | 0.00145 | -1.26219879 |
| Ttc9 | 0.00144 | -1.30017714 |
| Armc5 | 0.0161 | -0.6259479 |
| Ece1 | 0.12257 | 0.48552511 |
| Itgal | 0.00295 | -0.78815855 |
| Pitpna | 0.83142 | 0.03375422 |
| Arhgap19 | 0.05976 | -2.1726161 |
| Frmd5 | 0.00893 | 1.12496496 |
| Mepce | 0.31669 | -0.27287488 |
| Pdgfrl | 0.13025 | -0.27014123 |
| Amhr2 | 0.0435 | -0.85619486 |
| C1qbp | 0.5273 | -0.18446262 |
| Bckdha | 0.01953 | -0.73126178 |
| Ganc | 0.01227 | 1.44316768 |
| Slc30a9 | 0.06731 | -0.39271489 |
| Ruvbl1 | 0.06088 | -0.34310097 |
| Gm16499 | 0.33463 | -0.83249394 |
| Lysmd4 | 0.88561 | -0.0430063 |
| Grm5 | 0.01378 | -2.10440827 |
| Hdhd3 | 0.00231 | -1.17535312 |
| Map7d2 | 0.00472 | 1.4206641 |
| Nmt2 | 0.00472 | 0.69236984 |
| Mybpc3 | 0.00144 | -1.02872328 |
| Map4 | 0.13289 | -0.27442937 |
| Kmt2a | 0.25904 | 0.19873747 |
| Lipn | 0.00145 | -1.22518835 |
| Ly86 | 0.63931 | 0.10436107 |
| Ipo8 | 0.00342 | -1.15321667 |
| Lgals1 | 0.00144 | -1.20939583 |
| Mcfd2 | 0.10539 | 0.38109165 |
| Wfdc2 | 0.00144 | -1.9577886 |
| Hoga1 | 0.32397 | -0.41352897 |
| Map1lc3a | 0.04678 | -0.92994203 |
| Xrn1 | 0.00158 | 1.12201181 |
| Mefv | 0.01574 | -1.45135169 |
| Msrb2 | 0.67824 | 0.12966843 |
| Gabbr1 | 0.13769 | 1.067706 |
| Pla2g6 | 0.23871 | -0.37923921 |
| Il17rb | 0.34297 | 0.38185176 |
| Hist1h1e | 0.00144 | -1.415771 |
| Aspm | 0.32378 | -0.5712537 |
| Slc2a1 | 0.38239 | -0.32141817 |
| Adap2 | 0.0171 | 0.9247266 |
| Ccl8 | 0.00144 | -2.14761032 |
| Rad17 | 0.00144 | 1.33107224 |
| Emilin3 | 0.00193 | -1.74444757 |
| Psen1 | 0.78611 | 0.05683161 |
| Phldb2 | 0.93013 | -0.03314241 |
| Il4ra | 0.0043 | -0.73591805 |
| 5330439B14Rik | 0.00144 | -1.26560808 |
| Klb | 0.16135 | -0.7405572 |
| Hspb8 | 0.53819 | -0.32056346 |
| Abcc10 | 0.0281 | -0.95564746 |
| Gnb4 | 0.02747 | -0.43276862 |
| Gdf1 | 0.00257 | -1.05163801 |
| Ly6g6c | 0.0027 | -1.00854108 |
| Prrt3 | 0.00144 | -1.28898079 |
| Fkbp2 | 0.07546 | -0.45545723 |
| Mat1a | 0.00144 | -1.26560808 |
| Sowahb | 0.00144 | -1.27716959 |
| 5830472F04Rik | 0.28183 | 0.29098754 |
| Fads3 | 0.01357 | -0.8523402 |
| Chst12 | 0.01284 | -1.08272747 |
| Nelfb | 0.01094 | -0.73371716 |
| Tenm1 | 0.00144 | -1.28116048 |
| Unkl | 0.31499 | -0.48832327 |
| Fam161b | 0.00144 | 1.29417137 |
| Zbtb17 | 0.0378 | -0.60914035 |
| Gbp7 | 0.00144 | 2.72412225 |
| Srrm3 | 0.02008 | -1.1726161 |
| Timp3 | 0.03178 | 0.67783511 |
| Plk4 | 0.06182 | 0.85715895 |
| Vis1 | 0.75868 | 0.09177775 |
| Mast2 | 0.34507 | -0.18376282 |
| Rnh1 | 0.17988 | -0.54317751 |
| Pik3ip1 | 0.00267 | -0.7964324 |
| Fry | 0.46606 | 0.19259313 |
| Scube3 | 0.00144 | -1.29051721 |
| Snapc3 | 0.00406 | 2.36124378 |
| Tbc1d2b | 0.01516 | -0.5318416 |
| Hacd4 | 0.29637 | 0.22545074 |
| Cenpm | 0.13464 | -0.95329254 |
| Tslp | 0.09042 | 1.20948594 |
| Ap5m1 | 0.00193 | 0.87754408 |
| Ehhadh | 0.02309 | 0.66095041 |
| Mkl2 | 0.25421 | -0.23542959 |
| Sars2 | 0.03419 | -0.70178752 |
| Camkmt | 0.01479 | 1.28067075 |
| Tex10 | 0.47039 | 0.1175373 |
| Ptbp1 | 0.00257 | -1.07216451 |
| Kcnk4 | 0.02296 | -0.86419189 |
| Ccdc105 | 0.00144 | -1.28116048 |
| Gm10658 | 0.69099 | -0.19134707 |
| Senp7 | 0.42543 | -0.22086364 |
| Cyyr1 | 0.0801 | -0.54916799 |
| Gpx4 | 0.00548 | -0.99820384 |
| Tm2d3 | 0.24907 | -0.45183286 |
| Galnt15 | 0.50255 | 0.17400593 |
| G6pc | 0.52248 | -0.30368648 |
| Ncs1 | 0.0421 | -0.95678805 |
| Olr1 | 0.00145 | -1.25646236 |
| Tpm3 | 0.04362 | 0.43785791 |
| Adgra2 | 0.049 | 0.5613461 |
| Prpf38a | 0.49748 | 0.11012266 |
| 4930562F07Rik | 0.00144 | -1.30713006 |
| Tnnc1 | 0.24441 | -0.35094284 |
| Abl2 | 0.48578 | 0.13012362 |
| Thbs2 | 0.01164 | -0.57822047 |
| Tbp | 0.30018 | 0.33234827 |
| Tbp | 0.32882 | -0.25701341 |
| Svs4 | 0.00145 | -1.22376446 |
| Ppp2r2c | 0.00272 | -1.09759392 |
| Zcchc8 | 0.06397 | 0.34768116 |
| Atp6ap2 | 0.00149 | 1.27844176 |
| G2e3 | 0.06413 | 1.19353653 |
| Cxcl9 | 0.74123 | 0.15137135 |
| Fdxr | 0.06452 | -0.53603649 |
| Eef2k | 0.05991 | -0.51263201 |
| Atn1 | 0.08683 | -0.44902095 |
| Ube2r2 | 0.80521 | -0.05811232 |
| Cyp7b1 | 0.47222 | 0.23827605 |
| Sytl3 | 0.00144 | -1.36353418 |
| Gpx7 | 0.01705 | -0.63583159 |
| Gatm | 0.3509 | -1.21332629 |
| Dtl | 0.00144 | -1.28898079 |
| Got1l1 | 0.00144 | -1.28116048 |
| Tlr5 | 0.17806 | 0.52731763 |
| Osbpl10 | 0.00145 | -0.99814672 |
| Crct1 | 0.00144 | -1.16492156 |
| Ostm1 | 0.08187 | 0.42619 |
| Snn | 0.4642 | -0.49748606 |
| Ccl6 | 0.17197 | -0.31255139 |
| Rgl2 | 0.40076 | -0.14008402 |
| Arl14 | 0.00144 | -1.48396829 |
| Vps39 | 0.96816 | 0.00884873 |
| Amph | 0.91962 | -0.11935153 |
| Prkcd | 0.00414 | 0.68747806 |
| Hoxb3 | 0.4638 | -0.4855206 |
| Calm4 | 0.67832 | -0.1369777 |
| Dfna5 | 0.02036 | 1.24634643 |
| Apoc4 | 0.24329 | -0.76252921 |
| Saxo1 | 0.21463 | 0.72085677 |
| Neu2 | 0.02519 | -0.81722014 |
| Shc4 | 0.00317 | -1.11935153 |
| Cnrip1 | 0.75081 | 0.06364838 |
| Olfr1300-ps1 | 0.05896 | -2.21136712 |
| Lmo7 | 0.61978 | -0.14264657 |
| Tcf21 | 0.16226 | -0.42445167 |
| Olfr56 | 0.00144 | -1.29746368 |
| Sprr2b | 0.03886 | -0.45037215 |
| 1700021F07Rik | 0.00144 | -1.31884045 |
| Gbx1 | 0.01892 | -1.12000661 |
| Lipa | 0.23397 | -0.22240624 |
| Eya3 | 0.13956 | -0.4942989 |
| Crygs | 0.00144 | -1.22098834 |
| Col11a1 | 0.93801 | -0.16176086 |
| Kidins220 | 0.00168 | 1.36721263 |
| Appbp2 | 0.81831 | 0.03919317 |
| Dstn | 0.00752 | 1.01687186 |
| Pacrgl | 0.64067 | -0.09414063 |
| Gkap1 | 0.00144 | 1.62918344 |
| 1700095J03Rik | 0.00145 | -1.27532353 |
| Insl6 | 0.2593 | -0.39383249 |
| Stil | 0.04908 | -0.92450106 |
| Ybey | 0.13633 | -0.27846507 |
| Aplf | 0.06688 | 0.41958859 |
| Pygm | 0.95178 | 0.03470797 |
| F11r | 0.38533 | 0.14280782 |
| Piga | 0.00233 | 1.31625079 |
| Zfp438 | 0.9965 | -0.00097022 |
| Hpgd | 0.00764 | 0.80900307 |
| Gm11568 | 0.00144 | -1.31495282 |
| Olfr299 | 0.00144 | -1.28898079 |
| Spink8 | 0.00144 | -1.28898079 |
| Fpr-rs3 | 0.21217 | -0.82534271 |
| Adipoq | 0.68102 | -0.4438997 |
| Slc38a2 | 0.00262 | 1.67635937 |
| Map4k2 | 0.48556 | 0.12472878 |
| Rab12 | 0.75492 | 0.12197498 |
| Wdr13 | 0.00288 | 0.99072042 |
| Gnal | 0.00698 | -1.63079701 |
| Tph1 | 0.00177 | -1.15847607 |
| Osbp2 | 0.03603 | -0.86046823 |
| P4ha1 | 0.27447 | 0.23049345 |
| Wscd1 | 0.00144 | -2.13815131 |
| Rragd | 0.15309 | -0.30883337 |
| Myl9 | 0.65355 | 0.57829654 |
| Dchs1 | 0.01069 | -1.25909434 |
| Capn8 | 0.00144 | -1.31495282 |
| Evi5 | 0.00144 | 2.14951106 |
| Olfr1426 | 0.00144 | -1.27454685 |
| Cyp46a1 | 0.00144 | -1.5269926 |
| Txndc8 | 0.00144 | -1.31495282 |
| Col23a1 | 0.00144 | -1.29644839 |
| Ccnb2 | 0.74223 | 0.14392082 |
| Tmem223 | 0.18278 | -0.49817334 |
| Sectm1a | 0.11932 | -0.80350734 |
| Adck5 | 0.024 | -0.49221989 |
| Dpp8 | 0.00482 | 0.97425014 |
| Med13l | 0.23664 | 0.35174462 |
| Hmgn5 | 0.03144 | 1.44595234 |
| Sms | 0.08108 | -0.35185279 |
| Xlr4b | 0.73598 | 0.37300982 |
| Sfrp4 | 0.03972 | -0.47796551 |
| Tnfrsf13c | 0.09429 | -0.97516876 |
| BC017643 | 0.89194 | -0.0223532 |
| Pex11a | 0.00144 | -1.34850039 |
| Ier5 | 0.32939 | -0.29766129 |
| Spag9 | 0.02208 | 1.45841797 |
| Foxj1 | 0.00746 | -1.80417688 |
| Ift122 | 0.60196 | -0.09805887 |
| Slc19a3 | 0.15531 | -0.80911555 |
| Nlgn3 | 0.06946 | -1.165124 |
| Azgp1 | 0.97751 | 0.00979518 |
| Olfr812 | 0.30416 | -1.36973941 |
| Tgif2 | 0.01362 | -0.69733811 |
| Wbscr22 | 0.25774 | -0.31071651 |
| Dcstamp | 0.01134 | -1.04486201 |
| Cldn10 | 0.00418 | -1.26434088 |
| Chil6 | 0.02958 | -0.89162376 |
| Pard6a | 0.01748 | -0.87739783 |
| Ush2a | 0.00144 | -1.29746368 |
| Macrod2 | 0.08406 | -2.38562916 |
| Tff3 | 0.11422 | -3.31959361 |
| Pdcd2 | 0.92305 | 0.02601759 |
| Cemip | 0.00144 | -1.30017714 |
| Meg3 | 0.00461 | 2.0165605 |
| Rac3 | 0.02964 | -0.5243394 |
| F2rl1 | 0.57093 | -0.53216292 |
| Nup153 | 0.12005 | -0.32598518 |
| Elf5 | 0.00144 | -1.31495282 |
| Ehd4 | 0.12866 | 0.29163521 |
| Cd3e | 0.00422 | -1.45030804 |
| Psmd5 | 0.2699 | 0.23930729 |
| Car2 | 0.38037 | 0.33719271 |
| Bpifb2 | 0.00144 | -1.20555712 |
| Ybx2 | 0.02264 | 0.49082517 |
| Ankrd27 | 0.30071 | -0.24380741 |
| St8sia1 | 0.02606 | -1.17180719 |
| Gulp1 | 0.00144 | -1.28898079 |
| Rnase2a | 0.00736 | -0.98702008 |
| Sele | 0.234 | 0.45246006 |
| Ces2f | 0.0061 | -1.54197809 |
| Ring1 | 0.48727 | -0.26779837 |
| Rrp36 | 0.15222 | -0.33628632 |
| Gm9779 | 0.10918 | 0.40018637 |
| Ptpn13 | 0.00381 | 1.11397207 |
| Prl4a1 | 0.00144 | -1.29085005 |
| Kcnab3 | 0.00163 | -1.29258142 |
| Tmcc2 | 0.049 | -0.72966033 |
| Tmem219 | 0.00144 | -1.09246483 |
| Gstm3 | 0.01697 | -0.59252802 |
| Camkv | 0.03538 | 0.56814245 |
| Npy | 0.00226 | -1.42479991 |
| Ccdc82 | 0.0069 | 2.76659415 |
| 4930430F08Rik | 0.00732 | 1.22643881 |
| Unc79 | 0.36805 | -0.44612778 |
| Tlx2 | 0.0124 | -0.90992284 |
| Zfp772 | 0.00196 | 1.01391116 |
| Vps13b | 0.81601 | -0.12011054 |
| Nfkb2 | 0.04588 | -0.5947326 |
| Sh2d4a | 0.11041 | 0.39499692 |
| Hecw2 | 0.00222 | 2.16303767 |
| Msh3 | 0.00735 | -0.86234674 |
| Ercc6 | 0.00144 | 2.4347187 |
| Manba | 0.80369 | 0.0394783 |
| Acot8 | 0.00281 | -0.88541636 |
| Lbp | 0.00435 | -0.76708996 |
| Gm11961 | 0.19468 | -0.26939051 |
| Actl9 | 0.00767 | -1.36364815 |
| Drg1 | 0.98535 | 0.00448479 |
| Zfp788 | 0.00304 | 1.64255739 |
| Cyp2f2 | 0.00145 | -1.22376446 |
| Csnk1a1 | 0.0415 | 0.45213898 |
| Olfr1226 | 0.03016 | -0.94550042 |
| Lzts1 | 0.00222 | -0.91129996 |
| Mycbp2 | 0.00144 | 0.96699116 |
| Olfr943 | 0.0017 | -1.24950944 |
| Zdhhc16 | 0.39237 | -0.16867987 |
| Smn1 | 0.56848 | -0.12797325 |
| Slc1a5 | 0.44634 | 0.15182347 |
| Cipc | 0.00917 | 0.63619466 |
| Ros1 | 0.00974 | -1.31632485 |
| Lpgat1 | 0.15207 | -0.31324105 |
| Pnpt1 | 0.00144 | 1.48575327 |
| Kdm4d | 0.00145 | -1.18741414 |
| Uqcc3 | 0.0041 | -0.75368203 |
| Carkd | 0.10897 | -0.36003443 |
| Brinp3 | 0.00144 | -1.27992547 |
| Hexb | 0.04229 | -0.40307055 |
| Coa6 | 0.88904 | -0.05312107 |
| Usp9y | 0.00144 | -1.21977358 |
| Aacs | 0.01064 | -0.71080512 |
| Lrriq4 | 0.00145 | -1.2388121 |
| Vmn1r18 | 0.00145 | -1.22518835 |
| Iqgap2 | 0.55156 | 0.28266564 |
| Ssxb1 | 0.06116 | -0.90286462 |
| Zfp524 | 0.00201 | -1.04143796 |
| Pygl | 0.07511 | 0.35013491 |
| Cyp4f14 | 0.5356 | 0.26355873 |
| Pou5f2 | 0.00277 | 1.39178904 |
| Ssc5d | 0.00195 | -1.13801122 |
| Pdp1 | 0.01261 | 0.58613744 |
| Tlr2 | 0.28003 | -0.20492257 |
| Zfp459 | 0.73989 | -0.12805443 |
| Ildr2 | 0.03273 | 0.84083583 |
| Nes | 0.02893 | 0.60423428 |
| Otud6b | 0.00523 | 1.22233367 |
| Cmc4 | 0.00312 | 0.82597817 |
| Parp11 | 0.52988 | 0.116729 |
| Smad5 | 0.03674 | -0.47149001 |
| Vash2 | 0.00601 | -1.09465341 |
| Mzb1 | 0.83565 | 0.05620449 |
| Cpne6 | 0.09506 | -0.90018134 |
| Csnk1a1 | 0.01727 | 0.51248351 |
| Lrrc52 | 0.00429 | 1.31653282 |
| Oser1 | 0.59604 | 0.08708153 |
| Rab3il1 | 0.01252 | -0.90260899 |
| Plekhb1 | 0.0142 | -0.68743489 |
| Acot1 | 0.04435 | -0.92616205 |
| Plce1 | 0.00144 | -1.28116048 |
| Arhgef25 | 0.01373 | -0.5319949 |
| Anxa9 | 0.45183 | 0.42531396 |
| Mamdc2 | 0.85421 | -0.04637551 |
| Kng1 | 0.00152 | -1.25878908 |
| Klf6 | 0.01013 | 0.987539 |
| Jag2 | 0.00653 | -0.65251476 |
| Phactr3 | 0.33466 | -0.21061293 |
| 1810018F18Rik | 0.00144 | -1.28116048 |
| 2010204K13Rik | 0.00145 | -1.2388121 |
| Ube2c | 0.22988 | -0.59814168 |
| Brca2 | 0.02738 | 0.47697357 |
| Atp2a2 | 0.63636 | 0.10201773 |
| Pgm5 | 0.53522 | -0.12550772 |
| Anxa3 | 0.14335 | 0.35661217 |
| Olfr429 | 0.00144 | -1.30713006 |
| Acta2 | 0.71894 | 0.21186453 |
| P3h4 | 0.35973 | -0.33601241 |
| P3h4 | 0.01689 | -0.7553014 |
| Rtn4r | 0.12167 | -0.74258467 |
| Cog4 | 0.00858 | -0.60797338 |
| Gm34753 | 0.00144 | -1.29085005 |
| Pla2g4b | 0.17566 | -0.35970691 |
| Ptpn21 | 0.965 | -0.00943473 |
| Tepp | 0.07821 | -0.75387968 |
| Usp5 | 0.06208 | -0.71293825 |
| Padi3 | 0.05865 | -2.35840832 |
| 1700030K09Rik | 0.80592 | 0.04082085 |
| Tagln | 0.02239 | -0.92107727 |
| 2700029M09Rik | 0.68099 | 0.08929058 |
| Olfr835 | 0.00144 | -1.28375735 |
| Pnoc | 0.00144 | -1.27699683 |
| Ccdc74a | 0.00144 | -1.29878664 |
| Txnrd3 | 0.03229 | -0.40469017 |
| Thap3 | 0.01297 | -0.56252131 |
| 2010003K11Rik | 0.00176 | -1.24266807 |
| Esx1 | 0.00144 | -1.33059316 |
| Olfr593 | 0.00144 | -1.31518407 |
| Dnajb13 | 0.09361 | -1.10247618 |
| Mrpl36 | 0.36645 | -0.28478843 |
| 1110008F13Rik | 0.17108 | -0.44913797 |
| Olfr395 | 0.00145 | -1.26219879 |
| Cdk1 | 0.51491 | -0.26331551 |
| Higd2a | 0.13433 | -0.55910615 |
| Dnajc7 | 0.00165 | 1.38429866 |
| Ftsj3 | 0.05777 | 0.4317411 |
| Nyx | 0.02855 | 0.88987404 |
| Serp1 | 0.0195 | 0.52058154 |
| Pldi | 0.24218 | 1.17907198 |
| 1700006A11Rik | 0.00144 | -1.30713006 |
| Kif3c | 0.06992 | -0.41414289 |
| H2-Oa | 0.00144 | -1.34666234 |
| Thsd4 | 0.02526 | -0.94538552 |
| Olfr808 | 0.00144 | -1.27165604 |
| Trp53i13 | 0.00363 | -1.07912869 |
| Cndp2 | 0.56084 | -0.19376557 |
| Sncb | 0.05697 | -0.79833634 |
| Sertad2 | 0.03628 | -0.48725248 |
| 4930597L12Rik | 0.1873 | -0.58929915 |
| Fastkd5 | 0.00398 | 0.80364403 |
| Zfp90 | 0.63085 | -0.10584389 |
| Nrsn1 | 0.4753 | 0.32517393 |
| Tbxas1 | 0.9761 | -0.00843634 |
| Slc16a10 | 0.1445 | -0.54955713 |
| Mea1 | 0.03189 | -0.66103438 |
| Dnm3 | 0.5015 | 0.18734952 |
| Xpnpep1 | 0.11481 | -0.44572166 |
| Nkiras1 | 0.0338 | 0.39648056 |
| Cnbp | 0.01153 | 1.13168528 |
| Kctd8 | 0.80201 | 0.16943721 |
| Cer1 | 0.00144 | -1.29049509 |
| Gimap7 | 0.00144 | -1.27165604 |
| Cd22 | 0.13205 | -0.54810544 |
| Khdc3 | 0.16933 | -0.61050293 |
| Hyal4 | 0.03846 | -0.51626005 |
| Padi6 | 0.00247 | -1.41779016 |
| Zfp78 | 0.01132 | 1.81265336 |
| Mospd2 | 0.00144 | 2.50322835 |
| Cox6b1 | 0.21503 | -0.52379456 |
| Atp6ap1 | 0.67147 | 0.18227872 |
| Fam46c | 0.00525 | -1.13423233 |
| Kcnk16 | 0.00144 | -1.30017714 |
| Tgm4 | 0.58927 | -0.42438627 |
| Gm26844 | 0.00144 | -1.27165604 |
| Cyp2c55 | 0.70317 | 0.14630066 |
| Ankar | 0.00144 | -1.29085005 |
| Ptpn12 | 0.00144 | 1.16701933 |
| Plekhh2 | 0.03194 | -0.87533641 |
| Scube1 | 0.33198 | -0.18368601 |
| Igfbp1 | 0.00176 | -1.24674691 |
| Olfr919 | 0.00144 | -1.30713006 |
| Gtdc1 | 0.00511 | 0.99458836 |
| Slc25a22 | 0.44283 | 0.36578091 |
| Adra1a | 0.08699 | -0.50900622 |
| Rasa2 | 0.00144 | 1.5706114 |
| Ccdc83 | 0.00144 | -1.31627578 |
| 9930038B18Rik | 0.00144 | -1.26934956 |
| Cfdp1 | 0.00868 | 0.6733589 |
| Tmbim7 | 0.00202 | -1.37517696 |
| 5730409E04Rik | 0.22931 | -0.21024983 |
| Flnb | 0.0074 | 1.05450882 |
| 6430573F11Rik | 0.08909 | 0.55979599 |
| Zfp777 | 0.0085 | -0.68878137 |
| Vmn1r21 | 0.00145 | -1.24191372 |
| Bbox1 | 0.12444 | -1.36190996 |
| 1810026J23Rik | 0.35396 | 0.35549102 |
| Emp3 | 0.00144 | -1.0699971 |
| Cldnd1 | 0.01318 | -0.60512956 |
| Dok2 | 0.00329 | -1.00728467 |
| Ufl1 | 0.00144 | 1.33743336 |
| Csn2 | 0.00145 | -1.24191372 |
| Cdc37l1 | 0.0195 | 0.52567243 |
| Rpl18a | 0.00273 | -0.92695466 |
| Esf1 | 0.00453 | 2.18580806 |
| Ptchd3 | 0.56106 | -0.29768555 |
| Gipc2 | 0.07113 | 0.40574835 |
| 6430590A07Rik | 0.00865 | 1.33693373 |
| Lrrc38 | 0.56513 | 0.10115801 |
| Map10 | 0.67351 | 0.06753766 |
| Sbno1 | 0.06783 | -0.50646485 |
| D430042O09Rik | 0.40344 | 0.36951785 |
| Phf13 | 0.05565 | -0.3690209 |
| Dguok | 0.09805 | -0.4682797 |
| Slc12a3 | 0.15173 | -0.66049663 |
| Mcmbp | 0.95805 | 0.01440628 |
| Deptor | 0.02655 | 0.50128523 |
| Pirb | 0.02007 | -0.59944736 |
| 4931406C07Rik | 0.00424 | 0.8621633 |
| Hsd17b4 | 0.51919 | 0.14651606 |
| Chst10 | 0.79654 | -0.175749 |
| Arid1b | 0.57846 | 0.1689787 |
| Glra1 | 0.00144 | -1.49614311 |
| Mturn | 0.00144 | -1.21607873 |
| Fut7 | 0.0158 | -0.86373663 |
| Olfr1472 | 0.1149 | -2.81885782 |
| Olfr695 | 0.05015 | -3.45001542 |
| B130052P14Rik | 0.00719 | 1.15963024 |
| Tas2r116 | 0.00144 | -1.28898079 |
| Ly6g6d | 0.00144 | -1.29085005 |
| Ssmem1 | 0.00589 | -1.11030563 |
| Piwil1 | 0.00144 | -1.29864962 |
| Spry2 | 0.03137 | -0.51192582 |
| Olfr1277 | 0.06932 | -0.91089797 |
| Ikzf4 | 0.31967 | 0.25742586 |
| Tmem203 | 0.00818 | -0.82108937 |
| Serpina5 | 0.31748 | -0.44973035 |
| Otx1 | 0.01297 | -0.99644699 |
| Olfr574 | 0.08179 | -1.16184234 |
| C920025E04Rik | 0.00362 | -1.02401141 |
| Hydin | 0.00144 | -1.31014844 |
| Dbh | 0.0896 | -1.32320878 |
| Ccr4 | 0.00144 | -1.28898079 |
| Rnf219 | 0.00145 | -1.24191372 |
| Fam188b | 0.7519 | 0.13173678 |
| Cebpd | 0.00202 | -1.01961857 |
| Ptgr1 | 0.01044 | -0.78085074 |
| Swi5 | 0.07496 | -0.6773368 |
| Tanc1 | 0.01606 | 0.50300845 |
| Acp6 | 0.97232 | -0.01095896 |
| Mpc1 | 0.1561 | 0.49821318 |
| Slamf8 | 0.84678 | 0.1013354 |
| Frs3 | 0.12834 | -0.38867956 |
| Tmtc2 | 0.08097 | -0.85903142 |
| Syce1 | 0.51467 | -0.42050285 |
| Trmt11 | 0.00222 | 1.12663367 |
| Shroom1 | 0.00203 | -1.38047548 |
| Slu7 | 0.01868 | 0.71164317 |
| Pdzph1 | 0.00144 | -1.31495282 |
| Hivep2 | 0.00761 | 0.84327009 |
| Fgf16 | 0.12517 | -0.37018067 |
| Slc35c1 | 0.01272 | -0.80999358 |
| Mrps34 | 0.00145 | -1.12499197 |
| Gng7 | 0.34252 | -0.92517729 |
| Olfr1454 | 0.00144 | -1.24613586 |
| 0610011F06Rik | 0.04258 | -0.70131508 |
| Mum1 | 0.0202 | -0.52384945 |
| 1700001K19Rik | 0.00349 | -0.71389334 |
| Ppa1 | 0.48174 | 0.21435361 |
| Bccip | 0.69841 | 0.06472778 |
| 5830417I10Rik | 0.21579 | -0.2065864 |
| Cbll1 | 0.02796 | -0.46908548 |
| Trappc2l | 0.04313 | -0.67911956 |
| Acyp2 | 0.02891 | 0.79182124 |
| Ces2a | 0.19513 | -0.35315819 |
| Prps1l1 | 0.0043 | -1.11764773 |
| Olfr65 | 0.00144 | -1.28116048 |
| Aldh3b2 | 0.00151 | -1.6869987 |
| Arhgef19 | 0.3006 | -0.36702608 |
| Usp6nl | 0.02408 | 1.34734021 |
| Ano4 | 0.16718 | -0.60691711 |
| Ano4 | 0.19949 | -0.90923422 |
| Diap2 | 0.01754 | 0.9951178 |
| Specc1l | 0.32921 | 0.33497588 |
| Dnajc12 | 0.15622 | 0.69262609 |
| Slc36a1os | 0.10412 | -0.77074223 |
| Slc12a1 | 0.00144 | -1.27165604 |
| Ankrd42 | 0.45821 | 0.24140883 |
| Itfg1 | 0.05279 | 0.38473054 |
| Trp53tg5 | 0.32044 | -0.65217379 |
| Gm6583 | 0.06401 | -0.9676386 |
| Slc41a3 | 0.66744 | 0.23502688 |
| Itga2 | 0.0061 | -1.87316213 |
| Pip5k1a | 0.08486 | -0.46344714 |
| Stox2 | 0.63386 | -0.14305186 |
| Tial1 | 0.03483 | 0.64241241 |
| 4931423N10Rik | 0.03731 | -0.89043112 |
| Hsd17b2 | 0.04199 | -1.20381157 |
| Krt25 | 0.00144 | -1.29878664 |
| Fanci | 0.00269 | 1.09360935 |
| Tmem53 | 0.01069 | -1.00294482 |
| Micu2 | 0.85245 | 0.03938857 |
| E130119H09Rik | 0.01569 | -0.70579199 |
| Lrrc10 | 0.12151 | -0.43765518 |
| Olfr267 | 0.00144 | -1.26560808 |
| Vmn1r54 | 0.14744 | -0.84899822 |
| BC100451 | 0.4854 | -0.36205221 |
| Ptcd3 | 0.74471 | -0.12999731 |
| Foxred2 | 0.00144 | -1.30017714 |
| Pf4 | 0.01633 | -0.54638583 |
| Kansl1l | 0.00144 | 2.04952536 |
| Ttc5 | 0.00389 | 1.32521818 |
| Rpl37a | 0.00144 | -1.31111399 |
| Pcdh10 | 0.00144 | -1.28116048 |
| Olfr19 | 0.00145 | -1.2388121 |
| Gm16488 | 0.00144 | -1.22098834 |
| Thoc2 | 0.00426 | 1.45271068 |
| Fgf15 | 0.15206 | -2.17731617 |
| Ppfia1 | 0.10919 | 0.2992364 |
| Sh3pxd2a | 0.23597 | -0.19205698 |
| Dph2 | 0.39667 | -0.24266407 |
| Arfip1 | 0.00146 | 1.50463328 |
| Fam110b | 0.03791 | -0.60226361 |
| Mtrf1 | 0.00151 | 1.08839351 |
| Fgfr1op2 | 0.00208 | 0.86310567 |
| Crat | 0.07987 | -0.59583728 |
| Celsr1 | 0.36938 | -0.28499146 |
| Atg16l1 | 0.01066 | -0.60272966 |
| Cap1 | 0.83792 | -0.03913971 |
| Stk19 | 0.04599 | -0.45592487 |
| Mlkl | 0.26633 | 0.2380967 |
| Exosc5 | 0.00233 | -0.85511007 |
| Sox15 | 0.14973 | -0.31008832 |
| Bcap31 | 0.51962 | -0.12722441 |
| Frrs1 | 0.07057 | 0.41143494 |
| Efhc1 | 0.0724 | 0.54468624 |
| Pogk | 0.00544 | 0.75620056 |
| Ggt6 | 0.31713 | -0.71247905 |
| Prkg1 | 0.01634 | 0.49219307 |
| Gm9931 | 0.00144 | -1.28116048 |
| Fam187b | 0.00144 | -1.31627578 |
| Lgalsl | 0.0028 | 0.92219045 |
| Olfr1195 | 0.00158 | -1.2446827 |
| Gp5 | 0.00158 | -1.21223637 |
| Ggh | 0.04886 | 0.46363672 |
| Map4k4 | 0.1615 | 0.24558287 |
| Dnajc21 | 0.00644 | 1.16151225 |
| Gipc1 | 0.05586 | -0.66631993 |
| A330107A15Rik | 0.00144 | -1.40883618 |
| Map2k2 | 0.00973 | -0.79071506 |
| Rhov | 0.00199 | -1.36883861 |
| Tmprss12 | 0.00144 | -1.23532597 |
| Insig2 | 0.00876 | 0.97331848 |
| Padi1 | 0.49831 | -0.11629786 |
| Arhgap9 | 0.06719 | -0.56593263 |
| Relt | 0.06988 | -0.74868206 |
| Kcnh5 | 0.43024 | -0.84562043 |
| M6pr | 0.04351 | 0.38515919 |
| 1700024P04Rik | 0.07199 | -0.87222379 |
| Olfr911-ps1 | 0.00144 | -1.30017714 |
| Gpnmb | 0.01038 | -1.33351359 |
| Cmtm5 | 0.00339 | -1.15032504 |
| Olfr1246 | 0.09476 | -2.74121057 |
| Pmf1 | 0.10614 | -0.45087014 |
| Serpinb11 | 0.17239 | -0.62966415 |
| Ctnna2 | 0.00144 | -1.42249344 |
| Pycard | 0.14708 | -0.48400094 |
| Olfr93 | 0.02887 | -1.03595468 |
| Vti1b | 0.13026 | 0.30425868 |
| Keap1 | 0.00144 | -1.08746642 |
| Ipcef1 | 0.00592 | -1.07650415 |
| Sobp | 0.2494 | -0.22232608 |
| Cldn34c4 | 0.009 | -1.1398278 |
| Cyb5r1 | 0.52089 | -0.13971793 |
| Haus7 | 0.32568 | 0.16652938 |
| Kif1c | 0.00694 | -1.0926355 |
| Sil1 | 0.00144 | -1.12696439 |
| Arhgap22 | 0.0357 | -0.50497414 |
| Mapre2 | 0.56887 | 0.09723711 |
| Slc6a13 | 0.64829 | -0.34684359 |
| Fam133b | 0.10058 | 0.76106538 |
| Coa5 | 0.00203 | -0.83766378 |
| Mzf1 | 0.13454 | -0.38916123 |
| Kctd1 | 0.12351 | -0.53070001 |
| Rbm28 | 0.06469 | 0.34096047 |
| Zp3r | 0.00144 | -1.3348153 |
| Ube2dnl2 | 0.00158 | -1.2446827 |
| Tulp3 | 0.36883 | -0.19081319 |
| Gm5124 | 0.93533 | 0.0234838 |
| Zfp566 | 0.00414 | 1.0588294 |
| Nrros | 0.00996 | -0.57000673 |
| Ascl1 | 0.55593 | 0.31483355 |
| Oasl1 | 0.04002 | -0.62264325 |
| Gsk3a | 0.36467 | -0.24222895 |
| Emp2 | 0.73026 | 0.08127348 |
| Egr3 | 0.10249 | -0.70895209 |
| Dsc1 | 0.35599 | -0.42226218 |
| Lrp2bp | 0.00144 | -1.22925777 |
| Dynll2 | 0.04819 | -0.39961335 |
| Dab1 | 0.00152 | -1.19607921 |
| 5730559C18Rik | 0.00144 | -1.28116048 |
| Heg1 | 0.11071 | -1.02084269 |
| Cldn2 | 0.49985 | -0.21630852 |
| Trak1 | 0.29978 | 0.34724846 |
| Duox2 | 0.01458 | 1.15812732 |
| Rps9 | 0.01458 | -0.86150909 |
| Sertad1 | 0.0733 | -0.48928323 |
| Evi5l | 0.08104 | 0.62197426 |
| 5430405H02Rik | 0.00145 | -1.24191372 |
| Olfr1537 | 0.49431 | -0.23996403 |
| Arrb1 | 0.00419 | -0.86194881 |
| Eftud2 | 0.49034 | -0.18407161 |
| Ccl7 | 0.13638 | 0.88223241 |
| Tmc2 | 0.63314 | -0.24058174 |
| Lingo3 | 0.71303 | 0.22219762 |
| Kin | 0.00144 | 1.5581216 |
| AI317395 | 0.05388 | 0.54822534 |
| Zfp871 | 0.01061 | 0.63139761 |
| Qrich1 | 0.23806 | -0.20409744 |
| Hoxb5 | 0.01833 | -1.9170679 |
| Grin2c | 0.23566 | -0.58444936 |
| Olfr790 | 0.00144 | -1.25118919 |
| Gart | 0.86612 | -0.05311807 |
| Nudt22 | 0.006 | -0.93855294 |
| Gpr182 | 0.00144 | -1.46311027 |
| Necap2 | 0.00402 | -0.82616927 |
| Hivep1 | 0.10252 | 0.35139401 |
| Tasp1 | 0.00403 | 0.86007371 |
| Tnfaip8 | 0.00144 | 1.30186768 |
| Med12 | 0.00158 | 0.95656684 |
| Rsph9 | 0.00144 | -1.19447188 |
| Nr2e3 | 0.75414 | -0.31138452 |
| Ccdc33 | 0.00145 | 1.86295056 |
| Zbtb8a | 0.38279 | -0.36301943 |
| Amfr | 0.13834 | -0.35422915 |
| D930027P08Rik | 0.13641 | 0.54694162 |
| Arhgef40 | 0.03538 | -0.93280023 |
| Olfr1178 | 0.00144 | -1.27730451 |
| Sprr2d | 0.00144 | -1.30017714 |
| Actl10 | 0.00158 | -1.2446827 |
| Ppfia2 | 0.06823 | -0.91763571 |
| Grid2ip | 0.00144 | -1.26560808 |
| Col26a1 | 0.03211 | -0.85403919 |
| Lipe | 0.07604 | -0.48822591 |
| Tmem159 | 0.90159 | 0.03034697 |
| Epor | 0.00861 | -0.91732273 |
| Ap1s2 | 0.00537 | 1.04664756 |
| Cul4a | 0.01387 | 0.66044948 |
| Asap1 | 0.00144 | 1.04131739 |
| Gm36283 | 0.00144 | -1.29051721 |
| Cryz | 0.87324 | -0.03190409 |
| Acadsb | 0.00144 | 2.04083205 |
| Lmbr1l | 0.03805 | -0.44152913 |
| Olfr114 | 0.00144 | -1.46433706 |
| Jmjd8 | 0.34235 | -0.28261736 |
| Fam83e | 0.00654 | -0.69938939 |
| BC023105 | 0.00152 | -1.25878908 |
| 1700019O17Rik | 0.13938 | -0.74338418 |
| Ube2e2 | 0.12391 | -0.3242598 |
| Gm6607 | 0.00144 | -1.08395336 |
| Qk | 0.03685 | 0.40094397 |
| Dtwd1 | 0.8376 | 0.06560058 |
| Hpn | 0.48649 | -0.1245858 |
| Olfr1184 | 0.00144 | -1.32766452 |
| Mcoln2 | 0.02989 | -0.91202478 |
| Mrpl11 | 0.0238 | -0.63418887 |
| Anapc4 | 0.34091 | 0.20657958 |
| Olfr1504 | 0.00145 | -1.25646236 |
| Myo19 | 0.00389 | 1.46410307 |
| Herpud1 | 0.8703 | -0.0448739 |
| Gja6 | 0.00144 | -1.20030135 |
| Gsap | 0.22108 | 0.35409476 |
| 4930548G14Rik | 0.00144 | -1.26560808 |
| 4930524N10Rik | 0.00767 | -1.36425238 |
| Vegfc | 0.45977 | 0.15855751 |
| Tpi1 | 0.03596 | -0.67598463 |
| Agr3 | 0.00144 | -1.25646236 |
| Tert | 0.16779 | 0.36456982 |
| Olfr867 | 0.00212 | -1.42249344 |
| Nucb1 | 0.11095 | -0.38878491 |
| Vmn1r212 | 0.00144 | -1.30713006 |
| Klhl6 | 0.32703 | -0.22215117 |
| Bcas3 | 0.01033 | -0.7144868 |
| Nme7 | 0.00321 | 0.91741883 |
| Tut1 | 0.05073 | -0.44178463 |
| Krt17 | 0.22716 | -0.22282997 |
| Phc1 | 0.90023 | -0.03323443 |
| 1700019A02Rik | 0.55518 | -0.27137782 |
| Gprc5d | 0.10939 | -1.18292847 |
| Vmn1r236 | 0.00144 | -1.37964577 |
| Bcas1 | 0.02418 | -2.91270969 |
| Rdm1 | 0.01403 | -0.97504496 |
| Purb | 0.00144 | 1.46784201 |
| 4930510E17Rik | 0.00218 | -1.18878227 |
| Ppapdc2 | 0.00269 | 0.82841637 |
| Chmp4c | 0.00405 | 1.02594413 |
| Sstr1 | 0.00144 | -1.28898079 |
| Npas3 | 0.00999 | -1.61033016 |
| Dhrs11 | 0.30138 | 0.46031121 |
| Rbm4 | 0.04612 | -0.4919007 |
| Tmem231 | 0.21474 | -0.75145126 |
| Wdr31 | 0.21334 | -0.33197514 |
| Itgb2l | 0.02872 | -1.91379509 |
| Pex7 | 0.05104 | 0.73079759 |
| Spink6 | 0.00149 | -1.19348234 |
| Tlr9 | 0.04032 | -0.44053697 |
| Wrnip1 | 0.46147 | -0.14339127 |
| Lace1 | 0.03429 | 0.90954691 |
| Gnai2 | 0.00158 | -0.87940306 |
| Tefm | 0.10401 | -0.82107543 |
| Ppp1r14a | 0.00144 | -1.47605723 |
| Aplp1 | 0.0205 | -0.48645524 |
| Rnf183 | 0.14756 | -2.49876633 |
| Gstt4 | 0.09288 | -0.86648736 |
| Sap30 | 0.83398 | 0.06271617 |
| Slc16a6 | 0.87935 | 0.03381594 |
| Trrap | 0.90341 | 0.05304839 |
| Gm9744 | 0.70979 | -0.21502783 |
| B9d1os | 0.00144 | 1.14732708 |
| Gfod1 | 0.16958 | -0.68146471 |
| Pdcd6 | 0.05233 | 0.49384737 |
| Map1s | 0.00875 | -0.67232175 |
| Uqcrh | 0.00295 | -0.77277819 |
| Hmha1 | 0.17094 | -0.29700436 |
| Myom2 | 0.72317 | 0.07756833 |
| Ndufa3 | 0.18726 | -0.60957651 |
| Cth | 0.31936 | 0.76814797 |
| Fam185a | 0.04789 | 0.95741979 |
| Celf1 | 0.92063 | 0.01706492 |
| Ten1 | 0.34988 | -0.40741101 |
| Prss56 | 0.00149 | -1.28183002 |
| Rab11fip3 | 0.05 | -0.50039707 |
| Prl3a1 | 0.6094 | 0.37500819 |
| Car3 | 0.97059 | 0.04206289 |
| St8sia3 | 0.00145 | -1.22376446 |
| Ubash3a | 0.0903 | -2.66394886 |
| Selenbp2 | 0.14709 | -0.44916704 |
| Cntrl | 0.00577 | 0.69984522 |
| Paqr7 | 0.05342 | -0.9055509 |
| Rdx | 0.00152 | 1.56665243 |
| Fam20a | 0.00479 | -0.73959766 |
| Gm3643 | 0.02441 | 1.24736386 |
| Il10 | 0.15332 | -0.76914284 |
| 4931402H11Rik | 0.00144 | -1.28116048 |
| Slco6d1 | 0.00144 | -1.30713006 |
| Gstm6 | 0.64191 | 0.21332126 |
| Gpr33 | 0.00152 | -1.25878908 |
| Slc25a12 | 0.22463 | 0.40642874 |
| Slc46a1 | 0.08708 | -0.58715062 |
| G6pc3 | 0.01075 | -0.85375199 |
| Commd5 | 0.00144 | -1.11302723 |
| Alox8 | 0.11603 | -1.86758854 |
| Ada | 0.00742 | -0.70949215 |
| Olfr745 | 0.00538 | -1.52832083 |
| Dnajc30 | 0.73364 | -0.14942935 |
| Lrp10 | 0.04941 | -0.65982282 |
| Gmpr2 | 0.00834 | 0.84185874 |
| Cfap126 | 0.06654 | -0.55272262 |
| Anxa3 | 0.02118 | -0.95131328 |
| Gpd1l | 0.00144 | -1.63686777 |
| Tst | 0.17583 | -0.44317553 |
| Hist2h2be | 0.22791 | 0.27021574 |
| Ccdc13 | 0.15338 | -0.69106903 |
| Ptafr | 0.30849 | -0.42670264 |
| Supt5 | 0.03337 | -0.41489399 |
| Ndp | 0.00145 | -1.30919717 |
| Msh5 | 0.00314 | -1.14703327 |
| Msh5 | 0.70327 | -0.15905114 |
| Fcer1a | 0.55111 | -0.30921639 |
| Dennd2c | 0.00196 | -1.22750594 |
| Ebp | 0.00155 | -0.96926857 |
| Msx1os | 0.06265 | -2.30124649 |
| Izumo3 | 0.00144 | -1.53315402 |
| 1700001G17Rik | 0.72633 | -0.12617054 |
| 4833420G17Rik | 0.0268 | -0.56935887 |
| Fam188a | 0.00144 | 1.1208133 |
| Hes6 | 0.05564 | -0.51259984 |
| Prss16 | 0.00144 | -1.29746368 |
| Neto2 | 0.0211 | 0.76683637 |
| Pus10 | 0.01303 | 1.01080364 |
| Tbc1d25 | 0.00154 | -1.12191763 |
| Prok2 | 0.00144 | -1.29085005 |
| Smpd1 | 0.00685 | -0.96982848 |
| Cdc5l | 0.00258 | 0.84045022 |
| Gm16551 | 0.43181 | -0.48931634 |
| 4933411G06Rik | 0.00144 | -1.20579466 |
| Hmgxb4 | 0.00994 | 1.83336693 |
| Pbx4 | 0.00463 | -1.34827422 |
| Grik5 | 0.01817 | -0.59615732 |
| Pus7l | 0.97258 | 0.00820663 |
| Mrpl13 | 0.49334 | 0.1378283 |
| C4b | 0.00144 | -1.6090514 |
| Gm12132 | 0.00144 | -1.27165604 |
| Cspp1 | 0.73815 | -0.32496967 |
| Fam134b | 0.69108 | 0.13329535 |
| Oas1c | 0.31772 | -0.29359909 |
| Cldn16 | 0.00144 | -1.30713006 |
| Fgb | 0.0222 | -1.41315531 |
| Ttyh1 | 0.57333 | -0.29269736 |
| Ttyh1 | 0.0049 | -1.08617297 |
| Ppbp | 0.0251 | -0.90790694 |
| Psma6 | 0.15085 | 0.45464395 |
| Retnlb | 0.15557 | -0.56706712 |
| Mbnl1 | 0.02561 | 0.54744592 |
| 4930539N22Rik | 0.01853 | -0.90776023 |
| 1700084M14Rik | 0.00144 | -1.31884045 |
| Smchd1 | 0.00372 | -1.14963365 |
| Lrig3 | 0.05403 | -0.49637274 |
| Tnpo3 | 0.52183 | -0.26528643 |
| Sptbn1 | 0.03345 | -0.47277617 |
| Lmod3 | 0.06704 | 0.3231428 |
| Olfr869 | 0.00144 | -1.28116048 |
| Megf8 | 0.00194 | -0.88283713 |
| Smad6 | 0.00996 | -0.6795546 |
| Mlip | 0.06565 | 0.55006396 |
| Mlip | 0.05207 | 0.62485429 |
| Diap1 | 0.12548 | -0.31500049 |
| Cpt1a | 0.02757 | 0.5243042 |
| Cnn2 | 0.05033 | -0.39670544 |
| Pgc | 0.00149 | -1.22582153 |
| Tbx20 | 0.34637 | -0.26626212 |
| Lppr2 | 0.85011 | -0.21236838 |
| Arrdc5 | 0.05144 | -1.52594014 |
| Pgm1 | 0.91092 | -0.05829699 |
| Thsd1 | 0.01419 | -0.57140546 |
| Mxra8 | 0.00624 | -0.6976107 |
| Snx3 | 0.7836 | 0.11173111 |
| Grhpr | 0.10377 | -0.58131107 |
| Slc25a38 | 0.53774 | -0.22514644 |
| Edrf1 | 0.58134 | -0.09697034 |
| Ctsj | 0.12891 | -0.88303095 |
| Adgrl1 | 0.00591 | -0.7881376 |
| Zbed5 | 0.04419 | 0.88286605 |
| Pex19 | 0.3357 | -0.38525382 |
| Lacc1 | 0.0502 | 1.11869729 |
| Nadk | 0.0747 | -0.31857882 |
| Clcc1 | 0.05826 | -0.79283721 |
| Pla2g2c | 0.15155 | -0.79411719 |
| 1700020I14Rik | 0.10239 | -0.56947219 |
| Hist2h4 | 0.00144 | -1.36395892 |
| Gdpd3 | 0.73858 | 0.1331512 |
| Olfr801 | 0.09878 | -1.09371416 |
| Atp4a | 0.97652 | 0.01080716 |
| Anxa5 | 0.50304 | 0.10273967 |
| Olfr365 | 0.00144 | -1.28116048 |
| Dnttip2 | 0.0152 | 1.11686292 |
| Cdcp1 | 0.00905 | -1.10278386 |
| Sult3a1 | 0.00144 | -1.31495282 |
| Cib4 | 0.10152 | -1.11957445 |
| Ucp1 | 0.00144 | -1.29746368 |
| Tssk1 | 0.94171 | 0.02030072 |
| Dhx30 | 0.02323 | -0.54029075 |
| Mgp | 0.00785 | -0.69529647 |
| Anks3 | 0.81667 | 0.04418015 |
| Olfr1116 | 0.00413 | -1.71951531 |
| Src | 0.23956 | -0.822106 |
| Nppb | 0.04895 | -0.87128049 |
| Mmp7 | 0.00145 | -1.18741414 |
| Khdrbs3 | 0.06107 | -0.35171108 |
| Ulbp1 | 0.00144 | 2.00573643 |
| Lztfl1 | 0.00144 | 1.85776198 |
| Fam132a | 0.11635 | -0.41583954 |
| Tbc1d7 | 0.51757 | 0.27038902 |
| Nt5c2 | 0.05739 | 0.6967222 |
| Ivd | 0.89644 | 0.04366643 |
| Mad2l1bp | 0.05855 | -0.55388218 |
| Mmp20 | 0.00144 | -1.27165604 |
| Kif27 | 0.02531 | -1.95327411 |
| Smg9 | 0.00937 | -0.64538106 |
| Jph4 | 0.07233 | -0.41065025 |
| C1qtnf3 | 0.08667 | -2.20244344 |
| Olfr1444 | 0.00632 | -0.74386126 |
| Zfp384 | 0.93816 | -0.05624763 |
| Xiap | 0.00713 | 0.99087069 |
| Fam213b | 0.00144 | -1.12185882 |
| Ahsg | 0.25473 | -0.22873771 |
| Ncapg2 | 0.06888 | 1.29298564 |
| Sf3b6 | 0.12566 | -0.30912328 |
| Wdr54 | 0.98779 | -0.0119038 |
| Nxph4 | 0.01131 | -0.61099584 |
| Sirt4 | 0.63602 | -0.11502491 |
| Zfp93 | 0.20626 | 0.30477719 |
| Vnn1 | 0.00149 | -1.2703832 |
| Nin | 0.0019 | 0.95110633 |
| Txn1 | 0.52705 | -0.14448009 |
| Cnih3 | 0.0017 | -1.24950944 |
| Nqo1 | 0.30633 | -0.26515996 |
| Olfr141 | 0.01026 | -0.80025924 |
| Mt4 | 0.15219 | -0.51980649 |
| Mecp2 | 0.00867 | 0.68689565 |
| Fxyd3 | 0.05966 | -0.96435792 |
| Rnaseh2a | 0.00533 | -0.88917844 |
| Rnaseh2a | 0.98321 | -0.00935009 |
| Btbd2 | 0.00294 | -0.9313 |
| Ctss | 0.04439 | 0.53001 |
| Crem | 0.00631 | 1.0853619 |
| Tas1r3 | 0.04749 | -0.65023383 |
| Cnga2 | 0.05478 | 0.89825975 |
| Dach2 | 0.00144 | -1.29051721 |
| Smarcd3 | 0.00719 | -0.73859023 |
| 4632433K11Rik | 0.76086 | 0.05459037 |
| Capn3 | 0.54352 | 0.39575536 |
| Morn1 | 0.53714 | -0.12862927 |
| Alpi | 0.00144 | -1.28898079 |
| Pfdn4 | 0.02848 | -0.62833263 |
| Cldn6 | 0.00353 | -1.08965911 |
| Spsb1 | 0.13635 | -0.49218402 |
| Fam84a | 0.36691 | 0.44305042 |
| Fam160a2 | 0.34086 | -0.26585465 |
| Fbxo17 | 0.03858 | -0.92260074 |
| Mrpl21 | 0.00147 | -0.85922785 |
| Slfn2 | 0.02076 | -0.52230163 |
| Shbg | 0.12269 | -3.18792276 |
| Amph | 0.02414 | -0.88685044 |
| 2310003H01Rik | 0.13158 | -0.44574541 |
| Ikbkg | 0.00653 | 1.05011805 |
| Gm20758 | 0.03992 | -0.90748867 |
| Fer | 0.04998 | 0.82556987 |
| Katnal2 | 0.05628 | -0.94104075 |
| Mmrn1 | 0.32459 | 0.33994968 |
| Gba | 0.04706 | -0.49696712 |
| Flot1 | 0.17863 | -0.41974228 |
| Klhl7 | 0.00144 | 1.0494165 |
| Olfr535 | 0.00144 | -1.31884045 |
| Tmem14a | 0.61377 | -0.09303852 |
| Rars2 | 0.00323 | 1.35787947 |
| Clca1 | 0.0369 | -0.92709582 |
| Mrgbp | 0.22188 | -0.23207317 |
| Zmat4 | 0.08588 | 0.4454968 |
| Tbpl1 | 0.28209 | 0.19681705 |
| Tbpl1 | 0.20297 | 0.21747294 |
| Arl11 | 0.13425 | -1.00729694 |
| Paqr8 | 0.26027 | 0.26933152 |
| Vmn1r206 | 0.18926 | -1.95842575 |
| Odf3b | 0.13417 | -0.57646862 |
| Pth2r | 0.00144 | -1.27699683 |
| Zfp420 | 0.00201 | 1.72284347 |
| 1700006J14Rik | 0.00334 | -0.96390278 |
| Dpagt1 | 0.09542 | -0.38394594 |
| Pxt1 | 0.59249 | -0.24234201 |
| Elp4 | 0.06607 | 0.56791968 |
| Csn1s2a | 0.00144 | -1.29085005 |
| Ocel1 | 0.00275 | -1.030479 |
| Timm22 | 0.04081 | -0.73820656 |
| Irf7 | 0.003 | -0.94861081 |
| Hspa14 | 0.01013 | 0.70743776 |
| Timm9 | 0.03314 | 0.87600842 |
| Shroom4 | 0.20454 | 0.7748002 |
| Slc12a7 | 0.42468 | -0.26448853 |
| Zfp383 | 0.00678 | 0.81536802 |
| Tmem123 | 0.00144 | 1.75351826 |
| Bspry | 0.23045 | -0.41025738 |
| Cdcp2 | 0.00221 | -1.10308723 |
| Slc9b2 | 0.10612 | -0.65072139 |
| Zdhhc11 | 0.00144 | -1.27165604 |
| Hsd3b1 | 0.31107 | 0.33109763 |
| Caly | 0.03592 | -0.93414486 |
| Fv1 | 0.08465 | -1.70241888 |
| 2010107G12Rik | 0.00145 | -1.2388121 |
| Tubb6 | 0.10702 | -0.50795712 |
| Ascc3 | 0.00286 | 0.95773522 |
| Wdr12 | 0.89227 | 0.03322406 |
| Slc30a3 | 0.00144 | -1.41443942 |
| Lrrc8b | 0.12175 | 0.44428259 |
| Mblac2 | 0.52284 | 0.21269232 |
| Olfr24 | 0.06442 | 0.44294917 |
| Reep4 | 0.18904 | -0.21478468 |
| Olfr983 | 0.00144 | -1.27165604 |
| Clic5 | 0.01729 | 0.69300335 |
| Olfr1338 | 0.00144 | -1.29746368 |
| Srd5a1 | 0.48729 | -0.14947567 |
| Ftmt | 0.15353 | -0.75486497 |
| Srrt | 0.06142 | 0.47553389 |
| Ccr7 | 0.23091 | -0.72555066 |
| Fam219aos | 0.24541 | -1.01541182 |
| Emc2 | 0.00144 | 1.36398506 |
| Ebag9 | 0.20875 | -0.28295683 |
| Tmem106b | 0.00145 | 1.30224447 |
| Eaf1 | 0.00925 | 0.63496263 |
| Arhgef40 | 0.04552 | -0.54326289 |
| BB085087 | 0.13225 | 0.55676128 |
| Ptprs | 0.00319 | -0.81954584 |
| 4921509C19Rik | 0.00144 | -1.27454685 |
| Klk1b27 | 0.00144 | -1.28898079 |
| Dclk3 | 0.68042 | -0.37327423 |
| Pkd2 | 0.04894 | 0.5017039 |
| Polr2d | 0.30138 | -0.31343897 |
| 4931400O07Rik | 0.00144 | -1.28116048 |
| Olfr641 | 0.08815 | -2.62590741 |
| Hsf3 | 0.00144 | -1.26560808 |
| Gnmt | 0.74253 | -0.12211741 |
| Foxb2 | 0.00145 | -1.25646236 |
| Pdcd2l | 0.21839 | -0.3717131 |
| Batf3 | 0.04396 | -0.62918536 |
| Rnmt | 0.00598 | 0.97522124 |
| 5830416P10Rik | 0.0017 | -1.36238117 |
| S100a14 | 0.22407 | -0.261509 |
| Fancg | 0.05898 | -0.42582237 |
| Rab15 | 0.61419 | -0.16340539 |
| 2310007L24Rik | 0.64731 | 0.39134313 |
| Mdn1 | 0.00753 | 0.6028566 |
| Pde10a | 0.01992 | 0.59925596 |
| Hspb3 | 0.01101 | -0.68431523 |
| Neurl2 | 0.00145 | -1.21276951 |
| Prss2 | 0.15597 | -0.70012802 |
| Tfap2c | 0.00144 | -1.29085005 |
| Paqr3 | 0.00239 | 1.13077247 |
| Rbm33 | 0.04091 | -0.41904855 |
| Plekhf1 | 0.9176 | -0.05075947 |
| Vmn1r38 | 0.02495 | -1.83972784 |
| Vps37c | 0.27059 | -0.41035516 |
| Sfxn1 | 0.62414 | 0.07754081 |
| Robo2 | 0.24871 | -0.63437949 |
| Defb13 | 0.01053 | -1.36567627 |
| Lrrc18 | 0.00149 | -1.19348234 |
| Jam2 | 0.15026 | 0.2803635 |
| Gpr87 | 0.00144 | -1.28898079 |
| Mov10l1 | 0.63187 | 0.31081921 |
| Nudcd2 | 0.72826 | -0.06035282 |
| Mis12 | 0.02159 | 0.89866772 |
| Cd80 | 0.66415 | -0.38474144 |
| Pccb | 0.3729 | 0.40233966 |
| Odf2l | 0.00144 | 2.84742084 |
| Manf | 0.90732 | -0.03030358 |
| Fn3krp | 0.3179 | -0.52959474 |
| Tmem119 | 0.00216 | -1.00536439 |
| Sc5d | 0.19681 | 0.22335868 |
| 1700016A09Rik | 0.27126 | -0.60336465 |
| Ednra | 0.00144 | -1.28116048 |
| Sema6b | 0.00144 | -1.31136456 |
| Trim10 | 0.0368 | -0.98360257 |
| Smyd5 | 0.18486 | -0.43906668 |
| Rab20 | 0.71903 | 0.20252091 |
| Hdac4 | 0.00421 | -0.81125447 |
| Cabp7 | 0.00699 | -1.50342899 |
| Mrgpra3 | 0.02611 | -0.94534324 |
| Prop1 | 0.01838 | -1.2171999 |
| Kap | 0.00144 | -1.55489656 |
| Pik3r2 | 0.00314 | -0.78869297 |
| Itga11 | 0.05468 | -0.58937844 |
| Apaf1 | 0.00753 | 1.20334203 |
| Jmjd1c | 0.07819 | 0.37570492 |
| Olfr1445 | 0.21281 | -0.53337895 |
| Aktip | 0.95989 | -0.00929813 |
| Rnaseh2c | 0.00144 | -1.30511507 |
| Zfp236 | 0.15292 | 0.32660098 |
| Taf9 | 0.00615 | 1.38757351 |
| 0610012G03Rik | 0.83638 | -0.10220311 |
| Ppp1r2 | 0.00776 | 2.28328291 |
| 6330403K07Rik | 0.18007 | -1.23534012 |
| Sap30bp | 0.08381 | -0.31994305 |
| Zfp51 | 0.00144 | 2.22713921 |
| Arhgap8 | 0.06606 | -0.36360364 |
| Tuft1 | 0.79028 | 0.07068344 |
| Tpst1 | 0.19978 | -0.24594572 |
| Ube2j2 | 0.75765 | -0.10462333 |
| Slc36a4 | 0.99775 | -0.00061893 |
| Nedd8 | 0.07518 | -0.53236957 |
| Myl1 | 0.13574 | -0.43341507 |
| Tspan15 | 0.04177 | -1.53109372 |
| Ranbp3l | 0.00144 | -1.26560808 |
| Lsamp | 0.04765 | -0.66381445 |
| Ext1 | 0.97164 | -0.0063093 |
| Krt1 | 0.00144 | -1.31495282 |
| 1810058I24Rik | 0.35484 | -0.27080935 |
| Ssr3 | 0.00206 | 1.02607238 |
| Ctsg | 0.52441 | -0.56903935 |
| Hist1h1a | 0.56254 | -0.21244316 |
| Calr | 0.43042 | -0.15348993 |
| Tmc4 | 0.54957 | 0.32915573 |
| Gje1 | 0.03883 | -1.99019407 |
| Dnah9 | 0.01082 | -1.24231203 |
| Irgm2 | 0.89841 | 0.02961468 |
| 4930428F12Rik | 0.00144 | -1.28116048 |
| Sirt6 | 0.17723 | -0.39009242 |
| Slc31a2 | 0.00144 | 1.39626722 |
| Dirc2 | 0.02355 | -0.53207298 |
| Rtp1 | 0.07148 | -2.74064717 |
| Psma2 | 0.92523 | -0.0171857 |
| Pola1 | 0.00144 | 1.17017914 |
| Il6ra | 0.06823 | 0.54657391 |
| Tusc1 | 0.06337 | -0.48281271 |
| Tmtc1 | 0.02341 | 0.56860263 |
| Sema6d | 0.89655 | -0.04597117 |
| Gng12 | 0.79191 | 0.06450359 |
| Gdf5 | 0.23285 | -0.70132398 |
| Fkbp7 | 0.99046 | 0.00210944 |
| Defb6 | 0.02967 | -0.6073339 |
| C2cd4b | 0.11478 | -0.6106033 |
| 4930563D23Rik | 0.38757 | -0.5293674 |
| Zfp105 | 0.01186 | 0.70578501 |
| Zmat3 | 0.5187 | 0.10899386 |
| Tgfa | 0.00351 | -1.40660463 |
| Ints6 | 0.00154 | 1.30549431 |
| Ifi35 | 0.00462 | -0.69619189 |
| Faf1 | 0.69653 | 0.06873309 |
| Olfr552 | 0.00145 | -1.22376446 |
| Mark2 | 0.00493 | -0.82045705 |
| Defb15 | 0.00144 | -1.27165604 |
| Ddt | 0.51156 | -0.18432172 |
| Plvap | 0.0021 | -0.86304944 |
| Casp7 | 0.13044 | -0.27009605 |
| Smim7 | 0.07151 | -0.41031483 |
| Dynll1 | 0.00911 | -0.709524 |
| Naa15 | 0.0023 | 0.82340474 |
| Mmrn2 | 0.00284 | -0.89863288 |
| LOC102638268 | 0.00353 | -1.08965911 |
| A130010J15Rik | 0.28676 | -0.16344987 |
| Grm2 | 0.14235 | -0.6426468 |
| Rab19 | 0.0063 | -1.13941301 |
| Naprt | 0.00683 | -0.77547606 |
| Gm41572 | 0.00144 | 1.93339363 |
| Pgrmc2 | 0.03823 | 0.48047818 |
| Hmx3 | 0.03323 | -1.25694302 |
| Olfr644 | 0.52848 | 0.8641866 |
| Cfb | 0.00275 | -1.45910338 |
| Pramef12os | 0.00149 | -1.22582153 |
| Ftcd | 0.00377 | -1.55726529 |
| Ap3d1 | 0.16725 | -0.30829218 |
| Mrpl20 | 0.00543 | -0.91762072 |
| Cox19 | 0.01928 | -0.72826008 |
| Txndc11 | 0.3007 | -0.17848162 |
| 2010109A12Rik | 0.48902 | -0.26412115 |
| Parg | 0.02069 | 0.67098141 |
| Zfp42 | 0.00144 | -1.31495282 |
| Ezr | 0.53506 | 0.16568171 |
| Tpo | 0.00144 | -1.30017714 |
| Cep164 | 0.01153 | -1.28357978 |
| Zfp654 | 0.00383 | 1.07785856 |
| Gm4980 | 0.00615 | -0.71355692 |
| Olfr1016 | 0.00144 | -1.36878954 |
| Zdhhc8 | 0.00218 | -0.82476401 |
| Klk1b3 | 0.97196 | -0.02072047 |
| Myl4 | 0.16453 | -2.30093389 |
| Ppm1l | 0.01039 | 0.64700107 |
| Ints2 | 0.00154 | 0.87040835 |
| C130060K24Rik | 0.01694 | -2.44780261 |
| Chn2 | 0.16736 | -0.27287715 |
| Rnf157 | 0.0018 | -1.21153109 |
| Lrrn1 | 0.0042 | 0.99101098 |
| Krt27 | 0.01892 | -0.81246844 |
| Efs | 0.00144 | -1.01377481 |
| Olfr1101 | 0.00442 | -1.92597591 |
| Olfr862 | 0.00145 | -1.27532353 |
| Daxx | 0.00158 | -0.88432986 |
| Dqx1 | 0.00144 | -1.28375735 |
| Cebpz | 0.13137 | 0.60768293 |
| Ccdc51 | 0.85602 | -0.07616369 |
| Eef1b2 | 0.19014 | -0.33332488 |
| Tmem182 | 0.23507 | 0.31995924 |
| Marf1 | 0.00294 | 1.32000604 |
| Irx4 | 0.54848 | -0.26399691 |
| Slc4a11 | 0.00144 | -1.46929575 |
| Antxrl | 0.30815 | -0.52935082 |
| Chst4 | 0.20182 | -0.53094138 |
| Vps41 | 0.00771 | 0.72083759 |
| Tbc1d16 | 0.00144 | -1.39981165 |
| Cxcr6 | 0.20453 | 0.9469406 |
| Slc6a20a | 0.00144 | -1.29085005 |
| Arhgap1 | 0.67641 | -0.09448391 |
| Bmp8b | 0.00144 | -1.27165604 |
| Sirt5 | 0.53829 | -0.18614053 |
| Olfr368 | 0.01481 | -0.81796175 |
| Nip7 | 0.04931 | -0.51398341 |
| Ankrd13b | 0.01753 | -0.70379464 |
| Carns1 | 0.65592 | -0.10741079 |
| Trpc6 | 0.01708 | -1.09513407 |
| Taf1c | 0.01347 | -0.54636645 |
| Mogat2 | 0.25146 | 0.73989913 |
| Olfr61 | 0.00144 | -1.20579466 |
| Sprr2k | 0.43429 | -0.17246465 |
| Nup50 | 0.14293 | -0.31361749 |
| Nfe2l2 | 0.00144 | 1.18925575 |
| Txndc16 | 0.00588 | 1.16529499 |
| Pea15a | 0.00341 | -0.8819744 |
| Mospd1 | 0.0395 | 0.72020402 |
| Iyd | 0.00144 | -1.26934956 |
| Rpp25l | 0.00512 | -0.95017595 |
| Tmem127 | 0.05807 | -0.39937943 |
| Polr3g | 0.00974 | 1.46323451 |
| Gm9856 | 0.90336 | 0.04657987 |
| Tor4a | 0.00144 | -1.02836199 |
| Upf3a | 0.06314 | 0.3400133 |
| Rhcg | 0.14136 | -0.89155079 |
| Dgke | 0.0048 | 0.75507892 |
| Irx5 | 0.00258 | -0.83740003 |
| Dpp9 | 0.80892 | 0.07661932 |
| Tube1 | 0.62747 | -0.15457172 |
| Ptch2 | 0.60554 | -0.12884891 |
| Clcn6 | 0.3955 | -0.17837137 |
| Pip5k1b | 0.43744 | 0.17422313 |
| Fancd2os | 0.00144 | -1.28898079 |
| Hsd11b2 | 0.04042 | -2.46974983 |
| Arl13b | 0.11459 | 0.33792333 |
| Prkar2a | 0.06495 | 0.95699256 |
| Vmn1r43 | 0.00144 | -1.30713006 |
| Lyrm7 | 0.35312 | -0.37428558 |
| Zfp112 | 0.11829 | 0.30985176 |
| Psrc1 | 0.27818 | -0.37312722 |
| Chrm5 | 0.00144 | -1.91759367 |
| 4930505A04Rik | 0.00318 | -1.16999514 |
| Ostf1 | 0.12412 | -0.73276558 |
| Arhgap11a | 0.00201 | 1.14647791 |
| Cldn11 | 0.00838 | -1.03947881 |
| Aars | 0.03489 | -0.54079819 |
| Trim29 | 0.1848 | -0.93278871 |
| Bpifb6 | 0.00471 | -1.60136614 |
| Smad1 | 0.17771 | -0.25921643 |
| Oog1 | 0.00149 | -1.21097148 |
| Zfp771 | 0.00184 | -1.00782248 |
| Tsr1 | 0.00518 | 1.06417465 |
| Cdhr4 | 0.00724 | 0.8096128 |
| Echs1 | 0.84028 | -0.07212887 |
| Comp | 0.00223 | -1.28035191 |
| L3hypdh | 0.15093 | 0.35211329 |
| Olfr1230 | 0.00144 | -1.28116048 |
| Ccne1 | 0.30257 | -0.26583228 |
| Grhl2 | 0.00614 | -1.77859821 |
| Abcb8 | 0.011 | -1.45251733 |
| 4933439C10Rik | 0.13559 | 0.5322336 |
| Phgdh | 0.00144 | -1.51451976 |
| Rnf138rt1 | 0.44646 | 0.21612389 |
| Shisa2 | 0.07577 | -1.17592474 |
| Slc20a1 | 0.01086 | 0.83244809 |
| Slc27a4 | 0.05979 | -1.13427549 |
| Trub1 | 0.00982 | 0.6634938 |
| Ccl20 | 0.93548 | -0.04906203 |
| Enpp1 | 0.02302 | 1.74579539 |
| Oard1 | 0.22054 | 0.24144005 |
| Icam1 | 0.62011 | 0.11476563 |
| Cdipt | 0.01114 | -0.65199874 |
| Col24a1 | 0.04685 | -0.91948177 |
| Itgb4 | 0.00277 | -1.12867347 |
| Cfp | 0.07993 | -0.34391154 |
| Vps16 | 0.53497 | 0.10428729 |
| Misp | 0.44519 | 0.19229106 |
| Krtap4-2 | 0.00144 | -1.29864962 |
| Col6a4 | 0.19028 | 1.61271481 |
| Bola3 | 0.41605 | -0.21007449 |
| Ngfrap1 | 0.05577 | -0.36683263 |
| Herc1 | 0.00689 | 1.30010035 |
| Il1f9 | 0.00317 | -1.9493323 |
| Fgfr4 | 0.00976 | -0.66929054 |
| Apoa1 | 0.01317 | -1.74379373 |
| Kntc1 | 0.17019 | -0.48677988 |
| Akt2 | 0.08187 | -0.56537156 |
| 1500011B03Rik | 0.81046 | -0.07813459 |
| Grifin | 0.00235 | 2.02508733 |
| Trappc4 | 0.00638 | -0.85421802 |
| Fam193b | 0.06726 | 0.55276968 |
| 1700020D05Rik | 0.14945 | 0.29506076 |
| Gm38496 | 0.01693 | -0.96347022 |
| Cd200r1 | 0.00144 | -1.36353418 |
| Olfr140 | 0.06205 | -0.88151665 |
| Srcap | 0.04774 | -0.4999911 |
| Ly6h | 0.00144 | -1.30713006 |
| Slc22a21 | 0.11165 | -0.28483974 |
| Ints3 | 0.01358 | -0.74213273 |
| Zrsr1 | 0.00144 | 1.17889631 |
| Far1 | 0.00389 | 1.88758976 |
| Ndc1 | 0.13744 | -0.30768654 |
| Myt1 | 0.00144 | -1.28898079 |
| Adamts9 | 0.00144 | 3.31626868 |
| Ly75 | 0.00417 | 1.14628404 |
| Antxr1 | 0.37399 | 0.17707264 |
| Tspan31 | 0.01055 | -0.73700978 |
| Prkrip1 | 0.05681 | -0.38807304 |
| Pyroxd2 | 0.00144 | -1.27992547 |
| Nipal4 | 0.00144 | -1.28116048 |
| Mei1 | 0.26272 | -1.14323192 |
| Muc15 | 0.0029 | -1.20702967 |
| Serpinb9b | 0.0031 | -1.40845703 |
| St8sia5 | 0.28721 | -0.32568141 |
| Proscos | 0.00884 | -0.95870613 |
| Rfc1 | 0.39914 | 0.17791657 |
| Myo1e | 0.24493 | 0.20275689 |
| AI464131 | 0.7231 | 0.15818287 |
| Ankrd35 | 0.52441 | -0.17252676 |
| Il10rb | 0.3402 | 0.19294936 |
| Pdk1 | 0.1132 | 0.37614115 |
| Dnlz | 0.10771 | -0.70633345 |
| Serpinb6c | 0.44419 | 0.32500214 |
| Zfp579 | 0.04534 | 0.35628165 |
| Gcg | 0.00152 | -1.62530097 |
| Olfr822 | 0.12541 | -0.3230041 |
| Asb10 | 0.00698 | -0.88462499 |
| Calcb | 0.00144 | -1.28898079 |
| Particl | 0.05074 | -0.34760754 |
| Rps4l | 0.0021 | -1.46630878 |
| 1110001J03Rik | 0.00172 | -1.26123481 |
| Abhd4 | 0.08991 | -0.52767007 |
| Hgh1 | 0.00912 | -0.63315128 |
| Fig4 | 0.21035 | 0.59677218 |
| Trrap | 0.01103 | -0.73874372 |
| Hist1h2aa | 0.00144 | -1.38296771 |
| Msh6 | 0.0129 | 0.56272504 |
| Zscan4d | 0.00166 | -1.37728565 |
| Fcer1g | 0.04988 | -0.36637439 |
| Smcr8 | 0.10868 | 0.34104495 |
| Ecm1 | 0.00144 | -1.00442994 |
| Mepe | 0.67239 | -0.08489893 |
| Arhgef38 | 0.00144 | -1.25118919 |
| Nradd | 0.00767 | -0.78982602 |
| Maf | 0.89206 | -0.05230486 |
| Olfr464 | 0.00144 | -1.29051721 |
| Lpcat1 | 0.01249 | -0.72545098 |
| Efhc2 | 0.00261 | -1.11284335 |
| Smr3a | 0.10067 | -0.93967261 |
| Synm | 0.04768 | 0.57120878 |
| Ccdc58 | 0.3405 | 0.19634021 |
| Rpe65 | 0.00144 | -1.28898079 |
| Cdk10 | 0.04126 | -0.38413327 |
| Ap1s3 | 0.70755 | -0.34787111 |
| Alkbh3 | 0.1038 | 0.52468045 |
| Oscar | 0.33395 | -0.59986879 |
| 1500015O10Rik | 0.04794 | -0.92198523 |
| Bckdk | 0.05153 | -0.73755801 |
| B4galnt2 | 0.12922 | -1.42999165 |
| Rnd2 | 0.00355 | -0.86934798 |
| Exoc2 | 0.37718 | 0.14667918 |
| Cox16 | 0.01592 | -0.59690522 |
| Ly6g6d | 0.00144 | -1.17445806 |
| Atp6v0b | 0.08614 | -0.53270859 |
| Sp5 | 0.00144 | -1.28116048 |
| 1700003O11Rik | 0.00159 | -1.26431393 |
| Fmnl1 | 0.01793 | -0.60936841 |
| Psmb2 | 0.80811 | -0.12311033 |
| Fzd2 | 0.0169 | -0.82809427 |
| A930016O22Rik | 0.0021 | -1.05818962 |
| Sptbn2 | 0.03623 | -0.72925142 |
| Dmd | 0.00145 | -1.22376446 |
| Olfr1412 | 0.00158 | -1.2446827 |
| 2610100L16Rik | 0.00144 | -1.29746368 |
| Uba5 | 0.00144 | 1.15619566 |
| Smad7 | 0.42596 | 0.13872232 |
| Olfr1339 | 0.01257 | -1.28246319 |
| Gm15411 | 0.00804 | -0.65152001 |
| Lhx5 | 0.00144 | -1.29746368 |
| Ltbp4 | 0.0015 | -0.94553558 |
| Olfr895 | 0.00144 | -1.25927895 |
| Olig3 | 0.00145 | -1.24191372 |
| Pcdhb6 | 0.68129 | -0.42953978 |
| Tex43 | 0.01925 | -1.02536766 |
| Cpt2 | 0.00365 | -0.88589625 |
| Cuedc2 | 0.00355 | -0.89112699 |
| Tax1bp1 | 0.00144 | 2.15432053 |
| Tax1bp1 | 0.00144 | 1.91890019 |
| Ccdc116 | 0.0519 | -0.66585775 |
| Mdga1 | 0.3528 | -0.32878494 |
| Heatr5b | 0.05689 | 0.81530324 |
| Gm16489 | 0.03459 | -1.04583451 |
| Stab2 | 0.02097 | -0.55584309 |
| Zswim8 | 0.69761 | -0.09258932 |
| Hoxb8 | 0.00145 | -1.2388121 |
| Nkx6-2 | 0.00592 | -1.70723639 |
| Gpld1 | 0.2808 | -0.19900605 |
| Uvrag | 0.02957 | 0.40677861 |
| Olfr836 | 0.04383 | -1.24948708 |
| Trap1 | 0.45659 | 0.23777626 |
| Atp5sl | 0.87285 | -0.07223746 |
| Klhl8 | 0.38994 | -0.15777947 |
| Btla | 0.59079 | -0.21868187 |
| Pan2 | 0.15449 | 0.5311061 |
| Zfp750 | 0.00777 | -1.05183729 |
| 2300002M23Rik | 0.00144 | -1.26560808 |
| Tssk2 | 0.00229 | -1.27300595 |
| Tmem37 | 0.29099 | -0.31881305 |
| Taldo1 | 0.00974 | -0.68980055 |
| Nrp | 0.47941 | 0.23020518 |
| Srebf1 | 0.00543 | -1.20698517 |
| Gm5483 | 0.00151 | -1.17792995 |
| Kif1bp | 0.04782 | 0.67816478 |
| Vmn1r225 | 0.01175 | -2.6825468 |
| Cdh10 | 0.37949 | -0.59631329 |
| Mettl2 | 0.31012 | 0.41710757 |
| Vwa9 | 0.07737 | 0.47010969 |
| Abhd17a | 0.01499 | -0.73402873 |
| Laptm5 | 0.00144 | -1.37566765 |
| Gpatch2 | 0.00251 | 0.89742533 |
| Defb2 | 0.00144 | -1.27165604 |
| Rnmtl1 | 0.2868 | -0.37453912 |
| Col9a2 | 0.07102 | -0.73501379 |
| Tmem213 | 0.00218 | -1.18878227 |
| Mon2 | 0.4174 | 0.13016365 |
| Cdc45 | 0.1155 | 1.33030735 |
| Pus1 | 0.11028 | -0.4255069 |
| Cldn14 | 0.23597 | -0.7334754 |
| Eme1 | 0.1847 | -0.37533713 |
| Catsperd | 0.00145 | -1.22376446 |
| Lmntd2 | 0.00144 | -2.72599504 |
| Lrrc34 | 0.00197 | -1.41950631 |
| Rfpl4 | 0.13971 | -1.18169874 |
| Hnrnpa1 | 0.46674 | 0.13213244 |
| Clip4 | 0.48446 | 0.27027961 |
| Anp32e | 0.00144 | 1.67781587 |
| Cts3 | 0.00222 | -1.1481805 |
| Aif1 | 0.05853 | -0.5476739 |
| Whsc1 | 0.6709 | 0.14049561 |
| Agmat | 0.24466 | -0.2633527 |
| Pten | 0.00484 | 1.76469428 |
| 0610007P14Rik | 0.00778 | -0.70142636 |
| Rhbg | 0.00144 | -1.25118919 |
| Ankrd7 | 0.00144 | -1.30017714 |
| Slc38a5 | 0.00144 | -1.78617062 |
| Vpreb1 | 0.00336 | -1.18012674 |
| Furin | 0.00453 | -0.9724663 |
| 4930548H24Rik | 0.19464 | -0.75222507 |
| Ubxn2a | 0.00145 | 0.97393128 |
| Kalrn | 0.22231 | -0.35543925 |
| Abhd12 | 0.02731 | -0.93945247 |
| Myo9b | 0.23768 | -0.22691679 |
| Olfr178 | 0.00144 | -1.32313902 |
| Mier2 | 0.03087 | -0.63342591 |
| Mier2 | 0.27081 | -0.4247347 |
| Txlna | 0.02726 | -0.44320985 |
| Gm10209 | 0.01075 | -0.75400986 |
| Matk | 0.59694 | -0.5874323 |
| Ap4b1 | 0.69522 | 0.07159388 |
| Ap4b1 | 0.03459 | 0.46590309 |
| Zfp704 | 0.05664 | 0.45769941 |
| Fgf2 | 0.64928 | -0.16142213 |
| Dbi | 0.00145 | -0.92821913 |
| Crhr2 | 0.43801 | -0.1844291 |
| Cnih1 | 0.59554 | 0.09744798 |
| Zkscan14 | 0.25687 | -0.25226461 |
| Capns1 | 0.80044 | -0.1220571 |
| Fam187a | 0.07608 | -0.79344826 |
| Catsperg2 | 0.27957 | -0.49514228 |
| Wasf2 | 0.02374 | -0.50106099 |
| Tnfrsf19 | 0.87566 | 0.03470505 |
| Smok3a | 0.00144 | -1.27165604 |
| Ppm1j | 0.50682 | -0.20247786 |
| Igfbp4 | 0.00158 | -1.17584801 |
| Ctns | 0.00591 | -0.67534688 |
| Uck2 | 0.88407 | 0.06645497 |
| Has3 | 0.68152 | -0.21958226 |
| Olfr648 | 0.30149 | -0.28452463 |
| Tfap2d | 0.44159 | -0.69273628 |
| Flt1 | 0.90382 | 0.0311681 |
| Ccdc150 | 0.00144 | 1.91021332 |
| Zfp280d | 0.00144 | 3.20672075 |
| Fn3k | 0.00387 | -1.07460759 |
| Rnf130 | 0.99356 | 0.00143426 |
| Bsdc1 | 0.01532 | -0.53943139 |
| Kctd13 | 0.29504 | -0.25787124 |
| Clec5a | 0.00145 | -1.22376446 |
| Tyms | 0.547 | -0.12658526 |
| Tppp | 0.00151 | 0.97124973 |
| Dnajc11 | 0.10193 | -0.43332887 |
| Auts2 | 0.03919 | -0.4695499 |
| Klhl2 | 0.02521 | 0.73890404 |
| Zscan30 | 0.31672 | -0.87208391 |
| Zfp653 | 0.64698 | -0.08765582 |
| Vmn1r208 | 0.00144 | -1.28898079 |
| Olfr992 | 0.00144 | -1.31884045 |
| Grin3b | 0.93331 | -0.02665079 |
| D17Wsu92e | 0.0791 | -0.5290848 |
| En2 | 0.02713 | -2.43201521 |
| Tceb2 | 0.01277 | -0.61600265 |
| Zfand4 | 0.00145 | 2.82994471 |
| Pcsk9 | 0.00144 | -1.30713006 |
| Trrap | 0.45536 | -0.11563701 |
| Cts7 | 0.00156 | -1.1341237 |
| Snrnp40 | 0.059 | -0.40939597 |
| Mtrf1l | 0.00144 | 1.1786255 |
| Rbmxl2 | 0.02872 | 0.50242674 |
| Rundc3a | 0.91845 | 0.03994321 |
| Prss46 | 0.00144 | -1.28116048 |
| Reg3d | 0.26692 | -0.6770965 |
| Gatb | 0.23934 | -0.25843069 |
| Slc16a1 | 0.89841 | 0.02765884 |
| Marveld3 | 0.00144 | -1.52265203 |
| Nsl1 | 0.00144 | -1.54819245 |
| 4933424G06Rik | 0.00145 | -1.2388121 |
| 4931428L18Rik | 0.00144 | -1.28116048 |
| Ssu2 | 0.33544 | -0.20552075 |
| Zfyve21 | 0.58935 | 0.22889766 |
| Gabrg1 | 0.00149 | -1.19696847 |
| E130201H02Rik | 0.04673 | -0.40544845 |
| Timm10b | 0.00144 | -1.28614893 |
| Gsto2 | 0.09958 | -0.91380383 |
| 2810428I15Rik | 0.0023 | -1.04829512 |
| Fam122a | 0.50133 | 0.14730493 |
| Sugt1 | 0.38429 | 0.15024183 |
| Arl2bp | 0.83729 | -0.04003968 |
| Med21 | 0.06764 | 0.50408598 |
| Ncstn | 0.01403 | -0.82303846 |
| Plod2 | 0.36847 | 0.26003356 |
| 4930597G03Rik | 0.00144 | -1.25927895 |
| Sufu | 0.17771 | -1.01736284 |
| Mars2 | 0.94381 | 0.03274658 |
| Pcna | 0.04597 | 0.57241651 |
| Hsdl1 | 0.80282 | 0.07796682 |
| C4bp-ps1 | 0.0364 | 0.6615648 |
| Mdm1 | 0.24607 | 0.36277041 |
| Acot4 | 0.21131 | 0.53282557 |
| Uchl4 | 0.00234 | 1.26296102 |
| Slc22a7 | 0.00144 | -1.33632959 |
| Timm21 | 0.00823 | 1.03624588 |
| Olfr401 | 0.10605 | -1.07999339 |
| Spopl | 0.00231 | 0.87099706 |
| Srp72 | 0.01384 | 0.61469982 |
| Scamp4 | 0.32883 | -0.3709207 |
| 1700069L16Rik | 0.00144 | -1.27165604 |
| Vkorc1 | 0.00149 | -0.87560426 |
| Tns2 | 0.02708 | -0.43446654 |
| Ccz1 | 0.02316 | 0.48216098 |
| Pex16 | 0.02362 | -0.70654337 |
| Nme3 | 0.03132 | -0.56907556 |
| Gnrhr | 0.00144 | -1.30017714 |
| 4930562C15Rik | 0.00144 | -1.89526431 |
| Neurod6 | 0.00144 | -1.26934956 |
| Dusp19 | 0.97319 | 0.01803581 |
| 1700020L24Rik | 0.03721 | -0.47190919 |
| 4930573O21Rik | 0.00144 | 2.09870896 |
| Gm11346 | 0.08665 | 0.83045326 |
| Tshz1 | 0.22869 | 0.21759067 |
| Svep1 | 0.0026 | -0.85687229 |
| Prame | 0.11354 | -2.88463662 |
| Fam111a | 0.00506 | 1.49945999 |
| Actn4 | 0.00618 | -0.72093428 |
| Fryl | 0.10095 | 0.81783722 |
| Arhgef18 | 0.4412 | -0.16218568 |
| Kti12 | 0.60336 | -0.13279128 |
| Cklf | 0.1813 | 0.23975808 |
| Mef2d | 0.00389 | -0.81507374 |
| Snx22 | 0.02377 | -0.76131859 |
| Daam1 | 0.53755 | -0.31732616 |
| Dchs1 | 0.00144 | -1.19449176 |
| Tkt | 0.00445 | -0.77264459 |
| Tcf3 | 0.00144 | -1.13156541 |
| Erc2 | 0.1141 | -0.59444041 |
| Olfr67 | 0.00144 | -1.27165604 |
| Tspan2 | 0.00265 | 1.14993496 |
| Eif2b1 | 0.36646 | 0.25444138 |
| Fndc3a | 0.45545 | -0.18751256 |
| Gbgt1 | 0.00152 | -1.25878908 |
| Fap | 0.00144 | 1.605429 |
| Lmo4 | 0.11325 | -0.37616439 |
| Ankrd6 | 0.5433 | 0.18891888 |
| Ces1c | 0.00145 | -1.22376446 |
| Ddx51 | 0.80853 | -0.05343181 |
| Aadac | 0.00144 | -1.27730451 |
| Defb35 | 0.05251 | -0.80017931 |
| Slc25a26 | 0.89537 | 0.0532367 |
| Qprt | 0.09415 | -0.63304885 |
| Vsx2 | 0.00574 | -1.52432995 |
| Fbxo5 | 0.00145 | 1.40445775 |
| Spryd4 | 0.69148 | 0.15421783 |
| Osbp | 0.60295 | 0.17551521 |
| Ndufs2 | 0.9226 | -0.05646482 |
| Ddx58 | 0.02229 | -0.74150097 |
| Slc41a1 | 0.95054 | 0.01673506 |
| Olfr887 | 0.00144 | -1.25118919 |
| Hacd1 | 0.49633 | 0.31837271 |
| Hacd1 | 0.99416 | -0.00267434 |
| Tm6sf2 | 0.30425 | -0.90048592 |
| Fam171b | 0.42314 | 0.25155581 |
| Wdr19 | 0.05938 | -0.9887972 |
| Atp6v1d | 0.09745 | 0.30441428 |
| 9330154K18Rik | 0.05058 | -2.01895358 |
| Rnps1 | 0.0584 | -0.43446563 |
| Hmcn1 | 0.00144 | 2.39288852 |
| Ntrk2 | 0.00144 | -1.30713006 |
| Gfpt1 | 0.01876 | -1.03763442 |
| Sidt1 | 0.7191 | 0.18621922 |
| Olfr447 | 0.02373 | -0.9419902 |
| Smap1 | 0.06119 | -0.42614011 |
| Gabra4 | 0.00144 | -1.30017714 |
| Olfr344 | 0.57915 | -0.36897173 |
| Fut2 | 0.1766 | -0.71564996 |
| Tigd2 | 0.00144 | 1.73700117 |
| Ap4m1 | 0.02549 | -0.48061941 |
| Nmnat1 | 0.1986 | -0.32930369 |
| Tex13a | 0.00144 | -1.28898079 |
| Zmynd10 | 0.96434 | 0.01018182 |
| Sostdc1 | 0.06542 | -0.88764561 |
| Med25 | 0.4409 | -0.35724396 |
| Eml6 | 0.01478 | 1.51098549 |
| Xpc | 0.46019 | -0.14089374 |
| Vim | 0.52597 | -0.10483278 |
| Smo | 0.0297 | -1.74240493 |
| Prss39 | 0.00144 | -1.29049509 |
| Dmxl1 | 0.02364 | 1.28741434 |
| Ccbl2 | 0.22479 | 0.35263618 |
| Haus3 | 0.00144 | 2.47444944 |
| Tmem234 | 0.00864 | -0.62200977 |
| Ttc4 | 0.45443 | -0.11443731 |
| Zfp157 | 0.00392 | 2.32066891 |
| Mrgpre | 0.0459 | -0.49424734 |
| Smcp | 0.02642 | -1.12343037 |
| Frmd4b | 0.01185 | 0.78178585 |
| Lrrn3 | 0.15557 | -0.46880587 |
| Pdk3 | 0.09558 | 0.36345253 |
| Tbcd | 0.02637 | -0.43343359 |
| Pcnxl4 | 0.01186 | 0.71636304 |
| Xab2 | 0.00315 | -0.74052877 |
| Tmem19 | 0.17014 | 0.23593384 |
| Olfr1242 | 0.00144 | -1.29049509 |
| Crebrf | 0.70849 | 0.06268048 |
| Car8 | 0.00227 | 2.87362144 |
| Gm39523 | 0.00592 | -1.43006774 |
| Dapl1 | 0.00144 | -1.61548004 |
| Cpb1 | 0.00144 | -1.29085005 |
| Ubxn6 | 0.00528 | -0.85217528 |
| Olfr125 | 0.1902 | -0.73216731 |
| Exosc10 | 0.06967 | -0.33258294 |
| Exosc10 | 0.04302 | -0.41360913 |
| Dkkl1 | 0.88413 | -0.0354532 |
| Cbx6 | 0.0044 | -0.78908482 |
| Slc3a1 | 0.04929 | 0.52832083 |
| D8Ertd738e | 0.0026 | -0.85212407 |
| Etnppl | 0.00694 | -2.15772972 |
| BC002189 | 0.0839 | 1.22518089 |
| Pola2 | 0.96993 | 0.00904439 |
| Itih1 | 0.0622 | -0.8873676 |
| Ifngr1 | 0.08413 | -0.39076618 |
| Ifi27l2b | 0.00624 | -1.34981112 |
| Fam63b | 0.02199 | 0.61084424 |
| Tfap4 | 0.20844 | -0.51042448 |
| Gng3 | 0.10963 | -0.8164358 |
| Gdi1 | 0.00144 | 1.06739492 |
| Ang | 0.45443 | -0.11701353 |
| Aco1 | 0.55569 | 0.18298934 |
| Slc43a3 | 0.15455 | 0.29288113 |
| Gm9961 | 0.07463 | -0.84305934 |
| Mrgprb5 | 0.00144 | -1.30713006 |
| Wisp2 | 0.00242 | -1.41807929 |
| LOC102641521 | 0.1024 | 0.69991762 |
| Ube2e1 | 0.00401 | 0.86126941 |
| Tjp1 | 0.00144 | 2.1370454 |
| Tgfb1 | 0.00232 | -0.78871356 |
| Tmem230 | 0.42166 | 0.19296687 |
| Mpeg1 | 0.1112 | 0.39236596 |
| Epc2 | 0.0141 | 0.68572542 |
| Bloc1s4 | 0.23084 | -0.33297938 |
| Mep1b | 0.00144 | -1.33249771 |
| Hyal3 | 0.06058 | -0.94359587 |
| Pacs2 | 0.07452 | -0.33829017 |
| Ercc1 | 0.02259 | -0.49584177 |
| Zmynd19 | 0.39286 | 0.20338768 |
| Rcor3 | 0.44519 | 0.18433441 |
| Nkpd1 | 0.86284 | 0.09717175 |
| 2900026A02Rik | 0.00614 | -0.66493753 |
| Wdfy1 | 0.00284 | -0.79463566 |
| Larp6 | 0.00228 | -1.36529814 |
| Cwc25 | 0.10839 | 0.4196143 |
| Cs | 0.00275 | -0.79925608 |
| Kremen1 | 0.08901 | -0.43629175 |
| Styx | 0.30288 | 0.16956609 |
| Dzip1 | 0.30215 | -0.68805624 |
| Olfr186 | 0.00145 | -1.2388121 |
| Cpa6 | 0.05612 | -0.67110422 |
| Slc40a1 | 0.98117 | -0.00788076 |
| Rgs14 | 0.02446 | -0.70724097 |
| Slc35f5 | 0.30847 | 0.26288451 |
| Spic | 0.34355 | -0.75048496 |
| B3gnt9 | 0.1124 | -0.27736697 |
| Zpld1 | 0.00144 | -1.32343611 |
| Sema3f | 0.00835 | -0.61001218 |
| Ppp1r21 | 0.00417 | 0.83380339 |
| Sf3a2 | 0.00483 | -0.93368646 |
| 1700111E14Rik | 0.00145 | -1.2388121 |
| Relb | 0.00858 | -0.6711817 |
| Phlpp2 | 0.00897 | 0.82417449 |
| Tmem47 | 0.00144 | 1.91150337 |
| Scrt2 | 0.06317 | -0.46289318 |
| Mrgprb2 | 0.01685 | -1.08368725 |
| Kcnn4 | 0.0305 | -0.67017033 |
| Ins2 | 0.31548 | -1.4590105 |
| Adgrv1 | 0.0705 | -0.68838423 |
| Adgrv1 | 0.09282 | 0.63370141 |
| Fam220a | 0.0039 | -1.11868593 |
| Wash1 | 0.01602 | -0.59815125 |
| Fsip2 | 0.00144 | -1.29051721 |
| Fahd1 | 0.00145 | 1.03280095 |
| Gykl1 | 0.00144 | -1.55648401 |
| Lrrc9 | 0.62942 | -0.41155739 |
| Pnpla3 | 0.16322 | -0.69159608 |
| Fam131b | 0.02302 | -1.4189721 |
| B3gnt5 | 0.00144 | -1.24613586 |
| Cfap74 | 0.18829 | -2.15523689 |
| Egfem1 | 0.00144 | -1.28116048 |
| Fxr2 | 0.99839 | -0.00058965 |
| Zhx1 | 0.12538 | 1.53318272 |
| Thrb | 0.01794 | 0.74656521 |
| Gprasp1 | 0.01458 | 0.86009863 |
| Tbcc | 0.00646 | -0.76390453 |
| Slc11a2 | 0.94503 | 0.01532595 |
| Ndufs4 | 0.34426 | -0.18111849 |
| Msx2 | 0.61092 | -0.43530983 |
| Mxd1 | 0.44174 | -0.97814692 |
| Olfr1086 | 0.09853 | -2.54821106 |
| Rorb | 0.0034 | -1.09848529 |
| Efnb1 | 0.00144 | -1.36376667 |
| Dydc1 | 0.99847 | 0.00125595 |
| Anp32b | 0.2118 | 0.22260346 |
| Cd55 | 0.10461 | 0.48790833 |
| Ppil4 | 0.00144 | 1.51429023 |
| Aff3 | 0.39063 | -0.49364651 |
| Clgn | 0.38363 | -0.28242704 |
| Mmachc | 0.93763 | -0.02426596 |
| Olfr402 | 0.08983 | -2.54909284 |
| Pcdha4 | 0.00144 | -1.28898079 |
| Snrpb2 | 0.06452 | 0.34124773 |
| Cox17 | 0.00144 | -1.4557091 |
| Rpl22 | 0.00145 | -0.87457844 |
| Tekt4 | 0.01441 | -0.83995986 |
| Ifih1 | 0.00629 | 1.46545575 |
| Clec2l | 0.62465 | -0.15231343 |
| Ifnab | 0.07902 | -0.53331937 |
| Trmt2a | 0.03902 | -0.40872539 |
| Nfkbiz | 0.00144 | 2.51120422 |
| Olfr1367 | 0.00144 | -1.27165604 |
| Rab13 | 0.63112 | 0.15175895 |
| Abi2 | 0.07939 | 1.41663585 |
| Serpina12 | 0.00144 | -1.29746368 |
| Tshz3 | 0.32072 | 0.25763584 |
| Uqcr10 | 0.00653 | -0.83228445 |
| Iigp1 | 0.0044 | 1.88178 |
| Nampt | 0.00212 | 1.37167999 |
| Ddx1 | 0.00184 | 0.84687727 |
| Vrk3 | 0.02931 | -0.57167768 |
| Oasl2 | 0.80811 | 0.05176154 |
| 4930535E02Rik | 0.00144 | 2.2964338 |
| Mbd3l1 | 0.00353 | -1.08965911 |
| Tnni3 | 0.00286 | -0.80344249 |
| Taco1 | 0.95549 | -0.02512071 |
| Mfsd7c | 0.96071 | 0.01410961 |
| Stip1 | 0.48401 | -0.16025644 |
| Sprr2f | 0.00144 | -1.24811894 |
| Gm5577 | 0.00144 | -1.30017714 |
| Gmppb | 0.03843 | -0.53113377 |
| 1700017G19Rik | 0.05073 | -1.24686229 |
| Myf6 | 0.37367 | -0.59332616 |
| Krtap19-9b | 0.00144 | -1.32313902 |
| Dse | 0.0226 | -0.80009402 |
| Glod5 | 0.42145 | -0.3256388 |
| Llgl1 | 0.01604 | -0.57553561 |
| Epx | 0.78896 | 0.47144791 |
| Gpsm1 | 0.04404 | -0.66499466 |
| Ebf2 | 0.96407 | -0.0096993 |
| Rnf125 | 0.00144 | 1.42843787 |
| Wbscr16 | 0.82771 | -0.08288542 |
| Actr6 | 0.00372 | 1.18497988 |
| Wwp2 | 0.00354 | -0.84890594 |
| Mtmr10 | 0.00144 | 1.21004301 |
| Ccnl2 | 0.00144 | 1.68815459 |
| Olfr187 | 0.48282 | -0.18585887 |
| Cyp2d12 | 0.24805 | -0.29206278 |
| Rcan3 | 0.77323 | -0.04727294 |
| Magi2 | 0.00144 | -1.12824751 |
| Morn4 | 0.1389 | -1.73264646 |
| Cabp1 | 0.21406 | 0.33764948 |
| Zfp946 | 0.00244 | -1.36776064 |
| Fam169a | 0.03231 | -0.87105313 |
| Six4 | 0.92434 | -0.01609214 |
| Mecr | 0.11841 | -0.3927862 |
| Rab9b | 0.04915 | 0.83502587 |
| Ppp2r5b | 0.00644 | -0.85939007 |
| 4930401B11Rik | 0.1559 | -1.38386953 |
| Sidt2 | 0.01695 | -0.67341309 |
| Prom2 | 0.00144 | -1.26560808 |
| Cyp4f39 | 0.07203 | -1.63079701 |
| Glipr1l1 | 0.10341 | -0.9970647 |
| Eif2ak1 | 0.22548 | 0.33962459 |
| Mcts1 | 0.02231 | 0.51896966 |
| Cd177 | 0.00145 | -1.22193674 |
| Mgat3 | 0.73872 | -0.12073375 |
| Hoxc12 | 0.0024 | -1.68952541 |
| Gjb5 | 0.00144 | -1.24811894 |
| Mrpl53 | 0.36688 | -0.33018451 |
| Slc44a3 | 0.04466 | -0.48252227 |
| Mroh8 | 0.00144 | -1.29049509 |
| Snhg11 | 0.04217 | -1.12166561 |
| Tnf | 0.17456 | 0.75037725 |
| Thpo | 0.08305 | 0.37330131 |
| Zfp74 | 0.00144 | 3.88058736 |
| Svs6 | 0.00152 | -1.25878908 |
| Heatr6 | 0.01751 | -0.50973616 |
| Grik4 | 0.65608 | -0.21486719 |
| Ndufa2 | 0.00144 | -0.93093364 |
| Mstn | 0.23678 | -0.76637293 |
| Mdh2 | 0.01184 | -0.80306172 |
| Ldb1 | 0.02833 | -0.4700191 |
| Efna4 | 0.75884 | -0.15957739 |
| Lrrc59 | 0.33823 | 0.24835358 |
| Mrpl10 | 0.75817 | -0.12599 |
| Ctsd | 0.00606 | -0.91634761 |
| Ddx10 | 0.00245 | 2.27682394 |
| Ido2 | 0.01899 | -0.98659835 |
| Tmem201 | 0.34464 | -0.47092711 |
| Ctrc | 0.01947 | -0.80061374 |
| Dll4 | 0.0036 | -1.13547594 |
| C1ra | 0.21161 | -0.2537411 |
| Slc2a3 | 0.00144 | -1.29878664 |
| Ryk | 0.83612 | -0.034969 |
| Arsi | 0.2214 | -0.53377544 |
| Rad21 | 0.01085 | 0.78086977 |
| 8430436N08Rik | 0.00145 | -1.22376446 |
| 4921508D12Rik | 0.00144 | -1.28116048 |
| Prp2 | 0.00935 | -0.67444719 |
| Pih1d1 | 0.09767 | -0.69116031 |
| Gngt1 | 0.00144 | -1.29085005 |
| Sox6os | 0.11931 | -0.6510441 |
| Bmp4 | 0.04539 | 0.68449719 |
| Kank2 | 0.03575 | -0.65090629 |
| Pdzd2 | 0.15026 | -0.95959232 |
| Vps4a | 0.40241 | 0.22943155 |
| Golgb1 | 0.03973 | -0.38273778 |
| Klre1 | 0.06127 | -2.95308534 |
| Mpzl3 | 0.17863 | -0.58311644 |
| Art3 | 0.00373 | 1.12688945 |
| Aldh1a7 | 0.73797 | -0.13945262 |
| Tenm3 | 0.01819 | 0.57420018 |
| 4930513O06Rik | 0.01911 | -2.81906197 |
| Rnf103 | 0.00144 | 1.27191701 |
| 3110079O15Rik | 0.02075 | -1.00628634 |
| Fras1 | 0.00767 | -1.02946836 |
| Tfpi | 0.20398 | 0.54141956 |
| Pde9a | 0.80045 | -0.12612801 |
| Gm2381 | 0.00145 | -1.2388121 |
| Slk | 0.00158 | 1.57425846 |
| Clec4d | 0.10557 | 0.73372014 |
| Arhgap26 | 0.19633 | -0.76440037 |
| Meox2 | 0.00144 | 1.69920665 |
| Itga9 | 0.82582 | 0.03969564 |
| Cstf3 | 0.00329 | 0.74780691 |
| Fadd | 0.3594 | -0.32425041 |
| Slc2a10 | 0.89287 | 0.0248634 |
| Emb | 0.83355 | -0.22555921 |
| Zpbp2 | 0.63564 | 0.08522273 |
| Tmigd3 | 0.04719 | -0.75246261 |
| Akr1c20 | 0.03824 | -3.48763538 |
| 2900005J15Rik | 0.08816 | 0.69098776 |
| Zmynd19 | 0.7028 | -0.0971381 |
| Olfr399 | 0.00687 | -1.56681324 |
| Olfr483 | 0.09196 | -0.87605567 |
| Zfp418 | 0.0097 | 0.61366755 |
| Nphp3 | 0.54298 | -0.19162046 |
| Gm16491 | 0.02285 | 0.95828898 |
| Rnf2 | 0.00144 | 1.12283096 |
| Rab17 | 0.48109 | -0.31697398 |
| Pdzd7 | 0.03914 | -0.83398185 |
| Gm41289 | 0.00144 | -1.29864962 |
| Procr | 0.26657 | -0.29721587 |
| Hmx1 | 0.37475 | -0.42352707 |
| Fst | 0.00225 | -1.68011993 |
| 1700040A12Rik | 0.82014 | 0.09503044 |
| Exosc7 | 0.78814 | 0.06649031 |
| Usp1 | 0.00144 | 1.13848783 |
| Pla1a | 0.12518 | -0.33862297 |
| Morc4 | 0.3812 | 0.16630533 |
| Stx6 | 0.00993 | -0.66734742 |
| Rasa4 | 0.03084 | -0.61613864 |
| Eva1b | 0.00144 | -1.13566145 |
| Gm4836 | 0.00144 | -1.28116048 |
| Tubb3 | 0.00144 | -1.18720765 |
| Wwp1 | 0.00218 | 1.09539608 |
| Ndufa13 | 0.00144 | -1.13971744 |
| Ddx17 | 0.00257 | 0.93922578 |
| Fxyd5 | 0.00553 | -0.921799 |
| Pdzd11 | 0.00834 | -0.60553241 |
| Gulp1 | 0.01077 | 1.28129149 |
| Zbtb46 | 0.45246 | 0.18311331 |
| Olfr557 | 0.0019 | -1.4508388 |
| Krt34 | 0.00144 | -1.26560808 |
| Epc1 | 0.17421 | 0.26250388 |
| Hmgb4 | 0.01933 | -1.2534661 |
| Slc25a51 | 0.18166 | 0.47848915 |
| Olfr1225 | 0.91229 | -0.15569533 |
| Ckm | 0.23906 | -0.56291084 |
| A730056A06Rik | 0.22639 | -0.68831316 |
| Dok5 | 0.00144 | -1.28116048 |
| Cep72 | 0.01743 | 0.99274313 |
| Tjap1 | 0.69524 | -0.07689818 |
| Scx | 0.09985 | -0.60613604 |
| Cars2 | 0.24455 | -0.4311406 |
| Lcn6 | 0.00144 | -1.30784197 |
| Ncam1 | 0.37565 | 0.27001334 |
| Bpifb9a | 0.33548 | 0.38781263 |
| Prkacb | 0.00144 | -1.44864523 |
| Ikbkg | 0.06327 | 0.6807524 |
| Id3 | 0.00301 | -0.93356901 |
| Ubr4 | 0.22248 | -0.23306131 |
| Gm38426 | 0.07343 | -0.48023613 |
| Tpte | 0.06823 | -0.62153296 |
| Fcgbp | 0.00144 | -1.32313902 |
| Dip2a | 0.05972 | -1.34201762 |
| Bcl2 | 0.8157 | 0.05289504 |
| Galnt3 | 0.0019 | -1.15423558 |
| Arl4a | 0.78036 | 0.05470106 |
| Lcn8 | 0.00144 | -1.21743532 |
| Prss28 | 0.00144 | -1.28116048 |
| Slain2 | 0.02825 | 0.53490918 |
| Cyp4f41-ps | 0.23433 | -0.33722097 |
| Nrm | 0.00285 | -1.31869861 |
| Myoz1 | 0.03193 | -0.60502265 |
| Rpn1 | 0.02138 | -0.47678238 |
| Fxyd7 | 0.38061 | -0.26805725 |
| Gm10413 | 0.72802 | -0.14106388 |
| Mllt11 | 0.06661 | 0.50429569 |
| Aadat | 0.0974 | -1.61741551 |
| Ndufs1 | 0.03983 | 0.52367452 |
| Nckap5 | 0.00424 | 1.06699605 |
| Bclaf1 | 0.00242 | 1.85955002 |
| Cuedc1 | 0.00554 | -1.08519309 |
| Entpd6 | 0.9231 | -0.03992374 |
| Strbp | 0.81565 | -0.0731096 |
| Abca8a | 0.00899 | 0.8072634 |
| Neurod2 | 0.00898 | 0.97232104 |
| Ear6 | 0.00145 | -1.26219879 |
| Cep85 | 0.59359 | 0.17660848 |
| LOC102637942 | 0.004 | -0.92384594 |
| Adam24 | 0.00144 | -1.28116048 |
| 2810474O19Rik | 0.00709 | 2.17029977 |
| Ccdc159 | 0.46641 | -0.14972064 |
| Olfr1260 | 0.00144 | -1.31233397 |
| Atg10 | 0.0017 | 1.01843069 |
| Pfkp | 0.91515 | 0.0207032 |
| Cxcl13 | 0.60228 | -0.62645369 |
| Sohlh2 | 0.99249 | 0.00231549 |
| Wrap53 | 0.00233 | -0.85829061 |
| Them7 | 0.16864 | -0.6570291 |
| B3gnt7 | 0.00144 | -1.21868187 |
| Rpap2 | 0.01345 | 0.63088686 |
| Oplah | 0.00294 | -1.13552937 |
| Rbpms2 | 0.77728 | 0.09435675 |
| 4933411O13Rik | 0.00144 | -1.25118919 |
| Fcmr | 0.15047 | -0.41750763 |
| Mpp7 | 0.00413 | 1.27216834 |
| Guca2a | 0.005 | -1.12538086 |
| Bbs1 | 0.56376 | -0.21563008 |
| Atp5c1 | 0.13523 | -0.44639445 |
| Aoc3 | 0.08539 | -0.30346931 |
| Blzf1 | 0.00163 | 1.06164754 |
| Galntl5 | 0.00144 | -1.29746368 |
| Ptprq | 0.00144 | -1.31884045 |
| Gimap9 | 0.44625 | 0.21524584 |
| Nr3c2 | 0.00144 | -1.34536341 |
| Gstt3 | 0.00298 | -0.93720644 |
| Cmtm7 | 0.01511 | -0.71433955 |
| 1700011E24Rik | 0.64467 | -0.22897744 |
| Map3k12 | 0.09312 | -0.30929993 |
| E230024E03Rik | 0.05018 | -2.18810912 |
| 2900092C05Rik | 0.00144 | -1.38527672 |
| Ccdc94 | 0.05522 | -0.69538555 |
| Cpsf4l | 0.00152 | -1.18830552 |
| Tank | 0.00144 | 1.7297152 |
| Olfr1066 | 0.00144 | -1.33059316 |
| Oprk1 | 0.00144 | -1.25927895 |
| Sts | 0.00499 | -0.80377964 |
| Srebf2 | 0.00327 | -0.83437676 |
| Ncf4 | 0.02808 | -0.58902231 |
| Mettl14 | 0.06177 | 0.79940329 |
| Ndst2 | 0.09076 | -0.35941539 |
| 2310040G07Rik | 0.00177 | -0.93203418 |
| Gnl3 | 0.00144 | 1.56928653 |
| Kras | 0.01932 | -0.65380678 |
| Caap1 | 0.00144 | 1.77853234 |
| 1700018A14Rik | 0.10458 | -0.892713 |
| Tespa1 | 0.06001 | -1.07662251 |
| Cacna1c | 0.03944 | 0.69288255 |
| Zfp783 | 0.52418 | 0.28870416 |
| Ift43 | 0.10569 | -0.40850686 |
| Det1 | 0.01784 | -0.75351453 |
| Gm15688 | 0.00158 | -1.2446827 |
| Athl1 | 0.00479 | -0.76006911 |
| 2310003L06Rik | 0.00144 | -1.23984951 |
| Camk2b | 0.00226 | -1.52304766 |
| 4933402J07Rik | 0.03953 | -1.0150293 |
| Gm15498 | 0.05938 | -0.9887972 |
| Stard9 | 0.20088 | 0.29205224 |
| Rhox5 | 0.00144 | -1.22776752 |
| Tcaim | 0.00144 | 2.1625907 |
| Hebp1 | 0.72705 | -0.108985 |
| Olfr643 | 0.00144 | -1.31884045 |
| Fstl4 | 0.79285 | -0.13669765 |
| Med8 | 0.61695 | 0.14524792 |
| Mchr1 | 0.00254 | -1.23443484 |
| Glud1 | 0.1267 | 0.26937041 |
| Bcat1 | 0.08802 | -0.82215549 |
| Olfr342 | 0.00216 | -1.17166665 |
| N6amt2 | 0.19378 | -0.3812486 |
| Acsm1 | 0.00144 | -1.24900642 |
| Aire | 0.0015 | -1.32982311 |
| Bcorl1 | 0.00961 | -0.60895627 |
| Fign | 0.25268 | -0.31774994 |
| Spaca3 | 0.00854 | -1.22485794 |
| Spaca3 | 0.03271 | -0.61095135 |
| Srpk3 | 0.23599 | -0.27796157 |
| Prap1 | 0.2028 | -0.40064442 |
| Ptchd1 | 0.05225 | -0.7079796 |
| Hoxc10 | 0.05122 | -0.86718816 |
| Fam13b | 0.00181 | 1.50925291 |
| Tmem135 | 0.04266 | 0.86147562 |
| Pcsk5 | 0.00144 | -1.28116048 |
| Gm13999 | 0.68282 | -0.08455461 |
| Myoc | 0.01635 | -0.6416091 |
| Polr3b | 0.23292 | 0.26241626 |
| Olfr1047 | 0.00145 | -1.22376446 |
| Phip | 0.00876 | 2.12733826 |
| Rprd1a | 0.0594 | 0.38156618 |
| Gemin7 | 0.00355 | -0.84894944 |
| Eya1 | 0.00144 | -1.33632959 |
| Olfr585 | 0.00144 | -1.28116048 |
| Dennd6b | 0.02562 | 0.84464178 |
| Cux1 | 0.09199 | -0.58287617 |
| Gm19522 | 0.00145 | 1.17800234 |
| Plk3 | 0.33104 | -0.3013384 |
| Rpf2 | 0.02122 | 0.62152314 |
| Cd36 | 0.00971 | 0.71515953 |
| Olfr701 | 0.00145 | -1.22376446 |
| Lmbrd2 | 0.0557 | 0.40125313 |
| Vmn2r29 | 0.29137 | 0.32861398 |
| P2ry13 | 0.69851 | 0.18415859 |
| Abca7 | 0.0036 | -0.70657592 |
| Shd | 0.11893 | 0.40587201 |
| Epn3 | 0.00455 | -0.71660832 |
| Arfgef1 | 0.00144 | 1.4232052 |
| Rbp4 | 0.73989 | -0.16020862 |
| Ift22 | 0.15865 | 0.41128837 |
| Ptx3 | 0.59509 | -0.60188184 |
| Baz1a | 0.20844 | 0.2437642 |
| Spata1 | 0.00144 | 2.94369599 |
| Olfr374 | 0.78749 | -0.10668349 |
| Lrrc63 | 0.01767 | -1.4406427 |
| Cndp1 | 0.00144 | -1.32766452 |
| Olfr1366 | 0.11962 | -0.90769809 |
| Flt3l | 0.04107 | -0.54491694 |
| Olfr354 | 0.00144 | -1.32599123 |
| Rchy1 | 0.01674 | 0.73218969 |
| Cxcl16 | 0.00391 | -0.95248342 |
| Bco1 | 0.00144 | -1.31495282 |
| Guca1b | 0.89503 | -0.03075111 |
| Fgd5 | 0.17503 | 0.23830345 |
| Fgd5 | 0.88232 | 0.09096755 |
| Vmn1r215 | 0.00144 | -1.28116048 |
| Ints4 | 0.00503 | -0.83081411 |
| Maml2 | 0.02094 | 0.72489423 |
| Mbl1 | 0.12108 | -0.61790352 |
| Yars2 | 0.04466 | 0.96906986 |
| Flywch1 | 0.0222 | -0.70261906 |
| Olfr1342 | 0.88194 | -0.07190956 |
| Nphs2 | 0.00149 | -1.19696847 |
| Olfr572 | 0.00149 | -1.22582153 |
| Brcc3 | 0.2642 | 0.19457836 |
| Lce3c | 0.00144 | -1.31884045 |
| Csnk2b | 0.0417 | -0.65560803 |
| Med4 | 0.0979 | 0.30056359 |
| Crls1 | 0.17984 | 0.60289917 |
| Gm13561 | 0.00247 | -1.34636174 |
| AI854703 | 0.13075 | -0.46450809 |
| Spata32 | 0.16042 | -0.32179239 |
| 4632428N05Rik | 0.03518 | -0.63682281 |
| Zfp433 | 0.00144 | 2.63878561 |
| Dpf2 | 0.40274 | -0.14810203 |
| Smox | 0.10553 | -0.27662698 |
| Ptgis | 0.00248 | -0.84517229 |
| Pou6f1 | 0.1127 | -0.65449137 |
| Frmpd3 | 0.98468 | 0.0290853 |
| Il16 | 0.07692 | -0.48368408 |
| Tusc2 | 0.08855 | -0.53435147 |
| Arap3 | 0.00201 | 1.24205535 |
| Cgref1 | 0.00144 | -1.43052218 |
| Fam189b | 0.0322 | -0.42494243 |
| Auh | 0.20925 | 0.38248367 |
| Fxr1 | 0.00144 | 1.65283826 |
| Wdr86 | 0.0135 | -0.73543985 |
| Angpt4 | 0.00145 | -1.2388121 |
| Olfr420 | 0.00144 | -1.25118919 |
| Mrpl54 | 0.00175 | -1.18081427 |
| Ackr2 | 0.56943 | -0.27383342 |
| Plod3 | 0.02177 | -0.57745214 |
| Gpc6 | 0.01557 | 1.07740739 |
| Tarm1 | 0.00144 | -1.30017714 |
| Slc6a18 | 0.03775 | -0.93497606 |
| Vps51 | 0.75136 | -0.13332311 |
| Tmed10 | 0.93825 | 0.0162231 |
| Psmd14 | 0.2441 | -0.48832327 |
| Pcolce | 0.00874 | -0.70910867 |
| Kctd12 | 0.00405 | 1.14136301 |
| Ccdc71 | 0.00363 | -0.84467781 |
| Zmynd8 | 0.03556 | -0.5294734 |
| 3830417A13Rik | 0.04023 | -1.20789141 |
| Marco | 0.48318 | 0.18415951 |
| Slc7a6os | 0.03338 | 0.42096543 |
| Olfr1413 | 0.02611 | -0.94534324 |
| Olfr1121 | 0.059 | -0.79876211 |
| Atp8b3 | 0.33482 | -1.9107718 |
| Fam175b | 0.00161 | -1.40263465 |
| Olfr1262 | 0.00144 | -1.28898079 |
| Kdm8 | 0.94054 | -0.0269514 |
| Nlrp14 | 0.00929 | -1.0433256 |
| Rwdd2b | 0.49542 | -0.2424822 |
| Gm608 | 0.43907 | 0.14067656 |
| Pttg1 | 0.0863 | -0.47082811 |
| Eif3b | 0.0031 | -0.7360032 |
| Sos2 | 0.01499 | 0.65436277 |
| Cops4 | 0.91691 | -0.02112781 |
| Rcsd1 | 0.02233 | -0.52173173 |
| Glipr1 | 0.56526 | 0.16613448 |
| Rgs9 | 0.32628 | -0.72376788 |
| Tm4sf4 | 0.86559 | 0.08697811 |
| Ptprz1 | 0.0016 | 1.475441 |
| 9030624J02Rik | 0.47298 | 0.20875731 |
| Ccdc124 | 0.00306 | -0.92215431 |
| Olfr1143 | 0.03746 | -0.55303782 |
| Polh | 0.90962 | -0.03900804 |
| Tnfrsf23 | 0.00144 | -1.24466058 |
| Gsc | 0.00144 | -1.30713006 |
| Gfi1b | 0.00144 | -1.53650525 |
| Zcchc5 | 0.00879 | -1.05401892 |
| Neurog2 | 0.05658 | -0.85356047 |
| Dcakd | 0.40383 | -0.48080161 |
| Spink12 | 0.00144 | -1.28116048 |
| BC022687 | 0.15139 | -0.37526495 |
| Cyp20a1 | 0.01576 | 0.54200181 |
| Krtap17-1 | 0.00144 | -1.27165604 |
| Lpin2 | 0.26757 | 0.55864624 |
| Olfr876 | 0.00144 | -1.31495282 |
| 1700030L22Rik | 0.00144 | -1.28116048 |
| Galnt2 | 0.02703 | -0.64569547 |
| Zbtb48 | 0.03003 | -0.41104742 |
| Capn10 | 0.08373 | -0.36880115 |
| Pdzd2 | 0.60832 | -0.14612057 |
| Ctr9 | 0.00144 | 0.96741619 |
| Olfr142 | 0.00152 | -1.25878908 |
| Pdgfa | 0.02534 | -0.58417029 |
| Tfeb | 0.66104 | -0.17604813 |
| Nagpa | 0.01165 | -0.70301924 |
| Ceacam18 | 0.00144 | -1.28898079 |
| Sntg2 | 0.00144 | -1.30737044 |
| Fra10ac1 | 0.01356 | 0.78440711 |
| Zfp410 | 0.03833 | 0.53970242 |
| Psmb9 | 0.00198 | -1.35896092 |
| Zfp160 | 0.00321 | 3.15682269 |
| Ces1e | 0.02823 | 0.80165149 |
| Nmrk2 | 0.00793 | -1.14255222 |
| Olfr617 | 0.00144 | -1.27454685 |
| Ptov1 | 0.18782 | -0.6045668 |
| Ptov1 | 0.00386 | -0.80145122 |
| Olfr139 | 0.25365 | -0.85452962 |
| Nudt16l1 | 0.00785 | -0.63999166 |
| Ly6g5b | 0.04064 | -0.54203769 |
| Slc18b1 | 0.83814 | -0.03179211 |
| Ubtd1 | 0.03549 | -0.76863944 |
| Olfr894 | 0.00144 | -1.28116048 |
| Nox4 | 0.07345 | -0.76378171 |
| Ddias | 0.32538 | 0.29725421 |
| Tpx2 | 0.08684 | 0.724323 |
| Anapc13 | 0.02833 | -0.57376855 |
| Onecut1 | 0.00145 | -1.2388121 |
| Olfr1302 | 0.00222 | -1.1481805 |
| Pfdn6 | 0.06367 | -0.49851065 |
| Grb7 | 0.00144 | -1.90665306 |
| Gabrb1 | 0.00144 | -1.31884045 |
| C630043F03Rik | 0.00371 | 1.72361064 |
| Tmbim4 | 0.56062 | 0.27796362 |
| Tle6 | 0.00769 | -0.68993736 |
| Dtd1 | 0.75989 | 0.11014316 |
| Fam50a | 0.00308 | 0.97053519 |
| Abhd8 | 0.04567 | -0.7557288 |
| Tmem98 | 0.17804 | -0.29753443 |
| 1700123O20Rik | 0.02642 | -0.54030848 |
| Gm5154 | 0.0583 | -0.81750407 |
| LOC102634128 | 0.00144 | -1.2383131 |
| Dnajb8 | 0.02877 | 1.20251668 |
| Fam101a | 0.00955 | -1.75400351 |
| Dnah1 | 0.00144 | -1.28116048 |
| Phyhip | 0.00286 | -1.00447154 |
| Flna | 0.73753 | 0.1138632 |
| Gon4l | 0.45596 | -0.11860656 |
| Sorbs2 | 0.08728 | 0.48004971 |
| Nkx2-3 | 0.12732 | -0.82093021 |
| 2210016L21Rik | 0.00144 | -1.29891725 |
| E2f2 | 0.01692 | -0.91402466 |
| Olfr1048 | 0.1632 | 0.42431434 |
| C1ql3 | 0.08858 | -1.25025713 |
| Arhgap29 | 0.36712 | 0.33438222 |
| A930002I21Rik | 0.00804 | -1.14019189 |
| Strc | 0.01395 | -1.01228913 |
| Krt84 | 0.03932 | -1.92826409 |
| Egr1 | 0.00165 | 2.02945299 |
| Polr2g | 0.05374 | -0.50097799 |
| Adamtsl2 | 0.26618 | -0.27882826 |
| 4921536K21Rik | 0.0049 | -1.54772759 |
| Adamts6 | 0.90936 | -0.05465585 |
| Prr30 | 0.00736 | 1.48622171 |
| Tmed5 | 0.1837 | 0.4309374 |
| Cldnd2 | 0.47814 | -0.39463993 |
| Tango2 | 0.5225 | -0.19662941 |
| Sod1 | 0.00144 | -1.07025264 |
| Rnf135 | 0.83976 | -0.05639892 |
| Lman1l | 0.03019 | -0.97372708 |
| Tspan33 | 0.05778 | -0.54452849 |
| Psmb1 | 0.94992 | -0.02534022 |
| Zbtb21 | 0.00447 | -1.07435095 |
| Pold1 | 0.00593 | -0.68042318 |
| Itih3 | 0.93087 | -0.03635032 |
| Il9 | 0.10339 | -0.8216566 |
| Ifrd1 | 0.0071 | 1.1082971 |
| Slirp | 0.8802 | 0.02565228 |
| Nsun7 | 0.00152 | -1.25878908 |
| Apod | 0.00751 | -0.78624707 |
| Rims1 | 0.00682 | -0.91270338 |
| Fate1 | 0.00158 | -1.2446827 |
| Slc36a2 | 0.24766 | 0.35504742 |
| Papss2 | 0.23627 | 0.38040894 |
| Zfp125 | 0.52119 | 0.36343251 |
| Zfp799 | 0.0102 | 0.57687176 |
| Cep83 | 0.52563 | -0.19200049 |
| Nap1l2 | 0.00144 | -1.26560808 |
| Mpg | 0.00791 | -0.6156662 |
| Mertk | 0.0027 | 1.63309063 |
| Klf14 | 0.00409 | -1.29012157 |
| Fscn1 | 0.0023 | -0.88806036 |
| Ercc3 | 0.22797 | 0.28024145 |
| 2310022B05Rik | 0.48431 | -0.29755177 |
| Tor1a | 0.0114 | -0.57208453 |
| Trdmt1 | 0.00409 | 0.96558383 |
| Gin1 | 0.00217 | 1.78612484 |
| Narfl | 0.04117 | -0.62829745 |
| Ankzf1 | 0.93163 | -0.01557937 |
| Olfr1216 | 0.00144 | -1.30017714 |
| Duoxa2 | 0.02072 | -1.39528885 |
| Slc35g3 | 0.86019 | -0.06595238 |
| Rala | 0.03044 | -0.50948899 |
| Tmem101 | 0.01926 | -0.75663118 |
| Rfx5 | 0.08405 | -0.62942497 |
| 9030612E09Rik | 0.00187 | 1.58939016 |
| Spink1 | 0.00144 | -1.36377172 |
| Clpb | 0.00492 | -0.76301876 |
| Smg5 | 0.05611 | -0.41861559 |
| Best2 | 0.1515 | 0.55529417 |
| Reln | 0.23328 | 0.31382339 |
| Eif3e | 0.01469 | 0.61165049 |
| Hs3st1 | 0.00144 | -1.32144095 |
| Micalcl | 0.33257 | -0.5381555 |
| Tmprss11d | 0.00144 | -1.26560808 |
| Gclc | 0.01282 | 0.65713019 |
| Tns1 | 0.67998 | -0.07048045 |
| Gabpa | 0.00198 | 1.00228438 |
| Mroh9 | 0.00897 | -1.87732577 |
| Olfr361 | 0.00144 | -1.25118919 |
| Slfn5 | 0.00144 | 2.17595423 |
| Rnf32 | 0.35596 | -0.38547358 |
| Krt71 | 0.00152 | -1.25878908 |
| Sh3bp2 | 0.35435 | -0.21913923 |
| Fads2 | 0.00311 | -0.81633035 |
| Fyb | 0.46192 | 0.22473677 |
| Zic2 | 0.00144 | -1.26301121 |
| D330050I16Rik | 0.41496 | -0.267004 |
| Tnfaip2 | 0.45492 | -0.25512222 |
| B3gat3 | 0.08231 | -0.62047867 |
| Vamp7 | 0.00144 | 2.07120316 |
| Rnf167 | 0.0533 | -0.58731069 |
| Kif13b | 0.15391 | -0.93369864 |
| Mitf | 0.46986 | -0.11826975 |
| Mad1l1 | 0.01322 | -0.50310772 |
| Ms4a3 | 0.00145 | -1.2388121 |
| Lilra5 | 0.6186 | -0.15281235 |
| Ttc33 | 0.02649 | 0.61628084 |
| 1700022I11Rik | 0.00144 | -1.28116048 |
| Olfr1356 | 0.00144 | -1.31884045 |
| Clk2 | 0.01438 | 0.80291857 |
| Cdkn1a | 0.21145 | -0.30348361 |
| Cct2 | 0.32707 | 0.24482918 |
| Slc25a23 | 0.6621 | 0.08896788 |
| Ptbp2 | 0.00144 | 1.33120014 |
| Mcm3ap | 0.60927 | 0.12442941 |
| Irf6 | 0.50431 | -0.45194837 |
| Prg3 | 0.00144 | -1.31884045 |
| Stx7 | 0.00144 | 1.01495597 |
| Rlim | 0.12107 | 0.31658578 |
| 1700088E04Rik | 0.1605 | -0.28569602 |
| Snrpe | 0.856 | -0.04163964 |
| Snrpe | 0.05651 | -0.38173481 |
| Abi2 | 0.00171 | 1.56524241 |
| Fbrsl1 | 0.00416 | -0.88942358 |
| Prg2 | 0.00145 | -1.2388121 |
| Klc2 | 0.37985 | -0.34454643 |
| Prkd1 | 0.00257 | 1.3037031 |
| Ifna9 | 0.07233 | -0.50761311 |
| Prss41 | 0.12461 | -0.97747523 |
| Dhx58 | 0.00144 | -1.30017714 |
| D630045J12Rik | 0.73046 | 0.17709212 |
| Eif4g3 | 0.51474 | -0.36053792 |
| Blvra | 0.32601 | -0.20329818 |
| Fbxo36 | 0.94133 | 0.02794906 |
| Dstyk | 0.00144 | -0.99118165 |
| Fbxo38 | 0.00751 | 0.61868863 |
| Supt20 | 0.30877 | 0.27337986 |
| Obp1b | 0.00144 | -1.25118919 |
| Spin4 | 0.14674 | 0.57032346 |
| Tnnt1 | 0.01234 | 0.7235801 |
| Olfr1307 | 0.46773 | -0.78554459 |
| Syde2 | 0.01381 | 1.05460291 |
| Heatr1 | 0.23604 | 0.20274119 |
| Pla2g4e | 0.05586 | -0.99616812 |
| Myh11 | 0.5686 | 0.29422731 |
| Lig4 | 0.00144 | 3.40944457 |
| Magea4 | 0.00144 | -1.31884045 |
| Commd10 | 0.0409 | 0.40863464 |
| Mob3c | 0.00234 | 0.9425167 |
| Fgg | 0.00437 | -1.37014133 |
| Ubqln4 | 0.48142 | -0.2013948 |
| Lrrc3b | 0.01414 | 0.73649876 |
| Chil1 | 0.246 | -0.8945068 |
| Atp5e | 0.00144 | -1.17747873 |
| Rexo1 | 0.05111 | -0.45598672 |
| Olfr1271 | 0.08162 | -2.59905674 |
| Uqcrfs1 | 0.44957 | -0.22304667 |
| Lgi2 | 0.18223 | -0.40760549 |
| Vapa | 0.92328 | -0.01575382 |
| Hist2h2bb | 0.00144 | -1.26428512 |
| Ccdc67 | 0.52618 | -0.52195162 |
| Rai1 | 0.01145 | -0.84412865 |
| Rab11b | 0.08636 | -0.51547767 |
| Scara5 | 0.02226 | 0.5016847 |
| Cep128 | 0.00589 | -1.11030563 |
| Arl8b | 0.07041 | 0.49069676 |
| Gzmg | 0.00144 | -1.27165604 |
| Agpat5 | 0.00735 | 2.11162822 |
| Gm30003 | 0.00144 | -1.28116048 |
| Fzd4 | 0.11287 | 0.32494567 |
| Atp6v0d1 | 0.6926 | -0.15399759 |
| Dus2 | 0.0855 | -0.34534416 |
| Aaed1 | 0.00415 | -0.71862301 |
| Mbtd1 | 0.00144 | 1.72802961 |
| Ehd3 | 0.0762 | -0.32466105 |
| Zfp266 | 0.00286 | 1.44416799 |
| 4930554P06Rik | 0.00144 | -1.20579466 |
| Zfpm1 | 0.00144 | -1.0834476 |
| Slc22a6 | 0.00203 | -1.23529212 |
| Srst | 0.0272 | -1.11008548 |
| Hoxa6 | 0.00548 | -1.74712279 |
| Olfr1009 | 0.01084 | -1.78801071 |
| Tmem143 | 0.95615 | -0.03864699 |
| Mcm6 | 0.82792 | 0.04953662 |
| Kcnk7 | 0.09769 | -0.96570761 |
| Tfap2b | 0.12259 | -2.87473181 |
| Krt32 | 0.0065 | -1.79639904 |
| Immp2l | 0.00577 | -1.02929066 |
| Ccng2 | 0.04861 | 0.53684185 |
| Spen | 0.05098 | -0.36463223 |
| Olfr418 | 0.03669 | -0.94779588 |
| Tspan9 | 0.20225 | -0.62898317 |
| Spata17 | 0.15522 | -0.46062355 |
| Ocstamp | 0.92303 | -0.03672764 |
| Arhgap17 | 0.18844 | 0.21766739 |
| Csnk1d | 0.16351 | -0.29717498 |
| Clec11a | 0.02391 | -0.644784 |
| 1700007B14Rik | 0.00144 | -1.22925777 |
| Lgals3bp | 0.0031 | -0.74669489 |
| Itgb7 | 0.01025 | -1.23979257 |
| Ifit3 | 0.80213 | 0.07740488 |
| Fem1c | 0.62835 | 0.08870446 |
| Rtn4rl2 | 0.33337 | 0.38297221 |
| Zfpl1 | 0.00301 | -0.76170871 |
| BC004004 | 0.01681 | -0.65541274 |
| Apobec2 | 0.14061 | -0.42291898 |
| Alcam | 0.20849 | 0.51549612 |
| Sec11c | 0.97393 | 0.00745758 |
| Tmem9 | 0.04779 | -0.71084244 |
| Syt7 | 0.68612 | 0.0977752 |
| Doc2g | 0.69311 | -0.13314869 |
| Slurp1 | 0.04824 | -0.86696801 |
| U2af2 | 0.08244 | -0.34159844 |
| Ttc9b | 0.00145 | -1.2388121 |
| D630022N01Rik | 0.00737 | -0.8913225 |
| Disc1 | 0.01173 | 0.74785008 |
| Veph1 | 0.00144 | -1.28116048 |
| Olfr48 | 0.01897 | -2.87903806 |
| Spp1 | 0.14584 | -0.75138333 |
| Lancl3 | 0.17969 | 1.09764044 |
| Olfr1221 | 0.23295 | -1.18527205 |
| Pdzd3 | 0.96993 | -0.02025099 |
| Olfr561 | 0.00144 | -1.30017714 |
| Ppp1r32 | 0.45064 | 0.15312421 |
| Dnase1 | 0.00144 | -1.31884045 |
| Usp53 | 0.36017 | 0.18849213 |
| Loxhd1 | 0.32785 | -0.59483314 |
| Jam3 | 0.759 | 0.06003241 |
| Chga | 0.05025 | -0.90748867 |
| Olfr1351 | 0.00814 | -1.636331 |
| Nr1i3 | 0.7133 | 0.23583421 |
| Tstd3 | 0.0046 | 1.03487173 |
| Serinc1 | 0.00271 | 1.18058084 |
| Slc2a8 | 0.44725 | -0.27864756 |
| Sepn1 | 0.01921 | -0.55580039 |
| Olfr332 | 0.06122 | -1.79565677 |
| Fads1 | 0.78571 | 0.04710347 |
| Mkl2 | 0.05299 | -1.73900778 |
| 1700093J21Rik | 0.03869 | 0.76323001 |
| Abi3bp | 0.00774 | 2.08872276 |
| Serpina3m | 0.00243 | -1.32392165 |
| Pdc | 0.00144 | -1.29051721 |
| Fbxo46 | 0.00989 | -0.83608729 |
| Jund | 0.00166 | -1.00565947 |
| Inhbe | 0.52411 | -0.31635741 |
| Dhx16 | 0.01368 | -0.54378222 |
| Hras | 0.06093 | -0.69737232 |
| Angptl1 | 0.07152 | 0.47929654 |
| Olfr638 | 0.06102 | -0.98161948 |
| Phyhd1 | 0.093 | 0.43760639 |
| Fbxw9 | 0.07393 | -0.41732873 |
| Larp7 | 0.59546 | -0.13573817 |
| Ndufc2 | 0.01939 | -0.76269599 |
| Ap1g2 | 0.05645 | -0.40871923 |
| Coprs | 0.06882 | -0.60903518 |
| Clic1 | 0.00144 | -1.24625428 |
| Ccsap | 0.74432 | 0.31288251 |
| Slc26a10 | 0.80608 | 0.06116928 |
| Tpm2 | 0.4429 | -0.17308548 |
| Lgi3 | 0.73984 | 0.22427182 |
| Orc1 | 0.00985 | -0.86187431 |
| Olfr320 | 0.00145 | -1.2388121 |
| Vps13c | 0.39418 | -0.22275996 |
| Mcm4 | 0.01727 | 0.58708528 |
| Mical1 | 0.00427 | -0.78224515 |
| Krtap19-2 | 0.00144 | -1.28898079 |
| Krt19 | 0.01044 | -2.04412373 |
| Rab39b | 0.00144 | -1.29085005 |
| Shc4 | 0.02107 | -0.73723327 |
| Exd2 | 0.00271 | 1.0415219 |
| Trappc11 | 0.00775 | 0.79703466 |
| Cds2 | 0.96627 | -0.00972907 |
| Arhgap24 | 0.14341 | 0.68956157 |
| Stx8 | 0.32451 | -0.26452062 |
| Acrbp | 0.01083 | 1.29204514 |
| Gm4681 | 0.00144 | -1.28898079 |
| Snai3 | 0.8371 | -0.10882923 |
| Tpk1 | 0.00145 | 2.58658946 |
| Grp | 0.00144 | -1.29085005 |
| Baiap3 | 0.21975 | -0.41957809 |
| Phf7 | 0.50774 | 0.18909309 |
| Setd1a | 0.02343 | -0.53983895 |
| Mvd | 0.01917 | -0.74154374 |
| Prkch | 0.28018 | 0.26620594 |
| Ifna4 | 0.00144 | -1.25118919 |
| Zfp688 | 0.01316 | -0.59958024 |
| Isca1 | 0.44965 | 0.20169244 |
| Hic1 | 0.00254 | -0.807843 |
| Trpv3 | 0.00144 | -1.30761071 |
| Prr13 | 0.00454 | -0.71624857 |
| Urb1 | 0.12957 | -0.71398314 |
| Timp4 | 0.04322 | -0.67631224 |
| Nisch | 0.02051 | -0.68994872 |
| Jak3 | 0.50062 | -0.14904275 |
| Cyp3a11 | 0.3286 | 0.33830647 |
| Gm7008 | 0.6379 | 0.3106398 |
| Camk2n2 | 0.02707 | -0.60753388 |
| Tnni2 | 0.00158 | -0.90518323 |
| Nsun3 | 0.0044 | 1.31523312 |
| Krt75 | 0.03429 | -1.64468471 |
| Cox15 | 0.00727 | 1.37984584 |
| Lefty1 | 0.00364 | -1.05608013 |
| Dync1h1 | 0.01245 | -0.5581154 |
| Slc25a30 | 0.33028 | 0.27580241 |
| Ero1lb | 0.00145 | 1.87398632 |
| Rab35 | 0.16115 | -0.30644678 |
| Gm15866 | 0.00144 | -1.28116048 |
| Ppie | 0.0307 | -0.61831051 |
| Zpbp | 0.03752 | 1.06800532 |
| Cpsf2 | 0.04412 | 0.37199675 |
| Cluh | 0.0484 | -0.64431049 |
| Apcs | 0.00733 | -1.14281464 |
| Churc1 | 0.23328 | -0.38683336 |
| Reg3g | 0.19145 | -1.63246372 |
| Svil | 0.0036 | 1.1109641 |
| Tmc6 | 0.00259 | -0.91067429 |
| Kcnk7 | 0.0552 | -0.64851741 |
| Pou3f4 | 0.26133 | -2.24517788 |
| Nkx2-4 | 0.23478 | -0.7433352 |
| Zfp809 | 0.06104 | 1.28856569 |
| Icam5 | 0.15977 | -0.52912975 |
| Sult1c2 | 0.21516 | -0.47924126 |
| Zfp580 | 0.01193 | -0.59702715 |
| Ubac2 | 0.01458 | -0.80459677 |
| Crygn | 0.00144 | -1.25118919 |
| G6pd2 | 0.09961 | -2.04879009 |
| Commd4 | 0.06035 | -0.38361069 |
| Olfr748 | 0.00149 | -1.25408 |
| Magee2 | 0.00415 | -1.18931869 |
| 4930568D16Rik | 0.03685 | -1.38032643 |
| Sharpin | 0.02231 | -0.66409085 |
| Fetub | 0.15023 | -0.54741572 |
| Cript | 0.00438 | 0.80664052 |
| 2410015M20Rik | 0.05075 | -0.66336869 |
| Bmp5 | 0.06922 | -1.19982069 |
| Mal2 | 0.00144 | -1.52594014 |
| Tnnc2 | 0.02272 | -0.70736726 |
| Prdx1 | 0.70326 | 0.14610362 |
| Fam98a | 0.00538 | 0.82967773 |
| Svs5 | 0.02324 | -1.65679785 |
| Zfyve27 | 0.0189 | -0.59775341 |
| Olfr322 | 0.00144 | -1.23984951 |
| Olfr1020 | 0.00768 | -0.91006235 |
| Marcks | 0.88408 | -0.02882449 |
| Lcp2 | 0.72305 | -0.1057304 |
| Olfr570 | 0.65479 | -0.31540864 |
| Mpv17l | 0.00144 | 1.79436978 |
| Lmod2 | 0.00518 | 0.69850087 |
| Tex16 | 0.02727 | -1.92773096 |
| Rnf216 | 0.01998 | -0.46118551 |
| Olfr397 | 0.41685 | 0.40988469 |
| St5 | 0.01303 | -0.64108 |
| Mrpl38 | 0.08501 | -0.52237349 |
| Dbx2 | 0.12734 | -0.61998985 |
| Ism1 | 0.03215 | -0.43390515 |
| Dhrs4 | 0.75218 | 0.1792187 |
| Ldhal6b | 0.76763 | -0.0788474 |
| Slc16a2 | 0.00687 | -0.78691782 |
| Vps26b | 0.00337 | 0.85663618 |
| Homer2 | 0.24915 | 0.40551463 |
| Cyb561d1 | 0.01997 | -0.53874052 |
| Sirt7 | 0.84131 | 0.03862904 |
| Sema6a | 0.07091 | 0.56958914 |
| Ppt1 | 0.03649 | -0.6698357 |
| AW552889 | 0.04702 | -1.1399855 |
| Itgae | 0.47312 | 0.24839537 |
| Hells | 0.17957 | 0.55332387 |
| Rap1a | 0.0054 | 1.46807504 |
| Chd3os | 0.06572 | -0.70311749 |
| C1qb | 0.06641 | -0.54714626 |
| Olfr656 | 0.81288 | -0.10453659 |
| Jph1 | 0.07284 | -0.50243999 |
| Ccnl1 | 0.00144 | 1.88635043 |
| Lipk | 0.00144 | -1.26560808 |
| Vps45 | 0.32332 | -0.23198063 |
| Upk2 | 0.16678 | -0.49716129 |
| Tyms-ps | 0.82089 | 0.0618596 |
| Pcsk7 | 0.92475 | 0.01713246 |
| Plgrkt | 0.01629 | 0.73936475 |
| Rab39 | 0.66008 | 0.10602526 |
| Cnfn | 0.90476 | 0.07837036 |
| Myod1 | 0.00144 | -1.25118919 |
| Tbce | 0.00171 | 1.89393461 |
| Odf4 | 0.52934 | 0.2723712 |
| Lta | 0.01367 | -1.23334054 |
| Nup37 | 0.08773 | 0.80173185 |
| Krtap6-2 | 0.00144 | -1.26934956 |
| Serinc4 | 0.00144 | -1.21148704 |
| Olfr1100 | 0.06483 | -0.88148784 |
| Cnn1 | 0.03789 | -1.30417595 |
| Zcchc18 | 0.36948 | 0.20601571 |
| Peli2 | 0.00707 | 0.74356419 |
| Lztfl1 | 0.00759 | 0.83039756 |
| Csrp2bp | 0.54591 | -0.26272653 |
| Pdk4 | 0.76531 | -0.06283173 |
| Exoc4 | 0.20394 | 0.37835111 |
| Amt | 0.04078 | 0.86413535 |
| Rbpms | 0.28912 | 0.28680261 |
| Tmem256 | 0.00145 | -1.26219879 |
| Dync1li2 | 0.00215 | -1.05400793 |
| Akr1cl | 0.13921 | -2.7891159 |
| Ndel1 | 0.2763 | 0.17508197 |
| Olfr1189 | 0.04302 | -2.05172912 |
| Gstt2 | 0.00813 | -0.91978074 |
| Il17c | 0.00874 | -0.93360403 |
| 0610010K14Rik | 0.13217 | -0.50175565 |
| C130081A10Rik | 0.95678 | -0.01771134 |
| Gm33589 | 0.00144 | -1.29085005 |
| Ang2 | 0.29426 | -0.27468318 |
| Txndc17 | 0.61808 | -0.09667097 |
| 1700021F05Rik | 0.7985 | -0.04172047 |
| Med11 | 0.0023 | -0.86242821 |
| Scel | 0.10152 | -0.82496539 |
| Slc25a45 | 0.23226 | -0.28761044 |
| Impg1 | 0.00144 | -1.28116048 |
| Taf5l | 0.41978 | -0.17457254 |
| Gm14123 | 0.00592 | -1.07650415 |
| Cd109 | 0.00327 | 1.45878443 |
| Rnase10 | 0.12248 | 0.73938866 |
| Mboat1 | 0.21736 | -0.22696466 |
| Trav9d-4 | 0.00193 | -1.24713717 |
| Arl5c | 0.00144 | -1.28116048 |
| Nckap5l | 0.01909 | -0.49174156 |
| Fcrl1 | 0.00145 | -1.24191372 |
| Fcrl1 | 0.03158 | -0.9668438 |
| 2210013O21Rik | 0.17988 | -0.2557901 |
| Ctnnd1 | 0.06332 | -0.85008993 |
| Gm13288 | 0.07481 | -0.33312871 |
| Fbl | 0.00608 | -0.67786327 |
| Endog | 0.10461 | -0.71499154 |
| Snapc4 | 0.97378 | -0.00828323 |
| Lsm3 | 0.56173 | -0.17975295 |
| Pacrg | 0.46168 | -0.34500553 |
| Ptges2 | 0.07538 | -0.74310592 |
| Tmem167b | 0.04317 | -0.53589181 |
| Olfr1 | 0.00149 | -1.2703832 |
| Smok2a | 0.00144 | -1.28116048 |
| Amotl2 | 0.01044 | -0.65136215 |
| Opn5 | 0.04341 | -0.98185074 |
| Llgl2 | 0.10753 | -0.55253815 |
| Llgl2 | 0.55269 | -0.29254199 |
| Ramp1 | 0.60532 | -0.19684679 |
| Spast | 0.04512 | 0.48475281 |
| Timm10 | 0.13153 | -0.48989895 |
| Slc38a7 | 0.25674 | -0.3095464 |
| Ecsit | 0.01095 | -0.75505652 |
| Sat1 | 0.01668 | -0.78495335 |
| Rasal1 | 0.00144 | -1.28116048 |
| Hexim1 | 0.2774 | 0.17545963 |
| Prex1 | 0.00144 | -0.99000083 |
| Poc1b | 0.54619 | -0.14247602 |
| Il17ra | 0.08027 | 0.55744724 |
| Stoml1 | 0.01685 | -0.72749018 |
| Olfr1161 | 0.00179 | -1.70284149 |
| Tmem141 | 0.00701 | -0.94410427 |
| Ube2q1 | 0.55096 | -0.09586641 |
| Nudt21 | 0.41748 | -0.18440334 |
| Npc2 | 0.03712 | -0.60863165 |
| Actn1 | 0.13106 | 0.25298817 |
| Irx6 | 0.04695 | -0.89294925 |
| Clnkos | 0.00144 | -1.30713006 |
| Noc3l | 0.06963 | -0.31625996 |
| Nfu1 | 0.35286 | 0.38091406 |
| Kcnj9 | 0.0018 | -1.38022641 |
| Fezf1 | 0.00145 | -1.24191372 |
| Nek5 | 0.00175 | -1.26015721 |
| Utp14a | 0.41502 | -0.19662206 |
| Htatsf1 | 0.04839 | 1.02710808 |
| Dusp4 | 0.85816 | -0.04679853 |
| Ogfrl1 | 0.00144 | 1.03906436 |
| Zc3h3 | 0.02397 | -0.89967717 |
| Snx2 | 0.00145 | 1.30600635 |
| Actl11 | 0.78678 | -0.15235919 |
| Adam18 | 0.00852 | -1.0980267 |
| Mrap | 0.05356 | -0.5070035 |
| Lrg1 | 0.06839 | -0.68516235 |
| P3h1 | 0.0795 | -0.32931289 |
| Extl1 | 0.02236 | -0.85376164 |
| Snrnp27 | 0.70104 | 0.10698393 |
| 1700112E06Rik | 0.02501 | -0.59413101 |
| Ift20 | 0.08968 | -0.32307927 |
| Rragc | 0.0296 | 0.58192439 |
| Arxes1 | 0.0076 | -1.07716047 |
| Irf5 | 0.94953 | -0.01120341 |
| Armcx4 | 0.00144 | 1.15294959 |
| Rhag | 0.00144 | -1.26560808 |
| Sdk2 | 0.01664 | -1.18812606 |
| Tle4 | 0.02619 | 0.5164086 |
| Zcchc7 | 0.02078 | 1.15899084 |
| Syn2 | 0.67 | 0.09909342 |
| Noc4l | 0.2385 | -0.33283407 |
| Hspb7 | 0.64196 | -0.18016607 |
| Gmeb2 | 0.80525 | 0.12290744 |
| Arhgap33 | 0.53559 | -0.11935755 |
| Aspdh | 0.00337 | -0.91599644 |
| Agpat4 | 0.00417 | -0.79201075 |
| Rhox9 | 0.00144 | -1.28116048 |
| Sgta | 0.00586 | -0.87862864 |
| Krtap19-3 | 0.00144 | -1.26560808 |
| Scn4a | 0.00146 | -1.05581708 |
| Tbrg4 | 0.98286 | 0.01271206 |
| Ddx41 | 0.14594 | -0.37048275 |
| Dohh | 0.00144 | -1.25267157 |
| Pafah2 | 0.46488 | 0.18813911 |
| Hsf4 | 0.66526 | -0.18906702 |
| Prkra | 0.60454 | 0.12186328 |
| Tmem180 | 0.01224 | -0.57971136 |
| Slc2a12 | 0.86836 | -0.04719363 |
| Tsc2 | 0.08178 | -0.4829915 |
| Hspb11 | 0.31055 | 0.25490538 |
| Pdgfra | 0.81845 | 0.059318 |
| Tnni3k | 0.88864 | 0.02574078 |
| Sptlc1 | 0.55128 | 0.14789213 |
| Snora34 | 0.02534 | 0.76099889 |
| Pi4k2a | 0.05001 | -0.57988182 |
| Gm26698 | 0.13358 | -0.73605578 |
| Zbtb5 | 0.02825 | -0.60316586 |
| Psmb8 | 0.00181 | -1.2112448 |
| Upk3bl | 0.0346 | -0.61851809 |
| Upk3bl | 0.00852 | -1.47522534 |
| Cep76 | 0.05857 | 0.72117199 |
| 5830432E09Rik | 0.00144 | -1.26560808 |
| Ube2t | 0.53604 | 0.20389748 |
| Thap4 | 0.07289 | -0.59754678 |
| Kdelr2 | 0.01402 | -0.5292716 |
| Metap2 | 0.00144 | 1.73529745 |
| Olfr111 | 0.00144 | -1.26560808 |
| Rab1b | 0.01431 | -0.83126024 |
| 6820408C15Rik | 0.07505 | -0.49887909 |
| Thop1 | 0.01686 | -0.80484949 |
| Tnrc6b | 0.12922 | 0.79129083 |
| Tmem263 | 0.00353 | -1.06721129 |
| BC016548 | 0.00144 | -1.31495282 |
| Rab23 | 0.10648 | 0.50435653 |
| Prph | 0.00787 | -1.56387812 |
| Plk1 | 0.12588 | -0.55739993 |
| Zfp748 | 0.00144 | -1.24613586 |
| Prrt2 | 0.9981 | 0.00204294 |
| Adam1b | 0.00144 | -1.20579466 |
| Rilpl2 | 0.13614 | -0.38521703 |
| Gpr162 | 0.00914 | -0.86090801 |
| Hhipl2 | 0.64549 | -0.34929823 |
| Ccdc129 | 0.01974 | -2.82041465 |
| Baalc | 0.22545 | -0.25466237 |
| Apbb1 | 0.63468 | -0.38008335 |
| Adam5 | 0.34469 | -2.33417956 |
| Nt5m | 0.55233 | -0.23808569 |
| Klra12 | 0.00356 | -1.73046824 |
| Slc25a20 | 0.93894 | 0.04540189 |
| Parp4 | 0.01106 | -0.82323266 |
| Xrcc1 | 0.04233 | -0.45492582 |
| Slc35f6 | 0.23932 | -0.39720919 |
| Traf1 | 0.15557 | -0.36230546 |
| Pgbd5 | 0.12555 | -3.09211111 |
| Morf4l1 | 0.73973 | -0.05505132 |
| Ptpn3 | 0.0058 | -0.87986457 |
| Slc39a2 | 0.00469 | -1.01429296 |
| Ehd2 | 0.00579 | -0.65750263 |
| Galnt10 | 0.00144 | -1.37779255 |
| Ly6d | 0.0165 | -0.85307933 |
| Eprs | 0.04564 | -0.4171368 |
| Slc25a37 | 0.04024 | 0.5633713 |
| A930010G16Rik | 0.71537 | -0.09504194 |
| Isoc2b | 0.006 | -0.86613734 |
| Amn | 0.04312 | -0.7134285 |
| Cox6c | 0.00289 | -0.91556817 |
| Olfr473 | 0.02443 | -1.8485875 |
| Cd151 | 0.0075 | -0.92573741 |
| Ube2ql1 | 0.32579 | -0.49619465 |
| Gp6 | 0.00636 | -1.55768461 |
| Ppp1r2-ps7 | 0.0244 | -1.76651198 |
| Omd | 0.00887 | 2.20351448 |
| Heatr5b | 0.06929 | 0.92778782 |
| Scn1b | 0.14431 | -0.41795408 |
| S100a10 | 0.01128 | -0.66292576 |
| Mlh3 | 0.4526 | 0.23701332 |
| Vipr2 | 0.04272 | 0.5679247 |
| Ppp1cb | 0.32256 | 0.27436261 |
| Pgam1 | 0.29197 | -0.27663424 |
| Cd79b | 0.23054 | -0.2565396 |
| 2200002J24Rik | 0.00144 | -1.20052149 |
| Olfr13 | 0.00186 | -1.38908362 |
| Akap12 | 0.07184 | 0.46834569 |
| Hand1 | 0.00238 | -1.77135533 |
| Olfr1364 | 0.00257 | -1.15440876 |
| Ddx55 | 0.01299 | 0.62761694 |
| Olfr732 | 0.00144 | -1.27165604 |
| Palm2 | 0.94127 | 0.032157 |
| Setd6 | 0.08709 | 0.33383638 |
| Ces1h | 0.00144 | -1.29051721 |
| Ttc39a | 0.2722 | 0.42039949 |
| Cnpy2 | 0.15375 | -0.32658614 |
| Uba3 | 0.00144 | 1.81950708 |
| 6430562O15Rik | 0.005 | 1.76960325 |
| Usp53 | 0.00144 | -1.31495282 |
| Nthl1 | 0.04721 | -0.44432761 |
| Arid4b | 0.13462 | -2.7143902 |
| Sugp1 | 0.07108 | -0.3978159 |
| Mmp2 | 0.03162 | -0.59839189 |
| Mesp1 | 0.00144 | -1.29746368 |
| Zfp87 | 0.02183 | 0.51637782 |
| Fancc | 0.05237 | -0.48365323 |
| Klhdc2 | 0.23932 | 0.42828853 |
| Defb7 | 0.11082 | -0.55748912 |
| Csf2ra | 0.00186 | 0.91680333 |
| Hykk | 0.13435 | -1.29975456 |
| Acad9 | 0.06996 | 0.51720306 |
| Pqlc1 | 0.04208 | -0.52992162 |
| Dmrtb1 | 0.15113 | -1.67886275 |
| Cyp3a41a | 0.96737 | -0.02909344 |
| Ddah2 | 0.00144 | -1.1178629 |
| Trerf1 | 0.95529 | -0.03389006 |
| Sla | 0.3223 | -0.71908196 |
| Ercc8 | 0.09749 | -0.34850531 |
| Slc17a9 | 0.42084 | -0.67937838 |
| Selo | 0.31091 | -0.23032193 |
| Gtf3c1 | 0.00261 | -0.9820379 |
| AA986860 | 0.13723 | -0.25865511 |
| Ipp | 0.01156 | 0.62481692 |
| Zkscan4 | 0.76569 | -0.1453051 |
| Hspa1l | 0.00178 | -1.19691941 |
| Prpf31 | 0.05196 | -0.38268745 |
| Rasl11a | 0.04662 | -1.77580762 |
| Slc16a4 | 0.00169 | 1.78226759 |
| Dscam | 0.00145 | -1.35893853 |
| Btg3 | 0.00187 | 1.02526962 |
| Olfr357 | 0.14486 | -2.81486694 |
| Ambn | 0.59293 | -0.46179934 |
| Krtap3-3 | 0.67565 | -0.4000082 |
| Eefsec | 0.18118 | -0.31578527 |
| Tarsl2 | 0.00269 | 1.15859146 |
| Pcbp3 | 0.06084 | -0.43836347 |
| Abhd1 | 0.50596 | -0.21403771 |
| Nbea | 0.00399 | 1.37201124 |
| Sh3yl1 | 0.07119 | 0.52728306 |
| Il20ra | 0.00144 | -1.27165604 |
| Pappa | 0.03833 | -0.55341007 |
| Ccdc176 | 0.04751 | -0.78251674 |
| Cwf19l2 | 0.00144 | 1.39257425 |
| Nid1 | 0.03297 | 0.52439426 |
| Mcpt2 | 0.03843 | -1.22820142 |
| Arhgap4 | 0.53138 | -0.19132864 |
| Il1rl1 | 0.00144 | -1.28898079 |
| 3010003L21Rik | 0.17047 | -0.42081128 |
| 1700003M02Rik | 0.00144 | -1.30017714 |
| Lcn9 | 0.40041 | -0.53196053 |
| Olfr1378 | 0.00144 | -1.26934956 |
| Olfr1223 | 0.00144 | -1.26560808 |
| Rab9 | 0.00144 | 1.08638771 |
| 4921530L21Rik | 0.00144 | -1.28898079 |
| Setmar | 0.3232 | 0.25576407 |
| Tmem81 | 0.03368 | -0.44328367 |
| Nkx6-3 | 0.00145 | -1.36286464 |
| Banp | 0.26385 | -0.22605297 |
| Olfr972 | 0.00144 | -1.28898079 |
| Vps26a | 0.00406 | 0.99590969 |
| Snta1 | 0.02526 | -0.72029472 |
| Abhd16a | 0.35233 | -0.3517687 |
| BC025446 | 0.00727 | -1.07804057 |
| Rpl28 | 0.00291 | -0.9662837 |
| Serpina4-ps1 | 0.13931 | -0.73264646 |
| Gprc6a | 0.00144 | -1.31884045 |
| Gng5 | 0.00144 | -1.26560808 |
| Prim2 | 0.42219 | 0.15617181 |
| Qser1 | 0.00144 | 1.30432249 |
| Ifnar2 | 0.13276 | -0.31700333 |
| P2ry12 | 0.52359 | -0.77867844 |
| Rn18s | 0.00144 | -1.14992741 |
| Dnajc8 | 0.0142 | 0.65513997 |
| Sdhaf1 | 0.13213 | -0.48310979 |
| Adprh | 0.05851 | -0.57699679 |
| Slc36a1 | 0.12221 | -0.68245487 |
| 4930402K13Rik | 0.07785 | -2.31072167 |
| Aldh6a1 | 0.00157 | 1.55328834 |
| Mtnr1b | 0.07447 | -0.81940862 |
| Numa1 | 0.00389 | -0.74714612 |
| Angptl4 | 0.12095 | -1.25289773 |
| Stap1 | 0.71266 | 0.22438749 |
| Kctd10 | 0.07317 | -0.30429214 |
| Tdg | 0.03014 | -0.41395214 |
| Rhoq | 0.02116 | 0.76390069 |
| Tnnt2 | 0.53688 | -0.09560627 |
| Mhrt | 0.7242 | -0.13280717 |
| Myh4 | 0.08011 | -1.83648437 |
| Vopp1 | 0.9355 | 0.01364593 |
| Trpm1 | 0.00144 | -1.25118919 |
| Bmp2k | 0.6257 | 0.09271014 |
| Slc7a15 | 0.04484 | -1.68658069 |
| Gzmm | 0.07973 | -0.41800071 |
| Ubl7 | 0.02025 | -0.55770846 |
| 4933434E20Rik | 0.49811 | 0.20377002 |
| Pcdhb17 | 0.02413 | 0.79575343 |
| Ovgp1 | 0.01652 | 2.11884103 |
| Cd53 | 0.01061 | 1.00420001 |
| Ddx26b | 0.0015 | 2.10532717 |
| Dsn1 | 0.00144 | 1.61941055 |
| 2410131K14Rik | 0.54723 | -0.26347935 |
| Glce | 0.07935 | 0.41539372 |
| Adcy2 | 0.00197 | -1.07716427 |
| Cd320 | 0.71981 | -0.18762857 |
| Efemp1 | 0.73852 | 0.11917353 |
| Ern2 | 0.56446 | -0.26654463 |
| Aldob | 0.75288 | -0.34232243 |
| Galnt14 | 0.00144 | -1.26934956 |
| Lrrc4 | 0.02106 | -1.04453848 |
| Pipox | 0.03157 | 0.99260041 |
| Inpp5d | 0.20431 | -0.2585553 |
| Golga7b | 0.00785 | -0.8331952 |
| Rnf43 | 0.02018 | -1.13435495 |
| Gins1 | 0.2341 | 0.34127729 |
| Gins1 | 0.16336 | 1.02665092 |
| Pdpr | 0.02743 | 0.78094425 |
| Senp7 | 0.01515 | 0.50540203 |
| 1190003K10Rik | 0.05227 | -1.64168332 |
| Cryl1 | 0.11223 | -0.34727111 |
| Atp6v0e | 0.07899 | -0.61678272 |
| Mgst3 | 0.06128 | -0.68455463 |
| Fabp4 | 0.13819 | -0.49062922 |
| Cyb5b | 0.85191 | -0.09394935 |
| Lsm5 | 0.10257 | -0.37706633 |
| Aebp1 | 0.00514 | -0.79718233 |
| Tipin | 0.01156 | 0.60693632 |
| Srsf9 | 0.00809 | -0.6318144 |
| Dexi | 0.0523 | -0.73085824 |
| Kcne3 | 0.43948 | 0.29747981 |
| Otub2 | 0.39214 | -0.32578845 |
| Zfpm2 | 0.57134 | -0.10971391 |
| Prox2 | 0.31929 | -0.23489239 |
| Emc7 | 0.27423 | 0.28946507 |
| Dido1 | 0.00932 | 0.62828814 |
| Fam204a | 0.00144 | 1.37672246 |
| Man2b1 | 0.0735 | -0.40956942 |
| Rps6kc1 | 0.96672 | -0.00952014 |
| Krt33b | 0.00144 | -1.27992547 |
| Plac8l1 | 0.00144 | -1.26428512 |
| Des | 0.71181 | -0.23367108 |
| Mrpl46 | 0.24451 | -0.25603366 |
| Cox5a | 0.09805 | -0.48139803 |
| Olfr1228 | 0.00144 | -1.31884045 |
| Ctnnbip1 | 0.00629 | -0.63601308 |
| Calb1 | 0.00144 | -1.24466058 |
| Afap1 | 0.22715 | -0.21263142 |
| Chn1 | 0.05364 | 0.53658781 |
| Agpat6 | 0.82118 | -0.07492348 |
| Agpat6 | 0.00195 | -0.92207841 |
| Tmem114 | 0.00413 | -1.81210614 |
| Krtap4-7 | 0.00144 | -1.27165604 |
| Spag7 | 0.14042 | 0.83524363 |
| Slco1a4 | 0.36556 | 0.58804241 |
| 1810055G02Rik | 0.8177 | -0.05955578 |
| Scin | 0.00216 | 1.235111 |
| Csl | 0.47171 | -0.21263765 |
| Ermp1 | 0.63459 | -0.22990975 |
| Hoxa3 | 0.0029 | -1.17200505 |
| H60a | 0.34978 | -0.40318615 |
| Lrfn1 | 0.00371 | -1.38485247 |
| Ltn1 | 0.0067 | -0.67874327 |
| Slx1b | 0.07633 | -0.4338262 |
| Zcchc4 | 0.00557 | 1.5566494 |
| Fhl1 | 0.43169 | -0.13914727 |
| Apoc2 | 0.00512 | -1.02897488 |
| Aldh1a1 | 0.92382 | -0.02067176 |
| Asic2 | 0.00144 | -1.26560808 |
| Pnpla5 | 0.11769 | -1.4277965 |
| Nudt16 | 0.16383 | -0.25283154 |
| Pyhin1 | 0.27591 | 0.20739499 |
| Uts2 | 0.00145 | -1.24082201 |
| Esyt1 | 0.00929 | -0.68634966 |
| Sv2c | 0.00144 | -1.28116048 |
| Wbp4 | 0.00471 | 1.02047132 |
| Olfr1188 | 0.00144 | -1.25927895 |
| Phf20l1 | 0.24707 | 0.1962663 |
| Chp2 | 0.15268 | -0.49281533 |
| Tspan14 | 0.01122 | -0.56114094 |
| Gucy1b2 | 0.02486 | 0.78242193 |
| Eya2 | 0.20764 | -0.39187155 |
| Rho | 0.04991 | -0.93578497 |
| 4930579F01Rik | 0.3172 | -0.28180433 |
| Lin7c | 0.00144 | 1.92246792 |
| Dcn | 0.32276 | -0.24785734 |
| Cyct | 0.33671 | 0.45622105 |
| Cisd2 | 0.12765 | 0.50602439 |
| Ints7 | 0.52732 | -0.11804128 |
| Tspan1 | 0.00144 | -1.29085005 |
| Olfr1393 | 0.52061 | -0.26536508 |
| Ndnl2 | 0.01706 | -0.47843623 |
| Clvs2 | 0.00144 | -1.30713006 |
| Senp7 | 0.05683 | 0.37378026 |
| Ulk4 | 0.03273 | -1.10366211 |
| Agbl1 | 0.00144 | -1.29746368 |
| 2010300C02Rik | 0.08224 | 0.46064295 |
| Tspyl4 | 0.00144 | -1.28116048 |
| Reg2 | 0.00144 | -1.27165604 |
| Tmem120a | 0.01861 | -0.74452693 |
| Hcn3 | 0.00144 | -1.28116048 |
| Gzmb | 0.76162 | -0.25432027 |
| Rpa1 | 0.66702 | -0.07699906 |
| Aox1 | 0.0045 | 0.76369931 |
| Dicer1 | 0.00547 | 0.78292217 |
| Asb4 | 0.00202 | -1.11565287 |
| Jmjd6 | 0.29292 | -0.31379139 |
| Enpp3 | 0.39685 | 0.30176152 |
| Stk4 | 0.14827 | -0.32298762 |
| Olfr672 | 0.00163 | -1.29258142 |
| Clrn1 | 0.09575 | -0.90052009 |
| Abce1 | 0.00144 | 1.55216357 |
| Clptm1 | 0.01138 | -0.82248146 |
| Srl | 0.60306 | -0.18651352 |
| Ugcg | 0.00492 | 0.75619873 |
| Top1mt | 0.24968 | -0.25538672 |
| Cib3 | 0.00145 | -1.22376446 |
| Pqlc3 | 0.00307 | 0.82247367 |
| Ankrd16 | 0.05292 | -0.73420972 |
| Mybbp1a | 0.03678 | -0.56211129 |
| Surf1 | 0.24132 | -0.37212901 |
| Vmn1r74 | 0.00149 | -1.22582153 |
| BC003331 | 0.01725 | 0.49514004 |
| Kif18a | 0.00585 | 1.1443198 |
| Dyx1c1 | 0.04265 | 0.48086641 |
| Cst3 | 0.0126 | -0.78502803 |
| Mbd5 | 0.04086 | 0.85108455 |
| Clcn2 | 0.06009 | 0.47992664 |
| Rb1cc1 | 0.34895 | 0.36410698 |
| 6030426L16Rik | 0.99196 | -0.0098446 |
| Cbl | 0.32694 | -0.66459205 |
| Fermt2 | 0.00417 | -0.67495476 |
| 4933433C11Rik | 0.00145 | -1.33579318 |
| Rnf145 | 0.87902 | 0.04140103 |
| Slc25a41 | 0.0062 | -1.33031495 |
| Tcl1b4 | 0.02703 | -1.24214498 |
| Kctd15 | 0.26958 | -0.17948716 |
| Fam151b | 0.1925 | 0.27705456 |
| Ubiad1 | 0.03811 | -0.73756055 |
| Idh2 | 0.85769 | 0.08939268 |
| Cgrrf1 | 0.00174 | -0.87707627 |
| Colgalt2 | 0.33955 | -0.21703999 |
| Gpihbp1 | 0.00518 | -0.82669335 |
| Akr1b7 | 0.00368 | -1.10379914 |
| Arpc1b | 0.51344 | -0.12849507 |
| Tmem218 | 0.15419 | 0.44335902 |
| 4930442E04Rik | 0.00144 | -1.26934956 |
| Tex15 | 0.32425 | -0.41741819 |
| Il20 | 0.00144 | -1.28898079 |
| Ttc32 | 0.15949 | 0.52511371 |
| Sema4g | 0.00144 | -1.20555712 |
| LOC102637286 | 0.05701 | -0.65728248 |
| Syt2 | 0.00144 | -1.24811894 |
| Gm12100 | 0.00144 | 1.45773061 |
| 2700068H02Rik | 0.01249 | -2.38994952 |
| Btbd7 | 0.00144 | 1.91941364 |
| Zfp119b | 0.00144 | 2.32834181 |
| Mef2b | 0.36456 | -0.34734552 |
| Olfr1414 | 0.01705 | -2.54110897 |
| Eif4ebp1 | 0.28386 | -0.3320975 |
| Slc6a17 | 0.55988 | -0.18256295 |
| Ssx2ip | 0.37612 | 0.18621788 |
| Pabpc6 | 0.02475 | -0.74331659 |
| Pspc1 | 0.00211 | 1.1129354 |
| Trappc9 | 0.01428 | -0.65482336 |
| Cyp2j9 | 0.31652 | -0.79849781 |
| Gm30582 | 0.00144 | -1.28898079 |
| D6Wsu163e | 0.03227 | 0.49228953 |
| Fam13a | 0.02541 | 0.65200604 |
| Phlda3 | 0.00144 | -1.16256362 |
| Dgkq | 0.69205 | 0.09601307 |
| Zfp598 | 0.94133 | 0.01326716 |
| Dpep3 | 0.00145 | -1.26219879 |
| Reg1 | 0.0088 | -1.08115135 |
| 5430419D17Rik | 0.00149 | -1.25408 |
| Nckipsd | 0.09502 | -0.34049996 |
| Myo1d | 0.00144 | 1.56251074 |
| Slc25a40 | 0.00145 | 1.22480695 |
| Bsg | 0.86931 | 0.11552929 |
| Snrnp25 | 0.00223 | -1.03899978 |
| Mcts2 | 0.17109 | -0.28799089 |
| Slc19a2 | 0.1892 | 0.29095218 |
| Zfand1 | 0.13966 | 0.3715437 |
| Zfand1 | 0.12351 | 0.28396535 |
| Slc2a13 | 0.00144 | -1.25118919 |
| Chchd1 | 0.69355 | -0.08332825 |
| Tmem56 | 0.00289 | -1.20921372 |
| Zc3h10 | 0.01471 | -0.53873366 |
| Shprh | 0.02121 | 1.25652127 |
| Rapgef2 | 0.98774 | 0.02094734 |
| Slc25a31 | 0.42748 | 0.22632 |
| Zfp322a | 0.10103 | 0.46679595 |
| Ufd1l | 0.14453 | 0.2389015 |
| Sgip1 | 0.40344 | 0.52251515 |
| Tmem165 | 0.00785 | 0.59476718 |
| Zfp810 | 0.00144 | 2.5941328 |
| Olfr100 | 0.65067 | -0.16286561 |
| Plk5 | 0.03687 | -0.48345498 |
| Rnf20 | 0.75752 | -0.10093564 |
| Vmn1r80 | 0.00197 | -1.16119516 |
| Ncbp1 | 0.06109 | 0.33447147 |
| Olfr1039 | 0.04555 | -1.3121725 |
| Acot7 | 0.02987 | -0.62843945 |
| Sis | 0.07117 | -0.91485959 |
| Tlcd2 | 0.13929 | 0.69646511 |
| Fcgr3 | 0.89741 | 0.03086061 |
| Olfr1106 | 0.00879 | -1.05401892 |
| Zfp423 | 0.22221 | -0.6319649 |
| 9330179D12Rik | 0.0017 | -1.19401501 |
| Cops2 | 0.00144 | 1.85477309 |
| Pdia4 | 0.02942 | -0.49767134 |
| Polr3b | 0.20397 | -0.32495777 |
| Daam2 | 0.80125 | 0.11114124 |
| Baiap2l2 | 0.87852 | -0.08869774 |
| Crb3 | 0.00145 | -1.22376446 |
| Tlx3 | 0.02772 | -0.98161948 |
| Cops3 | 0.03296 | 0.70571675 |
| Sftpc | 0.09194 | -2.03398747 |
| Ccl22 | 0.00144 | -1.28116048 |
| Rfx2 | 0.59215 | -0.22783526 |
| St7l | 0.36225 | -0.26779489 |
| Yipf1 | 0.2979 | -0.36483761 |
| Pth1r | 0.0052 | -0.87879165 |
| Ppib | 0.00268 | -0.79019932 |
| Itgb5 | 0.00144 | -1.07401841 |
| Cacng7 | 0.97567 | 0.01132518 |
| Ifi44l | 0.00158 | -1.2446827 |
| Cwc22 | 0.00188 | 2.32594746 |
| Igsf3 | 0.00144 | -1.01610189 |
| 1700066C05Rik | 0.37082 | 0.86046128 |
| Glb1 | 0.01089 | -0.71113152 |
| Olfr341 | 0.08855 | -2.71495302 |
| Alas2 | 0.10299 | -0.63777695 |
| Rsrc2 | 0.00722 | 1.15156125 |
| Gbp8 | 0.02215 | 0.97658171 |
| Plekhg3 | 0.01063 | 1.28025155 |
| Lenep | 0.66814 | -0.09965362 |
| Was | 0.02967 | -0.54769073 |
| Zbtb25 | 0.03932 | 0.47885706 |
| Prune2 | 0.00144 | 1.35685351 |
| Ube2cbp | 0.91459 | -0.0227759 |
| Lrtm1 | 0.19616 | -0.47968281 |
| Golga2 | 0.03419 | -0.41186953 |
| Fam206a | 0.01982 | -0.92665949 |
| 1700093K21Rik | 0.00144 | -1.20030135 |
| Chek1 | 0.17912 | 0.51311122 |
| Pus3 | 0.00144 | 2.25065509 |
| Lonp2 | 0.04466 | 0.47787236 |
| Arfip2 | 0.28441 | -0.17985603 |
| Olfr71 | 0.00586 | -1.04374818 |
| Ppa2 | 0.32966 | 0.33917386 |
| Ddx25 | 0.54073 | -0.35654067 |
| Serpina3g | 0.01477 | -1.47966397 |
| Sppl2a | 0.00144 | 2.08612592 |
| Hydin | 0.01076 | -1.69581904 |
| Zfp959 | 0.00144 | 2.20832858 |
| Mcemp1 | 0.21049 | 0.52688867 |
| Hpx | 0.23509 | -1.14134209 |
| Gm15631 | 0.15654 | -0.72368185 |
| Olfr799 | 0.00969 | -1.02369437 |
| Cd47 | 0.19137 | -0.40701911 |
| Hsbp1 | 0.69524 | -0.09888562 |
| 2410004B18Rik | 0.49747 | 0.20222546 |
| Spert | 0.43845 | -0.32029462 |
| Crip3 | 0.03403 | 0.93090798 |
| Josd2 | 0.04678 | -0.62956929 |
| Utp3 | 0.07683 | 0.36492259 |
| Gm39091 | 0.00144 | -1.28116048 |
| Papd4 | 0.00239 | 1.0525868 |
| Armt1 | 0.52483 | 0.32182398 |
| Wrn | 0.75039 | -0.07607512 |
| Lin7b | 0.19873 | -0.48241262 |
| Il27 | 0.01699 | -0.5380958 |
| Trmt61a | 0.66313 | -0.10011642 |
| Olfr821 | 0.70983 | -0.26165185 |
| Mcm3 | 0.02411 | -0.53328662 |
| Man2a1 | 0.00462 | 1.36859988 |
| Cyb561a3 | 0.01737 | -0.68122083 |
| Evpl | 0.00463 | -1.2594253 |
| Krt18 | 0.00543 | -1.11634695 |
| Clec2d | 0.03238 | -0.73661036 |
| C330018D20Rik | 0.34035 | 0.20010484 |
| Ctnna1 | 0.01597 | 0.58977379 |
| Fbxo25 | 0.45613 | -0.14676539 |
| Cmpk1 | 0.03039 | 0.51557693 |
| Adgb | 0.18831 | -0.58664225 |
| Pbp2 | 0.38941 | -0.42895101 |
| Wdr35 | 0.37206 | -0.17179672 |
| Vti1a | 0.05369 | -0.33662342 |
| Mfi2 | 0.01387 | -1.0387752 |
| Mylip | 0.12507 | 0.41164879 |
| Satb1 | 0.45977 | -0.13244045 |
| Hps4 | 0.01088 | -0.62285329 |
| Med1 | 0.78203 | 0.04403224 |
| Gm38484 | 0.04706 | -1.38743727 |
| COX2 | 0.00254 | -0.88121534 |
| Coro7 | 0.0249 | -0.49616469 |
| Fam96b | 0.03062 | -0.84844759 |
| Slc6a12 | 0.01625 | -1.11217381 |
| Wfdc15a | 0.09575 | -0.90052009 |
| Agt | 0.00144 | -1.33649132 |
| Vmn1r53 | 0.00145 | -1.25646236 |
| Thyn1 | 0.03081 | -0.50212206 |
| Vimp | 0.58021 | 0.13458324 |
| Eaf2 | 0.22981 | 0.45283263 |
| Fam71e1 | 0.54177 | -0.1367348 |
| Snx21 | 0.0182 | -0.81324386 |
| Cend1 | 0.21078 | -1.0006969 |
| Vapb | 0.00144 | -1.06920212 |
| Gtf2h2 | 0.00662 | 1.49229782 |
| Cd9 | 0.00144 | -1.31884045 |
| Trim28 | 0.603 | -0.18958282 |
| Sdc4 | 0.05847 | -0.53758686 |
| Mfsd8 | 0.00449 | 1.46536267 |
| Bhlhb9 | 0.00144 | 1.34051796 |
| Ptrhd1 | 0.01002 | -0.88905382 |
| Hkdc1 | 0.00144 | -1.24466058 |
| Nfkbid | 0.80412 | -0.12737967 |
| Dok7 | 0.0085 | -0.76177598 |
| Olfr622 | 0.00314 | -1.14703327 |
| Hypm | 0.26793 | -0.39031223 |
| Olfr1220 | 0.00144 | -1.29085005 |
| Ankrd12 | 0.13238 | 0.57311598 |
| Plcxd1 | 0.01729 | -1.21364624 |
| Agpat1 | 0.00144 | -1.09374502 |
| Hnf4g | 0.80935 | -0.25761975 |
| Pkhd1 | 0.00144 | -1.29051721 |
| Perm1 | 0.48373 | -0.21596741 |
| Ugt3a1 | 0.00549 | -1.16183131 |
| Tecpr2 | 0.22251 | -0.19509417 |
| Ptp4a3 | 0.04395 | -0.82320743 |
| Grap | 0.00756 | -0.72869923 |
| Bri3bp | 0.24659 | -0.26279019 |
| Htr5b | 0.00144 | -1.28116048 |
| Ovol2 | 0.00144 | -1.33632959 |
| Ablim2 | 0.96826 | -0.01522101 |
| Hspb6 | 0.7878 | 0.25116481 |
| Ssna1 | 0.09682 | -0.56439031 |
| Plekhd1os | 0.00285 | -1.22098834 |
| Cmtm3 | 0.02053 | -0.53841669 |
| Olfr522 | 0.4003 | -0.48827464 |
| 4930519F09Rik | 0.48317 | -0.10614204 |
| Pgls | 0.00144 | -1.28207662 |
| Bhmt2 | 0.00145 | -1.22376446 |
| Fam192a | 0.26758 | -0.26637797 |
| Mapkbp1 | 0.09419 | -0.75334397 |
| Ccdc30 | 0.34668 | -0.63893744 |
| Dazap2 | 0.75077 | -0.05389302 |
| Chd5 | 0.48436 | -0.31721233 |
| Tnrc6c | 0.00813 | 0.56943374 |
| Purg | 0.00144 | 1.37420884 |
| Gm16976 | 0.04003 | -1.43179544 |
| Tnp2 | 0.03789 | -0.68774302 |
| Sult1a1 | 0.03041 | 0.67344887 |
| Lyz2 | 0.13425 | 0.5157623 |
| Fasn | 0.00836 | -0.98421943 |
| Abi3bp | 0.14497 | 0.39411306 |
| N6amt1 | 0.37492 | -0.15405599 |
| Ubl4b | 0.00144 | -1.30017714 |
| Sfxn4 | 0.9854 | -0.00576034 |
| Olfr1136 | 0.00144 | -1.28898079 |
| Trat1 | 0.16516 | -0.78544604 |
| Cln5 | 0.029 | 0.51049528 |
| Gps2 | 0.90556 | 0.03606915 |
| Actr3b | 0.26879 | 0.31717658 |
| Txnrd1 | 0.31299 | 0.25189119 |
| Prl5a1 | 0.00144 | -1.22925777 |
| Otulin | 0.12658 | -0.33619266 |
| Ppp2r5c | 0.05613 | 0.42254286 |
| Mcee | 0.20909 | -0.40481601 |
| Trhde | 0.32353 | -0.40064513 |
| Rock2 | 0.18822 | 0.53904254 |
| Rab24 | 0.86548 | 0.04406833 |
| Gtf2a2 | 0.21729 | 0.56020348 |
| Zfp707 | 0.01607 | -0.54117791 |
| Zxdc | 0.97966 | -0.00733748 |
| Wasf3 | 0.03408 | -1.4554796 |
| Grcc10 | 0.00144 | -1.56249546 |
| Smyd2 | 0.32019 | -0.24167064 |
| Atp10a | 0.66922 | -0.14425649 |
| Pth2 | 0.47366 | -0.21751227 |
| Kif24 | 0.13031 | -0.36460926 |
| Mrps31 | 0.09339 | 0.38457031 |
| Usp42 | 0.02399 | 0.49507808 |
| Osmr | 0.00788 | 1.22335069 |
| Zfp143 | 0.01808 | 0.60796173 |
| Acat1 | 0.10686 | 0.30438846 |
| Utp20 | 0.04225 | 0.42822069 |
| Rpp40 | 0.17246 | 0.39572164 |
| Tmx4 | 0.00501 | 1.82515585 |
| Krt4 | 0.04178 | -1.10038984 |
| Stard5 | 0.02672 | -0.59980903 |
| Olfr610 | 0.03894 | -1.9269609 |
| Dgka | 0.00815 | 0.85971297 |
| Retsat | 0.39374 | -0.43044335 |
| Cxcr3 | 0.0026 | -1.82480044 |
| Glyat | 0.00772 | -1.44654337 |
| Syt10 | 0.99913 | -0.00274093 |
| Tmc5 | 0.23668 | 0.36060135 |
| Asz1 | 0.00144 | -1.26934956 |
| Anks4b | 0.00149 | -1.19348234 |
| Sult6b1 | 0.00279 | 1.15501248 |
| Nle1 | 0.33489 | -0.18589608 |
| Fdx1l | 0.12431 | -0.51877181 |
| Ptprn | 0.00144 | -1.30713006 |
| C2cd3 | 0.42139 | 0.3236994 |
| Pgf | 0.07069 | -0.44760689 |
| Igf1r | 0.38658 | -0.13739139 |
| Olfr347 | 0.00144 | -1.27165604 |
| Rab12 | 0.00225 | -1.27642686 |
| Perp | 0.03176 | 0.67324837 |
| Sectm1b | 0.35837 | -0.33793527 |
| Sectm1b | 0.0111 | -1.02598223 |
| Cetn1 | 0.05282 | -0.79057592 |
| Ylpm1 | 0.00254 | 1.04611292 |
| Ints5 | 0.03849 | -0.99888171 |
| Pcgf2 | 0.15709 | -0.92821162 |
| Uba1 | 0.84211 | 0.09691381 |
| Trpc3 | 0.00361 | 1.33426304 |
| Zfp764 | 0.62309 | 0.09734048 |
| Tgfb2 | 0.71655 | 0.13642907 |
| Slc24a2 | 0.04835 | 0.7798249 |
| Nat1 | 0.54521 | -0.32464418 |
| Mpo | 0.09889 | -0.65676535 |
| Cd3eap | 0.0022 | -0.84093115 |
| Mesp2 | 0.04707 | -1.17253574 |
| LOC102631780 | 0.00144 | -1.29746368 |
| Iffo1 | 0.81311 | 0.05179793 |
| Pddc1 | 0.00625 | -0.88688695 |
| Bod1 | 0.31971 | -0.2068588 |
| Defb11 | 0.80817 | 0.06126573 |
| Cmtm2a | 0.52382 | -0.11128442 |
| Hpdl | 0.0134 | -0.9280683 |
| Eepd1 | 0.15554 | -0.37740152 |
| Pcdhb21 | 0.05499 | 0.4525631 |
| Csf3 | 0.00527 | -1.50365397 |
| Paxbp1 | 0.00144 | 1.96815618 |
| Col2a1 | 0.32985 | -0.19682869 |
| Wdr18 | 0.01889 | -0.74142176 |
| Ccdc109b | 0.1063 | -0.6919657 |
| Lgals8 | 0.02987 | 0.51938815 |
| Wipf3 | 0.71 | 0.09104581 |
| Ccdc6 | 0.0237 | 0.67366993 |
| 8430408G22Rik | 0.00668 | -0.72667558 |
| Srpx | 0.58143 | 0.21278356 |
| 2210407C18Rik | 0.24719 | 0.32379286 |
| Bdp1 | 0.11576 | -0.38230953 |
| Kdr | 0.11139 | -0.41826733 |
| Irf2 | 0.83845 | -0.05473832 |
| Ppfia4 | 0.00282 | -0.87258679 |
| Btn1a1 | 0.00144 | -1.24613586 |
| Olfr832 | 0.00144 | -1.20555712 |
| Gm5134 | 0.00145 | -1.2388121 |
| Olfr73 | 0.00144 | -1.27699683 |
| Vmn1r192 | 0.14798 | 0.4649563 |
| Atg4a | 0.00144 | 1.40441651 |
| Lima1 | 0.1186 | 0.38542127 |
| Card11 | 0.02099 | -0.87792022 |
| Nr5a1 | 0.00286 | -1.29537735 |
| Prkd3 | 0.84622 | 0.1626261 |
| Gadd45g | 0.20154 | -0.34001903 |
| Kcnk6 | 0.00229 | 0.79248637 |
| Madd | 0.00145 | -1.25646236 |
| Igf2bp2 | 0.14299 | 0.79606932 |
| Usp11 | 0.38869 | 0.20453782 |
| Nid2 | 0.04764 | 0.40422673 |
| Nfs1 | 0.33022 | 0.36467829 |
| CYTB | 0.01091 | -0.57446376 |
| Nars | 0.01805 | 0.51117115 |
| Mxd3 | 0.16381 | -0.73810771 |
| Slc35f4 | 0.04506 | -0.58483569 |
| Txlnb | 0.00144 | 1.45927358 |
| Abcb5 | 0.02369 | -1.70395532 |
| Gm26994 | 0.00144 | -1.26934956 |
| Letm1 | 0.56743 | 0.27566021 |
| Gemin2 | 0.15435 | 0.27928099 |
| Mia3 | 0.06426 | 0.40152813 |
| Cnep1r1 | 0.00283 | 0.95312835 |
| AF529169 | 0.46221 | -0.87819776 |
| Olfr878 | 0.00145 | -1.2388121 |
| Dgkd | 0.42462 | -0.14247562 |
| Dbf4 | 0.00144 | 1.29007055 |
| Ccm2 | 0.12488 | -0.45145293 |
| Mcat | 0.19407 | -0.44209509 |
| Rxrb | 0.47037 | -0.27192774 |
| Rrn3 | 0.54395 | -0.11851449 |
| Pnliprp2 | 0.00144 | -1.34945434 |
| Glt28d2 | 0.00565 | 1.10379493 |
| Smim11 | 0.01179 | -0.66618849 |
| Lce1e | 0.00144 | -1.33632959 |
| Blm | 0.36608 | -0.20870937 |
| Crip2 | 0.00167 | -1.0583857 |
| Naa38 | 0.00159 | -1.22019605 |
| Taf4a | 0.00192 | 1.74548097 |
| Tmem86a | 0.1278 | -0.51676598 |
| Parp1 | 0.19901 | 0.53170079 |
| Ssr2 | 0.28777 | -0.39482138 |
| Mrps10 | 0.28548 | -0.33446084 |
| Qtrt1 | 0.12384 | -0.55114721 |
| Nr2c2ap | 0.02344 | -0.6250447 |
| Psip1 | 0.00405 | 1.43670387 |
| Gsdma | 0.04658 | -2.023711 |
| Camsap3 | 0.02736 | -0.58439411 |
| Banf1 | 0.53207 | -0.24749175 |
| Zufsp | 0.00327 | 1.33786596 |
| Shmt2 | 0.08317 | -0.61842289 |
| Olfr1330 | 0.01019 | -1.52939908 |
| Myh9 | 0.01195 | -0.68813173 |
| Mmp12 | 0.00346 | -1.22679402 |
| Fabp2 | 0.11438 | -0.70984427 |
| Lmna | 0.00144 | -1.23087427 |
| Olfr577 | 0.09035 | -2.4791351 |
| Dyrk1a | 0.10245 | -0.28145259 |
| Fkbp6 | 0.06115 | -1.63031101 |
| 1700121C10Rik | 0.22017 | -0.58282041 |
| Smim10l1 | 0.00144 | 1.95949708 |
| Cd6 | 0.01504 | -0.77453956 |
| Olfr488 | 0.00144 | -1.26560808 |
| Fbf1 | 0.08175 | -0.30623767 |
| Arhgef5 | 0.73787 | -0.05217317 |
| Cyp4x1 | 0.01702 | -1.00335122 |
| Ccbe1 | 0.02758 | -0.69751078 |
| Zfp346 | 0.98076 | 0.00686534 |
| Tdrd12 | 0.89804 | -0.05735596 |
| Spag9 | 0.00475 | 2.62914608 |
| Fbxo9 | 0.0153 | 0.61047896 |
| Gm40055 | 0.00144 | -1.30017714 |
| Pspn | 0.00145 | -1.22376446 |
| Prl2c3 | 0.00144 | -1.28116048 |
| Galnt5 | 0.00144 | -1.27165604 |
| Hist1h1t | 0.02236 | -1.1220352 |
| Nup188 | 0.14521 | -0.40170254 |
| 1700041B01Rik | 0.00227 | -1.41580707 |
| Atp7a | 0.01452 | 0.51048172 |
| Fpr1 | 0.23018 | -1.3258774 |
| Ccdc34 | 0.29526 | 1.3644575 |
| Olfr698 | 0.00144 | -1.25118919 |
| Dhrs7 | 0.02394 | -0.49206 |
| Slc14a1 | 0.44126 | -0.72655019 |
| Ptges | 0.17895 | -0.36150107 |
| Mtbp | 0.00343 | 0.89581474 |
| 1810062O18Rik | 0.70861 | 0.13021676 |
| Slc30a7 | 0.01044 | 1.43045097 |
| Tnks | 0.01677 | -0.82281634 |
| Zfr | 0.09647 | 0.58185605 |
| Xdh | 0.59036 | 0.13433873 |
| Cd300e | 0.9299 | -0.03243809 |
| Olfr1222 | 0.0644 | -1.95971112 |
| Shroom2 | 0.04213 | 1.21193131 |
| P4hb | 0.79204 | -0.06840594 |
| Orm3 | 0.17554 | 0.34991299 |
| Npy2r | 0.00144 | -1.28898079 |
| Elp6 | 0.86926 | -0.02945513 |
| Man2b2 | 0.00206 | -0.81726841 |
| Serpine3 | 0.00144 | -1.29281267 |
| Ypel5 | 0.0051 | 0.76923702 |
| Frmd5 | 0.10744 | -0.86649803 |
| Cox6a1 | 0.0034 | -1.05306504 |
| Rgma | 0.00144 | -1.36523171 |
| Cfap54 | 0.59764 | -0.19407757 |
| Calb2 | 0.00144 | -1.47430191 |
| Tnrc6a | 0.49332 | -0.27331913 |
| 4930449E01Rik | 0.00883 | -0.63519437 |
| Dfna5 | 0.03849 | -1.19366761 |
| Drosha | 0.65671 | 0.21797268 |
| Snap91 | 0.05831 | 0.68012926 |
| Sgce | 0.38922 | 0.20077908 |
| Phf10 | 0.37696 | -0.14616485 |
| Malsu1 | 0.43172 | -0.22047239 |
| Tgfbrap1 | 0.40946 | 0.50025283 |
| Adamts15 | 0.00786 | -0.91179057 |
| Aifm2 | 0.00639 | -0.77639506 |
| Ppox | 0.43008 | 0.13875899 |
| Pfkl | 0.03381 | -0.72912165 |
| Zfp455 | 0.00144 | 2.30938335 |
| Plekha6 | 0.14402 | 0.27716414 |
| Hoxa5 | 0.42473 | -0.56859576 |
| N4bp1 | 0.41958 | -0.13856191 |
| Olfr791 | 0.1605 | -1.56620514 |
| Galr2 | 0.02388 | -1.10247618 |
| Arsa | 0.00777 | -0.60466102 |
| Apoc3 | 0.00144 | -1.29051721 |
| Osgep | 0.4024 | -0.26876187 |
| Atp6v1f | 0.00957 | -0.71351061 |
| Tk2 | 0.74331 | 0.06905565 |
| Papolb | 0.00144 | -1.29049509 |
| 4933428G20Rik | 0.01941 | -0.69048772 |
| Fam118a | 0.05669 | 0.97031078 |
| Cd40lg | 0.00144 | -1.25118919 |
| 2810459M11Rik | 0.00158 | 1.48433512 |
| Olfr52 | 0.51953 | -0.37111382 |
| Rnaset2b | 0.0346 | 0.95498898 |
| Nsmaf | 0.00145 | 1.05579663 |
| Naca | 0.91431 | -0.02313686 |
| Olfr1299 | 0.00144 | -1.28898079 |
| Olfr691 | 0.05338 | -1.07725077 |
| Gprc5c | 0.02992 | -0.80563374 |
| Dchs2 | 0.00144 | -1.44803386 |
| Itga8 | 0.03912 | 1.21616878 |
| Kcnn2 | 0.07833 | 0.45138148 |
| Olfr584 | 0.04344 | -3.35746129 |
| Dcpp1 | 0.00144 | -1.29085005 |
| Gpr89 | 0.00919 | -0.69441116 |
| Usp40 | 0.30032 | -0.20444166 |
| Cyp11a1 | 0.00144 | -1.25927895 |
| Nav1 | 0.40959 | -0.43732487 |
| 2700099C18Rik | 0.81903 | 0.11442565 |
| 4933425L06Rik | 0.00258 | -1.07351702 |
| Pex14 | 0.0202 | -0.85816956 |
| Nepn | 0.44851 | -0.22144846 |
| Praf2 | 0.02398 | -0.52572891 |
| 1810058N15Rik | 0.00144 | -1.74019658 |
| Nfasc | 0.07596 | -0.44676208 |
| Abcb11 | 0.04624 | -0.8143145 |
| Bcl2l15 | 0.05393 | 0.75586996 |
| Cep104 | 0.22952 | 0.33900435 |
| Gm40999 | 0.00144 | -1.26560808 |
| Vgll3 | 0.26841 | -0.3307303 |
| Uri1 | 0.20951 | -0.64108828 |
| 9130023H24Rik | 0.59622 | -0.12713909 |
| Snx20 | 0.00144 | -1.21139049 |
| Zmynd12 | 0.03186 | -0.58430238 |
| Hmgcl | 0.28237 | -0.39766926 |
| Tamm41 | 0.01748 | -0.56951545 |
| Mybphl | 0.41605 | -2.08986045 |
| Toe1 | 0.24012 | 0.22978816 |
| Fosl1 | 0.12877 | -1.7153297 |
| Olfr352 | 0.0017 | -1.24950944 |
| Olfr164 | 0.02241 | -1.76426058 |
| Mcur1 | 0.83506 | 0.05424121 |
| Olfr214 | 0.00145 | -1.26219879 |
| Gcm2 | 0.09722 | -0.68472505 |
| Ppif | 0.46524 | 0.54307415 |
| Dnaja4 | 0.05893 | -0.43125908 |
| 1700094D03Rik | 0.02901 | 0.84968026 |
| 1700047K16Rik | 0.58724 | 0.10986734 |
| Myl9 | 0.00735 | -1.0197354 |
| Surf2 | 0.04634 | 0.43501077 |
| Mpp7 | 0.00144 | 1.60697982 |
| Vmn1r78 | 0.00144 | -1.31884045 |
| Cmtm8 | 0.14703 | -0.26837422 |
| 2010107G23Rik | 0.95539 | -0.03296454 |
| Bnip1 | 0.58625 | -0.18545448 |
| S1pr4 | 0.06605 | -1.21087725 |
| 1700011L22Rik | 0.00145 | -1.61510759 |
| 1700019N19Rik | 0.89325 | -0.15147971 |
| Cst8 | 0.02975 | -1.04918573 |
| Defb41 | 0.00144 | -1.31495282 |
| Cckar | 0.00144 | -1.27165604 |
| Cbln1 | 0.05298 | -1.1493145 |
| Olfr498 | 0.00144 | -1.29085005 |
| Gtf3a | 0.06827 | -0.49822032 |
| 1700047A11Rik | 0.00144 | -1.30713006 |
| Tdrd9 | 0.00145 | -1.18741414 |
| Iqcb1 | 0.00144 | 1.19734106 |
| Pop7 | 0.01651 | -0.73847973 |
| Rxra | 0.07733 | -0.59878896 |
| Syde1 | 0.00316 | -0.76524349 |
| Ppp5c | 0.12263 | -0.40512555 |
| Kifap3 | 0.01293 | 0.93374756 |
| Ankrd54 | 0.00538 | -0.71916888 |
| Ddah1 | 0.01087 | 0.66458925 |
| Ido1 | 0.14204 | 0.57548274 |
| Hey2 | 0.3325 | 0.87185027 |
| Ano3 | 0.03146 | -2.24765993 |
| Pqlc2 | 0.01633 | -0.63329019 |
| Mir99ahg | 0.00497 | 0.74091934 |
| 4930579K19Rik | 0.08299 | -0.47032765 |
| Cldn15 | 0.01013 | -0.95342981 |
| Nup160 | 0.11341 | 0.42182319 |
| Rqcd1 | 0.72405 | 0.18697098 |
| Lsm8 | 0.15824 | 0.24565076 |
| Ywhah | 0.54879 | 0.09476392 |
| 4930483K19Rik | 0.00144 | -1.30017714 |
| Slco4c1 | 0.00144 | -1.29746368 |
| Xkr5 | 0.06873 | -1.25947464 |
| Sgk3 | 0.02214 | 0.52854853 |
| Gm11959 | 0.1707 | 0.55804631 |
| Lpp | 0.18304 | 0.22246698 |
| Eif4g2 | 0.00144 | 1.56969977 |
| Sec13 | 0.94883 | -0.02867599 |
| Dll1 | 0.01589 | -0.53820213 |
| Hnrnpf | 0.08522 | 0.37268967 |
| Olfr620 | 0.00144 | -1.26560808 |
| 9530022L04Rik | 0.00149 | -1.22582153 |
| Olfr1258 | 0.00144 | -1.40286221 |
| Chordc1 | 0.00144 | 1.73388364 |
| 1700021A07Rik | 0.00144 | -1.27699683 |
| Syne1 | 0.0205 | 1.22782396 |
| 1700121L16Rik | 0.00144 | -1.30713006 |
| Gm9801 | 0.0111 | -1.02598223 |
| Ear4 | 0.00158 | -1.2446827 |
| Atp8a2 | 0.06105 | -0.67008296 |
| Olfr155 | 0.12133 | -2.86133799 |
| Snai2 | 0.01683 | 1.0042913 |
| St3gal2 | 0.0501 | -0.72115 |
| Eif2s3x | 0.02001 | 1.04527858 |
| Rerg | 0.02531 | 0.76769956 |
| Rpn2 | 0.34569 | -0.15805201 |
| Rcn1 | 0.95497 | -0.01323641 |
| B4gat1 | 0.65063 | -0.11979709 |
| Plau | 0.00144 | -1.28375735 |
| Kctd5 | 0.67979 | -0.08760645 |
| 4931403M11Rik | 0.00144 | -1.32766452 |
| Elavl3 | 0.04266 | -0.72033063 |
| Hoxd1 | 0.0202 | -1.67786946 |
| 1810010H24Rik | 0.33023 | 0.21967658 |
| 0610039K10Rik | 0.00144 | -1.28116048 |
| Siglecg | 0.80448 | 0.08486154 |
| Ntrk1 | 0.00145 | -1.04197543 |
| Gm1549 | 0.00144 | -1.30713006 |
| Abca12 | 0.0015 | -1.15488942 |
| Prorsd1 | 0.04716 | 0.70859041 |
| Clpx | 0.00144 | 1.77456214 |
| Gm10143 | 0.00144 | -1.28116048 |
| Cluap1 | 0.00326 | 0.95951503 |
| BC017158 | 0.04247 | -0.40190832 |
| Tapbp | 0.00144 | -1.02656972 |
| Olfr12 | 0.04158 | -0.76126432 |
| Fam155a | 0.01094 | -1.11366036 |
| Stxbp3 | 0.00144 | 2.22794672 |
| Cpm | 0.67239 | 0.07451084 |
| Tpcn1 | 0.18114 | -0.50481656 |
| Loxl1 | 0.00342 | -0.96245294 |
| Rtp4 | 0.44507 | -0.14998578 |
| Serac1 | 0.37017 | -0.22625771 |
| Slc29a2 | 0.02692 | -0.60802371 |
| Olfr646 | 0.05351 | -2.8691101 |
| 9530076L18 | 0.26904 | -0.61216709 |
| Olfr218 | 0.00144 | -1.48296698 |
| Depdc1b | 0.88832 | -0.14073344 |
| Afap1l1 | 0.18982 | 0.24418277 |
| Tmem38a | 0.11466 | -0.47526936 |
| Ccdc185 | 0.0028 | -1.16569658 |
| Whrn | 0.79257 | -0.09469952 |
| Itgax | 0.01054 | -0.74669186 |
| Pepd | 0.06231 | -0.48451176 |
| Ccdc23 | 0.09362 | -0.48507858 |
| Olfr160 | 0.00144 | -1.31884045 |
| Gna12 | 0.18016 | -0.44068629 |
| Ucma | 0.01281 | -1.99595737 |
| Fam114a1 | 0.02668 | 0.58054819 |
| Arg1 | 0.00144 | -1.29864962 |
| Scgb3a2 | 0.04838 | -0.60301688 |
| Tmem54 | 0.00144 | -1.29128727 |
| Adcy8 | 0.22312 | -0.52453077 |
| Vmn1r15 | 0.00144 | -1.29085005 |
| Rasl10a | 0.6419 | 0.13556909 |
| 1700013F07Rik | 0.41613 | -0.92862938 |
| Ugt2b37 | 0.00144 | -2.32987701 |
| Trim46 | 0.08792 | -0.79166045 |
| Gm16740 | 0.00144 | -1.4406427 |
| Cib2 | 0.9355 | 0.03482949 |
| Topbp1 | 0.10683 | 1.1133719 |
| Atp5d | 0.00805 | -0.94660799 |
| Maob | 0.78632 | -0.05769255 |
| Pxk | 0.00877 | 0.73516857 |
| Nek1 | 0.64438 | -0.18616132 |
| Myom1 | 0.83852 | -0.03450549 |
| Draxin | 0.00144 | -1.31884045 |
| Vmn2r89 | 0.36717 | -0.61446255 |
| Ltb | 0.00494 | -0.9685226 |
| Olfr985 | 0.01594 | -1.26528643 |
| Lgals7 | 0.00597 | -1.47542158 |
| Usp17la | 0.65165 | -0.28794062 |
| Dlg4 | 0.0015 | -1.15488942 |
| Armc12 | 0.00238 | -1.36389994 |
| Entpd4 | 0.61784 | 0.2198513 |
| Pcdhb8 | 0.00272 | -1.09759392 |
| Daam1 | 0.4113 | -0.15180777 |
| Cobl | 0.19369 | -0.37367276 |
| Cfl2 | 0.00151 | 1.09886792 |
| Mastl | 0.55814 | -0.38107763 |
| Capza3 | 0.00144 | -1.28116048 |
| Tubd1 | 0.1193 | 0.25587983 |
| Fmo2 | 0.08312 | 0.55156563 |
| Trmt10c | 0.00144 | 1.36400241 |
| Slit3 | 0.00144 | -1.22907422 |
| St3gal1 | 0.00309 | -0.77266277 |
| Gm20939 | 0.00144 | 2.46217138 |
| Gm10632 | 0.01078 | -0.94724597 |
| Kazn | 0.01141 | -0.55124388 |
| 2610015P09Rik | 0.00144 | -1.26428512 |
| AI854517 | 0.10347 | -2.78995022 |
| Tmem217 | 0.16525 | -0.76531096 |
| Ski | 0.00325 | -0.77806863 |
| B3galt5 | 0.00144 | -1.44780261 |
| Gspt2 | 0.83872 | 0.1863732 |
| P2ry10 | 0.03638 | -0.82638928 |
| Olfr1453 | 0.35372 | 0.30120515 |
| Ciz1 | 0.19702 | -0.21935863 |
| Asb14 | 0.09323 | 0.32138088 |
| Araf | 0.81093 | -0.04335053 |
| Olfr686 | 0.12717 | -1.05175941 |
| Knop1 | 0.02755 | -0.67846812 |
| Tti2 | 0.07011 | 1.64250973 |
| Rbbp8 | 0.00156 | 1.63061273 |
| Hoxd3os1 | 0.00144 | -1.25927895 |
| Map3k9 | 0.23067 | -0.73506104 |
| Atp1a1 | 0.14833 | -0.38562747 |
| Tlr4 | 0.00205 | 1.56744965 |
| Ppp6r2 | 0.01141 | -0.73187581 |
| Ppih | 0.0114 | -0.64713219 |
| Sigmar1 | 0.1855 | -0.33710484 |
| Srm | 0.03217 | -0.64069316 |
| Nefh | 0.01134 | -1.02600084 |
| Tbccd1 | 0.03139 | 0.53772996 |
| Crb2 | 0.00589 | -1.02871888 |
| Slc30a1 | 0.00144 | 1.54616241 |
| 2810405F15Rik | 0.00145 | -1.2388121 |
| Cox6b2 | 0.00147 | -0.92787693 |
| Ano2 | 0.00145 | -1.22376446 |
| Wwc2 | 0.51536 | -0.1005014 |
| Csrp2 | 0.05471 | -0.43898763 |
| Srek1ip1 | 0.00152 | 0.90031708 |
| Fem1c | 0.22561 | 0.49530737 |
| 4933440N22Rik | 0.00208 | -1.160578 |
| 4933405O20Rik | 0.00144 | -1.30017714 |
| BC048403 | 0.61267 | -0.0834593 |
| 1700125H20Rik | 0.00145 | -1.16044081 |
| Slc46a3 | 0.72672 | -0.08323256 |
| Piwil4 | 0.00202 | -1.98953082 |
| D10Wsu102e | 0.02869 | 0.93363135 |
| Rcc2 | 0.00144 | -1.11817565 |
| Trappc12 | 0.00158 | -1.2446827 |
| Spef1 | 0.01606 | -0.58220547 |
| Rims4 | 0.00145 | -1.2388121 |
| Slc22a26 | 0.52916 | -0.39387652 |
| Olfr63 | 0.31996 | -0.59646605 |
| Bcl2l1 | 0.07657 | -0.61775298 |
| Impa2 | 0.05287 | -0.94325803 |
| Fcamr | 0.06374 | -2.11182987 |
| Wdr90 | 0.1242 | -0.35373556 |
| Gpx6 | 0.00171 | -1.70052101 |
| Kel | 0.00144 | -1.36353418 |
| Gfm2 | 0.81934 | -0.06277522 |
| Gm4755 | 0.00144 | -1.30017714 |
| Pcbd2 | 0.34613 | -0.33696484 |
| Spock1 | 0.00144 | -1.28116048 |
| Bfsp2 | 0.06811 | -0.55399977 |
| Neurog1 | 0.0025 | -1.09086029 |
| Arl16 | 0.40111 | -0.26614814 |
| Fastkd1 | 0.16603 | 0.61548491 |
| Scaf4 | 0.03448 | -0.39888506 |
| Lhx8 | 0.02485 | -2.21485912 |
| 2010111I01Rik | 0.01227 | -1.59413507 |
| Prmt7 | 0.0106 | -0.76789282 |
| Cog6 | 0.05796 | 0.72175826 |
| Fam163b | 0.07774 | -2.34484129 |
| Hmces | 0.34788 | -0.1591765 |
| Lix1 | 0.00824 | -0.66889056 |
| Dctn3 | 0.00948 | -0.82375262 |
| Vit | 0.05176 | -0.64698009 |
| Clnk | 0.00144 | -1.29864962 |
| Ugt2a3 | 0.00144 | -1.2383131 |
| Recql | 0.00144 | 1.81646931 |
| Zc3h12a | 0.67501 | 0.22869905 |
| Naa35 | 0.14925 | 0.53763351 |
| Kctd6 | 0.31112 | 0.31637241 |
| Sh3tc1 | 0.00144 | -1.24963934 |
| Pcgf6 | 0.0577 | 0.6680626 |
| Prpf6 | 0.7773 | -0.04463334 |
| Hcls1 | 0.08399 | -0.35008853 |
| Birc6 | 0.0128 | 0.61310064 |
| Dph1 | 0.02993 | -0.61252813 |
| Zfyve26 | 0.00144 | -1.25118919 |
| Plcz1 | 0.00144 | -1.20579466 |
| Olfr530 | 0.05455 | -0.8301823 |
| Scamp1 | 0.00366 | -0.74526555 |
| Chmp7 | 0.00187 | -0.83594125 |
| Sf3a3 | 0.81442 | -0.04012565 |
| Ucp2 | 0.00467 | -0.81234624 |
| Tmod1 | 0.14389 | 0.55091138 |
| Kif1bp | 0.80611 | 0.11313386 |
| Palld | 0.00219 | -0.78344334 |
| Ppat | 0.01968 | 0.64197903 |
| Vmn1r72 | 0.00144 | -2.093482 |
| Triqk | 0.14199 | 0.44214923 |
| Msi1 | 0.14792 | -0.45830201 |
| Nt5dc2 | 0.72457 | 0.25884944 |
| Fcgr1 | 0.01929 | -0.55236102 |
| Anxa13 | 0.561 | -0.11779558 |
| Gadd45a | 0.00908 | 0.94891447 |
| Kcnn1 | 0.92402 | -0.03298854 |
| Lss | 0.00735 | 1.45059854 |
| Casp2 | 0.40974 | -0.18021019 |
| Tmem170 | 0.01296 | 0.82884223 |
| Orc6 | 0.10091 | 0.31027979 |
| Olfr514 | 0.00144 | -1.26560808 |
| Cacna1f | 0.52503 | -0.46941966 |
| Ahcyl1 | 0.46822 | 0.16715204 |
| Nono | 0.10391 | 0.27186359 |
| Sgsm2 | 0.00144 | -1.30017714 |
| Foxs1 | 0.2724 | -0.37105458 |
| D8Ertd82e | 0.48856 | -0.38894502 |
| Rhot2 | 0.01551 | -0.79655513 |
| Upf1 | 0.00736 | -0.69803691 |
| Nrbp2 | 0.00144 | 1.0068637 |
| Fstl5 | 0.32037 | -0.69418165 |
| Mrgpra1 | 0.00144 | -1.31495282 |
| Proc | 0.00664 | -0.89386023 |
| Baz2b | 0.00144 | 1.39057798 |
| 4930452B06Rik | 0.4929 | -0.18089058 |
| Adgrb1 | 0.11975 | -0.46712024 |
| Cep192 | 0.00795 | 1.54030758 |
| Ccdc30 | 0.53508 | -0.1882662 |
| Vmn1r62 | 0.00144 | -1.31884045 |
| Ints1 | 0.0024 | -0.95452989 |
| Slc39a13 | 0.11438 | -0.43976202 |
| D730050B12Rik | 0.29885 | -0.38766853 |
| Arf4 | 0.0183 | 0.69745712 |
| Cyc1 | 0.33892 | -0.35562492 |
| Cutc | 0.06177 | 0.66187286 |
| Cmss1 | 0.01422 | -1.17158916 |
| Aanat | 0.00192 | -1.5358119 |
| Nek7 | 0.92944 | -0.01448016 |
| Zc3h7a | 0.00145 | 1.79177665 |
| Nap1l5 | 0.00144 | 1.36274255 |
| 2610021A01Rik | 0.86548 | -0.05236446 |
| Ankrd2 | 0.08671 | 0.99326643 |
| Lrp11 | 0.19809 | 0.44974753 |
| Alkbh5 | 0.21541 | -0.32616901 |
| Nfkbia | 0.0148 | -0.73209736 |
| Ogn | 0.00144 | 1.94781467 |
| Klk1b4 | 0.03381 | -1.00597397 |
| Nedd4 | 0.00429 | -0.75357611 |
| Olfr689 | 0.00144 | -1.26560808 |
| Ppargc1b | 0.19352 | -0.2926447 |
| Atp5g3 | 0.00468 | -0.7682339 |
| Stom | 0.00454 | -0.87882408 |
| Zfp954 | 0.12656 | -0.25270446 |
| Ifitm6 | 0.23019 | 0.22134002 |
| Unc5b | 0.00144 | -1.31424436 |
| Gde1 | 0.89899 | -0.04275738 |
| 5730559C18Rik | 0.02756 | -1.40179533 |
| Rhbdd2 | 0.42495 | -0.37808631 |
| Cxcl17 | 0.00843 | -1.12617054 |
| Zgrf1 | 0.13061 | 1.11391001 |
| Ticam1 | 0.15149 | -0.48848247 |
| Rasl11b | 0.04124 | -0.61746127 |
| Gnb3 | 0.06011 | 1.25154989 |
| Ccdc88c | 0.05421 | -1.35327673 |
| Fkbp1b | 0.98375 | -0.0035657 |
| Cul7 | 0.00144 | -1.216876 |
| Rab32 | 0.55099 | -0.16185247 |
| 4930402H24Rik | 0.10443 | -0.42959649 |
| Dnajc5g | 0.09187 | -1.02681088 |
| 4732419C18Rik | 0.00144 | -1.28116048 |
| Tob1 | 0.0015 | -0.87094392 |
| Cd200 | 0.0358 | 1.0491846 |
| Mmp11 | 0.03767 | -0.53733506 |
| Mfsd7b | 0.00937 | 2.30447143 |
| Impad1 | 0.38846 | 0.19991036 |
| Il25 | 0.0795 | -0.71113304 |
| Olfr1080 | 0.00144 | -1.28116048 |
| Ell | 0.04236 | -0.40063849 |
| Snrnp200 | 0.7816 | 0.07576078 |
| Olfr618 | 0.00144 | -1.20579466 |
| 4930594C11Rik | 0.00145 | -1.2388121 |
| Thoc5 | 0.00144 | -1.26934956 |
| Acbd4 | 0.01275 | -0.58795259 |
| Yaf2 | 0.64407 | -0.08044544 |
| Cd72 | 0.06012 | -0.4544563 |
| Xpot | 0.00817 | 0.80305876 |
| Chdh | 0.05309 | -2.28731663 |
| P4htm | 0.07346 | -0.49434491 |
| Ttbk2 | 0.02408 | 1.35667833 |
| Wnk1 | 0.00953 | 1.26689147 |
| Klhl36 | 0.00209 | -0.92784594 |
| Tmco6 | 0.00785 | -0.74009533 |
| Uba6 | 0.00149 | -1.22582153 |
| Ica1 | 0.44884 | 0.15619788 |
| Haghl | 0.05089 | -0.35392924 |
| Tbl1xr1 | 0.29356 | -0.17058962 |
| Nus1 | 0.9915 | -0.00322701 |
| Uts2r | 0.00177 | -1.15941448 |
| Gpx3 | 0.00717 | -1.12857146 |
| Fis1 | 0.00291 | -1.06401915 |
| Cox14 | 0.00292 | -1.07926723 |
| 2310033P09Rik | 0.02842 | -0.50549365 |
| Adam15 | 0.00151 | -1.10687655 |
| Olfr521 | 0.15451 | -0.7674174 |
| Trappc8 | 0.00144 | 1.61351077 |
| Pecr | 0.4302 | 0.18696978 |
| Hspb9 | 0.00404 | -1.18820846 |
| Ccno | 0.5085 | -0.26199803 |
| Gm6498 | 0.03281 | -0.56316 |
| Tnc | 0.1279 | -0.73276558 |
| Thbd | 0.76595 | -0.06333587 |
| Pafah1b1 | 0.00144 | 1.55832343 |
| 2210011G09Rik | 0.0052 | 1.47094967 |
| Mug2 | 0.0096 | -0.99768241 |
| Arhgap30 | 0.40125 | -0.24997759 |
| Mobp | 0.64046 | -0.29329057 |
| Sel1l3 | 0.40376 | -0.31973713 |
| Ptprb | 0.01051 | 1.53831457 |
| Sytl2 | 0.00753 | 1.30079103 |
| Raver2 | 0.00144 | -1.60312538 |
| Calca | 0.00144 | -1.40054462 |
| Hint3 | 0.17249 | -0.35278861 |
| Ppp6r3 | 0.79458 | 0.04757634 |
| Olfr930 | 0.00691 | -2.29207674 |
| Gulo | 0.59064 | -0.55935026 |
| Ighg3 | 0.00369 | -1.16508618 |
| Znhit2 | 0.00182 | -1.08004041 |
| Nyap2 | 0.00144 | -1.30713006 |
| AI661453 | 0.00144 | -1.12664931 |
| Sval1 | 0.84052 | 0.10681108 |
| Insig1 | 0.00144 | -1.16173988 |
| Mst1 | 0.02802 | -0.85336261 |
| Selk | 0.46743 | -0.13400367 |
| Fabp7 | 0.1115 | 0.71188789 |
| Olfr654 | 0.02622 | -0.9501226 |
| Ddx3x | 0.07013 | 0.56106851 |
| Psmg3 | 0.0024 | -0.96114651 |
| Olfr693 | 0.01187 | -1.00553399 |
| Npnt | 0.04341 | -0.98185074 |
| Txndc12 | 0.08089 | 0.63214698 |
| Olfr273 | 0.38651 | -0.51052647 |
| Nek4 | 0.5726 | 0.10098761 |
| Npm2 | 0.00868 | 1.48683396 |
| Ube2e3 | 0.75506 | 0.09461752 |
| Dis3l | 0.49125 | 0.1125004 |
| Spef2 | 0.00144 | -1.31518407 |
| Spg11 | 0.12176 | 0.30399198 |
| Naip1 | 0.00149 | -1.19696847 |
| Olfr1341 | 0.00227 | -1.16712279 |
| Gtpbp3 | 0.02531 | -0.71762361 |
| Olfr1353 | 0.00144 | -1.30713006 |
| Mpzl3 | 0.82371 | -0.36615507 |
| F8 | 0.00144 | 1.5242577 |
| Limk2 | 0.57636 | -0.09238163 |
| Aff3 | 0.00144 | -1.24811894 |
| Hrh3 | 0.09404 | -0.51594378 |
| Olfr391-ps | 0.053 | -1.89999369 |
| 9430002A10Rik | 0.02149 | 1.69772972 |
| Mettl6 | 0.19838 | 0.69176483 |
| Tmtc2 | 0.00144 | 2.05250753 |
| Clip1 | 0.07005 | 0.43736538 |
| Aldoart2 | 0.00145 | -1.2388121 |
| Tbc1d15 | 0.00144 | 1.56368399 |
| Tmem45a | 0.25966 | -0.23648391 |
| Msh4 | 0.00144 | -1.27454685 |
| Otud5 | 0.20756 | -0.2192683 |
| Slc52a2 | 0.00363 | -0.87986085 |
| Rhof | 0.06413 | -1.95292288 |
| Myo18b | 0.15377 | -0.88678144 |
| BC006965 | 0.02818 | -2.12185752 |
| Cfap52 | 0.06151 | -0.73910238 |
| Jmjd7 | 0.32247 | -0.35728159 |
| Arhgap20os | 0.00144 | -1.28116048 |
| Tradd | 0.00149 | -0.95664792 |
| Slc25a29 | 0.17345 | -0.52566661 |
| Nup133 | 0.57559 | 0.09125374 |
| Usp13 | 0.02257 | -0.54410442 |
| Hpd | 0.00144 | -1.29049509 |
| Usp49 | 0.02618 | 0.62432917 |
| Fpr2 | 0.01309 | -1.0762729 |
| Psmd6 | 0.02152 | 0.88030798 |
| Haus6 | 0.26011 | 1.01128562 |
| Chrng | 0.00144 | -1.20555712 |
| Tex38 | 0.01102 | 1.21670112 |
| Nlrp4b | 0.00144 | -1.27165604 |
| Zscan2 | 0.02966 | -0.69430431 |
| Kremen2 | 0.08388 | -0.69192595 |
| Rps18 | 0.00144 | -1.04780589 |
| 1700037C18Rik | 0.00417 | -0.72096601 |
| Adck5 | 0.39578 | -0.39855012 |
| Cd209g | 0.37762 | -0.33333333 |
| 1700012B07Rik | 0.00642 | -1.65700698 |
| Qars | 0.29483 | -0.30147596 |
| Krt23 | 0.00145 | -1.2388121 |
| Cdh16 | 0.52672 | -0.16230033 |
| Serpinh1 | 0.00212 | -1.12488336 |
| Fxyd1 | 0.04724 | -0.63046072 |
| 3110001I22Rik | 0.00144 | 1.29676848 |
| 1700034O15Rik | 0.23951 | -0.83725274 |
| Olfr1212 | 0.0702 | -2.75590907 |
| Lhpp | 0.45257 | -0.21591717 |
| Wdr33 | 0.02094 | 0.893509 |
| Ncor2 | 0.05983 | -0.40437896 |
| Zfp275 | 0.02891 | 0.63086931 |
| Frzb | 0.46068 | 0.16525745 |
| Ccl2 | 0.08404 | 1.08264184 |
| Rbl1 | 0.00144 | 2.11682499 |
| Il2rb | 0.01058 | -1.43087178 |
| Ier3 | 0.0496 | -0.66104374 |
| Hist1h2ae | 0.01514 | -1.2599325 |
| Olfr434 | 0.07501 | -0.6282881 |
| LOC105243826 | 0.00144 | -1.49245118 |
| Foxd3 | 0.00215 | -1.13215694 |
| Ttll12 | 0.01875 | -0.74137646 |
| Adgre4 | 0.00705 | 0.88212102 |
| 2310036O22Rik | 0.03855 | -0.48147173 |
| Bdkrb1 | 0.16069 | 0.49121004 |
| Lzts2 | 0.02298 | -0.50931416 |
| Mylk | 0.94395 | -0.02461647 |
| Znrd1 | 0.06093 | -0.47471701 |
| 5033430I15Rik | 0.02054 | -0.67557683 |
| 4930463O16Rik | 0.34247 | -0.47267223 |
| Ccdc117 | 0.01881 | 0.67628478 |
| Dhx32 | 0.9502 | 0.01349677 |
| 4930503E14Rik | 0.00144 | -1.27165604 |
| Dnd1 | 0.37514 | -0.27177988 |
| Asphd2 | 0.00144 | -1.11202882 |
| Fem1a | 0.05375 | -0.69638182 |
| Cxx1c | 0.01547 | -0.72298409 |
| Pdcd1 | 0.35911 | -0.83378946 |
| Olfr1306 | 0.00144 | -1.40179533 |
| T | 0.01926 | -0.98294245 |
| Pigz | 0.04042 | -0.72675383 |
| Cdkn3 | 0.15389 | -0.99191259 |
| Rnf113a1 | 0.34086 | -0.20022578 |
| Olfr1394 | 0.67119 | -0.15591744 |
| Lig1 | 0.00609 | -0.62878459 |
| Olfr568 | 0.00144 | -1.28375735 |
| Rbbp6 | 0.00144 | 1.5136732 |
| 4930544G11Rik | 0.01269 | -1.14134209 |
| Cryba4 | 0.01142 | -1.13595675 |
| Slc25a2 | 0.04375 | 1.46214785 |
| Ccdc61 | 0.02617 | -0.47573389 |
| Marf1 | 0.02066 | 1.04018215 |
| Cd3d | 0.04945 | -0.83504064 |
| Hhatl | 0.10948 | -0.71795789 |
| Cd160 | 0.00145 | -1.22376446 |
| Nrbf2 | 0.02477 | 0.70105293 |
| Dolk | 0.19849 | -0.37509472 |
| Rnd3 | 0.36912 | 0.21592879 |
| Gfm1 | 0.02518 | 0.67205878 |
| Smarcb1 | 0.00877 | -0.71966061 |
| LOC102641994 | 0.11539 | 1.03232195 |
| Polr1b | 0.73211 | 0.1092811 |
| Fam217b | 0.31521 | 0.17158756 |
| 4930407I10Rik | 0.00144 | -1.4178219 |
| Aak1 | 0.0048 | 0.87070498 |
| Plcb1 | 0.01204 | 1.20224225 |
| Cd74 | 0.02741 | -0.65158127 |
| Hyal2 | 0.00144 | -1.1544389 |
| 4930473A02Rik | 0.00144 | -1.25118919 |
| Mrgprh | 0.83575 | 0.08493885 |
| Krtap22-2 | 0.00144 | -1.30017714 |
| Gstm2 | 0.09239 | -0.33546954 |
| Tsc22d4 | 0.20823 | -0.47985623 |
| 9530077C05Rik | 0.18855 | 0.44192331 |
| Cstad | 0.11643 | 0.52583507 |
| Tmed3 | 0.00203 | -0.85305337 |
| Akr1c14 | 0.00144 | 2.56623672 |
| Gm9789 | 0.0402 | -1.62671129 |
| 4930557A04Rik | 0.018 | -2.8553999 |
| 9930104L06Rik | 0.98131 | -0.00777369 |
| LOC105243269 | 0.00149 | -1.19348234 |
| Adamts18 | 0.01351 | -1.54647009 |
| Rpl36a | 0.00286 | -0.75832047 |
| Bphl | 0.73274 | 0.11099546 |
| Cpsf7 | 0.61676 | 0.1002861 |
| Msh2 | 0.15714 | -0.24989445 |
| Erbb4 | 0.0318 | -0.45824774 |
| Brat1 | 0.9903 | -0.00268131 |
| Anxa1 | 0.00575 | 0.71424905 |
| Zbtb26 | 0.04502 | 1.31518689 |
| Drd4 | 0.00144 | -1.25830842 |
| Fibin | 0.82409 | 0.06996876 |
| Icmt | 0.14403 | -0.35637878 |
| Cyp2e1 | 0.27688 | -0.98229348 |
| Olfr1510 | 0.00144 | -1.27992547 |
| Gm14296 | 0.00604 | 2.11336281 |
| Zfp874b | 0.01855 | 0.93157143 |
| Jmjd4 | 0.1992 | -0.30853351 |
| Zfp735 | 0.00144 | -1.30737044 |
| Armc1 | 0.0477 | 0.41864678 |
| Kdm5d | 0.00153 | 1.09894495 |
| Slc1a6 | 0.24041 | -0.66792611 |
| Tinag | 0.00144 | -1.30017714 |
| 4930415F15Rik | 0.99408 | -0.0030428 |
| Arhgef37 | 0.00144 | -1.29051721 |
| St14 | 0.00192 | -1.08088827 |
| Nbas | 0.06575 | -0.95081427 |
| Hspd1 | 0.0059 | 0.85661894 |
| Ccdc126 | 0.07062 | 0.41913581 |
| Hexa | 0.06517 | -0.55147693 |
| Pacsin3 | 0.01105 | -0.68130288 |
| Maats1 | 0.18002 | -0.57415534 |
| Grik1 | 0.00144 | -1.26934956 |
| Svop | 0.40105 | -0.34902275 |
| C3ar1 | 0.39434 | -0.24073732 |
| Adgra3 | 0.00932 | -0.65792071 |
| Micu3 | 0.0031 | -1.03331983 |
| Rhoc | 0.00144 | -1.24973024 |
| Eapp | 0.00789 | 0.84260487 |
| Mrps28 | 0.06777 | -0.66641301 |
| H2-M10.5 | 0.00207 | -1.72354019 |
| Snapc1 | 0.00291 | 1.09045339 |
| As3mt | 0.17299 | 0.3416417 |
| Ltb4r2 | 0.00331 | -2.75270735 |
| Mtor | 0.06686 | 0.74118606 |
| Urod | 0.00298 | -0.92341036 |
| Sass6 | 0.15427 | 0.59555198 |
| Hsdl2 | 0.03313 | 0.51348461 |
| Cactin | 0.00413 | -0.68887637 |
| Igsf23 | 0.18241 | -0.60860947 |
| Khsrp | 0.0029 | -0.9020101 |
| Lhcgr | 0.00144 | -1.28116048 |
| Krtap9-1 | 0.92924 | 0.01578612 |
| Olfr609 | 0.91927 | -0.05248638 |
| Ppapdc1b | 0.05194 | 0.43297551 |
| Lrrc42 | 0.87164 | -0.03057925 |
| Gpr158 | 0.2541 | -0.58089308 |
| Fam179b | 0.85115 | 0.0505953 |
| Sun1 | 0.35503 | -0.15496728 |
| Tspan5 | 0.02197 | -0.43988828 |
| Clasp2 | 0.28494 | -0.2247216 |
| Olfr703 | 0.38087 | -0.37434134 |
| Nasp | 0.01667 | 0.9114405 |
| Cep57 | 0.00145 | 1.51726468 |
| Zfp940 | 0.01915 | 0.75942739 |
| S100a6 | 0.00144 | -1.19181122 |
| Coro2a | 0.09722 | -0.94353485 |
| Rad9a | 0.05916 | -0.36097124 |
| Psmc3 | 0.89878 | 0.06071434 |
| LOC545466 | 0.00144 | -1.29085005 |
| Klrd1 | 0.07109 | 0.68437806 |
| Pla2g1b | 0.00144 | -1.33182817 |
| Zbtb25 | 0.03241 | -1.15703151 |
| Aldoart1 | 0.04163 | -2.04883077 |
| Ccdc108 | 0.68599 | -0.16959392 |
| Cmklr1 | 0.11046 | -0.50174927 |
| Bend6 | 0.00144 | -1.27730451 |
| Supt16 | 0.11514 | -0.44931244 |
| Ndufa9 | 0.03645 | -0.39996713 |
| Sdf2l1 | 0.18263 | -0.33927014 |
| Slc12a6 | 0.00145 | 0.93824339 |
| Tcta | 0.45603 | 0.34516301 |
| Zfp207 | 0.12268 | 0.25907063 |
| Tgfbr1 | 0.1358 | -0.40384855 |
| Zfp655 | 0.21136 | 0.22129999 |
| Kirrel3 | 0.20401 | -0.76120625 |
| 6330415B21Rik | 0.00145 | -1.24191372 |
| Slc4a7 | 0.00144 | -1.05244703 |
| E330017A01Rik | 0.00489 | -1.04391646 |
| 4930404N11Rik | 0.75483 | -0.13812169 |
| Ndufa11 | 0.07101 | -0.80556471 |
| Rell1 | 0.03104 | 0.90137927 |
| Cyp1a1 | 0.77754 | -0.41404288 |
| A230056P14Rik | 0.06533 | -2.71047292 |
| Col6a2 | 0.03444 | -0.58836709 |
| Pagr1a | 0.00382 | -0.83273057 |
| Socs1 | 0.00147 | -1.42862278 |
| Mtdh | 0.0179 | -1.2456552 |
| Olfr1245 | 0.04118 | -2.38211884 |
| Syap1 | 0.00144 | 1.86793685 |
| Cav2 | 0.00144 | 3.54986339 |
| Cts8 | 0.0088 | -1.78877407 |
| Mfsd2a | 0.00852 | -1.11379527 |
| Ccdc175 | 0.00144 | -1.31495282 |
| Dennd2d | 0.35393 | 0.46025313 |
| Tkfc | 0.05099 | -0.45610385 |
| Plcg2 | 0.17195 | -0.29639542 |
| Fam173a | 0.00719 | -0.64642721 |
| Ptgs1 | 0.16247 | -0.31180401 |
| Treml1 | 0.00144 | -1.48031818 |
| Kif3b | 0.48904 | 0.14095842 |
| Ppargc1a | 0.64843 | 0.1046921 |
| Swap70 | 0.92011 | 0.02534817 |
| Hint2 | 0.00313 | -0.88849715 |
| H2-DMb1 | 0.00207 | -1.21758427 |
| Rprm | 0.00144 | -1.97507491 |
| Vmn1r11 | 0.00144 | -1.24811894 |
| Tecr | 0.77118 | -0.16259511 |
| Myg1 | 0.05067 | -0.6498266 |
| Itfg2 | 0.84931 | -0.06451276 |
| 3830406C13Rik | 0.04089 | 0.50476698 |
| 1700049J03Rik | 0.00145 | -1.01905435 |
| Vldlr | 0.07399 | -0.3161588 |
| Gltpd2 | 0.68834 | -0.13891799 |
| Hinfp | 0.18476 | -0.56348521 |
| Gemin5 | 0.07764 | -0.37392499 |
| Plcd3 | 0.03032 | -0.52199976 |
| Ufsp1 | 0.16201 | -0.4905839 |
| Vps36 | 0.00144 | -1.14562854 |
| 1700008P02Rik | 0.00455 | -1.21759263 |
| Ctsh | 0.61986 | -0.11626444 |
| Cstf1 | 0.08388 | -0.42527021 |
| Tas1r2 | 0.00339 | -1.69203433 |
| Abcc2 | 0.00144 | -1.27165604 |
| Cenpc1 | 0.00144 | 2.14022404 |
| Zfp81 | 0.32535 | 0.41505071 |
| Defb22 | 0.10207 | -0.80127102 |
| Rab11a | 0.60259 | 0.09453918 |
| Rnf19a | 0.00158 | 1.05655225 |
| Ccdc3 | 0.11607 | -0.56637784 |
| Fam3c | 0.37689 | -0.18327985 |
| Sdpr | 0.00232 | 1.38245036 |
| Apmap | 0.47698 | 0.17312443 |
| Gm38404 | 0.00144 | -1.25118919 |
| Rit2 | 0.00144 | -1.20555712 |
| Rbmx | 0.00238 | 0.79816508 |
| Fam160b2 | 0.00256 | 0.78315519 |
| Ift22 | 0.02726 | -0.43885565 |
| Igfals | 0.94822 | 0.05162931 |
| Speer3 | 0.00145 | -1.24082201 |
| 4921539E11Rik | 0.00145 | -1.22376446 |
| Chst8 | 0.03323 | -0.80782266 |
| Gpc1 | 0.00144 | -1.32160063 |
| Olfr802 | 0.00144 | -1.28116048 |
| Akr1c20 | 0.00659 | -1.53340342 |
| 1700018B24Rik | 0.59638 | 0.52857255 |
| Olfr1350 | 0.52995 | 0.3521325 |
| Vmn2r58 | 0.00145 | -1.2388121 |
| AU040320 | 0.10737 | -0.46402433 |
| Ccl28 | 0.00144 | -1.50188002 |
| Pebp4 | 0.00144 | -1.25118919 |
| Pebp4 | 0.36554 | -0.54103254 |
| Olfr1239 | 0.00144 | -1.29085005 |
| 4930486L24Rik | 0.01173 | 1.22273339 |
| Top1 | 0.0984 | 0.48309373 |
| Szt2 | 0.1569 | -0.2431668 |
| Stra8 | 0.00144 | -1.31884045 |
| Pnpla7 | 0.74109 | 0.12222532 |
| Olfr1030 | 0.67391 | 0.33908547 |
| Phyh | 0.06415 | 0.60145622 |
| Micall2 | 0.00505 | -0.74635676 |
| Txndc15 | 0.109 | 0.45847471 |
| Dok1 | 0.00144 | -1.49638838 |
| Dcps | 0.00144 | -0.99500916 |
| Fgl1 | 0.00669 | -0.64735396 |
| Rnf141 | 0.51602 | 0.11325717 |
| Acpp | 0.04551 | -0.91232186 |
| D17H6S53E | 0.00224 | -1.38102847 |
| Mprip | 0.563 | -0.10947909 |
| Gm39749 | 0.21482 | 0.32142503 |
| 2310007B03Rik | 0.00144 | -1.30713006 |
| Olfr1411 | 0.49593 | -0.4898865 |
| 4933421I07Rik | 0.00454 | -1.67929197 |
| Hsd17b3 | 0.01471 | -1.0877005 |
| Chd5 | 0.00144 | -2.96482293 |
| Psd3 | 0.0094 | 0.97593064 |
| Chst11 | 0.4629 | 0.32377689 |
| Mrps18a | 0.20287 | -0.41626029 |
| Glp1r | 0.17422 | -1.02473254 |
| Spaca7 | 0.21671 | -0.64632352 |
| Fut4-ps1 | 0.01511 | -1.15835073 |
| Gar1 | 0.06407 | 0.80735404 |
| Atad1 | 0.00275 | 1.07657122 |
| 4930507A01Rik | 0.0093 | -1.06147486 |
| Trim30b | 0.0747 | -0.80118062 |
| 5830418P13Rik | 0.85613 | -0.11928676 |
| Tex264 | 0.00755 | -0.83098516 |
| Pcdh8 | 0.00144 | -1.28116048 |
| Star | 0.00361 | 0.97702565 |
| Sycp2 | 0.01484 | -1.11996532 |
| Ak5 | 0.23577 | -2.40538682 |
| 4631423B10Rik | 0.32332 | 0.27481825 |
| Clybl | 0.53281 | -0.17256511 |
| 2310001K24Rik | 0.11071 | -0.69606859 |
| Mansc1 | 0.17374 | -0.32296674 |
| Ppp1r15b | 0.01538 | 0.54768472 |
| Kcnma1 | 0.00376 | -1.60893423 |
| Kdm2a | 0.11912 | 0.54583264 |
| Cbln3 | 0.1678 | -3.17342529 |
| Dcun1d5 | 0.10692 | 0.61925743 |
| Immp1l | 0.39325 | -0.26622346 |
| Morn3 | 0.1755 | -0.78968988 |
| Ino80dos | 0.00564 | -1.17300636 |
| Exoc7 | 0.14496 | -0.40306522 |
| Zfp354c | 0.24381 | -0.70718733 |
| Gbe1 | 0.05193 | 0.53110453 |
| Dmgdh | 0.00144 | -1.31495282 |
| Sox17 | 0.78268 | -0.06882668 |
| 6430531B16Rik | 0.00144 | -1.28116048 |
| 4930414N06Rik | 0.00144 | -1.27165604 |
| Itpka | 0.43747 | -0.61020962 |
| Sord | 0.66582 | -0.11434808 |
| Fam83d | 0.00361 | -1.6278193 |
| Syce2 | 0.50672 | -0.14687099 |
| 8430429K09Rik | 0.00252 | 0.95177007 |
| Prkg2 | 0.65519 | 0.24965937 |
| Sec31a | 0.94591 | -0.01435996 |
| Mrpl9 | 0.18703 | 0.57259831 |
| Olfr837 | 0.00145 | -1.22376446 |
| Baz2a | 0.1249 | -0.26709708 |
| Actl7a | 0.37714 | 0.35684823 |
| Ntm | 0.21383 | 0.67120726 |
| Fam46a | 0.6671 | 0.09902434 |
| Mmp21 | 0.00144 | -1.30713006 |
| Map2k3 | 0.9277 | -0.03885262 |
| Cdh18 | 0.01456 | -1.03105883 |
| Gm7968 | 0.15638 | 0.63117725 |
| Naa25 | 0.00263 | 0.82749082 |
| Bhlhe40 | 0.57482 | -0.12561107 |
| Irgc1 | 0.00144 | -1.28116048 |
| Mlph | 0.00145 | -1.22376446 |
| Ankrd33 | 0.62448 | 0.18755592 |
| Cmbl | 0.64901 | 0.11244119 |
| Snf8 | 0.01716 | -0.65096666 |
| Tmem89 | 0.23886 | 0.7223818 |
| Ccar1 | 0.00167 | 2.17533791 |
| Emc9 | 0.00418 | -0.86472092 |
| Ndufa10 | 0.91776 | -0.06322181 |
| 2010109I03Rik | 0.00144 | -1.28116048 |
| Rnf186 | 0.00175 | -1.26015721 |
| Tmem45b | 0.02573 | -1.45178997 |
| 1700018B08Rik | 0.00144 | -1.30783085 |
| Clec4f | 0.01649 | -1.57743861 |
| Tdrd7 | 0.0866 | 0.35728187 |
| Gm568 | 0.0243 | -0.83373138 |
| Fundc1 | 0.02433 | 0.63321755 |
| Rev3l | 0.00144 | 1.27284548 |
| Pls3 | 0.00453 | 1.14761118 |
| Arl10 | 0.55004 | 0.11342987 |
| Prima1 | 0.09524 | -1.16005524 |
| Il1r1 | 0.02955 | 0.72485764 |
| Lhfpl4 | 0.00144 | -1.5016806 |
| Fibcd1 | 0.00144 | -1.33632959 |
| H2-M10.1 | 0.00144 | -1.26428512 |
| Olfr1206 | 0.00144 | -1.28898079 |
| Wsb1 | 0.00144 | 1.18731309 |
| Slc6a11 | 0.00144 | -1.26301121 |
| 1700067P10Rik | 0.02654 | -0.8216566 |
| Agtrap | 0.17519 | -0.48143703 |
| Olfr166 | 0.3031 | -0.44139278 |
| Vmn1r40 | 0.00144 | -1.26934956 |
| Klf17 | 0.01001 | -0.83819106 |
| Psg19 | 0.03876 | -1.91285507 |
| Hist1h1b | 0.0395 | -1.05370856 |
| Gosr2 | 0.44358 | -0.2614976 |
| Plekhs1 | 0.10403 | -1.57119093 |
| 1700056E22Rik | 0.03375 | -0.75138019 |
| Tnfrsf17 | 0.16323 | -0.7380701 |
| Syngr1 | 0.01116 | -0.83159029 |
| Phkb | 0.3067 | 0.35982329 |
| Uspl1 | 0.08974 | 0.31033006 |
| Igh-VJ558 | 0.06654 | -1.19300441 |
| Gfpt1 | 0.00144 | 1.54616995 |
| Dusp15 | 0.00144 | -1.28116048 |
| 2210409D07Rik | 0.00764 | -1.20075482 |
| Apitd1 | 0.01831 | -0.86696351 |
| Olfr1085 | 0.00144 | -1.26560808 |
| Ficd | 0.93794 | -0.03621451 |
| Egr4 | 0.00144 | -1.27165604 |
| Mmaa | 0.61295 | -0.08342623 |
| Esco1 | 0.00144 | 2.13071572 |
| Pnck | 0.95258 | -0.02721465 |
| Dync1li1 | 0.85207 | -0.05423182 |
| Smc6 | 0.12182 | 0.95365534 |
| Ceacam11 | 0.02605 | -0.85001139 |
| Cep83 | 0.00144 | 1.64828931 |
| Nhlrc2 | 0.08025 | -0.32528756 |
| Ccnh | 0.00462 | 1.79375848 |
| Lrguk | 0.07337 | -0.86419189 |
| Tmed7 | 0.00144 | 1.68322136 |
| S100a13 | 0.00144 | -1.00684651 |
| Hacd4 | 0.0044 | 1.2171225 |
| Rad51b | 0.50218 | 0.15428545 |
| Psmb5 | 0.00992 | -0.9340987 |
| Pole2 | 0.00302 | 1.21750116 |
| Itpr1 | 0.04839 | 0.76383224 |
| Slc25a3 | 0.39258 | 0.54057008 |
| Spata16 | 0.00144 | -1.28898079 |
| Spata16 | 0.00144 | -1.45144986 |
| Bnip3l | 0.01062 | 0.59269468 |
| Rab14 | 0.03404 | -0.44892348 |
| Fmo3 | 0.00144 | -1.31495282 |
| Acr | 0.30348 | -0.29616639 |
| 4930579G18Rik | 0.5442 | -0.1581795 |
| Atf5 | 0.00144 | -1.09581504 |
| Trpv4 | 0.0028 | -0.76249493 |
| Alg9 | 0.0841 | -0.5409019 |
| Sftpb | 0.00144 | -1.32599123 |
| Chchd10 | 0.0039 | -0.96266813 |
| Habp4 | 0.87968 | 0.06081288 |
| Hoxd3os1 | 0.33618 | 0.18018946 |
| Rnf112 | 0.0029 | -1.20702967 |
| Tmem64 | 0.05033 | 0.43626326 |
| Aox3 | 0.00964 | -1.68047009 |
| Mon1a | 0.00144 | -1.03528224 |
| Car10 | 0.0105 | -0.9810057 |
| Rasip1 | 0.00347 | -0.79985124 |
| Vmn1r202 | 0.00144 | -1.25118919 |
| 3110039I08Rik | 0.00144 | 2.20413725 |
| Kptn | 0.00266 | -1.21776218 |
| 2210016H18Rik | 0.00144 | 1.27684314 |
| A1cf | 0.01051 | -1.1474527 |
| Cntnap4 | 0.00145 | -1.2388121 |
| Olfr881 | 0.01971 | -1.70065414 |
| Idh3a | 0.00281 | 1.04558692 |
| Csad | 0.01116 | 0.61683996 |
| Mrfap1 | 0.84847 | 0.03787009 |
| Aagab | 0.49489 | -0.13064357 |
| Tmem131 | 0.39321 | 0.31440448 |
| Il34 | 0.00144 | -1.09636527 |
| Slc7a10 | 0.00144 | -1.48661212 |
| Fam208a | 0.0402 | 0.81968508 |
| Acsl4 | 0.9409 | -0.01691401 |
| 1700017B05Rik | 0.00144 | -1.31495282 |
| Six1 | 0.50029 | -0.29641168 |
| Enox2 | 0.02421 | 0.53140026 |
| Reg3a | 0.00145 | -1.25646236 |
| Wdr63 | 0.00144 | -1.29085005 |
| Arhgap42 | 0.59467 | 0.26967863 |
| Rgsl1 | 0.16385 | -0.84891609 |
| Wbscr25 | 0.01369 | -1.28551253 |
| Ddr2 | 0.04168 | 0.87453954 |
| Il11 | 0.94184 | -0.01322338 |
| Gkn3 | 0.03141 | -0.82958999 |
| Abtb1 | 0.02439 | -0.64303484 |
| Olfm3 | 0.02016 | -0.98313485 |
| 1700061G19Rik | 0.25427 | -0.85251799 |
| Gdap10 | 0.0107 | 1.12344032 |
| Frg1 | 0.00396 | -1.21885932 |
| 2010012O05Rik | 0.29146 | -0.17903822 |
| Cdc42ep3 | 0.19609 | 0.2515988 |
| Slc25a11 | 0.08987 | -0.60045849 |
| Them5 | 0.00144 | -1.26560808 |
| Dxo | 0.00397 | -0.73338614 |
| Dxo | 0.006 | -0.62510083 |
| Lrfn3 | 0.00322 | -0.89217401 |
| Topors | 0.03724 | 0.58111233 |
| Tbc1d10c | 0.09513 | -0.56730074 |
| Litaf | 0.17894 | -0.25263973 |
| Olfr356 | 0.00144 | -1.31978601 |
| Faim | 0.31072 | 0.17506228 |
| Zfy2 | 0.00144 | -1.25118919 |
| 4930517O19Rik | 0.00144 | -1.64106147 |
| Sgpp2 | 0.00144 | -1.22925777 |
| 9030625N01Rik | 0.23351 | -0.32367364 |
| Ugt3a2 | 0.117 | -0.80836422 |
| Thbs3 | 0.02907 | -0.46715165 |
| L3mbtl1 | 0.00144 | -1.26560808 |
| Nr1i2 | 0.0224 | -1.8110964 |
| Tmem71 | 0.06286 | 0.50198138 |
| Mup4 | 0.12608 | -1.45715661 |
| Olfr225 | 0.00144 | -1.28116048 |
| Mfn2 | 0.03312 | -0.53781718 |
| Dopey1 | 0.78907 | 0.07316901 |
| Peli3 | 0.08074 | -0.42266029 |
| Cyp8b1 | 0.00921 | -1.67747383 |
| Kctd16 | 0.00195 | -1.19169742 |
| Zc2hc1a | 0.00784 | 0.83899187 |
| Abcf1 | 0.00802 | -0.58765049 |
| Limd1 | 0.41175 | -0.14955829 |
| Snrk | 0.00835 | 0.68005337 |
| Adpgk | 0.16121 | -0.22998768 |
| Shcbp1l | 0.8015 | -0.24986772 |
| Hspa9 | 0.02618 | 0.66841995 |
| Olfr1179 | 0.0049 | -1.08617297 |
| Clca3a2 | 0.00144 | 1.99417629 |
| Apoo | 0.11382 | 0.55228375 |
| A230072E10Rik | 0.4565 | -0.48290343 |
| Rbm18 | 0.47286 | -0.12106545 |
| Adm | 0.16998 | 0.23552323 |
| Tmem57 | 0.06332 | 0.39162153 |
| Ly6a | 0.01868 | -0.61755534 |
| Abhd3 | 0.00879 | 1.09220482 |
| Ric8 | 0.08725 | -0.35817499 |
| Vnn3 | 0.00144 | -1.26428512 |
| Ldb3 | 0.51731 | -0.29057825 |
| Nr0b2 | 0.46248 | 0.26534943 |
| Mpp6 | 0.01344 | -0.76150316 |
| Ppm1k | 0.00391 | 1.65655681 |
| Arhgef10 | 0.46337 | -0.15901574 |
| Dlat | 0.01225 | 0.70662897 |
| Ppig | 0.451 | 0.21747288 |
| Lman1 | 0.02877 | -0.9205913 |
| Olfr1395 | 0.19609 | -0.84862089 |
| Epn2 | 0.00402 | -0.68038682 |
| Lag3 | 0.26782 | -0.69965283 |
| Ribc2 | 0.00144 | -1.28116048 |
| Dmp1 | 0.00144 | -1.29085005 |
| Crym | 0.85074 | -0.12613248 |
| 2900011O08Rik | 0.00149 | -1.22582153 |
| 4933427E11Rik | 0.00145 | -1.2388121 |
| Fam69b | 0.76803 | -0.14093154 |
| Ppp1r3f | 0.02698 | -0.63782379 |
| Ralgapa2 | 0.22772 | 0.25025713 |
| Tra2b | 0.00826 | 0.84040737 |
| Gpr160 | 0.3381 | -0.35227615 |
| Galnt5 | 0.89094 | 0.03975846 |
| Rab36 | 0.00144 | -1.29878664 |
| Pigf | 0.02041 | 0.45885065 |
| Lipc | 0.00144 | -1.29085005 |
| Olfr1167 | 0.00144 | -1.27165604 |
| Vmn1r193 | 0.00758 | -1.90665306 |
| Acrv1 | 0.0903 | -2.47976183 |
| Pank1 | 0.51181 | 0.17714091 |
| Prss27 | 0.04517 | -1.97403711 |
| Zfp30 | 0.0741 | 0.31835407 |
| Itpripl1 | 0.01859 | -0.593529 |
| Rnf24 | 0.02331 | 0.6053099 |
| Galnt9 | 0.03773 | -1.99369153 |
| Dusp8 | 0.05947 | -0.68411457 |
| Muc13 | 0.00145 | -1.22376446 |
| 1700018F24Rik | 0.0891 | -2.45349378 |
| Tmed9 | 0.21468 | -0.26286728 |
| Nans | 0.13625 | -0.39993615 |
| Gm35168 | 0.00144 | -1.27165604 |
| Plin5 | 0.1647 | -0.63616976 |
| 0610009L18Rik | 0.01127 | -0.98118689 |
| Cpb2 | 0.10571 | 0.42988707 |
| Npc1l1 | 0.05261 | 0.53188913 |
| Ceacam3 | 0.00157 | -1.19247859 |
| Opalin | 0.00144 | -1.26560808 |
| Cxxc1 | 0.63903 | -0.1034002 |
| Phf11a | 0.02365 | 0.80684561 |
| Srsf2 | 0.02161 | 0.60240707 |
| Kdelc1 | 0.4506 | 0.28279046 |
| Rbm3 | 0.0431 | 0.47088617 |
| Nek11 | 0.00145 | -1.2388121 |
| Tub | 0.00144 | -1.24811894 |
| Itgb2 | 0.43886 | -0.12971696 |
| Pex11b | 0.26841 | -0.33240212 |
| Irgm1 | 0.06568 | 0.51194628 |
| Ddx19b | 0.3591 | 0.16595662 |
| Ptcd2 | 0.77701 | 0.09721013 |
| Ccdc166 | 0.00218 | -1.17286405 |
| Olfr738 | 0.00409 | -1.16668909 |
| Tmem8c | 0.76731 | -0.17013629 |
| Slco1c1 | 0.07174 | -2.3367719 |
| Olfr1507 | 0.09386 | -1.62981933 |
| Rnf138 | 0.09929 | -0.38335807 |
| Fbxw26 | 0.00144 | -1.34462115 |
| Tex33 | 0.64439 | -0.45556744 |
| LOC102633266 | 0.00144 | -1.24613586 |
| Tyrobp | 0.02147 | -0.54711461 |
| Birc7 | 0.00144 | -1.28116048 |
| Dynlrb1 | 0.84998 | -0.0703261 |
| Mlx | 0.76486 | -0.1044398 |
| T2 | 0.15052 | 0.5507326 |
| Fkbp15 | 0.1232 | 0.87947108 |
| Spats2 | 0.00145 | -1.14964547 |
| Fbxo33 | 0.01022 | 0.69520789 |
| Olfr461 | 0.00144 | -1.29085005 |
| Mill1 | 0.02008 | -1.79260595 |
| Foxi2 | 0.00144 | -1.28116048 |
| 2810021J22Rik | 0.00248 | 1.32194754 |
| Ogt | 0.00144 | 2.01042119 |
| Dlx5 | 0.10229 | -0.76638697 |
| Dlc1 | 0.03763 | 1.16811173 |
| Sdhd | 0.88956 | 0.06032894 |
| Cntnap2 | 0.00243 | -1.33333333 |
| Pcdh7 | 0.00144 | 1.59280296 |
| Nphp1 | 0.01562 | 0.57670254 |
| Rgs1 | 0.50685 | -0.6919771 |
| Amdhd2 | 0.00144 | -0.99598084 |
| Elp3 | 0.02368 | -0.49587025 |
| St8sia2 | 0.06825 | -1.63195396 |
| Glg1 | 0.01713 | -0.48796453 |
| Polr1d | 0.73634 | -0.10807633 |
| Arhgef4 | 0.49682 | -0.46349823 |
| Ptpn14 | 0.45372 | -0.21204294 |
| Prl6a1 | 0.00144 | -1.28116048 |
| Csgalnact1 | 0.10144 | 0.522441 |
| Cpne4 | 0.01147 | -1.06301127 |
| Hoxd4 | 0.52705 | 0.41630147 |
| Slc26a5 | 0.00144 | -1.20555712 |
| Recql4 | 0.04115 | -0.49532651 |
| Gstm5 | 0.7495 | -0.10606087 |
| Gpr27 | 0.64731 | 0.14611372 |
| Sar1b | 0.07329 | 0.94578541 |
| Stim2 | 0.3752 | -0.13704074 |
| 4930425F17Rik | 0.00144 | -1.40078216 |
| Aspa | 0.3362 | -0.40279805 |
| Arl6ip5 | 0.48401 | -0.20661326 |
| Fkrp | 0.84062 | 0.1066825 |
| Dock9 | 0.01166 | 0.90585199 |
| Mettl9 | 0.01032 | 1.03720417 |
| Ccdc32 | 0.44737 | -0.31990181 |
| Sdr16c6 | 0.00144 | -1.26911831 |
| Gm10627 | 0.01786 | -0.83053208 |
| Def8 | 0.16641 | -0.49521129 |
| Pinlyp | 0.03201 | -1.50434376 |
| Mpp6 | 0.459 | 0.20427358 |
| Tsks | 0.48026 | -0.32032021 |
| Adcyap1r1 | 0.01952 | -0.6532852 |
| Angptl6 | 0.00284 | -1.00130167 |
| Gpatch3 | 0.00725 | -0.82643719 |
| Nfkbib | 0.24481 | -0.27696667 |
| Tmem70 | 0.05027 | 0.61291154 |
| Rnf114 | 0.10777 | -0.42448538 |
| Csrp2bp | 0.04754 | -0.35958227 |
| Fbrs | 0.98087 | 0.00652886 |
| Lpl | 0.00144 | 1.74342705 |
| Slc39a6 | 0.02012 | 0.72285456 |
| Fundc2 | 0.19679 | -0.27821594 |
| Klhl1 | 0.59812 | -0.39134313 |
| Ttc26 | 0.00144 | -1.55701652 |
| Dusp26 | 0.00929 | -1.30588311 |
| Cacnb3 | 0.00144 | -1.1486811 |
| Ccer1 | 0.00144 | -1.31884045 |
| Pifo | 0.00144 | -1.2383131 |
| 4933427G17Rik | 0.22464 | -0.60372005 |
| Tmod4 | 0.10565 | 0.8455318 |
| Klrg2 | 0.50928 | 0.19775016 |
| Smo | 0.05555 | -0.37978227 |
| Tpsg1 | 0.01893 | -1.86781639 |
| 4930415O20Rik | 0.64059 | -0.13511323 |
| Phf8 | 0.26673 | -0.20269089 |
| Scrg1 | 0.41328 | -0.36347569 |
| LOC100043315 | 0.00144 | -1.31518407 |
| Rab4b | 0.15153 | -0.40028562 |
| 4931429I11Rik | 0.02173 | -1.02207431 |
| BC037034 | 0.83663 | -0.04530739 |
| Zdhhc3 | 0.88233 | -0.04587548 |
| Hey1 | 0.1686 | -0.34726938 |
| Igsf6 | 0.00144 | 2.38180041 |
| Hus1b | 0.0198 | -1.22229819 |
| Gal | 0.03406 | -0.43943964 |
| Brdt | 0.58445 | 0.32220163 |
| Tmem176a | 0.01116 | -0.66822758 |
| Nop2 | 0.04119 | -0.47428707 |
| Usp29 | 0.00144 | -1.28898079 |
| Psma5 | 0.03756 | -0.45427749 |
| Retnla | 0.82009 | 0.08410687 |
| Tacstd2 | 0.25205 | -0.57676729 |
| Tnfsf13b | 0.00144 | 1.88805921 |
| Adam25 | 0.52232 | -0.34246256 |
| Mmd2 | 0.00144 | -1.28116048 |
| 1700034I23Rik | 0.14651 | 0.38144339 |
| Uros | 0.74877 | -0.11959301 |
| Hoxb2 | 0.12125 | 0.87861047 |
| Dnajc22 | 0.37093 | -0.62482304 |
| Olfr44 | 0.11382 | -2.9298373 |
| Tspyl4 | 0.03825 | 0.82255085 |
| Marcksl1 | 0.00528 | -0.68267147 |
| Lppr1 | 0.00144 | -1.29085005 |
| Lhx2 | 0.16498 | -0.79168531 |
| Cpox | 0.02333 | 0.62429901 |
| Krt76 | 0.00144 | -1.30017714 |
| C230029F24Rik | 0.00144 | -1.28898079 |
| Ddrgk1 | 0.02957 | -0.52167449 |
| Adipor2 | 0.64449 | -0.2112907 |
| Slc10a7 | 0.01222 | 1.08757478 |
| Prpf40b | 0.35756 | -0.14952795 |
| Ip6k2 | 0.71862 | -0.06353357 |
| Olfr710 | 0.00144 | -1.29746368 |
| Klrg1 | 0.00144 | -1.25118919 |
| Ankmy2 | 0.13925 | 0.37886157 |
| Opa3 | 0.20262 | -0.52977653 |
| Sp1 | 0.11703 | -0.33357573 |
| Abcg5 | 0.00144 | -1.28116048 |
| S100a8 | 0.05203 | 1.53798941 |
| Ppp3cb | 0.32714 | 0.27817856 |
| Adcy10 | 0.11663 | -2.93096767 |
| Kpna1 | 0.02094 | 0.91991117 |
| Pon3 | 0.00472 | -0.674046 |
| Lmbr1 | 0.05871 | 0.52314017 |
| Pla2g2a | 0.00589 | -1.11030563 |
| Spz1 | 0.00144 | -1.28898079 |
| Atf7 | 0.0482 | 0.5903224 |
| Muc6 | 0.00684 | -1.13117744 |
| Ryr1 | 0.08698 | -0.53506795 |
| Atp2b2 | 0.00145 | -1.20192485 |
| Mbd2 | 0.49565 | 0.23118266 |
| Lpin2 | 0.00435 | 0.82010604 |
| Olfr853 | 0.00144 | -1.27165604 |
| Ppp6c | 0.05593 | -0.38825338 |
| Tspan13 | 0.50325 | -0.14437948 |
| Zfp768 | 0.00144 | -1.15108746 |
| Ctsz | 0.00326 | -0.73588683 |
| Tiam2 | 0.92208 | 0.04261557 |
| Sf3b5 | 0.00144 | -1.27699683 |
| Padi4 | 0.77709 | -0.09944326 |
| Omg | 0.26683 | -1.14807284 |
| Fhdc1 | 0.69551 | -0.08297788 |
| Coq10b | 0.01215 | 0.56517164 |
| Bcl2l13 | 0.51422 | 0.28866462 |
| Mmp3 | 0.4431 | -0.28669728 |
| Mfge8 | 0.00144 | -1.37535868 |
| 2210011C24Rik | 0.46261 | -0.20288448 |
| Smarcad1 | 0.00327 | 0.7559791 |
| Hdac9 | 0.48578 | 0.47325688 |
| Cyp1b1 | 0.00327 | 1.69339425 |
| Mrpl43 | 0.04878 | -0.76925038 |
| Col7a1 | 0.26296 | 0.28139915 |
| Med31 | 0.38476 | -0.17842062 |
| 2810013P06Rik | 0.03454 | 0.41045803 |
| Riok2 | 0.00286 | 0.77808607 |
| Cav3 | 0.01371 | -0.84191215 |
| Olfr513 | 0.91258 | 0.06604487 |
| Plac1 | 0.00749 | -1.05513651 |
| Blcap | 0.00478 | -0.69502713 |
| Serf1 | 0.79935 | -0.07357756 |
| Atxn2 | 0.04173 | -0.38005153 |
| Ptgs2 | 0.00591 | 1.99422558 |
| Kif4 | 0.00516 | 1.00794891 |
| Ercc6 | 0.00145 | -1.22376446 |
| Hdc | 0.08015 | -0.53379437 |
| Jdp2 | 0.32707 | -0.17995614 |
| H2-Ob | 0.06825 | -0.94332221 |
| Foxred1 | 0.3331 | 0.39426695 |
| Bax | 0.00221 | -0.9903789 |
| Birc2 | 0.00144 | 1.24080008 |
| Ahrr | 0.61512 | 0.15172072 |
| Vmn1r13 | 0.00144 | -1.28116048 |
| Dlk2 | 0.02753 | -0.60803799 |
| Nxph3 | 0.00144 | -1.16029081 |
| Chchd4 | 0.78881 | 0.1295982 |
| Zfp92 | 0.00149 | -1.32850014 |
| Traip | 0.05719 | -1.00840415 |
| Pcp4 | 0.64581 | -0.24652355 |
| Psca | 0.01728 | -0.85661854 |
| Fam49a | 0.05187 | 1.09319886 |
| Olfr27 | 0.00144 | -1.29746368 |
| Mki67 | 0.00421 | 2.12764611 |
| Evi5l | 0.63397 | -0.10509818 |
| Il17rd | 0.06845 | 0.46152995 |
| A830012C17Rik | 0.01552 | -1.59761845 |
| Mrps9 | 0.22783 | 0.25028297 |
| Ctsl | 0.05579 | 0.4064709 |
| Atf6b | 0.00767 | -0.67140376 |
| Rffl | 0.05947 | -0.42847155 |
| Ces1g | 0.0268 | -1.83080902 |
| Olfr494 | 0.00144 | -1.28898079 |
| Cntn6 | 0.12396 | -1.2122742 |
| Zfp831 | 0.00149 | -1.2703832 |
| Slc51a | 0.05084 | -0.57000491 |
| 9430076C15Rik | 0.13742 | -2.90298441 |
| Sorbs1 | 0.02342 | -0.52617633 |
| Chit1 | 0.36811 | 0.36039494 |
| BC010981 | 0.03054 | 0.53777636 |
| Isx | 0.00233 | -1.53651605 |
| Cyr61 | 0.83708 | -0.08989741 |
| Fam216a | 0.45135 | -0.11675748 |
| Gpc4 | 0.16339 | -0.2540153 |
| 1110007C09Rik | 0.39206 | -0.32217982 |
| Olfr338 | 0.16516 | -1.02772844 |
| Alox15 | 0.00145 | -1.26219879 |
| Cope | 0.07413 | -0.63646457 |
| Stx12 | 0.63277 | 0.09229385 |
| Tshb | 0.00144 | -1.27992547 |
| 4930488N24Rik | 0.45621 | -0.19382701 |
| Mmgt1 | 0.01116 | 1.55255789 |
| Vmn1r16 | 0.00144 | -1.28116048 |
| Irgq | 0.00144 | -1.31817693 |
| Magoh | 0.68593 | 0.09509699 |
| Zfp317 | 0.57157 | 0.18052876 |
| Sh2b3 | 0.13362 | -0.35420881 |
| Adgre1 | 0.36798 | -0.19539085 |
| Reep5 | 0.02398 | 1.06660354 |
| l7Rn6 | 0.22678 | 0.56141866 |
| Olfr195 | 0.1134 | -1.13351264 |
| Csn1s2b | 0.01001 | -0.87563308 |
| Rnf123 | 0.64178 | -0.24553636 |
| Olfr1214 | 0.00144 | -1.28116048 |
| Tmem206 | 0.74287 | -0.05892446 |
| Nkapl | 0.07289 | 0.74243041 |
| Tgds | 0.03027 | 0.63664155 |
| Uevld | 0.00145 | -1.2388121 |
| Ctps | 0.24813 | -0.18148244 |
| Slc25a13 | 0.00583 | 1.08511083 |
| Fbxw2 | 0.61041 | -0.07957216 |
| Cacna1d | 0.92943 | -0.04246704 |
| Psd | 0.00144 | -1.10356032 |
| Stard8 | 0.44876 | 0.13784427 |
| Pter | 0.00707 | 0.86314756 |
| Plscr2 | 0.00422 | 1.46425251 |
| Epg5 | 0.00144 | -1.33632959 |
| Hsd17b7 | 0.07262 | -0.58048723 |
| Spata2l | 0.10348 | -0.43724804 |
| Ubr2 | 0.0081 | -1.07522545 |
| Pyurf | 0.05072 | 0.54706077 |
| Rabgap1l | 0.03506 | -0.9858148 |
| Tas2r140 | 0.18846 | -0.45419003 |
| Dhx38 | 0.15298 | -0.32389193 |
| Mettl5 | 0.00285 | 1.07366507 |
| Zfp276 | 0.17574 | -0.22943499 |
| Asxl2 | 0.00652 | 0.73942402 |
| Prpf39 | 0.00144 | 2.66902099 |
| 4430402I18Rik | 0.08336 | -2.59503092 |
| Igfn1 | 0.00149 | -1.25408 |
| Otx2 | 0.00145 | -1.26219879 |
| Stat1 | 0.33509 | -0.26027311 |
| Zfp763 | 0.01109 | 1.02671083 |
| Epas1 | 0.06366 | 0.35221534 |
| H1fnt | 0.30215 | -0.30582931 |
| Tifa | 0.15415 | 0.33988196 |
| Fgf18 | 0.36957 | -0.21007837 |
| Tceal1 | 0.01047 | 0.73783515 |
| Lect2 | 0.00159 | -1.02182267 |
| Dusp28 | 0.2096 | -0.51919773 |
| Kdm5a | 0.17057 | 0.56130651 |
| Trdn | 0.00144 | 1.73311911 |
| Rev1 | 0.04315 | 0.74389132 |
| 2310050C09Rik | 0.50196 | -0.48019086 |
| Lrrc6 | 0.26996 | -0.71044259 |
| Wdr24 | 0.0171 | -0.64938734 |
| Brf1 | 0.54149 | 0.10966839 |
| Gm20559 | 0.32723 | -0.15216728 |
| Dgcr14 | 0.47761 | -0.15164503 |
| Mroh1 | 0.00336 | -0.78555352 |
| 4930417O22Rik | 0.27445 | -0.67951775 |
| Suox | 0.25132 | -0.41077304 |
| Sds | 0.09342 | -0.76971149 |
| Herc3 | 0.00719 | 1.118607 |
| Acer2 | 0.02177 | -0.61418696 |
| Ptger2 | 0.37806 | -0.610025 |
| Gsg1l | 0.2587 | 0.40845067 |
| Abcb4 | 0.23263 | 0.23820738 |
| Speer4f | 0.00328 | -1.72725015 |
| Pycr2 | 0.00327 | -0.96051331 |
| Dtx3 | 0.00533 | 0.87391936 |
| Stxbp5 | 0.81771 | -0.07776279 |
| Foxf2 | 0.00144 | -1.28116048 |
| Fam212a | 0.0049 | -0.95588569 |
| Ccdc80 | 0.53701 | -0.12420197 |
| Cse1l | 0.00194 | 1.14337455 |
| Hormad2 | 0.05346 | -0.86696801 |
| Yars | 0.91929 | 0.03202174 |
| Cd274 | 0.75267 | 0.15583634 |
| Tdp1 | 0.11581 | -0.26390291 |
| Myoz2 | 0.57482 | 0.16189926 |
| Arhgap25 | 0.19485 | -0.23680627 |
| Miip | 0.08499 | -0.37680035 |
| Nipsnap3a | 0.00186 | -1.2317955 |
| Poglut1 | 0.01792 | 0.53210595 |
| Olfr180 | 0.00144 | -1.22925777 |
| Pcyox1l | 0.04819 | -0.75815054 |
| Zdhhc4 | 0.406 | -0.31171669 |
| Tfap2b | 0.00145 | -1.26219879 |
| Zfp618 | 0.16744 | -1.20215176 |
| Trpv3 | 0.00144 | -1.29746368 |
| Skint7 | 0.13562 | -0.68230032 |
| Trim60 | 0.07847 | -1.13352232 |
| Bbc3 | 0.00184 | -0.85161419 |
| Ezh2 | 0.00767 | 1.78927874 |
| Lamb2 | 0.05389 | -0.51785554 |
| Rab42 | 0.19721 | 0.48431678 |
| Olfr1109 | 0.00828 | -2.16818754 |
| Olfr705 | 0.15 | -0.66183348 |
| Asb9 | 0.05537 | -0.5670685 |
| Clmn | 0.79548 | 0.28599367 |
| Chrm3 | 0.00158 | -1.15468698 |
| Casp8 | 0.00144 | 1.25937625 |
| Adgrg3 | 0.71989 | -0.07329034 |
| Rnf144a | 0.00876 | 0.79987816 |
| Smim10l2a | 0.05149 | -0.74782014 |
| Aamp | 0.6452 | -0.21617587 |
| Ttll6 | 0.02979 | -2.00139593 |
| Gm9767 | 0.01463 | -1.31812226 |
| Adck1 | 0.24103 | -0.40191026 |
| 3200001D21Rik | 0.24715 | -0.7933679 |
| Cfap46 | 0.00144 | -1.28116048 |
| Ptprcap | 0.01518 | -0.60175238 |
| Alox5 | 0.03891 | -0.70887718 |
| Cfap43 | 0.00144 | -1.56519132 |
| Hn1 | 0.15451 | -0.44348051 |
| Olfr1208 | 0.00145 | -1.22376446 |
| Ralgapb | 0.55428 | -0.22398506 |
| Fbxl15 | 0.00696 | -0.77351781 |
| Vwa5b2 | 0.00144 | -1.31495282 |
| Tppp3 | 0.00406 | -0.96630728 |
| Rps27l | 0.05946 | -0.5596465 |
| Rps27l | 0.03226 | -0.66311537 |
| 4930583I09Rik | 0.04191 | -1.22098834 |
| Acta1 | 0.16846 | -0.97497831 |
| Sesn3 | 0.00209 | -0.87912139 |
| Mlxip | 0.59578 | 0.16840774 |
| Nop14 | 0.00201 | 1.43982063 |
| Rec8 | 0.00571 | -0.74383979 |
| Clec7a | 0.07008 | 0.79590672 |
| Kif2b | 0.00144 | -1.3230041 |
| Ebf3 | 0.00757 | 0.99144521 |
| Tpp2 | 0.01478 | 0.8242252 |
| Pbx2 | 0.00163 | -0.84558478 |
| Olfr15 | 0.02618 | -1.53469044 |
| Myl7 | 0.23668 | -2.91989258 |
| Mt2 | 0.00682 | 1.52841729 |
| Tram2 | 0.02554 | -0.69951843 |
| Polr2i | 0.00144 | -1.11109674 |
| Cenpf | 0.41184 | 0.29689412 |
| 1700019M22Rik | 0.04908 | -0.90497976 |
| 1700025F22Rik | 0.00144 | -1.39762478 |
| Samd8 | 0.81065 | -0.0815822 |
| Slamf9 | 0.00145 | -1.17895281 |
| Dazl | 0.14414 | -2.81358235 |
| Ccr2 | 0.55691 | -0.34995113 |
| Mapk4 | 0.56763 | -0.26583269 |
| Ddit4 | 0.15043 | -0.28144963 |
| Pcolce2 | 0.00833 | -0.59421973 |
| Muc4 | 0.08553 | -1.26078043 |
| Rftn2 | 0.88365 | -0.03074034 |
| Scnn1g | 0.00145 | -1.2388121 |
| Tex19.1 | 0.00144 | -1.80489508 |
| ND4 | 0.72 | -0.05989948 |
| Ugt2b1 | 0.00243 | -1.12283767 |
| Optn | 0.01695 | 0.71286011 |
| Ufsp2 | 0.10051 | 0.30406446 |
| Klk1 | 0.01808 | -1.66716321 |
| Ppp3cc | 0.57761 | -0.1034658 |
| Ppp3cc | 0.08527 | 0.44673363 |
| Abcb1b | 0.2084 | 0.30981104 |
| 4931402G19Rik | 0.00144 | -1.28116048 |
| H2afx | 0.00916 | -0.99702507 |
| Olfr1298 | 0.00144 | -1.30017714 |
| Alg12 | 0.85028 | -0.04655904 |
| Gdf2 | 0.00144 | -1.3570277 |
| 4930591A17Rik | 0.38599 | -1.14032054 |
| Aqp3 | 0.06145 | -2.33944222 |
| 1700011A15Rik | 0.05545 | -0.9451231 |
| Rmnd5b | 0.33761 | -0.26004403 |
| Dpys | 0.23739 | -0.67937838 |
| AI837181 | 0.00879 | -0.8051416 |
| AB041806 | 0.04302 | -0.92844287 |
| Atp2a1 | 0.04337 | 0.78937726 |
| Rgs17 | 0.72966 | 0.06896003 |
| Rsbn1 | 0.00145 | 1.22177671 |
| Zfp618 | 0.31281 | 0.24431226 |
| Rab3gap2 | 0.31843 | -0.15955559 |
| 2610507B11Rik | 0.00399 | -0.74273851 |
| 6330403L08Rik | 0.09734 | -0.43143203 |
| Oraov1 | 0.14804 | 0.26632709 |
| Plin1 | 0.02885 | -1.47249332 |
| Nbl1 | 0.00572 | -0.68550366 |
| Acer2 | 0.00422 | 0.99629371 |
| Smc1b | 0.00208 | -1.15498993 |
| Lamb1 | 0.01078 | 0.54623187 |
| Gm20172 | 0.06978 | -2.40392363 |
| Hapln3 | 0.01071 | -0.72704007 |
| Cadm3 | 0.01097 | -0.56841843 |
| Sbf1 | 0.05689 | 1.07027603 |
| Tktl1 | 0.00144 | -1.25927895 |
| Nkain1 | 0.07267 | -0.77046581 |
| Snapc5 | 0.00542 | 0.81547098 |
| Rtbdn | 0.17153 | 0.5907865 |
| Meikin | 0.00144 | -1.33632959 |
| Qrsl1 | 0.17936 | 0.58570348 |
| Skap2 | 0.27061 | 0.21710369 |
| Nell2 | 0.00144 | -1.31495282 |
| Copb2 | 0.27108 | 0.19263289 |
| Traf3ip3 | 0.15546 | -0.59098163 |
| Slc10a2 | 0.00144 | -1.26934956 |
| Tssc1 | 0.06794 | -1.04792753 |
| Zcchc6 | 0.39237 | 0.24185701 |
| Aox4 | 0.00202 | -1.38883422 |
| Gm4922 | 0.0731 | -0.8956869 |
| Ptcra | 0.00144 | -1.30713006 |
| 4833431D13Rik | 0.86756 | 0.08092324 |
| Macc1 | 0.14083 | -0.70598628 |
| Prkag2os1 | 0.00144 | -1.31495282 |
| 4931431F19Rik | 0.00332 | -0.74955609 |
| Hsd17b1 | 0.20624 | -1.12143435 |
| Olfr1163 | 0.00144 | -1.28116048 |
| Galk1 | 0.00144 | -1.11970949 |
| Zfp574 | 0.00938 | -0.67245959 |
| Olfr697 | 0.1433 | -1.14845401 |
| Tmem237 | 0.01076 | 0.55294647 |
| Olfr1497 | 0.0434 | -0.93768952 |
| Adcy6 | 0.74465 | -0.06232295 |
| Rpl3l | 0.44066 | -0.27158722 |
| Vmn1r42 | 0.01465 | -0.93998499 |
| Trpm6 | 0.00144 | -1.29049509 |
| Tmem216 | 0.01404 | -0.79317186 |
| Drc7 | 0.08227 | 0.69953285 |
| Piwil2 | 0.157 | 0.48650888 |
| Lat2 | 0.04747 | -0.39008631 |
| Fdxacb1 | 0.32616 | 0.1758185 |
| Dynlt3 | 0.02565 | -0.43296273 |
| Tex261 | 0.00144 | -1.28932819 |
| Otog | 0.8545 | -0.12201738 |
| Skor1 | 0.12252 | -0.47554419 |
| Dusp16 | 0.84766 | -0.12887087 |
| E430018J23Rik | 0.13743 | -0.29920857 |
| Myo1c | 0.03515 | -0.5935869 |
| Shq1 | 0.28841 | 0.43449424 |
| Ankrd60 | 0.00144 | -1.43619207 |
| Prss42 | 0.03202 | -1.60197432 |
| Zscan20 | 0.07836 | -0.3795936 |
| Micall1 | 0.9052 | -0.02171662 |
| Ldhc | 0.00185 | -0.99379477 |
| Krt8 | 0.08627 | -0.73201942 |
| Fam114a2 | 0.25665 | 0.22790724 |
| Olfr915 | 0.00144 | -1.31495282 |
| Cel | 0.16783 | -0.54710352 |
| Naaa | 0.25219 | -0.27571652 |
| Lap3 | 0.47683 | -0.14748976 |
| Trpc4ap | 0.39276 | -0.27033671 |
| Ubr7 | 0.04612 | 0.7069514 |
| Ywhab | 0.01974 | -0.51237877 |
| Col5a3 | 0.24139 | -0.26082064 |
| Sap30l | 0.0066 | -0.75291407 |
| Prss30 | 0.32209 | -0.36190996 |
| Sox14 | 0.06546 | -1.45539167 |
| Tcirg1 | 0.12878 | -0.37823561 |
| Tmem202 | 0.24142 | 0.75527094 |
| Rhd | 0.03954 | -0.70853312 |
| Gm10634 | 0.01129 | -0.86478707 |
| Ptpn1 | 0.00144 | -0.91333121 |
| Prkdc | 0.00704 | 1.22342793 |
| Tmem194 | 0.67774 | -0.18996439 |
| Gsn | 0.00144 | -1.27991442 |
| Arap2 | 0.01128 | 0.82937407 |
| Cilp | 0.16187 | -0.31831464 |
| Actg2 | 0.61909 | 0.29341519 |
| Arhgap15 | 0.01916 | 1.5386979 |
| A030005L19Rik | 0.00333 | 1.58276375 |
| Lhfpl3 | 0.00503 | -1.50546878 |
| Rhot1 | 0.00144 | 1.57457723 |
| Banf2 | 0.00394 | -1.45057155 |
| Gpn2 | 0.19431 | -0.35412711 |
| Map2k1 | 0.04167 | 0.72136814 |
| Dkk2 | 0.90767 | -0.13583692 |
| 4930503E24Rik | 0.00144 | -1.27730451 |
| Plekhg6 | 0.05065 | -1.40477304 |
| Tns1 | 0.0175 | -0.62961587 |
| Sppl3 | 0.00188 | -0.94149795 |
| Tmem173 | 0.01638 | -0.60943921 |
| B3gnt3 | 0.02733 | -0.83104121 |
| 1700022A21Rik | 0.00651 | -1.0995294 |
| Gpr151 | 0.00144 | -1.31884045 |
| Tecpr1 | 0.00397 | -0.70493967 |
| Tap2 | 0.8301 | 0.14303569 |
| Olfr642 | 0.00144 | -1.3348153 |
| Tm4sf1 | 0.03993 | -0.52421374 |
| Zfp7 | 0.76261 | 0.11409786 |
| Mycl | 0.00144 | -1.34324911 |
| Olfr578 | 0.08653 | -0.86086849 |
| Nusap1 | 0.00236 | 1.55320586 |
| Pi15 | 0.0525 | -0.45674304 |
| Olfr918 | 0.00145 | -1.25646236 |
| Pcdhb14 | 0.20124 | 0.37958892 |
| Col13a1 | 0.53865 | -0.16710193 |
| Rrad | 0.00942 | -1.49643441 |
| Smgc | 0.46565 | -0.23475336 |
| Gng8 | 0.01008 | -1.07424264 |
| Serpina3k | 0.61837 | -0.44315161 |
| Sim1 | 0.00144 | -1.24466058 |
| Exo1 | 0.43362 | 0.36122664 |
| Polr2c | 0.00235 | -0.86019619 |
| Myh8 | 0.00144 | -1.47681752 |
| AI853363 | 0.00144 | -1.27165604 |
| Plcd1 | 0.08284 | -0.31364871 |
| Inhba | 0.0065 | 1.8860673 |
| Kmt2e | 0.00256 | 2.0647596 |
| Hprt | 0.00509 | 0.71845048 |
| Nr4a1 | 0.03969 | -1.1653996 |
| Trpv5 | 0.00144 | -1.30017714 |
| Bcl2l12 | 0.00245 | -1.11413592 |
| Boll | 0.08215 | -0.84262967 |
| Mapk13 | 0.36191 | -0.58457564 |
| Apol7c | 0.01431 | -1.27355877 |
| Adam21 | 0.00492 | 1.21897078 |
| Cgnl1 | 0.12137 | 0.3392942 |
| Zfp35 | 0.75575 | -0.07875369 |
| 4930447A16Rik | 0.00144 | -1.27730451 |
| Ubn2 | 0.00367 | 0.9132026 |
| Vmn1r211 | 0.03567 | -1.96932322 |
| Ctsll3 | 0.00144 | -1.29878664 |
| Mcl1 | 0.03043 | 0.46374569 |
| Mfsd11 | 0.77312 | -0.11856212 |
| Cd93 | 0.06595 | 0.40575893 |
| Agpat2 | 0.81605 | 0.19489124 |
| Pdxdc1 | 0.00144 | -1.29049509 |
| Col4a3bp | 0.00146 | 1.48254157 |
| Shisa4 | 0.00814 | -0.82859568 |
| Cbr2 | 0.0018 | -0.91000204 |
| 0610009O20Rik | 0.64848 | -0.14559516 |
| B4galt4 | 0.05734 | 0.56499379 |
| Sv2c | 0.97065 | -0.01948209 |
| Il4 | 0.00144 | -1.29085005 |
| Tmcc1 | 0.68105 | -0.07552528 |
| Olfr1284 | 0.07416 | -1.65466624 |
| H2-T23 | 0.00158 | -1.11665913 |
| Ptrf | 0.002 | -1.04371094 |
| 4930448N21Rik | 0.25127 | 0.66463449 |
| Gnai3 | 0.00466 | 1.01370678 |
| Smim14 | 0.11145 | -0.46266774 |
| Bex2 | 0.00144 | -1.29085005 |
| Aplp2 | 0.48528 | 0.11173964 |
| Btnl9 | 0.00144 | 1.74419616 |
| Akirin1 | 0.07542 | 0.32753442 |
| Akt1 | 0.53709 | -0.15382792 |
| Eif1ax | 0.50734 | 0.14816924 |
| Ska2 | 0.00145 | -1.21051401 |
| Rnase4 | 0.16154 | 0.27653783 |
| Vstm2b | 0.00144 | -1.29085005 |
| Olfr1509 | 0.05227 | -3.0115858 |
| 4930533N22Rik | 0.00144 | -1.25118919 |
| Mrps18b | 0.05888 | -0.64035134 |
| Kcnip2 | 0.30119 | 0.83783879 |
| Utf1 | 0.02746 | -1.1626427 |
| Gng5 | 0.25891 | -0.31279086 |
| Spock3 | 0.00144 | -1.30017714 |
| Tcl1 | 0.7207 | -0.10055499 |
| Olfr1211 | 0.00144 | -2.06489626 |
| Pvr | 0.0166 | 0.66256501 |
| Ctdsp2 | 0.00422 | -0.7227587 |
| Olfr1198 | 0.05946 | -1.12694059 |
| Nog | 0.00252 | -2.20430484 |
| Ppp1r12a | 0.00209 | 1.00752674 |
| 1700001P01Rik | 0.39863 | -0.26219879 |
| Parp8 | 0.00515 | 0.78745517 |
| Zswim7 | 0.01865 | -0.5830659 |
| Uggt1 | 0.01955 | -0.53456952 |
| Dlx6os2 | 0.03372 | -0.45736771 |
| Mmadhc | 0.15379 | 0.60524446 |
| 9430038I01Rik | 0.01822 | -0.73910278 |
| Cd4 | 0.00144 | -1.28116048 |
| Metap1d | 0.00157 | 0.86338209 |
| Slco6c1 | 0.01123 | -1.04967308 |
| Gtf2e1 | 0.11889 | 0.37696613 |
| Ndst1 | 0.01089 | -0.64944956 |
| St8sia4 | 0.00814 | 1.47773895 |
| Polr1a | 0.00786 | -0.69739748 |
| Rit1 | 0.07412 | -0.68566401 |
| Fam175b | 0.0687 | -0.98336503 |
| Ccdc87 | 0.00144 | -1.20555712 |
| Prl7a2 | 0.48896 | -0.62065939 |
| Plcb3 | 0.43877 | -0.12579343 |
| Rnf40 | 0.02302 | -0.61601244 |
| Guk1 | 0.65063 | -0.23921676 |
| Mastl | 0.03754 | -1.27951219 |
| Olfr659 | 0.51623 | 0.72028467 |
| Icam4 | 0.61328 | 0.21483049 |
| Tsfm | 0.50989 | -0.29327078 |
| Alox5ap | 0.44646 | -0.16977916 |
| Cml1 | 0.0082 | -0.85197569 |
| Xpo7 | 0.14327 | -0.23828689 |
| Dap3 | 0.00974 | -0.57974131 |
| Mtmr9 | 0.44798 | -0.19186114 |
| Pelp1 | 0.09834 | -0.4076163 |
| Olfm2 | 0.00144 | -1.28116048 |
| Ints2 | 0.00158 | -1.17515383 |
| Ahsa2 | 0.00158 | 1.53747836 |
| Ddx18 | 0.02208 | 0.94699902 |
| Klhl40 | 0.08379 | -0.78996704 |
| Nfkbie | 0.0609 | -0.40577735 |
| Zc3h11a | 0.44276 | 0.1498485 |
| Dusp27 | 0.01131 | -0.68584209 |
| Fabp9 | 0.10583 | -0.51614554 |
| Xcl1 | 0.01586 | -1.08799441 |
| Cdk3-ps | 0.05774 | -0.41381966 |
| Ecel1 | 0.0074 | -0.71756998 |
| Sfxn5 | 0.40449 | 0.14421073 |
| Sdhb | 0.71085 | -0.23792424 |
| 1700001L19Rik | 0.00149 | -1.22582153 |
| Cxxc5 | 0.02824 | -0.58528399 |
| Lpar6 | 0.02621 | 0.7044992 |
| Ceacam14 | 0.00152 | -1.25878908 |
| Nubpl | 0.83373 | -0.04572935 |
| Nfxl1 | 0.21219 | -0.44503869 |
| Hyal5 | 0.00145 | -1.22376446 |
| Tjp3 | 0.179 | -1.01698052 |
| 4930447K03Rik | 0.17896 | -0.81070367 |
| Trpc7 | 0.00584 | -1.15337513 |
| Sct | 0.03961 | -0.60811354 |
| Rpa2 | 0.19643 | -0.34831649 |
| Ddx60 | 0.22891 | 0.54596769 |
| Sdcbp2 | 0.49999 | 0.1184035 |
| Adam6b | 0.15358 | -0.42592742 |
| Pms2 | 0.22321 | 0.2398429 |
| Cdkl1 | 0.47487 | -0.19300852 |
| Nod1 | 0.00585 | -0.62834696 |
| 1190005I06Rik | 0.01138 | -1.19666531 |
| Rbm5 | 0.00252 | 0.80500319 |
| Sesn1 | 0.0806 | 0.41837038 |
| Tbc1d10b | 0.00427 | -0.85201823 |
| B4galnt1 | 0.11104 | -0.37697355 |
| Adprhl1 | 0.18405 | -0.36592538 |
| Olfr1491 | 0.00145 | -1.26219879 |
| Tmem126a | 0.12876 | 1.04532767 |
| Adh7 | 0.00144 | -1.25118919 |
| Dmap1 | 0.00455 | -0.76217738 |
| Sapcd1 | 0.39929 | 0.26222811 |
| 1700006E09Rik | 0.00144 | -1.28898079 |
| Psma7 | 0.00547 | -0.8021344 |
| Clec1b | 0.05421 | -0.61159336 |
| Insrr | 0.01958 | -0.93429899 |
| Tmem202 | 0.00144 | -1.26560808 |
| Usf1 | 0.00144 | -1.49896882 |
| Ppp2r5d | 0.00145 | -0.93589037 |
| Ltbp3 | 0.0965 | -0.34729962 |
| E030002O03Rik | 0.00144 | -1.28116048 |
| Olfr601 | 0.00144 | -1.48396829 |
| Nvl | 0.0389 | 1.21184558 |
| Kcnmb4os1 | 0.00144 | -1.27730451 |
| Cnp | 0.12877 | -3.20324953 |
| Neurl1b | 0.07153 | -0.35200239 |
| Tmco4 | 0.04006 | -0.59332289 |
| Plxnb3 | 0.28316 | 0.25360066 |
| Hiatl1 | 0.00154 | 1.13749862 |
| Paxip1 | 0.12545 | 0.40738543 |
| Npff | 0.09907 | 0.55285432 |
| Commd9 | 0.15274 | -0.41914589 |
| Nwd1 | 0.22401 | -0.39664464 |
| Olfr706 | 0.01318 | -0.9524986 |
| Zswim1 | 0.06784 | -0.59487174 |
| Ice1 | 0.00144 | 1.59108152 |
| Acy3 | 0.78984 | 0.09063008 |
| Myo5c | 0.0784 | 1.12771596 |
| Stab1 | 0.00301 | -0.85745579 |
| Cnot7 | 0.17967 | 0.34626721 |
| Pla2g2d | 0.30946 | 0.25072054 |
| Hoxb6 | 0.08989 | 0.35826145 |
| Thumpd3 | 0.00144 | 1.44240801 |
| Zwilch | 0.9072 | 0.07926784 |
| 1700029P11Rik | 0.17273 | -0.53463686 |
| Gm40625 | 0.00325 | -1.15314387 |
| C130026I21Rik | 0.00144 | -1.25118919 |
| Clint1 | 0.01135 | 0.6638843 |
| Dnajc4 | 0.04178 | -0.72169064 |
| Ik | 0.00286 | 0.94247831 |
| Zfp239 | 0.00144 | -1.23532597 |
| Nkain3 | 0.00151 | -1.17792995 |
| Fam171a1 | 0.13697 | 0.26920651 |
| Mill2 | 0.76135 | 0.05738526 |
| Igsf3 | 0.12946 | -0.26589067 |
| Cd163 | 0.01074 | 1.19721582 |
| Pi4k2b | 0.63398 | 0.26321068 |
| Cab39 | 0.00639 | -0.62070658 |
| Mad2l1 | 0.0414 | 0.70351245 |
| Slc34a3 | 0.01006 | -2.13362648 |
| Tubgcp5 | 0.4128 | -1.17289236 |
| Phactr3 | 0.0643 | -0.99936063 |
| Ccdc71l | 0.00715 | -0.61481153 |
| Tnfrsf14 | 0.12585 | -1.03643316 |
| 1700128E19Rik | 0.01395 | -1.01228913 |
| Ccdc114 | 0.02724 | -1.0081862 |
| Ell2 | 0.00151 | 1.1814257 |
| Pth | 0.00144 | -1.31495282 |
| Ptchd2 | 0.04721 | -1.95900781 |
| Ppfibp2 | 0.29716 | 0.15918884 |
| Itga3 | 0.07943 | -0.58033277 |
| Srsf12 | 0.00144 | -1.31495282 |
| Efcab3 | 0.0167 | -1.20082152 |
| Hdgfl1 | 0.05632 | -2.22373749 |
| H2-Q10 | 0.48789 | 0.2928455 |
| Cdc42ep2 | 0.01342 | -0.81624224 |
| A730008H23Rik | 0.01462 | -0.75422738 |
| A930018P22Rik | 0.01409 | -1.44131614 |
| Birc5 | 0.39647 | -0.35928779 |
| Pbk | 0.06525 | 0.95613882 |
| Olfr961 | 0.00144 | -1.65909331 |
| Chst7 | 0.00144 | -0.97782983 |
| Adcy4 | 0.0037 | -0.85947406 |
| Cts6 | 0.00145 | -1.22376446 |
| Fpgt | 0.00144 | 2.58698577 |
| E330009J07Rik | 0.00997 | 1.7590392 |
| Eif2b2 | 0.01906 | -0.67373073 |
| Acsm5 | 0.16945 | 0.65053358 |
| Pcsk2 | 0.11223 | 0.6871092 |
| Plxna2 | 0.00144 | 1.21571913 |
| Etnk1 | 0.00387 | -0.76595124 |
| Lrfn2 | 0.00463 | -1.02842441 |
| Map1a | 0.19881 | -0.99324424 |
| Lypd8 | 0.00298 | -1.42363422 |
| Nxph1 | 0.00144 | -1.28116048 |
| Smyd3 | 0.00871 | -0.63206933 |
| 1700003G18Rik | 0.95641 | 0.07776773 |
| Mfap1a | 0.00776 | 0.71201579 |
| Crhr1 | 0.05226 | -0.88511191 |
| Olfr1218 | 0.06507 | -0.85340528 |
| Cd2ap | 0.02182 | 0.80313435 |
| Slc5a4b | 0.00145 | -1.25646236 |
| Olfr124 | 0.02964 | -1.19645932 |
| Pkd2l2 | 0.00422 | 1.60202577 |
| Htatip2 | 0.89628 | 0.03691144 |
| Zfp72 | 0.00522 | 1.50750144 |
| Stau2 | 0.00778 | 0.78713789 |
| Fcho1 | 0.00761 | -0.9926579 |
| Slfn3 | 0.0353 | 1.40994793 |
| Arhgef26 | 0.00145 | -1.25646236 |
| Sec23a | 0.18914 | 0.6395566 |
| Kcnc4 | 0.73135 | 0.16517581 |
| 2310002L09Rik | 0.00646 | 1.12115413 |
| Isyna1 | 0.09187 | -0.37640129 |
| Rap1a | 0.00144 | 1.62626228 |
| Ceacam20 | 0.27747 | -0.3692513 |
| Tat | 0.70992 | 0.08537138 |
| 4933440M02Rik | 0.00702 | -1.09418462 |
| Trp53inp2 | 0.11874 | -0.43146337 |
| Mpzl1 | 0.21021 | -0.21196128 |
| Gbas | 0.24224 | 0.43850809 |
| Ociad1 | 0.03935 | 0.77311305 |
| Olfr472 | 0.00149 | -1.25408 |
| Cenpp | 0.1177 | 0.37518981 |
| 4930430O22Rik | 0.22798 | -0.91464386 |
| Susd1 | 0.02283 | -1.48433239 |
| Kcnq4 | 0.05019 | -0.4526358 |
| Dynlrb2 | 0.27385 | -0.63039704 |
| Cep135 | 0.01698 | 1.85034756 |
| 4930502E18Rik | 0.78978 | -0.14758973 |
| Top2b | 0.0015 | 1.05505501 |
| Phykpl | 0.1325 | 0.3657064 |
| Tead2 | 0.01045 | -0.55300549 |
| Gabrb2 | 0.00548 | 1.38753623 |
| Dnah7a | 0.78617 | 0.16142463 |
| Gm5176 | 0.8451 | -0.09617671 |
| Vmn1r205 | 0.00144 | -1.28116048 |
| Vmn1r27 | 0.00145 | -1.22376446 |
| Gm32819 | 0.18556 | -0.39942229 |
| Rhou | 0.06356 | -0.46903172 |
| En1 | 0.01946 | -1.02877696 |
| Dpep1 | 0.00254 | -0.90091791 |
| Rpl38 | 0.00144 | -1.21420107 |
| Csn3 | 0.00144 | -1.27699683 |
| Csn3 | 0.00296 | -1.24082201 |
| Olfr380 | 0.00144 | -1.24811894 |
| Adamts2 | 0.00173 | -1.01136089 |
| 2310030G06Rik | 0.95982 | 0.01255871 |
| Bri3bp | 0.02276 | -0.82349676 |
| Mdh1b | 0.0259 | -1.11764526 |
| Zfp711 | 0.00144 | -1.33632959 |
| Rpp30 | 0.01423 | 1.03510915 |
| Cks1b | 0.00618 | -0.7006406 |
| Capn15 | 0.42733 | 0.47518382 |
| Tubgcp2 | 0.96788 | 0.01591245 |
| Sh2d2a | 0.19498 | -0.36228716 |
| Gga1 | 0.02276 | -0.80646509 |
| Lce1l | 0.13044 | 0.41742879 |
| Enpp3 | 0.00145 | -1.22376446 |
| Pgap2 | 0.09337 | -0.48615711 |
| Ptf1a | 0.13733 | -0.66714732 |
| Nr1h5 | 0.00144 | -1.31978601 |
| Cacng2 | 0.44681 | -0.18883857 |
| Rpusd2 | 0.0068 | -1.13602825 |
| Vmn1r85 | 0.00144 | -1.31495282 |
| Sptbn4 | 0.92306 | -0.01633168 |
| Mypop | 0.00348 | -0.8683 |
| Rhobtb2 | 0.12154 | -0.41819571 |
| Rraga | 0.01528 | 0.56735626 |
| Coa3 | 0.00768 | -0.8045556 |
| Ptpmt1 | 0.55935 | 0.11832592 |
| Rgcc | 0.626 | -0.10282097 |
| Tcam1 | 0.00144 | -1.31495282 |
| Dennd6a | 0.46295 | -0.14386855 |
| 4930562A09Rik | 0.06654 | -1.04844645 |
| Sra1 | 0.99847 | -0.00047845 |
| Snx33 | 0.00482 | -0.69207485 |
| Inpp4b | 0.42647 | -0.14766094 |
| Sprr4 | 0.1625 | -0.72880437 |
| Zfand3 | 0.00728 | -0.76921087 |
| 6530403H02Rik | 0.00144 | -1.25927895 |
| Pcmt1 | 0.00335 | 0.8034635 |
| 2700097O09Rik | 0.01106 | 0.58742486 |
| Ovol1 | 0.00158 | -1.2446827 |
| Olfr25 | 0.00144 | -1.28898079 |
| Prr5l | 0.02305 | -0.49410095 |
| Insc | 0.36997 | 0.33321421 |
| Zcchc6 | 0.29685 | 0.29722008 |
| Olfr908 | 0.00907 | -1.03958412 |
| Plcb4 | 0.00144 | 2.04089091 |
| Mdk | 0.04907 | -0.39360432 |
| Zdhhc25 | 0.43621 | -0.48360738 |
| Ggnbp2 | 0.00144 | 1.84479315 |
| Olfr981 | 0.00144 | -1.30253725 |
| B3gnt4 | 0.10531 | -2.74663006 |
| Gm9079 | 0.68107 | -0.08544256 |
| Pnrc2 | 0.02743 | 0.64012408 |
| Epyc | 0.99687 | 0.00128411 |
| Pkp2 | 0.0919 | 0.29896214 |
| Gsdma2 | 0.00595 | -1.16184234 |
| Htra1 | 0.04806 | -0.65072441 |
| Calcrl | 0.00144 | 2.25009186 |
| Smndc1 | 0.01165 | 0.81481511 |
| Myh2 | 0.07944 | -1.08010478 |
| Egfl8 | 0.06833 | -0.7335406 |
| Snx17 | 0.01178 | -0.5965155 |
| Cln8 | 0.04098 | -0.59381589 |
| Tnrc18 | 0.00144 | -1.47183229 |
| Grm6 | 0.00144 | -1.30626366 |
| 4930456L15Rik | 0.02264 | -0.92886545 |
| Tmem184b | 0.00671 | -0.64715748 |
| Dtwd2 | 0.70152 | -0.08412187 |
| Arl8a | 0.02178 | -0.52516274 |
| Gnpat | 0.21293 | 0.41015599 |
| Ppm1f | 0.04605 | -0.38730534 |
| Jade1 | 0.01245 | 0.83505357 |
| 9430016H08Rik | 0.07615 | 0.51010869 |
| Klhl4 | 0.00144 | 2.45665202 |
| Akap6 | 0.00792 | 0.70987858 |
| Adgrv1 | 0.00144 | -1.28898079 |
| Popdc2 | 0.10586 | -0.5006945 |
| Tm9sf3 | 0.00396 | 0.92365093 |
| Capn12 | 0.00145 | -1.24191372 |
| Rlf | 0.00144 | 1.16139078 |
| Slc26a4 | 0.07191 | 0.75004624 |
| Klhl38 | 0.05556 | 0.57893704 |
| Ppil6 | 0.17269 | 0.26523784 |
| Fam49b | 0.02703 | 0.73232378 |
| Tk1 | 0.613 | -0.2302863 |
| Pds5b | 0.00325 | 1.51916949 |
| Nr1d1 | 0.01613 | -0.61476472 |
| Khdrbs2 | 0.73721 | -0.09507126 |
| Rtcb | 0.8371 | -0.05928921 |
| Pask | 0.42215 | 0.14801998 |
| Lmo2 | 0.77749 | -0.05848075 |
| Lamb3 | 0.1518 | 0.48464886 |
| Tbc1d23 | 0.00144 | 1.53297172 |
| Gm39214 | 0.00144 | -1.29049509 |
| Ric1 | 0.40895 | 0.17702598 |
| Slc39a10 | 0.00209 | 1.45896682 |
| Polr3d | 0.30824 | 0.31021525 |
| Cd81 | 0.00567 | -0.76530207 |
| Cd81 | 0.08565 | -0.51335147 |
| Casp9 | 0.18394 | -0.43347742 |
| Plin3 | 0.0069 | -0.75264212 |
| Chmp3 | 0.18387 | 0.30170743 |
| Tln2 | 0.00144 | -1.24357682 |
| Zbed3 | 0.22561 | -0.39856331 |
| Dock5 | 0.33912 | -0.28487876 |
| Pnmal1 | 0.04008 | -2.00403383 |
| Fmnl2 | 0.00464 | 1.22350993 |
| H2-Ea-ps | 0.35354 | -0.68032393 |
| Olfr922 | 0.00158 | -1.22727468 |
| D730001G18Rik | 0.04217 | -1.39929302 |
| Tspo | 0.00144 | -1.16653541 |
| Arf6 | 0.00206 | -0.87613256 |
| Mesdc2 | 0.19698 | 0.24111265 |
| 4930595L18Rik | 0.00164 | -1.29347279 |
| Ehbp1l1 | 0.01527 | -0.53344819 |
| Abcg1 | 0.06992 | -0.557779 |
| Tmem254a | 0.09395 | -0.40363186 |
| Tbc1d10a | 0.00158 | -0.8940365 |
| Tmeff1 | 0.08444 | 0.66901428 |
| Emilin1 | 0.00158 | -0.95282229 |
| Rspo2 | 0.00144 | -1.28116048 |
| Mlycd | 0.03571 | -0.90665755 |
| Ikbke | 0.93078 | -0.03234385 |
| Olfr1463 | 0.00144 | -1.31884045 |
| Igbp1 | 0.09954 | 0.31140674 |
| Ctdsp1 | 0.0222 | -0.54798493 |
| Olfr17 | 0.31587 | -0.59534814 |
| Vps33b | 0.00145 | 0.97367857 |
| Man1c1 | 0.79663 | -0.09227802 |
| Micall1 | 0.04122 | -0.72113314 |
| Eef2kmt | 0.21833 | -0.34611964 |
| Gm9968 | 0.08237 | -1.22919252 |
| Csnk1g3 | 0.0051 | 0.91861926 |
| Lrfn5 | 0.01722 | -0.92735116 |
| AI314180 | 0.22481 | -0.32543694 |
| Krtap21-1 | 0.71085 | 0.38208112 |
| Tpr | 0.19814 | 0.44456941 |
| Zfp33b | 0.00144 | 1.71394374 |
| Klk1b9 | 0.21406 | -0.81392177 |
| Trim62 | 0.00144 | 1.1732025 |
| Adck3 | 0.51091 | -0.29330792 |
| 1700037H04Rik | 0.04353 | -0.70211692 |
| 1700037H04Rik | 0.0047 | -0.81584903 |
| Stk24 | 0.75771 | -0.06025854 |
| Cln3 | 0.09266 | -0.32600581 |
| Fam81a | 0.78118 | -0.080628 |
| Tnks2 | 0.02815 | 0.44465496 |
| Ranbp10 | 0.97518 | -0.00592161 |
| Arsg | 0.2049 | 0.28651662 |
| Tspan32 | 0.8139 | -0.13684067 |
| Ttc21b | 0.23291 | -0.5455697 |
| L2hgdh | 0.0108 | 0.74073319 |
| 4930533I22Rik | 0.00145 | -1.2388121 |
| Apobec4 | 0.00144 | -1.28116048 |
| Cd47 | 0.19295 | 0.27378779 |
| Pfn1 | 0.00144 | -1.17848646 |
| Vstm5 | 0.49601 | -0.37665893 |
| Chmp2b | 0.05905 | 0.34000985 |
| Olfr786 | 0.00144 | -1.31884045 |
| Gng2 | 0.01673 | -0.5332268 |
| Gdf9 | 0.00272 | -0.81020886 |
| Fkbp8 | 0.17408 | -0.53253847 |
| B230118H07Rik | 0.23657 | 0.21395925 |
| Aldoc | 0.03194 | -0.64957317 |
| Unc5d | 0.00144 | -1.49567869 |
| Gm41082 | 0.59554 | -0.22838147 |
| Fam227b | 0.00144 | -1.57331972 |
| 4930431F12Rik | 0.08983 | -1.94489184 |
| Gm36371 | 0.54253 | -0.10157293 |
| Pacsin1 | 0.06868 | 0.9222645 |
| Rnpepl1 | 0.00556 | -0.82841518 |
| Msl3l2 | 0.17847 | -0.34130333 |
| Kmt2c | 0.26974 | 0.19175072 |
| Cdca7l | 0.42258 | -0.51442107 |
| Ppp2r2b | 0.00144 | -1.28116048 |
| Rnf165 | 0.30753 | 0.3385122 |
| Tacc3 | 0.91459 | -0.02795567 |
| Angptl7 | 0.13155 | 0.54400807 |
| Nfatc1 | 0.02409 | -0.49001859 |
| Mmp15 | 0.02872 | -0.74193544 |
| Zfp119a | 0.00144 | 2.20219589 |
| Zfyve16 | 0.14772 | 0.53970003 |
| Cela1 | 0.07102 | -2.28222384 |
| Tmem125 | 0.00144 | -1.33632959 |
| Olfr599 | 0.00144 | -1.29085005 |
| H2-Q2 | 0.00227 | -1.07408888 |
| Bpifa5 | 0.05454 | 0.61303106 |
| Ola1 | 0.00459 | 0.89728385 |
| Pxdc1 | 0.00901 | -0.6089247 |
| Aknaos | 0.00144 | -1.29051721 |
| 1700123K08Rik | 0.03261 | 1.25447052 |
| Fbxo31 | 0.02293 | -0.67535335 |
| Slc41a2 | 0.24587 | 0.44757642 |
| Tsku | 0.03442 | -0.68098471 |
| Tmem11 | 0.10852 | -0.51365741 |
| Prss51 | 0.00144 | -1.27165604 |
| Plcl2 | 0.78725 | 0.04389027 |
| Med10 | 0.86469 | 0.03452208 |
| Serpinb9d | 0.00144 | -1.26428512 |
| Prp2 | 0.00592 | -1.42017472 |
| Six5 | 0.22221 | -0.47018122 |
| Ddx28 | 0.7962 | -0.08582811 |
| Ttn | 0.42019 | 0.1577371 |
| Tmprss6 | 0.1554 | -1.09224884 |
| Gm19272 | 0.00145 | -1.2388121 |
| Mfap3l | 0.00356 | -1.36083344 |
| Gm5115 | 0.00301 | 1.01748708 |
| Odf3 | 0.29452 | -0.29374144 |
| Alpk2 | 0.63372 | -0.17002014 |
| Prmt2 | 0.00915 | -0.84741223 |
| Oxa1l | 0.50467 | 0.21212517 |
| Gm12977 | 0.02785 | -1.75208529 |
| Wdr53 | 0.10451 | 0.27402172 |
| Kcns3 | 0.79965 | 0.16573688 |
| Rictor | 0.52814 | -0.13104084 |
| 1110008E08Rik | 0.15938 | -1.06019075 |
| Mturn | 0.13103 | 0.30961076 |
| Olfr820 | 0.00144 | -1.29085005 |
| Gfer | 0.05963 | -0.75445667 |
| Rgs10 | 0.0789 | -0.33868722 |
| Tex26 | 0.03272 | -1.28838569 |
| Tex26 | 0.46095 | 0.33261718 |
| Mtr | 0.00669 | -0.79620281 |
| Fiz1 | 0.90175 | 0.05568192 |
| Als2cr11 | 0.00499 | -1.49245118 |
| Ankrd44 | 0.02757 | 0.81664213 |
| Mdh1 | 0.32458 | 0.53711571 |
| Mettl7a1 | 0.00182 | 1.60080584 |
| Zscan5b | 0.07277 | -2.39927973 |
| Efna5 | 0.02003 | -0.7171018 |
| Polr3a | 0.56252 | 0.15048527 |
| Ribc1 | 0.10659 | -0.44215795 |
| 1700019L03Rik | 0.02773 | -0.91258439 |
| Chst3 | 0.00144 | -1.24613586 |
| 4932442E05Rik | 0.00144 | -1.27165604 |
| Mmel1 | 0.06264 | 0.46094551 |
| Slc2a4 | 0.03174 | -0.63743491 |
| Iffo2 | 0.00761 | -1.19970893 |
| Cxcl2 | 0.05346 | -0.9445058 |
| Mrpl52 | 0.11976 | -0.52097873 |
| 2410016O06Rik | 0.00715 | -0.68401847 |
| 2410004P03Rik | 0.01501 | -0.68789329 |
| Kif1c | 0.31779 | -0.22403227 |
| Gm16532 | 0.00144 | -1.27699683 |
| Scamp1 | 0.10734 | 0.31418323 |
| Lemd3 | 0.00144 | 1.1314994 |
| Gng10 | 0.02281 | -0.72863204 |
| Olfr665 | 0.06465 | -2.15423558 |
| Ddx59 | 0.03731 | 0.67201446 |
| Eogt | 0.16459 | 0.25705176 |
| Anxa10 | 0.00144 | -1.58979569 |
| Hbp1 | 0.23006 | 0.24426488 |
| Usp28 | 0.23256 | 0.2997958 |
| Wfs1 | 0.42161 | -0.25504025 |
| Vmn2r10 | 0.00144 | -1.32313902 |
| Trp63 | 0.0494 | -1.97980955 |
| Tfpi2 | 0.863 | 0.05400492 |
| Tac1 | 0.0304 | -0.82733346 |
| Bag5 | 0.04259 | 0.36899886 |
| Lipt1 | 0.00145 | -1.26219879 |
| Pcgf1 | 0.07002 | 0.41677276 |
| Emd | 0.002 | -0.9370629 |
| Olfr594 | 0.00144 | -1.27165604 |
| Rps6kl1 | 0.81195 | -0.04759306 |
| Ddost | 0.02484 | -0.61800873 |
| Dleu2 | 0.02028 | 0.90327828 |
| Col4a5 | 0.20628 | 0.31143355 |
| Chrd | 0.09224 | -1.25453685 |
| Olfr389 | 0.00409 | -1.75582677 |
| Casp6 | 0.03864 | 0.52659334 |
| Il17b | 0.04514 | -2.16274728 |
| Atp11c | 0.00893 | 0.93956566 |
| 9530053A07Rik | 0.0015 | -1.20535468 |
| Plac9a | 0.00144 | -1.49702654 |
| BC021614 | 0.00144 | -1.55888744 |
| Rnf4 | 0.83597 | 0.03773449 |
| Slc37a1 | 0.31555 | -0.31069947 |
| Ptprb | 0.00648 | 0.88759711 |
| Prodh | 0.85057 | 0.04287339 |
| Slc6a17 | 0.34079 | 0.65777521 |
| Tmem132c | 0.00145 | -1.26219879 |
| Tfb1m | 0.00145 | -1.22376446 |
| Hmx2 | 0.062 | -2.85704897 |
| Gm26637 | 0.06971 | 0.55102241 |
| Fstl1 | 0.03315 | -0.50123532 |
| Emc4 | 0.38572 | -0.21054729 |
| Tmem59l | 0.8404 | 0.06895367 |
| Gpr180 | 0.92899 | 0.02788319 |
| Pxn | 0.03023 | -0.44784333 |
| Arhgap12 | 0.00169 | 1.22075428 |
| Suz12 | 0.00182 | 1.07577602 |
| Parp12 | 0.01332 | 0.5148691 |
| Actrt2 | 0.00144 | -1.50707897 |
| Atp6v1e2 | 0.86263 | 0.09792762 |
| Tpmt | 0.3975 | 0.19905563 |
| Wipi2 | 0.26514 | -0.236392 |
| Oit3 | 0.00144 | -1.28898079 |
| Frem2 | 0.00422 | -0.78447781 |
| Polr2k | 0.7781 | 0.05797199 |
| Tead1 | 0.03868 | 0.47159156 |
| Zfp280d | 0.62115 | 0.24121277 |
| Vgll1 | 0.00144 | -1.28116048 |
| Sephs1 | 0.36789 | 0.18591119 |
| Sephs1 | 0.11664 | -0.28811806 |
| Cox11 | 0.59079 | 0.10490578 |
| Haus4 | 0.01102 | -0.73329305 |
| Evx2 | 0.36474 | -0.22843755 |
| Epha1 | 0.07335 | -0.52573537 |
| Rnls | 0.07803 | 1.50458639 |
| Stk17b | 0.01913 | 0.7761815 |
| Slc6a3 | 0.00144 | -1.30017714 |
| Cpa3 | 0.98703 | -0.01305053 |
| Ccr1l1 | 0.17422 | -0.74101761 |
| Tbc1d20 | 0.01167 | -0.72449562 |
| 2310039H08Rik | 0.00438 | -1.05773957 |
| Tnpo3 | 0.0564 | 0.37352225 |
| Nap1l3 | 0.24405 | 0.74433427 |
| Scnn1a | 0.8504 | 0.06718089 |
| Col19a1 | 0.00152 | -1.25878908 |
| Psma8 | 0.18016 | 0.24756464 |
| Ap2b1 | 0.31443 | -0.18431751 |
| Nrde2 | 0.42056 | -0.23715169 |
| Klf4 | 0.04511 | 0.4696662 |
| Ppp3ca | 0.00145 | 1.46804973 |
| Has2 | 0.24049 | 0.37049117 |
| Hdac11 | 0.71153 | -0.17035761 |
| Stpg1 | 0.00406 | -1.01402135 |
| Fam131a | 0.07446 | -0.60898593 |
| Tmem208 | 0.00409 | -0.82382011 |
| Ntng1 | 0.72029 | -0.23948209 |
| Adamtsl3 | 0.03934 | 0.63936147 |
| Htra2 | 0.0383 | -0.53986062 |
| Macrod1 | 0.01131 | -0.82168461 |
| Spata9 | 0.00144 | -1.31495282 |
| Il1b | 0.03355 | 1.1946448 |
| Tgfbi | 0.78675 | -0.05234006 |
| Scn2b | 0.80597 | -0.18274868 |
| Olfr60 | 0.05278 | -0.73456296 |
| Mcm10 | 0.07927 | 0.53292364 |
| Chd8 | 0.22627 | -0.22456291 |
| Skida1 | 0.00526 | -1.02478608 |
| Nat3 | 0.01697 | 1.02797961 |
| Cog7 | 0.02952 | 0.41262708 |
| Pfkfb3 | 0.68169 | 0.11508357 |
| Tmtc3 | 0.15319 | -0.56974188 |
| Eri3 | 0.00144 | -1.24900642 |
| Lama4 | 0.63013 | 0.07480269 |
| Lrrc41 | 0.01694 | -0.88217348 |
| Klk10 | 0.00223 | -1.00712147 |
| Ndufv2 | 0.24673 | 0.80047679 |
| Cxadr | 0.14559 | -0.62361743 |
| Cep41 | 0.00966 | -0.95832451 |
| Tmem261 | 0.02897 | -0.65926611 |
| Olfr733 | 0.00144 | -1.27165604 |
| Aoc1 | 0.02941 | -1.07095875 |
| Serpinb1c | 0.01379 | -0.72407471 |
| Atp6v0e2 | 0.06949 | -0.51628417 |
| Sart3 | 0.07364 | -0.31967949 |
| 8430419K02Rik | 0.04698 | -0.91567309 |
| Olfr716 | 0.00145 | -1.2388121 |
| Golga5 | 0.0026 | 1.06278487 |
| Selp | 0.03733 | -0.59631222 |
| Neurl4 | 0.00352 | -0.77278528 |
| Rlbp1 | 0.00144 | -1.24353547 |
| Chst13 | 0.00145 | -1.22376446 |
| Dpy19l2 | 0.00144 | -1.33632959 |
| Rin1 | 0.27559 | -0.79990046 |
| Ptpn2 | 0.00144 | 1.32076787 |
| Cthrc1 | 0.00144 | -1.29864962 |
| Prl7a1 | 0.00144 | -1.27165604 |
| Kcne1 | 0.42696 | -0.36528263 |
| Slc27a6 | 0.04441 | -0.9225944 |
| Golgb1 | 0.00878 | 0.83832006 |
| Wdr47 | 0.00194 | 1.15234993 |
| Hpca | 0.00144 | -1.23155796 |
| Ctc1 | 0.0975 | -0.48037004 |
| Muc20 | 0.1332 | -0.55835935 |
| Gusb | 0.16398 | -0.24836946 |
| Cptp | 0.34716 | -0.29389644 |
| Gpr50 | 0.68241 | 0.12055616 |
| Gpr83 | 0.72915 | -0.15646337 |
| Olfr677 | 0.00145 | -1.25646236 |
| Eif3j1 | 0.00144 | 1.26172886 |
| Nudt2 | 0.8921 | 0.0311849 |
| Lrrc45 | 0.16432 | 0.35232404 |
| Alpk3 | 0.0243 | -0.6492547 |
| Dscaml1 | 0.00144 | -1.36211029 |
| Sarnp | 0.10712 | 0.34349691 |
| Traf3ip2 | 0.39947 | -0.19532884 |
| 1110037F02Rik | 0.00481 | 0.91325644 |
| Olfr690 | 0.00144 | -1.3124439 |
| Wnt3a | 0.00145 | -1.2388121 |
| Vcam1 | 0.19284 | 0.30087388 |
| Tsta3 | 0.04533 | -0.63131506 |
| Pcnp | 0.00144 | 1.44204051 |
| Nebl | 0.11515 | -0.47450907 |
| Vmn1r82 | 0.00158 | -1.2446827 |
| Ino80b | 0.05995 | -1.56234828 |
| Tmem30a | 0.01893 | 2.39588728 |
| Cyp4f13 | 0.03945 | 0.54923078 |
| Fcer2a | 0.64868 | 0.26007638 |
| Pptc7 | 0.78385 | 0.07027534 |
| Mrpl18 | 0.32342 | 0.27280946 |
| Hdx | 0.97438 | 0.04435254 |
| Ppp1r2-ps9 | 0.00144 | -1.22098834 |
| Glrx | 0.8623 | 0.07782461 |
| Slc17a8 | 0.93982 | -0.05160569 |
| Cacng1 | 0.18438 | -1.87798958 |
| Olfr502 | 0.38966 | -0.50049856 |
| Ly6g5c | 0.05989 | -0.89966591 |
| Ercc4 | 0.05607 | -0.40963994 |
| Spata21 | 0.26932 | -0.83113712 |
| Ttc21a | 0.25126 | -0.70343834 |
| Alkbh4 | 0.01344 | -0.58205941 |
| Rtn2 | 0.02768 | -0.76674408 |
| Ccdc79 | 0.00144 | -1.28898079 |
| Prtn3 | 0.09181 | -1.03199009 |
| Cyp2c44 | 0.00144 | -1.31518407 |
| Prrx1 | 0.0103 | -0.70721745 |
| Atoh8 | 0.00209 | -0.82256687 |
| Prss54 | 0.01062 | -1.12200941 |
| Olfr1204 | 0.00368 | -1.10379914 |
| Afm | 0.01176 | -1.37182574 |
| Hcrtr1 | 0.87834 | -0.16450211 |
| Zbtb24 | 0.48778 | -0.32065242 |
| Rpl36al | 0.80009 | -0.05943786 |
| Akap3 | 0.0049 | -1.29358273 |
| Msmo1 | 0.16982 | 0.28262038 |
| Tctex1d2 | 0.27933 | 0.22103706 |
| Apln | 0.29389 | 0.33133114 |
| Slc35b1 | 0.04874 | -0.37610513 |
| Polr1e | 0.82436 | -0.0474804 |
| Gtdc1 | 0.13076 | -2.25880927 |
| Rab31 | 0.93405 | -0.01582423 |
| Sqrdl | 0.26391 | 0.34492983 |
| Cxcl14 | 0.39167 | -0.20966052 |
| Pcdh10 | 0.00431 | -1.44280189 |
| Agr2 | 0.06187 | -1.15736866 |
| N4bp3 | 0.002 | -0.92622923 |
| Stc2 | 0.00206 | -0.94750729 |
| Acbd6 | 0.03234 | -0.68482531 |
| Zdhhc24 | 0.01606 | -0.6295298 |
| Wdr92 | 0.45587 | 0.15568252 |
| Egflam | 0.01876 | 0.59824685 |
| Ndufab1 | 0.01635 | -0.58888403 |
| Metrn | 0.04924 | -0.66400686 |
| Gm7544 | 0.54446 | -0.55302443 |
| Mterf4 | 0.03993 | 0.90000674 |
| Ttl | 0.00145 | -0.92106284 |
| Chtf18 | 0.00235 | -0.93581542 |
| Dio2 | 0.29947 | 0.28844844 |
| Olfr906 | 0.02731 | -0.93945247 |
| Gltscr2 | 0.15171 | -0.4782645 |
| Prss36 | 0.03004 | -0.78749514 |
| Cr2 | 0.02862 | -2.15078827 |
| Gm40824 | 0.00944 | 1.86585335 |
| Ston1 | 0.00145 | -1.05664167 |
| Ppp1r12c | 0.58396 | -0.08871677 |
| Trappc5 | 0.00145 | -1.05321983 |
| Ak3 | 0.84148 | 0.04474318 |
| Mcm8 | 0.00214 | 1.35859512 |
| Olfr113 | 0.00916 | -1.06669723 |
| Camkk1 | 0.01633 | -0.65434929 |
| Tnfsf14 | 0.00556 | -1.18756951 |
| Slc7a13 | 0.00203 | -1.26528643 |
| 3930402G23Rik | 0.02792 | -0.87144901 |
| Pmel | 0.16356 | -0.74468518 |
| Klhl25 | 0.00144 | -1.69934328 |
| Ralgapa1 | 0.00529 | 0.79449343 |
| Ercc6l | 0.1411 | -0.84519499 |
| Ulk3 | 0.39116 | 0.13946723 |
| Ptger4 | 0.95697 | -0.01437803 |
| Psmd4 | 0.14 | -0.43534279 |
| Tmem117 | 0.18778 | 0.49354812 |
| Por | 0.09202 | -0.37969987 |
| Pla2g5 | 0.68794 | 0.26818901 |
| 1810008B01Rik | 0.00173 | -1.6426896 |
| Cachd1 | 0.00144 | -1.28116048 |
| Olfr1297 | 0.00144 | -1.55798917 |
| Guca1a | 0.0088 | -1.08115135 |
| Fam180a | 0.00144 | -1.29049509 |
| Shisa7 | 0.91729 | -0.07779831 |
| Fmod | 0.0325 | -1.00578121 |
| Anxa8 | 0.01184 | -1.10489666 |
| Olfr969 | 0.00144 | -1.56519132 |
| Ankrd32 | 0.00144 | 1.35348663 |
| Lkaaear1 | 0.11524 | -0.60203383 |
| Tgm6 | 0.01902 | -0.99270387 |
| Lrrc69 | 0.04184 | -1.12274058 |
| Krt1 | 0.03568 | -1.58581667 |
| 1110008P14Rik | 0.14484 | -0.51282653 |
| Ncf1 | 0.01853 | -0.67631755 |
| Vmn1r227 | 0.30134 | 0.43789394 |
| Sypl2 | 0.27913 | -0.68321026 |
| Large | 0.4279 | 0.16012213 |
| Hfe2 | 0.40139 | 0.66442642 |
| Rpl37 | 0.75668 | -0.11599602 |
| Lsm1 | 0.91751 | 0.04029955 |
| LOC102633151 | 0.03608 | -0.65359453 |
| Cacna1b | 0.00144 | -1.26560808 |
| Fezf2 | 0.00144 | -1.28116048 |
| Lce1i | 0.0028 | -2.21802803 |
| Zfp773 | 0.01962 | 0.77800147 |
| Reep3 | 0.22732 | 0.30764305 |
| Seh1l | 0.01443 | 1.02540949 |
| Ahcyl1 | 0.00742 | 1.23194009 |
| Slc39a5 | 0.22933 | -0.66013988 |
| Uroc1 | 0.00145 | -1.22376446 |
| Mrgprf | 0.00144 | -1.30447385 |
| Gtf2a1l | 0.53953 | -0.47414291 |
| 9130230L23Rik | 0.07604 | -0.90206792 |
| Akna | 0.20854 | -0.27326918 |
| Snx15 | 0.21174 | -0.4526777 |
| Xylt1 | 0.00412 | -1.01275811 |
| Klf11 | 0.43132 | 0.2035481 |
| A630089N07Rik | 0.5909 | 0.61919229 |
| Cd5l | 0.00144 | 1.60388253 |
| Ube3b | 0.0071 | -0.89756732 |
| Ndufb11 | 0.00308 | -1.08213604 |
| Creld1 | 0.23945 | -0.38058732 |
| Otor | 0.07879 | -2.61096334 |
| Mtmr6 | 0.00353 | 1.05735824 |
| 1700040L02Rik | 0.16205 | 0.41924244 |
| Uox | 0.00144 | -1.29644839 |
| Tnfrsf8 | 0.34057 | -0.46618313 |
| Pcsk4 | 0.53977 | -0.10931247 |
| Kif20b | 0.38363 | 0.37809286 |
| Klhl30 | 0.31058 | -0.42645546 |
| Olfr1038-ps | 0.00144 | -1.31303925 |
| Mlf1 | 0.00144 | 1.20718425 |
| Fgf3 | 0.00159 | -1.26431393 |
| Olfr76 | 0.00145 | -1.24191372 |
| Krtap13 | 0.00182 | -1.14330452 |
| Bag2 | 0.40057 | 0.3442433 |
| Cd34 | 0.01023 | -0.58431096 |
| Cep19 | 0.24085 | 0.20958533 |
| Olfr493 | 0.00144 | -1.40054462 |
| Thg1l | 0.06348 | -0.54683661 |
| Zfp827 | 0.01833 | -0.55202319 |
| Exoc3l4 | 0.00516 | -0.85076337 |
| Phf19 | 0.02471 | -0.78598518 |
| Shcbp1 | 0.0028 | 1.69078899 |
| Sec61g | 0.01066 | -0.63381498 |
| Tmem63c | 0.7606 | 0.07863896 |
| Krt33a | 0.00447 | -1.07435095 |
| Emc7 | 0.00144 | 2.8054986 |
| Efhb | 0.04798 | -0.69432979 |
| C8a | 0.00144 | -1.28116048 |
| Palb2 | 0.02477 | -1.25055601 |
| Olfr1442 | 0.00144 | -1.21868187 |
| Hk2 | 0.98914 | -0.00307777 |
| Hba-x | 0.00145 | -1.22376446 |
| Hdac9 | 0.01894 | -0.70184484 |
| L3mbtl4 | 0.01277 | -1.40009704 |
| Gbp2 | 0.12919 | 0.63970956 |
| 4930539H15Rik | 0.54346 | -0.63843428 |
| Cdrt4 | 0.32664 | -0.6743612 |
| Chchd6 | 0.00258 | -1.10173515 |
| Taf8 | 0.00823 | -0.65899369 |
| Trem3 | 0.00144 | -1.36769783 |
| Atp8b5 | 0.12248 | -0.97533314 |
| Slc18a1 | 0.15514 | -0.84051778 |
| Pnmal1 | 0.62448 | 0.5842617 |
| Vars | 0.01262 | -0.71275776 |
| Tsg101 | 0.13193 | 0.60041858 |
| Ccnt2 | 0.00144 | 2.32880795 |
| Olfr1053 | 0.00157 | -1.19247859 |
| Rbm10 | 0.74173 | 0.05598306 |
| Vmn1r216 | 0.027 | -1.15543933 |
| Vmn1r66 | 0.01061 | -1.79881118 |
| Mbd4 | 0.48287 | 0.16084613 |
| Apobr | 0.61603 | -0.10849017 |
| Foxh1 | 0.00549 | -0.71216103 |
| Cecr6 | 0.00144 | -1.29746368 |
| Csnk2a2 | 0.59538 | 0.10145659 |
| Wbscr17 | 0.02476 | -0.80610421 |
| Olfr398 | 0.00144 | -1.5154554 |
| Abcb9 | 0.00465 | -1.14994538 |
| Zc3h13 | 0.14672 | 0.45450721 |
| Ctnnal1 | 0.89579 | -0.03534489 |
| Ebi3 | 0.10918 | -0.33259711 |
| Mterf2 | 0.00337 | 0.84140505 |
| Sh3tc2 | 0.01014 | -0.54734337 |
| Kprp | 0.00315 | -1.47751319 |
| Sar1a | 0.02024 | -0.52294452 |
| Rarb | 0.72818 | 0.0711709 |
| Pdap1 | 0.84803 | -0.03414373 |
| Ydjc | 0.00144 | -1.31884045 |
| 1700104B16Rik | 0.00144 | -1.28116048 |
| Ncapd2 | 0.05554 | -0.46462782 |
| Dtx1 | 0.00158 | -1.12661271 |
| Wls | 0.28179 | 0.27011086 |
| Taf12 | 0.64823 | 0.11635889 |
| Cfap97 | 0.00144 | 1.68116085 |
| Dctn5 | 0.27222 | 0.18453977 |
| Morn5 | 0.85609 | -0.11398579 |
| Tubgcp3 | 0.00414 | 0.69882916 |
| Olfr870 | 0.29055 | -0.44896825 |
| Crtap | 0.21911 | -0.27439588 |
| 4930522N08Rik | 0.15251 | -0.87819776 |
| LOC102643054 | 0.00158 | -1.2446827 |
| Xrcc5 | 0.50577 | 0.12772909 |
| Unc119 | 0.14073 | -0.55464303 |
| Rnf26 | 0.00323 | -0.87233151 |
| Rln3 | 0.06416 | -1.68927208 |
| Nup85 | 0.01471 | -0.55268263 |
| Olfr242 | 0.00144 | -1.29746368 |
| Stat3 | 0.00144 | -1.0441023 |
| Hrct1 | 0.03618 | -1.28028691 |
| Olfr912 | 0.00144 | -1.26560808 |
| Mapkapk5 | 0.99299 | 0.00224836 |
| Moxd2 | 0.01347 | -1.01042984 |
| Olfr1054 | 0.05989 | -0.7911528 |
| Klhl15 | 0.03714 | 0.60094636 |
| Larp1b | 0.00144 | 1.04369974 |
| Lfng | 0.11541 | -0.9658684 |
| Lipf | 0.31074 | -0.35404032 |
| Pnpla8 | 0.00479 | 0.97134592 |
| Cenpi | 0.21287 | -0.41131778 |
| C230071I02Rik | 0.00225 | 3.24479568 |
| Gm35725 | 0.00144 | -1.28116048 |
| 4931417E11Rik | 0.03227 | -1.90441487 |
| Pomp | 0.01175 | -0.55745207 |
| Foxo4 | 0.72127 | 0.09582915 |
| Prdx4 | 0.0374 | -0.4125887 |
| Grsf1 | 0.02207 | 0.79437051 |
| Wdhd1 | 0.00144 | -1.25118919 |
| Zbtb12 | 0.01165 | -0.7536079 |
| Tcp11x2 | 0.00356 | -0.93369864 |
| Shtn1 | 0.88289 | 0.04334819 |
| Rasgef1a | 0.0538 | 0.64993898 |
| Klk1b1 | 0.00286 | -1.17314862 |
| Lrp12 | 0.13294 | -0.33946824 |
| Cachd1 | 0.23513 | -0.21506034 |
| Olfr945 | 0.3302 | -0.39984382 |
| Neurod4 | 0.00144 | -1.32599123 |
| Pak1ip1 | 0.01013 | 0.57574052 |
| Snrpg | 0.0429 | -0.38130546 |
| 4930444G20Rik | 0.0047 | -1.62656859 |
| Grina | 0.41638 | -0.30388262 |
| Fto | 0.98334 | -0.00718426 |
| Dock7 | 0.52284 | 0.3794053 |
| Pin1 | 0.25161 | -0.51322904 |
| 9330171B17Rik | 0.04698 | -0.91567309 |
| Arhgap24 | 0.04933 | 0.61900831 |
| Sfswap | 0.36995 | -0.21096114 |
| Med26 | 0.60277 | -0.13135949 |
| Cacna2d4 | 0.00144 | -1.28898079 |
| Siglecf | 0.00144 | -1.29746368 |
| Olfr744 | 0.00767 | -0.61526471 |
| Elmo2 | 0.42161 | 0.28037453 |
| Lamc1 | 0.93103 | -0.01429804 |
| Olfr272 | 0.00144 | -1.22925777 |
| Pik3ap1 | 0.0225 | -0.99954352 |
| Fam69a | 0.0134 | 1.01132279 |
| 2700038G22Rik | 0.00627 | 1.39655936 |
| Cidea | 0.00144 | -1.21696355 |
| Cd83 | 0.062 | -0.47169488 |
| Olfr1256 | 0.00144 | -1.26301121 |
| Tmem107 | 0.00508 | -0.89500163 |
| Lypd5 | 0.25676 | -0.58435668 |
| A330027G23Rik | 0.00144 | -1.29085005 |
| Zfp108 | 0.06583 | 0.32650406 |
| BC055324 | 0.90784 | -0.02467214 |
| Gckr | 0.04443 | -1.14985379 |
| Ttc36 | 0.00144 | -1.28898079 |
| Parvb | 0.16529 | -0.46958558 |
| Thap7 | 0.00144 | -1.24740707 |
| Obox6 | 0.00145 | -1.12248092 |
| H2-K1 | 0.00393 | -1.07612778 |
| Grb2 | 0.00408 | -0.73046447 |
| Gabra6 | 0.05772 | -0.84899822 |
| Ap4s1 | 0.38249 | 0.19156769 |
| Neurl3 | 0.18723 | -0.37386242 |
| Mien1 | 0.23331 | -0.52468527 |
| Eppin | 0.00144 | -1.27165604 |
| Rab28 | 0.02625 | 0.4797282 |
| Zfp57 | 0.01197 | 1.72146944 |
| Ddx60 | 0.60315 | -0.52249413 |
| Exo5 | 0.2522 | 0.27841159 |
| Cacnb4 | 0.37023 | -1.59032792 |
| Ltv1 | 0.01084 | 1.10134092 |
| Parp6 | 0.10148 | 0.31731226 |
| Nkx2-2 | 0.09749 | -2.66555715 |
| 5730407I07Rik | 0.3754 | -0.22395686 |
| Tekt5 | 0.24379 | -0.48557893 |
| Atad5 | 0.98681 | -0.0064778 |
| Afap1l2 | 0.0366 | -0.57338211 |
| Ly6c2 | 0.00989 | -0.67500863 |
| Clta | 0.32012 | -0.21614138 |
| Olfr393 | 0.00232 | -1.15063496 |
| Cct7 | 0.04275 | -0.56281862 |
| Pigk | 0.82395 | 0.08046496 |
| Rnase2b | 0.00158 | -1.17515383 |
| Tktl2 | 0.1263 | -2.94003547 |
| Soat2 | 0.00144 | -1.25118919 |
| Chaf1a | 0.10122 | -0.40735821 |
| Slc4a1 | 0.05472 | -1.11462195 |
| Cx3cl1 | 0.00144 | -1.18636866 |
| Ist1 | 0.10793 | 0.30531245 |
| Ireb2 | 0.00144 | 1.56124119 |
| Amdhd1 | 0.09091 | -1.28873383 |
| Prr16 | 0.00144 | -1.24865146 |
| Itih2 | 0.08605 | -1.97029589 |
| Adcy1 | 0.00601 | 0.79319993 |
| Khdc1b | 0.00144 | -1.31495282 |
| Fam102b | 0.42108 | 0.1706301 |
| Hadh | 0.00144 | -1.25712448 |
| Olfr920 | 0.31211 | -0.54060996 |
| Bscl2 | 0.11501 | -0.50341076 |
| Plekhj1 | 0.01874 | -0.6603934 |
| Foxc2 | 0.15276 | -0.38646378 |
| 1110004E09Rik | 0.41611 | 0.2029356 |
| Sncaip | 0.00457 | 0.97257903 |
| Vwc2l | 0.00144 | -1.27165604 |
| Btd | 0.00369 | -0.90592948 |
| Atf7ip2 | 0.00149 | -1.19696847 |
| Papss1 | 0.10258 | -0.27255966 |
| Mast1 | 0.07644 | -0.43306934 |
| Trpc1 | 0.2677 | 0.40358123 |
| Phlda1 | 0.08862 | -0.47671903 |
| Neil3 | 0.00144 | -1.28116048 |
| Ercc2 | 0.16357 | -0.36387884 |
| Olfr1079 | 0.00144 | -1.28898079 |
| Zfp330 | 0.00566 | 0.70697251 |
| Mtdh | 0.02574 | -0.68021794 |
| Olfr259 | 0.00222 | -1.1481805 |
| Adnp2 | 0.5678 | -0.09343713 |
| Dnmt1 | 0.00651 | -0.67625731 |
| 1700017D01Rik | 0.00144 | -1.28116048 |
| Csf1 | 0.25492 | -0.23209916 |
| Ube2q2 | 0.00769 | 0.82019774 |
| Baiap2l1 | 0.09654 | 0.65624281 |
| Gpatch11 | 0.00186 | 1.0268928 |
| Fam149a | 0.02578 | -0.42025798 |
| Arl13a | 0.00166 | -1.21062785 |
| Gtse1 | 0.00144 | -1.36433367 |
| Spin1 | 0.01178 | 0.56762792 |
| Shroom3 | 0.27919 | 0.4858315 |
| Vps4b | 0.00144 | 1.6614962 |
| Dppa3 | 0.13197 | -2.60866298 |
| Esco2 | 0.0507 | -0.88511728 |
| 4933408B17Rik | 0.32393 | 0.38155247 |
| Sec14l4 | 0.30102 | -0.35296456 |
| Il13 | 0.02828 | -1.09082921 |
| Anapc10 | 0.00144 | 1.26440225 |
| Rint1 | 0.00582 | 0.65091961 |
| Vmn1r172 | 0.00145 | -1.25646236 |
| Tusc3 | 0.03579 | 0.6055236 |
| Pcid2 | 0.44382 | 0.21742209 |
| Gk2 | 0.03031 | -1.02137678 |
| Prss34 | 0.10905 | -0.40650601 |
| Alx3 | 0.0057 | -0.66837935 |
| Olfr433 | 0.00144 | -1.55463795 |
| Wfdc1 | 0.89982 | 0.05530753 |
| 1110059G10Rik | 0.02776 | -0.5679006 |
| Slco3a1 | 0.13754 | -0.31172217 |
| Pcbp4 | 0.00267 | -0.95568055 |
| 1700010B08Rik | 0.00144 | -1.28116048 |
| Lrrc1 | 0.03528 | 0.51142578 |
| Pate4 | 0.00144 | -1.28898079 |
| Sgcg | 0.29989 | 0.38579621 |
| Zic1 | 0.00149 | -1.22582153 |
| Zbtb49 | 0.45522 | -0.14965516 |
| Ttr | 0.00564 | -1.8433998 |
| Thrsp | 0.95641 | 0.00982426 |
| Tbx10 | 0.00415 | -1.4501028 |
| Gtf3c4 | 0.02537 | -0.51946403 |
| Prpf4 | 0.78135 | 0.04562667 |
| Rtkn2 | 0.18293 | -1.8162492 |
| Arr3 | 0.00259 | -1.20572989 |
| Fga | 0.01102 | -1.13684067 |
| Lrp1 | 0.00462 | -0.71984361 |
| Nlrc3 | 0.15584 | 0.40968505 |
| Supv3l1 | 0.35379 | 0.63566414 |
| 1700012B09Rik | 0.56035 | -0.25457847 |
| 1700012B09Rik | 0.00144 | -1.29049509 |
| Cdk7 | 0.01734 | 0.72578709 |
| Ccs | 0.15751 | -0.4388188 |
| Cdk5rap1 | 0.07638 | 0.87889651 |
| Fbxo21 | 0.00295 | -0.99779545 |
| Ccdc134 | 0.74864 | 0.05401799 |
| Nucb2 | 0.00169 | 1.50191256 |
| Iqcf1 | 0.00999 | -0.77449829 |
| Nelfe | 0.00144 | -1.44322926 |
| Rbm46os | 0.14191 | -0.62218389 |
| Slc26a8 | 0.00144 | -1.50634882 |
| Mb | 0.00326 | -0.76319069 |
| Slc25a25 | 0.16596 | -0.36931573 |
| Slc25a25 | 0.06702 | -0.4998725 |
| Ms4a6c | 0.88779 | 0.03479629 |
| Mrpl23 | 0.00144 | -1.18286667 |
| Dclk1 | 0.01762 | 1.36666462 |
| Bbs4 | 0.00179 | 1.1096269 |
| Dcun1d3 | 0.99922 | 0.00017334 |
| Mgat4e | 0.00149 | -1.22582153 |
| Hint1 | 0.00983 | -0.86944192 |
| Ghdc | 0.18792 | -0.29460222 |
| Mrpl14 | 0.00197 | -1.18207943 |
| Olfr1495 | 0.00144 | -1.36353418 |
| Pno1 | 0.01796 | 0.51923393 |
| Srcap | 0.00145 | -1.2388121 |
| Spata5 | 0.24683 | 0.18861293 |
| Scube2 | 0.33256 | 0.46193447 |
| Gm32014 | 0.00144 | -1.27165604 |
| Olfr591 | 0.00144 | -1.28898079 |
| Dido1 | 0.73892 | -0.14839015 |
| Diap2 | 0.12406 | 0.65240902 |
| Zfp74 | 0.08117 | 0.43079703 |
| Stk10 | 0.89891 | 0.0221858 |
| Setdb2 | 0.00144 | 1.83267663 |
| Pinx1 | 0.00144 | 3.58907415 |
| Myh1 | 0.0927 | -1.56681324 |
| Rem2 | 0.02394 | -3.17016316 |
| 2410022M11Rik | 0.00454 | -1.13119243 |
| Efcab6 | 0.00144 | -1.28898079 |
| Col18a1 | 0.00144 | -1.07222959 |
| Chgb | 0.0024 | 1.20268873 |
| Ndc80 | 0.87467 | 0.0580162 |
| 2510039O18Rik | 0.01111 | -0.77775815 |
| Arpc1a | 0.07984 | -0.35550122 |
| Olfr411 | 0.12416 | -1.31986748 |
| Gm5893 | 0.00144 | -1.25118919 |
| Stard10 | 0.29047 | -0.72815914 |
| Rnf31 | 0.00223 | -0.80506811 |
| H2-M2 | 0.00158 | -1.02176887 |
| Nmd3 | 0.00144 | -1.30017714 |
| Acot6 | 0.00454 | 0.96535414 |
| Sipa1 | 0.02206 | -0.54949919 |
| 4930405H06Rik | 0.00177 | -1.15847607 |
| Hibch | 0.12125 | 1.01716989 |
| Jsrp1 | 0.00144 | -1.39796783 |
| Gm36736 | 0.00144 | -1.35619208 |
| Rsph14 | 0.04239 | -0.79272898 |
| Pik3c2g | 0.80937 | -0.12168312 |
| Agfg1 | 0.0318 | 0.49763451 |
| Olfr1166 | 0.16682 | -0.67277555 |
| Gzmf | 0.00144 | -1.32599123 |
| Ankrd13a | 0.22953 | -0.20490828 |
| BB283564 | 0.45271 | 0.76750002 |
| Ap1m1 | 0.43128 | -0.19719023 |
| Dcp2 | 0.00301 | -0.8440762 |
| Abca14 | 0.00144 | -1.31518407 |
| Atp8b4 | 0.13639 | -0.65254168 |
| Wfdc5 | 0.00144 | -1.27165604 |
| Ccdc181 | 0.33325 | 0.25240558 |
| Klhl13 | 0.08172 | -0.81320339 |
| Orc2 | 0.00558 | 1.40764178 |
| Scrn1 | 0.10449 | 0.31005989 |
| Pnma1 | 0.00145 | -1.2388121 |
| Med23 | 0.46146 | -0.12942782 |
| Mcm5 | 0.00967 | -0.73824249 |
| Ppan | 0.00392 | -0.76162278 |
| Dscam | 0.35393 | -0.38574658 |
| Nol12 | 0.0044 | -0.70758688 |
| Dad1 | 0.07469 | -0.59628658 |
| Olfr1134 | 0.0117 | -0.71947645 |
| Ears2 | 0.41605 | 0.18376211 |
| Cldn5 | 0.02029 | -0.95567161 |
| Ccrn4l | 0.00668 | 1.54102646 |
| Pim3 | 0.31735 | -0.21798046 |
| Sult2a8 | 0.00152 | 1.14868575 |
| Cabin1 | 0.01739 | -0.49727215 |
| Ankrd61 | 0.08311 | 0.66675486 |
| 2310067E19Rik | 0.04595 | 0.88297113 |
| Folh1 | 0.00144 | -1.29085005 |
| Snrpb | 0.2677 | -0.34819978 |
| Klrk1 | 0.00761 | -1.73246858 |
| Adh4 | 0.00778 | -1.27223093 |
| Podxl2 | 0.22619 | -0.51979189 |
| Osgin1 | 0.00149 | -0.97114397 |
| Tmcc3 | 0.06212 | 0.46789186 |
| Rnf121 | 0.00583 | -0.73716947 |
| 4933417G07Rik | 0.72253 | 0.16188499 |
| Kbtbd2 | 0.27169 | -0.20797505 |
| Atp2c2 | 0.00144 | -1.48396829 |
| Gm15674 | 0.01545 | -1.19703824 |
| Akr1c6 | 0.13381 | 0.46995309 |
| Ppp1r27 | 0.08646 | -0.56655114 |
| Grm1 | 0.002 | -1.19650662 |
| Dhrs7c | 0.16087 | -0.34155443 |
| Galnt12 | 0.2914 | -0.20843156 |
| Tbc1d31 | 0.0692 | -1.09836216 |
| Adora1 | 0.05993 | -0.36286282 |
| LOC102639145 | 0.00144 | -1.31884045 |
| Akr7a5 | 0.0569 | -0.90221961 |
| Ube2s | 0.01647 | -0.78524559 |
| Arhgef28 | 0.97258 | 0.00739776 |
| Zfp191 | 0.04715 | 0.49034837 |
| Unc13a | 0.03287 | -0.58376785 |
| Tnfaip8l3 | 0.14741 | -2.87494705 |
| Myf5 | 0.00275 | -1.3673208 |
| Cd248 | 0.00144 | -1.33972729 |
| Gpr179 | 0.10722 | -1.01182033 |
| Max | 0.111 | 0.29474805 |
| Zfp617 | 0.00144 | 1.52806424 |
| Fbxo7 | 0.02487 | -0.45209767 |
| Gca | 0.02018 | 1.06368315 |
| Dnajb11 | 0.50242 | 0.12039672 |
| 4930549C01Rik | 0.56012 | -0.32309875 |
| Slc39a9 | 0.09687 | 0.54091051 |
| Tmem132d | 0.00144 | -1.28116048 |
| Nop56 | 0.02771 | -0.42653164 |
| H60a | 0.32794 | -0.49717353 |
| Eif4e3 | 0.00725 | 0.84150561 |
| Olfr460 | 0.02671 | -1.00277612 |
| 4933436I01Rik | 0.00144 | -1.26934956 |
| Nprl2 | 0.89939 | -0.06248564 |
| Neurl3 | 0.96112 | -0.00846293 |
| Zc3h11a | 0.01038 | 0.76582427 |
| Mgme1 | 0.3063 | -0.15665952 |
| Slc9b1 | 0.01118 | 1.38781933 |
| Cabp2 | 0.05575 | -0.98829873 |
| Serpinb5 | 0.00144 | -1.29746368 |
| Gm31711 | 0.48537 | 0.3891524 |
| Six3 | 0.12453 | -2.0653291 |
| Acad10 | 0.07793 | -0.34232719 |
| Kcnma1 | 0.07524 | -2.45718629 |
| Sgms2 | 0.24271 | 0.48973755 |
| Mmp25 | 0.00144 | -1.29085005 |
| Hrh2 | 0.00158 | -1.2446827 |
| 1500015L24Rik | 0.05709 | -0.86363514 |
| Smim12 | 0.00416 | -0.96904392 |
| G6pdx | 0.02659 | -0.45946902 |
| Magohb | 0.20423 | 0.31495861 |
| Adam2 | 0.29716 | -0.83158823 |
| Olfr520 | 0.03603 | -1.92667811 |
| Krt5 | 0.25003 | -1.61043272 |
| Tesk2 | 0.81253 | 0.07995509 |
| Fbln5 | 0.0364 | -0.43962368 |
| Pah | 0.05995 | -0.86068167 |
| Tbrg1 | 0.59553 | 0.11390333 |
| Sebox | 0.32494 | -0.32781738 |
| Pik3c2b | 0.55165 | -0.29592532 |
| Slc11a1 | 0.51852 | 0.38749493 |
| Atg4c | 0.60745 | -0.19321348 |
| Efna3 | 0.00144 | -1.28116048 |
| Rnf149 | 0.05575 | 0.40511194 |
| Rrp15 | 0.14744 | 0.25235055 |
| Calm2 | 0.00833 | 0.85057925 |
| Shoc2 | 0.47318 | -0.13447476 |
| Abi3 | 0.00908 | -0.67821605 |
| Mkrn1 | 0.6812 | 0.06955574 |
| Hbq1a | 0.50834 | -0.61350726 |
| Dnal4 | 0.01106 | -0.81231199 |
| Gm8179 | 0.89214 | -0.15567059 |
| Slc46a2 | 0.49813 | -0.26035232 |
| Ptdss2 | 0.27504 | -0.33177212 |
| Mmp1a | 0.00144 | -1.28116048 |
| Ccl9 | 0.6709 | -0.08268392 |
| Ankdd1b | 0.00145 | -1.22376446 |
| Adamtsl4 | 0.71848 | -0.08294962 |
| Zfp935 | 0.0023 | 2.37246088 |
| Prkar1b | 0.02771 | -1.53998216 |
| Slc29a3 | 0.00273 | -1.07037255 |
| Vps13b | 0.00144 | -1.29978795 |
| Il5 | 0.04139 | -0.887803 |
| 4930581F22Rik | 0.20562 | 0.67901809 |
| Gnb5 | 0.76964 | 0.12783747 |
| Fkbp4 | 0.21385 | -0.43257367 |
| Myo1e | 0.1725 | 0.30310814 |
| Gpr108 | 0.03031 | -0.5378712 |
| Tsga13 | 0.00144 | -1.39629016 |
| Dpy30 | 0.54788 | -0.18607729 |
| Wdr64 | 0.483 | -0.33906976 |
| Rilpl1 | 0.30421 | 0.35187296 |
| F12 | 0.00144 | -1.29864962 |
| Ifitm7 | 0.00245 | -0.83656866 |
| Phpt1 | 0.01333 | -0.67646267 |
| Pfkfb2 | 0.0043 | 0.89290627 |
| Zfp101 | 0.00203 | 2.3469708 |
| Cnksr3 | 0.55737 | -0.1732187 |
| Olfr675 | 0.00145 | -1.20627532 |
| Mmp13 | 0.00192 | 1.20716279 |
| Il17rd | 0.44227 | 0.23126262 |
| Olfr1340 | 0.02074 | -2.0311688 |
| Ttbk2 | 0.00184 | -0.95615441 |
| Pla2g12b | 0.0019 | -1.13608632 |
| Nr2f6 | 0.01086 | -0.9224287 |
| Aimp1 | 0.95415 | -0.00944254 |
| Dld | 0.00149 | 1.90878402 |
| Pcdhb7 | 0.29112 | 0.27938455 |
| Cr1l | 0.06899 | 0.35853526 |
| Smarcd2 | 0.00656 | -0.71280105 |
| Cfc1 | 0.09978 | -0.35792919 |
| Ccdc183 | 0.00145 | -1.42017585 |
| Hbs1l | 0.23835 | 0.23230769 |
| Tsen15 | 0.64664 | 0.10108024 |
| Paf1 | 0.00682 | 0.61496775 |
| Barhl1 | 0.15856 | -0.25371932 |
| Stk39 | 0.2197 | 0.22154114 |
| Fbxo15 | 0.00145 | -1.22193674 |
| Fbxo15 | 0.00717 | -1.0591794 |
| Polr2m | 0.99819 | 0.00044326 |
| Slfn4 | 0.00144 | -1.29085005 |
| Prrx2 | 0.62965 | -0.18246369 |
| Pkhd1l1 | 0.00144 | -1.30713006 |
| Pdpr | 0.14247 | 0.31802124 |
| Bcor | 0.78529 | -0.07523319 |
| Pou2af1 | 0.10893 | 0.39218498 |
| Csdc2 | 0.2243 | -0.4771425 |
| Htr6 | 0.00144 | -1.29085005 |
| Olfr295 | 0.00171 | -1.08480094 |
| Desi2 | 0.2808 | -0.17865128 |
| Tmem74bos | 0.01041 | -1.5623083 |
| Atp5g1 | 0.19132 | -0.59987187 |
| Aff2 | 0.05631 | -2.67369332 |
| Sympk | 0.00254 | -0.82080829 |
| Tssk3 | 0.00144 | -1.20555712 |
| Fancd2 | 0.00144 | -1.33632959 |
| Pcif1 | 0.01528 | -0.61553653 |
| Hook1 | 0.08023 | 0.72588295 |
| Olfr531 | 0.12603 | -0.83178373 |
| Rnf139 | 0.0123 | 0.84365 |
| Upb1 | 0.03171 | 0.68621317 |
| Efcc1 | 0.12313 | -2.57506527 |
| Gm11100 | 0.0479 | -1.40106479 |
| Stx1a | 0.57641 | 0.15656622 |
| Npr1 | 0.25896 | -0.56642177 |
| Vmn1r230 | 0.00144 | -1.29085005 |
| Mug1 | 0.00506 | -1.0591794 |
| Phactr1 | 0.00144 | -0.99329449 |
| Heatr3 | 0.3185 | 0.2379412 |
| Pcgf3 | 0.08922 | 0.70844993 |
| Eif5b | 0.03754 | 0.47818127 |
| Cecr5 | 0.37949 | 0.42573249 |
| Clec2i | 0.42006 | -0.32181002 |
| Col1a2 | 0.0125 | -0.58307629 |
| Ccdc53 | 0.75174 | -0.0943463 |
| Tex40 | 0.01886 | -0.75680075 |
| Ndufa13 | 0.00643 | -0.81495236 |
| Amer2 | 0.00144 | -1.29085005 |
| Eml3 | 0.03308 | -0.44800071 |
| Ptpre | 0.95499 | -0.00977535 |
| Zfyve1 | 0.53396 | 0.14155712 |
| Rbks | 0.77721 | -0.05284982 |
| Nod2 | 0.30478 | 0.23170084 |
| Zfp428 | 0.00156 | -0.99432792 |
| 4933402N22Rik | 0.01843 | 0.9493323 |
| Hbegf | 0.30762 | -0.31091917 |
| Brwd1 | 0.00569 | 0.88121018 |
| Tmem214 | 0.01678 | -0.69854185 |
| Fcgr4 | 0.17496 | -0.36020563 |
| C1qa | 0.14092 | -0.46798465 |
| Olfr807 | 0.00144 | -1.31627578 |
| Usmg5 | 0.1723 | -0.43198389 |
| Necab2 | 0.62041 | -0.31421354 |
| Dcxr | 0.17992 | -0.45071637 |
| Cd69 | 0.01344 | -1.32064399 |
| Trim54 | 0.00748 | -0.67692761 |
| E230016M11Rik | 0.21381 | 0.67068246 |
| Ccdc132 | 0.00144 | 1.76991484 |
| Lmbrd2 | 0.00145 | 1.36069994 |
| Pcsk6 | 0.14228 | -0.32132593 |
| Olfr1087 | 0.00144 | -1.28898079 |
| Map2 | 0.98582 | -0.00880195 |
| Mlh1 | 0.96742 | -0.00694177 |
| Paox | 0.00144 | -1.03475606 |
| Dbp | 0.00708 | -0.71828265 |
| Cux2 | 0.00332 | -0.74995392 |
| Scarb2 | 0.93026 | 0.0300481 |
| Gm16223 | 0.04061 | -0.82286232 |
| Hist1h2ba | 0.80269 | 0.09932676 |
| Shfm1 | 0.01385 | -0.56724472 |
| Ago2 | 0.00144 | -1.22679371 |
| Igdcc3 | 0.11948 | -0.28397448 |
| Appl2 | 0.00144 | 1.96378315 |
| Rfx7 | 0.0446 | -0.70669403 |
| Prkx | 0.0041 | 0.80706296 |
| Ppm1f | 0.10873 | -1.81660917 |
| Hlx | 0.00674 | -1.25617689 |
| Fam57b | 0.10106 | -0.33986063 |
| Gbx2 | 0.88795 | -0.05160604 |
| 2010204K13Rik | 0.03382 | -0.46432124 |
| Flt4 | 0.17477 | -0.32020859 |
| Cgnl1 | 0.45256 | -0.83819106 |
| Fam92a | 0.00144 | 1.53989606 |
| 4930539E08Rik | 0.00261 | -1.69986771 |
| Kif26b | 0.68727 | 0.26833488 |
| Tssc4 | 0.00144 | -0.96035337 |
| 1700080E11Rik | 0.04343 | -1.39211709 |
| 1700080E11Rik | 0.12562 | -0.81981054 |
| Vasp | 0.00623 | -0.72987584 |
| Top3b | 0.02849 | -0.47610308 |
| Tead4 | 0.41346 | -0.15059906 |
| Ttc9c | 0.00222 | -0.92277615 |
| Mbl2 | 0.00767 | -1.02946836 |
| H1foo | 0.81991 | -0.07411552 |
| Ces1f | 0.00144 | -1.29864962 |
| Fau | 0.00144 | -1.16418038 |
| Hibch | 0.00206 | 0.99910917 |
| Olfr1093 | 0.24782 | -0.52221195 |
| Mrpl1 | 0.0065 | 0.67157151 |
| Gm21464 | 0.50769 | -0.30844754 |
| Iqce | 0.0958 | -0.76505373 |
| Cep104 | 0.00158 | -0.94306249 |
| Ephx3 | 0.00191 | -1.05147592 |
| Mamld1 | 0.93062 | 0.03469579 |
| Itfg3 | 0.06565 | -0.61099418 |
| Trappc10 | 0.00145 | -1.22376446 |
| Gabrr1 | 0.0729 | 0.57119503 |
| Puf60 | 0.00713 | -0.69515528 |
| Ndufb9 | 0.00151 | -1.04361654 |
| Ctrl | 0.2466 | -1.04530038 |
| Prnd | 0.03758 | 0.6419206 |
| Tas2r105 | 0.00144 | -1.27165604 |
| Slc6a20b | 0.00144 | -1.29049509 |
| Stac3 | 0.34585 | 0.2455103 |
| Myl3 | 0.00148 | -0.92824196 |
| Rdh19 | 0.17351 | -0.58622266 |
| Tie1 | 0.00604 | -0.81385028 |
| Utp18 | 0.00144 | -1.29864962 |
| Stat4 | 0.0933 | 1.51769561 |
| Nrg3 | 0.00144 | -1.29085005 |
| Nup205 | 0.24414 | -0.31852586 |
| Olfr143 | 0.00144 | -1.28375735 |
| Prdm2 | 0.03216 | 1.04147773 |
| Amigo2 | 0.07345 | -0.54990711 |
| Mdm4 | 0.27481 | 0.33704254 |
| Tlr8 | 0.00208 | -1.15498993 |
| Edrf1 | 0.00416 | 0.98381067 |
| Tspan10 | 0.06975 | -1.05893713 |
| Atxn7l3b | 0.17926 | -0.22435426 |
| Lpar5 | 0.49503 | -0.82895847 |
| 4930483J18Rik | 0.18749 | -0.77904961 |
| Olfr1133 | 0.00145 | -1.2388121 |
| 1700109H08Rik | 0.00306 | 1.04345264 |
| Ntmt1 | 0.05471 | -0.51784829 |
| Rps19bp1 | 0.05278 | -0.52489351 |
| Kndc1 | 0.00144 | -1.26934956 |
| Kat6b | 0.01381 | 0.62981864 |
| Txnl1 | 0.32168 | 0.18138023 |
| Usp25 | 0.00242 | 0.87777007 |
| Gimap5 | 0.00436 | -0.69765106 |
| Ndrg2 | 0.28734 | -0.25628449 |
| Syvn1 | 0.04941 | -0.50255369 |
| Trip11 | 0.01298 | 1.3111116 |
| Ankrd36 | 0.05345 | -0.73135572 |
| Gak | 0.07791 | -0.33487784 |
| Fam195b | 0.00144 | -1.30563248 |
| Ffar2 | 0.00341 | -1.57829302 |
| Cmtr2 | 0.44714 | 0.17501069 |
| Inpp5f | 0.00368 | 2.0527679 |
| Pklr | 0.00144 | -1.39713491 |
| Vps13d | 0.225 | 0.2228507 |
| Tmem74 | 0.97734 | 0.04009808 |
| Got2 | 0.19571 | -0.43611795 |
| Cyb5r2 | 0.00589 | -1.20973681 |
| Neurog3 | 0.00144 | -1.85408081 |
| Flii | 0.00488 | -0.83901243 |
| Atg101 | 0.00182 | -0.98955801 |
| Mtfp1 | 0.01878 | -0.84574003 |
| Lpo | 0.30424 | -0.51027184 |
| 4930578C19Rik | 0.77527 | 0.06593632 |
| Pcmtd1 | 0.06431 | -0.39722364 |
| Trp53inp1 | 0.00144 | 1.88271628 |
| Pelo | 0.00735 | -0.71357735 |
| Olfr843 | 0.00145 | -1.26219879 |
| Grk6 | 0.04174 | -0.46076966 |
| Car14 | 0.1611 | -0.44048452 |
| Zfp146 | 0.29346 | 0.95042256 |
| Fbxl4 | 0.02077 | 0.63157483 |
| Adamts10 | 0.44449 | 0.2162487 |
| Olfr683 | 0.00144 | -1.31518407 |
| F10 | 0.11606 | 0.95454105 |
| Lrp2 | 0.01677 | -0.88042742 |
| Olfr235 | 0.00145 | -1.2388121 |
| Mkl1 | 0.00244 | -0.88746821 |
| Maged1 | 0.0171 | -0.56611067 |
| Pramel1 | 0.2336 | -0.50337576 |
| Sec24a | 0.03202 | 1.50273351 |
| Cd86 | 0.46292 | 0.25367991 |
| Ccdc43 | 0.77436 | 0.07802353 |
| Bzw2 | 0.93276 | 0.02624428 |
| Kcnmb1 | 0.00144 | -1.29051721 |
| Fam60a | 0.21562 | 0.46141267 |
| Olfr893 | 0.0017 | -1.19401501 |
| Cenpj | 0.00163 | 1.89041562 |
| Srr | 0.01318 | -0.53716129 |
| Unkl | 0.04905 | 0.4269383 |
| Colec11 | 0.00912 | -0.66230394 |
| Odf2 | 0.0033 | 0.81959857 |
| Dhrs2 | 0.09739 | -2.62964816 |
| Pip | 0.00144 | -1.58053394 |
| Lipe | 0.00144 | -1.33632959 |
| Zdhhc14 | 0.21797 | -0.25014594 |
| Tnik | 0.04354 | 0.50252 |
| Slc39a7 | 0.00193 | -0.86339081 |
| Olfr935 | 0.00153 | -1.24063982 |
| Pdrg1 | 0.08004 | -0.44741972 |
| Mto1 | 0.30302 | 0.39033739 |
| Vmn1r22 | 0.00144 | -1.30713006 |
| Dock11 | 0.00144 | 2.14011654 |
| Gprc5b | 0.8001 | 0.04514517 |
| Lingo4 | 0.93446 | 0.06309649 |
| Atp7a | 0.013 | 0.87180068 |
| Slc10a6 | 0.00903 | 0.98494254 |
| Ptpn11 | 0.12548 | -0.27836839 |
| Bcam | 0.0126 | -0.88529376 |
| 1700009J07Rik | 0.00144 | -1.44112336 |
| Iqca | 0.00144 | -1.27165604 |
| Nim1k | 0.79997 | 0.06035741 |
| Ibtk | 0.86568 | -0.09045426 |
| Tada1 | 0.06857 | 0.34349053 |
| Pik3r5 | 0.00144 | -1.26934956 |
| Nkx2-5 | 0.01362 | -0.91728693 |
| Osbpl2 | 0.16676 | 0.3723313 |
| Gorasp2 | 0.4497 | 0.19802596 |
| 2410024N13Rik | 0.00144 | -1.30713006 |
| Cdhr1 | 0.00144 | -1.24353547 |
| 1810046K07Rik | 0.08063 | -0.90203488 |
| Pttg1ip | 0.90556 | -0.03088613 |
| Eif3l | 0.00602 | -0.65316195 |
| Egr2 | 0.00144 | 3.57406834 |
| Nsmce1 | 0.0264 | -0.62156129 |
| Lyzl1 | 0.0494 | -0.94846032 |
| Cmah | 0.48224 | -0.28057931 |
| Shank1 | 0.14285 | -0.83920241 |
| Cd14 | 0.69799 | -0.09334424 |
| Med19 | 0.13535 | -0.29275235 |
| Memo1 | 0.01236 | 0.62159873 |
| Kcnc4 | 0.00216 | -1.10905491 |
| Gpatch4 | 0.0958 | 0.50225558 |
| C2cd5 | 0.00144 | 1.72673775 |
| Stard3nl | 0.27731 | 0.22510872 |
| Usp2 | 0.80658 | -0.10484521 |
| Palm3 | 0.00717 | -1.0591794 |
| Nit1 | 0.82655 | -0.05279232 |
| Sh2d1a | 0.00144 | -1.30713006 |
| Cxcl12 | 0.01699 | -0.68685446 |
| Gm9534 | 0.025 | -1.02383124 |
| Pak2 | 0.01226 | 0.52079428 |
| Itih4 | 0.01894 | -0.71046705 |
| Ifrd2 | 0.00635 | -0.83746232 |
| Gsdmd | 0.10766 | -0.35125996 |
| Hal | 0.00144 | -1.26560808 |
| Lurap1l | 0.00783 | -0.69430301 |
| Arhgdig | 0.01206 | -1.14278404 |
| Gas6 | 0.00148 | -0.91414338 |
| Olfr653 | 0.00144 | -1.28116048 |
| Apoe | 0.00271 | -0.7911252 |
| Sdhaf4 | 0.61072 | 0.18275203 |
| Palmd | 0.07187 | 0.331362 |
| Bcl11b | 0.03498 | -0.66253078 |
| Cacna2d2 | 0.01258 | -1.12385295 |
| Wnt10a | 0.00144 | -1.24811894 |
| Olfr487 | 0.01886 | -1.59515861 |
| Dnajc27 | 0.2758 | 0.32647535 |
| Cd163l1 | 0.07126 | -1.2680266 |
| Fcho2 | 0.04989 | 0.65577531 |
| Shprh | 0.22466 | -0.45251575 |
| Gli3 | 0.04711 | 0.59257808 |
| Golm1 | 0.00826 | 0.92677371 |
| Fam65c | 0.00144 | -1.27730451 |
| Tmem35 | 0.94156 | 0.02147493 |
| Csf2 | 0.0612 | -1.5739529 |
| Proz | 0.09529 | 0.6932424 |
| Hnrnpm | 0.00381 | 0.90536783 |
| Samhd1 | 0.01933 | 0.49219147 |
| Asb5 | 0.03741 | 0.83716286 |
| LOC102641377 | 0.07346 | 0.51473364 |
| Dpp8 | 0.02386 | 0.48093348 |
| Grwd1 | 0.00486 | -0.90425223 |
| 1700016H13Rik | 0.16768 | -0.5141808 |
| Cspg5 | 0.04977 | -1.50686642 |
| Haus1 | 0.64614 | 0.07294695 |
| Sema4c | 0.07196 | -0.39431736 |
| Ddx54 | 0.0148 | -0.59566642 |
| Dock10 | 0.08815 | -0.84051778 |
| 4933411G11Rik | 0.00144 | -1.30017714 |
| Mycbpap | 0.02162 | 0.53709538 |
| Tbc1d9 | 0.50436 | 0.18923448 |
| Gpr17 | 0.14746 | -2.99916961 |
| Vmn1r148 | 0.00144 | -1.49363712 |
| Apobec3 | 0.28051 | -1.93870515 |
| D530014G21Rik | 0.00144 | -1.33632959 |
| Olfr345 | 0.00158 | -1.2446827 |
| B3gnt8 | 0.00821 | -1.02662795 |
| Ndufa5 | 0.00579 | -0.65853398 |
| Olfr1499 | 0.00144 | -1.29085005 |
| Napg | 0.00144 | 1.28961345 |
| Noc2l | 0.20319 | -0.28963859 |
| Nsmf | 0.07051 | -0.35280167 |
| Zbtb3 | 0.14567 | -0.38632039 |
| Atxn7l1 | 0.17606 | 0.67079571 |
| Tuba1a | 0.00256 | -0.95285251 |
| Hba-a1 | 0.00591 | -1.11849496 |
| Bst2 | 0.00649 | -0.84523276 |
| Reg3b | 0.00144 | -1.29049509 |
| Utp15 | 0.38504 | 0.33921156 |
| Dhx38 | 0.38821 | -0.53642272 |
| Mvk | 0.01133 | -0.64559376 |
| Ppp1r3d | 0.25226 | -0.21958874 |
| Mcpt1 | 0.00144 | -1.28898079 |
| Ccdc89 | 0.69682 | -0.07964624 |
| Parvb | 0.00144 | -1.6729924 |
| Slc52a3 | 0.00144 | -1.35071562 |
| Etd | 0.2143 | -0.56774937 |
| Olfr190 | 0.47059 | 0.54311245 |
| Rps10 | 0.00693 | -0.92682459 |
| Cct3 | 0.04935 | 0.70733426 |
| Gls2 | 0.94682 | 0.01616886 |
| Lpcat2 | 0.54943 | -0.13303243 |
| Tdp2 | 0.00177 | 1.06668377 |
| 2810004N23Rik | 0.77527 | -0.07910691 |
| Cnpy1 | 0.00151 | -1.17792995 |
| Xylb | 0.33069 | 0.4591705 |
| Bnc2 | 0.44925 | -0.34211711 |
| Atp5f1 | 0.17905 | 0.40722322 |
| Mast4 | 0.07534 | 0.5479115 |
| Nefl | 0.0028 | -1.16569658 |
| Prim1 | 0.00514 | 1.06030828 |
| Klf1 | 0.07656 | -1.03275883 |
| Ifnar1 | 0.62661 | -0.0999105 |
| Mob1a | 0.00625 | 0.95689723 |
| Klhl24 | 0.00887 | 0.97773762 |
| Ttc30a1 | 0.87415 | -0.02587251 |
| Ammecr1l | 0.50996 | 0.17117196 |
| Hacd3 | 0.0023 | -1.08234321 |
| Kcnt2 | 0.81828 | 0.08982657 |
| Bace1 | 0.96522 | -0.00963849 |
| Tifab | 0.00144 | -1.31495282 |
| Cnbd2 | 0.00177 | 1.85136598 |
| St5 | 0.00687 | -1.06998228 |
| Atad3aos | 0.0052 | -1.12932173 |
| Fam214a | 0.00144 | 1.66348477 |
| Lum | 0.77303 | 0.08460677 |
| Leng1 | 0.24555 | 0.1969956 |
| Lama3 | 0.00796 | 1.7973066 |
| Gpatch1 | 0.0044 | 0.65671164 |
| Ptpn7 | 0.02261 | -0.45968495 |
| Chil3 | 0.19949 | -0.83124338 |
| Cd5 | 0.00232 | -1.23247196 |
| Cask | 0.04527 | 0.47684735 |
| Olfr1270 | 0.06896 | -2.34149455 |
| Prdm11 | 0.00864 | 1.55201756 |
| B3galt4 | 0.00308 | -0.89129714 |
| Crebl2 | 0.81311 | -0.04241744 |
| Irak3 | 0.29887 | -0.52590119 |
| Saa2 | 0.26243 | -0.9108115 |
| Kcnj4 | 0.03183 | -0.82159396 |
| Pou2f2 | 0.23052 | -0.67851591 |
| Eva1c | 0.22182 | -0.31758817 |
| Gls | 0.24106 | 0.33786896 |
| Gzmk | 0.37598 | -0.64122924 |
| Fzd5 | 0.00836 | -1.05193127 |
| Ap1s1 | 0.00212 | -0.97772743 |
| 1700047A11Rik | 0.90139 | 0.17678016 |
| Ccdc70 | 0.00992 | -1.14963365 |
| Avil | 0.01979 | 1.30945386 |
| Ttc5 | 0.28414 | -0.19884468 |
| Acy1 | 0.86836 | -0.07619547 |
| Ticam2 | 0.00144 | -1.29864962 |
| Osbpl1a | 0.0881 | 0.33014771 |
| Psme4 | 0.01923 | 0.53736583 |
| Gmnn | 0.91848 | 0.02470085 |
| Ago2 | 0.87747 | 0.0258775 |
| Dnase2b | 0.99738 | 0.00077005 |
| Aup1 | 0.16998 | -0.43647183 |
| Zfp770 | 0.02601 | 0.43158032 |
| Slc22a18 | 0.00171 | -1.03368452 |
| Mcm7 | 0.00667 | -0.62844382 |
| Hirip3 | 0.00314 | 0.78395249 |
| Tufm | 0.01554 | -0.66953643 |
| Lbx2 | 0.00144 | -1.32146639 |
| Twist2 | 0.00503 | -0.71283879 |
| C1galt1 | 0.1253 | 0.40841805 |
| Pbld2 | 0.38557 | 0.20341073 |
| Cox4i1 | 0.00237 | -0.93889547 |
| Socs4 | 0.01243 | 0.89751644 |
| Anxa2 | 0.04073 | -0.50154357 |
| Mrpl33 | 0.0199 | -0.54072359 |
| Ckmt2 | 0.28095 | -0.26541357 |
| Olfr1323 | 0.0714 | -2.38888118 |
| Gbp3 | 0.00268 | 2.04352465 |
| Arhgef7 | 0.50065 | -0.21750651 |
| Batf2 | 0.00145 | -1.23831704 |
| Hibadh | 0.31119 | 0.22445798 |
| Gga2 | 0.40961 | 0.36497894 |
| Snai1 | 0.78449 | 0.08637816 |
| Tmem191c | 0.04845 | 0.57218016 |
| Rpl8 | 0.00233 | -1.03000549 |
| Pygo1 | 0.2827 | -0.50517071 |
| Tmem150a | 0.59048 | 0.1536981 |
| AU022252 | 0.45042 | 0.15194359 |
| Ppl | 0.18303 | 0.46572331 |
| Tssk4 | 0.67171 | 0.10692989 |
| Fitm1 | 0.95111 | -0.04598076 |
| Apoc1 | 0.00783 | -1.55640785 |
| Dda1 | 0.14706 | -0.38808597 |
| Mrps16 | 0.00499 | -0.96121414 |
| Eef1e1 | 0.20826 | 0.43318516 |
| Txndc2 | 0.31151 | -0.76777081 |
| Adamdec1 | 0.00144 | -1.30713006 |
| Sh3kbp1 | 0.06916 | -0.32609888 |
| Snrpa1 | 0.12503 | 0.44305564 |
| Mta2 | 0.42763 | -0.14766018 |
| Nme4 | 0.63707 | -0.20961775 |
| Tcp1 | 0.01931 | 1.05222049 |
| Cenpe | 0.04614 | 1.08972455 |
| Spr | 0.00281 | -1.02289234 |
| Nsg2 | 0.96337 | -0.02199135 |
| Atl2 | 0.00242 | -0.83790537 |
| Bdp1 | 0.0319 | 0.91253742 |
| 2810025M15Rik | 0.00326 | -0.74313534 |
| Olfr1428 | 0.0056 | -0.91232186 |
| Rufy1 | 0.20552 | 0.39544402 |
| Olfr600 | 0.00144 | -1.31518407 |
| Spaca4 | 0.7166 | 0.12485679 |
| Carhsp1 | 0.06143 | -0.37042043 |
| Zwint | 0.08332 | 0.60175283 |
| Ogdhl | 0.23771 | 0.68455948 |
| Schip1 | 0.03864 | 1.01441707 |
| 2810403A07Rik | 0.01574 | 0.72643944 |
| Rin2 | 0.05006 | 0.42421114 |
| Rbm12b1 | 0.00775 | 0.9687978 |
| Bdh1 | 0.0063 | 1.44611857 |
| Ptcd1 | 0.72509 | 0.1165101 |
| Serpina7 | 0.14456 | -0.75632578 |
| 4933405L10Rik | 0.77869 | -0.13518307 |
| Zfp623 | 0.50707 | 0.21864791 |
| Bsn | 0.00467 | -1.20764345 |
| Toporsl | 0.00144 | -1.38185621 |
| Armc7 | 0.05331 | -0.61637287 |
| Folr2 | 0.0734 | -0.44434904 |
| Exosc3 | 0.19972 | -0.20910358 |
| Dnajc9 | 0.83251 | 0.03818433 |
| Bcr | 0.00209 | -0.79739956 |
| Wnt5a | 0.84448 | -0.11207864 |
| Tsx | 0.0044 | -1.37929289 |
| Trappc3 | 0.78341 | -0.09018288 |
| Inmt | 0.01198 | -1.20381871 |
| Oca2 | 0.00145 | -1.2388121 |
| Mblac1 | 0.00144 | -0.9246226 |
| Elfn2 | 0.01356 | -1.10879133 |
| Pde5a | 0.00144 | -1.27165604 |
| E130012A19Rik | 0.75459 | 0.09873041 |
| Tspo2 | 0.021 | -0.57502497 |
| Fcgrt | 0.01593 | -0.89397944 |
| Med30 | 0.64784 | 0.14699109 |
| Olfr888 | 0.00144 | -1.29085005 |
| Enpep | 0.09457 | 0.3222999 |
| Edar | 0.4533 | -1.03204691 |
| Chrnb3 | 0.00145 | -1.2388121 |
| Ophn1 | 0.00144 | 1.23915748 |
| Coq7 | 0.11104 | -0.3145504 |
| Coq7 | 0.93189 | -0.03317851 |
| Kng2 | 0.00145 | -1.2388121 |
| Olfr512 | 0.00144 | -1.56449565 |
| Wbp2nl | 0.00144 | -1.28116048 |
| Ccdc103 | 0.06827 | -0.88524119 |
| Tc2n | 0.01455 | -1.22298239 |
| Gnl2 | 0.00586 | 1.33106945 |
| Sesn2 | 0.00185 | -0.87116815 |
| Abcb6 | 0.00396 | -0.89644658 |
| 4930448F12Rik | 0.01879 | -1.8301317 |
| Slc22a2 | 0.00218 | -1.18878227 |
| Ccl25 | 0.01107 | 0.64033626 |
| Scg3 | 0.16523 | 0.47634863 |
| Rfx3 | 0.35293 | 0.21895508 |
| Itgb6 | 0.38767 | -0.21104928 |
| Cep170 | 0.99902 | 0.002097 |
| Ifit2 | 0.0039 | 2.38466297 |
| Morc3 | 0.00158 | 1.03281356 |
| Cspg4 | 0.00194 | -0.95672307 |
| Apobec1 | 0.85479 | 0.1039044 |
| Ndufb3 | 0.02576 | -0.45364934 |
| Alb | 0.00145 | -1.6102572 |
| Kcne1l | 0.00276 | -1.10776704 |
| 4930488L21Rik | 0.00144 | -1.30017714 |
| Efemp2 | 0.00144 | -1.11466552 |
| 1700026D08Rik | 0.00144 | -1.35112763 |
| Bin3 | 0.01509 | -0.52385294 |
| Map2k5 | 0.81596 | 0.05129802 |
| Crybg3 | 0.39043 | -0.18523052 |
| Yod1 | 0.00144 | 2.4019766 |
| Lcorl | 0.08773 | 0.61055963 |
| 4930470F04Rik | 0.00144 | -1.45950387 |
| Wbp1 | 0.0049 | -1.04055571 |
| Cenpv | 0.345 | -0.3529259 |
| Cbx3 | 0.01487 | 0.84575196 |
| Gm35405 | 0.00159 | -1.26431393 |
| Tcof1 | 0.00144 | -1.3712276 |
| Nsf | 0.00439 | -0.84763715 |
| Copb1 | 0.00173 | 0.96962141 |
| 1500035N22Rik | 0.22063 | -0.99852824 |
| Uhrf1bp1l | 0.35789 | -0.31005788 |
| Dnajc1 | 0.00891 | 0.85219977 |
| Pcdhb15 | 0.6566 | -0.39792391 |
| Smpd4 | 0.01163 | -0.53133897 |
| Nrg4 | 0.87986 | 0.0464243 |
| Car7 | 0.17237 | -0.2724283 |
| Snx1 | 0.09212 | -0.28547403 |
| Ccdc120 | 0.15269 | -1.71915997 |
| Arhgef9 | 0.01968 | -0.82668151 |
| Kcnh6 | 0.00144 | -1.28116048 |
| Rnf213 | 0.10188 | 0.36501156 |
| Ocrl | 0.05104 | 1.09838292 |
| Serpina3n | 0.00827 | -1.01817494 |
| Sin3a | 0.44452 | 0.11924109 |
| Gcat | 0.08706 | -0.59124243 |
| Myo16 | 0.05237 | -2.34797237 |
| Zbbx | 0.04168 | -2.77572696 |
| Bud13 | 0.24271 | -0.42750687 |
| Junb | 0.90338 | -0.03555956 |
| Prkaa1 | 0.00208 | 1.09604187 |
| Dctpp1 | 0.00798 | -0.92412644 |
| Mtdh | 0.09906 | 0.27996191 |
| Elof1 | 0.00413 | -0.92468015 |
| Gm6377 | 0.00144 | -1.24811894 |
| Fam181b | 0.00545 | -0.86389632 |
| Ggnbp1 | 0.00144 | -1.12936707 |
| Scrn2 | 0.18678 | -0.51112584 |
| Poll | 0.01378 | -0.73551045 |
| Zscan12 | 0.0402 | 0.52159558 |
| Ankk1 | 0.18485 | -0.94138241 |
| A430035B10Rik | 0.00144 | 1.39685247 |
| Rc3h2 | 0.12544 | -0.26776589 |
| Cdk19 | 0.12496 | 0.3190928 |
| Vmn1r199 | 0.00144 | -1.26560808 |
| Fyco1 | 0.00214 | -0.94722338 |
| Mcm2 | 0.01117 | -0.97442789 |
| Pcnxl2 | 0.00144 | -1.30052723 |
| B3galnt2 | 0.00144 | 1.35718206 |
| Triobp | 0.00699 | -0.65063004 |
| A230050P20Rik | 0.94891 | 0.01802705 |
| Wipi1 | 0.70518 | -0.07005662 |
| Bend5 | 0.24556 | -0.1906561 |
| Dab2 | 0.17648 | 0.25228187 |
| Tcaf1 | 0.03872 | 0.41298881 |
| Tcaf1 | 0.21175 | 0.33205593 |
| Mmab | 0.05298 | -0.57313533 |
| Soga3 | 0.34044 | -0.45551088 |
| Cdk5 | 0.43694 | -0.22444309 |
| Oaz3 | 0.68858 | 0.36190681 |
| Snrpc | 0.01643 | -0.70369667 |
| Psd2 | 0.18011 | -0.79404543 |
| E2f4 | 0.46062 | 0.62320861 |
| Rgn | 0.13276 | 0.4161002 |
| Efcab14 | 0.48593 | -0.10617447 |
| Plekhd1 | 0.00528 | -1.53085223 |
| Sdc2 | 0.0017 | 1.3725505 |
| Gm34014 | 0.09217 | -0.35203865 |
| LOC102639958 | 0.19961 | -0.49514228 |
| Hiat1 | 0.00229 | 1.02207475 |
| Gjd4 | 0.91889 | 0.1489828 |
| Serpina3c | 0.19378 | -0.90497243 |
| C630050I24Rik | 0.02931 | -2.15775259 |
| Ctgf | 0.05045 | 0.4857957 |
| Cfd | 0.83114 | -0.27442312 |
| Dnajc15 | 0.62234 | -0.10978168 |
| Arid4a | 0.00257 | 0.77591444 |
| Smim3 | 0.94062 | -0.02315294 |
| Paqr3 | 0.00144 | -1.29085005 |
| Usp14 | 0.00403 | -1.27106364 |
| Mob2 | 0.18344 | -0.40217376 |
| Zc2hc1b | 0.2398 | -1.68783431 |
| Glrx5 | 0.10017 | -0.6043289 |
| Atp2b3 | 0.5677 | 0.31884399 |
| Slc25a33 | 0.41294 | 0.30029656 |
| Ltbr | 0.00144 | -1.06444382 |
| Zfp511 | 0.94889 | -0.01239507 |
| Eps15 | 0.60404 | 0.111447 |
| Elfn1 | 0.71381 | -0.16221615 |
| Phlpp1 | 0.04359 | 0.50878325 |
| Dbx1 | 0.00497 | -1.52918587 |
| Ccdc39 | 0.19824 | -0.71979811 |
| Igsf9 | 0.67463 | 0.57753987 |
| Bcl7a | 0.07208 | -0.34169691 |
| 6330416G13Rik | 0.02341 | -0.62635091 |
| 1700113H08Rik | 0.82084 | -0.08861726 |
| Ppp1r3g | 0.77474 | -0.18508806 |
| Igsf10 | 0.05151 | 0.68781374 |
| Pld6 | 0.72593 | 0.18163677 |
| Abcg3 | 0.00145 | -1.22376446 |
| Traf3ip1 | 0.00192 | 1.17528129 |
| Sel1l | 0.11277 | 0.41274142 |
| 4930590J08Rik | 0.01531 | -1.38616337 |
| Psmb4 | 0.0039 | -0.84578222 |
| Prl | 0.00144 | -1.20555712 |
| Klk1b8 | 0.51805 | -0.20394639 |
| Alg3 | 0.44778 | -0.34967947 |
| Cuzd1 | 0.03002 | 1.03664243 |
| Dok7 | 0.01363 | -0.83858463 |
| Hmbs | 0.49311 | -0.24545634 |
| Chmp2a | 0.52321 | -0.14499042 |
| Gsto1 | 0.59431 | 0.24521413 |
| Rbfa | 0.14655 | -0.59491129 |
| Etfb | 0.00144 | -1.20094879 |
| Abtb2 | 0.58081 | -0.25404295 |
| Zfp672 | 0.05969 | -0.46154623 |
| Ank | 0.17005 | 0.50966952 |
| Cdca8 | 0.21157 | -0.48359022 |
| Dennd2a | 0.02369 | -0.47038345 |
| Nudt14 | 0.01223 | -0.9343423 |
| Brms1 | 0.01929 | -0.56031561 |
| Zfp296 | 0.40073 | -0.24058166 |
| Stard3 | 0.33687 | -0.20176755 |
| Zdhhc7 | 0.07676 | -0.48663464 |
| Pllp | 0.00211 | -1.01228913 |
| Oas1f | 0.00613 | -0.92909658 |
| 1700080G18Rik | 0.03023 | -0.79757399 |
| Cahm | 0.00144 | -1.27165604 |
| Klc3 | 0.20253 | -0.44792114 |
| LOC545964 | 0.00242 | -1.17446216 |
| Alyref | 0.00145 | -1.10766759 |
| Agbl4 | 0.01097 | -1.10038984 |
| Npdc1 | 0.00144 | -0.97232595 |
| Vmn1r195 | 0.09911 | -1.47829169 |
| Asb12 | 0.64257 | -0.15365404 |
| Fcrla | 0.35637 | 0.43257571 |
| Ccndbp1 | 0.55203 | 0.10716408 |
| 1810065E05Rik | 0.00144 | -1.29746368 |
| Tmem150c | 0.05231 | 1.03455059 |
| Olfr419 | 0.00543 | -1.12233866 |
| Glod4 | 0.66514 | -0.09190809 |
| Mettl3 | 0.08711 | 0.53170807 |
| Olfr1489 | 0.00203 | -1.26528643 |
| Gp9 | 0.00144 | -1.45975588 |
| Specc1 | 0.44039 | -0.19494073 |
| Zfp273 | 0.00144 | 2.23127352 |
| Kif23 | 0.84151 | 0.17283032 |
| Lrrc26 | 0.00144 | -1.28116048 |
| Dmrtc1c2 | 0.00144 | -1.33632959 |
| 4921507P07Rik | 0.0075 | -1.08536055 |
| Dnajb1 | 0.17228 | -0.40207128 |
| Fndc1 | 0.06474 | -0.38070658 |
| Slc6a1 | 0.00145 | -1.2551153 |
| Krt42 | 0.00144 | -1.28116048 |
| Zdhhc12 | 0.02283 | -0.66096427 |
| Kcne2 | 0.00152 | -1.25878908 |
| Emcn | 0.69212 | 0.13413766 |
| Cep85l | 0.00725 | 1.10075297 |
| Fabp12 | 0.19464 | -0.73352156 |
| Smpdl3a | 0.22385 | 0.27448131 |
| Psg16 | 0.13374 | -0.8956869 |
| Tas2r108 | 0.00144 | -1.39713491 |
| Ankrd53 | 0.00144 | -1.42249344 |
| Map2k3os | 0.48721 | 0.2175014 |
| Phax | 0.0018 | 0.97665613 |
| Grem2 | 0.00144 | -1.28116048 |
| Trim24 | 0.00144 | 1.75152729 |
| Golt1b | 0.01591 | 0.64626509 |
| Nrip1 | 0.00546 | 0.71253008 |
| Pcnxl2 | 0.00293 | -1.29732355 |
| Nol7 | 0.00144 | 2.05840512 |
| Iqcg | 0.3455 | -0.41538934 |
| 4930465K10Rik | 0.47091 | -0.44168745 |
| Ikbip | 0.04554 | 0.43872593 |
| Usp49 | 0.72533 | 0.10371787 |
| Rnf220 | 0.36383 | -0.30879439 |
| Arpc2 | 0.46261 | 0.13506525 |
| Xpo5 | 0.60188 | 0.10099104 |
| Pias3 | 0.26166 | -0.22816606 |
| Krt80 | 0.20232 | -0.52968897 |
| Cops6 | 0.01681 | -0.63735727 |
| R3hcc1 | 0.3636 | -0.23379519 |
| Tmtc3 | 0.00144 | 1.98711871 |
| Urb1 | 0.37046 | 0.17915656 |
| Pkp1 | 0.00144 | -1.28116048 |
| Gsg2 | 0.73437 | -0.109882 |
| Gp1bb | 0.192 | -0.36038 |
| A730036I17Rik | 0.19082 | 0.59151319 |
| B230208H11Rik | 0.84887 | -0.12499113 |
| Zfp318 | 0.02441 | -1.05690317 |
| Atp1b1 | 0.25616 | -0.22572901 |
| LOC102641083 | 0.00408 | 1.17649022 |
| 9230112D13Rik | 0.03643 | -0.96062012 |
| Zfp444 | 0.00419 | -0.99956366 |
| Zfp444 | 0.00149 | -0.8632383 |
| Fam184a | 0.59558 | -0.17003323 |
| Slc35b3 | 0.00831 | 0.86832801 |
| Olfr924 | 0.00144 | -1.28116048 |
| C8b | 0.00144 | -1.31495282 |
| Ifi202b | 0.00144 | -1.29746368 |
| Ccdc30 | 0.687 | -0.29331822 |
| Tmprss11g | 0.00144 | -1.29049509 |
| Cd52 | 0.00401 | -1.24733205 |
| Usp47 | 0.01811 | 1.00163434 |
| Tnp1 | 0.28897 | -0.543427 |
| Tle3 | 0.00144 | -1.59742099 |
| Pdgfrb | 0.00241 | -0.84218398 |
| Zfp248 | 0.35143 | -0.65647235 |
| Aurka | 0.06625 | -0.6243457 |
| Lypd3 | 0.05995 | -1.31819469 |
| LOC102641619 | 0.54075 | -0.51540399 |
| Mpv17 | 0.63201 | 0.1512445 |
| Ropn1l | 0.00363 | -0.9689795 |
| Olfr1044 | 0.00373 | -1.08684252 |
| Jchain | 0.09114 | 0.40883121 |
| Tnfaip8l2 | 0.01622 | -0.96654519 |
| Ptprv | 0.00144 | -1.29085005 |
| Lamp1 | 0.27046 | -0.19682711 |
| Jagn1 | 0.71811 | -0.13523182 |
| Mgat2 | 0.23546 | -0.19232401 |
| Gsdmc | 0.43856 | -0.59864001 |
| Mkrn2 | 0.42202 | 0.20799388 |
| Vps28 | 0.01703 | -0.66723012 |
| Mycbp | 0.78203 | -0.06193722 |
| Crtam | 0.22751 | -1.87218688 |
| Olfr1362 | 0.0111 | -1.02598223 |
| Mtmr3 | 0.00144 | -1.25511559 |
| Ube4a | 0.50357 | 0.50187962 |
| Mgst2 | 0.56109 | -0.2049858 |
| Rps6ka1 | 0.00144 | -1.43579193 |
| 1700023E05Rik | 0.00144 | -1.27165604 |
| Rrp7a | 0.01558 | -0.95349509 |
| Ipo9 | 0.85352 | 0.04229688 |
| Letm2 | 0.86113 | -0.03222448 |
| Ptchd1 | 0.05239 | -0.84985239 |
| Srsf11 | 0.50184 | -0.13312868 |
| Spcs1 | 0.60611 | 0.1365375 |
| P3h3 | 0.01295 | -0.57365771 |
| Gabrd | 0.11663 | -0.93437967 |
| Apbb2 | 0.08798 | -0.29190352 |
| Trabd | 0.00714 | -0.82358824 |
| Diras1 | 0.04217 | -1.08600544 |
| Adam7 | 0.00144 | -1.26560808 |
| Actc1 | 0.48943 | -0.24053995 |
| Gng13 | 0.00294 | -1.14809462 |
| Nfxl1 | 0.04639 | 1.688244 |
| Psmd8 | 0.96788 | -0.02035178 |
| Cep63 | 0.77824 | -0.06797161 |
| Phospho1 | 0.03548 | -1.38204068 |
| Zfp572 | 0.01335 | -1.18861789 |
| Nkx2-6 | 0.44002 | -0.30891119 |
| Mxd4 | 0.00828 | -0.67267424 |
| Usp44 | 0.00168 | 1.44219482 |
| Msantd1 | 0.34323 | 0.15639555 |
| Fam228b | 0.48295 | -0.13017887 |
| Pou6f2 | 0.10553 | 0.44661039 |
| Nudt7 | 0.53293 | 0.16235814 |
| Cox7a1 | 0.00228 | -0.93344826 |
| Rnft1 | 0.00174 | 1.21245581 |
| Olfr510 | 0.00144 | -1.24811894 |
| 5830411J07Rik | 0.11793 | -0.5517388 |
| Zfp710 | 0.00478 | -0.72949766 |
| Fam117a | 0.00155 | -0.98568512 |
| Chic2 | 0.00384 | -0.68039039 |
| Acoxl | 0.62318 | -0.32459223 |
| Itsn2 | 0.00406 | 2.15573802 |
| Sdf2 | 0.04608 | -0.58291398 |
| Cpsf3l | 0.59896 | -0.118973 |
| Klhdc3 | 0.04519 | -0.44089053 |
| Rad51 | 0.06573 | 0.64250137 |
| Ptprm | 0.00299 | 0.9280299 |
| Lpgat1 | 0.19609 | -0.21166362 |
| Sh3pxd2b | 0.57841 | -0.13885014 |
| Stx18 | 0.04909 | 0.47284879 |
| Igf1 | 0.02516 | -0.63315146 |
| 4921511H03Rik | 0.01669 | -1.66292519 |
| Hand2 | 0.29044 | -0.23655418 |
| Pon2 | 0.01028 | 1.47615315 |
| Olfr784 | 0.00654 | -1.54969761 |
| Cpne9 | 0.8139 | 0.13718284 |
| Sugp2 | 0.00969 | -0.62701214 |
| Ccdc15 | 0.69424 | -0.16742952 |
| Kctd19 | 0.00266 | -2.14429661 |
| Dpp3 | 0.00639 | -0.77240393 |
| Slain1 | 0.00144 | -1.28116048 |
| Clec4a3 | 0.30654 | 0.28264978 |
| 3300002I08Rik | 0.24283 | -1.95126312 |
| Olfr485 | 0.16934 | -1.83138444 |
| Nfatc3 | 0.00882 | -0.61614677 |
| 1600014C23Rik | 0.00288 | -1.06232977 |
| Ica1l | 0.62895 | 0.18921291 |
| Gm24463 | 0.09529 | -0.50332177 |
| Ccdc184 | 0.02368 | -0.78947542 |
| Grin3a | 0.00145 | -1.26219879 |
| Mocs3 | 0.02352 | -0.72204046 |
| Rhobtb1 | 0.11201 | -0.33490863 |
| Pcdhb20 | 0.50352 | 0.1725218 |
| Csf2rb2 | 0.40839 | 0.28657475 |
| Tmed11 | 0.00144 | -1.27165604 |
| Col19a1 | 0.00144 | -1.28898079 |
| Nsdhl | 0.08838 | 1.0523663 |
| Ptgr2 | 0.56384 | 0.10424429 |
| Bicc1 | 0.81041 | -0.05825553 |
| Msln | 0.07399 | -1.80622504 |
| Gm12580 | 0.00144 | -1.40263095 |
| Atp11b | 0.00144 | 1.32449652 |
| Dennd1a | 0.12118 | -0.4001681 |
| Mvb12a | 0.0016 | -0.94008423 |
| Ranbp3 | 0.03533 | -0.7865716 |
| Krtdap | 0.0031 | -1.09550759 |
| Napsa | 0.00462 | -1.36943057 |
| Nemf | 0.01993 | 1.1929301 |
| Arrb2 | 0.01893 | -0.56211273 |
| Ccdc92 | 0.01126 | -0.81073672 |
| Ssu72 | 0.92408 | -0.02907157 |
| Vmn1r171 | 0.00144 | -1.28898079 |
| Neto1 | 0.00145 | -1.34348037 |
| Ambp | 0.00474 | -2.31423666 |
| Acyp1 | 0.80521 | 0.04587377 |
| Clec4g | 0.21341 | -0.28042894 |
| Svip | 0.00947 | 0.93157893 |
| Rbms1 | 0.00144 | 1.76596024 |
| Rnf14 | 0.00545 | 0.69589106 |
| Sigirr | 0.00175 | -1.16288886 |
| Prl2a1 | 0.00144 | -1.28898079 |
| Zic4 | 0.30617 | -0.40604025 |
| Lpcat3 | 0.24907 | -0.43728342 |
| Zar1 | 0.01102 | -2.68255642 |
| Pax1 | 0.01564 | -0.98859476 |
| Sall4 | 0.02229 | -0.61302002 |
| Nefm | 0.07933 | -0.9309806 |
| Stk26 | 0.38533 | -0.44296029 |
| Mcpt4 | 0.02663 | -1.42923433 |
| Lrp8 | 0.00144 | -1.30713006 |
| Lrit1 | 0.00144 | -1.31884045 |
| Col22a1 | 0.07906 | -0.61388903 |
| Tram1l1 | 0.78369 | 0.35949283 |
| Cops8 | 0.29834 | 0.52740067 |
| 9230104L09Rik | 0.01995 | -0.74823419 |
| Cdkn2a | 0.00144 | -1.29746368 |
| Cct5 | 0.04502 | -0.38629367 |
| 2410002F23Rik | 0.27641 | -0.19124184 |
| Olfr95 | 0.11171 | -0.86722663 |
| Vps13b | 0.17775 | 0.31615166 |
| Sit1 | 0.87152 | -0.07355315 |
| Pgrmc1 | 0.00762 | 1.2258044 |
| Ubr2 | 0.61509 | -0.10653421 |
| 1700063H04Rik | 0.01655 | -0.88663264 |
| Slc22a29 | 0.00144 | -1.36353418 |
| Rbm19 | 0.00324 | -0.81926501 |
| R3hdm1 | 0.93548 | -0.0141603 |
| Chn2 | 0.00144 | -1.25118919 |
| Dtx4 | 0.00335 | -1.09177199 |
| 4930431F10Rik | 0.00145 | -1.2388121 |
| Efna5 | 0.05579 | -0.46179836 |
| Krtap7-1 | 0.06046 | -0.47716713 |
| Klf2 | 0.00144 | -1.20611698 |
| Ifnb1 | 0.00144 | -1.32766452 |
| Colec10 | 0.00767 | -1.61259868 |
| B630019A10Rik | 0.00144 | -1.28898079 |
| Slc22a16 | 0.00224 | -0.84350491 |
| Aim1 | 0.89712 | 0.03989511 |
| Trpa1 | 0.30639 | -0.54716176 |
| Astn1 | 0.07401 | 0.7518348 |
| Steap4 | 0.00465 | 0.79834921 |
| Tmem42 | 0.31436 | -0.26197092 |
| 4930578I07Rik | 0.00144 | -1.30713006 |
| Vipas39 | 0.96106 | 0.01002839 |
| Tdh | 0.07882 | -0.4457491 |
| Zfp318 | 0.04298 | 0.74160021 |
| Epo | 0.36506 | -0.6173949 |
| Timm44 | 0.1885 | -0.20690929 |
| Olfr1010 | 0.01361 | -1.32681573 |
| Olfr1337 | 0.00144 | -1.28116048 |
| Myh8 | 0.07088 | -0.35713225 |
| Wdr59 | 0.07365 | -0.41619509 |
| Mbd1 | 0.00144 | 1.14384915 |
| Lyl1 | 0.00144 | -0.9883371 |
| Mrpl12 | 0.02808 | -0.88507007 |
| Pprc1 | 0.60306 | -0.09719701 |
| Olfr1126 | 0.00144 | -1.30017714 |
| Eps8l3 | 0.00144 | -1.29085005 |
| Rab11fip2 | 0.75838 | -0.11331845 |
| Zfp429 | 0.00192 | 1.65683317 |
| Prdm8 | 0.2356 | -0.37690855 |
| Paics | 0.03055 | 0.63962276 |
| Cd59a | 0.06693 | 0.47082286 |
| Aspn | 0.00144 | 2.14951744 |
| Glt8d2 | 0.3336 | -0.46690174 |
| Sulf1 | 0.56035 | 0.12682954 |
| Rnf207 | 0.01375 | 1.26862385 |
| Antxr2 | 0.01103 | 0.59445423 |
| Rspo1 | 0.4669 | -0.61497032 |
| Sdhaf3 | 0.00149 | -1.21097148 |
| Inppl1 | 0.0911 | -0.54937677 |
| Abhd11 | 0.01009 | -0.79262946 |
| Filip1l | 0.00144 | 2.04205143 |
| Ep300 | 0.00151 | -1.17792995 |
| Asb8 | 0.55712 | 0.20770539 |
| Ap2a1 | 0.08639 | -0.55165637 |
| Oxld1 | 0.02385 | -0.75618231 |
| 1110008L16Rik | 0.1784 | 0.78806326 |
| Anks1 | 0.07182 | -0.46414621 |
| Fbxw11 | 0.98681 | -0.0032972 |
| Ube3c | 0.79071 | 0.05455311 |
| Mrpl32 | 0.28764 | 0.35751901 |
| Uhrf2 | 0.00386 | 0.89011504 |
| Trim17 | 0.41539 | -0.36694348 |
| 1700063A18Rik | 0.00144 | -1.29085005 |
| Pskh1 | 0.26176 | -0.57899467 |
| Cables2 | 0.1146 | -0.26931416 |
| Orm2 | 0.00144 | -1.28898079 |
| Sap130 | 0.02446 | -0.49935348 |
| Ipo11 | 0.91103 | 0.02983272 |
| Clec10a | 0.00144 | -1.34162187 |
| Anapc1 | 0.38317 | -0.1790811 |
| Eif3m | 0.01543 | 0.52248761 |
| Sec24d | 0.00597 | 1.36323395 |
| Zfp191 | 0.37268 | 0.14753224 |
| Ambra1 | 0.47804 | -0.23461975 |
| Ddx50 | 0.00144 | 1.55608812 |
| Dock7 | 0.00144 | 1.993443 |
| Med18 | 0.1018 | -0.32375308 |
| Slitrk1 | 0.03295 | -0.87047824 |
| Rp9 | 0.00144 | -1.42771059 |
| 4930519P11Rik | 0.00144 | -1.14277448 |
| Pmm2 | 0.57652 | 0.14600403 |
| Ankhd1 | 0.17645 | 0.44491512 |
| Wdr36 | 0.07016 | 0.4987774 |
| Slco1b2 | 0.09902 | -2.55937312 |
| Dcaf6 | 0.33459 | 0.18726103 |
| Slc23a1 | 0.46672 | -0.87112453 |
| Islr | 0.00361 | -0.88184325 |
| Cops5 | 0.23353 | 0.22645067 |
| Ccl3 | 0.00818 | -1.01564035 |
| Stmn3 | 0.00144 | -1.34668127 |
| Eva1a | 0.61976 | 0.11393228 |
| Asun | 0.00144 | 1.21728164 |
| Gbf1 | 0.24494 | -0.30233701 |
| 4933413L06Rik | 0.00144 | -1.28116048 |
| Zg16 | 0.2356 | -2.28160988 |
| Cmc2 | 0.03799 | -0.61649258 |
| Nadsyn1 | 0.42145 | -0.17197184 |
| Slc4a9 | 0.02241 | 0.72573965 |
| Mocos | 0.03436 | -0.53651667 |
| Fhl2 | 0.4251 | -0.23334712 |
| 1700029H14Rik | 0.42551 | -0.49517567 |
| Atp6v1g1 | 0.08869 | -0.30384582 |
| Hspbp1 | 0.11843 | -0.59077512 |
| Adad2 | 0.03956 | -0.79997687 |
| A430107J10Rik | 0.00144 | -1.20555712 |
| Cysltr1 | 0.0344 | 1.13563464 |
| Sptan1 | 0.38441 | 0.18636033 |
| Rgr | 0.00145 | -1.26219879 |
| Wbp5 | 0.04033 | 0.52827706 |
| E130006N16Rik | 0.02036 | 0.62048352 |
| Acat3 | 0.03905 | -0.6687655 |
| Fam26e | 0.92325 | 0.07080883 |
| Olfr51 | 0.00145 | -1.22376446 |
| Sprr1a | 0.00563 | -2.0022068 |
| 1700012P22Rik | 0.022 | -0.91339291 |
| Pcbd1 | 0.06058 | 0.63880519 |
| Zfp532 | 0.75781 | -0.37227051 |
| Fank1 | 0.30327 | -0.43644269 |
| Cpxm1 | 0.00144 | -1.42127208 |
| 1700122O11Rik | 0.73777 | -0.1379531 |
| Wdr45 | 0.33894 | -0.22743322 |
| Slc38a3 | 0.1809 | -0.33247121 |
| Clpp | 0.00174 | -0.96377029 |
| Cep97 | 0.13575 | 0.48454511 |
| Robo4 | 0.00793 | -0.61003696 |
| Peg12 | 0.11864 | -0.88719749 |
| Ccdc102a | 0.07257 | -0.30932603 |
| Zgpat | 0.02441 | -0.61129405 |
| Mapkap1 | 0.13618 | 0.59668402 |
| Tmem68 | 0.00144 | 1.31029525 |
| Morn4 | 0.24131 | 0.25611854 |
| Trip12 | 0.00208 | -1.15498993 |
| Wfdc3 | 0.17172 | -1.01068391 |
| Ddx27 | 0.0137 | 0.6483448 |
| Abcd4 | 0.91161 | -0.01946773 |
| Prm2 | 0.00636 | -0.85422758 |
| Eif1b | 0.21285 | -0.2951863 |
| Gjc1 | 0.38182 | 0.1705019 |
| Btc | 0.00144 | -1.26560808 |
| Nudt17 | 0.00144 | -1.29746368 |
| Aptx | 0.10344 | -0.28316568 |
| Zfp286it | 0.00145 | -1.2388121 |
| Olfr1348 | 0.0227 | -1.23485194 |
| Tmem29 | 0.29861 | 0.36736606 |
| Mthfs | 0.95854 | -0.01373921 |
| Cisd1 | 0.21956 | -0.38275653 |
| Angel2 | 0.53596 | -0.09459204 |
| Rnf13 | 0.01763 | 0.60729821 |
| Wnt5b | 0.20655 | -0.35482371 |
| Vdac3 | 0.55308 | -0.11539941 |
| Ugdh | 0.02317 | 0.72061245 |
| Ttc3 | 0.00144 | 1.21482388 |
| Tpbpa | 0.09302 | -1.47009156 |
| Slc17a7 | 0.2164 | -1.55282807 |
| Papl | 0.00149 | -1.2703832 |
| Vmn1r75 | 0.00635 | -1.30849771 |
| Calml3 | 0.00212 | -1.14445472 |
| Pex1 | 0.03069 | -0.64373955 |
| Card14 | 0.03172 | -0.81332982 |
| Fcna | 0.31852 | 0.36010505 |
| Fam78b | 0.03993 | -0.47894082 |
| Etv2 | 0.00144 | -1.21945192 |
| Cyp2g1 | 0.00158 | -1.2446827 |
| Cst7 | 0.00144 | -1.5296438 |
| Srpr | 0.46274 | -0.13971787 |
| Zadh2 | 0.3433 | 0.22584786 |
| Ccdc137 | 0.01876 | 0.62876657 |
| Cdh1 | 0.00144 | -1.28116048 |
| Samd3 | 0.00144 | -1.29746368 |
| Klhl10 | 0.04863 | 0.89062352 |
| Olfm1 | 0.00219 | -0.84960191 |
| Fam209 | 0.74559 | -0.25807088 |
| Dhx8 | 0.75101 | 0.07121727 |
| Mybph | 0.04162 | 0.82155147 |
| Mybph | 0.93888 | -0.04232963 |
| Chek2 | 0.38127 | 0.16860422 |
| Sall2 | 0.00955 | -0.60153209 |
| 4930438A08Rik | 0.05481 | -1.51362551 |
| Zw10 | 0.00378 | 0.74641812 |
| Rb1 | 0.05837 | 0.57651883 |
| Tanc1 | 0.22243 | 0.29409055 |
| Usp19 | 0.07562 | -0.46188169 |
| Calcoco1 | 0.15712 | -0.32006954 |
| Pnliprp1 | 0.00163 | -1.63998225 |
| Ttc7b | 0.05285 | 0.58349818 |
| Krtap31-1 | 0.66677 | -0.14427073 |
| Gpank1 | 0.00158 | -0.9816745 |
| Olfr921 | 0.38922 | 0.41297517 |
| Olfr796 | 0.07399 | -0.87715749 |
| Lypd2 | 0.05699 | -0.48741973 |
| Avp | 0.52821 | -0.33970294 |
| Ddx52 | 0.00177 | 1.4792687 |
| Zmat2 | 0.05608 | -0.35264227 |
| Nudt12 | 0.00144 | -1.15766275 |
| Dgki | 0.0959 | -2.39399647 |
| Mppe1 | 0.11466 | 0.36911644 |
| Gng11 | 0.0037 | -0.83873807 |
| Galm | 0.02169 | 1.23748714 |
| Cyp4f15 | 0.06841 | -1.04191855 |
| Ms4a4b | 0.37648 | 0.37963904 |
| Ube2v2 | 0.00288 | 1.18464754 |
| Rnf219 | 0.00144 | 1.61526477 |
| Ccdc136 | 0.26498 | -1.14834799 |
| Eif4ebp2 | 0.02772 | -0.6268308 |
| 4930522H14Rik | 0.00144 | -1.28898079 |
| Coch | 0.05862 | -0.887655 |
| Capzb | 0.68124 | -0.11624402 |
| Zfp474 | 0.00144 | -1.27992547 |
| Panx1 | 0.00318 | -0.84971176 |
| Pqbp1 | 0.01274 | -0.6582641 |
| Nfat5 | 0.04035 | 0.90718978 |
| Corin | 0.07614 | 0.32352832 |
| Mink1 | 0.29493 | -0.2298144 |
| Slpi | 0.00144 | -1.30713006 |
| Krtap13-1 | 0.00145 | -1.2388121 |
| 2410012M07Rik | 0.02574 | -0.96920392 |
| Rhpn2 | 0.52607 | -0.10623274 |
| Zfp212 | 0.0191 | -0.47451709 |
| Shkbp1 | 0.00459 | -0.69449081 |
| Rnf148 | 0.11058 | -0.62700399 |
| Gpr15 | 0.00144 | -1.28116048 |
| Chac1 | 0.31118 | 0.38894921 |
| Olfr1176 | 0.00144 | -1.28898079 |
| Dusp12 | 0.3208 | -0.16064416 |
| Asb13 | 0.00144 | 1.15730271 |
| Coq9 | 0.63747 | 0.35199583 |
| Lpar3 | 0.21843 | 0.44918616 |
| Arpp19 | 0.02668 | -0.46353819 |
| 1700052K11Rik | 0.29314 | -0.21142621 |
| Gramd4 | 0.0714 | -0.35277869 |
| Cyp2c70 | 0.79593 | 0.07388275 |
| Stxbp2 | 0.62267 | 0.16585183 |
| Stxbp2 | 0.69794 | 0.10773283 |
| Oas1e | 0.04355 | -0.70451259 |
| Nfia | 0.13243 | -0.42490679 |
| Ncl | 0.00144 | 1.94901736 |
| Smim24 | 0.04348 | -0.54987146 |
| Kitl | 0.00144 | 3.0012243 |
| Lmln | 0.5902 | 0.31632803 |
| Olfr1043 | 0.58767 | 0.14239785 |
| Prickle3 | 0.04336 | -0.55068753 |
| Adgrb3 | 0.00149 | -1.28183002 |
| Mcph1 | 0.95182 | 0.02219925 |
| Csrp3 | 0.49496 | -0.18829344 |
| 3110021A11Rik | 0.8458 | 0.07691395 |
| Cdt1 | 0.00418 | -0.87081608 |
| Cbx4 | 0.02025 | -0.54605515 |
| Serpina6 | 0.00144 | -1.28898079 |
| A230107O07Rik | 0.17784 | -0.76132008 |
| Olfr466 | 0.00144 | -1.43671944 |
| Nck2 | 0.64776 | 0.16304178 |
| Scly | 0.13528 | -0.35546605 |
| Lats2 | 0.86366 | 0.03375764 |
| Ppp2r3a | 0.17857 | 0.93894897 |
| Kif20a | 0.28897 | -0.29314032 |
| Gm4013 | 0.05612 | -0.56405839 |
| Npepps | 0.52746 | 0.10351285 |
| Kif7 | 0.00144 | -1.26934956 |
| Glyr1 | 0.0123 | 0.53200142 |
| Fbxo24 | 0.00317 | -1.12815152 |
| Slc38a1 | 0.04549 | 0.69399583 |
| Idh1 | 0.00164 | -0.88015424 |
| Phf6 | 0.00144 | 1.67801553 |
| Pcdh15 | 0.07394 | -2.36292941 |
| 2310010J17Rik | 0.00271 | -0.83637363 |
| Alppl2 | 0.28617 | -0.55025259 |
| 4930474N05Rik | 0.00144 | -1.50566664 |
| 1700013H16Rik | 0.00144 | -1.26934956 |
| Krtap14 | 0.00144 | -1.29049509 |
| 2810049E08Rik | 0.01281 | -0.93369864 |
| Ccdc141 | 0.1184 | -0.47487266 |
| Olfr958 | 0.00144 | -1.27165604 |
| Rsl1 | 0.00375 | 1.30621425 |
| Cysltr2 | 0.00144 | -1.24811894 |
| Dph5 | 0.53306 | 0.17796713 |
| Capns2 | 0.9369 | 0.07113455 |
| Cst12 | 0.00159 | -1.26431393 |
| Olfr1217 | 0.00144 | -1.29085005 |
| Tsacc | 0.00532 | -0.76976024 |
| Plet1 | 0.07063 | -0.99005572 |
| Ppm1d | 0.01283 | -0.60156524 |
| Rptor | 0.30959 | -0.40239068 |
| Tnfrsf12a | 0.51064 | -0.33859198 |
| Lrrc66 | 0.00257 | -1.1333102 |
| Serpina9 | 0.61317 | 0.16281393 |
| Exd1 | 0.59769 | -0.77478663 |
| Dpy19l4 | 0.00232 | 2.72706645 |
| Ptpn20 | 0.02711 | -2.22144687 |
| Usp54 | 0.8365 | -0.08346663 |
| Kcnh2 | 0.81938 | -0.1298068 |
| Clca2 | 0.06817 | -2.85018416 |
| Ctnna3 | 0.01909 | -1.43787403 |
| Ngdn | 0.8905 | 0.02694107 |
| Gypa | 0.16279 | -2.53921704 |
| BC005537 | 0.00245 | 0.9730126 |
| Cog8 | 0.13739 | -0.54512537 |
| Tpbpb | 0.59149 | -0.42285753 |
| Bbip1 | 0.00144 | 3.41975251 |
| Fkbp11 | 0.00438 | -1.06911584 |
| Mrps30 | 0.82339 | 0.07254844 |
| Olfr480 | 0.00144 | -1.26560808 |
| Tmco5b | 0.00144 | -1.27165604 |
| Zcchc13 | 0.00144 | -1.60060526 |
| Psmd3 | 0.00302 | -0.88082737 |
| Ppp1r13b | 0.11157 | -0.34487575 |
| Zfp493 | 0.00518 | -0.81972457 |
| Zfp869 | 0.00309 | 0.90097108 |
| Tek | 0.00398 | 1.29504046 |
| Hspa12b | 0.01529 | -0.59698664 |
| Tecpr2 | 0.12459 | -0.70217194 |
| Psd3 | 0.24272 | -0.53807514 |
| Vmn1r73 | 0.00824 | -1.00868743 |
| Spink2 | 0.04443 | -1.10645854 |
| Idh3b | 0.31489 | 0.42112703 |
| Fcgr2b | 0.38045 | 0.2327325 |
| Lrmp | 0.42372 | 0.21350987 |
| Atxn1l | 0.02682 | 0.42955914 |
| Cacybp | 0.00144 | 2.01427948 |
| Slc35a5 | 0.00312 | 1.20420756 |
| Sstr4 | 0.00208 | -1.22889094 |
| Slc7a11 | 0.00144 | -1.49245118 |
| Ccl21a | 0.00484 | -0.90155246 |
| Scd2 | 0.18399 | -0.2729439 |
| Ehd2 | 0.78925 | -0.54984973 |
| Pthlh | 0.17053 | 0.26867651 |
| Mob4 | 0.01261 | 0.5766344 |
| H2-T3 | 0.03113 | -1.58703982 |
| Atf6 | 0.04436 | -0.4843714 |
| Sec63 | 0.00144 | 2.53186428 |
| Gnao1 | 0.04918 | -0.61971396 |
| Bfsp1 | 0.01106 | -1.4508388 |
| B2m | 0.56298 | -0.13908765 |
| Akr1b8 | 0.02239 | -0.51801697 |
| Olfr231 | 0.00144 | -1.22925777 |
| Atp6v1g2 | 0.17814 | -0.22336864 |
| Vsig4 | 0.14893 | -0.49646161 |
| 4930485G23Rik | 0.00144 | -1.31495282 |
| Zfp84 | 0.00144 | 0.99145974 |
| Rbak | 0.00395 | -1.16169443 |
| Slc16a8 | 0.03454 | -0.61434621 |
| Lynx1 | 0.08903 | -0.46665607 |
| 4930533L02Rik | 0.00144 | -1.46547784 |
| Uqcrc2 | 0.7567 | -0.06415599 |
| Zmiz1 | 0.11488 | -0.97695128 |
| Mtx3 | 0.00145 | 1.85235895 |
| Scgb1a1 | 0.01844 | -1.9331996 |
| Dennd3 | 0.0234 | -0.45824648 |
| Grrp1 | 0.00144 | -1.21230386 |
| Nr4a3 | 0.04338 | -1.32466826 |
| Fbxl19 | 0.15025 | -1.39686297 |
| Serinc5 | 0.03548 | 0.55260006 |
| Cyld | 0.01784 | 1.04935094 |
| Olfr883 | 0.22617 | -0.9063479 |
| Chmp1a | 0.01458 | -0.80666062 |
| Tescl | 0.0627 | -1.27849118 |
| Prcc | 0.00144 | -1.06738909 |
| Sval2 | 0.00145 | -1.26219879 |
| Zcrb1 | 0.08428 | -0.292559 |
| Pdss1 | 0.3873 | 0.16160299 |
| Atg2b | 0.05474 | 0.80160561 |
| Elp5 | 0.08667 | -0.56502169 |
| Ddx20 | 0.00671 | 0.72502187 |
| Sh3bgr | 0.00722 | -0.87741443 |
| Clic4 | 0.72942 | -0.06637034 |
| Ppp2r5e | 0.00845 | -0.71892659 |
| Slitrk2 | 0.01818 | -1.08200933 |
| AW146154 | 0.00144 | 2.85078871 |
| Slc22a8 | 0.07072 | -0.79209082 |
| Ccdc38 | 0.05944 | -1.33249771 |
| Hagh | 0.2496 | -0.38569544 |
| Ppp1r37 | 0.01788 | -0.71576447 |
| Ppp1r37 | 0.00505 | -0.80683684 |
| Ptch1 | 0.02479 | -0.93294124 |
| Prpf4b | 0.5212 | 0.18484353 |
| Kif15 | 0.00145 | -1.22376446 |
| Irf9 | 0.35424 | -0.16115748 |
| Zfp91 | 0.00144 | 1.94394649 |
| Cabs1 | 0.00144 | -1.28898079 |
| Hsd11b1 | 0.01913 | -0.5111618 |
| Sesn3 | 0.0081 | 0.9195622 |
| Drosha | 0.74691 | 0.05088842 |
| Olfr829 | 0.00145 | -1.2388121 |
| Vmn1r47 | 0.05326 | -0.60529738 |
| Chst11 | 0.12874 | -0.64831955 |
| Arl14ep | 0.02673 | 0.45516155 |
| Sec61a2 | 0.05367 | 0.66495755 |
| Nelfcd | 0.19041 | -0.37361661 |
| Zfp503 | 0.16682 | -0.43540687 |
| Olfr624 | 0.0029 | -1.20702967 |
| D230025D16Rik | 0.45305 | -0.23242449 |
| Enc1 | 0.33186 | 0.19570783 |
| Gzmn | 0.00145 | -1.26219879 |
| Fgf12 | 0.70949 | 0.33912838 |
| Otud7a | 0.0088 | -1.08115135 |
| Rbmx2 | 0.00145 | 1.09909785 |
| Gm14461 | 0.00144 | -1.27165604 |
| Dapk2 | 0.02185 | -0.70074009 |
| Cdx4 | 0.00144 | -1.42017585 |
| Olfr1241 | 0.00144 | -1.29085005 |
| Dgat2l6 | 0.01692 | -1.03712037 |
| Gan | 0.32904 | -0.19246371 |
| Glp2r | 0.02162 | -1.25524905 |
| Sptan1 | 0.03833 | 0.44018127 |
| Tmem51 | 0.00279 | -1.09254889 |
| Dzip1 | 0.17847 | -0.6893905 |
| Serpina3a | 0.00144 | -1.28898079 |
| Dcun1d2 | 0.60117 | -0.55709285 |
| Cdca4 | 0.87844 | 0.03051509 |
| Rec114 | 0.06919 | -1.05625599 |
| Dkk4 | 0.00144 | -1.30017714 |
| Vwce | 0.00144 | -1.28116048 |
| Gm31825 | 0.80344 | -0.08447472 |
| Mettl13 | 0.51151 | -0.21153413 |
| Igf2r | 0.03442 | -0.40902845 |
| Lzic | 0.49172 | 0.21970218 |
| Hars | 0.00236 | -0.83917867 |
| Lce1b | 0.00144 | -1.40202659 |
| Acaa2 | 0.02749 | -0.64780833 |
| Aqp1 | 0.02715 | -0.58509727 |
| Coq5 | 0.53153 | 0.28333451 |
| Cdca5 | 0.11051 | -0.74805525 |
| Nmi | 0.03703 | 0.56136457 |
| Oxct2a | 0.04257 | -1.97334567 |
| Wdr74 | 0.25391 | -0.41007215 |
| Unc45a | 0.00415 | -0.70654889 |
| Tmem192 | 0.41422 | -0.29592225 |
| Tgfb3 | 0.07665 | -0.30958298 |
| Ppp1r14c | 0.0018 | -0.86725167 |
| Ccdc93 | 0.04815 | -0.45462737 |
| Anapc15 | 0.43801 | 0.17084939 |
| Nat2 | 0.80044 | 0.05546941 |
| Mpp1 | 0.0107 | 0.57474599 |
| Rfxap | 0.97392 | -0.00636709 |
| Cpne5 | 0.13795 | -0.68631549 |
| Olfr414 | 0.01218 | -0.59719772 |
| Col4a1 | 0.7476 | -0.05667405 |
| Olfr381 | 0.43479 | -0.43235114 |
| Cyp2d26 | 0.33861 | -0.27951417 |
| Gtpbp2 | 0.27574 | 0.3202682 |
| Mydgf | 0.00144 | -1.11384728 |
| Tecrl | 0.00356 | 0.75860667 |
| Zfp637 | 0.05505 | -0.6572868 |
| Dus3l | 0.19606 | -0.26560574 |
| Synpo | 0.01264 | -0.58610894 |
| Kera | 0.00144 | -1.28116048 |
| Irg1 | 0.36224 | 0.46384972 |
| Nup98 | 0.2139 | 0.21651525 |
| Vmn1r58 | 0.00144 | -1.26911831 |
| Ttll5 | 0.00918 | -1.43923229 |
| Olfr1209 | 0.00144 | -1.25927895 |
| Bub1 | 0.75968 | 0.13684067 |
| Olfr503 | 0.00601 | -1.09465341 |
| Fshr | 0.185 | -0.64767237 |
| Arf2 | 0.17885 | -0.27062877 |
| Olfr74 | 0.9318 | -0.07019941 |
| Lamp5 | 0.00144 | -1.22925777 |
| Ercc6l | 0.01818 | 0.77417847 |
| Nubp1 | 0.0062 | -0.85345456 |
| 4930555K19Rik | 0.0239 | -0.52707384 |
| Smc1a | 0.03581 | 0.55079419 |
| Klk4 | 0.027 | -0.51100894 |
| Gmcl1 | 0.01261 | 0.75902661 |
| 4930512B01Rik | 0.00144 | -1.31495282 |
| Gpbp1 | 0.01874 | 0.99895603 |
| Naa50 | 0.01007 | 0.88494219 |
| Slc37a3 | 0.15548 | 0.28696203 |
| Grpel1 | 0.90948 | 0.05382615 |
| Liph | 0.02 | -1.88152579 |
| Degs2 | 0.01412 | -1.32939179 |
| Daglb | 0.00562 | -0.69381965 |
| Nup50 | 0.01755 | 0.52513555 |
| Ctf1 | 0.97359 | -0.01776855 |
| Cbr4 | 0.09234 | 0.56657317 |
| Creld2 | 0.57379 | -0.10019156 |
| Uchl5 | 0.00144 | 1.36276703 |
| Zfp451 | 0.29351 | -0.33165816 |
| Rdh7 | 0.00144 | -1.29878664 |
| Mamstr | 0.14815 | -0.37870329 |
| Usp20 | 0.00528 | -0.91106526 |
| Muc5b | 0.00434 | -1.0948178 |
| Vwa7 | 0.02465 | -0.73131833 |
| Nipa1 | 0.005 | 0.80386397 |
| Olfr154 | 0.0391 | -0.97701755 |
| Kdm6b | 0.00293 | -0.97551848 |
| Adgrl3 | 0.00144 | -1.29746368 |
| BC089597 | 0.00232 | -1.32389779 |
| Rac2 | 0.00383 | -0.78049666 |
| 4933401B06Rik | 0.9818 | -0.0084422 |
| 4930546C10Rik | 0.52181 | -0.13142631 |
| Bmi1 | 0.02882 | -0.59982246 |
| Olfr355 | 0.05772 | -0.84279299 |
| Tab1 | 0.00379 | -0.83099079 |
| Slc25a39 | 0.68011 | -0.26505154 |
| 1110038F14Rik | 0.03323 | -0.45959859 |
| Slc35a4 | 0.56507 | -0.20039483 |
| Mrps17 | 0.68458 | 0.15116067 |
| Anapc11 | 0.00584 | -0.8813515 |
| Zc3h18 | 0.00975 | -0.57190089 |
| Them4 | 0.01682 | -0.46858441 |
| Cenpo | 0.91115 | -0.02128073 |
| Pex5l | 0.37085 | 0.54278357 |
| Ints4 | 0.52539 | 0.11347126 |
| Ascc2 | 0.08958 | -0.31219254 |
| Zeb2 | 0.90266 | 0.03727669 |
| Arl10 | 0.10892 | -0.90088491 |
| Fam132b | 0.00144 | -1.28116048 |
| Dnah6 | 0.12324 | -1.04181877 |
| Pde4d | 0.00203 | -1.42660238 |
| Cul4b | 0.0018 | 1.59493598 |
| Olfr713 | 0.00908 | -1.21110851 |
| Mmp10 | 0.14538 | -0.78034564 |
| Olfr868 | 0.01314 | -1.70574357 |
| P2ry14 | 0.06073 | 0.44833927 |
| Polr2f | 0.02371 | -0.81971096 |
| Lmnb1 | 0.05631 | 0.4147949 |
| Rabl3 | 0.67859 | -0.12081417 |
| Taf13 | 0.00145 | 1.1059847 |
| Olfr181 | 0.00144 | -1.30017714 |
| Cd68 | 0.01333 | -0.65318929 |
| Mina | 0.26776 | 0.18404165 |
| Echdc1 | 0.00144 | 1.44137481 |
| Asb17 | 0.79054 | -0.29354395 |
| Snx31 | 0.00144 | -1.30017714 |
| Cyp2d26 | 0.05929 | -0.5591377 |
| Fbxl8 | 0.00381 | -0.97773226 |
| Rmdn2 | 0.00213 | 0.83555323 |
| Snx10 | 0.00144 | 1.112034 |
| 9330151L19Rik | 0.00367 | 0.87269553 |
| Adh6-ps1 | 0.00144 | -1.26560808 |
| Wdr44 | 0.01013 | 1.24935591 |
| Kif16b | 0.00454 | 0.90364922 |
| Pa2g4 | 0.00489 | -0.76435295 |
| Chadl | 0.16407 | -2.11719083 |
| Fam26d | 0.02624 | -1.8586312 |
| Spred1 | 0.00846 | 1.03681197 |
| Synpo2 | 0.26996 | -0.22855542 |
| Il17re | 0.03684 | -0.56861574 |
| Deb1 | 0.0772 | -0.37442099 |
| Ap3b1 | 0.03984 | 0.41083998 |
| Toporsos | 0.01315 | -1.17842634 |
| Aga | 0.12265 | 0.62715431 |
| Cstf2 | 0.99252 | -0.00200706 |
| Nckap1l | 0.41861 | -0.17385543 |
| 1700003F12Rik | 0.00144 | -1.29878664 |
| Mettl5os | 0.39153 | 0.44090954 |
| Olfr1462 | 0.0285 | -3.39190515 |
| Ofcc1 | 0.00144 | -1.27730451 |
| Sqstm1 | 0.03966 | -0.53442661 |
| Naa30 | 0.00144 | 1.37431622 |
| Usp36 | 0.00144 | -1.71472311 |
| Amacr | 0.30483 | 0.53329259 |
| Olfr1055 | 0.00144 | -1.27699683 |
| Zfp644 | 0.56839 | 0.10536241 |
| Eef1a1 | 0.14892 | -0.30763344 |
| Ahctf1 | 0.00144 | 1.80684174 |
| Drg2 | 0.27171 | -0.31073045 |
| Dgcr6 | 0.00667 | -0.89699837 |
| Wdr6 | 0.0108 | -0.67943034 |
| Wdr82 | 0.03681 | -0.39383557 |
| S100g | 0.00144 | -1.31884045 |
| 2700049A03Rik | 0.09289 | 0.29777109 |
| Pitpnm3 | 0.01148 | -1.49533741 |
| Spag16 | 0.00144 | -1.26560808 |
| Ppp1r18 | 0.28306 | 0.20898476 |
| Zxda | 0.00144 | 2.29607063 |
| Scpep1 | 0.74892 | -0.05641029 |
| Themis3 | 0.00144 | -1.26560808 |
| Sncg | 0.00144 | -1.2000711 |
| Sgk1 | 0.97754 | 0.00633142 |
| Lmo3 | 0.00144 | -1.30017714 |
| Zbtb43 | 0.02913 | -0.46703672 |
| Elmod1 | 0.00464 | -1.24291503 |
| Pfkm | 0.05786 | -0.44974087 |
| Leap2 | 0.02241 | -1.04309434 |
| Fam20c | 0.1141 | -0.55147318 |
| Olfr933 | 0.09653 | -0.43637342 |
| Bik | 0.09794 | -0.52515409 |
| Galt | 0.00645 | -0.95017093 |
| Figf | 0.72012 | 0.0804781 |
| Camsap2 | 0.00145 | 1.19508055 |
| 5830468F06Rik | 0.71857 | -0.26975626 |
| Ethe1 | 0.40009 | -0.37527213 |
| Rab5a | 0.00144 | 1.71481879 |
| Cables1 | 0.01868 | -0.60083644 |
| Slc27a1 | 0.09211 | -0.60346624 |
| Bpifa3 | 0.28486 | -0.75582677 |
| Zfp354a | 0.00191 | 1.10654939 |
| Zdhhc2 | 0.00992 | 1.01933917 |
| 6330409D20Rik | 0.39934 | -0.56912595 |
| Naga | 0.02326 | -0.58738861 |
| Olfr53 | 0.01234 | -1.10835412 |
| Ilvbl | 0.38745 | -0.37549291 |
| Ephx2 | 0.00377 | -0.92877805 |
| Lad1 | 0.50378 | 0.33427681 |
| Rapgef3 | 0.00469 | -0.93364822 |
| Olfr92 | 0.00144 | -1.28898079 |
| Cyp17a1 | 0.00144 | -1.80268081 |
| Olfr1240 | 0.00415 | -1.06195552 |
| 1700010I14Rik | 0.29139 | 0.55277606 |
| Dpt | 0.12027 | 0.32124534 |
| 4933411K16Rik | 0.34972 | -0.27041279 |
| Rbm14 | 0.00209 | -1.00317399 |
| Tex12 | 0.00144 | -1.53131709 |
| Cfap69 | 0.00144 | 2.86884716 |
| Ccdc22 | 0.02459 | -0.49461123 |
| Lsm4 | 0.00534 | -0.95058989 |
| Coq6 | 0.96628 | 0.03455297 |
| Xpo6 | 0.14746 | 0.50562688 |
| Abcc5 | 0.01455 | 0.69082034 |
| Dhrs7b | 0.00144 | -1.08907837 |
| Gm19665 | 0.14555 | -0.60918938 |
| P2rx3 | 0.11683 | 1.10972414 |
| Lrig1 | 0.67278 | -0.10697863 |
| Rexo2 | 0.72016 | -0.05593101 |
| 4921513I03Rik | 0.00144 | -1.45426645 |
| Med27 | 0.00542 | -0.79528311 |
| Hdac2 | 0.47954 | 0.25956844 |
| Ctxn3 | 0.02812 | -0.82819921 |
| L3mbtl3 | 0.01685 | -0.54903915 |
| Disp2 | 0.00144 | -1.30713006 |
| Tbcb | 0.00767 | -0.80705248 |
| Ralgps2 | 0.55385 | 0.09986732 |
| Rragd | 0.76497 | 0.10977236 |
| Usp42 | 0.00192 | -1.21383057 |
| Slc5a5 | 0.00144 | -1.33632959 |
| Asb2 | 0.11977 | -0.70346063 |
| Fam83h | 0.00219 | -0.96491296 |
| Iqcd | 0.00915 | -0.66287755 |
| Shank3 | 0.15825 | 0.34684471 |
| Scamp2 | 0.00186 | -0.8483188 |
| Olfr339 | 0.02122 | -0.96350362 |
| Apol8 | 0.07044 | -2.32283133 |
| Dpysl3 | 0.02701 | -0.49189428 |
| Pot1b | 0.42859 | 0.38323098 |
| P2rx6 | 0.72806 | -0.06723913 |
| Trim63 | 0.10843 | -0.65104531 |
| Surf4 | 0.00459 | -0.85818482 |
| Ankrd26 | 0.50682 | 0.21024509 |
| Rif1 | 0.073 | 0.71190174 |
| Vmn1r81 | 0.0284 | -2.1879863 |
| Scara3 | 0.01098 | -1.0152386 |
| Fam162a | 0.13884 | -0.28689411 |
| Nup153 | 0.00517 | 0.82633153 |
| Edn1 | 0.68813 | 0.11079977 |
| Prss37 | 0.53606 | 0.24985724 |
| Cstb | 0.0242 | -0.57901958 |
| Defb12 | 0.00144 | -1.27165604 |
| Ikbkap | 0.86823 | 0.03709265 |
| Clns1a | 0.27437 | 0.37306427 |
| H1fx | 0.01129 | -1.24532345 |
| Cdh13 | 0.00729 | -0.66302143 |
| Ccna1 | 0.00144 | -1.27165604 |
| Cpne1 | 0.03332 | -0.61912647 |
| Hao2 | 0.00158 | -1.2446827 |
| Trim13 | 0.05314 | 0.45131924 |
| Parm1 | 0.00144 | -0.97571629 |
| Amz1 | 0.00209 | -1.0091321 |
| Batf | 0.07129 | -0.39827726 |
| Ccl17 | 0.00315 | -2.28832214 |
| Rbbp7 | 0.00688 | 1.01787859 |
| Ctu1 | 0.01707 | -0.63686424 |
| Dusp8 | 0.01369 | -0.78885868 |
| Ppt1 | 0.69412 | -0.0766398 |
| Kifc3 | 0.06764 | -0.55369287 |
| Cpsf6 | 0.00144 | 1.37797762 |
| Ccdc57 | 0.10461 | -0.37803748 |
| Il2 | 0.01726 | -1.45179897 |
| Eci3 | 0.02079 | -0.56169601 |
| Foxn1 | 0.0164 | -0.77209214 |
| Clstn3 | 0.07382 | -0.50459635 |
| Tmem109 | 0.00284 | -0.9770888 |
| Bcl7c | 0.04131 | -0.68007104 |
| Olfr348 | 0.00145 | -1.22376446 |
| Gkn2 | 0.00144 | -1.23270322 |
| Jakmip1 | 0.00144 | -1.27454685 |
| Prpf19 | 0.4533 | -0.29427587 |
| Slx1b | 0.03189 | -0.47240076 |
| Myt1l | 0.83127 | -0.22703745 |
| Uaca | 0.00158 | 0.90195509 |
| Slc26a11 | 0.97966 | 0.00465516 |
| Olfr421-ps1 | 0.00144 | -1.29085005 |
| Hps6 | 0.00191 | -1.00691212 |
| F13b | 0.00144 | -1.29746368 |
| Upk1b | 0.12434 | -0.87369633 |
| Ap1s2 | 0.00877 | 1.18668666 |
| Atp1b4 | 0.00144 | -1.31884045 |
| Olfr1131 | 0.1368 | 0.46483224 |
| Cryba1 | 0.0385 | -2.09235163 |
| Cenpq | 0.00144 | 2.03188837 |
| Ch25h | 0.38465 | -0.33796718 |
| Ttc27 | 0.10407 | 0.41515868 |
| Cyp27a1 | 0.7381 | -0.15656942 |
| Il1f5 | 0.01086 | -1.64046339 |
| Slc5a3 | 0.44151 | -0.16841154 |
| Slc7a8 | 0.00144 | -1.26078455 |
| Olfr159 | 0.00241 | -1.26581942 |
| Sp3 | 0.00716 | 0.81762212 |
| St6galnac2 | 0.6337 | 0.09616614 |
| Spire2 | 0.00144 | -1.33632959 |
| Polr1c | 0.01348 | -0.7421346 |
| Pitpnm2 | 0.00144 | -1.02869695 |
| Piezo2 | 0.00159 | -1.26431393 |
| Wdr3 | 0.00568 | 0.71910355 |
| Igtp | 0.86019 | -0.03135712 |
| Hoxd11 | 0.00144 | -1.26934956 |
| 1810011O10Rik | 0.70403 | 0.10363566 |
| Angel1 | 0.04852 | -0.48026694 |
| Gsta4 | 0.27908 | -0.26813312 |
| Ahi1 | 0.92943 | 0.06011159 |
| B230217C12Rik | 0.14201 | -0.38514294 |
| Apopt1 | 0.5043 | 0.24428932 |
| Bean1 | 0.00158 | -0.96390573 |
| Nudt19 | 0.92234 | 0.03062347 |
| Zic5 | 0.00144 | -1.28116048 |
| Slc22a17 | 0.01723 | -0.69576114 |
| Gcfc2 | 0.00144 | 1.68247606 |
| Gpr149 | 0.00144 | -1.33059316 |
| Dstyk | 0.05399 | 0.38254668 |
| Rin3 | 0.51244 | -0.12048863 |
| Sucla2 | 0.00144 | 1.60082623 |
| Srp14 | 0.00431 | -0.71511481 |
| Ubash3a | 0.59074 | -0.33598831 |
| Gfod2 | 0.21945 | -0.31107733 |
| Arl15 | 0.45452 | 0.15338713 |
| Wdr77 | 0.95341 | 0.01881032 |
| Akr1c19 | 0.03042 | 0.78928387 |
| C030040A22Rik | 0.02157 | -1.51405921 |
| Loxl3 | 0.10867 | -0.32135533 |
| Tmem87a | 0.04117 | 0.7952179 |
| Il1f8 | 0.05482 | -0.87082785 |
| Cdk2ap2 | 0.02388 | -0.75149939 |
| Olfr606 | 0.00739 | -2.33242168 |
| Smc4 | 0.00295 | 1.69192985 |
| Cyp2d10 | 0.48267 | -0.22958969 |
| Rnd1 | 0.84421 | -0.04923488 |
| Skint2 | 0.00144 | -1.26301121 |
| Olfr478 | 0.00144 | -1.29085005 |
| Heatr5b | 0.00252 | -0.91288969 |
| 4921524J17Rik | 0.0019 | 0.9671307 |
| Dock4 | 0.35632 | -0.72245358 |
| Znrd1as | 0.02176 | 0.63560579 |
| 1700010D01Rik | 0.00527 | -2.60466328 |
| Olfr1404 | 0.00144 | -1.27165604 |
| Olfr926 | 0.00158 | -1.17515383 |
| Mrps35 | 0.80185 | 0.07025865 |
| Nudt9 | 0.25026 | -0.25622131 |
| Slc1a1 | 0.51054 | 0.1385608 |
| Mst1r | 0.25026 | -0.24036424 |
| Zfp13 | 0.00145 | -1.20374009 |
| Klk1b11 | 0.00144 | -1.20555712 |
| Rab3a | 0.79593 | -0.12017281 |
| Ddah1 | 0.82235 | -0.12113993 |
| Hes5 | 0.00144 | -1.26560808 |
| Cd207 | 0.01353 | -1.46775591 |
| Scgb3a1 | 0.02707 | -0.59774528 |
| Vsig1 | 0.54069 | -0.39376174 |
| C3 | 0.00489 | -0.89430841 |
| Lingo2 | 0.00145 | -1.22376446 |
| Sf3b2 | 0.00144 | -1.45489501 |
| Qsox2 | 0.09913 | -0.40005472 |
| Ttyh2 | 0.65621 | -0.23293041 |
| Ing5 | 0.01761 | -0.65314445 |
| Gm9777 | 0.05587 | -0.66003199 |
| Vmn1r25 | 0.05933 | -0.45335355 |
| Dmrta2 | 0.00144 | -1.29085005 |
| Pik3r4 | 0.13853 | 0.34935449 |
| Apoa1bp | 0.01017 | -0.80041077 |
| Tekt2 | 0.26321 | 0.85753935 |
| Rabgef1 | 0.98166 | -0.00529802 |
| Coro1b | 0.01458 | -0.65650207 |
| Vtn | 0.0068 | -0.83481678 |
| Uqcrc1 | 0.00144 | -1.08431157 |
| Vcp | 0.43804 | 0.13769979 |
| Neil1 | 0.38239 | -0.2048783 |
| Neil1 | 0.67793 | -0.13433138 |
| Olfr687 | 0.00144 | -1.27165604 |
| E130308A19Rik | 0.73538 | -0.06704136 |
| Havcr2 | 0.01304 | 1.04093164 |
| Ltb4r1 | 0.00655 | -1.7423598 |
| Eif2d | 0.5517 | -0.19584941 |
| Copa | 0.14289 | 0.30348419 |
| Pcdhb3 | 0.08086 | 0.45854623 |
| Vmn1r233 | 0.00144 | -1.29085005 |
| Cys1 | 0.08053 | -0.60237323 |
| Os9 | 0.03784 | -0.38702714 |
| Erich2 | 0.00144 | -1.28898079 |
| Olfr109 | 0.00144 | -1.28116048 |
| Paqr4 | 0.00357 | -0.92239113 |
| 4931414P19Rik | 0.11194 | -0.54865104 |
| Iars2 | 0.00537 | 0.69402725 |
| Ccdc86 | 0.12629 | -0.41236165 |
| Nufip1 | 0.41983 | 0.15714506 |
| Lyplal1 | 0.64973 | 0.24698391 |
| S100a5 | 0.00144 | -1.31495282 |
| 4931409K22Rik | 0.00145 | -1.22376446 |
| Polr2h | 0.64325 | -0.14282873 |
| Pon1 | 0.11693 | -1.45926182 |
| Prl3b1 | 0.63948 | -0.24321901 |
| Adrbk2 | 0.02986 | 0.63392919 |
| Cfap20 | 0.74444 | 0.08099416 |
| Mrps18c | 0.58788 | 0.08744494 |
| Gpr65 | 0.04431 | 0.9083571 |
| Ghrh | 0.776 | 0.17128483 |
| Slc25a4 | 0.00298 | -0.78474114 |
| Msantd4 | 0.01203 | 0.60993967 |
| Actn2 | 0.34779 | -0.33896699 |
| Gpsm2 | 0.68064 | -0.13083351 |
| Chrna7 | 0.00144 | -1.33632959 |
| Wfdc21 | 0.81046 | -0.12509638 |
| Tnmd | 0.34715 | -0.65565707 |
| Zbtb4 | 0.03867 | 0.43990135 |
| Lrrc25 | 0.00648 | -0.93547417 |
| Vmn2r1 | 0.00144 | -1.29746368 |
| Plb1 | 0.00144 | -1.28898079 |
| Zmynd15 | 0.116 | -0.37911056 |
| Ubr1 | 0.00342 | -0.86715964 |
| Kdsr | 0.08667 | 0.35342143 |
| Nav2 | 0.91372 | -0.04526065 |
| 2610303G11Rik | 0.25341 | -0.67577354 |
| Mcf2l | 0.00823 | 0.73878424 |
| Slc44a4 | 0.11561 | -2.92665932 |
| Olfr571 | 0.00303 | -0.86178794 |
| Bhlha9 | 0.00144 | -1.37833222 |
| Ccdc50 | 0.10353 | 0.34954968 |
| Mrpl34 | 0.13308 | -0.56865053 |
| Socs2 | 0.09639 | 0.44111345 |
| Oxct1 | 0.02816 | -0.46342786 |
| Slc5a8 | 0.08566 | -3.32320379 |
| Arl3 | 0.75845 | 0.089243 |
| Abcf1 | 0.47136 | -0.14056635 |
| Slc25a54 | 0.00144 | -1.27699683 |
| Krt35 | 0.00144 | -1.30713006 |
| BC031181 | 0.76565 | -0.09699344 |
| Ubqln3 | 0.14221 | -0.6724031 |
| 4930555G01Rik | 0.00144 | 2.0194684 |
| Kif5a | 0.06409 | 0.39267002 |
| 9330178D15Rik | 0.0378 | -0.93613993 |
| Rbm26 | 0.00263 | 1.58407678 |
| Lsm10 | 0.02362 | -0.77164652 |
| Kif21b | 0.00144 | -1.86224005 |
| Pparg | 0.54124 | 0.16851212 |
| Cttnbp2 | 0.36848 | 0.56998717 |
| Celf3 | 0.00144 | -1.56688218 |
| Htra3 | 0.00145 | -0.97867798 |
| Fam195a | 0.02858 | -0.67513703 |
| Speg | 0.72468 | -0.05845185 |
| Nr0b1 | 0.0037 | -1.11214255 |
| Nsmce4a | 0.28774 | -0.16528506 |
| Nsmce4a | 0.06374 | -0.34591843 |
| Tnfrsf13b | 0.00144 | -1.43490912 |
| Camk1 | 0.23662 | -0.35268335 |
| Cmtm2b | 0.00149 | -1.22582153 |
| Zfp64 | 0.00267 | -0.84813163 |
| Traf4 | 0.00145 | -1.18777496 |
| Tnfrsf1a | 0.00489 | -0.73491158 |
| Tbx6 | 0.83796 | -0.06450757 |
| Syngr2 | 0.72949 | -0.19437169 |
| Rasl12 | 0.00144 | -1.07053446 |
| Lax1 | 0.03871 | -1.17326465 |
| Col6a6 | 0.04854 | -0.69754057 |
| Cited2 | 0.06898 | 0.51924129 |
| Gtf3c5 | 0.44217 | -0.16600236 |
| Ece1 | 0.02588 | -0.78913637 |
| Trip13 | 0.03535 | 1.08854182 |
| Atp6v1h | 0.00577 | 0.76703012 |
| Eci1 | 0.3064 | -0.34280064 |
| Crp | 0.01607 | -1.02218921 |
| Nomo1 | 0.00144 | -0.97707576 |
| Alg14 | 0.08634 | -0.29817349 |
| 2010005H15Rik | 0.34614 | -0.59191007 |
| Aasdh | 0.70194 | -0.12440287 |
| Nat14 | 0.44157 | -0.15234684 |
| S100a4 | 0.00144 | -1.54728628 |
| Lypd6b | 0.04028 | -0.5910307 |
| Rad52 | 0.0106 | 0.58631785 |
| Olfr212 | 0.00144 | -1.30713006 |
| Trub2 | 0.13964 | -0.44508265 |
| Itpr3 | 0.00605 | -0.87614827 |
| Lingo1 | 0.09861 | -0.80894128 |
| Rsph6a | 0.00305 | -1.69227187 |
| Gtf2h4 | 0.00985 | -0.81953252 |
| Gpr12 | 0.11347 | -0.79997687 |
| Sspo | 0.75379 | 0.19627529 |
| Gh | 0.00369 | -2.14134209 |
| Krtap6-5 | 0.00217 | -1.1219484 |
| Rnase6 | 0.08337 | -0.82470882 |
| Olfr148 | 0.00144 | -1.27165604 |
| Atrip | 0.00455 | -0.75354624 |
| Dusp10 | 0.25221 | -0.38848787 |
| Rpp25 | 0.00561 | -0.96537337 |
| Gm36633 | 0.30326 | -0.41281749 |
| Sumo1 | 0.26576 | 0.19487362 |
| Gfod1 | 0.07413 | -0.51546089 |
| Fam76b | 0.00144 | 1.84320086 |
| Usp13 | 0.33069 | 0.25839172 |
| Tmem158 | 0.00187 | -1.11590686 |
| Mpc2 | 0.7508 | -0.09076915 |
| Ppp1r35 | 0.00144 | -1.25459666 |
| Fbn2 | 0.63838 | -0.66300112 |
| Sft2d2 | 0.26941 | 0.1850032 |
| Kat8 | 0.18951 | -0.21531938 |
| Dpp6 | 0.54069 | -0.32018937 |
| Ip6k1 | 0.01955 | -0.48666113 |
| Cyp2c29 | 0.00144 | -1.30017714 |
| Trim8 | 0.00144 | -1.04616837 |
| Fam162b | 0.00149 | -1.19348234 |
| Gm16486 | 0.40951 | -0.42584466 |
| Cacna1s | 0.00343 | -1.06507336 |
| Cyb561d2 | 0.00872 | -0.96624084 |
| Spesp1 | 0.00144 | -1.28116048 |
| Rsph4a | 0.00144 | -1.55701652 |
| Vwf | 0.03132 | -0.66535842 |
| Svs1 | 0.00144 | -1.25118919 |
| Zkscan2 | 0.54763 | 0.24716036 |
| Rest | 0.00665 | 0.79786065 |
| Slc39a4 | 0.00345 | -1.07635548 |
| Ttc7 | 0.02151 | -0.84597769 |
| Adamts19 | 0.01807 | 0.74728006 |
| Penk | 0.00146 | -1.27177856 |
| Hoxa11 | 0.00144 | -1.2784435 |
| Muc5ac | 0.90972 | -0.08863135 |
| Herpud2 | 0.40896 | 0.14024207 |
| C1qc | 0.00288 | -0.96483759 |
| Bckdhb | 0.04415 | 0.96595264 |
| 1700105P06Rik | 0.00144 | -1.44283895 |
| 2310007H11Rik | 0.00144 | -1.28797948 |
| Tmem225 | 0.00144 | -1.25927895 |
| Vps33a | 0.45946 | 0.15660934 |
| Pglyrp2 | 0.48842 | -0.56032024 |
| Jph3 | 0.52421 | -0.40400929 |
| Ugt2b36 | 0.00144 | -1.29085005 |
| Clcf1 | 0.00174 | 1.48736473 |
| Samd11 | 0.00144 | -1.25118919 |
| Zbtb41 | 0.03024 | 1.84604631 |
| Tyr | 0.00849 | -1.8549422 |
| Tns1 | 0.42151 | 0.26875147 |
| Myog | 0.00144 | -1.30626366 |
| Olfr495 | 0.00144 | -1.27891018 |
| Tex10 | 0.04972 | 1.05736624 |
| Ccdc85a | 0.00284 | 0.96230586 |
| Lgals3 | 0.00144 | -1.37391636 |
| Herc4 | 0.00144 | 1.6626057 |
| Smarcd1 | 0.54068 | -0.18915853 |
| Stub1 | 0.00432 | -0.86045857 |
| 4930469K13Rik | 0.00144 | -1.28116048 |
| Adamtsl5 | 0.02891 | -0.49340455 |
| Klc4 | 0.13414 | -0.37964811 |
| Hgfac | 0.48012 | 0.34718354 |
| Tfr2 | 0.0134 | -1.7093186 |
| Dsp | 0.01552 | 0.77554418 |
| St6gal1 | 0.09489 | -0.86048351 |
| Pls1 | 0.0015 | -1.15488942 |
| Prmt3 | 0.00491 | 0.73790731 |
| Gmeb2 | 0.23852 | -0.20601394 |
| Psme1 | 0.11044 | -0.38887935 |
| Serpinb2 | 0.00144 | -1.28898079 |
| Samd14 | 0.00158 | -0.90859215 |
| Cnppd1 | 0.00144 | -0.98033022 |
| Htr7 | 0.04109 | -0.6331169 |
| Olfr640 | 0.00144 | -1.3421574 |
| Ak8 | 0.0384 | -1.00214209 |
| Serpinb3c | 0.45977 | 0.33879886 |
| Gucy2c | 0.27621 | -0.24592538 |
| Fn1 | 0.01541 | -0.52401514 |
| Fam26f | 0.02637 | -1.23143833 |
| Aqr | 0.05103 | -0.52188333 |
| Prdx3 | 0.85355 | -0.04354766 |
| Snw1 | 0.499 | 0.11832333 |
| Snw1 | 0.19953 | -0.26434964 |
| Zfp169 | 0.01206 | -1.26915226 |
| Apon | 0.0475 | -3.27353365 |
| Krtap9-3 | 0.00193 | -1.35393509 |
| Map3k3 | 0.14098 | -0.34680708 |
| Prss58 | 0.00592 | -1.55889052 |
| 4930542C16Rik | 0.04929 | -2.08644492 |
| Gm31251 | 0.72942 | 0.18304539 |
| Tap1 | 0.00372 | -1.13638571 |
| Oprm1 | 0.00144 | -1.28116048 |
| Srf | 0.07002 | -0.59479713 |
| Opcml | 0.69676 | -0.1237726 |
| Mns1 | 0.06262 | 1.60929717 |
| Mif4gd | 0.01423 | -0.87317179 |
