# Supplementary material for: UTP14A, DKC1, DDX10, PinX1, and ESF1 Modulate Cardiac Angiogenesis Leading to Obesity-Induced Cardiac Injury
Source: J Diabetes Res. 2022 Jun 13;2022:2923291. doi: 10.1155/2022/2923291 (PMC9208995; doi:10.1155/2022/2923291)
Supplement: Supplementary Materials — GSE98226 datasets were downloaded from GEO (http://www.ncbi.nlm.nih.gov/geo/). Supplemental File 1: DEGs. Supplemental File 2: GO enrichment analysis. Supplemental File 3: KEGG pathway analysis. Supplemental File 4: correlation between UTP14A, DKC1, DDX10, PinX1, and ESF1 expression and cardiac angiogenesis. [file 2923291.f1.zip › Supplemental File 2 (1).docx]

Supplemental File 2 : GO Enrichment Analysis

| Category | Term | Count | PValue | Genes | Fold Enrichment |
| --- | --- | --- | --- | --- | --- |
| GOTERM_MF_DIRECT | GO:0003676~nucleic acid binding | 94 | 5.20E-20 | N6AMT1, GM14322, ZFP445, ZCCHC11, RBM25, PRDM9, RBM26, ZFP407, GM14326, ZFP329, PINX1, GM14325, PRDM5, CHD6, ZBTB20, CHD5, AW146154, ZFP119A, ALKBH8, ZFP119B, BC002059, TBK1, ZFP74, ZFP160, ZFP874A, DHX15, ZFP120, ZFP442, ZFP760, ZFP810, ZFP931, GM14410, ZFP930, ZFP455, ZFP935, ZFP934, ZFP932, DDX10, ZFP938, ZFP936, GM14296, DDX50, GM20939, PTBP3, SFPQ, ZEB1, ZFP40, MATR3, ZSCAN26, GM10778, ZFP575, ZFPM2, ZCCHC7, AU041133, YTHDC2, ZFP948, RNPC3, GM38396, WRN, ZMAT1, ZFP182, ZFP52, ZFP51, 2410141K09RIK, ZFP53, DHX36, ZFP101, ZFP386, ZFP781, TTC14, RBM39, ZRSR2, ZFP677, EGR1, EGR2, NPM1, ZFP433, ZFP950, EGR3, ZFP758, GM14391, ZFP719, ZFP959, ZFP958, SMARCA1, GM14393, CNOT4, SYNJ1, FUBP1, HNRNPA2B1, EU599041, ZFP273, ZFP157, TARDBP | 2.802804986 |
| GOTERM_BP_DIRECT | GO:0006355~regulation of transcription, DNA-templated | 110 | 2.00E-10 | ZFP326, GM14124, TCERG1, ZFP445, PRDM9, CCNT2, ZFP329, PRDM5, ZBTB20, ARID4B, AW146154, ZFP119A, CCAR1, ZFP119B, YY2, HMGN5, ZFP160, CCNL1, ZFP442, GM14410, ZFP455, RALGAPA1, GM20939, DMTF1, ZFP40, ZSCAN26, ZFP575, ZFPM2, CLOCK, ASF1A, KMT2E, INO80B, GCM1, DLX6, ZFP182, ZFP52, ZFP51, ZFP53, DHX36, ZFP101, SCAI, BPTF, EGR1, EGR2, EGR3, ZFP758, GM14391, NR1D2, SMARCA1, GM14393, CNOT4, FUBP1, FEV, GM14322, SETD2, GM14326, GM14325, CHD6, CHD5, FAM208A, MYSM1, SCML2, ZFP74, ZFP874A, SNAPC3, RB1CC1, ZMYM5, NFKBIZ, ZFP120, ZFP280D, ZFP760, ZFP810, ZFP931, ZFP930, TSC22D1, ZFP935, ZFP934, ZFP932, ZFP938, ZFP936, GM14296, PHF20L1, SFPQ, ZEB1, RBL1, GM10778, VGLL2, NFAT5, AU041133, ZFP948, GM38396, 2410141K09RIK, ZFP386, ZFP781, RBM39, ZFP677, ZFP950, ZFP433, ZFP719, ZFP959, ZFP958, ASXL3, ESF1, EU599041, ZFP273, SSBP2, ZFP157, TARDBP, MPHOSPH8, TRIM33 | 1.833529375 |
| GOTERM_MF_DIRECT | GO:0046872~metal ion binding | 145 | 1.81E-09 | ZFP326, GM14124, ITSN2, ZFP445, ATP8A2, PRDM9, ZFAND4, ZFP329, RFESD, PRDM5, LTN1, ZBTB20, AW146154, ZFP119A, ALKBH8, ZFP119B, BC002059, YY2, ZFP160, NEK1, CAR8, ZFP442, TRIM23, PRKCG, GM14410, POSTN, ZFP455, ESCO1, ZBTB38, LIG4, ATP11C, GTPBP8, YOD1, QTRTD1, GM20939, RC3H2, RNF125, NAIP6, ZFP40, MATR3, ZSCAN26, AMY1, ZFP575, ZFPM2, KMT2E, INO80B, ZCCHC7, GCM1, ADAMTS12, ZMAT1, ZFP182, ZFP52, ZFP51, ZFP53, TRPM7, ZFP101, EDEM3, EGR1, ZRSR2, EGR2, EGR3, ZFP758, GM14391, MICAL2, LNPEP, NR1D2, CP, GM14393, CNOT4, ALB, GNAS, PHF14, GM14322, ZCCHC11, RBM26, ZFP407, GM14326, CPQ, GM14325, CHD5, EFCAB14, ZC3H6, MYSM1, ADAMTS5, ZFP74, ZC3H7A, ZFP874A, ZMYM5, ZFP120, TRIM69, ZFP280D, ZFP760, RBM6, ZFP810, ZFP931, FGA, ZFP930, APOBEC3, ZFP935, ZFP934, PDE4D, ZFP932, ITGA1, ZFP938, ZFP936, GM14296, YDJC, PHF20L1, ZEB1, CYP21A1, TAX1BP1, FAM20B, GM10778, THOP1, AU041133, UHRF2, ROCK2, PON2, RPE, ZFP948, PRICKLE1, GM38396, WRN, ZNRF3, 2410141K09RIK, PLAGL1, PGGT1B, PDLIM5, MBNL3, EPS15, ZFP386, GH, ZFP677, ZFP950, ZFP433, ZFP719, ZFP959, ZFP958, CDC42BPA, TMLHE, ASXL3, EU599041, ZFP273, ZFP157, TRIM33 | 1.594080338 |
| GOTERM_CC_DIRECT | GO:0005622~intracellular | 82 | 7.96E-09 | GM14322, GM14124, ZFP445, PRDM9, GM14326, LRRK2, GM14325, AW146154, SENP7, DOCK11, ZFP74, ZFP160, ZFP874A, ZFP120, CAR8, ZFP442, ZFP760, TRIM23, ZFP810, ZFP931, PRKCG, GM14410, GUCY1A2, ZFP930, ZFP455, ZFP935, ZFP934, ZFP932, BC005561, ATG10, ZFP938, TRNT1, ZFP936, GM14296, GM20939, RNF125, PIK3CA, ZFP40, EXOC4, GM10778, AU041133, ROCK2, SAA3, ZFP948, MTIF2, AKAP5, GM38396, SLFN9, WRN, ZMAT1, ZFP182, ARL13B, ZFP52, ZFP51, 2410141K09RIK, ZFP53, EIF2S3Y, STX3, EVI5, ZFP101, ZFP386, SOCS4, ZFP781, TBC1D8B, ZFP677, ZFP433, ZFP950, ZFP758, CAV2, GM14391, XPO7, LNPEP, ZFP958, CDC42BPA, GM14393, BCL2, EU599041, ZFP273, ZFP157, OGT, TRIM33, RPS24 | 1.942789388 |
| GOTERM_MF_DIRECT | GO:0044822~poly(A) RNA binding | 62 | 1.05E-07 | ZFP326, TCERG1, ZCCHC11, RBM26, HNRNPU, HNRNPR, FAM208A, MKI67, CCAR1, RPGR, IFIT2, LGALS3, HMGN5, ZC3H7A, DHX15, UTP14A, RBM6, SEC63, PNISR, APOBEC3, DDX10, PRPF4B, DDX50, RC3H2, PCDHGA9, PTBP3, FAM133B, SFPQ, DKC1, MATR3, KCTD12, LUC7L2, ZCCHC7, ROCK2, YTHDC1, CSTF3, YTHDC2, NOL7, MTIF2, MNDAL, FIP1L1, DHX36, MBNL3, SPTBN1, TRMT10A, RBM39, NOP58, NPM1, API5, HIST1H4M, PCDH20, UPF3B, ANKHD1, CNOT4, FUBP1, CAPRIN1, HNRNPA2B1, MPHOSPH10, ESF1, R3HDM1, TARDBP, RPS24 | 2.054618776 |
| GOTERM_CC_DIRECT | GO:0005634~nucleus | 204 | 1.60E-05 | TCERG1, FOXI3, CCNF, HNRNPU, HNRNPR, SMC3, MKI67, ALKBH8, LGALS3, CCNL1, TRIM23, PRKCG, ESCO1, YOD1, HECW2, ZSCAN26, KIF20B, ZFPM2, CLOCK, ASF1A, MEG3, CSTF3, RNPC3, MNDAL, ZFP182, DHX36, SPTBN1, SCAI, ZRSR2, EGR1, EGR2, EGR3, HIST1H4M, UPF3B, MICAL2, MEMO1, NR1D2, SMARCA1, FUBP1, MPHOSPH10, FBXL3, CDK12, RPS24, ATR, SETD2, ZCCHC11, NIPBL, SCML2, CASP12, ZMYM5, DHX15, ZFP120, UTP14A, UPF2, APOBEC3, TSC22D1, STRADA, PIFO, TSC22D2, ZFP932, ZFP938, CACYBP, PHF20L1, PLCB4, ZEB1, CENPC1, PLCB1, VGLL2, NOL7, AKAP7, PRICKLE1, WRN, UBN2, PLAGL1, MBNL3, FILIP1L, PAK2, GH, TRMT10A, NPM1, GDF15, ZFP719, XPO7, ZFP959, PNMA2, RANBP6, TJP1, PRPF39, NEDD4, DLC1, STK17B, HNRNPA2B1, CCNG1, MPLKIP, TARDBP, MPHOSPH8, CENPQ, ZFP326, ZFP445, CCNT2, PRDM9, ZFP329, PRDM5, ZBTB20, ARID4B, ZFP119A, ZFP119B, YY2, HMGN5, CWC22, NFATC2IP, NEK1, PUS3, PNISR, RALGAPA1, MAGI3, ZBTB38, LIG4, PRPF4B, DMTF1, DKC1, PLAA, MATR3, PHIP, ZFP575, TOX, DGKH, KMT2E, GCM1, INO80B, ZCCHC7, YTHDC1, DLX6, YTHDC2, ZMAT1, EVI5, BPTF, API5, CAV2, CNOT4, CLK1, FEV, ALB, BCL2, GNAS, PHF14, OGT, BRWD3, RBM25, FLT1, PINX1, CHD6, CHD5, FAM208A, DUSP16, MYSM1, GRM1, CDC73, SENP7, ZFP74, RB1CC1, SNAPC3, NFKBIZ, TRIM69, ZFP280D, RBM6, CERS4, VBP1, DDX50, RAD23B, MUP2, PTBP3, SFPQ, RBL1, SPOPL, USP40, LUC7L2, AGPAT5, NFAT5, SLFN5, USP15, UHRF2, ROCK2, PON2, LIN9, FAM169A, SLFN9, MALT1, HECTD2, FIP1L1, RHOX8, GBP3, FKTN, XLR, RBM39, NOP58, SWT1, TNIP1, ASXL3, ESF1, ERCC8, ERCC6, SSBP2, TRIM33 | 1.284003354 |
| GOTERM_BP_DIRECT | GO:0006397~mRNA processing | 21 | 3.54E-04 | ZFP326, RBM39, ZRSR2, RBM25, RBM26, YTHDC1, CSTF3, HNRNPU, PRPF4B, PTBP3, LGALS3, PRPF39, SFPQ, FIP1L1, HNRNPA2B1, CWC22, DHX15, CDK12, MBNL3, PLCB1, TARDBP | 2.477438802 |
| GOTERM_BP_DIRECT | GO:0008380~RNA splicing | 17 | 6.66E-04 | ZFP326, RBM39, ZRSR2, RBM25, YTHDC1, HNRNPU, PRPF4B, PTBP3, LGALS3, PRPF39, SFPQ, HNRNPA2B1, CWC22, DHX15, CDK12, MBNL3, TARDBP | 2.679608773 |
| GOTERM_BP_DIRECT | GO:0050688~regulation of defense response to virus | 4 | 9.14E-04 | APOBEC3, ELMOD2, IFIT1, GBP4 | 18.99369748 |
| GOTERM_BP_DIRECT | GO:0051056~regulation of small GTPase mediated signal transduction | 5 | 0.001073518 | RALGAPA1, IQSEC3, ARHGAP18, CDC42BPA, SIPA1L3 | 10.55205415 |
| GOTERM_CC_DIRECT | GO:0005813~centrosome | 24 | 0.001303211 | IQCB1, ITSN2, CEP126, DYNC1I2, NPM1, ROCK2, PDE4D, SCLT1, 1700029J07RIK, HAUS3, CCP110, RPGRIP1L, RPGR, NIN, ODF2L, WRN, CSPP1, CNTRL, MPLKIP, NEK1, CEP95, RTTN, KIF20B, LRRCC1 | 2.09017341 |
| GOTERM_MF_DIRECT | GO:0032947~protein complex scaffold | 7 | 0.0014217 | HOMER1, CAV2, RB1CC1, MAGI3, PKP2, AKAP7, MPP7 | 5.612740142 |
| GOTERM_BP_DIRECT | GO:0042384~cilium assembly | 11 | 0.002161886 | IQCB1, CEP126, ARL13B, INTU, SCLT1, NEK1, EXOC5, BBIP1, RPGRIP1L, FNBP1L, RPGR | 3.239235229 |
| GOTERM_BP_DIRECT | GO:0048015~phosphatidylinositol-mediated signaling | 6 | 0.002357646 | PIK3CA, INPP5F, SYNJ1, IRS2, CAR8, OGT | 6.331232493 |
| GOTERM_BP_DIRECT | GO:0007049~cell cycle | 29 | 0.003444658 | KMT2E, CCNT2, UHRF2, GM16505, CCNF, LIN9, MKI67, SMC3, CDC73, CCAR1, NIPBL, RB1CC1, CNTRL, NEK1, HMCN1, EVI5, LRRCC1, ESCO1, STRADA, HAUS3, LIG4, CD2AP, RBL1, DMTF1, CENPC1, CCNG1, GAS2, MPLKIP, KIF20B | 1.794192921 |
| GOTERM_CC_DIRECT | GO:0005654~nucleoplasm | 71 | 0.003898902 | ZFP326, PRDM9, GOLIM4, CHD6, ZBTB20, HNRNPU, HNRNPR, HNMT, SMC3, DUSP16, GIMAP8, ALKBH8, HMGN5, ZMYM5, CWC22, LRRCC1, IQCB1, PNISR, STRADA, HAUS3, LIG4, CACYBP, PRPF4B, RAD23B, RC3H2, SFPQ, ZEB1, RBL1, DMTF1, DKC1, FAM20B, PKP2, MATR3, ZSCAN26, KIF20B, ZFPM2, CLOCK, KMT2E, NFAT5, UHRF2, YTHDC1, CSTF3, MTIF2, LIN9, RNPC3, A230046K03RIK, BC005624, WRN, UBN2, PLAGL1, PAK2, BPTF, RBM39, EGR1, EGR2, NPM1, API5, HIST1H4M, MPP5, ANKHD1, TNIP1, FUBP1, HNRNPA2B1, MPLKIP, ESF1, FBXL3, ERCC6, TARDBP, CENPQ, TRIM33, ATR | 1.390073337 |
| GOTERM_MF_DIRECT | GO:0000166~nucleotide binding | 72 | 0.004731033 | RBM25, FLT1, ATP8A2, RBM26, LRRK2, CHD6, CHD5, HNRNPU, HNRNPR, MKI67, SMC3, GIMAP8, ALKBH8, TBK1, FPGT, DHX15, NEK1, RBM6, TRIM23, PRKCG, GUCY1A2, ABCA8B, MAGI3, DDX10, ATP11C, LIG4, GTPBP8, PRPF4B, TRNT1, DDX50, PTBP3, SFPQ, PIK3CA, NAIP6, FAM20B, MATR3, KIF20B, SLFN5, ROCK2, YTHDC2, AKAP7, NLRP9B, MTIF2, RNPC3, WRN, NWD1, ARL13B, GRK4, DHX36, TRPM7, EIF2S3Y, ABCB1A, PAK2, GBP3, RBM39, ZRSR2, TPK1, UPF3B, CDC42BPA, SMARCA1, CLK1, CNOT4, NIN, SYNJ1, STK17B, HNRNPA2B1, GNAS, CDK12, ABCE1, TARDBP, RPS24, ATR | 1.37170863 |
| GOTERM_BP_DIRECT | GO:0060271~cilium morphogenesis | 12 | 0.005373257 | IQCB1, CEP126, ARL13B, INTU, TBC1D32, SCLT1, NEK1, NPHP3, BBIP1, RPGRIP1L, FNBP1L, RPGR | 2.681463174 |
| GOTERM_MF_DIRECT | GO:0008094~DNA-dependent ATPase activity | 5 | 0.006471685 | DHX36, CHD6, ERCC6, SMARCA1, BPTF | 6.586378738 |
| GOTERM_MF_DIRECT | GO:0008026~ATP-dependent helicase activity | 5 | 0.006471685 | WRN, DHX36, DHX15, CHD6, CHD5 | 6.586378738 |
| GOTERM_CC_DIRECT | GO:0031091~platelet alpha granule | 4 | 0.006531423 | TREML1, FGA, VAMP7, STXBP3 | 10.10250482 |
| GOTERM_BP_DIRECT | GO:0048496~maintenance of organ identity | 3 | 0.006533251 | IQCB1, PKP2, NPHP3 | 22.79243697 |
| GOTERM_BP_DIRECT | GO:0045636~positive regulation of melanocyte differentiation | 3 | 0.006533251 | KITL, BCL2, ADAMTS9 | 22.79243697 |
| GOTERM_MF_DIRECT | GO:0004842~ubiquitin-protein transferase activity | 18 | 0.007867476 | TRIM12C, UHRF2, LTN1, KLHL4, MALT1, RC3H2, CNOT4, ZNRF3, RNF125, HECTD2, NAIP6, HECW2, NEDD4, TRIM69, ERCC8, FBXL3, MPHOSPH8, TRIM23 | 2.036524469 |
| GOTERM_BP_DIRECT | GO:0048286~lung alveolus development | 6 | 0.008284531 | MEG3, GH, PHF14, ZFP157, RC3H2, STRA6 | 4.74842437 |
| GOTERM_CC_DIRECT | GO:0005681~spliceosomal complex | 10 | 0.010007173 | ZFP326, LGALS3, ZRSR2, RBM25, API5, HNRNPA2B1, CWC22, DHX15, HNRNPU, PRPF4B | 2.785617137 |
| GOTERM_MF_DIRECT | GO:0008022~protein C-terminus binding | 13 | 0.011794289 | SCLT1, LIG4, AKAP7, MKI67, SYNJ2BP, CD2AP, TJP1, NIPBL, SYNJ1, NEDD4, ERCC6, VGLL2, MPDZ | 2.294202737 |
| GOTERM_BP_DIRECT | GO:0007420~brain development | 13 | 0.012099254 | STXBP3, NOG, IRS2, RPGRIP1L, SMARCA1, HNMT, PTPRG, NIPBL, WRN, SYNJ1, CNTN4, PLCB1, BPTF | 2.2862784 |
| GOTERM_BP_DIRECT | GO:0006396~RNA processing | 7 | 0.012309773 | PRPF39, DKC1, CSTF3, YTHDC2, DHX36, TRNT1, CCNL1 | 3.642626914 |
| GOTERM_MF_DIRECT | GO:1990247~N6-methyladenosine-containing RNA binding | 3 | 0.014016359 | YTHDC1, YTHDC2, HNRNPA2B1 | 15.80730897 |
| GOTERM_MF_DIRECT | GO:0001047~core promoter binding | 7 | 0.015900548 | SFPQ, DHX36, HNRNPU, KANSL1L, CLOCK, MNDAL, CCAR1 | 3.44248062 |
| GOTERM_CC_DIRECT | GO:0005737~cytoplasm | 199 | 0.016503007 | ITSN2, CCNF, HNRNPU, CCP110, HNRNPR, IFIT1, SMC3, FNBP1L, MKI67, ALKBH8, IFIT2, CCAR1, LGALS3, TBK1, CSPP1, CEP95, RTTN, HMCN1, CCNL1, TRIM23, PRKCG, ATG10, LCE1I, QTRTD1, RC3H2, CD2AP, EML5, ASTL, HECW2, ZSCAN26, KIF20B, ZFPM2, CLOCK, MEG3, INTU, STXBP3, MNDAL, SCFD1, INPP5F, DHX36, TRPM7, SPTBN1, SCAI, EGR1, EGR2, HOMER1, IQSEC3, UPF3B, IFI44, BBIP1, TREML1, KITL, FBXL3, SPRR1A, RPS24, ZCCHC11, UCMA, LYST, ZMYM5, CNTRL, DHX15, LRRCC1, UPF2, APOBEC3, TSC22D1, STRADA, PIFO, TSC22D2, CACYBP, PLCB4, ZEB1, VAMP7, CENPC1, GAS2, EXOC4, EXOC5, PLCB1, VGLL2, DYNC1I2, PRUNE2, AKAP7, PRICKLE1, AKAP5, PPP1R2, BC005624, WRN, 4932431P20RIK, MBNL3, FILIP1L, EPS15, PAK2, NPM1, GDF15, KLK13, XPO7, MPP5, RANBP6, TJP1, ETAA1, MKLN1, NEDD4, DLC1, HNRNPA2B1, MPLKIP, TARDBP, MPHOSPH8, CEP126, CCNT2, IRS2, HNMT, ALDH1L2, GIMAP8, TBC1D32, FPGT, CWC22, NFATC2IP, NEK1, CAR8, PNISR, POSTN, RALGAPA1, LIG4, 1700029J07RIK, RPGRIP1L, ERMN, DMTF1, NAIP6, ELMO1, PLAA, MATR3, DGKH, TRIM12C, INO80B, ZCCHC7, FAM114A1, YTHDC2, TTC41, ITPR2, ARHGAP18, BTBD1, GRK4, MPDZ, EVI5, SOCS4, BPTF, API5, CAV2, DENND4C, ANKHD1, CNOT4, CLK1, SYNJ1, CAPRIN1, ALB, BCL2, GNAS, OGT, RBM25, CPQ, LRRK2, PINX1, DUSP16, CDC73, RPGR, RB1CC1, TRIM69, CCDC50, IQCB1, FGA, VBP1, PDE4D, HAUS3, RAD23B, ASPN, SFPQ, SPOPL, USP40, PPP1R12B, THOP1, NFAT5, USP15, ROCK2, NLRP9B, MALT1, FBXL20, NWD1, HECTD2, KRIT1, PDLIM5, GBP3, SPAG9, NOP58, SCLT1, CDC42BPA, NIN, TNIP1, PPP2R2B, SSBP2, ABCE1 | 1.136931718 |
| GOTERM_MF_DIRECT | GO:0008270~zinc ion binding | 42 | 0.016519375 | KMT2E, ZFP326, TRIM12C, GCM1, ZCCHC7, ZCCHC11, ZFP407, ZFAND4, UHRF2, LTN1, CHD5, PRICKLE1, ADAMTS12, ALKBH8, PTMS, MLLT6, ADAMTS5, ZNRF3, ZMAT1, ZMYM5, PGGT1B, TRIM69, CAR8, PDLIM5, ADAMTS9, TRIM23, BPTF, PRKCG, EGR1, APOBEC3, MICAL2, LNPEP, NR1D2, QTRTD1, RC3H2, CNOT4, RNF125, ASTL, ALB, PHF14, MATR3, TRIM33 | 1.441038399 |
| GOTERM_BP_DIRECT | GO:0000278~mitotic cell cycle | 5 | 0.017145761 | PPP2R2B, CNTRL, CENPC1, PRDM5, PAK2 | 4.998341442 |
| GOTERM_CC_DIRECT | GO:0020005~symbiont-containing vacuole membrane | 3 | 0.017458312 | GBP6, GBP7, GBP3 | 14.2066474 |
| GOTERM_CC_DIRECT | GO:0016607~nuclear speck | 12 | 0.020105453 | KMT2E, PNISR, NPM1, RBM25, YTHDC1, CWC22, TRIM69, CDK12, CCNL1, PLCB1, TARDBP, LUC7L2 | 2.217623009 |
| GOTERM_MF_DIRECT | GO:0003723~RNA binding | 32 | 0.021193574 | RBM25, RBM26, YTHDC1, YTHDC2, HNRNPU, IFIT1, RNPC3, ALKBH8, IFIT2, ZMAT1, FIP1L1, DHX36, CWC22, UPF2, PUS3, RBM39, ZRSR2, NPM1, DDX10, TRNT1, DDX50, RC3H2, PTBP3, CNOT4, SFPQ, SYNJ1, DKC1, FUBP1, CAPRIN1, HNRNPA2B1, MATR3, TARDBP | 1.513178295 |
| GOTERM_BP_DIRECT | GO:0048312~intracellular distribution of mitochondria | 3 | 0.02193667 | LRRK2, SYNJ2BP, OGT | 12.66246499 |
| GOTERM_BP_DIRECT | GO:0007216~G-protein coupled glutamate receptor signaling pathway | 3 | 0.02193667 | TRPM1, HOMER1, GRM1 | 12.66246499 |
| GOTERM_BP_DIRECT | GO:0044406~adhesion of symbiont to host | 3 | 0.02193667 | GBP6, GBP7, GBP3 | 12.66246499 |
| GOTERM_BP_DIRECT | GO:0031397~negative regulation of protein ubiquitination | 5 | 0.022127779 | PRKCG, PINX1, SPOPL, OGT, GBP4 | 4.632609141 |
| GOTERM_MF_DIRECT | GO:0004386~helicase activity | 9 | 0.024069937 | WRN, YTHDC2, DHX36, DHX15, CHD6, DDX10, CHD5, SMARCA1, DDX50 | 2.573282856 |
| GOTERM_MF_DIRECT | GO:0003700~transcription factor activity, sequence-specific DNA binding | 35 | 0.025047105 | GM14124, GCM1, TCERG1, NFAT5, ZFP445, FOXI3, ZFP948, ZFP160, ZFP182, ZFP52, ZFP51, 2410141K09RIK, ZFP53, ZFP442, ZFP386, ZFP677, EGR1, ZFP930, EGR2, ZFP433, ZFP935, TSC22D1, ZFP758, ZFP934, ZBTB38, TSC22D2, GM14391, NR1D2, ZEB1, ZFP40, FEV, EU599041, ZFP273, ZSCAN26, CLOCK | 1.461982143 |
| GOTERM_BP_DIRECT | GO:0006283~transcription-coupled nucleotide-excision repair | 3 | 0.026949962 | UVSSA, ERCC8, ERCC6 | 11.39621849 |
| GOTERM_BP_DIRECT | GO:0051726~regulation of cell cycle | 8 | 0.02832583 | NPM1, SFPQ, RBL1, CCNF, CCNG1, BCL2, PLCB1, TARDBP | 2.713385354 |
| GOTERM_CC_DIRECT | GO:0043005~neuron projection | 19 | 0.030113135 | PRKCG, HOMER1, LRRK2, ITGA1, CACYBP, HNMT, GRM1, WRN, VAMP7, SYNJ1, NAIP6, HNRNPA2B1, EXOC4, TRPM7, STX3, PDLIM5, MPDZ, OGT, BPTF | 1.713817781 |
| GOTERM_BP_DIRECT | GO:0071346~cellular response to interferon-gamma | 6 | 0.033052152 | GBP6, CCL8, GBP7, CCL17, GBP4, GBP3 | 3.351828967 |
| GOTERM_CC_DIRECT | GO:0005938~cell cortex | 9 | 0.037064165 | FGA, MKLN1, GRK4, NEDD4, ITPR2, HMCN1, FNBP1L, ERMN, CD2AP | 2.367774566 |
| GOTERM_BP_DIRECT | GO:0006351~transcription, DNA-templated | 63 | 0.037210038 | ZFP326, TCERG1, ZFP445, SETD2, PRDM9, CCNT2, ZFP329, PRDM5, CHD6, ZBTB20, CHD5, ARID4B, FAM208A, MYSM1, CDC73, CCAR1, HMGN5, YY2, ZFP74, RB1CC1, SNAPC3, ZMYM5, NFKBIZ, ZFP120, CCNL1, ZFP280D, TSC22D1, ZFP932, SFPQ, ZEB1, RBL1, DMTF1, ZSCAN26, ZFP575, ZFPM2, VGLL2, CLOCK, ASF1A, KMT2E, INO80B, NFAT5, GCM1, LIN9, ZFP182, DHX36, SCAI, BPTF, RBM39, EGR1, EGR2, EGR3, SWT1, NR1D2, SMARCA1, CNOT4, FUBP1, FEV, ASXL3, ESF1, SSBP2, TARDBP, MPHOSPH8, TRIM33 | 1.269605243 |
| GOTERM_CC_DIRECT | GO:0005730~nucleolus | 32 | 0.038311943 | INO80B, TCERG1, ZCCHC7, ZCCHC11, PINX1, NOL7, MKI67, MALT1, MNDAL, WRN, HMGN5, DHX15, TRIM69, UTP14A, SPTBN1, TRMT10A, ZRSR2, NPM1, NOP58, UPF3B, PNMA2, DDX50, CD2AP, NIN, DKC1, MPHOSPH10, ESF1, CDK12, ERCC6, KIF20B, MPHOSPH8, CLOCK | 1.43978691 |
| GOTERM_BP_DIRECT | GO:0035264~multicellular organism growth | 8 | 0.039046341 | MEG3, ZFP950, MFSD7B, PDE4D, GNAS, NEK1, ERCC6, RC3H2 | 2.532492997 |
| GOTERM_BP_DIRECT | GO:0043484~regulation of RNA splicing | 4 | 0.039710845 | CLK1, ZFP326, CDK12, MBNL3 | 5.239640684 |
| GOTERM_CC_DIRECT | GO:0005694~chromosome | 16 | 0.039798468 | SETD2, PRDM9, ESCO1, PINX1, FAM208A, PRPF4B, MKI67, SMC3, UVSSA, DHX36, CENPC1, MPHOSPH10, CENPQ, MPHOSPH8, CLOCK, ATR | 1.762064794 |
| GOTERM_CC_DIRECT | GO:0016363~nuclear matrix | 7 | 0.043120227 | ZFP326, TCERG1, NPM1, SFPQ, ERCC8, MATR3, SMC3 | 2.733925273 |
| GOTERM_BP_DIRECT | GO:0043161~proteasome-mediated ubiquitin-dependent protein catabolic process | 9 | 0.043212215 | EDEM3, BTBD1, PLAA, ERCC8, SPOPL, FBXL3, CLOCK, RAD23B, CD2AP | 2.294540635 |
| GOTERM_BP_DIRECT | GO:0042832~defense response to protozoan | 4 | 0.043289843 | GBP6, GBP7, LYST, GBP3 | 5.064985994 |
| GOTERM_CC_DIRECT | GO:0097431~mitotic spindle pole | 3 | 0.044595718 | NIN, CNTRL, KIF20B | 8.742552245 |
| GOTERM_MF_DIRECT | GO:0043024~ribosomal small subunit binding | 3 | 0.046783427 | NPM1, MTIF2, ABCE1 | 8.511627907 |
| GOTERM_BP_DIRECT | GO:0042752~regulation of circadian rhythm | 5 | 0.047351805 | PRKCG, SFPQ, ROCK2, FBXL3, NR1D2 | 3.652634131 |
| GOTERM_CC_DIRECT | GO:0000785~chromatin | 8 | 0.049299888 | NIPBL, SFPQ, HMGN5, ESCO1, NEDD4, HNRNPA2B1, CHD5, SMC3 | 2.40535829 |
| GOTERM_BP_DIRECT | GO:0044029~hypomethylation of CpG island | 2 | 0.051849779 | MEG3, PIK3CA | 37.98739496 |
| GOTERM_BP_DIRECT | GO:0048753~pigment granule organization | 2 | 0.051849779 | BCL2, LYST | 37.98739496 |
| GOTERM_BP_DIRECT | GO:0048511~rhythmic process | 8 | 0.052127276 | PRKCG, SFPQ, ROCK2, HNRNPU, FBXL3, NR1D2, CLOCK, OGT | 2.374212185 |
| GOTERM_BP_DIRECT | GO:0007160~cell-matrix adhesion | 6 | 0.054328327 | FGA, MKLN1, SNED1, ITGA10, ITGA1, ADAMTS12 | 2.922107304 |
| GOTERM_BP_DIRECT | GO:0006275~regulation of DNA replication | 3 | 0.057700756 | ESCO1, ZBTB38, SMC3 | 7.597478992 |
| GOTERM_MF_DIRECT | GO:0003924~GTPase activity | 11 | 0.059078275 | GBP6, F830016B08RIK, GBP7, LRRK2, GNAS, MTIF2, GTPBP8, EIF2S3Y, GBP4, TRIM23, GBP3 | 1.94124847 |
| GOTERM_BP_DIRECT | GO:0018105~peptidyl-serine phosphorylation | 8 | 0.061550882 | PRKCG, CLK1, TBK1, LRRK2, BCL2, RICTOR, PAK2, ATR | 2.284956088 |
| GOTERM_BP_DIRECT | GO:0048589~developmental growth | 4 | 0.063468135 | NIPBL, GNAS, BCL2, STRA6 | 4.341416567 |
| GOTERM_MF_DIRECT | GO:0032403~protein complex binding | 16 | 0.064626016 | DYNC1I2, LRRK2, NOG, AKAP5, MYSM1, CD2AP, WRN, SYNJ1, PPP2R2B, KCNQ1OT1, KRIT1, EXOC4, ERCC8, ERCC6, SPTBN1, RPS24 | 1.648434455 |
| GOTERM_BP_DIRECT | GO:2000114~regulation of establishment of cell polarity | 3 | 0.064826868 | KRIT1, RICTOR, KIF20B | 7.122636555 |
| GOTERM_BP_DIRECT | GO:0060322~head development | 3 | 0.064826868 | DLX6, RPGRIP1L, STRA6 | 7.122636555 |
| GOTERM_MF_DIRECT | GO:0070034~telomerase RNA binding | 3 | 0.068268135 | DKC1, PINX1, HNRNPU | 6.915697674 |
| GOTERM_MF_DIRECT | GO:0048029~monosaccharide binding | 3 | 0.068268135 | LGALS3, RPE, OGT | 6.915697674 |
| GOTERM_CC_DIRECT | GO:0005732~small nucleolar ribonucleoprotein complex | 3 | 0.072599911 | NOP58, DKC1, MPHOSPH10 | 6.685481129 |
| GOTERM_BP_DIRECT | GO:0006915~apoptotic process | 22 | 0.07403519 | RBM25, ASAH2, API5, DCC, PRUNE2, MALT1, CCAR1, IFIT2, SCML2, CASP12, NAIP6, PPP2R2B, DLC1, STK17B, BCL7B, ELMO1, BCL2, GAS2, TAX1BP1, TRIM69, PAK2, OGT | 1.466180156 |
| GOTERM_BP_DIRECT | GO:0070232~regulation of T cell apoptotic process | 2 | 0.076759699 | LGALS3, GIMAP8 | 25.32492997 |
| GOTERM_BP_DIRECT | GO:0019065~receptor-mediated endocytosis of virus by host cell | 2 | 0.076759699 | CAV2, EPS15 | 25.32492997 |
| GOTERM_BP_DIRECT | GO:0031344~regulation of cell projection organization | 2 | 0.076759699 | PIFO, ERMN | 25.32492997 |
| GOTERM_BP_DIRECT | GO:0051276~chromosome organization | 4 | 0.077304988 | SFPQ, LIG4, SMC3, ATR | 3.998673153 |
| GOTERM_MF_DIRECT | GO:0034595~phosphatidylinositol phosphate 5-phosphatase activity | 2 | 0.07899304 | INPP5F, SYNJ1 | 24.58914729 |
| GOTERM_BP_DIRECT | GO:0048546~digestive tract morphogenesis | 3 | 0.079887927 | SFRP5, BCL2, STRA6 | 6.331232493 |
| GOTERM_MF_DIRECT | GO:0005515~protein binding | 125 | 0.080709373 | ITSN2, CCNT2, HNRNPU, CCP110, IRS2, IFIT1, SMC3, ZFP119A, CCAR1, LGALS3, TBK1, TBC1D32, CSPP1, NFATC2IP, LEPR, CAR8, CCNL1, PRKCG, RALGAPA1, MAGI3, ATG10, LIG4, QTRTD1, NEAT1, RC3H2, CD2AP, ASTL, DKC1, ELMO1, PKP2, PLAA, KCTD12, KIF20B, ZFPM2, CLOCK, INO80B, GCM1, STXBP3, ITPR2, INPP5F, KCNQ1OT1, ZFP53, DHX36, MPDZ, SPTBN1, SCAI, STRA6, EGR2, HOMER1, IQSEC3, LNPEP, NR1D2, GDF7, TREML1, SYNJ1, KITL, FUBP1, BCL2, GNAS, FBXL3, OGT, ATR, ZCCHC11, LRRK2, PINX1, CHD5, DUSP16, GRM1, CDC73, RPGR, SCML2, CASP12, RB1CC1, IQCB1, SUN3, GRIFIN, APOBEC3, DCC, STRADA, PDE4D, ITGA1, PIFO, CACYBP, GAB3, RAD23B, ASPN, SFPQ, PLCB4, ZEB1, RBL1, VAMP7, PIK3CA, CENPC1, EXOC4, TAX1BP1, PLCB1, VGLL2, ROCK2, AKAP7, PRICKLE1, MALT1, WRN, PLN, FIP1L1, PLAGL1, KRIT1, RICTOR, STX3, PDLIM5, EPS15, PAK2, GBP4, TTC14, SPAG9, RBM39, NPM1, XPO7, MPP5, TJP1, MKLN1, NIN, NEDD4, PLP1, SSBP2, TARDBP | 1.12670213 |
| GOTERM_BP_DIRECT | GO:0014911~positive regulation of smooth muscle cell migration | 4 | 0.082187119 | FGA, EGR1, POSTN, BCL2 | 3.896143073 |
| GOTERM_CC_DIRECT | GO:0005911~cell-cell junction | 10 | 0.085259726 | PRKCG, TJP1, TMEM47, MAGI3, KRIT1, PKP2, RPGRIP1L, STX3, CDC42BPA, CD2AP | 1.884795675 |
| GOTERM_BP_DIRECT | GO:0060325~face morphogenesis | 4 | 0.087198529 | NIPBL, ZFP950, NOG, STRA6 | 3.798739496 |
| GOTERM_BP_DIRECT | GO:0002548~monocyte chemotaxis | 4 | 0.087198529 | LGALS3, CCL8, FLT1, CCL17 | 3.798739496 |
| GOTERM_CC_DIRECT | GO:0030133~transport vesicle | 5 | 0.088185309 | PIGR, VAMP7, ASTL, CAV2, GNAS | 2.959718208 |
| GOTERM_MF_DIRECT | GO:0004004~ATP-dependent RNA helicase activity | 5 | 0.09081406 | YTHDC2, DHX36, DHX15, DDX10, DDX50 | 2.927279439 |
| GOTERM_BP_DIRECT | GO:0040018~positive regulation of multicellular organism growth | 4 | 0.092335694 | GH, NIPBL, ATP8A2, BCL2 | 3.706087313 |
| GOTERM_BP_DIRECT | GO:0035458~cellular response to interferon-beta | 4 | 0.092335694 | GBP6, F830016B08RIK, IFIT1, GBP3 | 3.706087313 |
| GOTERM_BP_DIRECT | GO:0016569~covalent chromatin modification | 12 | 0.09276782 | KMT2E, SETD2, RBL1, HMGN5, PRDM9, PRDM5, CHD6, CHD5, SMARCA1, OGT, MYSM1, ASF1A | 1.713717066 |
| GOTERM_CC_DIRECT | GO:0032391~photoreceptor connecting cilium | 4 | 0.092963663 | IQCB1, PCDHB16, RPGRIP1L, RPGR | 3.696038348 |
| GOTERM_BP_DIRECT | GO:0048278~vesicle docking | 3 | 0.095902499 | CAV2, EXOC5, STX3 | 5.698109244 |
| GOTERM_BP_DIRECT | GO:0007088~regulation of mitotic nuclear division | 3 | 0.095902499 | CAV2, MKI67, KIF20B | 5.698109244 |
| GOTERM_MF_DIRECT | GO:0047485~protein N-terminus binding | 7 | 0.097986665 | NPM1, SCFD1, NIPBL, EXOC4, EXOC5, ERCC6, PDLIM5 | 2.206718346 |
