# Supplementary material for: UTP14A, DKC1, DDX10, PinX1, and ESF1 Modulate Cardiac Angiogenesis Leading to Obesity-Induced Cardiac Injury
Source: J Diabetes Res. 2022 Jun 13;2022:2923291. doi: 10.1155/2022/2923291 (PMC9208995; doi:10.1155/2022/2923291)
Supplement: Supplementary Materials — GSE98226 datasets were downloaded from GEO (http://www.ncbi.nlm.nih.gov/geo/). Supplemental File 1: DEGs. Supplemental File 2: GO enrichment analysis. Supplemental File 3: KEGG pathway analysis. Supplemental File 4: correlation between UTP14A, DKC1, DDX10, PinX1, and ESF1 expression and cardiac angiogenesis. [file 2923291.f1.zip › Supplemental File 3 (1).docx]

Supplemental File 3 : KEGG Pathway Analysis

| Category | Term | Count | % | PValue | Genes | Fold Enrichment | Bonferroni | Benjamini | FDR |
| --- | --- | --- | --- | --- | --- | --- | --- | --- | --- |
| KEGG_PATHWAY | mmu04730:Long-term depression | 7 | 1.013024602 | 0.001399066 | PRKCG, GUCY1A2, PLCB4, GNAS, ITPR2, PLCB1, GRM1 | 5.621488984 | 0.24633646 | 0.175221201 | 0.174353769 |
| KEGG_PATHWAY | mmu04540:Gap junction | 8 | 1.157742402 | 0.001734863 | PRKCG, TJP1, GUCY1A2, PLCB4, GNAS, ITPR2, PLCB1, GRM1 | 4.556954525 | 0.295837932 | 0.175221201 | 0.174353769 |
| KEGG_PATHWAY | mmu04071:Sphingolipid signaling pathway | 9 | 1.302460203 | 0.003612055 | PRKCG, CERS4, PLCB4, ASAH2, PIK3CA, PPP2R2B, ROCK2, BCL2, PLCB1 | 3.555527019 | 0.518551469 | 0.211060848 | 0.210015992 |
| KEGG_PATHWAY | mmu04270:Vascular smooth muscle contraction | 9 | 1.302460203 | 0.004179423 | PRKCG, GUCY1A2, CALCRL, PLCB4, ROCK2, GNAS, ITPR2, PPP1R12B, PLCB1 | 3.471538191 | 0.570877369 | 0.211060848 | 0.210015992 |
| KEGG_PATHWAY | mmu04015:Rap1 signaling pathway | 11 | 1.591895803 | 0.011566723 | PRKCG, PLCB4, FLT1, PIK3CA, KITL, MAGI3, KRIT1, GNAS, LPAR4, PLCB1, SIPA1L3 | 2.518036788 | 0.904639975 | 0.462640894 | 0.460350592 |
| KEGG_PATHWAY | mmu04070:Phosphatidylinositol signaling system | 7 | 1.013024602 | 0.013741809 | PRKCG, PLCB4, PIK3CA, SYNJ1, ITPR2, PLCB1, DGKH | 3.535163176 | 0.938890899 | 0.462640894 | 0.460350592 |
| KEGG_PATHWAY | mmu04611:Platelet activation | 8 | 1.157742402 | 0.01696438 | FGA, GUCY1A2, PLCB4, PIK3CA, ROCK2, GNAS, ITPR2, PLCB1 | 2.991588467 | 0.968450722 | 0.481307035 | 0.478924327 |
| KEGG_PATHWAY | mmu04970:Salivary secretion | 6 | 0.868306802 | 0.01973781 | PRKCG, GUCY1A2, PLCB4, GNAS, ITPR2, PLCB1 | 3.81718918 | 0.982170676 | 0.481307035 | 0.478924327 |
| KEGG_PATHWAY | mmu04510:Focal adhesion | 10 | 1.447178003 | 0.02471058 | PRKCG, FLT1, PIK3CA, CAV2, ROCK2, ITGA10, ITGA1, BCL2, PPP1R12B, PAK2 | 2.366534355 | 0.993617812 | 0.481307035 | 0.478924327 |
| KEGG_PATHWAY | mmu04725:Cholinergic synapse | 7 | 1.013024602 | 0.027048343 | PRKCG, PLCB4, PIK3CA, BCL2, ITPR2, KCNQ5, PLCB1 | 3.034609098 | 0.996069616 | 0.481307035 | 0.478924327 |
| KEGG_PATHWAY | mmu04724:Glutamatergic synapse | 7 | 1.013024602 | 0.029157209 | PRKCG, PLCB4, HOMER1, GNAS, ITPR2, PLCB1, GRM1 | 2.981833287 | 0.997464405 | 0.481307035 | 0.478924327 |
| KEGG_PATHWAY | mmu04925:Aldosterone synthesis and secretion | 6 | 0.868306802 | 0.030138043 | PRKCG, PLCB4, CYP21A1, GNAS, ITPR2, PLCB1 | 3.417715894 | 0.997932694 | 0.481307035 | 0.478924327 |
| KEGG_PATHWAY | mmu04921:Oxytocin signaling pathway | 8 | 1.157742402 | 0.032627098 | PRKCG, GUCY1A2, PLCB4, ROCK2, GNAS, ITPR2, PPP1R12B, PLCB1 | 2.612653928 | 0.998769812 | 0.481307035 | 0.478924327 |
| KEGG_PATHWAY | mmu05143:African trypanosomiasis | 4 | 0.578871201 | 0.033357913 | PRKCG, PLCB4, PLCB1, THOP1 | 5.598544131 | 0.998943982 | 0.481307035 | 0.478924327 |
| KEGG_PATHWAY | mmu04720:Long-term potentiation | 5 | 0.723589001 | 0.044527742 | PRKCG, PLCB4, ITPR2, PLCB1, GRM1 | 3.711156147 | 0.999899062 | 0.557864008 | 0.555102305 |
| KEGG_PATHWAY | mmu04915:Estrogen signaling pathway | 6 | 0.868306802 | 0.048584166 | PLCB4, PIK3CA, GNAS, ITPR2, PLCB1, GRM1 | 2.99922007 | 0.999957262 | 0.557864008 | 0.555102305 |
| KEGG_PATHWAY | mmu04918:Thyroid hormone synthesis | 5 | 0.723589001 | 0.053288467 | PRKCG, PLCB4, GNAS, ITPR2, PLCB1 | 3.499090082 | 0.999984297 | 0.557864008 | 0.555102305 |
| KEGG_PATHWAY | mmu04141:Protein processing in endoplasmic reticulum | 8 | 1.157742402 | 0.054454223 | EDEM3, CASP12, BCL2, STT3A, PLAA, YOD1, RAD23B, SEC63 | 2.332726721 | 0.999987757 | 0.557864008 | 0.555102305 |
| KEGG_PATHWAY | mmu04728:Dopaminergic synapse | 7 | 1.013024602 | 0.054662227 | PRKCG, PLCB4, PPP2R2B, GNAS, ITPR2, PLCB1, CLOCK | 2.55903603 | 0.999988289 | 0.557864008 | 0.555102305 |
| KEGG_PATHWAY | mmu04924:Renin secretion | 5 | 0.723589001 | 0.055615022 | GUCY1A2, PLCB4, GNAS, ITPR2, PLCB1 | 3.449807123 | 0.999990448 | 0.557864008 | 0.555102305 |
| KEGG_PATHWAY | mmu04971:Gastric acid secretion | 5 | 0.723589001 | 0.057995763 | PRKCG, PLCB4, GNAS, ITPR2, PLCB1 | 3.401893135 | 0.999994263 | 0.557864008 | 0.555102305 |
| KEGG_PATHWAY | mmu04310:Wnt signaling pathway | 7 | 1.013024602 | 0.066659271 | PRKCG, PLCB4, ROCK2, SFRP5, CACYBP, PRICKLE1, PLCB1 | 2.431991688 | 0.999999113 | 0.612053308 | 0.609023341 |
| KEGG_PATHWAY | mmu04020:Calcium signaling pathway | 8 | 1.157742402 | 0.073134295 | PRKCG, PLN, PLCB4, LTB4R2, GNAS, ITPR2, PLCB1, GRM1 | 2.177211607 | 0.999999783 | 0.640521518 | 0.637350619 |
| KEGG_PATHWAY | mmu05161:Hepatitis B | 7 | 1.013024602 | 0.076101566 | PRKCG, EGR2, TBK1, EGR3, CASP12, PIK3CA, BCL2 | 2.348704302 | 0.999999886 | 0.640521518 | 0.637350619 |
| KEGG_PATHWAY | mmu04961:Endocrine and other factor-regulated calcium reabsorption | 4 | 0.578871201 | 0.088115985 | PRKCG, PLCB4, GNAS, PLCB1 | 3.768250857 | 0.999999992 | 0.711977156 | 0.708452516 |
