# Supplementary material for: UTP14A, DKC1, DDX10, PinX1, and ESF1 Modulate Cardiac Angiogenesis Leading to Obesity-Induced Cardiac Injury
Source: J Diabetes Res. 2022 Jun 13;2022:2923291. doi: 10.1155/2022/2923291 (PMC9208995; doi:10.1155/2022/2923291)

Supplemental File 4 : Correlation between UTP14A, DKC1, DDX10, PinX1 and ESF1 expression and cardiac angiogenesis

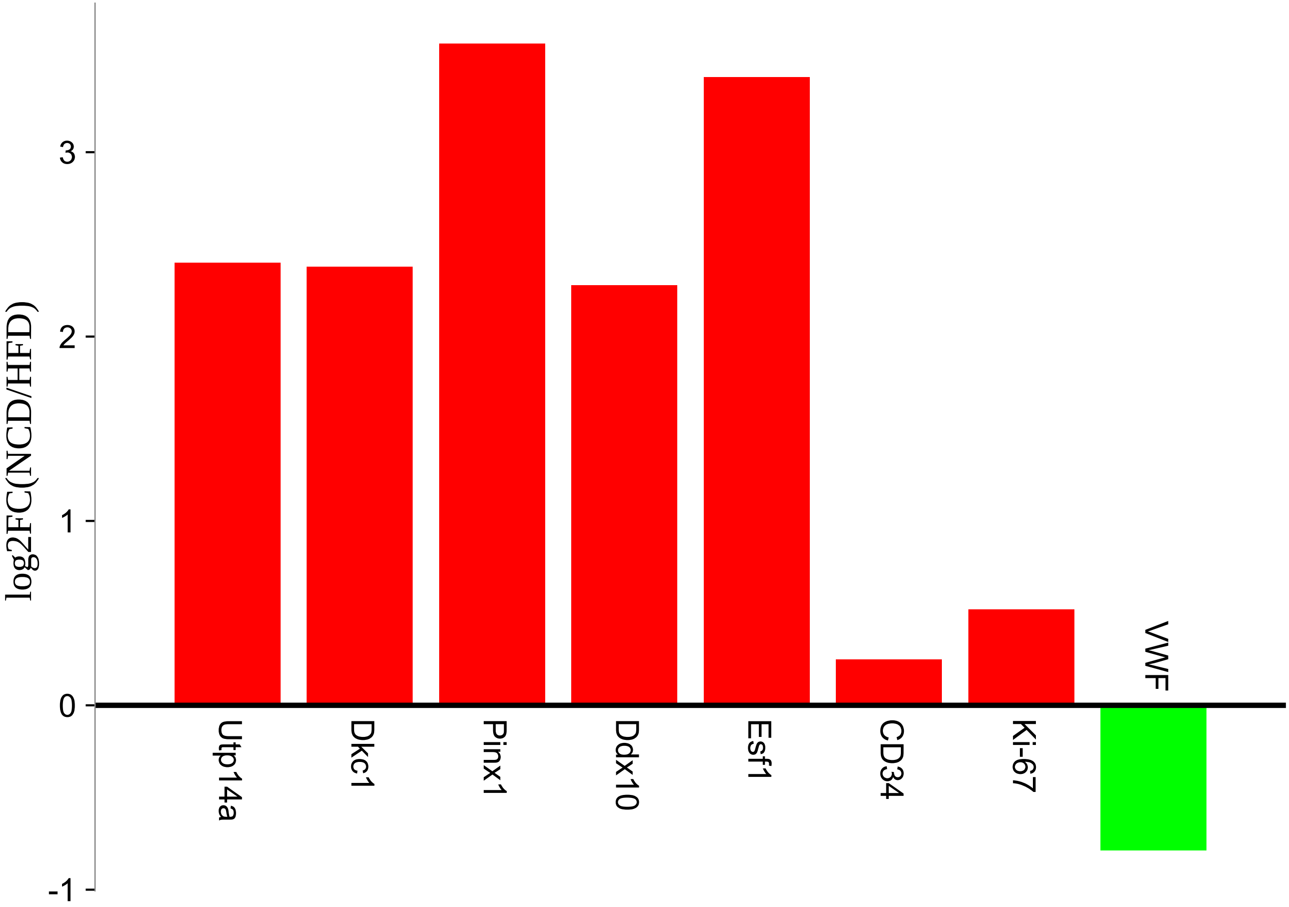

Supplement: Supplementary Materials — GSE98226 datasets were downloaded from GEO (http://www.ncbi.nlm.nih.gov/geo/). Supplemental File 1: DEGs. Supplemental File 2: GO enrichment analysis. Supplemental File 3: KEGG pathway analysis. Supplemental File 4: correlation between UTP14A, DKC1, DDX10, PinX1, and ESF1 expression and cardiac angiogenesis. [file 2923291.f1.zip › Supplemental File 4 (1).pdf]
